# ePoster

**DOI:** 10.1111/ene.70193

**Published:** 2025-06-21

## Saturday, June 21 2025

## Infectious Diseases

## EPO‐001

### Convulsivant status epilepticus over the course of HHV‐6 encephalitis in a type 2 diabetic patient

#### A. Struta ^1^; L. Popa^1^; S. Petrescu^2^; H. Nicolae^2^; C. Panea^2^

##### ^1^Neurology, Elias Emergency University Hospital, Bucharest, Romania; ^2^Neurology, Carol Davila University of Medicine and Pharmacy, Bucharest, Romania

**Background and aims:** Human Herpes Virus 6 (HHV‐6) is an omnipresent virus in the pediatric population, often associated with an exanthematic disease. HHV‐6 encephalitis in immunocompetent adults is exceptional, with the main clinical and radiological features frequently resembling limbic encephalitis.

**Methods:** A 68‐year‐old patient with a history of type 2 diabetes, arterial hypertension, and hypercholesterolemia, presented to the emergency room for two new‐onset bilateral tonic‐clonic seizures (during the day of admission), confusion, fever, headache, photophobia (insidious progression over the last four days). Non‐contrast brain computed tomography was unremarkable. Recent medical history included contact with a two‐year‐old who showed symptoms of acute upper respiratory tract infection. The patient also reported significant voluntary weight loss (approximately 13 kilograms in one month). The spinal tap revealed increased CSF (cerebrospinal fluid) protein levels (1,33g/L). CSF cultures were positive for HHV‐6 and antiviral therapy with Ganciclovir was initiated. HIV (human immunodeficiency virus) serology was negative. Five days after admission, the patient developed convulsive status epilepticus and was transferred to Acute Critical Care where he received anti‐seizure medication and required mechanical ventilation. Brain imaging by contrast magnetic resonance (performed six days after admission) revealed a T2/FLAIR hypersignal (gliotic) lesion in the central midbrain, but no abnormalities of the temporal lobes/limbic structures.

**Results:** The clinical progression was favorable after receiving anti‐viral, supportive, and anti‐seizure medication (no epileptic seizures and extubation after 5 days).

**Conclusion:** Although rare and most frequently associated with hematopoietic stem cell transplantation, HHV‐6 primary infection/reactivation can cause severe neurological complications in adults, albeit sometimes lacking radiologic findings of limbing encephalitis.

**Disclosure:** Nothing to disclose.

## EPO‐002

### Early recognition of cerebral neuroschistosomiasis: Diagnostic and therapeutic insights in endemic regions

#### A. Teixeira Vaz; C. Lopes Figueiredo; M. Valente; L. Machado Dumont; R. Queiroga; C. Veloso; F. Brito; A. Penalva

##### Neurology, Santa Casa de São Paulo, São Paulo, Brazil

**Background and aims:** Cerebral neuroschistosomiasis is a rare complication of Schistosoma mansoni infection, which is highly prevalent in Brazil, particularly in endemic regions like Pernambuco. ‐ Although spinal cord involvement is more common, cerebral manifestations can occur and pose diagnostic challenges. ‐ This study discusses the diagnostic and therapeutic challenges of cerebral neuroschistosomiasis, illustrated by a unique clinical case.

**Methods:** This study analyzes the clinical presentation, diagnostic process, imaging findings, and therapeutic outcomes of a rare case of cerebral schistosomiasis. A comprehensive review of the literature was conducted to contextualize the case findings and provide an evidence‐based discussion on diagnostic challenges and therapeutic strategies in endemic regions.

**Results:** Clinical Features: A 16‐year‐old female presented with a 2‐week history of right hemicranial headache, nausea, vomiting, and left homonymous hemianopia, with no systemic symptoms, highlighting the importance of isolated focal neurological signs. ‐ Imaging Findings: Brain MRI revealed characteristic cortical and subcortical signal changes with linear and micronodular enhancement. ‐ Differential Diagnosis: Included neoplasms, brain abscesses, and demyelinating diseases. However, epidemiological history and characteristic imaging patterns strongly suggested cerebral neuroschistosomiasis. ‐ Treatment: Praziquantel and corticosteroids led to complete symptom resolution, underscoring the efficacy of this approach.

**Figure:**
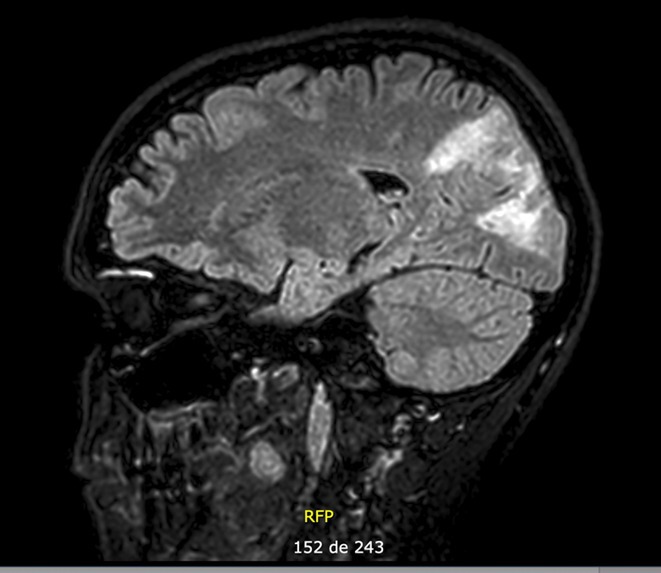
**FIGURE 1** Brain MRI in sagittal flair

**Figure:**
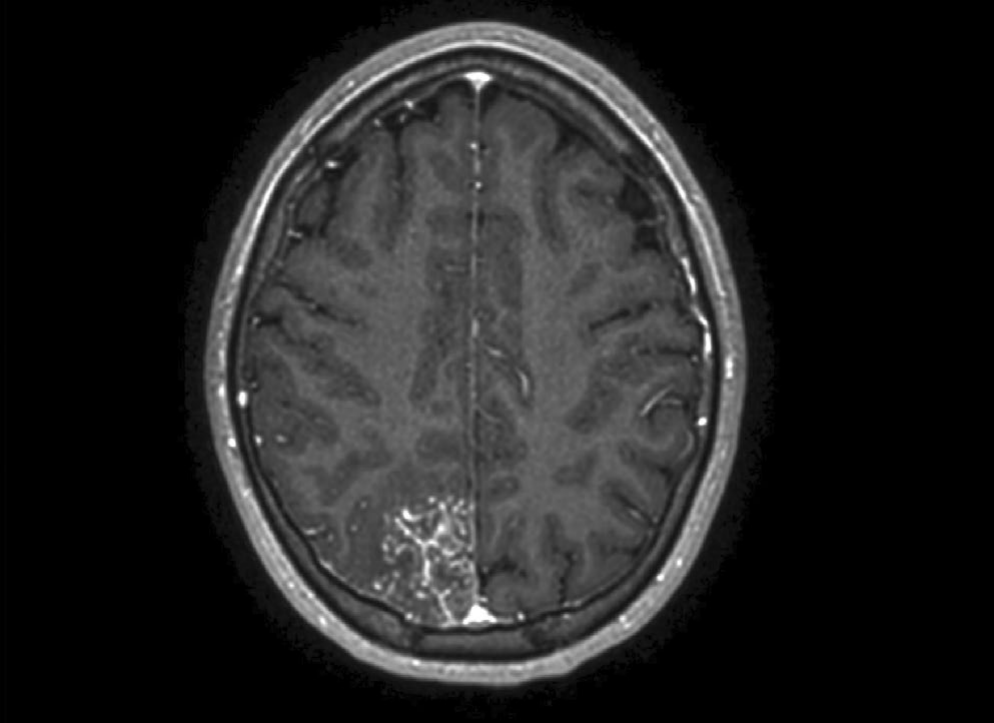
**FIGURE 2** Brain MRI in axial T1

**Figure:**
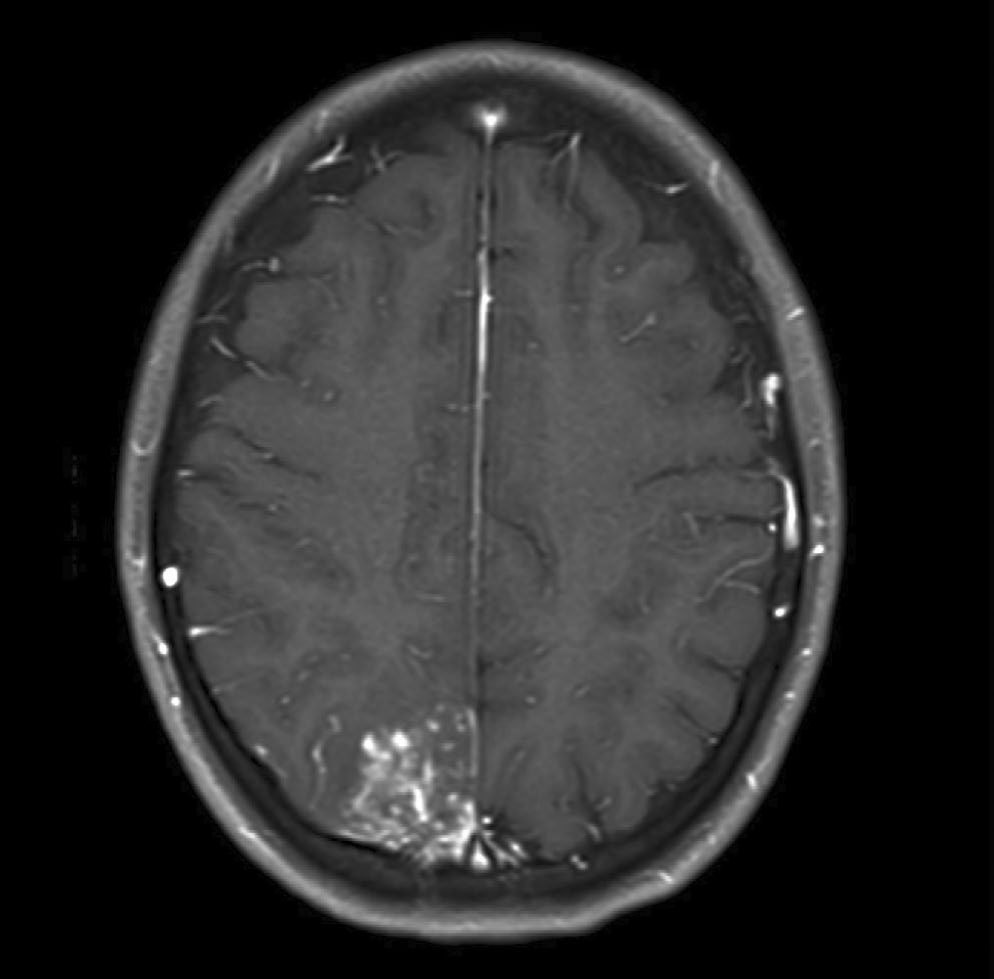
**FIGURE 3** Brain MRI in axial T1‐weighted post‐contrast

**Conclusion:** ‐ Cerebral neuroschistosomiasis should be considered in patients from endemic regions presenting with atypical neurological symptoms. ‐ The combination of clinical history, characteristic imaging findings, and serology is essential for early diagnosis. ‐ Proper treatment can prevent severe complications and significantly improve patient outcomes.

**Disclosure:** Nothing to disclose.

## EPO‐003

### EBV encephalitis in disguise: Navigating diagnostic obstacles in a polyallergic patient with severe hyponatremia

#### A. Ștefănache ^1^; S. Dumitrescu^1^; A. Iacob^1^; D. Ștefani^1^; C. Sîrbu^2^

##### ^1^Department of Neurology, Central Military Emergency University Hospital “Dr. Carol Davila”, Bucharest, Romania; ^2^Clinical Neuroscience Department, Carol Davila University of Medicine and Pharmacy, Bucharest, Romania

**Background and aims:** Epstein‐Barr virus (EBV) is a rare cause of encephalitis in immunocompetent adults, with diagnosis often complicated by nonspecific clinical presentation that overlaps with other neurological conditions. In this case, the diagnostic process was complicated by severe hyponatremia, which raised suspicion of a paraneoplastic SIADH, delaying the identification of the cause of encephalopathy.

**Methods:** A 54‐year‐old polyallergic female presented with confusion and worsening general condition over 6 days, without fever or elevated inflammation markers. Initial head CT suggested an expansive process in the parieto‐temporo‐occipital region. Non‐contrast brain MRI performed 6 days later showed cerebellar and occipital lesions, suggesting an ischemic event. Severe hyponatremia raised suspicion of paraneoplastic SIADH, prompting a chest‐abdomen‐pelvis CT that revealed a possible pancreatic tumor. Due to progressive ventricular dilation lumbar puncture was contraindicated, delaying the diagnosis; cerebrospinal fluid was later obtained through an external ventricular drain.

**Results:** Following a contrast‐enhanced MRI with prior desensitization for allergic reactions, both encephalitis and cerebral metastases were considered, as the lesions were non‐enhancing. Based on clinical suspicion, antiviral therapy, corticosteroids and cephalosporins were initiated. Serology showed positive EBV IgG and IgM, and EBV was detected in the cerebrospinal fluid, confirming EBV encephalitis. Despite aggressive treatment, the patient's condition worsened and she ultimately succumbed to the disease.

**Conclusion:** This case highlights the challenges in diagnosing EBV encephalitis, particularly when paraneoplastic SIADH complicate the clinical picture. It underscores the importance of a comprehensive differential diagnosis and the critical role of a multidisciplinary approach in overcoming diagnostic challenges and facilitating accurate treatment.

**Disclosure:** Nothing to disclose.

## EPO‐004

### The role of cognitive reserve in mediating HANDs in older adults living with‐treated HIV in Tanzania

#### E. Kuhoga ^1^; B. Mbwele^1^; M. Sadler^2^; N. Reddy^2^; E. Shime^1^; K. Mdee^1^; K. Mpoki^1^; M. Benson^1^; R. Walker^2^; G. Livingston^3^; W. Gray^4^; S. Paddick^2^

##### ^1^Department of Epidemiology, Bio‐Statistics and Clinical Research, University of Dar es Salaam‐Mbeya College of Health and Allied Sciences, UDSM‐MCHAS, Mbeya, Tanzania; ^2^Newcastle University, Newcastle Upon Tyne, UK; ^3^Division of Psychiatry, University College London, London, UK, ^4^5Northumbria Healthcare NHS Foundation Trust, North Tyneside General Hospital, North Shields, UK

**Background and aims:** HIV‐associated neurocognitive disorders (HAND) are a spectrum of cognitive impairments in chronic HIV infection. HAND is common in sub‐Saharan Africa (SSA), despite combination antiretroviral therapy (cART). Older people appear to be at increased risk. It is unknown if cognitive reserve (CR), which is protective in neurodegenerative dementias, protects against HAND.

**Methods:** Cross‐sectional observational study completed in hospital outpatient clinics in Southwest Tanzania. We assessed HIV‐positive participants aged ≥50 years established on cART using a neuropsychological test battery, functional assessment, informant history and depression screen. Control participants were HIV‐negative individuals attending chronic disease clinics. We used operationalised Frascati criteria for HAND diagnosis. CR was measured using the Cognitive Reserve Index (CRI) and other proxy measures.

**Results:** The prevalence of HAND was 64.4% (n = 219/343). Lower CRI score [odds ratio (OR) = 0.971, p = 0.009] and less formal education (OR = 4.364, p = 0.026) were independent risk factors for HAND but HIV‐severity measures were not. Unemployment and low‐skilled manual work were associated with increased risk of HAND in bivariate analysis but not in multivariable analysis.

**Conclusion:** Higher total CRI score and more formal education appeared to be protective against HAND, in this cohort. Potentially, cognitively and socially stimulating activities and exercise could increase cognitive reserve in later life. Cognitive reserve could possibly be more important than HIV‐disease severity in risk of HAND in older people with treated HIV.

**Disclosure:** Nothing to disclose.

## EPO‐005

### Clinical and neurological outcomes in patients with confirmed west Nile Virus: A descriptive study

#### E. Rraklli ^1^; I. Alushi^1^; V. Papa^2^

##### ^1^Department of Neurology, Regional Hospital Center of Berat, Tirana, Albania; ^2^Department of Infection, Regional Hospital Center of Berat, Tirana, Albania

**Background and aims:** West Nile Virus (WNV) is a mosquito‐borne infection that can lead to severe neurological complications. Pre‐existing conditions such as arterial hypertension and diabetes mellitus may affect the severity and outcomes of WNV infection. This study examines the clinical and neurological outcomes in WNV patients, with a focus on the impact of comorbidities

**Methods:** The study included 22 patients diagnosed with WNV through serological and PCR testing. Demographic data, including age and comorbidities (arterial hypertension and diabetes mellitus), were collected. Patients were monitored for acute neurological complications such as encephalitis, meningitis, and paralysis. A three‐month follow‐up assessed the long‐term effects of the infection

**Results:** The cohort had a mean age of 63 years, with a balanced male and female distribution. Comorbidities were prevalent, with 54.5% diagnosed with arterial hypertension and 36.4% with diabetes mellitus. These conditions were linked to more severe WNV‐related complications. During the acute phase, encephalitis was the most common neurological complication, followed by axonal neuropathy. At the one‐month follow‐up, several sequelae were observed, including cognitive and behavioral disorders (18.2%), myalgia (27.3%), persistent axonal neuropathy (4.5%), tremor (4.5%), and cranial nerve paralysis (4.5%). Additional rare complications included epilepsy (4.5%) and subacute thyroiditis (4.5%).

**Figure:**
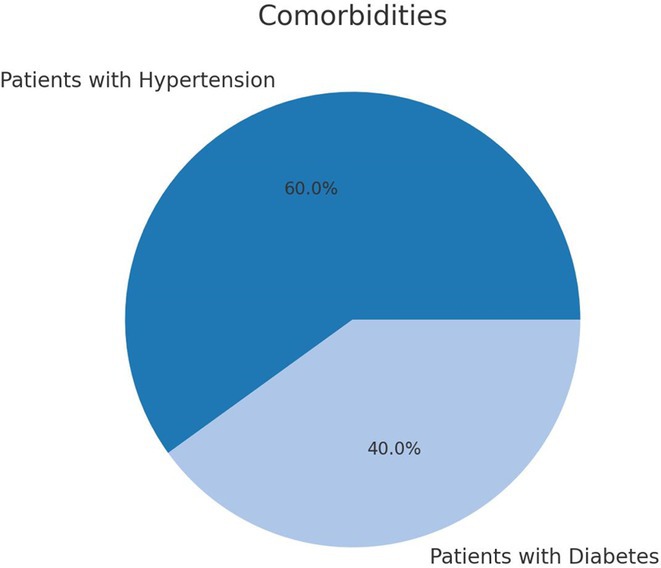
**FIGURE 1** Comorbidities Prevalent in WNV infection

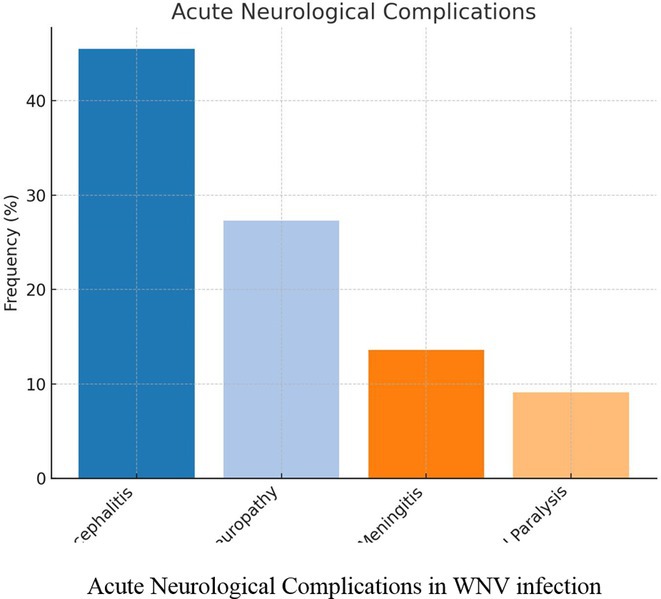

**Figure:**
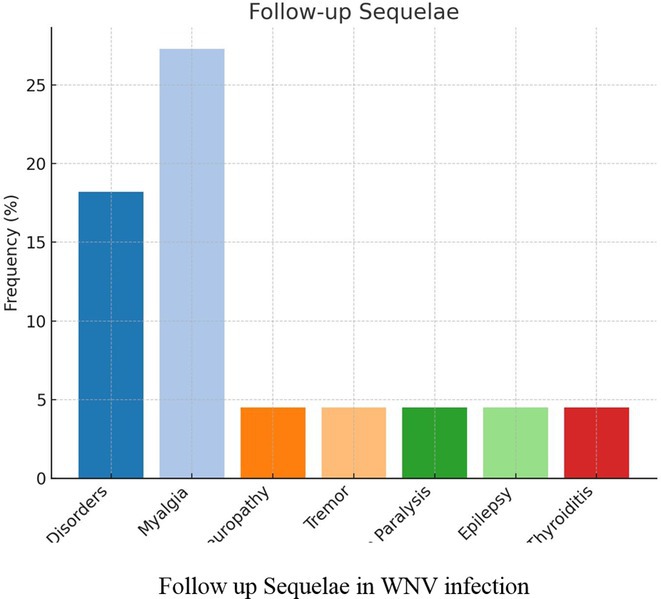
FIGURE 2

**Conclusion:** Pre‐existing health conditions contributed to more severe WNV complications and slower recovery. The study emphasizes the need for ongoing neurological care and monitoring for individuals with comorbidities

**Disclosure:** Nothing to disclose.

## EPO‐006

### Cerebrospinal fluid findings in patients with acute COVID‐19 delirium: a multicenter retrospective data study

#### F. Boesl ^1^; L. Pechstein^1^; J. Hardenberg^2^; K. Rubarth^2^; T. Nguyen^2^; N. Akbari^3^; S. Treskatsch^4^; C. Franke^1^; S. Angermair^4^

##### ^1^Department of Neurology and Experimental Neurology, Charité – Universitätsmedizin Berlin, Berlin, Germany; ^2^Institute of Medical Informatics, Charité – Universitätsmedizin Berlin, Berlin, Germany; ^3^Institute of Biometry and Clinical Epidemiology, Charité – Universitätsmedizin Berlin, Berlin, Germany; ^4^Department of Anesthesiology and Intensive Care Medicine, Germany

**Background and aims:** Delirium is a frequent neuropsychiatric complication in critically ill COVID‐19 patients. Pathophysiological mechanisms leading to delirium remain unclear, with hypothesized contributors including systemic inflammation, blood‐brain barrier disruption, and neuroinflammation. Cerebrospinal fluid analysis (CSF) could be crucial to better understand underlying mechanisms, but CSF data on delirium are limited.

**Methods:** We retrospectively analyzed the clinical and CSF data of 55 critically ill COVID‐19 patients with delirium admitted to one of the seven COVID‐19 intensive care units of Charité ‐ University Medicine Berlin between February 2020 and December 2021. Delirium was diagnosed using the Confusion Assessment Method for the ICU. CSF parameters assessed included pleocytosis, blood‐brain barrier integrity, intrathecal immunoglobulin synthesis, total protein, lactate, neurofilament light chain protein, and anti‐neuronal autoantibodies.

**Figure:**
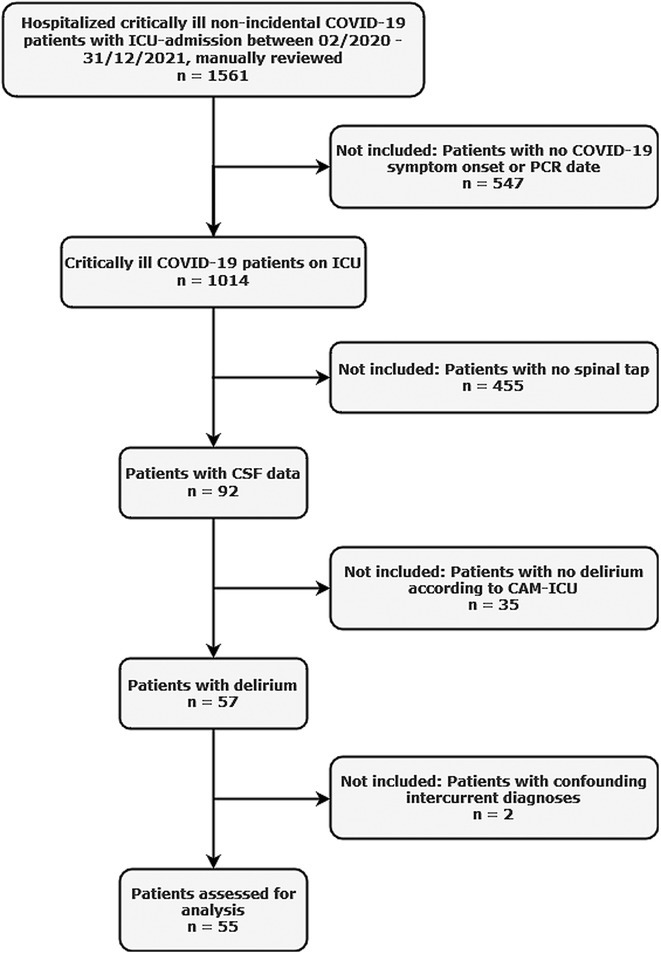
**FIGURE 1** Flowchart of patient selection

**Results:** Blood‐brain barrier disruption was observed in 53% of patients, while pleocytosis occurred in only 4%. Elevated CSF total protein and lactate were noted in 43% and 22% of patients. Intrathecal immunoglobulin synthesis was rare (4%). Anti‐neuronal autoantibodies were infrequent in CSF (2%), but more common in serum (41%). Notably, CSF neurofilament light chain protein levels were elevated in 81% of patients, indicating significant axonal injury.

**Conclusion:** Our findings highlight CSF alterations in critically ill COVID‐19 patients with delirium, characterized by frequent blood‐brain barrier disruption, neuronal injury, and protein leakage, but minimal cellular immune response or intrathecal immunoglobulin synthesis. The presence of anti‐neuronal autoantibodies in serum suggests a potential autoimmune contribution, though their direct pathogenic role remains uncertain. These results suggest that multifactorial mechanisms, including systemic inflammation and blood‐brain barrier dysfunction drive COVID‐19‐related delirium.

**Disclosure:** Nothing to disclose.

## EPO‐007

### Lyme radiculoneuritis presenting with hyperacute lower limb weakness and a benign course, an atypical Bannwarth Syndrome

#### G. Mullane, O. O’Toole

##### Cork University Hospital, Cork, Ireland

**Background and aims:** We present a 49‐year‐old lady who presented with an episode of acute lower limb weakness. The patient reported instantaneous bilateral lower limb weakness, resulting in a fall to the ground. The patient did not have any pre‐existing medical conditions and did not take any regular medications. There were no sensory symptoms and no upper limb weakness. She denied any recent illnesses or trauma. Examination demonstrated bilateral lower limb weakness and hyporeflexia. There were no sensory deficits and upper limb examination was unremarkable.

**Methods:** Neurophysiological evaluation was requested, which demonstrated reduced right common peroneal motor amplitude, but otherwise motor amplitudes and conduction velocities were within accep limits. Sensory responses were normal. Electromyography demonstrated florid acute denervation in both lower limbs in an L2‐S2 distribution as well as upper limbs to a lesser degree. MRI brain and whole spine were unremarkable. Cerebrospinal fluid analysis demonstrated mildly elevated protein.

**Results:** Following review at a tertiary neurology centre 8 months following initial presentation, motor strength had normalised with no residual deficits. Further review noted frequent international travel and subsequent Borrelia burgdorferi serology revealed positive IgG and IgM suggesting recent infection. The patient completed a course of doxycycline. Subsequent neurophysiology showed complete resolution of previous features.

**Conclusion:** This case highlights an atypical hyperacute presentation of Lyme radiculoneuritis. While this frequently presents with concurrent neuropathic pain and sensory symptoms, both were absent in this case, broadening the clinical spectrum of this condition and highlighting Lyme disease as a rare but important differential in acute flaccid paresis.

**Disclosure:** Nothing to disclose.

## EPO‐008

### The accuracy of Bacterial Meningitis (BMS) in identifying pediatric patients high risk for bacterial meningitis

#### J. Maruquin ^1^; J. Viado^2^

##### ^1^Department of Pediatrics, Mariano Marcos Memorial Hospital and Medical Center/Batac City, Philippines; ^2^Department of Pediatrics, Mariano Marcos Memorial Hospital and Medical Center/Batac City, Philippines

**Background and aims:** Bacterial and aseptic meningitis have similar presentations but vastly different treatments and outcomes. Early identification of bacterial meningitis is crucial for appropriate evaluation and antibiotic therapy. The Bacterial Meningitis Score (BMS) helps clinicians distinguish between the two.

**Methods:** This cross‐sectional single‐center study involved 75 pediatric patients, aged 29 days to 18 years, suspected of meningitis, seen in the Emergency Room from March to November 2023 at a tertiary hospital in the Philippines. Eligible patients were selected based on inclusion and exclusion criteria. Lumbar punctures were performed to obtain cerebrospinal fluid (CSF) for analysis. High‐risk predictors were scored using the Bacterial Meningitis Score (BMS): positive CSF Gram (2 points), CSF absolute neutrophil count ≥1000 cells/μL (1 point), CSF protein ≥80 mg/dL (1 point), peripheral absolute neutrophil count ≥10,000 cells/μL (1 point), and seizures prior to or during presentation (1 point). Predictors were classified as very low risk (BMS=0) or not very low risk (BMS ≥1).

**Figure:**
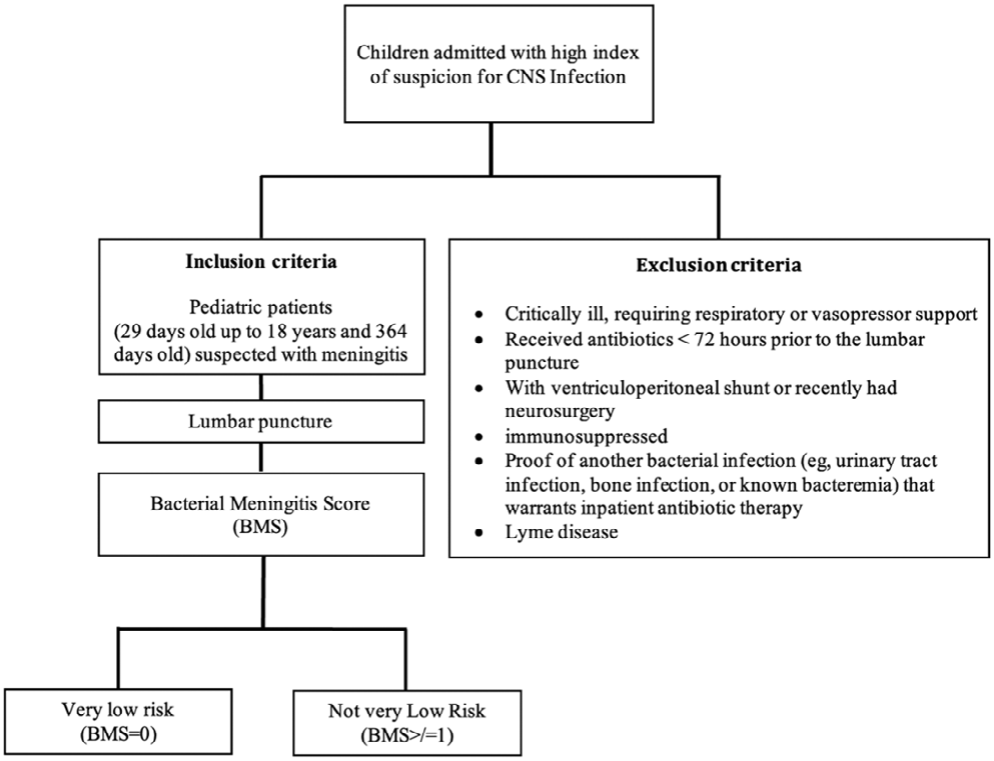
**FIGURE 1** Patient Flow Diagram

**Results:** The sensitivity of the Bacterial Meningitis Score (BMS >/=1) for bacterial meningitis is 100% (95% CI), and the specificity is 19.70% (95% CI). The positive and negative likelihood ratios were 1.23 (positive predictive value 24%, 95% CI, 1.10 – 1.53) and 0 (negative predictive value 100%, 95% CI, 0.01 – 1.68), respectively.

**Figure:**
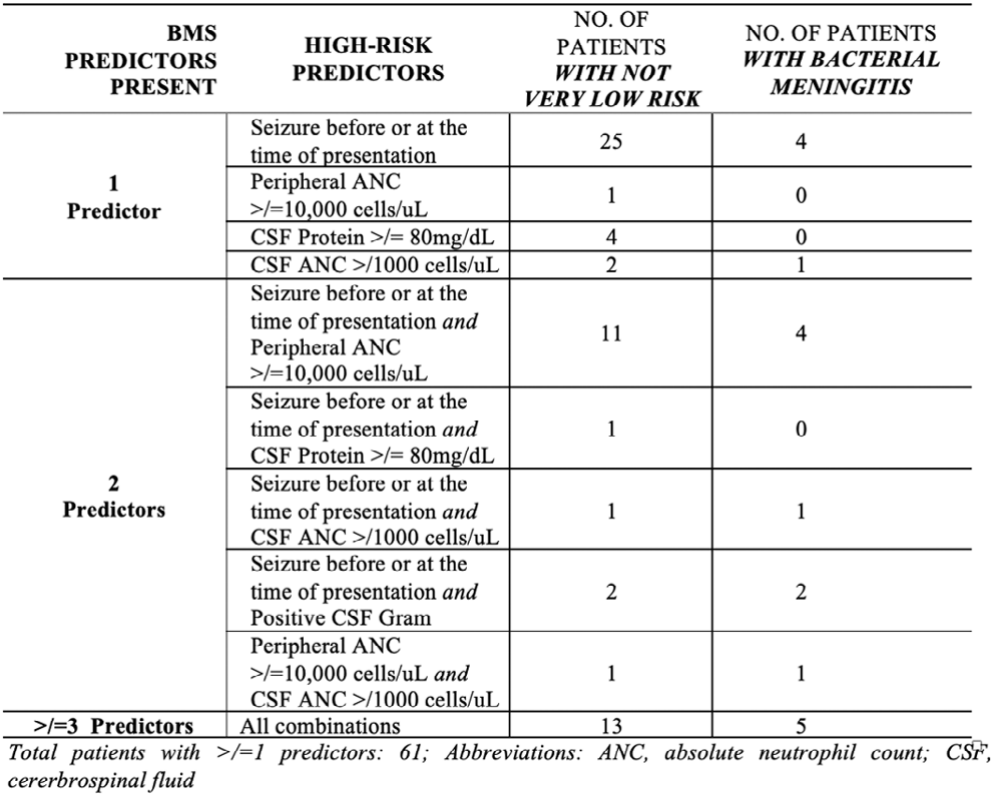
**FIGURE 2** Risk of Bacterial Meningitis for Patients with 1, 2, or 3 or more Bacterial Meningitis Score Predictors

**Figure:**
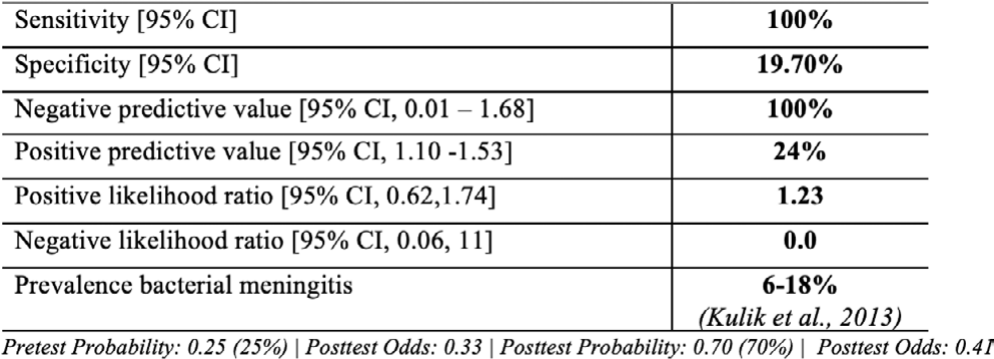
**FIGURE 3** Performance of Bacterial Meningitis Score (BMS)

**Conclusion:** The BMS's high sensitivity and negative predictive value effectively rule out bacterial meningitis. However, its low specificity and positive predictive value require cautious interpretation of positive results. It's a useful initial screening tool, but further evaluation and testing are necessary for diagnosis

**Disclosure:** Nothing to disclose.

## EPO‐009

### Bridging the gap: Uncovering cardiovascular risks in HIV patients – A pilot study

#### L. Ngarka; M. Ngwayu; L. Kuate; L. Nfor; M. Mengnjo; A. Njamnshi

##### Yaounde Central Hospital, Yaounde, Cameroon

**Background and aims:** The advent of Highly Active Antiretroviral Therapy (HAART) has significantly improved the life expectancy of HIV‐positive patients, yet it has also coincided with a rising prevalence of cardiovascular disease (CVD), particularly among this population. HIV itself is a recognized independent risk factor for CVD. This study aims to identify cardiovascular risk factors in HIV‐infected individuals at diagnosis and evaluate the feasibility of dual diagnosis.

**Methods:** Conducted over nine months at the Day Hospital of Etoug‐ebe Baptist Hospital in Yaoundé, this cross‐sectional study enrolled patients who tested positive for HIV during screening campaigns. We used adapted questionnaires and simple body measurements to assess cardiovascular risk factors.

**Results:** The study included 73 participants, predominantly female, with an average age of 35.6 years. Notably, hypertension was prevalent in 16.4% of participants, while 8.2% were classified as obese, and 26% demonstrated abdominal obesity. Conversely, 9.6% of participants were underweight. Age and sex had no significant impact on hypertension prevalence, and no clinical signs of atherosclerosis were observed.

**Conclusion:** The high prevalence of cardiovascular disease risk factors among HIV‐positive patients highlights the need for concurrent diagnosis of both conditions. Implementing dual diagnosis is feasible and essential for guiding effective care strategies, ultimately reducing morbidity and mortality.

**Disclosure:** Nothing to disclose.

## EPO‐010

### West Nile Virus: An uncommon cause of meningoencephalitis in Ukraine: A case report

#### M. Liksunova ^1^; A. Afanasieva^1^; M. Bodnar^1^; T. Slobodin^2^

##### ^1^Department of Neurology, LLC “Dobrobut‐Clinic”, Kyiv, Ukraine; ^2^Department of Neurology, Bogomolets National Medical University, Kyiv, Ukraine

**Background and aims:** The West Nile virus (WNV) is an RNA virus from the Flaviviridae family, transmitted by mosquitoes. WNV neuroinvasive disease is a rare life‐threatening complication of WNV infection that is becoming more common in Europe in summertime.

**Methods:** A single case presentation.

**Results:** A 67‐year‐old man was transferred from a local clinic with impaired level of consciousness with a two‐week history of fever, nausea, vomiting, general weakness. The patient lives in a lakeside locality. A papular rash on the skin and bilateral subconjunctival hemorrhages were detected. Neurological examination revealed: disorientation, aphasia and positive meningeal signs. Brain MRI showed signs of leptomeningeal post‐contrast enhancement. In CSF: cytosis – 20 cells/mm^3^, predominantly lymphocytes, protein 1020 mg/L, glucose 3.0 mmol/L. However, the IV acyclovir was started, the patient developed rapid deterioration with a short episode of critical bradycardia and asystole, which led to the mechanical ventilation. The blood tests for sarcoidosis, tick‐borne encephalitis, syphilis, and HIV were negative. The cerebrospinal fluid analyses for Herpesviruses were also negative and acyclovir was withdrawn. Pulse therapy with methylprednisolone was started, which led to the restoration of consciousness and discontinuation of mechanical ventilation. On day 10, WNV infection was diagnosed by positive serology (IgG and IgM). Two weeks later patient was discharged, asymptomatic except for mild ataxia and hearing loss.

**Figure:**
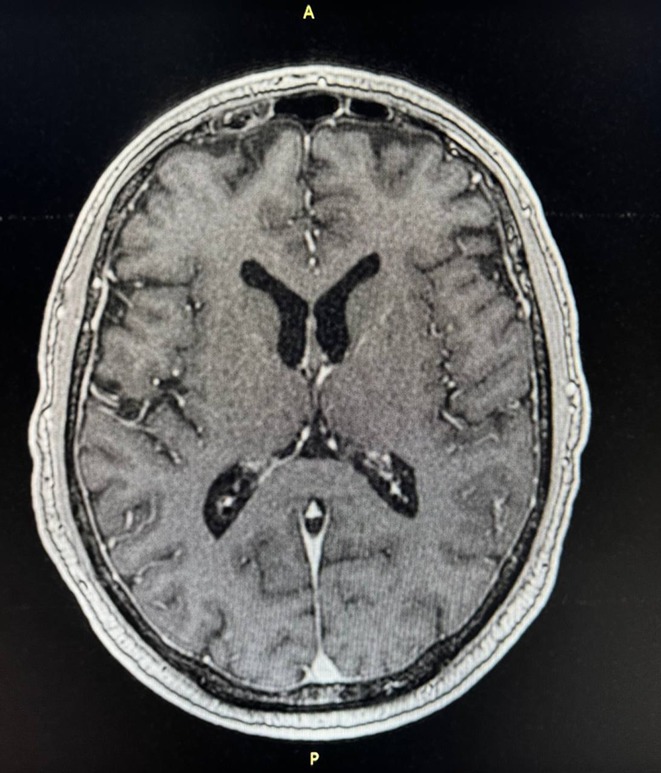
**FIGURE 1** Patient's brain MRI T1 post contract sequence, axial projection ‐leptomeningeal post‐contrast enhancement.

**Figure:**
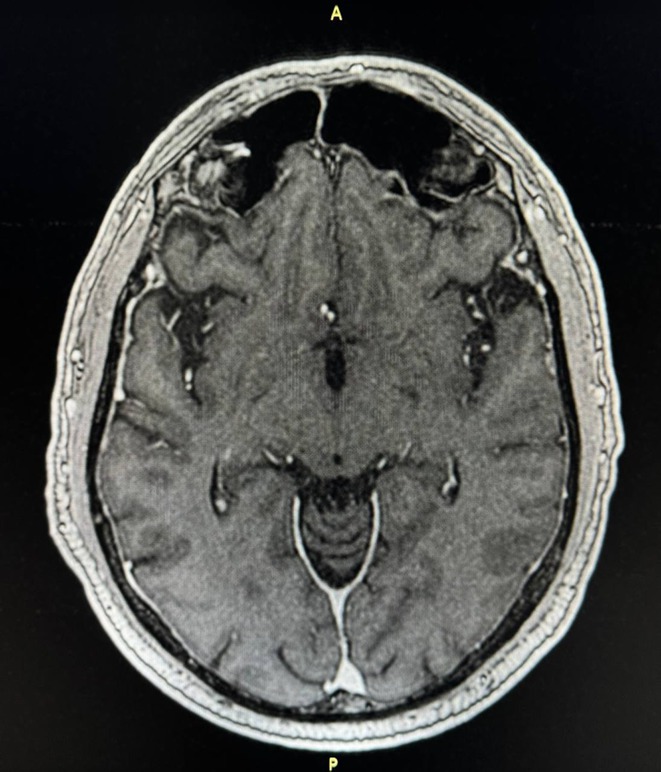
**FIGURE 2** Patient's brain MRI T1 post contract sequence, axial projection ‐leptomeningeal post‐contrast enhancement.

**Figure:**
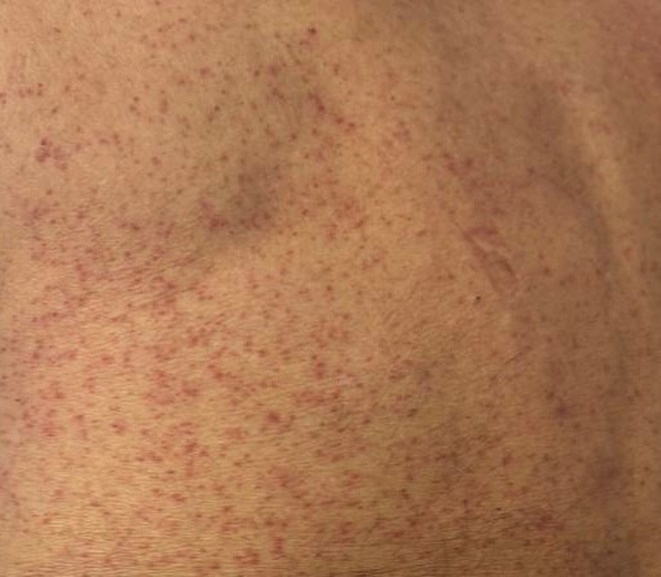
**FIGURE 3** A maculopapular rash on a patient's back.

**Conclusion:** We report this case to emphasize that elderly male patients have a higher risk of developing WNV neuroinvasive disease with high mortality rate. Despite its rarity, WNV is becoming endemic in Europe, highlighting the need for increased awareness.

**Disclosure:** Nothing to disclose.

## EPO‐011

### An unexpected “Souvenir”

#### M. Gilot ^1^; R. Lopez^1^; V. Hernando^1^; P. Nuñez^1^; C. Ferreiro^1^; M. Tamarit^2^; P. Del Valle^1^; I. Hernando^1^; C. Ballester^1^; M. Herrezuelo^1^; M. Elguezabal^1^

##### ^1^University Hospital Severo Ochoa, Madrid, Spain; ^2^University Hospital Getafe, Madrid, Spain

**Background and aims:** Melioidosis is a rare infection caused by the bacteria Burkholderia pseudomallei. It is an endemic infection in Southeast Asia and Northern Australia. Its diagnosis can be challenging because it is an imported pathogen that can mimick other diseases. This is why it is crucial to know this entity and its connection to recent travel history to high‐risk areas.

**Methods:** We describe the case of a 42‐year‐old woman with no relevant medical history. She was referred to the emergency department following a generalized tonic‐clonic epileptic seizure while sleeping. The neurological exam showed a right upper quadrantanopia. The medical history highlighted a recent travel to Vietnam, where she swam in rivers and the sea. In the final days of the trip, she experienced headache, cough, and a mild feverish sensation, treated with amoxiciline.

**Results:** A brain MRI revealed a heterogeneous left temporo‐parietal lesion with irregular anular gadolinium enhance; a pyogenic abscess or a high‐grade glial neoplasm were the main differential diagnoses. A brain biopsy was performed and microbiological culture showed growth of Burkholderia pseudomallei. The patient was treated with Meropenem for 8 weeks, followed by Cotrimoxazole for 6 months. No further seizures were reported and a 3‐month follow‐up MRI showed persistent radiological improvement.

**Figure:**
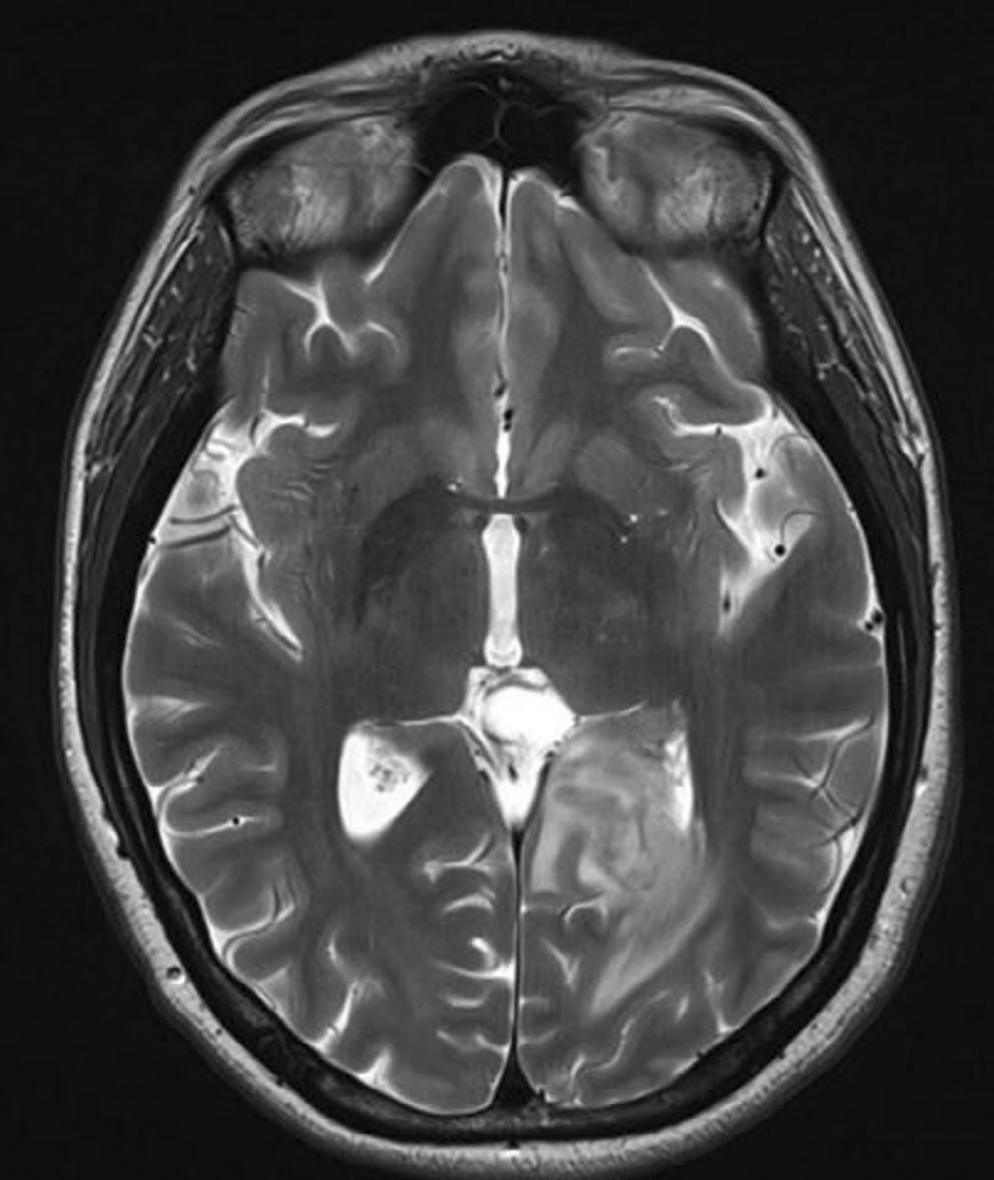
**FIGURE 1** MRI

**Figure:**
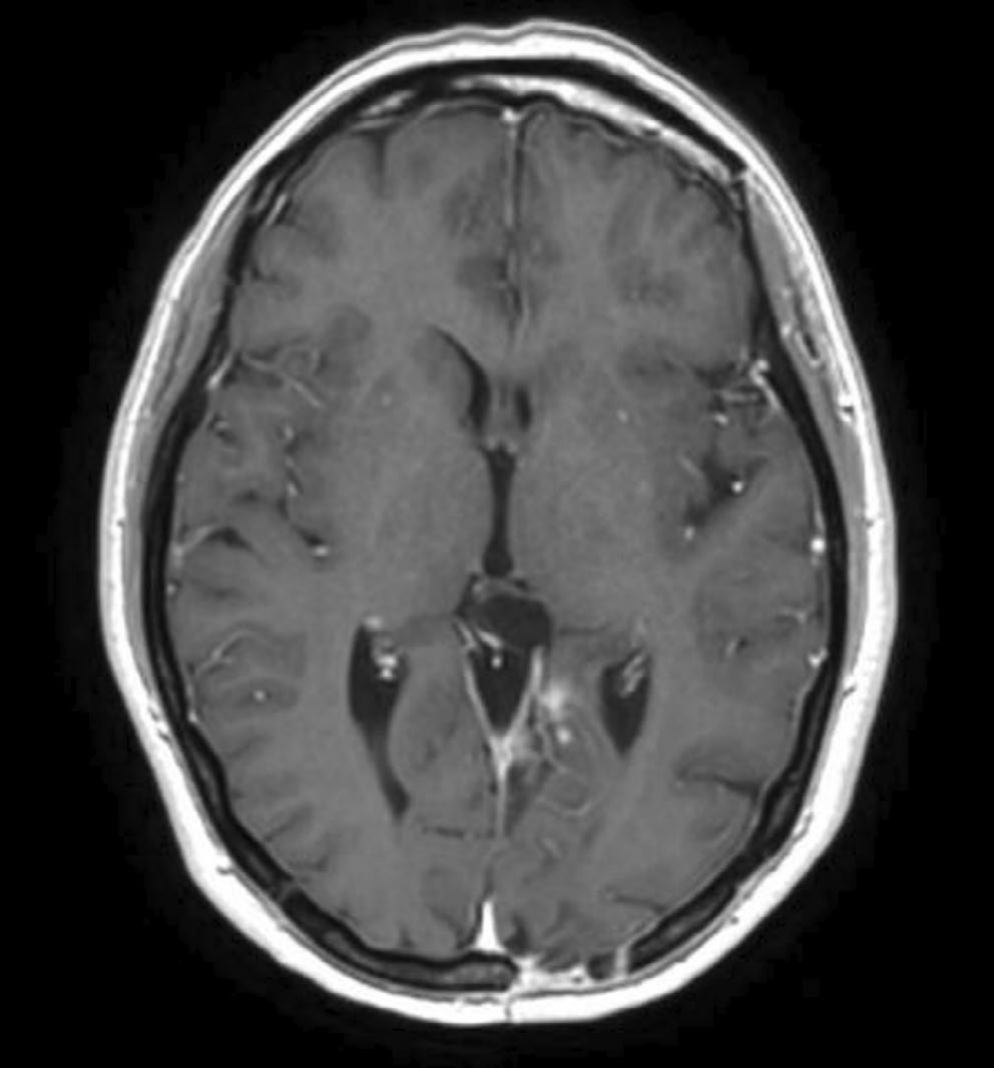
**FIGURE 2** Follow up MRI

**Conclusion:** The case underscores the importance of addressing abscess microbiology with brain biopsy, crucial to diagnose Melioidosis. In closing, we want to raise awareness among Occidental clinicians about the growing number of cases in European regions due to the continued tourism to endemic countries.

**Disclosure:** Nothing to disclose.

## EPO‐012

### Gender‐related differences in subjects with persisting COVID‐19 loss of smell: baseline data from the SMELL‐trial

#### N. De Cleene; K. Seppi; B. Heim

##### Department of Neurology, Medical University of Innsbruck (MUI), Innsbruck, Austria

**Background and aims:** Olfactory dysfunction is one of the most common symptoms of COVID‐19. Indeed, incidence of loss of smell after SARS‐CoV‐2 infection has been estimated by meta‐analysis to be around 50% with subjective recovery rates ranging from around 3 to 90%. Moreover, recent studies have shown that up to 7% of patients remain anosmic for more than 12 months after the onset of COVID‐19 infection.

**Methods:** The SMELL‐trial is a monocentric RCT evaluating the efficacy of olfactory training in individuals with persisting COVID‐19 associated loss of smell (>3 months post‐infection). Olfactory performance was measured using the identification and discrimination subscales of the Sniffin’ Sticks. Data regarding demographics, comorbidities, Quality of Life (QoL,), mental health, well‐being and subjective olfactory impairment (olfactory visual analogue scale, patient global impression of severity) were collected.

**Results:** A total of 70 individuals were included. There were more female (64%) participants. Mean age was 54 years (SD: 14.5) and mean OD duration of 20 months (SD: 11.4). No differences between both sexes were seen for age, BMI, or symptom duration, neither for comorbidities nor smoking history. There were no gender‐related differences for QoL or health, nor for well‐being and mood. No differences were seen for subjective olfactory impairment. However, men had lower scores on both subscales of the Sniffin’ Sticks (identification: 6.85 vs. 9.16, discrimination: 8.81 vs. 10.11).

**Conclusion:** In the SMELL‐trial cohort, there were no gender‐related differences in QoL, well‐being and mood as well as measures of subjective olfactory impairment, while objective assessment of olfactory function was worse in men compared to women.

**Disclosure:** NDC reports no financial disclosures. BH reports honoraria from Novartis AG, BIAL, AbbVie and grants from the Austrian science fund (FWF) outside the submitted work. KS reports honoraria from the International Parkinson and Movement Disorders Society, grants from the FWF Austrian Science Fund, the Michael J. Fox Foundation, and the International Parkinson and Movement Disorder Society, as well as personal fees from Teva, UCB, Lundbeck, AOP Orphan Pharmaceuticals AG, AbbVie, Roche, and Grünenthal outside the submitted work.

## EPO‐013

### The gut‐brain axis in Post‐COVID‐19 condition: a cross‐sectional microbiome and neuroimaging study

#### S. Wetz; J. Walders; R. Dadsena; K. Reetz

##### Department of Neurology, University Hospital RWTH Aachen, Aachen, Germany

**Background and aims:** Post‐COVID‐19 condition (PCC) affects 15% of people worldwide previously infected with SARS‐CoV‐2, having a lasting impact on society, with fatigue and cognitive impairment as the most common symptoms. While the pathogenic mechanisms remain unclear, gut dysbiosis is increasingly recognized to play a key role in PCC pathogenesis, disrupting proper gut‐brain communication and serotonin metabolism. Here, we investigated gut microbiota of PCC patients in relation to neurological manifestations and neuroimaging changes.

**Methods:** In this cross‐sectional study, consisting of 42 subjects (29 females, 46.7±11.3 years) and 30 healthy controls (22 females, 53.9±17.8 years) we analyzed gut microbiota using 16S rRNA gene amplicon sequencing and its relation to neuropsychological performance, reported symptoms and neuroimaging alterations of resting‐state functional and structural magnetic resonance imaging (MRI).

**Results:** We observed significantly altered gut microbiota in PCC patients (PERMANOVA, p<0.001) with a higher abundance of the genera Bacteroides and Alistipes, and a lower abundance of Blautia and Agathobacter compared to healthy controls (Kruskal‐Wallis test, p<0.05, LDA score>2.5). Moreover, we detected brainstem alterations that strongly correlated with both self‐reported fatigue and microbiome dysbiosis.

**Figure:**
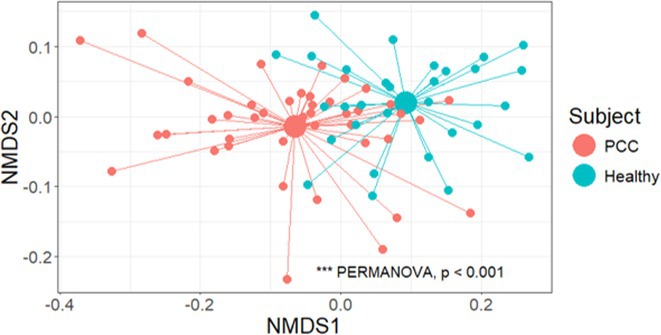
**FIGURE 1** Non‐metric Multi‐dimensional Scaling Plot (NMDS) of gut composition of PCC patients and of a healthy control group, based on weighted Unifrac as dissimilarity measure.

**Figure:**
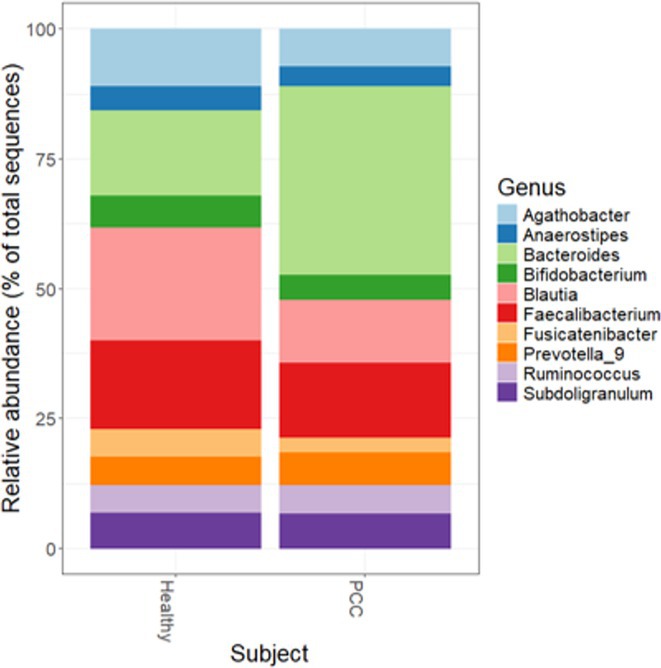
**FIGURE 2** Average relative abundance of top 10 genera found in gut microbiota in PCC patients and a healthy control group.

**Conclusion:** This pioneering study comprehensively investigates the gut‐brain axis in PCC linking gut microbiome alterations to both neurological symptoms and MRI‐detectable brain changes. Our results suggest a significant role of microbiome disruptions in the manifestation of brain changes and key neurological symptoms in PCC. These findings highlight the potential for microbiome‐targeted interventions including dietary modifications, pre‐ and probiotics, selective serotonin reuptake inhibitors, and vagus nerve stimulation, to restore gut‐brain homeostasis and alleviate symptoms.

**Figure:**
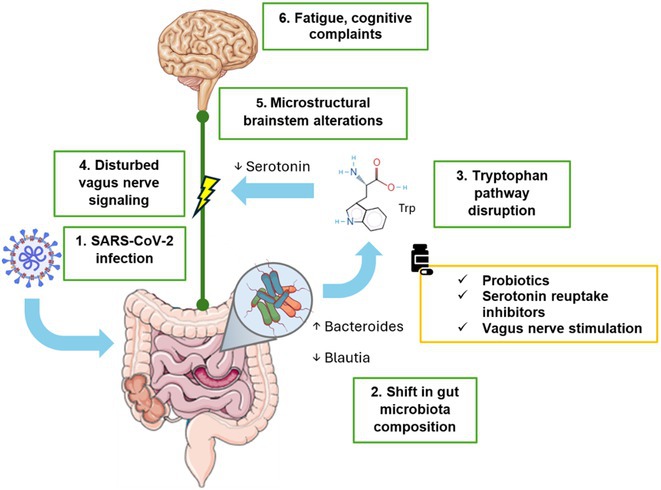
**FIGURE 3** Hypothesized mechanism of gut dysbiosis in Post COVID pathogenesis.

**Disclosure:** Sophie Wetz and Dr. Julia Walders are funded by the Else‐Kröner‐Fresenius Grant (2022_EKEA.58). Dr. Ravi Dadsena and Prof. Dr. Kathrin Reetz have nothing to disclose.

## EPO‐014

### Rapid progression and fatal outcome of HHV‐6 encephalitis in an immunocompromised adult: A case report

#### S. Petrescu; D. Morosanu; R. badea; C. Tiu

##### Department of Neurology, Emergency University Hospital, Bucharest, Romania

**Background and aims:** Human herpesvirus 6 (HHV‐6) is a neurotropic virus with a strong affinity for the central nervous system (CNS). While primary infection is typically self‐limited during childhood, the reactivation of HHV‐6 can lead to serious neurological conditions, particularly in immunocompromised individuals.

**Methods:** We report the case of a 67‐year‐old female with a history of end‐stage kidney disease who developed progressive neurological symptoms, beginning with speech difficulties, quickly evolving into motor deficits, and altered mental status. Diagnostic workup included cerebrospinal fluid (CSF) analysis, revealing positive polymerase chain reaction (PCR) results for HHV‐6, along with findings suggestive of inflammation. Serological testing showed a high viral load of HHV‐6. Magnetic resonance imaging (MRI) demonstrated non‐enhancing hyperintensities on fluid‐attenuated inversion recovery (FLAIR) and diffusion‐weighted imaging (DWI) sequences in the left hemisphere, particularly in the cortical regions, with associated cortical hemorrhages. Abnormal signal changes indicative of encephalitis were also observed in the right frontal and temporal lobes.

**Figure:**
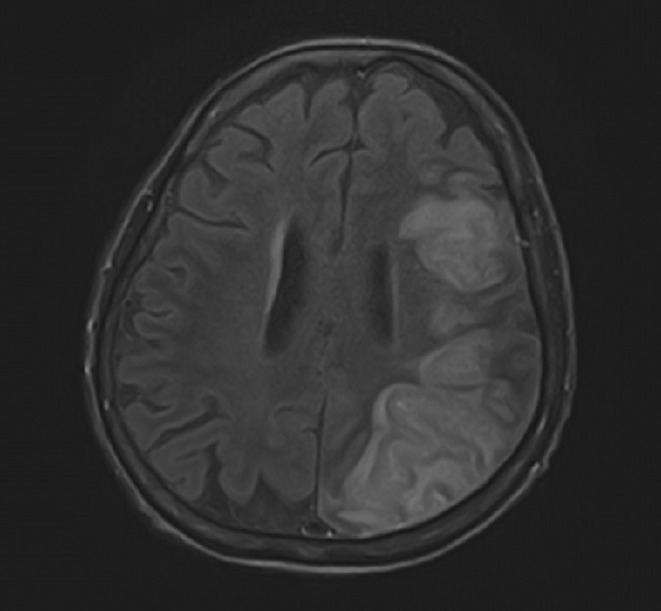
**FIGURE 1** MRI axial FLAIR image

**Figure:**
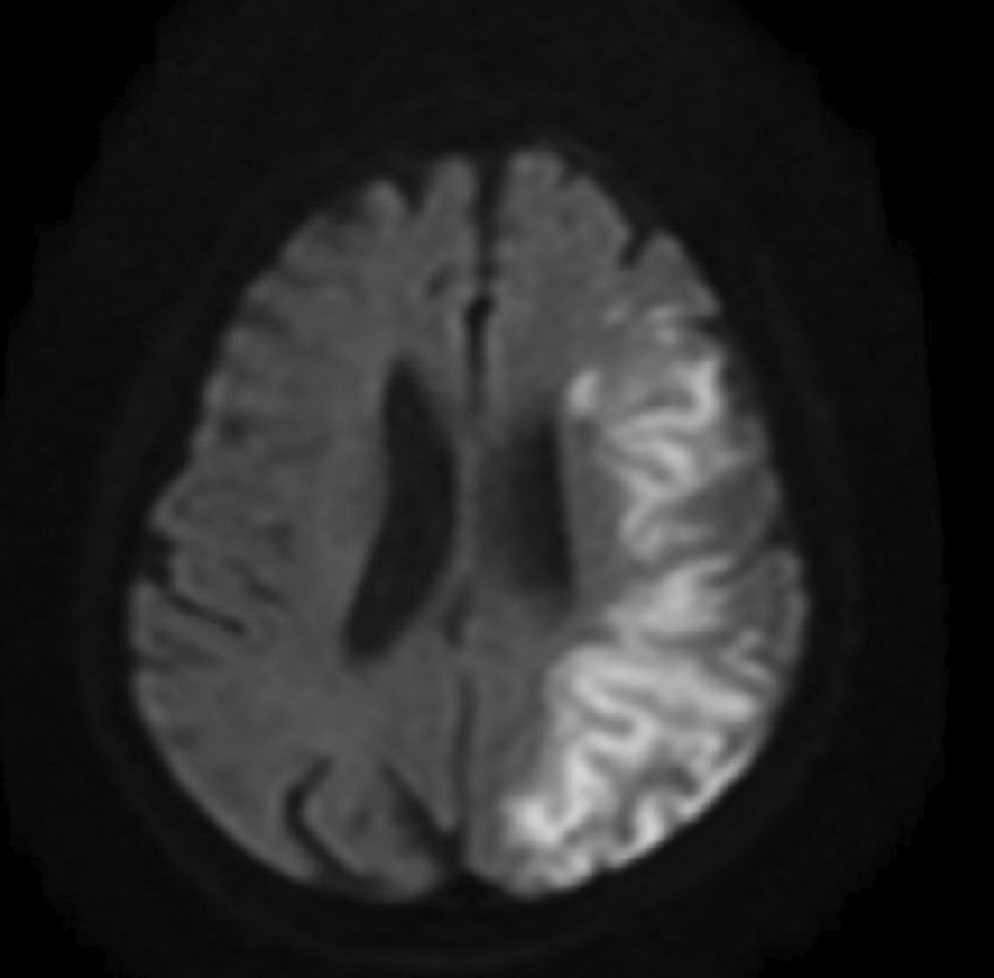
**FIGURE 2** MRI axial DWI image

**Figure:**
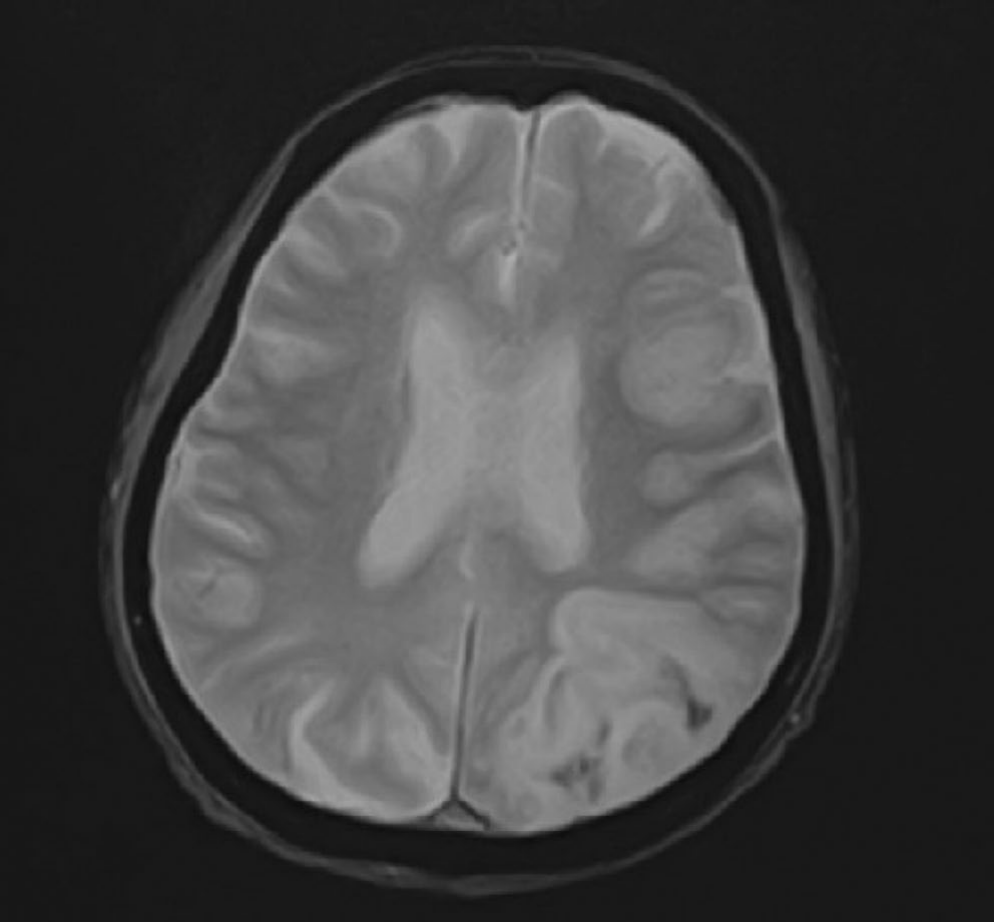
**FIGURE 3** MRI axial SWI image

**Results:** The patient was promptly initiated on antiviral therapy, in addition to antibiotics and supportive care. Despite these interventions, the patient's condition continued to deteriorate, showing a poor response to treatment, and she ultimately succumbed to the illness.

**Conclusion:** This case highlights the significant threat posed by HHV‐6 encephalitis, particularly in immunocompromised patients, underscoring the importance of early detection, comprehensive diagnostic workup, and timely therapeutic intervention. Although early diagnosis and treatment are critical, the prognosis may remain poor in cases with extensive neurological involvement.

**Disclosure:** Nothing to disclose.

## EPO‐015

### Heidenhain variant of Creutzfeldt‐Jakob disease: A diagnostic challenge in patients with visual disturbances

#### T. Fidan Çolak; B. Doğan; İ. Güngör

##### Department of Neurology, Faculty of Medicine, Ondokuz Mayıs University, Samsun, Turkey

**Background and aims:** Creutzfeldt‐Jakob disease (CJD) is a rare, fatal neurodegenerative disorder caused by misfolded prion proteins. The Heidenhain variant of CJD (HvCJD) is characterized by early visual disturbances, including visual impairment, altered color perception, and cortical blindness, often preceding cognitive and motor symptoms. This variant poses a diagnostic challenge, as patients typically seek ophthalmologic evaluation before a neurological diagnosis is made.

**Methods:** This report presents two cases of HvCJD with varying clinical manifestations. Both patients underwent cranial MRI, EEG, and CSF analysis, including testing for 14‐3‐3 protein. The diagnostic criteria of the World Health Organization for probable CJD were used.

**Figure:**
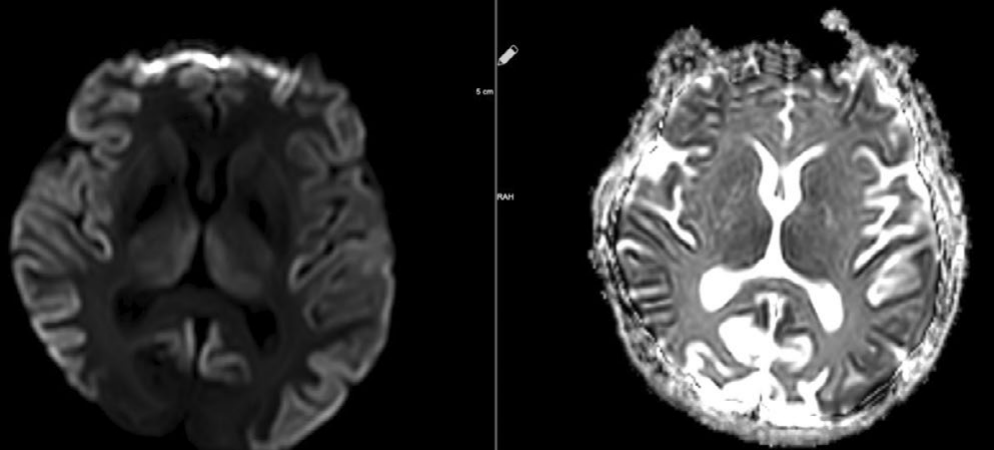
**FIGURE 1** Diffusion restrictions in the bilateral frontoparieto‐occipital lobes and bilateral thalami

**Figure:**
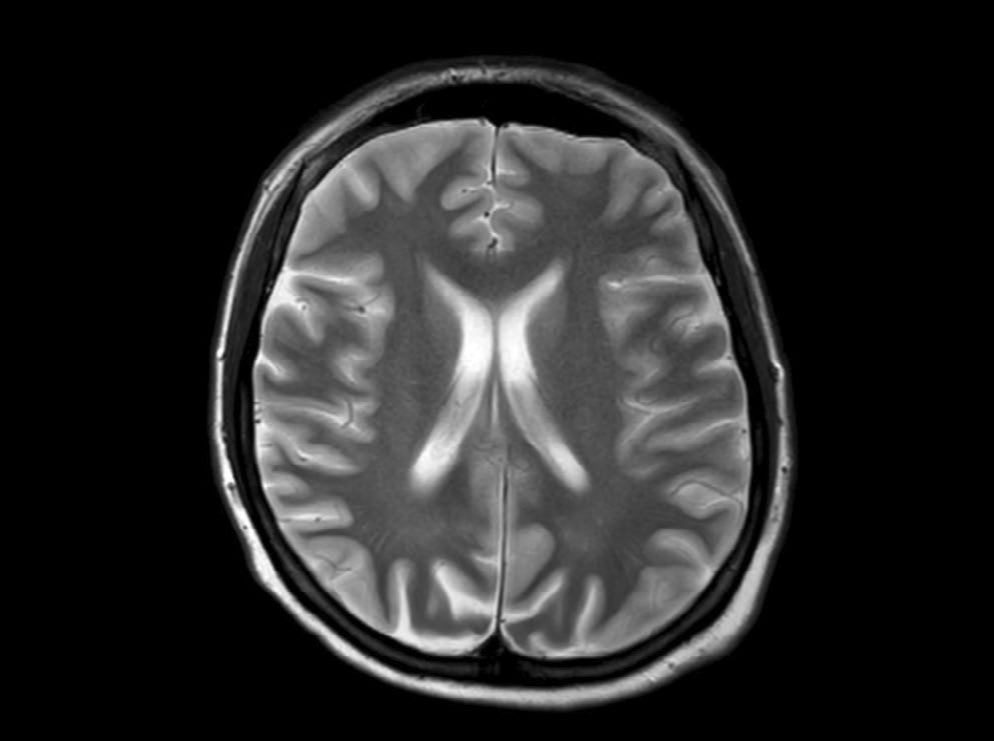
**FIGURE 2** FLAIR hyperintensities in the bilateral cortical surfaces of frontoparietooccipital lobes

**Figure:**
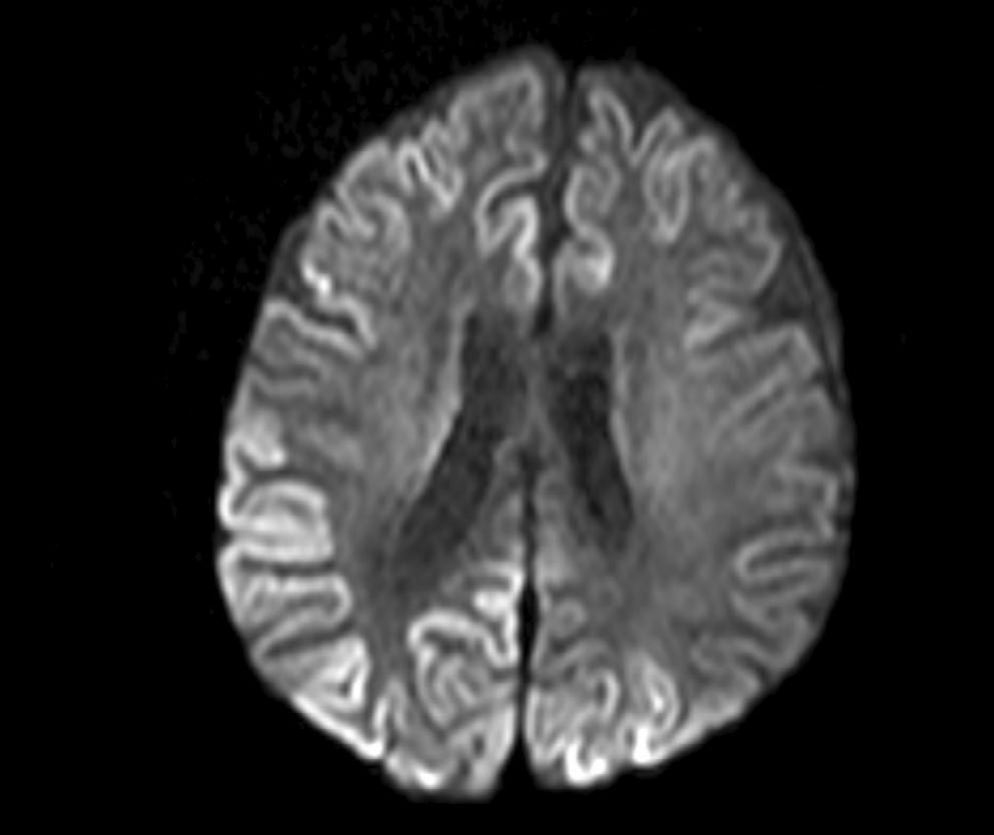
**FIGURE 3** Diffusion‐restricted signal changes in the bilateral frontoparietal and temporo‐occipital cortical surfaces, with more pronounced involvement in the right parieto‐occipital region

**Results:** The first case is a 35‐year‐old female with progressive visual impairment, behavioral changes, and cortical blindness, progressing to akinetic mutism. The second case is a 74‐year‐old male with visual impairment, ataxia, and rapidly progressive dementia. Both patients showed characteristic MRI, EEG, and CSF findings, supporting the diagnosis of probable CJD.

**Conclusion:** These cases emphasize the clinical heterogeneity of HvCJD and the importance of considering it in the differential diagnosis of unexplained visual disturbances and rapid neurological decline. Early recognition is critical to reduce diagnostic delays and the risk of prion disease transmission. Increased awareness among healthcare providers, especially ophthalmologists, is essential for timely diagnosis and management.

**Disclosure:** Nothing to disclose.

## Epilepsy 1

## EPO‐016

### Progression of negative myoclonus

#### A. Sinokki ^1^; L. Säisänen^2^; J. Hyppönen^2^; K. Silvennoinen^3^; R. Kälviäinen^4^; E. Mervaala^2^; P. Karjalainen^1^; S. Rissanen^1^

##### ^1^Department of Technical Physics, University of Eastern Finland, Kuopio, Finland; ^2^Kuopio Epilepsy Center, Department of Clinical Neurophysiology, Kuopio University Hospital, Full Member of ERN EpiCARE, Kuopio, Finland; ^3^Institute of Clinical Medicine, School of Medicine, Faculty of Health Sciences, University of Eastern Finland, Kuopio, Finland; ^4^Kuopio Epilepsy Center, Neurocenter, Kuopio University Hospital, Full Member of ERN EpiCARE, Kuopio, Finland

**Background and aims:** Negative myoclonus (NM) is characterized as a short absence (<500 ms) of muscle tone and is one of the main motor symptoms in progressive myoclonus epilepsies. NM may result in dropping items or falling. NM can be quantified by measuring silent periods (SP) in electromyography (EMG). The aim of the study is to determine whether progression of NM over one year can be detected using an EMG‐based analysis method.

**Methods:** One‐year follow‐up study was conducted for 18 progressive myoclonus epilepsy type 1 (EPM1) patients. SPs in EMG were detected from a 15‐minute segment from the beginning of Unified Myoclonus Rating Scale (UMRS) assessment. NM severity was rated using a standardized scoring system (0 = no NM, 3 = severe NM) during the UMRS assessment. Three EMG features were calculated: number of SPs, average SP duration, and ratio of SP to muscle activity.

**Figure:**
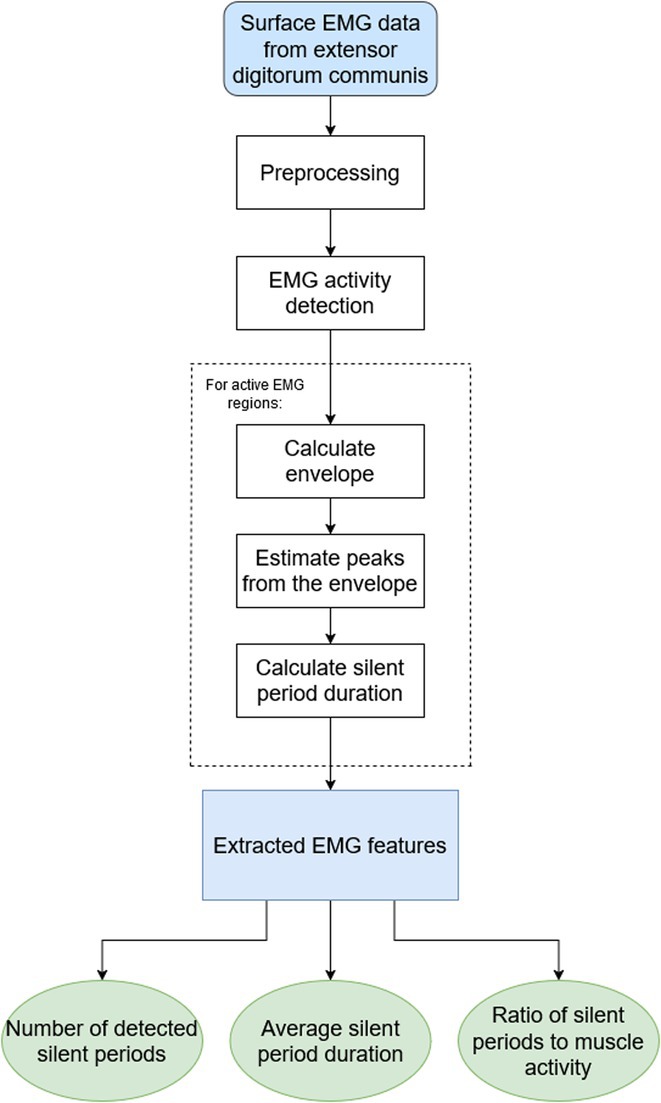
**FIGURE 1** Flowchart of the electromyography analysis method and silent period detection.

**Results:** NM severity increased in seven subjects. Among these, two showed an increase in EMG features, while others showed no significant change or a decrease. Subjects with higher NM severity (2 – 3) had higher ratio of SP to muscle activity on both visits. Nine patients scored as “no NM” had average SP duration below 100 ms in both assessments.

**Figure:**
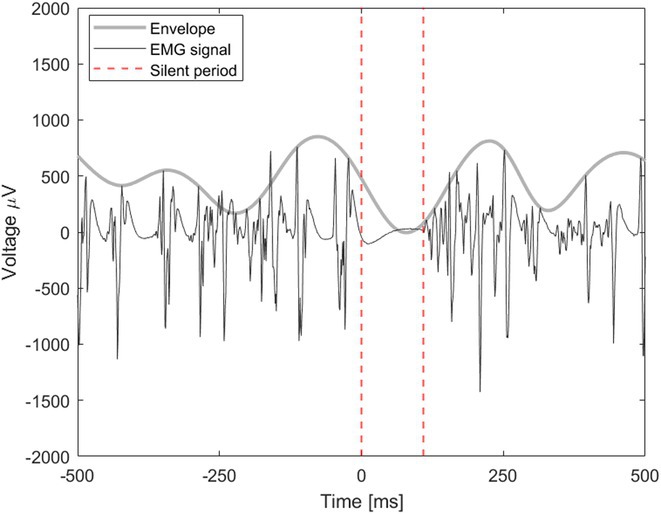
**FIGURE 2** Example of a silent period detected from electromyography signal (black), extracted envelope curve (gray), and silent period starting from t=0s.

**Conclusion:** Results suggest that one year may be too short time to observe meaningful NM progression. Additionally, the observed decrease in SPs may reflect natural fluctuations seen during short‐term evaluations such as UMRS. EMG‐based measurements during long‐term home monitoring would most probably improve reliability and provide greater clinical relevance.

**Disclosure:** A.S. Nothing to disclose. L.S. Nothing to disclose. J.H. Nothing to disclose. K.S. has received speaker's honorarium by Jazz Pharma. R.K. has received speaker's honoraria from Eisai, Jazz Pharmaceuticals, Orion, and UCB and honoraria for membership of the advisory boards/consultation of Angelini Pharma, Eisai, Jazz Pharmaceuticals, Orion and UCB. E.M. Nothing to disclose. P.A.K. is a co‐founder in Adamant Health Ltd. He is an inventor in patent applications WO2019166557A1 and WO2020174122A1. S.M.R. is a co‐founder in Adamant Health Ltd that develops EMG‐based analysis software. She is an inventor in patent applications WO2019166557A1 and WO2020174122A1.

## EPO‐017

### Treatment patterns in adult patients with focal‐onset seizures initiating cenobamate in USA

#### A. lovera ^1^; J. Leach^1^; E. Alvarez‐Baron^1^; P. Lipone^1^; C. Benoist^1^; R. Bosan^2^; E. Eworuke^2^; A. Comandini^1^; E. Salvatori^1^; A. Cattaneo^1^

##### ^1^Global Medical Department, Angelini Pharma S.p.A., Rome, Italy; ^2^IQVIA, Falls Church, USA

**Background and aims:** Antiseizure medications (ASMs) are a mainstay for patients with epilepsy. Cenobamate is a novel ASM indicated for adults with focal onset seizures (FOS). Real‐world data on the treatment patterns associated with cenobamate use will shed light on its efficacy and safety.

**Methods:** Retrospective analysis of IQVIA PharMetrics Plus database was conducted on US adult patients with FOS and a cenobamate claim (index date) between 1/4/2020 to 30/4/2023. Patients had a minimum of 6‐month continuous enrolment pre‐ and post‐index date. Patients were on cenobamate monotherapy (≥90 days prescription claims) or polytherapy (overlapping ≥90 days’ supply cenobamate and other ASMs). All other patients were categorized as undetermined. Then, a 90‐day window was used to determine patients that continued, switched, augmented (prescription for an additional ASM) or discontinued cenobamate regimen.

**Results:** Among the 1,132 patients included, 45.1% were on polytherapy, 1.5% on monotherapy, and 53.5% on undetermined regimen. In the polytherapy group, the largest proportion of patients were on cenobamate and 1 additional ASM (47%), followed by 2 additional ASMs (36%) and 3+ additional ASMs (17%). Most patients continued the identified treatment regimen (66.2%), did not augment (91.5%), and did not switch (97.7%). 11.2% discontinued cenobamate, 20.6% discontinued additional ASMs.

**Conclusion:** Most patients used cenobamate with only 1 additional ASM. Our study showed high patient adherence and minimal changes in treatment regimen (low rates of augmentation and switching). As seen here, cenobamate discontinuation rate was in line with the literature and lower than the one of co‐ASMs.

**Disclosure:** Angelini is the sponsor and funded IQVIA to conduct the study.

## EPO‐018

### Long‐term outcomes in patients with generalized tonic‐clonic seizures following VNS therapy: Interim CORE‐VNS 36 months

#### A. Suller‐Marti^1^; M. Keezer^2^; R. Verner^3^; A. Andrade^1^; M. Veilleux^4^; K. Myers^5^; J. Burneo^6^; G. Giannicola^3^; M. Dibue ^3^; P. Roncon^3^

##### ^1^Department of Clinical Neurological Sciences, Schulich School of Medicine and Dentistry, Western University, London, Ontario, Canada and Department of Pediatrics, Schulich School of Medicine and Dentistry, Western University, London, Ontario, Canada; ^2^Department of Neurosciences and School of Public Health, Université de Montréal, Montreal, Quebec, Canada; ^3^LivaNova PLC (or a subsidiary), Houston, USA; ^4^Department of Neurology and Neurosurgery, Montreal Neurological Institute and Hospital, Montreal, Quebec, Canada; ^5^Research Institute of the McGill University Medical Center, Montreal, Quebec, Canada; ^6^Department of Clinical Neurological Sciences, Schulich School of Medicine and Dentistry, Western University and Department of Epidemiology and Biostatistics, Schulich School of Medicine and Dentistry, Western University, London, Ontario, Canada

**Background and aims:** Generalized tonic‐clonic seizures (GTCs) are substantially devastating with considerable health risks. This study examines the long‐term outcomes of VNS in patients with GTCs.

**Methods:** Patients were enrolled into a prospective, multicenter observational registry called CORE‐VNS (NCT03529045). Participants with primary GTCs were selected for analysis (focal seizures at baseline and LGS were excluded). Selected study participants completed a 3‐month retrospective baseline period and after the VNS implant, were followed for up to 36 months.

**Results:** Fifty‐nine participants received an initial VNS implant, with 12 implanted within 5 years of epilepsy diagnosis and 47 after more than 5 years. Earlier implant recipients were younger (median age 9.7 vs. 25.9 years). Participants had previously failed a median of 6 antiseizure medications (ASMs). Among 40 participants completing 36 months of follow‐up, the responder rate (≥50% seizure reduction) was 70% (N=28; 95% CI: 56.0–81.7%), with a median seizure frequency change (MSFC) of ‐83.2% (95% CI: ‐100% to ‐53.3%). Earlier implant recipients demonstrated a higher responder rate (83.3% vs. 67.7%) and greater MSFC (‐94.3% vs. ‐76.4%). Quality of life improved for 27% of participants, regardless of implant timing. Adverse events were reported by 35.6% (N=21), with the most frequent being dysphonia (11.9%, N=7), dyspnea (6.8%, N=4), implant site pain (5.1%, N=3), and cough, oropharyngeal pain, and implant site infection (3.4%, N=2).

**Figure:**
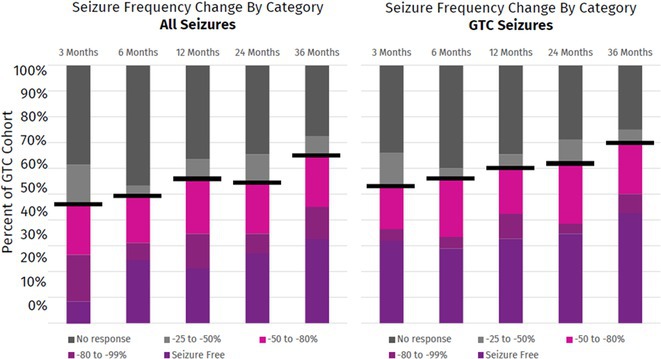
**FIGURE 1** Seizure Frequency Change by Category

**Figure:**
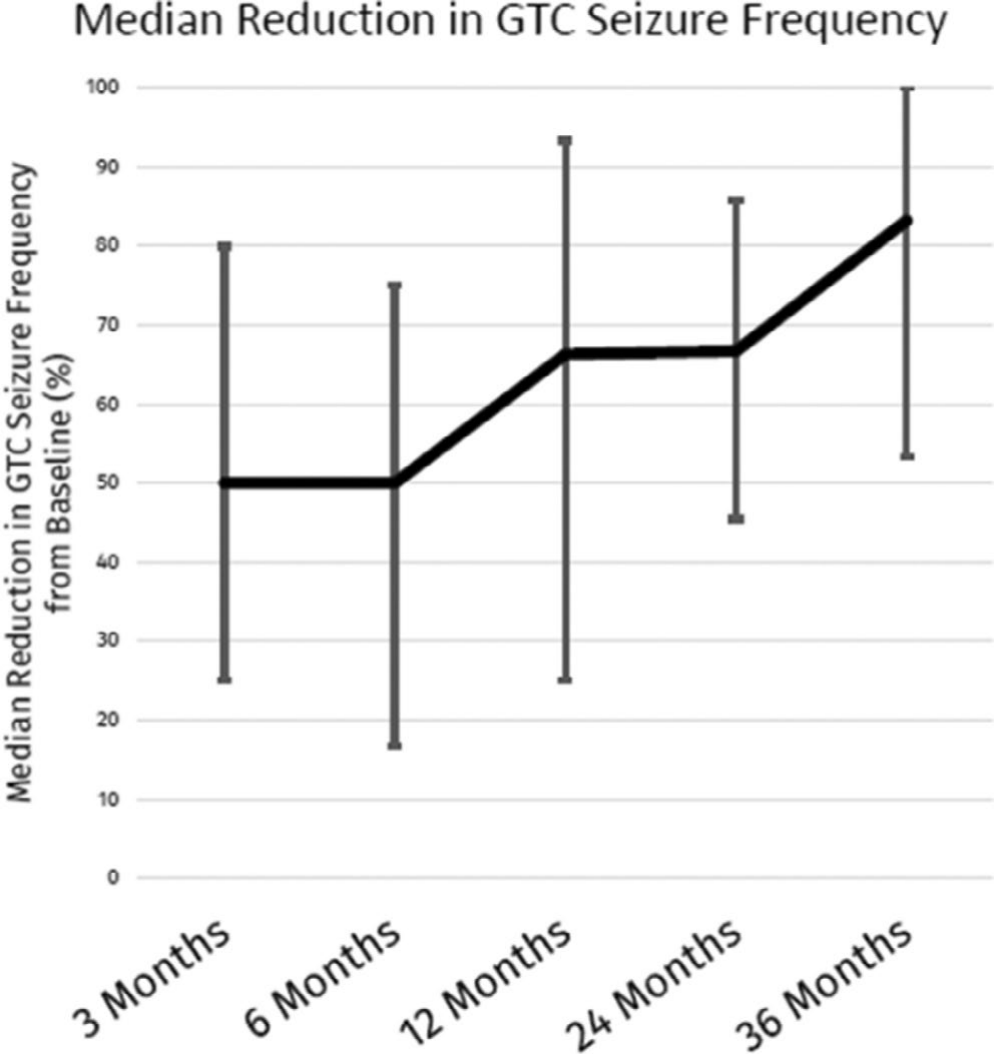
**FIGURE 2** Median Reduction in GTC Seizure Frequency

**Conclusion:** Adjunctive VNS was well‐tolerated and effective in reducing GTC frequency, with a 36‐month responder rate of 70% and an MSFC of up to ‐94.3%. These findings reflect the modern experience of VNS in GTC management.

**Disclosure:** RV, PR, MD, GG are employees of LivaNova PLC (or a subsidiary) and hold stock or stock options with the company, who is the manufacturer of the VNS Therapy System. All other authors are investigators associated with the CORE‐VNS Study, and in that capacity they or their institutions receive compensation from LivaNova for study‐related activities. No author received direct compensation from LivaNova related to this abstract.

## EPO‐019

### Prediction of short‐term outcome in non‐convulsive status epilepticus: External validation of the SACE score

#### C. Silvestri ^1^; G. Giovannini^3^; N. Orlandi^3^; L. Taruffi^2^; M. Burani^2^; M. Malerba^3^; A. Padvani^1^; S. Meletti^3^

##### ^1^Department of Continuity of Care and Frailty, U.O. Neurologia 2, University of Brescia, Brescia, Italy; ^2^Department of Biomedical Metabolic Sciences and Neurosciences, University of Modena and Reggio Emilia, Italy; ^3^Neurophysiology Unit and Epilepsy Centre, Azienda Ospedaliera‐Universitaria of Modena, Italy

**Background and aims:** Non‐convulsive status epilepticus (NCSE) lacks prominent motor symptoms, making initial diagnosis critical for improved outcomes. This study evaluated the SACE (Seizures, Age, Coma, EEG evolution) score in predicting NCSE outcomes and compared it with other prognostic tools (STESS, mSTESS, EMSE‐EACE). The primary outcome was 30‐day survival.

**Methods:** In a single‐center, retrospective study, 276 NCSE patients were analyzed from an initial cohort of 423. Bivariate logistic regression was used to identify which EEG patterns from Salzburg Consensus Criteria are significantly associated with in‐hospital and 30‐day survival. We tested performance of the SACE score in our NCSE population. Spearman's correlation coefficient examined the relationship between the SACE scores and the modified Rankin Scale (mRS) at 30 days.

**Results:** The cohort's mean age was 71.7 years (60% female), 27% of patients presented with coma at NCSE onset. Patients with EEG typical spatio‐temporal ictal (TSE) patterns (n=37) had higher survival rates (86.5% in‐hospital, 81.1% at 30 days) than those without (70.7% and 61.5%, respectively). The original SACE score demonstrated sensitivity of 0.508, specificity of 0.737, Youden's Index (YI) of 0.246, and AUC of 0.623. We developed a refined SACE score by adjusting parameter (sensitivity 0.39, specificity 0.758, YI 0.147, AUC 0.661) outperforming existing tools in predicting 30‐day outcomes.

**Figure:**
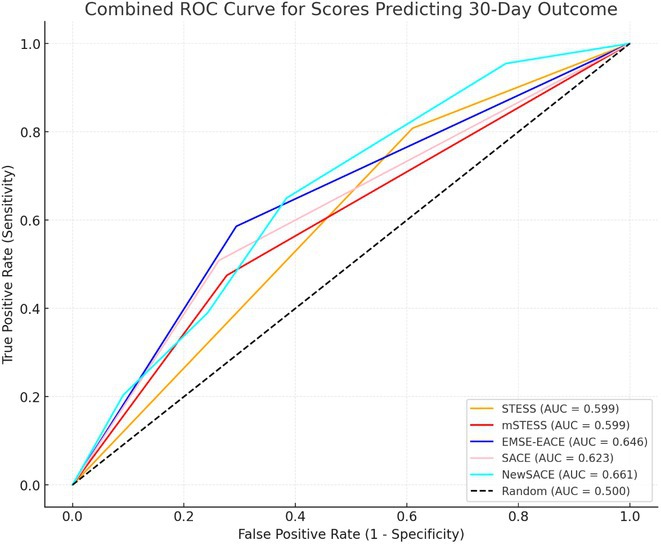
FIGURE 1

**Figure:**
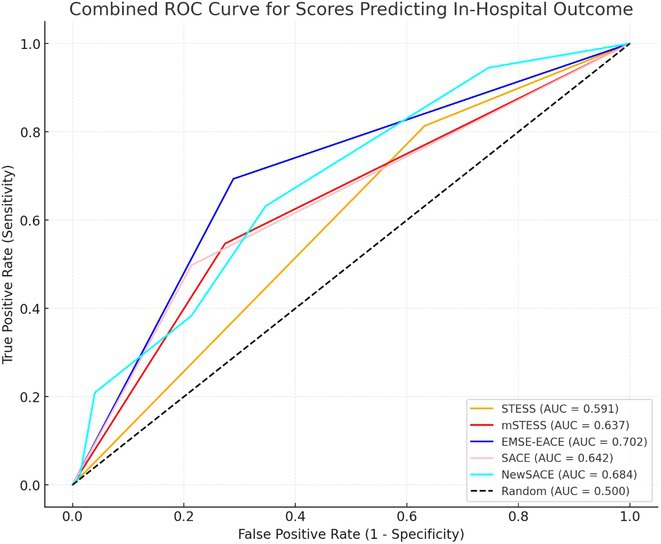
FIGURE 2

**Conclusion:** We confirmed that EEG TSE patterns predicted 30‐day survival. A refined SACE scores, which assigns two points for a history of seizures, showed a slightly better performance. A moderate correlation was identified between the SACE score and mRS 30‐day (Spearman correlation coefficient 0.508; p < 0.001).

**Disclosure:** Nothing to disclose.

## EPO‐020

### Perampanel as the only add‐on adjunctive therapy in highly active epilepsy

#### D. Tedeschi ^1^; A. Pascarella^1^; M. Pasquale^1^; D. Abelardo^1^; S. Gasparini^1^; O. Marsico^1^; R. Cutellè^1^; A. Santoro^1^; C. Mummolo^1^; V. Cianci^2^; U. Aguglia^1^; E. Ferlazzo^1^; PEROC Study Group^3^

##### ^1^Department of Medical and Surgical Sciences, Magna Græcia University of Catanzaro, Italy; ^2^Regional Epilepsy Center, Great Metropolitan “Bianchi‐Melacrino‐Morelli Hospital”, Reggio Calabria, Italy; ^3^Italy

**Background and aims:** People with highly frequent seizures often show resistance to therapy and poor prognoses, posing significant challenges for clinicians. Perampanel (PER) is a new generation antiseizure medication effective in focal and generalized seizures. This study aimed to evaluate the 12‐month effectiveness of PER as only add‐on treatment for people with highly active epilepsy in a real‐world setting.

**Methods:** Data from the Italian retrospective, longitudinal, multicenter perampanel as Only Concomitant Antiseizure Medication (PEROC) study were analyzed. Patients were grouped by baseline seizure frequency in a) <5, b) 5–20, and c) >20 seizures/month. Outcomes included PER retention, responder rates (>=50% seizure reduction), seizure‐free rates, and adverse events (AEs).

**Results:** The study included 485 patients: 354 with <5, 79 with 5–20, and 52 with >20 seizures/month at baseline, respectively. Retention rates at 12 months were 75.1%, 65.3%, and 58.1% respectively, without significant differences (p=0,077). Poor tolerability was the main reason for treatment withdrawal. At the 1‐year follow‐up, seizure frequency significantly decreased in all groups (p<0.001), with responder rates of 71.2%, 61.8%, and 63.2% in the three subgroups, showing no significantly differences between the groups. Seizure‐free rates were lower in patients with >20 (15.8%) and 5–20 seizures/month (23.5%) compared <5 seizures/month (49.5%; p=0.001). AEs, mainly dizziness, irritability, and drowsiness, occurred in 30% of patients, with similar rates across groups (p= 0.092).

**Figure:**
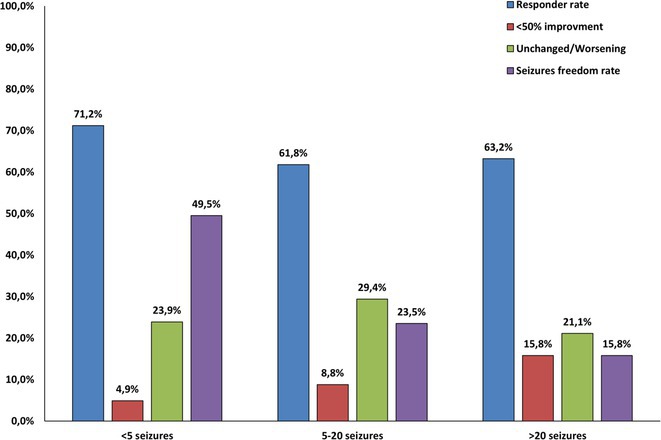
**FIGURE 1** Proportion of responder patients, patients achieving seizure freedom, patients with 20 per month)

**Conclusion:** PER demonstrated good efficacy and tolerability as only concomitant antiseizure medication in a real‐world setting in patients with highly active epilepsy, revealing a valuable treatment option in this population.

**Disclosure:** Nothing to disclose.

## EPO‐021

### What matters most to people living with epilepsy? A review of published qualitative research relating to health outcomes

#### J. Mitchell ^1^; J. Doherty^2^; R. Batchelor^3^; A. Noble^4^; P. Williamson^5^; A. Marson^1^

##### ^1^Institute of Systems, Molecular and Integrative Biology (ISMIB) University of Liverpool, Liverpool, UK; ^2^The Walton Centre NHS Foundation Trust, Liverpool, UK; ^3^The Oxford Institute of Clinical Psychology Training and Research, University of Oxford, Oxford, UK; ^4^Departments of Public Health, Policy and Systems, Institute of Population Health, Policy and Systems, University of Liverpool, Liverpool, UK; ^5^Department of Health Data Science, University of Liverpool, Liverpool, UK

**Background and aims:** There is a wealth of research examining the lived experience perspective from adults living with epilepsy across a range of geographical and cultural contexts. This review synthesises outcomes discussed by adults when asked about their lived experience.

**Methods:** MEDLINE was searched using an established qualitative methodological search filter. Studies reporting qualitative primary evidence of the views and experiences of adults living with epilepsy were included. Two independent researchers identified, screened and extracted data from relevant articles. Population and study characteristics were extracted in addition to verbatim quotes relevant to outcomes.

**Results:** Database searching returned 614 articles, of which 74 were included for outcome synthesis. The views of over 2474 people with epilepsy and 658 caregivers were identified (median 20 per study, range 4‐632) from 6 continents. Of included studies, 77% used in‐depth interviews, 12% used focus groups, 4% used a mixture of focus groups and in‐depth interviews, and 7% used mixed methods approaches. A total of 140 granular outcomes were coded from the included studies. ‘Social and role functioning’ and ‘emotional functioning’ outcomes were the most frequently coded, supporting the notion that living with epilepsy represents more than living with seizures.

**Figure:**
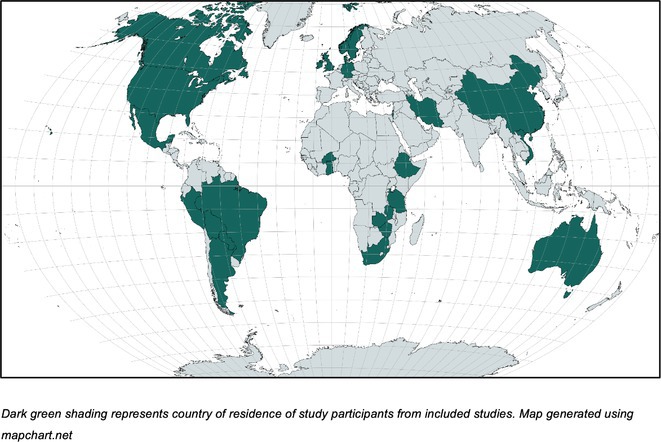
**FIGURE 1** Geographical location of adults with epilepsy in included qualitative studies

**Conclusion:** The results have wide‐reaching implications, providing new insights on what matters most to people living with epilepsy in a diverse range of geographic, societal and cultural settings. The results should be used to inform clinical practice, research, and inform which patient‐centric outcomes should be measured in future clinical trials.

**Disclosure:** Nothing to disclose.

## EPO‐022

### Safety, efficacy and pharmacokinetic analysis of intravenous VAL‐1221 treatment in Lafora disease

#### L. Muccioli; M. Tappatà; E. Esposito; A. Caravelli; C. Cancellerini; E. Pasini; R. Minardi; E. Pizzi; S. Mazzone; V. Carelli; L. Vignatelli; J. Fiori; R. Michelucci; F. Bisulli

##### IRCCS Istituto delle Scienze Neurologiche di Bologna‐Full member of the ERN EpiCARE, Bologna, Italy

**Background and aims:** Lafora Disease (LD) is a progressive myoclonus epilepsy with onset in previously healthy adolescents leading to death in young adulthood, with no effective treatments currently available. This study investigates the safety, efficacy and pharmacokinetics of intravenous VAL‐1221, the first potentially disease‐modifying treatment administered to LD patients.

**Methods:** Monocentric analysis of a 12‐month compassionate use program for VAL‐1221 (20 mg/kg every other week) in LD patients. Safety was assessed through adverse events (AE) monitoring. Efficacy was evaluated using clinical scales (including the Lafora disease clinical performance scale‐LDPS) and neuroimaging. Pharmacokinetic studies were performed using liquid chromatography‐high resolution mass spectrometry (LC‐HRMS) to analyze plasma and CSF samples.

**Results:** Five patients (range: 17‐24 years; 3 females) at intermediate to advanced stages of LD received VAL‐1221. Four patients completed the 12‐month treatment course, while one discontinued after 8 months due to status epilepticus. We observed 5 mild infusion‐related AEs (skin rash, n=1; hypotension, n=4) and no other treatment‐related AEs. Efficacy analyses showed a trend of deterioration from baseline to 12 months across various measures. The pharmacokinetic of VAL‐1221 was characterized by plasma concentrations (Cmax 100‐150 ug/mL; T1/2 60 min), but the drug was undetectable in CSF in all three assessed patients.

**Figure:**
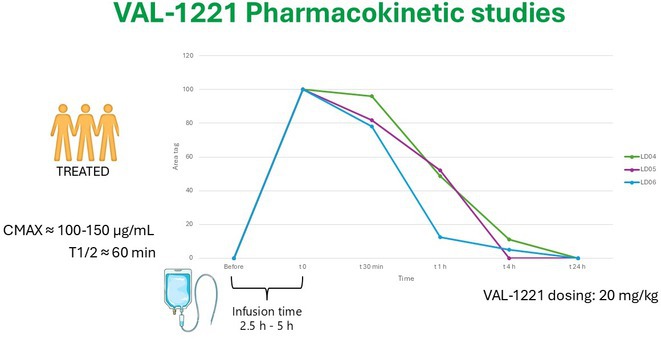
**FIGURE 1** VAL‐1221 pharmacokinetics

**Conclusion:** VAL‐1221 drug demonstrated an acceptable safety and tolerability profile. Evidence of disease progression and the lack of identification of the drug in CSF suggest that intravenous VAL‐1221 does not cross the blood‐brain barrier and is therefore ineffective for treating LD in this formulation. Further studies are needed to explore alternative administration methods.

**Disclosure:** Nothing to disclose.

## EPO‐023

### MOGAD spectrum and epilepsy: A review on age‐dependent radiologiacal and clinical profiles

#### M. Rubin ^1^; G. Cutillo^2^; V. Viti^3^; C. Zanetta^4^; M. Margoni^5^; P. Preziosa^1^; A. Bellini^6^; M. Rocca^1^; M. Filippi^7^

##### ^1^Neuroimaging Research Unit, Division of Neuroscience, and Neurology Unit, IRCCS San Raffaele Scientific Institute, and Vita‐Salute San Raffaele University, Milan, Italy; ^2^Neurology Unit, Neurorehabilitation Unit, Neurophysiology Service, IRCCS San Raffaele Scientific Institute, and Vita‐Salute San Raffaele University, Milan, Italy; ^3^Neurology Unit, Neurorehabilitation Unit, IRCCS San Raffaele Scientific Institute, and Vita‐Salute San Raffaele University, Milan, Italy; ^4^Neurology Unit, and Neurorehabilitation Unit, IRCCS San Raffaele Scientific Institute, Milan, Italy; ^5^Neuroimaging Research Unit, Division of Neuroscience, Neurology Unit, and Neurorehabilitation Unit, IRCCS San Raffaele Scientific Institute, Milan, Italy; ^6^Neurology Unit, Neurorehabilitation Unit, Neurophysiology Service, IRCCS San Raffaele Scientific Institute, Milan, Italy; ^7^Neurology Unit, Neurorehabilitation Unit, Neurophysiology Service, and Neuroimaging Research Unit, Division of Neuroscience, IRCCS San Raffaele Scientific Institute, and Vita‐Salute San Raffaele University, Milan, Italy

**Background and aims:** Anti‐MOG antibody‐associated disease (MOGAD) is a rare acute demyelinating syndrome presenting differently across lifespan. Albeit frequent, epilepsy remains a relatively understudied MOGAD‐related symptom. We aimed to characterize age‐dependent imaging, biohumoral and clinical signatures in MOGAD patients with epilepsy.

**Methods:** We systematically reviewed online repositories up to April 2024, selecting 126 case reports/series on MOGAD patients with seizures/epilepsy (Figure 1). Data were reported as percentages or medians with interquartile ranges, and comparisons were performed using Chi‐square and Mann‐Whitney U tests, as appropriate.

**Figure:**
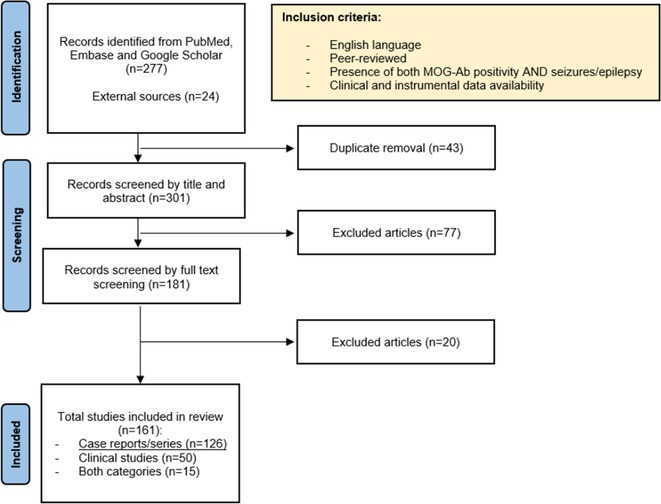
**FIGURE 1** Flowchart of the screening process. The keywords used for the searches were (“myelin oligodendrocyte glycoprotein” OR “MOG” OR “MOGAD”) AND (“epilepsy” OR “seizure”). Adapted from Page MJ et al, BMJ 2021.

**Results:** We included 273 MOGAD patients, 124 presenting with adult‐onset and 149 with pediatric‐onset disease. Pediatric‐onset patients were more often female (p=0.012), presented more commonly with acute demyelinating encephalomyelitis (p<0.001), and experienced encephalopathy more frequently (p=0.001). Adult‐onset patients more often presented with cortical encephalitis and typical MOGAD onset (p<0.007) and had higher cerebrospinal fluid pleiocytosis (p=0.005). Antibody co‐positivity was seen in 30% of patients, predominantly with anti‐NMDAR IgG (87%). MRI revealed no significant age‐related differences in temporal lobe involvement or leptomeningeal enhancement. Both groups exhibited epileptic manifestations primarily at disease onset, with no significant age‐related difference concerning generalized manifestations, but pediatric patients more likely experiencing status epilepticus (p=0.003). Despite no significant differences in immunotherapy, anti‐seizure medication (ASM) and ASM polytherapy use, pediatric‐onset patients showed higher rates of residual deficits and chronic epilepsy at follow‐up (p<0.007) (Figure 2).

**Figure:**
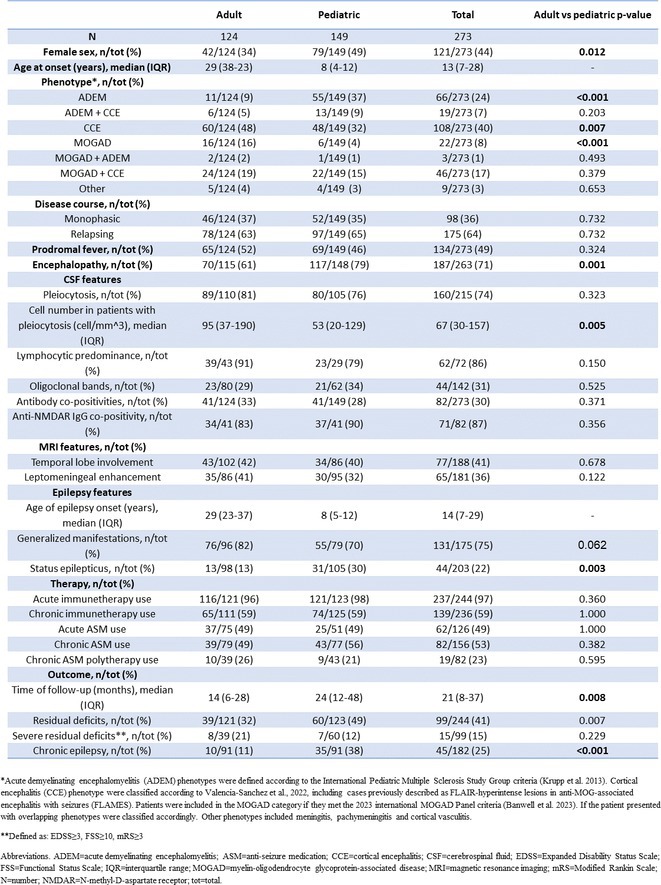
**FIGURE 2** Demographic, clinical, biohumoral, MRI and electroclinical characteristics in patients with MOGAD and epilepsy, according to age of onset. For each feature, analyses were performed on the total number of patients with available data.

**Conclusion:** Current literature addresses epilepsy as a significant comorbidity in MOGAD, with relevant rates of chronic progression. Significant age‐dependent differences in clinical presentation and outcomes underscore the importance of age‐specific approaches.

**Disclosure:** MR, GC, VV, AB nothing to disclose. CZ consulting or speaking fees from Alexion, Astrazeneca, Biogen, Bristol Myers Squibb, Janssen, Merck, Novartis, Roche, Sandoz, Sanofi. MM grants and personal fees from Sanofi Genzyme, Merck Serono, Roche, Biogen, Amgen and Novartis. PP speaker fees from Roche, Biogen, Novartis, Merck, Bristol Myers Squibb, Genzyme, Horizon and Sanofi; grants from Italian Ministry of Health (MSAL) and FISM. MF received compensation for consulting services or speaking activities from Alexion, Almirall, Bayer, Biogen, Celgene, Chiesi Italia SpA, Eli Lilly, Genzyme, Janssen, Merck‐Serono, Neopharmed Gentili, Novartis, Novo Nordisk, Roche, Sanofi Takeda, and TEVA; Advisory Boards for Alexion, Biogen, Bristol‐Myers Squibb, Merck, Novartis, Roche, Sanofi, Sanofi‐Aventis, Sanofi‐Genzyme, Takeda; scientific direction of educational events for Biogen, Merck, Roche, Celgene, Bristol‐Myers Squibb, Lilly, Novartis, Sanofi‐Genzyme; he receives research support from Biogen Idec, Merck‐Serono, Novartis, Roche, MSAL, the Italian Ministry of University and Research (MUR), and FISM. MAR received consulting fees from Biogen, Bristol Myers Squibb, Eli Lilly, Janssen, Roche, and speaker honoraria from AstraZaneca, Biogen, Bristol Myers Squibb, Bromatech, Celgene, Genzyme, Horizon Therapeutics Italy, Merck Serono SpA, Novartis, Roche, Sanofi and Teva, she receives research support from the MS Society of Canada, MSAL, MUR, and FISM.

## EPO‐024

### Changes in antiseizure medications and their serum concentrations during pregnancy in women: A real‐world experience

#### M. Trevisan ^1^; C. Silvestri^1^; A. Klyuchnik^1^; L. Purin^1^; E. Rolla^1^; M. Pasolini^2^; M. Tentorio^2^; P. Costa^2^; L. Broglio^2^; U. Leggio^2^; S. Gazzina^2^; A. Padovani^1^

##### ^1^Neurology Unit, Department of Clinical and Experimental Sciences, University of Brescia, Brescia, Italy; ^2^Neurophysiopathology Unit and Regional Centre for Epilepsy, Department of Continuity of Care and Frailty, ASST Spedali Civili di Brescia, Brescia, Italy

**Background and aims:** Pregnancy is known to impact on pharmacokinetics of antiseizure medications (ASMs) due to changes of absorption, distribution, metabolism and catabolism. This is particularly relevant for women with epilepsy (WWE), as these changes can contribute to worsening or recurrence of seizures and consequent risks for the fetus. We retrospectively evaluated the pharmacokinetics of ASMs and the clinical course of WWE over three years with focusing on how dosage variation affects the circulating drug levels.

**Methods:** 47 pregnant WWE with at least one visit to the Regional Epilepsy Center were considered. Data collected included demographics, epilepsy type, seizure frequency 9 months before and during pregnancy, ASMs dosage and serum concentrations. Complete data for all trimesters were available for 24 patients. The concentration‐to‐dose (C/D) ratio was calculated at four time points: before pregnancy and during each trimester. Repeated measures ANOVA was used to analyze the C/D ratio changes between the timepoints (before pregnancy vs each trimester).

**Results:** C/D ratio showed a mean significant decrease (‐38.5%) in the first trimester (p<0.05). The variation of C/D ratio in the first trimester for levetiracetam and lamotrigine was ‐20% and ‐52.2% respectively. Despite most of the patients underwent an increase in ASM dosage during pregnancy, a persistent reduction in the C/D ratio was observed (p<0.05). Nevertheless, seizure frequency did not increase.

**Conclusion:** Our study suggests that pregnancy induces changes in ASM concentrations, which are not fully mitigated by therapeutic adjustments. Nevertheless, seizure frequency appeared to be stable, suggesting that other biological factors may contribute to seizure control.

**Disclosure:** Nothing to disclose.

## EPO‐025

### The effect of the vigilance state on the epileptogenic network: An SEEG study

#### N. Biagioli ^1^; F. Pizzo^2^; A. Marchi^2^; L. Isabelle^2^; C. Benar^3^; F. Bartolomei^2^

##### ^1^Department of Neurology, University of Modena and Reggio Emilia, Modena, Italy; ^2^Service d’ Epileptologie et de Rythmologie Cérébrale, Hôpital de la Timone, Marseille, France; ^3^Dynamap, INS, APHM, Hôpital de la Timone, Marseille, France

**Background and aims:** Unlike interictal anomalies, it's unclear how sleep modulates seizures and thalamo‐cortical connectivity during seizures. This study aims to clarify how vigilances states influence the epileptogenic network, and its relations with thalamus during ictal/interictal periods

**Methods:** 36 patients with SEEG implantation presenting seizures during sleep (S) and wakefulness (W), characterized by low voltage fast activity pattern (LVFA) and thalamic electrode, were selected. EI was used to identify contacts belonging to 3 network zones (EZN, PZN, NIZ) and to assess S/W's epileptogenicity variations. Nonlinear correlation coefficient(h^2^) evaluated functional connectivity (FC) differences, comparing S/W, within zones and between zones and the thalamus. FC analysis was performed across four periods: Background (BG), Pre‐LVFA, LVFA, and Post‐LVFA, based on their relationship to rapid discharge.

**Figure:**
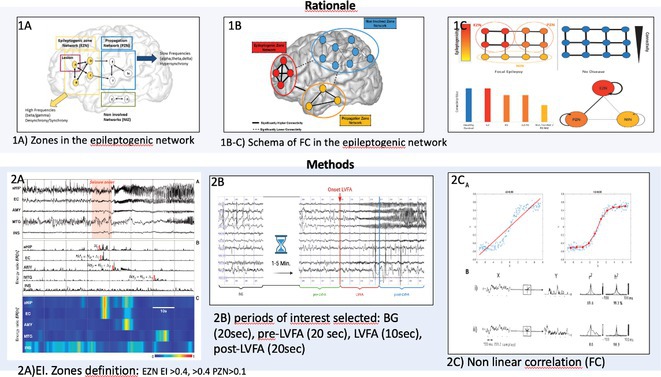
**FIGURE 1** Rationale: Schematic representation of the epileptogenic network and FC changes. Methods: representation of the various methods used (EI with cut‐off, periods of interest, and non‐linear correlation)

**Results:** No differences found in the number of contacts across the zones between S/W. During the preictal period, no FC differences were observed in EZN between S/W, a significant increase in FC during S was detected during BG and Pre‐LVFA in the PZN (p<0.001, p=0.028) and in the NIZ (p<0.001). Inter‐zone analysis revealed increased FC during sleep in BG and Pre‐LVFA across all comparisons performed (p<0.001). An increase in FC during S was observed only in BG for EZN‐thalamus and PZN‐thalamus (p=0.004, p=0.001) and in BG and Pre‐LVFA for NIZ‐thalamus (p<0.001, p<0.001). No FC differences between S/W were observed during ictal periods (LVFA and post‐LVFA).

**Figure:**
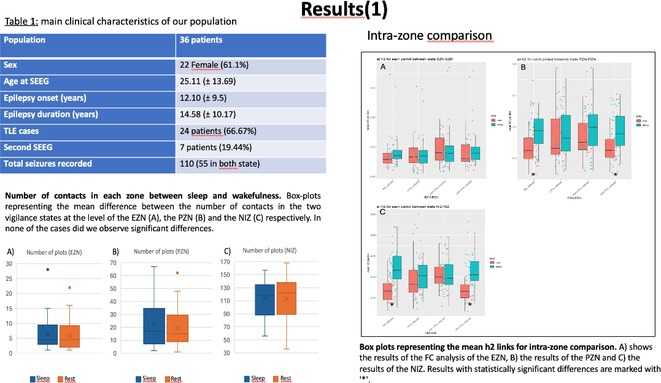
**FIGURE 2** Result (1): Table of population, number of plots and intrazone comparison of FC between S/W

**Figure:**
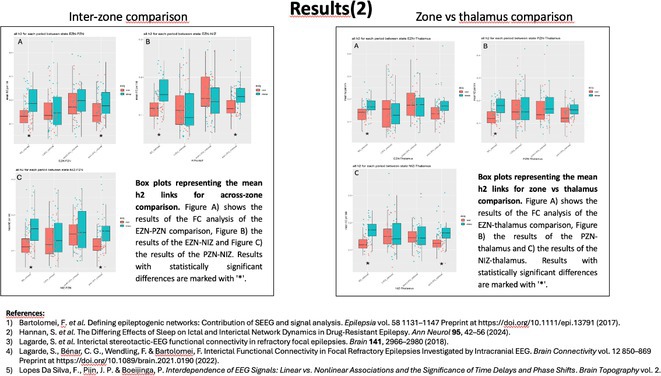
**FIGURE 3** Result (2): Inter‐zone and zones vs thalamus comparison of FC between S/W, bibliography

**Conclusion:** Sleep does not appear to modulate seizures. EZN exhibits a distinct FC profile that remains unaffected by vigilance state, unlike other zones. This may aid in differentiating it for pre‐surgical evaluation.

**Disclosure:** Nothing to disclose.

## EPO‐026

### Simultaneous SEEG and Scalp‐EEG for precise localization of epileptogenic zone in MRI‐negative temporal lobe epilepsy

#### N. Rast ^1^; M. Kovacevic^2^; S. Aull‐Watschinger^3^; J. Jud^1^; S. Bonelli^1^; C. Dorfer^4^; K. Rössler^4^; G. Kasprian^5^; E. Pataraia^1^

##### ^1^Department of Neurology, Medical University of Vienna, Vienna, Austria; ^2^Neurology Clinic, Clinical Center of Serbia, Belgrade, Serbia; ^3^Comprehensive Center for Clinical Neurosciences & Mental Health, Medical University of Vienna, Vienna, Austria; ^4^Department of Neurosurgery, Medical University of Vienna, Vienna, Austria; ^5^Department of Radiology, Division of Neuroradiology, Medical University of Vienna, Vienna, Austria

**Background and aims:** MRI‐negative focal epilepsy presents challenges in localizing the epileptogenic zone (EZ), crucial for achieving surgical seizure freedom. Simultaneous stereo‐electroencephalography (SEEG) and scalp‐electroencephalography (ScEEG) provide complementary insights by comparing ictal and interictal patterns, improving EZ delineation for diagnostic and therapeutic strategies.

**Methods:** This retrospective analysis included 10 adult patients with MRI‐negative drug‐resistant temporal lobe epilepsy (TLE) who underwent simultaneous SEEG and ScEEG at the Department of Neurology, Medical University of Vienna, between 2016 and 2023. SEEG electrodes were placed based on electroclinical hypotheses, and ScEEG according to the 10‐20 system. Seizures were classified based on ictal EEG latency of ScEEG and SEEG onset in: Group 1 (≤ 10 seconds), Group 2 (> 10 seconds), and Group 3 (no ScEEG changes). Seizure onset patterns (SOP) were categorized as ictal paroxysmal fast activity (iPFA), high‐amplitude polyspikes (HAP), or rhythmic sharp waves (rSW). Interictal epileptiform discharges (IEDs) were analyzed for morphology, amplitude, and distribution. The study was approved by the local Ethics Committee (EK 2161/2024).

**Results:** Median seizure onset latency was 6 seconds (range: 0–43). Group 1 SOPs showed iPFA and HAP (40.9%), Group 2 ‐ HAP (45.4%) and rSW (27.3%) and in Group 3 HAP and rSW was represented equally (44.4%). Scalp‐detected interictal populations were observed in 50% of SEEG‐recorded events, with sharp waves most frequent (33.3%). Interictal and ictal activity on deeper SEEG contacts rarely corresponded with ScEEG.

**Conclusion:** Deep epileptic discharges in MRI‐negative TLE often remain confined to SEEG. Combining SEEG and ScEEG enhances EZ delineation and supports optimized therapeutic planning.

**Disclosure:** Nothing to disclose.

## EPO‐027

### Unraveling the role of IL‐1β and PIP3 in epilepsy and sleep disturbances: Insights into the PI3K/AKT pathway

#### P. Yi ^1^; S. Huang^2^; F. Chang^2^

##### ^1^Department of Sport Management, Aletheia University, Taipei, Taiwan; ^2^Graduate Institute of Veterinary Medicine, National Taiwan University, Taipei, Taiwan

**Background and aims:** Epilepsy, a neurological disorder affecting 1% of the global population, is characterized by recurrent seizures and closely linked to sleep disturbances such as reduced sleep efficiency and sleep deprivation. These disturbances exacerbate seizure recurrence, creating a vicious cycle. The PI3K/AKT signaling pathway, critical for cellular survival and inflammation, has been implicated in epilepsy, with interleukin‐1β (IL‐1β) playing a central role. IL‐1β interacts with its receptor IL‐1R1, activating pathways such as NF‐κB, which are linked to seizures. However, the role of phosphatidylinositol‐3,4,5‐trisphosphate (PIP3) in this cascade and its connection to sleep disturbances remain unclear.

**Methods:** Using a pentylenetetrazol (PTZ)‐induced mouse model of epilepsy, we investigated the roles of IL‐1β signaling and PIP3. IL‐1R1 knockout mice were compared with wild‐type controls. Pharmacological inhibitors of the PI3K/AKT pathway were employed to study PIP3's function. Sleep parameters were assessed using polysomnography and behavioral analyses.

**Results:** IL‐1R1 knockout suppressed PIP3 expression and reduced NMDA receptor activity, mitigating epileptogenesis. Sleep disturbances, including fragmented sleep and reduced efficiency, were significantly improved by targeting IL‐1β signaling or inhibiting the PI3K/AKT pathway.

**Conclusion:** IL‐1β and PIP3 are pivotal in the PI3K/AKT pathway underlying epilepsy and associated sleep dysfunction. Suppression of IL‐1R1 or inhibition of the PI3K/AKT pathway not only mitigates seizures but also improves sleep disturbances. These findings highlight promising therapeutic strategies to address epilepsy and its comorbid sleep issues.

**Disclosure:** Nothing to disclose.

## EPO‐029

### From phenotype to genetics and back: A longitudinal study of monogenic developmental and epileptic encephalopathies

#### U. Costantino ^1^; O. Palumbo^2^; P. Palumbo^2^; M. Benvenuto^2^; M. Di Claudio^1^; F. Ciccone^1^; M. Carella^2^; G. D'Orsi^1^

##### ^1^Neurology Unit, Fondazione IRCCS‐Casa Sollievo della Sofferenza, San Giovanni Rotondo, Foggia, Italy; ^2^Division of Medical Genetics, Fondazione IRCCS Casa Sollievo della Sofferenza, San Giovanni Rotondo, Foggia, Italy

**Background and aims:** Developmental and epileptic encephalopathies (DEEs) are neurodevelopmental disorders characterized by early‐onset and drug‐resistant seizures, significant developmental delay or regression and distinctive EEG abnormalities. This study aimed to investigate the correlations between electro‐clinical phenotype and genetic findings and evaluate the natural history of persons with monogenic DEEs.

**Methods:** Between January 2023 and December 2024, 155 persons with DEEs underwent a comprehensive clinical‐instrumental study at the Neurology Unit of the Fondazione IRCCS Casa Sollievo della Sofferenza, San Giovanni Rotondo (FG), Italy, including detailed epilepsy history, 24‐hour video electroencephalography, neuropsychological assessment, brain 3T MRI and genetic analysis.

**Results:** The mean age at the time of genetic diagnosis was 6.26 years. NGS sequencing identified a genetic etiology in 61 patients, while SNP‐arrays analysis in 13. Pathogenic or likely pathogenic variants were identified in genes with an autosomal dominant inheritance pattern in 73.3% of patients, X‐linked inheritance in 6.6% and autosomal recessive inheritance in 20%. The most frequently reported genes were SCN1A (20%), followed by TSC1 (8%) and CACNA1A (5%). MRI findings included cortical atrophy in 15 patients, cerebellar atrophy in 8 and focal lesions in 13. Neuropsychological assessments demonstrated severe intellectual disability in 34.7% of patients. In 37% of patients, 24‐hour video electroencephalography revealed specific seizure types (e.g. “tonic seizures” in 7 patients) and facilitated the optimization of antiseizures medications.

**Figure:**
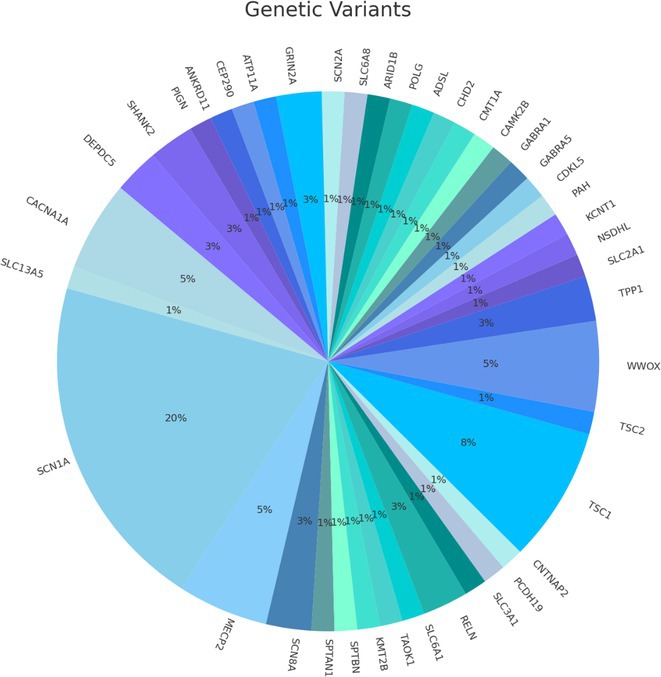
**FIGURE 1** An overview of gene's variants identified.

**Figure:**
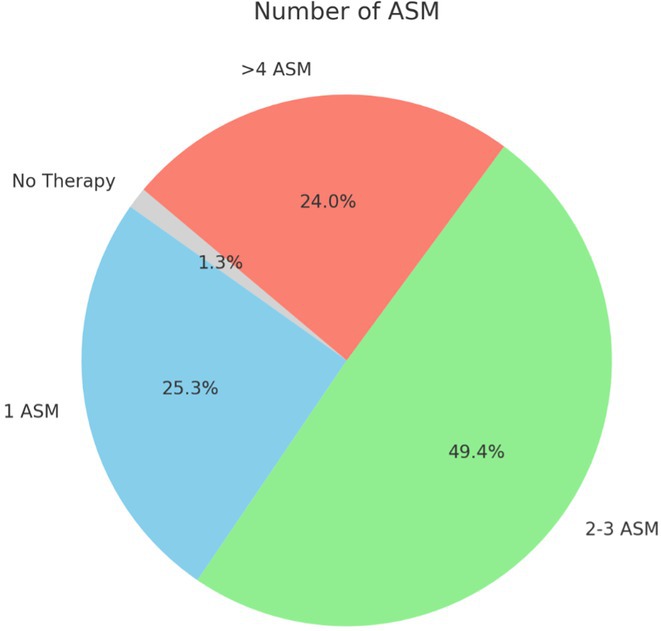
**FIGURE 2** Percentage of drug resistant epilepsy.

**Conclusion:** This longitudinal study highlights the crucial role of phenotype and genetic testing in the diagnostic workup of DEEs. Molecular diagnosis facilitated personalized treatments and improved our understanding of the underlying pathophysiology in a significant proportion of patients.

**Disclosure:** Nothing to disclose.

## EPO‐030

### Efficacy and tolerability of Cenobamate: Real‐world experience from a tertiary Italian centre

#### V. Viola ^1^; L. Muccioli^3^; L. Ferri^2^; L. Licchetta^2^; G. Bruschi^1^; R. Esposto^2^; L. Di Vito^2^; B. Mostacci^2^; F. Bisulli^3^

##### ^1^Department of Biomedical and Neuromotor Sciences, University of Bologna, Bologna, Italy; ^2^IRCCS Istituto delle Scienze Neurologiche di Bologna, Full Member of the European Reference Network for Rare and Complex Epilepsies (EpiCARE), Bologna, Italy, Department of Biomedical and Neuromotor Sciences, University of Bologna, Bologna, Italy; ^3^Department of Biomedical and Neuromotor Sciences, University of Bologna, Bologna, Italy; IRCCS Istituto delle Scienze Neurologiche di Bologna, Full Member of the European Reference Network for Rare and Complex Epilepsies (EpiCARE), Bologna, Italy

**Background and aims:** To evaluate real‐world efficacy and tolerability of cenobamate (CNB) in patients with drug‐resistant epilepsy (DRE) in an Italian tertiary hospital.

**Methods:** We conducted a single‐centre, retrospective, observational study collecting data from clinical records at Bellaria Hospital. Inclusion criteria were age ≥18 years, focal seizures and DRE. Primary effectiveness endpoints included seizure reductions (≥50‐80%, ≥80.1‐99%, 100%) or worsening at 3, 6 and 12 month visits. Safety endpoints included rates of adverse events (AEs) at 3, 6 and 12 month visits.

**Results:** The study included 96 patients with DRE, of whom 13.5% had developmental and epileptic encephalopathy (DEE). Etiology was known in 69% (11.5% genetic, 9.4% vascular malformation, 3% autoimmune, 3% infectious, 36.5% structural, 5% genetic and structural). Median number of prior and concomitant antiseizure medications (ASMs) were 6 and 2.5, respectively. Retention rates were 95%, 92% and 88.7%, and mean CNB dosages/day were 112.76 mg, 149.08 mg and 180.95 at 3, 6 and 12 months, respectively. At the last available visit, 28.6%, 20.6% and 15.9% had response rates of ≥50‐80%, ≥80.1‐99% and 100%, 17.5% did not respond and a further 17.5% worsened. At baseline, median epilepsy duration was 25.95 years and median number of seizures/month was 12.50, with a significant reduction (P < 0.001) at all timepoints. The cumulative percentages of patients with AEs and AEs leading to discontinuation were 46%, 39.6% and 38.7% at 3, 6 and 12 months.

**Conclusion:** In this highly refractory population, CNB demonstrated good efficacy regardless of etiology, prior and concomitant ASMs. AEs were frequent but mostly mild‐to‐moderate.

**Disclosure:** Nothing to disclose.

## Cerebrovascular Diseases 1

## EPO‐031

### Acute kidney injury in patients with spontaneous intracerebral hemorrhage – Is it a real problem?

#### A. Golenia ^1^; K. Ludwiniak^2^; O. Maciejewska^2^; P. Olejnik^1^; A. Opuchlik^1^; J. Małyszko^3^

##### ^1^Department of Neurology, Medical University of Warsaw, Poland; ^2^Department of Neurology, University Clinical Center, Poland; ^3^Department of Nephrology, Dialysis and Internal Medicine, Medical University of Warsaw, Poland

**Background and aims:** Acute kidney injury (AKI) is common in critically ill intensive care unit patients, including those with intracerebral hemorrhage (ICH). Spontaneous ICH accounts for 10‐15% of all strokes and is the leading cause of death and long‐term disability in people over 40 years of age worldwide, and the development of AKI in these patients further worsens their outcomes. The aim of the study was to determine the incidence and risk factors for AKI, as well as short‐term outcomes in patients with spontaneous ICH.

**Methods:** In this single‐center study, we retrospectively analyzed the data of consecutive patients diagnosed with spontaneous ICH.

**Results:** Of the 237 patients with spontaneous ICH included in the study, 13.5% of patients developed AKI. Risk factors for AKI were: severity of neurological deficit as measured by the National Institutes of Health Stroke Scale (NIHSS), larger hematoma volume, as well as higher baseline mean systolic and diastolic blood pressure. Furthermore, patients who developed AKI had higher levels of serum glucose, urea, serum creatinine and lower estimated glomerular filtration rate (eGFR) on hospital admission. In addition, the overall and in‐hospital mortality were much higher in patients with AKI than in those without AKI. Finally, adjusted regression analysis identified the in‐hospital use of nephrotoxic antibiotics as a major risk factor, increasing the likelihood of AKI by eightfold.

**Conclusion:** These findings highlight the importance of early identification of high‐risk patients and careful management of nephrotoxic agents to reduce the incidence and adverse outcomes of AKI in ICH patients.

**Disclosure:** Nothing to disclose.

## EPO‐032

### Complex post‐closure hallucinations as a sign of posterior callosal stroke: A case‐report

#### A. Sarnataro

##### Department of Neuroscience, Reproductive and Odontostomatological Sciences, University of Naples, Italy

**Background and aims:** Visual hallucinations triggered by eye closure are a very rare finding in clinical practice. Underlying pathomechanisms include cortex irritation and cortical release phenomenon caused by suppression of inhibitory cortical input to visual association areas.

**Methods:** We describe the case of a 77‐year‐old female with transient visual hallucinations at closed eyes after a posterior ischemic stroke due to basilar artery occlusion.

**Results:** At admission the patient presented with altered consciousness state and NIHSS scored 23 points. CT‐Angiography showed basilar artery occlusion, and patient underwent thrombectomy. The following day neurological examination was dramatically improved (NIHSS 0), and the patient complained only of complex visual hallucinations occurring at closed eyes. CT‐scan and EEG were negative while brain MRI (6th day) showed DWI restriction in splenium of corpus callosum, and FDG‐PET (8th day) revealed hypometabolism in right temporo‐occipital area. On day 10, visual hallucinations disappeared, and the patient was discharged.

**Conclusion:** Splenium of Corpus callosum is involved in integration network mainly for occipital cortex. Posterior callosal stroke might result in right temporo‐occipital area hypometabolism suggesting that closed‐eye hallucinations may be related to loss of inhibitory cortical input to visual association areas as described in Charles Bonnet Syndrome.

**Disclosure:** Nothing to disclose.

## EPO‐033

### Terson syndrome secondary to cerebral intraparenchymal haemorrhage

#### A. Montalvo ^1^; M. M. Roque^1^; S. Mano^2^; P. Nascimento Alves^3^; A. Fonseca^3^; T. Pinho e Melo^1^

##### ^1^Stroke Unit, Neurology Service, Department of Neuroscience and Mental Health, Local Health Unit of Santa Maria, Lisbon, Portugal; ^2^Ophthalmology Service, Local Health Unit of Santa Maria, Lisbon, Portugal; ^3^Faculty of Medicine, University of Lisbon, Lisbon, Portugal

**Background and aims:** Terson syndrome was first described as a vitreous haemorrhage secondary to subarachnoid haemorrhage (SAH). It is associated with worse prognosis in these patients. More recently, intracerebral haemorrhage and neoplasms have also been associated with this syndrome.

**Methods:** Case report.

**Results:** A 28‐year‐old male was admitted due to an explosive headache with nausea, vomiting and, 10 minutes later, altered mental status. He was sedated and intubated due to inefficient respiratory pattern in the pre‐hospital setting. Neurological examination showed absence of eye opening, anisocoria due to right mydriasis, unreactive pupils, motor response with flexion of the right upper limb and pathologic extension of the left upper limb (Glasgow Coma Scale 6). He had previous history of amblyopia secondary to congenital periocular hemangioma and strabismus, surgically corrected during childhood. Head‐CT showed a right temporal hematoma with significant mass effect. Decompressive craniectomy and hematoma evacuation were performed. Aetiological investigation, including analytical evaluation, and digital cerebral angiography were negative. Brain MRI showed a small haemorrhage on the posterior surface of the right eye. Ophtalmologic evaluation confirmed vitreous haemorrhage. There was progressive clinical improvement. At discharge, the patient presented left hemiparesis and was autonomous in activities of daily living (mRankin 2).

**Conclusion:** In this case, the acute intracerebral haemorrhage was responsible for a rapid increase in intracranial pressure that extended to the optic nerve sheath leading to vitreous haemorrhage. There was no impact on visual acuity, possibly due to previous amblyopia. In this case, MRI was essential for diagnosis the vitreous haemorrhage.

**Disclosure:** Nothing to disclose.

## EPO‐034

### Neglecting neglect: Optimising FASTED assessment for Prehospital LVO‐stroke prediction

#### C. Ip ^1,2^; A. Toleti^1^; R. Simister^1,2^

##### ^1^National Hospital for Neurology and Neurosurgery, London, UK,^2^ Clinical Video Assessment for Stroke (CVAS) Project Group

**Background and aims:** FAST‐ED is a validated screening tool for large vessel occlusion (LVO) ischaemic stroke detection. The London Prehospital Video Triage (PVT) service enables stroke specialists to assist in on‐scene assessment, but examining neglect via video remains challenging and may introduce delays. We evaluate the utility of neglect within FAST‐ED score.

**Methods:** We retrospectively analysed patients assessed via PVT between January and June 2023 and admitted to the National Hospital for Neurology and Neurosurgery. FAST‐ED's accuracy for LVO prediction was compared with two alternative models: neglect field excluded, and double weighting eye‐deviation. LVO was defined based on thrombectomy eligibility, including M1, proximal M2, P1, A1, and basilar occlusions.

**Results:** 424 patients were analysed: including 52 LVO stroke, 177 non‐LVO stroke, and 125 non‐strokes. Conventional FAST‐ED≥4 as LVO prediction demonstrated sensitivity 0.65 and specificity 0.86 among stroke patients, and specificity 0.89 among all patients. Mimics with FAST‐ED≥4 included functional (3), seizures related to sepsis (2), new mass lesion (1) and hyperglycaemia (1). If only MCA‐implicated LVOs are considered, FAST‐ED≥4 showed improved sensitivity of 0.74. Full FAST‐ED, FAST‐ED with neglect excluded, and FAST‐ED with neglect excluded while eye‐deviation score doubled, all showed similar predictive accuracies (AUC of 0.82, 0.82, and 0.81 respectively). For patients with confirmed neglect on admission, 69% were missed on PVT assessment.

**Figure:**
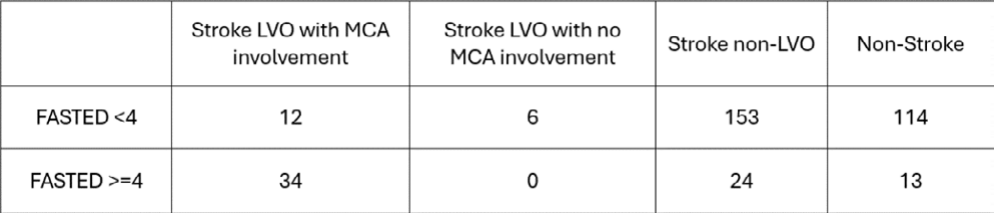
**TABLE 1** Case numbers for various final diagnosis groups and their FASTED score.

**Figure:**
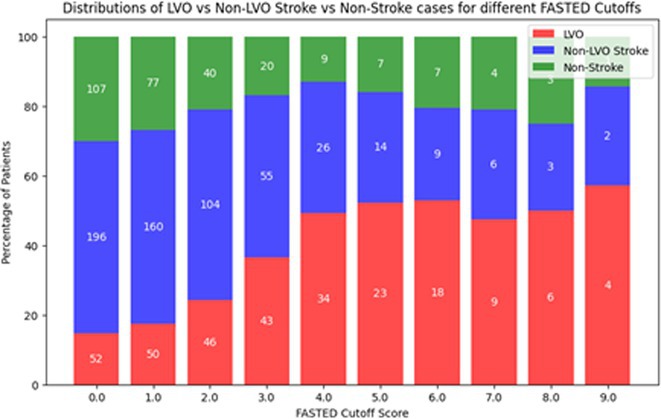
**FIGURE 1** Distribution of cases for various FASTED cut‐off scores

**Figure:**
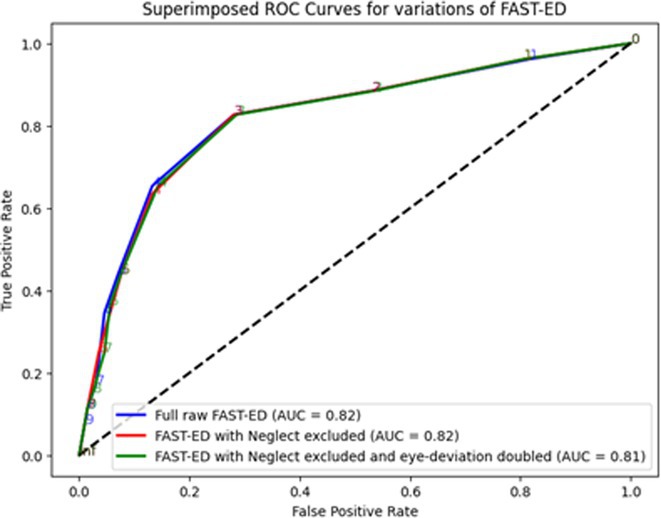
**FIGURE 2** Superimposed ROC curves for variations of FASTED scores

**Conclusion:** FAST‐ED is an effective tool for LVO identification, particularly for MCA LVO. Excluding neglect assessment does not significantly impact its accuracy. Specialist assessment via PVT would enhance the exclusion of clear non‐stroke with high FAST‐ED, and inclusion of non‐MCA LVO.

**Disclosure:** Nothing to disclose.

## EPO‐035

### NOTCH3 protein EGFr domain and white matter lesions effects on brain volume in CADASIL patients

#### E. Zacarias ^1^; N. Rifino^1^; M. Stanziano^2^; C. De Toma^1^; I. Canavero^1^; B. Storti^1^; A. Francia^1^; G. Boncoraglio^1^; G. Marinoni^1^; C. Strazzabosco^1^; A. Bersano^1^

##### ^1^Cerebrovascular Unit, Fondazione IRCSS Istituto Neurologico Carlo Besta, Milan, Italy; ^2^Neuroradiology Unit, Fondazione IRCCS Istituto Neurologico Carlo Besta, Milan, Italy

**Background and aims:** Epidermal growth factor‐like repeat domains (EGFrs) of the NOTCH3 protein have been classified into three risk groups that may predict disease severity in cerebral autosomal dominant arteriopathy with subcortical infarcts and leukoencephalopathy (CADASIL) patients. Extensive white matter hyperintensities (WMHs) in CADASIL patients are associated with increased brain volume. Still, no correlation has been explored when considering the EGFr risk category.

**Methods:** 70 CADASIL patients were classified into three risk groups with similar mean age according to the NOTCH3 EGFr domain altered: 26 high‐risk, 23 medium‐risk, and 21 low‐risk. Normalized brain volume (brain parenchyma volume/intracranial cavity volume) and normalized white matter (WM) lesion volume (WM lesion volume/intracranial cavity volume) were calculated using VolBrain DeepLesionBrain from volumetric T1w and FLAIR brain MRI sequences. A multivariate linear regression model was used to model the relationship between normalized brain volume and normalized WM lesion volume, considering the effect of EGFr risk category, age and sex.

**Results:** The regression analysis showed that brain volume significantly decreases with increasing age (p = 0.001), while it increases in individuals with higher EGFr risk categories (p = 0.010) and greater WM lesion volumes (p < 0.001). Sex does not have a significant effect (p = 0.298).

**Conclusion:** Our study confirms previous reports that greater WMH volumes are associated with increased brain volume in CADASIL patients, which may be caused by an overall increase in water content within cerebral tissue despite the loss of white matter components, while adding the EGFr risk category as another factor that seems to influence this correlation.

**Disclosure:** Nothing to disclose.

## EPO‐036

### Does emergency conversion to general anesthesia during mechanical thrombectomy increase the risk of poor outcome?

#### F. Kuris^1^; M. Valente ^1^; G. Gigli^1^; P. Paone^2^; D. Alimonti^2^; P. Gritti^3^; L. Longhi^3^; M. Passoni^3^; C. Deana^4^; L. D'Anna^5^; G. Merlino^1^

##### ^1^Clinical Neurology Unit and Stroke Unit, DMED, Udine, Italy; ^2^Neurology Unit, Bergamo, Italy; ^3^Neurointensive Care Unit, Bergamo, Italy; ^4^Department of Anaesthesia and Intensive Care Health Integrated, Udine, Italy; ^5^Department of Stroke and Neuroscience, Charing Cross Hospital, London, UK

**Background and aims:** The consequences of emergency conversion (EC) from non‐general anesthesia (non‐GA) to general anesthesia (GA) during mechanical thrombectomy (MT) are unknown. This study aimed to explore the functional outcomes of patients who underwent EC during MT.

**Methods:** We included consecutive patients with anterior large vessel occlusion and pre‐mRS ≤2 treated with MT in 3 thrombectomy capable centers between January 2022 and December 2023. Inverse probability weighting (IPW) reduced bias by indicating the anesthesia type on study outcomes. We used a weighted ordinal robust logistic regression analysis to explore the primary outcome of modified Rankin Scale (mRS) shift at 90 days in EC versus GA versus non‐GA. Secondary outcomes included 90‐day poor outcome, defined as an mRS >2, 90‐day mortality, symptomatic intracranial hemorrhage (sICH), and successful recanalization.

**Results:** We included 669 patients: 395 underwent GA, 189 non‐GA, and 85 EC. There was no significant shift for worse mRS scores at 90 days in EC versus GA (cOR 1.17, 95% CI 0.97‐1.41; p =0.091) and versus non‐GA (cOR 0.88, 95% CI 0.72‐1.07, p =0.201). Secondary outcomes were not different among the three groups, but the GA technique was an independent predictor of successful recanalization (aOR 1.90, 95% CI 1.30–2.80; p = 0.001).

**Figure:**
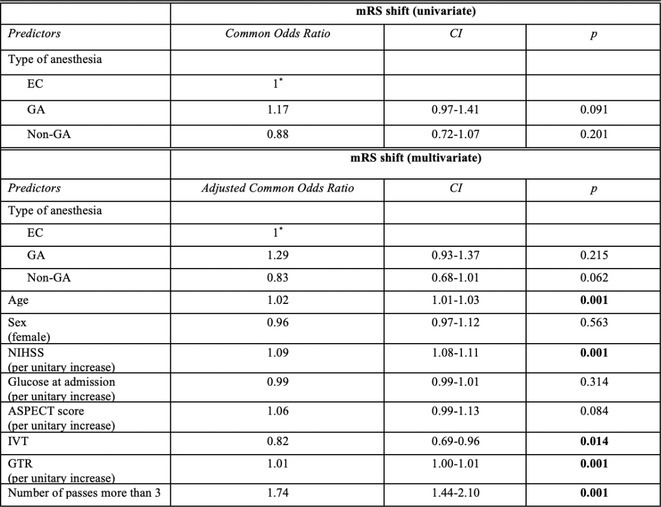
**FIGURE 1** Weighted shift analysis for mRS at 90 days. EC= emergency conversion; GA= general anaesthesia; NIHSS= National Institutes of Health Stroke Scale; ASPECT = Alberta Stroke Program Early CT; IVT= intravenous thrombolysis; GTR= groin‐to‐reperfusion. *Reference

**Figure:**
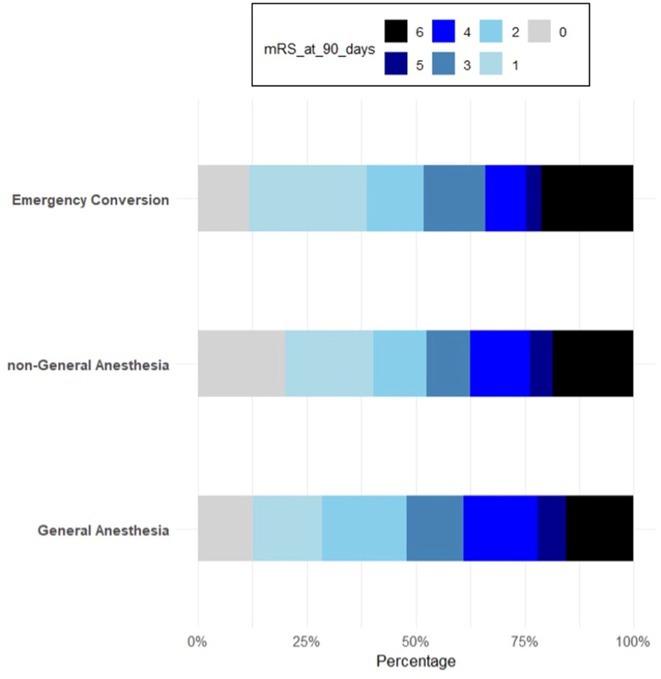
**FIGURE 2** Distribution of modified Rankin Scale (mRS) scores at 90 days in patients underwent Emergency Conversion (EC), non‐General Anesthesia (non‐GA), and General Anesthesia (GA).

**Conclusion:** Our study suggests that EC is not associated with worse functional outcome compared to patients undergoing primary GA or non‐GA.

**Disclosure:** Nothing to disclose.

## EPO‐037

### In‐hospital course of ischemic stroke without LVO treated with thrombolysis: Impact of Fazekas score, location, and size

#### F. Tazza; M. Pizzorno; E. Scarsi; C. Finocchi

##### Neurology Department, San Paolo Hospital, Savona, Italy

**Background and aims:** Whether leukoaraiosis worsens prognosis in acute ischemic stroke patients undergoing intravenous thrombolysis (IVT) remains debated. We aimed to assess how IVT influences outcomes in minor ischemic stroke without large vessel occlusion (LVO) and whether stroke size, location, or leukoaraiosis severity affects in‐hospital improvement.

**Methods:** We included acute ischemic stroke patients without LVO and confirmed by brain MRI. Demographic data, NIHSS at admission (NIHSS_in) and discharge (NIHSS_out), stroke location and size, and Fazekas score (sum of periventricular/subcortical values, range 0–6) were collected. Two‐way ANOVA was performed.

**Results:** Among 207 patients, 126 (60.9%) met inclusion criteria. 58.7% female, mean age was 76.6 ± 11.4 years, 17.5% IVT‐treated, mean NIHSS_in 4.55 ± 3.29, NIHSS_out 1.2 ± 1.87, hospital stay 11.65 ± 7.55 days. IVT and non‐IVT groups were similar in age/sex. Fazekas scores: 25.4% mild (0–2), 35.7% moderate (3–4), 37.3% severe (5–6). Stroke locations: 19.8% infratentorial, 19.8% deep, 50.8% hemispheric, 9.5% multifocal. IVT patients had significantly lower NIHSS_out (p < 0.001, covariate: NIHSS_in). Those with severe Fazekas scores showed greater NIHSS_out improvement with IVT (mean ‐1.7 ± 0.55; p = 0.002, covariate: NIHSS_in). Stroke location and size had no impact.

**Figure:**
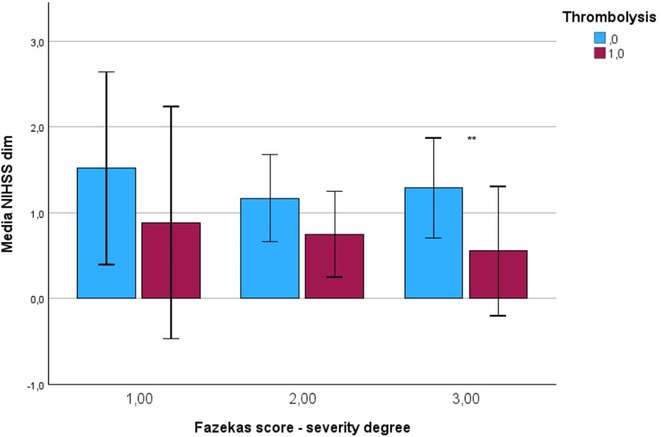
**FIGURE 1** NIHSS at discharge according to Fazekas score (1: mild, 2: moderate, 3: severe) in patients treated with intravenous thrombolysis (IVT) and those not treated with IVT.

**Conclusion:** In IVT‐treated patients, severe leukoaraiosis does not worsen outcomes. Instead, IVT appears particularly beneficial in these patients with reduced functional reserve.

**Disclosure:** F. Tazza, M. Pizzorno, E. Scarsi, C. Finocchi have nothing to disclose.

## EPO‐039

### Efficacy and safety of reteplase versus alteplase for the treatment of acute ischemic stroke: A meta‐analysis

#### M. Baker; M. Alkhawaldeh; M. G. Alshaikh Yousef

##### Faculty of Medicine, Jordan University of Science and Technology, Irbid, Jordan

**Background and aims:** Reteplase rise as a possible alternative to Alteplase in managing patients presenting with acute ischemic stroke within the eligible therapeutic window.

**Methods:** We systematically searched Medline, Scopus, and Cochrane Library up to August 2024. We Included RCTs comparing the efficacy and safety of Reteplase to that of Alteplase in adult patients who received thrombolytic therapy within 4.5 hours of symptoms onset and had excellent functional status before the onset of their stroke. Primary efficacy outcome was an excellent functional outcome, defined as a modified Rankin scale score of 0 or 1 within 90 days. Primary Safety outcome was assessed using the incidence of symptomatic intracranial hemorrhage within 36 hours. Other safety outcomes were the incidences of any adverse event, serious adverse event, and death within 90 days.

**Results:** Out of 376 records identified, 3 studies were included in this review, comprising 1772 patients, Reteplase showed higher excellent functional outcome (RR=0.89 95% CI= [0.84,0.94], P=<0.0001), higher dramatic recovery at 7‐30 days (RR=1.11, 95% CI=[1.04,1.18], p=0.002), and Barthel Index score of ≥61 at 90 days (RR=1.06, 95% CI=[1.01,1.12], P=0.02) in comparison to Alteplase. Symptomatic intracranial hemorrhage within 36 hours and mortality were not significantly different between the two arms (RR=1.06, 95% CI=[0.62,1.81], p=0.83), (RR=1.18, 95% CI=[0.72,192], p=0.51] respectively. However, the incidences of any adverse event or serious event within 90 days were significantly higher in the Reteplase arm (RR=1.12, 95% CI=[1.07,1.18], p=<0.00001), (RR=4.12, 95% CI=[2.79,6.07], p=<0.00001) respectively.

**Conclusion:** Reteplase seems to be more effective and less safe than Alteplase.

**Disclosure:** Nothing to disclose.

## EPO‐040

### Safety and efficacy of factor XIa inhibitors for prevention of thromboembolism: Systematic review and meta‐analysis

#### M. Noushad ^1^; N. Zahid^2^; F. Iqbal^3^; A. Siddique^3^; Nadeem^4^; W. Hussain^5^; A. Ishaq^6^; M. Ahmed^7^; N. Lal^3^; S. Abdullah^8^; H. Yousaf^9^; U. Jafar^10^; H. Ahmad Cheema^10^; R. Ahmed^11^

##### ^1^University Hospitals Plymouth NHS Trust, UK; ^2^Department of Medicine, Sialkot Medical College, Sialkot, Pakistan; ^3^Department of Medicine, Faisalabad Medical University, Faisalabad, Pakistan; ^4^Department of Medicine, Jinnah Medical & Dental College Karachi, Karachi, Pakistan; ^5^Department of Medicine, FMH College of Medicine & Dentistry, Lahore, Pakistan; ^6^Department of Medicine, Services Institute of Medical Sciences, Lahore, Pakistan; ^7^Department of Medicine, Liaquat University of Medical & Health sciences, Jamshoro, Pakistan; ^8^Department of Medicine, Allama Iqbal Medical College, Lahore, Pakistan; ^9^Department of Medicine, Asian Medical Institute, Kyrgyzstan, ^10^Department of Medicine, King Edward Medical University, Lahore, Pakistan, ^11^National Heart and Lung Institute, Imperial College London, UK

**Background and aims:** Stroke and thromboembolism remain the leading causes of mortality worldwide. Factor Xia (FXIa) might prevent thromboembolism without interfering with hemostasis, thus leading to a lower risk of bleeding compared to direct oral anticoagulants (DOACs).

**Methods:** We conducted a systematic search using PubMed, Embase, and Clincaltrials.gov to retrieve randomized controlled trials comparing FXIa inhibitors to placebo or DOACs in patients at risk of stroke or thromboembolism. All statistical analyses were carried out using RevMan 5.4, using a random effects model.

**Results:** Our meta‐analysis included 14 RCTs involving 30,952 patients. FXIa inhibitors significantly decreased the risk of major bleeding (RR 0.47, 95% CI: 0.33‐0.66, I2= 46%) with no significant change in systemic embolism or thromboembolism (RR 0.82, 95% CI: 0.66‐1.03, I2= 60%). There was no significant change between two groups when assessing the rate of all bleeding events (RR 0.78, 95% CI: 0.55‐1.11, I2= 73%), all‐cause mortality (RR 0.89, 95% CI: 0.70‐1.13, I2= 0%), ischemic stroke (RR 1.01, 95% CI: 0.47‐2.15, I2= 83%). FXIa inhibitors were associated with a reduced risk of intracranial hemorrhage (RR 0.44, 95% CI: 0.21‐0.95, I2= 1%). Although there was a higher rate of all adverse events in the FXIa inhibitors group (RR 1.58, 95% CI: 1.03‐2.40, I2= 95%), risk of serious adverse events (RR 1.16, 95% CI: 0.86‐1.55, I2= 74%) remained comparable between two groups.

**Figure:**
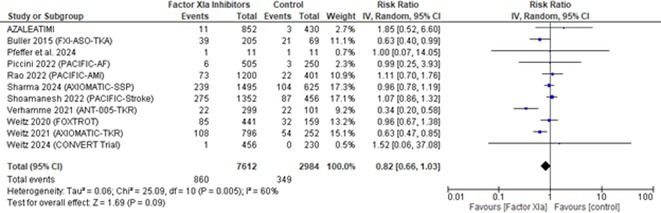
**FIGURE 1** Forrest plot for thromboembolism/systemic embolism.

**Figure:**
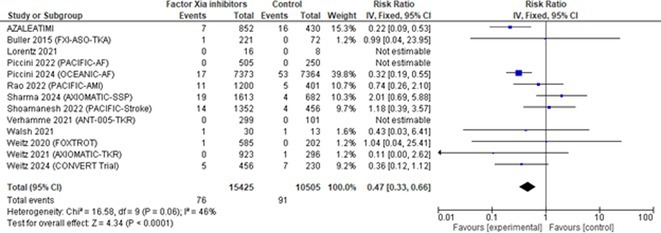
**FIGURE 2** Forrest plot for Major bleeding.

**Figure:**
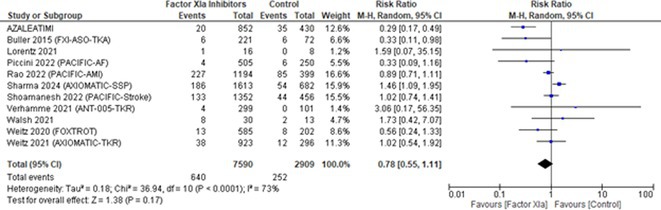
**FIGURE 3** Forrest plot for All bleeding events.

**Conclusion:** When compared with DOACs, FXIa inhibitors demonstrate favorable bleeding outcomes without changing thromboembolism, mortality, or other safety outcomes. Data from high‐quality, large‐scale RCTs is warranted for evidence of its clinical benefit.

**Disclosure:** Nothing to disclose.

## EPO‐041

### Left atrial appendage occlusion versus anticoagulant therapy alone after ischemic stroke despite anticoagulation

#### N. Mena García ^1^; M. Campos Jiménez^1^; G. Cabañas Engenios^1^; R. Pastor González^1^; M. Matute Lozano^1^; A. De Felipe Mimbrera^1^; R. Vera Lechuga^1^; J. Chico García^1^; L. Salido Tahoces^2^; J. Masjuan Vallejo^1^; S. García Madrona^1^; A. Cruz Culebras^1^

##### ^1^Stroke Unit, Neurology Department, Ramón y Cajal University Hospital, Madrid, Spain; ^2^Cardiology Department, Ramón y Cajal University Hospital, Madrid, Spain

**Background and aims:** Recurrent ischemic stroke in patients with atrial fibrillation (AF) despite oral anticoagulation remains a clinical challenge. Left atrial appendage occlusion (LAAO) is a potential mechanical intervention to mitigate this risk. We aim to compare the efficacy and safety of LAAO plus anticoagulation versus continued oral anticoagulant (OAC) therapy alone in secondary major events prevention.

**Methods:** This retrospective cohort study included patients with nonvalvular AF who experienced ischemic stroke despite anticoagulation. Outcomes were compared between two groups: LAAO recipients (mean follow‐up 4.7±3.3 years) and controls on OAC therapy (mean follow‐up 1.1±1.0 years). Multivariate Cox regression evaluated time to a first event, a composite of stroke, bleeding, and other systemic embolisms. Safety of the LAAO procedure was also evaluated.

**Results:** The study enrolled 108 patients, 45.4% women, with a mean age of 77.2±9.8 years. Both cohorts exhibited elevated CHA2DS2‐VASc scores, with a mean of ≥5±1.2. During the follow‐up, 20 patients experienced an event (4 in the LAAO group and 16 in the OAC group). The event rate was significantly lower in the LAAO group (0.082 vs 0.82 events per 100 patient‐years) with an aHR of 0.10 (CI 95% 0.01‐0.79, p=0.03). Multivariate analysis was adjusted for age and CHA2DS2‐VASc. Individually, each of the composite variables was not significant. Major procedural complications occurred in two patients (6%) undergoing LAAO (cardiorespiratory arrest and major bleeding).

**Conclusion:** LAAO showed superior event prevention compared to OAC alone in patients with nonvalvular AF, albeit with low‐frequency procedural risks. These findings support LAAO as an effective secondary prevention strategy in high‐risk population.

**Disclosure:** Nothing to disclose.

## EPO‐042

### Efficacy and safety of recombinant human prourokinase vs. alteplase in acute ischemic stroke: A meta‐analysis

#### N. Nhan ^1^; H. Vinh^1^; N. Vy^2^; T. Nghi^1^; M. Emmanuel Mark^3^; L. Oláh^4^

##### ^1^University of Debrecen Medical School, Debrecen, Hungary; ^2^The University of Melbourne, Melbourne, Australia; ^3^Department of Anesthesiology and Pain Medicine, Toronto General Hospital, Toronto, Canada; ^4^University of Debrecen Medical School, Department of Neurology, Debrecen, Hungary

**Background and aims:** Intravenous Alteplase at 0.9 mg/kg is the standard treatment for acute ischemic stroke in patients presenting within 4.5 hours. The role of recombinant human Prourokinase (rhPRO‐UK) in stroke management is less understood. This meta‐analysis evaluates the efficacy and safety of rhPRO‐UK compared to Alteplase.

**Methods:** A systematic search of PubMed and Cochrane databases identified randomized controlled trials (RCTs) comparing rhPRO‐UK to Alteplase in acute ischemic stroke. Outcomes assessed included: (1) Modified Rankin Scale (mRS) scores of 0–2 (good functional outcomes) at 90 days; (2) systemic bleeding; (3) symptomatic intracranial bleeding (as defined by SITS‐MOST); and (4) serious adverse events within 90 days. Heterogeneity was assessed using the I^2^ statistic, and random‐effects models were applied.

**Results:** Three RCTs including 1,179 patients treated with rhPRO‐UK were analyzed. At 90 days, rhPRO‐UK was non‐inferior to Alteplase in achieving mRS 0–2 outcomes (OR 1.01; 95% CI 0.83–1.23; p = 0.93; I^2^ = 0%). Risks of symptomatic intracranial bleeding (RR 0.53; 95% CI 0.18–1.59; p = 0.26; I^2^ = 25%) and serious adverse events (RR 0.95; 95% CI 0.78–1.16; p = 0.60; I^2^ = 0%) were comparable. The risk of systemic bleeding was lower with rhPRO‐UK (RR 0.73; 95% CI 0.58–0.92; p = 0.009; I^2^ = 57%).

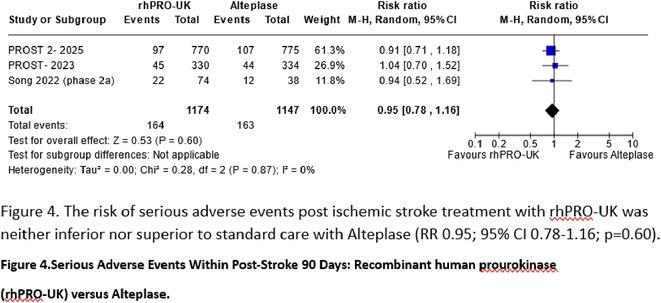

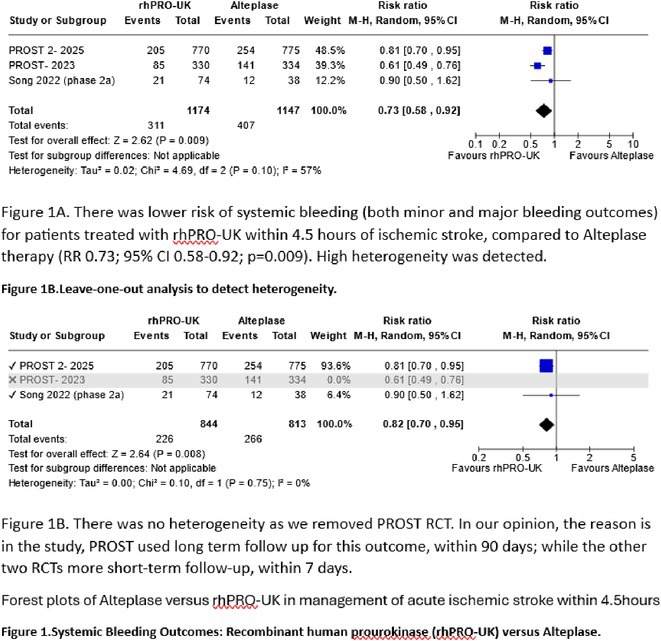

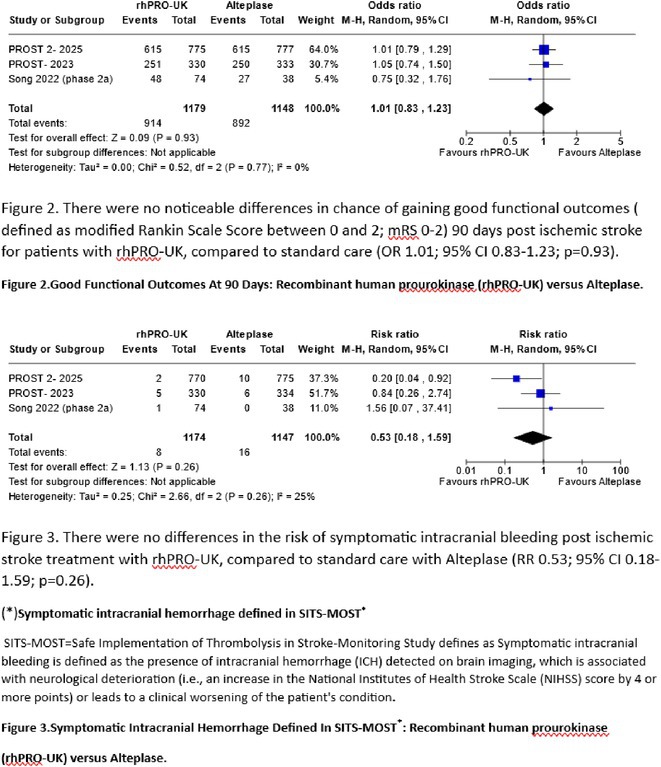

Symptomatic Intracranial Hemorrhage and Good Functional Outcomes at 90 Days: Recombinant human prourokinase (rhPRO‐UK) versus Alteplase.

**Conclusion:** RhPRO‐UK offers similar efficacy and safety to Alteplase in acute ischemic stroke, with a reduced risk of systemic bleeding. These findings, limited by potential biases such as sample size reductions, warrant further RCTs to confirm rhPRO‐UK's safety profile and explore its potential superiority.

**Disclosure:** Nothing to disclose.

## EPO‐043

### Predictive value of ABCD2 score and Canadian TIA Score for outpatient management of vascular neurological deficits

#### P. Scoppettuolo ^1^; S. El Sankari^1^; S. Ferrao Santos^1^; A. Sellimi^1^; I. Gunes Tatar^2^; A. Penaloza^3^; A. Peeters^1^

##### ^1^Neurology Department, Cliniques Universitaires Saint Luc, UCLouvain, Brussels, Belgium; ^2^Radiology Department, Cliniques Universitaires Saint Luc, UCLouvain, Brussels, Belgium; ^3^Emergency Department, Cliniques Universitaires Saint Luc, UCLouvain, Brussels, Belgium

**Background and aims:** The safety of outpatient management for transient ischemic attack (TIA) and minor acute ischemic stroke (MAIS) remains controversial. This study aimed to evaluate the predictive value of the ABCD2 score and the Canadian TIA Score (CTS) for acute ischemic lesions (AIL) and to identify independent predictors of recurrence in patients presenting with TIA or MAIS, to guide admission to stroke units.

**Methods:** We retrospectively reviewed medical records of patients with suspected TIA or MAIS between June 2022 and December 2023. ABCD2 and CTS scores were considered positive at cut‐offs of ≥4 and ≥9 points, respectively, indicating high acute ischemic stroke risk. Univariate and multivariate analyses compared patients with and without AIL, while ROC analysis assessed the predictive value of both scores.

**Figure:**
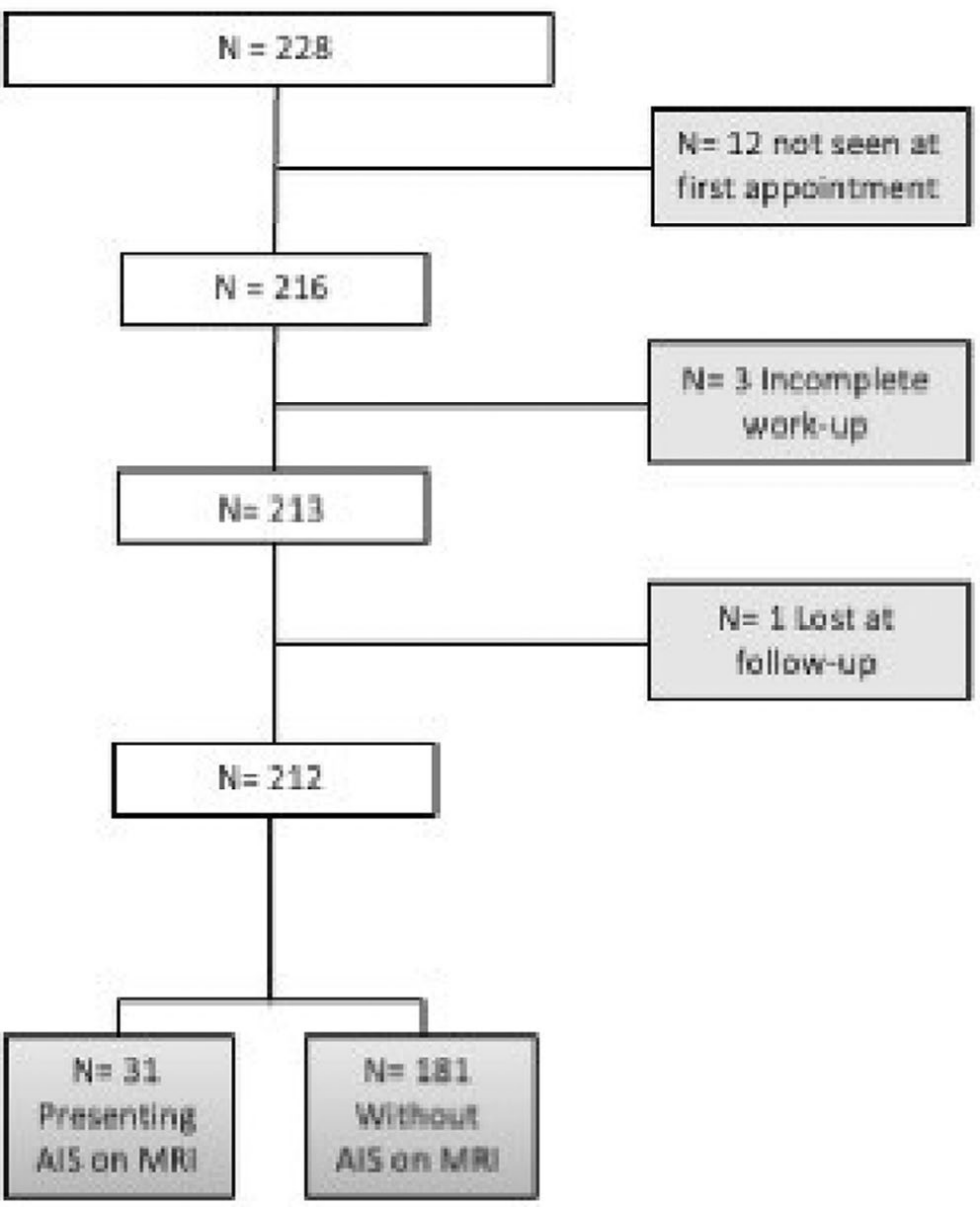
**FIGURE 1** Flowchart

**Results:** From 212 patients analyzed, thirty‐one (14.6%) had AIL and presented more often with motor deficits (29% vs. 11%, p=0.02), symptom duration ≥24 hours (41.9% vs. 9.9%, p<0.001), and shorter delays to MRI (8 vs. 16 days, p=0.03). Multivariate analysis identified early MRI acquisition (per day: OR=0.96, p=0.013), symptom duration ≥24 hours (OR=7.77, p<0.001), and motor deficits (OR=3.93, p=0.008) as independent predictors of AIL. Both ABCD2 and CTS scores demonstrated high negative predictive value (NPV) (90% and 88.7%, respectively) but moderate predictive accuracy (60% vs. 69.8%). Among 28 patients with recurrence, no significant differences were observed compared to the overall population.

**Figure:**
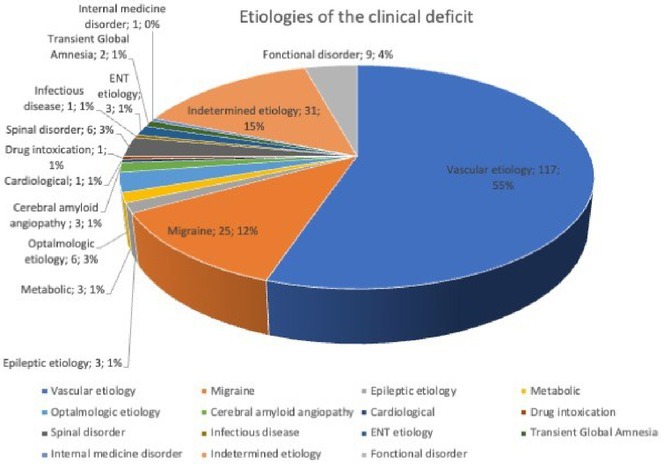
**FIGURE 2** Etiologies

**Conclusion:** ABCD2 score and CTS exhibit moderate accuracy for predicting AIL but provide high NPV, supporting their use in safely discharging patients with presumptive negative MRI findings.

**Disclosure:** Nothing to disclose.

## EPO‐044

### Management of unruptured intracranial aneurysms: 10‐year experience of a multidisciplinary committee

#### P. López‐Grueiro Valcarce ^1^; L. Pulido Fraiz^1^; C. Hervás Testal^1^; M. Alonso de Lenciñana Cases^1^; B. Fuentes Gimeno^1^; G. Ruiz Ares^1^; A. Fernández Prieto^2^; B. Marín Aguilera^2^; R. Frutos Martínez^2^; P. Navia Álvarez^2^; A. Álvarez Muelas^2^; Á. Gómez de la Riva^3^; B. Hernández García^3^; R. Rigual Robillo^1^; E. de Celis Ruiz^1^; L. Casado Fernández^1^; L. González Martín^1^; J. Rodríguez Pardo de Donlebún^1^

##### ^1^Neurology, Hospital Universitario La Paz, Madrid, Spain; ^2^Radiology, Hospital Universitario La Paz, Madrid, Spain; ^3^Neurosurgery, Hospital Universitario La Paz, Madrid, Spain

**Background and aims:** ESO guidelines report a risk of rupture for unruptured intracranial aneurysms (UIA) of 0.8%/patient‐year. Endovascular or surgical treatment is often indicated in UIAs with high‐risk features, with reported treatment failure of 10‐18% and disabling procedural complications of 4%. We analyzed the characteristics and prognosis of patients with UIA evaluated by a multidisciplinary committee.

**Methods:** Retrospective observational study of patients with UIA evaluated by a multidisciplinary committee (neurology, neurosurgery, neuroradiology) between 2011 and 2019. We compared basal characteristics and outcomes of treated and untreated patients.

**Results:** Seventy‐five patients were included, median age 60 years (71% women), with median follow‐up of 8.4 (6.5‐11.4) years after UIA diagnosis. Most frequent risk factors were hypertension (56%) and smoking (51%). Most common aneurysm locations were middle cerebral (36%) and internal carotid arteries (35%). Ten patients (13%) presented with compressive symptoms, while 87% were considered incidental UIAs. Thirty‐eight patients (51%) underwent intervention (25 endovascular and 13 surgical treatment), while 37 (49%) were managed conservatively. No significant differences were found between treatment groups in terms of age, maximum aneurysm diameter or PHASES score. Treated aneurysms were more frequently irregular (21% vs 5%). One (2.6%) fatal complication (and 2 non‐disabling) occurred in the treated group and 4 (10%) required reintervention. One fatal hemorrhage (global rupture rate of 0.3%/patient‐year) occurred in the conservative group. Significant aneurysm growth was reported in 4 patients during follow‐up, one of whom underwent embolization.

**Conclusion:** Evaluation of UIA management by a multidisciplinary committee is associated with low rates of fatal complications and rupture during follow‐up.

**Disclosure:** Nothing to disclose.

## EPO‐045

### Laboratory and radiological findings associated with increased stroke risk in atheosclerotic carotid disease

#### S. Yusufli ^1^; S. Soylu^2^; B. Yalçın^2^; G. Koral^2^; M. Sezgin^1^; M. Barburolu^3^; S. Sencer^3^; N. Yeşilot^1^; V. Yilmaz^2^; E. Tüzün^2^; C. Küçükali^2^; E. Ekizoğlu^1^

##### ^1^Department of Neurology, Istanbul University, İstanbul, Turkey; ^2^Department of Neuroscience, Istanbul University ‐ Aziz Sancar Experimental Medicine Research Institute, İstanbul, Turkey; ^3^Department of Radiology, Istanbul University, İstanbul, Turkey

**Background and aims:** The aim is to identify inflammatory and radiological biomarkers that can predict ischemic stroke risk.

**Methods:** 62 cases were examined in three groups: asymptomatic carotid stenosis (ACS), symptomatic carotid stenosis (SCS), and control group. In serum samples, inflammatory markers and natural immune system activity were evaluated by ELISA and flow cytometry. In the SCS group, they were repeated in the acute phase and at 6‐months. While all patients underwent vascular imaging, white matter lesions were classified according to the Fazekas scale using MR, and the presence of intraplaque hemorrhage was evaluated with MPRAGE. Cognitive functions of patients were determined using the ACE‐R and MMSE tests.

**Results:** In the SCS group, levels of IL‐6, IL‐1 beta, and M1 monocytes were higher compared to the ACS and control groups (p < 0.01, p < 0.05, p < 0.05, respectively). IL‐8 levels in the SCS group were higher than the control group (p < 0.05). In the ACS group, IL‐8 levels were higher in patients with heterogeneous plaques than homogeneous plaques (p = 0.04). CitH3 and PMN in the SCS and IL‐6 in the ACS were higher in patients with advanced carotid stenosis (p = 0.008, p = 0.009, p = 0.043, respectively). In the ACS group, MMSE and ACE‐R scores were found to be lower than the control group (p = 0.045, p = 0.005).

**Conclusion:** Increased inflammation and neutrosis in atherosclerotic carotid disease were associated with stroke. No clear relationship was demonstrated between severe white matter disorder and increased systemic inflammation.

**Disclosure:** Nothing to disclose.

## Cognitive Neurology/Neuropsychology

## EPO‐046

### Loneliness and prescription drug misuse in older patients: A study of patients on potentially addictive medication

#### S. Cheng^1^; T. Ghazal Siddiqui^1^; M. Gossop^2^; C. Lundqvist ^3^

##### ^1^Lørenskog, Norway; ^2^National Addiction Centre, Kings College, London, UK; ^3^Dept. Clinical Medicine, Campus Akershus University Hospital, University of Oslo; and HØKH Health Services Research, Akershus University Hospital, Løenskog, Norway

**Background and aims:** Medications with addictive potential are commonly prescribed to older patients despite consensus recommendations advocating caution. Studies have shown a link between loneliness and medication use, but rarely evaluate substance use disorder‐related outcomes in real‐life clinical settings. We explored the association between loneliness prolonged drug use and severity of drug dependence.

**Methods:** With a clinical sample including 246 consenting older patients, we conducted a consecutive cross‐sectional study at a large public regional hospital in Norway. We measured loneliness, using the De Jong‐Gierveld Loneliness Scale, and severity of prescription drug dependence by the Severity of Dependence Scale (SDS), and defined prolonged use as medication use exceeding duration recommended by clinical guidelines. We cross‐checked information from both patient interviews and electronic hospital registries. Multivariable logistic and linear regression models were used.

**Results:** The adjusted odds ratio of prolonged use for overall, social and emotional loneliness were 1.32 (95% CI: 1.07–1.61), 1.52 (95% CI: 1.05–2.20) and 1.41(95% CI: 1.05–1.91), respectively. The odds were higher for female and multimorbid patients, and lower for those with higher education. Loneliness was positively associated with the SDS score, adjusting for sociodemographic and clinical characteristics. The association was stronger when Z‐hypnotics were co‐used with opioids or benzodiazepines.

**Figure:**
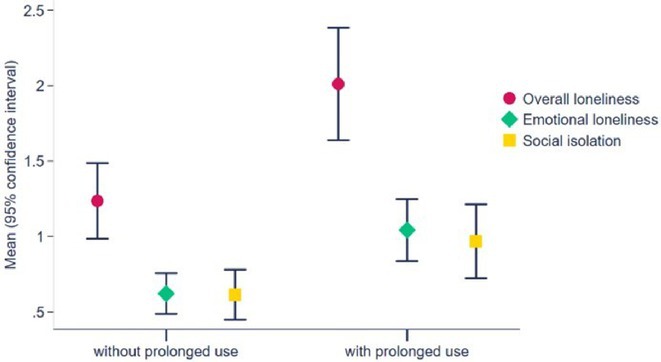
**FIGURE 1** Loneliness score in older patients with vs without prolonged use of potentially addictive meds.

**Figure:**
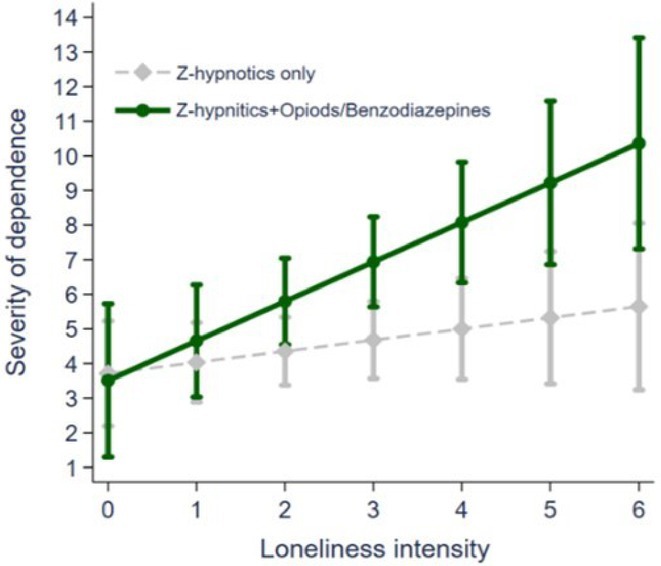
**FIGURE 2** Association between loneliness score and severity of dependence in older patients using potentially addictive meds.

**Conclusion:** In older patients, physicians should be aware that prolonged use and dependence on central nervous system inhibitory drugs are more probable when loneliness intensifies. A particular focus on female, lower‐educated, multimorbid patients, and those receiving concomitant medications is suggested.

**Disclosure:** CL has participated on an advisory board and received payment for lectures arranged by Abbvie Pharma AS, Novartis AS, and Roche AS, Norway. He has also received research sponsorship from Abbvie pharma. All other authors declare that they have no conflicts of interest.

## EPO‐047

### Emotion recognition deficits and brain functional network connectivity in frontotemporal degeneration

#### A. Gilioli ^1^; E. Canu^2^; C. Tripodi^1^; S. Basaia^1^; V. Castelnovo^1^; E. Sibilla^2^; F. Freri^1^; E. Spinelli^3^; G. Cecchetti^2^; G. Magnani^4^; F. Caso^4^; E. Aiello^5^; G. De Luca^5^; P. Caroppo^6^; S. Prioni^6^; C. Villa^6^; L. Tremolizzo^7^; I. Appollonio^7^; F. Verde^8^; N. Ticozzi^8^; V. Silani^8^; M. Gorno‐Tempini^9^; B. Poletti^10^; M. Filippi^11^; F. Agosta^3^

##### ^1^Neuroimaging Research Unit, Division of Neuroscience, IRCCS San Raffaele Scientific Institute, Milan, Italy; ^2^Neuroimaging Research Unit, Division of Neuroscience, and Neurology Unit, IRCCS San Raffaele Scientific Institute, Milan, Italy; ^3^Neuroimaging Research Unit, Division of Neuroscience, and Neurology Unit, IRCCS San Raffaele Scientific Institute, and Vita‐Salute San Raffaele University, Milan, Italy; ^4^Neurology Unit, IRCCS San Raffaele Scientific Institute, Milan, Italy; ^5^Department of Neurology and Laboratory of Neuroscience, IRCCS Istituto Auxologico Italiano, Milan, Italy; ^6^Fondazione IRCCS Istituto Neurologico Carlo Besta, Unit of Neurology 5 ‐ Neuropathology, Milan, Italy; ^7^Neurology Unit, IRCCS “Fondazione San Gerardo” and School of Medicine and Surgery, University of Milano‐Bicocca, Monza, Italy; ^8^Department of Neurology and Laboratory of Neuroscience, IRCCS Istituto Auxologico Italiano, and Department of Pathophysiology and Transplantation, “Dino Ferrari” Center, Università degli Studi di Milano, Milan, Italy; ^9^Memory and Aging Center, and Global Brain Health Institute, University of California San Francisco, San Francisco, California, USA, ^10^Department of Neurology and Laboratory of Neuroscience, IRCCS Istituto Auxologico Italiano, and Department of Oncology and Hemato‐Oncology, Università degli Studi di Milano, Milano, Italy, ^11^Neurology Unit, Neurorehabilitation Unit, Neurophysiology Service, and Neuroimaging Research Unit, Division of Neuroscience, IRCCS San Raffaele Scientific Institute, and Vita‐Salute San Raffaele University, Milan, Italy

**Background and aims:** We investigated emotion recognition (ER), functional brain connectivity of ER‐related networks and their relationship in a frontotemporal degeneration (FTD) cohort.

**Methods:** 109 FTD patients [46 bvFTD, 10 sbvFTD, 13 nfvPPA, 18 svPPA, 22 PSP] and 114 HC underwent the Comprehensive Affect Testing System‐abbreviated version (CATS‐A). Accuracy scores for basic and combined positive and negative emotions were compared across groups. Structural and resting‐state functional MRI were obtained for 79 patients and 49 HC. In 50 young HC, functional connectivity of six ER networks was reconstructed from key nodes: salience (SN), semantic appraisal, anterior default mode (aDMN), visuo‐associative, sensorimotor (SMN), and basal ganglia networks. Intra‐network direct functional connectivity (dFC) and graph‐based nodal properties were compared among groups and correlated with CATS‐A scores.

**Results:** FTD groups showed deficits in recognizing positive and, mainly, negative emotions compared to HC. SvPPA recognized happiness better than bvFTD and nfvPPA, while sbvFTD recognized worse disgust than svPPA. Compared to HC, all FTD patients exhibited altered dFC nodal properties, including higher path length, reduced nodal strength, local efficiency and clustering coefficient within the SN, SMN and aDMN. Lower accuracy in recognizing negative emotions correlated with altered aDMN properties in FTD and SMN in HC.

**Conclusion:** Our data confirmed ER deficits in FTD and suggested a differential role in ER valence based on the temporal FTD subtypes (svPPA vs sbvFTD). While HC relied on the SMN in processing negative emotions, FTD patients showed a shift to the aDMN, reflecting loss of specificity and compensatory mechanisms.

**Disclosure:** Funding. European Research Council (StG‐2016_714388_NeuroTRACK); Foundation Research on Alzheimer Disease. Co‐funding Next Generation EU [DM 1557 11.10.2022]. Disclosures: AG, CT, VC, ES, FF, EGS, GM, FC, ENA, GDL, PC, SP, CV, LT, IA, FV, MLGT nothing; EC, SB grants from Italian Ministry of Health (MSAL); GC speaker fees from Neopharmed Gentili; NT consulting fees from Amylyx Pharmaceuticals and Zambon Biotech SA. VS consulting or speaking fees from AveXis, Cytokinetics, Italfarmaco, Liquidweb srl, Novartis Pharma AG, Amylyx Pharmaceuticals, Biogen, Zambon Biotech SA; grants from MSAL, AriSLA, and E‐Rare Joint Transnational Call; BP consulting or speaking fees from Liquidweb srl; MF consulting or speaking fees from Alexion, Almirall, Bayer, Biogen, Bristol‐Myers Squibb, Celgene, Chiesi Italia SpA, Eli Lilly, Genzyme, Janssen, Merck‐Serono, Neopharmed Gentili, Novartis, Novo Nordisk, Roche, Sanofi Takeda, TEVA; scientific direction of educational events for Biogen, Merck, Roche, Celgene, Bristol‐Myers Squibb, Lilly, Novartis, Sanofi‐Genzyme; research support from Biogen Idec, Merck‐Serono, Novartis, Roche. FA speaker honoraria from Biogen Idec, Roche, Eli Lilly and GE Healthcare, and grants from MSAL, the Italian Ministry of University and Research, AriSLA, the ERC, the EU Joint Programme – Neurodegenerative Disease Research, Foundation Research on Alzheimer Disease.

## EPO‐048

### Neuropsychological tests predict amyloid status in MCI and mild dementia: Real‐world memory clinic insights

#### B. Pancaldi ^1^; A. Zilioli^1^; G. Busi^1^; F. Ferrari‐Pellegrini^2^; L. Ruffini^3^; M. Spallazzi^2^

##### ^1^Department of Medicine and Surgery, University of Parma, Parma, Italy; ^2^Department of Medicine and Surgery, University‐Hospital of Parma, Parma, Italy; ^3^Nuclear Medicine Unit, University Hospital of Parma, Parma, Italy

**Background and aims:** Alzheimer's disease (AD), the leading cause of dementia, necessitates accessible diagnostic tools to complement amyloid‐PET imaging and cerebrospinal fluid (CSF) analysis, particularly with the advent of disease‐modifying therapies. This study evaluated the predictive accuracy of individual and combined neuropsychological assessments for amyloid‐PET status in patients with mild cognitive impairment (MCI) or mild dementia, considering age and education level.

**Methods:** A retrospective analysis (2015–2019) included 118 adults aged 45–85 years with cognitive deficits. Exclusion criteria were CDR >1, MMSE <20, clinically significant neuroimaging abnormalities, or a history of major cerebrovascular events. Neuropsychological assessments. Amyloid‐PET was conducted within six months of baseline testing. Statistical analyses evaluated test performance using ROC curves and logistic regression and were conducted using the open‐source statistical software Jamovi (v. 2.3.21.0).

**Results:** The FCSRT immediate free‐recall (IFR) component (AUC = 0.70) and the recall of Rey‐Osterreith Complex Figure (AUC = 0.63) showed predictive value for amyloid positivity. While FCSRT displayed high sensitivity and Rey recall high specificity, combined test performance was modestly improved by including verbal fluency and age (AUC = 0.76). Subgroup analyses showed better predictive accuracy in patients younger than 75 years and with higher education levels (AUC = 0.88 for IFR; AUC = 0.78 for Rey recall).

**Figure:**
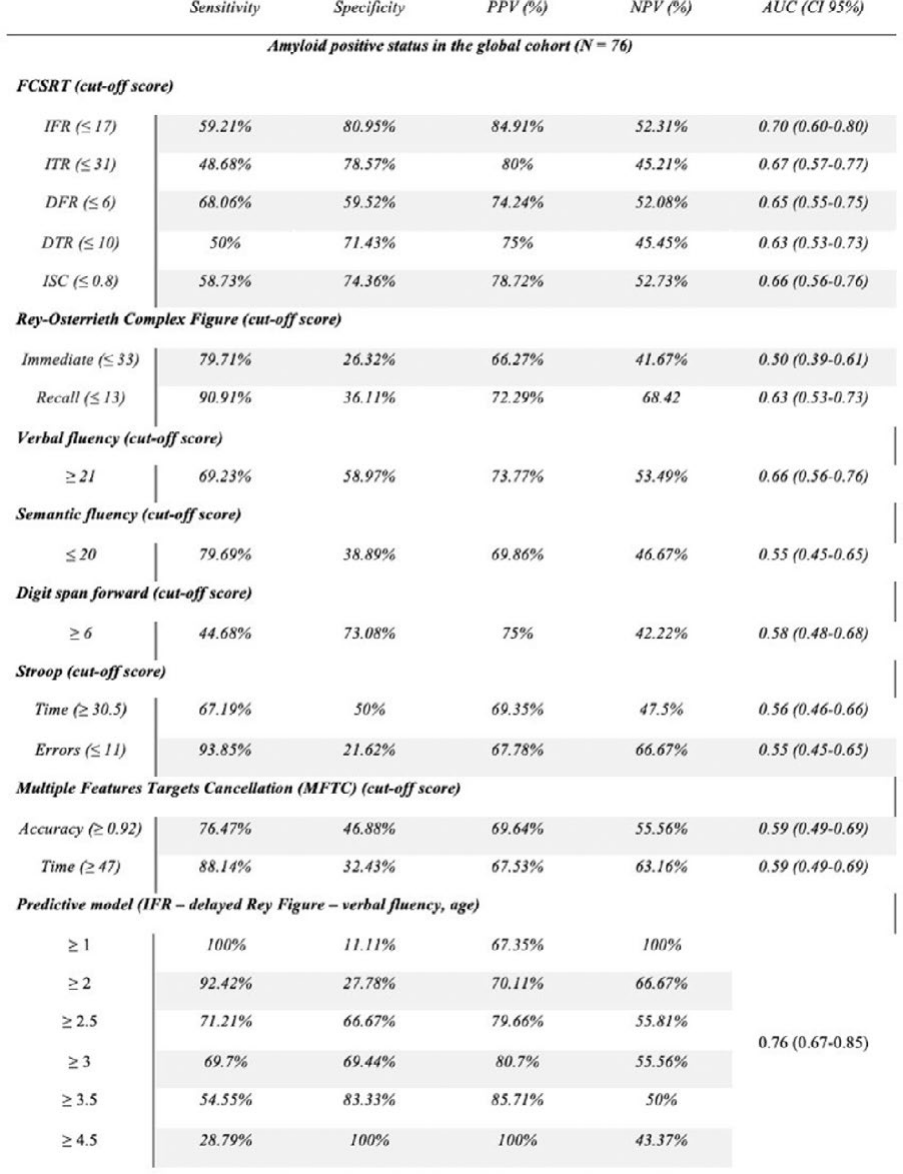
**TABLE 1** Predictive performance of neuropsychological tests and predictive model within the global cohort.

**Figure:**
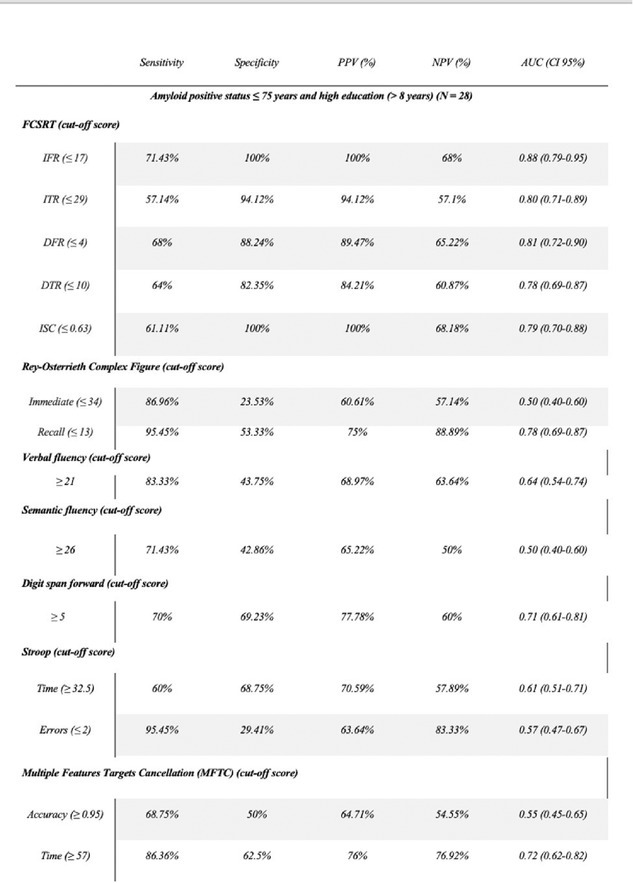
**TABLE 2** Predictive performance of neuropsychological tests within the cohort ≤ 75 years and with high education (> 8 years)

**Figure:**
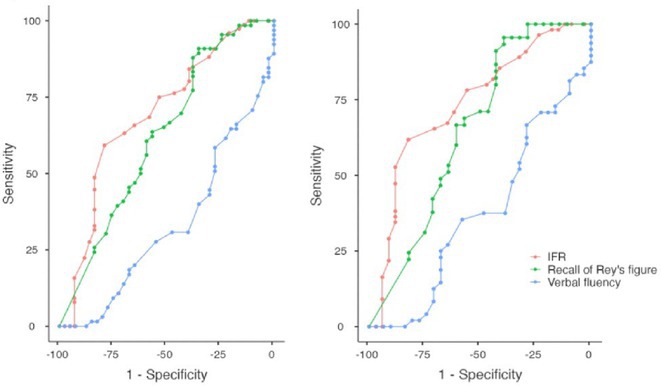
**FIGURE 1** ROC curves of IFR, recall of Rey's figure, and verbal fluency. On the left in the global cohort, on the right in the cohort ≤ 75 years.

**Conclusion:** These results emphasize the utility of neuropsychological assessments as non‐invasive, cost‐effective tools for identifying individuals requiring advanced diagnostic confirmation of AD, particularly in younger and more educated patients.

**Disclosure:** Nothing to disclose.

## EPO‐049

### Investigating the reality of functional neurological disorder diagnosis: A case study from a UK neurological centre

#### E. Nicholas; R. Nicholas; J. Varley

##### Department of Neurology, Imperial College London, London, UK

**Background and aims:** This study investigates diagnostic rates and clinical attitudes towards Functional Neurological Disorder (FND) at a large UK neurological centre without a dedicated FND service.

**Methods:** Inpatient episodes, outpatient appointments, and emergency department attendances from 1 January 2018 to 30 June 2024 were reviewed to determine FND diagnosis rates. A total of 9,698 medical records were assessed for coding accuracy, while a clinician survey explored neurologists’ understanding of the condition within the neurological centre. These findings were contextualised with historical understandings of functional disorders.

**Results:** FND was diagnosed in 19 patients over the study period. A diagnosis rate of 0.0135% was found in outpatient appointments and a rate of 0.0145% in emergency department attendances. Survey responses highlighted the influence of historical stigmas, outdated perceptions, and insufficient institutional support in shaping clinical attitudes and treatment decisions.

**Conclusion:** Despite accounting for 5‐15% of neurology patients and an estimated 8,000 new diagnoses in the UK annually, FND remains a marginalised condition at this centre. Persistent stigma and systemic underinvestment contribute to low diagnosis rates and inadequate care. Addressing these cultural and institutional barriers in essential for improving diagnosis and treatment for FND.

**Disclosure:** Nothing to disclose.

## EPO‐050

### Rubber hand illusion in Parkinson's disease

#### E. Canu ^1^; E. Sibilla^1^; E. Sarasso^2^; S. Basaia^3^; V. Castelnovo^1^; C. Tripodi^3^; F. Freri^3^; T. Ghisolfi^3^; M. Leocadi^3^; D. Corbetta^4^; A. Gardoni^3^; L. Zenere^3^; A. Grassi^3^; R. Balestrino^5^; M. Malcangi^5^; M. Volontè^5^; F. Agosta^6^; M. Filippi^7^

##### ^1^Neuroimaging Research Unit, Division of Neuroscience, and Neurology Unit, IRCCS San Raffaele Scientific Institute, Milan, Italy; ^2^Neuroimaging Research Unit, Division of Neuroscience, IRCCS San Raffaele Scientific Institute, Vita‐Salute San Raffaele University, Milan, Italy; and DINOGMI, University of Genoa, Genoa, Italy; ^3^Neuroimaging Research Unit, Division of Neuroscience, IRCCS San Raffaele Scientific Institute, Milan, Italy; ^4^Department of Rehabilitation and Functional Recovery, IRCCS Ospedale San Raffaele, Milan, Italy; ^5^Neurology Unit, and Neurorehabilitation Unit, IRCCS San Raffaele Scientific Institute, Milan, Italy; ^6^Neuroimaging Research Unit, Division of Neuroscience, and Neurology Unit, IRCCS San Raffaele Scientific Institute, and Vita‐Salute San Raffaele University, Milan, Italy; ^7^Neurology Unit, Neurorehabilitation Unit, Neurophysiology Service, and Neuroimaging Research Unit, Division of Neuroscience, IRCCS San Raffaele Scientific Institute, and Vita‐Salute San Raffaele University, Milan, Italy

**Background and aims:** To investigate the rubber hand illusion (RHI) in patients with Parkinson's disease (PD) compared to healthy controls (HC).

**Methods:** 37 PD and 30 HC underwent neuropsychological evaluation, RHI, and RHI‐questionnaires, and a functional MRI (fMRI) during a virtual reality motor task (VR‐motor task). VR‐motor‐task consisted in hand‐moving while observing a virtual hand in three conditions (synchronous, demultiplied, mismatch). We compared PD and HC with multivariate analyses examining the proprioceptive localization of the participant's index finger (PLIF) before stimulations, post‐stimulation RHI‐questionnaire scores, and fMRI activity. Linear regressions were used to analyze the relationship between proprioceptive drifts and RHI‐questionnaire scores. Linear mixed‐effects models examined PLIF changes after each stimulation and between pre‐stimulation phases.

**Results:** No PLIF differences were observed between groups in each pre‐stimulation phase. Compared to HC, PD patients showed higher RHI‐questionnaire scores. In all participants, we observed a positive relationship between RHI‐questionnaire scores and proprioceptive drifts toward the RH. Both groups showed significant PLIF changes toward the RH after the first left‐hand synchronous stimulation. In PD, we observed PLIF changes also after second synchronous left‐hand stimulation and after both first and second synchronous right‐hand stimulation. During fMRI, PD patients showed reduced left temporal‐parietal junction activity in the demultiplied condition.

**Conclusion:** In both groups, we confirmed the RHI after a first synchronous left‐hand stimulation. In PD, the RHI effect increased after a second stimulation and extended to the dominant hand. PD patients also showed higher subjective RH ownership in both conditions and hands. These findings suggest impaired self‐agency in PD.

**Disclosure:** Funding. Italian Ministry of Health (GR‐2018‐12366005). Disclosures: ES, ML, VC, CT, FF, TG, AG, LZ, AG, RB, MM, MAV have nothing to disclose; EC, ES, SB, DC receive research supports from the Italian Ministry of Health. MF received compensation for consulting services or speaking activities from Alexion, Almirall, Bayer, Biogen, Celgene, Chiesi Italia SpA, Eli Lilly, Genzyme, Janssen, Merck‐Serono, Neopharmed Gentili, Novartis, Novo Nordisk, Roche, Sanofi Takeda, and TEVA; Advisory Boards for Alexion, Biogen, Bristol‐Myers Squibb, Merck, Novartis, Roche, Sanofi, Sanofi‐Aventis, Sanofi‐Genzyme, Takeda; scientific direction of educational events for Biogen, Merck, Roche, Celgene, Bristol‐Myers Squibb, Lilly, Novartis, Sanofi‐Genzyme; he receives research support from Biogen Idec, Merck‐Serono, Novartis, Roche, the Italian Ministry of Health, the Italian Ministry of University and Research, and FISM. FA is Associate Editor of NeuroImage: Clinical, has received speaker honoraria from Biogen Idec, Roche, Eli Lilly and GE Healthcare, and receives or has received research supports from the Italian Ministry of Health, the Italian Ministry of University and Research, AriSLA (Fondazione Italiana di Ricerca per la SLA), the European Research Council, the EU Joint Programme – Neurodegenerative Disease Research (JPND), and Foundation Research on Alzheimer Disease (France).

## EPO‐051

### Cognitive performance in healthy females: A comparative study of menopause and non‐menopause groups

#### J. Dinic ^1^; M. Sarcevic^2^; P. Aleksic^2^; U. Lazic^2^; B. Salak Djokic^2^; V. Ilic^2^; G. Mandic Stojmenovic^3^; E. Stefanova^3^; T. Stojkovic^3^

##### ^1^University of Belgrade, Faculty of Medicine, Belgrade, Serbia; ^2^University Clinical Center of Serbia, Neurology Clinic, Belgrade, Serbia; ^3^University of Belgrade, Faculty of Medicine, University Clinical Center of Serbia, Neurology Clinic, Belgrade, Serbia

**Background and aims:** After age, sex is a major risk factor for Alzheimer's disease with lower lifetime exposure to ovarian hormones being the strongest cause. The cognitive effects of menopause are not fully understood, and studies on healthy females are lacking. This study aimed to explore differences in cognitive performance in healthy menopausal and non‐menopausal women.

**Methods:** We recruited 114 healthy female volunteers aged 30‐65 years among Belgrade University employees. The exclusion criteria were difficulties in communicative ability and/or safe engagement in interventions, and presence of any neurological, psychiatric, medical condition, or iatrogenic cause known to affect the brain structure and/or function. All participants underwent a detailed assessment including basic demographic data, data on vascular risk factors, mood scales, comprehensive neuropsychological assessment, and Cognitive Reserve Index Questionnaire.

**Results:** Among participants, 48 reached menopause at the mean age of 48.6 ± 4.89 years, while 66 were not yet in menopause. The mean age/education of the menopause group was 54.91 ± 4.8/18.60 ± 4.16 years, respectively, and 43.11 ± 6.91/19.31 ± 4.13 years in the non‐menopausal group. The menopausal group had significantly more subjective complaints in general (median 7.00 vs. 3.50, p=0.005 for positive answers on the MyCog scale), especially in the memory domain (median 3.00 vs. 1.00, p=0.038). Differences in raw scores of neuropsychological tests (MMSE, MOCA, ROCF memory score) did not persist after age correction.

**Conclusion:** The menopausal group reported more cognitive complaints, however, cognitive performance did not differ. Further research on healthy women is needed to understand the cognitive burden of menopause better.

**Disclosure:** This research was supported by Alzheimer's Association grant AACSFD‐17‐533520.

## EPO‐052

### An Italian scale for the assessment of motor speech disorders in patients with PPA and PSP

#### L. Lumaca ^1^; E. Canu^2^; A. Riva^1^; V. Castelnovo^2^; E. Sibilla^2^; C. Tripodi^1^; F. Freri^1^; T. Ghisolfi^1^; E. Spinelli^3^; G. Cecchetti^4^; M. Gorno Tempini^5^; M. Filippi^6^; F. Agosta^3^

##### ^1^Neuroimaging Research Unit, Division of Neuroscience, IRCCS San Raffaele Scientific Institute, Milan, Italy; ^2^Neuroimaging Research Unit, Division of Neuroscience, and Neurology Unit, IRCCS San Raffaele Scientific Institute, Milan, Italy; ^3^Neuroimaging Research Unit, Division of Neuroscience, and Neurology Unit, IRCCS San Raffaele Scientific Institute, and Vita‐Salute San Raffaele University, Milan, Italy; ^4^Neurology Unit, Neurophysiology Service, and Neuroimaging Research Unit, Division of Neuroscience, IRCCS San Raffaele Scientific Institute, Milan, Italy; ^5^Department of Neurology, Memory and Aging Center, University of California, San Francisco, California, USA; ^6^Neurology Unit, Neurorehabilitation Unit, Neurophysiology Service, and Neuroimaging Research Unit, Division of Neuroscience, IRCCS San Raffaele Scientific Institute, and Vita‐Salute San Raffaele University, Milan, Italy

**Background and aims:** This study provides an Italian scale to assess motor speech disorders (MSD) in patients with primary progressive aphasia (PPA) and progressive supranuclear palsy (PSP).

**Methods:** The motor speech evaluation scale (MSE) evaluates: sustained /a/, alternating and sequential motion rates (AMR, SMR), counting, and repeating 8 words 5 times each. MSE was administered to 75 patients (59 PPA, 16 PSP) and 63 healthy controls (HC‐MSE). Sixty‐seven patients and 61 matched HC (HC‐MRI) also underwent an MRI scan. Analysis included duration, voice quality, articulatory rate (AR), and accuracy, along with the average number of syllables (ANS) per repeated word. Patients were classified as having apraxia of speech (AOS, N=20), dysarthria (DYS, N=11), mixed conditions (MIX, N=7), or non‐MSD (N=37). Sequential Feature Selection (SFS) identified parameters differentiating MSD subtypes. Voxel‐based morphometry assessed grey matter (GM) atrophy in MSD cases.

**Results:** SFS distinguished AOS from non‐MSD considering age, AR of microscopico, and accuracy of artiglieria and segregazione (R^2^=1.00). Non‐MSD and DYS differed in AR of pagoda and ANS of artiglieria (R^2^=0.92). Age, ANS of pagoda, and AR of microscopico differentiated non‐MSD and MIX (R^2^=1.00). AOS and DYS differed in voice quality, AR of /papa/, SMR and spaghetti tasks, accuracy for /papa/, and ANS of artiglieria (R^2^=0.90). Compared to HC‐MRI, AOS cases showed left motor and premotor atrophy, while MIX cases left inferior frontal damage.

**Conclusion:** This study provides the first Italian scale to evaluate MSD in PPA and PSP. The MSE distinguishes AOS, DYS, and their co‐occurrence, supporting its use in neurodegenerative conditions.

**Disclosure:** Funding. European Research Council (StG‐2016_714388_NeuroTRACK); Foundation Research on Alzheimer Disease. Co‐funding by the Next Generation EU [DM 1557 11.10.2022]. Disclosures. LL, AR, VC, ES, CT, FF, TG, EGS, MLGT nothing to disclose; EC grants from Italian Ministry of Health (MSAL); GC speaker fees from Neopharmed Gentili; MF consulting or speaking fees from Alexion, Almirall, Bayer, Biogen, Celgene, Chiesi Italia SpA, Eli Lilly, Genzyme, Janssen, Merck‐Serono, Neopharmed Gentili, Novartis, Novo Nordisk, Roche, Sanofi Takeda, and TEVA; Advisory Boards for Alexion, Biogen, Bristol‐Myers Squibb, Merck, Novartis, Roche, Sanofi, Sanofi‐Aventis, Sanofi‐Genzyme, Takeda; scientific direction of educational events for Biogen, Merck, Roche, Celgene, Bristol‐Myers Squibb, Lilly, Novartis, Sanofi‐Genzyme; he receives research support from Biogen Idec, Merck‐Serono, Novartis, Roche, MSAL, Italian Ministry of University and Research (MUR), and FISM. FA is Associate Editor of NeuroImage: Clinical, has received speaker honoraria from Biogen Idec, Roche, Eli Lilly and GE Healthcare, and receives or has received research supports from MSAL, MUR, AriSLA, ERC, the EU Joint Programme – Neurodegenerative Disease Research, and Foundation Research on Alzheimer Disease (France).

## EPO‐053

### Obstructive sleep apnea severity is associated with operation but not maintenance deficits in working memory

#### L. Huang ^1^; Y. Long^2^; Y. Jin^1^; X. Ren^3^; Y. Bie^3^; Z. Sun^4^; X. He^1^

##### ^1^Departments of Psychology, University of Science and Technology of China, Hefei, China; ^2^Departments of Geriatrics, The First Affiliated Hospital of University of Science and Technology of China, Hefei, China; ^3^Department of Otolaryngology‐Head and Neck Surgery, The First Affiliated Hospital of University of Science and Technology of China, Hefei, China; ^4^Department of Neurology, First Affiliated Hospital of Anhui Medical University, Hefei, China

**Background and aims:** Obstructive sleep apnea (OSA) is a highly prevalent sleep disorder often linked to cognitive deficits, particularly impairments in working memory (WM). Baddeley's model suggests WM involves maintaining and operating information, with distinct functional characteristics and neural underpinnings. However, it remains unclear how OSA compromises WM, particularly its key components. Hence, we explored the association of OSA severity and deficits in different aspects of WM.

**Figure:**
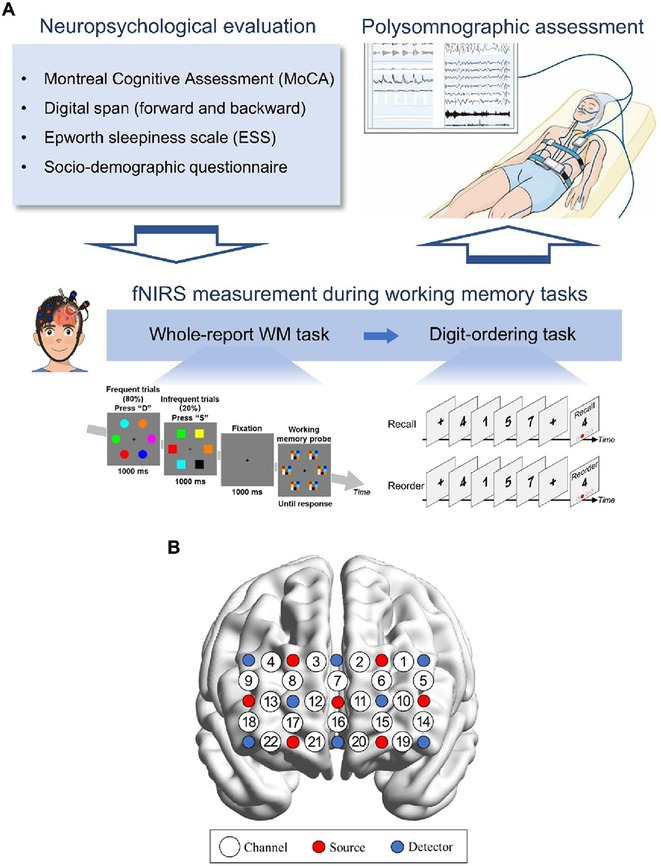
**FIGURE 1** (A) Study procedures and experimental design. (B) Spatial profiles of fNIRS channels.

**Methods:** Twenty‐three male participants with suspected OSA (Mage = 34.0 years) sequentially underwent neuropsychological evaluation, WM tasks, and polysomnographic assessment (Fig. 1A). During the WM tasks, prefrontal cortex activities were recorded using a 22‐channel functional near‐infrared spectroscopy (fNIRS), and quantified via fractional amplitude of low‐frequency fluctuations (fALFF) (Fig. 1B). WM maintenance and operation were evaluated using the whole‐report WM task and the digit‐ordering task, respectively. The apnea‐hypopnea index (AHI), nadir of oxygen saturation (SpO2), and the Epworth Sleepiness Scale (ESS) were used to quantify the severity of OSA.

**Results:** The severity of OSA was found to be significantly associated with deficits in WM operation (rESS = 0.476, p = 0.026), but not with WM maintenance (Fig. 2). A hierarchical regression model, including demographic characteristics, SpO2 nadir, and fALFF of channel 22, explained a significant proportion of the variance in WM operation (Adj. R2 = 0.40, p = 0.004).

**Figure:**
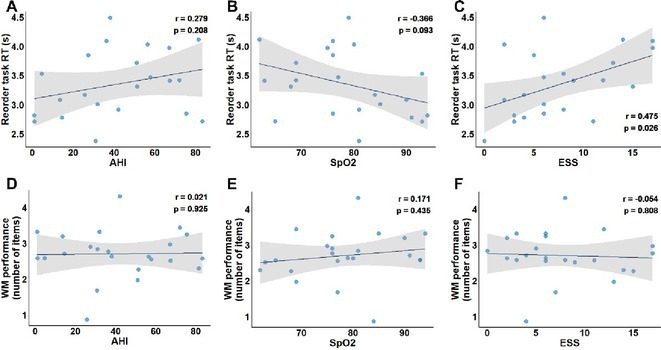
**FIGURE 2** Correlation analysis of the severity of OSA and participants’ performance in the digit‐ordering task (A‐C) and the whole‐report WM task (D‐F).

**Conclusion:** Worsen OSA is associated with slower response during WM operation, but not with the maintenance of WM, potentially due to reduced spontaneous activity in the prefrontal cortex.

**Disclosure:** Nothing to disclose.

## EPO‐054

### French version of the Northwestern Anagram Test to assess syntactic impairment in primary progressive aphasias

#### L. Bavelier ^1^; S. Ferrieux^1^; L. Grimont^1^; I. de Marcellus^1^; C. Rebillard^1^; M. Lannes^2^; C. Rousseau^2^; F. Puppo‐Capodano^1^; A. Rametti‐Lacroux^1^; I. Le Ber^3^; M. Teichmann^1^; D. Saracino^3^

##### ^1^Institute of Memory and Alzheimer Disease (IMMA), Reference Centre for Rare or Early Onset Dementias, Department of Neurology, AP‐HP ‐ Hôpital Pitié‐Salpêtrière, Paris, France; ^2^Sorbonne Université, Paris Brain Institute – Institut du Cerveau – ICM, Inserm U1127, CNRS UMR 7225, AP‐HP ‐ Hôpital Pitié‐Salpêtrière, Paris, France; ^3^Sorbonne Université, Paris Brain Institute – Institut du Cerveau – ICM, Inserm U1127, CNRS UMR 7225 and IMMA, Reference Centre for Rare or Early Onset Dementias, Department of Neurology, AP‐HP ‐ Hôpital Pitié‐Salpêtrière, Paris, France

**Background and aims:** Primary progressive aphasias (PPA) are neurodegenerative conditions including three main variants: nonfluent/agrammatic (nfa), semantic, and logopenic PPA. Several tests allow for assessing the linguistic core features for stringently classifying semantic and logopenic PPA, but there is no internationally available tool to quantify grammatical‐syntactic disorders guaranteeing correct classification of nfa‐PPA. The Northwestern Anagram Test (NAT) aimed at filling this gap. We aimed at validating its French version (NAT‐F).

**Methods:** The NAT‐F was applied to 10 PPA patients, 10 Alzheimer's disease (AD) patients and 50 healthy controls, using a digital tablet application (Figure 1). The NAT‐F was adapted from the initial English and Italian versions to probe for different French syntax structures (items) including canonical/active and non‐canonical/passive sentences. To provide a rapid testing procedure, we shortened the original English version generating and comparing two 22‐item versions (NAT‐F1, NAT‐F2).

**Figure:**
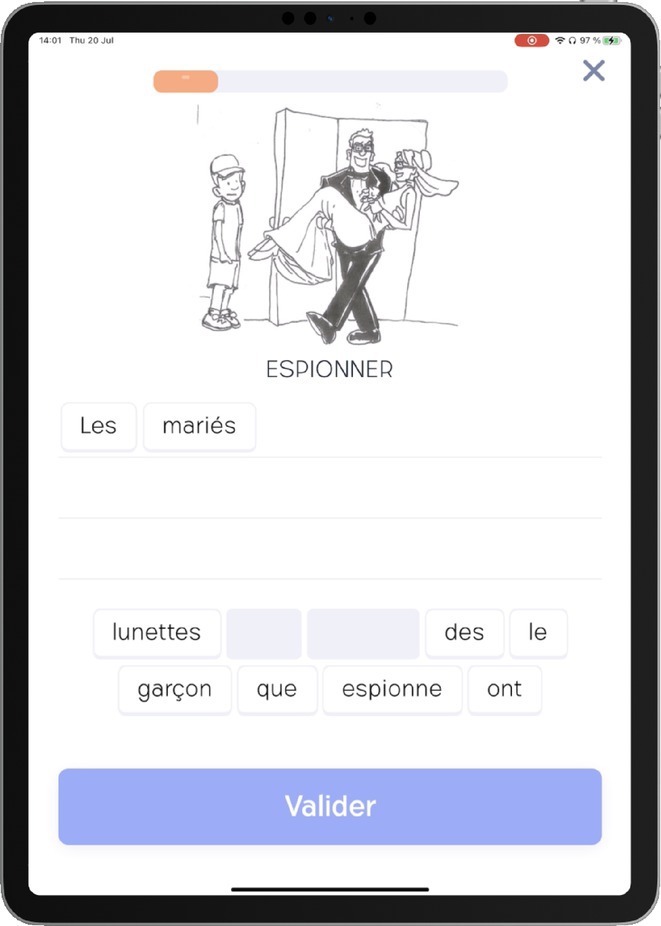
**FIGURE 1** Example of a NAT‐F item, featuring a non‐canonical object‐related sentence. The infinitive form of the verb is indicated and the words are provided in scrambled order. The task consists in reordering then within 60 seconds.

**Results:** Patients and controls were comparable for age (p=0.07), sex (p=0.56) and educational level (p=0.47). Performance of controls was correlated between NAT‐F1 and NAT‐F2 for both total scores (rho=0.61, p<0.0001) and item‐specific scores (p<0.001). NAT‐F scores of both versions were lower in PPA and AD patients compared to controls (p<0.001, Figure 2), especially for non‐canonical items. Within non‐canonical items, performance with passive sentences was poorer in PPA than in AD patients (p=0.002), and within PPA variants there was a trend for poorest performance in nfa‐PPA with passive and object‐relative sentences.

**Figure:**
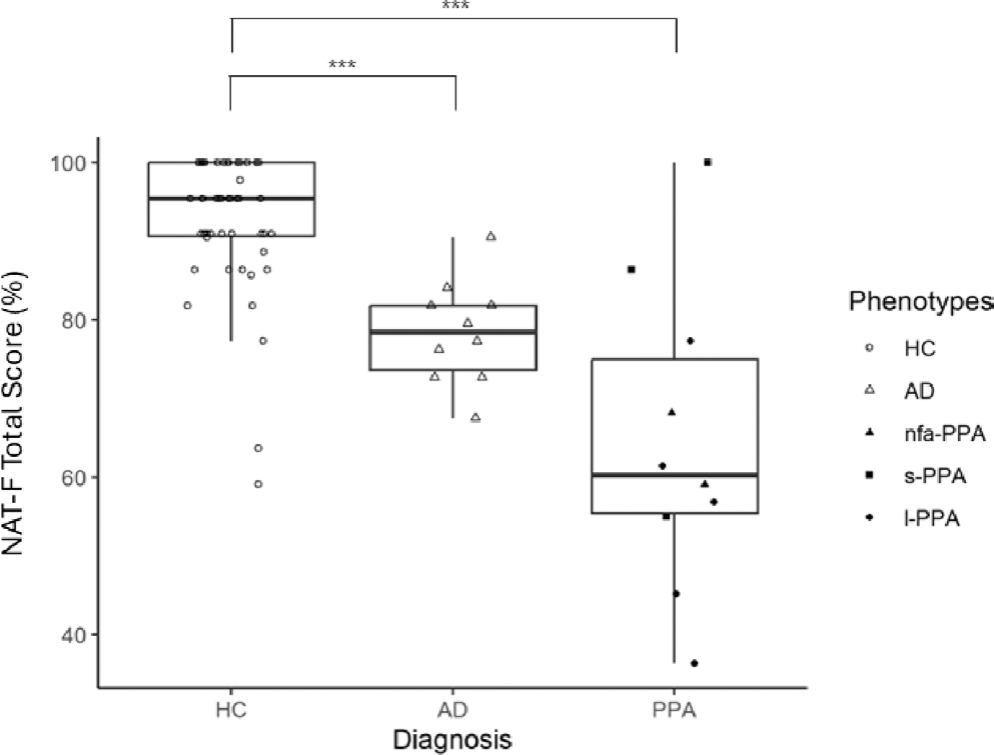
**FIGURE 2** Comparisons of NAT‐F total scores between the diagnostic groups (Kruskal‐Wallis and Dunn's post hoc test). ***: p < 0.0001. AD: Alzheimer's disease; HC: healthy controls; l‐PPA: logopenic PPA; nfa‐PPA: nonfluent/agrammatic PPA; s‐PPA: semantic PPA.

**Conclusion:** NAT‐F provides an international and clinically‐relevant tool for test‐based classification of nfa‐PPA. Definitive validation requires the assessment and comparison of larger populations of the three PPA main variants.

**Disclosure:** Nothing to disclose.

## EPO‐055

### Towards a functional protective model for the prodromal stage of alpha‐synucleinopathies

#### G. D'Este^2^; G. Carli^3^; C. Leitner^1^; E. Cini^1^; F. Berra^1^; A. Castelnuovo^1^; S. Marelli^2^; M. Zucconi^2^; F. Casoni^2^; A. Galbiati^1^; L. Ferini‐Strambi ^1^

##### ^1^Department of Psychology, “Vita‐Salute” San Raffaele University, Milan, Italy; ^2^Sleep Disorders Centre, Department of Clinical Neurosciences, Neurology, IRCCS San Raffaele Scientific Institute, Milan, Italy; ^3^Neurology department, university of Michigan, Ann Arbor, USA

**Background and aims:** Isolated Rapid Eye Movement Sleep Behavior Disorder (iRBD) is an early stage of synucleinopathies. While Slow Wave (SW) sleep is renowned for its protective effects against degeneration, its specific role in synucleinopathy‐related neurodegeneration remains poorly understood. Parkinson's disease‐related pattern (PDRP) expression on 18fluorodeoxyglucose PET ([18F]FDG‐PET) is a marker of neurodegeneration in iRBD. We aimed to investigate between SW and PDRP alongside cognitive performance in iRBD, with an exploratory focus on cognitive reserve (CR).

**Methods:** 41 polysomnography‐confirmed iRBD patients underwent [18F]FDG‐PET, neuropsychological assessment and CR Index questionnaire (CRIq). The PDRP expression was computed for each patient. SW density (SWd) and Slow Oscillations density (SOd) were calculated on frontal EEG derivations in N2 and N3 sleep with an automated algorithm.

**Results:** SWd and SOd (in N2 and N3 sleep) were positively correlated with cognitive screening and visuospatial abilities domains. PDRP was positively correlated with the TMT A score and negatively correlated with SWd in N3 sleep (all p<0.05). iRBD with Mild Cognitive Impairment (MCI) showed a reduction in SWd and SOd in both N2 and N3 sleep in comparison to patients without MCI (all p<0.05), but no difference in PDRP expression. Patients with MCI showed a non‐significant reduction at CRIq in comparison to cognitively normal patients.

**Figure:**
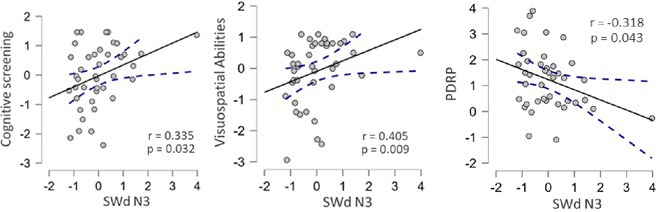
**FIGURE 1** Correlation analysis between SW sleep indexes, neuropsychological data and PDRP.

**Figure:**
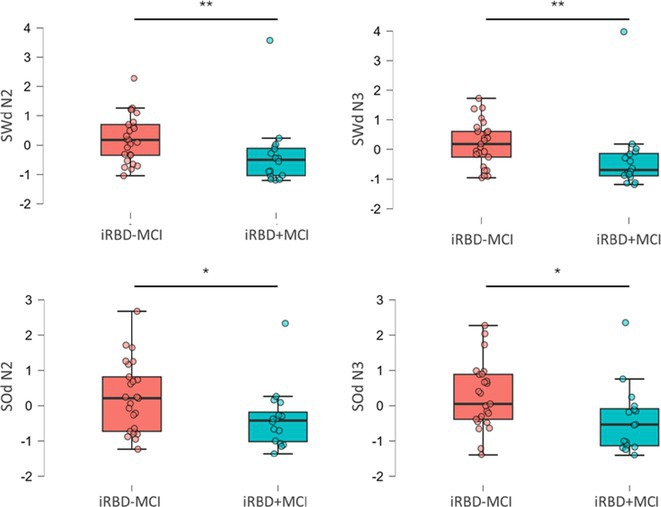
**FIGURE 2** Comparisons between iRBD‐MCI and iRBD+MCI patients on SW sleep indexes.

**Figure:**
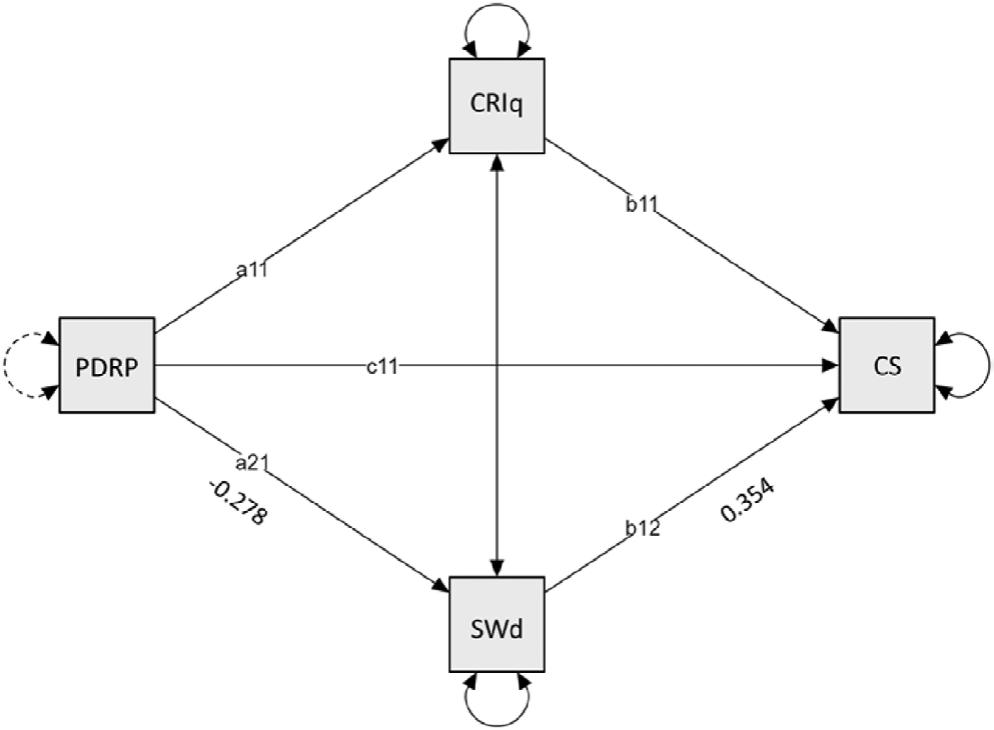
**FIGURE 3** Putative model for testing CR.

**Conclusion:** Our findings highlight a novel interaction between pathological burden and SW sleep dynamics in iRBD, which is also closely tied to cognitive deficits manifestation. Future longitudinal studies should investigate whether these associations might be moderated by CR as a possible protective factor against neurodegeneration.

**Disclosure:** Nothing to disclose.

## EPO‐056

### Associations between spatial navigation performance and Alzheimer's disease biomarkers

#### M. Laczó; N. Sapietova; V. Katerina; B. Sarka; V. Martin; J. Hort; J. Laczó

##### Memory Clinic, Department of Neurology, Charles University, Second Faculty of Medicine and Motol University Hospital, Prague, Czechia

**Background and aims:** Spatial navigation impairment is among the earliest cognitive deficits in Alzheimer's disease (AD). This study investigated the association between spatial navigation performance and AD biomarkers in individuals with amnestic mild cognitive impairment (aMCI).

**Methods:** This study included 80 amnestic MCI participants with AD (AD aMCI), 66 aMCI participants without AD (non‐AD aMCI), and 72 cognitively normal (CN) adults. Spatial navigation performance was assessed using real‐space and computerized versions of the human analogue of the Morris Water Maze task, which measured allocentric, egocentric, and delayed allocentric navigation performance. All participants underwent neuropsychological and brain assessments. Biomarker data for cerebrospinal fluid (CSF) levels of amyloid‐beta1‐42, total tau, phosphorylated tau181, and amyloid PET imaging were collected for all aMCI participants.

**Results:** On the real and virtual allocentric and delayed allocentric tasks, both AD and non‐AD aMCI participants performed worse than the CN group (p<=0.018), with AD aMCI participants performing worse than non‐AD aMCI participants (p=0.014). On the real and virtual egocentric tasks, AD aMCI participants performed worse than the CN group (p<=0.015), while the non‐AD aMCI and CN groups showed similar performance. On the virtual egocentric task, AD aMCI participants performed worse than the non‐AD aMCI participants (p<0.001), Performance on the real, virtual, and delayed allocentric tasks and the virtual egocentric task was associated with lower amyloid‐beta1‐42 levels and a higher p‐tau181/amyloid‐beta1‐42 ratio (beta≥0. 174, p<=0.015).

**Conclusion:** Spatial navigation impairments, particularly allocentric, are strongly linked to AD biomarkers in aMCI. These tasks are promising non‐invasive tools for early detection and monitoring of AD‐related pathology.

**Disclosure:** National Institute for Neurological Research (Programme EXCELES, ID Project No. LX22NPO5107) – Funded by the European Union – Next Generation EU, and the Institutional Support of Excellence 2 2. LF UK (Grant No. 6980382).

## EPO‐057

### Preoperative magnesium levels and risk of postoperative delirium in elderly non‐cardiac surgery patients: A cohort study

#### M. Yuan; X. Zhang; W. Mi

##### Anesthesia Surgery Center, PLA General Hospital, Beijing, China

**Background and aims:** Postoperative delirium (POD), common in elderly patients, is linked to neuroinflammation, oxidative stress, and synaptic dysfunction. This study explored the association between preoperative magnesium (Mg) levels and POD risk, aiming to identify optimal levels and underlying mechanisms.

**Methods:** This retrospective cohort study included patients ≥65 undergoing non‐neurosurgical, non‐cardiac procedures (2014–2021). Preoperative Mg levels were measured within 30 days before surgery, and POD occurrence within seven days post‐surgery was assessed using standardized criteria. Logistic regression adjusted for confounders (via DAG) examined the Mg‐POD relationship. Mg levels were divided into quintiles, with RCS analyses for nonlinear associations and subgroup analyses by sex, age, renal dysfunction, and diabetes.

**Results:** 53445 patients were analyzed, with 1,551 (2.9%) developing POD. Multivariable logistic regression showed Mg as a continuous variable was inversely associated with POD (OR=0.89, 0.85–0.93, p<0.001). Quintile analysis revealed a linear trend, with the lowest POD risk at Mg=0.89 mmol/L. Below it, each SD increase reduced POD risk (OR=0.75, 0.71–0.79); above it, risk increased (OR=1.04, 0.97–1.12). Adjusting for albumin transformed the U‐shaped association into a linear one, identifying albumin as a key confounder. Mediation analysis demonstrated albumin partially mediated the Mg‐POD relationship (direct effect c’=−0.047, indirect effect a*b=−0.022, both p<0.001), explaining 31.88% of the effect. Mediation was stronger in patients with renal insufficiency (57.14%) and diabetes (21.84%).

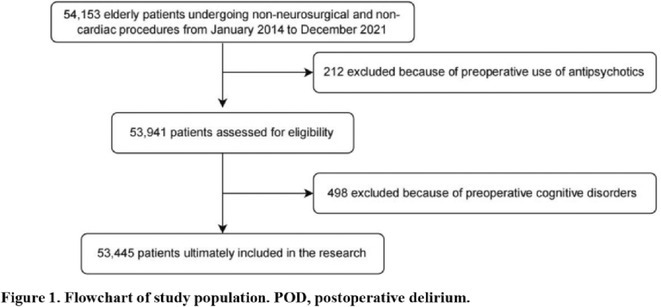

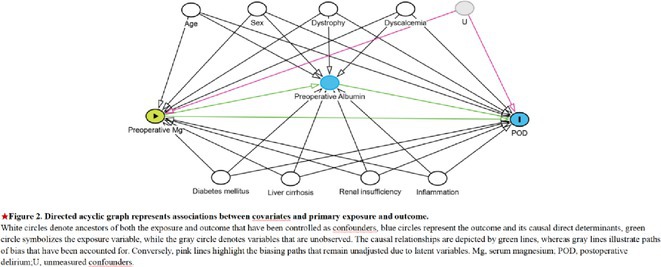

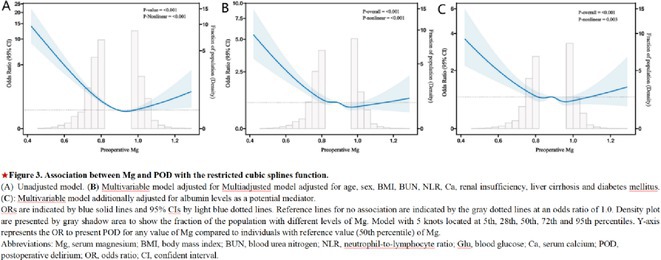

**Conclusion:** Maintaining optimal Mg levels may reduce POD risk, particularly in vulnerable populations.

**Disclosure:** Nothing to disclose.

## EPO‐059

### Machine Learning Prediction of non‐dementia cognitive symptoms using clinical, genetics, cognitive and imaging data

#### V. Cabreira

##### Centre for Clinical Brain Sciences, University of Edinburgh, Edinburgh, UK

**Background and aims:** Non‐neurodegenerative disorders can present with cognitive symptoms and superficially resemble dementia. Current diagnostic methods lack precision and standardisation. We assessed whether Machine learning (ML) models accurately predict non‐dementia‐related cognitive symptoms (functional cognitive disorder, FCD).

**Methods:** The UK Biobank contains 500,000 participants and clinical, cognitive and physical measurements, and imaging data. Participants were followed between 2006 and 2023. Participants with daily or almost daily cognitive symptoms were selected, excluding patients with dementia at baseline and other neurological disorders. Dementia at follow‐up was derived using linked health records. Five machine learning methods (random forest, decision tree, c50, logistic regression and Extreme Gradient Boosting) were developed, with ten‐fold cross‐validation. Performance metrics (internal validation) included accuracy, area under the receiver operating characteristic curve (AUC), sensitivity, specificity, and F1 score. The effect of individual qualitative and quantitative features in correctly predicting FCD was explored.

**Results:** 11862 participants (1407 dementias, 10455 FCD), with a mean age of 57‐years‐old (46% male). The optimal model (random forest) demonstrated an accuracy of 0.85, AUC of 0.87, sensitivity of 0.88, specificity of 0.61, PPV of 0.95, and an F1 score of 0.91. Age at recruitment, traumatic and adverse life events, and polygenic risk score were the most important predictors among 38 best performing variables.

**Figure:**
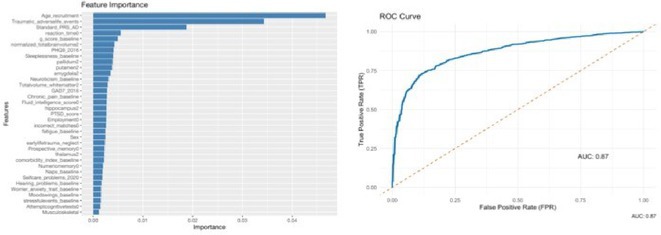
**FIGURE 1** A, Feature importance graph for the Random Forest classification model. B, The receiver operating characteristic curve for the best‐performing random forest model. AUC: area under the curve.

**Figure:**
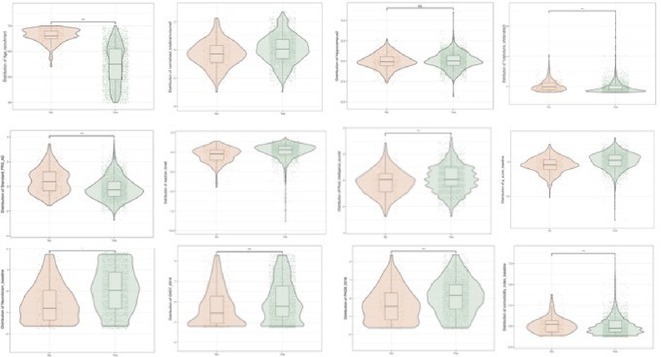
**FIGURE 2** Quantitative measures. Differences in age at recruitment, normalized total brain volume, hippocampal volume, total volume of white matter changes, polygenic risk scores (PRS) for Alzheimer’s disease, reaction time, fluid intelligence score, global cognitive function (g‐score), neuroticism score, anxiety (GAD‐7), depressive symptoms (PHQ‐9) and comorbidity index.

**Figure:**
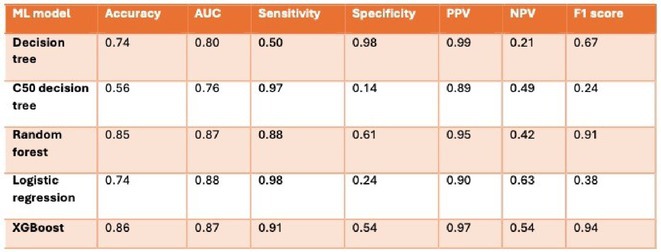
**TABLE 1** Performance of Machine learning models on a dataset of clinical history, cognitive, imaging and genetic data to discriminate dementia from FCD among participants with disabling cognitive symptoms.

**Conclusion:** Robust machine learning model performance may allow for early identification of individuals with non‐progressive cognitive symptoms (FCD). The use of routine clinical data is promising to aid clinical decision‐making with prognostic implications, and research studies. Prospective validation of these models in independent datasets is required to improve generalizability.

**Disclosure:** Nothing to disclose.

## Headache 1

## EPO‐060

### Is cognitive dysfunction an inevitable outcome in migraine?

#### E. Koçhan Kızılkılıç; B. Kılboz; S. Üçler

##### University of Health Sciences, Prof. Dr. Cemil Taşcıoğlu City Hospital, Department of Neurology, Istanbul, Turkey

**Background and aims:** Cognitive dysfunction, though not traditionally considered a core symptom of migraine, significantly impacts patients’ daily lives, particularly during attacks. This study evaluates the neuropsychological performance of patients with episodic and chronic migraine by comparing their baseline and attack‐period performance with healthy controls.

**Methods:** Thirty‐two patients with episodic migraine, 32 with chronic migraine, and 30 age‐ and gender‐matched healthy controls were included. Demographic data, HIT‐6 scores and headache characteristics were recorded. The Mini‐Mental State Examination, Beck Depression Inventory (BDI), Stroop Test, Clock Drawing Test, Forward and Backward Digit Span Tests (DST) were administered during attacks and repeated interictally. Healthy controls completed the same tests. Statistical evaluation included frequentist statistics, Spearman correlations, and beta regression.

**Results:** HIT‐6 scores were higher in the chronic migraine group compared to the episodic group. Chronic migraine patients had higher baseline BDI scores and lower DST scores than controls. All six tests showed significantly worse performance during attacks in both migraine groups (Figure 1). Attack severity correlated with cognitive impairment, particularly in the episodic migraine group (Figure 2). Beta regression revealed each monthly attack added 1.58 points to HIT‐6 scores, peaking at 20 attacks per month.

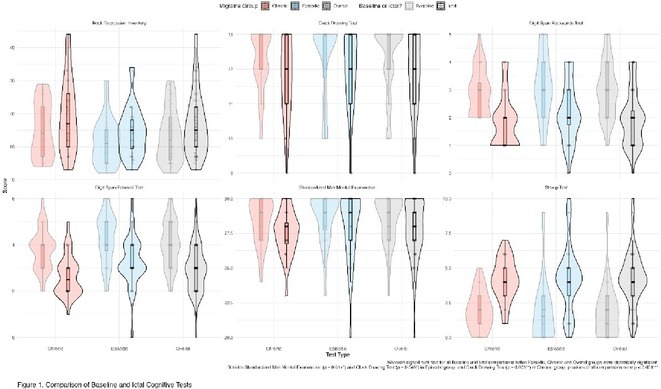

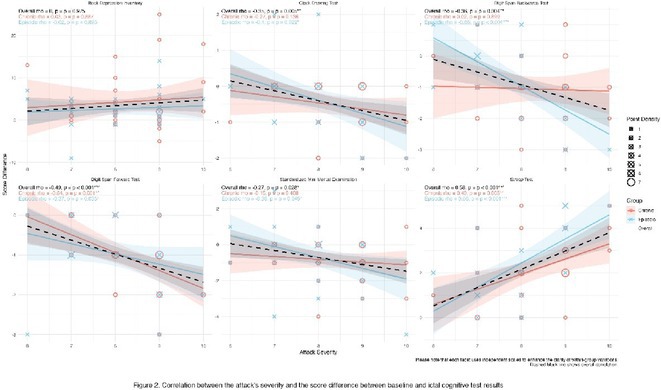

**Conclusion:** Cognitive impairment during ictal and interictal periods significantly contributes to migraine‐related disability, with attack frequency worsening the burden. Clinicians should monitor cognitive performance and adjust treatment plans to address this impact.

**Disclosure:** Nothing to disclose.

## EPO‐061

### Headache acceptance and migraine‐related disability: Validation of the Turkish headache acceptance questionnaire

#### E. Koçhan Kızılkılıç; B. Kılboz; M. Karahoca; E. Ünal; T. Aydın; S. Üçler

##### Department of Neurology, Prof. Dr. Cemil Taşcıoğlu City Hospital, Istanbul, Turkey

**Background and aims:** Mindfulness‐based therapies, such as Acceptance and Commitment Therapy (ACT), show promise in helping individuals lead active lives despite chronic pain. While pain acceptance has been studied in chronic pain, headache‐specific tools are needed due to the unique features of headaches. This study evaluated the validity and reliability of the Turkish version of the Headache Acceptance Questionnaire (HAQ), designed to measure headache‐related pain acceptance, and examined its relationship with migraine‐related disability.

**Methods:** From February to December 2024, migraine patients were assessed using the HAQ, HIT‐6, and MIDAS scales. Validation incorporated content validity, structural validity through factor analyses, and reliability testing using Cronbach's alpha and ICC. Correlations between HAQ scores and migraine‐related disability were analyzed.

**Results:** Among 184 patients (156 females) the Turkish HAQ demonstrated excellent content validity and strong structural validity (KMO=0.89, Bartlett's p<0.001). Reliability was excellent, with a Cronbach's alpha of 0.909 and ICC of 0.99 (p<0.001). HAQ scores were strongly and negatively correlated with HIT‐6 (r=‐0.86, p<0.001) and MIDAS (ρ=‐0.73, p<0.001).

**Figure:**
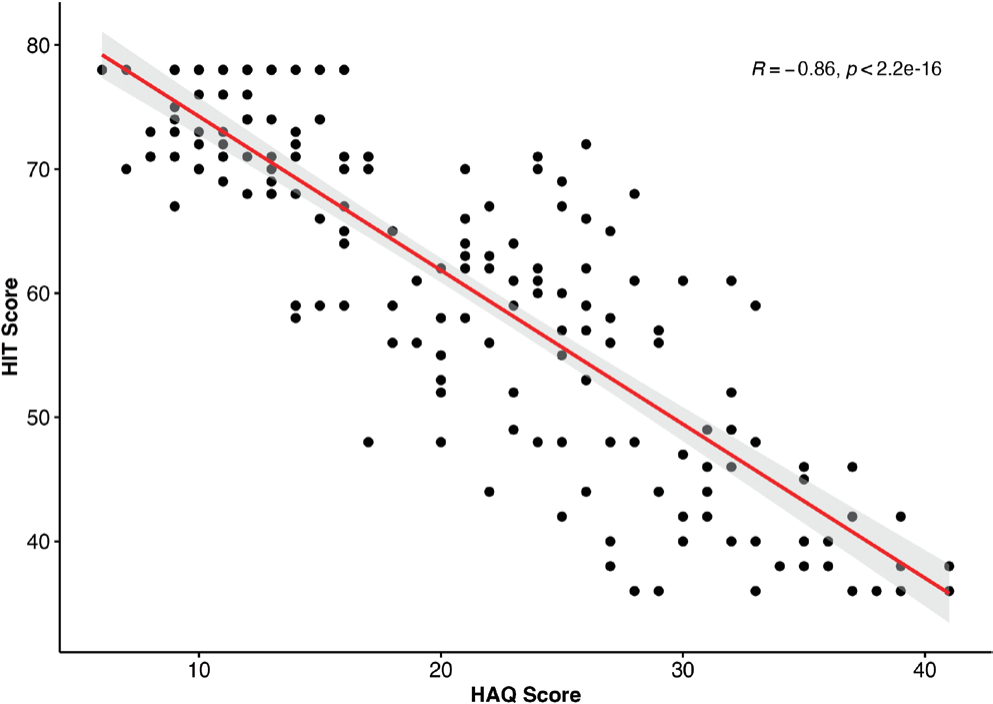
**FIGURE 1** Correlation between HAQ Score and HIT‐6 Score.

**Figure:**
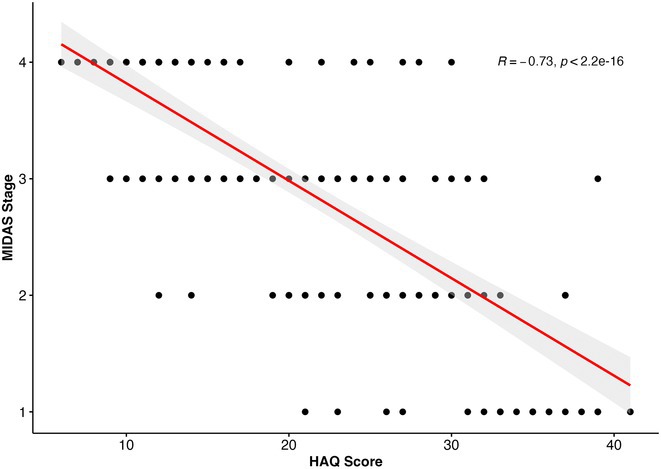
**FIGURE 2** Correlation between HAQ Score and MIDAS Stage.

**Conclusion:** The Turkish HAQ is a valid and reliable tool for assessing headache‐specific pain acceptance. The strong inverse relationship between headache acceptance and disability highlights the clinical importance of acceptance‐based therapies, such as ACT, in managing migraine.

**Disclosure:** Nothing to disclose.

## EPO‐063

### Real‐world safety and tolerability of fremanezumab in migraine prevention: Final outcomes of the PEARL study

#### M. Ashina^1^; D. Mitsikostas^2^; F. Amin^1^; P. Kokturk^3^; C. Schankin^4^; G. Sahin^5^; P. Pozo‐Rosich^6^; P. Dorman^7^; T. Nežádal^8^; I. Pavão Martins^9^; M. Sumelahti^10^; V. Ramirez Campos^11^; H. Akcicek^3^; C. Tassorelli ^12^

##### ^1^Department of Neurology, Danish Headache Center, Copenhagen University Hospital– Rigshospitalet Glostrup, Copenhagen, Denmark; Department of Clinical Medicine, University of Copenhagen, Copenhagen, Denmark; ^2^Department of First Neurology, Aeginition Hospital, National and Kapodistrian University of Athens, Athens, Greece; ^3^Teva Netherlands B.V, Haarlem, The Netherlands; ^4^Department of Neurology, Inselspital, University Hospital Bern, University of Bern, Bern, Switzerland; ^5^Department of Clinical Sciences of Lund, Lund University, Skåneuro Neurology Clinic, Lund, Sweden; ^6^Headache Unit and Research Group, Vall d’Hebron Hospital and Research Institute, Universitat Autonoma de Barcelona, Barcelona, Spain; ^7^The Newcastle upon Tyne Hospitals NHS Foundation Trust, Newcastle upon Tyne, UK; ^8^Institute of Neuropsychiatric Care, First Faculty of Medicine, Charles University, Prague, Czechia; ^9^Centro de Estudos Egas Moniz, Faculty of Medicine, University of Lisbon, Lisbon, Portugal, ^10^Faculty of Medicine and Health Technology, University of Tampere, Tampere, Pirkanmaa, Finland, ^11^Teva Branded Pharmaceutical Products R&D, Inc., West Chester, USA, ^12^Department of Brain and Behavioral Sciences, University of Pavia, Pavia, Italy; IRCCS C. Mondino Foundation, Pavia, Italy

**Background and aims:** PEARL (EUPAS35111) is a 24‐month observational, prospective, Phase 4 study evaluating the real‐world effectiveness and safety of fremanezumab for episodic and chronic migraine (EM, CM) prevention. Here we present safety and tolerability outcomes from the PEARL study after 24 months of follow up.

**Methods:** Eligible participants were adults with EM or CM receiving fremanezumab for migraine prevention, who maintained a daily headache diary prior to and throughout the study period. Primary endpoint: the proportion of participants with >=50% reduction in monthly migraine days (MMD) during the 6‐month period after fremanezumab initiation. Safety and tolerability were assessed by adverse event (AE) reporting.

**Results:** In total, 637/1128 (56.5%) participants with available data achieved the primary endpoint. All enrolled participants (N=1140) were included in the safety analysis. Overall, 642/1140 (56.3%) participants reported >=1 AE (Table 1). Drug‐related AEs were reported by 376 (33.0%) participants; the most common being general disorders and administration site conditions (n=253, 22.2%) and gastrointestinal disorders (n=79, 6.9%; Table 1). Drug‐related serious AEs were infrequent, occurring in four (0.4%) participants; the most common was drug hypersensitivity (n=2, 0.2%). AEs leading to treatment discontinuation were reported by 61 (5.4%) participants; the most frequent reasons were drug ineffectiveness (n=36, 3.2%), injection site erythema (n=5, 0.4%) and injection site pruritus (n=4, 0.4%).

**Figure:**
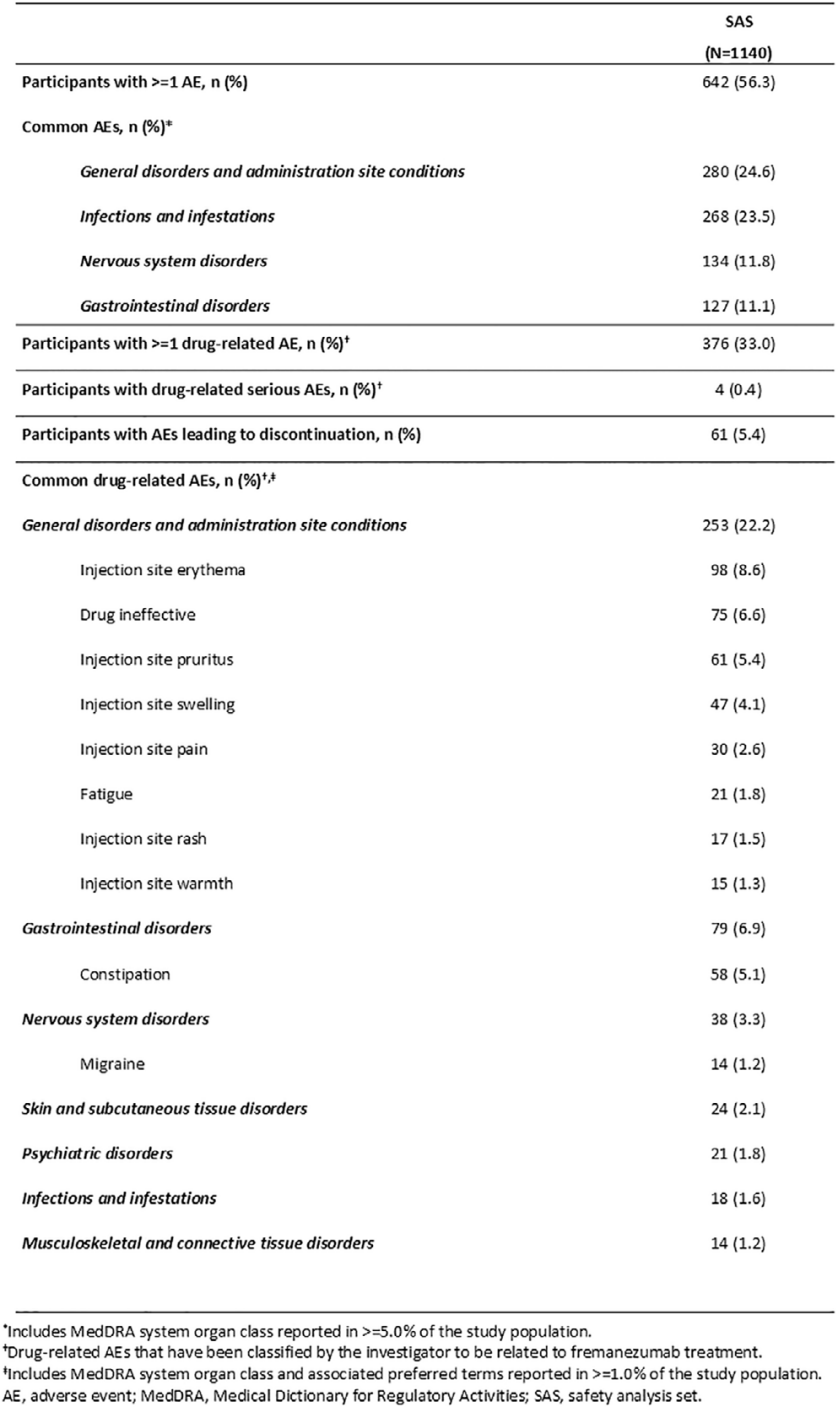
**TABLE 1** Safety analysis.

**Conclusion:** The favourable long‐term safety and tolerability of fremanezumab demonstrated in this analysis are consistent with the known safety profile of fremanezumab from previous PEARL interim analyses and randomised controlled trials, supporting its continued clinical use for migraine prevention.

**Disclosure:** MA: AbbVie, Amgen, AstraZeneca, Eli Lilly, GlaxoSmithKline, Lundbeck, Novartis, Novo Nordisk Foundation, Pfizer, Teva Pharmaceuticals. DM: Allergan, Amgen, Bayer, Biogen, Cefaly, electroCore, Eli Lilly, Genesis Pharma, Merck Serono, Merz, Mylan, Novartis, Roche, Sanofi Genzyme, Specifar, Teva Pharmaceuticals. FMA: AbbVie, Eli Lilly, Lundbeck, Novartis, Pfizer, Teva Pharmaceuticals. CJS: AbbVie, Allergan, Almirall, Amgen, Eli Lilly, Grünenthal, Lundbeck, MindMed, Novartis, Pfizer, Teva Pharmaceuticals, Zynnon, Baasch‐Medicus Foundation, Eye on Vision Foundation, German Migraine and Headache Society. GS: AbbVie, Lundbeck, Novartis, Pfizer, Teva Pharmaceuticals, Vinnova, Lund University, Swedish Neurological Association. PPR: AbbVie, Amgen, Biohaven EraNet NEURON, Chiesi, Eli Lilly, Lundbeck, Instituto Investigación Carlos III, MINECO, Novartis, Pfizer, RIS3CAT FEDER, Teva Pharmaceuticals. PJD: AbbVie, electroCore, Eli Lilly, Lundbeck, Novartis, Pfizer, Teva Pharmaceuticals. TN: AbbVie, Amgen, Eli Lilly, Glenmark, Lundbeck, Neurocrine Novartis, Organon, Pfizer, Teva Pharmaceuticals, UCB. IPM: AbbVie, Allergan, Eli Lilly, Lundbeck, Novartis, Organon, Pfizer, Teva Pharmaceuticals. MLS: AbbVie, Eli Lilly, Lundbeck, Novartis, Pfizer, Teva Pharmaceuticals. CT: AbbVie, Chordate, Dompé, Eli Lilly, Ipsen, Lundbeck, Novartis, Pfizer, Teva Pharmaceuticals, European Commission, Italian Ministry of Health, Migraine Research Foundation. PK, VRC, HA, study funding: Teva Pharmaceuticals.

## EPO‐064

### sNfL and GFAP levels in idiopathic intracranial hypertension: an exploratory study

#### N. Krajnc ^1^; S. Macher^1^; M. Michl^2^; N. Müller^1^; S. Zaic^1^; C. Mitsch^2^; W. Marik^3^; K. Novak^4^; C. Wöber^1^; B. Pemp^2^; G. Bsteh^1^

##### ^1^Department of Neurology, Medical University of Vienna, Vienna, Austria; ^2^Department of Ophthalmology, Medical University of Vienna, Vienna, Austria; ^3^Department of Neuroradiology, Medical University of Vienna, Vienna, Austria; ^4^Department of Neurosurgery, Medical University of Vienna, Vienna, Austria

**Background and aims:** Idiopathic intracranial hypertension (IIH) is a systemic disorder marked by increased intracranial pressure carrying the risk of blindness due to optic nerve damage. Serum neurofilament light chain (sNfL) and glial fibrillary acidic protein (GFAP) are emerging biomarkers of axonal damage and astrocytic activation, respectively.

**Methods:** From an ongoing prospective observational study including pwIIH, sNfL and GFAP levels were measured at baseline using single‐molecule array (Simoa) technology, and analyzed as Z‐scores adjusting for age, BMI and – for GFAP – sex. The ophthalmological outcomes included papilledema degree, visual outcomes (visual acuity, visual field), optical coherence tomography and transbulbar sonography.

**Results:** We included 23 pwIIH (mean age 34.3 years (SD 8.1), 95.7% female, median cerebrospinal fluid (CSF) opening pressure 33.0 cmCSF (IQR 26.9–35.4), median body mass index (BMI) 35.7 kg/m2 (IQR 31.1–43.3)). Mean sNfL and GFAP Z‐scores at baseline were 1.0 (1.0) and 0.5 (1.4), respectively. sNfL Z‐scores at baseline exhibited a non‐significant positive correlation with the GCL volume of the worse eye (r=0.38, p=0.079), whereas GFAP Z‐scores showed no correlation with any ophthalmological outcomes. Additionally, neither sNfL nor GFAP Z‐scores correlated with the CSF opening pressure.

**Conclusion:** sNfL might be associated with GCL volume, an established sensitive marker of optic nerve damage. In contrast, GFAP does not appear to correlate with any ophthalmological outcomes. Given that IIH entails impaired CSF homeostasis, the lack of correlation might be attributable to impaired outflow of biomarkers from the CSF into the bloodstream.

**Disclosure:** All authors declare no conflict of interest relevant to this study.

## EPO‐065

### Acute Trigeminal Autonomic Cephalalgia headache service: An effective rapid‐access pathway for headache patients

#### P. Amarasena; L. Alves; M. Njohjam; W. Sulaiman; M. Villar‐Martinez; N. Karsan; P. Goadsby

##### Headache Group, Wolfson Sensory, Pain and Regeneration Centre, King's College London, London, UK

**Background and aims:** An acute trigeminal autonomic cephalalgia (TAC) headache service is a rapid access consultation service for patients with TACs offering urgent advice and treatment. We conducted a service evaluation of the interventions offered to assess the effectiveness of this service.

**Methods:** Data from successive consultations (n=419) in the acute TAC headache service from 19/02/2018 to 29/07/2024 were collected from clinic letters as part of a service evaluation. Data were summarized as percentages or as medians with interquartile ranges.

**Results:** Data from 419 consultations of 190 patients were analyzed. Their median age was 49 years (IQR: 38‐58) and 67% were male. The highest number of referrals were received in March (11.5%) and November (9.8%) and 19% were new referrals. The distance to patients’ home ranged from 1 ‐ 423 kms (median 32, IQR: 8 ‐ 103). The majority were due to cluster headache (79%), followed by other primary headache (20%) and secondary headache (1%) disorders. In TAC patients, the median duration of the bout at the time of review was 3 weeks (IQR: 1‐8). Interventions included treatment revisions (51%), greater occipital nerve (GON) injections (13%), a combination of GON injection and treatment revisions (24%) or advice alone (11%). Interventions were effective in 62% of patients.

**Conclusion:** An acute TAC headache service is effective for patients with primary headache disorders, especially for cluster headache. This service effectively provides urgent and tailored guidance and treatment to patients with TAC headaches, minimizing the need to visit the emergency department.

**Disclosure:** PA, LA, MN, WS, MV and NK have nothing to disclose relevant to this submission. PJG reports, over the last 36 months, grants from Celgene and Kallyope, and personal fees from Aeon Biopharma, Abbvie, Aurene, CoolTech LLC, Dr Reddy's, Eli‐Lilly and Company, Linpharma, Lundbeck, Pfizer, PureTech Health LLC, Satsuma, Shiratronics, Teva Pharmaceuticals, Tremeau, and Vial, and personal fees for advice through Gerson Lehrman Group, Guidepoint, SAI Med Partners, Vector Metric, and fees for educational materials from CME Outfitters and WebMD, and publishing royalties or fees from Massachusetts Medical Society, Oxford University Press, UptoDate and Wolters Kluwer, and a patent magnetic stimulation for headache (No. WO2016090333 A1) assigned to eNeura without fee.

## EPO‐066

### Do EHF Criteria reflect response to acute treatments in resistant and refractory migraine? The REFINE study

#### I. Gragnaniello ^1^; R. Ornello^1^; A. Onofri^1^; C. Rosignoli^1^; V. Caponnetto^1^; D. Bayar^2^; M. Braschinsky^3^; M. Carnovali^4^; M. Gentile^5^; R. Gil‐Gouveia^6^; G. Iaccarino^7^; C. Lampl^8^; A. Leheste^3^; P. Martelletti^9^; C. Mazzanti^9^; D. Mitsikostas^10^; A. Munoz‐Vendrell^11^; R. Oliveira^6^; A. Ozge^2^; I. Pavao Martins^12^; P. Pozo‐Rosich^11^; M. Prudenzano^5^; K. Ryliskiene^13^; S. Sacco^1^

##### ^1^University of L'Aquila, Department of Applied Clinical Sciences and Biotechnology, L'Aquila, Italy; ^2^Mersin University Faculty of Medicine, Department of Neurology, Mersin, Turkey; ^3^Headache Clinic, Tartu, Estonia; ^4^Evangelical Hospital, Unna, Germany; ^5^Centro Cefalee ‐ Clinica Neurologica “L. Amaducci”, Azienda Ospedaliero‐Universitaria Policlinico Consorziale di Bari, Bari, Italy; ^6^Hospital da Luz ‐ Center for Interdisciplinary Research in Health, Universidade Católica Portuguesa, Lisbon, Portugal; ^7^Cefalee e Neurosonologia‐Policlinico Universitario Campus Bio‐medico, Rome, Italy; ^8^Department of Neurology and Headache Medical Centre, Konventhospital Barmherzige Brüder Linz, Linz, Austria; ^9^University Sapienza, Rome, Italy, ^10^First Neurology Department, Aeginition Hospital, Medical School, National and Kapodistrian University of Athens, Athens, Greece, ^11^Headache Unit and Research Group Vall d’Hebron University Hospital and Institute of Research, Universitat Autonoma de Barcelona, Barcelona, Spain, ^12^Faculdade de Medicine and Hospital Universitário de Santa Maria, Centro Hospitalar; Hospital Cuf Tejo, Lisbon, Portugal, ^13^Vilnius University Centre of Neurology ‐ Kardiolitos klinikos Centre of Neurology, Vilnius, Lithuania

**Background and aims:** The European Headache Federation (EHF) defines resistant and refractory migraine by failure of ≥3 (resistant) or all (refractory) pharmacological preventive treatment classes, without considering acute treatments. This study aimed to evaluate whether these definitions also correlate with response to acute treatment.

**Methods:** We conducted a multicenter, prospective, international study (REFINE) to test the EHF definitions in a real‐life setting in 15 European headache centers. The Headache Under Response to Treatment (HURT) Questionnaire assessed the impact of migraine on daily activities and effectiveness of acute medications in patients with resistant, refractory, and non‐resistant non‐refractory (NRNR) migraine.

**Results:** We included 689 patients, of whom 261 (37.9%) had resistant, 73 (10.6%) refractory, and 355 (51.5%) NRNR migraine. Patients with refractory migraine experienced more significant impairment in daily activities (HURT‐2) and social activities (HURT‐3) versus those with resistant and NRNR migraine (46 [63.0%] vs 110 [42.2%] vs 51 [14.4%], p<0.001; 37 [50.7%] vs 67 [25.7%] vs 25 [7.0%], p<0.001). Regarding acute medication efficacy (HURT‐5), more patients with refractory migraine stated that one dose “never” relieved their headache, and delayed or avoided taking acute medication due to concerns about adverse events (HURT‐7), versus those with resistant and NRNR migraine (29 [39.7%] vs 28 [10.7%] vs 25 [7.0%], p<0.001; 16 [21.9%] vs 7 [2.7%] vs 11 [3.1%], p<0.001).

**Conclusion:** Patients with refractory and resistant migraine report poorer acute treatment response compared with those with NRNR migraine, which significantly affect daily lives. Management of difficult‐to‐treat migraine should focus on optimizing acute treatments, alongside preventive therapies.

**Disclosure:** Nothing to disclose.

## EPO‐067

### Profile of triptan use and efficacy in migraine patients treated with Anti‐CGRP monoclonal antibodies

#### I. Sá Pereira ^1^; R. Rato^1^; R. Dias^2^; A. Costa^2^; M. Pinto^1^; M.J. Pinto^2^

##### ^1^Department of Neurology, São João Local Health Unit, Porto, Portugal; ^2^Department of Neurology, São João Local Health Unit, Porto; Department of Clinical Neurosciences and Mental Health, Faculty of Medicine of the University of Porto, Porto, Portugal

**Background and aims:** Anti‐CGRP monoclonal antibodies (mAbs) are migraine‐specific preventive drugs. Evidence suggests they may reduce the need for acute medications, such as triptans. Even so, some anecdotal cases have reported a loss of triptan efficacy during treatment with these agents.

**Methods:** Ongoing ambispective study involving migraine patients from a tertiary center treated with anti‐CGRP mAbs. The primary outcomes were the necessity and efficacy of triptans before and after starting mAbs therapy; a Likert scale was created to assess the latter.

**Results:** A total of 86 patients were included, predominantly women (84,88%), with a mean age of 42±12 years. Fremanezumab was the prevailing mAb (66,28%). Satisfactory to excellent results were reported by 71 patients (82.56%). Before starting mAbs, 69 patients (80.29%) used triptans, mainly zolmitriptan (36.23%) and eletriptan (34.78%). Among these, 57.97% (n=40) experienced some benefit: 27.54% satisfactory, 18.54% good, and 11.59% excellent. After initiating mAbs, 34.78% (n=24) no longer required triptans, indicating a significant reduction in the need for abortive treatments. Among patients continuing triptan use, 33.33% (n=15) reported a difference in efficacy, with 86.67% (n=13) noting improvement (p=0.004).

**Conclusion:** As previously suggested in other studies, preliminary findings indicate that anti‐CGRP mAbs may reduce the need for triptans in migraine management. Despite anecdotal reports of triptan efficacy loss, so far our data did not support this. Further studies are needed to explore this interaction.

**Disclosure:** Nothing to disclose.

## EPO‐068

### Consistency of response to rimegepant: A patient‐level interim analysis of a prospective real‐world observational study

#### L. Abraham ^1^; A. Urani^2^; G. Lambru^3^; R. Lipton^4^; P. Goadsby^5^; P. Pozo‐Rosich^6^; B. Galabova^1^; K. Fanning^7^; F. Dai^8^; K. Hygge Blakeman^9^

##### ^1^Pfizer R&D UK Ltd., Tadworth, Surrey; ^2^Aptar Digital Health, Paris, France; ^3^The Headache and Facial Pain Service, Guy's and St. Thomas' NHS Foundation Trust, London, UK; ^4^Montefiore Medical Center and Albert Einstein College of Medicine, Bronx, New York, USA; ^5^NIHR King's Clinical Research Facility, King's College Hospital/SLaM Biomedical Research Centre, King's College London, UK and University of California, Los Angeles, Los Angeles, USA; ^6^Headache and Neurological Pain Research Group, Vall d'Hebron Research Institute, Universitat Autònoma de Barcelona, Barcelona, Spain. Headache and Craniofacial Pain Unit, Neurology Department, Vall d'Hebron University Hospital, Barcelona, Spain; ^7^MIST Research, Wilmington, USA; ^8^Pfizer, Inc., New York, USA; ^9^Pfizer AB., Stockholm, Sweden

**Background and aims:** This study evaluated real‐world consistency of response to rimegepant for acute treatment of migraine at the individual patient level.

**Methods:** This prospective observational study was conducted using the Migraine Buddy® app with adults experiencing 3–14 headache days in the last 30 days and planning to use rimegepant during the next 30 days. Using a custom‐made interface, participants completed: a baseline survey; a 28‐day daily diary assessing time to meaningful pain relief (relief considered meaningful by the patient), time to meaningful functional improvement, and treatment satisfaction; and a questionnaire at study completion. Interim analyses assessed consistency of response, defined as achieving response in ≥2 of the first 3 rimegepant‐treated attacks or in ≥3 of the first 4 rimegepant‐treated attacks. Response was assessed via meaningful pain relief within 2 hr, meaningful functional improvement within 2hr, and report of satisfied/extremely satisfied with rimegepant.

**Results:** Among 118 participants with ≥3 rimegepant‐treated attacks, 62.7% achieved meaningful pain relief within 2 hr in ≥2 of the first 3 attacks, 60.2% achieved meaningful improvement in functioning within 2 hr in ≥2 of the first 3 attacks, and 75.4% reported treatment satisfaction. Among 95 participants with ≥4 rimegepant‐treated attacks, 48.4% achieved meaningful pain relief within 2 hr in ≥3 of the first 4 attacks, 48.4% achieved meaningful improvement in functioning within 2 hr in ≥3 of the first 4 attacks, and 69.5% reported treatment satisfaction.

**Conclusion:** Many patients with ≥3 or ≥4 rimegepant‐treated attacks achieved consistent response to rimegepant within 2 hr on endpoints of meaningful pain relief and functional improvement.

**Disclosure:** LA, FD, and KHB are employed by, and own stock in, Pfizer. AU is an employee of Aptar Digital Health, paid consultants to Pfizer. GL has received fees from Abbvie, TEVA, Lundbeck, Eli Lilly, Novartis, Pfizer, and Dr Reddy's. RBL received research support, grants, or fees from the NIH, FDA, NHF, Aeon, Allergan/AbbVie, Amgen, Axsome, Dr Reddy's, Eli Lilly, GlaxoSmithKline, Ipsen, Lundbeck, Pfizer, Merck, Teva and Vedanta; and holds options in Axon, Biohaven Pharmaceuticals, CoolTech, and Manistee. PJG received a grant from Kallyope; consulting fees from Aeon Biopharma, Abbvie, Aurene, CoolTech LLC, Dr Reddy's, Eli‐Lilly and Company, Epalex, Linpharma, Lundbeck, Pfizer, PureTech Health LLC, Satsuma, Shiratronics, Teva Pharmaceuticals, Tremeau, and Vial; consulting fees through Gerson Lehrman Group, Guidepoint, SAI Med Partners, Vector Metric; and fees from CME Outfitters and WebMD. PPR received honoraria or research support from AbbVie, Biohaven, Chiesi, Eli Lilly, Lundbeck, Medscape, Novartis, Pfizer and Teva Pharmaceuticals; received grants from AbbVie, AGAUR, EraNet Neuron, FEDER RIS3CAT, Instituto Investigación Carlos III, MICINN, Novartis, and Teva Pharmaceuticals. BG is employed by Pfizer. KMF is employed by MIST Research, which receives funding from AbbVie, Allay Lamp, NYC Langone Health, Juva Health, Migraine Canada, AESARA, and Aptar.

## EPO‐069

### Migraine patients treated with Fremanezumab: What happens when treatment is discontinued? Experience in a single center

#### E. Díaz Fernández; A. Lozano Ros; A. Guillem Mesado; A. Sánchez Soblechero

##### Neurology, Hospital Gregorio Marañón, Madrid, Spain

**Background and aims:** To describe the effect of discontinuing Fremanezumab in a cohort of patients with chronic migraine (CM) or episodic migraine (EM), following the European Headache Federation guidelines (2022).

**Methods:** Inclusion criteria: patients with CM or EM treated with monthly Fremanezumab for 12 to 18 months (December/2020‐December/2024) and follow‐up after discontinuation >6 months. Sample: 61 patients, two groups: patients who after discontinuation required restarting treatment (F1) and patients who did not (F0). Additionally, F1 is subdivided into patients who reinitiated treatment early (<=4 months) (F1.1) or late (F1.2). Demographic characteristics, disability scores (MIDAS and HIT‐6) and migraine days/month (MMD) were analyzed quarterly. Descriptive analysis of the total and analytical analysis between F0 and F1 and between F1.1 and F1.2 were performed (χ^2^, Mann‐Whitney U). The response to Fremanezumab, both initially and upon reinitiation in F1, was compared (Wilcoxon).

**Results:** Sixty‐one patients, mean age 53.1±10 years and age at onset 17.6±7.5 years, mostly women (n=56, 91.8%), 57 (93.4%) suffered from CM. Treatment reinitiation was required in 54 patients (88.5%), 46.3% early, without a significant loss of efficacy (p>0.05) after restart. Three patients required treatment reinitiation twice. At baseline, the mean MMD was 16.8±6.9, the mean HIT‐6 score was 67.7±6.3 and MIDAS score was 91.6±50.3. No statistically significant differences were found between F0 and F1 in demographic characteristics or baseline scores, except gender.

**Figure:**
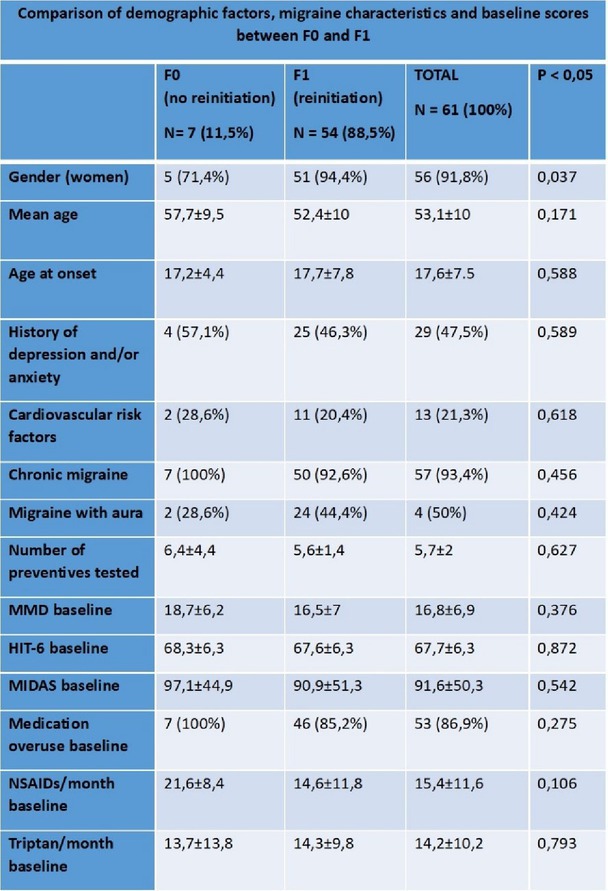
FIGURE 1

**Figure:**
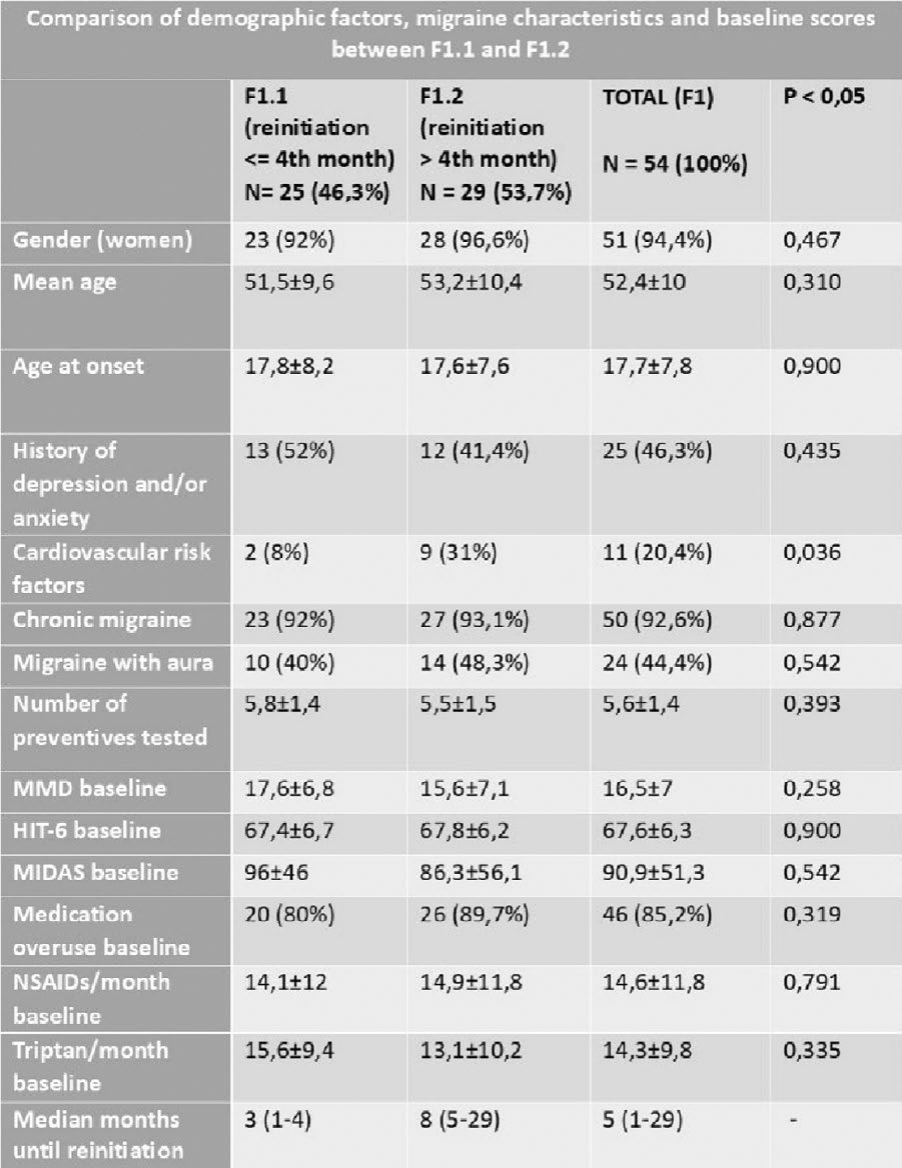
FIGURE 2

**Conclusion:** 88.5% of migraine patients had to reintroduce Fremanezumab, recovering its efficacy after reinitiation. This led to a change in our protocol, consisting of avoiding treatment discontinuation in patients with chronic migraine.

**Disclosure:** Nothing to disclose.

## EPO‐070

### “Comorbidities” of idiopathic intracranial hypertension: An Austrian population based cohort study

#### N. Müller^1^; N. Krajnc^1^; S. Zaic^1^; S. Macher^1^; C. Wöber^1^; W. Marik^2^; K. Novak^3^; B. Pemp^4^; B. Reichardt^5^; G. Bsteh ^1^

##### ^1^Department of Neurology, Medical University of Vienna, Vienna, Austria; ^2^Department of Neuroradiology, Medical University of Vienna, Vienna, Austria; ^3^Department of Neurosurgery, Medical University of Vienna, Vienna, Austria; ^4^Department of Ophthalmology, Medical University of Vienna, Vienna, Austria; ^5^Austrian Social Health Insurance Fund, Eisenstadt, Austria

**Background and aims:** Idiopathic intracranial hypertension (IIH) is a rare disorder characterized by headaches and papilledema. While a variety of diagnoses is frequently observed with IIH, population‐based data on comorbidities and co‐medication in IIH is scarce.

**Methods:** The Austrian health insurance register (>99% population coverage) was queried for patients discharged between 2016 and 2021 with ICD‐10 code G93.2 and/or acetazolamide (AZM) prescription. IIH was considered confirmed if G93.2 was assigned ≥2 times and AZM was prescribed ≥ once. Five obese controls (OBC, ICD‐10: E65/66/68) and five general population controls (GPC) were drawn from the register for each patient. Comorbidities and prescribed analgesics as well as antidepressants were extracted.

**Results:** Of 5,969 patients identified, 114 fulfilled the criteria for confirmed IIH. Compared to 114 OBC and 114 GPC matched for age and sex, IIH patients had significantly higher rates of any headache comorbidity (17.5%; 1.8% OBC, 0% GPC), migraine (10.5%; 1.8% OBC, 0% GPC), and depression (14.9%; 7.0% OBC, 0.9% GPC). Analgesic use was high (88.6%), with increased prescriptions of opioids (30.7%; 15.8% OBC, 5.3% GPC), antimigraine medications (19.3%; 5.3% OBC, 2.6% GPC), and CGRP inhibitors (8.8%; 0% OBC, 0% GPC).

**Figure:**
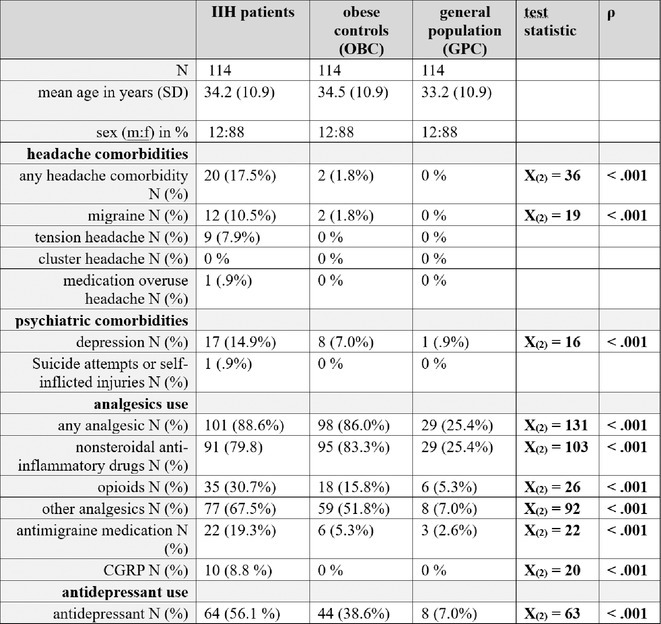
**TABLE 1** Demographics and frequency of invasive/non‐invasive therapies (SD = standard deviation, m = male, f = female).

**Conclusion:** IIH patients had more headache and psychiatric comorbidities than controls and relied more on symptomatic treatments. Frequent use of analgesics, antidepressants, and antimigraine medications highlights the disease burden. As the dataset is based on hospital discharge records, headache comorbidities are likely underreported in GPC. Outpatient data are needed for a more comprehensive assessment.

**Disclosure:** Funding There was no funding to this research. Competing interests Nina Müller1,2, Nik Krajnc1,2, Sina Zaic1,2, Stefan Macher1,2, Christian Wöber1,2, Wolfgang Marik2,3, Klaus Novak2,4, Berthold Pemp5, Berthold Reichardt6, and Gabriel Bsteh1,2 NM: declares no conflict of interest relevant to this study NK: has participated in meetings sponsored by, received speaker honoraria or travel funding from BMC/Celgene, Merck, Novartis, Roche and Sanofi‐Genzyme. SZ: declares no conflict of interest relevant to this study SM: declares no conflict of interest relevant to this study CW: has received honoraria consultancy/speaking from Apomedica, Curelator, Eli Lilly, Grünenthal, Hermes, Novartis, Pfizer, Ratiopharm/Teva, and Stada WM: declares no conflict of interest relevant to this study. KN: declares no conflict of interest relevant to this study. BP: has received honoraria for consultancy/speaking from Chiesi, GenSight and Santen. BR: declares no conflict of interest relevant to this study. GB: has participated in meetings sponsored by, received speaker honoraria or travel funding from Biogen, Celgene/BMS, Lilly, Merck, Novartis, Roche, Sanofi‐Genzyme and Teva, and received honoraria for consulting Biogen, Celgene/BMS, Novartis, Roche, Sanofi‐Genzyme and Teva. He has received unrestricted research grants from Celgene/BMS and Novartis.

## EPO‐071

### Optical nerve sheath diameter for prediction of post‐dural puncture headache: A pilot prospective observational study

#### A. Kunzmann; F. Merzou; D. Janitschke; S. Groppa; P. Lochner

##### Department of Neurology, Saarland University Medical Center, Homburg, Germany

**Background and aims:** Post‐puncture headaches are a frequently distressing complication for patients after diagnostic lumbar puncture (LP). We aimed to determine whether decreased ONSD after diagnostic LP can be used as a predictor for patients with post‐lumbar puncture syndrome (PDPH).

**Methods:** In this prospective observational study 87 patients, who received a diagnostic LP, ONSD measurements were recorded before (T0) and 1 hour after LP (T1). 76 patients became an additional value 24 hours after LP (T2). ONSD measurements were performed 48 hours (T3) and 72 hours (T4) after LP in patients, who presented with symptoms related to intracranial hypotension. Demographic data such as age, gender, BMI and chronic headaches were recorded at different times.

**Results:** All included patients showed a physiological reduction in ONSD after diagnostic LP, but no more substantial decrease in the PDPH group could be shown (Fig. 1). The PDPH group decreases significantly at T2 and T3 (Fig. 2). No statistical difference was found in terms of BMI, gender, liquor volume, needle size or previous headaches between the PDPH and Non‐PDPH groups (Table 1). Younger female patients were more likely to experience PDPH symptoms. The rate of PDPH development was 9.20% (n = 7). The ROC curve analysis showed the optimal ONSD cutoff value at 4.9 mm for predicting PDPH. Adopting this cutoff value, the sensitivity and specificity were 92.9 % and 85.7%, respectively.

**Figure:**
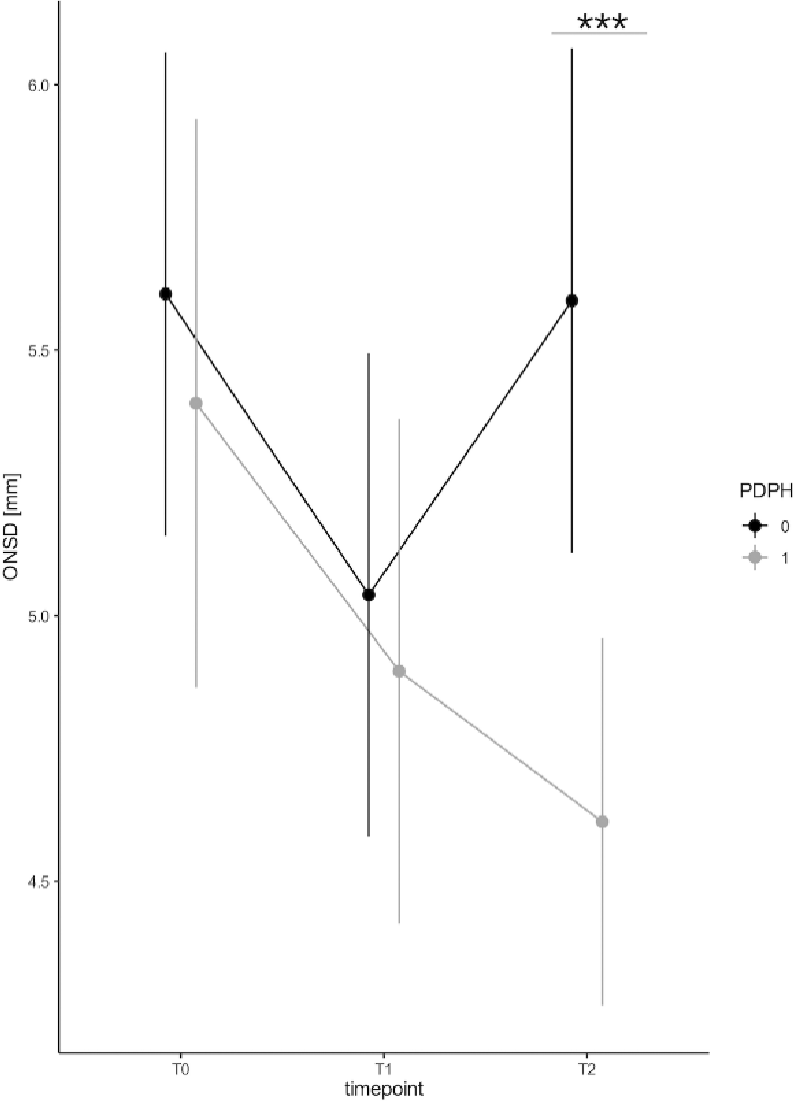
**FIGURE 1** The ONSD measures values of the PDPH group and Non‐PDPH group at different times.

**Figure:**
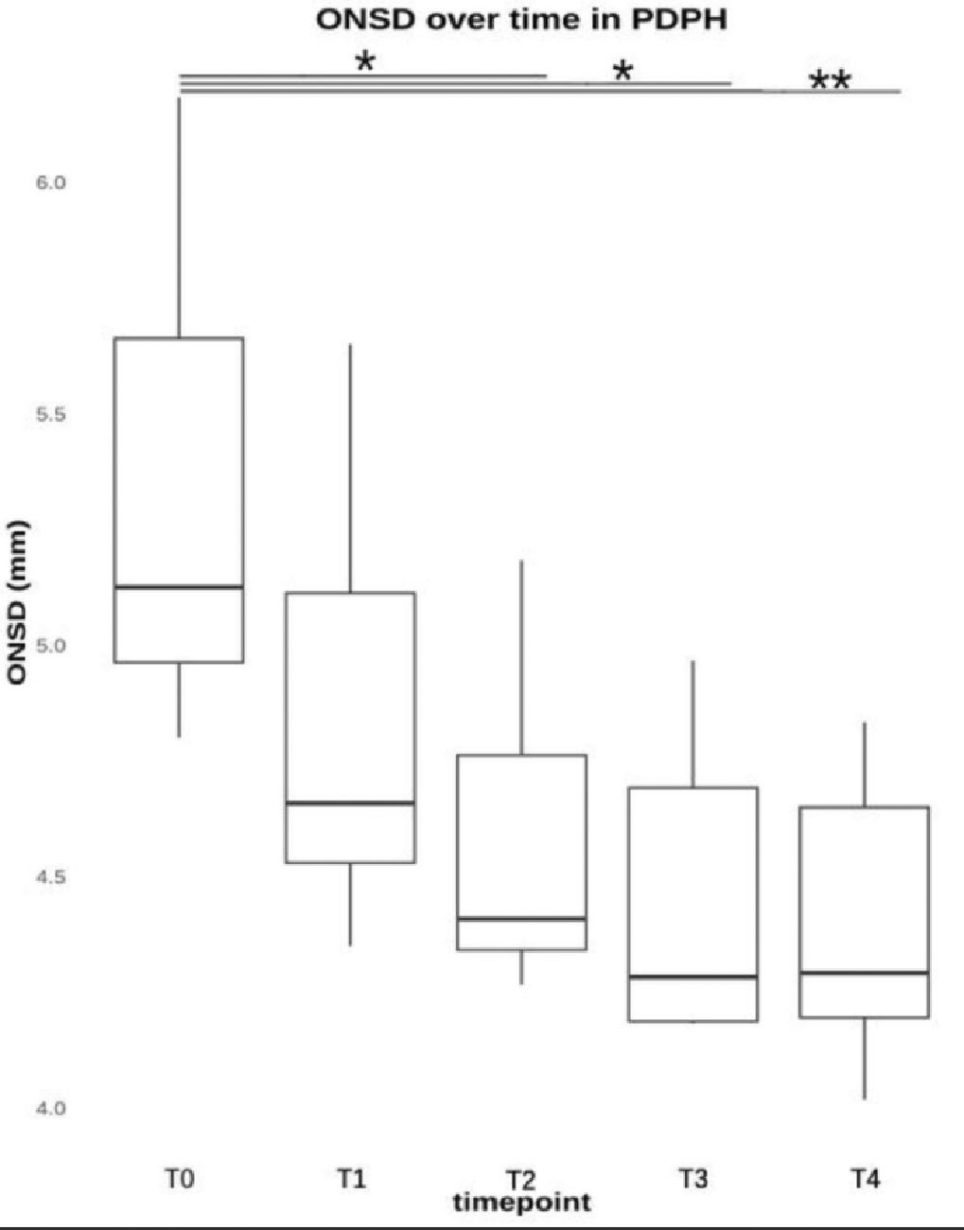
**FIGURE 2** The ONSD measurement values of the patients with post‐dural puncture headache (PDPH) at different times.

**Figure:**
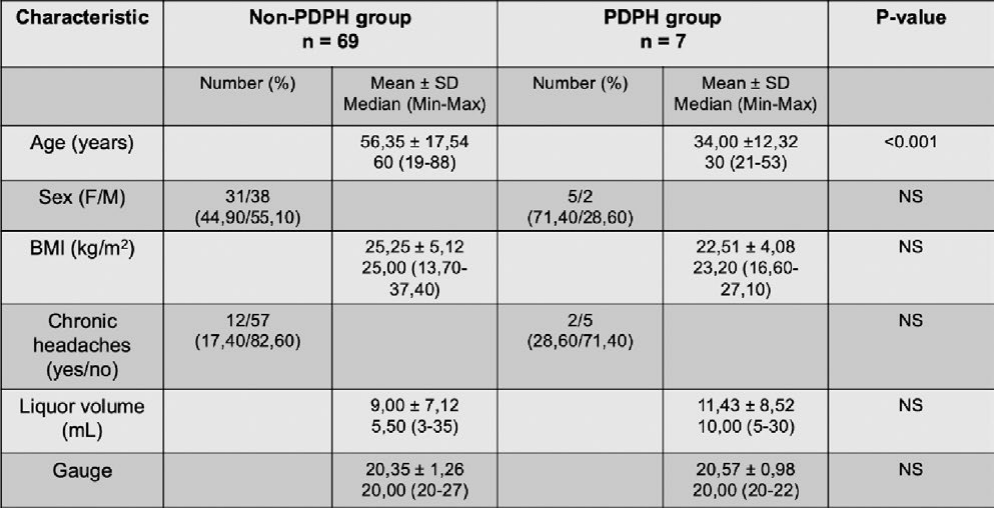
**TABLE 1** Demographic data of the PDPH and Non‐PDPH groups.

**Conclusion:** In conclusion, we showed that non‐invasive ultrasound can be an objective method for diagnosing headaches caused by intracranial hypotension after LP.

**Disclosure:** Nothing to disclose.

## EPO‐072

### Is there still a role for detoxification strategies in migraine therapeutic scenario?

#### V. Dortucci; M. Silvestro; I. Orologio; P. Sozio; F. Trojsi; M. Siciliano; G. Tedeschi; A. Tessitore; A. Russo

##### Headache Centre, Department of Advanced Medical and Surgical Sciences (DAMS), University of Campania “Luigi Vanvitelli”, Italy

**Background and aims:** Medication overuse headache (MOH) management relays on detoxification strategies able to withdraw from overused drugs but also to improve the responsiveness to treatments. Recent evidence supported the effectiveness of monoclonal antibodies acting on the CGRP pathway (CGRP‐mAbs) regardless withdrawal from overused drugs and detoxification. Since MOH can be distinguished in simple (MOH Type I) and complex (MOH Type II) phenotypes, we evaluated whether detoxification strategy can still have a role in patients with complex MOH to improve the response to preventive treatment with CGRP‐mAbs compared to simple MOH.

**Methods:** Two hundred chronic migraine patients affected by MOH and treated with subcutaneous CGRP‐mAbs underwent an extensive interview to assess clinical parameters of disease severity. The primary endpoint of the study was the differences in the percentage of patients achieving a >50% reduction in monthly headache days at the end of the first, third and sixth month of treatment with CGRP‐mAbs compared with the baseline among simple and complex MOH groups based on previous detoxification strategy.

**Results:** Dividing patients based on the diagnosis of MOH types and detoxification strategy (4 groups: patients with MOH type I performing or not detoxification strategy and patients with MOH type II performing or not detoxification strategy), no differences were found in the percentage of patients showing a >50% response in monthly headache attacks frequency nor after one month (p=0.132) nor after the third (p=0.184) and sixth month of treatment (p=0.113).

**Figure:**
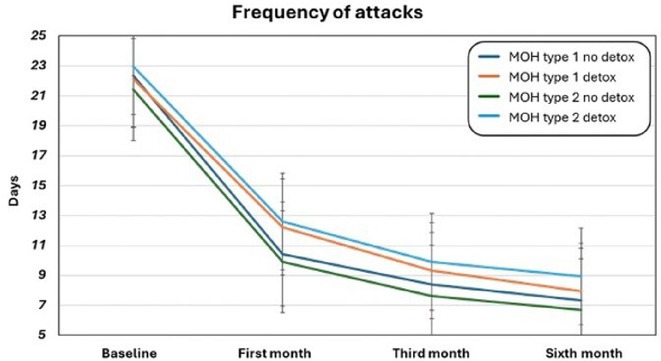
FIGURE 1

**Figure:**
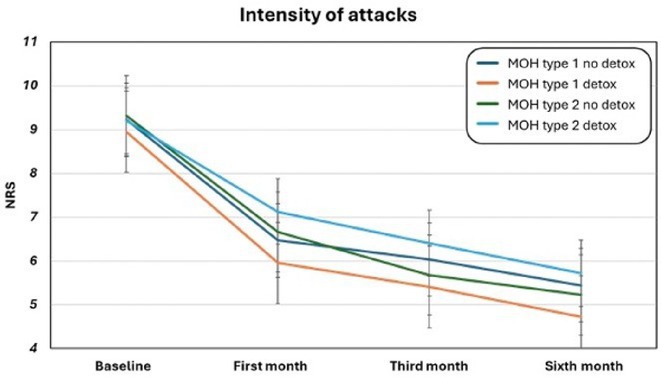
FIGURE 2

**Figure:**
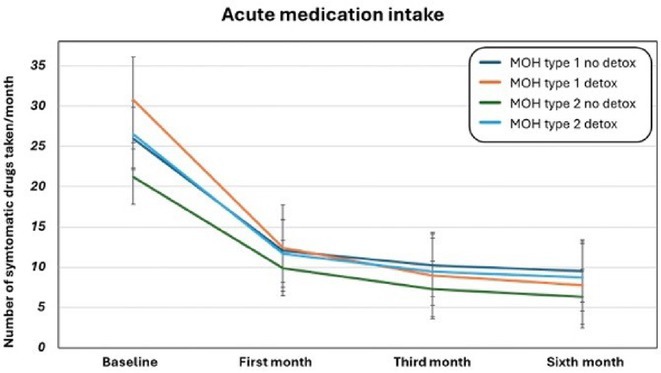
FIGURE 3

**Conclusion:** CGRP‐mAbs may be effective in MOH patients irrespective from both detoxification strategies and “complexity” of MOH.

**Disclosure:** Nothing to disclose.

## EPO‐073

### Impact of atogepant on patient‐reported outcomes for the preventive treatment of migraine in Japanese participants

#### Y. Matsumori ^1^; T. Yamamoto^2^; F. Sakai^3^; N. Imai^4^; G. Ahmadyar^5^; C. Castro^5^; K. Carr^5^; K. Nagy^5^; J. Bao^5^; T. Takeshima^6^

##### ^1^Sendai Headache and Neurology Clinic, Sendai, Miyagi, Japan; ^2^Saitama Medical University Hospital, Saitama, Japan; ^3^Saitama International Headache Center, Saitama, Japan; ^4^Department of Neurology, Japanese Red Cross Shizuoka Hospital, Shizuoka, Japan; ^5^AbbVie, North Chicago, USA; ^6^Headache Center and Department of Neurology, Tominaga Hospital, Osaka, Japan

**Background and aims:** Atogepant is an oral calcitonin gene–related peptide receptor antagonist approved in the US and EU for the preventive treatment of migraine in adults.

**Methods:** This open‐label, 52‐week, long‐term safety study evaluated atogepant 60 mg once daily (QD) for the preventive treatment of migraine in Japanese participants. The study enrolled participants with chronic migraine (CM) who completed the Phase 3 PROGRESS trial and de novo participants with episodic migraine (EM), aged 18–80 years with a >1‐year history of migraine and a history of 4–14 migraine days per month. The study included a 4‐week screening period (EM only), 52‐week open‐label treatment period of atogepant, and 4‐week safety follow‐up period. The primary endpoint was the safety and tolerability of atogepant. Exploratory health outcomes evaluated in the trial included change from baseline in the Migraine Specific Quality of Life Questionnaire v2.1 (MSQv2.1) Role Function‐Restrictive (RFR) domain score, the Activity Impairment in Migraine‐Diary (AIM‐D) Performance of Daily Activities (PDA) and Physical Impairment (PI) domain scores, and Headache Impact Test‐6 (HIT‐6) total score over 52 weeks.

**Results:** The modified intent‐to‐treat population included 150 CM PROGRESS completers and 30 de novo EM participants. Least‐square mean change from baseline at each time point assessed over the 52‐week treatment period demonstrated improvement in the MSQv2.1 RFR domain score (Figure 1), AIM‐D PDA and PI domain scores (Figure 2), and HIT‐6 total score (Figure 3) in CM and EM participants.

**Figure:**
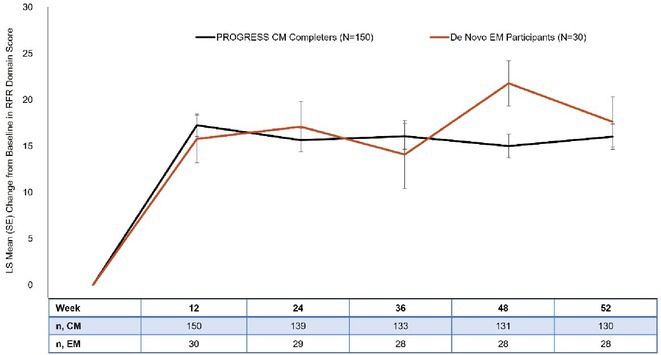
**FIGURE 1** Least Square Mean Change From Baseline in the MSQv2.1 RFR Domain Score Over 52 Weeks in PROGRESS CM Completers and De Novo EM Participants

**Figure:**
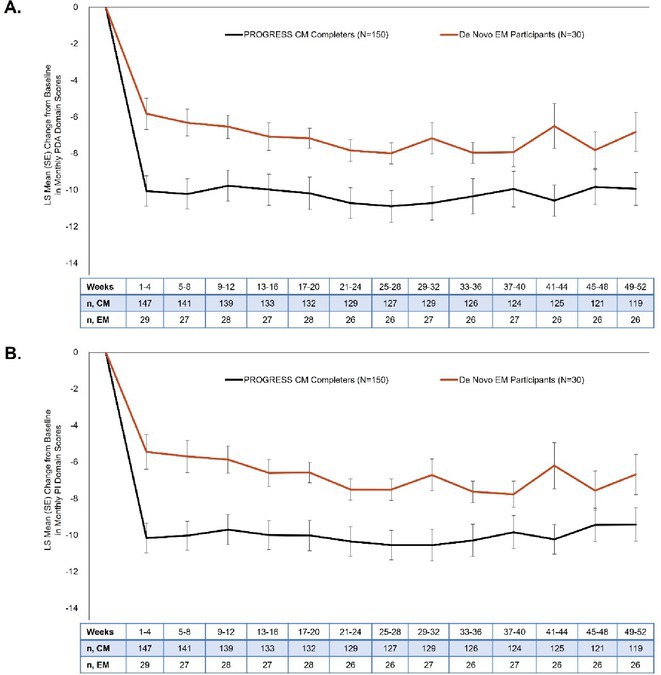
**FIGURE 2** Least Square Mean Change From Baseline in AIM‐D PDA (A) and PI (B) Domain Scores Over 52 Weeks in PROGRESS CM Completers and De Novo EM Participants

**Figure:**
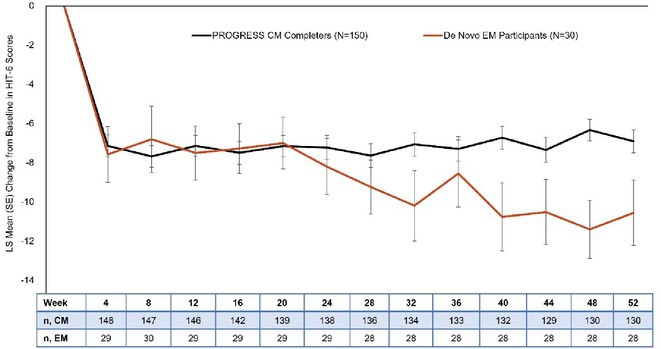
**FIGURE 3** Least Square Mean Change From Baseline in HIT‐6 Total Score Over 52 Weeks in PROGRESS CM Completers and De Novo EM Participants

**Conclusion:** Patient‐reported outcomes showed improvements from baseline with atogepant 60 mg QD treatment and these persisted over 52 weeks.

**Disclosure:** Yasuhiko Matsumori reports personal consultancy fees from Amgen, Astellas, BioPharma K.K., Daiichi Sankyo Co., Ltd, Eli Lilly Japan K.K., and Otsuka Pharmaceutical Co., Ltd. Takao Takeshima has been on the speakers’ bureau for Amgen K.K., Daiichi Sankyo Co., Ltd., Eli Lilly Japan K.K., and Otsuka Pharmaceutical Co., Ltd., and has received research funding/collaborative research expenses from AbbVie GK., Amgen K.K., Eli Lilly Japan K.K., Eisai Co., Ltd., Lundbeck Japan K.K., and Pfizer Japan Inc. Takao Takeshima also acted as an advisor to Hedgehog MedTech, Inc., Sawai Pharmaceutical Co., Ltd., and TEIJIN Pharma Ltd. Fumihiko Sakai is a consultant for Amgen, Eli Lilly, and Otsuka. Toshimasa Yamamoto has received honoraria from Eisai Co., Ltd., and Takeda Pharmaceutical Co., Ltd. Noboru Imai reports being an advisor for Sawai and received speaker fees from Daiichi Sankyo, Eli Lilly, Otsuka, and Amgen. Gina Ahmadyar, Colleen Castro, Karen Carr, Krisztian Nagy, and Jia Bao are employees of AbbVie and may own AbbVie stock.

## EPO‐074

### Low pulse pressure and high serum complement C1q are risk factors for hemodialysis headache

#### Z. Xiao

##### Department of Neurology, Renmin Hospital of Wuhan University, Wuhan, China

**Background and aims:** Hemodialysis headache (HDH) is a common complication in dialysis patients, affecting their quality of life. The etiology and triggering factors are not well understood. This study aimed to assess the prevalence and characteristics of HDH in Chinese patients undergoing hemodialysis and identify potential risk factors.

**Methods:** The study included two phases: a cross‐sectional observational study and a case‐control study. Participants underwent neurological exams, and demographic and medical data were collected. Serum levels of creatinine, uric acid, glucose, electrolytes, inflammatory markers (TNF‐α, IL‐1β, IL‐6, etc.), and blood pressure were measured before and after dialysis.

**Results:** he prevalence of HDH was 37.7% (183/485). HDH was typically a bilateral, moderate‐intensity tightening headache lasting less than 2 hours. In the case‐control study (50 HDH patients, 84 controls), pre‐dialysis pulse pressure (PP) was lower in the HDH group (51.5 ± 18.2 vs. 67.9 ± 14.9, p = 0.027). Pre‐dialysis C1q levels were significantly higher in the HDH group (201.5 vs. 189.0, p = 0.021). Lower pre‐dialysis PP (OR = 0.96) and body weight (OR = 0.95) decreased HDH risk, while higher C1q levels (OR = 1.02) increased the odds of HDH.

**Conclusion:** Low PP, low body weight, and high blood complement C1q may be potential risk factors associated with HDH.

**Disclosure:** Nothing to disclose.

## Movement Disorders 1

## EPO‐075

### Plasma NfL and extracellular vesicles profile predict cognitive impairment in Parkinson's disease

#### A. Imarisio ^1^; S. Berra^2^; M. Squillario^2^; F. Miraglia^3^; A. Cacciotti^3^; R. Calabrese^4^; C. Galandra^4^; L. Biscetti^5^; A. Di Fonzo^6^; P. Parchi^7^; R. Ghidoni^8^; G. Forloni^9^; E. Valente^1^

##### ^1^Department of Molecular Medicine, University of Pavia, Pavia, Italy; IRCCS Mondino Foundation, Pavia, Italy; ^2^LISCOMP Lab, IRCCS Ospedale Policlinico San Martino, Genoa, Italy; ^3^Brain Connectivity Laboratory, Department of Neuroscience & Neurorehabilitation, IRCCS San Raffaele Roma, Italy; 5 Department of Theoretical and Applied Sciences, eCampus University, Novedrate, Italy; ^4^IRCCS Mondino Foundation, Pavia, Italy; ^5^Section of Neurology, Italian National Research Center on Aging (IRCCS INRCA), Ancona, Italy; ^6^Foundation IRCCS Ca' Granda Ospedale Maggiore Policlinico, Neurology Unit, Milan, Italy; ^7^Istituto delle Scienze Neurologiche di Bologna, Italy; ^8^Molecular Markers Laboratory, IRCCS Istituto Centro San Giovanni di Dio, Brescia, Italy; ^9^Department of Neuroscience, Istituto di Ricerche Farmacologiche Mario Negri IRCCS, Milan, Italy

**Background and aims:** The mechanisms underlying cognitive decline in PD remain largely unclear. We investigated the relative contribution of a selected panel of biomarkers of neurodegeneration and variants in the GBA and APOE genes in driving cognitive dysfunction in PD.

**Methods:** We enrolled 222 PD patients in a multicentric, cross‐sectional study. The cohort was stratified in PD with normal cognition (PD‐NC, n = 119), PD with mild cognitive impairment (PD‐MCI, n = 82) and PD with dementia (PDD, n = 21). GBA and APOE genotypes were characterized in the whole cohort (Table 1). Plasma levels of neurofilament light chain (NfL), tau, p‐tau181, Aβ1‐40, Aβ1‐42, α‐synuclein, glial fibrillar acidic protein (GFAP), and small extracellular vesicles (EVs concentration and mean size) were analyzed using SIMOA and Nanoparticle Tracking Analysis.

**Figure:**
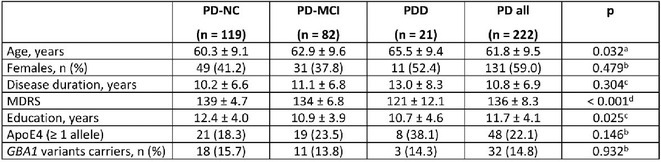
**TABLE 1** Demographics and clinical characteristics of the study cohort. a One‐way ANOVA; b Chi‐Square test; c Kruskal‐Wallis test; d Two‐way ANCOVA with group and sex as factors, age as covariate.

**Results:** Age‐adjusted ANCOVA showed higher Aβ1‐40, Aβ1‐42, NfL, p‐Tau, t‐Tau in PDD than PD‐NC. PD‐MCI showed higher NfL, p‐Tau and EVs mean size compared to PD‐NC (Fig. 1). A multinomial logistics regression model adjusted for demographics and genetic variables showed higher EVs mean size (p=0.023) and NfL concentration (p=0.037) associated to PD‐NC and PDD group, respectively (Table 2). There were no differences in plasma biomarker profiles between GBA‐PD and non‐GBA‐PD.

**Figure:**
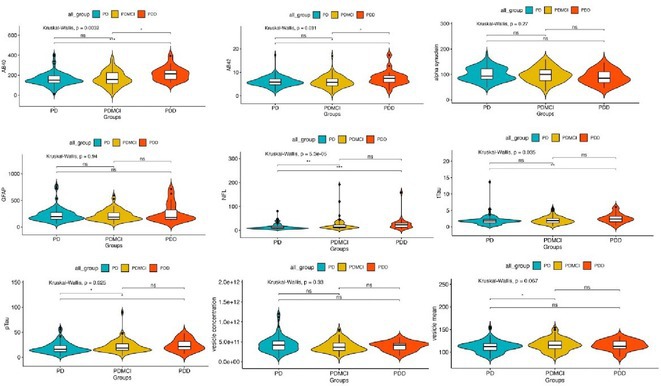
**FIGURE 1** Plasma biomarkers levels in PD‐NC, PD‐MCI e PDD groups.

**Figure:**
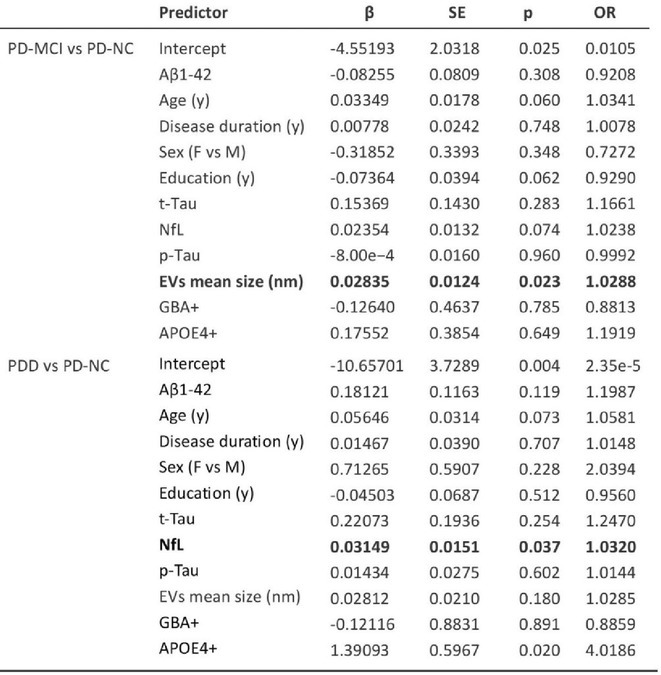
**TABLE 2** Multinomial logistic regression model for prediction of PD cognitive status.

**Conclusion:** We identified plasma NfL and EVs mean size as independent predictors of cognitive dysfunction in PD, independently from GBA and APOE status. The longitudinal follow‐up of this well‐characterized cohort holds promises in identifying novel biomarkers able to identify clusters of PD patients with distinct cognitive trajectories.

**Disclosure:** Nothing to disclose.

## EPO‐076

### Post Hoc analysis: Impact of prior catechol‐o‐methyltransferase Inhibitor use on foslevodopa/foscarbidopa optimization

#### A. Fasano^1^; B. Bergmans^2^; E. Freire‐Alvarez^3^; P. Odin^4^; L. Bergmann^5^; R. Gupta^5^; K. Onuk^5^; J. Samuelsson^5^; M. O'Meara ^5^; K. Chaudhuri^6^

##### ^1^Edmond J Safra Program in Parkinson's Disease, Morton & Gloria Shulman Movement Disorders Clinic, Toronto Western Hospital, UHN; Division of Neurology, University of Toronto; Krembil Brain Institute, Ontario, Canada; IRCCS Humanitas Research Hospital, Milan, Italy; ^2^AZ St‐Jan Brugge, Brugge, Belgium and Ghent University Hospital, Ghent, Belgium; ^3^Neurology Department, University General Hospital of Elche, Carrer Almazara, Elche, Spain; ^4^Lund University, Lund, Sweden; ^5^AbbVie Inc, North Chicago, USA; ^6^Institute of Psychiatry, Psychology & Neuroscience, King's College Hospital, London, UK

**Background and aims:** Prior (until conversion) catechol‐O‐methyltransferase inhibitors (COMTi) use may affect foslevodopa/foscarbidopa (LDp/CDp) dose conversion and optimization due to COMT inhibition and related pharmacodynamic effects. This post hoc analysis explored impacts of prior COMTi on dosage, efficacy, and safety of 24‐hour continuous subcutaneous LDp/CDp infusion.

**Methods:** Patients from a 52‐week open‐label study of LDp/CDp (NCT03781167) were grouped by prior COMTi (opicapone or entacapone) or no prior(non‐COMTi) use. Outcomes included dosing, change from baseline (CFB) in OFF‐time and ON‐time without troublesome dyskinesia (PD diary), CFB in dyskinesia time/impact (MDS‐UPDRS 4.1/4.2), and adverse events ([AE] including special interest: hallucinations and dyskinesia).

**Results:** Prior opicapone users had shorter PD duration and more OFF‐time at baseline than non‐COMTi (Table 1). Both prior COMTi groups had higher baseline levodopa(LD) equivalent doses (Table 1). Prior entacapone users showed longer optimization (Table 2). From weeks 1–2, median daily LD dose slightly decreased for prior opicapone but increased for prior entacapone and non‐COMTi (Figure 1). Dyskinesia time scores increased from baseline–day 2 in prior COMTi vs non‐COMTi(mean[SD]:prior opicapone,0.4[0.9],p=.066; prior entacapone, 0.3[1.0],p=.049; non‐COMTi,0.0[0.9]). No other relevant CFB differences were observed for dyskinesia, OFF time, or ON time without troublesome dyskinesia. Groups reported similar proportions of any AE (Table 2). Table 2 shows hallucination, dyskinesia, and serious AEs; however, sample sizes are a limitation. LDp/CDp discontinuation was similar among groups; most common reason was AE for prior opicapone and non‐COMTi, and withdrawn consent for prior entacapone (Table 2).

**Figure:**
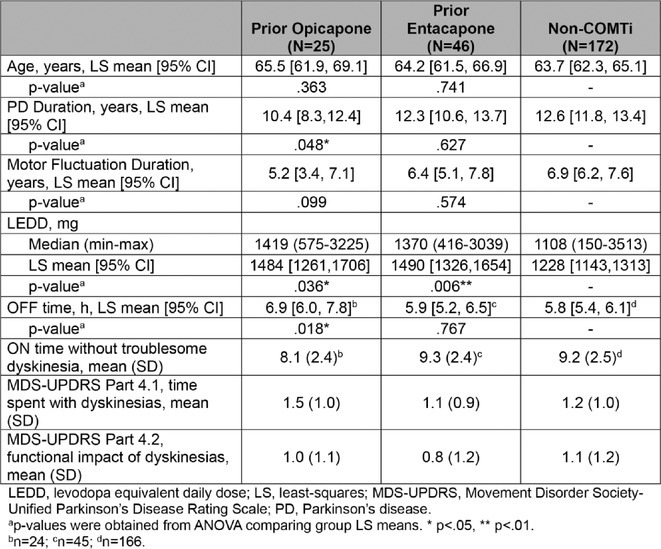
**TABLE 1** Demographics and Baseline Characteristics by Prior COMT Inhibitor Use.

**Figure:**
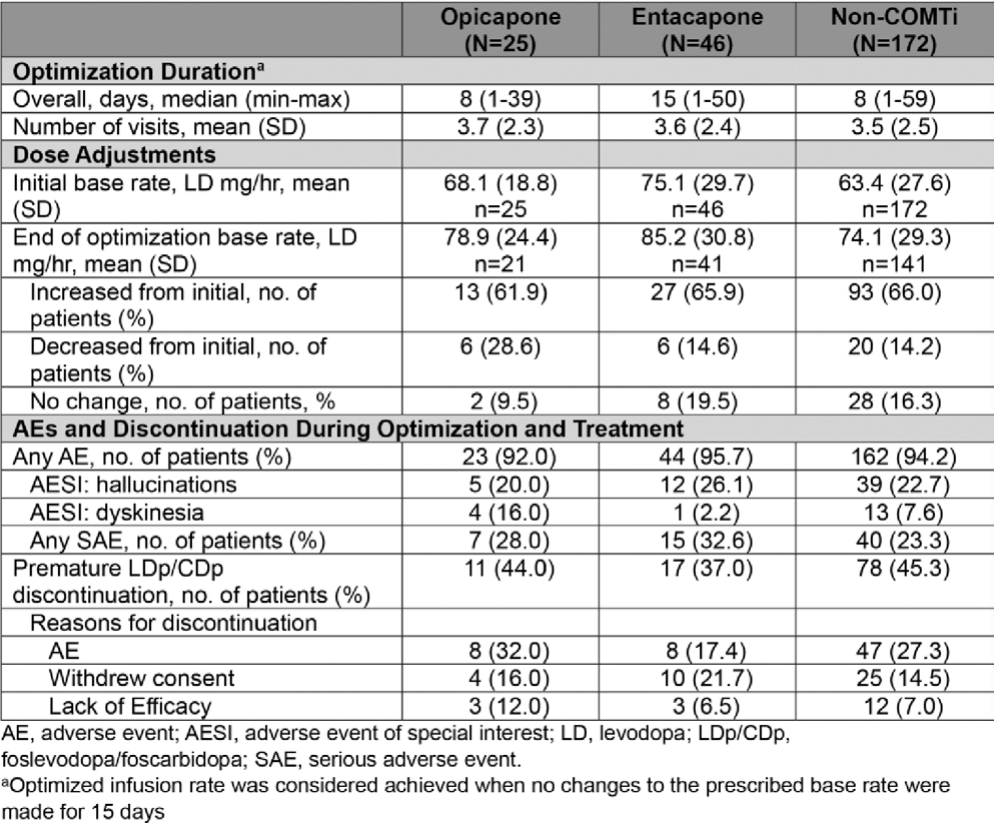
**TABLE 2** Dose Adjustments During the Initial Optimization Period and Overall AE Profile.

**Figure:**
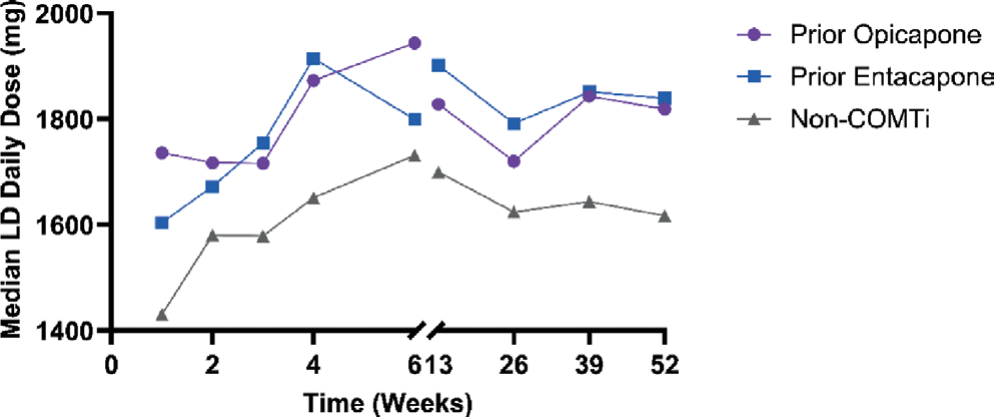
**FIGURE 1** Daily LD Dose from LDp/CDp Infusion Over 52 Weeks.

**Conclusion:** Prior COMTi use does not impact overall efficacy and safety of LDp/CDp. During initial days after conversion, closer monitoring for dosing‐related effects like dyskinesias may be advised.

**Disclosure:** AF received consulting fees from AbbVie, Abbott, Boston Scientific, Medtronic, and UCB; research support from Boston Scientific, Medtronic, Michael J. Fox Foundation for Parkinson's Research, and University of Toronto; honoraria as a speaker for AbbVie, Boston Scientific, Chiesi, Medtronic, Novartis, Teva, and UCB. BB has received advisor/speaker fees and/or grants from AbbVie Inc, EG, Ipsen, Merz, and Zambon EF has received advisory, consulting, and lecture fees from AbbVie, Almirall, Bial, Eisai, UCB, Teva, Neuraxpharm, Estada, and Zambon. He is an investigator on AbbVie studies. PO received compensation/grants/royalties from: AbbVie Inc, Bial, Britannia, Ever Pharma, Lobsor, Nordic Infucare, Stada, Zambon, Uni Med Verlag, UCB. PO's institution has received research support from AbbVie Inc, Parkinsonfonden, Swedish Research Council, and Region Skåne. LB, RG, JS, KO and MM are employees of AbbVie Inc. KRC has received educational funding from UCB; honoraria for sponsored symposia from UCB, AbbVie, Britannia, US Worldmeds, Otsuka, Medtronic, Zambon, Bial, Sunovion, Scion; acted as a consultant for AbbVie, UCB, Britannia Bial, Sunovion. This study was funded by AbbVie Inc. AbbVie Inc participated in the study design; study research; collection, analysis, and interpretation of data; and writing, reviewing, and approving this abstract for submission.

## EPO‐077

### Retinal asymmetrical degeneration in Parkinson's disease and rem sleep behavior disorder

#### C. Cicero ^1^; C. Terravecchia^1^; G. Mostile^1^; G. Donzuso^1^; L. Giuliano^1^; D. Contrafatto^2^; C. Chisari^1^; A. Nicoletti^1^

##### ^1^Department of Medical, Surgical, and Advanced Technologies “G.F. Ingrassia”, University of Catania, Catania, Italy; ^2^Neurologic Clinic, AOU Policlinico G. Rodolico‐San Marco, Catania, Italy

**Background and aims:** According to the Synuclein Origin and Connectome (SOC) model, Rem Sleep Behavior Disorder (RBD) patients represent the prodromal phase of “body first” Parkinson's Disease (PD) patients, characterized by a more symmetric disease presentation due to a more symmetric alpha‐synuclein spreading. Conversely, “Brain first” PD patients are predicted to have an asymmetrical spreading and clinical presentation, and no prodromal RBD. Thinning of the retinal layers has been described in both RBD and PD patients, however no study has ever assessed the presence of asymmetrical retinal degeneration.

**Methods:** Early PD “brain first” patients diagnosed according to the MDS‐PD diagnostic criteria were recruited, absence of RBD was assessed using the RBDSQ questionnaire (score <6). Isolated RBD patients were diagnosed via videopolysomnography. Macula layer's thickness was evaluated using Spectral‐Density Optical Coherence Tomography (SD‐OCT). Asymmetry index (AI) was computed for each macular layer.

**Results:** Thirteen RBD patients and 15 PD “brain first” were recruited. Mean disease duration for PD patients was 25.6 ± 13.5 months, with a mean UPDRS‐ME score of 25.5 ± 7.3. There was no difference in age, sex, and Moca score between the groups. Concerning macular layers, there was a significant higher AI in the outer plexiform layer (OPL) in PD vs RBD patients (10.7±8.5 vs 4.3±3.2; p=0.02). No differences were found in the other macular layers.

**Conclusion:** Our findings suggest a more asymmetrical retinal degeneration in “brain first” PD patients and a more symmetrical pattern in prodromal “body first” patients supporting the SOC model.

**Disclosure:** Nothing to disclose.

## EPO‐078

### OGA inhibition as a potential therapeutic approach for tauopathies: The prosper study, a phase 2 trial in PSP

#### G. Höeglinger^1^; L. Golbe^2^; A. Boxer^3^; Y. Compta Hirnyj^4^; H. Morris^5^; A. Colomé^6^; M. Nicolás^6^; L. Álvarez^6^; B. Fernández^6^; C. Sastré^6^; C. Varona ^6^

##### ^1^Ludwig‐Maximilians‐University Munich, Munich, Germany; ^2^Rutgers University, New Brunswick, USA and CurePSP, New York, USA; ^3^University of California, San Francisco Memory and Aging Center; ^4^Hospital Clinic de Barcelona; ^5^UCL Queen Square Institute of Neurology; ^6^Ferrer, Barcelona, Spain

**Background and aims:** Progressive supranuclear palsy (PSP) is a primary tauopathy characterized by the pathological aggregation of tau protein. Tau hyperphosphorylation and other post‐translational modifications contribute to its accumulation. O‐GlcNAcylation, a dynamic modification that competes with phosphorylation, is regulated by O‐GlcNAcase (OGA), which removes O‐GlcNAc moieties from tau. OGA inhibition has been shown to elevate tau O‐GlcNAcylation and to reduce the pathological aggregation of tau. Studies using different OGA inhibitors have consistently shown a reduction in tau‐related pathology in multiple tau models. FNP‐223, a selective orally administered OGA inhibitor, shows promise as a disease‐modifying therapy for PSP based on preclinical evidence.

**Figure:**
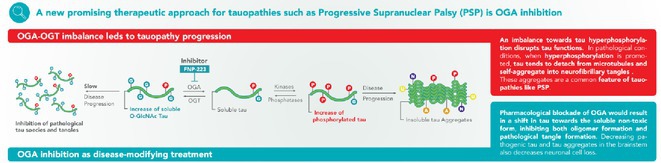
**FIGURE 1** FNP‐223 Mechanism of Action. FNP‐223 is a reversible, substrate‐competitive inhibitor of OGA, blocking O‐GlcNAc removal from tau. This inhibition increases glycosylated tau, preventing pathological aggregation while maintaining structure and function.

**Methods:** The PROSPER study is a Phase 2 randomized, double‐blind, placebo‐controlled trial assessing the efficacy, safety, and pharmacokinetics of FNP‐223. Eligible participants include patients diagnosed with possible or probable PSP‐Richardson's syndrome within three years of symptom onset. 220 participants are being recruited across 44 sites in Europe and the U.S. Participants are randomized 1:1 to receive oral FNP‐223 or placebo three times daily for 52 weeks. The primary endpoint is the change in the total PSPRS score. Secondary and exploratory endpoints will evaluate effects on progression rates of functionality, cognition, quality‐of‐life, neurodegeneration fluid biomarkers, and brain volume.

**Results:** Recruitment is ongoing. The study aims to provide critical data on the safety and efficacy of FNP‐223 in slowing disease progression.

**Conclusion:** FNP‐223 represents a promising therapeutic approach targeting tau pathology in PSP. The PROSPER study will determine its potential as a disease‐modifying agent for PSP, addressing an urgent unmet need in this population.

**Disclosure:** Prof. Dr. Med. Günter Höeglinger, Dr. Lawrence I. Golbe, Dr. Adam Boxer, Dr. Yaroslau Compta Hirnyj, Dr. Huw Morris are coordinating investigators for the PROSPER Study. Anna Colomé, Marta Nicolás, Lubia Álvarez, Begoña Fernández, Carla Varona, Carlos Sastré are employees of Ferrer, the sponsor of the PROSPER Study.

## EPO‐079

### Apomorphine sublingual film's efficacy in elderly patients with Parkinson's disease: Post‐hoc analysis of study CTH‐301

#### F. Moreira ^1^; L. Wojtecki^2^; W. Jost^3^; D. Santos‐Garcia^4^; J. Kassubek^5^; M. Fonseca^6^; G. Harrison‐Jones^6^; I. Pijuan^6^

##### ^1^Parkinson and Movement Disorders Unit, Neurology Department, CUF Coimbra Hospital, Coimbra, Portugal; ^2^Department of Neurology & Institute of Clinical Neuroscience and Medical Psychology, University Clinic Duesseldorf, Duesseldorf, Germany; ^3^Parkinson Klinik‐Ortenau, Wolfach, Germany; ^4^CHUAC, Complejo Hospitalario Universitario de A Coruña, A Coruña, Spain; ^5^Department of Neurology, University Hospital Ulm, Ulm, Germany; ^6^Bial – R&D Investments, S.A., Coronado, Portugal

**Background and aims:** Apomorphine sublingual film (SL‐APO) is indicated for the on‐demand treatment of OFF‐episodes in patients with Parkinson's disease (PD). This study evaluated the efficacy of SL‐APO in elderly (≥70 years) and younger (<70 years) patients over the long term.

**Methods:** The Phase 3, multicentre, non‐randomised, open‐label Study CTH‐301 assessed the long‐term (≥3 years) safety, tolerability and efficacy of SL‐APO. This post‐hoc analysis evaluated the efficacy of SL‐APO in patients aged <70 and ≥70 years. Assessments included SL‐APO dose, discontinuation rate due to lack of efficacy, changes in Movement Disorder Society‐Unified PD Rating Scale (MDS‐UPDRS) Part III scores from pre‐ to post‐dose at Weeks 24, 36 and 48, and percentage of patients with a full‐ON response within 30 minutes post‐dose at Weeks 24, 36 and 48.

**Results:** Of the 369 de novo (not previously exposed to SL‐APO) patients included in Study CTH‐301, 253 (68.6%) were aged <70 years and 116 (31.4%) ≥70 years. The mean SL‐APO optimised dose was similar for the <70 and the ≥70 years age groups (19.6 mg vs 21.2 mg; p=0.09). Both groups achieved a clinically meaningful reduction in MDS‐UPDRS Part III at all time points (Figure 1). More than 75% of patients in both groups reported a full‐ON response at all visits (Figure 2). The rate of discontinuation due to lack of efficacy was low and comparable across age groups (6.3% [16/253] vs 6.0% [7/116]).

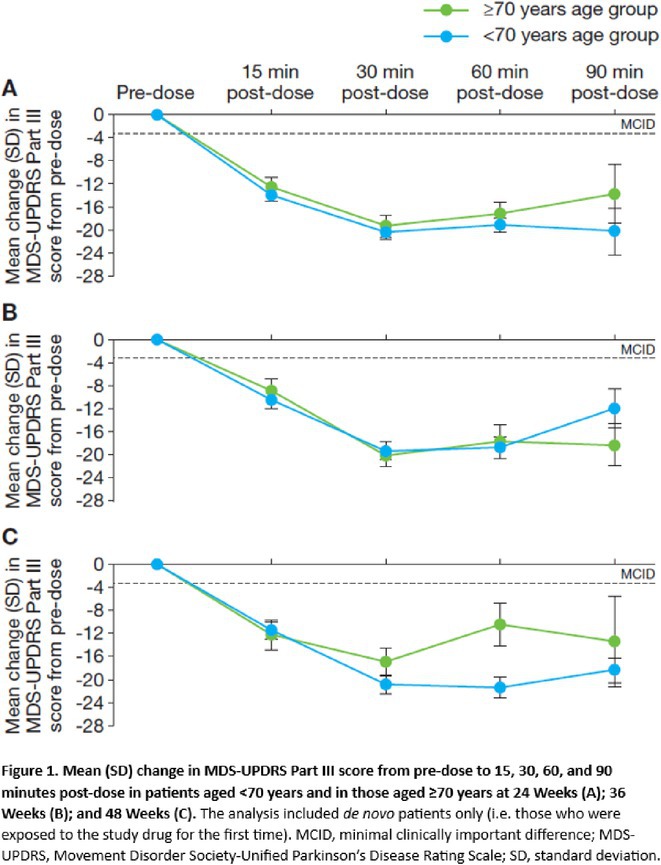

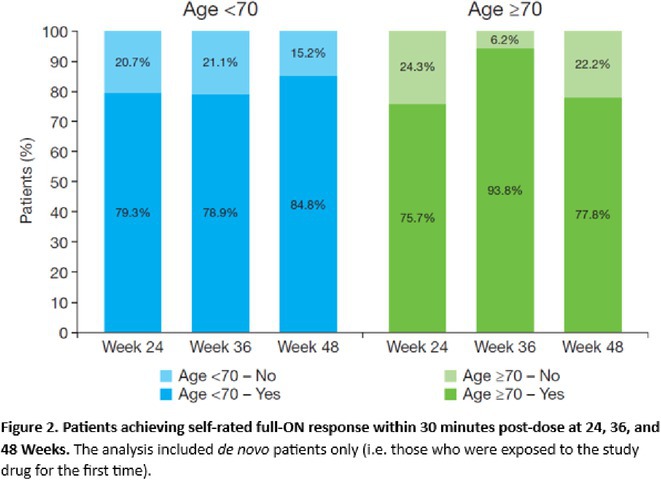

**Conclusion:** SL‐APO was efficacious over the long‐term as an on‐demand treatment for OFF episodes in elderly patients with PD.

**Disclosure:** FM is a consultant for Bial, AbbVie, and Zambon, and has received honoraria for educational presentations from Bial and Zambon. LW has received a speaker honorarium and travel payments from Bial. WHJ is a speaker and/or consultant for Bial, Britannia, Desitin and Zambon. DSG has received honoraria for educational presentations and advice service from AbbVie, UCB Pharma, Lundbeck, KRKA, Zambon, Bial, Italfarmaco, Archimedes, Esteve, Qualigen, Teva, Merz, Orionpharma and Stada. He also received grants from the Spanish Ministry of Economy and Competitiveness [PI16/01575] co‐founded by ISCIII (Concesión de subvenciones de Proyectos de Investigación en Salud de la convocatoria 2020 de la Acción Estratégica en Salud 2017‐2020 por el proyecto “PROGRESIÓN NO MOTORA E IMPACTO EN LA CALIDAD DE VIDA EN LA ENFERMEDAD DE PARKINSON” y “Concesión de Contrato para la intensificación de la actividad investigadora en el Sistema Nacional de Salud, Convocatoria 2021, Instituto de Salud Carlos III”). JK has received honoraria or consultation fees from AbbVie, Bial, Biogen, Desitin, Esteve, Licher MT, Medtronic, NeuroDerm, Novartis, STADA, UCB Pharma, and Zambon; in addition, he is Specialty Chief Editor for Frontiers in Neurology (section Applied Neuroimaging) and Associate Editor (Neurology) for Therapeutic Advances in Chronic Disease. MMF, GH‐J, and IP are employees of Bial. Study supported by Bial.

## EPO‐080

### Effect of opicapone on non‐motor burden in people with Parkinson's disease‐related sleep disturbances: The OASIS study

#### J. Ferreira^1^; M. Gago^2^; R. Costa^3^; M. Fonseca^4^; H. Brigas ^3^; J. Holenz^3^; C. Trenkwalder^5^

##### ^1^IMM João Lobo Antunes Institute of Molecular Medicine, Faculty of Medicine, University of Lisbon, Lisbon, Portugal; ^2^Department of Neurology, ULS Alto Ave, Hospital da Senhora da Oliveira, Guimarães, Portugal; ^3^BIAL – Portela & Ca S.A., Coronado, Portugal; ^4^BIAL ‐ R&D Investments, S.A. Coronado, Portugal; ^5^Paracelsus‐Elena Klinik, Kassel, Germany

**Background and aims:** Sleep disturbances are common and challenging to manage in Parkinson's disease (PD) and patients often present with a high non‐motor symptoms (NMS) burden. This study's objective was to assess if enhancing levodopa effectiveness with the catechol‐O‐methyl transferase inhibitor opicapone (OPC) may alleviate NMS in patients with PD‐related sleep issues and motor fluctuations.

**Methods:** This post‐hoc analysis of the 6‐week, open‐label, single‐arm OpicApone in Sleep dISorder (OASIS) study evaluated OPC 50 mg as levodopa add‐on therapy. Changes from baseline to Week 6 in Movement Disorder Society Non‐Motor Rating Scale (MDS‐NMS) domains were analysed, as well as tolerability.

**Results:** Of the 16 patients in the OASIS, 15 completed treatment. At baseline, the mean (standard error; SE) MDS‐NMS score was 131.3 (26). Following 6 weeks of OPC treatment, there was a significant reduction in the MDS‐NMS score, with a mean change of ‐28.9 (95% confidence interval: ‐44.7 to ‐13.2; p=0.0015). The mean (SE) MDS‐NMS sleep and wakefulness domain decreased by ‐6.4 (2.6) points (‐31%; p=0.025) (Table 1), with significant improvements in insomnia (‐2.8 [1.1]; ‐43%; p=0.03) and in unintentional daytime sleep episodes (‐2.2 [0.8]; ‐41%, p=0.02) (Figure 1). Mean (SE) reductions were seen across other MDS‐NMS domains, including depression, anxiety, apathy, gastrointestinal, pain and other symptoms (Table 1). OPC was well‐tolerated.

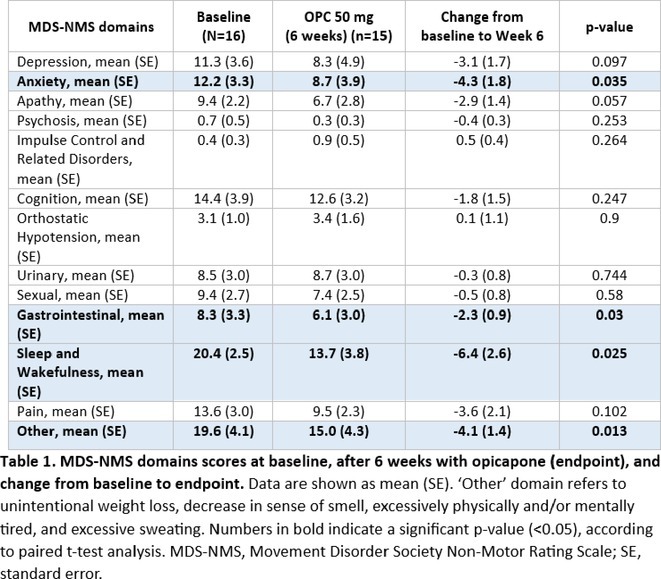

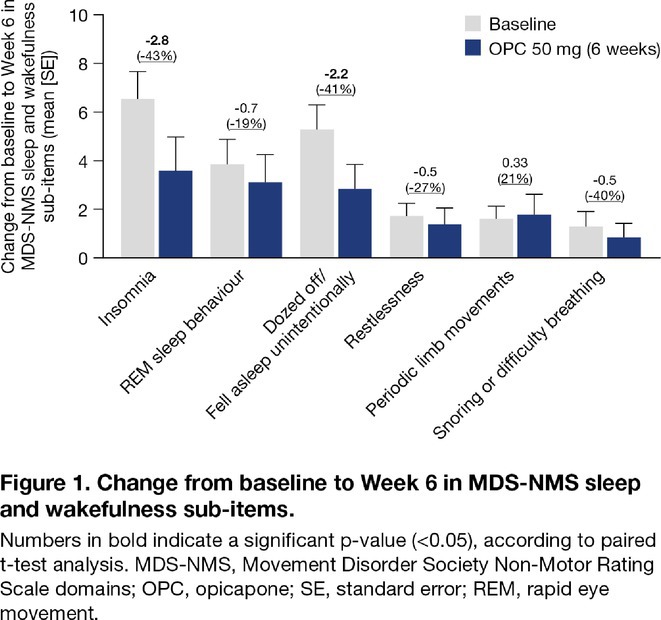

**Conclusion:** OPC significantly reduced NMS burden, particularly improving sleep‐related issues, such as insomnia and daytime sleepiness, in PD patients with motor fluctuations and sleep disturbances, highlighting its potential to address both motor and non‐motor challenges in this population.

**Disclosure:** JJF has received grants from GlaxoSmithKline, Grunenthal, Fundação MSD (Portugal), TEVA, MSD, Allergan, Novartis and Medtronic. JFF also received consultancy and speaker fees, and participated in advisory boards for GlaxoSmithKline, Novartis, TEVA, Lundbeck, Solvay, BIAL, Merck‐Serono, Merz, Ipsen, Biogen, Acadia, Allergan, Abbvie, Sunovion Pharmaceuticals, Zambon, Affiris and Angelini. MFG has received payment/honoraria for lectures from Zambon, Bial Portugal, Takeda and Amicus Therapeutics, and payment/honoraria for advisory boards from Abbvie and Bial Portugal. MMF, RC, HB and JH are employees of Bial. CT has received consulting/independent contractor fees from AbbVie, UCB, Roche, Bial, Ono and Boehringer, speakers honoraria from AbbVie, STADA, Bial and Alexion, and receives royalties from Thieme Publisher, License fee: PDSS‐2, grant and contracted research support from The Michale J. Fox Foundation, EU: Era‐Net program, BRAVA Project, and is an employee (full or part‐time) of Paracelsus‐Elena Hospital, Kassel.

## EPO‐081

### Evaluation of non‐motor symptoms in Parkinson's disease with and without a GBA mutation: A cross sectional study

#### I. Muro ^1^; C. Albalat^1^; M. Pascual^2^; J. Masabanda^1^; F. Sanchez Cuesta^2^; E. Casas Peña^1^; P. Lorenzo Barreto^1^; B. González^1^; L. López Manzanares^1^; J. Romero^2^

##### ^1^Movement disorder Unit, La Princesa hospital, Madrid, Spain; ^2^Neurorehabilitation and brain damage Unit, Beata Maria Ana hospital, Madrid, Spain

**Background and aims:** Glucocerebrosidase gene mutations (GBA+) are the main genetic risk factor for Parkinson's disease (PD). Earlier onset and a higher burden of motor and non‐motor symptoms (NMS) have been described, but the whole range of non‐motor manifestations including balance have not been assessed.

**Methods:** A cross‐sectional observational study was conducted on 40 non demented PD patients (20 GBA+), matched by age, sex and PD duration. Motor and non‐motor variables were assessed using standardized tests and validated scales.

**Results:** Both groups were comparable in age and disease onset (5.05y GBA+ and 5.75y GBA‐). Non‐Motor Symptoms Scale score for GBA+ was higher (82.85 vs 71.05, p=0.914).Higher scores were also seen for GBA+ in King's Parkinson's Disease Pain Scale, modified Fatigue Impact Scale, Questionnaire for Impulsive Compulsive Disorders in Parkinson's Disease Rating Scale, Apathy Evaluation Scale and Beck Depression Inventory (see table 1). Instrumental posturography revealed lower global stability index and balance index in GBA+ patients (P=0.051 and P=0.091). Computerized reaction time tasks showed a tendency to slower execution and more mistakes in GBA+ patients with no statistically significant differences (see table 2).

**Figure:**
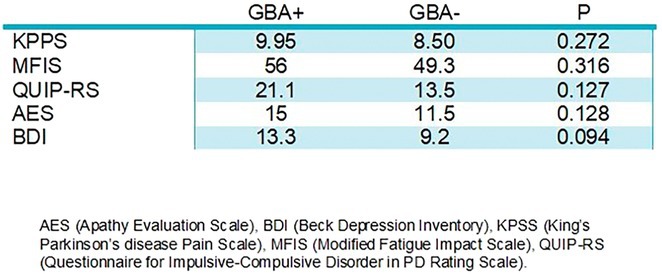
**TABLE 1** non motor symptoms assessment.

**Figure:**
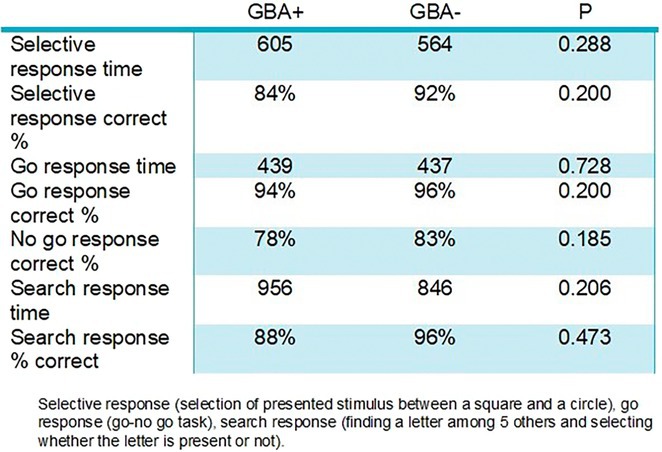
**TABLE 2** Computerized cognitive processing tasks

**Conclusion:** Higher burden of NMS in GBA+ was found as expected, as well as poorer balance performance, even though patients were in early stages and a tendency towards lower scores on cognitive processing speed. We will assess the progression of NMS and balance to identify potential markers to define a GBA+ phenotypic profile. These findings could guide the need for genetic testing in clinical practice.

**Disclosure:** Nothing to disclose.

## EPO‐082

### Expanding the clinical spectrum of STUB1‐related disorder: Dystonia and GPi‐DBS as therapeutic option

#### J. Kaprzak^1^; A. Sulek^2^; T. Janiszewska^3^; J. Slawek^1^; J. Dulski ^4^

##### ^1^Division of Neurological and Psychiatric Nursing, Faculty of Health Sciences, Medical University of Gdansk, Gdansk, Poland; Neurology Department, St Adalbert Hospital, Copernicus PL Ltd., Gdansk, Poland; ^2^Department of Genetics, Institute of Psychiatry and Neurology, Warsaw, Poland; Faculty of Medicine, Lazarski University, Warsaw, Poland; ^3^Department of Genetics, Antoni Jurasz University Hospital No. 1, Bydgoszcz, Poland; ^4^Division of Neurological and Psychiatric Nursing, Faculty of Health Sciences, Medical University of Gdansk, Gdansk, Poland; Neurology Department, St Adalbert Hospital, Copernicus PL Ltd., Gdansk, Poland; Department of Neurology, Mayo Clinic Florida, USA

**Background and aims:** STUB1 mutations cause hereditary ataxias, with additional features including dementia and extrapyramidal symptoms. There is no literature on the therapeutic role of deep brain stimulation (DBS) in this disorder.

**Methods:** We personally evaluated proband and her two siblings, and reviewed clinical records and videos of their mother. Genetic investigations included targeted assays for C9orf72, SCA1, 2, 3, 6, 7, 8, 12, 17, DRPLA, DYT6, and mitochondrial disorders. Exome sequencing was performed in the proband, followed by Sanger sequencing in her siblings and mother.

**Results:** Proband, a 50‐year‐old female, presented with cervical dystonia at age 33, which was generalized by age 45. Brain MRI showed marked cerebellar atrophy (Figure 1). Neuropsychological evaluation revealed cognitive deficits (ACE‐III score: 80/100). CSF analysis found elevated total tau (181 pg/ml) with normal phospho‐tau (29 pg/ml) and beta‐amyloid (891 pg/ml). At 48, the patient underwent bilateral GPi‐DBS, resulting in moderate improvement (CGI=+2). Proband's mother developed dysarthria at 41, followed by spastic tetraparesis, cerebellar ataxia, and dementia. By age 80, she was significantly disabled, with limited verbal contact. Her brain MRI revealed pronounced cerebellar atrophy. Proband's brother reported muscle fatigue and tremulousness at 41, and electromyography indicated neurogenic involvement. The sister, aged 52, remained asymptomatic. Exome sequencing identified heterozygous STUB1 c.146A>G (p.Tyr49Cys) variant in the proband. Sanger sequencing confirmed the variant in the mother and brother, but not in the unaffected sister. Genetic testing for other hereditary conditions was negative.

**Figure:**
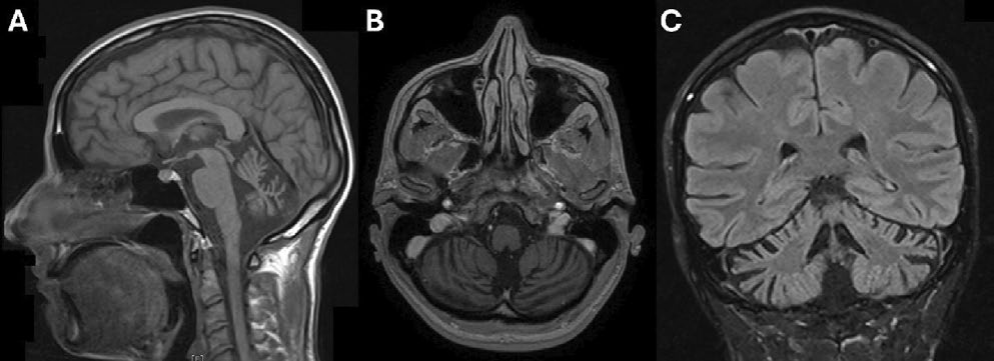
**FIGURE 1** Brain MRI of the proband at 46 years old demonstrating cerebellum atrophy on T1‐weighted sagittal (A) and axial (B), and T2‐weighted coronal (C) sequences.

**Conclusion:** STUB1 mutations can present with dystonia and cognitive impairment without ataxia. GPi‐DBS may offer therapeutic benefits in STUB1‐related dystonia.

**Disclosure:** J. Kasprzak reports no disclosures relevant to the manuscript. A. Sulek reports no disclosures relevant to the manuscript. T. Janiszewska reports no disclosures relevant to the manuscript. J. Slawek serves as a co‐editor‐in‐chief of Neurologia i Neurochirurgia Polska. He received consultancies from Allergan, Abbvie, Ipsen, Everpharma, Merz, Novartis, Biogen, Roche, TEVA. He received speakers’ bureau honoraria from Allergan, Abbvie, Ipsen, Everpharma, Merz, Novartis, Biogen, Roche, TEVA. He has intellectual property rights for “Application of Hydrogen Peroxide and 17β ‐Estradiol and its Metabolites as Biomarkers in a Method of Diagnosing Neurodegenerative Diseases In Vitro” (WO/2023/234790). J. Dulski is partially supported by the Polish Minister of Science (Scholarship for Outstanding Young Scientists, SMN/19/1279/2023), and the Haworth Family Professorship in Neurodegenerative Diseases fund (90052067). He serves as an editorial board member of Neurologia i Neurochirurgia Polska. He received speakers’ bureau honoraria from VM Media Ltd., Radosław Lipiński 90 Consulting, Ipsen. He has intellectual property rights for “Application of Hydrogen Peroxide and 17β ‐Estradiol and its Metabolites as Biomarkers in a Method of Diagnosing Neurodegenerative Diseases In Vitro” (WO/2023/234790).

## EPO‐083

### Effectiveness of opicapone added to different levodopa doses in Parkinson's: Post‐Hoc analysis of the ADOPTION trials

#### J. Ferreira ^1^; J. Lee^2^; H. Ma^3^; B. Jeon^4^; W. Poewe^5^; A. Antonini^6^; F. Stocchi^7^; D. Rodrigues^8^; M. Fonseca^9^; H. Brigas^8^; J. Holenz^8^; O. Rascol on behalf of studies’ investigators^10^

##### ^1^IMM ‐ oão Lobo Antunes Institute of Molecular Medicine, Faculty of Medicine, University of Lisbon, Lisbon, Portugal; ^2^Department of Neurology, SMG‐SNU Boramae Medical Center, Seoul, Republic of Korea; ^3^Department of Neurology, Hallym University Sacred Heart Hospital, Anyang, Republic of Korea; ^4^Department of Neurology, Seoul National University Hospital, Seoul, Republic of Korea; ^5^Department of Neurology, Medical University of Innsbruck, Innsbruck, Austria, ^6^7Department of Neurosciences, University of Padova, Padova, Italy; ^7^University San Raffaele Roma and Institute for Research and Medical Care IRCCS San Raffaele, Roma, Italy; ^8^BIAL – Portela & Ca S.A., Coronado, Portugal; ^9^BIAL R&D Investments, S.A., Portugal, ^10^University of Toulouse 3, University Hospital of Toulouse, INSERM; Clinical Investigation Center CIC1436 Departments of Neurosciences and Clinical Pharmacology and NS‐Park/FCRIN network; Toulouse, France

**Background and aims:** This study assessed the efficacy of opicapone 50 mg compared to an additional 100 mg dose of levodopa in reducing OFF‐time in Parkinson's disease (PD) patients experiencing the first signs of motor fluctuations using different levodopa dosing regimens.

**Methods:** The ADOPTION clinical program included two 4‐week, randomised (1:1), open‐label studies in South Korea and Europe. PD patients with early wearing‐off received opicapone 50 mg or an increased dose of levodopa (increased by 100 mg/day) as add‐on to standard levodopa therapy. In this exploratory post hoc analysis, change from baseline in absolute OFF‐time (primary endpoint) was evaluated in two subgroups: patients receiving ≤400 mg/day of levodopa (low‐dose group) and those receiving >400–≤600 mg/day of levodopa (moderate‐dose group) at baseline (Table 1).

**Figure:**
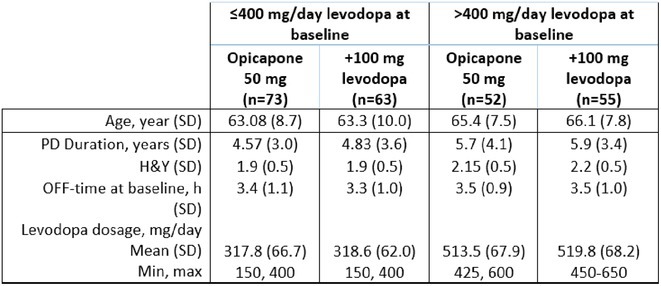
**TABLE 1** Baseline characteristics (randomised set). H&Y and Yahr; PD, Parkinson’s disease; SD, standard deviation.

**Results:** OFF‐time reduction was consistently greater with opicapone 50 mg than the increased levodopa dose. In the low‐dose group, mean (95% confidence interval) OFF‐time reduction was ‐60.12 (‐85.77, ‐34.47) minutes with opicapone versus ‐40.40 (‐64.98, ‐15.83) minutes with levodopa. In the moderate‐dose group, OFF‐time reduction was ‐66.27 (‐86.27, ‐46.18) minutes with opicapone and ‐24.91 (‐55.22, 5,4) minutes with 100 mg levodopa (Figure 1). While opicapone maintained a consistent effect across levodopa dose ranges, the additional 100 mg levodopa dose showed a trend toward reduced efficacy at higher baseline levodopa doses.

**Figure:**
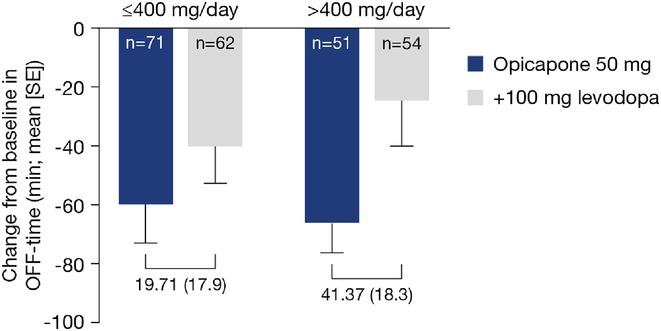
**FIGURE 1** Mean (SE) change from baseline to end of study treatment in absolute OFF‐time. SE, standard error; min, minutes.

**Conclusion:** Opicapone consistently reduced OFF‐time by ~1 h independently from the total levodopa dose at baseline and was more efficacious than an increased levodopa dose, suggesting that it is an effective strategy for early motor fluctuations in PD patients.

**Disclosure:** JJF received grants/honoraria from GlaxoSmithKline, Grunenthal, Fundação MSD, TEVA, MSD, Allergan, Novartis and Medtronic, GlaxoSmithKline, Novartis, TEVA, Lundbeck, Solvay, BIAL, Merck‐Serono, Merz, Ipsen, Biogen, Acadia, Allergan, Abbvie, Sunovion Pharmaceuticals, Zambon, Affiris and Angelini. JYL received grants/honoraria from NRF, SMG‐SNU, Eisai Korea, Bial, SK Chemicals. BJ received grants from Peptron and Abbvie Korea. WP received honoraria from Alterity, AbbVie, Affiris, AstraZeneca, Axovant, BIAL, Biogen, Britannia, Lilly, Lundbeck, NeuroDerm, Neurocrine, Denali Pharma, Orion Pharma, Roche, Stada, Sunovion, Takeda, UCB, Zambon, Michael J. Fox Foundation, EU FP7 and Horizon 2020. AA received compensation/support from UCB, Boehringer Ingelheim, Britannia, AbbVie, Zambon, BIAL, NeuroDerm, Theravance Biopharma, Roche, Chiesi Pharma, Lundbeck, Horizon, Ministry of Education University and Cariparo Foundation. FS received honoraria from Lundbeck, UCB, Chiesi, Zambon, Britannia, Cynapsus, Sunovion, Kyowa, Abbvie, Neuroderm, Biogen and BIAL. OR provided consultancy for/received grants from institutions including AbbVie, Adamas, Acorda, Addex, AlzProtect, ApoPharma, AstraZeneca, Axovant, BIAL, Biogen, Britannia, Buckwang, CereSpir, Clevexel, Denali, INC Research, IPMDS, Lundbeck, Lupin, Merck, Novartis, CHU, France‐Parkinson, INSERM, Michael J. Fox Foundation and Cure Parkinson UK. DMR, MMF, HB, GC‐F and JH are Bial employees.

## EPO‐084

### PSMF1 variants: A rare cause of early‐onset Parkinson's disease

#### L. Magistrelli ^1^; L. Straniero^2^; M. Brumana^2^; E. Contaldi^1^; G. Pezzoli^3^; I. Isaias^4^; R. Asselta^2^

##### ^1^ASST G. Pini‐CTO, Parkinson Institute of Milan, Milan, Italy; ^2^Department of Biomedical Sciences, Humanitas University, Pieve Emanuele, Milan, Italy; IRCCS Humanitas Research Hospital, Rozzano, Milan, Italy; ^3^Fondazione Pezzoli per il Morbo di Parkinson, Milan, Italy; ^4^ASST G. Pini‐CTO, Parkinson Institute of Milan, Milan, Italy; Department of Neurology, University Hospital Würzburg, Würzburg, Germany

**Background and aims:** Biallelic Proteasome Inhibitor Subunit 1 (PSMF1) variants have been described in patients with early‐onset Parkinson's disease (PD) mainly presenting with atypical aspects.

**Methods:** 1091 DNA samples (collected at the Parkinson Institute of Milan between 2002 and 2023) from early onset PD patients and/or positive family history were analysed with whole‐exome sequencing.

**Results:** Biallelic PSMF1 variants were detected in one patient (estimated prevalence: 0.0009). This woman presented bradykinesia, rest tremor of the right hand, micrography started at the age of 46. She suffered from a depressive syndrome started at the age of 27. Her maternal grandmother was diagnosed with PD at the age of 80. No hyposmia, constipation, or rapid eye movement sleep behaviour disorder were referred. Brain MRI was unremarkable, 123I‐FP‐CIT single‐photon emission computed tomography was compatible with parkinsonism. Ropinirole improved tremor but induced hallucinations and confusion at higher doses. Chronic treatment with levodopa was started with benefit. Two years later (four years from the first symptoms onset) she developed motor fluctuations (wearing‐off, morning akinesia, peak‐dose dyskinesia). At neurological examination no atypical signs were present. The patient died at the age of 64. Genetic analysis revealed the presence of two PSMF1 variants: c.129+2T>C (affecting position +2 of the donor splice site of exon 1) and c.725G>A (p. R242H). Both have already been reported; interestingly, the c.129+2T>C variant have not been associated to PD yet.

**Conclusion:** We confirm that biallelic PSMF1 variants cause a very rare form of early‐onset PD, which may also present with a more typical clinical phenotype.

**Disclosure:** Nothing to disclosure.

## EPO‐085

### Drug use and long‐term Parkinson's disease risk: A systematic screening approach

#### L. Vignatelli ^1^; E. Baldin^1^; L. Belotti^1^; F. Baccari^1^; M. Schettino^1^; P. Cortelli^1^; G. Calandra‐Buonaura^1^; T. Riise^2^; F. Nonino^1^; C. Zenesini^1^

##### ^1^IRCCS Istituto Scienze Neurologiche, Bologna, Italy; ^2^University of Bergen, Norway

**Background and aims:** A systematic drug‐wide approach might be useful to identify drugs that (1) could be repurposed to treat Parkinson's disease (PD) or (2) could help explain PD pathogenesis (Romanowska 2023). The aim of this study was to investigate the drugs associated with a reduced or increased risk of PD in two cohorts of the Bologna Local Health Trust (BLHT), identified by both clinical diagnosis and health administrative databases.

**Methods:** Historical cohort study design with time‐dependent exposure. Population: residents in BLHT (680,000) aged ≥ 25 years without PD diagnosis, follow‐up 2010‐2023. Exposure: use of 94 drug classes (ATC codes, level 2) ascertained by administrative prescriptions data after at least 5 years. Endpoint: PD onset identified through (1) the ParkLink Bologna cohort or (2) a validated algorithm launched on healthcare databases. Associations between drug classes and PD risk estimated by means of Cox proportional hazard models.

**Results:** We identified 824 individuals with PD from ParkLink and 8,585 from algorithm. Protective association (Table 1) was found for ATC codes B03 “Antianemic”, C01 “Cardiac”, C03 “Diuretics”, H02 “Corticosteroids for systemic use” M04 “Antigout preparations”. Codes N06 “Psychoanaleptics” and G04 “Urologicals” showed an increased risk (Table 1).

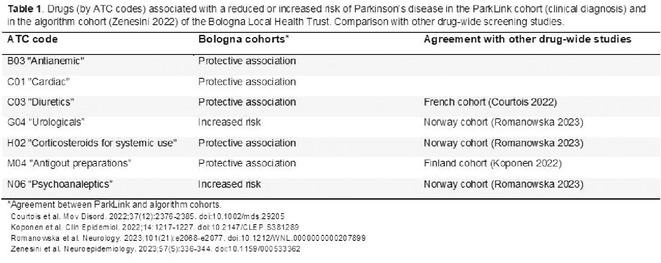

**Conclusion:** Specific drug classes may impact PD onset and progression, as suggested by consistent findings among different geographic and healthcare settings (Courtois 2022, Koponen 2022, Romanowska 2023) (Table 1). Many classes with anti‐inflammatory activity may have a protective role. Further studies exploring these associations at the 3rd to 5th level of ATC codes are warranted.

**Disclosure:** Nothing to disclose.

## EPO‐086

### sGFAP is elevated in essential tremor patients with late disease onset and short disease duration

#### L. Gattermeyer‐Kell; M. Khalil; D. Kern; S. Franthal; P. Katschnig‐Winter; M. Kögl; R. Demjaha; C. Tafrali; E. Hofer; R. Schmidt; C. Enzinger; P. Schwingenschuh

##### Department of Neurology, Medical University of Graz, Graz, Austria

**Background and aims:** The role of neurodegeneration in essential tremor (ET) remains debated, particularly in patients with late disease onset. Neuropathological studies have identified structural changes in the Purkinje cells and its connections. Recent studies additionally suggested a role of cerebellar astrocytes. Increased levels of serum glial fibrillary acidic protein (sGFAP), an astrocytic intermediate filament, were found in various neuroinflammatory and neurodegenerative diseases. The objective of this case‐control study was to investigate the role of sGFAP in ET focusing on early‐stage late‐onset patients.

**Methods:** sGFAP was quantified by single molecule array at baseline and 5‐years‐follow‐up in 36 ET‐patients and 36 age‐matched healthy controls. ET‐patients were assessed using the Fahn‐Tolosa‐Marin‐Tremor‐Rating‐Scale. The ET group was stratified (1) by median age at onset and median disease duration in early‐stage late‐onset and early‐onset/late‐stage ET, and (2) by median sGFAP‐level at baseline.

**Results:** Early‐stage late‐onset ET‐patients had higher baseline‐sGFAP than controls (p=0.023) and higher follow‐up‐sGFAP and annual sGFAP‐increase than both controls (p=0.023; p=0.007) and early‐onset/late‐stage ET‐patients (p=0.021; p=0.024). Baseline sGFAP‐level correlated with tremor severity at follow‐up in the early‐stage late‐onset (rs=0.704, p=0.011) but not in the early‐onset/late‐stage group. Patients with high compared to low sGFAP‐baseline levels had later disease onset (p<0.001) and sGFAP‐increase was associated to tremor progression only in high sGFAP‐patients (p=0.041). ET‐plus and pure‐ET‐patients did not differ in any of the sGFAP‐parameters.

**Conclusion:** sGFAP is elevated in early stages of late‐onset ET and associated to tremor progression, substantiating the role of a pathophysiological substrate in ET in this subgroup.

**Disclosure:** Nothing to disclose.

## EPO‐087

### The impact of antidopaminergic medications on assessment of function, cognition, and motor features on HD outcomes

#### R. Reilmann^1^ ; M. Geva^2^, A. Tan^2^, K. Chen^2^, W. Feng^2^, R. Hand^2^, A. Cruz‐Herranz^2^, M. Hayden^2^

##### ^1^George‐Huntington‐Institute, Muenster, Germany; ^2^Prilenia Therapeutics B.V., Naarden, The Netherlands

**Background and aims:** Antidopaminergics medications (ADMs; VMAT2 inhibitors and antipsychotics) are essential for Huntington's disease (HD) symptom management. However, potent anti‐dopamine activity of ADMs is associated with faster progression in measures of HD, including function (TFC), cognition (SWR/SDMT), motor (TMS), and the composite measure of progression (cUHDRS). No prospective randomized controlled trials (RCT) have addressed the impact of ADMs in HD over time. The Phase 3 PROOF‐HD trial (NCT04556656) evaluated the efficacy and safety of pridopidine in HD, and it is the first double‐blind, placebo‐controlled study presenting data assessing the impact of ADMs on a placebo group.

**Methods:** Participants on‐ or off‐ADMs in the placebo arm were compared across HD outcome measures. The most frequently used ADMs included olanzapine (18%), risperidone (18%), deutetrabenazine (13%), tetrabenazine (9%), aripiprazole (8%), tiapride (11%), and quetiapine (5%).

**Results:** By week 78, on‐ADM participants (n = 133) showed greater declines across all measures compared with off‐ADM participants (n = 112): TFC (Δ = ‐1.31 vs. ‐0.46, p<0.0001), cUHDRS (Δ = ‐1.29 vs. ‐0.49, p<0.0001), SDMT (Δ = ‐2.36 vs. ‐0.38, p=0.0003), SWR (Δ = ‐4.12 vs. ‐0.73, p=0.02), and TMS (Δ = 3.95 vs. 1.12, p = 0.01).

**Conclusion:** These findings underscore the impact of ADMs on HD outcomes, highlighting the need for careful consideration in patient‐care and clinical trial design. Future HD trials should account for ADM use, e.g., using natural history controls, to ensure accurate assessment of disease progression and treatment efficacy.

**Disclosure:** This study was sponsored by Prilenia Therapeutics.

## EPO‐088

### Patient profiles and first‐line treatment in Parkinson's disease: Study on the 2/100th French Nationwide claims database

#### B. Degos^1^; D. Devos^2^; C. Giordana^3^; A. Sommet^4^; O. Rascol^5^; M. Bennani ^6^; T. Rouaud^7^

##### ^1^Dynamics and Pathophysiology of Neuronal Networks Team, Center for Interdisciplinary Res. in Biology, Collège de France, CNRS/INSERM, Univ. PSL, Paris; Neurology Dept, Avicenne Univ. Hosp., Sorbonne Paris Nord Univ., NS‐Park/FCRIN Network, Bobigny; France; ^2^Univ. Lille, INSERM, CHU Lille, U1172 – LilNCog – Lille Neurosciences & Cognition, Lille, France; Department of Medical pharmacology CHU Lille, Lille, France; ^3^Neurology Department, Centre Hospitalier Universitaire de Nice, Université Côte d'Azur, Nice, France; ^4^Department of Medical Pharmacology, CIC 1436, Toulouse University Hospital, Toulouse, France; ^5^University of Toulouse 3, University Hospital of Toulouse, INSERM; Clinical Investigation Center CIC1436 Departments of Neurosciences and Clinical Pharmacology and NS‐Park/FCRIN network; Toulouse, France; ^6^Qualees, Paris, France; ^7^Department of Neurology, NS‐PARK/FCRIN Network, Nantes University Hospital, Nantes, France

**Background and aims:** The care pathways of patients with Parkinson's disease (PD) in France are poorly understood outside expert centers and clinical trials. What is the reality of their management? We describe the socio‐demographic and clinical characteristics of PD patients, as well as the first‐line antiparkinsonian treatment choices.

**Methods:** Retrospective cohort study of patients over 20 years of age identified has having PD in the sample of the French Nationwide claims database (ESND) from 01/01/2015 to 31/12/2021 by declaration of a long‐term illness, hospitalization for PD, or at least three dispensations of antiparkinsonian drugs within one year. The demographic and clinical profiles of patients, as well as the first‐line antiparkinsonian treatments, stratified by patient age at treatment initiation, was described.

**Results:** Among the 11,095 PD patients analysed, 50.1% were men. First‐line treatments for patients aged ≥70 years (63.3%) included levodopa + decarboxylase inhibitor (64.9%), piribedil (9.8%), and pramipexole (9.2%), with an average duration of 2.8 years, and a single treatment in 69.9% of cases. For patients aged <70 years, first‐line treatments included pramipexole (31.0%), levodopa + decarboxylase inhibitor (18.8%), and ropinirole (17.9%), with an average treatment duration of 3 years and a single treatment line in 53.6% of cases.

**Conclusion:** This study highlights differences in therapeutic strategies based on patient age at treatment initiation: a predominance of levodopa + decarboxylase inhibitor in patients over 70 years old and dopamine agonists in younger patients. These results are in line with the local recommendations of the time.

**Disclosure:** This research was conducted with support from Orion Pharma. BD, DD, CG, AS, OR and TR are members of Orion's advisory board. BD received honoraria for presentations from Merz and Ipsen. CG received honoraria from Abbvie and Ipsen. MB is president of the Contract Research Organization Qualees in charge of the study.

## EPO‐089

### Analysis of single PDQ‐39 items comprising the PDQ‐8 shows foslevodopa/foscarbidopa therapy improved PDQ‐8 summary index

#### P. Svenningsson^1^; A. Johansson^1^; P. Odin^2^; L. Bergmann ^3^; R. Gupta^3^; J. Samuelsson^3^; J. Parra^3^; F. Bergquist^4^

##### ^1^Karolinska University Hospital, Karolinska Institutet, Stockholm, Sweden; ^2^Skåne University Hospital, Lund University, Lund, Sweden; ^3^AbbVie Inc, North Chicago, USA; ^4^Sahlgrenska University Hospital, University of Gothenburg, Gothenburg, Sweden

**Background and aims:** The 39‐item Parkinson's Disease Questionnaire (PDQ‐39) is a disease‐specific instrument evaluating Parkinson's disease (PD) patients quality of life (QoL); however, this assessment is time‐consuming. The shorter PDQ‐8, comprised of 1 representative item from each of the 8 PDQ‐39 domains, is reported to have similar validity and responsiveness as PDQ‐39. Horváth et al. used the 8 representative items from PDQ‐39 assessments to calculate the PDQ‐8 Summary Index (PDQ‐8‐SI) and minimal clinically important difference (MCID) for PDQ‐8‐SI and PDQ‐39‐SI (5.94, 4.72, respectively). Our objective was to similarly derive and analyse PDQ‐8‐SI from PDQ‐39 assessments in advanced PD patients treated with 24‐hour foslevodopa/foscarbidopa (LDp/CDp) continuous subcutaneous infusion.

**Methods:** The PDQ‐8‐SI was calculated post hoc from 1 active‐controlled and 1 open‐label phase 3 trial using the approach above in patients treated with LDp/CDp or oral levodopa/carbidopa immediate‐release therapy for 12 weeks (NCT04380142), or with LDp/CDp for 52 weeks (NCT03781167), respectively. Nominal P values were calculated via one‐sample t‐test and analysis of covariance, or two‐sided paired‐sample t‐test.

**Results:** Figure 1 reports active‐controlled trial within‐group change from baseline and vs oral PDQ‐8‐SI improvement. Figure 2 reports open‐label trial PDQ‐8‐SI change from baseline. Table 1 reports each trial's calculated PDQ‐8‐SI and previously reported PDQ‐39‐SI. LDp/CDp overall safety was previously reported as generally well tolerated.

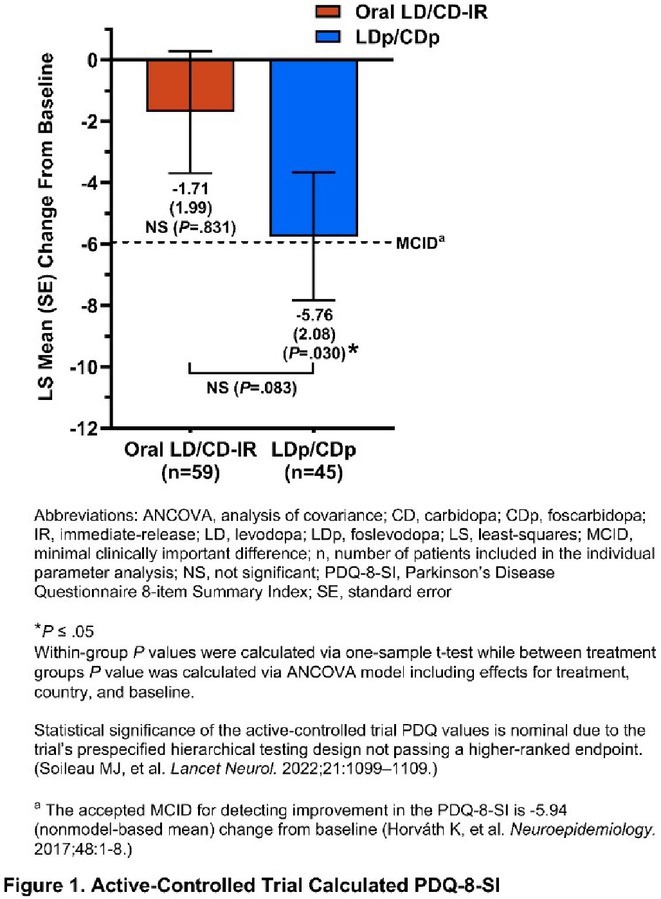

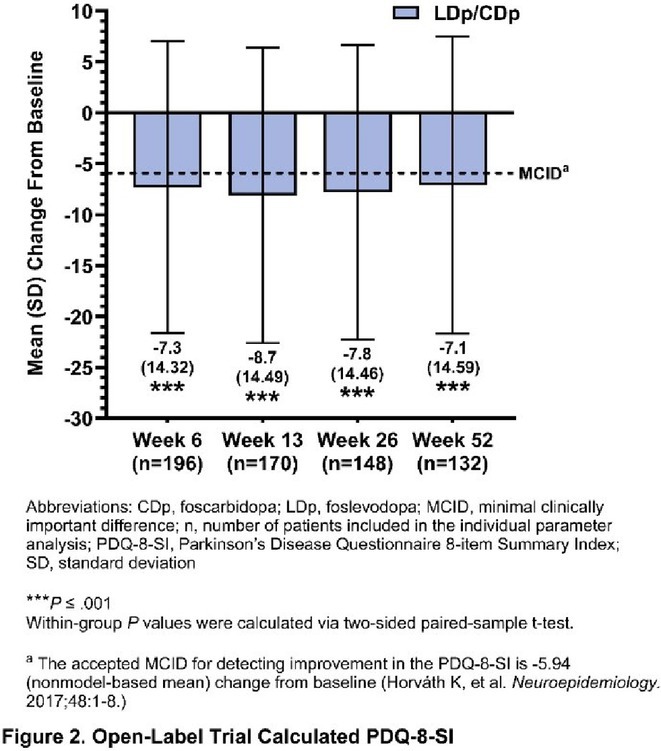

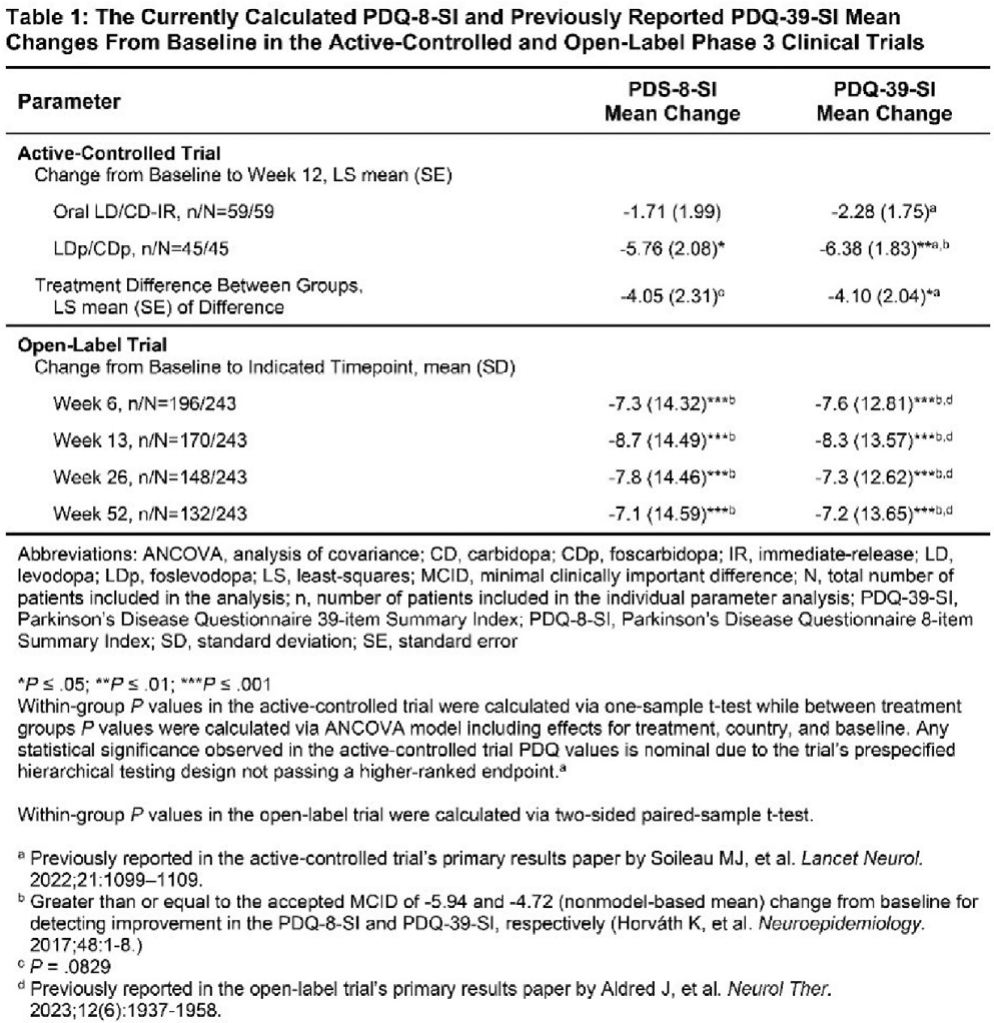

**Conclusion:** Both trial's calculated PDQ‐8‐SI showed similar nominally significant improvements vs baseline near/over MCID in LDp/CDp‐treated patients, as previously reported for PDQ‐39‐SI. These results suggest evaluating QoL in PD patients using PDQ‐8 could obtain results similar to PDQ‐39 with reduced patient/caregiver assessment time burden.

**Disclosure:** This analysis was supported by AbbVie Inc. Financial disclosures (currently or within the past 2 years): PS: is a study investigator and has received compensation from AbbVie Inc, Lundbeck, and Takeda for advisory fees; has received speaker activities honoraria from Zambon; and, has received research support from Knut and Alice Wallenberg Foundation, Stockholm City Council, Swedish Parkinson Fund, and the Swedish Brain Foundation. AJ served as an investigator in clinical trials sponsored by AbbVie Inc, BIAL, Integrative Research Laboratories and Neurolixis, and his institution has received honoraria from AbbVie Inc for his educational presentations. PO received compensation for: consultancy and speaker‐related activities from AbbVie Inc, Bial, Britannia, EVER Pharma, Nordic Infucare, STADA, and Zambon; and, for serving on scientific advisory or data safety monitoring boards for AbbVie Inc. PO's institution has received research support from AbbVie Inc, Lund University Medical Faculty, SFO Multipark, Parkinsonfonden, the Swedish Parkinson Academy, the Swedish Research Council, and Health Care Region Skåne. LB, RG, JS, and JCP are fulltime employees of AbbVie Inc, and may hold AbbVie Inc stock and/or stock options. FB has received compensation for lectures and advisory services plus in‐kind donations of PKG reports for clinical studies from GKC, and honorarium for an advisory board from AbbVie Inc. FB owns stock options in Dizlin Pharmaceuticals AB.

## MS and Related Disorders 1

## EPO‐090

### First results of the Multiple Sclerosis Autonomy Scale (MSAS) questionnaire

#### C. Donze^1^; G. Paillot^2^; C. Mekies^3^; M. Cohen^4^; L. Brechenmacher^5^; A. Civet^5^; D. Pau^5^; C. Mouzawak^6^; P. Vermersch ^7^

##### ^1^Hôpital saint Philibert, Groupement des Hôpitaux de l'Institut Catholique de Lille, Faculté de médecine et de maïeutique de Lille, Lomme, France; ^2^Association Aventure Hustive, Saint‐Malo, France; ^3^RAMSAY Clinique des Cèdres, Neurologie, Toulouse, France; ^4^Université Nice Cote d’Azur, UR2CA‐URRIS CRCSEP CHU Nice Pasteur, Service de Neurologie, Nice, 06002, France; ^5^Roche SAS, 4 cours de l'ile Seguin, 92000 Boulogne‐Billancourt, France; ^6^Structure régionale neuro SEP SYNAPSE, Le Vésinet, France; ^7^Universite Lille, INSERM UMR1172 LilNCog, CHU Lille, FHU Precise, Lille, France

**Background and aims:** Many MS patients have symptoms that impact on their autonomy, defined as being able to perform the roles that are most important to oneself, with or without help. MSAS is a new questionnaire that aims at evaluating patient autonomy in multiple sclerosis. Our current study's primary objective is to validate the psychometric properties of the MSAS questionnaire.

**Methods:** The FOCAL‐MS2 study will confirm the psychometric properties of the short version of the questionnaire. 210 pairs of healthcare professionals and patients were recruited to test the questionnaire for 1 year. This analysis describes the characteristics of patients and MSAS questionnaire results at inclusion. MSAS scores are standardized on 0‐100 scale, higher scores representing higher burden of autonomy.

**Results:** From the 210 patients included from January to April 2024, 199 completed the MSAS questionnaire at baseline: 74% women, mean age at diagnosis 34.3 +/‐ 9.9 years; 132 (66.3%) had a relapsing remitting form of MS (RRMS), 23 (11.5%) with primary progressive (PPMS) and 44 (22.1%) with secondary progressive (SPMS). The most important dimension for patients was support from their partner (mean score: 89.5±15.6) and consideration from the care team (mean score: 86.5±14.8). The least important dimension was the image projected to others (mean score: 69.7±24.0).

**Conclusion:** The population included in the study covers the different profiles of MS patients. Not all the dimensions are equally important for each patient, making it possible to identify individualised priorities that have a real impact on autonomy.

**Disclosure:** The authors declared the following potential conflicts of interest with respect to the research, authorship, and/or publication of this article: Cecile Donze: consulting and speaking honoraria from Biogen, BMS, Coloplast, Merck, Novartis, Roche, Sanofi, and Teva; Claude Mekies: honoraria for scientific advisory boards participation or contribution to scientific meetings from Bayer, Biogen, BMS, Celg`ene, Eisai, Genzyme, GSK, Janssen, Lilly, Merck, Novartis, Pfizer, Roche, Sandoz, Sanofi, and Teva; Geraud Paillot: no conflict of interest; Patrick Vermersch: honoraria, contribution to meetings from AB Science, Biogen, BMS‐Celgene, Imcyse, Janssen, Merck, Novartis, Roche, Sanofi‐Genzyme, Teva, and research supports from Merck, Novartis, and Sanofi‐Genzyme; Guillaume Montagu: as an employee of unknowns SAS, consulting for Alexion, Astellas, BMS, Celltrion, Chiesi, Gilead, Novo Nordisk, Pfizer, and Roche; Lucie Brechenmacher, Alexandre Civet and David Pau are employees from Roche SAS, France; Catherine Mouzawak: honoraria for consulting, scientific advisory boards participation, or other activities from Biogen, Janssen, Merck, Novartis, Sanofi, and Roche; Mikael Cohen: consulting honoraria from Ad Scientiam, Alexion, Biogen, BMS, Horizon Therapeutics, Merck, Novartis, and Roche.

## EPO‐091

### Inter‐network connectivity and consolidated resilience in adult and pediatric multiple sclerosis patients

#### D. Mistri^1^; M. Margoni^2^; P. Valsasina^1^; A. Meani^1^; M. Filippi^3^; M. Rocca ^4^

##### ^1^Neuroimaging Research Unit, Division of Neuroscience, IRCCS San Raffaele Scientific Institute, Milan, Italy; ^2^Neuroimaging Research Unit, Division of Neuroscience, Neurology Unit, and Neurorehabilitation Unit, IRCCS San Raffaele Scientific Institute, Milan, Italy; ^3^Neurology Unit, Neurorehabilitation Unit, Neurophysiology Service, and Neuroimaging Research Unit, Division of Neuroscience, IRCCS San Raffaele Scientific Institute, and Vita‐Salute San Raffaele University, Milan, Italy; ^4^Neurology Unit, and Neuroimaging Research Unit, Division of Neuroscience, IRCCS San Raffaele Scientific Institute, and Vita‐Salute San Raffaele University, Milan, Italy

**Background and aims:** Consolidated resilience (CR) is a measure that combines cognitive reserve and cognitive resilience. Our aim was to assess whether CR moderates the association between inter‐network functional connectivity and cognition in different age groups of multiple sclerosis (MS) patients.

**Methods:** A total of 268 MS patients underwent cognitive and 3.0T MRI assessment. CR scores were computed using a partial least‐squares path model combining proxy‐based measures of cognitive reserve (years of education and intelligence quotient) with residual‐based measures of cognitive resilience. Resting state fMRI scans were acquired from 25 adult MS patients, 24 sex‐ and disease duration‐matched pediatric MS patients. MS patients were re‐evaluated after a median follow‐up of 2.4 years (interquartile range 1.6–3.3 years). We identified the main cognitive networks by independent component analysis and calculated inter‐network connectivity through pairwise correlations. As a summary measure of connectivity strength for each network, we calculated the degree, i.e., the weighted sum of inter‐network correlation coefficients. Linear mixed models were used to evaluate the effect of CR and degree on cognitive changes.

**Results:** Linear mixed models showed significant three‐way interactions (CR x time x network degree) for verbal memory scores (frontoparietal network [β = 1.42, p = 0.010]) in pediatric MS patients, and for attentional/executive scores (salience network [β = 1.42, p = 0.010]; executive control network [β = ‐0.70, p = 0.008]) in adult MS patients.

**Conclusion:** The association between higher CR and better cognitive outcomes in MS patients is moderated by specific RS FC patterns between cognitive networks, which change with age.

**Disclosure:** DM, PV, AM have nothing to disclose. MM reports grants and personal fees from Sanofi Genzyme, Merck Serono, Roche, Biogen, Amgen and Novartis. MF received compensation for consulting services or speaking activities from Alexion, Almirall, Bayer, Biogen, Celgene, Chiesi Italia SpA, Eli Lilly, Genzyme, Janssen, Merck‐Serono, Neopharmed Gentili, Novartis, Novo Nordisk, Roche, Sanofi Takeda, and TEVA; Advisory Boards for Alexion, Biogen, Bristol‐Myers Squibb, Merck, Novartis, Roche, Sanofi, Sanofi‐Aventis, Sanofi‐Genzyme, Takeda; scientific direction of educational events for Biogen, Merck, Roche, Celgene, Bristol‐Myers Squibb, Lilly, Novartis, Sanofi‐Genzyme; he receives research support from Biogen Idec, Merck‐Serono, Novartis, Roche, the Italian Ministry of Health, the Italian Ministry of University and Research, and FISM. MAR received consulting fees from Biogen, Bristol Myers Squibb, Eli Lilly, Janssen, Roche, and speaker honoraria from AstraZaneca, Biogen, Bristol Myers Squibb, Bromatech, Celgene, Genzyme, Horizon Therapeutics Italy, Merck Serono SpA, Novartis, Roche, Sanofi and Teva, she receives research support from the MS Society of Canada, the Italian Ministry of Health, the Italian Ministry of University and Research, and FISM.

## EPO‐092

### Incidence of infection in 500 adults 55 years and older with multiple sclerosis treated with ocrelizumab

#### S. Lee^1^; R. Scheu^2^; F. Mateen ^1^

##### ^1^Department of Neurology, Massachusetts General Hospital, Boston, USA; ^2^Department of Public Health and Primary Care, School of Clinical Medicine, University of Cambridge, Cambridge, UK

**Background and aims:** Phase III clinical trials for Ocrelizumab, a disease modifying therapy for multiple sclerosis (MS), excluded patients >55 years; however, Ocrelizumab is widely used to treat people with MS 55 years and older.

**Methods:** A retrospective, observational cohort study was conducted at a high‐volume academic medical center in Boston, USA. The electronic medical records of 500 randomly selected adults aged 55 and older, who received at least one dose of Ocrelizumab IV (June 2017‐April 2024).

**Results:** Participants were 65% female, 92% White and, at last follow up, were aged: 55–59 years (n=133), 60–64 years (n=151), 65–69 years (n=105), 70–74 years (n=70), 75–79 years (n=29), and ≥ 80 years (n=12). Presentations were RRMS (45%), SPMS (22%), PPMS (15%), and atypical (2%). Average MS duration was 20 years. The median number of Ocrelizumab doses was 6. Ocrelizumab was a third‐line or later treatment in 55%. Hypogammaglobulinemia (IgG<600 mg/dL) occurred at least once in 52%. 30% of those tested (n=117) were JCV‐antibody seropositive (index level >1.5). During 21867.1 person‐months of observation, there were 882 infections, including urinary tract infections (n=420), COVID‐19 (n=172), upper respiratory tract infections (n=104), sepsis (n=12) and PML (also exposed to natalizumab, n=1). 105 hospitalizations and 1 death were related to infections. There were 32.5 non‐COVID infections per 1,000 person‐months of Ocrelizumab IV in older adults.

**Conclusion:** We depict a cohort of older adults clinically selected to take Ocrelizumab for MS and their safety profile.

**Disclosure:** F. Mateen has received research funding from EMD Serono, Genentech, Horizon Therapeutics, Novartis, and TG Therapeutics. S. Lee and R. Scheu have nothing to disclose.

## EPO‐093

### Cord lesion burden and atrophy in late‐onset multiple sclerosis: A comparative study with adult‐onset multiple sclerosis

#### G. Guido ^1^; N. Tedone^2^; P. Valsasina^3^; P. Preziosa^1^; F. Esposito^4^; M. Filippi^5^; M. Rocca^1^

##### ^1^Neuroimaging Research Unit, Division of Neuroscience, and Neurology Unit, IRCCS San Raffaele Scientific Institute, and Vita‐Salute San Raffaele University, Milan, Italy; ^2^Neuroimaging Research Unit, Division of Neuroscience, IRCCS San Raffaele Scientific Institute, and Vita‐Salute San Raffaele University, Milan, Italy; ^3^Neuroimaging Research Unit, Division of Neuroscience, IRCCS San Raffaele Scientific Institute, Milan, Italy; ^4^Neurology Unit, Division of Neuroscience, IRCCS San Raffaele Scientific Institute, Milan, Italy; ^5^Neurology Unit, Neurorehabilitation Unit, Neurophysiology Service, and Neuroimaging Research Unit, Division of Neuroscience, IRCCS San Raffaele Scientific Institute, and Vita‐Salute San Raffaele University, Milan, Italy

**Background and aims:** Late‐onset multiple sclerosis (LOMS) is defined when clinical onset starts after age 50. This study aimed to characterize LOMS patients in terms of cervical cord lesions and atrophy, compared to disease duration (DD)‐matched adult‐onset MS (AOMS), and correlate their cord damage with clinical scores.

**Methods:** A total of 101 MS patients (81 AOMS/20 LOMS, mean DD=2.1 years in both groups) and 83 healthy controls (HC, 57/26 matched with AOMS and LOMS, respectively) underwent following 3T MRI scans: (i) sagittal/axial cord T2‐weighted scans for cord lesion count and volume; (ii) brain 3D T1‐weighted MRI for mean upper cord cross‐sectional area (MUCCA) assessment; and (iii) in a sub‐group of n=73 patients, phase‐sensitive inversion‐recovery at C2/3 and C3/4 for cord grey matter (GM) area assessment. Patients also underwent disability, 9‐hole peg test (9HPT) and Timed 25‐foot walking test (T25‐FW) evaluation.

**Results:** Spinal cord lesion number/volume were not different between LOMS and AOMS. Also, we found no differences in MUCCA/cord GM area between LOMS/AOMS and their respective HC groups, nor between LOMS and AOMS. However, when looking at patients with longer DD (≥5 years), LOMS showed lower C3/4 GM area than AOMS (p=0.017). In LOMS, a higher cord lesions number correlated with higher T25‐FW (r=0.70, p=0.04), while a higher cord lesion volume with higher disability (r=0.51, p=0.02) and T25‐FW (r=0.58, p=0.007).

**Conclusion:** While lesion damage is comparable between LOMS and AOMS, cord GM atrophy seems to become more severe in LOMS vs AOMS as DD increases.

**Disclosure:** GG, NT, PV, FE have nothing to disclose. PP received speaker honoraria from Roche, Biogen, Novartis, Merck, Bristol Myers Squibb, Genzyme, Horizon and Sanofi. He received research support from Italian Ministry of Health and Fondazione Italiana Sclerosi Multipla (FISM). MF received compensation for consulting services or speaking activities from Alexion, Almirall, Bayer, Biogen, Celgene, Chiesi Italia SpA, Eli Lilly, Genzyme, Janssen, Merck‐Serono, Neopharmed Gentili, Novartis, Novo Nordisk, Roche, Sanofi Takeda, and TEVA; Advisory Boards for Alexion, Biogen, Bristol‐Myers Squibb, Merck, Novartis, Roche, Sanofi, Sanofi‐Aventis, Sanofi‐Genzyme, Takeda; scientific direction of educational events for Biogen, Merck, Roche, Celgene, Bristol‐Myers Squibb, Lilly, Novartis, Sanofi‐Genzyme; he receives research support from Biogen Idec, Merck‐Serono, Novartis, Roche, the Italian Ministry of Health, the Italian Ministry of University and Research, and FISM. MAR received consulting fees from Biogen, Bristol Myers Squibb, Eli Lilly, Janssen, Roche, and speaker honoraria from AstraZaneca, Biogen, Bristol Myers Squibb, Bromatech, Celgene, Genzyme, Horizon Therapeutics Italy, Merck Serono SpA, Novartis, Roche, Sanofi and Teva, she receives research support from the MS Society of Canada, the Italian Ministry of Health, the Italian Ministry of University and Research, and FISM.

## EPO‐094

### Identifying new candidate biomarkers through proteomic analysis of cerebrospinal fluid in multiple sclerosis

#### G. Salemi ^1^; F. Vaglica^2^; M. Lo Pinto^3^; F. Geraci^2^; S. Scilabra^3^; P. Cancemi^2^; P. Ragonese^1^

##### ^1^BIND Department, University of Palermo, Italy; ^2^STEBICEF Department, University of Palermo, Italy; ^3^Proteomics Group, Department of Research, ISMETT‐IRCCS, Ri.MED Foundation, Palermo, Italy

**Background and aims:** Multiple Sclerosis (MS) is a chronic disease recognized as a complex disorder influenced by various factors, where the interplay between genetic predispositions and environmental elements contributes to disease onset. Recognizing these differences may help anticipate how the disease will evolve and elucidate the distinct ways MS patients respond to various treatments. Key biomarkers play a role in enhancing patient diagnosis, treatment strategies, and prognostic evaluations. These biomarkers, (e.g., cytokines, chemokines, RNAs), highlight the diverse contributions of the immune system to the pathophysiology and progression of MS. Unfortunately, none of them demonstrate specificity for MS

**Methods:** Here we define a proteomics MS signature by using mass spectrometry in cerebrospinal fluid (CSF) of MS patients (n = 15) compared to not affected controls (NC) (n = 12).

**Results:** A total of 964 proteins were identified. Among these, 72 were exclusively found in MS patients, while 46 proteins were differentially expressed between NC and MS. MS patients exhibit unique proteins, such as proteolytic enzymes, cell adhesion factors, and those linked to stress and immune activity. Moreover, an upregulation of IGFBP‐2 and a downregulation of IGF‐2 was found. This imbalance could impair the neuroprotective effects of IGF‐1, contributing to neurodegeneration. Machine learning was employed to assess which of the proteomic collection of identified proteins could be used to better classify MS compared to NC. It identified proteins effective in distinguishing MS from NC (Figure).

**Figure:**
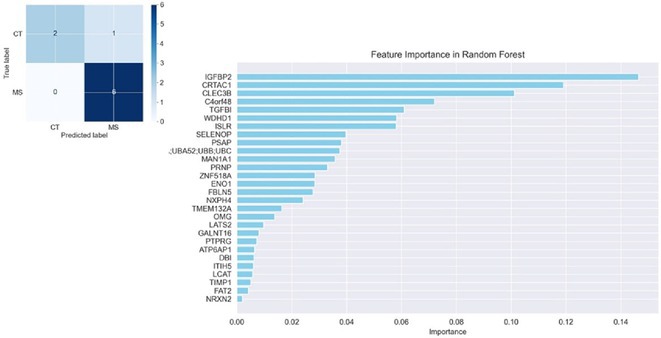
**FIGURE 1** Putative candidate markers from CSF proteomic analyses

**Conclusion:** The putative proteomic biomarkers here identified, provide valuable information for MS management. IGFBP2 was validated as promising MS biomarkers in CSF.

**Disclosure:** Nothing to disclose.

## EPO‐095

### Efficacy and safety of Ofatumumab in the first year of clinical practice: A multicenter study

#### I. Gómez‐Estévez^1^; J. Chico García^2^; S. Pastor‐Yvorra^3^; I. Moreno Torres^4^; I. Esain González^5^; A. García Leal^6^; B. Pilo de La Fuente^7^; F. Valenzuela Rojas^8^; L. Borrega Canelo^9^; N. Juárez Torrejón^10^; M. Gómez‐Moreno^11^; L. García‐Vasco^1^; L. Costa‐Frossard Françaluci^2^; C. González‐Ávila^3^; Orviz García aida^4^; R. Blasco Quilez rosa^5^; I. Puertas Muñoz^6^; Y. Aladro Benito^7^; S. De la Fuente Batista^4^; J. Sabin Muñoz^5^; C. Oreja‐Guevara ^1^

##### ^1^Hospital Clínico San Carlos, IdISSC, Madrid, Spain; ^2^Hospital Universitario Ramón y Cajal, IRYCIS, Universidad de Alcalá, Madrid, Spain; ^3^Hospital General de Villalba, Madrid, Spain; ^4^Hospital Universitario Fundación Jiménez Díaz, Madrid, Spain; ^5^Hospital Puerta de Hierro, Madrid, Spain; ^6^Hospital Universitario La Paz, Madrid, Spain; ^7^Hospital Universitario de Getafe, Madrid, Spain; ^8^Hospital Central de La Defensa Gómez Ulla, Madrid, Spain; ^9^Hospital Universitario Fundación Alcorcón, Madrid, Spain, ^10^Hospital Universitario Severo Ochoa, Madrid, Spain, ^11^Hospital Universitario Infanta Leonor, Madrid, Spain

**Background and aims:** The main goal was to analyze the efficacy and safety in MS patients treated with ofatumumab in real‐world clinical practice.

**Methods:** Retrospective multicentre cohort study included patients treated with Ofatumumab using data from the EMCAM (Multiple Sclerosis Study group of Madrid) cohort. We described the clinical and demographic characteristic of the patients and analyzed the effectiveness and safety in the first year of treatment.

**Results:** A total of 285 patients were included. The clinical and demographic characteristics are shown in table 1. 15.1% of patients switched to ofatumumab due to relapse, 31.2% due to radiological activity and 18.7% for both reasons. 35% switched for other reasons such as safety concerns or adverse events (AE). 9 patients have discontinued treatment three by personal choice, two due to radiological disease activity, two due to relapse, one for both reasons and one for safety reasons. After one year of treatment with ofatumumab, the ARR decreased significantly to 0.02 and the mean of gadolinium‐enhancing lesions decreased to 0.04. EDSS remained stable at one year 2,2 (0‐8). Baseline IgG and IgM levels were 10.12 (4.33‐17.2) and 1.17 (0.11‐5.37) G/L respectively. After twelve months IgG levels had decreased to 9.9 (4‐17.7) and IgM to 0.86 (0.14‐4.58) G/L. Adverse events were reported by 26.7% of patients after the first dose, with 67.7% of these experiencing mild flu‐like symptoms. Three patients experienced herpes simplex during treatment.

**Figure:**
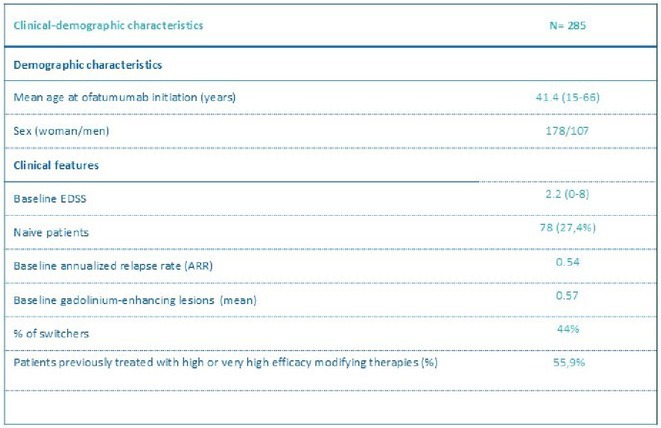
**FIGURE 1** Clinical and demographic characteristics

**Conclusion:** Ofatumumab was well‐tolerated, with no severe adverse events. After one year, significant reductions in ARR and Gd+ lesions were observed, supporting its efficacy and safety in treating relapsing multiple sclerosis.

**Disclosure:** none of the co‐authors have any conflict of interest. This work has not been funded.

## EPO‐096

### McDonald criteria application by German neurologists suggests a need for further training

#### J. Heinemann ^1^; D. Yankov^1^; A. Solomon^2^; S. Rauer^1^; H. Wiendl^1^; R. Dersch^1^

##### ^1^Department of Neurology, Medical Center, University of Freiburg, Faculty of Medicine, Freiburg, Germany; ^2^Larner College of Medicine at the University of Vermont, Department of Neurological Sciences, University Health Center ‐ Arnold 2, 1 South Prospect Street, Burlington, USA

**Background and aims:** McDonald criteria (MC) are a globally accepted standard for the diagnosis of multiple sclerosis (MS). Misdiagnosis of MS is a common problem that has significant clinical consequences for patients. Misapplication of MC is a potential source of MS misdiagnosis. Recent research has identified elements of the criteria that are frequently misunderstood by neurologists in the US. The aim of this study was to assess application of MC by neurologists in Germany.

**Methods:** A previously developed survey instrument was modified and distributed to neurology residents and specialists (general neurology, not MS‐subspecialists).

**Results:** 68 neurologists (42 neurology residents (NR) and 26 neurology specialists (NS) completed the survey. We found frequent misapplication of MC. Symptoms atypical for MS were mistaken as typical by 31% of participants. Understanding of MRI dissemination in space criteria was poor. Periventricular and juxtacortical lesions were incorrectly identified by 46% and 55%, respectively. Most participants accepted purely anamnestic reports of previous neurological symptoms without objective clinical evidence as sufficient to prove dissemination in time.

**Conclusion:** Training and continuing education on MS diagnostic criteria needs to be improved, especially concurrent with dissemination of future iterations of MC.

**Disclosure:** Nothing to disclose.

## EPO‐097

### Characterizing functional and structural imaging features of cognitive phenotypes in multiple sclerosis

#### K. Jain^1^; P. Valsasina^1^; D. Mistri^1^; A. Meani^1^; P. Preziosa ^2^; M. Filippi^3^; M. Rocca^2^

##### ^1^Neuroimaging Research Unit, Division of Neuroscience, IRCCS San Raffaele Scientific Institute, Milan, Italy; ^2^Neurology Unit, and Neuroimaging Research Unit, Division of Neuroscience, IRCCS San Raffaele Scientific Institute, and Vita‐Salute San Raffaele University, Milan, Italy; ^3^Neurology Unit, Neurorehabilitation Unit, Neurophysiology Service, and Neuroimaging Research Unit, Division of Neuroscience, IRCCS San Raffaele Scientific Institute, and Vita‐Salute San Raffaele University, Milan, Italy

**Background and aims:** This study aims at identifying distinct cognitive phenotypes in a large cohort of MS patients and characterize their clinical, structural MRI and resting state (RS) functional connectivity (FC) features.

**Methods:** We enrolled 369 right‐handed MS patients and 168 age‐and sex‐matched healthy controls (HC). Participants underwent neurological and neuropsychological evaluation using the Rao Brief Repeatable Battery of Neuropsychological tests. 3.0T MRI was used to derive atrophy measures and RS FC in seven cognitive networks. Latent Profile Analysis on cognitive Z‐scores identified MS cognitive phenotypes.

**Results:** Four cognitive phenotypes were detected: preserved cognition (PC) [n=67 (18.2%)], mild single domain‐verbal fluency involvement (MSD‐VF) [n=37 (10.0%)], mild single domain‐attention (MSD‐A) involvement [n=197 (53.4%)], and severe‐multidomain (SMD) involvement [n=68 (18.4%)]. SMD had worse clinical and atrophy features compared to the remaining phenotypes (p=range <0.001‐0.04). PC patients had similar RS FC to HC, while MSD‐VF patients presented with increased RS FC of the executive control (ECN) (p=0.03) and language (p=0.02) network compared to SMD patients. Conversely, SMD patients had significantly decreased default‐mode network (DMN) RS FC compared to other phenotypes (p=0.01‐0.04). Finally, we observed significant quadratic trends (corresponding to increased RS FC in MSD‐VF followed by decreased RS FC in MSD‐A and SMD) for DMN (p=0.005), ECN (p=0.005) and language (p<0.001) networks.

**Conclusion:** While PC patients exhibited normal RS FC, SMD patients presented widespread structural abnormalities and severe DMN RS FC decrease. Observed functional changes may represent a continuum from compensation in the earlier MS stages to a complete breakdown when impairment becomes severe.

**Disclosure:** Disclosures. KJ was funded by MSIF and ECTRIMS in the form of MSIF‐ECTRIMS McDonald Fellowship. PV, DM and AM have nothing to disclose. PP received speaker honoraria from Roche, Biogen, Novartis, Merck, Bristol Myers Squibb, Genzyme, Horizon and Sanofi. He received research support from Italian Ministry of Health and Fondazione Italiana Sclerosi Multipla (FISM). MF received compensation for consulting services or speaking activities from Alexion, Almirall, Bayer, Biogen, Celgene, Chiesi Italia SpA, Eli Lilly, Genzyme, Janssen, Merck‐Serono, Neopharmed Gentili, Novartis, Novo Nordisk, Roche, Sanofi Takeda, and TEVA; Advisory Boards for Alexion, Biogen, Bristol‐Myers Squibb, Merck, Novartis, Roche, Sanofi, Sanofi‐Aventis, Sanofi‐Genzyme, Takeda; scientific direction of educational events for Biogen, Merck, Roche, Celgene, Bristol‐Myers Squibb, Lilly, Novartis, Sanofi‐Genzyme; he receives research support from Biogen Idec, Merck‐Serono, Novartis, Roche, the Italian Ministry of Health, the Italian Ministry of University and Research, and FISM. MAR received consulting fees from Biogen, Bristol Myers Squibb, Eli Lilly, Janssen, Roche, and speaker honoraria from AstraZaneca, Biogen, Bristol Myers Squibb, Bromatech, Celgene, Genzyme, Horizon Therapeutics Italy, Merck Serono SpA, Novartis, Roche, Sanofi and Teva, she receives research support from the MS Society of Canada, the Italian Ministry of Health, the Italian Ministry of University and Research, and FISM.

## EPO‐098

### Cognitive impairment and serum neurofilament level predict disease progression in pediatric multiple sclerosis

#### M. Achille ^1^; G. Lucisano^2^; L. Margari^3^; M. Simone^3^

##### ^1^Neurology Unit, Department of Translational Biomedicines and Neurosciences, University of Bari Aldo Moro, Piazza Giulio Cesare 11, 70124 Bari, Italia; ^2^Center for Outcomes Research and Clinical Epidemiology ‐ CORESEARCH, Pescara, Italy; ^3^Child and Adolescence Neuropsychiatry Unit, Department of Precision and Regenerative Medicine and Jonic area (DiMepreJ), University of Bari Aldo Moro, Piazza Giulio Cesare n.11, 70124 Bari, Italia

**Background and aims:** To identify predictors of disease progression in pediatric multiple sclerosis (POMS).

**Methods:** Thirty‐five POMS patients (median age: 15; 13.0‐16.0), under treatment with disease‐modifying therapies (DMTs) were followed up for 18 months and underwent physical disability (via expanded disability status scale ‐ EDSS) and cognitive performance (via Symbol Digit modalities test ‐ SDMT) assessments, and MRI. Serum neurofilament light chain (sNfl) and glial fibrillary acidic protein (GFAP) levels were measured at baseline and at the end of follow‐up. Radiological progression was defined as an increase in T2 and/or Gd‐positive lesions; clinical progression was defined as an EDSS increase of >=0.5 points.

**Results:** At baseline, mean EDSS score was 2.0, median SDMT was 44.0, median sNfl and GFAP levels were 18.1 pg/mL and 103.63 pg/mL, respectively. At follow‐up, 37.1% experienced clinical progression, and 62.8% radiological progression. 63% showed cognitive improvement. As for biomarkers, 68% experienced a reduction in sNfl levels; GFAP levels remained stable. 54% of patients switched to high‐efficacy therapy (HETs). A statistically significant association was found between lower SDMT scores and higher sNfl levels; between radiological progression and lower SDMT scores; between clinical progression and lower baseline EDSS scores. A trend of association was found between radiological progression and higher baseline sNfl levels. Cerebellar involvement and frequent relapses were significatively associated with switching to HETs.

**Figure:**
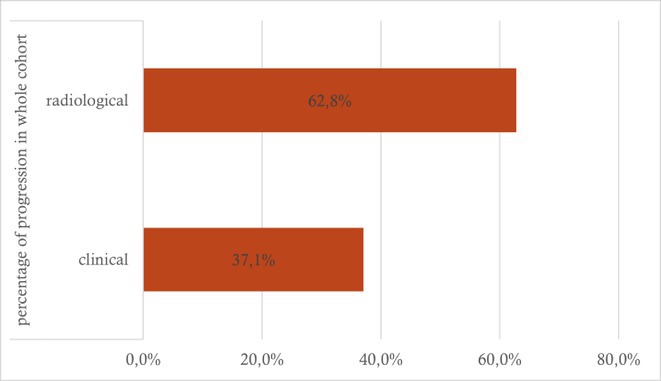
**FIGURE 1** Disease progression.

**Figure:**
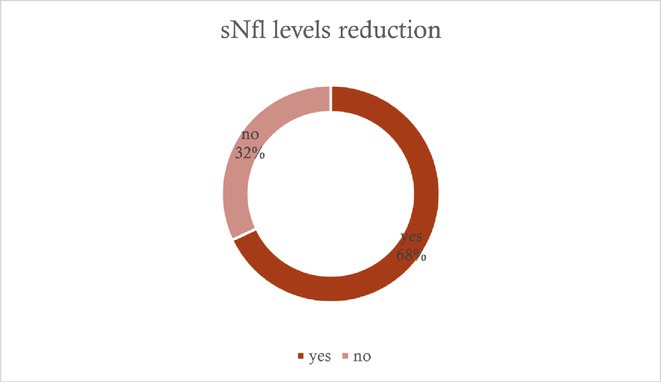
**FIGURE 2** sNfl levels reduction.

**Conclusion:** Cognitive impairment and elevated sNfl levels at disease onset predict disease progression. This confirms the need for early cognitive and neurodegeneration assessments to inform targeted therapies.

**Disclosure:** Nothing to disclose.

## EPO‐099

### Ocrelizumab virtually eliminates new cortical lesions in people with multiple sclerosis

#### N. Cavalli ^1^; G. Boffa^1^; E. Cipriano^1^; C. Lapucci^2^; V. Boccia^1^; S. Al Qudsi^1^; T. Sirito^1^; G. Lombardi^1^; E. Leveraro^1^; S. Magon^3^; A. Uccelli^1^; M. Cellerino^1^; M. Inglese^1^

##### ^1^Department of Neurosciences, Rehabilitation, Ophthalmology, Genetics and Maternal and child sciences, University of Genoa, Genoa, Italy; ^2^Neurology Unit, San Martino Hospital, Genoa, Italy; ^3^Pharma research and early development, Neuroscience and rare diseases Roche innovation center Basel, F. Hoffmann‐La Roche Ltd., Basel, Switzerland

**Background and aims:** Cortical lesions (CLs) are a common finding in multiple sclerosis (MS) but data regarding the impact of treatment on their evolution are lacking. This study evaluates CLs status and new CLs formation in a cohort of patients treated with ocrelizumab (OCR), their correlation with neurological disability and with white matter lesions (WMLs) accrual.

**Methods:** 92 relapsing‐remitting MS patients [62(67%) women, mean(SD) age 39(9.9) years, median(IQR) baseline EDSS 2.5(2)] underwent clinical, neuropsychological assessment and brain 3T‐MRI at baseline and two years after OCR start. CLs were manually segmented using Artificial Intelligence‐Driven Imaging Reconstruction (AIDIR) sequences.

**Results:** At baseline, 22 patients (24%) had no CLs, 31 (34%) had 1 CL, 17 (19%) had 2 CLs, and 22 (24%) had>=3 CLs. Higher CLs number was associated with higher disease duration (r=0.2, p=0.06), lower SDMT (r=‐0.22, p=0.07) and lower total brain volume (r=‐0.32, p=0.002). 17 patients (19%) experienced WMLs accrual and 16 patients (17%) disability progression over follow‐up, but none of them developed new CLs. We observed the formation of only one CL at follow‐up in a female patient (29 years‐old, disease duration 2.9 years). This patient maintained NEDA‐3 status, showed improved physical disability (EDSS 3.5 to 2.5) and no cognitive decline despite baseline SDMT impairment (z score ‐1.4 to ‐0.21).

**Conclusion:** In a cohort of patients treated with OCR only one new CL in a patient without concurrent WMLs accrual was detected. These data support OCR efficacy in minimizing lesion accrual and suggest distinct mechanisms underlying CLs and WMLs development in MS.

**Disclosure:** This study was partially supported by a grant from Roche

## EPO‐100

### Abstract withdrawn

## EPO‐101

### Lesion location and functional connections reveal cognitive impairment networks in multiple sclerosis

#### P. Preziosa ^1^; A. Franceschini^2^; P. Valsasina^2^; D. Mistri^2^; M. Margoni^3^; F. Esposito^4^; M. Rocca^1^; M. Filippi^5^

##### ^1^Neuroimaging Research Unit, Division of Neuroscience, and Neurology Unit, IRCCS San Raffaele Scientific Institute, and Vita‐Salute San Raffaele University, Milan, Italy; ^2^Neuroimaging Research Unit, Division of Neuroscience, IRCCS San Raffaele Scientific Institute, Milan, Italy; ^3^Neuroimaging Research Unit, Division of Neuroscience, Neurology Unit, and Neurorehabilitation Unit, IRCCS San Raffaele Scientific Institute, Milan, Italy; ^4^Neurology Unit, IRCCS San Raffaele Scientific Institute, Milan, Italy; ^5^Neurology Unit, Neurorehabilitation Unit, Neurophysiology Service, and Neuroimaging Research Unit, Division of Neuroscience, IRCCS San Raffaele Scientific Institute, and Vita‐Salute San Raffaele University, Milan, Italy

**Background and aims:** Cognitive impairment, fatigue, and depression are common in multiple sclerosis (MS) and may result from white matter (WM) lesion disruption of regional functional connectivity. We explored whether WM lesions functionally connected to specific brain regions contribute to these MS‐related manifestations.

**Methods:** A total of 596 MS patients underwent 3T brain MRI acquisition, neurologic assessment, and neuropsychological evaluation using the Brief Repeatable Battery, Modified Fatigue Impact Scale (MFIS), and Montgomery‐Asberg Depression Rating Scale (MADRS). Voxel‐wise lesion probability maps were compared between MS patients’ groups based on cognition, fatigue, or depression. WM lesion distributions were linked to a brain functional connectivity atlas to map lesion network associations. Lesion network maps were then compared among MS patients’ subgroups (p<0.05, family‐wise error‐corrected).

**Results:** One hundred twenty‐six (27.2%) MS patients were cognitively‐impaired. These patients had significantly more widespread WM lesions, more functionally connected to bilateral hippocampi, thalami, cerebellum, and temporo‐occipital cortices compared to cognitively preserved patients. Lesion networks were similar for impaired processing speed/attention. Verbal memory deficits were linked to WM lesions connected to hippocampi, parahippocampi, left lingual gyrus, and right cerebellum, while verbal fluency deficits involved connections to thalami and cerebellum. Visual memory deficits corresponded only to widespread WM lesion distribution. No significant lesion distribution or network differences were found for patients with fatigue (MFIS score>38, 184/493 [37.3%]) or depression (MADRS score>9, 192/495 [38.8%]).

**Conclusion:** Regional WM lesions disrupting connections to hippocampus, thalamus, cerebellum, and temporo‐occipital cortices contribute to cognitive impairment. Lesion network map may clarify mechanisms underlying cognitive deficits in MS.

**Disclosure:** PP received speaker honoraria from Roche, Biogen, Novartis, Merck, Bristol Myers Squibb, Genzyme, Horizon and Sanofi. He received research support from Italian Ministry of Health and Fondazione Italiana Sclerosi Multipla (FISM). AF, PV, DM, FE have nothing to disclose. MM reports grants and personal fees from Sanofi Genzyme, Merck Serono, Roche, Biogen, Amgen and Novartis. MAR received consulting fees from Biogen, Bristol Myers Squibb, Eli Lilly, Janssen, Roche, and speaker honoraria from AstraZaneca, Biogen, Bristol Myers Squibb, Bromatech, Celgene, Genzyme, Horizon Therapeutics Italy, Merck Serono SpA, Novartis, Roche, Sanofi and Teva, she receives research support from the MS Society of Canada, the Italian Ministry of Health, the Italian Ministry of University and Research, and FISM. MF received compensation for consulting services or speaking activities from Alexion, Almirall, Bayer, Biogen, Celgene, Chiesi Italia SpA, Eli Lilly, Genzyme, Janssen, Merck‐Serono, Neopharmed Gentili, Novartis, Novo Nordisk, Roche, Sanofi Takeda, and TEVA; Advisory Boards for Alexion, Biogen, Bristol‐Myers Squibb, Merck, Novartis, Roche, Sanofi, Sanofi‐Aventis, Sanofi‐Genzyme, Takeda; scientific direction of educational events for Biogen, Merck, Roche, Celgene, Bristol‐Myers Squibb, Lilly, Novartis, Sanofi‐Genzyme; he receives research support from Biogen Idec, Merck‐Serono, Novartis, Roche, the Italian Ministry of Health, the Italian Ministry of University and Research, and FISM.

## EPO‐102

### Increased health utilisation before an MS diagnosis: Evidence from routine healthcare data in Wales

#### J. Witts^1^; R. Middleton ^1^; R. Nicholas^2^

##### ^1^Swansea University, Disease Registers Group; ^2^Department of Brain Sciences ‐ Faculty of Medicine

**Background and aims:** Using an algorithm to identify people with Multiple Sclerosis (pwMS) in routine healthcare data we determined if there was any evidence of an MS prodrome by looking at healthcare utilisation in Wales in those before the age of 16 (pre‐16) and before the diagnosis of MS (pre‐Dx) was made.

**Methods:** Using the Secure Anonymised Information Linkage (SAIL) Databank of 4.6 Million people in Wales, we identified inpatient admissions (top 10), GP attendances (top 10) and prescriptions (top 20) in pwMS and separate propensity matched controls (by gender and year of birth) pre‐16 and also pre‐Dx. We identified entries unique to MS, excluding MS/demyelinating codes, and assessed whether entries common to both groups were significantly different

**Results:** Pre‐16 (N=313), admissions unique to MS included constipation (4.8%) and dental caries (3.5%); there was no significant difference in the 6/10 ICD‐10 codes shared between pwMS/control cohorts. Pre‐Dx (N=5,309), sensory symptoms (4.3%), paraesthesia (3.8%), headaches (3.7%) and urinary tract infections (3.6%) were unique to pwMS with the remainder 4/10 not being different between pwMS/control cohorts. For GP attendances (pwMS N=4798 pre‐16, N=9648 pre‐Dx), there were higher rates of attendances but no unique causes for attendance. In pwMS versus controls pre‐16, they had more respiratory infections (pwMS 14.2%, p<0.001), consistent with this pre‐16 they had higher rates of penicillin use. Pre‐Dx they had more vaccinations and used more antibiotics, paracetamol, anti‐inflammatories, hydrocortisone and PPI inhibitors.

**Conclusion:** pwMS have higher healthcare utilisation pre‐16 and pre‐Dx. This requires further study but does imply a MS prodrome.

**Disclosure:** None of the authors have anything to disclose related to this abstract.

## EPO‐103

### Relationship between neutrophil to lymphocyte ratio and neurofilament light‐chain levels in patients with MS

#### S. Iacono ^1^; G. Schirò^1^; M. Andolina^1^; G. Sorbello^1^; A. Calì^1^; L. Agnello^2^; M. Ciaccio^2^; G. Salemi^1^; P. Ragonese^1^

##### ^1^Department of Biomedicine, Neuroscience and Advanced Diagnostics, University of Palermo, Italy; ^2^Department of Biomedicine, Neurosciences and Advanced Diagnostics, Institute of Clinical Biochemistry, Clinical Molecular Medicine and Clinical Laboratory Medicine, University Hospital Policlinico, Palermo, Italy

**Background and aims:** The pathogenesis of Multiple Sclerosis (MS) is unclear wherein the balance between peripheral and intrathecal immune systems may affect both relapsing activity and disease progression. Neutrophilic to lymphocyte ratio (NLR) has been proposed as possible surrogate biomarker for neuroinflammation. On the other hand, the prognostic value of cerebrospinal fluid (CSF) neurofilament light‐chain levels gained increasing interest as marker of neurodegeneration. Aims of this study are to evaluate the relationship between NLR, cerebrospinal NfL and relapse activity in patients with MS.

**Methods:** individuals who underwent diagnostic work‐up through lumbar puncture (LP) and with a definite diagnosis of MS were included. We obtained NLR and CSF‐NfL for each patient. The Spearman's coefficient was employed to estimate the relationship between NLR and CSF‐NfL. Finally, NLR and CSF‐NfL were compared between pwMS with or without at least one relapse within 3 months preceding LP by using Mann and Whitney test.

**Results:** a total of 40 pwMS (age: 40.6 ± 13.6 years; female n=27, 67.5%; mean EDSS 2.4 ± 2) were included. Seventeen pwMS (42.5%) had at least one relapse before LP. A significant direct correlation was found between NLR and CSF‐NfL (r=0.35; p=0.028; Fig. 1). CSF‐NfL were higher in pwMS with a relapse before LP (p=0.024; Fig. 2) while NLR was similar between groups (p=0.57; Fig. 3).

**Figure:**
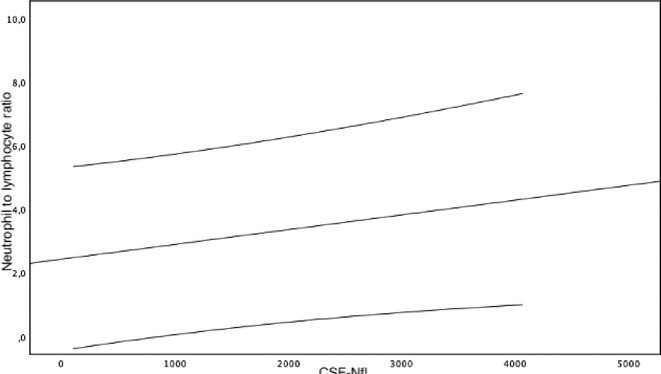
**FIGURE 1** Scatterplot showing the linear correlation between NLR and CSF‐NfL.

**Figure:**
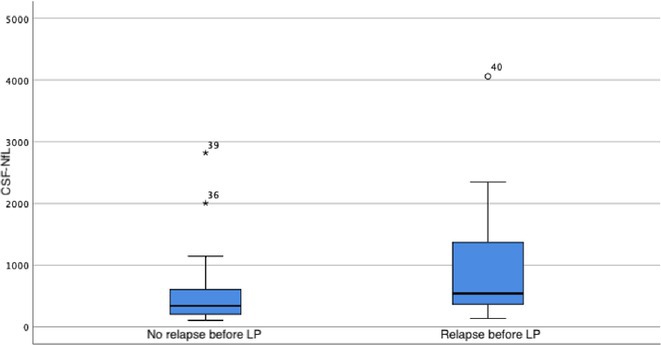
**FIGURE 2** Box‐plots showing the comparison of CSF‐NfL levels in patients with or without MS relapses before lumbar puncture.

**Figure:**
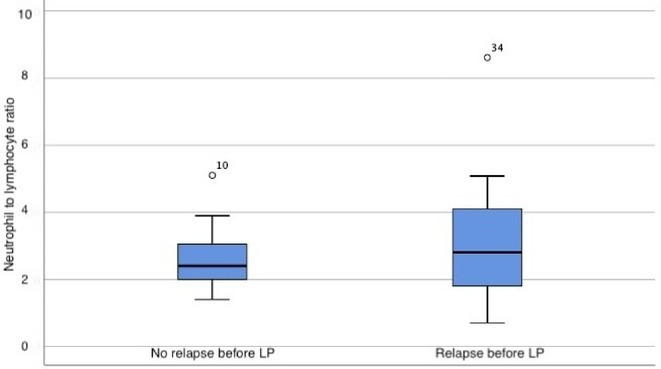
**FIGURE 3** Box‐plots showing the comparison of NLR in patients with or without MS relapses before lumbar puncture

**Conclusion:** Despite it did not change with relapse activity in pwMS, our results suggest a possible role of NLR as a simple and inexpensive surrogate biomarker of axonal damage.

**Disclosure:** Nothing to disclose.

## EPO‐104

### Inter‐eye difference of ganglion cell layer alone in identifying optic neuritis in multiple sclerosis

#### N. Krajnc ^1^; F. Föttinger^1^; M. Ponleitner^1^; B. Kornek^1^; F. Leutmezer^1^; S. Macher^1^; P. Rommer^1^; C. Schmied^1^; K. Zebenholzer^1^; G. Zulehner^1^; T. Zrzavy^1^; T. Berger^1^; B. Pemp^2^; G. Bsteh^1^

##### ^1^Department of Neurology, Medical University of Vienna, Vienna, Austria; ^2^Department of Ophthalmology, Medical University of Vienna, Vienna, Austria

**Background and aims:** The 2024 McDonald criteria for diagnosing multiple sclerosis (MS) include optic nerve involvement as a fifth region for establishing dissemination in space. Optic neuritis (ON) can be detected through optical coherence tomography (OCT) using an inter‐eye absolute or percentage difference (IEAD, IEPD) in ganglion cell‐inner plexiform layer (GCIPL) thickness.

**Methods:** This cross‐sectional retrospective study included people with MS (pwMS) who underwent an OCT scan. Diagnostic accuracy was assessed using ROC analysis.

**Results:** A total of 241 pwMS (mean age 34.7 years (SD 9.7), 70.1% female) were included. GCL IEAD (AUC 0.88, cut‐off ≥0.04mm3 or ≥1.4μm, 80.0% sensitivity, 86.5% specificity) and IEPD (AUC 0.89, cut‐off ≥4%, 79.0% sensitivity, 87.2% specificity) demonstrated excellent diagnostic accuracy for unilateral ON, showing non‐inferiority to the established GCIPL IEAD/IEPD. An improvement in diagnostic performance of both models was observed in a subanalysis of pwMS with subclinical ON (AUC 0.95, sensitivity 93.8%, specificity 87.2%).

**Conclusion:** GCL IEAD and IEPD provide strong diagnostic accuracy for identifying unilateral ON and can be effectively used as an alternative to GCIPL IEAD/IEPD to facilitate implementation in clinical routine.

**Disclosure:** All authors declare no conflict of interest relevant to this study.

## Muscle and Neuromuscular Junction Disorder 1

## EPO‐105

### Increased prevalence of extrathymic neoplasm in myasthenia gravis patients a population‐based matched case‐control study

#### A. Wilf‐Yarkoni; T. Kab; M. Hellmann; I. Lotan; T. Shohat; K. Pardo

##### Rabin Medical Center, Tel‐Aviv, Israel

**Background and aims:** Myasthenia gravis (MG) is known to be associated with thymic neoplasms. However, an increased prevalence of extrathymic neoplasms has also been reported. This study aims to evaluate the rates of malignancy in MG patients, while also accounting for risk factors such as co‐morbidities and immunomodulatory treatments.

**Methods:** We conducted a case–control study using Clalit health services (CHS) database, applying innovative machine learning (ML) algorithm developed by our group to avoid diagnosis misclassification. We included MG patients aged 18 and older, along with a sex‐ and age‐matched control group in a 1:3 ratio. We compared the prevalence and hazard ratios of extrathymic neoplasms between the groups.

**Results:** 1,558 patients with a high probability of MG according to our ML model, were included in our cohort, alongside a control group of 4,674 non‐myasthenic individuals. MG patients had higher prevalence of malignancy prior to the MG diagnosis with OR of 1.85 (P < 0.001), and higher incidence of malignancy after the MG diagnosis with HR of 1.64 (P < 0.001). Mean time between MG diagnosis and malignancy was 3.89 years (IQR, 1.19‐8.83). The most prevalent extrathymic neoplasms after MG diagnosis were, lung, digestive, Skin cancer, and hematologic cancers. Age and male sex were associated with increased risk for developing cancer, while the present of tymoma and treatments were not.

**Conclusion:** Male and older MG patients have a higher prevalence of solid and hematologic neoplasms compared to non‐myasthenic controls. Our ML model provided an accurate and reliable assessment of the MG population.

**Disclosure:** Nothing to disclose.

## EPO‐106

### Rituximab in the treatment of acetylcholine receptor antibody‐positive – Myasthenia Gravis: A single center study

#### D. Tzavella; E. Strataki; C. Chrysovitsanou; V. Zouvelou

##### 1st Neurology Department, National and Kapodistrian University of Athens, Greece, Eginition Hospital, ERN EURO‐NMD

**Background and aims:** Rituximab, a monoclonal antibody targeting CD20 B cells, has a well‐established role in myasthenia gravis (MG) with muscle‐specific kinase receptor (MuSK) antibodies. However, its efficacy and optimal timing in acetylcholine receptor antibody‐positive (AChR‐Ab+) MG remain contentious.

**Methods:** This single‐center, observational study evaluated 26 AChR‐Ab+ MG patients who received rituximab as the sole non‐steroidal immunosuppressant for at least one therapeutic cycle and were observed for a minimum of 3 months.

**Results:** Patients aged 25–85 years (mean age at first infusion: 61.6). Among the cohort 12 (46%) had very late‐onset MG (≥65 years), 16 (64%) were newly diagnosed (<2 years from diagnosis), 4 (15%) had thymoma‐associated MG, and 6 (23%) had refractory MG. Dosing regimens included four weekly doses of 500 mg (16 patients, 62%), two doses of 500 mg two weeks apart (6 patients, 23%), and a single 500 mg dose (4 patients, 15%). At six months, 11 patients (42%) achieved pharmacologic remission, including those with refractory ocular and dysphagia symptoms. Additionally, 3 (11.5%) showed minimal manifestations, 8 (31%) demonstrated clinical improvement, and 1 (4%) remained unchanged. AChR‐Ab titers decreased by 35% (data for 10 patients). Corticosteroid intake was reduced by 36%, and none required rescue therapy. At twelve months, 15 (58%) maintained their clinical status. No data available for the rest.

**Conclusion:** Rituximab demonstrated a favorable safety profile with no adverse events, even on very late‐onset MG. It achieved sustained pharmacologic remission and a significant steroid‐sparing effect, benefiting refractory MG patients and those with newly diagnosed or chronic subtypes.

**Disclosure:** Nothing to disclose.

## EPO‐107

### Higher risk of fractures in myasthenia gravis patients versus general population–national healthcare database study

#### E. Sobieszczuk ^1^; P. Szczudlik^1^; B. Koń^2^; A. Pawlewicz^2^; A. Kostera‐Pruszczyk^1^

##### ^1^Department of Neurology, Medical University of Warsaw, Warsaw, Poland, ERN EURO‐NMD; ^2^Department of Analysis, Quality Monitoring and Optimisation of Health Services, National Health Fund, Warsaw, Poland

**Background and aims:** Methods NFZ is a mandatory health insurance in Poland. MG is the only indication for reimbursement of pyridostygmine bromide and ambenonium chloride. Aim: to determine incidence of fractures in myasthenia gravis as compared with control population (ConP).

**Methods:** MG patients receiving reimbursed pyridostigmine or ambenonium dispensed between 1.01.2013‐31.12.2023 were identified. A control group matched for age and gender was randomly assigned. All fractures diagnosed in 2023 by ICD‐10 codes were analyzed by localization, compared between MG and ConP.

**Results:** There were 10,300 MG patients in 2023; F (61%), mean age 62 years, median age 66 years. Patients with MG had 158,8% higher chance for osteoporotic fracture (p<0.001, R2=0.104). Independent risk factors for osteoporotic fractures were: age, female sex, MG, adrenal insufficiency, diabetes, cataract, using steroids >180 days. MG patients had 32% higher chance for other than osteoporotic fracture (p<0.01, R2 = 0.025) with independent risk factors: age, female sex, MG, adrenal insufficiency, cataract. MG patients had 127.9% higher chance for spine fractures (p<0.001, R2=0.043). MG patients treated with glucocorticosteroids (GCS) >180 days had 149.9% higher risk of having spine fractures than MG patients not treated with GCS (p<0.001, R2=0.05), with MG, age and female gender being an independent risk factors. MG patients treated with GCS >180 days had in general 58.6% higher risk of having any nonosteoporotic fracture than MG patients not treated with GCS (p<0.01, R2=0.017), MG, age and female gender were an independent risk factors.

**Conclusion:** MG patients have higher risk of fractures in comparison with general population.

**Disclosure:** Nothing to disclose.

## EPO‐108

### Burden of remaining symptoms and fluctuations of generalized myasthenia gravis on patients' daily lives – BEYOND study

#### F. Saccà ^1^; S. Lehnerer^2^; M. Bonaria Uccheddu^3^; D. Sufragiu^4^; C. Campbell^5^; D. Bridge^6^; S. Pope^6^; C. Chassat^6^; G. Rodrigues^7^; M. Mangelaars^8^; W. Noel^9^; C. Gary^10^

##### ^1^Department of Neurosciences and Reproductive and Odontostomatological Sciences, University of Naples Federico II, Naples, Italy; ^2^Charité – Universitätsmedizin Berlin, corporate member of Freie Universität Berlin and Humboldt‐Universität zu Berlin, Department of Neurology with Experimental Neurology, Charitéplatz 1, 10117 Berlin, Germany; ^3^European Myasthenia Gravis Association (EuMGA), La Louviere, Belgium; Associazione Italiana Miastenia (AIM), Milan, Italy; ^4^Associazione Italiana Miastenia (AIM), Milan, Italy; European Myasthenia Gravis Association (EuMGA), La Louviere, Belgium; ^5^Myaware, Derby, UK; ^6^Adelphi Values PROVE, Bollington, UK; ^7^Johnson & Johnson Innovative Medicine, Madrid, Spain; ^8^Johnson & Johnson Innovative Medicine, Tilburg, the Netherlands; ^9^Johnson & Johnson Innovative Medicine, Brussels, Belgium, ^10^Johnson & Johnson Innovative Medicine, Issy‐Les‐Moulineaux, France

**Background and aims:** Generalised myasthenia gravis (gMG) is a rare autoimmune disorder affecting the neuromuscular junction with varying clinical manifestations that disrupt patients’ daily lives; an estimated 30% of patients with gMG experience remaining symptoms despite treatment. The study objective is to describe how these remaining symptoms and their fluctuations may affect a person's daily activities while living with gMG.

**Methods:** A Delphi panel was conducted with adult patients with gMG (n=18 from France, Germany, Italy, the UK and Sweden), aiming to reach consensus regarding the burden of symptoms across key domains, following completion of two questionnaire rounds.

**Results:** Interim results from the first Delphi panel round (n=18) are summarised here. Respondents highlighted the impact of remaining symptoms in five key domains: daily activities, social life, mental and personal well‐being, work and education, and future plans. Participants noted still requiring adaptations for personal hygiene (e.g. shower stool) and support with completing household chores (e.g., cooking, cleaning). Fluctuations, described as the variation in the intensity and predictability of remaining symptoms, significantly affected patients' daily lives, with participants highlighting the need to adapt their daily routines and future plans.

**Conclusion:** This study is the first to investigate the specific impact of remaining symptoms of gMG on patients’ daily lives and how fluctuations may further aggravate this impact. These interim results demonstrate the burden of remaining symptoms and their fluctuations for patients; minimising these would likely improve patients’ daily lives. Full study results will be presented at the 2025 EAN congress.

**Disclosure:** This study was sponsored by J&J Innovative Medicine. FS: public speaking honoraria from Alexion, argenx, Biogen, Genpharm, Medpharma, Madison Pharma, Neopharm Israel, Sanofi, Zai Lab; compensation for Advisory boards or consultation fees from Alexion, Amgen, argenx, AstraZeneca, Alexis, Biogen, Dianthus, J&J, Lexeo, Novartis, Reata, Roche, Sandoz, Sanofi, Takeda, UCB, Zai Lab; PI in clinical trials for Alexion, argenx, Dianthus, Immunovant, Lediant, Novartis, Prilenia, Remgen, Sanofi. SL: speaker or consultancy honoraria or financial research support (paid to her institution) from Alexion, argenx, Biogen, Hormosan, HUMA, J&J, Merck, UCB and Roche. MBU: honoraria for consulting services from Alexion, J&J, Merck, UCB Pharma and UCB S.A.; speaking fees from Alexion, UCB Pharma and UCB S.A.; consulting services to Argenx. DS: honoraria for consulting services from Novartis Pharma AG and J&J. CC: Employee of Myaware which received speaker honoraria for her services from UCB, Argenx and J&J. GR, MM, WN, CG: Employees of J&J; may hold stock or stock options in J&J. DB, SP, CC: Employees of Adelphi Values PROVE, which received funding from J&J for the conduct of the study and for abstract development.

## EPO‐109

### Oral cladribine capsules for generalised myasthenia gravis: design of the actively‐recruiting phase 3 myclad study

#### H. Kaminski^1^; J. Howard Jr^2^; G. Cutter^3^; K. O’Connor^4^; M. Dimachkie^5^; J. Palace ^6^; A. Meisel^7^; R. Mantegazza^8^; A. Nolting^9^; S. Gopalakrishnan^9^; C. Le Bolay^10^; A. Javor^11^; N. Alexandri^9^; D. Jack^12^; K. Rejdak^13^

##### ^1^Department of Neurology & Rehabilitation Medicine, School of Medicine & Health Sciences, George Washington University, Washington DC, USA; ^2^Department of Neurology, University of North Carolina at Chapel Hill, Chapel Hill, North Carolina, USA; ^3^School of Public Health – Biostatistics, University of Alabama at Birmingham, Birmingham, Alabama, USA; ^4^Yale School of Medicine, Yale University, New Haven, Connecticut, USA; ^5^Department of Neurology, University of Kansas Medical Center, Kansas City, Kansas, USA; ^6^Nuffield Department of Clinical Neurosciences, University of Oxford, Oxford, UK; ^7^Department of Neurology with Experimental Neurology, Charité – Universitätsmedizin Berlin, Berlin, Germany; ^8^Neuroimmunology and Neuromuscular Diseases, IRCCS Carlo Besta, Milan, Italy; ^9^Merck Healthcare KGaA, Darmstadt, Germany, ^10^Merck Santé S.A.S., Lyon, France, an affiliate of Merck KGaA, Darmstadt, Germany, ^11^Ares Trading SA, Eysins, Switzerland, an affiliate of Merck KGaA, Darmstadt, Germany, ^12^Merck Serono Ltd., Middlesex, UK, an affiliate of Merck KGaA, Darmstadt, Germany, ^13^Department of Neurology, Medical University of Lublin, Lublin, Poland

**Background and aims:** Myasthenia Gravis (MG) is an autoantibody‐mediated disorder of neuromuscular junction transmission, characterised by fluctuating muscle weakness. Cladribine is an immune reconstitution therapy (IRT) that selectively targets B and T cells and has shown preliminary efficacy in a pilot study of participants with generalised MG (gMG). We present the design of MyClad, a Phase 3 trial evaluating efficacy and safety of oral cladribine capsules (CladC) versus placebo in gMG.

**Methods:** MyClad is a 3‐year Phase 3 trial aiming to recruit 240 participants with gMG Class II–IVa, irrespective of autoantibody serotype. In the first double‐blind placebo‐controlled period (24 weeks), participants will be randomised 1:1:1 to two short courses of placebo or low‐ or high‐dose CladC (Figure). In the following blinded extension period (24 weeks), placebo recipients will be re‐randomized to low‐ or high‐dose CladC. During this period and a third double‐blind follow‐up period of 96 weeks, participants may be retreated with CladC if clinically needed. The primary endpoint is change from baseline (CFB) to Week 24 in MG‐Activities of Daily Living (MG‐ADL) score for each CladC dose versus placebo. Secondary endpoints include CFB to Week 24 in Quantitative MG (QMG), MG Composite and MG 15‐Item Quality of Life Scale revised scores, proportions of MG‐ADL and QMG responders, time from CladC full dose to retreatment or rescue treatment, safety and CladC pharmacokinetics.

**Figure:**
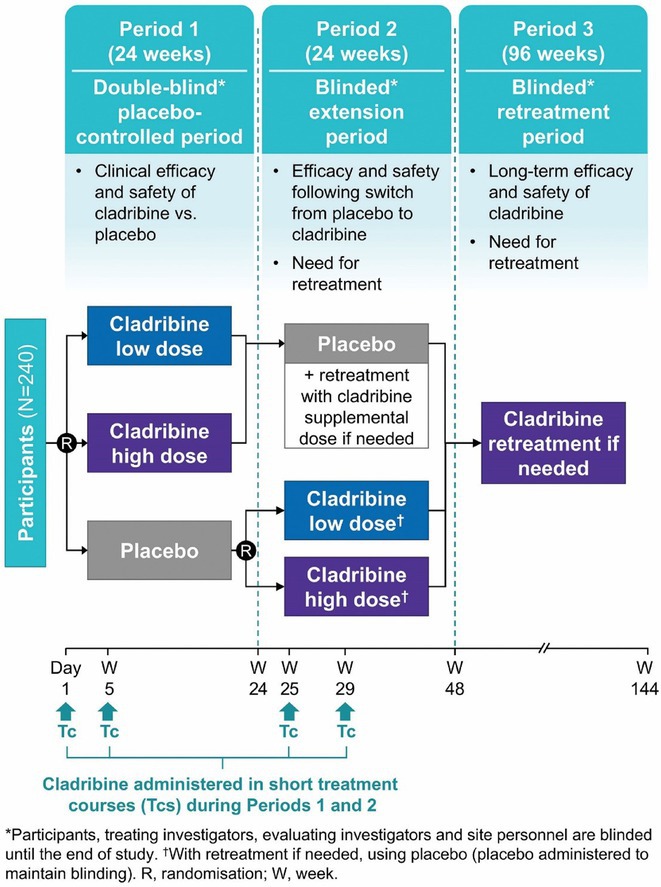
**FIGURE 1** MyClad study design.

**Results:** Some participants have been recruited; enrolment is ongoing.

**Conclusion:** MyClad aims to establish efficacy, duration of effect and safety of IRT to target B and T cell‐mediated autoimmunity with CladC in gMG.

**Disclosure:** The MyClad study (Clinicaltrials.gov: NCT06463587) is sponsored by Merck Healthcare KGaA, Darmstadt, Germany, an affiliate of Merck KGaA, Darmstadt, Germany. AN, SG, CLB, AJ, NA and DJ are employees of Merck KGaA or its affiliates. Other authors or their institutions have multiple financial and/or non‐financial relationships with research organisations or pharmaceutical companies; further information will be included in the presentation.

## EPO‐110

### Myasthenia gravis medication related to pregnancy, a registry‐based cohort study

#### J. Lindroos ^1^; N. Gilhus^1^; M. Bjørk^1^; J. Hoff^2^; C. Cesta^3^; K. Furu^4^; J. Cohen^4^

##### ^1^Department of Neurology, Haukeland University Hospital, Bergen, Norway; ^2^Faculty of Health Studies at VID Specialized University, Bergen, Norway; ^3^Centre for Pharmacoepidemiology, Karolinska Institutet, Stockholm, Sweden; ^4^Department of Chronic Diseases and Centre for Fertility and Health, Norwegian Institute of Public Health, Oslo, Norway

**Background and aims:** The unpredictable disease course of myasthenia gravis (MG) during pregnancy influences medication needs. Both medications and MG‐autoantibodies transferred from mother to child could potentially harm the foetus. Medication‐use patterns in relation to pregnancy are unknown in MG but should be mapped to disentangle the effect of maternal disease from in‐utero medication‐exposure. Our aim was to determine medication use related to MG‐pregnancies, and to distinguish periods with stable and unstable disease.

**Methods:** We included all MG‐pregnancies resulting in birth in Norway 2010‐2020 and Sweden 2008–2019 using nationwide health‐ and population registries. MG‐pregnancies were identified by a maternal MG‐diagnosis from specialist care, or multiple pyridostigmine‐purchases. Medication‐use was assessed through filled prescription records from one year before pregnancy to six months postpartum. Next, MG‐pregnancies will be grouped based on similar medication‐use patterns by group‐based trajectory modelling, a machine‐learning method, and described through clinical parameters, such as hospitalizations.

**Results:** We identified 321 MG‐pregnancies of 225 women from a background population of 1,962,396 pregnancies. In preliminary analyses, 37% used pyridostigmine before pregnancy, 34% during, and 29% after pregnancy. Prednisolone and azathioprine were used in 10% and 7% of pregnancies, respectively. No pregnancies were exposed to methotrexate, mycophenolate mofetil, or cyclophosphamide.

**Conclusion:** Most parturients with MG had not used any MG‐medications before, during, or after pregnancy, indicating mild and stable MG. In the next step, to be presented at the congress, we will describe medication‐use trajectories in MG‐pregnancies. Assuming that add‐on therapy in pregnancy is a sign of disease‐worsening, these trajectories will describe disease severity in pregnancy.

**Disclosure:** JLVL has received financial support from UCB. NEG has received financial support from Grifols, UCB, Argenx, Janssen, Johnson&Johnson, Merck, Roche, Alexion, Immunovant, Huma, Denka, Amgen, and Dianthus. MHB has received speaker honoraria and/or consultancy honoraria from Teva, Eisai, AbbVie, Pfizer, Novartis, Lundbeck, Angelini Pharma, Jazz pharmaceuticals, and Lilly during the last five years, none in relation to the topic in the abstract. JMC declare no conflicts of interest. CEC, KF and JMC reports participation in research projects funded by pharmaceutical companies, all with funds paid to their institution (no personal fees) and with no relation to the work reported in this paper.

## EPO‐111

### Fatigue assessed by neuro‐QoL in phase 3 vivacity‐MG3 trial of nipocalimab vs placebo in generalized myasthenia gravis

#### J. Vissing ^1^; K. Gandhi^2^; S. Pease^2^; N. Imran^2^; M. Ait‐Tihyaty^2^; I. Turkoz^3^; G. Coteur^4^; C. Gary^5^; Z. Choudhry^3^; S. Ramchandran^3^

##### ^1^Department of Neurology, University of Copenhagen, Copenhagen, Denmark; ^2^Johnson & Johnson, Raritan, USA; ^3^Johnson & Johnson, Horsham, USA; ^4^Johnson & Johnson, Raritan, USA; IPATH Solutions, Wemmel, Belgium; ^5^Johnson & Johnson, Issy‐les‐Moulineaux, France

**Background and aims:** Generalized myasthenia gravis (gMG) is an autoantibody‐mediated disease, with muscle weakness, considerable fatigue and associated impacts. Fatigue often correlates with gMG disease severity(1) emphasising need to manage both effectively. In Vivacity‐MG3 (NCT04951622), nipocalimab+standard‐of‐care (SOC) demonstrated improved and sustained efficacy vs placebo+SOC. We evaluated changes in Neuro‐QoL‐Fatigue, patient‐reported assessment of fatigue and its associated impact, and disease‐severity measures between Vivacity‐MG3 arms.

**Figure:**
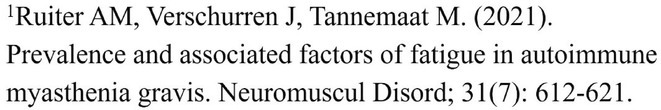
Reference

**Methods:** Mean changes‐from‐baseline (CFB) in Neuro‐QoL‐Fatigue total score over 24 weeks(W) were compared using mixed‐model‐repeated‐measures. Proportion of patients achieving meaningful‐within‐person‐improvement (MWPI) of 6.7‐points from baseline at 24W were examined using Chi‐square test statistics. Logistic regression models evaluated likelihood of sustaining MWPI for >=8,12,16, or 20W. Mean CFB at 24W was evaluated by stratifying patients based on baseline disease‐severity scores observed above median on Myasthenia‐Gravis‐Activities‐of‐Daily‐Living (MG‐ADL) and Quantitative‐Myasthenia‐Gravis (QMG) scales (severe disease defined as MG‐ADL >9 and QMG >15).

**Results:** LS‐mean (95% CI) difference in CFB on Neuro‐QoL‐Fatigue was greater (p=0.001) with nipocalimab+SOC (‐7.4[‐11.94, ‐2.93]) by 4W and numerically higher at 24W (‐4.3[‐9.16, 0.62]) vs placebo+SOC. At 24W, 6.2% more patients on nipocalimab+SOC (42/67) achieved MWPI than placebo+SOC (35/62) (p>0.05). Nipocalimab+SOC‐treated patients were approximately twice more likely to sustain MWPI for >=8,12,16, 20W (p<0.05). Among those with severe disease at baseline, mean improvement was numerically greater at 24W with nipocalimab+SOC vs placebo+SOC (difference=‐9.0; 95% CI=‐22.0, 4.1).

**Conclusion:** Nipocalimab+SOC‐treated patients showed improvement on Neuro‐QoL‐Fatigue as‐early‐as W4. Nipocalimab+SOC‐treated patients were also significantly more likely to sustain MWPI over time. Patients with more severe disease at baseline showed numerically greater improvements with nipocalimab+SOC than placebo+SOC.

**Disclosure:** This study was sponsored by Johnson & Johnson. John Vissing: Received consultant fees for serving on advisory boards for Alexion Pharmaceuticals Inc., Argenx BV, Dianthus Therapeutics, Horizon Therapeutics (now Amgen Inc.), Janssen, Regeneron, Roche, and UCB Pharma SA. Kavita Gandhi, Sheryl Pease, Nida Imran, Maria Ait‐Tihyaty, Ibrahim Turkoz, Charlotte Gary, Zia Chaudhry, and Sindhu Ramchandren: Employees of Johnson & Johnson, may hold stocks/stock options in Johnson & Johnson. Geoffroy Coteur: Owner of IPATH Solutions and received consultant fees from Johnson & Johnson.

## EPO‐112

### Herding‐like behaviour in myasthenia gravis treatment decisions: exploring underlying mechanisms

#### R. Villaverde^1^; R. Gómez‐Ballesteros^2^; V. Reyes^3^; T. Armangué^4^; L. Querol^5^; G. Gutiérrez‐Gutiérrez^6^; J. Sotoca^7^; A. Ares^8^; E. Salas^2^; P. Díaz‐Abós^2^; A. Squaglia^2^; J. Maurino ^2^; E. Cortés‐Vicente^5^

##### ^1^Department of Neurology, Hospital Universitario Morales Meseguer, Murcia, Spain; ^2^Medical Department, Roche Farma, Madrid, Spain; ^3^Department of Neurology, Hospital Regional Universitario de Málaga, Málaga, Spain; ^4^Department of Pediatric Neuroimmunology, Hospital Sant Joan de Déu, Barcelona, Spain; ^5^Department of Neurology, Hospital de la Santa Creu i Sant Pau, Barcelona, Spain; ^6^Department of Neurology, Hospital Universitario Infanta Sofía, Madrid, Spain; ^7^Department of Neurology, Hospital Universitario Vall d’Hebron, Barcelona, Spain; ^8^Department of Neurology, Complejo Asistencial Universitario León, León, Spain

**Background and aims:** Herding‐like behaviour occurs when physicians follow colleagues’ recommendations instead of making independent decisions. It can lead to suboptimal outcomes in dynamic contexts, such as adopting new treatments for generalized myasthenia gravis (gMG). This study aimed to assess herding‐like behavior and associated factors among neurologists managing gMG.

**Methods:** An electronic survey study was conducted with the Spanish Society of Neurology. Neurologists provided demographic, professional, and behavioural characteristics/traits. Herding‐like behaviour was assessed using a simulated case scenario: 42‐year‐old woman with gMG stable for 3 years on pyridostigmine and azathioprine. Three months earlier, she experienced transient lower‐extremity weakness, resolved spontaneously within 2‐3 weeks. Her neurological examination remained unchanged (MG‐ADL=0), with normal blood tests and no new medications. Seeking a second opinion, she was advised by a neuromuscular specialist to switch to ravulizumab. Agreeing with this recommendation, contrary to established guidelines, was classified as herding. Relationships between herding‐like behaviour and neurologists’ characteristics were assessed using Chi‐square and Mann‐Whitney‐U tests.

**Results:** 149 neurologists participated (mean age [SD]: 39.0±9.4 years, 54.4% male, median experience managing gMG [IQR]: 7 [3‐15] years; 32.2% fully dedicated to gMG; median gMG patients attended/month: 10 [5‐20]). Herding‐like behaviour was present in 38.9% (n=58/149). Neurologists with herding were older, not specialized in gMG, and more experienced (all p<0.01). Those without herding worked at reference hospitals, had specific gMG consultations, treated more patients, participated in clinical trials, and attended neuromuscular congresses (p<0.05).

**Conclusion:** Herding‐like behaviour was observed in over one‐third of neurologists. Addressing its impact and promoting specific interventions may enhance clinical decision‐making and patient care.

**Disclosure:** GGG has received compensation for consulting services from CSL Behring, Biogen, Alter, Takeda, Akcea, Lupin Neuroscience, Roche, Alexion, and Argenx; congresses support from Alter, Esteve, Sanofi‐Genzyme, Pfizer, and UCB Pharma; has scientific relation with Lilly, Alexion, Genzyme, Takeda, Biogen, Pfizer, and Alter; books with Exeltis, Alter, Esteve, Andrómaco, and Bristol‐Myers; and has received grants and awards from Lilly, UCB Pharma, and CSL Behring. RGB, ES, PDA, and JM are employees of Roche Pharma Spain. JS has received travel/congress support and compensation for consulting services from Roche, Biogen, UCB, and Argenx. AA has received speaking honoraria, consultation services compensation, or travel support for congress and scientific meetings attendance from Almirall, Bayer, Biogen, BMS, Janssen, Merck, Novartis, Roche, Sanofi, and Teva. LQ received speaker honoraria from Merck, Sanofi, Roche, Biogen, Grifols and CSL Behring; provided expert testimony for Grifols, Johnson & Johnson, Annexon Pharmaceuticals, Sanofi, Novartis, Takeda, and CSL‐Behring; and received research funds from Roche, UCB, and Grifols. ECV has received speaking and advisory boards honoraria from UCB Pharma, Alexion, Argenx, and J&J. The rest of the authors declare no conflict of interest for this work.

## EPO‐113

### Psychometric evaluation of resistance to change questionnaire: reluctance to adopt new treatments in myasthenia gravis

#### V. Reyes^1^; J. Maurino ^2^; T. Armangué^3^; L. Querol^4^; G. Gutiérrez‐Gutiérrez^5^; J. Sotoca^6^; A. Ares^7^; R. Villaverde^8^; E. Salas^2^; P. Díaz‐Abós^2^; E. Cortés‐Vicente^4^; R. Gómez‐Ballesteros^2^; J. Ballesteros^9^

##### ^1^Department of Neurology, Hospital Regional Universitario de Málaga, Málaga, Spain; ^2^Medical Department, Roche Farma, Madrid, Spain; ^3^Department of Pediatric Neuroimmunology, Hospital Sant Joan de Déu, Barcelona, Spain; ^4^Department of Neurology, Hospital de la Santa Creu i Sant Pau, Barcelona, Spain; ^5^Department of Neurology, Hospital Universitario Infanta Sofía, Madrid, Spain; ^6^Department of Neurology, Hospital Universitario Vall d’Hebron, Barcelona, Spain; ^7^Department of Neurology, Complejo Asistencial Universitario León, León, Spain; ^8^Department of Neurology, Hospital Universitario Morales Meseguer, Murcia, Spain; ^9^Department of Neurosciences and CIBERSAM, University of Basque Country (UPV/EHU), Leioa, Spain

**Background and aims:** Resistance to change is a well‐recognized phenomenon in healthcare, impacting innovative treatments’ adoption. Limited research has examined the validity of tools designed to assess this behaviour in the evolving therapeutic landscape of generalized myasthenia gravis (gMG). This study aimed to evaluate the dimensionality and item characteristics of the Resistance to Change questionnaire among neurologists treating gMG patients.

**Methods:** An electronic survey study was conducted with the Spanish Society of Neurology. Resistance to adopting gMG‐selective therapies was assessed using a 34‐item self‐administered questionnaire evaluating resistance trait, openness to change, perceived usefulness, ease of use, value, peer influence, self‐efficacy, and organizational support. A non‐parametric item response theory approach (Mokken analysis) was used to examine the questionnaire's dimensional structure, with scalability coefficients and reliability assessed via Cronbach's alpha.

**Results:** A total of 149 neurologists were included (mean age [SD]: 39.0±9.4 years, 54.4% male, median experience managing gMG [IQR]: 7 [3‐15] years). The questionnaire showed good internal reliability for the overall scale (0.88; 95% CI: 0.84 to 0.90) and its dimensions (range: 0.84 to 0.94). The Mokken analysis suggested two dimensions. First dimension included nine initial items (resistance trait and openness to change) and showed a scalability of 0.67 (strong scale). The second dimension comprised the rest of items and showed a scalability of 0.39 (weak scale).

**Conclusion:** The Resistance to Change questionnaire showed good internal reliability and a dimensional structure with a strong component related to resistance new gMG treatments introduction among neurologists. This tool may help identify and assess barriers to adopting novel medical interventions.

**Disclosure:** GGG has received compensation for consulting services from CSL Behring, Biogen, Alter, Takeda, Akcea, Lupin Neuroscience, Roche, Alexion, and Argenx; congresses support from Alter, Esteve, Sanofi‐Genzyme, Pfizer, and UCB Pharma; has scientific relation with Lilly, Alexion, Genzyme, Takeda, Biogen, Pfizer, and Alter; books with Exeltis, Alter, Esteve, Andrómaco, and Bristol‐Myers; and has received grants and awards from Lilly, UCB Pharma, and CSL Behring. RGB, ES, PDA, and JM are employees of Roche Pharma Spain. JS has received travel/congress support and compensation for consulting services from Roche, Biogen, UCB, and Argenx. AA has received speaking honoraria, consultation services compensation, or travel support for congress and scientific meetings attendance from Almirall, Bayer, Biogen, BMS, Janssen, Merck, Novartis, Roche, Sanofi, and Teva. LQ received speaker honoraria from Merck, Sanofi, Roche, Biogen, Grifols and CSL Behring; provided expert testimony for Grifols, Johnson & Johnson, Annexon Pharmaceuticals, Sanofi, Novartis, Takeda, and CSL‐Behring; and received research funds from Roche, UCB, and Grifols. ECV has received speaking and advisory boards honoraria from UCB Pharma, Alexion, Argenx, and J&J. JB has collaborated in this study through a research contract between the UPV/EHU and Roche Farma Spain. The rest of the authors declare no conflict of interest for this work.

## EPO‐114

### Extended therapeutic experience with efgartigimod in myasthenia gravis: A multicenter study

#### L. Fuchs ^1^; I. Vigiser^2^; H. Kolb^2^; K. Regev^2^; V. Drory^3^; A. Dori^4^; D. Magalashvili^4^; G. Kenan^6^; Y. Mechnik Steen^7^; M. Hellmann^8^; A. Wilf‐Yarkoni^8^; T. Friedman Korn^9^; A. Vaknin‐Dembinsky^9^; A. Bsoul^10^; S. Shelly^10^; A. Karni^2^

##### ^1^Faculty of Medical & Health Sciences, Tel Aviv University, Tel Aviv, Israel; ^2^Neuroimmunology and MS Unit, Neurology Institute, Tel Aviv Sourasky Medical Center, Tel Aviv, Israel; ^3^Neuromuscular Diseases Unit, Neurology Institute, Tel Aviv Sourasky Medical Center, Tel Aviv, Israel; ^4^Department of Neurology, Sheba Medical Center, Ramat‐Gan, Israel; ^6^Department of Neurology, Shamir Medical Center, Rishon Le'Zion, Israel; ^7^Department of Neurology, Soroka Medical Center, Beersheba, Israel; ^8^Department of Neurology, Rabin Medical Center, Petah Tikva, Israel; ^9^Department of Neurology, Hadassah Hebrew University Medical Center, Jerusalem, Israel, ^10^Department of Neurology, Rambam Medical Center, Haifa, Israel

**Background and aims:** Efgartigimod has shown effectiveness in treating seropositive generalized myasthenia gravis (SP gMG). This extended study evaluated the efficacy and safety outcomes.

**Methods:** A multicenter study of 51 SP gMG patients (26 females, 25 males), assessed the impact of efgartigimod on MG‐ADL scale, response patterns, steroid sparing, and safety.

**Results:** The cohort (mean age 58.9 years, range 19–87) had a median disease duration of 4 years (range 0.6–39). Patients were followed for a median of 8 months (range 2–29), receiving a median of 3 treatment cycles (range 1–13). After the first cycle, 78.6% showed ≥2‐point MG‐ADL improvement (median 7 to 2; p = 0.0001), 33.3% achieved minimal symptom expression. Among 33 patients followed >1 year, MG‐ADL scores improved (median 6 to 2; p < 0.0001). Treatment was continued by 45.5% of patients, 24.2% did not need retreatment and treatment changed in 27.3% due to insufficient efficacy and in 3.0% for insurance reasons. In 29 evaluable patients, response patterns between cycles were: improvement with intermittent worsening to baseline (58.6%), improvement with less severe worsening (24.1%), and sustained improvement (17.2%). Of 31 prednisone users (mean dose 30.0 ± 14.6 mg), 58% had dose reductions (16.6 ± 22.01 mg; p = 0.001). Efgartigimod was well tolerated, with no treatment‐related serious adverse events.

**Conclusion:** Efgartigimod demonstrated significant clinical benefits in SP gMG, including symptom reduction, steroid‐sparing effects, and was safe. Treatment regimens were personalized, depending on response patterns ranging from single cycles to continuous maintenance of treatment cycles.

**Disclosure:** Nothing to disclose

## EPO‐115

### Molecular and ultrastructural basis of the neuromuscular junction defect in PURA syndrome

#### M. Mroczek ^1^; C. Preusse^2^; M. Bielak^3^; A. Sobolewska^3^; A. Della Marina^4^; V. Dobelmann^5^; S. Iyadurai^6^; M. Chrościńska‐Krawczyk^3^; T. Ruck^7^; H. Goebel^2^; W. Stenzel^2^; A. Roos^8^

##### ^1^University of Basel, Basel, Switzerland; ^2^Department of Neuropathology, Charité ‐ Universitätsmedizin Berlin, corporate member of Freie Universität Berlin and Humboldt‐Universität zu Berlin, Germany, Berlin; ^3^University Children Hospital, Department of Child Neurology, Lublin, Poland; ^4^University Duisburg‐Essen, Department of Pediatric Neurology, Centre for Neuromuscular Disorders, Centre for Translational, Neuro‐ and Behavioral Sciences, Essen, Germany; ^5^Department of Neurology, Medical Faculty, Heinrich Heine University Düsseldorf, Düsseldorf, Germany; ^6^Johns Hopkins All Children's Hospital, Division of Neurology, St. Petersburg, US; ^7^Department of Neurology, Medical Faculty, Heinrich Heine University Düsseldorf; Department of Neurology with Heimer Institute for Muscle Research, University Hospital Bergmannsheil, Bochum, Germany; ^8^University Duisburg‐Essen, Department of Pediatric Neurology, Centre for Neuromuscular Disorders, Centre for Translational, Neuro‐ and Behavioral Sciences, Essen, Germany; Department of Neurology, Medical Faculty, Heinrich Heine University Düsseldorf, Germany

**Background and aims:** PURA‐related neurodevelopmental disorder is an ultrarare congenital genetic condition caused by pathogenic autosomal dominant variants in the PURA gene. Although the disease is primarily classified as a central developmental disorder, some phenotypic features such as positive effect of salbutamol/pyridostigmine bromide on muscle weakness indicate an endplate defect. The aim of this study is to decipher the structural and molecular basis of the endplate involvement in PURA syndrome.

**Methods:** We performed myopathological, ultrastructural, proteomic, and qPCR studies on the muscle biopsy from a 3‐months‐old patient carrying the pathogenic (c.159del; p.(Leu54Cysfs*)) PURA variant. In addition, proteomic signature of extracellular vesicles and thrombospondin‐4 (marker protein of neuromuscular junction function) level were examined in sera derived from 8 PURA‐patients.

**Results:** Electron microscopy revealed structural endplate alterations consistent with perturbed synaptic transmission. Those include rarefication of postsynaptic clefts and vesicular alterations within endothelial capillary cells and the myofibres. Proteomics demonstrated dysregulation of structural proteins similar to those seen in congenital myopathies where treatment with endplate stimulators is frequently successful. Therefore, we suspect a defect in the vesicular transport of proteins from muscle to endplate. Further investigations, such as vesicle proteomics, thrombospondin‐4 measurement, are currently being performed to better understand the molecular mechanisms of the disease.

**Conclusion:** In individuals with PURA Syndrome, the functional deficit observed at the neuromuscular junction may be attributable to aberrant vesicle transport.

**Disclosure:** Nothing to disclose.

## EPO‐116

### Safety profile of Duchenne muscular dystrophy gene therapy

#### S. Popovich; L. Kuzenkova; E. Uvakina; T. Podkletnova; O. Globa

##### Center of child Psychologen Urology, National Medical Center for Children's Health, Moscow, Russian Federation

**Background and aims:** Delandistrogene moxeparvovec is a gene therapy available for ambulatory patients aged four through five years for whom there is no other therapeutic option of pathogenetic therapy in the Russian Federation at the expense of the charity foundation “Circle of Goodness” in May 2024.

**Methods:** 22 patients received gene therapy at the National Research Medical Center for Children's Health, Moscow, from July 2024 to December 2024. 29 weeks. We analyzed the types and timing of serious adverse events, its treatment and outcomes.

**Results:** 4 patients developed SAE. 2 patients developed acute liver injury with increased GGT and total bilirubin at 6‐8 weeks after gene therapy. Both patients required pulse therapy with methylprednisolone, followed by oral prednisone with its gradual withdrawal. Another 2 patients developed immune ‐ mediated myositis in the 4 week after gene therapy. The first patient started treatment with methylprednisolone pulse therapy followed by oral prednisone with its gradual withdrawal and IVIG on the fourth day of symptoms development. The motor decline were restored within four weeks to their level before treatment. The second patient started receiving therapy for myositis after 10th day of the onset of clinical symptoms. He required the use of invasive ventilation, methylprednisolone pulse therapy, IVIG, followed by oral prednisone and tacrolimus with full recovery in 10 weeks after starting treatment.

**Conclusion:** Our experience is useful for determining key periods of development of SAE after Delandistrogen moxeparvovec. Also we offer therapeutic options for complications.

**Disclosure:** Sofiya Popovich HONORARIA Novartis, PTC, Roche, Janssen, Astrazeneca Lyudmila Kuzenkova HONORARIA Novartis, PTC, Roche, Janssen, Astrazeneca Evgeniya Uvakina Tatyana Podkletnova HONORARIA PTC, Roche Oxana Globa HONORARIA Novartis.

## EPO‐117

### Implementation of FEES and the standardised FEES scores in the diagnosis of dysphagia in SMA ‐ DYS‐SMA trial

#### S. Hamzic ^1^; H. Krämer‐Best^1^; A. Hahn^2^; J. Wagner‐Dörr^1^; M. Butz^3^; t. braun^1^; E. Sawatzki^1^; M. Jünemann^1^; P. Schramm^1^; H. Khilan^1^; O. Alhaj Omar^1^

##### ^1^Department of Neurology, University Medical Centre Giessen and Marburg, Campus Giessen, Giessen, Germany; ^2^Department of Child Neurology, University Medical Centre Giessen and Marburg, Campus Giessen, Giessen, Germany; ^3^Kerckhoff Clinic, Bad Nauheim, Germany

**Background and aims:** Spinal muscular atrophy (SMA) is an autosomal recessive neuromuscular disease characterised by progressive degeneration of the 2nd motor neurons of the motor anterior horn. The clinical characteristics of SMA are mainly characterised by progressive muscle weakness and atrophy. The bulbar symptoms, including dysphagia, pose a major therapeutic challenge with the frequent need for artificial feeding and a high risk of pulmonary complications. The aim of the DYS‐SMA study (ClinTrials RegNo. NCT04773470; Investigator Initiated Trial supported by Roche Pharma AG) was to implement the Flexible Endoscopic Evaluation of Swallowing (FEES) and standardised FEES scores in the descriptive evaluation of swallowing pathomechanisms in patients with SMA type 1‐3 treated at the University Hospital Giessen.

**Methods:** In the prospective, interventional, open, explorative and diagnostic study, 40 SMA patients were included in the study after detailed medical consultation. The study‐related interventions were the clinical dysphagia screening and the FEES at two points in time (at visit 1 (T1) and 4 months after visit 1 (T2).

**Results:** The initial clinical assessment revealed dysphagia in 38.5% of SMA patients (n=15). After the subsequent initial FEES, the number of confirmed diagnoses of dysphagia increased to 84.6% (n=33). In our trial, most severe dysphagic symptoms are found in SMA type 1 and most frequent in SMA type 2. SMA type 3 shows only mild dysphagia.

**Conclusion:** The trial results indicate that FEES is a valid and very easy to implement imaging instrument for the diagnosis of dysphagia in SMA. Clinical assessments are insufficient to adequately diagnose dysphagia in SMA.

**Disclosure:** The trial was financially supported by Roche Pharma AG.

## EPO‐118

### Usefulness of repetitive nerve stimulation of the hypoglossal nerve in patients with Myasthenia Gravis

#### S. Bernardo ^1^; A. Oliveira^2^; C. Alves^3^; S. Palma^3^; P. Pereira^3^

##### ^1^ULS Amadora‐Sintra, Lisbon, Portugal; ^2^ULS Estuário do Tejo, Vila Franca de Xira; ^3^ULS Almada‐Seixal, Almada, Portugal

**Background and aims:** Most repetitive nerve stimulation (RNS) protocols for diagnosing Myasthenia Gravis (MG) do not assess muscles with bulbar functions. Objectives: To describe a new, non‐invasive RNS technique of the hypoglossal nerve and recorded in the submental complex muscles (SMC). The study also aimed to determine the technique's overall sensitivity, particularly in predominantly bulbar forms.

**Methods:** This observational, retrospective study included 102 individuals who underwent RNS for suspected myasthenic syndrome. Of these, 25 with a definitive MG diagnosis were selected and grouped according to their initial MGFA classification. RNS was preferably performed on five nerves: hypoglossal, accessory, radial, facial, and ulnar.

**Results:** Among the 25 diagnosed patients, 18 underwent both RNS and SFEMG, while 7 underwent RNS alone. The trapezius muscle demonstrated the highest overall sensitivity (48%) and in ocular forms (33%). The SMC had the second‐highest overall sensitivity (40%) and was the most sensitive for detecting bulbar forms (80%), followed by the trapezius (56%). In one patient with mild bulbar symptoms (MGFA IIb), the SMC was the only muscle assessed that showed a decrement.

**Conclusion:** Despite limitations due to its retrospective nature and nerve selection bias, this study revealed that the SMC was the most sensitive muscle in bulbar forms, underscoring its usefulness in evaluating patients with suspected myasthenic syndrome. Particularly in bulbar‐dominant MG, the SMC may be the only muscle showing abnormalities. Thus, this technique should be considered for inclusion in protocols for assessing myasthenic patients.

**Disclosure:** Nothing to disclose.

## EPO‐119

### Gender differences in myasthenia gravis: A retrospective cohort study

#### T. Millner ^1^; G. Nadais^2^; F. Silveira^2^; L. Braz^3^; M. Pinto^3^

##### ^1^Neurology Department, São João Local Health Unit, Porto, Portugal/Neurology Department, Central Hospital of Funchal, Madeira, Portugal; ^2^Neurology Department, São João Local Health Unit, Porto, Portugal; ^3^Neurology Department, São João Local Health Unit, Porto, Portugal/Department of Clinical Neurosciences and Mental Health, Faculty of Medicine, University of Porto, Porto, Portugal

**Background and aims:** Myasthenia gravis (MG) is an autoimmune disorder causing fluctuating muscle weakness. Early‐onset MG predominantly affects females, while late‐onset MG is more common in males. Generalized MG is more frequent in women, and ocular MG is more common in men. Other gender differences lack clear evidence. This study evaluates gender‐related differences in a single‐center MG cohort.

**Methods:** This retrospective study analyzed electronic health records, including age at diagnosis, clinical presentation, antibody status, electrophysiological studies, comorbidities, and treatment use. Statistical analysis was conducted using chi‐square and Mann‐Whitney U tests.

**Results:** Among 96 MG patients (55.2% female), 25% had ocular and 75% generalized MG. Half were early‐onset and half late‐onset. Anti‐acetylcholine receptor antibodies were present in 71.9% of cases, 3 had anti‐MuSK antibodies, and 27.1% were seronegative. Generalized MG was more frequent in women (p<0.001), while ocular MG was more common in men (p<0.001). Men with ocular MG more often presented with isolated ptosis (p=0.020). IVIG use was significantly higher in women with generalized MG (p=0.027), though there were no gender differences in exacerbations or MGFA Post‐Intervention Status. Other variables, including age of onset, thymus pathology, antibody status, nerve stimulation abnormalities, treatment use (pyridostigmine, corticosteroids, immunosuppressants), and mortality, showed no significant gender differences.

**Conclusion:** Our findings confirm gender differences in MG subtypes. The higher IVIG use in women, despite similar disease severity, may reflect a gender‐related perception of disease burden, warranting further study.

**Disclosure:** Nothing to disclose.

## Neuroimaging

## EPO‐120

Abstract withdrawn

## EPO‐121

### Longitudinal changes in [18F]FDG PET brain metabolism as a prognostic marker in autoimmune encephalitis

#### D. Cerne ^1^; G. Benvenuto^1^; S. Raffa^2^; S. Morbelli^3^; A. Uccelli^1^; D. Arnaldi^1^; P. Mattioli^1^; F. Villani^4^; L. Roccatagliata^5^; A. Lechiara^6^; E. Mobilia^6^; L. Benedetti^7^; F. Massa^1^

##### ^1^Department of Neuroscience, Rehabilitation, Ophthalmology, Genetics, Maternal and Child Health (DINOGMI), University of Genoa, Genoa, Italy; ^2^Department of Nuclear Medicine, IRCCS San Martino Polyclinic Hospital, Genoa, Italy; ^3^Unit of Nuclear Medicine, Città della Salute e della Scienza di Torino, Turin, Italy; ^4^Division of Neurophysiology and Epilepsy Center, IRCCS San Martino Polyclinic Hospital, Genoa, Italy; ^5^Department of Health Science (DISSAL), University of Genoa, Genoa, Italy; ^6^Autoimmunity Laboratory, IRCCS San Martino Polyclinic Hospital, Genoa, Italy; ^7^IRCCS San Martino Polyclinic Hospital, Genoa, Italy

**Background and aims:** Recent advancements in autoimmune encephalitis (AE) have enhanced diagnosis and management, but predicting long‐term outcomes remains challenging. This study aimed to evaluate longitudinal changes in brain [18F]FDG PET patterns in AE patients to identify specific regional metabolic variations and predict clinical outcomes.

**Methods:** This retrospective study involved 22 AE patients who underwent brain [18F]FDG PET at baseline (BS) and after treatment (follow‐up, FU). PET scans were analyzed voxel‐wise using paired t‐tests to compare metabolic activity between BS and FU. Significant clusters with at least 100 voxels and p<0.05 were identified. Volume of interest (VOI) values were correlated with clinical outcomes using partial Spearman's tests, and a general linear regression model (GLM) assessed the prognostic significance of VOI values.

**Results:** Three VOIs showed significant metabolic differences: VOI‐A (relatively hypermetabolic) included the caudate‐thalamus‐parahippocampal region, right amygdala, and anterior cingulate cortex; VOI‐B1 and VOI‐B2 (relatively hypometabolic) corresponding to the right fusiform gyrus, precuneus and temporo‐parietal cortex, respectively. Key findings include: i) lower metabolism in VOI‐B1 at BS correlated with higher CASE scores at FU (p=0.014); ii) relapsing patients had lower VOI‐B1 values at BS (p=0.026). At FU, higher metabolism in VOI‐A (p=0.021) was noted in patients with mRS>2 at BS, alongside lower metabolism in VOI‐B1 and VOI‐B2 (p=0.036 and p=0.043). Lower metabolism in VOI‐B1 at BS predicted relapse (p=0.011) and higher CASE scores at FU (p=0.021). Lower metabolism in VOI‐B2 at FU predicted mRS>2 (p=0.028).

**Figure:**
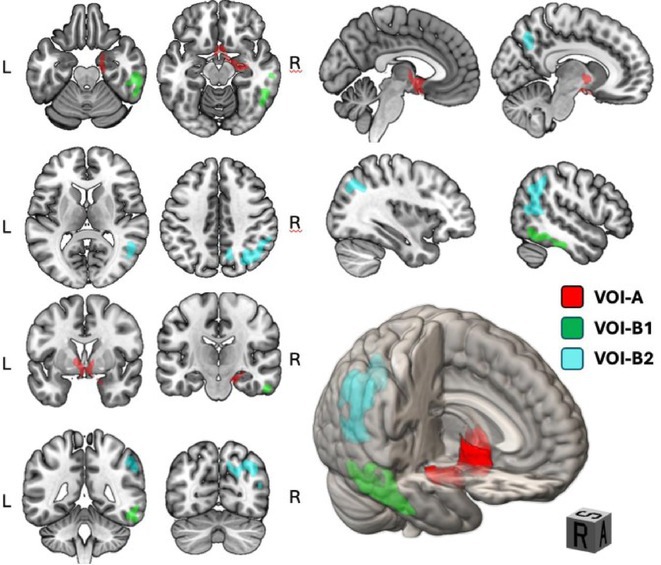
**FIGURE 1** Relative hypermetabolic (VOI‐A) and hypometabolic (VOI‐B1 and B2) regions in BS compared to FU

**Figure:**
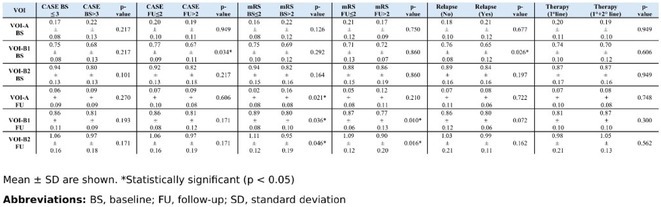
**FIGURE 2** Comparison between VOIs and clinical outcomes

**Figure:**
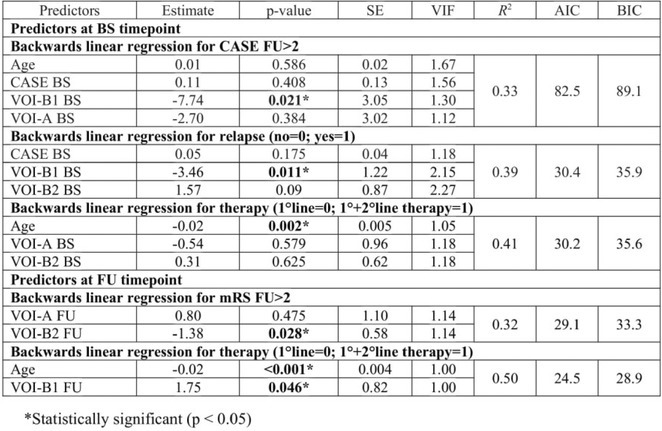
**TABLE 1** Backwards general linear regression model.

**Conclusion:** Quantitative brain [18F]FDG PET analysis can provide prognostic information in AE, identifying hypometabolism in specific regions as a prognostic marker.

**Disclosure:** Nothing to disclose.

## EPO‐122

### Stepwise connectivity from the disease epicenter aligns with ALS‐related gene expression in C9orf72 ALS patients

#### E. Spinelli ^1^; I. Bottale^1^; A. Ghirelli^1^; S. Basaia^2^; A. Cattani^3^; V. Castelnovo^4^; E. Canu^4^; T. Russo^5^; P. Schito^5^; Y. Falzone^5^; T. Domi^6^; L. Pozzi^6^; P. Carrera^7^; M. Filippi^8^; F. Agosta^1^

##### ^1^Neuroimaging Research Unit, Division of Neuroscience, and Neurology Unit, IRCCS San Raffaele Scientific Institute, and Vita‐Salute San Raffaele University, Milan, Italy; ^2^Neuroimaging Research Unit, Division of Neuroscience, IRCCS San Raffaele Scientific Institute, Milan, Italy; ^3^Vita‐Salute San Raffaele University, Milan, Italy; ^4^Neuroimaging Research Unit, Division of Neuroscience, and Neurology Unit, IRCCS San Raffaele Scientific Institute, Milan, Italy; ^5^Neurology Unit, IRCCS San Raffaele Scientific Institute, Milan, Italy; ^6^Experimental Neuropathology Unit, Division of Neuroscience, Institute of Experimental Neurology, IRCCS San Raffaele Scientific Institute, Milan, Italy; ^7^Unit of Genomics for Human Disease Diagnosis, IRCCS San Raffaele Scientific Institute, Milan, Italy; ^8^Neurology Unit, Neurorehabilitation Unit, Neurophysiology Service, and Neuroimaging Research Unit, Division of Neuroscience, IRCCS San Raffaele Scientific Institute, and Vita‐Salute San Raffaele University, Milan, Italy

**Background and aims:** Stepwise functional connectivity (SFC) detects whole‐brain functional couplings of a selected region of interest at increasing topological distances. This study applied SFC to test the hypothesis that stepwise architecture propagating from the disease epicenter would be related with patterns of gene expression in patients with amyotrophic lateral sclerosis (ALS) carrying C9orf72 repeat expansion.

**Methods:** Thirty patients with C9orf72‐ALS and 35 age‐matched healthy controls underwent brain MRI on a 3T scanner. The region of interest was defined as the peak of atrophy observed using voxel‐based morphometry in C9orf72‐ALS patients. We tested the correlation between SFC architecture propagating from the disease epicenter in healthy conditions and the expression pattern of the major causative genes in ALS, as obtained from the Allen Human Brain Atlas.

**Results:** The disease epicenter was identified in the right frontal superior cortex in C9orf72‐ALS patients. Significant correlations were identified between SFC topological distance from the right frontal superior cortex and TARDBP expression (r=0.262, p=0.017). Similar results were obtained analyzing the correlation between SFC maps and C9orf72 expression (r=0.260, p= 0.018).

**Conclusion:** This study provides insights into the relationship between the topology of target functional networks in ALS patients with C9orf72 expansion and the transcriptomic patterns of ALS‐related genes. Specifically, this study suggests that higher gene expression in regions more proximal to the disease epicenter might shape pathological spreading along the SFC architecture in C9orf72‐ALS patients.

**Disclosure:** Funding. ERC (StG‐2016_714388_NeuroTRACK); Foundation Research on Alzheimer Disease. Co‐funding by the Next Generation EU [DM 1557 11.10.2022]. Disclosures: MF consulting fees from Alexion, Almirall, Biogen, Merck, Novartis, Roche, Sanofi; speaking fees from Bayer, Biogen, Celgene, Chiesi Italia SpA, Eli Lilly, Genzyme, Janssen, Merck‐Serono, Neopharmed Gentili, Novartis, Novo Nordisk, Roche, Sanofi, Takeda, and TEVA; participation in Advisory Boards for Alexion, Biogen, Bristol‐Myers Squibb, Merck, Novartis, Roche, Sanofi, Sanofi‐Aventis, Sanofi‐Genzyme, Takeda; scientific direction of educational events for Biogen, Merck, Roche, Celgene, Bristol‐Myers Squibb, Lilly, Novartis, Sanofi‐Genzyme; he receives research support from Biogen Idec, Merck‐Serono, Novartis, Roche, the Italian Ministry of Health, the Italian Ministry of University and Research, and Fondazione Italiana Sclerosi Multipla. F. Agosta is Associate Editor of NeuroImage: Clinical, has received speaker honoraria from Biogen Idec, Roche, Eli Lilly and GE Healthcare, and receives or has received research supports from the Italian Ministry of Health, the Italian Ministry of University and Research, AriSLA (Fondazione Italiana di Ricerca per la SLA), the European Research Council, the EU Joint Programme – Neurodegenerative Disease Research (JPND), and Foundation Research on Alzheimer Disease (France).

## EPO‐123

### Altered hypothalamic functional connectivity in amyotrophic lateral sclerosis

#### F. Freri ^1^; E. Spinelli^2^; E. Canu^3^; F. Roselli^4^; V. Castelnovo^3^; H. Müller^4^; J. Kassubek^4^; A. Ludolph^4^; M. Filippi^5^; F. Agosta^2^

##### ^1^Neuroimaging Research Unit, Division of Neuroscience, IRCCS San Raffaele Scientific Institute, Milan, Italy; ^2^Neuroimaging Research Unit, Division of Neuroscience, and Neurology Unit, IRCCS San Raffaele Scientific Institute, and Vita‐Salute San Raffaele University, Milan, Italy; ^3^Neuroimaging Research Unit, Division of Neuroscience, and Neurology Unit, IRCCS San Raffaele Scientific Institute, Milan, Italy; ^4^Department of Neurology, University of Ulm, and German Center for Neurodegenerative Diseases (DZNE), Ulm, Germany; ^5^Neurology Unit, Neurorehabilitation Unit, Neurophysiology Service, and Neuroimaging Research Unit, Division of Neuroscience, IRCCS San Raffaele Scientific Institute, and Vita‐Salute San Raffaele University, Milan, Italy

**Background and aims:** Hypermetabolism is a newly identified clinical feature of amyotrophic lateral sclerosis (ALS), associated with shorter survival. In ALS cases compared to healthy controls (HC), hypothalamic volume reduction and white matter (WM) alterations between hypothalamus, orbitofrontal and insular regions have been reported. These changes suggest a relationship between patients’ hypermetabolic state and hypothalamic dysfunction. However, the hypothalamic functional connectivity and its association with clinical severity and WM connectivity in ALS remain unclear.

**Methods:** Seventy‐one ALS patients and thirty‐nine HC underwent structural and resting‐state functional MRI. In each subject, the bilateral hypothalamus was manually segmented, and a seed‐based resting‐state functional connectivity (RS‐FC) analysis was performed between this region and the rest of the brain. Hypothalamic RS‐FC was then compared among groups. Furthermore, in ALS, the relationship between RS‐FC significant changes, ALSFRS scores‐defined by ALS Functional Rating Scale (ALSFRS)‐ and the WM tract integrity‐assessed through tract‐based spatial statistics‐ were investigated.

**Results:** Compared to HC, ALS patients exhibited increased hypothalamic RS‐FC with the caudate nuclei bilaterally. Additionally, in patients, greater disease severity and decreased WM integrity of the genu of corpus callosum correlated with increased RS‐FC between hypothalamus, caudate nucleus and orbitofrontal cortex bilaterally.

**Conclusion:** Our findings support hypothalamic alterations in ALS. These changes may be related to the hypermetabolic clinical feature in these patients, but further studies are needed to verify this association and its impact on patients’ survival. The early detection of the hypothalamic changes in ALS could be useful for prognostic stratification and for monitoring the effect of interventions.

**Disclosure:** Funding: The EU Joint Programme Neurodegenerative Disease Research – HiCALS project; Next Generation EU/National Recovery and Resilience Plan, Investment PE8‐Project Age‐It. Disclosures: FF, EGS, FR, VC, HPM, JK, ACL report no disclosures. EC receives grants from Italian Ministry of Health. MF received compensation for consulting services or speaking activities from Alexion, Almirall, Bayer, Biogen, Celgene, Chiesi Italia SpA, Eli Lilly, Genzyme, Janssen, Merck‐Serono, Neopharmed Gentili, Novartis, Novo Nordisk, Roche, Sanofi Takeda, and TEVA; Advisory Boards for Alexion, Biogen, Bristol‐Myers Squibb, Merck, Novartis, Roche, Sanofi, Sanofi‐Aventis, Sanofi‐Genzyme, Takeda; scientific direction of educational events for Biogen, Merck, Roche, Celgene, Bristol‐Myers Squibb, Lilly, Novartis, Sanofi‐Genzyme; he receives research support from Biogen Idec, Merck‐Serono, Novartis, Roche, the Italian Ministry of Health, the Italian Ministry of University and Research, and FISM. FA received speaker honoraria from Biogen Idec, Roche, Eli Lilly, GE Healthcare; receives research supports from IMH, IMUR, AriSLA, ERC, EU JPND Research, Foundation Research on AD (France).

## EPO‐124

### Insights from multi‐shell diffusion and functional MRI analysis in trigeminal neuralgia

#### F. Valtorta ^1^; S. Basaia^1^; L. Albano^2^; E. Pompeo^3^; D. Emedoli^4^; E. Sibilla^5^; E. Sarasso^6^; P. Mortini^3^; F. Agosta^7^; M. Filippi^8^

##### ^1^Neuroimaging Research Unit, Division of Neuroscience, IRCCS San Raffaele Scientific Institute, Milan, Italy; ^2^Neuroimaging Research Unit, Division of Neuroscience, and Neurosurgery and Gamma Knife Radiosurgery Unit, IRCCS San Raffaele Scientific Institute, and Vita‐Salute San Raffaele University, Milan, Italy; ^3^Neurosurgery and Gamma Knife Radiosurgery Unit, IRCCS San Raffaele Scientific Institute, and Vita‐Salute San Raffaele University, Milan, Italy; ^4^Department of Rehabilitation and Functional Recovery, IRCCS Ospedale San Raffaele, Milan, Italy; ^5^Neuroimaging Research Unit, Division of Neuroscience, and Neurology Unit, IRCCS San Raffaele Scientific Institute, Milan, Italy; ^6^Neuroimaging Research Unit, Division of Neuroscience, IRCCS San Raffaele Scientific Institute, Vita‐Salute San Raffaele University, Milan, Italy; and DINOGMI, University of Genoa, Genoa, Italy; ^7^Neuroimaging Research Unit, Division of Neuroscience, and Neurology Unit, IRCCS San Raffaele Scientific Institute, and Vita‐Salute San Raffaele University, Milan, Italy; ^8^Neurology Unit, Neurorehabilitation Unit, Neurophysiology Service, and Neuroimaging Research Unit, Division of Neuroscience, IRCCS San Raffaele Scientific Institute, and Vita‐Salute San Raffaele University, Milan, Italy

**Background and aims:** This study aimed to investigate microstructural alterations in gray matter (GM) and white matter (WM) in Trigeminal Neuralgia (TN) patients. Additionally, it explored the neural correlates of pain in TN patients during an observation functional MRI (fMRI) task.

**Methods:** Thirteen TN patients and 29 controls were enrolled and underwent multi‐shell diffusion brain MRI and an fMRI task. TN patients were re‐evaluated 3‐months post‐Gamma Knife radiosurgery (GKRS). Fractional anisotropy (FA), and Intra‐Cellular Volume Fraction (ICVF) maps were computed using the NODDI model. Then, tract‐based spatial statistics (TBSS) and GM‐based spatial statistics (GBSS) were performed to estimate WM and GM damage between groups. fMRI task required patients to observe facial gestures during daily activities or specific movements that triggered pain. Brain activity was analysed between baseline and 3‐months follow‐up.

**Results:** TBSS showed reduced FA in the brainstem and decreased ICVF along the anterior thalamic radiation and near the periaqueductal grey in TN patients. GBSS revealed lower ICVF in the temporal lobe and higher ICVF in subcortical regions, insula and temporal pole, suggesting GM microstructural alterations. At 3‐months ICVF increased in TN patients in precentral gyri, insula and temporal pole. At baseline, TN patients showed widespread brain activation, which decreased and shifted post‐GKRS, reflecting changes in pain processing.

**Conclusion:** WM involvement suggests alterations beyond sensory and motor pathways, while microstructural GM changes may reflect persistent nociceptive stimuli. Moreover, these findings enhance our understanding of TN's neural mechanisms and GKRS's effect on brain function.

**Disclosure:** Funding: Italian Ministry of Health (MSAL) (GR‐2021‐12374601). Disclosures: SB, ES grants from MSAL. FV, DE, ES, EP nothing to disclose. LA, PM grants from Boston Scientific. LA research support from Fondazione Cariplo. FA speaker fees from Biogen Idec, Italfarmaco, Roche, Zambon and Eli Lilly; grants from MSAL, Italian Ministry of University and Research (MUR), AriSLA, the European Research Council, the EU Joint Programme – Neurodegenerative Disease Research (JPND), and Foundation Research on Alzheimer Disease (France). MF received compensation for consulting services from Alexion, Almirall, Biogen, Merck, Novartis, Roche, Sanofi, speaking activities from Bayer, Biogen, Celgene, Chiesi Italia SpA, Eli Lilly, Genzyme, Janssen, Merck‐Serono, Neopharmed Gentili, Novartis, Novo Nordisk, Roche, Sanofi, Takeda, and TEVA, participation in Advisory Boards for Alexion, Biogen, Bristol‐Myers Squibb, Merck, Novartis, Roche, Sanofi, Sanofi‐Aventis, Sanofi‐Genzyme, Takeda, scientific direction of educational events for Biogen, Merck, Roche, Celgene, Bristol‐Myers Squibb, Lilly, Novartis, Sanofi‐Genzyme, he receives research support from Biogen Idec, Merck‐Serono, Novartis, Roche, MSAL, MUR, and Fondazione Italiana Sclerosi Multipla.

## EPO‐125

### Role of brain MRI in PFBC (Primary Familial Brain Calcification): Results from a single‐center Italian cohort

#### G. Bonato ^1^; G. Librizzi^3^; A. Adraman^3^; M. Corazza^1^; A. Fabris^4^; F. Pistonesi^1^; C. Bertolin^5^; L. Salviati^5^; A. Antonini^1^; R. Manara^4^; M. Carecchio^1^

##### ^1^Parkinson and Movement Disorders Unit, Centre for Rare Neurological Diseases (ERN‐RND), Department of Neuroscience, University of Padova, Padova, Italy; ^3^Padova Neuroscience Center (PNC), University of Padova, Padova, Italy; ^4^Neuroradiology Unit, DIMED, University Hospital of Padova, Padova, Italy; ^5^Medical Genetics Unit, Department of Women and Children's Health, University of Padova, Padova, Italy

**Background and aims:** PFBC (Primary Familial Brain Calcification) is a rare genetic neurodegenerative disorder characterized by bilateral calcium deposition in basal ganglia, featuring movement disorders, psychiatric or cognitive disturbances. Correlations between genetic, clinical and radiological data are scarce. CT scan is the gold standard technique to image brain calcification, whereas the role of MRI is currently limited and no clear correlations between genetic PFBC subtypes, clinical phenotypes and radiological features are known.

**Methods:** 45 PFBC subjects and 67 matched healthy controls from the ERN‐RND Center of Padua University underwent 3T brain MRI (T1, FLAIR, SWI sequences, FreeSurfer cortical thickness analysis), genetic testing (NGS Illumina NextSeq), clinical and neuropsychological evaluations.

**Results:** FLAIR and SWI sequences proved sensitive in identifying brain calcifications. White matter involvement and leukopathy in both cerebrum and cerebellum were significantly associated with cognitive impairment (OR 5.7, p=0.02; mean MoCA 21 vs 26, p=0.004). The presence of dentate nuclei calcifications was a significant predictor of a genetic diagnosis (OR 7.3, p=0.03) and of behavioral‐psychiatric symptoms (91% vs 56%, p=0.04). All MYORG mutation carriers (n=5) showed punctate calcifications in the brainstem in a region approximately corresponding to hypoglossal nerve nucleus, possibly contributing to dysarthria, a typical feature of this genetic subtype. Cortical thickness reduction was observed in the left premotor cortex in PFBC (p<0.05), whereas no alterations were documented in asymptomatic PFBC subjects compared to HC. Cerebellar atrophy was demonstrated in MYORG subjects.

**Figure:**
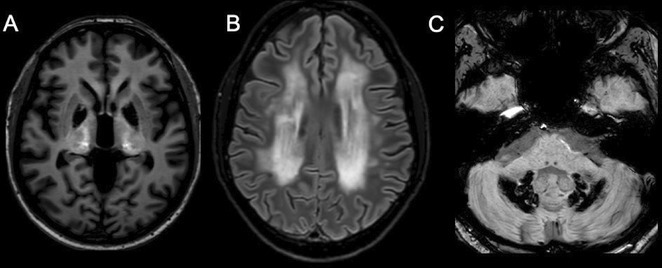
**FIGURE 1** Brain MRI in PFBC. A: T1 sequence: basal ganglia calcifications in PFBC. B: FLAIR sequence: extensive leukoencephalopathy in a PDGFB mutation carrier. C: SWI sequence: dentate nuclei and brainstem calcifications in a MYORG mutation carrier.

**Figure:**
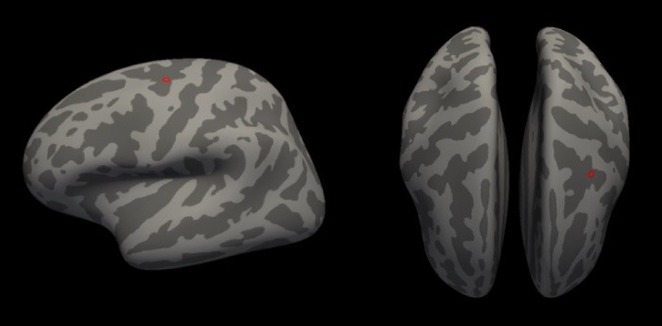
**FIGURE 2** Cortical Thickness analysis results: in red the area (left premotor cortex) with significant atrophy in PFBC vs healthy controls.

**Conclusion:** Beside CT scan, brain MRI may be a useful tool to diagnose PFBC with potentially relevant prognostic correlates.

**Disclosure:** Nothing to disclose.

## EPO‐126

### Glymphatic dysfunction as an indicator of disease burden and a potential biomarker in anti‐NMDAR encephalitis

#### H. Cai; D. Wu; Z. Lu; Z. Kang; B. Zhang

##### Department of Neurology, the Third Affiliated Hospital of Sun Yat‐sen University, Guangzhou, China

**Background and aims:** Glymphatic dysfunction is closely associated with the progression of neuroinflammation, indicating a potential mechanism underlying anti‐N‐methyl‐D‐aspartate receptor (anti‐NMDAR) encephalitis. To date, no studies have reported about glymphatic dysfunction in anti‐NMDAR encephalitis. The use of the diffusion tensor imaging analysis along the perivascular space (DTI‐ALPS) index, free water in white matter (FW‐WM) and perivascular space volume fraction (PVSVF) represent a noninvasive but conventional method for evaluating glymphatic function and disease severity. This study aimed to explore the utility of these biomarkers for evaluating glymphatic dysfunction in patients with anti‐NMDAR encephalitis and establish their effectiveness in differentiating patients from healthy controls.

**Methods:** In the present study, we enrolled 20 patients with anti‐NMDAR encephalitis and 18 age‐ and sex‐matched healthy controls (HCs). Glymphatic function was assessed using DTI‐ALPS index, FW‐WM, and PVSVF. All participants underwent follow‐up 3 months after discharge. Correlation analyses between magnetic resonance imaging (MRI) indices of glymphatic function and clinical factors were performed.

**Figure:**
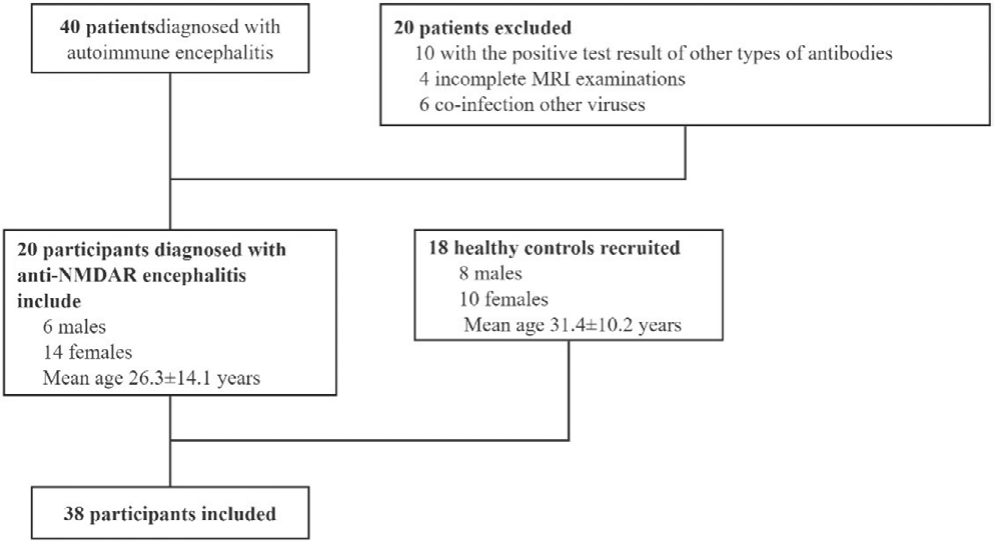
**FIGURE 1** Patient selection flowchart.

**Results:** Patients with anti‐NMDAR encephalitis demonstrated a significantly lower DTI‐ALPS index and higher FW‐WM and PVSVF values than HCs. Similar findings were observed in patients with negative conventional structural MRI findings. Significant correlations were identified between the MRI indices and clinical factors, including the Clinical Assessment Scale for Auto‐immune Encephalitis score, the number of clinical symptoms, and other related clinical factors.

**Figure:**
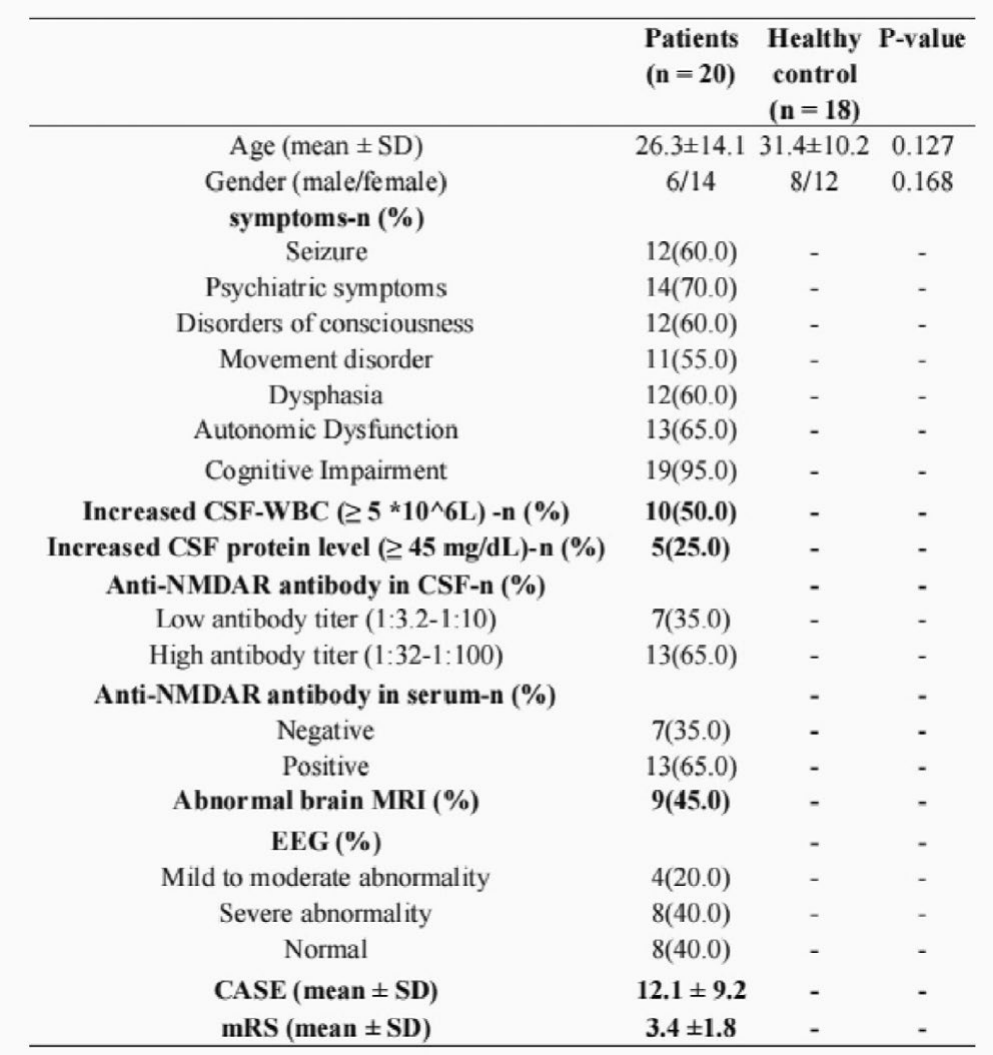
**TABLE 1** Demographics and clinical characteristics of patients with anti‐NMDAR encephalitis.

**Figure:**
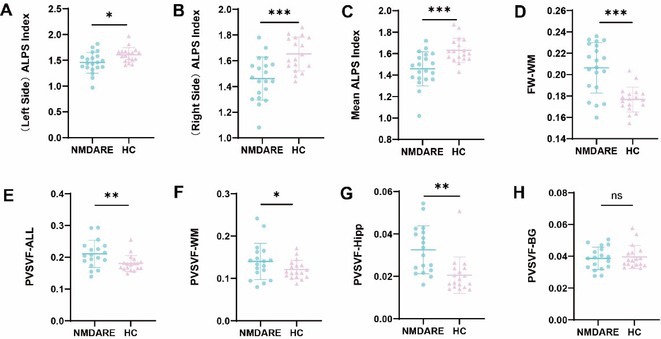
**FIGURE 2** Comparisons of MRI indices between anti‐NMDAR encephalitis patients and HCs.

**Conclusion:** Our study revealed glymphatic dysfunction in patients with anti‐NMDAR encephalitis using MRI indices. PVSVF, DTI‐ALPS index, and FW‐WM are effective in distinguishing patients from HCs, offering deeper insights into disease progression beyond traditional MRI findings.

**Disclosure:** Nothing to disclose.

## EPO‐127

### Brain microstructural damage in multiple sclerosis using T1W/T2W ratio: An Italian neuroimaging network initiative study

#### L. Storelli^1^; E. Pagani^1^; M. Margoni^2^; A. Meani^1^; P. Preziosa ^3^; A. Gallo^4^; A. Bisecco^4^; P. Pantano^5^; C. Piervincenzi^6^; N. De Stefano^7^; R. Cortese^7^; M. Rocca^3^; M. Filippi^8^

##### ^1^Neuroimaging Research Unit, Division of Neuroscience, IRCCS San Raffaele Scientific Institute, Milan, Italy; ^2^Neuroimaging Research Unit, Division of Neuroscience, Neurology Unit, and Neurorehabilitation Unit, IRCCS San Raffaele Scientific Institute, Milan, Italy; ^3^Neuroimaging Research Unit, Division of Neuroscience, and Neurology Unit, IRCCS San Raffaele Scientific Institute, and Vita‐Salute San Raffaele University, Milan, Italy; ^4^Department of Advanced Medical and Surgical Sciences, and 3T MRI‐Center, University of Campania “Luigi Vanvitelli”, Naples, Italy; ^5^Department of Human Neurosciences, Sapienza University of Rome, Rome, Italy, and IRCCS NEUROMED, Pozzilli, Italy; ^6^Department of Human Neurosciences, Sapienza University of Rome, Rome, Italy; ^7^Department of Medicine, Surgery and Neuroscience, University of Siena, Siena, Italy; ^8^Neurology Unit, Neurorehabilitation Unit, Neurophysiology Service, and Neuroimaging Research Unit, Division of Neuroscience, IRCCS San Raffaele Scientific Institute, and Vita‐Salute San Raffaele University, Milan, Italy

**Background and aims:** This study assessed T1w/T2w ratios from MRI scans as a potential marker for myelin content in multiple sclerosis (MS) using a large, multicenter cohort from the Italian Neuroimaging Network Initiative (INNI).

**Methods:** This cross‐sectional study included 272 healthy controls (HC) and 918 MS patients from the INNI repository. MRI data, including sagittal 3D T1‐weighted and axial 2D T2‐weighted images, were collected. T1w/T2w ratios were calculated using a pipeline based on Ganzetti et al. that involved intensity bias correction, calibration, and histogram normalization. Tissue segmentation masks were overlaid to obtain the ratio values. Z‐scores for MS were calculated by fitting linear mixed models on HC data. The study also explored associations between T1w/T2w ratios in various brain regions and disease duration and Expanded Disability Status Scale (EDSS) scores.

**Results:** Compared to HC, the T1w/T2w ratio was lower in white matter (WM) lesions of all MS phenotypes, in relapsing‐remitting MS (RRMS) normal appearing WM (NAWM), and in the cortex for both RRMS and secondary progressive MS (all p<0.001). The ratio was higher in the thalamus, caudate, putamen, pallidum, and hippocampus of MS patients (all p<0.046). In relapse‐onset MS, lower T1w/T2w ratios were observed in WM lesions, NAWM, and the cortex at EDSS <3.0 (all p≤0.003). Higher ratios were noted in the thalamus, caudate, and putamen at EDSS≥4.0 (all p≤0.05). Longer disease duration and higher EDSS correlated with changes in T1w/T2w ratios across brain regions.

**Conclusion:** The T1w/T2w ratio may serve as a clinically relevant marker of demyelination, neurodegeneration, and iron accumulation in MS.

**Disclosure:** Funding. Supported by Fondazione Italiana Sclerosi Multipla (FISM2023/S/1) and financed or co‐financed with the ‘5 per mille’ public funding. Disclosures. Nothing to disclose.

## EPO‐128

### Handwriting difficulties in Parkinson's disease: technological assessment and resting‐state fMRI correlates

#### L. Zenere^1^; E. Sarasso ^2^; A. Gardoni^3^; E. Canu^4^; R. Balestrino^5^; A. Grassi^1^; M. Forghieri^6^; D. Emedoli^7^; S. Basaia^1^; V. Castelnovo^4^; E. Sibilla^4^; M. Malcangi^5^; M. Volontè^8^; D. Corbetta^7^; F. Agosta^9^; M. Filippi^10^

##### ^1^Neuroimaging Research Unit, Division of Neuroscience, IRCCS San Raffaele Scientific Institute, Milan, Italy; ^2^Neuroimaging Research Unit, Division of Neuroscience, IRCCS San Raffaele Scientific Institute, Vita‐Salute San Raffaele University, Milan, Italy; and DINOGMI, University of Genoa, Genoa, Italy; ^3^Neuroimaging Research Unit, Division of Neuroscience, IRCCS San Raffaele Scientific Institute, and Vita‐Salute San Raffaele University, Milan, Italy; ^4^Neuroimaging Research Unit, Division of Neuroscience, and Neurology Unit, IRCCS San Raffaele Scientific Institute, Milan, Italy; ^5^Neurology Unit, and Neurorehabilitation Unit, IRCCS San Raffaele Scientific Institute, Milan, Italy; ^6^Vita‐Salute San Raffaele University, Milan, Italy; ^7^Department of Rehabilitation and Functional Recovery, IRCCS Ospedale San Raffaele, Milan, Italy; ^8^Neurology Unit, IRCCS San Raffaele Scientific Institute, Milan, Italy; ^9^Neuroimaging Research Unit, Division of Neuroscience, and Neurology Unit, IRCCS San Raffaele Scientific Institute, and Vita‐Salute San Raffaele University, Milan, Italy, ^10^Neurology Unit, Neurorehabilitation Unit, Neurophysiology Service, and Neuroimaging Research Unit, Division of Neuroscience, IRCCS San Raffaele Scientific Institute, and Vita‐Salute San Raffaele University, Milan, Italy

**Background and aims:** Handwriting is a complex activity requiring cognitive and motor abilities, often impaired in people with Parkinson's Disease (pwPD). Proper handwriting assessment is essential to develop and evaluate the effect of rehabilitation protocols. To assess handwriting alterations in pwPD compared to healthy controls (HC) and to identify the functional neural correlates of handwriting changes through using resting‐state fMRI functional connectivity (RS‐FC) analysis.

**Methods:** Forty pwPD and 30 age‐ and sex‐matched HC underwent handwriting and hand dexterity assessments, neuropsychological evaluation, and RS‐fMRI. A tablet‐based handwriting assessment included four tasks: Systematic Screening for Handwriting Difficulties‐SOS test (copying a text), funnel test (coloring a shape), closed loop task (drawing specific symbols), and repetitive cursive loop task (writing repeated symbols). SOS test was executed also on paper. RS‐fMRI analysis used MELODIC to identify RS‐FC differences, and correlations with clinical variables significantly differing between groups were assessed.

**Results:** Compared to HC, pwPD showed smaller word size, slower drawing speed, and poorer performance in the handwriting tasks on tablet. SOS test on paper confirmed slower writing speed, smaller size, and lower writing quality in pwPD. RS‐FC analysis revealed decreased connectivity in the basal ganglia, cerebellum, ventral default mode, and visual networks, alongside increased RS‐FC in the salience and executive control networks. Correlations showed that smaller writing amplitude and poorer handwriting quality were associated with altered RS‐FC in motor and cognitive networks.

**Conclusion:** PwPD exhibited handwriting impairments that were correlated to RS‐FC changes in motor and cognitive networks, highlighting the neurological basis of handwriting difficulties in pwPD.

**Disclosure:** Funding: Italian Ministry of Health (MSAL) (GR‐2018‐12366005). Disclosures: LZ, AG, EC, RB, AGr, DE, VC, ES, MM, MAV: nothing. ES, SB and DC grants form MSAL. MF is Editor‐in‐Chief of the Journal of Neurology, Associate Editor of Human Brain Mapping, Neurological Sciences, and Radiology; received compensation for consulting services from Alexion, Almirall, Biogen, Merck, Novartis, Roche, Sanofi; speaking activities from Bayer, Biogen, Cel‐ gene, Chiesi Italia SpA, Eli Lilly, Genzyme, Janssen, Merck‐Serono, Neo‐ pharmed Gentili, Novartis, Novo Nordisk, Roche, Sanofi, Takeda and TEVA; participation in Advisory Boards for Alexion, Biogen, Bristol‐Myers Squibb, Merck, Novartis, Roche, Sanofi, Sanofi‐Aventis, Sanofi‐Genzyme, Takeda; scientific direction of educational events for Biogen, Merck, Roche, Celgene, Bristol‐Myers Squibb, Lilly, Novartis, Sanofi‐Genzyme; he receives research support from Biogen Idec, Merck‐Serono, Novartis, Roche, MSAL, the Italian Ministry of University and Research and Fondazione Italiana Sclerosi Multipla. F.A. is Associate Editor of NeuroImage: Clinical, has received speaker honoraria from Biogen Idec, Roche, Eli Lilly and GE Healthcare, and receives or has received research supports from MSAL, the Italian Ministry of University and Research, AriSLA, the European Research Council, the EU Joint Programme—Neurodegenerative Disease Research and Foundation Research on Alzheimer Disease (France).

## EPO‐129

### Structural connectivity between thalamic nuclei and hippocampus in temporal lobe epilepsy

#### S. Yildirim ^1^; R. Stepponat^2^; F. Fischmeister^2^; M. Tomschik^1^; V. Schmidbauer^2^; F. Khalaveh^1^; J. Koren^3^; C. Baumgartner^3^; E. Pataraia^4^; S. Bonelli^4^; K. Rössler^1^; G. Kasprian^2^; C. Dorfer^1^

##### ^1^Department of Neurosurgery, Medical University of Vienna, Vienna, Austria; ^2^Division of Neuroradiology and Musculoskeletal Radiology, Medical University of Vienna, Vienna, Austria; ^3^Department of Neurology, Clinic Hietzing, Vienna, Austria; ^4^Department of Neurology, Medical University of Vienna, Vienna, Austria

**Background and aims:** Hippocampal sclerosis (HS) is the most prevalent structural alteration in temporal lobe epilepsy (TLE), while thalamic atrophy is frequently observed in cases with extratemporal manifestations. This study aimed to investigate differences in the structural connectivity of individual thalamic nuclei between TLE patients and healthy controls.

**Methods:** Thirty‐six TLE patients who underwent pre‐surgical magnetic resonance imaging (MRI) and 18 healthy controls were enrolled in this study. Patients were subdivided into TLE with HS (TLE‐HS) and MRI‐negative TLE (TLE‐MRneg). Probabilistic tractography and whole‐brain segmentation, including the thalamus, were performed to determine the number of streamlines per voxel between the thalamic nuclei and hippocampus. Connectivity strength and volume of regions were correlated with clinical data.

**Results:** The volume of the entire thalamus ipsilateral to seizure onset was significantly decreased in TLE‐HS compared to controls (Mann‐Whitney‐U test: pFDR < 0.01) with the anterior thalamic nuclei (ANT) as important contributor. Furthermore, decreased ipsilateral connectivity strength between the hippocampus and ANT was detected in TLE‐HS (pFDR < 0.01) compared to TLE‐MRneg and controls which correlated negatively with the duration of epilepsy (ρ = ‐0.512, p = 0.025) and positively with seizure frequency (ρ = 0.603, p = 0.006). Moreover, ANT volume correlated negatively with epilepsy duration in TLE‐HS (ρ = ‐0.471, p = 0.042).

**Conclusion:** ANT showed atrophy and decreased connectivity in TLE‐HS, which correlated with epilepsy duration and seizure frequency. Network analyses may enhance the understanding of seizure origin and propagation, and provide the promising potential to improve the selection of ideal DBS candidates and targets.

**Disclosure:** Nothing to disclose.

## EPO‐130

### Imaging blood‐brain barrier dysfunction in drug‐resistant epilepsy: a multi‐center feasibility study

#### N. Cafri ^1^; F. Beninnger^1^; A. Friedman^3^; I. Goldberg^1^; B. Wandschneider^3^; M. Campbell^4^

##### ^1^Department of Neurology, Rabin Medical Center, Beilinson Hospital and Tel‐Aviv University, Israel; ^3^Department of Clinical and Experimental Epilepsy, UCL Queen Square Institute of Neurology, London; ^4^FutureNeuro, Science Foundation Ireland Research Centre for Chronic and Rare Neurological Diseases, Royal College of Surgeons in Ireland, University of Medicine and Health Sciences, Dublin, Ireland

**Background and aims:** Dysfunction of the blood‐brain barrier (BBBD) has been linked to various neurological disorders, including epilepsy. This study aims to utilize dynamic contrast‐enhanced MRI (DCE‐MRI) to identify and compare brain regions with BBBD in patients with epilepsy (PWE) and healthy individuals.

**Figure:**
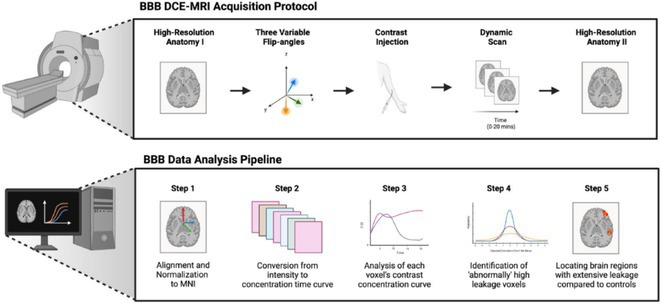
**FIGURE 1** BBBD analysis uses DCE‐MRI to capture brain images pre‐ and post‐contrast, followed by alignment, curve conversion, voxel analysis, and identification of abnormal leakage regions.

**Methods:** We scanned 50 drug‐resistant epilepsy (DRE) patients and 58 control participants from four global specialized epilepsy centers using dynamic contrast‐enhanced MRI (DCE‐MRI). The presence and extent of BBBD were analyzed and compared between PWE and healthy controls.

**Results:** Both greater brain volume and higher number of brain regions with BBBD were significantly present in PWE compared to healthy controls (p < 10‐7). No differences in total brain volume with BBBD were observed in patients diagnosed with either focal seizures or generalized epilepsy, despite variations in the affected regions. Overall brain volume with BBBD did not differ in PWE with MRI‐visible lesions compared with non‐lesional cases. BBBD was observed in brain regions suspected to be related to the onset of seizures in 82% of patients (n = 39) and was typically identified in, adjacent to, and/or in the same hemisphere as the suspected epileptogenic lesion (n = 10).

**Figure:**
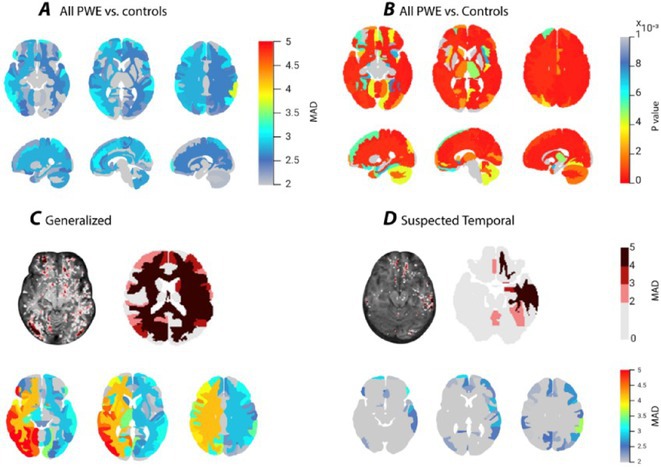
**FIGURE 2** Imaging patients with epilepsy reveals a correlation between blood‐brain barrier dysfunction and seizure onset zone diagnosis.

**Conclusion:** These findings are consistent with pre‐clinical studies that highlight the role of BBBD in the development of DRE and identify microvascular stabilization as a potential therapeutic strategy.

**Figure:**
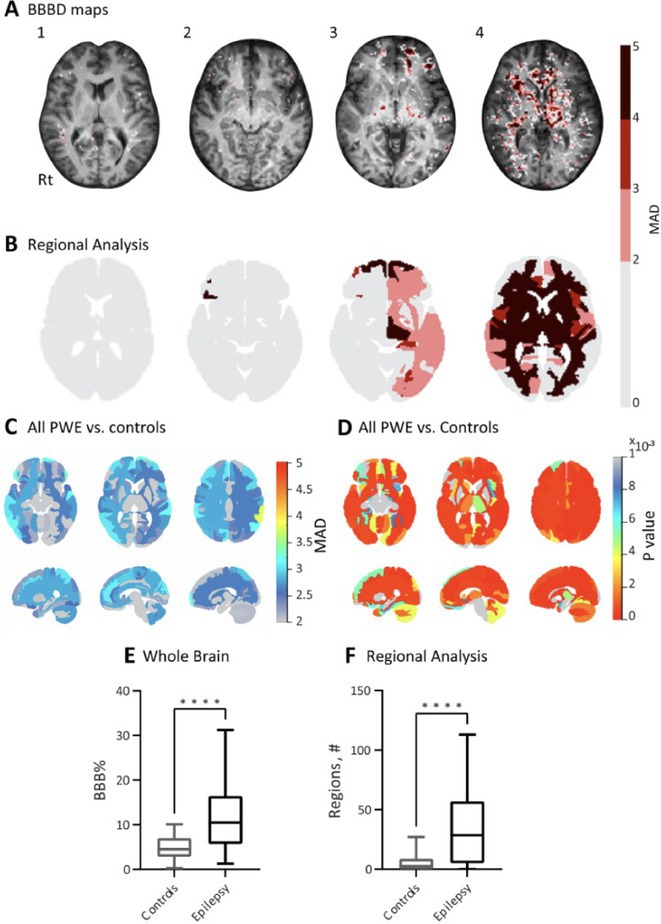
**FIGURE 3** DCE‐MRI reveals persistent BBBD in epileptic patients.

**Disclosure:** Nothing to disclose.

## EPO‐131

### Longitudinal evolution of Neuromelanin‐MRI signal in the substantia nigra in Parkinson's disease

#### P. Lillebostad ^1^; A. Lundervold^1^; C. Tzoulis^2^

##### ^1^Department of Biomedicine, University of Bergen, Bergen, Norway; ^2^Neuro‐SysMed, Department of Neurology, Haukeland University Hospital, Bergen, Norway

**Background and aims:** Neuromelanin‐sensitive MRI (NM‐MRI) of the substantia nigra (SN) is increasingly utilized in Parkinson's disease (PD) research, showing reduced signal intensity and volume loss in patients. Although group differences are well established, the temporal evolution within individuals is less studied. To investigate, we analyzed NM‐MRI scans from the Parkinson's Progression Markers Initiative (PPMI) database (www.ppmi‐info.org).

**Methods:** We processed longitudinal NM‐MRIs from 382 participants (168 PD patients, 214 prodromal). Scans from each visit were rigidly aligned and averaged. We used an Attention U‐Net model to automate segmentation of the SN and a reference region in the crus cerebri, and calculated the contrast ratio (CR), contrast‐to‐noise ratio (CNR), and volume. Analyses were conducted with and without thresholding.

**Results:** 114 PD patients exhibited a reduced CR, while 54 showed increases between the first and last scans (1.4 years mean duration). A weaker trend was observed in the prodromal group (126 decreased vs. 88 increased). No significant changes were detected in CNR nor volume across thresholds (0, 1.5, 3.0), with these measures displaying more variability. With a linear curve fit, we estimated a CR loss of 2% per year in the patients (p=10^‐6^) and 0.7% in the prodromal cohort (p=0.044).

**Figure:**
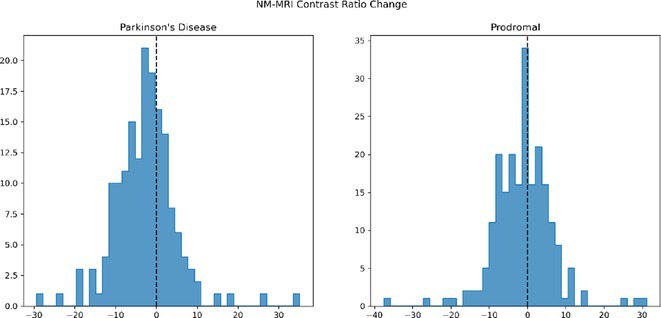
**FIGURE 1** Percent change in CR between first and last measurements in prodromal and diagnosed Parkinson's disease.

**Conclusion:** The CR of the SN to crus cerebri offers a greater longitudinal stability than CNR and volume, and may prove useful for monitoring individual neurodegeneration on a relatively short timescale. The higher rate of CR loss in PD patients supports an accelerated neurodegeneration post‐diagnosis than in the prodromal stage.

**Disclosure:** Nothing to declare

## EPO‐132

### 7T 3D‐MR spectroscopic imaging reveals tissue‐ and lesion‐specific oxidative stress in multiple sclerosis

#### R. Rumbak ^1^; E. Niess^1^; A. Dal‐Bianco^2^; F. Niess^1^; B. Strasser^1^; L. Hingerl^1^; A. Kloss‐Brandstätter^3^; G. Grabner^4^; T. Berger^2^; W. Bogner^1^; P. Rommer^2^

##### ^1^High‐field MR Center, Department of Biomedical Imaging and Image‐guided Therapy, Medical University of Vienna, Vienna, Austria; ^2^Department of Neurology, Medical University of Vienna, Vienna, Austria; ^3^Department of Engineering & IT, Carinthia University of Applied Sciences, Villach, Austria; ^4^Department of Medical Engineering, Carinthia University of Applied Sciences, Klagenfurt, Austria

**Background and aims:** Oxidative stress is pivotal in Multiple Sclerosis (MS) pathogenesis, yet its in vivo mechanisms remain underexplored. Glutathione (GSH), the primary antioxidant countering oxidative stress, is difficult to measure due to low concentration and imaging limitations. Utilizing advanced 7T spectroscopy, we examined lesion‐ and tissue‐specific oxidative stress, highlighting GSH's potential as a biomarker to enhance MS diagnostics and therapy.

**Methods:** This study included 18 MS patients (8F/10M, 47 ± 11 years) and 12 controls (6F/6M, 31 ± 7 years). Lesions were categorized as subcortical, juxtacortical, leukocortical, and intracortical using MP2RAGE and FLAIR. Segmented lesion masks were dilated and subtracted from WM/GM CSI masks, while control masks were derived directly. GSH and metabolites were quantified via Echo‐less 3D MRSI and expressed as tCr ratios. Student's t‐tests compared lesions to tissue types, and Pearson correlations assessed metabolite relationships for each lesion type with p‐values adjusted for multiple comparisons.

**Figure:**
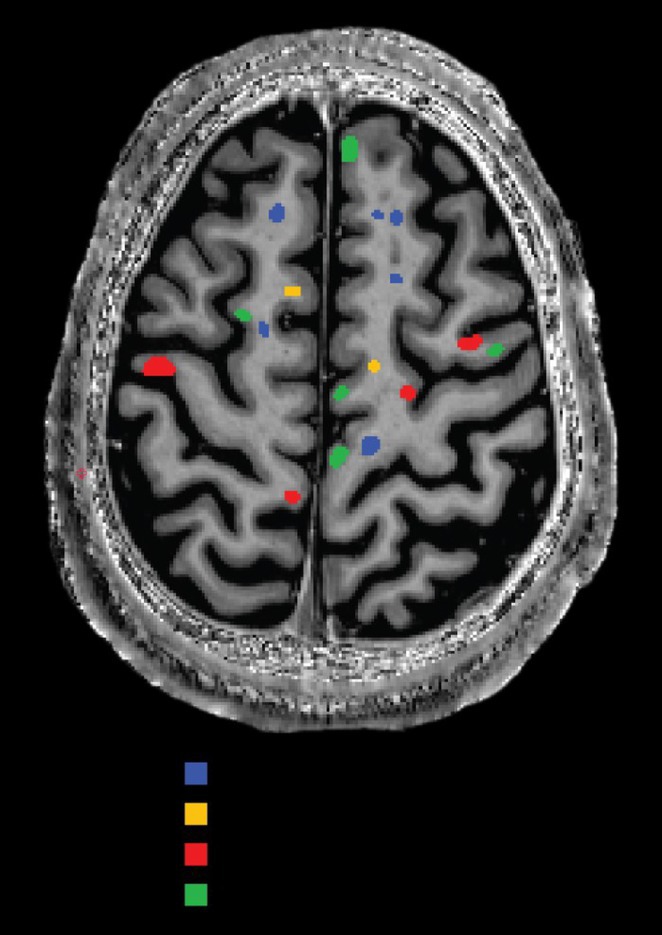
**FIGURE 1** Segmentation and categorization of lesions. Color‐coded regions represent subcortical (blue), juxtacortical (yellow), leukocortical (red), and intracortical (green) lesions on MP2RAGE.

**Results:** GSH/tCr was higher and Ins/tCr lower in all cortex‐proximal lesions than surrounding tissue. Glu/tCr was elevated in subcortical lesions but lower in other types. Juxtacortical lesions showed positive correlations between tCho/tCr and GSH/tCr, and NAA/tCr and Ins/tCr. Glu/tCr positively correlated with NAA/tCr in leukocortical and intracortical lesions.

**Figure:**
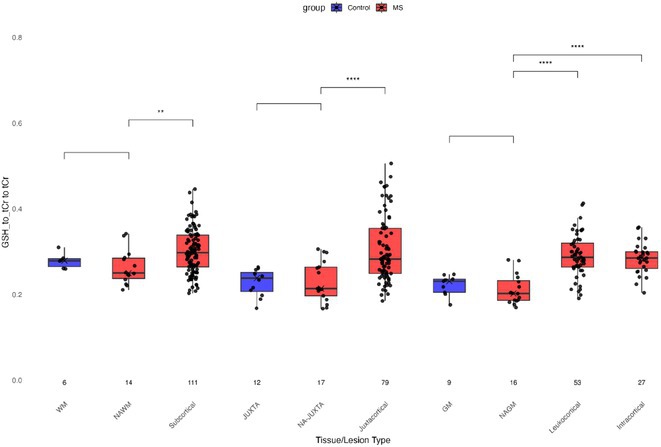
**FIGURE 2** GSH/tCr ratios across tissue and lesion types. Significantly elevated GSH/tCr levels in lesions (red) compared to healthy tissues (blue) and normal appearing tissue (red), highlighting oxidative stress patterns. (**p < 0.01; ****p < 0.0001)

**Figure:**
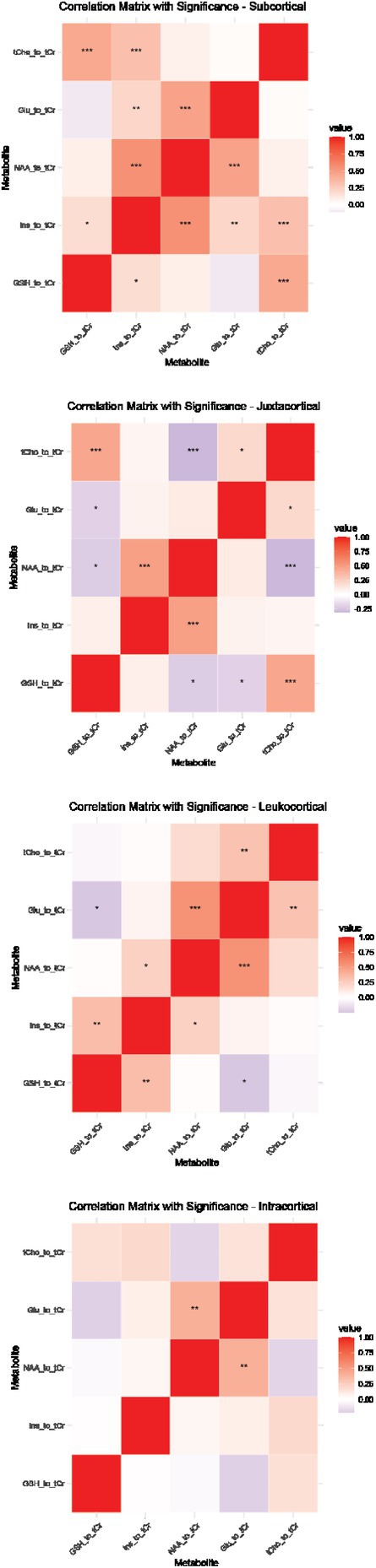
**FIGURE 3** Correlation matrices with significance for tCho/tCr, Glu/tCr, NAA/tCr, Ins/tCr, and GSH/tCr ratios across subcortical, juxtacortical, leukocortical, and intracortical lesions. Significant correlations are marked (*p < 0.05, **p < 0.01, ***p < 0.001).

**Conclusion:** These findings are the first to reveal metabolic alterations and correlations, including altered GSH levels in cortex‐proximal lesions, utilizing advanced 7T 3D echo‐less MRSI, providing unprecedented spatial insights into oxidative stress mechanisms in MS.

**Disclosure:** This research was funded in whole, or in part, by the Austrian Science Fund (FWF):[10.55776/DFH50].

## EPO‐133

### H/M ratio cut‐offs from standardized MIBG scintigraphy for diagnosing Parkinson's disease and related disorders

#### S. Taguchi ^1^; H. Suzuki^2^; T. Nakura^1^; H. Saiki^1^; M. Doyu^2^

##### ^1^Parkinson's Disease Advanced Therapy Center, Aichi Medical University Hospital, Nagakute, Japan; ^2^Neurology

**Background and aims:** ¹^23^I‐Metaiodobenzylguanidine (MIBG) myocardial scintigraphy, included in the global diagnostic criteria for Parkinson's disease (PD), previously relied on non‐standardized data. This study evaluates the diagnostic accuracy of standardized analysis for distinguishing PD from related disorders. We also aim to establish optimal heart‐to‐mediastinum (H/M) ratio cut‐offs for PD diagnosis using standardized methods.

**Methods:** We enrolled 268 parkinsonism patients (122 males, median age 71 years [Inter‐quartile range: IQR 61–77], median disease duration 2 years [IQR 1–6]), diagnosed based on international standard criteria. ¹^23^I‐MIBG myocardial scintigraphy was performed, and H/M ratios were calculated using standardized protocols.

**Results:** Among 268 patients, 202 had PD and 66 had non‐PD (Multiple system atrophy 24, Progressive supranuclear palsy 34, Corticobasal syndrome 8). H/M ratios in PD were lower in both early (median 1.90 [IQR 1.65–2.34]) and late phases (median 1.60 [IQR 1.36–2.08]) vs. non‐PD (early: 2.90 [IQR 2.34–3.32]; late: 3.13 [IQR 2.29–3.57]) (p < 0.001, respectively; Kruskal‐Wallis test). Receiver operating characteristic (ROC) analysis identified early‐phase cut‐off 2.24 (specificity 0.81, sensitivity 0.70, AUC 0.81) and late‐phase cut‐off 1.90 (specificity 0.81, sensitivity 0.70, AUC 0.83).

**Conclusion:** Our standardized analysis demonstrated a lower sensitivity for PD diagnosis compared to conventional methods. This finding is likely due to the predominance of early‐stage PD in our cohort, where minimal cardiac sympathetic nerve damage is expected due to early Lewy pathology. Future studies should focus on cases with a relatively longer disease duration.

**Disclosure:** Nothing to disclose.

## EPO‐134

### Cerebral amyloid angiopathy prevalence in Alzheimer's disease according to different Boston criteria versions

#### V. De Franco ^1^; F. Mazzacane^2^; E. Prodi^3^; C. Imbimbo^2^; M. Malik^2^; M. Cotta Ramusino^1^; G. Perini^1^; A. Costa^1^; A. Pichiecchio^3^

##### ^1^Unit of Behavioral Neurology and Center for Cognitive Disorders and Dementias (CDCD), IRCCS Mondino Foundation, Pavia, Italy; ^2^Department of Brain and Behavioral Sciences, University of Pavia, Pavia, Italy; ^3^Department of Neuroradiology, Advanced Imaging and Radiomics Center, IRCCS Mondino Foundation, Pavia, Italy

**Background and aims:** Cerebral amyloid angiopathy (CAA) commonly coexist with Alzheimer's disease (AD), exacerbating cognitive deterioration and complicating treatment strategies. Boston 2.0 criteria have shown increased sensitivity for CAA diagnosis, but few studies have explored the impact of their application in AD patients. The aim of our study was to assess the prevalence of CAA in a cohort of typical AD patients, according to the different versions of the Boston criteria.

**Methods:** A retrospective cohort of 108 consecutive AD patients, diagnosed using the Albert/McKhann criteria with biomarker support, was analyzed. Patients were included if an adequate MRI scan was available to assess hemorrhagic and non‐hemorrhagic [centrum semiovale perivascular spaces (csPVS); and multispot white matter (WMH) hyperintensities pattern] CAA radiological biomarkers.

**Results:** The prevalence of CAA (possible and probable) rose from 35% to 79% using the Boston v2.0 criteria. The prevalence of probable CAA increased from 19% to 21% and 32%, with v1.0, v1.5 and v2.0 criteria respectively. Transitioning from v1.5 to v2.0 criteria, the reclassification was due to the presence of severe csPVS in 8 (13.3%) cases, WMH multisport pattern in 35 (57.4%) cases and both markers in 18 (29.5%) cases.

**Figure:**
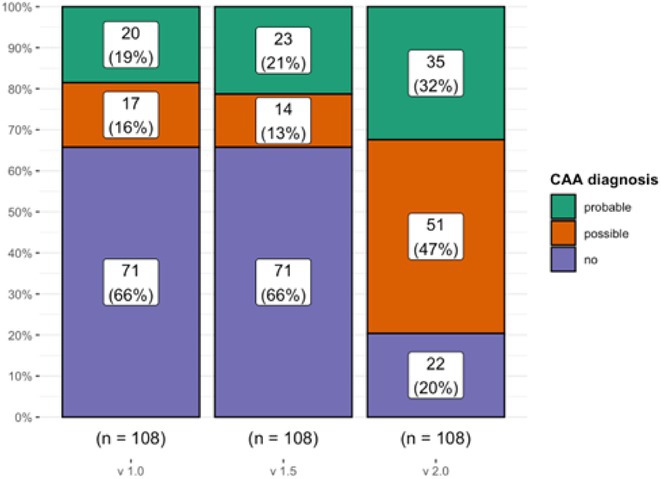
**FIGURE 1** CAA prevalence in our population using the different versions of Boston criteria.

**Figure:**
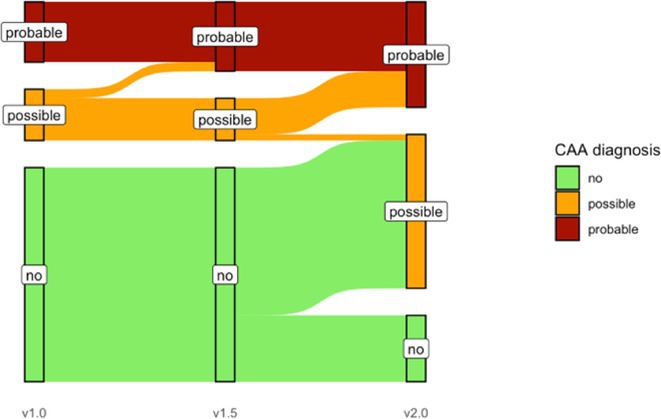
**FIGURE 2** Sankey diagram showing the change in the diagnosis transition between different versions of Boston criteria.

**Conclusion:** The Boston 2.0 criteria significantly increased the prevalence of CAA diagnosis in AD patients, but the clinical significance regarding prognosis and treatment decisions deserve further studies, as their specificity in non‐hemorrhagic patients remains uncertain. A careful interpretation of neuroradiologica data is essential to tailor the therapeutic choices to the patient, especially in relation to the use of anticoagulants and anti‐amyloid immunotherapy.

**Disclosure:** Nothing to disclose.

## Neuropathies

## EPO‐135

### Oligoclonal bands in blood and cerebrospinal fluid of patients with immune‐mediated neuropathies

#### A. Sekulic ^1^; I. Bozovic^2^; A. Palibrk^2^; M. Jankovic^2^; S. Peric^2^; J. Drulovic^1^; I. Basta^1^

##### ^1^Faculty of Medicine, University of Belgrade, Serbia; ^2^Neurology Clinic, University Clinical Centre of Serbia, Serbia

**Background and aims:** Albuminocytological dissociation is usually seen in patients with Guillain Barre syndrome (GBS) and chronic inflammatory demyelinating polyradiculoneuropathy (CIDP). Data on immunoglobulin oligoclonal bands (OCBs) are scarce. The aim was to analyze the presence of OCBs in cerebrospinal fluid (CSF) and serum of patients with GBS and CIDP.

**Methods:** During a ten‐year old period, 344 patients were primarily diagnosed with GBS among whom 213 (62%) had OCB analysis ‐ 64% males, age 53±16 years, GBS disability scale (GDS) at nadir 3.4±1.1. During the same period, 169 patients were diagnosed with CIDP among whom 125 (74%) had OCB analysis ‐ 72% males, age at onset 56±15 years, duration 18±27 months, mean INCAT 3.6±2.1.

**Results:** Eighteen (8%) GBS patients had CSF OCBs ‐ two had only CSF bands (one later developed CIDP and one connective tissue disease (CTD)), one had CSF bands and a smaller number of serum bands (diagnosed with Lyme disease), and 15 patients had parallel bands (two had CTD, two systemic vasculitis, one Hodgkin lymphoma, one monoclonal gammopathy, and one Lyme disease). Thirteen (10%) CIDP patients had OCB ‐ five only CSF bands (one previously had meningoencephalitis and one was diagnosed with multiple sclerosis), one CSF bands and a smaller number of serum bands (with Sjogren's syndrome), and seven parallel bands (two had CTD and four paraprotein). Seven CIDP patients had parallel monoclonal bands due to paraprotein.

**Conclusion:** CSF oligoclonal bands are not common in immune‐mediated neuropathies and if found, further analysis should be performed to seek for other diseases.

**Disclosure:** Nothing to disclose.

## EPO‐136

### Neuroleukemeiosis presenting similar to AIDP

#### A. Elsaddig

##### Neurology Department, Salford Royal Hospital, Manchester, England, UK

**Background and aims:** Neuroleukemeiosis, a rare condition where leukemic cells infiltrate peripheral nerves. We present a case which mimicked Acute Inflammatory Demyelinating Polyneuropathy (AIDP).

**Methods:** Case presentation of a 33‐year‐old male diagnosed with Acute Myeloid Leukemia (AML) in May 2024, undergoing chemotherapy with cytarabine, daunorubicin, and midostaurin. In July 2024, he developed left facial nerve palsy following a diarrheal illness, progressing to severe neurological symptoms, including shoulder and back pain, ascending paraesthesia, bilateral lower facial weakness, diplopia, bulbar dysfunction, paraparesis of upper limbs and asymmetrical lower limb weakness. Neurological examination revealed multiple cranial nerve palsies, including bilateral lower facial nerve palsy, right oculomotor nerve palsy with pupillary involvement, bulbar speech, tongue immobility, and global areflexia. Motor deficits showed severe upper limb paralysis and asymmetrical lower limb weakness. Sensory impairments included diminished vibration and proprioception.

**Results:** Initial cerebrospinal fluid (CSF) analysis on day 7 showed normal findings, while nerve conduction studies on day 14 confirmed non‐length‐dependent acquired demyelinating polyneuropathy. MRI of the lumbosacral spine revealed cauda equina thickening and enhancement, MR brain imaging ruled out cranial nerve enhancement or leptomeningeal pathology. Despite five days of intravenous immunoglobulin (IVIG) therapy, symptoms worsened. Repeat CSF flow cytometry confirmed leukemic infiltration (99% CD33‐positive cells). Oncological treatment escalated but unfortunately patient didn’t survive.

**Figure:**
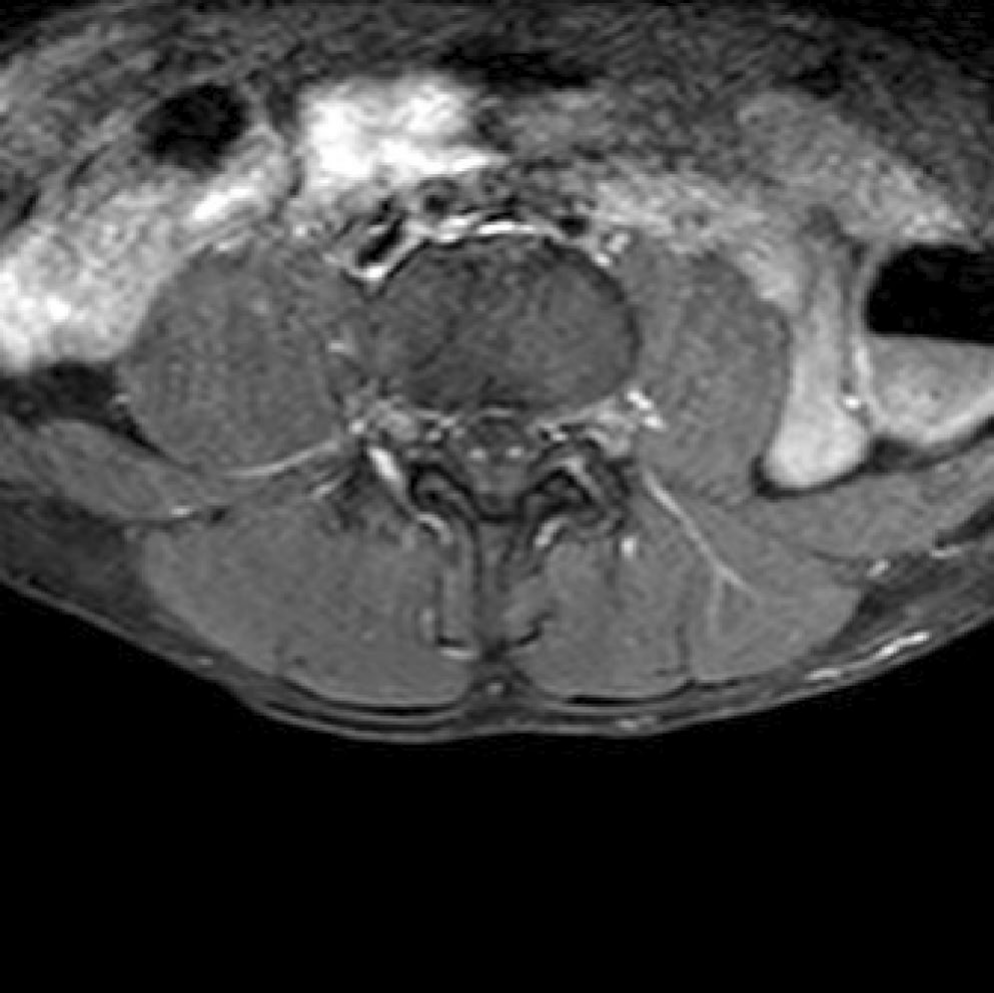
**FIGURE 1** Enhanced thickened lumbosacral nerve roots on MR lumbosacral spine scan with GAD.

**Figure:**
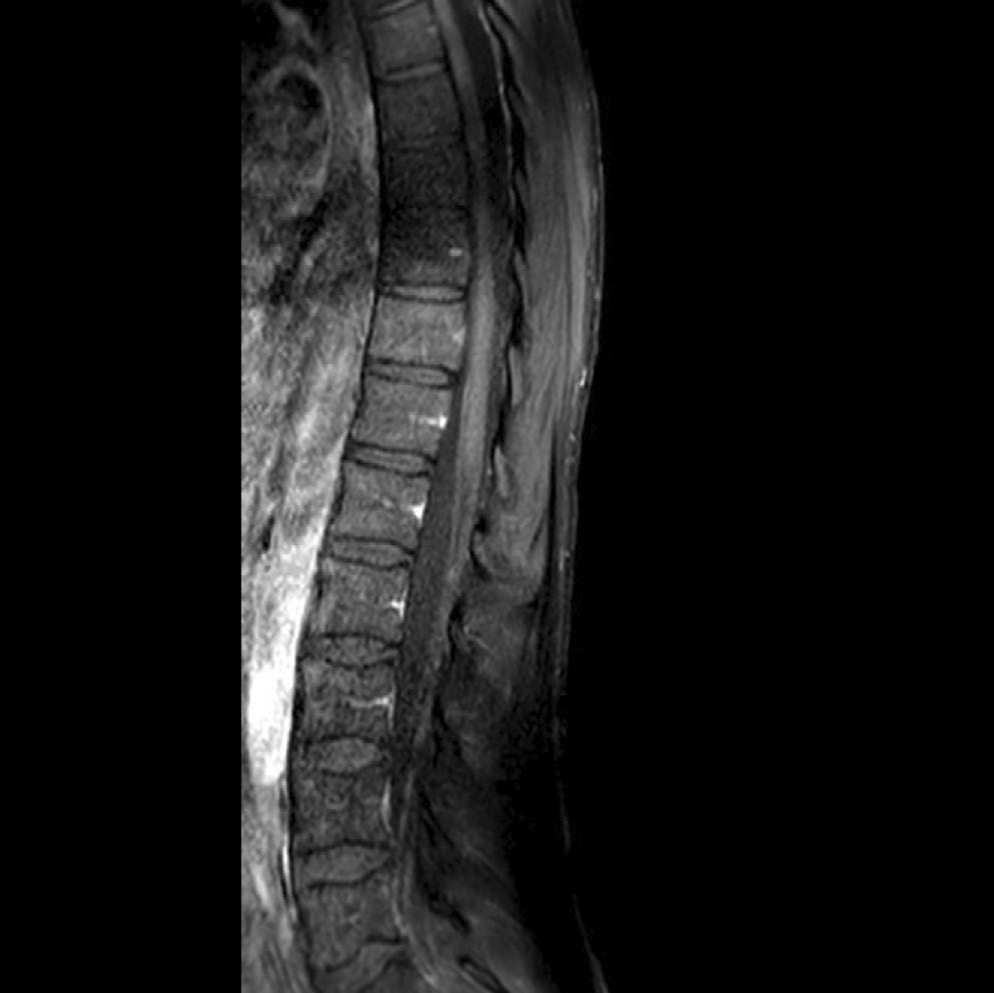
**FIGURE 2** Thickened enhancing cauda equina nerve roots on MR lumbosacral spine scan with contrast.

**Conclusion:** This case highlights the rarity of neuroleukemeiosis and its potential to mimic AIDP. Key differentiators include asymmetrical weakness, worsening clinical state despite treatment, and imaging findings. Prompt recognition and diagnostic differentiation are crucial for appropriate management.

**Disclosure:** nothing to disclose.

## EPO‐137

### Rapidly progressive treatment refractory CIDP neuropathy, think of Paranodal neuropathy

#### A. Elsaddig

##### Neurology Department, Salford Royal Hospital, Manchester, England, UK

**Background and aims:** Paranodal neuropathies are a group of inflammatory neuropathies that are due to antibodies targeting nodal and paranodal antibodies in peripheral nerves. They share several clinical features to AIDP and CIDP, but with additional autonomic and systemic features. Here we present a case of neurofascin 155 IgG4 paranodal neuropathy

**Methods:** Case presentation of a 57 years old female who over a 4‐month period started having pins and needles in hands and feet, that ascended upwards, associated with ataxia, loss of dexterity in upper limbs and weakness in lower limbs requiring a use of wheelchair, significant loss of weight and constipation. Bedside examination showed bilateral subclinical facial muscle weakness, neck flexion weakness, and symmetrical proximal and distal weakness in 4 limbs, associated with global areflexia, impaired vibration up to iliac spine and impaired proprioception up to knees.

**Results:** Investigation supported a demyelinating inflammatory polyneuropathy, which included sural sparing pattern, significant reduced conduction velocity and prolonged distal motor and f waves. Lumbar puncture showed elevated CSF protein with normal white cells. Given the additional autonomic and systemic features plus facial weakness and significantly raised protein, paranodal antibodies panel sent and showed positive IgG4 for NF155.

**Figure:**
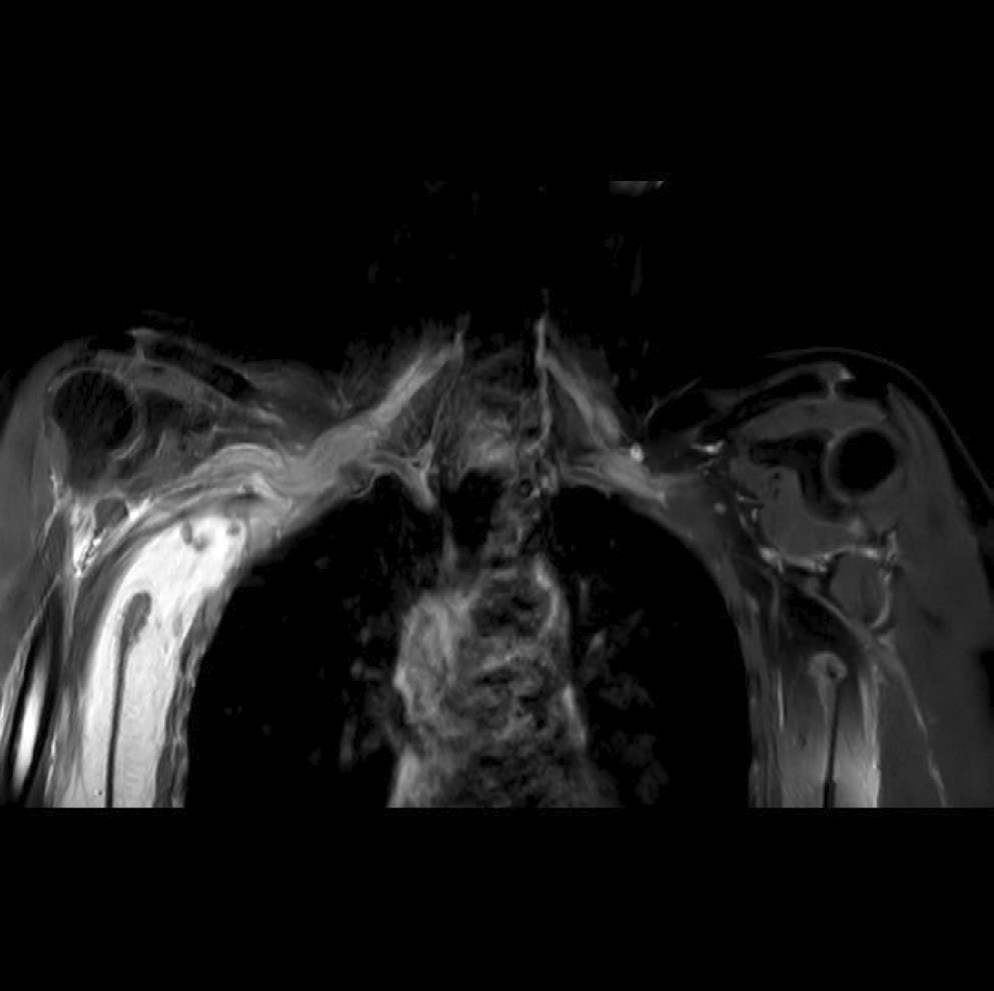
**FIGURE 1** Brachial plexus with contrast showing avid enhancement and thickening of brachial plexus.

**Figure:**
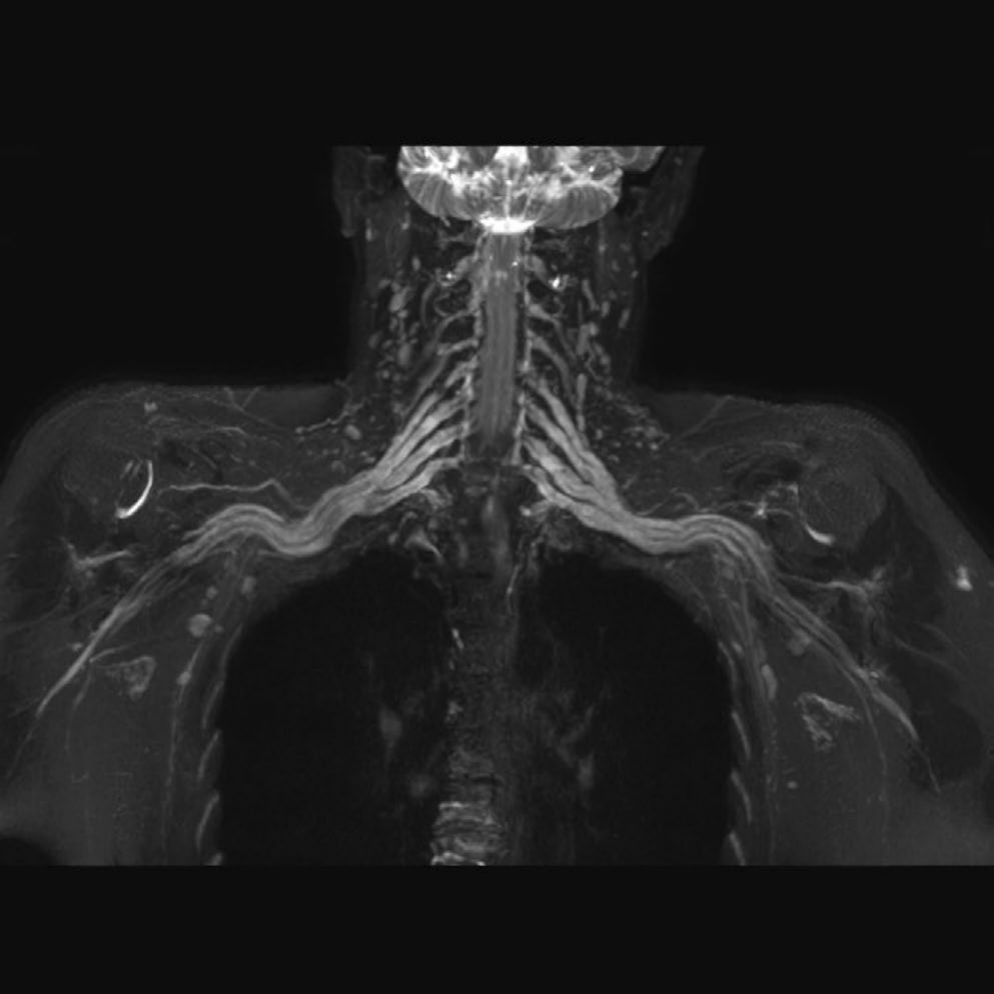
**FIGURE 2** Coronal reconstructed MIP MPR MRi scan showing thickening of brachial plexus.

**Figure:**
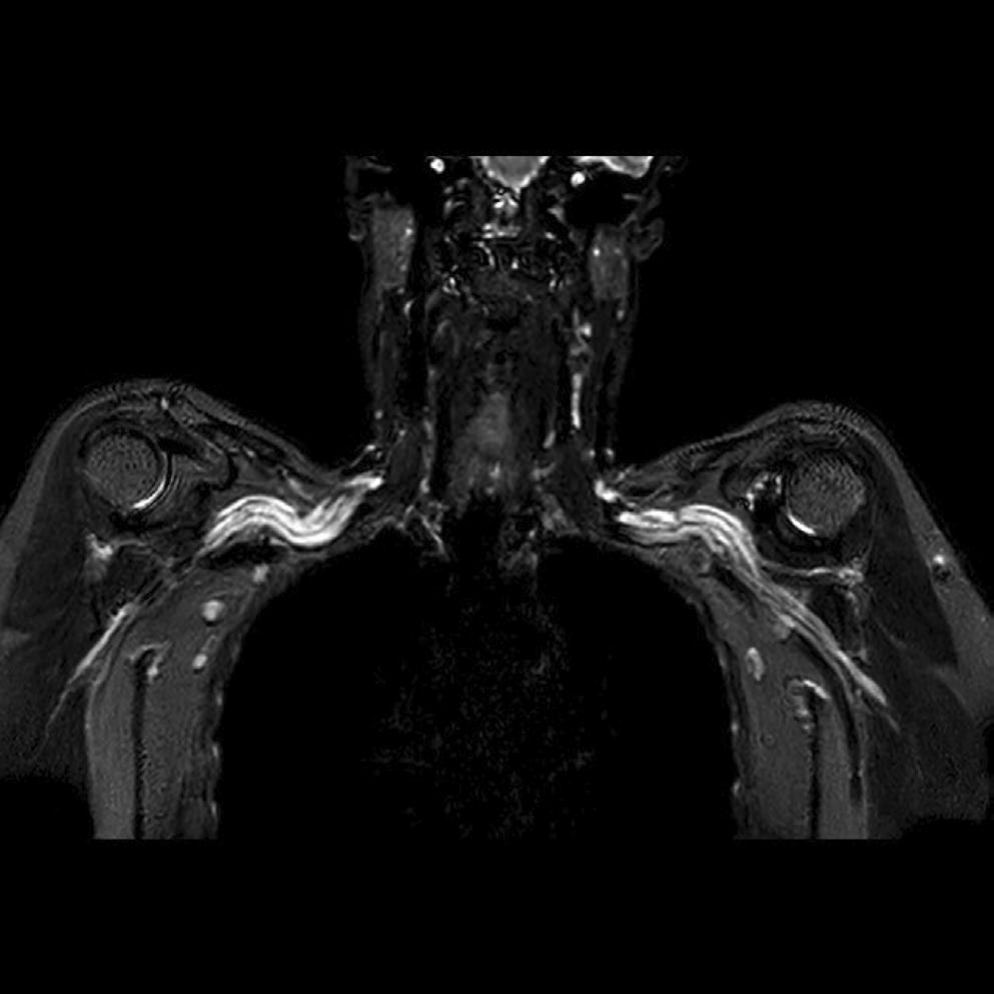
**FIGURE 3** High signal of brachial plexus on STIR sequence.

**Conclusion:** Rapidly progressive symmetrical “CIDP” polyneuropathy with cranial, autonomic and systemic features with suboptimal response to IvIg should raise suspicion of paranodal neuropathies which respond better to B cell depleting therapy (Rituximab) and plasma exchange.

**Disclosure:** nothing to disclose.

## EPO‐138

### A case of ANCA‐associated vasculitis mimicking lumbar disc herniation

#### T. Eyigurbuz^1^; B. Ozberk Pamuk ^2^; E. Coban^1^; N. Kale^1^

##### ^1^Department of Neurology, Bagcilar Training and Research Hospital, Istanbul, Turkey; ^2^Department of Neurology, Samsun Training and Research Hospital, Samsun, Turkey

**Background and aims:** ANCA‐associated vasculitides are autoimmune disorders characterized by small‐vessel inflammation, often leading to systemic manifestations and peripheral neuropathy. Although vasculitic neuropathy typically presents as mononeuritis multiplex, asymmetric sensorimotor polyneuropathy can also occur.

**Methods:** A 50‐year‐old male presented with severe right lower extremity pain and foot drop. Lumbar disc herniation was initially suspected, and a discectomy was performed, but symptoms persisted. Within a month, he developed night sweats, weight loss, and progressive limb weakness. Physical examination revealed maculopapular erythematous lesions in the pretibial region. Laboratory tests, including C‐reactive protein (CRP), erythrocyte sedimentation rate (ESR), and proteinase 3 (PR3)‐ANCA, were performed. Imaging studies, such as cranial and spinal magnetic resonance imaging (MRI), and cerebrospinal fluid (CSF) analysis were conducted. Electromyography (EMG) was performed, and a sural nerve biopsy was taken for confirmation.

**Results:** Laboratory tests showed elevated CRP and ESR with positive PR3‐ANCA. MRI and CSF analysis were unremarkable. EMG revealed axonal polyneuropathy affecting motor and sensory fibers. Sural nerve biopsy confirmed vasculitis. The patient initially received 1g intravenous steroid therapy but showed no significant clinical improvement. Subsequently, treatment with intravenous immunoglobulin (IVIg) and cyclophosphamide led to a marked reduction in inflammatory markers and significant relief of neuropathic pain.

**Figure:**
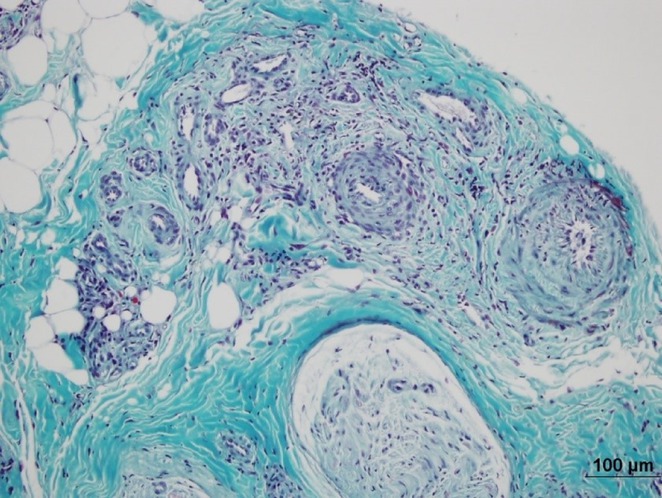
**FIGURE 1** The sural nerve biopsy stained with modified Gomori's trichrome (MGT) shows perivascular inflammation.

**Conclusion:** This case highlights the need to consider vasculitis in the differential diagnosis of acute‐subacute neuropathies. Early recognition and immunosuppressive therapy are essential to prevent irreversible nerve damage.

**Disclosure:** Nothing to disclose.

## EPO‐140

### Immunoglobulin vs efgartigimod: Indirect comparisons in chronic inflammatory demyelinating polyneuropathy (CIDP)

#### D. Cornblath^1^; J. Wilson^2^; M. Baquié ^3^; C. Wissmann^3^; E. Clodi^4^

##### ^1^Department of Neurology, Johns Hopkins University, Baltimore, USA; ^2^Numerus Ltd, Wokingham, UK; ^3^Octapharma AG, Lachen, Switzerland; ^4^Octapharma PPG, Vienna, Austria

**Background and aims:** As no relevant head‐to‐head randomized control trials (RCTs) have been published in CIDP, matching‐adjusted indirect comparisons (MAIC) were conducted to evaluate the efficacy of Panzyga® intravenous immunoglobulin (IVIg) maintenance therapy versus efgartigimod subcutaneous neonatal Fc receptor (FcRn) inhibiting therapy in patients with CIDP.

**Methods:** Individual patient data (IPD) from the 1.0g/kg arm (N=69) and 2.0g/kg arm (N=36) IVIg phase 3 ProCID trial (NCT02638207) were respectively weighted to match aggregate baseline characteristics of patients from Stage A of subcutaneous efgartigimod PH20 phase 2 trial ADHERE (N=322; NCT04281472). ProCID patients who achieved confirmed Evidence of Clinical Improvement (ECI) were weighted (in both arms respectively) to match patients in the efgartigimod arm at the start of ADHERE Stage B (N=111). Based on clinically important baseline characteristics (age and sex), patients were matched. Weighted estimates from ProCID were compared (unanchored MAIC) against reported estimates in ADHERE for various clinical outcomes.

**Results:** A MAIC compared outcomes between ProCID patients and those in ADHERE's Stage A treatment period: Confirmed ECI (treatment responders), change from baseline in adjusted Inflammatory Neuropathy Cause and Treatment (aINCAT) score, Inflammatory Rasch built Overall Disability Scale (I‐RODS) score, grip strength (dominant and non‐dominant hands), and Medical Research Council (MRC) sum score. Another MAIC assessed deterioration following confirmed ECI in both ProCID treatment arms with efgartigimod patients in ADHERE Stage B respectively. Detailed results will be presented at the conference.

**Conclusion:** Although head‐to‐head RCTs are the reference, MAICs represent valuable comparative evidence for evaluating the growing treatment options for CIDP.

**Disclosure:** David R Cornblath (Consultant: Annexon Biosciences, Avilar Therapeutics, Boehringer Ingelheim, Dianthus Therapeutics, Grifols S.A., Hansa Medical AB, Johnson & Johnson, Nuvig Pharma, Octapharma AG, Pfizer, Inc; Data Safety Monitoring Board: Avidity Bio, Passage Bio, Sanofi, Vertex; Technology Licensing: Worldwide Clinical Trials, Inc., Beijing 3E‐Regenacy Pharmaceuticals Co., Ltd., Passage Bio, CMIC, MedImmune Ltd., Fundacion GELTAMO, RWS Life Sciences; Scientific Advisory Board: Algotherapeutics, Nervosave) Jason C. Wilson (employee: Numerus Ltd, service provider: Octapharma AG) Mathurin Baquié (employee: Octapharma AG) Christoph Wissmann (employee: Octapharma AG) Elisabeth Clodi (employee: Octapharma PPG)

## EPO‐141

### The effects of efgartigimod therapy on autoimmune nodopathy

#### H. Tai; S. Niu; Z. Zhang

##### Beijing Tiantan Hospital, Capital Medical University

**Background and aims:** Autoimmune nodopathy is a polyneuropathy characterized by IgG antibodies targeting the nodo‐paranodal region. Efgartigimod, an FcRn inhibitor lowering IgG and pathological autoantibodies, offers potential treatment. This study assessed efgartigimod's efficacy and safety in autoimmune nodopathy.

**Methods:** Three patients with autoimmune nodopathy (identified nodo‐paranodal antibodies: NF155, CNTN1, NF186, Caspr1; IgG subtypes determined) participated in this open‐label pilot study. Disease severity was assessed using aINCAT, MRC sum score, and I‐RODS scores. Efgartigimod (10 mg/kg) was administered in four weekly infusions. Weekly clinical evaluations were conducted during and after treatment.

**Results:** Treatment was well‐tolerated. Patient 1 (anti‐NF186 IgG3) showed significant improvement after three doses, with sustained benefit but relapse after three weeks. Patient 2 (anti‐NF155 IgG4) showed moderate improvement after four doses, with sustained benefit. Patient 3 (anti‐NF155 IgG1/IgG4) showed improvement with efgartigimod and prior steroid treatment, with sustained benefit. Response varied based on antibody target and subtype.

**Conclusion:** Efgartigimod showed efficacy and safety in treating autoimmune nodopathy, suggesting a promising novel therapy. However, response variability highlights the need for further investigation into the influence of specific antibody targets and subclasses.

**Disclosure:** There are no disclosures to report.

## EPO‐142

### Multifocal predominant motor neuropathy in tangier disease: A case report

#### J. Alves ^1^; C. Bernardes^1^; A. Ramalho^2^; S. Moreira^2^; L. Almendra^1^; A. Matos^1^

##### ^1^Neurology Department, Coimbra University Hospital, Coimbra Local Health Unit, Coimbra, Portugal; ^2^Internal Medicine Department, Coimbra University Hospital, Coimbra Local Health Unit, Coimbra, Portugal

**Background and aims:** Tangier Disease (TD) is a rare genetic disorder caused by mutations in the ABCA1 gene, resulting in low HDL levels and cholesterol buildup. Peripheral neuropathy occurs in about 50% of cases with heterogeneous clinical and electrophysiological patterns, from multifocal neuropathy, syringomyelia‐like neuropathy, and, less commonly, distal symmetric polyneuropathy.

**Methods:** Case report.

**Results:** A 49‐year‐old male was referred to neurology appointment due to two months of paraesthesia and progressive loss of dexterity in his right upper limb. His medical history included smoking, excessive alcohol use, hypertension, type 2 diabetes, psoriasis and bilateral hip arthroplasty at age 35. Since 47, he has been monitored for extremely low HDL cholesterol (HDL <3 mg/dL) and severe hypertriglyceridemia (644 mg/dL), unresponsive to fenofibrate. Follow‐up revealed hepatosplenomegaly and pancytopenia. At 48, he had a myocardial infarction. There was no family history of lipid abnormalities or early‐onset vascular disease. Neurological examination revealed weakness grade 3/5 in right wrist extension and 4/5 in fingers abduction and dysesthesia in the right median nerve distribution. Nerve conduction studies suggested an acquired demyelinating predominantly motor neuropathy with motor conduction blocks and temporal dispersion in the median and fibular nerves bilaterally and right ulnar nerve at non‐compressible sites, and absent right median nerve SNAP. Genetic testing identified a ABCA1 gene variant (c.164A>G p.(His55Arg)) of uncertain significance, raising suspicion of Tangier disease.

**Conclusion:** Tangier Disease should be considered in patients with demyelinating multifocal motor or sensory‐motor neuropathy and severe lipid abnormalities. This case highlights the importance of a multidisciplinary approach, for accurate diagnosis and management.

**Disclosure:** Nothing to disclose.

## EPO‐143

### When to consider CANVAS: A tertiary center's perspective on diagnostic clues

#### M. Almeida; L. Silva; A. Alferes; A. Jorge; A. Geraldo; L. Almendra; J. Lemos; A. Matos

##### Department of Neurology, Coimbra University Hospitals, Coimbra, Portugal

**Background and aims:** Cerebellar Ataxia with neuropathy and bilateral vestibular areflexia syndrome (CANVAS) is a recently described syndrome that has become the focus of growing current interest, frequently cited as the most common cause of recessive genetic ataxia worldwide. The typical features are chronic progressive gait unsteadiness, eye movement abnormalities, including loss of vestibulo‐ocular reflex (VOR), and polyneuropathy. Our goal was to characterize the clinical and paraclinical findings in 17 genetically confirmed CANVAS patients.

**Methods:** We retrospectively included patients tested for CANVAS in our tertiary center. Data retrieved from patients’ records including demografic, symptoms, neurological assessment as well as EMG and neuro‐ophtalmological evaluation and brain MRI findings were further analyzed.

**Results:** From a total of 38 patients tested for CANVAS, 17 were positive. Our CANVAS patients present a mean age of 66.76 years‐old (SD 6.33). Most have experienced imbalance for about a decade, with this symptom being universal followed by oscillopsia. Downbeat nystagmus was present in 82% of patients and horizontal VOR was severely impaired in 75 % of patients. About 94% present the diagnostic triad, while the remaining did not have vestibular areflexia. All patients had abnormal SNAPs, which were absent in 82% of our sample. CMAPs were universally preserved. Clinical polyneuropathy was reported in 82% of the patients. Cerebellar atrophy was visible in 50% of patients.

**Conclusion:** Our results globally align with the literature. Clinical assessment alone may be sufficient to strongly suspect of CANVAS for an up‐to‐date clinician.

**Disclosure:** Nothing to disclose.

## EPO‐144

### Effect of efgartigimod PH20 SC on lower limb function in chronic inflammatory demyelinating polyneuropathy in ADHERE

#### S. Rinaldi^1^; J. Allen^2^; J. Lin^3^; M. Stettner ^4^; I. Merkies^5^; J. Guptill^6^; C. Hewamadduma^7^; G. Istas^8^; A. De Roeck^8^; S. Kuwabara^9^; G. Lauria^10^; L. Querol^11^; N. Suresh^12^; C. Karam^13^; T. Skripuletz^14^; A. Echaniz‐Laguna^15^; B. Van Hoorick^8^; R. Yamasaki^16^; R. Lewis^17^; P. van Doorn^18^

##### ^1^Nuffield Department of Clinical Neurosciences, University of Oxford, Oxford, UK; ^2^Department of Neurology, University of Minnesota, Minneapolis, USA; ^3^Department of Neurology, Huashan Hospital, Fudan University, Shanghai, China; ^4^Department of Neurology and Center for Translational Neuro‐ and Behavioral Sciences (C‐TNBS), University Medicine Essen, Essen, Germany; ^5^Curaçao Medical Center, Willemstad, Curaçao; ^6^Department of Neurology, School of Medicine, Duke University, Durham, USA; argenx, Ghent, Belgium; ^7^Sheffield Institute for Translational Neurosciences (SITRAN), University of Sheffield, Sheffield, UK; Sheffield Teaching Hospitals NHS Foundation Trust, Sheffield, UK, ^8^argenx, Ghent, Belgium; ^9^Department of Neurology, Graduate School of Medicine, Chiba University, Chiba, Japan, ^10^IRCCS Fondazione Istituto Neurologico Carlo Besta, Milan, Italy; Department of Medical Biotechnology and Translational Medicine, University of Milan, Milan, Italy, ^11^Department of Neurology, Neuromuscular Diseases Unit, Hospital de La Santa Creu I Sant Pau, Universitat Autònoma de Barcelona, Barcelona, Spain; Centro De Investigación Biomédica en Red en Enfermedades Raras (CIBERER), Madrid, Spain, ^12^Lakeland Regional Health, Lakeland, USA, ^13^Department of Neurology, University of Pennsylvania, Philadelphia, USA, ^14^Department of Neurology, Hannover Medical School, Hanover, Germany, ^15^French National Reference Center for Rare Neuropathies (CERAMIC), Bicêtre University Hospital, Public Hospital Network of Paris, Le Kremlin‐Bicêtre, France, ^16^Department of Neurology, Kyushu University Hospital, Fukuoka, Japan, ^17^Department of Neurology, Cedars‐Sinai Medical Center, Los Angeles, USA, ^18^Department of Neurology, Erasmus University Medical Center, Rotterdam, The Netherlands

**Background and aims:** In the ADHERE trial (NCT04281472), subcutaneous (SC) efgartigimod PH20 (coformulated with recombinant human hyaluronidase PH20), a neonatal Fc inhibitor, reduced relapse risk and improved disability scores in chronic inflammatory demyelinating polyneuropathy (CIDP). This post hoc analysis explores the effect of efgartigimod PH20 SC on lower limb function.

**Methods:** Participants had active CIDP and were off treatment or on standard treatments (withdrawn during ≤12‐week run‐in). Participants received weekly efgartigimod PH20 SC 1000 mg (stage A). Responders were randomised (1:1) to weekly efgartigimod PH20 SC 1000 mg or placebo for ≤48 weeks (stage B). Outcomes included changes from run‐in baseline (after participants withdrew prior treatments) to stage B last assessment in adjusted Inflammatory Neuropathy Cause and Treatment (aINCAT) leg score, selected Inflammatory Rasch‐Built Overall Disability Scale (I‐RODS) individual items, and Timed Up and Go (TUG) test.

**Results:** 322 participants entered stage A; 221 were randomised and treated in stage B (efgartigimod PH20 SC: 111, placebo: 110). Tables 1 and 2, respectively, report participants who improved to an INCAT leg score of 0 or 1, and ≥1 points in I‐RODS individual items at stage B last assessment. At stage B last assessment, mean (SE) TUG test completion time (seconds) in efgartigimod‐treated participants decreased by −2.6 (0.66) from 16.5 (1.32) at run‐in baseline, while in placebo‐treated participants it decreased by −1.4 (1.48) from 19.9 (3.33).

**Figure:**
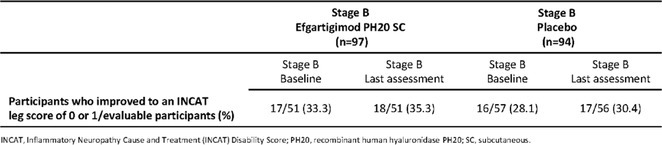
**TABLE 1** Percentage of participants who had an INCAT leg score of ≥2 at run‐in baseline and improved to a score of 0 or 1.

**Figure:**
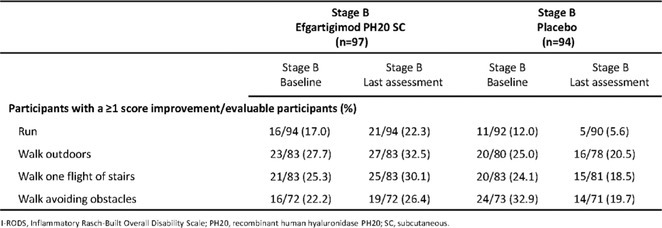
**TABLE 2** Percentage of participants who had a score of 0 or 1 in selected individual I‐RODS items at run‐in baseline and improved ≥1 points.

**Conclusion:** Numerically more efgartigimod PH20 SC–treated participants with CIDP in ADHERE stage B experienced improvements from run‐in baseline in lower limb function compared with placebo‐treated participants.

**Disclosure:** As the disclosures of all the authors included in this abstract exceed 1500 characters, disclosures will be provided to the congress so they can be accurately reflected in the Congress Abstract book.

## EPO‐145

### Microvascular decompression for hyperactive dysfunction syndrome: A single‐centre experience

#### M. Segura‐Lozano ^1^; C. Castillo‐Rangel^2^; O. Carranza‐Rentería^1^; A. Munguía‐Rodríguez^1^

##### ^1^Neurología Segura. Hospital Angeles Morelia. México; ^2^ISSTE 1ro de Octubre, CDMX, México

**Background and aims:** Hyperactive dysfunction syndromes (HDS) arise from vascular compression at the root entry/exit zone of cranial nerves. Commonly encountered neuropathies include trigeminal neuralgia (TN) in its classical (CTN), secondary (STN), or idiopathic (ITN) forms, hemifacial spasm (HFS), and glossopharyngeal neuralgia (GPN). Microvascular decompression (MVD) addresses the root cause of HDS and is considered an effective surgical treatment.

**Methods:** We retrospectively analyzed medical and surgical records of 1378 patients with HDS who underwent MVD at our center between 2014 and 2024.

**Results:** Between January 2014 and December 2024, 3624 patients with HDS were evaluated, of whom 1378 underwent MVD. Among operated patients TN was manifested in 1199 (87%), HFS in 83 (6%) and GPN in 17 (1.2%). Combined HDS was observed in 79 (5.7%) patients. The mean age of symptom onset was 47.8 years for CTN, 45.3 for STN, 37.4 for ITN, 47.3 for HFS, and 53.2 for GPN. Female predominance was observed across all HDS types, with ITN showing the highest female ratio. Right‐sided involvement was most frequent with ITN having the highest bilateral occurrence. Transient postoperative complications occurred in 21%, predominantly CSF leaks (13%) and facial paralysis (2%). No perioperative deaths were reported. Recurrence was observed in 5.8% over follow‐ups ranging from 1 month to 10 years.

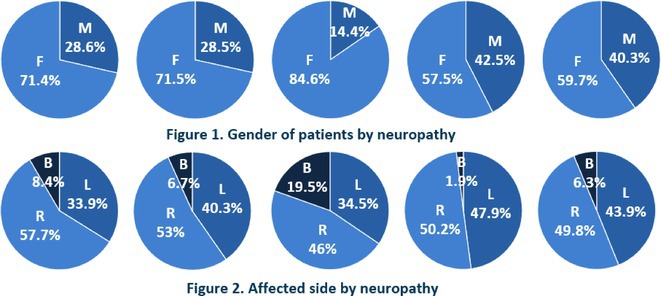

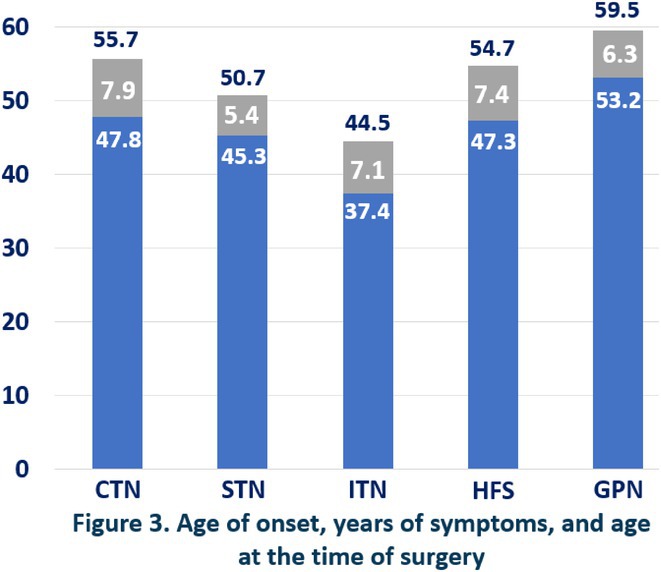

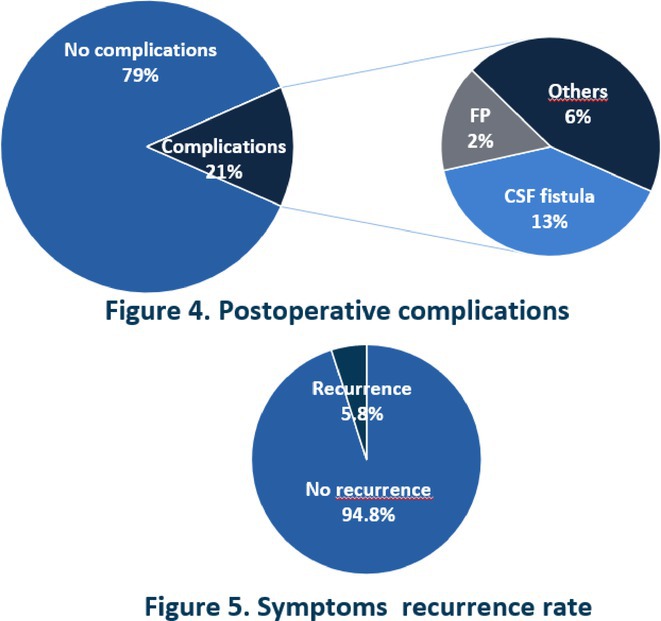

**Conclusion:** MVD is a safe and effective surgical treatment for HDS, offering significant symptom relief with minimal complications. This series, among the largest reported in Latin America, demonstrates outcomes consistent with international standards.

**Disclosure:** Nothing to disclose.

## EPO‐146

### Developing a blood‐based biomarker targeting alpha‐synuclein fragments for the early diagnosis of PD

#### M. Benmarce ^1,2^; K. Henriksen^1,3^; S. Nielsen^1^; M. Karsdal^1^

##### ^1^Nordic Bioscience, Herlev, Denmark; ^2^Technical University of Denmark, Lyngby, Denmark; ^3^Department. of Molecular and Medical Biology, Roskilde University Center, Roskilde, Denmark

**Background and aims:** Developing a sensitive immunoassay that detects alpha‐synuclein fragments cleaved by calpain I in peripheral blood for the early diagnosis of Parkinson's Disease (PD). It has been previously established that these fragments contribute to the formation of aggregates, which are associated with the early onset of the disease.

**Methods:** An antibody was generated to specifically target alpha‐synuclein fragments cleaved by calpain I. A competitive ELISA was developed to analyze serum samples from clinical PD cohorts with a mean age of 64.2 years. These cohorts exhibited hypokinesia, postural instability, muscle rigidity, and tremor, having been diagnosed for one and a half years. Additionally, the SH‐SY5Y neuroblastoma cell model was used to bridge brain pathology to peripheral biomakers and further validate the immunoassay.

**Results:** The developed antibody demonstrated specificity for α‐synuclein fragments. A competitive ELISA was developed and validated for measurements in serum, it can significantly distinguish between healthy and PD serum samples. Moreover, alpha ‐synuclein fragments were detected in the supernatant of apoptotic SH‐SY5Y cells.

**Conclusions:** Alpha‐Synuclein fragments cleaved by calpain I represent key early drivers of PD pathology. This blood‐based biomarker holds promise for early diagnosis and may provide crucial insights into patient eligibility for targeted therapeutic interventions in PD.

**Disclosure:** I work and own shares at Nordic Bioscience.

## EPO‐147

### Polyradiculoneuropathies associated with immune checkpoint inhibitors: Are we facing a new nosological entity?

#### M. Trimboli

##### Department of Neurology, AOU Renato Dulbecco, Catanzaro, Italy

**Background and aims:** Immune checkpoint inhibitors (ICIs) are increasingly used to treat advanced cancers. While they enhance survival, ICIs can cause immune‐related adverse events (irAEs) affecting the peripheral nervous system, notably acute inflammatory demyelinating polyneuropathy (AIDP) and chronic inflammatory demyelinating polyneuropathy (CIDP). Early diagnosis is challenging, leading to potential misclassification and suboptimal management.

**Methods:** A 48‐year‐old woman with melanoma, undergoing pembrolizumab therapy, developed progressive lower limb weakness, sensory disturbances, and areflexia after two cycles of treatment. Neurological evaluation suggested AIDP, and she was treated with intravenous immunoglobulin (IVIg), leading to initial improvement. However, 100 days later, the patient experienced a relapse with widespread weakness in all limbs, prompting a re‐evaluation and reclassification of her condition as acute‐onset CIDP (A‐CIDP). This case highlights the difficulty in distinguishing between AIDP and A‐CIDP, especially when considering the nuances of ICI‐related neuropathies.

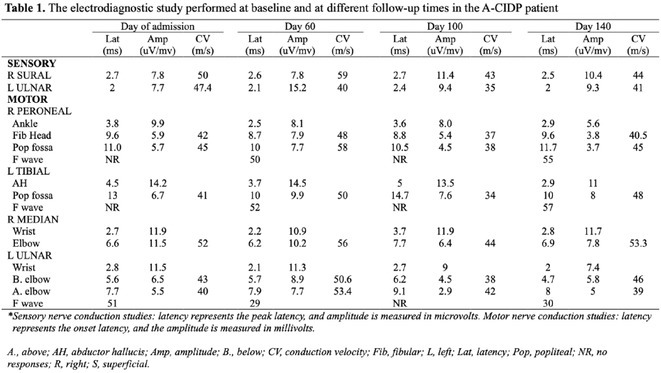

**Results:** A review identified 51 AIDP and 10 CIDP cases linked to ICI therapy. Symptoms included weakness, paresthesia, and gait instability, with NCS often showing demyelination. Most improved with steroids and/or IVIg, though some AIDP cases progressed to A‐CIDP, suggesting potential misdiagnosis in ICI‐induced neuropathies.

**Conclusion:** This case underscores the diagnostic challenges of ICI‐related neuropathies, particularly A‐CIDP, which may mimic AIDP. The immune dysregulation induced by ICIs often leads to atypical, aggressive presentations. Early cessation of ICIs and timely immunosuppressive therapy are crucial to prevent lasting disability. Recognizing A‐CIDP enables better outcomes through personalized treatment and proactive monitoring.

**Disclosure:** Nothing to disclose.

## EPO‐148

### Femoral Nerve Injury causes – Retrospective study

#### S. Palma; C. Alves; P. Pereira

##### Almada‐Seixal Local Health Unit, Almada, Portugal

**Background and aims:** According literature, femoral nerve mononeuropathy is iatrogenic in 60% of cases. It is considered a rare condition, and its incidence may be underestimated. Electroneuromyography (ENMG) is frequently used in this clinical context. This study aims to determine the main risk factors, etiologies, and clinical course of this mononeuropathy in patients referred for electrophysiological evaluation.

**Methods:** Patients diagnosed with femoral mononeuropathy from the ENMG database between January 2020‐July 2024, were included.

**Results:** 12 patients were included (58.3% male; median age of 61 years), referred for ENMG due to suspected femoral mononeuropathy. All had unilateral symptoms. Three main etiological mechanisms were identified: peri‐surgical stretching, with four cases associated with hip prosthesis procedures and four cases with intra‐abdominal surgeries; compressive, with three cases due to retroperitoneal hematoma in hospitalized patients on low molecular weight heparin (LMWH) anticoagulation; and direct trauma, with one case caused by a stab injury. Follow‐up data were available for 75% of cases, of which 88.8% underwent physiotherapy. Despite symptomatic improvement, 66.6% of patients continued to experience symptoms. One case achieved complete recovery.

**Conclusion:** Medical‐surgical iatrogenesis is the most common cause of femoral nerve mononeuropathy, accounting for 91.6% of cases in this cohort, with surgical causes being the most frequent. This highlights the importance of surgical awareness to minimize its occurrence. The presence of symptoms consistent with femoral nerve injury in patients on anticoagulation, particularly LMWH, should raise suspicion of retroperitoneal hematoma. Early diagnosis could allow timely suspension of anticoagulation (when possible), which may significantly impact prognosis.

**Disclosure:** Nothing to disclose.

## EPO‐149

### A Delphi panel to identify optimal outcome measures in chronic inflammatory demyelinating polyneuropathy (CIDP)

#### Y. Rajabally ^1^; G. Boggia^2^; D. Riley^3^; S. Riley^3^; J. Peatman^3^; M. Mangelaars^4^; C. Gary^5^; C. Fix^6^; E. Nobile‐Orazio^7^

##### ^1^Aston University, Birmingham, UK; ^2^Janssen‐Cilag, a Johnson & Johnson company, Italy; ^3^Adelphi Values PROVE™, Bollington, Cheshire, UK; ^4^Janssen‐Cilag BV, a Johnson & Johnson company, Netherlands; ^5^Janssen‐Cilag, a Johnson & Johnson company, France; ^6^GBS/CIDP Foundation International, USA; ^7^IRCCS Humanitas Research Hospital, University of Milan, Italy

**Background and aims:** CIDP is a rare, heterogenous, autoimmune polyradiculoneuropathy causing disability. Current clinical outcome assessments (COAs) may not capture all relevant aspects of the disease experience.

**Methods:** A multi‐stakeholder modified Delphi study is ongoing to identify optimal COAs for CIDP, involving 18 health care providers (HCPs) and 18 patients from nine European countries. Preliminary results from the first‐round survey from 13 HCPs and 10 patients are presented; full results are anticipated in 2025.

**Results:** Preliminary first‐round results from 13 HCPs highlighted mobility, gait/balance, muscle/grip strength, functional independence, pain, mood, and fatigue as key domains for assessment. Ten patients recognised these as important, together with quality of life (QoL), sleep, and cognition. Pain management was prioritised by most patients (n=9/10) and was also deemed highly important by HCPs (n=11/13). Additionally, HCPs (n=10/13) noted time constraints and subjectivity as barriers to pain assessment, while patients (n=5/10) noted that pain relief could ameliorate other outcomes such as fatigue. HCPs (n=8/13) noted the visual analogue scale is frequently or always used in clinical practice, while patients (n=6/10) raised concerns regarding the use of scales to quantify the extent of their pain highlighting the need for more adapted assessment tools.

**Conclusion:** Given the clinical heterogeneity of CIDP, identifying a core set of COAs is essential. Preliminary findings suggest that beyond current outcomes valued by clinicians, several other elements are of relevance to patients. Multi‐stakeholder alignment is needed to refine and optimise existing tools to reflect the full impact of CIDP on patients’ lives from both HCP‐and patient‐centric perspectives.

**Disclosure:** The study was funded by Johnson and Johnson Innovative Medicine, which provided Adelphi Values PROVE with funding for the review; GMB, CG, and MM are Johnson and Johnson employees and may hold stock or stock options. YAR has received speaker/consultancy honoraria from LFB, Polyneuron, Argenx, Takeda, Grifols, Janssen, Sanofi, Dianthus, has received educational sponsorships from LFB and CSL Behring and has obtained research grants from LFB. EN‐O has received speaker/consultancy honoraria from Argenx, Takeda, CSL Behring, Dianthus, Janssen, Kedrion, LFB, Roche and has received a research grant from Takeda. CF is a GBS/CIDP Foundation International employee.

## Peripheral Nerve Disorders

## EPO‐150

### HyperCPKemia in Guillain–Barré syndrome: A cohort study

#### A. Kumar ^1^; N. Lall^2^; D. Joshi^1^; V. Singh^1^; A. Pathak^1^; R. Chaurasia^1^; V. Mishra^1^; J. Chaurasia^1^

##### ^1^Department of Neurology, Institute of Medical Sciences, Banaras Hindu University, Varanasi India; ^2^Radiotherapy & Radiation Medicine, Institute of Medical Sciences, Banaras Hindu University, Varanasi, India

**Background and aims:** Elevated creatine kinase (CK) levels have been observed in some Guillain‐Barré syndrome (GBS) patients, particularly those with the acute motor axonal neuropathy (AMAN) subtype. This may indicate muscle damage and could be a marker of disease severity in specific GBS subtypes. Further research is needed to comprehend the implications fully.

**Methods:** We prospectively enrolled 106 patients with AIDP, AMAN, AMSAN, and unclassified groups whose serum CK levels were measured during their stay in the hospital between April 2022 and December 2024. Patients were classified into four different subtypes of GBS, based on nerve conduction studies, and further sub‐classified into normal CK (serum CK ≤ 26‐140 U/L) and CK elevation (serum CK ≥140 U/L) groups, respectively. The clinical features were compared among these groups.

**Results:** Out of 106 patients, 39 (36.8%), 55 (51.8%), and 12 (11.3%) were of AIDP, AMAN, AMSAN, and unclassified subtypes, respectively. Clinical characteristics were similar among normal CK (n=63) and CK elevation (n=43) groups. In our study, patients in the CK elevation group had autonomic dysfunctions. Among the two groups, the frequency of CK elevation was significantly higher (p ≤ 0.05) in the AMAN subtype of GBS as evaluated by MRC sum (23.4 ± 13.6 & 32.3 ±12.6) from admission to discharge, respectively.

**Conclusion:** CK elevation in AMAN subtype of GBS was associated with autonomic dysfunctions, potentially indicating disease severity in specific subtypes. Therefore, increased CK levels within the first four weeks of symptom onset may be a marker for axonal degeneration and poor prognosis in GBS.

**Disclosure:** Nothing to disclose.

## EPO‐151

### Pan‐neurofascin nodo‐paranodopathy presenting as fulminant Guillain‐Barré Syndrome – case report and literature review

#### A. Cabral; C. Mota; S. Casanova; A. Campos; J. Ribeiro; M. Malaquias

##### Neurology Department, Local Health Unit of Gaia Espinho, Porto, Portugal

**Background and aims:** Autoimmune nodo‐paranodopathy (AINP) associated with pan‐neurofascin antibodies (Ab‐PanNF) is a rare subtype of autoimmune neuropathy that can present as a severe, prolonged, and sometimes fatal illness. However, timely recognition and appropriate treatment can lead to full or near‐complete recovery, often with a monophasic course. Rituximab (RTX) is emerging as the most effective treatment, capable of inducing remission.

**Methods:** We present a single case report and a literature review on pan‐neurofascin nodo‐paranodopathy.

**Results:** We describe a patient initially diagnosed with Guillain‐Barré Syndrome (GBS) who experienced partial recovery before worsening abruptly, developing tetraplegia, lower cranial nerve involvement, and dysautonomia. A diagnosis of AINP with Ab‐PanNF was established, leading to RTX initiation and remarkable improvement. Our literature review summarizes 35 reported cases, highlighting key features of this novel entity and identifying red flags that should prompt early Ab‐PanNF testing. These include age >60 years, rapidly progressive and severe GBS‐like presentation, fulminant relapse after initial improvement, prolonged mechanical ventilation, and refractoriness to standard therapies.

**Conclusion:** Early recognition of AINP by neurologists and intensive care physicians is crucial, as prompt antibody‐depleting therapy can dramatically alter patient outcomes.

**Disclosure:** Nothing to disclose.

## EPO‐152

### Radiation induced vasculopathy leading to ischemic lumbar radiculopathy

#### A. Thet ^1^; A. Al‐Samak^2^; M. Thura^3^; S. Tun^1^; K. Lwin^3^

##### ^1^Department of Neurophysiology, Pinderfields Hospital, Wakefield, UK; ^2^Department of Neurophysiology, Harrogate District Hospital, Harrogate, UK; ^3^Department of Neurophysiology, Calderdale Royal Hospital, Halifax, UK

**Background and aims:** Delayed effects of radiation can be manifold. A case of radiation induced vasculopathy leading to ischemic radiculopathy is presented here. Objective: Radiation related vasculopathy and radiculo plexopathy are rare. The latency period between radiation exposure and the onset of symptoms ranged from 6 months to 44 years. The incidence of radiation‐induced lumbosacral plexopathy following pelvic radiotherapy ranged from 0.3% to 1.3%.

**Methods:** Case Report.

**Results:** 74‐year‐old lady had a year history of claudication symptoms in right leg. Right leg pain with walking was mentioned. While driving, she had to stop her car as sudden onset severed sharp pain appeared at the groin radiating to knee along the anterior thigh. Since then pins and needles sensation started from her right sole to the leg. She had right vulvar cancer treated with radiation 22 years earlier. Her right lower limb arterial pulses were not palpable including right femoral artery. Left side was normal. Right ankle jerk was absent. ABPI was 0.61. NCS were normal. EMG suggested the right L5/S1 radiculopathy. MRI of LS spine was unremarkable. MRA revealed moderate to severe stenosis of right common femoral artery while other vessels were patent.

**Conclusion:** A sudden onset intense groin pain limiting her functioning was followed by sensory symptoms in the distal lower limb. Patient had ischemic one lower limb symptoms. Primary cause of vasculopathy is linked to radiotherapy she received years ago. Spinal roots ischemia is suggested by sudden development of radiculopathy symptoms. It is a case of radiation vasculopathy leading to ischemic radiculopathy.

**Disclosure:** “Nothing to disclose.”

## EPO‐153

### Comparative analysis of paranodal antibody assays in autoimmune nodopathies: Accuracy and inter‐laboratory agreement

#### B. Rugginini ^1^; E. Vegezzi^2^; S. Masciocchi^3^; A. Jentzer^4^; E. Zardini^3^; C. Morandi^3^; S. Scaranzin^3^; F. Zuliani^3^; L. Benedetti^5^; J. Devaux^6^; M. Gastaldi^3^

##### ^1^Department of Brain and Behavioural Sciences, University of Pavia, Pavia, Italy; ^2^IRCCS Mondino Foundation, Pavia, Italy; ^3^Neuroimmunology Laboratory, IRCCS Mondino Foundation, Pavia, Italy; ^4^The Department of Immunology, CHU, Montpellier, France; ^5^San Martino Hospital, Istituto di Ricovero e Cura a Carattere Scientifico, Genoa, Italy; ^6^Institut de Génomique Fonctionnelle, Université de Montpellier, CNRS, INSERM, France

**Background and aims:** Autoimmune nodopathies (AN) are a subgroup of neuropathies that harbour antibodies targeting paranodal/nodal proteins (PNAbs): NF155, NF186, CNTN1 and CASPR1. However, laboratory strategies for their detection are not standardized. We aimed to evaluate the performances of PNAbs detection assays in two expert Neuroimmunology Centres.

**Methods:** A cohort of chronic inflammatory demyelinating polyneuropathy (CIDP) patients (n=45), pathological controls (n=34, 6 immune‐mediated neuropathies, 12 non‐immune‐mediated neuropathies, 16 normal pressure hydrocephalus), and healthy controls (n=10) were tested using a commercial CBA (CCBA) or in‐house assays: CBA on living cells (LCBA) and ELISA. PNAbs positivity required confirmation by both in‐house tests at either Centre. CIDP patients with PNAbs were considered AN and “true positives.” We assessed analytic performances using sensitivity, specificity and accuracy, and inter‐laboratory agreement using Fleiss' Kappa test with 95% confidence intervals (CI).

**Results:** In‐house assays detected PNAbs in 21 AN patients (NF155 = 11, PanNF =1, CNTN1 = 6, CASPR1 = 3), with an overall accuracy of 99.6% (sensitivity: 99.6%; specificity: 100%), with substantial agreement between Centres (Fleiss’ kappa: 0.668, 95% CI: 0.528 – 0.778). CCBA demonstrated comparable performance (accuracy: 98.4%; sensitivity: 92.8%; specificity: 98.8%). ELISA showed the lowest accuracy (95.5%; sensitivity: 97.6%; specificity: 95.4%). Substantial overall inter‐laboratory agreement for PNAbs (Fleiss’ kappa: 0.735, 95% CI: 0.635 – 0.817) was observed, with only 3/89 (3.4%) discordant samples. CCBA showed the highest agreement (0.914), while ELISA the lowest (0.712).

**Conclusion:** PNAbs assays have overall good performances and reproducibility. CCBAs represent a reliable alternative to in‐house assays for their detection.

**Disclosure:** Nothing to disclose.

## EPO‐154

### GBS at the Norfolk and Norwich Hospital (NNUH): A retrospective study regarding our practice and adherence to guidelines

#### D. Ibrahim; F. Olaoye; J. Dairo; M. Cao; O. Elkhayatt

##### Neurology department, Norfolk and Norwich University Hospital, Norwich, UK

**Background and aims:** Guillain‐Barre Syndrome (GBS) is an autoimmune polyradiculoneuritis causing ascending paralysis, sensory impairment and potential bulbar and autonomic dysfunction. Diagnosis is clinical and supported by CSF analysis and NCS/EMG. In the UK, the incidence is estimated at around 2/100,000/year, with 3‐7% mortality. National guidelines enforce early IVIg/PLEX treatment and close FVC monitoring. Our study aims to evaluate our department's performance in diagnosing and treating the condition according to the guidelines and identify areas requiring improvement.

**Methods:** A retrospective analysis was conducted on GBS patients admitted to NNUH (January 2019 ‐ July 2023). Data analysed included demographic details, Hughs score, CSF analysis, NCS/EMG and FVC.

**Results:** Thirty‐nine GBS patients were identified (M:F = 2:1, average age 59). 66% presented with typical ascending paralysis. Other presentations included ataxia, ophthalmoplegia, and bulbar palsy. Average Hughs score was 2.8 ‐ 4. IVIg were administered to 80% of patients (100% of patient requiring it due to Hughs > 2). Albuminocytologic dissociation was detected in 48.7% cases. Supportive NCS/EMG were found in 49% patients. 23% patients required ITU admission (FVC<1.5). Incidence was 0.89/100.000/year with 10% mortality.

**Conclusion:** NNUH showed very good guideline compliance regarding investigations, monitoring and escalation of GBS patients. All eligible cases received prompt immunotherapy and ventilatory support in ITU. CSF analysis and NCS/EMG were performed to support the diagnosis, reflecting good clinical practice. Our study showed lower incidence and higher mortality in our catchment area compared to the national average, suggesting underdiagnosis of milder cases which did not reach hospital attention.

**Disclosure:** Nothing to disclose.

## EPO‐155

### Electrophysiological evaluation of peripheral nerve injuries after the february 6 earthquake in Turkey

#### A. Yildirim^1^; E. Akbas ^1^; R. İnan^2^; T. Eyigurbuz^3^; İ. Guclu Altun^4^; B. Hosver^1^; T. Adatepe^3^

##### ^1^Neurology, İstanbul Medeniyet University, Göztepe City Hospital, İstanbul, Turkey; ^2^University of Health Sciences, Kartal Dr. Lütfi Kırdar City Hospital, Neurology Clinic; ^3^University of Health Sciences, Bağcılar Training and Research Hospital, Neurology Clinic; ^4^Çanakkale 18 Mart University, Faculty of Medicine, Department of Neurology

**Background and aims:** The February 6, 2023, earthquake in Turkey caused over 50,000 deaths and more than 100,000 injuries. This study aims to evaluate the peripheral nerve, plexus, and root injuries of earthquake‐affected patients in terms of EMG findings.

**Methods:** The clinical and EMG findings of patients trapped under rubble and admitted to three different centers after the earthquake were retrospectively evaluated.

**Results:** Among the 47 patients examined in the EMG laboratory, 19 were male and 28 were female. The average age of the patients undergoing EMG was 29.5 years, with the youngest being 6 years old and the oldest 69 years old. Seventeen of the patients were under the age of 18. Upper extremity injuries were found in 12 patients, lower extremity injuries in 34 patients, and both upper and lower extremity injuries in 1 patient. The average duration of being trapped under rubble was 29.5 hours. EMG examinations revealed peripheral nerve injury in 37 patients, plexus injury in 8 patients, root injury in 1 patient, and both plexus and peripheral nerve injury in 1 patient. The most common peripheral nerve injury was found in the peroneal nerve at a rate of 46%, followed by sciatic and tibial nerve injuries.

**Conclusion:** Severe peripheral nerve injuries were identified in earthquake survivors, causing significant disability. Long‐term follow‐up is necessary to assess nerve regeneration and the effectiveness of physiotherapy.

**Disclosure:** Nothing to disclose.

## EPO‐156

### Intraepineurial FF as a novel biomarker in TTR amyloidosis patients and asymptomatic carriers using MR neurography

#### E. Sole Cruz ^1^; E. Fortanier^1^; C. Michel^2^; M. Hostin^2^; E. Delmont^1^; D. Ben Dahan^2^; S. Attarian^1^

##### ^1^Reference Center for Neuromuscular Diseases and ALS, La Timone University Hospital, Aix‐Marseille University, Marseille, France; ^2^Center for Magnetic Resonance in Biology and Medicine, Aix‐Marseille University, Marseille, France

**Background and aims:** Transthyretin familial amyloid polyneuropathy (ATTRv‐PN) is a rare and progressive neurodegenerative disorder characterized by axonal neuropathy. Early detection of disease onset and progression is crucial for timely therapeutic intervention. Intra‐epineurial fat fraction (FF) could reflect lipid droplets in amyloid deposits or epineurial fatty‐rich replacement due to nerve fibers loss. This study investigates the utility of quantitative magnetic resonance imaging (qMRI) biomarkers, particularly intra‐epineurial FF, in differentiating ATTRv‐PN from asymptomatic carriers (ATTRv‐C) and healthy controls (HC).

**Methods:** 53 patients with TTR mutations, (31 symptomatic ATTRv‐PN, 22 ATTRv‐C), and 24 HC were included and imaged, Sciatic and tibial nerves were segmented on anatomical sequences. qMRI parameters including FF, MTR (Magnetization transfer ratio) and volume were computed from the sciatic and tibial masks to quantify nerve morphology, structural integrity, and intraepineurial fat fraction‐like. Correlations between qMRI metrics, clinical and electrophysiological parameters were calculated.

**Figure:**
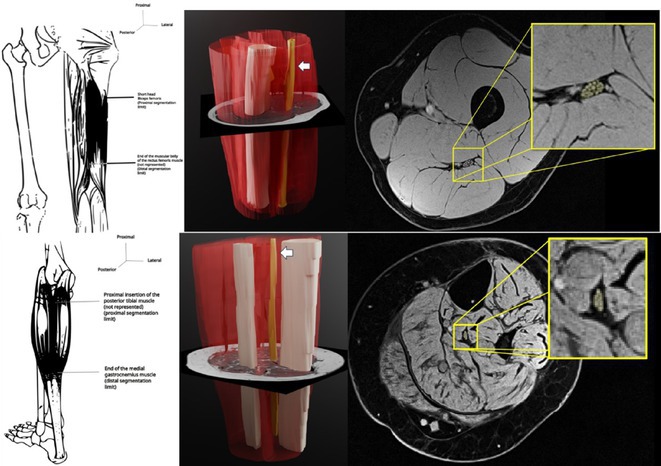
**FIGURE 1** Anatomical Landmarks for segmentation limits are shown in the left column. Sciatic nerve (upper row) and tibial nerve (lower row) are marked by a white arrow (3D reconstruction, central column) and marked in yellow (right column).

**Results:** Symptomatic ATTRv‐PN patients exhibited significantly higher intra‐epineurial FF in both sciatic (median value 32.4% IQR [24.4‐38.1]) and tibial nerves (median value 13.7%, IQR [9.97‐20.7]) compared to controls (sciatic median value 22.3%, IQR [16.6‐28.5]; tibial median value 9.74%, IQR [6.36‐12.5] respectively) (p<0.05). Intra‐epineurial FF values were positively correlated in both uni‐ and multivariate analysis with clinical and electrophysiological scores. ATTRv‐C also showed increased FF compared to controls (p<0.05). Additionally, MTR and nerve volumes exhibited less pronounced differences across groups.

**Figure:**
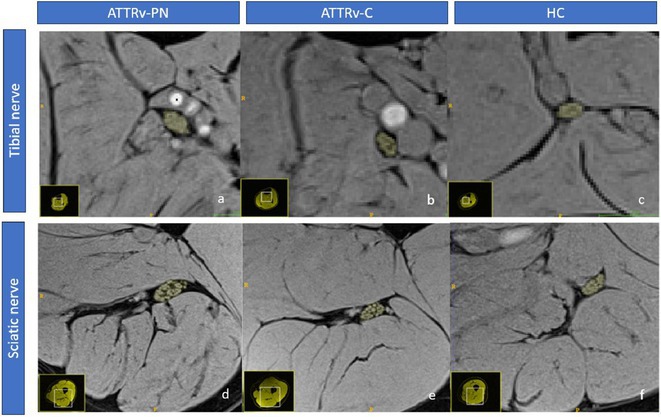
**FIGURE 2** Enlarged views of nerve ROIs. Tibial nerve ROI in a symptomatic ATTRv‐PN patient (a); ATTRv‐C patient (b) and healthy control (c); Sciatic nerve ROI in an ATTRv‐PN patient (d); ATTRv‐C patient (e) and healthy control (f).

**Figure:**
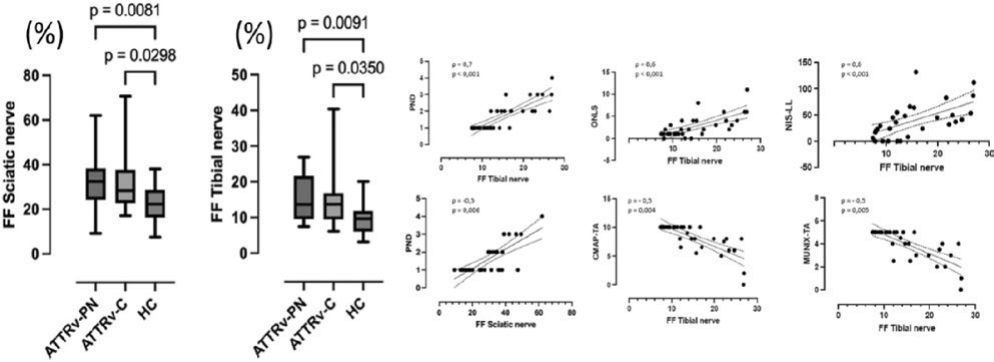
**FIGURE 3** represents Intra‐Epineurial FF differences between ATTRv‐PN, ATTRv‐C and HC, and correlations with clinical and electrophysiological scores

**Conclusion:** These results suggest that intra‐epineurial FF is a promising qMRI biomarker for identifying early changes in ATTRv‐PN, with potential for broader application in other axonal neuropathies.

**Disclosure:** Nothing to disclose.

## EPO‐157

### Diagnostic comparison of nerve ultrasound in immune‐mediated neuropathies

#### I. Glāzere ^1^; G. Ķauķe^1^; S. Mironovs^1^; V. Ķēniņa^3^

##### ^1^Pauls Stradins Clinical University Hospital, Department of Neurology, Riga, Latvia; ^3^Riga Stradins University, Faculty of Continuing Education, Riga, Latvia

**Background and aims:** Chronic immune‐mediated neuropathies represent a diverse group of peripheral nerve disorders with variable clinical phenotype and course, as well as response to treatment. Recognizing rare forms like anti‐myelin‐associated‐glycoprotein (MAG) neuropathy from chronic inflammatory demyelinating polyneuropathy (CIDP) would improve patient management and treatment strategy.

**Methods:** 4 CIDP and 4 anti‐MAG neuropathy patients were selected for detailed nerve ultrasound analysis, performed by single trained expert in nerve ultrasound. All patients were previously clinically evaluated, as well as other relevant data as treatment, duration of the disease and age at onset were documented.

**Results:** 8 male patients were enrolled ‐ 4 CIDP and 4 anti‐MAG neuropathy patients. For patient base line demographic and treatment data, see Table Nr 1. At the time of enrolment, the median age was 62 years (range 50‐79 years). The median age at onset for CIDP patients was 59 years, for anti‐MAG neuropathy patients – 52 years. Detailed nerve ultrasound results are represented in Table Nr 2.

**Figure:**
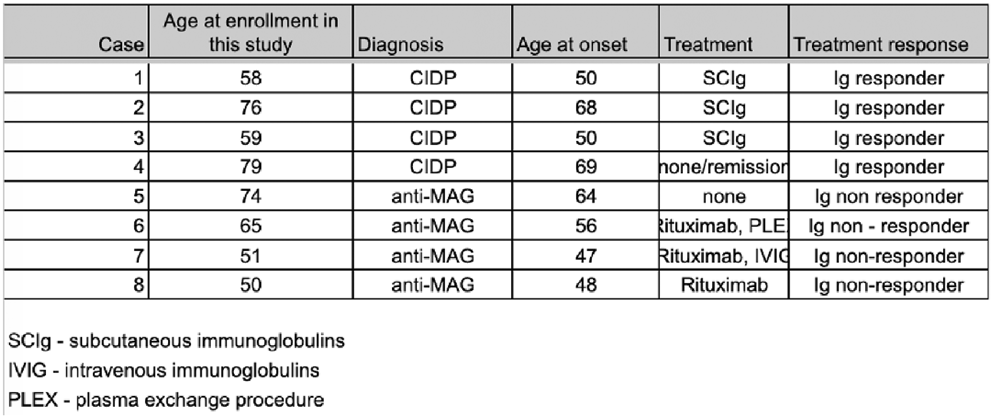
**TABLE 1** Patient demographic data and characteristics.

**Figure:**
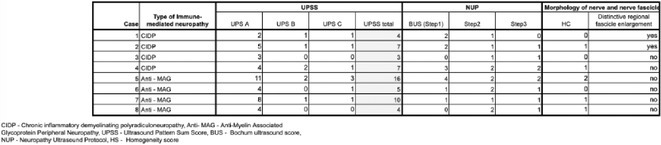
**TABLE 2** Comparison of ultrasound findings in CIDP and Anti‐MAG antibody neuropathy.

**Conclusion:** High resolution nerve ultrasound can be used as a complementary diagnostic tool to nerve conduction studies and serological findings to differentiate between hereditary, immune ‐ mediates and axonal polyneuropathies. Our small case study coincides with and complements the limited number of publications on the usefulness of nerve ultrasound for differentiation between CIDP and anti ‐ MAG antibody polyneuropathy. Anti ‐ MAG antibody neuropathy shows more homogenous nerve enlargement, tends to affect distal nerves more while in CIDP there are more noticeable fascicular changes with or without nerve enlargement.

**Disclosure:** Nothing to disclose.

## EPO‐158

### Immunoadsorption versus plasma exchange in Guillain‐Barre syndrome: A meta‐analysis and systematic review

#### L. Danganan; E. Montemayor; R. Dejan Jr.

##### East Avenue Medical Center

**Background and aims:** Guillain‐Barre Syndrome is a debilitating neurological disease with an incidence of 1.1 ‐ 1.8 per 100,000. Autoantibodies affecting peripheral nerve membranes play an important role in understanding the pathophysiology and treatment of it. Treatment options for its cure continue to unfold and evolve. Different clinical trials resulted in increased interest in therapeutic apheresis for treatment of severe and refractory disease. Conflicting results of immunoadsorption compared to plasma exchange in the management of Guillain‐Barre Syndrome led us to synthesize available evidence from published studies.

**Methods:** Review Manager software was used for this review and classified the outcomes into primary (curative effect) and secondary (safety profile and relapse rate). Quality assessment and statistical data analysis were conducted using the said software.

**Figure:**
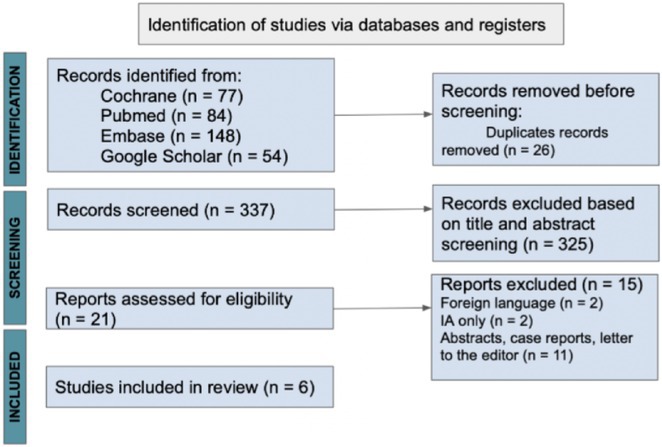
**FIGURE 1** Flowchart of the systematic literature search on four electronic databases according to the PRISMA guidelines.

**Results:** The odds of achieving at least one grade disability and functional improvement was similar for patients treated with immunoadsorption and plasma exchange (OR: 0.77; 95% CI: 0.34 ‐ 1.74; p = 0.53). Reduced risk of complications for patients treated with immunoadsorption group as compared to plasma exchange group (RR: 0.69; 95% CI: 0.43 ‐ 1.11; p = 0.13) was noted. Increased risk of relapse for patients who underwent immunoadsorption (RR: 1.70; 95% CI: 0.96 ‐ 3.00; p = 0.07).

**Figure:**
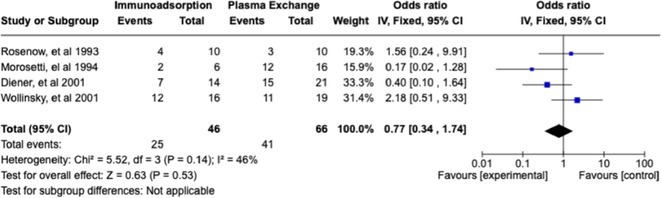
**FIGURE 2** A forest plot showing analysis of patients that had achieved at least 1 score reduction in the Hughes scale 4 weeks or functional improvement after GBS treatment.

**Figure:**
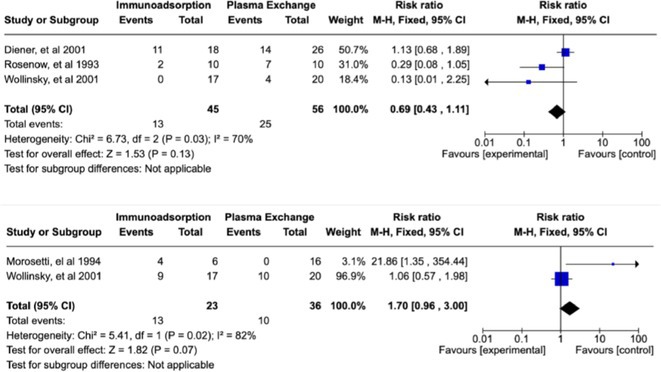
**FIGURE 3** A forest plot showing analysis of patients that had complications after the GBS treatment and that had relapse or lack of clinical improvement after the GBS treatment.

**Conclusion:** Immunoadsorption is at least as effective as plasma exchange in the treatment of Guillain‐Barre Syndrome based on its curative effect by lowering its disability and improving functional score. Immunoadsorption showed reduced complications but relapse rates were higher compared to plasma exchange.

**Disclosure:** Nothing to disclose.

## EPO‐159

### Romberg test in the 21st century: Differences in sway patterns in patients with vestibular and proprioceptive disorders

#### S. Mermelstein^1^; L. Joffily^2^; P. Castro ^3^; T. Ellmers^4^; I. Bocai^5^; M. Jardim^1^; J. Allum^6^; D. Kaski^7^

##### ^1^Department of Neurology, Pedro Ernesto University Hospital, Rio de Janeiro, Rio de Janeiro, Brazil. Department of Neurology, Gaffré‐Guinle University Hospital, Rio de Janeiro, Rio de Janeiro, Brazil; ^2^Department of Neurology, Gaffré‐Guinle University Hospital, Rio de Janeiro, Rio de Janeiro, Brazil. Department of Otorhinolaryngology (ORL), Gaffré‐Guinle University Hospital, Rio de Janeiro, Rio de Janeiro, Brazil; ^3^School of Allied Health Sciences, Faculty of Health and Life Sciences, De Montfort University, Leicester, UK. Universidad del Desarrollo, Escuela de Fonoaudiologia, Facultad de Medicina Clinica Alemana, Santiago, Chile; ^4^Department of Brain Sciences, Imperial College London, Charing Cross Hospital, London, UK; ^5^Department of Otorhinolaryngology (ORL), Gaffré‐Guinle University Hospital, Rio de Janeiro, Rio de Janeiro, Brazil; ^6^Department of Otorhinolaryngology (ORL), University Hospital Basel, Basel, Switzerland; ^7^SENSE research unit, Department of Clinical and Movement Neurosciences, Institute of Neurology, University College London, London, UK

**Background and aims:** The Romberg Test (RT) is a traditional bedside test for static balance, but its nonspecific original description has cast doubt over its diagnostic utility. The development of variants, designed to improve the assessment of vestibular causes of imbalance, has expanded the scope of balance evaluation. These, combined with advanced assessment techniques, have intensified the debate regarding the diagnostic utility of the RT. This study aims to evaluate whether there are specific patterns of sway during the RT and its variants that differentiate individuals with vestibular and proprioceptive deficits.

**Methods:** We assessed 6 healthy individuals and 18 patients whose main complaint was imbalance. Patients were divided into three groups: unilateral vestibular loss, bilateral vestibular loss, isolated peripheral neuropathy. Participants underwent clinical history collection, physical examination, and ancillary tests such as vestibular function testing as appropriate. Each participant performed four stance tasks, recorded using the SwayStar.

**Results:** Greater sway in the stance task on foam surface were more indicative of the presence of vestibulopathy compared to neuropathy. Conversely, patients with peripheral neuropathy exhibited greater imbalance in tasks on firm ground compared to tasks on foam surface. Patients with bilateral vestibular loss swayed more than the unilateral vestibular loss group, but both groups behave similarly and thus differed from the neuropathy group.

**Conclusion:** This study identified distinct patterns of postural sway associated with vestibulopathy and neuropathy during stance tasks, providing clear differentiation between these conditions. These findings underscore the potential of targeted stance tasks to enhance diagnostic accuracy in clinical practice.

**Disclosure:** Nothing to disclose.

## EPO‐160

### Fluid biomarkers in Charcot‐Marie‐Tooth disease

#### R. Bellanti ^1,2^; C. Kramarz^2^; M. Skorupinska^2^; M. Laura^2^; A. Rossor^2^; S. Rinaldi^1^; M. Reilly^2^; M. Lunn^2^

##### ^1^Nuffield Department of Clinical Neurosciences, University of Oxford, Oxford, UK; ^2^Department of Neuromuscular Diseases, Queen Square Institute of Neurology, University College London, London, UK

**Background and aims:** Objective, responsive biomarkers are required to assess disease progression and inform clinical trials in Charcot‐Marie‐Tooth disease (CMT), the most common inherited neuropathy. Peripherin and periaxin, biomarkers of peripheral nerve axonal damage and acute demyelination, respectively, have recently been validated in the inflammatory neuropathies Guillain‐Barré syndrome (GBS) and chronic inflammatory demyelinating polyradiculoneuropathy (CIDP). Here, we evaluate whether peripherin and periaxin are elevated in CMT, and their potential utility for longitudinal monitoring alongside neurofilament light chain (NfL) and existing outcome measures.

**Methods:** We measured serum peripherin, periaxin, and NfL in patients with CMT1A (n=12), CMT2A (n=8) and CMTX (n=8), and compared levels to healthy controls (HC, n=20).

**Results:** Peripherin was higher in CMT1A, CMT2A, and CMTX compared to HC (all p<0.05). NfL was higher in all neuropathy groups versus HC (all p<0.001). Periaxin was elevated in two out of eight CMT2A patients, two out of eight CMTX patients, and below detection limit in CMT1A. Strong correlations were observed between peripherin and clinical tests: stair climb test CMT1A (rho = ‐0.912, p=0.0006) and 6‐minute walk test in CMTX (rho = ‐1, p=0.0167).

**Conclusion:** Fluid biomarkers show promise in CMT. We aim to selectively measure periaxin in patients with clinical evidence of disease progression, where elevated levels may indicate active demyelination. Larger cohorts are being tested to assess the individual and combined contributions of all three biomarkers to clinical evaluation.

**Disclosure:** Nothing to disclose.

## EPO‐161

### Fluid biomarkers in chronic inflammatory neuropathies: comparative analysis and evaluation of diagnostic utility

#### R. Bellanti ^1,2^; D. Lester^1^; N. Dubuisson^1^; M. Turner^1^; A. Thompson^1^; M. Lunn^2^; S. Rinaldi^1^

##### ^1^Nuffield Department of Clinical Neurosciences, University of Oxford, Oxford, UK; ^2^Department of Neuromuscular Diseases, Queen Square Institute of Neurology, University College London, London, UK

**Background and aims:** Objective, responsive biomarkers are required to assess disease progression and inform clinical trials in the inflammatory neuropathies. Peripherin and periaxin, biomarkers of peripheral axonal damage and demyelination, respectively, have recently been validated in Guillain‐Barré syndrome (GBS) and chronic inflammatory demyelinating polyradiculoneuropathy (CIDP). Here, we evaluate their potential clinical utility in chronic immune‐mediated neuropathies, alongside neurofilament light chain (NfL) and existing outcome measures.

**Methods:** Clinical data were retrospectively analysed. Peripherin and NfL were measured in samples from patients with multifocal motor neuropathy (MMN, n=11), POEMS syndrome (n=14), anti‐MAG neuropathy (n=14), multiple sclerosis (MS, n=20), and healthy controls (HC, n=20).

**Results:** Peripherin levels were higher in MMN and POEMS compared to HC (p = 0.01). NfL was higher in MMN vs HC (p = 0.0053), and the peripherin/NfL ratio was higher in MMN compared to MS (p = 0.029). In three POEMS patients with longitudinal data, peripherin closely mirrored VEGF levels over time.

**Conclusion:** Fluid biomarkers show potential in the inflammatory neuropathies. Work is underway to measure peripherin, periaxin, and NfL in larger, deeply phenotyped patient cohorts. We will compare MMN levels to lower motor neuron‐predominant motor neurone disease, and evaluate correlation with paraprotein levels, antibody titres (GM1, GQ1b, MAG), VEGF, and existing outcome measures.

**Disclosure:** Nothing to disclose.

## EPO‐162

### Comprehensive evaluation of outcomes after nerve transfers for brachial plexus injuries

#### Š. Brušáková ^1^; I. Humhej^2^; J. Ceé^1^; I. Holečková^3^

##### ^1^Department of Neurology, Masaryk Hospital Krajská Zdravotní a.s., Sociální Péče 3316/12A, Ústí nad Labem, Czechia; ^2^Neurosurgical Department, Faculty of Health Studies J. E. Purkynje University, Masaryk Hospital Krajská Zdravotní a.s., Sociální Péče 3316/12A, Ústí nad Labem, Czechia; ^3^Neurosurgical Department, Faculty of Medicine in Pilsen, Charles University, Pilsen, Czechia

**Background and aims:** Nerve transfers are a key surgical strategy for managing brachial plexus injuries (BPIs). This study provides a comprehensive assessment of their clinical, neurophysiological, and patient‐reported outcomes.

**Methods:** We analyzed 34 nerve transfers in 21 patients treated over an 11‐year period. Evaluations included muscle strength grading (MRC), functional capacity (Mallet scores), disability impact (DASH), and electrophysiological data to monitor synkinesis and voluntary activation. Additional metrics included pain levels and quality of life assessments.

**Results:** Of the nerve transfers, 58.8% achieved M3 or greater muscle strength, and 14.7% reached M4 or higher. Synkinesis‐free recovery occurred in 29.4% of cases. A significant correlation was observed between early reinnervation (≤6 months) and better muscle strength (Rs = 0.528). Patient‐reported outcomes showed an inverse relationship between improved strength and disability (Rs = ‐0.510). Early surgical intervention emerged as the most critical factor for success.

**Figure:**
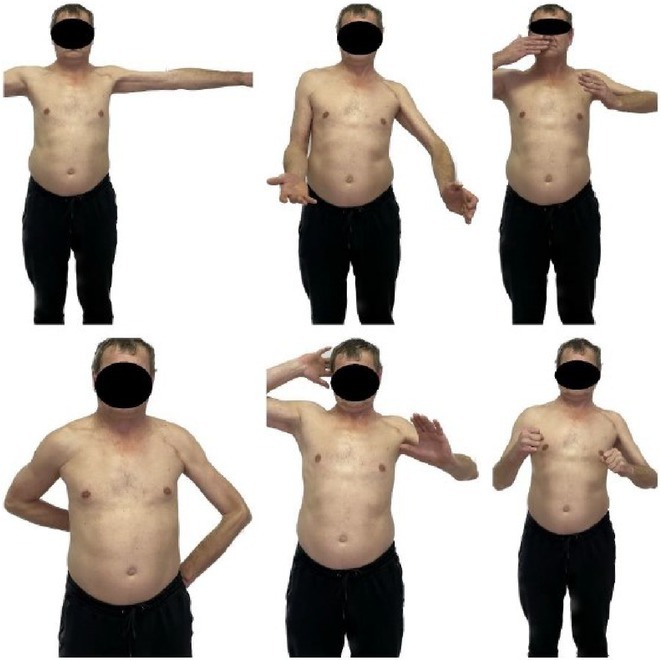
**FIGURE 1** Mallet scale.

**Figure:**
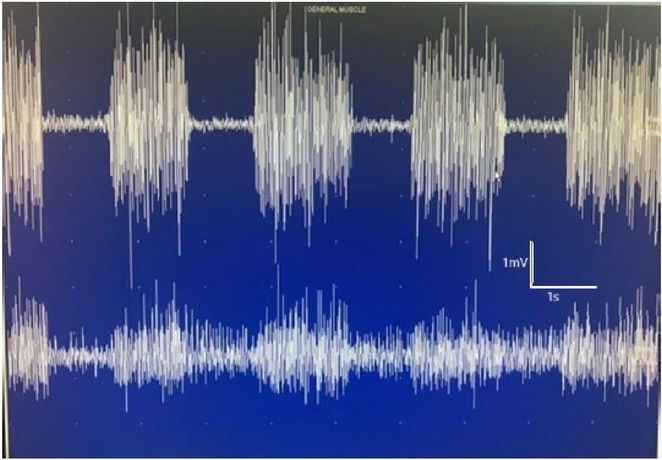
**FIGURE 2** Early reinnervation changes in needle EMG.

**Figure:**
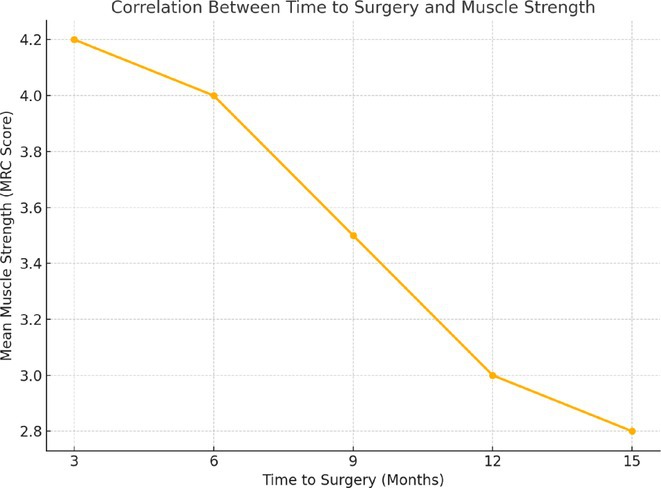
**FIGURE 3** Correlation between muscle strength and time to surgery.

**Conclusion:** Nerve transfers significantly improve functional outcomes in BPI patients. Early intervention is essential for maximizing neuroplasticity and reducing long‐term disability. This study highlights the multifactorial nature of recovery, emphasizing the need for personalized, multidisciplinary care approaches.

**Disclosure:** Nothing to disclose.

## EPO‐163

### Brachial neuritis in hepatitis E: Pleomorphic presentations in three cases

#### G. Bonifacio; T. Disu; D. Allen; C. Osman

##### Neurosciences, Wessex Neurological Centre, Southampton, UK

**Background and aims:** Brachial neuritis (BN), also known as neuralgic amyotrophy, is a rare condition with an annual incidence of 1 in 1000. Its aetiology is multifactorial, often without specific risk factors. Hepatitis E virus (HEV) has been identified as a potential trigger, with immune‐mediated mechanisms implicated in its pathophysiology.

**Methods:** We present three cases of HEV‐associated BN, highlighting their clinical variability and outcomes.

**Results:** Case 1: A 54‐year‐old male presented with bilateral shoulder pain followed by profound bilateral upper limb weakness after acute HEV infection. ALT was >2000 U/L, and MRI revealed patchy denervation oedema in selected muscle groups, supported by EMG findings. Corticosteroid treatment led to partial recovery over one year. Case 2: A 65‐year‐old male developed acute interscapular pain followed by orthopnoea but no weakness. HEV serology was positive (mildly raised ALT). CXR showed bilateral raised hemidiaphragms. EMG revealed bi‐diaphragmatic denervation (confirmed on videofluoroscopy). The patient remains on home non‐invasive ventilation. Case 3: A 65‐year‐old female presented with bilateral sequential shoulder pain followed by right scapular winging and bilateral upper limb weakness. HEV serology was positive (raised ALT), MRI showed denervation oedema, but early EMG findings were normal. Corticosteroids resulted in gradual recovery.

**Conclusion:** These cases highlight the pleomorphic presentations of HEV‐associated BN, ranging from diaphragmatic weakness to scapular winging or profound arm weakness, with variable recovery. Routine HEV testing is crucial in BN presentations. While corticosteroid therapy has been controversial, it has alleviated pain in these cases. Further research is needed to understand long‐term outcomes.

**Disclosure:** All authors declare no conflicts of interest related to this study. This work was conducted independently and received no specific funding or sponsorship.

## EPO‐164

### Reactivity to HNK‐1 glycans and complement activation in anti‐MAG antibody‐associated neuropathy

#### Y. Fukami ^1^; M. Kuwahara^2^; H. Ogata^3^; S. Furukawa^1^; S. Yagi^1^; Y. Nagai^2^; N. Isobe^3^; H. Koike^4^; M. Katsuno^5^

##### ^1^Department of Neurology, Nagoya University Graduate School of Medicine, Nagoya, Japan; ^2^Department of Neurology, Kindai University Faculty of Medicine, Osaka, Japan; ^3^Department of Neurology, Neurological Institute, Graduate School of Medical Sciences, Kyushu University, Fukuoka, Japan; ^4^Division of Neurology, Department of Internal Medicine, Faculty of Medicine, Saga University, Saga, Japan; ^5^Department of Neurology and Department of Clinical Research Education, Nagoya University Graduate School of Medicine, Nagoya, Japan

**Background and aims:** Complement activation is hypothesized to play a significant role in the pathophysiology of anti‐myelin‐associated glycoprotein antibody‐associated neuropathy (MAGN), but its exact contribution remains unclear. This study investigated the relationship between IgM antibody reactivity to human natural killer‐1 (HNK‐1) carbohydrate, complement activation, and disease severity in MAGN. The inhibitory effects of classical complement pathway inhibitors were also evaluated.

**Methods:** Serum samples from 43 patients with MAGN (median age: 69 years) and 33 patients with chronic inflammatory demyelinating polyneuropathy (CIDP) (median age: 71 years) were analyzed. IgM antibody reactivity and complement C3c deposition were quantified using biotinylated synthetic HNK‐1 glycans immobilized on streptavidin‐coated plates. Correlations with clinical parameters were assessed, and the inhibitory effects of C1 inhibitor, anti‐C1q antibody, and C1s inhibitor were evaluated.

**Results:** All MAGN patient sera showed reactivity to HNK‐1 carbohydrate, absent in CIDP sera. A strong correlation was identified between HNK‐1 reactivity and complement C3c deposition (rs = 0.66, p < 0.0001), suggesting a role for complement activation in MAGN. Complement activation correlated with ataxia scores (rs = 0.33, p = 0.02) but not with other parameters. In vitro, complement activation was dose‐dependently inhibited by C1 inhibitor, anti‐C1q antibody, and C1s inhibitor.

**Conclusion:** The study highlights complement activation's potential role in MAGN pathophysiology and the utility of synthetic HNK‐1 glycans for studying this mechanism. While complement inhibitors demonstrated in vitro efficacy, further preclinical and clinical studies are required to evaluate their therapeutic potential.

**Disclosure:** Nothing to disclose.

## Sleep‐wake Disorders

## EPO‐165

### A Fragile Slumber: Understanding sleep in long‐COVID Syndrome: a cross‐sectional study

#### A. Liampas ^1^; C. Ioannou^2^; K. Christodoulou^2^; R. Louka^3^; G. Vavougios^1^; P. Zis^1^; P. Bargiotas^1^; G. Hadjigeorgiou^1^; A. Tofarides^4^; A. Artemiadis^1^

##### ^1^Medical School, University of Cyprus, Nicosia, Cyprus; ^2^Neurophysiology Unit, Department of Neurology, Nicosia General Hospital (SHSO), Nicosia, Cyprus; ^3^Department of Neurology, Nicosia General Hospital (SHSO), Nicosia, Cyprus; ^4^Department of Internal Medicine, Nicosia General Hospital (SHSO), Nicosia, Cyprus

**Background and aims:** Approximately 25% of people with COVID‐19 experience residual or new symptoms even one month after infection, while 10% continue to have symptoms after 12 months. These patients are often referred to as sufferers of Long‐COVID syndrome (LCs), of whom a significant proportion experiences sleep‐related disorders. Our aim was to establish the prevalence of sleep disorders in patients with LCs, and secondly to examine the associations of sleep quality with other parameters.

**Methods:** A cross‐sectional study was performed in the outpatient clinic of the Neurology Clinic Nicosia General Hospital from September 2022 to April 2024. All patients met the WHO definition (2021) for LCs.

**Results:** The sample consisted of 39 women and 12 men (N=51, mean age: 54.1 years ± 11.9). The mean PSQI index score was 9.2 ± 5.3 (0‐18), and 40 (78.4%) patients exhibited poor sleep quality. The PSQI score demonstrated a significant correlation with the following study parameters, in decreasing order: number of symptoms (r=0.675, p<0.001), fatigue (r=0.604, p<0.001), stress (r=0.546, p<0.001), anxiety (r=0.523, p<0.001) and depression (r=0.521, p<0.001). The multivariate analysis of associations of sleep quality revealed that the only statistically significant association of sleep quality was with fatigue (b = 0.85 ± 0.35, beta = 0.33, p = 0.02).

**Conclusion:** A high proportion of patients diagnosed with LCs experience poor sleep quality. The analysis revealed that fatigue, the psychological state of patients, and the number of physical symptoms of LCs collectively accounted for up to 49.6% of the variability in the sleep quality index.

**Disclosure:** Nothing to disclose.

## EPO‐166

### Sleep microstructure: Cyclic alternating pattern analysis in patients with Parkinson's disease and cognitive impairment

#### D. Sandri

##### Neurology Unit, Auxologico Piancavallo, Verbania, Italy

**Background and aims:** Sleep microstructure evaluates the subtle changes of graphoelements which constitute sleep electroencephalogram, specifically the cycling alternating pattern (CAP) considers the relationship between periodic activity (A‐phases) and background rhythm (B‐phases). CAP takes into account sequences of transient electrocortical events distinct from background activity and recurring at up to 1 minute intervals. A‐phases are part of an arousals hierarchy that arrange the sleeping brain to the flexibility around the referential state: the non‐CAP slow wave sleep; establishing CAP as a marker of sleep instability and preservation. In people with neurodegenerative disorders, sleep macrostructure prevents to appraise the subtle variations of graphoelements, whereas different studies displayed a significant reduction of CAP indexes in Alzheimer's disease, REM sleep behaviour disorder and Parkinson's disease(PD). This study analyzes CAP in PD patients with cognitive impairment, evaluating the correlation with neurodegeneration progression.

**Methods:** 16 PD patients with mild cognitive impairment (PDMCI) and 16 with PD dementia (PDD), diagnosed according to Movement Disorder Society's clinical, cognitive and functional criteria, have been recruited. Patients underwent an in‐lab full‐night polysomnography for sleep macrostructure and microstructure scoring.

**Results:** REM sleep percentage (%TST_R), CAP‐rate and A‐phases indexes decreased in PDD patients, with mayor statistical significance for A3‐phases and CAP‐rate in N1‐stage.

**Conclusion:** A logistic binomial regression model, combining %TST_R and CAP‐rate_N1, has been used to predict accurately patients as PDMCI and PDD. Despite the small sample, sleep microstructure seems to add information about neurodegeneration progression in PD cognitive decline. CAP‐rate could be an economical and non‐invasive neurophysiologic marker of neurodegeneration.

**Figure:**
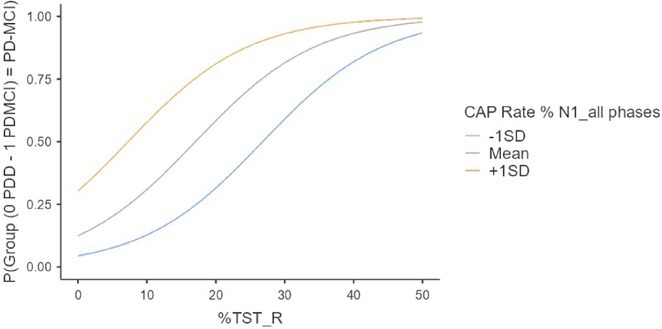
**FIGURE 1** Curve estimated via binomial regression logit model. On the x‐axis, the percentage of REM sleep, while the CAP‐rate_N1 allows to find the best fit of the standard deviations. The dependent variable on the ordinate axis goes from PDD=0 to PDMCI=1.

**Figure:**
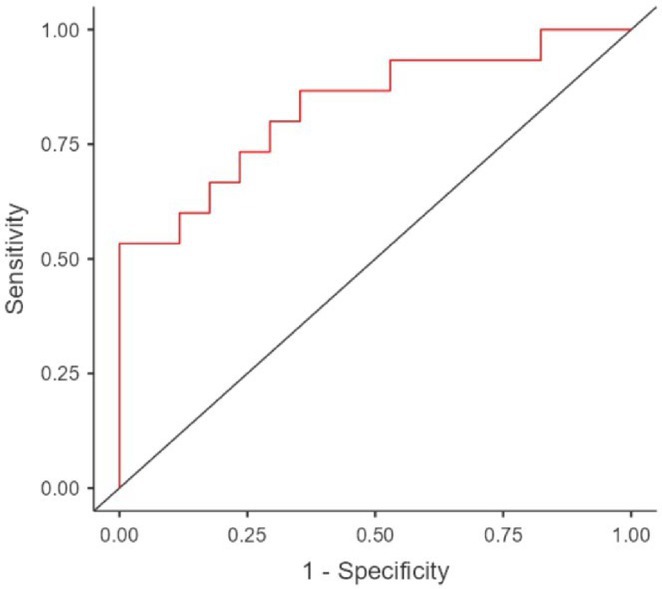
**FIGURE 2** ROC curve of the prediction model.

**Disclosure:** Nothing to disclose.

## EPO‐167

### New associations between chronic insomnia and medication overuse headache (MOH)

#### E. Lebedeva

##### The Ural State Medical University, International Medical Center “Europe‐Asia”, Yekaterinburg, Russian Federation

**Background and aims:** In our previous study we found that chronic insomnia was one of the most significant factors associated with medication overuse headache (MOH). This study aimed to analyze use of painkillers and clinical features of headaches in patients with MOH suffering from chronic insomnia compared with patients with MOH without insomnia in age‐ and gender‐matched patient groups.

**Methods:** A prospective case‐control study was done at the International Headache Center “Europe‐Asia” between March 2021 and December 2023. The study included 171 patients with MOH (mean age 43.3 years, 81.9% women) and 173 patients without MOH (mean age 41.4 years, 74.6% women).

**Results:** Among patients with MOH, 103 patients (60.2%) had chronic insomnia (mean age 46.1, 86.1% females) and 68 patients (39.8%) did not suffer from insomnia (mean age 39.0, 84.2% females). NSAIDs were used by 85% of patients with MOH and chronic insomnia and 87% of patients with MOH without insomnia. We found for the first time that nocturnal headaches (74.8%, p=0.04, OR 2,0; 95% CI 1.01‐3.8), use of analgesics at night (66%, p=0.005, OR 2.5, 95% CI 1.3‐4.6) and taking ≥ 2 doses of painkillers per day (66%, p=0.008, OR 2,3; 95% CI 1,2‐4,3) were significantly associated with chronic insomnia. Patients with chronic tension‐type headache suffered from chronic insomnia more frequent (40.8%, p=0.03, OR 2.1; 95% CI 1.1‐4.6).

**Conclusion:** Our findings stress the necessity of early treatment of chronic insomnia, early withdrawal of analgesics, especially with the tendency to their night use and night headaches.

**Disclosure:** Nothing to disclose.

## EPO‐168

### Sleep hygiene in epilepsy: Coffee‐to‐go or coffee‐to‐seize?

#### E. Balian ^1^; L. Atabekyan^2^; N. Nadryan^1^; H. Hovakimyan^2^; S. Khachatryan^1^

##### ^1^Department of Neurology and Neurosurgery, National Institute of Health, Yerevan, Armenia; ^2^Somnus Neurology Clinic, Yerevan, Armenia

**Background and aims:** Sleep and epilepsy share a well‐established bidirectional relationship. Caffeine, as a stimulant, is one of the key points of sleep hygiene, having an impact on sleep structure. Sleep disruption may lead to poor control of seizures in adults with epilepsy (AWE). We aimed to identify the potential effects of caffeine on sleep and epilepsy parameters in AWE and its implications for disease management.

**Methods:** AWE from a tertiary sleep and epilepsy center were divided into two groups: Non‐Coffee consumers (NCC), Coffee Consumers (CC). CC were divided into Non‐late (NLCC) and Late Coffee (LCC) Consumers (after 17:00). Sleep quality and excessive daytime sleepiness were assessed using validated Armenian version of Pittsburgh Sleep Quality Index and Epworth Sleepiness Scale respectively. Mann‐Whitney U and Chi‐square tests were used for statistics.

**Results:** Sample: n=175, mean age 35.4±13.7(18‐71), females‐47.4%(83). Of them, 85.7%(150) were CC, with LCC comprising 65.1%(97) of the latter. The results of the performed analyses to see whether patients in CC group differ from those from NCC are presented in Tables 1 and 2. We found significant links to leg movement and periodic leg movement indices, and for arousal index. Finally, we found important association between higher seizure frequency and CC. Table 3 shows results for NLCC vs LCC.

**Figure:**
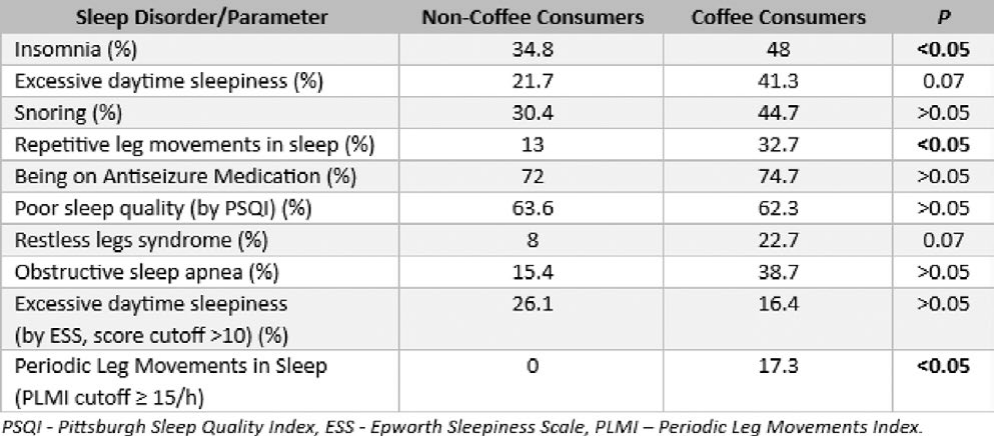
**FIGURE 1** Chi squared analysis of caffeine consumers vs non‐consumers in epilepsy patients.

**Figure:**
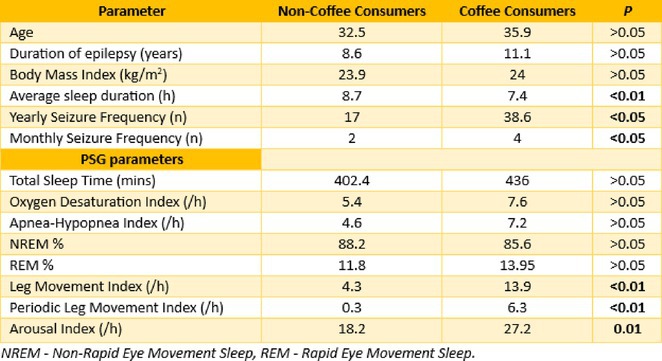
**FIGURE 2** Comparison of means related to polysomnographic parameters and seizure frequency depending on coffee consumption.

**Figure:**
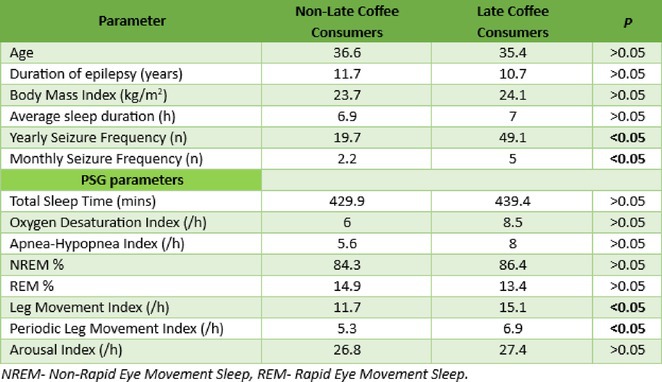
**FIGURE 3** Comparison of means for late coffee consumers in epilepsy.

**Conclusion:** Our results show that coffee may be a potential threat as an exacerbating factor for seizures in AWE, with evening consumption leading to worse outcomes. Sleep hygiene education is an important component of health management for epilepsy patients with impact on seizure frequency and sleep quality.

**Disclosure:** Nothing to disclose.

## EPO‐169

### Screening of sleep‐wake disturbances in acute ischemic stroke: The bernese sleep health questionnaire

#### F. Colò^1^; S. Bauer‐Gambelli ^1^; A. Vordter^3^; E. Vogt^2^; V. Brunetti^4^; S. Baillieul^5^; E. Rollo^4^; P. Bücke^2^; D. Seiffge^2^; M. Schmidt^1^; X. Yang^1^; J. Lippert^2^; C. Bassetti^1^

##### ^1^Interdisciplinary Sleep‐Wake‐Epilepsy‐Center, Inselspital, Bern University Hospital, Bern, Switzerland; ^2^Department of Neurology, Inselspital, Bern University Hospital, University of Bern, Bern, Switzerland; ^3^Swiss Sleep House Bern, Inselspital, Bern University Hospital, Bern, Switzerland; ^4^Department of Neurosciences, Università Cattolica del Sacro Cuore, Rome, Italy; ^5^Grenoble Alpes University, HP2 Lab., INSERM U1300 & Grenoble University Hospital, Grenoble, France

**Background and aims:** Sleep‐wake disturbances (SWD) are common in ischemic stroke and often persist, worsening outcomes when multiple SWD co‐occur. However, comprehensive SWD screening in acute stroke is often time‐consuming and impractical. This study assessed SWD prevalence in acute ischemic stroke patients using the new Bernese Sleep Health Questionnaire (BSHQ).

**Methods:** We enrolled all patients with acute ischemic stroke from the monocentric observational Risk of Atrial Fibrillation In Stroke patients with Sleep disordered breathing study, who completed the BSHQ between July 2022 and November 2024. The BSHQ, a 16‐item self‐reported questionnaire designed to screen for a wide range of SWDs, was administered to clinically stable patients within 72 hours of stroke onset at the Stroke Unit of the University Hospital Bern (Inselspital). The BSHQ assesses symptoms related to sleep‐disordered breathing (SDB), insomnia, excessive daytime sleepiness (EDS), fatigue, restless legs syndrome (RLS), and parasomnias.

**Results:** A total of 369 patients (146 women, 39.6%; mean age: 69.8 years) completed the BSHQ. An elevated risk for SDB, according to a NoSAS‐Score of ≥8, was found in 66.7%. Insomnia symptoms were reported by 15.4% at least three times per week, while 8.7% experienced RLS symptoms at least once weekly. EDS and fatigue symptoms (≥1x/week) were present in 30.6% and 35.8% of patients, respectively. Signs of parasomnias (≥1x/week) were identified in 4.3% of patients.

**Figure:**
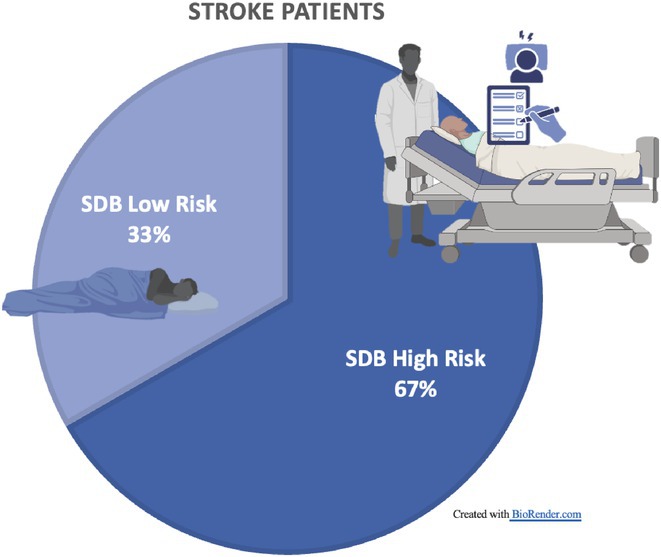
**FIGURE 1** An elevated risk for SDB, according to a NoSAS‐Score of ≥8, was found in 66.7% of patients.

**Conclusion:** The BSHQ revealed high rates of SWD in acute ischemic stroke patients, aligning with prior evidence. The BSHQ may serve as an effective screening tool for SWD in stroke patients, further validation studies are ongoing.

**Disclosure:** Nothing to disclose.

## EPO‐170

### Stress in adult DoA: A web‐based survey

#### G. Mainieri ^1^; M. Belingheri^2,3^; G. Loddo^4^; F. Provini^1,5^; C. Lombardi^6,7^; M. Riva^2,3^

##### ^1^IRCCS Istituto delle Scienze Neurologiche di Bologna, Bologna, Italia; ^2^School of Medicine and Surgery, University of Milano‐Bicocca, Italy; ^4^Department of Primary Care, Azienda AUSL di Bologna, Bologna, Italy; ^6^Istituto Auxologico Italiano, IRCCS, Sleep Disorders Center and Department of Cardiovascular, Neural and Metabolic Sciences, San Luca Hospital, Milan, Italy

**Background and aims:** Disorders of Arousal (DoA) are NREM parasomnias, encompassing three main clinical entities: sleepwalking (SW), confusional arousals (CA), and sleep terrors (ST). Clinical evidence suggests a bidirectional relationship between stress and DoA, wherein stress exacerbates parasomnia episodes, and the episodes themselves may contribute to psychological stress, impacting quality of life. The aim of our study was to evaluate the prevalence of these three DoA entities and their relationship with subjective perception of distress within a university population.

**Methods:** A web‐based survey was conducted among students aged 18 to 35 at Bicocca University of Milan between May and June 2023. The survey collected data on sociodemographic characteristics and lifestyle habits, along with responses to two validated Italian questionnaires: the Arousal Disorder Questionnaire (ADQ), used to assess the occurrence of DoA (1), and the General Health Questionnaire (GHQ‐12), a widely utilized tool for measuring current psychological distress (2).

**Results:** A total of 1,039 students completed the survey (259 males, 780 females), with a median age of 23.0 years (IQR: 21.0–25.0). The prevalence of SW, ST, and CA was 2.7%, 3.0%, and 5.9%, respectively. The overall GHQ score was 6.0 (IQR: 5.0 ‐ 8.0). Comparing subjects with or without DoA, perceived distress was significantly higher in individuals with ST (p=0.0359) and CA (p=0.0034), whereas no significant differences were observed for SW.

**Conclusion:** These findings align with prevalence rates reported in broader adult populations and confirm an association between DoA and stress, claiming for new targeted therapeutic strategies.

**Disclosure:** Nothing to disclose.

## EPO‐171

### ECS improves depression and sleep regulation through modulation of the microbial‐gut‐brain axis

#### J. Ji

##### Department of Anesthesiology, The Third Affiliated Hospital of Sun Yat‐sen University, Guangzhou, China

**Background and aims:** Electroconvulsive therapy (ECT) has shown potential to alleviate depressive symptoms, but its impact on the gut‐brain axis and microbiome is underexplored. This study investigates how ECT regulates depressive behaviors and the composition of the gut microbiota via the microbiota‐gut‐brain axis.

**Methods:** Chronic Unpredictable Mild Stress (CUMS) was used to induce depression in rats, which were divided into control, depression, and ECT treatment groups. Depressive behaviors were assessed by body weight, the open field test, sugar and water consumption, and the forced swimming test. Brain and intestinal histology, microcirculatory blood flow, and inflammatory factors (TNFα, IL1β, IL6) in intestinal tissues were measured by HE staining, immunofluorescence, and ELISA. Intestinal microbiota composition was analyzed via metagenomic sequencing. ANOVA and Kruskal‐Wallis tests were used for data analysis (P<0.05 considered significant).

**Results:** ECT treatment significantly improved depressive behaviors (P<0.01), reducing immobility time in the forced swimming and hanging tail tests (P<0.05). Histology revealed reduced intestinal inflammation (P<0.05), and immunofluorescence showed increased c‐Fos expression (P<0.05). ECT also significantly decreased TNFα, IL1β, and IL6 levels (P<0.01). Metagenomic sequencing revealed increased intestinal microbiota diversity, with a significant restoration of Bacteroidota and Verrucomicrobiota abundance (P<0.05).

**Figure:**
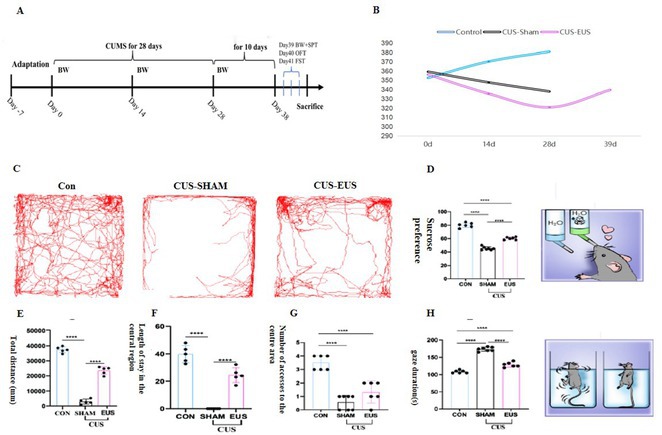
FIGURE 1

**Figure:**
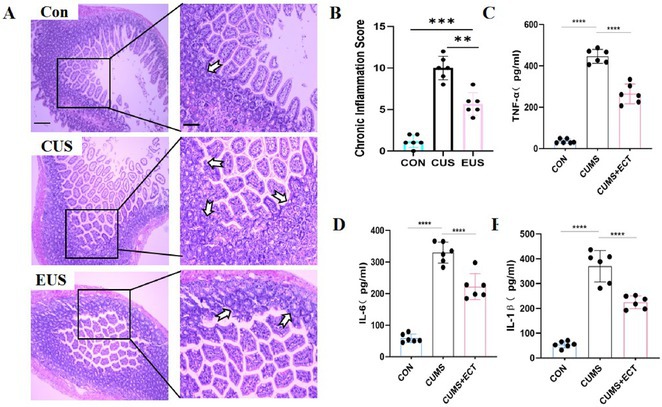
FIGURE 3

**Figure:**
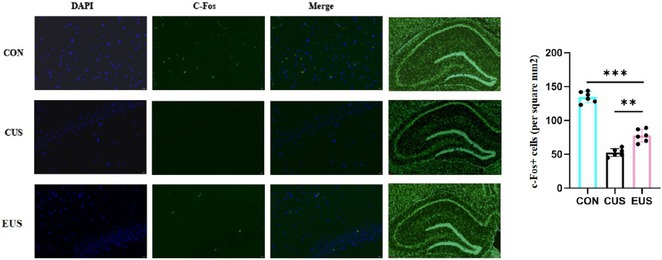
FIGURE 2

**Conclusion:** ECT alleviates depressive symptoms and improves sleep quality by regulating the gut microbiota and enhancing the function of the brain‐gut axis.

**Disclosure:** Nothing to disclose.

## EPO‐172

### Sleep in functional motor disorders: A case‐control polysomnographic study

#### J. Nepozitek ^1^; S. Dostalova^1^; M. Jirasek^2^; G. Chaloupkova^1^; Z. Forejtova^1^; L. Novakova^1^; V. Rottova^1^; V. Konvicna^1^; K. Sonka^1^; M. Edwards^3^; T. Serranova^1^

##### ^1^Department of Neurology and Center of Clinical Neuroscience, First Faculty of Medicine, Charles University and General University Hospital in Prague, Czechia; ^2^Department of Rehabilitation and Sports Medicine, Second Faculty of Medicine, Charles University and University Hospital Motol, Prague, Czechia; ^3^Institute of Psychiatry, Psychology and Neuroscience, King's College London, UK

**Background and aims:** Sleep problems are frequent in functional motor disorders (FMD). Surprisingly, objective correlates of impaired sleep and its relationship to other comorbidities have been understudied, and no polysomnographic study is available. We aimed to map the polysomnographic parameters in the context of self‐reported sleep and mood symptoms and search for comorbid sleep disorders in FMD and healthy controls.

**Methods:** Thirty‐seven patients (mean age (SD), 48.2(10.6) years) with clinically definite FMD and 37 controls (48.6(11.2) years) underwent structured medical and sleep history assessment, neurological examination, and polysomnography and completed questionnaires for sleep quality, sleepiness, depression, and anxiety.

**Results:** In FMD, specific sleep disorders were identified in our cohort, with 32% having restless legs syndrome, 38% clinically significant obstructive sleep apnea, and 8% periodic limb movements in sleep. FMD patients reported worse sleep quality (p< 0.001), higher sleepiness (p< 0.001), depression (p< 0.001), and anxiety (p< 0.001), and had longer REM sleep latencies (p< 0.001). Furthermore, statistical trends for longer sleep latencies (p=0.030), worse sleep efficiency (p=0.012), and higher wake and REM sleep ratios (p=0.013, resp. p=0.027) were found in FMD. In FMD, subjective sleep quality positively correlated with depression (rho=0.54; p<0.002) and anxiety (rho=0.61; p<0.001), and subjective sleepiness correlated with depression (rho=0.42; p=0.010). Self‐reported measures did not correlate with any polysomnographic parameters.

**Conclusion:** Polysomnography detected sleep structure changes in FMD. Sleep abnormalities, including impairments in REM sleep, should be considered in the management of FMD. Future studies should further explore the role of REM sleep disturbances in the pathophysiology of FMD.

**Disclosure:** This work was supported by the Czech Ministry of Health Project AZV NU20‐04‐0332, the project National Institute for Neurological Research (Programme EXCELES, ID Project No. LX22NPO5107) – Funded by the European Union – Next Generation EU; Charles University: Cooperation Program in Neuroscience; General University Hospital in Prague and Ministry of Health of the Czech Republic project MH CZ‐DRO‐VFN64165. Nothing to disclose.

## EPO‐174

### Impulsivity and impulse control disorders in restless legs syndrome

#### A. Frijo^1^; F. Casoni^2^; P. Proserpio^2^; A. Galbiati^1^; L. Ferini‐Strambi ^1^

##### ^1^Faculty of Psychology, Vita‐Salute San Raffaele University, Milan, Italy; ^2^Department of Clinical Neurosciences, Neurology‐Sleep Disorders Center, IRCCS San Raffaele Scientific Institute, Milan, Italy

**Background and aims:** This study aims to examine impulsivity and prevalence of Impulse Control Disorders (ICDs) in patients with Restless Legs Syndrome (RLS), while also exploring differences in impulsivity subtypes independent of ICD presence.

**Methods:** A total of 19 RLS patients and 19 controls were enrolled in the study. The frequency and severity of ICDs were assessed using the modified Minnesota Impulsive Disorders Interview (mMIDI), while disease severity was measured with the International Restless Legs Syndrome Study Group Rating Scale (IRLSRS). Impulsivity was evaluated using the Barratt Impulsiveness Scale‐11 (BIS‐11) and the Go/NoGo task, which assess attentional and motor impulsivity.

**Results:** The results revealed a higher prevalence of ICDs among RLS/WED patients. Notably, compulsive eating disorder was observed in 45% of RLS patients, compared to just 5% in the control group (p=0.005). All patients with ICDs were receiving dopaminergic treatment, suggesting a potential link between DA therapy and ICD development. Furthermore, RLS patients exhibited significantly higher attentional impulsivity than controls (p=0.047), which correlated with symptom severity (r=0.574, p < 0.001). However, no significant differences were found in motor impulsivity.

**Conclusion:** RLS patients exhibited heightened attentional impulsivity, which correlated with symptom severity, while motor impulsivity remained unaffected. Additionally, compulsive eating disorder emerged as a significant concern among RLS patients. Notably, all RLS patients with ICDs were undergoing dopaminergic treatment, highlighting the need for further research to explore this association and its potential connection to attentional impulsivity.

**Disclosure:** Nothing to disclose.

## EPO‐175

### iSPHYNCS: The internationalization and new approaches of the swiss primary hypersomnolence and narcolepsy cohort study

#### J. van der Meer^1^; E. Wenz^1^; L. Fregolente^1^; K. Zub^1^; J. Warncke^1^; O. Gnarra^2^; R. Morand^3^; A. Helmy^1^; Z. Zhang^4^; R. Khatami^4^; S. Von Manitius^5^; S. Miano^6^; M. Strub^7^; M. Tafti^8^; A. Datta^9^; S. Bürki^9^; R. Razaei^10^; U. Kallweit^10^; D. Bijlenga^11^; G. Lammers^11^; S. Mougiakakou^3^; A. Tzovara^12^; M. Tüzün ^13^; M. Schmidt^1^; C. Bassetti^1^

##### ^1^Sleep‐Wake Epilepsy Center, NeuroTec, Department of Neurology, Inselspital, Bern University Hospital, University of Bern, Bern, Switzerland; ^2^Department of Health Sciences and Technology, Institute of Robotics and Intelligent Systems, ETH Zurich, Switzerland; ^3^ARTORG Center for Biomedical Engineering Research, University of Bern, Bern, Switzerland; ^4^Clinic Barmelweid, Center for Sleep Medicine and Sleep Research, Barmelweid, Switzerland; ^5^Department of Neurology, Kantonsspital St. Gallen St, Gallen, Switzerland; ^6^Sleep and Epilepsy Center, Neurocenter of Southern Switzerland, Regional Hospital (EOC) of Lugano, Lugano, Switzerland; ^7^Centre for Sleep Medicine Basel, Basel, Switzerland; ^8^Department of Biomedical Science, Faculty of Biology and Medicine, University of Lausanne, Lausanne, Switzerland; ^9^Neuropaediatrics, University Children's Hospital Basel, Basel, Switzerland, ^10^Center for Narcolepsy and Hypersomnias, Professorship for Narcolepsy and Hypersomnolence Research, Department of Medicine, University Witten/Herdecke, Witten, Germany, ^11^Sleep‐Wake Center, Stichting Epilepsie Instellingen Nederland (SEIN), The Netherlands, ^12^Institute of Computer Science, University of Bern, Bern, Switzerland, ^13^Universitäre Psychiatrische Dienste Bern (UPD), Bern University Hospital, University of Bern, Bern, Switzerland

**Background and aims:** The international Swiss Primary Hypersomnolence and Narcolepsy Cohort Study (iSPHYNCS) aims to provide new data to improve diagnostics and the management of primary central disorders of hypersomnolence (CDH). The three main specific aims of iSPHYNCS are 1) discovery of new biomarkers, 2) assessment of treatment adherence, and 3) patient‐related outcomes, to set the ground for personalized patient treatment.

**Methods:** The study is ongoing at 10 study sites in Switzerland, Germany, the Netherlands and Italy, and plans to prospectively include 500 CDH patients and 60 healthy controls (HC) by the end of 2026. The multi‐modal approach includes questionnaires, clinical assessments, video‐polysomnography, the Multiple Sleep Latency Test (MSLT), vigilance tests, actigraphy, long‐term activity monitoring with Fitbit, immunological studies, quantitative hypocretin measurements, proteomics, gut microbiomics, and genetics/epigenetics. AI‐powered analyses, including unsupervised clustering, are used for data‐driven patient phenotyping.

**Results:** 281 participants, including 10 children, have been recruited. The study population comprises 71 individuals with narcolepsy type I (NT1), 171 individuals of the “narcoleptic borderland” (NBL), such as narcolepsy type 2, idiopathic hypersomnia and insufficient sleep syndrome, as well as 39 HC. Initial analyses reveal notable differences among NT1, NBL, and HC groups across various domains, including questionnaire responses, neuropsychiatric profiles, gut microbiome, polysomnographic, Fitbit and vigilance data.

**Figure:**
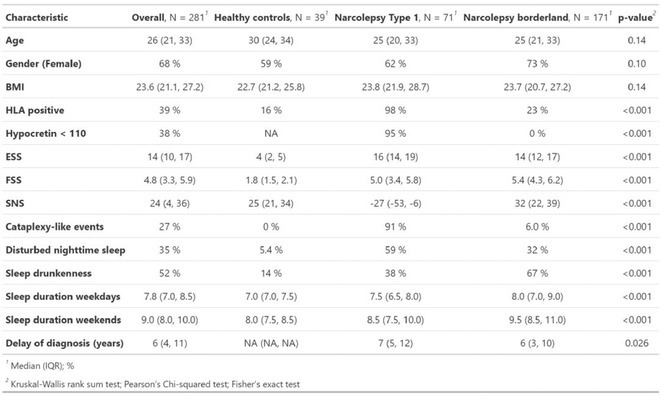
**TABLE 1** Description of the iSPHYNCS Population.

**Conclusion:** Following an initial three‐year phase in Switzerland, the internationalization of iSPHYNCS was successfully launched in 2023. Preliminary results suggest novel and promising clinical, biological, and digital markers of CDH. Proteomics and genetics/epigenetics analyses are currently being explored.

**Disclosure:** The authors declare no conflict of interest. The study is supported by the Swiss National Science Foundation (SNF 320030_185362; SNF 32003B_215721).

## EPO‐176

### Diencephalic–mesencephalic junction dysplasia, a congenital malformation that may cause hypersomnolence: a case report

#### O. Goyena; C. Sifre Peña; A. Gamboa Berastegui; M. Cortes Rubiales; A. Rodriguez Valer; S. Cajaraville; I. Sustacha; S. Fernandez Soberon; T. Gonzalez‐Pinto; A. Pinedo Brochado

##### Galdakao Hospital, Galdakao, Spain

**Background and aims:** Diencephalic‐mesencephalic junction dysplasia (DMJD) is a rare, congenital malformation. It leads to caudal expansion of the diencephalon and a short‐thick midbrain. Few cases of DMJD have been described in adults. We present a case of type B DMJD in a patient with hypersomnia and explore the potential causal relationship between both.

**Methods:** A 48‐year‐old male with a history of hypersomnolence for at least 8 years, which led to two traffic accidents. Initially his hypersomnolence was interpreted due to OSAS and morbidly obesity. Despite treatment with CPAP and bariatric surgery he continued to experience somnolence. A multiple sleep latency test (MSLT) showed daytime hypersomnolence without SOREMs. He had an HLA haplotype of HLA‐DRB115, DQA01:02, DQB1*06:02 and normal cerebrospinal fluid hypocretin levels. Brain MRI revealed type B DMJD with the characteristic butterfly sign.

**Results:** Only three adult cases of type B DMJD are reported in the literature. One patient clinically presented with frontotemporal dementia (FTD) another with involuntary movements and the third patient had headaches. Among the secondary causes of daytime hypersomnolence, structural lesions affecting the hypothalamus‐mesencephalon have been reported. After excluding other causes of hypersomnolence, and considering that this patient presents hypersomnolence associated with obesity we hypothesize that DMJD may be the cause of the patient's clinical presentation.

**Conclusion:** We highlight the importance of considering structural causes in patients with hypersomnolance and we present the first case reported in the literature of DMJD associated with hypersomnolence. Further studies are needed to establish a clearer association between both.

**Disclosure:** Nothing to disclose.

## EPO‐177

### Clinical decision support systems for oxygen‐enriched PAP therapy in obstructive sleep Apnea

#### S. Kistkins ^1^; A. Olsen^2^; D. Freimanis^3^; P. Osipovs^1^; D. Bliznuks^1^; A. Svaza^4^

##### ^1^Institute of Applied Computer Systems, Riga Technical University, Riga, Latvia; ^2^California Institute of Technology, Pasadena, USA; ^3^Research Institute, Pauls Stradins Clinical University Hospital, Riga, Latvia; ^4^Sleep Disorder Center, Riga, Latvia

**Background and aims:** Obstructive Sleep Apnea (OSA) treatment often uses positive airway pressure (PAP) devices, but manual titration poses challenges like patient discomfort and inaccurate SpO2 monitoring. This study evaluates a Clinical Decision Support System (CDSS) with Markov decision processes (MDP) to enhance PAP and oxygen titration, focusing on oxygenation and pCO2 control.

**Methods:** A single‐center observational study included 14 adults (mean age: 63±8 years, BMI: 41±8 kg/m^2^) with OSA‐induced hypoxemia. PAP titration was guided by SpO2 and pCO2 metrics during a one‐night protocol. Manual CPAP/BiPAP adjustments and oxygen supplementation were used to optimize SpO2 (>89%) and reduce apnea events. A paired t‐test assessed changes in AHI and SpO2, while Pearson correlation coefficients evaluated the relationship between pCO2 and IPAP during BiPAP therapy. MDPs modeled treatment state transitions to predict optimal adjustments.

**Figure:**
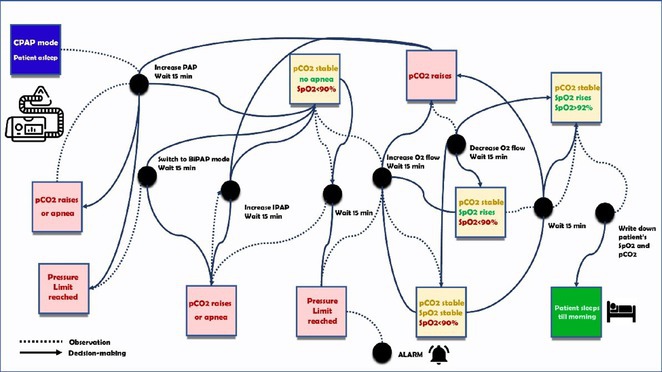
**FIGURE 1** Expert knowledge‐based clinical decision support framework for PAP and oxygen titration using Markov decision processes.

**Results:** The intervention significantly reduced the AHI (mean of delta AHI 48.9±31.9 events/hour (p<0.0001). Mean SpO2 increased by 9%±6.0% (p<0.0003). Transitioning from CPAP to BiPAP (N = 7) significantly reduced pCO2 (p<0.05 for each intervention) in 5 patients, with a strong negative correlation to increased IPAP (mean correlation coefficient: ‐0.71±0.06). Markov model simulations supported effective decision‐making in oxygen titration and demonstrated stable patient respiratory states.

**Figure:**
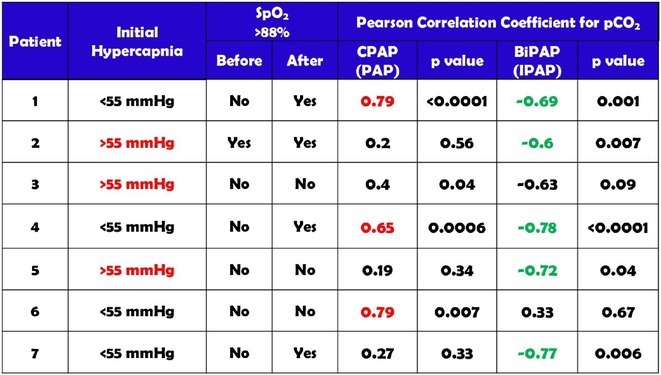
**FIGURE 2** Pearson correlation coefficients of pCO2 comparing the PAP modes.

**Conclusion:** The integration of MDPs into CDSS frameworks for PAP and oxygen titration has shown potential for improving OSA treatment outcomes. By facilitating precise adjustments to therapy, the system enhances SpO2 levels and reduces AHI, particularly in hypercapnic patients. Future work should focus on refining algorithms with larger datasets to achieve personalized respiratory care.

**Disclosure:** Nothing to disclose.

## EPO‐178

### Brief intervention to discontinue inappropriate z‐hypnotic use among older adults: A randomised controlled trial

#### T. Siddiqui ^1^; T. Simonsen^1^; M. Selle^1^; C. Lundqvist^2^

##### ^1^Health Services Research Unit (HØKH), Akershus University Hospital, Lørenskog, Norway, ^2^Health Services Research Unit (HØKH), Akershus University Hospital and Institute of Clinical medicine, Faculty of medicine, University of Oslo, Norway

**Background and aims:** Recommendation from healthcare guidelines suggest avoiding long‐term use of z‐hypnotics in older adults. Yet, inappropriate use (prolonged use at high doses) is common. We tested a brief intervention (BI) for discontinuing inappropriate z‐hypnotics use in older adults.

**Methods:** Triple‐blind two‐arm randomised controlled trial comparing BI conducted by trained GPs to business‐as‐usual (BAU) at baseline and six‐week follow‐up. Intention‐to‐treat (ITT) and per‐protocol (PP) analyses were performed, employing t‐test/Fisher's exact test. The pre‐defined primary outcome was the proportion of participants without inappropriate z‐hypnotic use (≥four weeks, ≥three times per week). Secondary outcomes included sleep complaints, pain levels, and cognition.

**Results:** Both study arms reduced inappropriate use and improved usage pattern (figure). No difference was found in the ITT analysis of BI and BAU at six‐week follow‐up (Fisher's exact test p‐value= 0.51, proportions no inappropriate use BAU = 71% and BI =57%). There were no significant differences between the BI and BAU groups in cognitive function (BI: mean = 18.12, SD = 2.15; BAU: mean = 17.61, SD = 2.89; t(31.3) = 0.59, p = 0.56), global sleep assessment (BI: mean = 6.90, SD = 3.40; BAU: mean = 7.56, SD = 4.19; t(28.458) = 0.51, p = 0.61), or pain levels (BI: mean = 1.67, SD = 1.96; BAU: mean = 1.75, SD = 2.05; t(31.64) = 0.12, p = 0.90). The PP analysis showed similar results.

**Figure:**
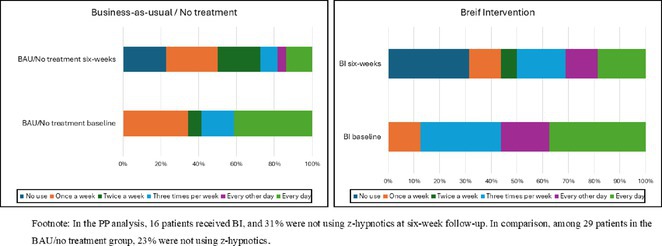
**FIGURE 1** Z‐ hypnotics frequency use in BI and BAU/no treatment group (per protocol analysis).

**Conclusion:** Although many patients reduced their use of z‐hypnotics, there was no significant difference in inappropriate use between the BI and BAU groups.

**Disclosure:** Disclosure: CL has participated on an advisory board and received payment for lectures arranged by Abbvie Pharma AS, Novartis AS, and Roche AS, Norway. He has also received research sponsorship from Abbvie pharma. All other authors declare that they have no conflicts of interest. CT registration: NCT06032715 (registered 17th Aug 2023).

## EPO‐179

### Efficacy and safety of daridorexant in women with insomnia disorder during menopausal transition: A subgroup analysis

#### Z. Schaedel ^1^; C. Bassetti^2^; P. Cassel^4^; S. Palacios^5^; C. Palmay^6^; R. Silvestri^7^; P. Stute^8^; F. Trémollieres^9^; T. Bakker^10^; O. Briasoulis^10^; S. Pain^10^; S. Bertisch^3^

##### ^1^Brighton & Hove Primary Care Ltd, Brighton, UK; ^2^Medizinische Fakultät Bern, Universität Bern, Bern, Switzerland; ^3^Department of Medicine, Brigham and Women's Hospital, Boston, USA; ^4^Frauearztinnen, Gießen, Germany; ^5^Clínica Palacios, Salud de la Mujer, Madrid, Spain; ^6^Midtown Health and Wellness Clinic, University of Toronto, Toronto, Canada; ^7^Centro Interdipartimentale per la Medicina del Sonno, UOSD di Neurofisiopatologia e Disordini del Movimento Dipartimento di Medicina Clinica e Sperimentale, AOU Messina, Italy; ^8^Frauenklinik, Inselspital Bern, Switzerland; ^9^Centre de Ménopause Hôpital, Toulouse, France, ^10^Idorsia Pharmaceuticals Ltd, Allschwil, Switzerland

**Background and aims:** Insomnia is common, burdensome, and under‐researched in women undergoing the menopausal transition. This is the first evaluation of the efficacy and safety of daridorexant (a novel insomnia treatment), specifically in an age group representative of the menopausal transition, enrolled in the phase 3 study NCT03545191.

**Methods:** In this randomized, double‐blind study, 930 patients with insomnia disorder received daridorexant 25 mg, 50 mg or placebo for 3 months. Subgroup analyses were performed among the 117 women aged 47‐55 years (25 mg n=43; 50 mg n=35; placebo n=39). Efficacy endpoints included change from baseline in polysomnography‐measured wake after sleep onset (WASO) and latency to persistent sleep (LPS), self‐reported total sleep time (sTST) and insomnia‐related daytime impairment (Insomnia Daytime Symptoms and Impacts Questionnaire [IDSIQ]).

**Results:** At Month 3, daridorexant 50 mg vs placebo decreased WASO and LPS by a least‐squares mean (LSM) of 13.8 min (95% CI ‐29.0, 1.4) and 14.7 min (‐30.0, 0.6) respectively, increased sTST by a LSM of 21.8 min (‐3.9, 47.4) and decreased (improved) IDSIQ total score by an LSM of ‐4.1 (‐14.4, 6.3) (Figure). These results were generally consistent with those of the overall study population. The incidence of somnolence/fatigue was low in both daridorexant groups and comparable to placebo. Comparable improvements in morning sleepiness (visual analogue scale score) were observed across groups.

**Figure:**
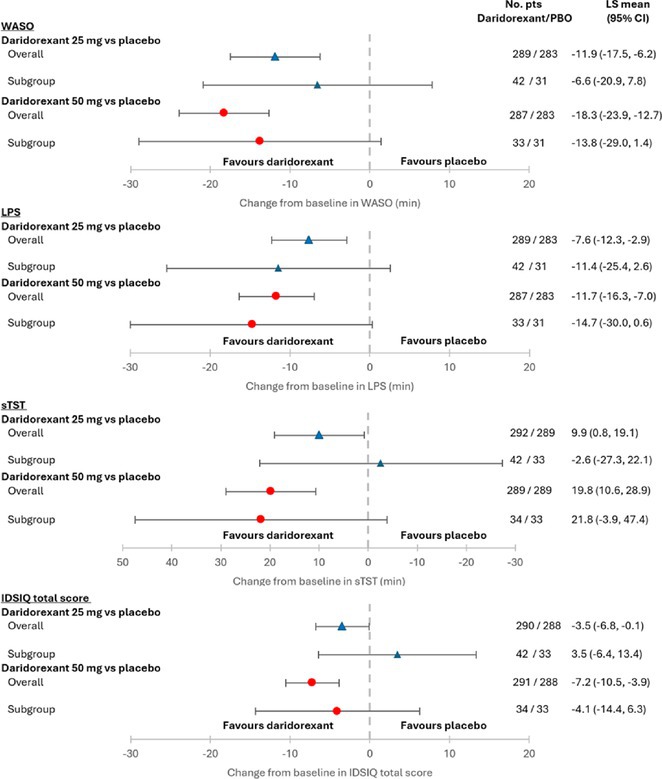
**FIGURE 1** Change from baseline to Month 3 in efficacy endpoints.

**Conclusion:** These post‐hoc exploratory analyses suggest that daridorexant 50 mg improves sleep outcomes and daytime functioning and is well tolerated in women aged 47‐55 years with insomnia disorder.

**Disclosure:** Schaedel Z has received speaker and advisory fees from Theramex, Besins, Idorsia, Astellas and Bayer. Bassetti C served as consultant for IDORSIA 2017‐2023. Bertisch SM has received consulting fees from Idorsia and Elemind. Cassel P has received honoraria for consultations and presentations and travel expense compensations from Idorsia, Dr. Pfleger Arzneimittel GmbH and from mementor Deutschland GmbH. Palacios S has no conflicts of interest to disclose. Palmay C has received speaking and moderating engagements/Honoraria from Pfizer, Merck, Allergan, Bayer, GSK, Galderma, Valeant, Lundbeck, AZ, Bausch Health, Lundbeck, Sunovion, Nuvopharm, Novartis, Lupin, Abbvie, Aspen, Moderna, Sunpharma, Searchlight, Moderna, Sanofi, Seqirus, Idorsia. She has received consulting fees from Dr. Ho Medical, MDBriefcase, The Rounds, Eisai, Sunovion, CCRN. PeerVoice, CCRN Board Director, CTC, Abbvie. She has contributed to Lawrence Park Magazine, CTC Primary Care Podcast, Co‐editor Primary Care Updates. Silvestri R has received honoraria for consultancies and presentations from Idorsia. Stute P has no conflicts of interest to disclose. Trémollieres F has received expert and/or conference fees from Astellas, Bayer, Besins Healthcare France and Theramex. Bakker T, Briasoulis O, Pain S are employees of Idorsia Pharmaceuticals Ltd.

## Sunday, June 22 2025

## Ageing and Dementia 1

## EPO‐180

### CSF synaptic biomarkers negatively correlate with disease duration: New insights into Alzheimer's disease synaptopathy

#### C. Martinuzzo ^1^; C. Trasciatti^1^; A. Pilotto^1^; C. Tolassi^2^; V. Quaresima^1^; S. Pelucchi^4^; L. D'Andrea^5^; R. Stringhi^4^; B. Aksan^5^; S. Caratozzolo^1^; A. Galli^1^; A. Rondina^1^; D. Mauceri^5^; E. Marcello^4^; M. di Luca^4^; A. Padovani^1^

##### ^1^Neurology Unit, Department of Clinical and Experimental Sciences, University of Brescia, Brescia, Italy; ^2^Neurology Unit, Department of Continuity of Care and Frailty, ASST Spedali Civili Brescia Hospital, Brescia, Italy; ^4^Department of Pharmacological and Biomolecular Sciences, Rodolfo Paoletti”, Università Degli Studi Di Milano, Milan, Italy; ^5^Department of Neurobiology, Interdisciplinary Centre for Neurosciences (IZN), Heidelberg University, Heidelberg, Germany

**Background and aims:** Synaptic dysfunction is an early event in Alzheimer's disease (AD). This study explores the relationship between cerebrospinal fluid (CSF) synaptic biomarkers (neurogranin, SNAP‐25, and CAP2) and biomarkers of neurodegeneration, glial cell activation, and inflammation in vivo.

**Methods:** We selected 60 AD patients based on an A+T+N+ CSF biomarker profile. We analyzed demographic variables, cognitive status (MMSE score), disease duration, and CSF biomarkers levels using SIMOA, Luminex, and standard ELISA in AD patients and 40 age‐ and sex‐matched controls (HC). Correlations were assessed through Spearman's partial correlation, adjusting for age and sex. Network analysis, collinearity diagnostic measures and backward multivariable linear regression were performed.

**Results:** AD patients exhibited significantly higher CSF levels of synaptic (p<0.001) and inflammatory markers (p<0.01) compared to HC. CAP2 showed the strongest positive correlation with inflammatory and neurodegeneration markers, but no correlation with biomarkers of amyloidosis. All synaptic biomarkers negatively correlated with disease duration, with CAP2 being the most negatively correlated. Network analysis revealed different relationships between synaptic, neuronal, glial, and inflammatory markers, with neurogranin being the most related to mild inflammatory changes.

**Conclusion:** Higher CSF synaptic and inflammatory biomarkers suggest a compensatory synaptic response to initial AD pathology and its link to inflammatory alterations. The negative correlation with disease duration indicates that AD progression sees these compensatory mechanisms overwhelmed, lowering CSF synaptic biomarkers levels. The lack of correlation with amyloidosis biomarkers suggests that synaptopathy is less driven by beta‐amyloid accumulation. These findings support the potential of a CSF synaptic biomarker panel for AD staging.

**Disclosure:** Nothing to disclose.

## EPO‐181

### Comparative analysis of serum NfL measurements: Agreement between SiMoA and ella platforms in ATTR polyneuropathy

#### D. Righi ^1^; G. Primiano^2^; A. Romano^2^; M. Luigetti^2^; C. Manco^1^; N. De Stefano^1^; D. Plantone^1^

##### ^1^Department of Medicine, Surgery and Neuroscience, University of Siena; ^2^Fondazione Policlinico Universitario A. Gemelli IRCCS, 00168, Rome, Italy

**Background and aims:** Neurofilament light chains (NfL) are neuron‐axonal proteins whose serum concentrations serve as biomarkers for neurological diseases. Due to their low serum levels, precise detection methods are critical. This study aimed to scrutinize the comparability of two techniques: Single Molecule Assay (SiMoA) and Ella automated immunoassay, analyzing serum NfL levels in ATTR polyneuropathy patients and carriers.

**Methods:** A cohort of 55 ATTRv patients and 55 carriers were recruited. We compared the two detection methods using Bland–Altman plots and Passing‐Bablock regression.

**Results:** The mean age of participants was 60 years (25‐75th percentile 47‐72), with 41 females. The median serum NfL concentration measured by Ella (28.4 pg/mL, 25‐75th percentile 9.6‐69.8) was significantly higher (p < 0.001) than that measured by SiMoA (8.9 pg/mL, 25‐75th percentile 5.7‐17.2). The Spearman correlation showed a strong positive correlation (r = 0.8, p < 0.001) between the results of the two methods. The t‐value was 5.2 and p< 0.001. Bland‐Altman analysis showed a mean bias of 15.5 pg/mL (LOA: ‐41.1 pg/mL to 72.0 pg/mL), indicating that Ella overestimated values by 15%. Passing‐Bablock regression showed a linear relationship between the two datasets (p = 0.44) and a slope of 1.72, confirming that Ella measurements were generally higher.

**Figure:**
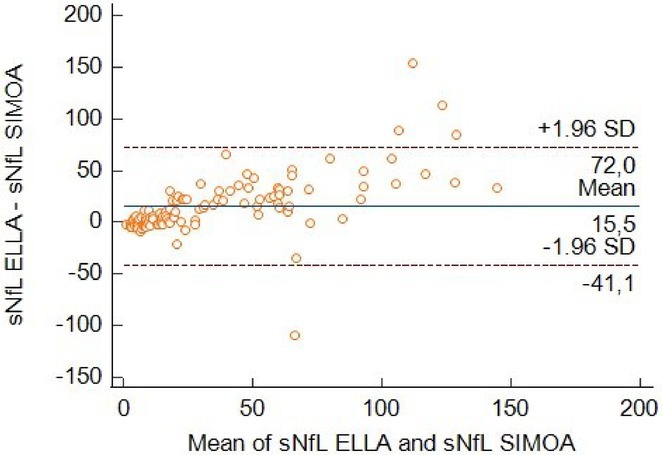
**FIGURE 1** The Bland‐Altman plot shows the differences between the two methods, with the median line representing the mean difference and the limits of agreement (+/‐ 1.96 SD) marking where 95% of differences fall. Random variation and no trends suggest potential ag.

**Figure:**
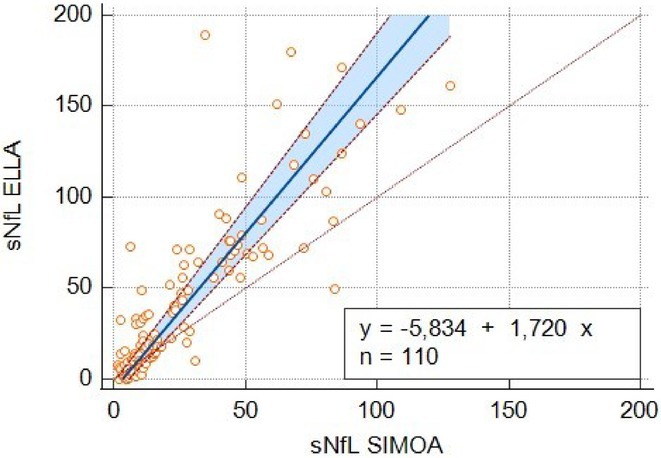
**FIGURE 2** The Passing‐Bablok regression plot shows a linear relationship between the two datasets, suggesting no significant proportional bias. The regression slope of 1.72 indicates that Ella measurements are generally higher than those with SIMOA.

**Figure:**
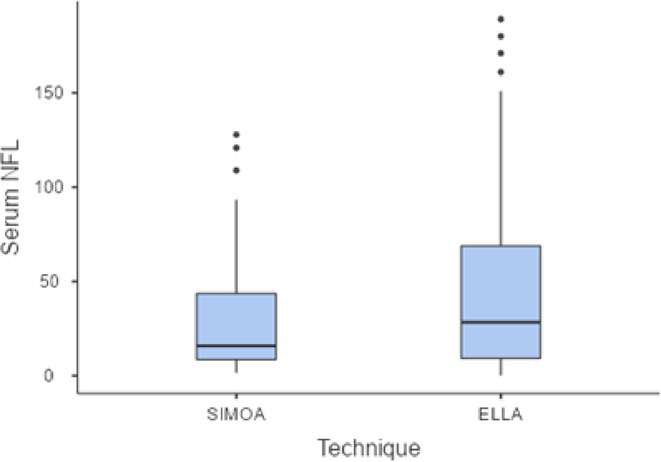
**FIGURE 3** The Paired T‐test plot shows a statistically significant difference between the means of the two measurement methods, considering the data variability.

**Conclusion:** Our findings underscore that both platforms are effective in measuring serum NfL, but Ella consistently yields higher values, especially at higher concentrations. Future studies should focus on standardizing conversion factors to reconcile discrepancies between the two methods.

**Disclosure:** Nothing to disclose.

## EPO‐182

### Serotonergic receptor maps overlap with cortical metabolism changes and CSF NPTX2 in prodromal Alzheimer's disease

#### F. Massa ^1^; B. Orso^2^; S. Garbarino^3^; V. Pelagotti^2^; F. De Cesari^2^; G. Bozzo^2^; S. Raffa^3^; M. Losa^2^; P. Mattioli^1^; A. Brugnolo^1^; N. Girtler^1^; D. Arnaldi^1^; S. Morbelli^4^; G. Sambuceti^5^; M. Pardini^1^

##### ^1^Department of Neuroscience, Rehabilitation, Ophthalmology, Genetics, Maternal and Child Health (DINOGMI), University of Genoa, and IRCCS Ospedale Policlinico San Martino, Genoa, Italy; ^2^Department of Neuroscience, Rehabilitation, Ophthalmology, Genetics, Maternal and Child Health (DINOGMI), University of Genoa, Italy; ^3^IRCCS Ospedale Policlinico San Martino, Genoa, Italy; ^4^Department of Medical Sciences, University of Turin, and Unit of Nuclear Medicine, Città della Salute e della Scienza di Torino, Turin, Italy; ^5^Department of Health Science (DISSAL), University of Genoa, and IRCCS Ospedale Policlinico San Martino, Genoa, Italy

**Background and aims:** Alzheimer's disease (AD) is characterized by progressive cognitive decline, often beginning with Mild Cognitive Impairment (MCI). Early molecular changes and neurodegeneration interact with diffuse projection systems, including dopaminergic, noradrenergic, and serotonergic pathways. Understanding these interactions may enhance knowledge of the disease pathology.

**Methods:** This study assessed baseline [18F]FDG‐PET metabolism and its relationship with monoaminergic receptor maps and cerebrospinal fluid (CSF) biomarkers in 49 MCI‐AD patients. We compared [18F]FDG‐PET scans to 40 matched healthy controls, analyzing spatial correlations between hypometabolism and neurotransmitter receptor/transporter distributions. Fisher's Z‐transformed correlations were used in linear models with distinct CSF biomarkers associated with AD pathology (Aβ42/Aβ40, pTau181, t‐Tau, NPTX2, neurogranin), and NFL, controlling for demographic and cognitive variables.

**Results:** Patients exhibited significant hypometabolism in bilateral temporo‐parietal regions. Negative correlations emerged between hypometabolism and receptor distributions for 5‐HT1A (r=−0.36, p<0.001), D1 (r=−0.15, p=0.03), and mGluR5 (r=−0.14, p=0.02), indicating that reduced glucose uptake in MCI‐AD was strongest in areas with normally high receptor densities. This suggests that these regions, which typically have abundant receptors, are particularly affected by metabolic decline. CSF NPTX2 levels correlated with the co‐localization of hypometabolism and 5‐HT1A (β=0.003, p=0.038) with no significant associations for other CSF markers.

**Conclusion:** Our findings highlight the role of synaptic dysfunction in AD progression, particularly affecting serotonergic cortical targets while relatively sparing noradrenergic pathways. CSF NPTX2 may serve as a biomarker for disease staging and monoaminergic vulnerability. Further research into synaptopathy and projection system deterioration may enhance understanding of early AD pathology.

**Disclosure:** Nothing to disclose.

## EPO‐183

### Improving MRI‐based ML models predicting AD progression in SCD patients with segmentation of early‐affected structures

#### H. Hadžić; O. Lerch; J. Cerman; A. Škorvagová; K. Veverová; J. Hort

##### Memory Clinic, Department of Neurology, Second Faculty of Medicine, Charles University and Motol University Hospital, Prague, Czechia

**Background and aims:** Alzheimer's disease (AD) is a devastating neurodegenerative disorder with increasing prevalence worldwide. Anti‐amyloid therapies should be administered early, before disabling symptoms develop. Therefore, concept of subjective cognitive decline (SCD) was developed. MRI‐based evaluation of neurodegeneration holds promise for predicting clinical progression, but subtle early changes and the number of brain regions assessed necessitate multivariate analysis, such as machine learning (ML) methods. In this study, we aimed to compare performance of models based on standard segmentation (FreeSurfer 6.0) and on adding detailed segmentation of structures affected early in AD ‐ basal forebrain nuclei and medial temporal lobe subfields, obtained using in‐house segmentation pipeline.

**Methods:** Using data from Czech Brain Aging Study (n=93), we trained random forest regressor models on two datasets, using SCD data only: “standard model” using volumetric MRI features (FreeSurfer 6.0) and an “enriched model”, including segmentation‐derived features by custom protocol. SCD patients were categorized based on their clinical trajectory and biomarker status as biomarker‐positive progressors or biomarker‐negative nonprogressors. Model performance was evaluated using regression and classification metrics with area under receiver operating characteristic curve (ROC‐AUC) and compared using DeLong and paired t‐tests.

**Results:** The “enriched model” had higher ROC‐AUC (0.83 vs. 0.77) and reduced root mean squared error (0.427 vs. 0.436) compared to the “standard model”, though results lacked statistical significance.

**Figure:**
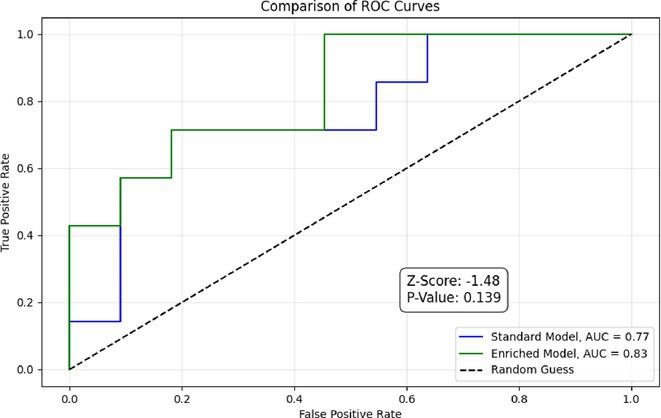
**FIGURE 1** Comparing ROC AUC of “standard model” and “enriched model” using DeLong test

**Conclusion:** Using detailed MRI segmentation protocols including structures affected early in the course of AD may have positive effect on ML model performance in predicting AD progression. Larger datasets and extended follow‐ups are needed to validate clinical applicability.

**Disclosure:** Nothing to disclose.

## EPO‐184

### Abstract withdrawn

## EPO‐185

### Efficacy and safety of sodium oligomannate and acetylcholinesterase inhibitors in Alzheimer's disease

#### L. Alkassas; A. Elrosasy; A. Ramadan; M. Elsayed Abouelmaged; N. Abdelhalim; H. Abd al‐azim; N. Aldeen Mahmoud; M. Kamal; S. Metwally; M. Qarma; A. Zabady; A. Sardahi; B. Touhami; N. Abdelhadi; O. Yousef

##### Faculty of Medicine, Cairo University, Cairo, Egypt

**Background and aims:** Alzheimer's disease (AD) is a prevalent neurodegenerative disorder. While current treatments like acetylcholinesterase inhibitors (AChEls) offer symptomatic relief, they do not halt disease progression. Sodium oligomannate (GV‐971) has emerged as a potential therapeutic agent with promising effects in preclinical studies. This meta‐analysis evaluates the efficacy and safety of sodium oligomannate, alone or in combination with AChEls, compared to donepezil or placebo in treating AD.

**Methods:** We systematically searched online databases for randomized controlled trials (RCTs) investigating GV‐971 in AD patients. Data from seven RCTs involving 1,352 participants were pooled and analyzed using RevMan 5.4.1, with outcomes including cognitive function, daily living activities, neuropsychiatric symptoms, and safety profiles.

**Figure:**
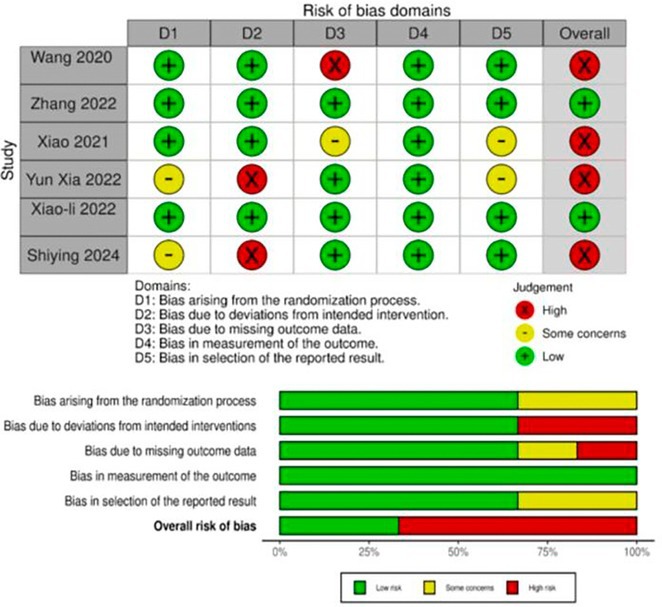
**FIGURE 2** Risk of bias according to ROB‐2.We assessed the risk of bias in the included studies using the Rob 2 tool, which is a Cochrane‐approved instrument for evaluating the quality of randomized controlled trials. This tool included five domains: bias from randomization process, allocation concealment, integrity of the intervention, bias of missing data, and bias missing outcome data, measurement outcomes and selection of the reported results. Four included studies showed a high risk of bias using RoB‐2 except Zhang 2022 and Xiao‐li 2022. The details of the RoB‐2 assessment are shown in Figure 2.

**Results:** Sodium oligomannate significantly improved cognitive function compared to placebo (MD = ‐2.96, 95% Cl ‐5.30 to ‐0.62, p = 0.01) and donepezil (MD = ‐5.67, 95% CI ‐9.17 to ‐2.17, p = 0.002) on the ADAS‐ Cog12 scale. When combined with AChEls, GV‐971 also demonstrated superior efficacy over AChels alone (MD = ‐3.25, 95% Cl ‐5.27 to ‐1.23, p = 0.002). However, secondary outcomes such as ADCS‐ADL and NPI showed mixed results, and no significant differences were observed in the MMSE scores. Safety analysis indicated no significant increase in adverse events compared to placebo.

**Figure:**
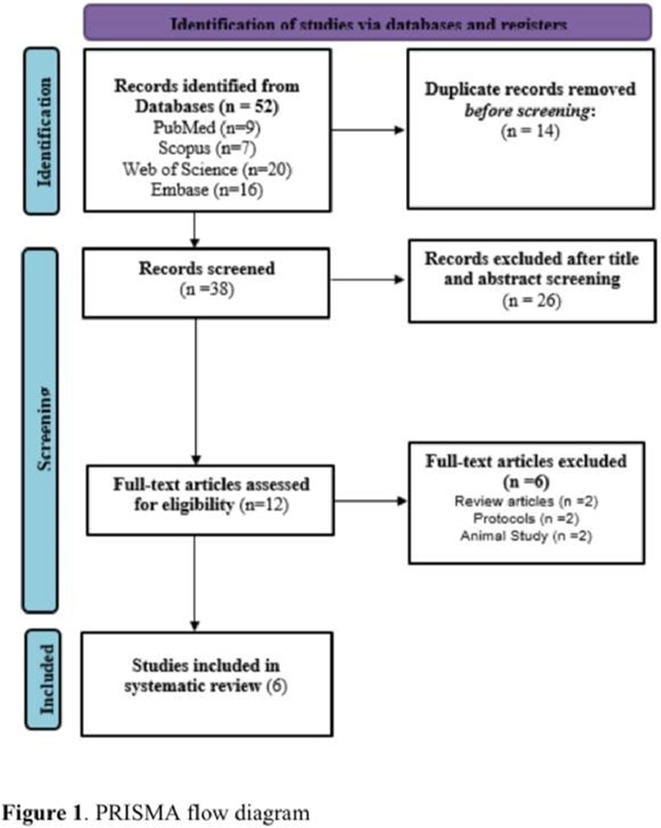
Our search retrieved 38 unique articles. Following the abstract screening, only 12 studies were eligible for full‐text screening. Finally, six randomized clinical trials (RCTs) were included in this review with 1252 patients.

**Figure:**
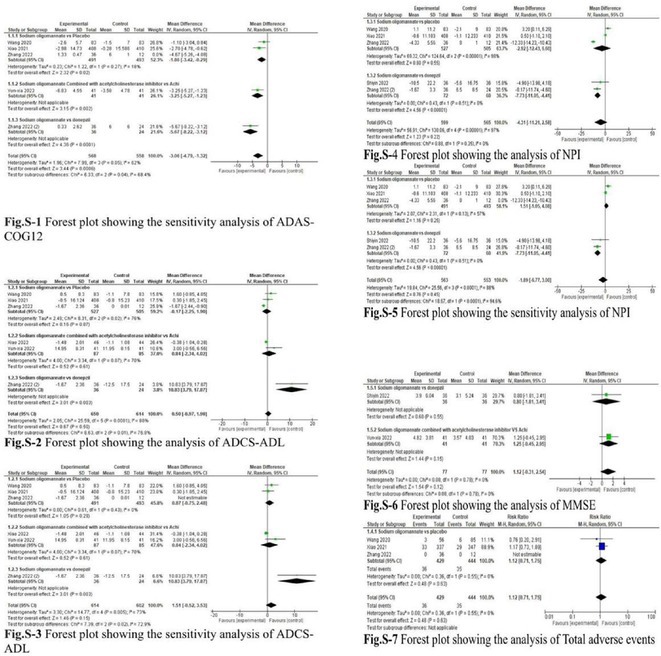
**FIGURE 3** Sodium oligomannate significantly improved cognitive function compared to placebo (MD = ‐2.96, 95% Cl ‐5.30 to ‐0.62, p = 0.01) and donepezil (MD = ‐5.67, 95% CI ‐9.17 to ‐2.17, p = 0.002) on the ADAS‐ Cog12 scale. When combined with AChEls, GV‐971 also d.

**Conclusion:** Sodium oligomannate shows promise in enhancing cognitive function in AD, particularly when compared to donepezil or placebo. While its safety profile is comparable to placebo, larger, well‐designed RCTs are necessary to validate these findings and explore the long‐term benefits of GV‐971, especially in combination therapies.

**Disclosure:** Nothing to disclose.

## EPO‐186

### Mini‐linguistic state examination: The French‐Canadian version of an international test for degenerative aphasias

#### L. Bavelier ^1^; S. Ferrieux^1^; L. Grimont^1^; I. de Marcellus^1^; C. Rébillard^1^; E. Poulin^2^; M. Lavoie^2^; R. Laforce^2^; M. Teichmann^1^

##### ^1^Department of Neurology, DMU Neurosciences, National Reference Center for « Rare or Early Onset Dementias », Pitié‐Salpêtrière University Hospital, Assistance Publique Hôpitaux de Paris (AP‐HP), Paris, France; ^2^Clinique Interdisciplinaire de Mémoire, Département des Sciences Neurologiques de l’hôpital universitaire‐ CHU de Québec, Québec (QC), Canada

**Background and aims:** There is need for rapid internationally applicable tests assessing language in neurodegenerative conditions to allow for homogenized diagnosis/classification of primary progressive aphasias (PPA), monitoring language decline, and providing endpoints in clinical/therapy trials. Such a tool, the ‘Mini‐Linguistic State Examination’ (MLSE) is currently developed within a worldwide network (22 countries). Our Paris‐Québec collaboration aimed at developing a French/Canadian version (fc‐MLSE), validating it with healthy controls, and applying it to patients.

**Methods:** The fc‐MLSE was adapted from the English version. Stimuli were selected to have similar linguistic complexity. Like the English version, the fc‐MLSE included 11 sub‐tests assessing 5 linguistic domains (motor‐speech, phonology, semantics, syntax, verbal working‐memory), providing a total‐score and 5 sub‐scores. It was applied to 182 controls to generate normative scores, and to 36 PPA patients (nonfluent/agrammatic [nfav‐PPA, n=8], logopenic [lv‐PPA, n=20], semantic [sv‐PPA, n=8]), and 6 Alzheimer's disease (AD) patients.

**Results:** Testing‐durations were ∼8/∼12 (controls/patients). Inter‐rater consistency was 92%. There were no ceiling effects in controls, and sub‐score results led to stratifications according to age‐ranges and educational levels. The fc‐MLSE distinguished PPA and AD patients from controls. PPA had lower total‐scores than AD patients. Sub‐scores distinguished PPA variants, showing highest error‐rates for motor‐speech, verbal working‐memory, and semantics in nfav‐PPA, lv‐PPA and sv‐PPA, respectively.

**Figure:**
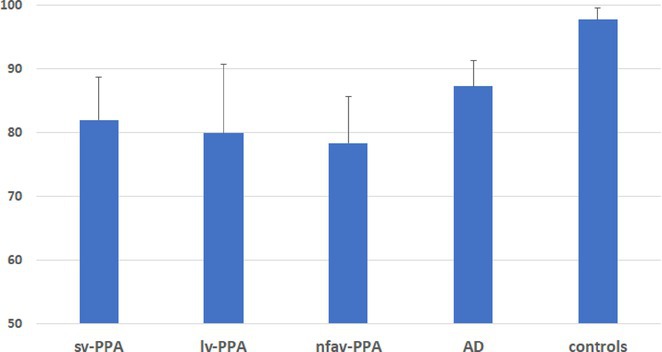
**FIGURE 1** fc‐MLSE total scores (/100) in patients with PPA variants and AD, and in healthy controls

**Figure:**
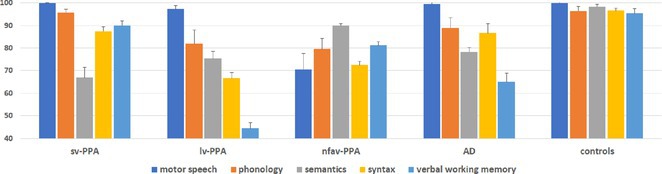
**FIGURE 2** Response accuracy (%) of patients with PPA variants and AD, and in healthy controls in the five language domains of the fc‐MLSE

**Conclusion:** The fc‐MLSE is a rapid, examiner‐consistent language test, validated with a large population of controls. It is suitable for classification of PPA variants, and will allow for follow‐up and trial monitoring in neurodegenerative diseases affecting language. The international use of MLSE versions will improve consistency/uniformity of language assessments.

**Disclosure:** Nothing to disclose.

## EPO‐187

Abstract withdrawn

## EPO‐188

### Distinguishing MCI and Alzheimer's disease: A machine learning framework using neuroimaging biomarkers

#### N. Bülbül ^1^; İ. Baytaş^2^; E. Kavalcı^2^; E. Karasu^2^; B. Okcu Korkmaz^1^; B. Belen^1^; İ. Musaoğlu^3^; A. Ovut^4^; N. Arslanoğlu^5^; M. Urhan^4^; H. Mutlu^3^; M. Özdağ^1^

##### ^1^Health Science University Sultan Abdulhamid Han Research and Training Hospital, Department of Neurology, Istanbul, Turkey; ^2^Bogazici University, Department of Computer Engineering, Istanbul, Turkey; ^3^Health Science University Sultan Abdulhamid Han Research and Training Hospital, Department of Radiology, Istanbul, Turkey; ^4^Health Science University Sultan Abdulhamid Han Research and Training Hospital, Department of Nuclear Medicine, Istanbul, Turkey; ^5^Health Science University Sultan Abdulhamid Han Research and Training Hospital, Department of Psychology, Istanbul, Turkey

**Background and aims:** It is still unknown and unclear what precise characteristics set people with mild cognitive impairment (MCI) apart from those who develop Alzheimer's disease (AD). Our goal is to use machine learning techniques to identify neuroimaging biomarkers that can predict the likelihood of developing AD from MCI.

**Methods:** A custom and local dataset of 251 visits of 237 patients was created. The clinical relevance of the data was verified by statistical methods and shown to be clinically consistent. In addition to MRI volumetric and density measurements, cognitive tests such as MMSE, ACE‐R and PET values were also included in the analysis, thus adopting a multimodal approach. We compared the statistical outcomes with the machine learning classification results. MRI measurements were extracted using FreeSurfer version 7.4.1.

**Results:** Forty‐one statistically significant MRI features and 15 significant PET features were detected. In MRI volumetric evaluation, structures such as left and right caudate nucleus, left amygdala, corpus callosum posterior, left hippocampus and left nucleus accumbens were associated with the risk of conversion from MCI to AD. For 27 patients, vector similarity was used to analyze the risk of conversion from MCI to AD. Based on MRI measurements, we demonstrated that some patients may have a higher risk of progression to AD.

**Conclusion:** It is important that we can use such machine learning models, thanks to their neuroimaging value, to distinguish between MCI and AD, to predict diagnosis and to identify early stage patients who are the target of future treatment options.

**Disclosure:** This project was supported by TUBITAK.

## EPO‐189

### In vivo GABA imaging using CEST MRI to investigate the therapeutic effect of RF‐EMF exposure in AD mice

#### S. Liu

##### Department of Radiology, Second Affiliated Hospital, Shantou University Medical College, Shantou, China

**Background and aims:** Radiofrequency–electromagnetic field(RF‐EMF) are beneficial in treating Alzheimer's disease(AD), but the underlying neurophysiological mechanisms remain unclear. It has been proposed that EMF promotes GABAergic neurogenesis, and abnormal GABA levels are an important influence in AD. Therefore, in vivo GABA imaging is essential. As an essential branch of molecular imaging, chemical exchange saturation transfer(CEST) magnetic resonance imaging(MRI) can provide vital information for quantitative imaging.

**Methods:** The AD mice received RF‐EMF treatment for 4 weeks. The level of GABA was assessed by CEST MRI in vivo and ELISA in vitro. GABA Receptor expression was assessed by western blot. Aquaporin‐4(AQP4) polarization and amyloid‐β(Aβ) accumulation were quantified by the immunohistochemistry. Neuronal functional status was examined by Nissl staining. Spatial learning memory function was evaluated by the Morris water maze test.

**Figure:**
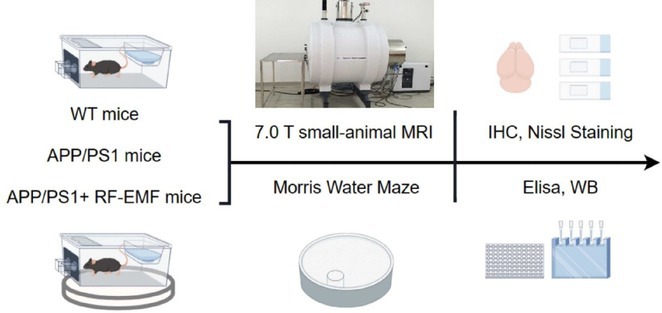
**FIGURE 1** Study design

**Results:** 1. MRI results showed that RF‐EMF could significantly increase the GABA signal in the hippocampus of AD mice. ELISA's results were consistent with the Variable Delay Multi Pulse(VDMP)‐CEST results, and VDMP‐CEST was more accurate in detecting changes in cortical GABA signals than Continuous Wave(CW)‐CEST. Correlation analysis revealed that the correlation of GABA signals with GABA levels was more significant with VDMP‐CEST than CW‐CEST. 2. With the pathological validations, we found RF‐EMF can elevate hippocampal GABA levels by polarizing AQP4, reducing Aβ accumulation and neuronal degeneration, improving cognitive impairment.

**Figure:**
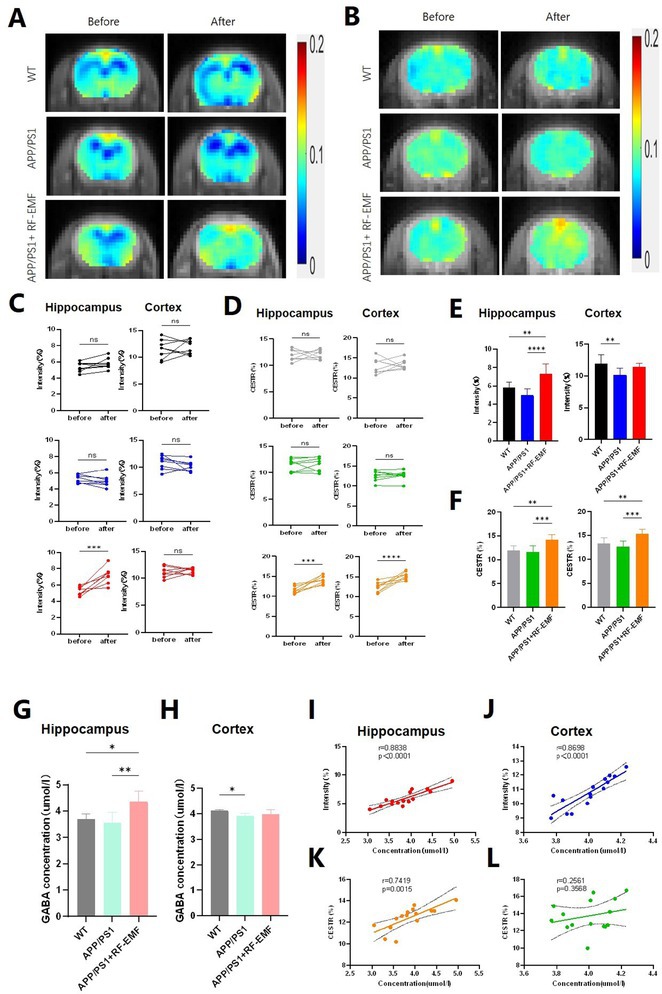
**FIGURE 2** Results of CEST MRI and ELISA

**Figure:**
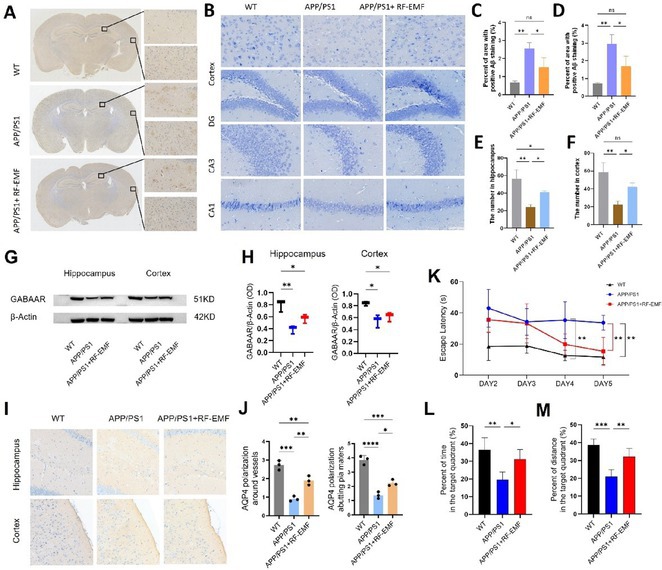
**FIGURE 3** Pathological validations and Behavioral Testing of the Therapeutic Effect of RF‐EMF on AD

**Conclusion:** 1. VDMP‐CEST enables non‐invasive in vivo GABA imaging 2. GABA levels in AD would be a specific and effective biomarker for monitoring the effect of RF‐EMF treatment.

**Disclosure:** Nothing to disclose.

## EPO‐190

### Electroencephalographic patterns as diagnostic endpoints across the dementia spectrum

#### J. Clarot; V. Zoteva; E. Vereycken; N. Colenbier; C. Neuray; P. van Mierlo

##### Clouds of Care, Ghent, Belgium

**Background and aims:** As dementia represents several neurodegenerative disorders with distinct clinical and cognitive symptoms, a robust tool for accurate differentiation is necessary. This study investigates the potential of electroencephalography (EEG) to distinguish between dementia subtypes, using machine learning techniques.

**Methods:** Routine EEG data were analysed from 386 participants, clinically categorised into Alzheimer's disease (AD), non‐Alzheimer's dementia (non‐AD), mild cognitive impairment (MCI), subjective cognitive decline (SCD), healthy controls (HC), and non‐cognitive symptomatic controls (SC). Spectral features (e.g., relative power, peak frequency, and aperiodic components) were extracted from the EEG recordings. Three machine learning models (random forest, gradient boosting, and linear support vector machine (SVM)) were trained for multiclass classification, using nested cross‐validation with stratified 6‐fold cross‐validation to optimise hyperparameters and assess performance.

**Results:** AD subjects exhibited significantly increased low‐frequency power and decreased high‐frequency power with elevated alpha3/alpha1 and alpha3/alpha2 ratios (alpha1 = 8‐9Hz, alpha2 = 9‐11Hz, alpha3 = 11‐13Hz) compared to controls and other subtypes. Non‐AD subjects showed increased delta and theta power compared to controls, with higher theta peak frequency than HC, MCI, and SCD. MCI exhibited elevated delta power compared to controls but no differences with SCD, along with lower theta peak frequency than controls and higher delta and theta peak frequencies compared to SCD. Gradient Boosting showed the best generalization performance (AUROC: 0.875, F1‐score: 0.608), outperforming Random Forest (AUROC: 0.860, F1‐score: 0.560) and Linear SVM (AUROC: 0.781, F1‐score: 0.391).

**Conclusion:** EEG shows potential as a non‐invasive tool to distinguish between dementia‐related conditions, aiding in diagnosis of this syndrome.

**Disclosure:** Julie Clarot, Emiel Vereycken, Nigel Colenbier, Velislava Zoteva are employees of Clouds of Care Caroline Neuray, Pieter van Mierlo are consultants and shareholders at Clouds of Care.

## EPO‐191

### Brexpiprazole for agitation in alzheimer's disease: A meta‐analysis of randomized controlled trials

#### Z. Bakir ^1^; R. Sudo^2^; M. Gobbo^3^

##### ^1^Department of Medicine, Sapienza University of Rome, Italy; ^2^Department of Medicine, Federal University of Grande Dourados, Brazil; ^3^Department of Medicine, Pontifical Catholic University of Rio Grande do Sul, Brazil

**Background and aims:** Agitation in Alzheimer's disease (AD) requires effective and well‐tolerated interventions. Recently, Brexpiprazole has emerged as a promising therapeutic avenue.

**Methods:** We systematically searched ClinicalTrials.gov, PubMed, Embase, and Cochrane Library for randomized controlled trials (RCT) comparing Brexpiprazole to placebo in patients with AD presenting with agitation. A random‐effects model was employed to compute mean differences and risk ratios using R software 4.3.1. The results were reported following the PRISMA guideline.

**Results:** A total of 3 double‐blind RCTs were included, comprising 1,028 patients with an average age of 74 years. Throughout a 12‐week mean follow‐up period, Brexpiprazole was associated with no changes in Clinical Global Impression‐Severity of illness (MD ‐0.19; 95% CI ‐0.38 to 0.00; p=0.05) and Neuropsychiatric Inventory‐Nursing Home scores (MD ‐1.51; 95% CI ‐3.63 to 0.62; p=0.16). However, there was a notable improvement in Cohen‐Mansfield Agitation Inventory score (MD ‐3.04; 95% CI ‐5.04 to ‐1.04; p<0.01). Additionally, no difference was observed for the incidence of at least 1 treatment‐emergent adverse events (TEAE) (RR 1.10; 95% CI 0.94 to 1.28; p=0.52), discontinuation due to TEAE (RR 1.50; 95% CI 0.81 to 2.78; p=0.20), dizziness (RR 1.04; 95% CI 0.52 to 2.11; p=0.86), extrapyramidal disorders (RR 2.60; 95% CI 0.44 to 15.40; p=0.99), and all‐cause death (RR 1.51; 95% CI 0.25 to 8.94; p=0.48).

**Figure:**
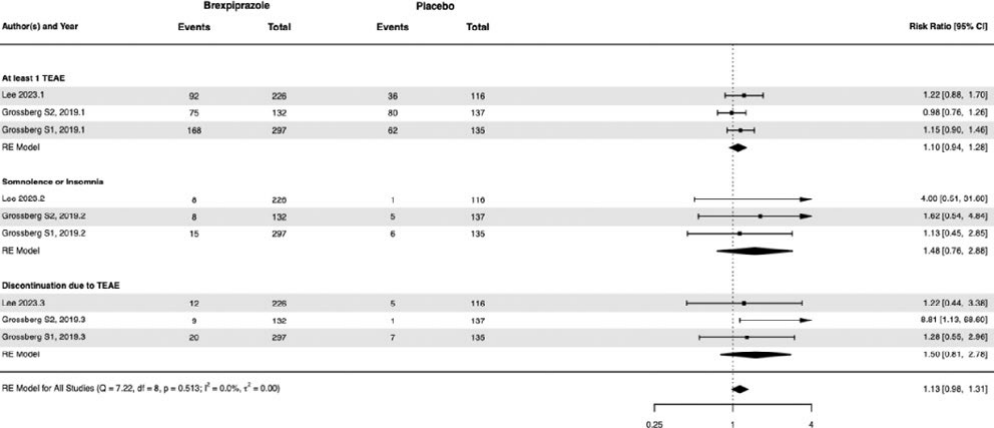
**FIGURE 1** Adverse Events

**Figure:**
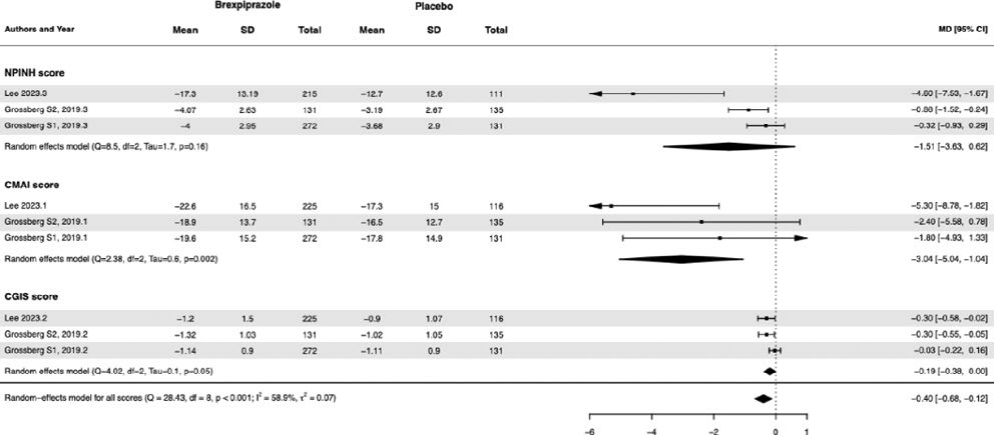
**FIGURE 2** Agitation score change

**Conclusion:** In this systematic review and meta‐analysis of 3 RCTs and 1,028 patients, Brexpiprazole was associated with a modestly favorable modulation in agitation score, concurrent with a positive safety profile.

**Disclosure:** Nothing to disclose.

## EPO‐192

### Tauopathy worsens with adiponectin deficiency and improves with adiporon treatment in mice with human tau mutation

#### Z. Zhang ^1^; L. Yick^1^; W. Zou^1^; H. Xue^1^; J. Kwan^1^; R. Ng^2^; K. Chan^1^

##### ^1^Division of Neurology, Department of Medicine, School of Clinical Medicine, Li Ka Shing Faculty of Medicine, The University of Hong Kong, Hong Kong SAR, Hong Kong; ^2^Division of Neuroscience, School of Biological Sciences, The University of Manchester, Manchester, UK

**Background and aims:** Tauopathy is characterized by the accumulation of hyperphosphorylated tau proteins in neurons. It is a pathological hallmark of Alzheimer's disease. Adiponectin (APN), an adipokine secreted from adipocytes, exerts anti‐inflammatory effects and promotes hippocampal neurogenesis. However, whether APN contributes to tau‐mediated neurodegeneration remains unknown. We aim to investigate the impact of APN deficiency on cognitive functions and neuropathologies in mice with tauopathy.

**Methods:** Cognitive functions of 9‐month‐old wildtype, APN knockout (APN‐/‐) mice, human tau P301S mutation transgenic (TauP301S) mice, and APN‐deficient tau (TauP301S; APN‐/‐) mice were examined by the novel object recognition (NOR) test. Hyperphosphorylated tau accumulation, microgliosis, and neuronal loss in the brain were analyzed by immunofluorescent staining. The therapeutic effect of an APN receptor agonist, adipoRon, was assessed by treating 6‐month‐old TauP301S and TauP301S; APN‐/‐ mice for three months.

**Results:** TauP301S; APN‐/‐ mice spent significantly less time exploring the novel object than wildtype during the NOR test. The immunoreactivity of hyperphosphorylated tau (AT8) and microglia markers ionized calcium‐binding adaptor molecule 1 (Iba1) in the brain of TauP301S; APN‐/‐ mice were significantly elevated compared with other groups. TauP301S; APN‐/‐ mice exhibited significantly fewer neuronal nuclei (NeuN) positive neurons than other groups. Importantly, chronic adipoRon treatment reduced AT8 immunoreactivity in TauP301S; APN‐/‐ mice markedly compared with vehicle‐treated TauP301S; APN‐/‐ mice.

**Conclusion:** APN deficiency aggravates tauopathy, characterized by increased hyperphosphorylated tau accumulation, microgliosis, and neuronal loss, which are reversed by adipoRon treatment. Further experiments will be conducted to study the underlying mechanism of how adipoRon improves tauopathy.

**Disclosure:** This project is supported by Health & Medical Research Fund.

## Autonomic Nervous System Diseases

## EPO‐193

### Craniocervical instability in patients with hypermobility syndrome: A surgical condition

#### A. Jenkins ^1^; J. O'Donnell^2^; C. Harvie^2^; R. Chung^3^

##### ^1^Mount Sinai Hospital; ^2^Jenkins NeuroSpine; ^3^University of Virginia Medical School

**Background and aims:** Craniocervical instability (CCI) is increasingly found to manifest from heritable connective tissue disorders. Hypermobile Ehlers‐Danlos Syndrome (hEDS) causes ligamentous laxity leading to an abnormal range of motion in joints, including the craniocervical junction. Symptoms of CCI often present as head and neck pain in addition to symptoms of autonomic dysfunction. We hypothesize that the hyper‐rotation of the atlanto‐axial joint and hyper‐flexion of the atlanto‐occipital joint causes repetitive stress on the sympathetic chain and transient mechanical compression and stretching of the venous system and surgical treatment improves symptoms for these patients.

**Methods:** A retrospective cohort study was performed on surgical patients diagnosed with hypermobile Ehlers‐Danlos syndrome, neck/head pain and symptoms of autonomic dysfunction, and radiographic findings of CCI at the O‐C1, C1‐2 or O‐C2 levels.

**Results:** Twelve patients underwent posterior cervical fusion at the level indicated for treatment (O‐C1: n=3, C1‐2: n=6, O‐C2: n=3). Radiologic findings showed an average C1‐2 angular displacement of 34 degrees in each direction. Clinical presentation included head and neck pain (100%), headaches (100%), dizziness (75%), POTS symptoms, (50%) vision disturbances (42%), brain fog (50%), vertigo (33.3%), tinnitus (42%), and gastrointestinal dysfunction (33.3). Post‐operatively, patients had significant improvement in overall (p=0.01), head (p=0.01), and neck (p=0.003) pain on the VAS. Post‐operatively, all 12 patients reported improvement in their neurological and autonomic symptoms (12.5 months mean follow up).

**Conclusion:** Our study demonstrates that fusion and stabilization of this junction leads to significant relief of patient's symptoms from CCI in the setting of hEDS.

**Disclosure:** Nothing to disclose.

## EPO‐194

### Early high‐efficacy disease‐modifying therapies may slow progression of autonomic dysfunction in people with MS

#### B. Ruška ^1^; I. Adamec^2^; L. Crnošija^2^; T. Gabelić^2^; B. Barun^2^; A. Junaković^2^; M. Krbot Skorić^3^; M. Habek^4^

##### ^1^Department of Neurology, Sveti Duh University Hospital, Zagreb, Croatia; ^2^Department of Neurology, University Hospital Center Zagreb, Zagreb, Croatia; ^3^Faculty of Electrical Engineering and Computing, University of Zagreb, Zagreb, Croatia; ^4^School of Medicine, University of Zagreb, Zagreb, Croatia

**Background and aims:** The research aimed to explore changes and predictors of autonomic dysfunction (AD) in people with multiple sclerosis (pwMS) from disease onset over six years.

**Methods:** A total of 121 pwMS were recruited at disease onset. After six years, data were available for 75 participants. Subjective AD was assessed with the Composite Autonomic Symptom Score‐31 (COMPASS‐31) questionnaire at the start and end of follow‐up. Objective tests of AD were performed at baseline and every two years, with results recorded using the Composite Autonomic Scoring Scale (CASS). Symptomatic dysautonomia was identified if COMPASS‐31 score was greater than the cohort median (7.913) and if CASS score was greater than 0.

**Results:** There were no significant changes in COMPASS‐31 and CASS results between the beginning and the end of follow‐up. However, a significant decline was observed in the cardiovagal index (p=0.001) and the sudomotor index (p=0.036 and p=0.001, respectively) at Years 4 and 6 compared to baseline. The number of participants with symptomatic dysautonomia increased significantly from Year 0 to Year 6 (p=0.049). Multivariable logistic regression analysis revealed that experiencing a relapse during the six years increased the likelihood of symptomatic dysautonomia by 388.6% (Exp(B) 3.886, 95% C.I. 1.019‐14.825, p=0.047). Conversely, transitioning to high‐efficacy disease‐modifying therapy (HET) reduced the probability of having a CASS score greater than 1 at Year 6 by 77.9% (Exp(B) 0.221, 95% C.I. 0.067‐0.734, p=0.014).

**Figure:**
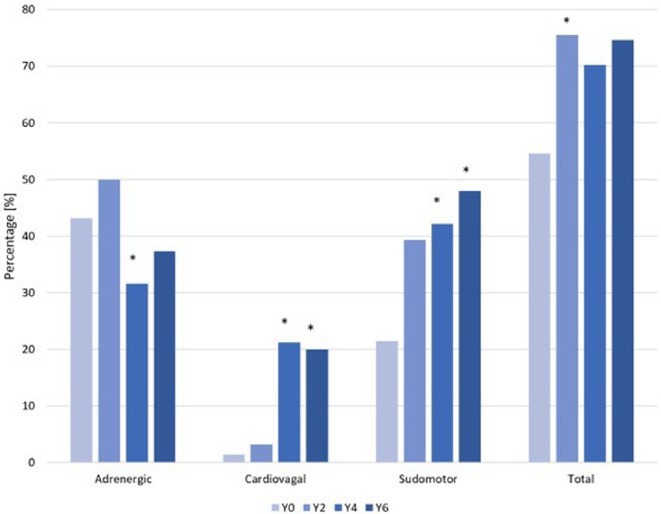
**FIGURE 1** Distribution of pathological CASS and its indices during a six‐year follow‐up. *statistically significant results in comparison to baseline for the same index

**Figure:**
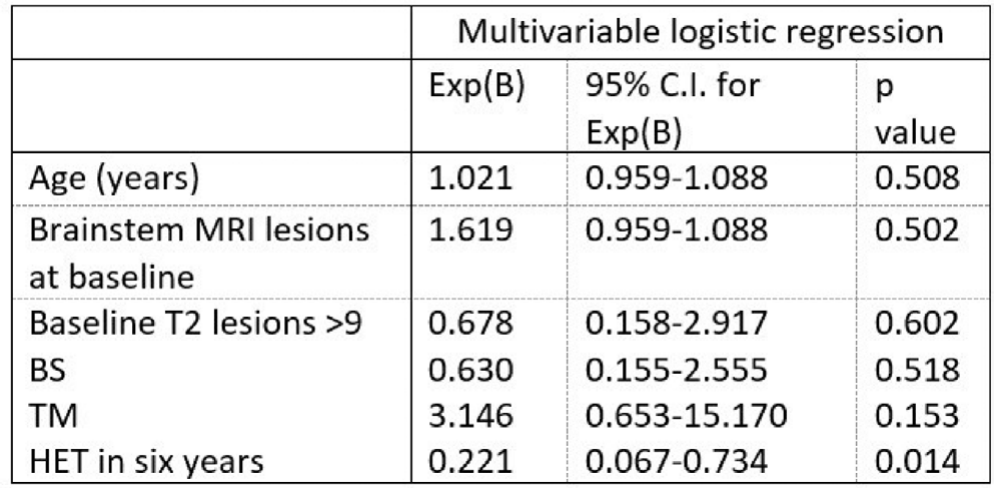
**TABLE 1** Results of the multivariable analysis identifying predictors of CASS>0 at year 6 visit.

**Conclusion:** Cardiovagal and sudomotor dysfunction progresses alongside disease duration in MS. The early initiation of HET in pwMS may reduce the risk of developing AD.

**Disclosure:** Nothing to disclose.

## EPO‐195

### The involvement of sensory and autonomic peripheral nervous system in Hypermobility Spectrum Disorder

#### D. Dell'Aversana ^1^; A. Trinchillo^1^; F. Masciarelli^1^; R. Iodice^1^; F. Vitale^1^; G. Caporaso^2^; R. Dubbioso^1^; L. Ruggiero^1^; V. Provitera^1^; S. Tozza^1^; L. Santoro^1^; F. Manganelli^1^; M. Nolano^1^; M. Nolano^2^

##### ^1^Department of Neuroscience, Reproductive Sciences and Odontostomatology, University of Naples “Federico II”, Naples, Italy; ^2^Skin Biopsy Laboratory, Department of Neurology, ICS, Istituti Clinici Scientifici Maugeri, Telese Terme, Italy

**Background and aims:** Hypermobile Spectrum Disorder (HSD) patients often have a history of sensory and autonomic symptoms. The aim of this study is to assess the peripheral involvement of sensory and autonomic nervous system in HSD.

**Methods:** 31 HSD patients (M/F=3/28; 36±14years) and 38 SFN patients (M/F=8/30; 52±14years) were recruited. Both groups underwent assessment of symptoms and sensory and autonomic dysfunction trough the “Small‐Fiber‐Neuropathy‐Symptoms‐Inventory‐Questionnaire” (SFN‐SIQ), “Composite‐Autonomic‐Symptoms‐Score” (COMPASS‐31), Quantitative Sensory Testing(QST), cardiovascular reflexes, sympathetic skin response (SSR) and Dynamic Sweat Test(DST). Cutaneous sensory and autonomic innervation was analyzed on punch biopsies from leg, thigh and fingertip applying indirect Immunofluerescence procedures.

**Results:** HSD patients were younger, with earlier onset of symptoms then SFN patients. They complained of generalized pain with involvement of the perineal region in a third of the cases. In both groups, abnormal QST for each sensory modality was observed. Autonomic symptoms involving the cardiovascular, gastrointestinal and sudomotor domains were significantly more frequent in HSD than in SFN patients. Evidence of Postural Orthostatic Tachycardia Syndrome(PoTS) was observed in half of HSD. DST showed a non‐length‐dependent reduction of sweat output per individual gland in HSD compared with SFN patients. Morphological analysis revealed a greater loss of pilomotor and sudomotor nerve fibers, with a mild non‐length‐dependent loss of epidermal nerve fibers (ENF) in HSD compared to SFN patients.

**Conclusion:** Small fibers involvement in HSD compared to SFN patients presents with generalized pain, involving perineal region and autonomic symptoms mostly involving cardiovascular and gastrointestinal domains. Morphological picture underlying this condition is a greater loss of autonomic nerves and a mild non‐length‐dependent loss of ENF.

**Disclosure:** Nothing to disclose.

## EPO‐196

### Chiari malformation and small fiber neuropathy associated with a novel COL6A5 mutation

#### F. Masciarelli ^1^; D. Dell'Aversana^1^; R. Iodice^1^; F. Vitale^1^; G. Caporaso^2^; R. Dubbioso^1^; L. Ruggiero^1^; V. Provitera^2^; S. Tozza^1^; L. Micale^3^; L. Santoro^1^; F. Manganelli^1^; M. Castori^3^; M. Nolano^1^

##### ^1^Department of Neurosciences, Reproductive Sciences and Odontostomatology, University Federico II of Naples, Naples, Italy; ^2^Neurology Department – Skin Biopsy, Lab Istituti Clinici Scientifici Maugeri, Spa SB Institute of Telese Terme ‐ IRCCS; ^3^UOC Medical Genetics, IRCCS Foundation ‐ Casa Sollievo della Sofferenza, Viale Cappuccini snc, 71013 San Giovanni Rotondo (FG), Italy

**Background and aims:** We describe a patient and her daughter with Chiari Malformation type‐I (CMI) and painful symptoms in which a COL6A5 gene mutation was found.

**Methods:** Patient and her daughter underwent Next‐Generation‐Sequencing with a gene panel for hereditary connective tissue disease. Variant detected was confirmed by Sanger sequencing. Patient underwent assessment of neurological symptoms and autonomic dysfunction trough the “Small‐Fiber‐Neuropathy‐Symptoms‐Inventory‐Questionnaire” (SFN‐SIQ), “Composite‐Autonomic‐Symptoms‐Score” (COMPASS‐31), cardiovascular reflexes, sympathetic skin response (SSR) and Dynamic Sweat Test (DST). Cutaneous sensory and autonomic innervation and Collagen VI presence in extracellular matrix were analyzed on punch biopsies from leg, thigh and fingertip applying indirect immunofluorescence procedures.

**Results:** Patient (57‐year‐old woman) and her daughter (33‐years‐old) had CMI. Both genetic analysis pointed out a heterozygous 5 bp‐deletion COL6A5 variant, predicted to introduce a premature stop codon. They complained diffuse and chronic musculoskeletal and burning pain, itchy scalp, allodynia and autonomic symptoms involving cardiovascular, gastrointestinal and sudomotor domains. Patient autonomic assessment showed a Postural Orthostatic Tachycardia Syndrome (PoTS) and a non‐length‐dependent reduction of sweat gland density and sweat output per gland at DST. Morphological analysis revealed a severe loss of sensory and autonomic nerve fibers. Collagen VI staining was reduced in extracellular matrix compared to healthy subject.

**Figure:**
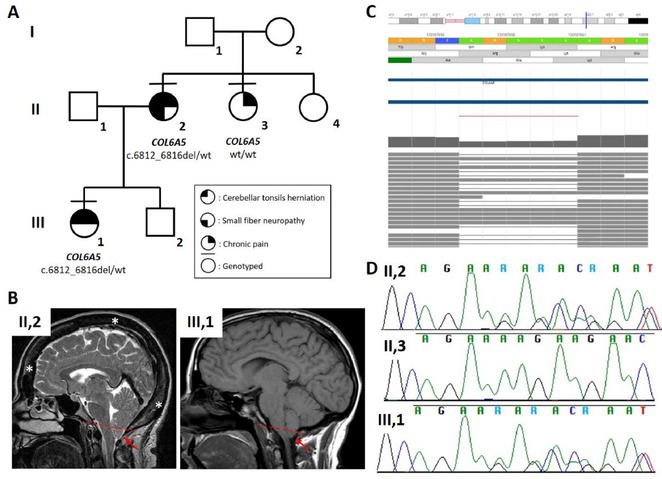
**FIGURE 1** Family, radiological and molecular features of the family.

**Figure:**
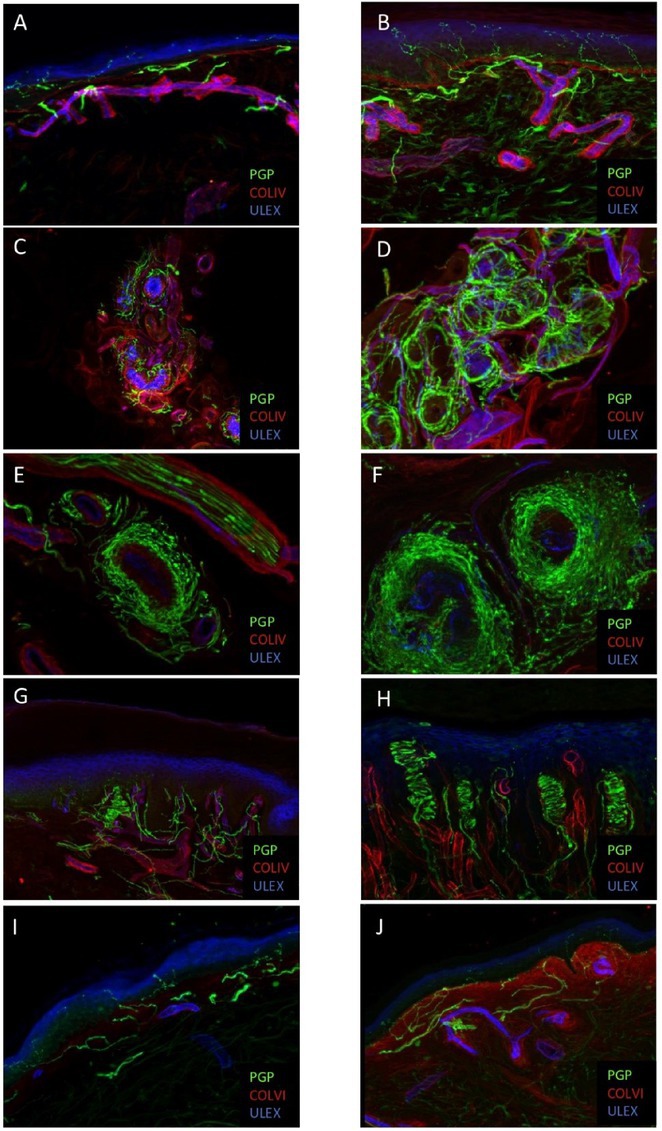
**FIGURE 2** Digital confocal images showing epidermal and dermal denervation in our patient (A, C, E, G, I) compared to a healthy control (B, D, F, H, J)

**Conclusion:** Patient received a small fibers neuropathy (SFN) diagnosis. The reduced collagen VI expression in extracellular matrix could indicate the pathogenicity of this variant. Mutation in the same gene locus has been described associated to CMI or to chronic itch, suggesting that the variant we found is likely pathogenic for a syndrome that associate both SFN and CMI.

**Disclosure:** Nothing to disclose.

## EPO‐197

### Characterizing sudomotor dysfunction in multiple sclerosis: Insights from QSART, SUDOSCAN, and COMPASS‐31

#### K. Jerčinović^1^; I. Adamec ^1^; T. Gabelić^1^; B. Barun^1^; M. Krbot Skorić^2^; M. Habek^1^

##### ^1^School of Medicine, University of Zagreb, Zagreb, Croatia; ^2^Department of Neurology, University Hospital Center Zagreb, Zagreb, Croatia

**Background and aims:** This study aimed to characterize sudomotor dysfunction in people with MS (pwMS) using the Quantitative Sudomotor Axon Reflex Test (QSART) and SUODSCAN.

**Methods:** Forty‐one consecutive treatment naïve pwMS were enrolled within 5 years from symptom onset. Symptoms of sudomotor dysfunction were evaluated with question 8 of the Composite Autonomic Symptom Score (COMPASS‐31). The sudomotor function was assessed with QSART, a test that measures the axon‐reflex‐mediated evaporated sweat response, and SUDOSCAN, which measures the electrochemical skin conductance of hands and feet through reverse iontophoresis.

**Results:** Symptomatic hyperhidrosis was present in 15 (36.6%) pwMS. Pathological result of the QSART (sudomotor index (SI) >0) was found in 11 (26.8%) pwMS, while SUDOSCAN results were pathological in 5 (12.20%) pwMS. pwMS with symptomatic hyperhidrosis had higher values on SUDOSCAN leg (83.00±5.26 vs 78.92±6.22, p=0.039), while there was no difference in the QSART results. There was no correlation between the results of the QSART and SUDOSCAN. However, pwMS with hypohidrosis on the QSART had the lowest results on the SUDOSCAN (Figure 1a and 1b). In contrast, pwMS with persistent hyperhidrosis or persistent sweating on the QSART had higher values on the SUDOSCAN (Figure 1a and 1b). pwMS with lesions present on the brainstem and spinal cord MRI had lower volumes on QSART compared to pwMS without lesions on both locations (distal leg 0.92±0.611 vs 0.92±0.611.65±1.46, p=0.037).

**Figure:**
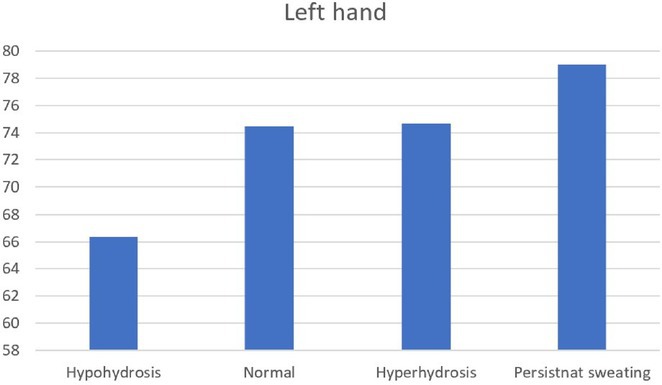
**FIGURE 1** Results of SUDOSCAN based on QSART

**Figure:**
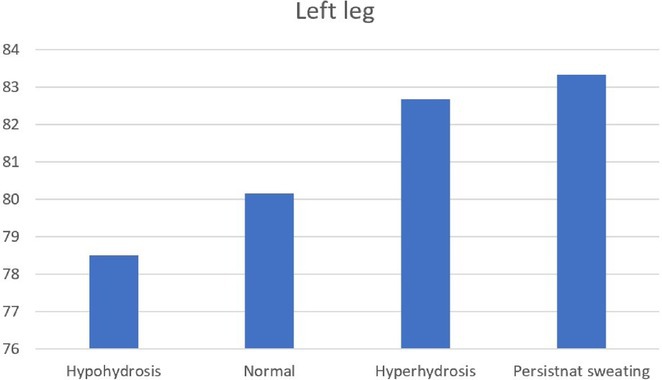
**FIGURE 2** Results of SUDOSCAN based on QSART

**Conclusion:** pwMS frequently have sudomotor dysfunction measured with COMPASS‐31, QSART, and SUDOSCAN. Detection of sudomotor problems in pwMS with different methodologies indicates differences in causes of sudomotor dysfunction in pwMS.

**Disclosure:** Funding: Croatian Science Foundation (IP‐2019‐10‐8200) Financial & competing interest disclosure KJ: Nothing to disclose. IA, TG, BB, MH: Received consultation and/or speaker fees from Biogen, Merck, Novartis, Roche, Astra Zeneca, Amgen. MKS: Received consultation and/or speaker fees from Roche.

## EPO‐198

### Monocular dynamic pupillometry in healthy controls and patients with autonomic dysfunction: a technical validation study

#### L. Sander ^1^; G. Oommen^2^; C. Brophy^2^; S. Bohus‐Roper^2^; G. Chiaro^2^; F. Bremner^3^; V. Iodice^4^

##### ^1^Autonomic Unit, NHNN, London, UK; Department of Brain, Repair and Rehabilitation, UCL Institute of Neurology, London, UK; Neurologic Clinic and Policlinic, Departments of Medicine and Clinical Research, University Hospital Basel and University of Basel,CH; ^2^Autonomic Unit, The National Hospital for Neurology and Neurosurgery, London, UK; ^3^Department of Neuro‐ophthalmology, The National Hospital for Neurology and Neurosurgery, London, UK; ^4^Autonomic Unit, The National Hospital for Neurology and Neurosurgery, London, UK; Department of Brain, Repair and Rehabilitation, University College London Queen Square Institute of Neurology, London, UK

**Background and aims:** Pupillometry is widely used to determine pupil diameter for corneal refractive surgery. Monocular devices are non‐invasive tools to assess pupillary parameters including ocular parasympathetic and sympathetic function. This study aims to generate normative pupillomotor autonomic values in healthy controls (HC) and to technically validate a handheld pupillometer.

**Methods:** 40 HC had monocular pupillometry using the PLR‐4000®(NeurOptics). Twelve patients with autonomic disorders additionally underwent binocular pupillometry (Neuroptics DP2000). Pupillary parasympathetic and sympathetic function were assessed by responses to light stimulus and to 0.5% apraclonidine eye drops, respectively. 4 HC had repeat assessment at a second timepoint.

**Results:** In HC, mean light reflex ratio was 42 ± 5.7% and median response to apraclonidine was ‐5.0 (‐8.8 – 2.8)%. Younger controls had larger mean resting pupils than older individuals (p=0.001, 95% CI = 0.4 – 1.4). There was no age‐related difference in mean light response (p=0.355). Results of normal pupillary function, parasympathetic, sympathetic, or combined denervation were comparable as assessed by the two pupillometers. Intra‐individual repeatability showed: median difference in resting pupil size 0.5mm (IQR 0.13 – 0.93mm), median light response difference 2 (1 – 4)%, median % difference in response to apraclonidine 8.4 (4.7 – 13.6)%.

**Conclusion:** The presented device provides accurate and reproducible assessments of pupillary parasympathetic and sympathetic function in healthy controls and patients with autonomic disorders. With normative data provided, it is a well‐tolerated, easily accessible tool to quantitatively assess autonomic ocular innervation. Further studies are needed to investigate its clinical use for autonomic screening and monitoring disease progression.

**Disclosure:** LS is supported by the University of Basel, Switzerland, and the Freiwillige Akademische Gesellschaft Basel. GO, CB, SB, GC, FB report no disclosures. VI is supported by the National Institute for Health Research, University College London Hospitals Biomedical Research Centre and by the Autonomic Charitable Trust (ACT) (Lord Bagri) Research Award. VI has received honoraria from Theravance Biopharma not related to this work.

## EPO‐199

### Clinical validation of autonomic ocular function assessments using monocular dynamic pupillometry

#### L. Sander ^1^; G. Oommen^2^; C. Brophy^2^; S. Bohus‐Roper^2^; F. Bremner^3^; V. Iodice^4^

##### ^1^Autonomic Unit, NHNN, London, UK; Department of Brain, Repair and Rehabilitation, UCL Institute of Neurology, London, UK; Neurologic Clinic and Policlinic, Departments of Medicine and Clinical Research, University Hospital Basel and University of Basel,CH; ^2^Autonomic Unit, The National Hospital for Neurology and Neurosurgery, London, UK; ^3^Department of Neuro‐ophthalmology, The National Hospital for Neurology and Neurosurgery, London, UK; ^4^Autonomic Unit, The National Hospital for Neurology and Neurosurgery, London, UK; Department of Brain, Repair and Rehabilitation, University College London Queen Square Institute of Neurology, London, UK

**Background and aims:** Pupillary function is frequently impaired in disorders affecting the autonomic nervous system. Dynamic pupillometry allows us to quantitatively evaluate ocular parasympathetic and sympathetic innervation. Monocular, handheld devices are easily accessible tools to assess various pupillary parameters. The aims of this study were to clinically validate the handheld pupillometer PLR‐4000® (NeurOptics) in patients with autonomic dysfunction.

**Methods:** In this prospective study, 100 patients with autonomic failure and intermittent autonomic disorders underwent pupillometry from April – December 2024 using the PLR‐4000® (NeurOptics). Pupillary parasympathetic and sympathetic function were assessed by responses to light stimulus and to 0.5% apraclonidine eye drops, respectively.

**Results:** In patients with neurodegenerative disorders (n=24), autonomic neuropathies (n=39), and autonomic ganglionopathies (n=9), pupillary abnormalities were very prevalent (52%, 45%, and 100%, respectively). In patients with alpha‐synucleinopathies, sympathetic denervation was the most common abnormality (9/21; 43%). 3/6 patients with autoimmune autonomic ganglionopathy presented with pupillary fatigue. All patients with intermittent autonomic disorders (n=28) presented with normal pupillary function.

**Conclusion:** Monocular dynamic pupillometry robustly measures pupillary parasympathetic and sympathetic function in patients with autonomic disorders. It is an easily accessible and valuable tool to quantitatively assess autonomic ocular innervation in addition to cardiovascular autonomic function testing. Further studies are needed to investigate its use as a tool for early autonomic screening, monitoring disease progression and response to treatment in different disorders.

**Disclosure:** LS is supported by the University of Basel, Switzerland, and the Freiwillige Akademische Gesellschaft Basel. GO, CB, SB, GC, FB report no disclosures. VI is supported by the National Institute for Health Research, University College London Hospitals Biomedical Research Centre and by the Autonomic Charitable Trust (ACT) (Lord Bagri) Research Award. VI has received honoraria from Theravance Biopharma not related to this work.

## EPO‐200

### Gastrointestinal motility and ANS function in children with inflammatory bowel disease and irritable bowel syndrome

#### A. Mocic Pavic^1^; P. Ruska^1^; M. Krbot Skoric^2^; M. Habek ^3^; I. Hojsak^1^

##### ^1^Children's Hospital Zagreb, Department of Pediatrics, Zagreb, Croatia; ^2^University Hospital Center Zagreb, Zagreb, Croatia; ^3^University of Zagreb, School of Medicine, Zagreb, Croatia

**Background and aims:** Electrogastrography (EGG) is a noninvasive method for the measurement of gastric myoelectrical activity that uses abdominal surface electrodes. This study aimed to investigate the differences in gastric motility measured with EGG and its correlation with standardized autonomic nervous system tests in children with inflammatory bowel disease (IBD), irritable bowel syndrome (IBS), and healthy children (HC).

**Methods:** Sixty‐two children were enrolled: 18 in the IBD, 26 in the IBS, and 18 in the HC group. ANS symptoms were evaluated with the Composite Autonomic Symptom Score (COMPASS‐31). COMPASS31 >7.913 was considered a clinically significant autonomic symptom burden. The severity and distribution of ANS function were quantitated using adrenergic, cardiovagal, and sudomotor indices of the Composite Autonomic Severity Scale (CASS). Gastric myoelectric activity was obtained from EGG in the preprandial and postprandial periods (standardized meal 300 kcal).

**Results:** There was no statistically significant difference in any of the EGG parameters between groups (all p>0.05). Autonomic symptom burden measured with COMPASS‐31 score negatively correlated with postprandial PDF (r=‐0.316, p=0.02). The gastrointestinal domain of the COMAPSS‐31 did not correlate with PDF (p>0.05). However, the orthostatic intolerance domain of the COMAPSS‐31 negatively correlated with postprandial PDF (r=‐0.364, p=0.007). Children with clinically significant autonomic symptom burden had lower values of postprandial PDF (2.8 (IQR 0.19) vs 2.9 (IQR 0.23), p=0.016). CASS and its indices did not correlate with any of the EGG parameters.

**Conclusion:** These findings indicate that children with higher and clinically significant autonomic symptom burden postprandially have lower levels of gastric activity.

**Disclosure:** AMP: Nothing to disclose. PR: Nothing to disclose. MKS: Received consultation and/or speaker fees from Sanofi Genzyme, Roche. MH: Received consultation and/or speaker fees from Biogen, Merck, Novartis, Roche, Astra Zeneca, Amgen. IH: Received honoraria for lectures from Sandoz, BioGaia, Ewopharma, Oktalpharma, Hipp, Nutricia, Biocodex, Nestle, and GM Pharma.

## EPO‐201

### Ultrasound assessment of peripheral nerve size in Guillain‐Barré syndrome: A systematic review and meta‐analysis

#### O. Alomari ^1^; B. Alrabadi^2^; T. A. Hussein^3^; R. Tawalbeh^1^; S. Shtayat^1^; R. Alnahdi^4^; R. A. Hussein^5^; A. Elgenidy^6^

##### ^1^Hamidiye International School of Medicine, University of Health Sciences, 3400, Istanbul Turkey; ^2^Faculty of Medicine, Jordan University of science and technology, IRBID, Jordan; ^3^Faculty of Medicine, Assiut University, Assiut, Egypt; ^4^Faculty of Medicine, Cairo University, Cairo, Egypt; ^5^Faculty of Medicine, Assiut University, Assiut, Egypt; ^6^Department of Neurology, Cairo University, Cairo 11652, Egypt

**Background and aims:** Guillain‐Barré syndrome (GBS) is an acute autoimmune disorder characterized by progressive muscle weakness and paralysis due to peripheral nerve damage. Changes in nerve size may serve as important biomarkers of nerve involvement. Ultrasound (US) has emerged as a non‐invasive tool for assessing peripheral nerve changes, including alterations in nerve size. This study aims to evaluate peripheral nerve size changes in patients with GBS using US.

**Methods:** A systematic literature search was conducted in PubMed, Scopus, Embase, and the Web of Science from inception until September 2024. Data extraction was performed using a standardized form.

**Results:** A total of 26 studies with 1,462 patients were identified, of which 18 were included in the analysis. Significant nerve size changes (p < 0.05) were observed across multiple anatomical regions, including the Cervical, Fibular, Median, Peroneal, Sural, Tibial, and Ulnar nerves. The most notable changes were found in the Tibial nerve at the popliteal region (MD 6.23, 95% CI 3.6 to 8.86), the Peroneal nerve (MD 2.09, 95% CI 1.31 to 2.88), and the Median nerve in the upper arm (MD 1.94, 95% CI 1.14 to 2.74) and forearm (MD 1.62, 95% CI 0.71 to 2.53).

**Figure:**
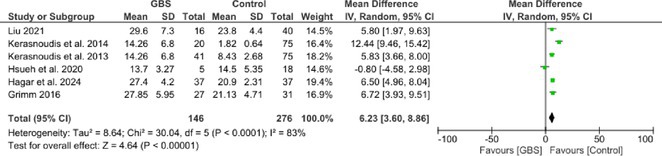
**FIGURE 1** Mean difference (MD) in nerve size for the Tibial nerve at the popliteal region in patients with Guillain‐Barré syndrome.

**Figure:**
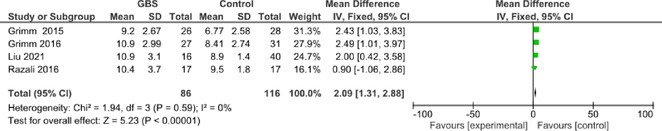
**FIGURE 2** Mean difference (MD) in nerve size for the Peroneal nerve in patients with Guillain‐Barré syndrome.

**Figure:**
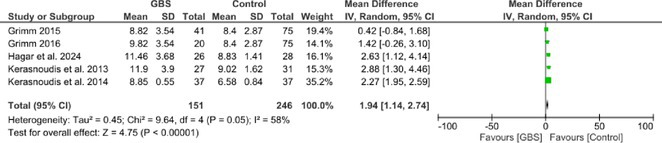
**FIGURE 3** Mean difference (MD) in nerve size for the Median nerve in the upper arm and forearm regions in patients with Guillain‐Barré syndrome.

**Conclusion:** US imaging reveals significant nerve size changes in multiple anatomical regions in GBS patients. These findings underscore the potential of US as a non‐invasive tool for monitoring nerve alterations and disease progression in GBS.

**Disclosure:** Nothing to disclose.

## EPO‐202

### The therapeutic effect of transcranial alternating current stimulation on persistent postural perceptual dizziness

#### W. Liu ^1^; Q. Liu^1^; A. Liu^2^

##### ^1^Beijing Fengtai You'anmen Hospital; ^2^Xuanwu Hospital, Capital Medical University

**Background and aims:** Persistent postural perceptual dizziness (PPPD) is a disease with chronic vestibular dysfunction as its main manifestation. There is no standardized treatment for PPPD, but transcranial alternating current stimulation (tACS) is a non‐invasive neuromodulation technique. Previous study using transcranial direct current stimulation (tDCS) at left dorsolateral prefrontal anode showed a significant reduction in DHI scores and some reduction in HAMD score; however, no study has studied the outcomes of tACS to PDDD. The aim of this study was to apply tACS for the treatment of PPPD and to investigate its therapeutic effect.

**Methods:** A total of 10 patients with PPPD were recruited in this study. The effect of tACS for PPPD was assessed by applying AC stimulation with a current size of 1.5 mA and a frequency of 10 Hz to five electrodes in the dorsolateral left prefrontal lobe of the subjects, and assessing the subjects' vertigo level, anxiety and depression status.

**Figure:**
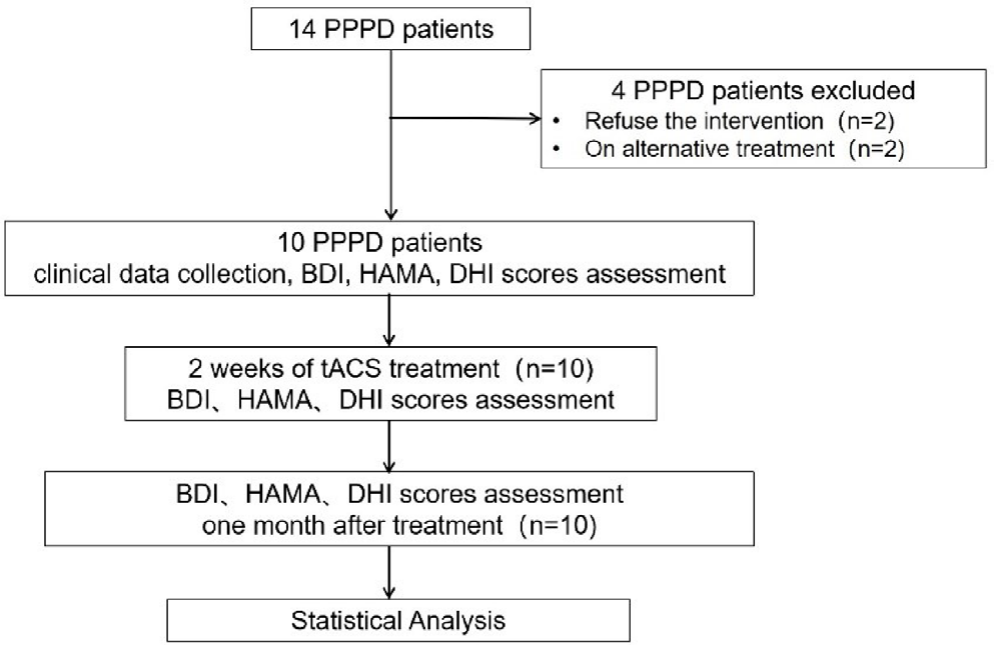
**FIGURE 1** Study flowchart

**Results:** Three scales were significant compared among the three groups at the baseline level before tACS, after tACS, and after one month of tACS, indicating that they were statistically different before and after the treatment, proving that there was a significant improvement in vertigo level, depressive state and anxiety state in the PPPD patients included before and after tACS.

**Figure:**
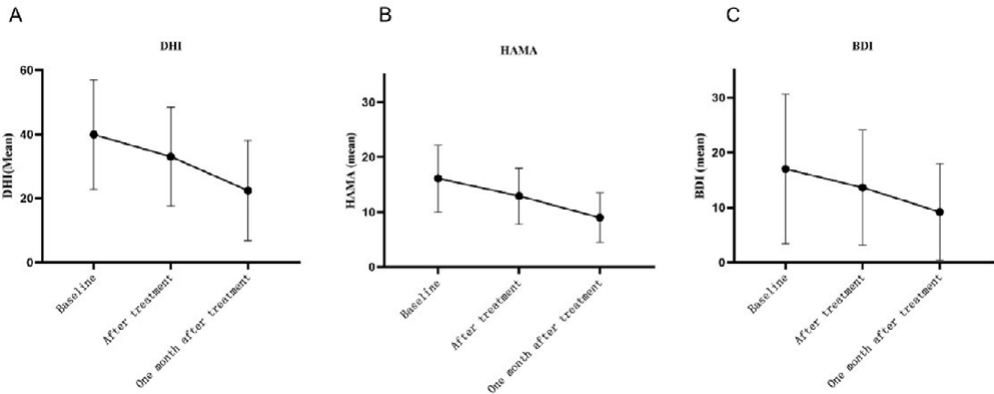
**FIGURE 2** BDI, HAMA and DHI scores changes after treatment

**Figure:**
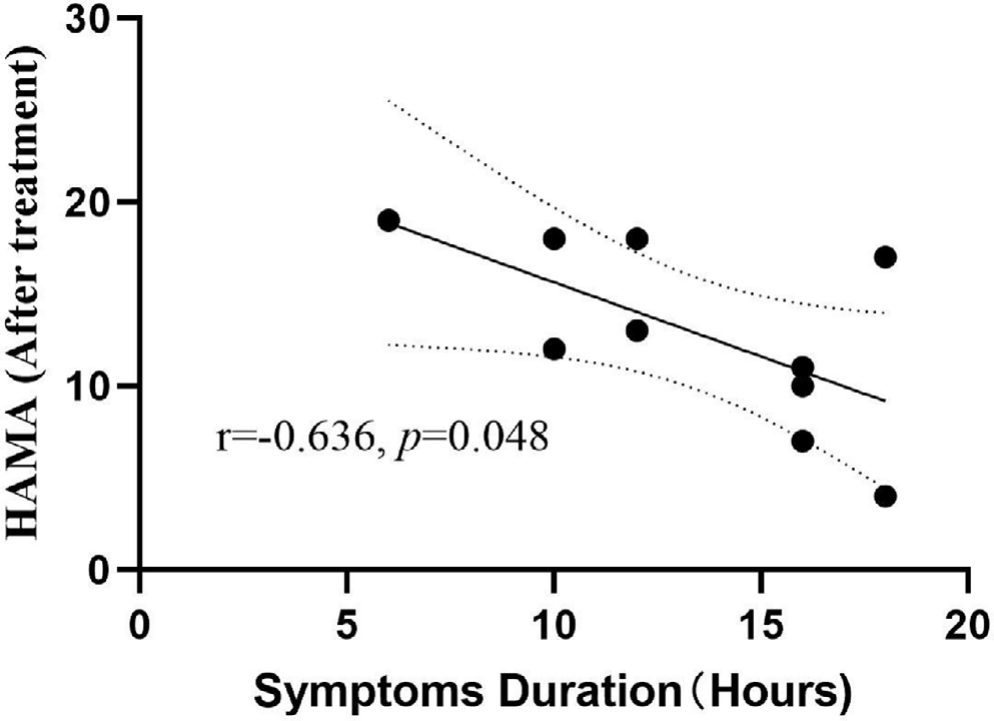
**FIGURE 3** Correlation between HAMA and symptoms duration after treatment

**Conclusion:** This study supports the application of tACS in PPPD patients to improve their anxiety and depression symptoms. Meanwhile, the results of this study suggest that DLPFC is involved in the pathogenesis of PPPD and may be a target for further research.

**Disclosure:** Nothing to disclose.

## EPO‐203

### Delayed orthostatic tachycardia – is the time frame for postural orthostatic tachycardia syndrome arbitrary?

#### Z. Siddiqi

##### University Of Alberta, Edmonton Alberta, Canada

**Background and aims:** Postural Orthostatic Tachycardia Syndrome (POTS) is defined as increase in heart rate by >30 bpm within 10 minutes of upright posture without significant orthostatic hypotension (OH). The basis of this time frame remains unclear. Patients who develop delayed orthostatic tachycardia (DOT) in the absence OH remain uncharacterized. The present study was aimed at defining the characteristics of the DOT group.

**Methods:** We reviewed clinical histories and laboratory tests performed in our laboratory for assessment of orthostatic intolerance (OI) in last 10 years. Laboratory tests included autonomic testing (sweat test, heart rate variability tests, and 45‐minute upright tilt table test) and quantitative sensory testing.

**Results:** Among 974 patients who underwent laboratory tests most common referral symptoms were orthostatic palpitations/tachycardia (49.3%), syncope (18.7%) and light‐headedness (7.3%). Among this cohort, 419 (43.0% ) had POTS, and 167/974 (17.1%) had DOT manifesting as onset/aggravation of presyncopal symptoms, with a mean HR increase by 49.7 bpm from baseline (Range: 40 BPM‐ 103 BPM) without OH, narrowing of pulse pressure, (x̄= 15.4, Range: 9.2 mm Hg‐ 24.8 mm Hg), and syncope (10.8%). Among the patients with delayed OI, 64.7% had small fiber/autonomic neuropathy.

**Figure:**
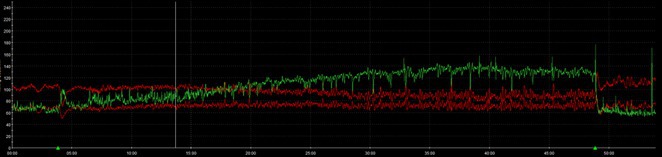
**FIGURE 1** A 45min upright TTT in a 35‐year‐old female with orthostatic symptoms shows delayed orthostatic tachycardia (green) without any significant orthostatic hypotension (red). The green triangles mark the start and ending of tilt test whereas the vertical line shows

**Conclusion:** A significant number of patients with OI have DOT on TTT without significant OH. Reduction in PP and small fiber/autonomic neuropathy in this group suggests that reduced cardiac output due to peripheral blood pooing causes DOT, which may be on the continuum of POTS and should be ruled out with appropriate testing protocols.

**Disclosure:** Nothing to disclose.

## Cerebrovascular Diseases 2

## EPO‐204

### Tenecteplase administration after the usual treatment window in acute ischemic stroke: A meta‐analysis

#### M. Ifzal^1^; S. Afzal^2^; S. Rizvi^3^; M. Muzaffar^4^; R. Ali^5^; M. Ikram^6^; M. Murtaza^7^; A. Mirza^8^; H. Ans^9^; L. Bucataru^10^; A. Ans^11^; R. Ahmed^12^; M. Ahmed ^13^; M. Ayyan^14^; G. Imbianozor^15^; A. Alareed^16^; M. Rehman^17^

##### ^1^Acute Medicine Unit, University Hospital of North Midlands, UK; ^2^Department of Medicine, Ziauddin Medical University, Karachi, Pakistan; ^3^Department of Medicine, Jinnah Medical and Dental College, Karachi, Pakistan; ^4^Jinnah Hospital Lahore, Pakistan; ^5^Liaquat National Hospital and Medical College, Karachi; ^6^Department of Medicine, Frontier Medical and Dental College, Abbottabad, Pakistan; ^7^Civil Hospital Sanghar, Sindh, Pakistan; ^8^Department of Medicine, Jinnah Medical and Dental college, Karachi, Pakistan; ^9^FMH College of Medicine and Dentistry, Pakistan, ^10^NHS Trust, UK, ^11^Department of Vascular Neurology, University of Pittsburgh Medical Center (UPMC), Pittsburgh, Pennsylvania (PA), ^12^Royal Brompton Hospital, Part of Guy's and St Thomas’ NHS Foundation Trust, UK, ^13^Department of Medicine, Rawalpindi Medical University, Rawalpindi, Pakistan, ^14^Department of Medicine, King Edward Medical University, Lahore, Pakistan, ^15^Royal Wolverhampton NHS Trust. New Cross Hospital, Wolverhampton, ^16^University Hospital Southampton NHS Foundation Trust, ^17^Department of Neurology, University of Alabama, Alabama, USA

**Background and aims:** Data regarding the efficacy and safety of tenecteplase (TNK) in patients with acute ischemic stroke (AIS) who present outside the standard treatment window are limited. This study aims to evaluate the role of TNK at a dose of 0.25 mg/kg, in treating AIS patients in an extended time window.

**Methods:** Searches were performed in PubMed, Scopus, Embase, and Cochrane CENTRAL to include randomized‐controlled trials (RCTs) comparing TNK (0.25 mg/kg) to no thrombolysis in AIS patients presenting after 4.5 hours of symptom onset or wake‐up AIS. The primary efficacy outcomes included a 3‐month excellent functional outcome (mRS ⩽1), and a good functional outcome (mRS ⩽2). Secondary safety outcomes assessed included symptomatic intracranial hemorrhage (sICH), and 3‐month all‐cause death. A random‐effects model was used to calculate summary estimates,

**Results:** 4 RCTs were included (n = 1632 patients) in the meta‐analysis. The pooled analysis demonstrated a significantly improved excellent functional outcome on 90 days (OR = 1.38, 95% CI: 1.12 to 1.69) and good functional outcome (OR = 1.23, 95% CI: 1.01 to 1.50) with TNK administration compared to control. No statistically significant association was observed for the two groups regarding all‐cause death (OR = 1.11, 95% CI: 0.82 to 1.50) and sICH (OR = 2.02, 95% CI: 0.93 to 4.38).

**Figure:**
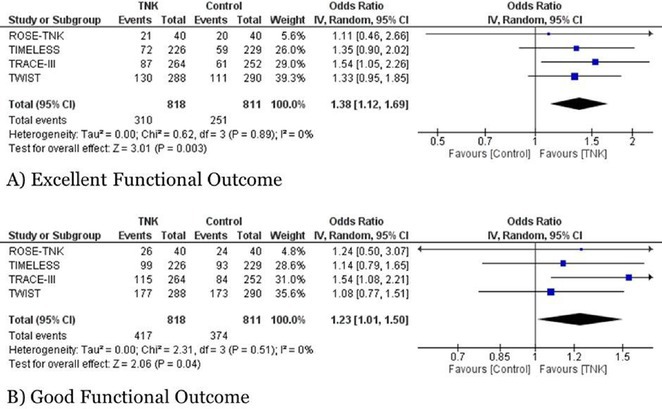
**FIGURE 1** Forest plots for (A) Excellent functional outcome and (B) Good functional outcome.

**Figure:**
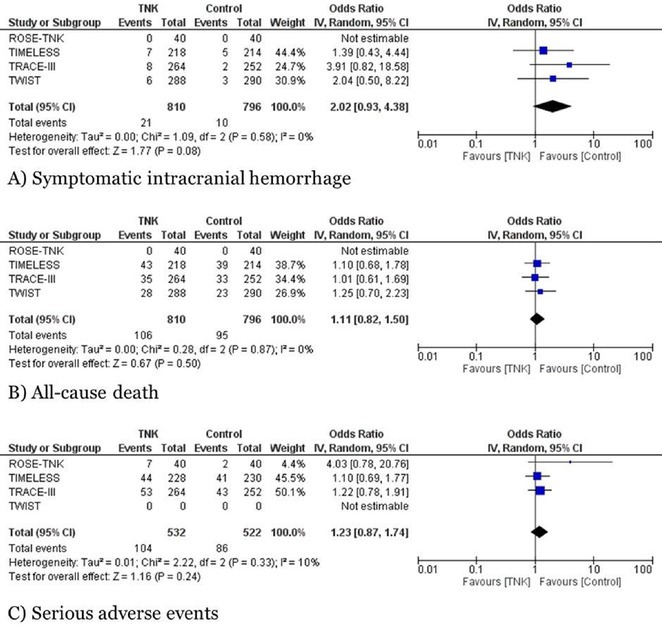
**FIGURE 2** Forest plots for (A) Symptomatic intracranial hemorrhage, (B) All‐cause death, and (C) Serious adverse events.

**Conclusion:** TNK administration outside the conventional treatment window in AIS patients leads to favorable neurological outcomes with a good safety profile.

**Disclosure:** Nothing to disclose.

## EPO‐205

### Recombinant human pro‐urokinase vs. alteplase within 4.5 hours of acute ischemic stroke: A meta‐analysis

#### T. Hashmi^1^; R. Zia^1^; A. Shafiq^2^; H. Ashraf^1^; M. Burhan^1^; M. Ahmed^1^; R. Ahmed^3^; G. Imbianozor^4^; A. Alareed ^5^; A. Mesmar^6^

##### ^1^Rawalpindi Medical University, Pakistan; ^2^Dow Medical College, Pakistan; ^3^Royal Brompton Hospital, London, UK; ^4^Royal Wolverhampton NHS Trust. New Cross Hospital, Wolverhampton; ^5^University Hospital Southampton NHS Foundation Trust; ^6^Sheikh Shakhbout Medical City

**Background and aims:** Recombinant human pro‐urokinase (rhPro‐UK) has emerged as a potential alternative to alteplase for patients with acute ischemic stroke (AIS) presenting within 4.5 hours of symptom onset. This meta‐analysis evaluates and compares the efficacy and safety of rhPro‐UK with alteplase in this patient population.

**Methods:** A comprehensive search was conducted on PubMed, Cochrane and EMBASE to find eligible RCTs comparing rhPro‐UK with r‐tPA in AIS patients treated within 4.5 hours of symptom onset. A random‐effects meta‐analysis was conducted using RevMan Web.

**Results:** Three RCTs encompassing 2,289 patients (rhPro‐UK: 1141; r‐tPA: 1148) met the inclusion criteria. The pooled analysis demonstrated no significant difference between rhPro‐UK and alteplase in achieving excellent functional outcome (mRS 0‐1 at 90d: RR = 1.04, 95% CI = 0.98 to 1.10; P = 0.17) and good excellent functional outcome (mRS 0‐2 at 90d: RR = 1.0, 95% CI = 0.96 to 1.05; P = 0.86). No statistically significant difference was observed for early neurological improvement (RR 1.05, 95% CI 0.96 to 1.15), symptomatic intracranial hemorrhage (RR = 0.52, 95% CI = 0.19 to 1.43), all‐cause mortality (RR 1.10, 95% CI 0.64 to 1.91) and severe adverse events (RR = 0.92, 95% CI = 0.75 to 1.13).

**Figure:**
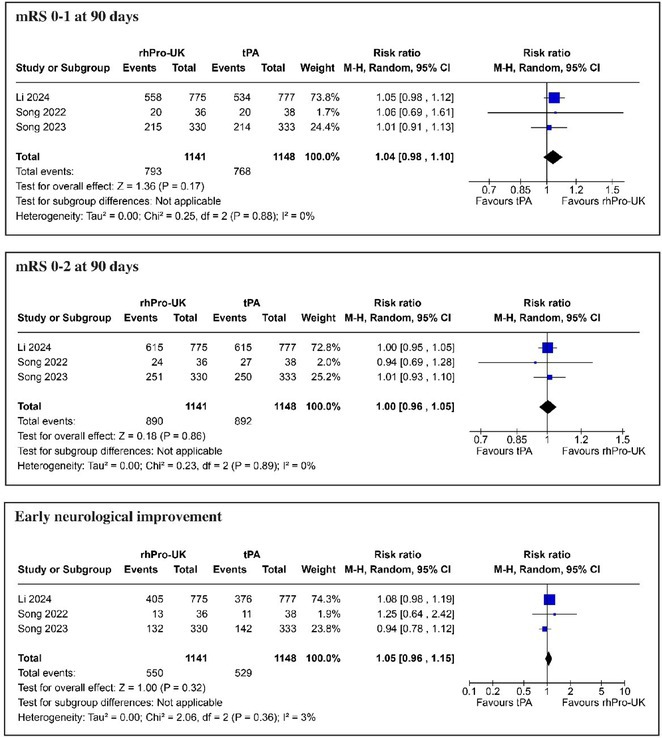
**FIGURE 1** Forest plots for (A) Excellent Functional Outcome, (B) Good functional Outcome, and (C) Early Neurological Improvement.

**Figure:**
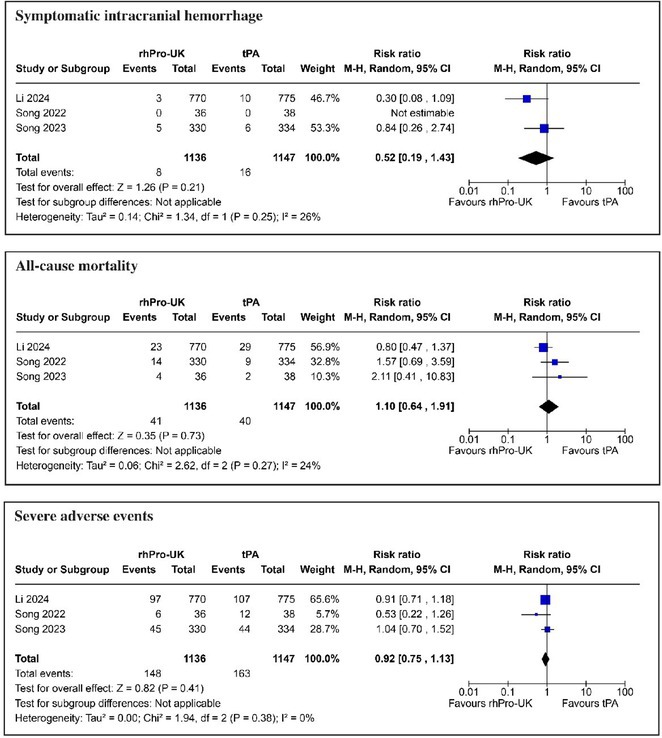
**FIGURE 2** Forest plots for (A) Symptomatic intracranial hemorrhage (B) All‐cause mortality, and (C) Serious adverse events.

**Conclusion:** This meta‐analysis found no significant differences between rhPro‐UK and alteplase in functional recovery, early neurological improvement, or safety outcomes, including symptomatic intracranial hemorrhage and all‐cause mortality. rhPro‐UK shows promise as a cost‐effective alternative, but further large‐scale RCTs are required to confirm its role in AIS management.

**Figure:**
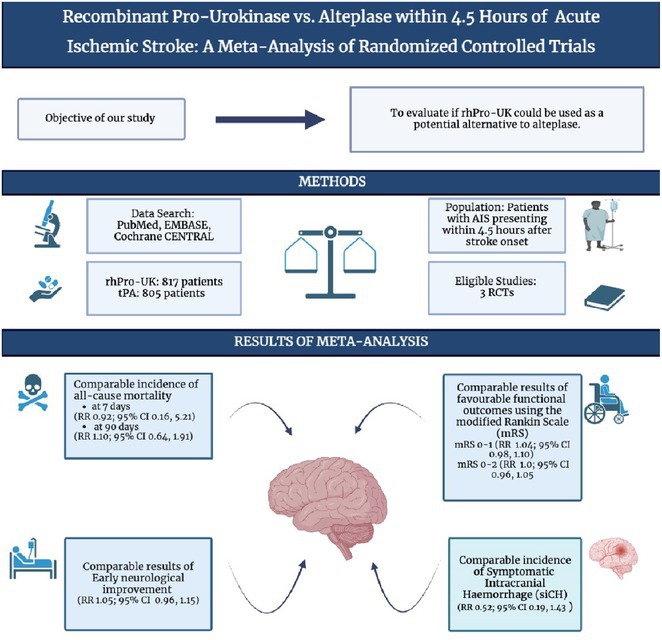
**FIGURE 3** Graphical abstract

**Disclosure:** Nothing to disclose.

## EPO‐206

### Paracentral Acute Middle Maculopathy: a retinal stroke which strokeologists should know about

#### D. Damas ^1^; S. Matos^1^; A. Jorge^1^; A. Martins^2^; G. Santo^1^; F. Silva^1^; J. Lemos^1^

##### ^1^Neurology Department, Coimbra University Hospital, Coimbra, Portugal; ^2^Faculty of Medicina, University of Coimbra, Coimbra, Portugal

**Background and aims:** Paracentral acute middle maculopathy (PAMM) represents a unique subtype of retinal stroke that has been associated to vascular risk factors, including carotid disease and microvascular retinopathy. It typically causes sudden monocular vision loss with strikingly normal fundoscopic examination. Only when performing optical coherence tomography (OCT), will one confirm the presence of hyperreflective bands in the inner retina indicating ischemia.

**Methods:** Case reports.

**Results:** Case 1. A 56‐year‐old female with arterial hypertension presented with acute scotoma in the right eye, without other neurological symptoms. Best corrected visual acuity (BCVA) was 16/20 on the right eye and fundus examination was unremarkable. Perimetry showed a central scotoma in the right eye. OCT revealed hyperreflective band in the inner nuclear and outer plexiform layers, consistent with PAMM. Vascular investigation showed no significant findings, and was attributed to hypertensive microvascular disease. Secondary prevention was initiated. Follow‐up showed perimetric improvement. Case 2. A 53‐year‐old male with arterial hypertension reported acute visual loss of right eye and left‐sided weakness. BCVA was 30/20 on the right eye and fundus examination was grossly normal. OCT showed hyperreflective bands in the right macula, compatible with PAMM. Vascular imaging revealed narrowing and intramural thrombus of the right internal carotid artery, suggesting carotid dissection. Antiplatelet therapy and rehabilitation was initiated.

**Conclusion:** Acute monocular vision loss with a normal fundoscopic examination can still reflect a retinal stroke, from hypertensive microvasculopathy to imminent atherosclerotic retinal artery occlusion. The detection of hyperreflective bands on OCT confirms PAMM and should prompt exclusion of cerebrovascular disease.

**Disclosure:** Nothing to disclose.

## EPO‐207

### Multiscale neural signals in seizure prediction: A comprehensive literature review and meta‐analysis

#### F. Abdelrahman ^2^; M. Mustafa^4^; A. Shariff^3^; M. Hegazy^2^; Z. Sayed^2^; N. Bekhit^2^; M. Elsayed^1^

##### ^1^MME Foundation; ^2^Newgiza University; ^3^Badr University in Cairo; ^4^Modern University for Technology and Information

**Background and aims:** Predicting epileptic seizures accurately remains a critical challenge in neuroscience, with profound implications for improving patient quality of life. This review and meta‐analysis evaluates the effectiveness of multiscale neural signal analysis in enhancing seizure prediction accuracy.

**Methods:** A systematic search of PubMed, Embase, and Cochrane Library identified studies utilizing electroencephalography (EEG), magnetoencephalography (MEG), or intracranial EEG (iEEG) data for seizure prediction. Key metrics extracted included prediction accuracy, sample size, signal processing methods, and machine learning algorithms. Meta‐analyses were performed using R to compute pooled effect sizes and confidence intervals.

**Results:** Thirty studies met inclusion criteria. Meta‐analysis demonstrated that multiscale neural signal analysis improves seizure prediction accuracy by an average of 15% (95% CI: 12%–18%) compared to traditional methods. Techniques that integrated time and frequency domain features, such as wavelet transforms and Fourier analysis, showed superior performance. Deep learning approaches, particularly long short‐term memory (LSTM) networks, achieved the highest predictive accuracy. Larger sample sizes (n > 50) and cross‐validation methods were associated with more robust outcomes.

**Conclusion:** Multiscale neural signal analysis significantly enhances seizure prediction accuracy, outperforming traditional approaches. Combining advanced signal processing with machine learning techniques, particularly deep learning, offers substantial promise for developing reliable predictive models. However, methodological standardization is needed to improve reproducibility and comparability across studies. Future research should focus on integrating multiscale signal analysis with emerging technologies to further refine and optimize seizure prediction methods.

**Disclosure:** Nothing to disclose.

## EPO‐208

### The effect of early dysphagia screening on stroke unit mortality: insights from RES‐Q registry

#### H. Ziąbska; A. Kobayashi; M. Karliński

##### Institute of Psychiatry and Neurology

**Background and aims:** Guidelines strongly recommend early dysphagia screening despite limited supporting evidence. We aimed to evaluate the effect of early dysphagia screening on hospital mortality in acute ischaemic stroke patients admitted to stroke units that perform dysphagia screening routinely.

**Methods:** We performed a retrospective analysis of patients with ischemic stroke admitted to 24 Polish stroke units from January 2022 to December 2024 and reported to the RES‐Q registry. Patients were stratified by baseline stroke severity, with adjustment for age, sex, baseline NIHSS, reperfusion therapy, and congestive heart failure.

**Results:** Of 14 788 reported to the registry, 388 were excluded due to the lack of dysphagia screening (n=53) or missing data (n=333). We observed a significant difference in the distribution of hospital mortality, favoring screening at 4‐24h after admission (388 of n=5437, 6.7%) over screening <4h (741 of n=7852, 9.4%) and screening >24h (106 of n=725, 14.6%). After stratification for stroke severity, we found that patients with baseline NIHSS 0‐5 subjected to dysphagia screening ≤24h were less likely to die compared to those screened >24h (1.4% vs. 4.9%, p=0.001; aOR 0.32, 95% CI: 0.12‐0.81). No such association was seen in patients with NIHSS 6‐16 (7.6% vs. 9.8%, p=0.174) or with NIHSS >16 (28.2% vs. 24.1%, p=0.147).

**Conclusion:** In stroke units that have already implemented dysphagia screening into routine practice, screening performed ≤24 hours of admission appears to reduce hospital mortality compared to more delayed screening. This refers particularly to patients with minor ischemic strokes who are typically considered the low‐risk population.

**Disclosure:** Nothing to disclose.

## EPO‐209

### Impact of sex and infarct volume on early functional outcomes after mechanical thrombectomy in acute ischemic stroke

#### H. Savsin ^1^; T. Homa^3^; D. Wrobel^1^; A. Slowik^2^; P. Wrona^2^

##### ^1^Student Scientific Group in Cerebrovascular Diseases, Jagiellonian University Medical College. Krakow Poland; ^2^Department of Neurology. Jagiellonian University Medical College, Krakow Poland; ^3^Department of Neurology, University Hospital, Krakow, Poland

**Background and aims:** The influence of factors like sex on outcomes after mechanical thrombectomy (MT) in acute ischemic stroke (AIS) patients remains uncertain. This study analyses pre‐MT computed tomography perfusion (CTP) imaging values and early neurological improvement (ENI), a 24‐hour post‐MT metric of outcome, to examine sex‐based differences in early recovery.

**Methods:** We retrospectively analyzed 573 consecutive patients with AIS in anterior circulation who underwent successful MT (TICI>=2b) at University Hospital, Krakow (2019–2023). We collected demographic and clinical factors including CTP imaging followed by post‐processing analysis with RAPID software. Early neurological improvement (ENI) was defined as a reduction of >=4 on the National Institutes of Health Stroke Scale (NIHSS) compared with the baseline score or an NIHSS <2 within 24 hours after MT. Early infarct volume (EIV) was calculated using CTP‐derived cerebral blood flow<30% volume.

**Results:** In the multivariate regression analysis, older age (OR=0.982 [0.967‐0.997]) and pre‐stroke diabetes (OR=0.643 [0.422‐0.978) decreased odds for ENI, whereas perfect recanalization (TICI>=2c) increased the odds for achieving ENI (OR=2.139 [1.403‐3.260], p<0.001). Although sex itself was not associated with ENI, an interaction between sex and EIV was observed (p=0.043), with EIV impacting female odds for ENI more substantially (OR=0.841 [0.764‐923 per 10ml, p<0.001) in comparison to males (OR=0.949 [0.887‐1.017, p=0.139).

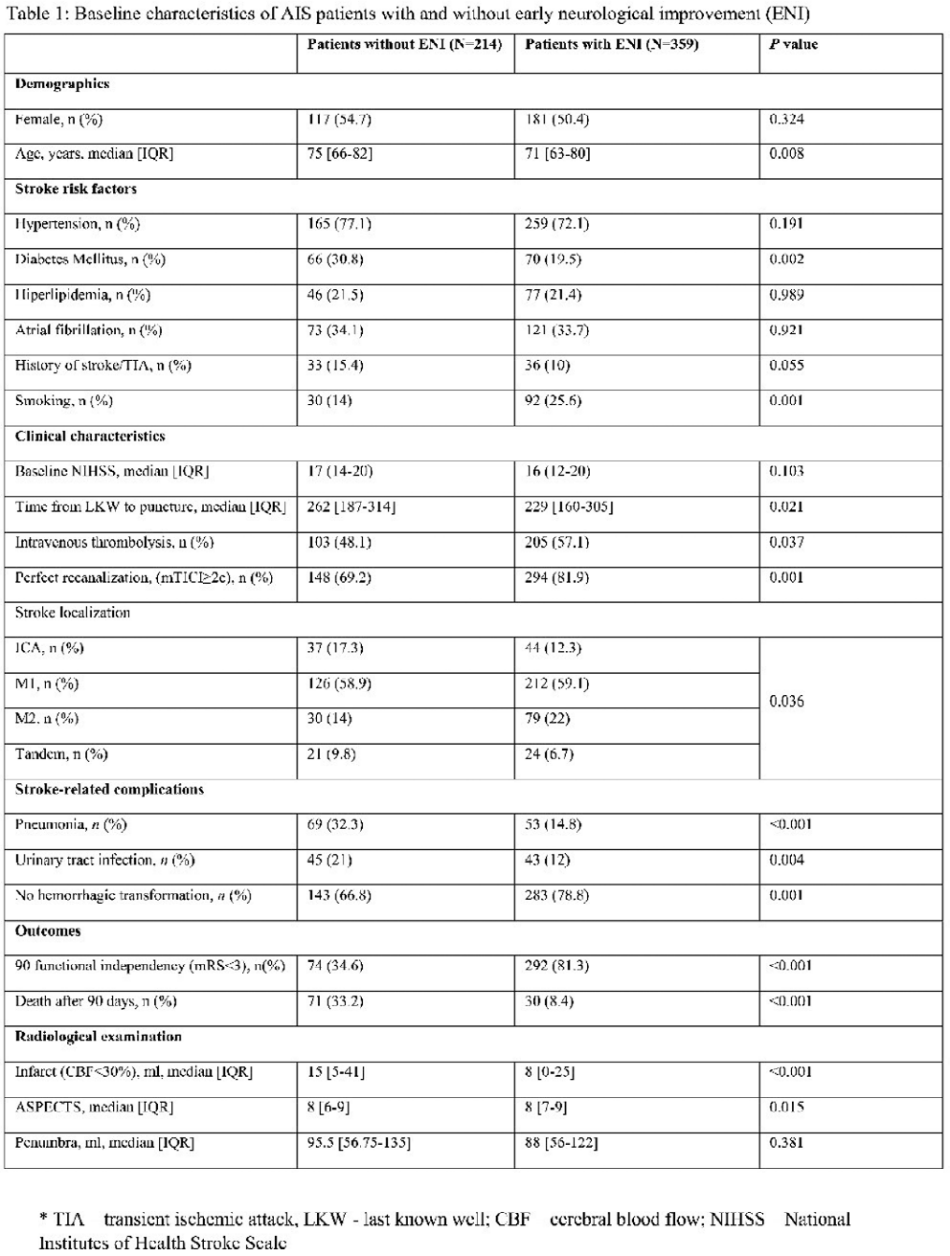

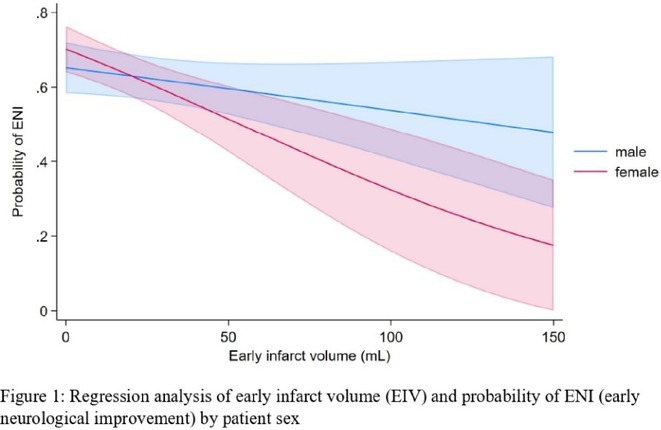

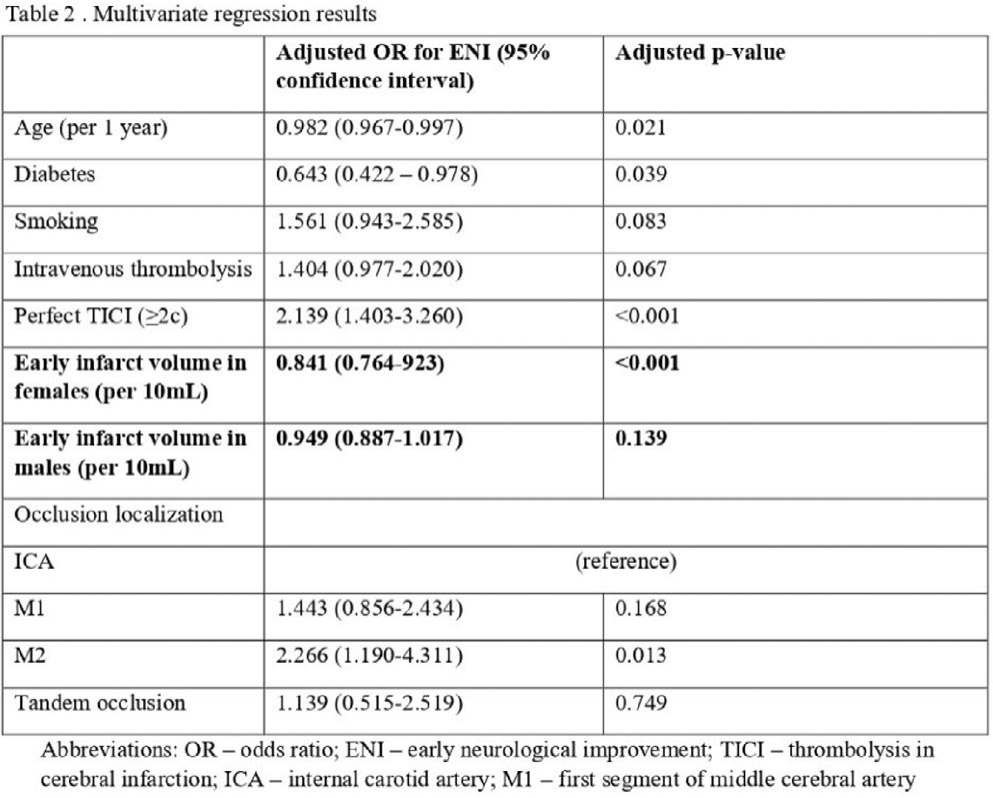

**Conclusion:** This study illustrates that pre‐procedural EIV in the female sex undergoing MT for anterior circulation AIS more significantly impacts early post‐stroke outcomes. This may stem from sex‐specific vulnerability to cerebral ischemia.

**Disclosure:** ERA‐NET‐NEURON/21/2020 BioStroke grant.

## EPO‐210

### Gender inequalities in stroke management in a comprehensive Italian hospital: A retrospective study on 9167 patients

#### I. Scala ^1^; M. Covino^3^; P. Rizzo^1^; M. Bisegna^2^; M. Monforte^1^; P. Calabresi^1^; G. Frisullo^1^

##### ^1^Neuroscience, Fondazione Policlinico Universitario A. Gemelli IRCCS, Rome, Italy; ^2^Neuroscience, Catholic University of the Sacred Heart, Rome, Italy; ^3^Emergency Department, Fondazione Policlinico Universitario A. Gemelli IRCCS, Rome, Italy

**Background and aims:** Several studies have demonstrated that risk factors and symptoms of stroke differ radically between the sexes. Furthermore, gender inequalities in stroke management have been widely described. The primary aim of this study was to analyze differences in stroke management between sexes. Moreover, we analyzed differences in in‐hospital pathways and symptoms at onset of stroke by sex.

**Methods:** In this retrospective, cohort study, we consecutively enrolled adult patients admitted to the Emergency Department (ED) of an Italian comprehensive stroke center for suspected stroke from 2015 to 2022. Univariate comparisons were performed using Mann‐Whitney, Kruskal‐Wallis and χ2‐tests, as appropriate. Binary and ordinal logistic regression models were used for the adjusted analyses.

**Results:** Overall, 9167 patients with suspected stroke were included in the study, of whom 4070 (44.4%) had a confirmed discharge diagnosis of Acute Ischemic Stroke (AIS). Considering the entire study population, we found that, in the adjusted analysis, male sex was an independent predictor of a lower likelihood of waiting ≥15 minutes in the ED (OR 0.694 95% CI (0.493‐0.978);p=0.037) and of being classified as non‐emergency code at triage (common OR 0.757 95% CI (0.580‐0.988);p=0.040). Considering patients with a discharge diagnosis of AIS, we found that women were significantly older than men (p<0.001) and had higher median NIHSS at onset (p<0.001). No gender differences were found in the rate of revascularization treatments, hospitalization, and in‐hospital mortality.

**Conclusion:** Although no differences were found in the rate of revascularization treatments administered, some sex inequalities in stroke management still persist and therefore require attention within in‐hospital pathways.

**Disclosure:** Nothing to disclose.

## EPO‐211

### Positron emission tomography for diagnosing cerebrovascular disorders: a scoping review with practical suggestions

#### B. Storti^1^; F. Sepe^2^; F. Giammello^3^; G. Fiume^4^; F. Rizzo^5^; M. Romoli^6^; M. Mosconi^7^; M. Bagnato^8^; M. Foschi^9^; I. Colonna ^10^; R. Ornello^9^

##### ^1^Cerebrovascular Unit, Fondazione IRCCS Istituto Neurologico Carlo Besta, Milan, Italy; ^2^UOC Malattie Cerebrovascolari/Stroke Unit IRCCS Fondazione Istituto Neurologico C. Mondino, Via Mondino 2, 27100, Pavia, Italy; ^3^Neurology and Stroke Unit, Neuchâtel Hospital Network, 2000 Neuchâtel, Switzerland; ^4^UOC Neurologia, IRCCS Centro Neurolesi Bonino‐Pulejo, Messina; ^5^Stroke Unit, Department of Neurology, Hospital Universitari Vall d’Hebron, Barcelona, Spain; ^6^Neurology and Stroke Unit, Department of Neuroscience, Bufalini Hospital, 47521 Cesena, Italy; ^7^Internal Cardiovascular and Emergency Medicine‐Stroke Unit, S. Maria della Misericordia Hospital, University of Perugia, Perugia, Italy; ^8^Department of Systems Medicine, Stroke Unit, University of Tor Vergata, Rome, Italy; ^9^Department of Biotechnological and Applied Clinical Sciences, University of L'Aquila, 67100 L'Aquila, Italy, ^10^Department of Neurology, “F. Ferrari” Hospital, Casarano, Italy

**Background and aims:** Positron emission tomography (PET) has several potential applications in cerebrovascular pathology; however, it is poorly available as it requires radioactive tracers and has high costs. This scoping review aims to evaluate the current literature on the clinical applications of PET after stroke to lay the foundation for a framework of clinical use.

**Methods:** We conducted a scoping review according to the PRISMA statement. PubMed and Scopus databases were searched (on January 20th, 2024) for relevant articles. Study inclusion criteria were: observational design (cohort, cross‐sectional, or case series studies with more than five patients), using PET with any technique, and reporting on clinical outcomes such as stroke recurrence, death, or clinical improvement. The Newcastle‐Ottawa Scale (NOS) was used to assess the risk of bias.

**Results:** Out of 9866 initially included records, 13 articles were selected. Various PET tracers have been employed across studies, mostly including 18FDG, 15O, 11C PIB, 18F‐florbetapir, and 18F‐florbetaben. The most common cerebrovascular diseases included primary angiitis of the central nervous system (PACNS) detected using fluorodeoxyglucose (FDG)‐PET and cerebral amyloid angiopathy (CAA) detected using amyloid tracer PET.

**Conclusion:** The use of cerebrovascular PET imaging tracers shows promise in assisting with diagnosis, though their current application remains restricted to experimental settings. Factors such as the use of different tracers and the evaluation of a limited number of patients hinder their diagnostic value. The most promising applications of PET imaging are in diagnosing CAA and PACNS. However, standardized protocols are still required.

**Disclosure:** Nothing to disclose.

## EPO‐212

### Thrombophilia screening in young ischemic stroke patients

#### J. Stefela ^1^; J. Vinklarek^1^; J. Brichta^1^; M. Cvikova^1^; D. Goldemund^1^; M. Reif^1^; V. Weiss^1^; M. Harsany^2^

##### ^1^Department of Neurology, St. Anne'ś University Hospital and Faculty of Medicine, Masaryk University, Brno, Czechia; ^2^Department of Neurology and International Clinical Research Centre, St. Anne'ś University Hospital and Faculty of Medicine, Masaryk University, Brno, Czechia

**Background and aims:** Approximately 10‐15% stroke patients are young adults aged between 18‐50 years. Often, standard diagnostic work‐up does not reveal stroke etiology. Thrombophilia testing is a common procedure although its clinical relevance remains unclear. Our aim was to assess routine thrombophilia testing and its clinical implications in young stroke.

**Methods:** A retrospective cohort study of ischemic stroke patients aged 18‐50 years hospitalized at our tertiary stroke centre between January 2020 and December 2024 was performed. We determined baseline patients’ characteristics, medical history, identified etiology and – if performed – results of the thrombophilia testing. Primary outcome was a positive thrombophilia screening. Association with age, gender, history of thromboembolism, patent foramen ovale (PFO) and cryptogenic stroke etiology was analysed.

**Results:** Out of 142 patients, 116 had a complete work‐up and 68 were tested for thrombophilia. Any positive result was identified in 17 patients (25%, figure 1). There was no difference in age, gender, baseline neurologic deficit or cardiovascular risk factors (table 1). In only 4 patients (6%), positive thrombophilia screening results were deemed relevant or led to management change. In a multivariate logistic regression, positive thrombophilia screening was associated with lower odds of having a PFO‐related stroke (OR 0.12 [0.22‐0.66], p=0.02) while there was no association with other prespecified risk factors.

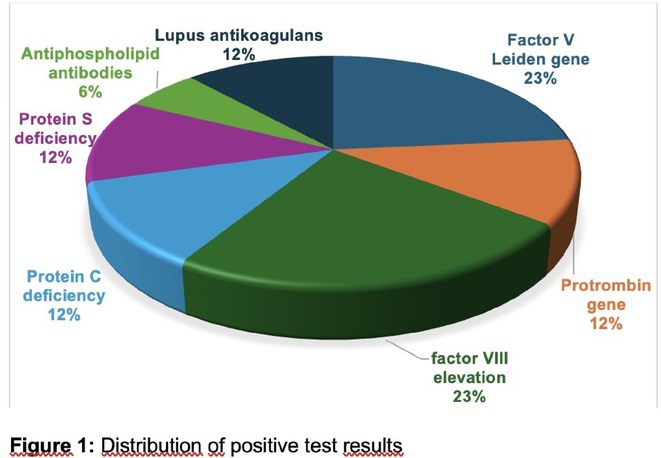

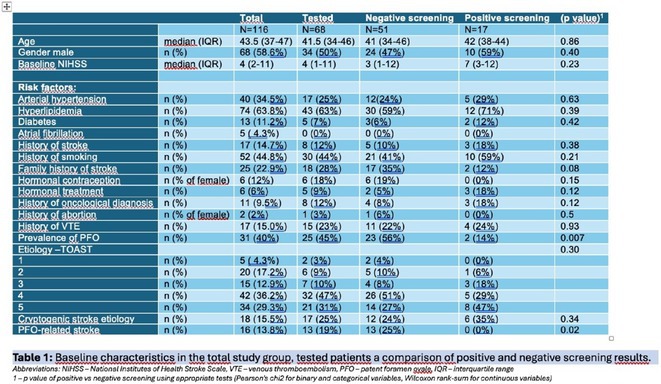

**Conclusion:** Routine thrombophilia screening in young stroke patients has low yield and its results are rarely clinically significant. There was high prevalence of cardiovascular risk factors in our young stroke cohort. Larger studies are necessary to assess testing for thrombophilia in different stroke subtypes.

**Disclosure:** Nothing to disclose.

## EPO‐213

### Pathological breathing patterns in unilateral lateral medullary infarction: a voxelwise lesion–behavior mapping study

#### K. Pavšič ^1^; J. Pretnar Oblak^1^; J. Avsenik^2^; L. Dolen Grošelj^3^; A. Vovk^4^; R. Berlot^5^; T. Rus^5^; F. Bajrović^1^

##### ^1^Department of Vascular Neurology and Neurological Intensive Care, University Medical Centre Ljubljana, Ljubljana, Slovenia; ^2^Institute of Radiology, University Medical Centre Ljubljana, Ljubljana, Slovenia; ^3^Institute of Clinical Neurophysiology, University Medical Centre Ljubljana, Ljubljana, Slovenia; ^4^Institute of Pathophysiology, Medical Faculty, University of Ljubljana, Ljubljana, Slovenia; ^5^Department of Neurology, University Medical Centre Ljubljana, Ljubljana, Slovenia

**Background and aims:** Acute unilateral lateral medullary infarction (ULMI) can damage the autonomic respiratory network and cause breathing disturbances in some patients. We aimed to find an association between lesion location/size and the occurrence of pathologic breathing patterns (PBP).

**Methods:** We prospectively followed 38 patients with ULMI (mean age 58±12 years, 30 (79%) men) hospitalized in our centre from 2015 to 2019. Polysomnography (PSG) and 1.5T MRI scans were performed in the acute phase. The presence of PBP such as tachypnea, moderate/severe ataxic breathing, and periodic breathing was documented from PSG. MRI lesions were mapped on the MNI‐152 template using a computational algorithm and manual correction. All lesions were flipped to the right side and were blurred using a 3mm FWHM spatial filter. Subtraction analysis using voxelwise t‐test was performed between patients with and without PBP (PBP vs non‐PBP, respectively), identifying regions where the lesion burden differed significantly at p=0.05.

**Results:** PBP occurred in 25(66%) patients. The main lesion cluster was larger in PBP group compared to non‐PBP group (2115 vs. 148 voxels). Subtraction analysis showed that PBP lesions more often affected a larger area of the rostral medulla, involving key autonomic, respiratory, and bulbar networks. An area in the inferior cerebellar peduncle (involved in vestibular and somatosensory functions) was statistically more frequently affected in non‐PBP group (Figure 1).

**Figure:**
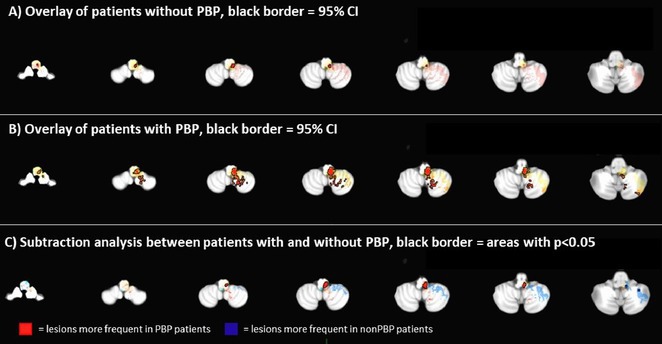
**FIGURE 1** Overlay maps of lesions in patients with (A) and without (B) pathological breathing patterns (PBP). C) Subtraction analysis using a voxewise t‐test between patients with and without PBP.

**Conclusion:** Our findings indicate that the presence of PBP in ULMI patients is associated with larger lesions in the medullary regions responsible for autonomic, respiratory, and bulbar functions.

**Disclosure:** There is nothing to declare.

## EPO‐214

### Stroke as a complication after transcatheter aortic valve implantation

#### R. Pastor^1^; A. Sánchez^1^; M. Filipchuk^1^; G. Cabañas^1^; M. Campos^1^; N. Mena ^1^; J. Cortina^1^; L. Gil^1^; J. Romero^1^; J. Masjuán^1^; L. Salido^2^; Á. Sánchez‐Recalde^2^; I. Corral^1^

##### ^1^Neurology Department, Hospital Universitario Ramón y Cajal, Madrid, Spain; ^2^Cardiology Department, Hospital Universitario Ramón y Cajal, Madrid, Spain

**Background and aims:** Stroke is one of the most concerning complications following transcatheter aortic valve implantation (TAVI). The aim of our study is to describe the characteristics of the event at our center and analyze factors potentially associated with stroke development.

**Methods:** We collected data from TAVIs performed at a tertiary university hospital between 2018‐2024 and conducted a retrospective descriptive study. Additionally, we evaluated the association of stroke with patient characteristics and vascular risk factors using multivariate analysis.

**Results:** A total of 574 patients were included, 17 (3%) of whom experienced a cerebrovascular event. Of these, 10 (59%) were women, with an average age of 84 years. Atherosclerosis in the supra‐aortic trunks was found by CT angiography in 53% of the cases. Binary logistic regression showed atrial fibrillation (AF) as the only risk factor with odds ratios of 11.68 (CI 95%: 2.123‐64.259, p=0.005), observed in 58.8% of stroke patients versus 32% of non‐stroke patients. The type of stroke was exclusively ischemic, 65% occurred within the first 24 hours and 35% between 24 and 48 hours after the procedure. Of the strokes, 76.5% were minor, and 23.5% were major. Only one patient was a candidate for fibrinolysis and one for mechanical thrombectomy. No patient died as a result of the cerebrovascular event.

**Conclusion:** Incidence of stroke was 3% in our series, generally presented as a minor stroke with no direct impact on mortality. Previous AF was the only risk factor.

**Disclosure:** Nothing to disclose.

## EPO‐215

### Discovery and validation of miRNAs in stroke: Profiling and bioinformatic target analysis

#### S. Ahmadova; C. Eyileten; Z. Wicik; A. Gasecka; M. Teresa Di Martino; J. Mucha; S. De Rosa; I. Jastrzebska; M. Postula; A. Czlonkowska

##### Department of Experimental and Clinical Pharmacology, Medical University of Warsaw, Center for Preclinical Research and Technology CEPT, Warsaw, Poland

**Background and aims:** MiRNAs and their target genes are recognized in the pathophysiology of ischemia.

**Methods:** Microarrays were performed with acute ischemic stroke vs control samples. Top non‐coding regulators of ischemia (miR‐18a‐5p, miR‐4467, miR‐199a‐5p and miR‐3135b) was validated.

**Results:** Microarray data identified 146 up and 258 downregulated DE probes. Target prediction showed 67 up and 125 downregulated mRNAs mapped by multiMir R package. Targets of upregulated top miRNAs were most associated with BDNF, IL‐2 signaling, FSH regulation of apoptosis, Axon guidance and TGF‐beta regulation of EC matrix. Downregulated miRNAs were most associated with Axon guidance, Neuronal system and Signaling by NGF. ANKRD52, AGO1 were targeted by all types of DE miRNAs. Most susceptible to regulation by upregulated miRNAs: ANKRD12 and HIFIA and downregulated miRNAs: GNAI2 and GRIN. qRT‐PCR analysis showed that miR‐18a‐5p was higher in stroke patients both at day l and 7 compared to control (p=0.001, p=0.009, respectively). MiR‐199a‐5p was higher in stroke group, and stayed higher at day? (p<0.001, p=0.002 respectively). MiR‐4467 and miR‐3135b were lower in stroke patients at day l compared to control (p<0.001, p<0.001, respectively). ROC curve showed diagnostic value for all studied miRNAs for acute stage of stroke (p<0.01).

**Figure:**
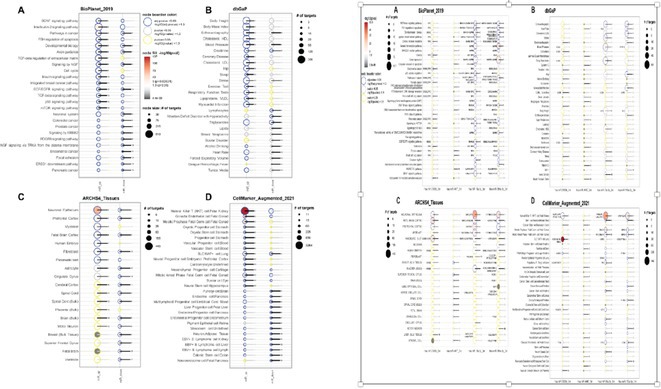
FIGURE 1

**Figure:**
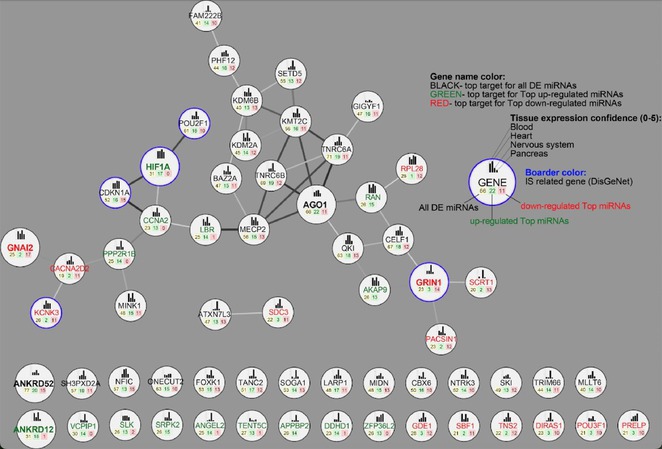
FIGURE 2

**Figure:**
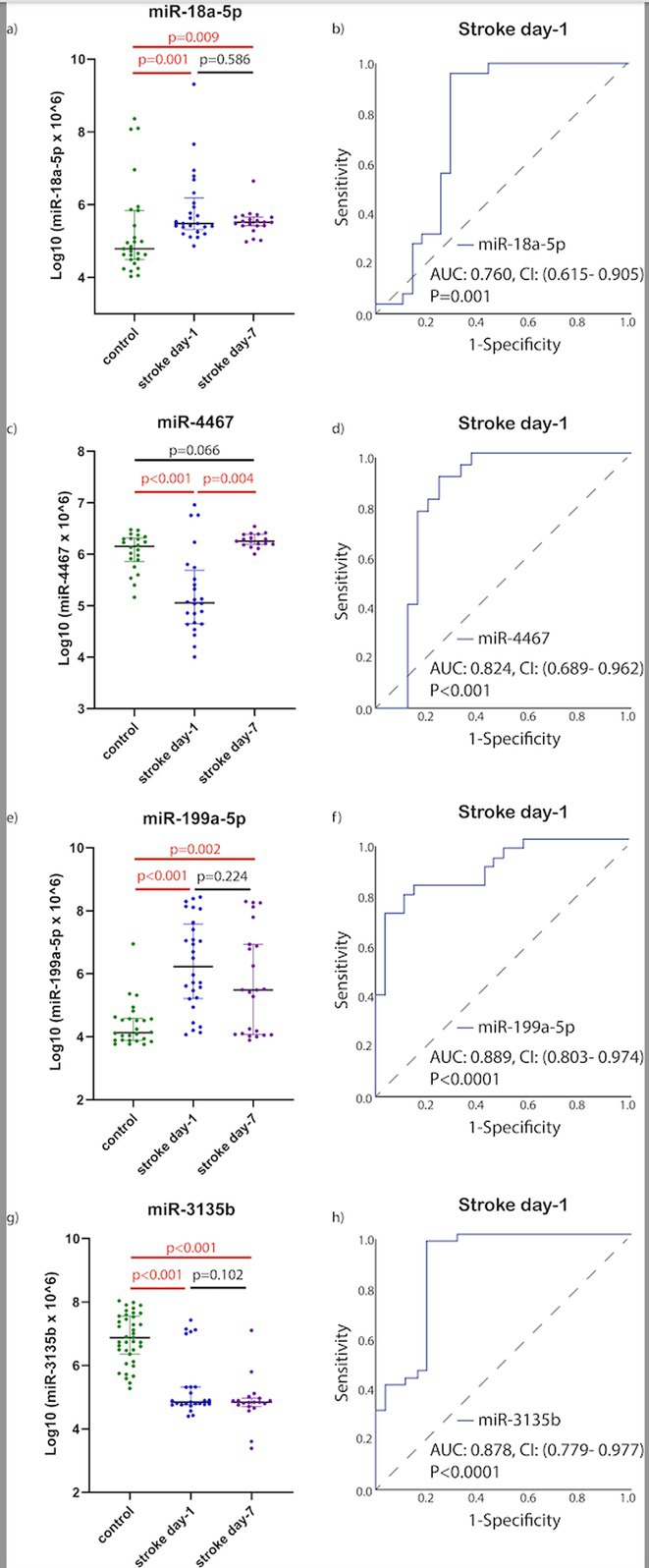
FIGURE 3

**Conclusion:** In our study microarray, validation and bioinformatic analysis results identified miR‐18a‐5p known to play a role in the pathophysiology of ischemia. Another promising biomarker of recovery from ischemia that was identified is miR‐3135b. The measurement techniques for determining miRNA are developing and may in the near future enable the use of these biomarkers in clinical practice. OPUS; 2018/31/B/NZ7/01137.

**Disclosure:** No competing interest.

## EPO‐216

### Surgical material cardioembolic stroke as a complication of ablative thoracoscopy

#### T. Jordà‐Baleri; M. Olivé‐Gadea; M. Ribó‐Jacobi; N. Rodríguez‐Villatoro; D. Campos‐Fernandez

##### Stroke Unit, Neurology Department, Hospital Universitari Vall d'Hebron, Barcelona, Spain

**Background and aims:** Perioperative cardioembolic stroke is severe complication of cardiac surgery. The particularity of this case lies in the thrombectomy's findings, where the extracted emboli consisted of exogenous surgical material. To our knowledge, no similar cases have been previously reported.

**Methods:** Description of a case report.

**Results:** A man in his late 70s, with a history of hypertension and refractory atrial fibrillation underwent thoracoscopic ablation. During the procedure, he experienced a rupture of the left superior pulmonary vein and left atrium, resulting in pericardial effusion, cardiac tamponade, and cardiogenic shock. An emergent pericardiocentesis followed by pericardiotomy to repair the atrial rupture were performed. A CT scan revealed acute infarction in the left hemisphere and left carotid artery thrombosis, and the patient was transferred to a Comprehensive Stroke Center in the area for mechanical thrombectomy. Recanalization (TICI 2b) was achieved with a single thrombectomy device pass. A thrombus which included textile/fibre‐like material was obtained, and embolization of exogenous material during emergent pericardiotomy was suspected as the stroke cause. Despite optimal medical therapy, the patient passed away a few days later.

**Figure:**
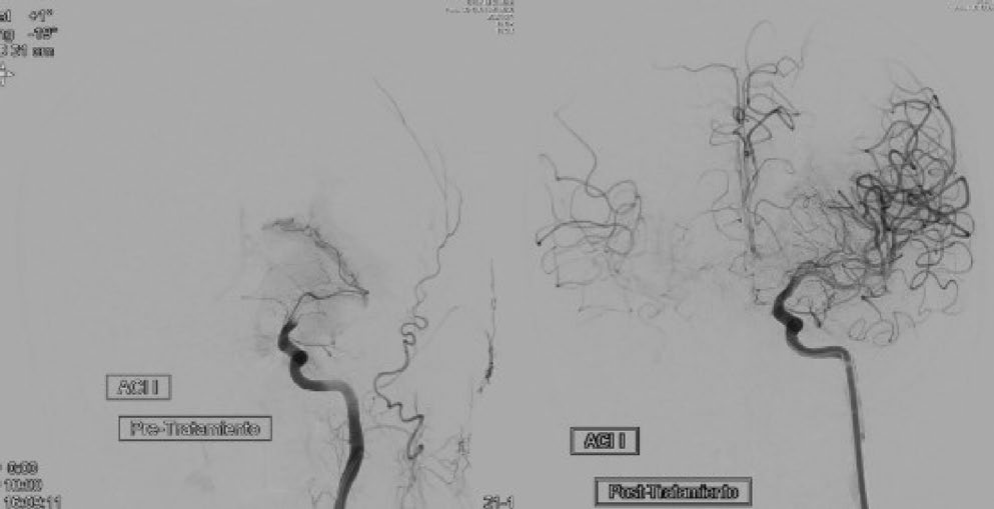
**FIGURE 1** Left internal carotid arteriography pre‐treatment and post‐treatment

**Figure:**
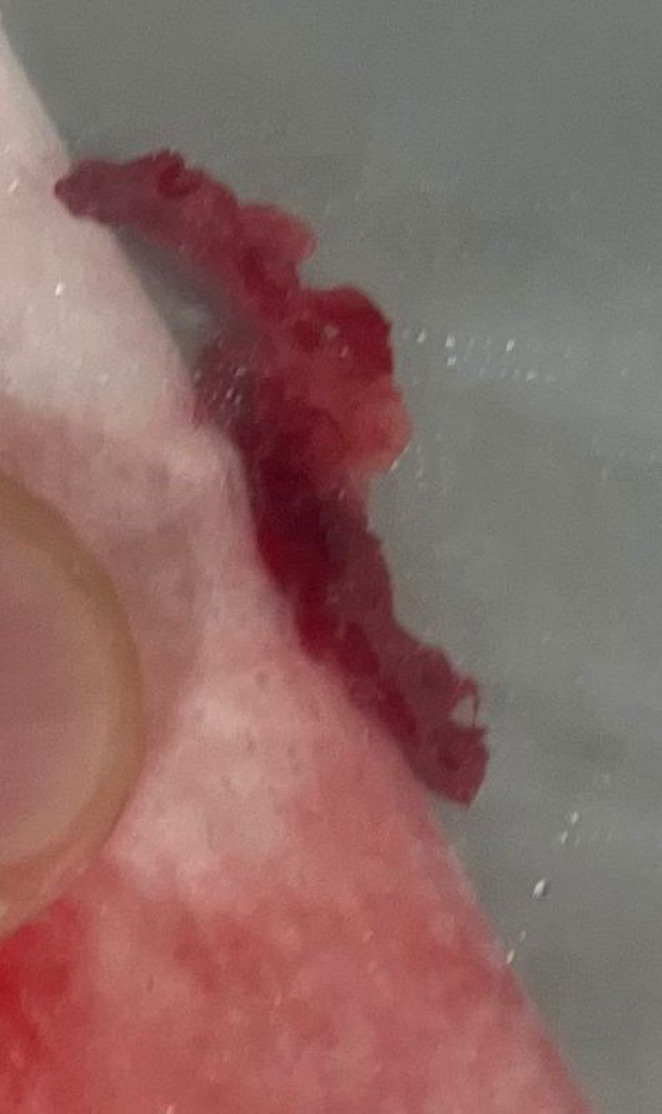
**FIGURE 2** Extracted material consisting of a surgical gauze and hematic content

**Conclusion:** Perioperative stroke still occurs in 2.6% of patients undergoing cardiac surgery despite the application of prevention measures. Despite being uncommon, foreign‐body cardioembolic stroke should be in the differential diagnosis, as it has previously been described.

**Disclosure:** Nothing to disclose.

## EPO‐217

### Locked‐in syndrome due to severe case of varicella zoster virus vasculitis

#### T. Unt; U. Thomson; K. Kannel; S. Ütt; A. Reitsnik; A. Vares; S. Mironenko; H. Jaakmees; A. Leheste; S. Mallene; K. Gross‐Paju

##### West Tallinn Central Hospital, Neurology and Psychiatry Clinic, Stroke Department, Tallinn, Estonia

**Background and aims:** Varicella zoster virus (VZV) vasculitis is a rare but serious complication arising from the reactivation of the varicella zoster virus. This condition primarily affects the elderly and immunocompromised individuals, posing significant challenges in diagnosis and management. The virus can lead to vasculitis by directly invading blood vessel walls or triggering an immune response that targets vascular structures. VZV vasculitis can have diverse clinical presentation, ranging from skin lesions and neurological deficits to systemic manifestations. Skin findings often include vesicular eruptions in a dermatomal distribution, resembling herpes zoster, while neurological involvement may lead to cerebrovascular accidents or encephalitis. The intricate interplay between viral factors and the host's immune response contributes to the complex pathogenesis of VZV vasculitis.

**Methods:** Case report.

**Results:** 69‐year‐old female patient with severe course of VZV‐vasculitis first presented with headache, meningismus, undulating dysartria and general malaise but over next week progressed to locked‐in syndrome due to vast ischemic damages in brainstem and cerebellar region, shown on images 1‐3. Diagnosis was confirmed by radiological findings and presence of VZV IgG antibodies in cerebrospinal fluid. The patient was treated with intravenous acyclovir for 21 days, along with intravenous methylprednisolone, without remarkable positive effect.

**Figure:**
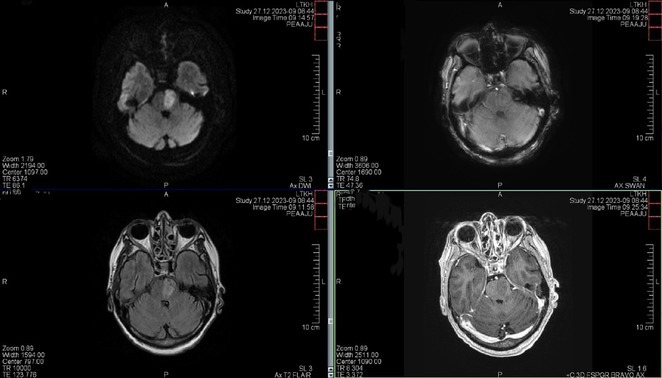
**FIGURE 1** Second MRI scan showing ischemic lesion in the brainstem on the left.

**Figure:**
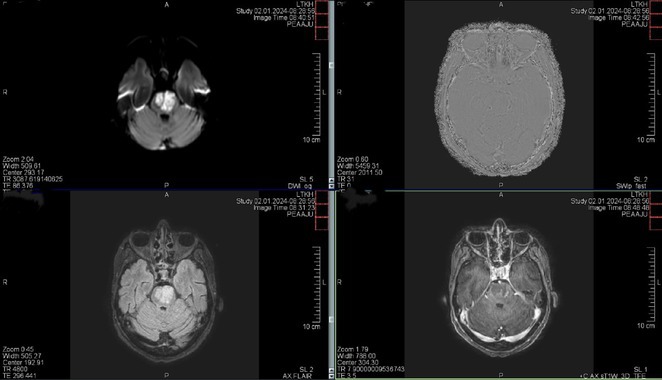
**FIGURE 2** Third MRI scan showing ischemic lesion's progression in the brainstem.

**Figure:**
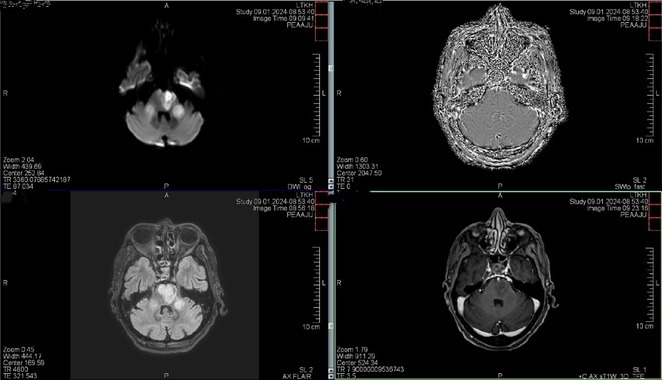
**FIGURE 3** Fifth MRI scan showing ischemic lesions’ dynamic in the brainstem and cerebellar peduncles.

**Conclusion:** Diagnosing VZV vasculitis requires multidisciplinary approach, combining clinical evaluation, imaging and laboratory analyses. Early recognition is crucial to initiate prompt antiviral and immunosuppressive therapy, aiming to mitigate the inflammatory response and prevent further vascular damage. This abstract emphasizes the need for awareness among clinicians, given the potential for severe morbidity and mortality associated with VZV vasculitis.

**Disclosure:** Nothing to disclose.

## Neurological Manifestation of Systemic Diseases and Neurotoxicology

## EPO‐218

### Subacute cerebellar syndrome, gait disorder and nistagmus due to hypomagnesemia

#### A. Oliveros‐Cid ^1^; L. Rondon Moreno^3^; A. Izaguerri Gracia^3^; J. Agorreta Ruiz^2^; A. Oliveros Juste^4^; M. Cid Lopez^4^

##### ^1^Neurology Unit. Fundación Neuropolis. Zaragoza. Spain. Neurology Section, Internal Medicine Department. Hospital Reina Sofía. Zaragoza. Spain; ^2^Internal Medicine Department. Hospital Reina Sofía. Tudela. Navarra. Spain; ^3^Neuroscience Research Unit. Fundación Neuropolis. Zaragoza. Spain; ^4^Neurology Unit. Fundación Neuropolis. Zaragoza. Spain

**Background and aims:** Hypomagnesemia is known to cause neurological symptoms such as tremor, tetany, and seizures, though it rarely presents as an acute cerebellar syndrome accompanied by oculomotor disturbances, with vertical nystagmus being the most frequently reported. We describe a case of pendular nystagmus and cerebellar ataxia in the context of severe hypomagnesemia.

**Methods:** A 62‐year‐old woman on long‐term proton pump inhibitor therapy presented with abdominal pain, nausea, dizziness, and diplopia following a gastroenteritis episode and intensification of treatment. Three days later, she developed gait ataxia, lateropulsion, instability, and primary gaze nystagmus with changes in orientation and direction during ocular pursuit. Over subsequent days, appendicular cerebellar syndrome and limb myoclonus emerged.

**Results:** Initial brain MRI and MR angiography revealed no abnormalities. Laboratory tests highlighted significant hypocalcemia (7.8 mg/dL) and hypomagnesemia (0.5 mg/dL). Magnesium replacement therapy was promptly initiated. Nystagmus resolved within hours, and ataxia improved over the following days. 5 months later, mild nystagmus during ocular pursuit and slight diplopia persisted, alongside a mildly unsteady but stable gait.

**Conclusion:** Severe hypomagnesemia can manifest as acute cerebellar ataxia, additional cerebellar symptoms and oculomotor abnormalities, particularly downbeat nystagmus. Pendular nystagmus is typically associated with demyelinating diseases, acquired brainstem lesions, cerebellar syndrome, genetic disorders, and metabolic conditions. This case underscores the importance of considering electrolyte imbalances in the differential diagnosis of acute cerebellar syndromes and highlights the potential for rapid neurological recovery with timely intervention. Further research is needed to elucidate the mechanisms underlying pendular nystagmus in hypomagnesemia.

**Disclosure:** Nothing to disclose.

## EPO‐219

### Neurological conditions after bariatric surgery, addressing more than complications

#### F. Assis Jacinto ^1^; G. Faria^2^; P. Salgado^1^

##### ^1^Neurology Department, Hospital Pedro Hispano, Matosinhos, Portugal; ^2^Obesity Surgical Treatment Unit, General Surgery Department, Hospital Pedro Hispano, Matosinhos, Portugal

**Background and aims:** Bariatric surgery (BS) as an obesity treatment has increased. Although reports suggest improvements in headaches and cognitive function post‐op, neurological conditions may arise, often linked to nutritional deficits. This study aims to describe neurological conditions associated with BS.

**Methods:** Six‐year retrospective study, descriptive analysis.

**Results:** We analysed 385 patients (83.9% female, mean age: 47.6 years (SD=11.2)). Comorbidities were mostly vascular risk factors. Procedures included gastric bypass (GB) (50.9%), sleeve (45.2%) and SADI‐S (3.9%). Vitamin supplementation began 3.1 days (SD=4.2) post‐op. Neurological complications occurred in 3.1% (all female, 11 (91.7%) post‐GB) at median onset‐time of 29.5 months (IQR=50‐20.25) post‐BS: 3 compressive mononeuropathies, 2 Wernicke's encephalopathies, 6 cognitive complaints (CC) and 1 Guillain‐Barré syndrome (GBS). CC cases (median onset: 24 months (IQR=52‐24) post‐op) were referred to Neurology consultation, with 2 improving after initial observation. Brain‐image showed subcortical atrophy in 1 case. CC were linked to B12‐vitamin deficiency and psychopathology in 2 cases each. Previous headaches were reported in 23 patients (13 with neurological follow‐up). In 19 migrainers, 8 reduced headache frequency post‐op, 3 discontinuing prophylaxis. Three patients (2 with suspected obstructive sleep apnea and 1 with idiopathic intracranial hypertension) experienced complete resolution.

**Conclusion:** Our sample had a low rate of neurological complains, consistent with literature. Nearly 50% of migrainers improved post‐BS, suggesting potential benefit. Although unclear, other factors than vitamin deficits, like hormonal changes, may link weight loss to cognitive impairment. Besides typical complications, we highlight the case of GBS, which could imply potential influence of BS on immune mediated responses.

**Disclosure:** Nothing to disclose.

## EPO‐220

### Evaluation of the role of omega‐3 fatty acids in nicotine‐induced neurotoxicity in pregnant Wistar rats and their pups

#### j. omole ^1^; Q. Alabi^2^; O. Ayoka^1^

##### ^1^Obafemi Awolowo University, Ile‐Ife, Osun State; ^2^Adeleke University, Ede, Osun State

**Background and aims:** This study investigated maternal and their pup neurobehaviour following nicotine exposure during gestational and postnatal periods and neuroprotective effect of Omega‐3 fatty acids.

**Methods:** The study used thirty pregnant female Wistar rats for this study. Groups I and II were treated with 1ml/kg/day of normal saline for 42 days; III was treated with 4 mg/kg/day of nicotine for 42 days; IV‐VI were co‐administered nicotine 4 mg/kg and 100, 300, 600 mg/kg/day of Omega‐3 fatty acids respectively for 42 days.

**Results:** The beam walk time of the mother rats in groups III and IV were significantly higher when compared with other groups. Similarly, the beam walks time of the pups of groups III and IV were significantly higher when compared with the pups of the mother rats in other groups. The brain dopamine and serotonin levels of mother rats in groups III, IV and V were significantly higher when compared with other groups. Also, the brain dopamine and serotonin levels of the pups in groups III and IV were significantly higher when compared with the pups in other groups. The reduced glutathione and catalase of mother and pup rats in groups III and IV were significantly lower when compared with other groups. Photomicrographs of cerebellum and hippocampus of the rats treated with nicotine showed scattered arrangement of pyramidal cells with vacuolated neurons. These alterations were significantly reversed with Omega‐3 fatty acids following nicotine exposure.

**Conclusion:** Omega‐3 fatty acids at 300 and 600mg/kg ameliorated nicotine‐induced neurotoxicity in mother rats and their pups.

**Disclosure:** Nothing to disclose.

## EPO‐221

### Two sisters with facial paresis and low back pain – genetic Neurosarcoidosis?

#### J. Neiva Correia; M. Saianda Duarte; V. Fonseca; J. Morgado; A. Arraiolos

##### Serviço de Neurologia, Hospital Beatriz Ângelo, Loures, Portugal

**Background and aims:** Sarcoidosis is a systemic immune‐mediated disease characterized by granulomatous inflammation affecting multiple organs. Neurosarcoidosis (NS) affects 5‐10% of patients with systemic sarcoidosis. The existence of familial forms suggests that genetic predisposition plays an important role in sarcoidosis pathogenesis.

**Methods:** Case report and literature review.

**Results:** Two sisters with no relevant personal medical history presented with similar symptoms, two years apart. TRS at 49 years developed severe low back pain (LBP), thoracic band‐like hypoesthesia and fever lasting four weeks, followed by left facial paresis. ERS at 54 years presented right facial paresis, followed one month later by left facial paresis, distal paresthesia, abdominal band‐like hyposthesia, gait imbalance, LBP, and urinary hesitation. Both cases presented CSF with pleocytosis and hyperproteinorrachia with no evidence of infection, elevated serum ACE levels, chest CT scans with lymphadenopathies, and lymph node biopsy with non‐necrotizing granulomas. The neuro axis MRI showed gadolinium enhancement of the left facial nerve in TRS, and along the roots of the cauda equina in ERS. Both patients improved with corticotherapy, supporting the diagnosis of NS.

**Conclusion:** The relevance of these cases lies in the identification of neurosarcoidosis in two sisters with a similar clinical presentation. Having a relative with sarcoidosis is a known risk factor, and both genetic and environmental factors appear to contribute to its pathophysiology. Furthermore, one of the patients presented with polyradiculopathy, an uncommon manifestation of neurosarcoidosis. Additional studies are needed to improve diagnosis and treatment, as well as to better understand the pathophysiology of this condition.

**Disclosure:** Nothing to disclose.

## EPO‐222

### Dementia in the patients with inflammatory arthritis: An analysis of data from national health insurance service

#### J. Lee

##### Neurology, National Health Insurance Service Ilsan Hospital, Koyang‐si, Republic of Korea

**Background and aims:** We evaluated the risk of dementia between the patients of inflammatory arthritis and matched population, using data from National Health Insurance Service.

**Methods:** We defined the patients of ankylosing spondylitis, seropositive rheumatoid arthritis, and psoriatic arthritis and enteropathic spondyloarthropathy, using the combination of main diagnosis. Control group was defined by 1:5 propensity score matching in each disease.

**Results:** In a multivariate analysis including dementia risk factors (hypertension, diabetes, dyslipidemia, and depression), a diagnosis of rheumatoid arthritis with a positive serologic test was associated with a greater incidence of dementia with HR 1.10, 95% CI 1.02‐1.20 (p=0.0159), the incidence of dementia was lower in the group that used biological agents than in the group that did not (HR 0.46, 95% CI 00.33‐0.65, p<0.0001). Among biological agents, the group using non‐TNF blocker had a significantly lower incidence of dementia (HR 0.37, 95% CI 0.21‐0.65, p= 0.0005).

**Conclusion:** In the case of rheumatoid arthritis with a positive serological test, this study also showed a high risk of developing dementia, similar to the results of other previous studies. Regarding changes according to the administration of biological agents, previous studies have shown that anti‐TNF antagonists lower the risk of developing dementia, but this study also showed that the administration of biological agents lowers the risk of developing dementia, and anti‐TNF antagonists Rather, in the case of non‐anti‐TNF antagonists, a statistically significant decrease was shown.

**Disclosure:** Nothing to disclose.

## EPO‐223

### Protecting the brain with cognitive reserve while healing hearts with coronary artery cardiac surgery

#### D. Megari

##### City College, University of York, Europe Campus, Thessaloniki, Greece

**Background and aims:** Postoperative cognitive decline (POCD), can range in severity from mild cognitive impairment to dementia and delirium, is one of the most serious side effects after coronary artery bypass grafting (CABG). Significant POCD, which includes a decline in cognitive functions and social functioning, is common in CABG patients. Cognitive reserve (CR), a protective factor that acts as a buffer against the effects of neuropathology, aging, and/or trauma, may be able to mitigate these negative effects. It describes the distinct ways that people approach tasks, which may allow them to be more resilient than others.

**Methods:** Using extracorporeal circulation, we evaluated 101 patients both prior to and four months following cardiopulmonary bypass surgery. The evaluation comprised measures of depression, anxiety, CR, and cognitive functions. CR was estimated using functional score, occupation, age, educational attainment, and vocabulary measurements.

**Results:** Focusing on median split, each patient was assigned to either the high (n=50) or low CR (n=51) group. On post‐surgery neuropsychological evaluation, patients with low CR were significantly more likely than those with high CR to exhibit post‐surgical cognitive decline in attention, memory, visuospatial perception, and executive functions, according to the effect of chi‐square tests.

**Conclusion:** Cognitive rehabilitation is essential due to severity of POCD and its impact on functioning, which are aspects of quality of life. CR may predict the neuropsychological effects of heart surgery, identify patients with low CR, and assist them in engaging in intervention programs that may slow cognitive aging, lower the risk of dementia, and improve their overall recovery following surgery.

**Disclosure:** Nothing to disclose.

## EPO‐224

### OverTTuRe study: Disease burden at diagnosis in ATTR amyloidosis patients with neurological impairment in Spain

#### L. Galán‐Dávila ^1^; P. García‐Pavía^2^; A. Eisman‐Maraver^3^; A. García‐López^3^; J. Sánchez‐Covisa^3^

##### ^1^Neurology Service, Hospital Clínico San Carlos, Madrid, Spain; ^2^Department of Cardiology, H. Puerta de Hierro Majadahonda and Centro Nacional de Investigaciones Cardiovasculares (CNIC), Madrid, Spain; ^3^Biopharmaceuticals Medical, AstraZeneca, Madrid, Spain

**Background and aims:** Amyloid Transthyretin (ATTR) amyloidosis is a debilitating and often misdiagnosed condition, characterized by the accumulation of transthyretin amyloid fibrils in various organs and tissues. The OverTTuRe study seeks to provide insights into the pre‐ and post‐diagnosis journeys of ATTR patients (all phenotypes) ‐ this communication focuses on the baseline data (up to diagnosis) of patients in Spain with neurological impairment (peripheral neuropathy ‐PN‐ and mixed).

**Methods:** Observational, retrospective chart review in 11 hospitals across Spain. 107 ATTR‐PN patients and 150 ATTR mixed patients with diagnosis from 2009 onwards were included (Figure 1).

**Figure:**
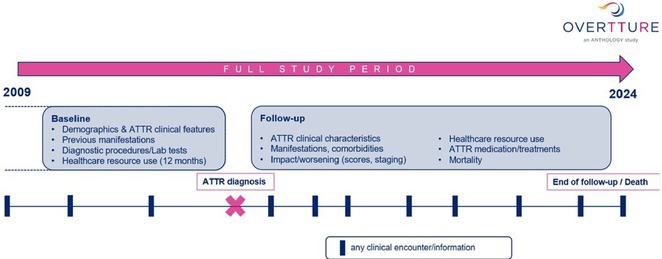
**FIGURE 1** Study flowchart.

**Results:** At diagnosis, mean age (SD) was 66.1 (16.1) years, and 35.8% were female. 75.9% had ATTRv (hereditary), with Val50Met‐late onset being the most predominant variant. 81.7% had a late onset (defined as age at diagnosis ≥ 50). From the first clinical manifestation associated with ATTR until diagnosis, the median time was 9.6 months (mean 44.9mos.), with notable differences between ATTR‐PN (5.2mos.) and mixed (20.2mos.). The NIS mean score was 13.7, with most patients at FAP stage 1 (82.5%) and a PND score of 1 or 2 (88.2%). Healthcare resource utilization (HCRU) prior to diagnosis were generally greater in mixed patients. Baseline data are summarized in Table 1. A wide range of clinical manifestations were already present at diagnosis (Table 2).

**Figure:**
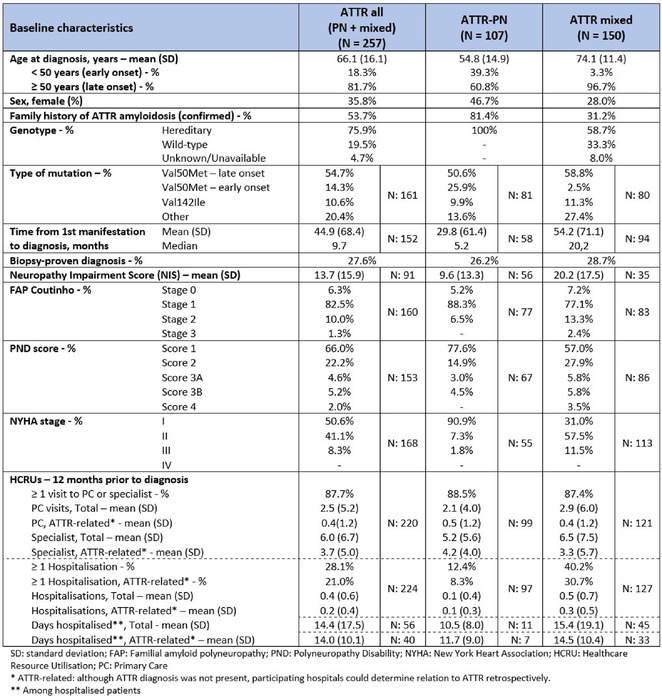
**TABLE 1** Baseline characteristics.

**Figure:**
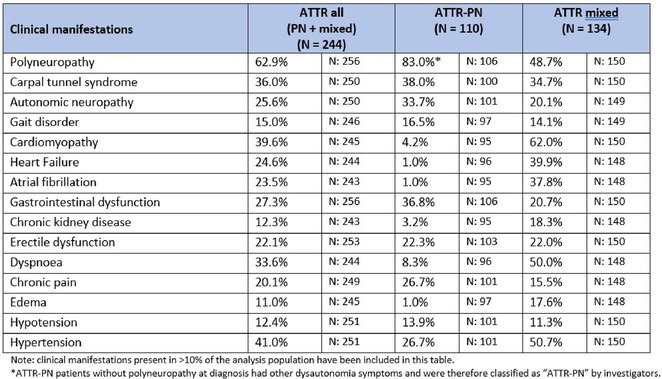
**TABLE 2** Clinical manifestations at baseline.

**Conclusion:** Our data underscore the substantial disease burden of the patients with ATTR‐PN and ATTR‐mixed amyloidosis already at diagnosis, partly due to a long and difficult diagnostic journey. These insights may help to establish improved recommendations for diagnostic procedures.

**Disclosure:** The OverTTure study is sponsored and funded by AstraZeneca.

## EPO‐225

### Lack of awareness of transthyretin amyloidosis with polyneuropathy and its impact on Spanish patients: ATENAS Study

#### L. Galán‐Dávila ^1^; M. Santos‐Rubio^2^; E. Bermúdez‐Ferreira^3^; A. Alejaldre‐Monforte^4^; S. Kapetanovic‐García^5^; C. Borrachero‐Garro^6^; F. Caimi‐Martínez^7^; A. García‐López^7^; R. Rubio‐Renau^8^; C. Moreno‐Tapia^8^; C. Solà‐Marsiñach^8^

##### ^1^Neurology Service, Hospital Clínico San Carlos, Madrid, Spain; ^2^Pharmacy Service, Hospital Universitario Juan Ramón Jiménez, Huelva, Spain; ^3^Member of Amilo (Spanish Association of Amyloidosis), Palma de Mallorca, Spain; ^4^Neurology Service, Hospital Clínic de Barcelona, Barcelona, Spain; ^5^Neurology Service, Hospital Universitario de Basurto, Bilbao, Spain; ^6^Internal Medicine Service, Hospital Universitario Juan Ramón Jiménez, Huelva, Spain; ^7^Biopharmaceuticals Medical, AstraZeneca, Madrid, Spain; ^8^Evidence Generation Department, A Piece of Pie, Barcelona, Spain

**Background and aims:** Transthyretin amyloidosis with polyneuropathy (ATTRv‐PN) is a rare, hereditary, progressive and systemic disease that can highly impact patients’ quality of life (QoL). However, studies addressing the patient's experience with ATTRv‐PN are scarce.

**Methods:** The ATENAS Study is an observational and cross‐sectional ongoing study describing the experiences of patients with ATTRv‐PN, their caregivers and specialized clinicians. For this communication, 15 patients partook in semi‐structured interviews. Interviews were recorded, and anonymized data were coded and analyzed thematically.

**Figure:**
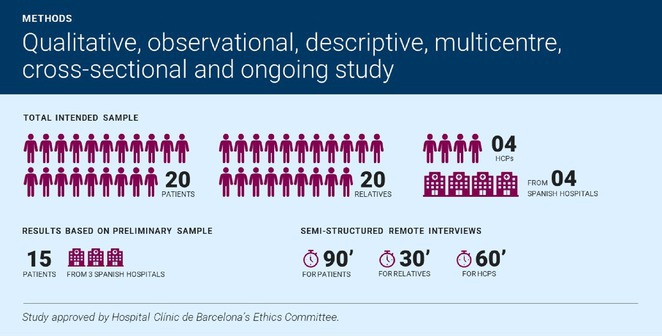
**FIGURE 1** ATENAS study design

**Results:** Diagnostic delays from first symptoms’ appearance until diagnosis –mean of 27 months for index patients and 9.5 months for patients with family history– were identified, reflecting lack of symptom and disease awareness among patients and healthcare professionals. The effect of ATTRv‐PN on QoL divided patients into two groups. Group 1 presented many symptoms, with diarrhea and loss of mobility the most incapacitating and affecting QoL, and blindness the most feared in the future. Lack of early detection of symptoms worsened impact on QoL. Group 2 had fewer symptoms, with loss of feeling on the extremities and fatigue the most common. ATTRv‐PN had been detected early, and patients reported good QoL. Polyneuropathy Disability (PND) score alone could not capture the severity of ATTRv‐PN from patients’ perspective since it was conditioned by the gravity of dysautonomia and patients’ expectations according to their age.

**Figure:**
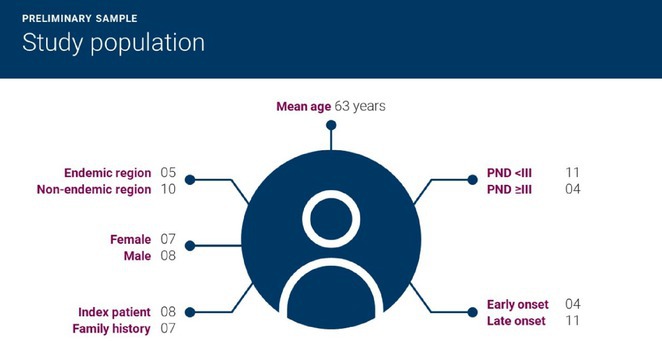
**FIGURE 2** Description of the study population

**Conclusion:** Early detection and treatment of ATTRv‐PN is crucial to delay the appearance of incapacitating symptoms and allow patients to live with good QoL. Increasing awareness of ATTRv‐PN can be a step towards this goal.

**Disclosure:** This study has been sponsored and fully funded by AstraZeneca Farmacéutica Spain.

## EPO‐226

### Emotional and social impact of transthyretin amyloidosis with polyneuropathy on patients and caregivers: ATENAS Study

#### L. Galán‐Dávila ^1^; M. Santos‐Rubio^2^; E. Bermúdez‐Ferreira^3^; A. Alejaldre‐Monforte^4^; S. Kapetanovic‐García^5^; C. Borrachero‐Garro^6^; F. Caimi‐Martínez^7^; A. García‐López^7^; R. Rubio‐Renau^8^; C. Moreno‐Tapia^8^; C. Solà‐Marsiñach^8^

##### ^1^Neurology Service, Hospital Clínico San Carlos, Madrid, Spain; ^2^Pharmacy Service, Hospital Universitario Juan Ramón Jiménez, Huelva, Spain; ^3^Member of Amilo (Spanish Association of Amyloidosis), Palma de Mallorca, Spain; ^4^Neurology Service, Hospital Clínic de Barcelona, Barcelona, Spain; ^5^Neurology Service, Hospital Universitario de Basurto, Bilbao, Spain; ^6^Internal Medicine Service, Hospital Universitario Juan Ramón Jiménez, Huelva, Spain; ^7^Biopharmaceuticals Medical, AstraZeneca, Madrid, Spain; ^8^Evidence Generation Department, A Piece of Pie, Barcelona, Spain

**Background and aims:** Transthyretin amyloidosis with polyneuropathy (ATTRv‐PN) is a rare, hereditary, and progressive disease caused by mutations in the transthyretin gene. Despite ATTRv‐PN's impact on quality of life (QoL), very few studies have examined the lived experience of patients and families.

**Methods:** The ATENAS Study is an observational and cross‐sectional ongoing study describing the experiences of patients with ATTRv‐PN, their relatives and specialized clinicians. For this communication, 15 patients and 8 relatives partook in semi‐structured recorded interviews. Data were anonymized, coded, and analyzed thematically.

**Figure:**
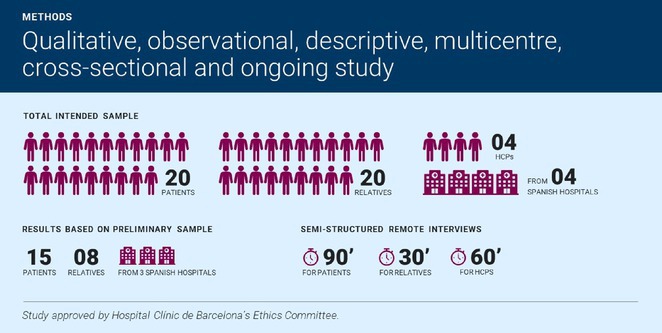
**FIGURE 1** ATENAS study design

**Results:** Patients with severe symptoms faced distress, frustration and shame. Their social and work lives were significantly impacted. Their relatives endured considerable emotional challenges due to drastic lifestyle changes and high patient dependency, with loss of work opportunities and personal income, and high impact on their social lives. Patients with mild symptoms experienced fewer emotional, social and work impacts. Their relatives did not consider themselves caregivers and remained optimistic about the course of ATTRv‐PN. Some patients and relatives thought ATTRv‐PN would manifest similarly in their offspring. Some patients and relatives with severe symptoms hoped the disease would manifest differently (or not at all) in their offspring. All hoped future medical advances would allow their offspring to live with good QoL.

**Figure:**
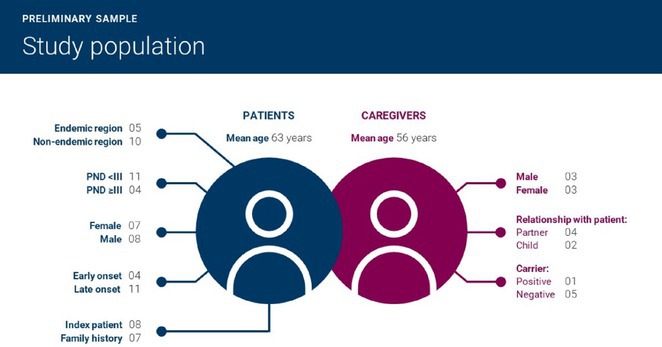
**FIGURE 2** Description of the study population

**Conclusion:** Severe disabling symptoms impacted the emotional, social and work lives of patients and relatives, requiring psychological and emotional support. Multidisciplinary teams treating these patients should be aware of it to improve their QoL.

**Disclosure:** This study has been sponsored and fully funded by AstraZeneca Farmacéutica Spain.

## EPO‐227

### The Hidden Dangers of Nitrous Oxide: Minutes of laughter and source of severe neurological sequelae

#### M. Elguezabal García; R. López Blanco; M. Gilot Sancho; M. Herrezuelo Lafuente; C. Ballester Martínez; I. Hernando Jiménez; V. Hernando Requejo; M. Sastre Real; N. Huertas González; N. Juárez Torrejón; C. Trevino Peinado

##### Neurology, Severo Ochoa University Hospital, Leganes, Spain

**Background and aims:** The rising use of nitrous oxide among young people, many of whom are unaware of its potential side effects, has become a growing epidemiological issue. This substance can cause significant neurological sequelae such as gait and sensory disturbances by cianocobalamine interference or depletion.

**Methods:** We describe the case of a 24‐year‐old male with a history of recreational drug abuse. He presented to the emergency department with a one‐month history of fingertip paresthesia and mild gait disturbances, progressing over two weeks to severe instability and ataxia. The patient reported daily use of 6 nitrous oxide canisters (666 g each) for the past 18 months. Neurological examination revealed distal paresis in the upper limbs, distal and proximal weakness in the lower limbs, hyperactive patellar reflexes and hypoactive Achilles reflexes, absence of vibratory sensation in extremities and bilateral dysmetria in the upper limbs, worsened by visual deprivation. Gait examination showed widened base and lower limb weakness.

**Results:** Diagnostic workup revealed a normal cranial CT and in the bloodwork a vitamin B12 deficiency stands out. Cervical spine MRI showed hyperintensity in anterolateral and dorsal columns, typical of subacute combined degeneration.

**Conclusion:** This case highlights the importance of considering substance abuse, such as nitrous oxide, in the differential diagnosis of young patients with neurological symptoms like subacute sensory ataxia, paraparesis and polyneuropathy. Nitrous oxide is becoming increasingly popular, but its use can lead to permanent sequelae. Early diagnosis and rapid treatment are crucial to prevent them.

**Disclosure:** Nothing to disclose.

## EPO‐228

### Neurology practice among solid organ transplant candidates and recipients: The European academy of neurology survey

#### V. Lo Re ^1^; G. Fiume^2^; M. Rizzo^3^; M. Lolich^4^; F. Avorio^1^; E. Lo Gerfo^1^; M. Pinzani^3^; A. Toscano^5^

##### ^1^Neurology Service, Department of Diagnostic and Therapeutic Services, IRCCS‐ISMETT (Istituto Mediterraneo per i Trapianti e Terapie ad alta specializzazione), University of Pittsburgh Medical Center Italy (UPMCI), Palermo, Italy; ^2^Neurology Unit, IRCCS Centro Neurolesi Bonino Pulejo, Messina, Italy; ^3^Department of Research, IRCCS‐ISMETT, UPMCI, Palermo, Italy; ^4^European Academy of Neurology, Vienna, Austria; ^5^Unit of Neurology and Neuromuscular Disorders, Department of Clinical and Experimental Medicine, University of Messina, Italy

**Background and aims:** Since neurological disorders can occur both before and after a solid organ transplant (SOT), the European Academy of Neurology (EAN) survey intended to provide a preliminary analysis of the extent and circumstances where neurologists are consulted in a transplantation setting.

**Methods:** A web‐based survey was prepared and sent to all EAN members on behalf of the EAN Panel on Neurocritical care.

**Results:** A total of 176 neurologists completed the survey. Only 1 out of 5 neurologists see SOT candidates, mainly for neurological comorbidities, although they are not always involved in multidisciplinary meetings to establish transplant eligibility; neurologists are more often involved when a neurological complication occurs after a SOT (31.8% of respondents), mainly delirium (26.7%); less than 1 out of 10 received specific training on neurological issues in the transplantation setting during their residency; lastly, only a small number of neurologists are involved in research programs in the emerging field of brain‐body interactions.

**Conclusion:** The survey clearly showed neurologists are seldom involved in SOT candidate evaluations even though a cognitive screening is recommended. Also, while post‐SOT neurological complications for which neurologists are consulted can be life‐threatening, only a minority of these professionals received specific training. The survey provided the basis for general guidelines to be agreed between the different associations/consortia of neurologists operating in this field.

**Disclosure:** ‐ Authors have no conflicts of interest to declare relating to this study.

## EPO‐229

### When the Brainstem isn't to blame: A diagnostic challenge

#### M. Andrade Ferreira ^1^; C. Cerqueira^2^; A. Ferreira^1^; F. Correia^1^

##### ^1^Neurology Department, Local Health Unit of Matosinhos, Matosinhos, Portugal; ^2^Neurorradiology Department, Local Health Unit of Matosinhos, Matosinhos, Portugal

**Background and aims:** A clinical presentation suggestive of brainstem involvement often leads to the assumption of central nervous system pathology. However, brain imaging might not reveal intracranial abnormalities, leading to a broader diagnostic process. We present a case report that highlights the importance of considering alternative causes for neurological symptoms suggestive of brainstem involvement.

**Methods:** We describe the clinical case of a patient with neurological symptoms indicative of brainstem involvement. He underwent multiple diagnostic tests, including brain MRI, chest CT, spine MRI, and biopsy for diagnosis confirmation.

**Results:** A 69‐year‐old man, with smoking habits, presented with sudden onset discoordination and weakness of the right lower limb. Neurological examination revealed Horner's syndrome of the left eye, dysphonia, ataxia and paresis of the right lower limb and ataxic gait. Brain MRI showed no abnormalities; however, chest CT revealed a mediastinal lesion extending into the vertebral canal from D1 to D4. Spine MRI confirmed spinal cord compression and invasion at this level. Biopsy identified the mass as a small cell lung cancer of the left apex (Pancoast tumour). Despite corticosteroid and radiotherapy treatment, the condition worsened, leading to paraparesis and urinary retention. The patient died 1 month after diagnosis.

**Figure:**
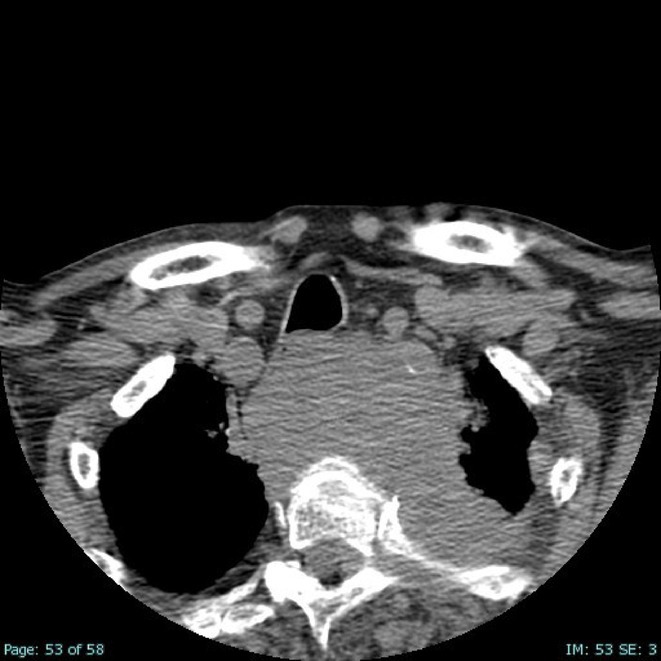
**FIGURE 1** Chest CT showing a large solid mass with origin in the left pulmonary apex and invasion of the aorta and mediastinum.

**Figure:**
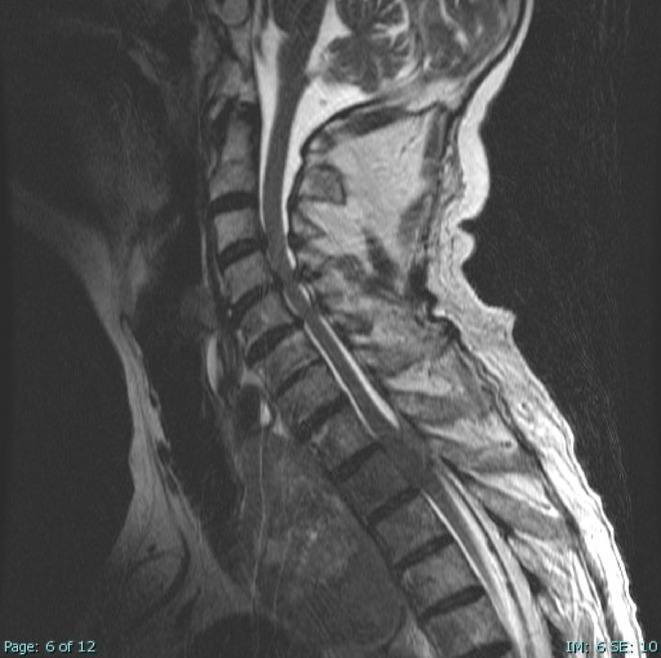
**FIGURE 2** Spine MRI showing a large mass with signs of bone invasion of the vertebral bodies from D1 to D4, extension into the intraspinal region at these levels and compression of the spinal cord.

**Conclusion:** Pancoast tumors can present with neurological deficits mimicking brainstem syndromes due to compression of the spinal cord, recurrent laryngeal nerve, and sympathetic pathways, prompting a diagnostic challenge in distinguishing between central and peripheral causes of these symptoms. Early recognition and advanced imaging are crucial to avoid misdiagnosis and ensure appropriate treatment.

**Disclosure:** Nothing to disclose.

## EPO‐230

### Upper extremities neuropathy, industrial mercury intoxication: Immunopathology mechanisms

#### N. Kolmykova; M. Kustov; D. Labunskiy; S. Kiryukhina; N. Kurgaev

##### Ogarev Mordovia State University

**Background and aims:** Distinctive features of the clinical picture of chronic mercury intoxication are tremor, erethism (increased neuropsychic excitability and irritability) and pronounced vegetative disorders, sleep disorders, emotional and cognitive impairment, gingivitis. Neuropathy is a pathology of nervous system, in which the structure of one or a group of nerves is damaged. There is pain syndrome, weakness, numbness of limbs, tingling. As a result, function of nerve fibers is impaired, after which they cannot correctly transmit electrical impulses. Nerve damage in our cases were caused by industrial mercury intoxication. Depending on extent of damage, toxic mononeuropathies and polyneuropathies are distinguished.

**Methods:** Under our observation were 76 mercury induced neuropathy patients, men and women aging from 29 to 57 years old. We performed stabilography, a method for quantitatively studying characteristics of posture control, based on measuring the coordinates of pressure center in plane of support, carried out using a stabiloplatform.

**Results:** It was revealed that mercury intoxication neuropathy affects predominantly upper extremities. In neuropathies and polyneuropathies, clinical syndromes characterized by isolated or diffuse damage to peripheral nerve fibers, unit of damage is mainly fibers that make up various nerves, probability of damage to which depends on their length, caliber, antigen composition, metabolic intensity, etc.

**Conclusion:** Clinical manifestations of polyneuropathies, considered as widespread, symmetrical, usually distal and progressive nerve damage, vary widely, differing in the rate of progression, severity of symptoms, the ratio of sensory and motor disorders, and presence of irritation symptoms. Immunoglobulins IgA, IgM and IgG were increased in almost all patients.

**Disclosure:** Nothing to disclose.

## EPO‐231

### Cerebral venous thrombosis associated with Behcet's disease in 24 Morrocan patients

#### J. Oumerzouk

##### Military Hospital Mohamed V of Rabat‐Morocco, Rabat, Morocco

**Background and aims:** Cerebral venous thrombosis (CVT) is the most common manifestation of vasculo‐Behc¸et's disease and may be superficial and/or deep localization. The aim of our study was to evaluate the clinical and radiological features of CVT associated with Behc¸et's disease in our population and to compare findings with previous studies.

**Methods:** We report a retrospective study of 24 cases of CVT secondary to Behc¸et's disease, collected between 1999 and 2019. The diagnosis of Behc¸et's disease was made in all cases according to the 2014 International Study Group Criteria for Behc¸et diseases. Patients received antithrombotic treatment, combined with corticosteroids, in six cases of superficial CVT and with immunosuppressants in cases of deep CVT.

**Results:** The diencephalic‐mesencephalic syndrome was found in 18 patients, whereas intracranial hypertension (71%) and headache (57%) were the most common presentations of superficial CVT. Unlike previous studies, magnetic resonance angiography and conventional angiography performed in our patients confirmed the predominance of deep venous thrombosis (18 cases), whereas superficial CVT was observed only in six cases.

**Conclusion:** Shortterm outcome was favorable, but sequelae of CVT were noted in 20 patients (75%). The outcome of patients was commonly mRS 02, however 70% of patients presenting with deep CVT at the beginning had a poor outcome (mRS 03) and we did not record any case of venous thrombosis relapse.

**Disclosure:** Nothing to disclose.

## EPO‐232

### Hyperglycemia‐induced acute neurological syndromes: Insights from a portuguese retrospective study

#### M. M. Roque ^1^; A. Montalvo^1^; F. Dourado Sotero^1^; A. Antunes^2^; L. Albuquerque^2^

##### ^1^Neurology Service, Department of Neurosciences and Mental Health, ULS Santa Maria, Lisbon, Portugal; ^2^Egas Moniz Study Center, University Neurology Clinic, Faculty of Medicine, University of Lisbon, Lisbon, Portugal

**Background and aims:** Uncontrolled hyperglycemia in patients with Diabetes Mellitus (DM) can present with various acute neurological symptoms, such as symptomatic seizures or movement disorders. We aim to characterize demographics, clinical presentation, laboratory, and imaging studies of patients with acute focal neurological manifestations associated with hyperglycemia.

**Methods:** Retrospective observational study including patients admitted to our Neurology department between January 2017 and August 2024 with acute neurological manifestations and a final causal diagnosis of hyperglycemia. Demographic, clinical, laboratory, and imaging data were collected and analyzed descriptively.

**Results:** Eight patients, all female with a mean age of 65 years, were included. Five had type 2 DM, two had type 1 DM, and one was newly diagnosed. The average disease duration was 20 years, with five patients exhibiting microvascular complications and one was newly diagnosed. Neurological manifestations included focal motor seizures (3), focal seizures with contralateral hemichorea‐hemiballism syndrome (1), isolated hemichorea‐hemiballism syndrome (2), central pontine myelinolysis (1), and acute diabetic neuropathy (1). Admission glucose levels exceeded 300 mg/dL in all cases, with a median HbA1c of 11.25%, indicating poor chronic glycemic control. MRI findings were typical in most cases, with specific patterns correlating to clinical presentations. Metabolic control led to neurological improvement in all patients.

**Conclusion:** Hyperglycemia remains a significant, underrecognized cause of neurological emergencies in Europe. Early recognition of diabetic causes in acute focal neurological syndromes, especially in emergency settings, is crucial for timely and effective treatment, emphasizing the central role of glycemic control in recovery.

**Disclosure:** Nothing to disclose.

## Headache 2

## EPO‐233

### Potential genetic link of chronic migraine with detoxification and nitric oxide pathways

#### A. Yakubova

##### Openlab “Gene and Cell Technologies”, Kazan Federal University, Kazan, Russian Federation

**Background and aims:** Chronic migraine (CM) is a multifactorial condition, affecting up to 5% of individuals diagnosed with migraine. This suggests the existence of specific genetic factors that contribute to its chronification. To explore this hypothesis, a comparative genetic study was conducted between CM patients and a healthy population control to identify genetic patterns and associations potentially associated with the chronic form of migraine.

**Methods:** Our study included 16 female CM patients. Population control data were derived from the European cohort of the 1000 Genomes Project. A targeted next‐generation sequencing approach was performed using the PGRNseq‐NDD panel, specifically designed to investigate 186 genes linked to inflammation, immune response, oxidative stress, neurodegeneration, metabolism and detoxification pathways.

**Results:** The analysis identified four single nucleotide polymorphisms (SNPs) with significantly different genotype distributions in the CM group compared to controls: rs34504481 in ARG1 (p = 0.049), rs8192925 in CES2 (p = 0.044), rs62359375 in MOCS2 (p = 0.035), and rs548541129 in SLCO2B1 (p = 0.010), (Fig. 1). These SNPs had not been previously reported in available genetic databases. Notably, the ARG1 gene is associated with increased nitric oxide production, while MOCS2, CES2, and SLCO2B1 are involved in xenobiotic and endogenous substance detoxification.

**Figure:**
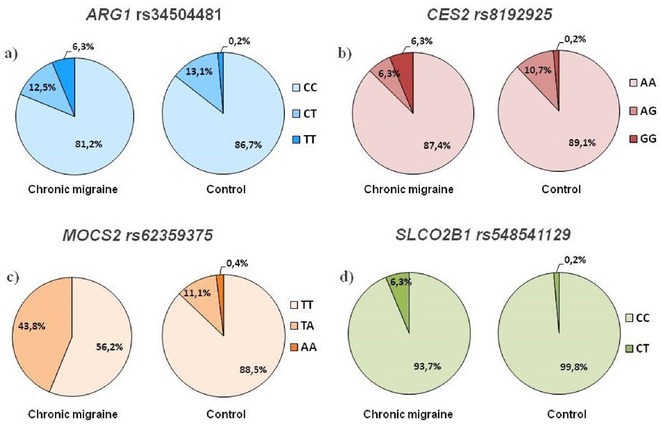
**FIGURE 1** Distribution of genotypes in chronic migraine patients and population control from the 1000 Genomes Project for the following single nucleotide polymorphisms: a) rs34504481 ARG1, b) rs8192925 CES2, c) rs62359375 MOCS2, d) rs548541129 SLCO2B1

**Conclusion:** Thus, this study highlights a potential link between CM and genetic variations in ARG1, CES2, MOCS2 and SLCO2B1 genes. The findings suggest that mechanisms such as increased nitric oxide production and impaired detoxification processes may contribute to the development of chronic migraine.

**Disclosure:** This study was supported by the Kazan Federal University Strategic Academic Leadership Program (PRIORITY‐2030).

## EPO‐234

### Study design of CORNERSTONE: A prospective observational study of atogepant effectiveness in routine clinical practice

#### E. Leroux ^1^; A. Gantenbein^2^; K. Carr^3^; Y. Liu^3^; B. Dabruzzo^3^; P. Gandhi^3^; S. Caughlin^3^; T. Alsaadi^4^; C. Tassorelli^5^; J. McVige^6^

##### ^1^Montreal Neurological Clinic, Montreal, QC, Canada; ^2^Neurology Department, ZURZACH Care, Bad Zurzach, Switzerland; ^3^AbbVie, North Chicago, USA; ^4^American Centre for Psychiatry & Neurology, UAE; ^5^Department of Brain and Behavioral Science, University of Pavia, Pavia, Italy; Headache Science and Neurorehabilitation Centre, IRCCS C. Mondino Foundation, Pavia, Italy; ^6^DENT Neurologic Institute, Amherst, NY

**Background and aims:** Atogepant is an oral calcitonin gene‐related peptide receptor antagonist approved for the preventive treatment of migraine. The Prospective Observational Study of Atogepant Effectiveness in Routine Clinical Practice(CORNERSTONE) study is designed to document the patient's real‐world experience and measure the effectiveness, tolerability, and safety of atogepant for the preventive treatment of migraine.

**Methods:** This multi‐country, prospective, observational study will enroll adult patients with a minimum 1‐year migraine diagnosis, who independently initiate atogepant per local standard of care prior to inclusion. The study includes a 28‐day pre‐atogepant retrospective recall period, 96‐week treatment period, and 30‐day follow‐up and safety period (Figure 1). Monthly Headache Days occurring in the 28 days prior to each study visit will be estimated by patient recall, optionally aided by a headache diary. Patients on other preventive migraine medications must maintain a stable regimen for at least three months before enrollment, and prescribers must confirm that there are no plans to alter this regimen during the first 12 weeks after enrollment.

**Figure:**
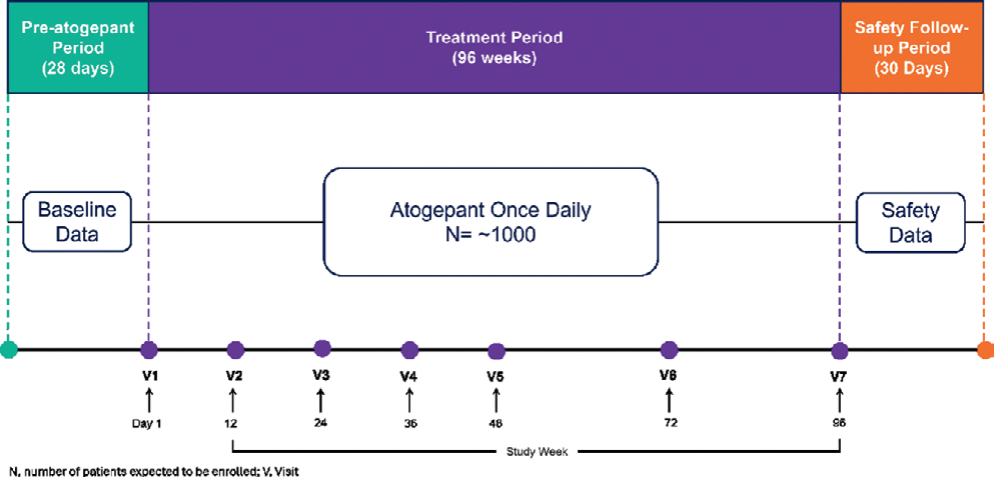
**FIGURE 1** Study design

**Results:** The study aims to enroll 1000 patients from 100 sites in 15 countries to provide necessary precision. The primary endpoint of the study is the achievement of “much better” or “very much better” at Week 12, as assessed by Patient Global Impression of Change (PGIC), recorded via electronic patient‐reported outcomes devices during clinical visits. Secondary endpoints include effectiveness and functional outcomes (Table 1). Adverse events and vitals will be monitored through the follow‐up safety period.

**Figure:**
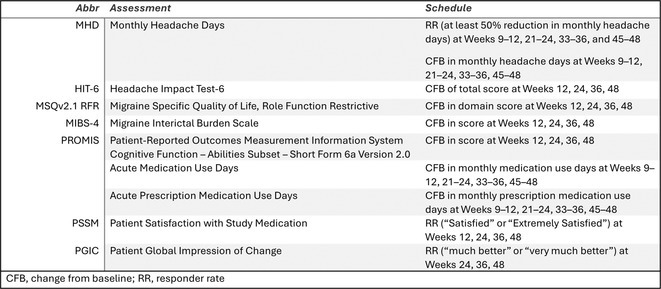
**TABLE 1** Secondary effectiveness and functional outcomes.

**Conclusion:** CORNERSTONE will provide clinically meaningful insights into the real‐world effectiveness of atogepant in routine clinical practice.

**Disclosure:** Elizabeth Leroux has received consultancy fees and/or speaker fees from Abbvie, Eli Lilly, Miravo, Lin Pharmaceutical, Lundbeck, Novartis, Paladin, Pfizer, and Teva. Andreas R. Gantenbein serves as consultant for, advisory board member of, or has received honoraria or research support from AbbVie, Allergan, Biomed, Curatis, Eli Lilly, Lundbeck, Neurolite, Novartis, Pfizer, and Teva. Karen Carr, Yingyi Liu, Brett Dabruzzo, Pranav Gandhi, and Sarah Caughlin are employees of AbbVie and may own AbbVie stock. Taoufik Alsaadi has received consultancy fees, speaker fees, and research grants from Novartis, Eli Lilly, GlaxoSmithKline, Lundbeck, Pfizer, Hikma and AbbVie. Cristina Tassorelli has participated in advisory boards for AbbVie, Dompé, Eli Lilly, Ipsen, Lundbeck, Medscape, Pfizer and Teva, and has lectured at symposia sponsored by AbbVie, Eli Lilly, Ipsen, Lundbeck, Pfizer and Teva. She is principal investigator or collaborator in clinical trials sponsored by AbbVie, Eli Lilly, Ipsen Lundbeck, Pfizer and Teva. She has received research grants from the European Commission, the Italian Ministry of Health, the Italian Ministry of University, the Migraine Research Foundation, and the Italian Multiple Sclerosis Foundation. Jennifer McVige has served as a speaker and/or received research support from Allergan (now AbbVie Inc.), Alder, Amgen/Novartis, Avanir, Biohaven, Eli Lilly, Lundbeck, and Teva.

## EPO‐235

### Effectiveness of anti‐CGRP monoclonal antibodies in chronic migraine refractory to onabotulinumtoxinA: Re‐MATe study

#### F. Salazar Hernández; M. Ruiz Perelló; B. Gómez Gozálvez; J. Bermejillo Barrera; A. Savolainen; D. López Segura; D. Vidal Mena; J. Fajardo Sanchís; E. Fages Caravaca; M. Cerdán Sánchez; A. Báidez Guerrero; M. Martínez Zarco; M. Ortega Ortega; J. García‐Carmona

##### Neurology Department, Hospital Universitario Santa Lucía de Cartagena, Murcia, Spain

**Background and aims:** Chronic migraine (CM) refractory to onabotulinumtoxin A (BoNT‐A) treatment refers to a condition where patients experience persistent, frequent migraines despite toxin injections. This form of CM remains resistant to conventional therapy, making it more challenging to manage and requiring alternative treatment approaches. We aimed to evaluate the clinical efficacy and safety of monoclonal antibodies targeting calcitonin gene‐related peptide (anti‐CGRP) in reducing the symptoms and frequency of migraine in patients diagnosed with CM refractory to BoNT‐A in clinical practice.

**Methods:** Re‐MATE (Real‐Migraine Antibodies Treatments Evidence) is an observational, retrospective study comparing the following variables at 3 and 6 months after initiating an anti‐CGRP treatment: number of monthly migraine days, number of days using rescue medication, retention rate at 6 months, and side effects.

**Results:** Seventy patients were included, 57 (81%) women, with an average age of 50.1±1.3 years, previously treated for 12.7±2.4 months with BoNT‐A. anti‐CGRP treatment significantly reduced the number of headache days and the use of rescue medication days per month (16.6±0.9 and 15.9±0.8, respectively) at 3 months (5.20±0.64, t1,58=12.5, p=0.001 and 4.69±0.62, t1,58=11.46, p=0.001, respectively) and at 6 months (4.07±0.67, t1,53=11.12, p=0.001; 3.91±0.72, t1,53=10.28, p=0.001). Furthermore, 3 (15%) patients were migraine‐free at 3 months and 5 (8%) at 6 months. Four (7%) patients reported side effects, and the adherence rate at 6 months was 91%.

**Conclusion:** Anti‐CGRP treatment was effective in patients diagnosed with CM refractory to BoNT‐A, showing high levels of compliance and safety at 6 months.

**Disclosure:** Nothing to disclose.

## EPO‐236

### Eptinezumab reduced disease burden in chronic migraine and medication‐overuse headache: Secondary RESOLUTION trial data

#### S. Tepper^1^; H. Schytz ^2^; R. Jensen^2^; C. Lundqvist^3^; G. Terwindt^5^; C. Tassorelli^6^; F. Vernieri^8^; M. Lantéri‐Minet^10^; A. Blumenfeld^12^; M. Josiassen^13^; G. Jansson^13^; A. Ettrup^13^; A. Mittoux^13^; R. Lipton^14^

##### ^1^The New England Institute for Neurology and Headache, Stamford, USA; ^2^Danish Headache Center, Department of Neurology, Rigshospitalet‐Glostrup, University of Copenhagen, Copenhagen, Denmark; ^3^Departments of Neurology and Health Services Research, Akershus University Hospital, Lørenskog, Norway; ^5^Department of Neurology, Leiden University Medical Centre, Leiden, Netherlands; ^6^Department of Brain and Behavioral Sciences, University of Pavia, Pavia, Italy; ^8^Unit of Headache and Neurosonology, Fondazione Policlinico Campus Bio‐Medico, Rome, Italy, ^10^Pain Department and FHU InovPain, Centre Hospitalier Universitaire de Nice, Nice, France, ^12^The Los Angeles Headache Center, Los Angeles, USA, ^13^H. Lundbeck A/S, Copenhagen, Denmark, ^14^Department of Neurology, Albert Einstein College of Medicine, New York, USA

**Background and aims:** The RESOLUTION trial assessed the efficacy and safety of eptinezumab vs placebo when given in addition to patient education in chronic migraine (CM) and medication‐overuse headache (MOH). The trial met its primary and all key secondary endpoints. Here we report the impact of eptinezumab vs placebo on multiple patient‐reported outcomes (PROs) measuring headache‐related burden and quality of life, migraine‐related disability, and work productivity and activity impairment.

**Methods:** RESOLUTION (NCT05452239) included a 28‐day screening (baseline) period and a 12‐week double‐blind, placebo‐controlled period. Adults (18‐75y) with CM and MOH (excluding opioid‐overuse headache) were randomised (1:1) to IV eptinezumab 100 mg or placebo, both with a Brief Educational Intervention about MOH prior to infusion. Secondary endpoints included several PROs assessing disease burden and health‐related quality of life at Weeks 4 and 12.

**Results:** Of 608 participants randomised, 596 (98.0%) completed the placebo‐controlled period. Baseline scores were indicative of moderate to severe disease burden. Eptinezumab treatment was associated with greater improvements compared to placebo at Week 4 across all PRO scores (Table; HIT‐6, mMIDAS, WPAI:M, MSQ, EQ‐5D‐5L, HADS, and TSQM‐9). Greater improvements with eptinezumab vs placebo were sustained at Week 12.

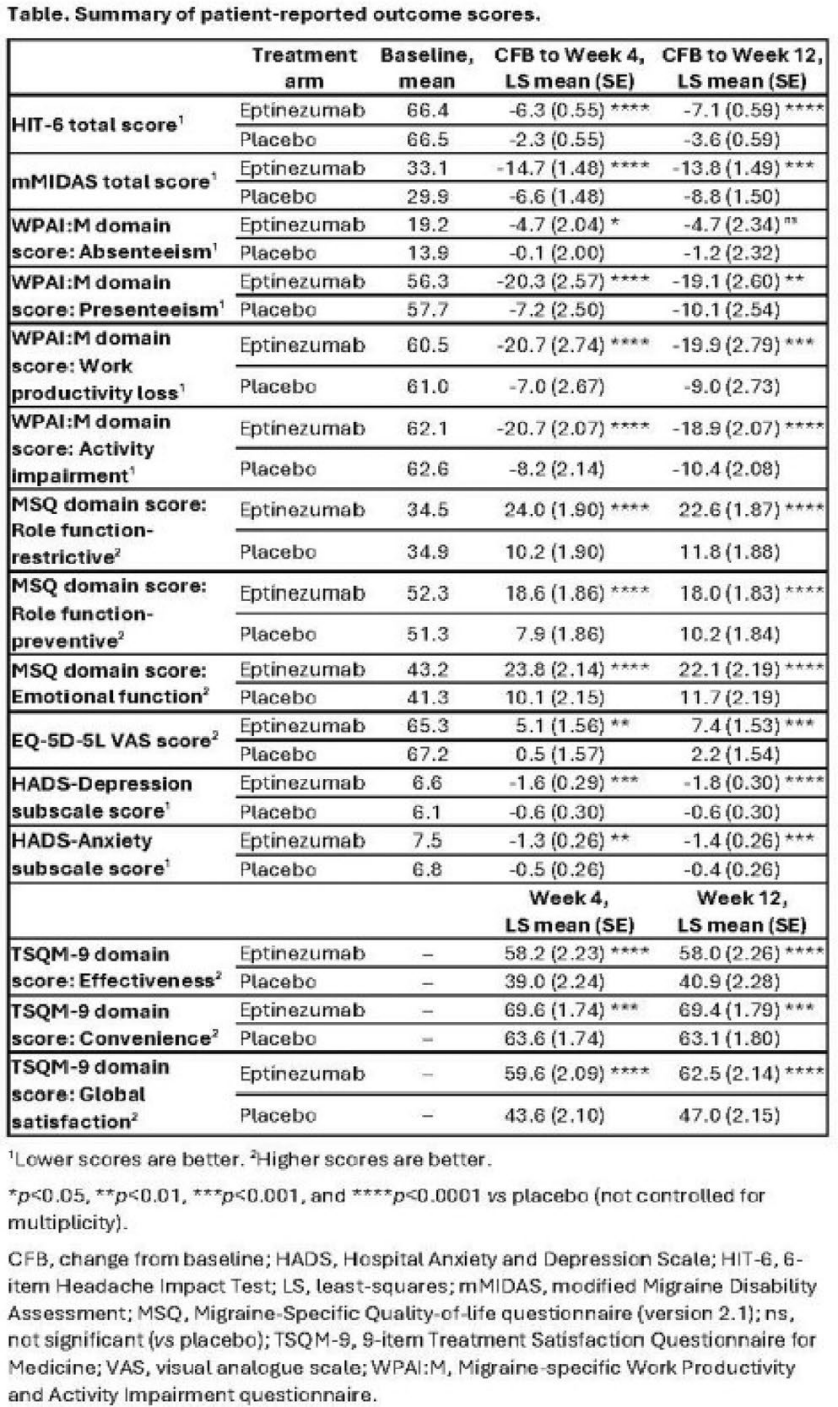

**Conclusion:** Eptinezumab resulted in greater improvements than placebo across all patient‐reported outcome measures at the first post‐baseline timepoint (Week 4) in patients with CM and MOH also receiving patient education. Improvements in headache‐related life impact, migraine‐related disability, work productivity and activity impairment, and health‐related quality of life continued to favour eptinezumab vs placebo at Week 12.

**Disclosure:** Trial sponsored by Lundbeck.

## EPO‐237

### IIH without papilledema in chronic migraineurs and revisiting of Friedman's diagnostic criteria

#### I. El Malky ^1^; H. Abdelkhalek^2^; M. Abdelhafiz^1^

##### ^1^South Valley Unversity, Egypt; ^2^Tanta University, Egypt

**Background and aims:** The application of revised Friedman's criteria to diagnose IIH WOP will prevent many patients from proper diagnosis and treatment. Our prospective study aimed to compare the prevalence of IIH WOP in case of following Friedman's criteria and in case of novel proposed criteria (OP > 200 mmH2O and radiological finding ≤ two), also reporting the predictive radiological signs for IIH WOP.

**Methods:** Patients underwent ophthalmologic, neurological evaluation, MRI, and a lumbar puncture (LP) with opening pressure (OP) measurement. CSF withdrawal was performed in patients with CSF OP > 200 mmH20. IIHWOP was defined according to Friedman's criteria. The effect of CSF withdrawal was evaluated clinically after two months.

**Results:** One hundred and two consecutive CM patients were enrolled (95 F, age 32.34 ± 9.45, and BMI 29.04 ± 5.89) without papilledema. Eighteen patients (17.65%) had OP greater than 250 mmH2O, and 20 patients (19.61%) with OP ≥ 200 mmH2O and ≤ 250 mH2O. Prevalence of suggested IIH WOP was applied only in three patients (2.9%). In case of violation of these criteria (Absent 6th nerve palsy, ICP > 200 mmH2O, and ≥ two radiological signs), more five patients were added to IIH WOP (7.8%). After CSF withdrawal, 85% of cases with OP > 200 mm H2O improved.

**Conclusion:** The prevalence of IIH WOP with the novel proposed diagnostic criteria increased to 7.8 % in comparison to 2.9% in the case of revised Friedman's criteria. Bilateral TSS was the predictor for IIH WOP.

**Disclosure:** Nothing to disclose.

## EPO‐238

### Sudden severe headache in the emergency department

#### A. Sjulstad^1^; O. Brekke^2^; F. Odeh^1^; H. Knutsen^1^; K. Alstadhaug ^3^

##### ^1^Department of Neurology, Nordland Hospital Trust, Bodø, Norway; ^2^Department of Clinical Biochemistry, Nordland Hospital Trust, Bodø, Norway; ^3^Institute of Clinical Medicine, UIT, The Arctic University of Norway, Tromsø, Norway

**Background and aims:** There is limited data on patients arriving the emergency department (ED) with sudden onset severe headache (SOSH), a subgroup of headache that may indicate a subarachnoid haemorrhage (SAH). Having applied the gold standard to differentiate SAH from other aetiologies, we wanted to assess the prevalence and the final diagnoses of such patients.

**Methods:** The medical records of every awake and alert patient with SOSH admitted to Nordland Hospital 2008‐2020 was scrutinized. Rehospitalisation or death associated with the headache was monitored until April 2022.

**Results:** A total of 588 patients, with mean age of 42.5 ± 17.9 years (61.6% female, 38.4% male), were identified, representing 0.4% of all the ED admissions. Half (49.7%) presented with thunderclap headache. Twenty percent (20.2%) were diagnosed with a secondary headache, of which half (9.9%) had an SAH, including 38 (6.5%) with an aneurysmal SAH. Most patients, 338 (57.5%), received an unspecific headache diagnosis. No deaths or readmissions attributed overlooked SAH were recorded by the final review in 2022.

**Conclusion:** SOSH represents only a small proportion of hospital admissions. At least 2 in 10 will have a secondary headache, and 1 in 10 will have a subarachnoid haemorrhage, of which two‐ thirds are attributed to an aneurysmal rupture. The majority of patients are discharged with an unspecific headache diagnosis. Our data underscore the importance of thorough evaluation of SOSH to identify SAH, but also the need for improved diagnostics to differentiate between other acute headache aetiologies.

**Disclosure:** Nothing to disclose.

## EPO‐239

### Refractory chronic cluster headache: Exploring the potential of repetitive transcranial magnetic stimulation

#### L. Portocarrero‐Sánchez ^1^; C. Rizea^1^; E. Díez‐Tejedor^2^; A. Sánchez‐Huertas^1^; M. León‐Ruiz^1^; J. Díaz‐de‐Terán^2^

##### ^1^Neurology, Hospital Universitario La Paz, Madrid, Spain; ^2^Neurology, Hospital La Paz Institute for Health Research – IdiPAZ (La Paz University Hospital – Universidad Autónoma de Madrid) Madrid, (Spain)

**Background and aims:** Chronic cluster headache (CCH) presents significant therapeutic challenges, particularly in refractory cases. Repetitive transcranial magnetic stimulation (rTMS) has emerged as a potential alternative, though evidence for its efficacy in CCH remains limited.

**Methods:** We conducted a randomized, double‐blind, placebo‐controlled, crossover pilot study to evaluate rTMS in patients with refractory CCH. Participants were randomized into two sequences: A (rTMS followed by sham) or B (sham followed by rTMS), each treatment period consisting of 10 consecutive working days, with a one‐month washout period and a three‐month follow‐up. The primary outcome was change in number of attacks per week (APW). Secondary outcomes included treatment tolerability, side effects and changes in attack intensity, duration, and rescue medication use.

**Results:** Eight patients were enrolled (5 in sequence A, 3 in sequence B), with three completing the full study. No significant effectiveness was achieved after rTMS period, although two patients experienced complete remission after day 4 of treatment. However, symptoms recurred 7 days after last treatment session. Secondary outcomes remained unchanged. Side effects were mild and transient, occurring in two cases (tingling and nuisance). Three of five dropouts were attributed to logistical challenges, such as the time commitment required for daily visits and a lack of perceived benefit early in treatment. The remaining two were due to lack of efficacy.

**Figure:**
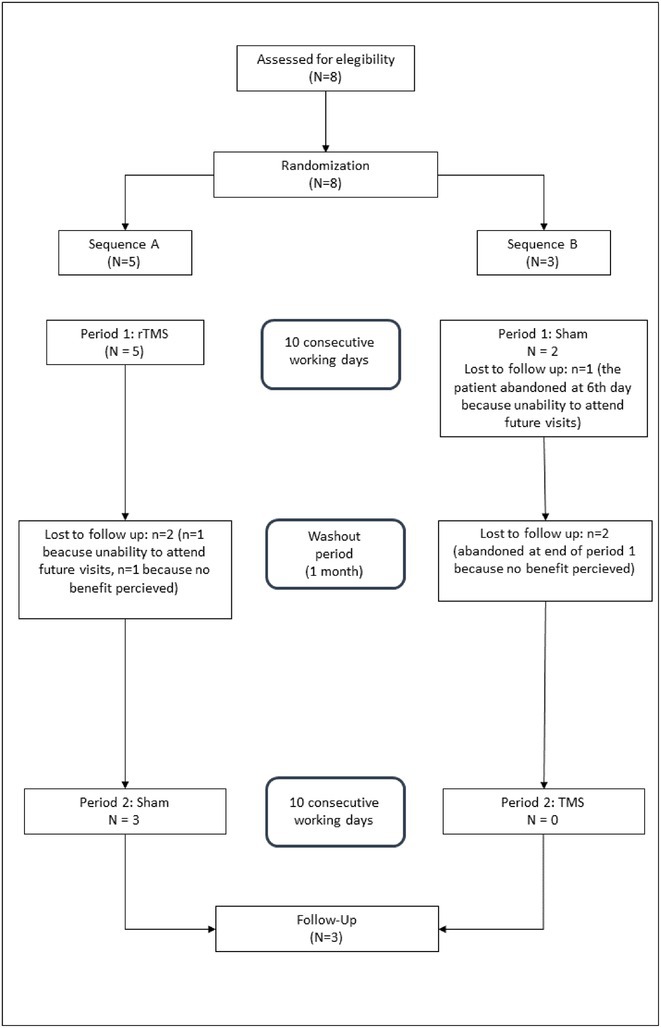
**FIGURE 1** Flow diagram.

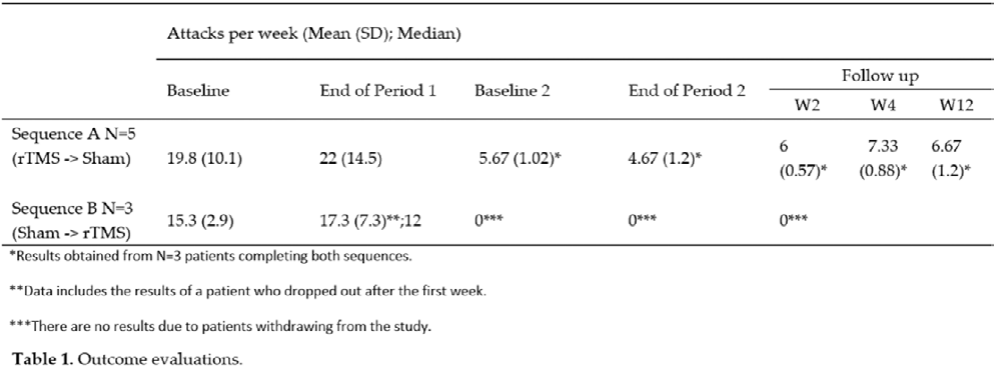

**Conclusion:** This pilot study suggests that rTMS may have some benefit in selected refractory CCH cases, but its effects appear short‐lived. Maintaining treatment adherence is a key challenge and it depends on the protocol used.

**Disclosure:** Nothing to disclose.

## EPO‐240

### PACAP6‐38 reduces nitroglycerin‐induced central sensitization by modulating synaptic plasticity in the TNC of rats

#### Z. Xiao

##### Department of Neurology, Renmin Hospital of Wuhan University, Wuhan, China.

**Background and aims:** Chronic migraine (CM) is a common neurological disorder with complex mechanisms. Pituitary adenylate cyclase‐activating peptide (PACAP) has been linked to migraine attacks, but targeting PACAP and its receptors shows varying therapeutic results. This study explores the effect of PACAP type I receptor (PAC1R) antagonist, PACAP6‐38, on nitroglycerin (NTG)‐induced central sensitization in CM.

**Methods:** CM was induced in Sprague‐Dawley rats via repeated NTG injections. Mechanical and thermal thresholds were measured, and central sensitization was assessed by c‐Fos expression. PACAP6‐38 was injected into the trigeminal nucleus caudalis (TNC). Synaptic proteins, phospho‐ERK1/2, p‐CREB, BDNF, and synaptic structures were analyzed by western blotting, immunofluorescence, TEM, and Golgi‐Cox staining.

**Results:** PACAP and PAC1R expression were elevated in the TNC following NTG injections. PACAP6‐38 treatment alleviated nociceptive sensitization, inhibited c‐Fos overexpression, restored synaptic structures, and reduced the ERK/CREB/BDNF pathway activation.

**Conclusion:** PACAP6‐38 improves NTG‐induced central sensitization by modulating synaptic plasticity in the TNC, likely through the ERK/CREB/BDNF pathway. This suggests that PACAP/PAC1R may be a novel target for migraine treatment.

**Disclosure:** Nothing to disclose.

## EPO‐241

### When two headaches collide: The importance of treating chronic migraine in cluster headache chronification

#### M. Villar Martinez; P. Amarasena; L. Bastos‐Alves; N. Karsan; D. Moreno‐Ajona; P. Goadsby

##### Wolfson Sensory Pain and Regeneration, Institute of Psychiatry, Psychology and Neuroscience, King's College London, UK

**Background and aims:** The extreme severity of cluster headache (CH) symptoms usually overshadows migraine symptoms in the clinical settings, leading both patients and clinicians potentially to dismiss the significance of migraine symptoms. In this study, we explore the relationship between the two, and the predictors that may contribute to increased risk of CH chronification.

**Methods:** Data from successive new consultations of patients with CH referred to the Acute Service at King's College Hospital, London from 2018‐2024, was collected (n=118). A generalised linear model using binomial distribution and logit link function was used to evaluate predictors of having chronic CH, taking episodic CH as reference and age, sex, age of cluster onset, years since the diagnosis, number of cluster preventives during the assessment, chronic migraine and number of abortive treatments used: triptans and oxygen.

**Results:** Our predictors significantly improved the model fit (χ = 20.79, Df = 7, P = 0.004). When abortive treatments were included in the model, despite not being itself significant, chronic migraine became a significant predictor of chronification (β=1.92, P=0.049). Older age of onset (β=0.04, P=0.042) and number of cluster preventives (β = 0.74, P<0.006) were predictors of chronification. The remaining variables were not significant.

**Conclusion:** Patients with both CH and CM who take acute treatments daily may have a higher likelihood of progressing to chronic cluster headache. This highlights the importance of not only treating cluster headache preventively but also addressing the underlying chronic migraine condition, which can play a pivotal role in chronification.

**Disclosure:** Nothing to disclose.

## EPO‐242

### Retinal changes in optical coherence tomography in migraine

#### M. Dauti; T. Kölsche; J. Lee

##### Department of Neurology, Medical Faculty and University Hospital Düsseldorf, Heinrich Heine University Düsseldorf, Germany

**Background and aims:** Migraine affects approximately 15% of the global population and significantly impairs quality of life. Structural and vascular retinal changes, including reductions in the retinal nerve fiber layer (RNFL) and ganglion cell layer (GCL), as well as enlargement of the foveal avascular zone (FAZ), have been associated with migraine. This study evaluates retinal alterations in migraine patients, examines longitudinal changes, and investigates associations with migraine subtypes and prophylactic therapies.

**Methods:** In this prospective study, 40 participants (20 migraine patients, 20 age‐ and sex‐matched controls) underwent ophthalmological examinations, optical coherence tomography (OCT), and OCT angiography (OCT‐A) at baseline, 6 months, and 12 months. Migraine patients were classified into episodic (25%) and chronic (75%) subtypes. Retinal layer thickness (RNFL and GCL) and vascular parameters including vessel density in the superficial, intermediate, and deep capillary plexuses and FAZ area, were assessed.

**Results:** Preliminary analysis of 40 participants (20 migraine patients, 20 controls) revealed no significant differences in RNFL or GCL thickness. However, the FAZ was significantly larger in migraine patients, particularly in the left eye (p < 0.01). Vessel density in the intermediate and deep capillary plexuses was also significantly reduced in the left eye, with a non‐significant trend in the right eye.

**Conclusion:** These initial findings indicate migraine‐associated retinal vascular changes, characterized by FAZ enlargement and reduced vessel density, predominantly in the left eye. Further analyses, planned as part of this ongoing study, aim to validate these results, stratify by migraine subtypes (e.g. with and without aura) and assess possible associations with prophylactic therapies.

**Disclosure:** MD, SK, AK, PN, JI, RJ, EA, AA, PJ, and RG declare no conflicts of interest. TK and VK have received travel grants from AbbVie, Ipsen, and Merz (unrelated to the submitted work). SGM has received honoraria, travel support, and research funding from Bayer, Biogen, Sanofi, Merck, Novo Nordisk, Genzyme, MSD, and Teva. PA has received honoraria, travel support, and research funding from Novartis, Biogen, Merz, Teva, Ipsen, Allergan, Celgene, Janssen Cilag, Roche, Merck, Sanofi, and Sandoz. JIL has received honoraria, travel support, and research funding from Boehringer Ingelheim, Daiichi Sankyo, Allergan, AbbVie, Ipsen, Novartis, Teva, Lilly, and Pfizer, as well as research grants from Merz to the University Hospital Düsseldorf for projects involving JIL.

## EPO‐243

### Does migraine predict dizziness?

#### D. Moreno; N. Karsan; M. Villar Martínez; P. Goadsby

##### NIHR King's Clinical Research Facility, & SLaM Biomedical Research Centre, and The Wolfson Sensory, Pain and Regeneration Centre (SPaRC), Institute of Psychiatry, Psychology and Neuroscience (IoPPN), King's College London, UK

**Background and aims:** Headache and dizziness combined account for more than 50% of consultations in Neurology outpatient clinics. Both vestibular migraine and Persistent Postural Perceptual Dizziness (PPPD), are common causes of dizziness. In this study, we aimed to understand the coexistence of these symptoms and whether the dizziness intensity can be predicted by migraine features.

**Methods:** Consecutive patients attending the General Neurology and Headache Clinic at Queen Elizabeth Hospital (Jan 2024‐Dec 2024, n=120) were asked to fill the Dizziness Handicap Inventory (DHI) and the Niigata PPPD Questionnaire (NPQ) if they experienced vestibular symptoms. DHI was developed to assess vestibular disorders and the NPQ for PPPD.

**Results:** Median age was 50 years (IQR, 72% females). A migraine diagnosis as per the 3‐item ID migraine could be applied to 112 patients. Ninety‐seven out of 120 (81%) complained of vestibular symptoms and completed the questionnaires. Median DHI was 14 (IQR 2‐36.5), median NPQ was 20 (IQR 5 – 43). A Wilcoxon test showed no significant difference between median DHI and NPQ (W=1467; P=0.210). Respective linear regression models with the DHI and NPQ scores as dependent variables, using gender, headache and migraine frequency, nausea, photophobia, osmophobia, movement sensitivity as predictor variables, showed migraine frequency as the only predictor variable for both (B=6.85 95% CI 4.22 – 9.47; P=0.004 for DHI and B=3.872 95% CI 0.181 – 7.562; P=0.04 for NPQ).

**Conclusion:** Even if the DHI and NPQ may reflect the severity of different aspects of vestibular symptoms, migraine frequency may predict more severe presentations of vestibular disorders and PPPD.

**Disclosure:** Nothing to disclose.

## EPO‐244

### Medication overuse headache in cluster headache

#### N. Lund ^1^; M. Søborg^1^; L. Carlsen^2^; R. Jensen^1^; A. Petersen^1^

##### ^1^Danish Headache Center, Dept.of Neurology, Rigshospitalet‐Glostrup, University of Copenhagen, Denmark; ^2^Department of Neurosurgery, University of Copenhagen, Rigshospitalet, Copenhagen, Denmark

**Background and aims:** Medication overuse headache (MOH) is a well‐described major driver of chronification in migraine. However, it is a topic of debate if MOH exists in cluster headache (CH). Therefore, we aimed to examine this and to describe the clinical characteristics associated with MOH in CH. Additionally, we aimed to explore the impact on CH treatment.

**Methods:** A large cohort of people diagnosed with CH, mainly deriving from a tertiary headache clinic, participated in a retrospective, semi‐structured interview investigating MOH according to existing criteria (ICHD‐3).

**Results:** A total of 433 people with CH were included with a male:female ratio of 2:1. Concurrent MOH could be diagnosed in 16%. Simple analgesics were the most frequently overused drug (52.2%), followed by triptans (37.3%), opioids (29.9%) and combination therapies (20.9%). Chronic phenotype (OR11.4, p<0.00001) and comorbid migraine (OR2.35, p<0.05) were associated with having concurrent MOH. Clinically, people with MOH had longer attack duration (30.0 vs. 20.0 minutes, p<0.01) and less effect of acute and preventive medication than those without MOH (20.0 vs. 55.9%, p<0.05 and 13.3 vs. 37.3%, p<0.01, respectively).

**Conclusion:** Our findings indicate that MOH can also occur in other headache disorders than migraine and tension‐type headache, including CH. If suspecting concurrent MOH in a patient with CH, we recommend that triptans should be continued due to the severity of CH attacks, but other analgesics could be discontinued, and preventive treatment sought to be optimized. Further prospective studies are warranted to identify better CH treatment and understand the effect of MOH on CH disease burden.

**Disclosure:** Nunu Lund has received a personal research grant from the Capital Region of Denmark's research foundation.

## EPO‐245

### A rare case of secondary paroxysmal hemicrania caused by a thoracic schwannoma affecting the sympathetic chain

#### R. Zwergal ^1^; F. Filppopulos^2^; A. Straube^1^; M. Wühr^2^; A. Zwergal^2^

##### ^1^Department of Neurology, LMU University Hospital, LMU Munich, Munich, Germany; ^2^German Center for Vertigo and Balance Disorders, LMU University Hospital, LMU Munich, Munich, Germany

**Background and aims:** Paroxysmal hemicrania (PH) is a rare headache disorder commonly classified as a trigeminal autonomic cephalalgia (TAC). Only few cases were reported, where a PH presentation could be convincingly aligned to a secondary pathology (e.g. a tumor).

**Methods:** Case description of a patient (male, 64y) with a rare cause of secondary PH.

**Results:** With an onset in 2011, the patient had frequent attacks of typical PH responsive to indometacin. During the attacks, the patient reported tearing and miosis of the ipsilateral eye, and a drop of heart rate (documented by a health tracker). Initially, MRI (brain and cervical spine) and neurological examination revealed no pathologies. In 2022, an MRI of the thoracic spine was performed due to new‐onset shoulder pain and tingling of legs. It depicted an extra‐spinal 2x1cm tumor at Th1‐level, locally compressing the spinal cord. After surgical removal in 2023 (pathology: schwannoma grade 1), the headache thereafter ceased completely. A reevaluation of the case led to the diagnosis of a secondary PH provoked by the Th1‐nerve root schwannoma.

**Conclusion:** A presumed sympathetic deficit has long been discussed in the pathophysiology of PH. The reported case highlights, that an affection of the Th1‐nerve root, which carries sympathetic fibers to the forehead/periorbital region, can induce PH possibly via the sympathetic‐trigeminal complex or an altered meningeal vasoregulation. Considering this, the role of sympathetic dysfunction in PH pathophysiology should be reevaluated.

**Disclosure:** Nothing to disclose.

## EPO‐246

### A new rat model of nocebo‐related nausea involving observational learning and conditioning mechanisms

#### Z. Xiao; Y. Zhang; Y. Lei

##### Department of Neurology, Renmin Hospital of Wuhan University, Wuhan, China.

**Background and aims:** The nocebo effect, such as nausea and vomiting, is one of the major reasons patients discontinue therapy. The underlying mechanisms remain unknown due to a lack of reliable experimental models. The goal of this study was to develop a new animal model of nocebo‐related nausea by combining observational learning and Pavlovian conditioning paradigms.

**Methods:** Male Sprague‐Dawley rats with nitroglycerin‐induced migraine were given 0.9% saline (a placebo) or LiCl (a nausea inducer) following headache relief, according to different paradigms.

**Results:** Both strategies provoked nocebo nausea responses, with the conditioning paradigm having a greater induction impact. The superposition of two mechanisms led to a further increase in nausea responses. A preliminary investigation of the underlying mechanism revealed clearly raised peripheral and central cholecystokinin (CCK) levels, as well as specific changes in the 5‐hydroxytryptamine and cannabinoid systems. Brain networks related to emotion, cognition, and visceral sense expressed higher c‐Fos‐positive neurons, including the anterior cingulate cortex (ACC), insula, basolateral amygdala (BLA), thalamic paraventricular nucleus (PVT), hypothalamic paraventricular nucleus (PVN), nucleus tractus solitarius (NTS), periaqueductal gray (PAG), and dorsal raphe nucleus‐dorsal part (DRD). We also found that nausea expectances in the model could last for at least 12 days.

**Conclusion:** The present study provides a useful experimental model of nocebo nausea that might be used to develop potential molecular pathways and therapeutic strategies for nocebo.

**Disclosure:** Nothing to disclose.

## EPO‐247

### Aryl hydrocarbon receptors alleviate migraine‐like pain in rats by regulating Treg/Th17 cell‐related balance

#### Z. Xiao

##### Department of Neurology, Renmin Hospital of Wuhan University, Wuhan, China.

**Background and aims:** Migraine is a neurovascular disorder with unclear pathophysiological mechanisms, but recent studies suggest immune dysfunction may play a role. The aryl hydrocarbon receptor (AHR), involved in autoimmune diseases, may be implicated in migraine, though its role remains unclear.

**Methods:** A chronic migraine rat model was created using repeated nitroglycerin (NTG) injections. Mechanical and thermal pain thresholds were measured. AHR expression in the trigeminal nucleus caudalis (TNC) was assessed, alongside Treg/Th17‐related factors. The AHR agonist ITE and antagonist CH‐223191 were used to examine their effects on pain behavior, c‐Fos, CGRP, AHR, and Treg/Th17 factors.

**Results:** NTG administration increased nociceptive hypersensitivity and enhanced c‐Fos and CGRP expression, while AHR levels in the TNC decreased. Treg/Th17‐related transcription factors showed an imbalance, with forkhead box protein P3 and STAT5 decreased, and RORγt and STAT3 increased. AHR agonist ITE alleviated pain behaviors and corrected Treg/Th17 imbalances, while AHR antagonist CH‐223191 worsened pain.

**Conclusion:** The AHR participates in the development of CM by regulating Treg/Th17‐related homeostasis. Therefore, treatments targeting the AHR/Treg/Th17 signaling pathway could be new effective interventions for CM treatment.

**Disclosure:** Nothing to disclose.

## Movement Disorders 2

## EPO‐248

### Fecal microbiota transplantation for Parkinson's disease: A systematic review and meta‐analysis

#### A. Menegaz de Almeida^1^; J. Martinez‐Lemus^2^; F. Moraes Tamashiro^3^; F. Westphal Filho^4^; P. Moss Lopes^4^; C. Miranda^5^; A. Silva Machado ^6^

##### ^1^Federal University of Mato Grosso, Sinop, Mato Grosso, Brazil; ^2^McGovern Medical School at UTHealth Houston, Houston, Texas, USA; ^3^Buenos Aires University, Buenos Aires, Argentina; ^4^Federal University of Amazonas, Manaus, Amazonas, Brazil; ^5^Bahiana School of Medical Sciences, Salvador, Bahia, Brazil; ^6^Cidade de São Paulo University, São Paulo, São Paulo, Brazil

**Background and aims:** Dysregulation of the microbiome–gut–brain axis is a key pathophysiological mechanism preceding the onset of motor symptoms in PD. FMT has been evaluated in several randomized controlled trials (RCTs) as a novel intervention for PD. However, it remains inconclusive whether FMT significantly improves PD symptoms.

**Methods:** MEDLINE, Web of Science, Scopus, and the Cochrane Library databases were systematically searched for RCTs comparing FMT with placebo in PD patients. We assessed efficacy using the Unified Parkinson's Disease Rating Scale (MDS‐UPDRS), the PD Questionnaire (PDQ‐39), Irritable Bowel Syndrome Scale (IBSS), and Wexner Constipation score.

**Results:** 3 RCTs and 145 patients were included, of whom 79 (54,48%) underwent FMT. UPDRS‐III showed no difference between groups (Mean Difference [MD] ‐2.30; 95% CI ‐4.94 to 0.34; p=0.08; I2=45%) but both MDS‐UPDRS‐II and MDS‐UPDRS‐IV subscales favored the FMT group (MD 0.78; 95% CI 0.34 to 1.23; p=0.0005; I2=0%; and MD 0.28; 95% CI 0.05 to 0.51; p=0.01; I2=0%, respectively). There was no difference in PDQ‐39, IBSS, and Wexner scale between groups (PDQ‐39 MD ‐1.35; 95% CI ‐3.55 to ‐0.86; p=0.23; I2=95%; IBSS MD 9.57; 95% CI ‐30.50 to 49.64; p=0.63; I2=40%; Wexner score MD ‐0.85; 95% CI ‐2.12 to 0.43; p=0.19; I2=87%).

**Figure:**
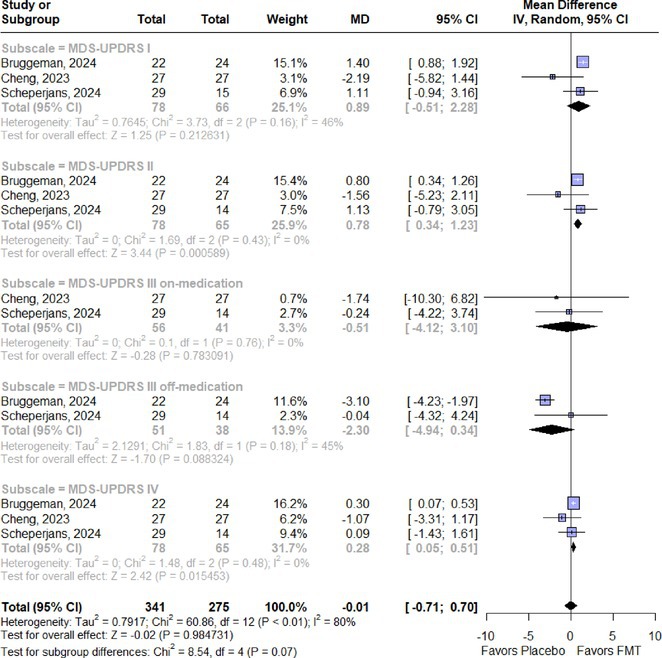
**FIGURE 1** Mean Unified Parkinson's Disease Rating Scale (MDS‐UPDRS) post treatment.

**Figure:**
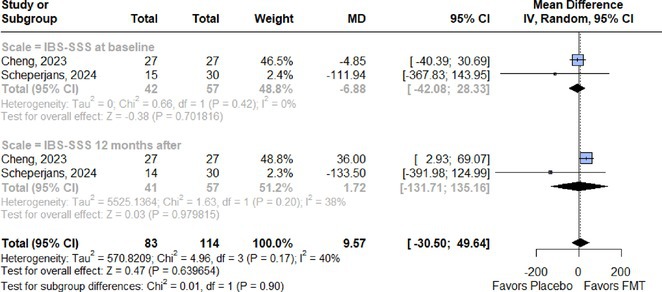
**FIGURE 2** Mean Irritable Bowel Syndrome Scale (IBSS) post treatment.

**Figure:**
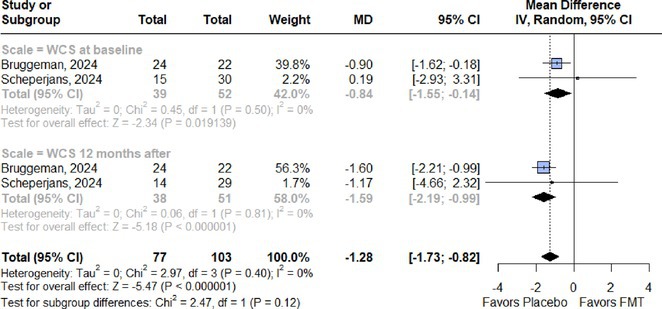
**FIGURE 3** Mean Wexner Constipation score post treatment.

**Conclusion:** Our findings indicate an improvement in Non‐Motor Experiences of Daily Activity (MDS‐UPDRS‐II) and Motor Complications (MDS‐UPDRS‐IV) in patients receiving FMT. However, we did not observe any impact on motor or constipation scores. Further trials without baseline differences between groups are needed to clarify the effects of FMT on PD.

**Disclosure:** Nothing to disclose.

## EPO‐249

### The impact of multimorbidity on symptom severity and quality of life in functional motor disorder

#### B. Křupková ^1^; M. Máčel^2^; P. Sojka^1^; L. Nováková^1^; K. Šútorová^1^; M. Jirásek^1^; T. Serranová^1^

##### ^1^Department of Neurology and Center of Clinical Neuroscience, Charles University, 1st Faculty of Medicine and General University Hospital in Prague, Prague, Czechia; ^2^Department of Psychology, Faculty of Arts, Charles University, Prague, Czechia

**Background and aims:** Functional motor disorder (FMD) is common, often associated with multiple persistent, disabling symptoms and impaired health‐related quality of life (HRQoL). The impact of co‐occurring physical illness comorbidities is underexplored; however, it may contribute to symptom manifestations and significantly affect HRQoL. Objective: To investigate the cumulative effect of multimorbidity on motor symptom severity, self‐reported symptom severity and HRQoL in FMD.

**Methods:** A total of 357 FMD patients (270 females, mean age=47.6 years, SD=12.8) years underwent detailed clinical evaluation including the Simplified FMD Rating Scale (SFMDRS) assessing motor severity. All patients completed an adapted Physical Health Questionnaire (PHQa) assessing subjective physical and neurological symptom severity, the Beck Depression Inventory (BDI), the Short Form Survey (SF‐12) for HRQoL. Based on reliable medical reports, a multimorbidity index (MMi) was calculated as a sum of major physical illness, neurological comorbidities including migraine and any psychiatric comorbidity.

**Results:** MMi significantly correlated with BDI (r=0.24, p<0.001), SFMDRS (r=0.13, p<0.05), SF‐12 (r=‐0.28, p<0.001), PHQa (r=0.34, p<0.001) scores. When controlling for age and gender a linear regression revealed the MMi was a significant predictor of PHQa (β=0.22, p<0.001) and SF‐12 (β= ‐0.25, p<0.001), but not motor symptom severity. These associations remained significant after correction for depression.

**Conclusion:** Our findings suggest that multimorbidity contributes substantially to the clinical complexity of FMD. Higher multimorbidity is associated with increased self‐reported somatic symptom severity and poor HRQoL. Further studies should investigate the role of multimorbidity in the pathophysiology of FMD to better understand its impact and inform targeted interventions.

**Disclosure:** Supported by NW24‐04‐00456.

## EPO‐250

### Intrajejunal levodopa infusion therapy – comparison of real‐world data from the ELEGANCE, GLORIA and DUOGLOBE registries

#### B. Popescu ^1^; J. Szász^2^; A. Dulamea^3^; V. Constantin^4^; M. Vasile^5^; L. Dumitrescu^1^

##### ^1^Department of Clinical Neurosciences, Colentina Clinical Hospital, and “Carol Davila” University of Medicine and Pharmacy, Bucharest, Romania; ^2^Department of Neurology, “George Emil Palade” University of Medicine, Pharmacy, Science and Technology, and Emergency Clinical County Hospital, Târgu Mureș, Romania; ^3^Department of Neurology, Fundeni Clinical Institute, and Department of Clinical Neurosciences “Carol Davila” University of Medicine and Pharmacy, Bucharest, Romania; ^4^Emergency Clinical County Hospital, Târgu Mureș, Romania; ^5^Central Military Emergency Hospital “Carol Davila”, and Department of Clinical Neurosciences “Carol Davila” University of Medicine and Pharmacy, Bucharest, Romania

**Background and aims:** The therapeutic options for patients with advanced Parkinson's disease (PD) who choose intrajejunal levodopa infusion treatment are levodopa–carbidopa intestinal gel (LCIG) or levodopa–entacapone–carbidopa intestinal gel (LECIG). GLORIA and DUOGLOBE are completed observational studies that have captured real‐world efficacy, safety and quality of life data (QoL) for LCIG. ELEGANCE is an ongoing international non‐interventional study (NCT05043103) collecting similar data on the routine clinical practice use of LECIG.

**Methods:** We compared published 12‐month outcomes data from GLORIA (Global LOng‐term Registry: DUODOPA® In patients with Advanced PD, a 2‐year international observational study) and DUOGLOBE (DUOdopa in Patients with Advanced Parkinson's Disease – a GLobal Observational Study Evaluating Long‐Term Effectiveness, a 3‐year international observational study) with those of the ELEGANCE planned interim analysis.

**Results:** The ELEGANCE analysis includes 167 patients with effectiveness data from at least one visit following their baseline assessment (V1) and who were followed up to V3 (6–12 months of treatment). The baseline demographics and clinical history of patients included in all three registries were similar (Table 1). Results showed significant reductions in daily OFF time following 12 months of LCIG or LECIG treatment with similar improvements across datasets in UPDRS part II, sleep parameters (PDSS‐2) and QoL (PDQ‐8).

**Figure:**
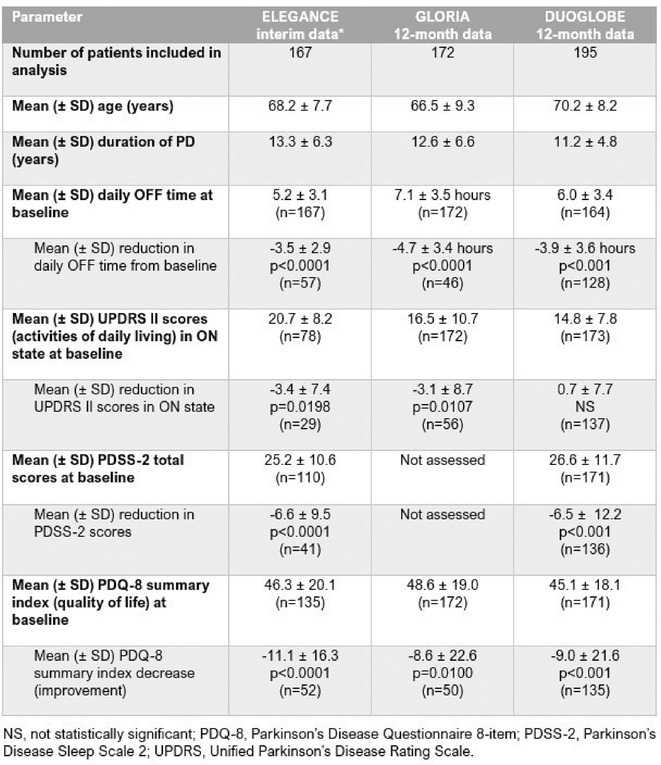
**TABLE 1** Comparison of study outcomes from the ELEGANCE interim analysis and the GLORIA and DUOGLOBE studies.

**Conclusion:** At an early stage of treatment with LECIG (up to 12 months) patients in ELEGANCE showed significant improvements in PD motor symptoms, sleep and QoL. These findings are directly comparable to observations after 12 months of treatment with LCIG in the GLORIA and DUOGLOBE studies.

**Disclosure:** Nothing to disclose.

## EPO‐251

### Limited success of GPi‐DBS in DYT‐THAP1 dystonia: Report of three cases

#### G. Yıldız; R. Yilmaz; M. Akbostancı

##### Department of Neurology, Ankara University, Ankara, Turkey

**Background and aims:** DYT‐THAP1 dystonia is a rare genetic movement disorder caused by mutations in the THAP1 gene. While globus pallidus internus deep brain stimulation (GPi‐DBS) is an established treatment for dystonia, its efficacy in DYT‐THAP1 remains inconsistent. Here, we report the clinical response to GPi‐DBS in three patients with DYT‐THAP1 dystonia.

**Methods:** A retrospective review of three DYT‐THAP1 patients treated with GPi‐DBS at Ankara University included data on age at onset, symptom duration prior to surgery, Burke‐Fahn‐Marsden Dystonia Rating Scale (BFMDRS) motor and disability scores, and speech outcomes.

**Results:** All patients exhibited spasmodic dysphonia with varying degrees of cervical, axial, and limb dystonia. Disease onset ranged from ages 7 to 25, with surgery performed after 4–27 years of symptoms. Post‐surgical motor improvement was limited, with no significant benefit observed in speech. When comparing pre‐ and post‐surgical BFMDRS scores, motor improvements of 18.8%, 0%, and 4.55% were observed. Also, no change was noted in the BFMDRS disability scores for any of the patients (Table 1). Two patients chose not to replace the battery after it had drained. Predictive factors such as early onset, long disease duration, and laryngeal involvement may have contributed to poor outcomes.

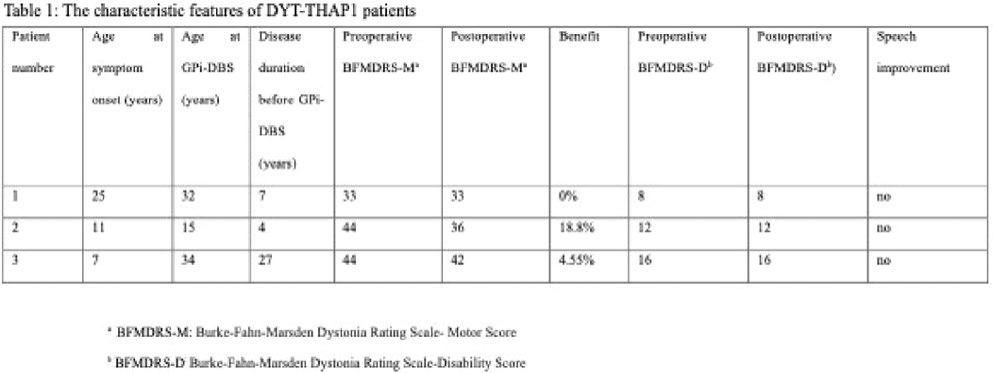

**Conclusion:** The variability in response to GPi‐DBS among patients with DYT‐THAP1 dystonia underscores the need for further studies to identify determinants of surgical efficacy. Our findings emphasize the critical role of patient‐specific factors in optimizing individualized treatment strategies and highlight the essential need for comprehensive reporting of both favorable and unfavorable outcomes to enhance evidence‐based clinical decision‐making.

**Disclosure:** Nothing to disclose.

## EPO‐252

### Subthalamic nucleus deep brain stimulation in a patient with digenic Parkinson's disease

#### D. Crosiers ^1^; F. Dijkstra^1^; L. Rumping^2^; M. De Praeter^3^; S. Tan^3^

##### ^1^Department of Neurology, Faculty of Medicine and Health Sciences, Antwerp University Hospital, Antwerpen, Belgium; University of Antwerp, Antwerp, Belgium; ^2^Department of Medical Genetics, Antwerp University Hospital; Faculty of Medicine and Health Sciences, University of Antwerp, Antwerpen, Belgium; ^3^Department of Neurosurgery, Antwerp University Hospital; Faculty of Medicine and Health Sciences, University of Antwerp, Antwerpen, Belgium

**Background and aims:** Homozygous deletions in PARK2 and PINK1 are both associated with autosomal recessive forms of Parkinson's disease (PD). We present a patient with a homozygous deletion in the PARK2, combined with a heterozygous deletion in the PINK1, who underwent bilateral subthalamic (STN) deep brain stimulation (DBS).

**Methods:** We present the clinical and genetic features of an early‐onset PD patient with pathogenic structural variants in PARK2 and PINK1. We also assessed local field potential recordings (LFP) using the DBS device. Finally, we provide a brief literature review on STN‐DBS in genetic PD.

**Results:** Favourable motor outcome of STN‐DBS is reported in PARK2‐ and PINK1‐associated PD. However, only few patients with limited follow‐up duration have been reported. LFP recordings in genetic PD patients undergoing DBS offer novel technology to longitudinally assess phenotypic features.

**Conclusion:** We report DBS outcome in a PD patient with digenic inheritance, and provide an overview of the reported monogenic PD patients undergoing DBS. Collection of additional data in longitudinal multicenter studies is needed to establish robust data and establish evidence‐based guidelines for device‐aided therapies in monogenic PD.

**Disclosure:** Nothing to disclose.

## EPO‐253

### Sex differences in Spinocerebellar ataxia type 1: clinical presentation and progression

#### F. Colucci ^1^; S. Stefanelli^2^; E. Contaldi^3^; A. Gozzi^2^; M. Pugliatti^4^; P. Antenucci^4^; J. Capone^2^; D. Gragnaniello^2^; M. Sensi^2^

##### ^1^Department of Clinical Neurosciences, Parkinson and Movement Disorders Unit, Fondazione IRCCS Istituto Neurologico Carlo Besta, Milan, Italy; ^2^Department of Neuroscience, Azienda Ospedaliero‐Universitaria S. Anna, Ferrara, Italy; ^3^Centro Parkinson e Parkinsonismi ASST Gaetano Pini‐CTO Milan Italy; ^4^Department of Neuroscience and Rehabilitation, University of Ferrara, Ferrara, Italy

**Background and aims:** Both motor and cognitive symptoms characterise spinocerebellar ataxia type 1 (SCA1), but sex‐specific differences in disease presentation and progression remain poorly understood. This study investigates the role of sex on motor and cognitive outcomes in SCA1 patients.

**Methods:** We conducted a monocentric, longitudinal observational cohort study at the University Hospital of Ferrara between 2021‐2024. Consecutively genetically confirmed SCA1 patients were evaluated at baseline and after 24±3 months. Assessments included comprehensive neuropsychological testing and auditory event‐related potentials (aERPs). Motor function was assessed using the Scale for Assessment and Rating of Ataxia (SARA).

**Results:** Sixteen SCA1 patients (9 males, seven females) were enrolled at baseline, with 10 patients (5 males, five females) completing follow‐up at 24±3 months. At baseline, while most cognitive functions were preserved in both sexes, male patients showed significantly worse performance in emotion attribution tasks than females (42.8±8.5 vs 53.1±5.7, p=0.029). At follow‐up, males demonstrated more pronounced deficits in verbal fluency, visual memory recall, and emotion attribution, while females maintained normal ranges across all tests. Although both sexes showed slightly worsening cognitive performance over time, the differences were not statistically significant. Motor impairment was more severe in males at follow‐up, though not significantly (SARA: 18.8±6.8 vs 14.0±6.5, p=ns). Analysis of aERPs revealed no differences between sexes at follow‐up.

**Conclusion:** These findings highlight both the importance of considering sex‐specific approaches in the clinical management of SCA1 patients and the higher values of neuropsychological assessment compared to neurophysiological approach to reach these slight changes over time.

**Disclosure:** no

## EPO‐254

### Impact of deep brain stimulation on impulsive‐compulsive behaviors in Parkinson's disease patients

#### L. Carretta^1^; F. Pinto ^2^; V. Ohannesian^3^; P. Teixeira^1^; L. Faria^1^; N. Oliveira^1^; R. Cipriano^1^; L. Almeida^4^; B. Ishizuka^3^; R. Silva^5^; R. Santos^6^; Y. Silva^7^; M. Leite^8^; C. Moura^9^; B. Pessoa^10^; P. Azevedo^11^

##### ^1^Department of Medicine, Escola Superior de Ciências da Santa Casa de Misericórdia de Vitória (EMESCAM), Vitória, Brazil; ^2^Department of Medicine, Estácio de Sá University, Rio de Janeiro, Brazil; ^3^Department of Medicine, Albert Einstein Israeli Faculty of Health Sciences (FICSAE), São Paulo, Brazil; ^4^Department of Medicine, Catholic University of Brasília, Brasília, Brazil; ^5^Department of Medicine, Federal University of Paraíba, João Pessoa, Brazil; ^6^Medical Education Institute Vista Carioca, Rio de Janeiro, Brazil; ^7^Healthcare Institution of South Iceland, Selfoss, Iceland; ^8^Department of Medicine, Santa Marcelina College, São Paulo, Brazil; ^9^Department of Neurology, Federal Fluminense University, Niterói, Brazil, ^10^Department of Neurosurgery, Federal Fluminense University, Niterói, Brazil, ^11^Department of Neurology, Santa Casa de Misericórdia de Vitória, Vitória, Brazil

**Background and aims:** Parkinson's Disease (PD) is a neurodegenerative disorder with motor and non‐motor complications, including impulse‐control disorders (ICDs), mainly due to dopaminergic medications used to treat the disease. While medication is the primary treatment, its effectiveness in managing impulsivity may be limited. Deep Brain Stimulation (DBS) alleviates motor symptoms, but its effect on ICDs is unclear. We aim to compare ICD between patients with PD treated with DBS as an adjunct to medication therapy versus medication alone.

**Methods:** We conducted a systematic review and meta‐analysis, searching PubMed, Embase, Cochrane Library, Web of Science, and Scopus. The inclusion criteria were patients with PD treated with DBS as an adjunct to medication therapy or alone. The primary outcomes analyzed were the Barratt Impulsiveness Scale (BIS) and decision‐making performance, assessed by the Iowa Gambling Task (IGT). Data on ICD severity, frequency, types, and adverse events were analyzed.

**Results:** For impulsiveness, measured by BIS, no significant difference was found between groups (MD = 0.47, 95% CI: ‐7.82 to 8.77; p = 0.91, I^2^ = 88%). Decision‐making performance, assessed by the Iowa Gambling Task (IGT), was better with DBS OFF than DBS ON (MD = ‐8.81, 95% CI: ‐16.45 to ‐1.18; p = 0.02, I^2^ = 23%), indicating potential adverse effects of DBS on impulsivity.

**Figure:**
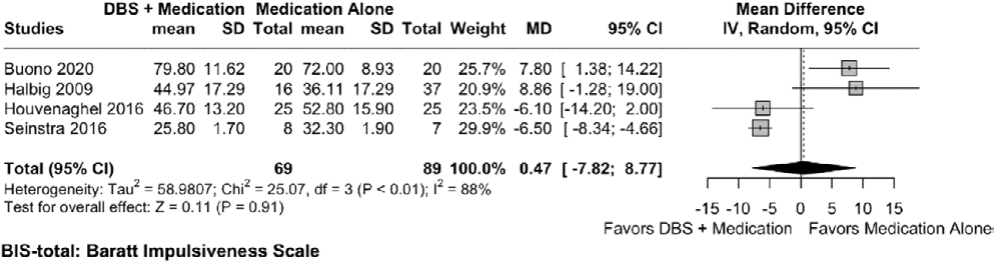
**FIGURE 1** Forest plot of Barratt Impulsiveness Scale (BIS)

**Figure:**
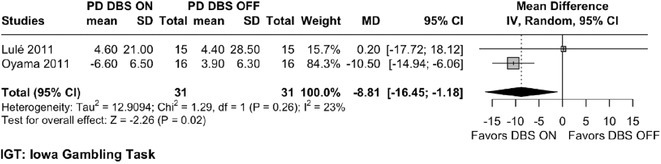
**FIGURE 2** Forest plot of Iowa Gambling Task (IGT)

**Conclusion:** Your findings suggest that managing ICDs with DBS in PD patients is promising. Findings may guide clinical decision‐making regarding the use of DBS in this population and inform future research directions.

**Disclosure:** Nothing to disclose.

## EPO‐255

### Actigraphic sleep monitoring in patients with Parkinson's disease and Deep Brain Stimulation

#### F. Daddoveri ^1^; E. Del Prete^2^; M. Maestri^1^; E. Bonanni^1^; U. Faraguna^3^; G. Siciliano^1^; R. Ceravolo^1^

##### ^1^Department of Clinical and Experimental Medicine, Neurology Unit, University of Pisa, Pisa, Italy; ^2^Department of Neuroscience, Neurology Unit, Azienda Ospedaliero Universitaria Pisana (AOUP), Pisa, Italy; ^3^Department of Translational Research and of New Surgical and Medical Technologies, University of Pisa, Pisa, Italy

**Background and aims:** Deep Brain Stimulation (DBS) represents an effective therapeutic strategy to improve both motor and non‐motor symptoms of Parkinson's disease (PD), such as sleep‐related issues. Actigraphy, based on the detection of triaxial accelerometric impulses, could become a useful innovation to monitor sleep even in these patients, allowing a continuous monitoring of patients’ conditions with better compliance and economic advantages.

**Methods:** We investigated the reliability of Actigraphy in the definition of sleep‐wake patterns, through a comparison with the gold‐standard Polysomnographic method, both in terms of epoch‐by‐epoch sleep recording and the definition of sleep‐related metrics such as Total Time of Sleep (TST), Wakefulness after Sleep Onset (WASO) and Sleep Efficiency (SE) in PD patients with Subthalamic DBS. Sleep recordings were collected using Axivity AX3 wrist Actigraphy and Polysomnography on 10 patients.

**Results:** An individually variable predictive capacity of Actigraphy in defining sleep‐wake patterns was present in our study, in agreement with literature. We estimated an average sensitivity of 74% in detecting sleep status, with an accuracy of 55%. Actigraphy globally underestimates TST values, but differences with Polysomnography are not statistically significant, given the wide variability detected between different patients’ data. Greater agreement was reported between the SE and WASO values.

**Figure:**
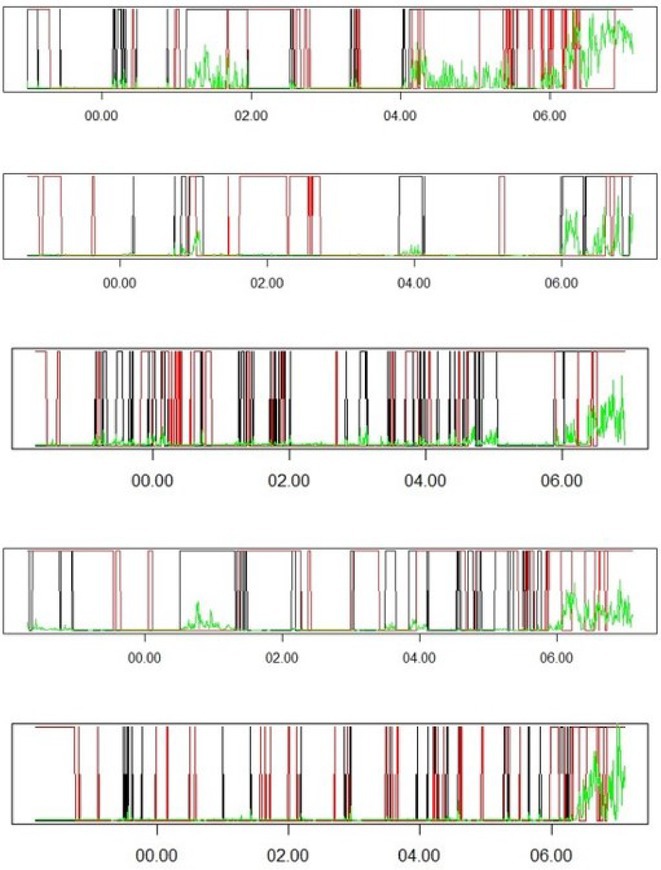
**FIGURE 1** Epoch by Epoch graphics of 5 patients, monitored across an entire night. In black, the Actigraphic binarized sleep‐wake trace; in red, the Polysomnographic trace; in green the trace of movement intensity detected by the Actigraphic device.

**Figure:**
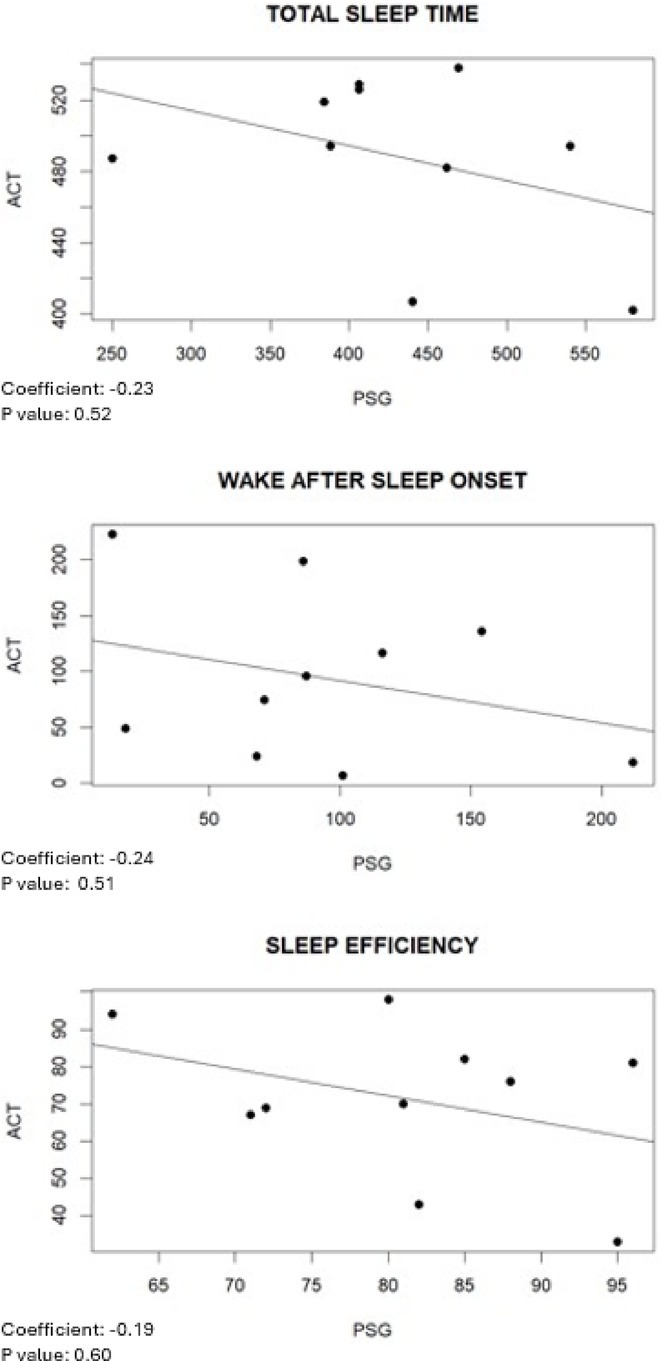
**FIGURE 2** Spearman correlations obtained by comparing Polysomnographic (PSG) and Actigraphic (ACT) sleep records. Each point of the graph represents a patient and his relative values: TST (minutes), WASO (minutes), SE (percentage).

**Figure:**
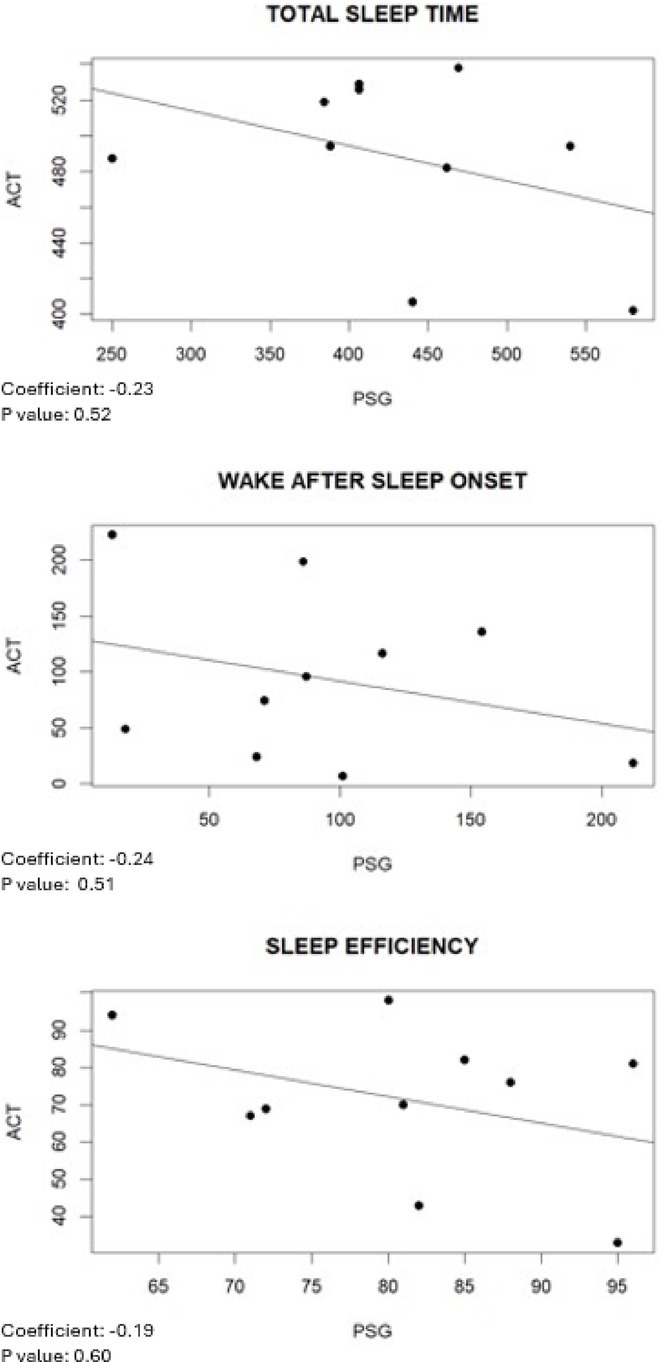
**FIGURE 3** Bland‐Altman plots comparing sleep measures of patients reveal an unsignificant tendency of Actigraphy to TST underestimation (mean difference = 55.3 minutes), WASO underestimation (1.9 minutes) and SE overestimation (10%) vs Polysomnography.

**Conclusion:** The study shows the potential applicability of Actigraphy in the assessment of sleep for PD patients undergoing DBS, with several clinical implications. However, we also highlight the inter‐patient variability in the reliability of the sleep‐related data obtained with this instrument in this population.

**Disclosure:** Nothing to disclose.

## EPO‐256

### Gender‐differences in efficacy and tolerability of opicapone in add on of levodopa. A real‐world observational study

#### G. Donzuso ^1^; G. Fazzina^1^; A. Salerno^1^; C. Terravecchia^1^; F. Contrafatto^1^; G. Mostile^1^; D. Contrafatto^2^; C. Cicero^1^; A. Nicoletti^1^

##### ^1^Department of Medical, Surgical Sciences and Advanced Technologies “GF Ingrassia”, University of Catania, Catania, Italy; ^2^AOU Policlinico G.Rodolico‐San Marco, Catania, Italy

**Background and aims:** Opicapone is a peripheral catechol‐O‐methyltransferase inhibitor, approved in add on to levodopa (LD) in Parkinson's disease (PD) patients with motor fluctuations. Aim of the study is to evaluate gender‐differences in efficacy and tolerability of opicapone in add on to LD treatment.

**Methods:** PD patients with motor fluctuations who started opicapone and who were followed up for at least 6 months were enrolled.

**Results:** Seventy‐seven PD patients (51 men; 66.2%) with a mean age at onset of 57.3±9.4 years and a disease duration of 11.0±4.2 years were enrolled. Baseline characteristics were not significantly different between sexes. At follow‐up a significant reduction of the total daily OFF time was observed. Overall 41.6% reported some adverse events (AEs) and incidence of AEs was significantly higher among women (65.4% versus 29.4%; p‐value 0.002). At multivariate analysis, adjusting by LEDD, female sex was significantly associated with the presence of AEs (OR 4.42; p‐value 0.004); 27.3% patients discontinued opicapone due to AEs and women had significantly higher odds of discontinuation (OR 3.00; p‐value 0.04).

**Figure:**
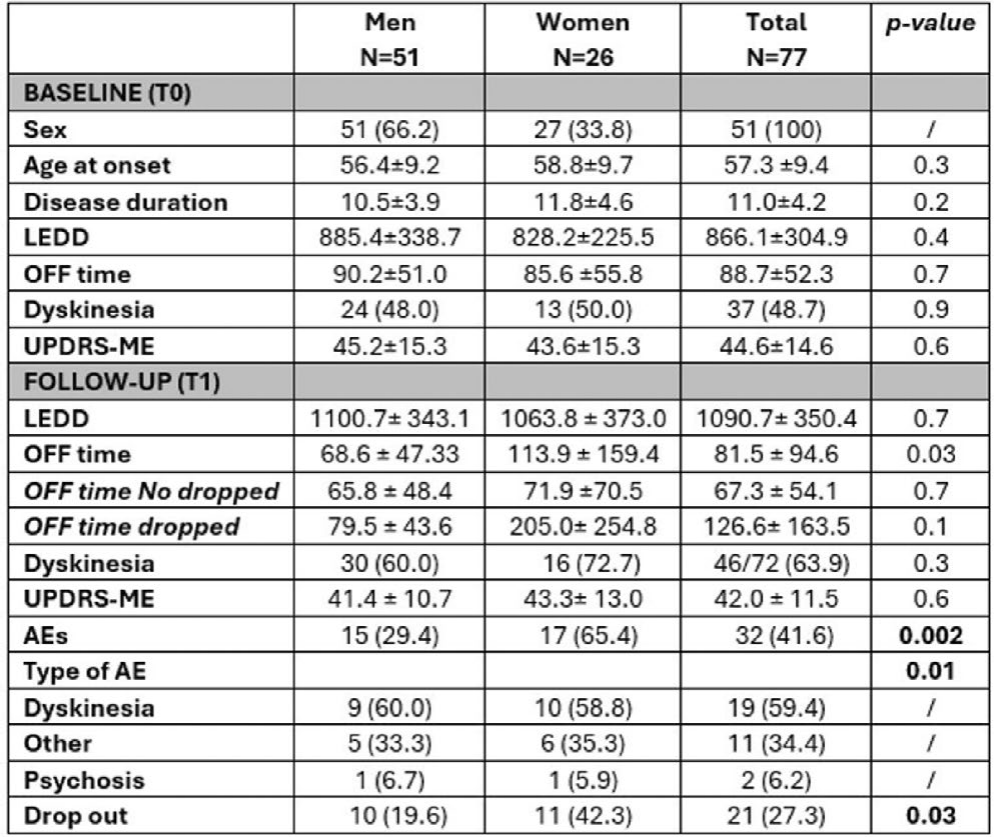
**TABLE 1** Gender‐differences in baseline and follow‐up clinical features in our PD sample.

**Conclusion:** Opicapone is highly effective for the treatment of motor fluctuations. Women experienced significantly higher AEs resulting in a higher frequency of drug discontinuation. The higher frequency of AEs, including dyskinesias, may be explained by higher levodopa bioavailability among women. To avoid an early discontinuation due to the presence of AEs in women with motor fluctuations, LD dosage should be reduced before the introduction of opicapone. Our study provides novel insights regarding gender‐differences in PD treatment, suggesting a personalized management for women with PD.

**Disclosure:** Nothing to disclose.

## EPO‐257

### Importance of moderate to vigorous physical activity for bone health in Parkinson's disease

#### M. Delgado‐Alvarado ^1^; C. de Dios^2^; M. Misiego‐Peral^3^; J. Sánchez‐de la Torre^3^; J. Riancho^1^; S. Setién^3^; Y. Jiménez‐López^3^; D. Gallo‐Valentín^3^; J. Infante^4^; E. Aurrecoechea^5^; R. López‐Maza^3^; M. Gómez‐España^5^; L. Riancho‐Zarrabeitia^5^

##### ^1^Neurology Department, Hospital Sierrallana, Torrelavega, Spain. IDIVAL, Santander Spain. CIBERNED, Madrid, Spain; ^2^Faculty of Medicine, University of Cantabria, Santander, Spain; ^3^Neurology Department, Hospital Sierrallana, Torrelavega, Spain; ^4^Neurology Department, University Hospital Marqués de Valdecilla‐IDIVAL, Santander, Spain. CIBERNED, Madrid, Spain; ^5^Rheumatology Department, Sierrallana Hospital, Torrelavega, Spain. IDIVAL, Santander, Spain

**Background and aims:** Parkinson's disease (PD) is associated with significant alterations in bone metabolism, yet the relationship between different intensities of physical activity and bone health in PD remains unexplored. This study aimed to assess bone health and its association with physical activity in PD patients.

**Methods:** PD patients underwent bone densitometry and physical activity was measured using a waist‐worn accelerometer (ActiGraph wGT3X‐BT) for seven days. Obtained data included total steps, time spent in light and moderate to vigorous physical activity (MVPA), and sedentary time. Demographic, clinical, and disease‐related data were collected.

**Results:** Among the 40 patients studied (mean age 70.5 ± 7.5 years; disease duration 6.5 ± 5 years), seven (17.5%) were osteoporotic. Patients with and without osteoporosis showed no differences in age (74.5 ± 8.6 vs. 69.7 ± 7.1; p=0.129), sex (males 57.1% vs. 63.6%; p=0.747), disease duration (7.5 ± 3.8 vs. 6.3 ± 5.2; p=0.194), or disease severity (UPDRS III: 30.5 ± 13.4 vs. 28.8 ± 14.7; p=0.630). Regression analysis, adjusting for age, sex, and BMI, revealed that total physical activity was positively associated with total hip bone mineral density (BMD) (β=0.310; p=0.026). Only MVPA was significantly related to total hip BMD (β=0.376; p=0.009), whereas light activity was not (β=0.109; p=0.457).

**Conclusion:** MVPA is associated with higher bone mineral density in PD patients. Encouraging this type of activity is recommended to support better bone health in this population.

**Disclosure:** Nothing to disclose.

## EPO‐258

### Subthalamotomy induced dyskinesia in Parkinson's disease: A sign of positive response rather than a problem

#### M. Ruiz Yanzi ^1^; M. Matarazzo^1^; I. Llera López^2^; E. Natera Villalba^1^; J. Máñez Miró^1^; R. Rodríguez Rojas^1^; J. Pineda Pardo^1^; M. del Álamo^1^; R. Martínez Fernández^1^; J. Obeso^1^

##### ^1^HM CINAC (Centro Integral de Neurociencias Abarca Campal), Hospital Universitario HM Puerta del Sur, HM Hospitales, Madrid, Spain; ^2^Hospital Universitario Rey Juan Carlos, Móstoles, Madrid, Spain

**Background and aims:** Unilateral magnetic resonance‐guided focused ultrasound (MRgFUS) subthalamotomy is a minimally invasive effective treatment for Parkinson's disease (PD) patients with asymmetrical motor signs. Dyskinesias may develop in the treated hemibody. We aimed to assess the effect of MRgFUS subthalamotomy in PD patients with dyskinesias post‐procedure in terms of patient‐reported changes, motor improvement and quality of life (QoL).

**Methods:** Thirty‐five PD patients underwent MRgFUS subthalamotomy in a randomized controlled trial. Retrospectively, they were grouped into those with (PD‐Dysk, n=13) and without dyskinesias (PD‐NoDysk, n=22) post‐procedure. The primary outcome was the between‐group difference in Patient's Global Impression of Change (PGI‐C) at four months. Secondary outcomes included long‐term PGI‐C, MDS‐UPDRS III scores, levodopa equivalent daily dose (LEDD) reduction, and QoL (PDQ‐39) at 4,12 and 24‐36 months. Mann‐Whitney U tests with Bonferroni correction were used.

**Results:** At four months, the PD‐dysk group reported greater improvement on PGI‐C (median 2.0 vs. 2.5, p=0.019), with a trend persisting at 24‐36 months (p=0.061). Motor improvement measured by MDS‐UPDRS III was comparable between groups except for greater reduction in treated‐side off‐medication score in the PD‐dysk group at 4 and 12 months (median ‐14 vs. ‐9.5; ‐14 vs. ‐9). LEDD reduction was greater in the PD‐dysk group at four months (‐175 vs 0, p=0.015) but not sustained. QoL scores showed no significant differences.

**Figure:**
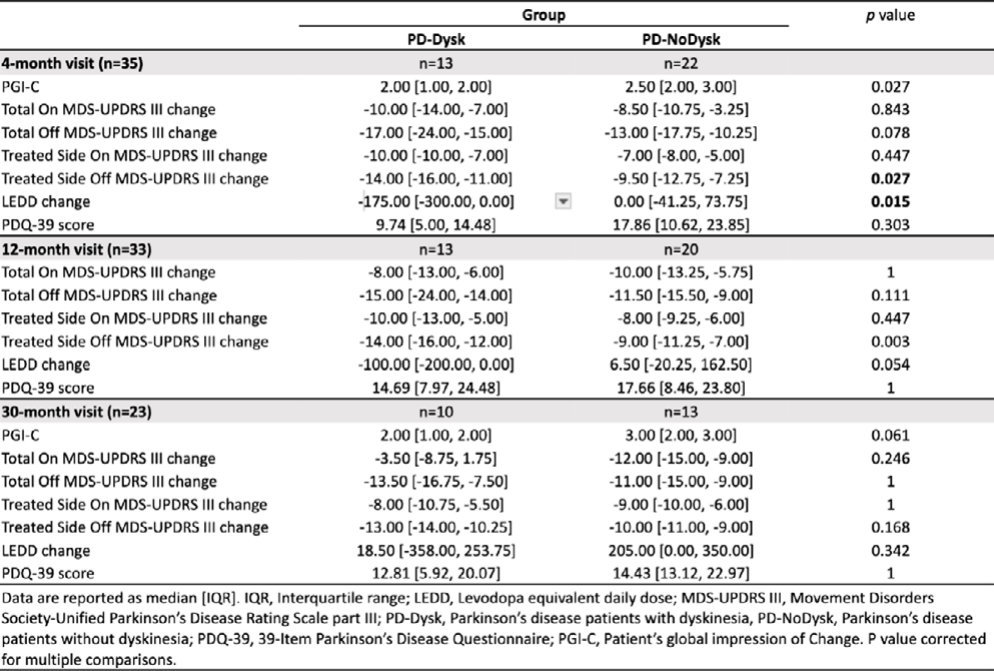
**TABLE 1** Analysis of primary and secondary outcomes.

**Conclusion:** PD patients with MRgFUS subthalamotomy‐related dyskinesias had a more favorable impression of health improvement than those without dyskinesias. Dyskinesias did not significantly impact QoL in either direction.

**Disclosure:** Nothing to disclosure.

## EPO‐259

### Non‐motor symptoms in GBA1‐Parkinson's disease: Analysis from the Parkinson's progression markers initiative

#### D. Ramos^1^; M. Fonseca ^2^; V. Di Foggia^1^; J. Holenz^1^; D. Simon^3^

##### ^1^Bial ‐ Portela & Cª, S.A.; Coronado, Portugal; ^2^Bial – R&D Investments, S.A., Coronado, Portugal; ^3^Department of Neurology, Beth Israel Deaconess Medical Center and Harvard Medical School; Boston, MA

**Background and aims:** Parkinson's Disease (PD) progression varies, particularly with non‐motor symptoms (NMS), which significantly affect quality of life. GBA‐PD is linked to GBA1‐gene mutations, encoding the beta‐glucocerebrosidase enzyme. On average, GBA‐PD shows earlier onset and a faster progression compared to idiopathic PD (iPD). Using data from the Parkinson's Progression Markers Initiative (PPMI), we compared GBA‐PD and iPD progression based on NMS severity.

**Methods:** PPMI, a multicenter, longitudinal cohort study was used (data‐cut: Dec‐11th, 2024). Non‐motor scales with continuous variables (cognitive, behaviour and sleep‐assessments) were included if the sample size at the longest follow‐up (6 years) was >= 40. Mixed‐Effects‐Model for Repeated‐Measures (MMRM), corrected for baseline value and disease duration, was employed.

**Results:** In total, 95 GBA‐PD and 495 iPD patients were included. At baseline, GBA vs iPD patients were 62.2 ± 10.55 (mean ± standard deviation) vs 64.1 ± 9.70 years‐old and had PD for 3.2 ±1.97 vs 2.2 ±1.18 years, respectively (Table 1). Patients with GBA‐PD consistently show worse severity and significantly faster progression in 54.5% of the scales vs iPD. Longitudinally, 6 non‐motor scales for GBA‐PD and 4 for iPD (out of 11) show Minimal Clinical Important Difference (MCID) from baseline, with GBA‐PD reaching MCID 1‐2 years earlier than iPD. Among group differences (Figure 1), four scales reached MCID: MDS‐UPDRS‐Part‐1A, Symbol‐Digit‐Modalities‐Score, Hopkins‐Verbal‐Learning‐Test, and State‐Trait‐Anxiety‐Index, with scores worse for GBA‐PD vs iPD.

**Figure:**
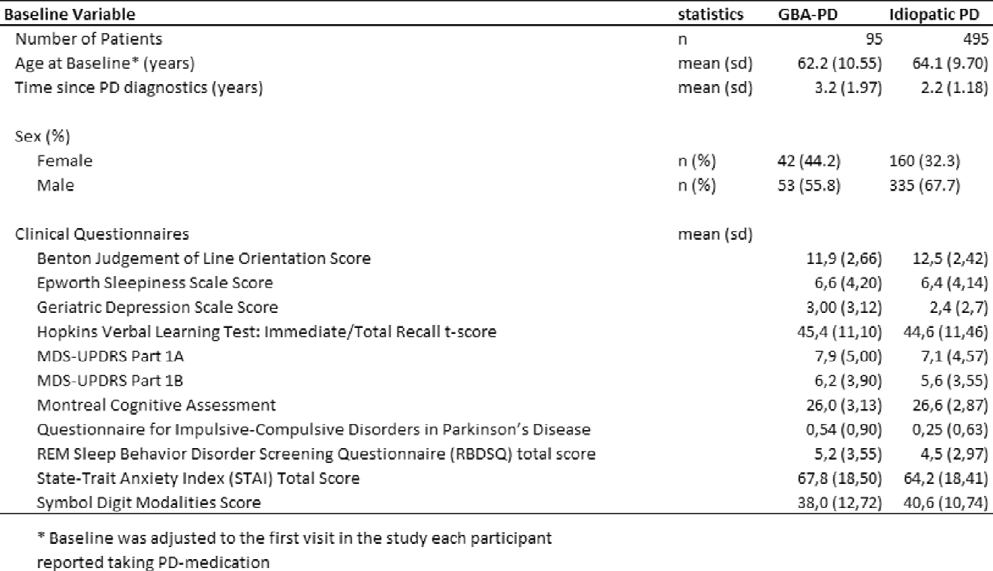
**TABLE 1** Baseline Characteristics.

**Figure:**
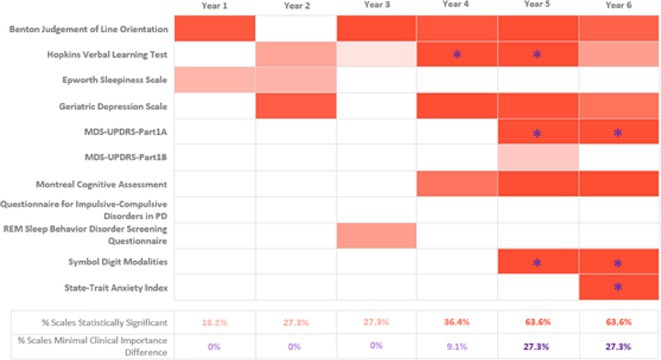
**FIGURE 1** Heatmap of yearly visits with statistically significant differences between GBA‐PD and iPD. Darker Red gradients indicate lower p‐values <= 0.05; Purple asterisks denote Minimal Clinically Important Difference (MCID) between groups.

**Conclusion:** This analysis suggests that, over 6‐years, NMS present with more severity and faster progression in GBA‐PD compared to iPD‐patients (adjusted for covariates baseline value and disease duration).

**Disclosure:** Daniel S. Ramos, Miguel M. Fonseca, and Valentina Di Foggia are employees of Bial.

## EPO‐260

### Mobile health technology postural and turning assessment in progressive supranuclear palsy and Parkinson's disease

#### T. Comunale ^1^; A. Pilotto^1^; A. Rizzardi^1^; C. Zatti^1^; M. Rizzetti^2^; A. Lombardi^1^; C. Hansen^3^; R. Romijnders^3^; B. Borroni^1^; W. Maetzler^3^; A. Padovani^1^

##### ^1^Department of Clinical and Experimental Sciences, Neurology Unit, University of Brescia, Italy; ^2^Parkinson's disease Rehabilitation unit, FERB Onlus Trescore Balneario, Bergamo, Italy; ^3^Department of Neurology, Christian‐Albrechts‐University of Kiel, Kiel, Germany

**Background and aims:** Progressive supranuclear palsy (PSP) is a neurodegenerative disease characterized by earlier postural instability compared to Parkinson's disease (PD). The aim of this study is to evaluate differences in postural and turning performances between patients with PSP and PD using Mobile Health Technology (MHT).

**Methods:** 250 subjects entered the study: 27 with PSP, 44 with PD who have experienced at least 1 fall in the last year, 63 with PD who have not experienced any fall in the last year and 116 healthy subjects. Static balance was evaluated with instrumented (lower back accelerometer, Rehagait®, Hasomed, Germany) 30‐s trials in side by side, semitandem and tandem positions. Turning was evaluated with instrumented Timed Up and Go test. Data were analysed to determine what parameters discriminate PSP from PD and HC and to detect correlations of technological measures with clinical assessment.

**Results:** Compared to HC and PD, PSP and PD fallers showed similar static parameters. PSP exhibited lower volume of perturbation compared to PD with falls. Turning parameters significantly differed between HC, PD without falls and PD fallers as well as PSP, with no differences between the latest groups.

**Conclusion:** PSP patients exhibit similar postural pattern compared to PD with falls but lower perturbation volume. Different pathophysiology and compensations mechanism are probably related to postural instability in these patients. Turning parameters instead are more sensitive in the detection of fallers and further studies are needed to determine if they could be used as markers of risk of falls or early markers of disease progression.

**Disclosure:** Nothing to disclose.

## EPO‐261

### Machine learning predicts risk of motor dysfunctions in Parkinson's Disease patients in a multicentric study

#### W. Endrizzi ^1^; M. Moroni^1^; F. Ragni^1^; S. Bovo^1^; C. Longo^2^; M. Chierici^1^; L. Gios^3^; D. Ottaviani^4^; R. Di Giacopo^4^; L. Avanzino^6^; R. Marchese^5^; F. Di Biasio^5^; M. Marenco^5^; A. Uccelli^5^; B. Giometto^2^; V. Osmani^1^; G. Jurman^1^; M. Malaguti^2^

##### ^1^Fondazione Bruno Kessler, Trento, Italy; ^2^Azienda Provinciale per i Servizi Sanitari (APSS) di Trento, Trento, Italy; ^3^TrentinoSalute4.0 – Competence Center for Digital Health of the Province of Trento, Trento, Italy; ^4^Department of Neurology, Santa Maria del Carmine Hospital, Azienda Provinciale per i Servizi Sanitari (APSS), Rovereto, Italy; ^5^IRCCS Ospedale Policlinico San Martino di Genova, Italy; ^6^Department of Experimental Medicine, Section of Human Physiology, University of Genoa

**Background and aims:** Dyskinesia, freezing, and motor fluctuations are conditions occurring during the course of Parkinson's Disease (PD) and significantly impacting the quality of life in patients. This study aims to identify patterns of clinical variables as predictors for each of the three outcomes—dyskinesia, freezing, and motor fluctuations—using a standardized Machine Learning (ML) strategy that ensures results reliability.

**Methods:** Demographic, motor, clinical, and pharmacological data of 265 PD patients followed at Movement Disorder Clinic in three Italian centers were retrospectively collected at four time points (baseline, after 12, 24, and 36 months) in the context of the project NeuroArtP3[1] (NET‐2018‐12366666). Four different classifiers were evaluated, namely Random Forest (RF), Extra Trees Classifier (ETC), XGBoost (XGB), and Logistic Regression (LR) on each outcome using a Randomized Nested Grid Search Cross Validation (RNGCV) strategy. Models performance was measured using Area Under the Receiver Operating Characteristic Curve (AUC), Matthews Correlation Coefficient (MCC) and F1‐score. To understand how clinical features influence each specific outcome, a SHAP explainability analysis was performed on the best‐performing classifiers.

**Results:** Best models’ performances (AUC) for 3 outcomes ranged from 0.81 to 0.86 and are shown in Figure 1. Significant predictors for the three motor dysfunctions are shown in Figure 2.

**Figure:**
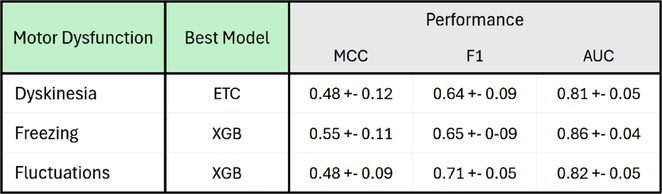
**FIGURE 1** Classification performances of the best models for each outcome.

**Figure:**
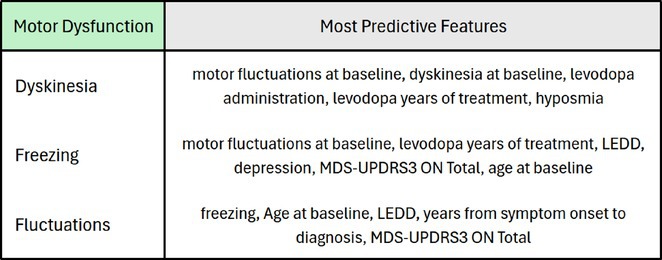
**FIGURE 2** Most important predictors for the three outcomes.

**Conclusion:** ML analysis showed a robust performance in predicting the risk of motor dysfunctions in PD patients. Comprehending the aspects that anticipate the risk of these outcomes could provide valuable insights for targeted clinical interventions.

**Disclosure:** Nothing to disclose.

## EPO‐262

### Limb‐kinetic apraxia of legs in Parkinson's disease: Prospective clinical investigation

#### Y. Balash ^1^; E. Mate^2^; R. Idries^3^; A. Eilam^4^; A. Korczyn^5^

##### ^1^Department of Neurology, Kaplan Medical Center, Rehovot, Israel; ^2^Department of Neurology, Kaplan Medical Center, Rehovot, Israel; ^3^Department of Neurology, Kaplan Medical Center, Rehovot, Israel; ^4^Department of Neurology, Kaplan Medical Center, Rehovot, Israel; ^5^Departments of Neurology and Physiology and Pharmacology, Faculty of Medicine, Tel‐Aviv University, Tel‐Aviv, Israel

**Background and aims:** The study of dynamic organization of motor acts is important for investigation of motor impairment, and a possible sign of a disorder of fronto‐parietal areas of the brain in Parkinson's disease (PD). We aimed to prospectively investigate whether limb‐kinetic apraxia in legs (LKA‐L) is a heretofore unrecognized manifestation of PD independent of bradykinesia and rigidity.

**Methods:** Patients with PD and healthy controls (HC) performed bipedal reciprocal coordination (BRC) and monopedal reciprocal coordination (MRC) tests as a foot modification of the Oseretzky exam (originally alternate antiphase clenching and unclenching of the fists of the right and left hands). While MRC allowed for alternating movements of one leg per unit of time, BRC required synchronous movements of both legs in antiphase. Leg movement rates and their quality were measured by video recording and compared statistically between the groups of PD and HC.

**Results:** The cohort consisted of 31 PD patients (mean age 69.3±7.1 years, 16 males) and 12 HC (mean age 69±6.2 years, 6 males). No differences between PD and HC groups were identified in MRC rate of performance, which were used as a measure of legs movement speed, although the quality of MRC movements was poorer in PD patients (p=0.022). BRC rate and its performance quality were significantly flawed in PD compared to controls (P = 0.002 and P = 0.003, respectively).

**Conclusion:** Testing for dynamic organization of LKA‐L revealed disorder in individuals with PD. LKA‐L analyses should be considered in the diagnosis of leg movements and gait disorders in PD.

**Disclosure:** Nothing to disclose.

## Movement Disorders 3

## EPO‐263

### Patient based retrospective reporting of motor and nonmotor symptoms in LRRK2‐related vs idiopathic Parkinson's disease

#### M. M. Roque ^1^; A. Valadas^1^; M. Coelho^2^; M. Rosa^3^; J. Ferreira^3^; L. Correia Guedes^1^

##### ^1^Neurology Service, Department of Neurosciences and Mental Health, ULS Santa Maria, Lisbon, Portugal; ^2^Egas Moniz Study Center, University of Lisbon, Faculty of Medicine, University of Lisbon, Lisbon, Portugal; ^3^Institute of Clinical Pharmacology, Faculty of Medicine, University of Lisbon, Lisbon, Portugal

**Background and aims:** The clinical phenotype of LRRK2‐PD overlaps with iPD, making routine clinical identification challenging without family history or in low mutation frequency populations. Our study aims to retrospectively compare motor and nonmotor symptoms in LRRK2‐ PD vs iPD.

**Methods:** We conducted a retrospective and cross‐sectional study comparing LRRK2‐PD with iPD in a single tertiary center. From a cohort of 654 Parkinson's disease patients who underwent genetic screening, 42 heterozygous carriers of LRRK2 mutations were identified. Of these, 24 were included and matched 1:2 with iPD patients (n=48) selected to match age at disease onset, disease duration, and gender. Participants completed structured questionnaires and underwent clinical evaluations using MDS‐UPDRS, NMSS, NMSQ, HADS, AES, MoCA, MMSE, and PDSS.

**Results:** Our retrospective questionnaire showed that LRRK2‐PD patients were less likely to report urinary urgency (p=0.014), REM sleep behavior disorder (p=0.004), pain (p=0.031), and falls (p=0.005). No significant differences between groups were identified for age at disease onset, disease duration and gender, in accordance with study design. Cross‐sectional evaluation found no significant differences between LRRK2‐PD and iPD groups in MDS‐UPDRS III, NMSS, NMSQ, HADS, AES, MoCA and MMSE total scores. Acknowledging the limitation of evaluating sub‐items in these scales, several showed significant differences between groups: LRRK2‐PD patients reported lower frequency/severity of nocturia (p=0.022), sexual dysfunction (p=0.014) and better overall sleep quality (p=0.02).

**Figure:**
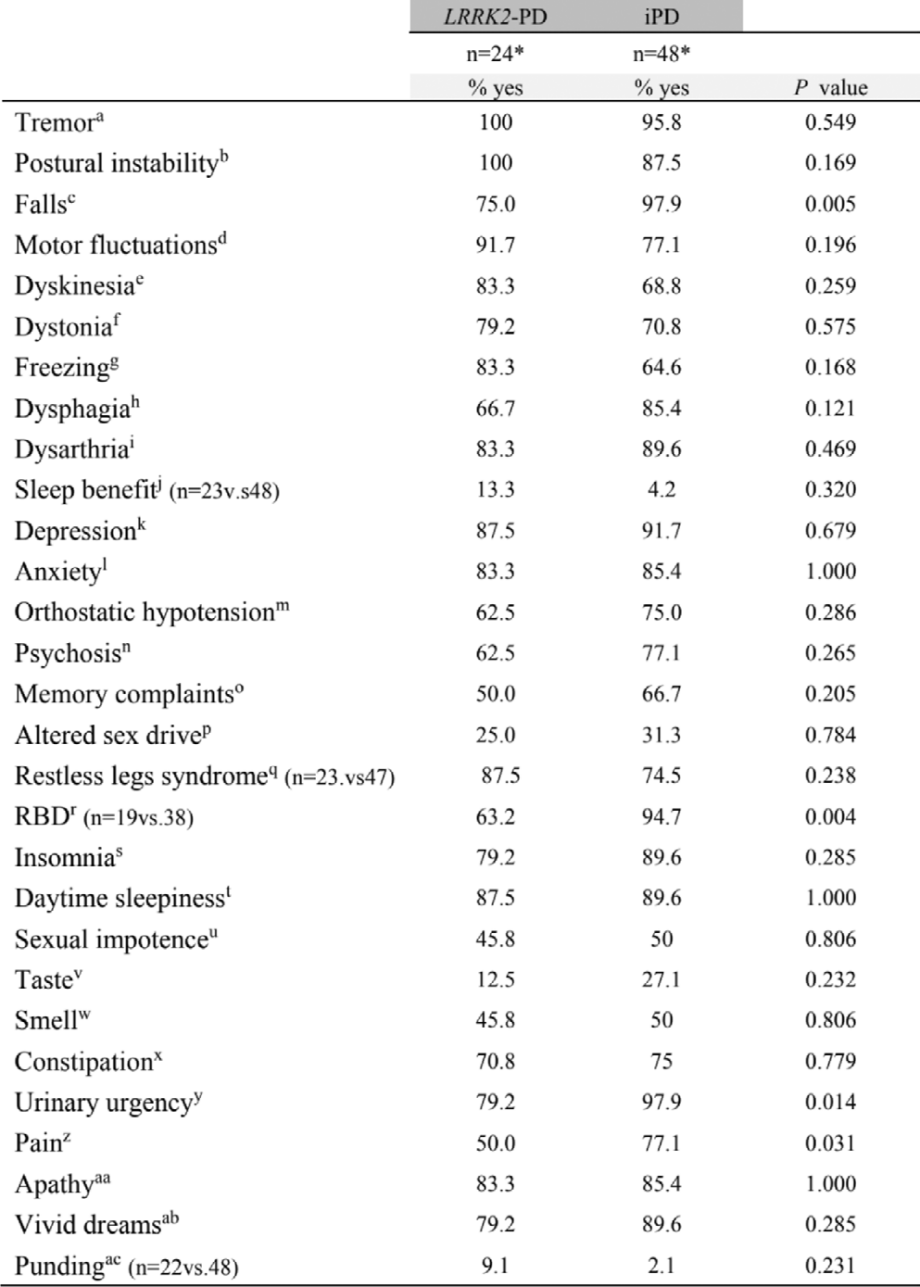
**TABLE 1** Reporting of ever having had motor and nonmotor symptoms.

**Figure:**
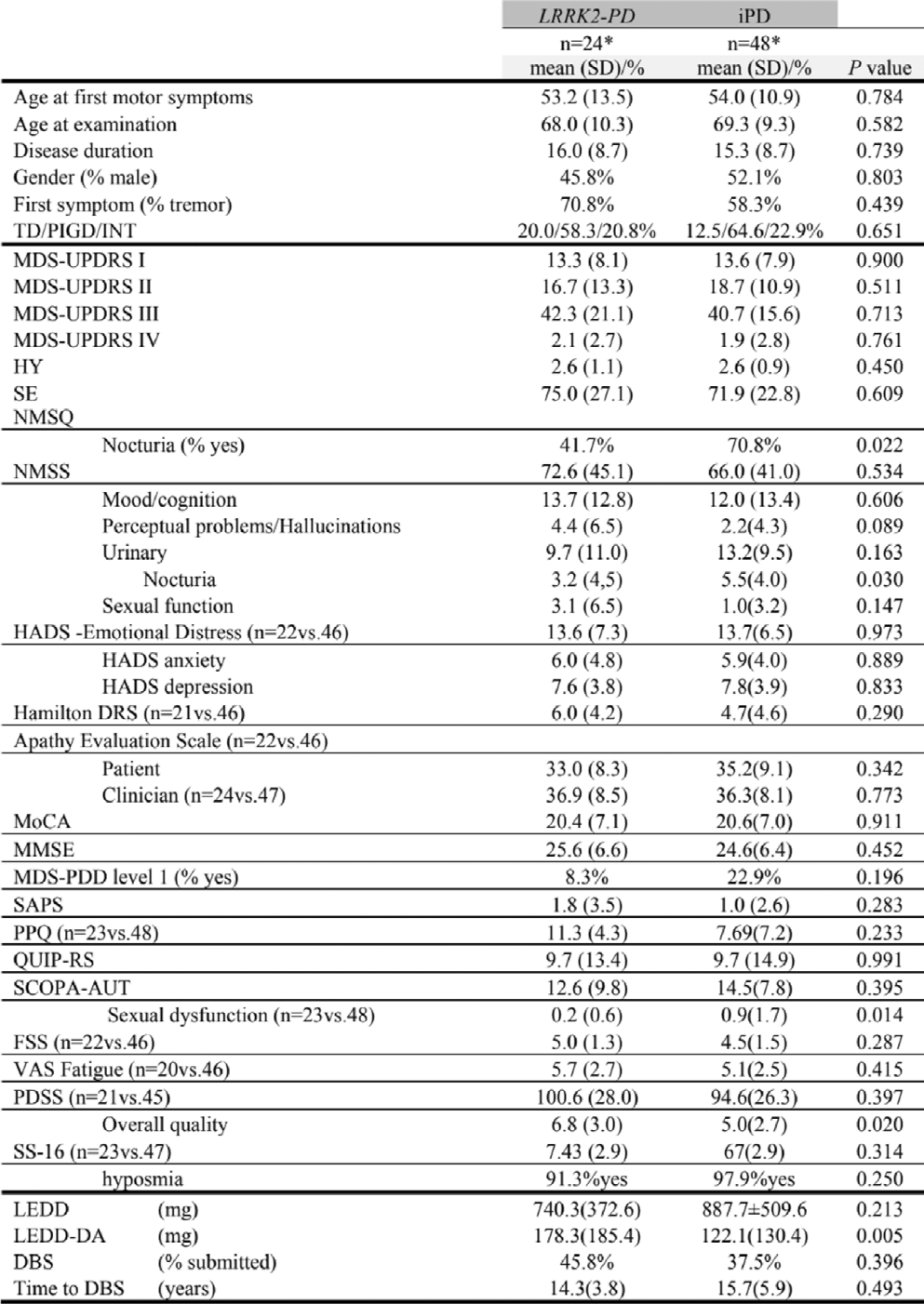
**TABLE 2** Demographic and clinical data comparing LRRK2‐PD patients vs iPD

**Conclusion:** Our retrospective questionnaire investigating if a motor and non‐motor symptom was ever present contributed to discriminate between LRRK2‐PD and iPD. Structured questionnaires like this one could contribute to inform clinical care and research in PD.

**Disclosure:** Nothing to disclose.

## EPO‐264

### 1H‐NMR metabolomic analysis identifies disrupted glutamate metabolism as serum signature in Parkinson's disease patients

#### A. Imarisio ^1^; C. Marino^2^; E. Napolitano^2^; I. Yahyavi^3^; M. Avenali^4^; G. Buongarzone^4^; C. Galandra^1^; M. Picascia^5^; M. Grimaldi^2^; C. Pacchetti^5^; F. Errico^6^; A. D'Ursi^2^; A. Usiello^3^; E. Valente^1^

##### ^1^Department of Molecular Medicine, University of Pavia, Pavia, Italy; Neurogenetics Research Centre, IRCCS Mondino Foundation,Pavia, Italy; ^2^Department of Pharmacy, University of Salerno, Fisciano, Salerno, Italy; ^3^Department of Environmental, Biological and Pharmaceutical Sciences and Technologies, Università degli Studi della Campania “Luigi Vanvitelli”, Caserta, Italy; CEINGE Biotecnologie Avanzate Franco Salvatore, Naples, Italy; ^4^Department of Brain and Behavioral Sciences, University of Pavia, Pavia, Italy; Parkinson's Disease and Movement Disorders Unit, IRCCS Mondino Foundation, Pavia, Italy; ^5^Parkinson's Disease and Movement Disorders Unit, IRCCS Mondino Foundation, Pavia, Italy; ^6^CEINGE Biotecnologie Avanzate Franco Salvatore, Naples, Italy; Department of Agricultural Sciences, University of Naples “Federico II”, Portici, Italy

**Background and aims:** Using a High‐Performance Liquid Chromatography targeted approach, we recently showed increased CSF and serum serine enantiomers levels as putative biomarker of Parkinson's disease (PD). Recent serum metabolomics evidence showed a dysregulation of multiple amino acids pathways in PD patients compared to healthy controls (HC). Here, we attempted to identify a metabolomic signature distinctive of PD through an untargeted approach.

**Methods:** We enrolled 69 idiopathic PD patients and 32 age‐matched HC. Untargeted metabolomics was carried out using Nuclear Magnetic Resonance (1H‐NMR) on serum samples. Partial least‐squares discriminant analysis (PLS‐DA) and pathway enrichment analysis were used to identify metabolites and biochemical pathways discriminating the two groups.

**Results:** Serum metabolomics identified two distinct clusters for PD patients and HC (Fig. 1a). Multivariate analyses revealed 11 metabolites independently associated with PD (i.e. with variable importance in projection score>1), including L‐glutamate, L‐proline, pyruvate and L‐serine (Fig. 1b). Univariate analyses showed (i) increased L‐glutamate and (ii) reduced L‐proline and 2‐oxoglutarate levels in PD compared to HC, all showing high predictive power for PD (AUC: 0.99, 0.89 and 0.94, respectively) (Fig. 2a‐b). Finally, pathway analysis identified 21 pathways overrepresented in PD at FDR<0.05, almost all involved in amino acids or energy metabolism. Among these, glycine‐serine pathway showed the best discriminating value (Fig. 3).

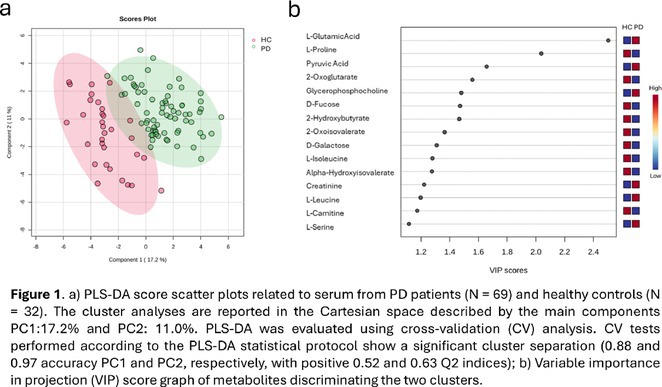

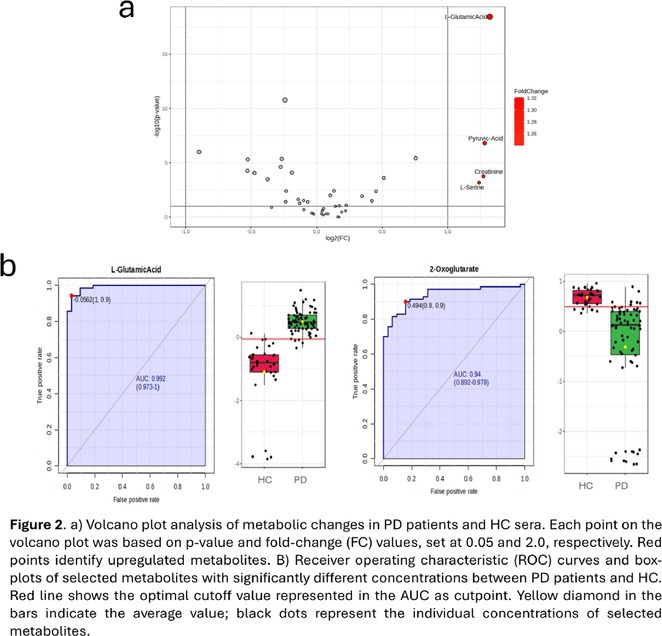

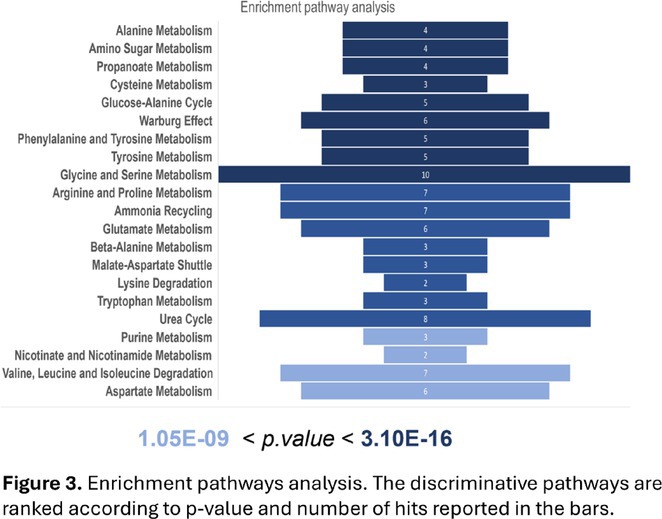

**Conclusion:** We identified serum glutamate levels and glycine‐serine metabolism dysregulation as distinctive signatures of PD. Our results support the hypothesis that dysregulated aminoacids and energy metabolism plays a key role in PD physiopathology and pave the way for future studies evaluating its diagnostic and prognostic value.

**Disclosure:** Nothing to disclose.

## EPO‐265

### Low vs. high‐frequency STN‐DBS in PD patients: results from a two‐center, randomized, double‐blind, crossover trial

#### C. Smeralda ^1^; D. De Monte^2^; A. Giannotta^1^; E. Belgrado^3^; A. Bernardini^2^; M. Valente^2^; C. Lettieri^2^; S. Rossi^1^

##### ^1^Siena Brain Investigation & Neuromodulation Lab (Si‐BIN lab), Unit of Neurology and Clinical Neurophysiology Department of Medicine, Surgery and Neuroscience, University of Siena, Siena, Italy; ^2^Clinical Neurology, Department of Medicine (DMED), University of Udine, Udine, Italy; ^3^Clinical Neurology, Department of Head‐Neck and Neuroscience, Azienda Sanitaria Universitaria Friuli Centrale (ASUFC), Udine, Italy

**Background and aims:** Subthalamic nucleus deep brain stimulation (STN‐DBS) effectively treats Parkinson's disease (PD). Whether low‐frequency stimulation (LFS) is more effective than high‐frequency stimulation (HFS) on gait and postural disturbances is not entirely elucidated yet.

**Methods:** We conducted a two‐center, randomized, double‐blind, crossover trial on sixteen STN‐DBS patients (7 females; age 69.7 ± 7.2). Of them, 7 were tremor predominant (TD), and 9 were postural instability/gait disorder (PIGD). Participants randomly received LFS (60 Hz) or HFS (130 Hz) during two separate visits, while in an OFF‐medication state. Stimulation amplitude was adjusted to maintain a stable total electrical energy delivered. The MDS‐UPDRS part III, the Berg Balance Scale (BBS), and the Time Up‐and‐Go (TUG) test wearing a wireless inertial sensor were performed both in OFF‐ and ON‐stimulation state.

**Results:** A significant time, frequency, and motor subtype interaction was found for MDS‐UPDRS total (F2,13=7.19, p=0.007), tremor (F2,13=10.5, p<0.001), gait score (F2,13=4.27, p=0.03), and BBS (F2,13=4.16, p=0.04). Post‐hoc tests for the tremor score indicated that in TD patients HFS ON state was significantly different from its OFF state (p<0.001) and LFS ON state (p=0.02), For the gait score and BBS in PIGD patients both LFS and HFS ON states were significantly different from their OFF states (p=0.02 and p=0.007, p=0.006 and p<0.001, respectively), but not from each other (p=0.77). A significant effect of time (F1,15=5.73, p<0.03), but not a significant time, frequency, and motor subtype interaction was found for TUG test duration.

**Conclusion:** LFS is equally effective as HFS in improving gait and postural stability in PIGD patients.

**Disclosure:** Nothing to disclose.

## EPO‐266

### Efficacy and safety of magnetic resonance‐guided focused ultrasound pallidothalamic tractotomy for Parkinson's Disease

#### C. Ferrer ^1^; A. Leruste^1^; A. Sanchez Fraga^1^; K. Gant^2^; S. Moola^3^; A. Maslov^3^; O. Borisenko^3^; A. Grinspan^2^

##### ^1^Insightec Europe GmbH, Munich, Germany; ^2^Insightec, Inc., Miami, USA; ^3^MTRC HEOR Ltd., Leeds, UK

**Background and aims:** Magnetic resonance‐guided focused ultrasound (MRgFUS) pallidothalamic tractotomy (PTT) is an incisionless therapy used in Parkinson's Disease (PD). A Phase III trial (NCT04728295) of staged‐bilateral PTT‐MRgFUS was completed and 12‐month follow up data are under FDA review. This systematic literature review (SLR) aims to review the published evidence to help contextualize those upcoming results.

**Methods:** An SLR was conducted in Medline and Medline In‐Process with no date restrictions (Table 1). Two reviewers independently screened titles and abstracts. Full‐text review and data extraction were completed, followed by a narrative synthesis of the findings.

**Figure:**
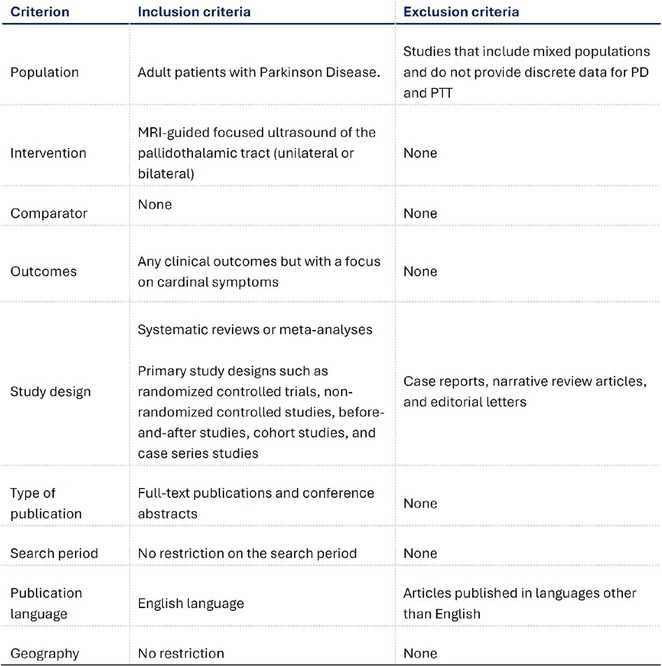
**TABLE 1** Inclusion and exclusion criteria.

**Results:** All reports were single‐centre studies conducted in Switzerland (5), Taiwan (2), and Japan (1) (Figure 1). Unilateral PTT‐MRgFUS (n=102) was an effective treatment reporting statistically significant reductions in tremor (84%), rigidity (70%), bradykinesia (73%), off‐medication dystonia (67%) and on‐medication dyskinesias (38%). Two staged‐bilateral studies (n=25) had a mean interval of 20±10 months between first and second PTT‐MRgFUS treatments. Total off‐medication UPDRS decreased by 52% (p<0.007). Mixed findings were reported for reductions in L‐Dopa intake. The most common adverse events included hypoesthesia, speech difficulties, hiccups, and reduced responsiveness to L‐Dopa. Recently, a modified approach was introduced by Chen et al, using dual‐target VIM‐PTT.

**Figure:**
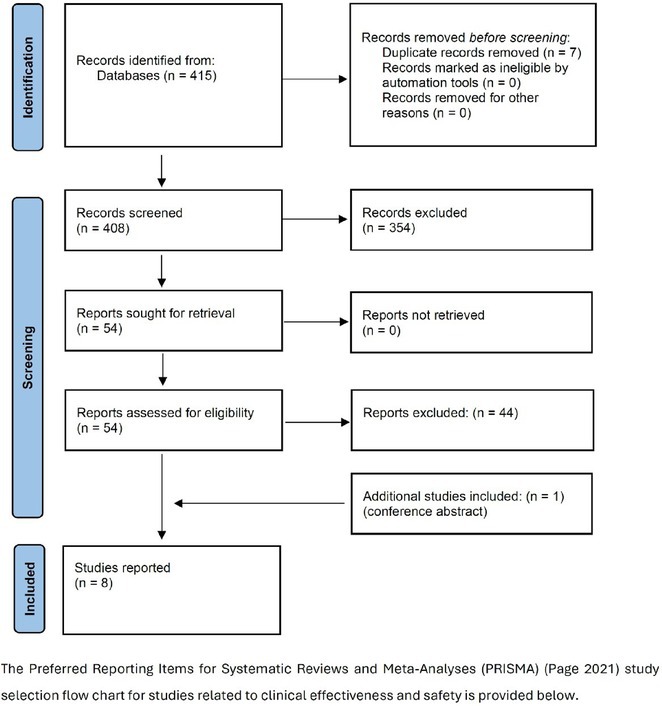
**FIGURE 1** PRISMA study selection flowchart.

**Conclusion:** This SLR provides preliminary evidence that PTT‐MRgFUS is effective in reducing motor symptoms and complications, with a generally favorable safety profile. Results from the upcoming Phase III study hold potential to expand bilateral treatment options to patients with idiopathic PD. It will be the first prospective global multicentre study investigating unilateral and staged‐bilateral PTT‐MRgFUS.

**Disclosure:** CF, AL, ASF, KG and AG are Insightec employees; SM, AM and OB are MTRC HEOR employees. Writing assistance was provided by Content Ed Net, Madrid, Spain.

## EPO‐267

### Light chain neurofilaments: A biomarker for differentiating Parkinson's Disease from Atypical Parkinsonism

#### J. Alves ^1^; D. Carneiro^1^; F. Moreira^2^; F. Matias^1^; P. Nunes Vicente^1^; C. Machado^1^; I. Santana^1^; A. Morgadinho^3^; I. Baldeiras^1^

##### ^1^Neurology Department, Coimbra University Hospital, Coimbra Local Health Unit, Coimbra, Portugal; ^2^Neurology Department, CUF Hospital of Coimbra, Coimbra, Portugal; ^3^Neurology Department, Hospital da Luz of Coimbra, Coimbra, Portugal

**Background and aims:** Differentiating Parkinson's disease (PD) from atypical parkinsonism (AP) is particularly challenging in the early stages. Emerging evidence suggests that neurofilament light chain (NfL) levels in CSF or serum may facilitate this distinction. This study evaluates the diagnostic utility of NfL in CSF and serum for distinguishing between PD and AP.

**Methods:** A retrospective analysis was conducted on clinical records of patients with a probable diagnosis of Parkinson's disease (PD), Multiple System Atrophy (MSA), Progressive Supranuclear Palsy (PSP), and Corticobasal Degeneration (CBD), all of whom underwent NfL testing in CSF and/or serum. NfL levels between PD and AP were compared using bivariate and multivariate analysis.

**Results:** We included 49 patients with parkinsonism: 35 PD and 14 AP (5 MSA, 7 PSP, 2 CBD). All had NfL testing in CSF, and 44 also in serum. No significant clinical‐demographic differences, except disease duration (PD: 8.0 years [IQR 3.0‐12.0]; AP: 2.5 years [IQR 2.0‐4.3]; p=0.015) and Hoehn and Yahr stage (PD: 2.0 [IQR 2.0‐3.0]; AP: 3.0 [IQR 3.0‐4.0]; p=0.002). Bivariate analysis showed higher NfL levels in AP patients compared to PD patients in CSF (p<0.001) and serum (p=0.012). No significant differences in NfL levels were found among AP subtypes. Multivariate analysis, adjusted to disease duration, Hoehn and Yahr score, and CSF/Serum NfL value, found a significant difference in CSF NfL levels (p=0.036), with high diagnostic accuracy (AUC 0.906).

**Figure:**
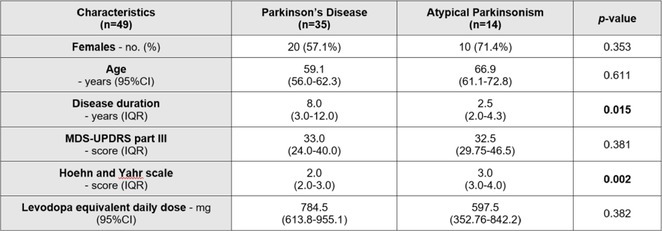
**FIGURE 1** Clinicodemographic characteristics and levels of study participants, stratified by final diagnosis

**Figure:**
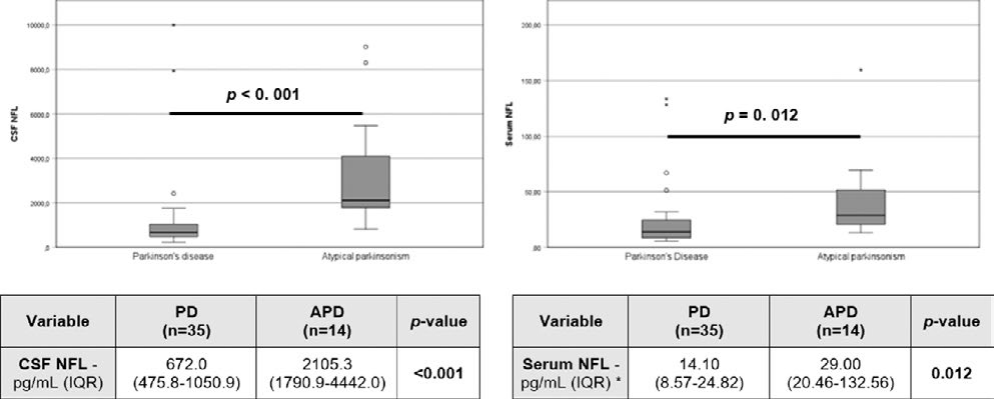
**FIGURE 2** Comparison of CSF and serum NfL levels between patients with PD and APD

**Figure:**
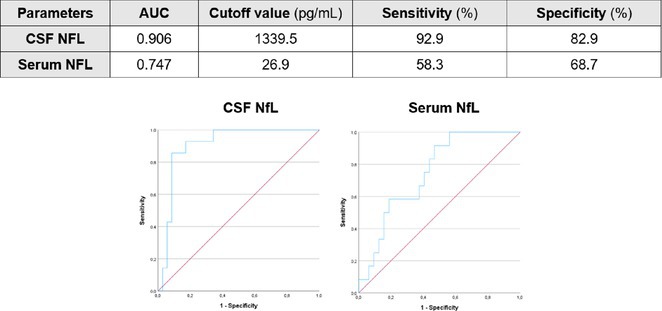
**FIGURE 3** Receiver operating characteristic (ROC) analysis of CSF and Serum NFL in the diagnosis of PD and APD

**Conclusion:** Elevated NfL levels in AP patients compared to PD support NfL as a promising biomarker for differentiation, aiding in therapeutic decisions and prognostic evaluation.

**Disclosure:** Nothing to disclose.

## EPO‐268

### Stepwise dual‐target MR‐guided Focused Ultrasound (dtMRgFUS) for Parkinson's disease: A 2‐year follow‐up of 3 cases

#### J. Chen ^1^; M. Lu^2^; C. Chun‐Ming Chen^3^; C. Tsai^4^

##### ^1^Neuroscience and Brain Disease Center, China Medical University, Taichung, Taiwan; ^2^Neuroscience Laboratory, Department of Neurology, China Medical University Hospital, Taichung, Taiwan; ^3^Department of Radiology, China Medical University Hospital, Taichung, Taiwan; ^4^School of Medicine, College of Medicine, China Medical University, Taichung, Taiwan

**Background and aims:** Magnetic resonance‐guided focused ultrasound (MRgFUS) is a new treatment for medication‐refractory Parkinson's disease (PD). Targets like the ventral intermediate nucleus (VIM) and pallidothalamic tract (PTT) have shown varying effectiveness. While single‐lesion treatments alleviate specific PD symptoms, our previous research studied the safety and efficacy of a dual‐lesion approach targeting both VIM and PTT over one year. This report presents the first three PD patients who underwent dual‐target MRgFUS (dtMRgFUS), with outcomes tracked over a two‐year follow‐up.

**Methods:** Three tremor‐dominant PD patients, previously reported, underwent dual‐target MRgFUS treatment, assessed using a comprehensive set of primary and secondary outcome measures. Individual brain MRI scans were used to navigate and precisely target the VIM and PTT. Primary outcomes included the off‐medication Clinical Rating Scale for Tremor (CRST) and the Unified Parkinson's Disease Rating Scale Part III (UPDRS‐III). Secondary outcomes included UPDRS Parts I, II, and IV, the Hoehn and Yahr scale, the Neuropsychiatric Inventory, the PD Quality of Life Questionnaire (PDQ‐39), the Non‐Motor Symptoms Scale (NMSS), and the Clinical Global Impression (CGI). Baseline data were compared with results obtained 6 months, 1 year, and 2‐year post‐treatment.

**Results:** Tremor severity and motor deficits, as measured by CRST‐Part B and UPDRS‐III, showed significant improvement following dual‐target ablation (P < 0.05, nonparametric Mann‐Whitney U tests). Non‐motor symptoms also demonstrated marked improvement at the 2‐year follow‐up. No severe adverse effects were reported.

**Conclusion:** Stepwise dual‐lesion targeting of the VIM and PTT using MRgFUS is a safe and effective therapeutic approach for Parkinson's disease patients over a 2‐year period.

**Disclosure:** Nothing to disclose.

## EPO‐269

### Exploring gender variations in the treatment of motor symptoms in Parkinson's disease

#### M. Cebuc ^1^; L. Rotaru^1^; I. Moldovanu^2^; S. Odobescu^2^; A. Lupușor^3^; I. Timotin^3^; S. Lozovanu^3^; V. Vovc^3^; S. Groppa^4^

##### ^1^Parkinson's Disease and Movement Disorder Center, Diomid Gherman Institute of Neurology and Neurosurgery, Chișinău, Republic of Moldova; ^2^Functional Neurology Research Unit, Diomid Gherman Institute of Neurology and Neurosurgery, Chișinău, Republic of Moldova; ^3^Department of Human Physiology and Biophysics, Nicolae Testemițanu State University of Medicine and Pharmacy, Chișinău, Republic of Moldova; ^4^Department of Neurology No.2, Nicolae Testemițanu State University of Medicine and Pharmacy, Chișinău, Republic of Moldova

**Background and aims:** Existing research highlights the role of gender in Parkinson's disease (PD) with higher prevalence, earlier onset, more severe forms and akinetic‐rigid phenotypes seen in males. The study aims to help tailor therapeutic strategies based on the patient's sex by analysing their dopaminergic medication profile.

**Methods:** This retrospective study involved 1741 PD patients (M‐54.9%, n=955; F‐45.1%, n=786) of the D. Gherman Institute of Neurology and Neurosurgery stratified based on gender (M‐ 69.01±7.68; F‐69.73±7.53 years, p=0.05). PD motor symptoms’ phenotype and severity, along to dopaminergic drugs’ type and dosages were analysed using IBM‐SPSS statistical tools.

**Results:** The male and female samples showed clinical homogeneity. Akinetic‐rigid phenotypes (52.6% vs. 54.1%, df=2, p=0.722) and severe forms were frequent (46.9% vs. 47.7%, df=2, p=0.366), with significantly more fluctuations (33.4% vs. 56.1%, df=1, p<0.01) and dyskinesia (31.1% vs. 50%, df=1, p<0.01) in women. LEDD had minimal variation across genders (1049.15±568.08 vs. 1100.83±584.54, p>0.05). Both had comparable frequencies of levodopa (99.2% vs. 98.7%, df=1, p=0.477) and dopamine agonists (17% vs. 15%, df=1, p=0.294) usage, but amantadine prevailed in women (9.3% vs. 17.7%, df=1, p<0.01). Females employed higher daily dosages (mg) of levodopa (1030.94, ±562.12 vs. 1063.62, ±588.10, p>0.05) and amantadine (225.56, ±89.07 vs. 227.94, ±82.04, p>0.05), whilst males of dopamine agonists (2.02±0.67 vs. 1.73±0.78, p=0.008).

**Conclusion:** Although samples were generally comparable, female participants had more motor complications and required greater dosages of preventive pharmacological intervention, likely due to metabolic peculiarities that need further research.

**Disclosure:** Nothing to disclose.

## EPO‐270

### Tracking the role of the sub‐thalamic nucleus in speech: a deep brain stimulation‐electroencephalography study

#### M. Mancuso ^1^; M. Fahimi^2^; F. Mollaei^3^; V. Litvak^2^; P. Limousin^4^

##### ^1^Human Neuroscience Department, La Sapienza University of Rome; Unit of Functional Neurosurgery, UCL; ^2^Wellcome centre for Neuroimaging, UCL; ^3^Psychology and Clinical Language Sciences, Reading University; ^4^Unit of Functional Neurosurgery, UCL

**Background and aims:** PD patients often developed hypokinetic dysarthria. Deep brain stimulation of the subthalamic nucleus (STN‐DBS) has mixed effects on speech, ranging from improvement to deterioration. An STN–temporal cortex loop oscillating in the alpha frequency band may serve a speech‐related function. DBS might therefore impart its negative effects on dysarthria by affecting this network. This study aims to characterize the role of the STN–temporal lobe circuitry in speech production using a speech task performed during STN‐DBS, alongside STN local field potentials (LFP) and EEG recordings. The primary objective is to evaluate whether the STN–temporal lobe network is involved in speech production, and if DBS can disrupt this network.

**Methods:** In this preliminary report, we studied 6 patients with bilateral STN implants, each assessed off‐medication (antiparkinsonian drugs were discontinued for 12 hours), in both on‐ and off‐STN‐DBS conditions. Patients performed a compensation speech paradigm task involving real‐time perturbation of vocal pitch and first formant. High‐density EEG and STN LFPs were recorded during these tasks. We evaluated STN–temporal circuit connectivity by computing coherence between STN LFPs and EEG signals across different task phases.

**Results:** We identified theta–alpha coherence between the left STN and the left frontal and posterior parieto‐temporal areas at rest. This coherence showed tendency for modulation during the vocal task. DBS activation tended to reduce this modulation

**Conclusion:** Our preliminary results suggested the presence of speech modulated coherence in the alpha–theta range between the STN and frontal–parietal areas, possibly originating from a tangential dipole in the temporal regions.

**Disclosure:** This research was funded by the Brain Entry Clinical Fellowship.

## EPO‐271

### Predictors of improvement in activities of daily living and QoL in patients treated with foslevodopa/foscarbidopa

#### A. Antonini^1^; B. Bergmans^2^; F. Gandor^3^; S. H. Isaacson^4^; D. Santos Garcia^5^; L. Bergmann^6^; J. Carlos Parra^6^; R. Gupta^6^; A. Saad ^6^; J. L. Aldred^7^

##### ^1^Parkinson and Movement Disorders Unit, Study Centre for Neurodegeneration, Department of Neuroscience, University of Padova, Padova, Italy; ^2^AZ St‐Jan Brugge, Brugge, Belgium and Ghent University Hospital, Ghent, Belgium; ^3^Department of Neurology, Macquarie University, Sydney, Australia; Department of Neurology, St. Vincent's Hospital, Sydney, Australia; Movement Disorders Hospital, Beelitz‐Heilstätten, Germany; Otto‐von‐Guericke‐University, Magdeburg, Germany; ^4^The Institute for Neurodegenerative Diseases, Boca Raton, USA; ^5^CHUAC, Complejo Hospitalario Universitario de A Coruña, A Coruña, Spain; ^6^AbbVie Inc., North Chicago, USA; ^7^Selkirk Neurology & Inland Northwest Research, PLLC, Spokane, USA

**Background and aims:** Treatment with foslevodopa/foscarbidopa (LDp/CDp) improved motor experiences of daily living (m‐EDL) and quality of life (QoL) for patients with advanced Parkinson's disease (aPD) in a 52‐week, multicountry, open‐label safety trial (NCT03781167). This post hoc analysis examined baseline characteristics linked to improvements in m‐EDL and QoL outcomes.

**Methods:** A multiple logistic regression model using backward elimination method was used for variable selection and estimating associations between baseline characteristics and efficacy outcomes, namely Movement Disorder Society‐Sponsored Unified Parkinson's Disease Rating Scale Part II (MDS‐UPDRS‐II) and Parkinson's Disease Questionnaire (PDQ‐39) Summary Index scores, representing m‐EDL and QoL, respectively (Figure 1). The models were adjusted for the respective baseline values of the outcomes. A minimal clinically important difference (MCID) was defined as <=‐3.05 for MDS‐UPDRS‐II and <=‐4.72 for PDQ‐39 Summary Index scores at the final visit from baseline.

**Figure:**
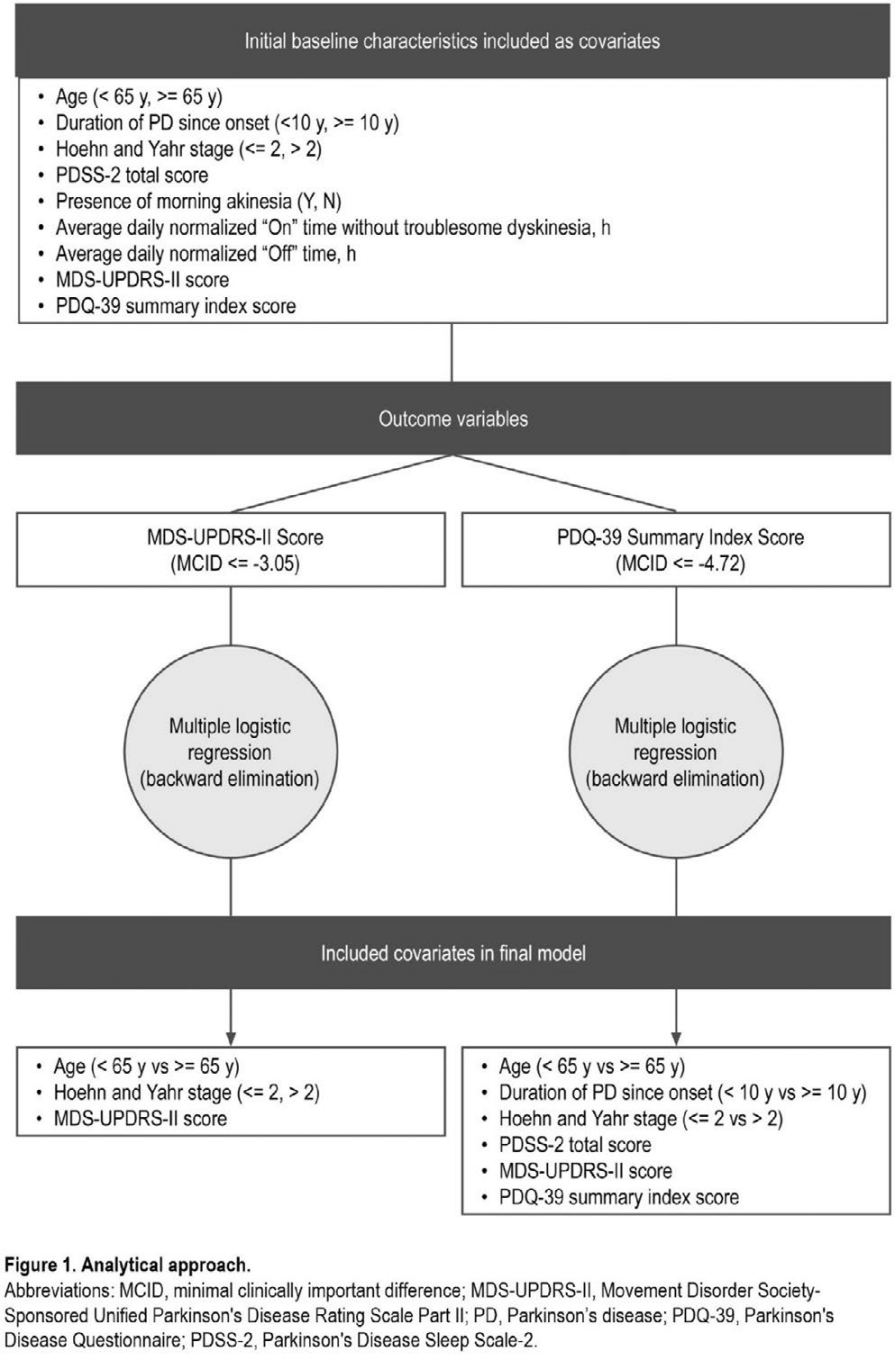
**FIGURE 1** Analytical Approach with FN

**Results:** Of the covariates assessed, patients <65 years had a 56% higher likelihood (Odds Ratio [95% CI] = 1.560 [1.06‐2.31]) of achieving an MDS‐UPDRS‐II Score MCID (P=.0258) than patients >=65 years (Table 1). Patients with Hoehn and Yahr (H&Y) stage <=2 had a 77.3% (1.773 [1.07‐2.95]) and 80.4% (1.804 [1.06‐3.05]) higher likelihood of achieving both an MDS‐UPDRS‐II Score MCID (P=.0270) and a PDQ‐39 Summary Index Score MCID (P=.0282) than patients ≥65 years and with H&Y stage >2 (Table 1).

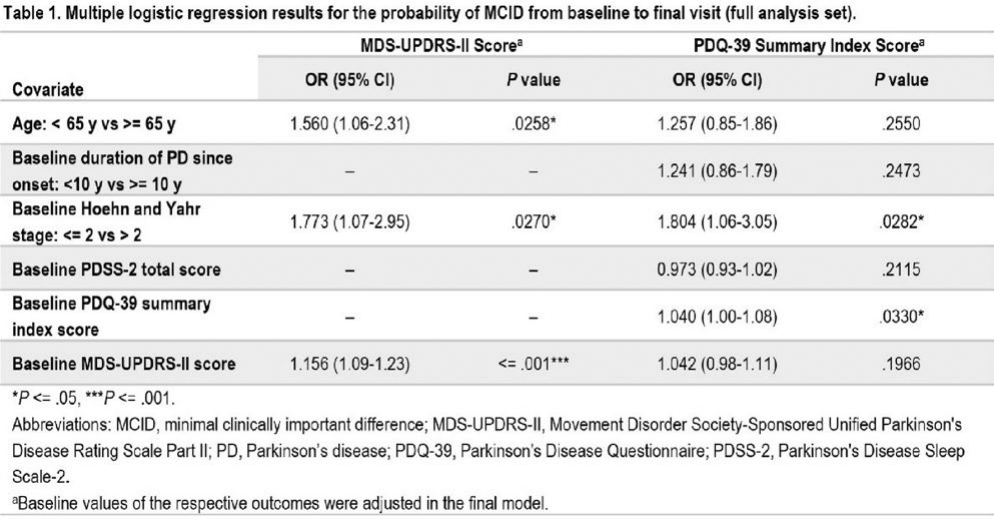

**Conclusion:** Although LDp/CDp enhances activities of daily living and QoL for patients with aPD, less advanced disease stage and younger age (<65 years) increase the likelihood of achieving clinically meaningful improvements in these outcomes.

**Disclosure:** AA has received fees, honoraria, and/or grants from AbbVie, Bayer, Biopharma, Bial, Britannia, Ever Pharma, Horizon 2020, Italian Ministry of University and Research, Italian Ministry of Health, Jazz, Medscape, Next Generation EU ‐ National Center for Gene Therapy and Drugs (Investment PE8 [Age‐It: “Ageing Well in an Ageing Society”]), Roche, Theravance, UCB, and Zambon. BB has received fees and/or grants from AbbVie, EG, Ipsen, Merz, and Zambon. FG has served as an advisory board member for AbbVie and has received honoraria from AbbVie, Bial, and Stada. SHI has received honoraria from Abbvie, Amneal, Cerevel, Mitsubishi Tanabe, Neuroderm, and Supernus. DSG has received fees, honoraria, and/or grants from Abbvie, UCB, Lundbeck, KRKA, Zambon, Bial, Italfarmaco, Teva, Archímedes, Esteve, Stada, Merz, and “Fundación Professor Novoa Santos”. LB, JCP, RG, and AS are full‐time employees of AbbVie and may hold AbbVie stock. JLA has received honoraria from AbbVie, Biogen, Roche, Takeda, Sage Therapeutics, Praxis, UCB, PhotoPharmics, Aptinyx, Athira, Revance, Acadia, Neurocrine, Sanofi, Merz, Scion, Sunovion, and Centogene AG.

## EPO‐272

### Noninvasive deep brain stimulation of subthalamic nucleus in Parkinson's disease: temporal and spatial properties

#### M. Lamoš ^1^; M. Bočková^1^; F. Missey^2^; J. Trajlínek^2^; A. do Nascimento Arantes^2^; P. Daniel^1^; J. Chrastina^3^; R. Jančálek^3^; E. Glowacki^4^; I. Rektorová^1^; A. Williamson^2^

##### ^1^First Department of Neurology, Masaryk University School of Medicine, St. Anne's Hospital, Brno, Czechia; ^2^Neuromodulation Technology Research, International Clinical Research Center, St. Anne's University Hospital, Brno, Czechia; ^3^Department of Neurosurgery, Masaryk University School of Medicine, St. Anne's Hospital, Brno, Czechia; ^4^Bioelectronics Materials and Devices, Central European Institute of Technology, Brno University of Technology, Brno, Czechia

**Background and aims:** Temporal Interference stimulation (TIS) is a novel non‐invasive brain electrical stimulation technique that has a potential to modulate deep brain regions. The focal modulation of TIS is possible using two high frequency signals (>1kHz), which interfere to create low frequency envelope modulating the target area. Recent work presented the capability of TIS to focus the subthalamic nucleus (STN) and to suppress STN beta oscillations in Parkinson's disease. Here, we present temporal and spatial characteristics of this modulation technique.

**Methods:** Implanted DBS leads were temporally externalized for local field potentials (LFP) recording in 8 patients with Parkinson's disease indicated for STN‐DBS. STN‐TIS was performed by 2 pairs (f1 = 9.00kHz; f2 = 9.13kHz, 2mA per pair max.) of scalp electrodes placed in frontoparietal regions for 3 minutes. The following 3 minutes of resting‐state were then used for LFP evaluation.

**Results:** Suppressed beta activity in STN re‐occurred back in approx. 120 seconds for STN‐TIS. In control condition, where conventional DBS was used, the after‐effect varied across group and in some patients was more immediate than TIS. No electrical field enhancement around the DBS lead was found in case of transcranial TIS.

**Conclusion:** TIS is a different type of neuromodulation, applied in a sinusoidal pattern at a sub‐threshold intensity; DBS is a pulsed pattern supra‐threshold intensity stimulation that generates action potentials. Despite these different mechanisms of action there is growing evidence that TIS has the potential to influence deep brain oscillatory activity and induce clinical effects in a way similar to DBS.

**Disclosure:** The work was supported by LX22NPO5107 (MEYS), European Union‐Next Generation EU.

## EPO‐273

### Slower turning predicts future Parkinson's disease diagnosis: A longitudinal study

#### M. Elshehabi ^1^; C. Hansen^1^; M. Hobert^2^; A. von Thaler^3^; K. Brockmann^3^; F. Metzger^4^; B. Galna^5^

##### ^1^Department of Neurology, University Medical Center Schleswig‐Holstein and Kiel University, Kiel, Germany; ^2^Department of Neurology, University Medical Center Schleswig‐Holstein and University of Lübeck, Lübeck, Germany; ^3^Department of Neurodegenerative Diseases, University Hospital Tübingen, and Center for Neurodegenerative Diseases, Tübingen, Germany; ^4^Geriatric Center and the Department of Psychiatry and Psychotherapy, University Hospital Tübingen, Tübingen, Germany; ^5^School of Allied Health (Exercise Science) | Centre for Healthy Ageing | Personalised Medicine Centre, Murdoch University, Australia

**Background and aims:** The use of wearable technology enables precise measurement of turning movements during walking. Cross‐sectional studies have shown that a decline in turning can be detected in the early clinical and even preclinical stages of Parkinson's disease (PD). This prospective longitudinal study aims to quantify the change in turning performance among older adults and determine if turning performance predicts future PD diagnosis.

**Methods:** A total of 933 participants (mean age = 66.1 years) from the TREND study were included for this analysis over five 2‐year intervals, with the development of clinically evident PD tracked. Participants walked up and down a 20‐meter hallway for one minute at their preferred pace, wearing a digital device on their lower back to capture turning. Longitudinal trajectories of turning performance were modelled using random effects linear mixed models to establish the interval between initial turning changes and PD diagnosis. Cox regression was used to assess whether initial turning measures could predict the time to PD onset, controlling for age and sex.

**Results:** Of all participants, 23 were diagnosed with idiopathic PD, an average of 5.3 years after baseline assessment. Slower peak angular velocity at baseline was associated with a higher hazard of PD diagnosis, with deviations from controls emerging approximately 8.7 years before diagnosis (Figure 1). Other parameters showed no prediction value of PD diagnosis.

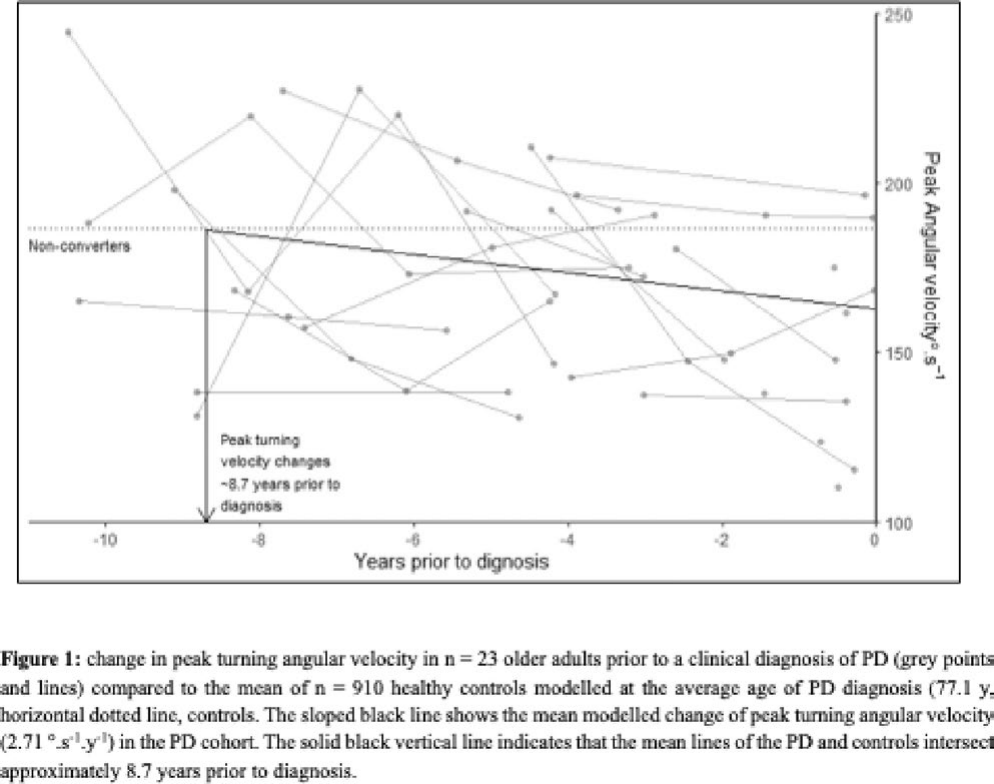

**Conclusion:** Peak angular velocity during turning appears to be a promising marker for identifying and tracking motor progression in the pre‐diagnostic phase of PD.

**Disclosure:** nothing to disclose.

## EPO‐274

### Unveiling the gut‐brain axis in Parkinson's disease: A meta‐analysis of randomized‐controlled trials

#### N. Papathanasiou

##### Department of Medicine, National Kapodistrian University of Athens, Athens, Greece

**Background and aims:** Gut dysbiosis has been associated with the pathogenesis of Parkinson's disease (PD). Studies show that probiotic supplements may mitigate PD symptoms. However, the effect of broader gut microbiota interventions on PD is not well‐defined. Thus, we performed a meta‐analysis to explore their role in managing PD symptoms.

**Methods:** We systematically searched MEDLINE, Scopus, and Cochrane databases from inception until December 05, 2024, for placebo‐controlled randomized trials that estimated the effects of probiotics, synbiotics, and fecal microbiota transplantation (FMT) on PD patients. To assess the efficacy of the different interventions, subsequent subanalyses were performed.

**Results:** A total of 11 RCTs comprising 756 patients were included. Of those, 410 (54%) received gut microbiota interventions. Follow‐up ranged from 4 weeks to 6 months. We found that gut microbiota interventions significantly improved motor symptoms (MDS‐UPDRS Total: SMD = ‐0.42; 95% CI ‐0.67 to ‐0.16; p = 0.001) and quality of life (PDQ‐39: SMD= ‐0.43; 95% CI ‐0.66 to ‐0.2; p= 0.0002) compared to placebo. Depression parameter was also significantly reduced in this group (SMD= ‐0.41; 95% CI ‐0.71 to ‐0.1; p= 0.009), with probiotics outweighing FMT. No significant changes in bowel movements and Bristol Stool Form Scale (BSFS) were noted. However, BSFS significantly increased (SMD = 0.55; CI 0.28 to 0.82; p < 0.00001) in patients with low (<= 3) baseline score.

**Figure:**
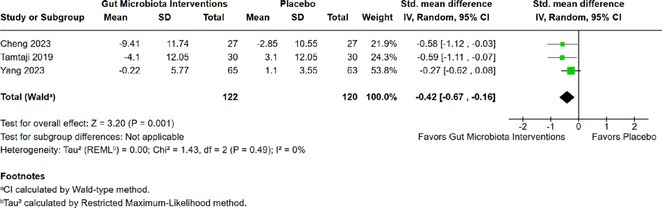
**FIGURE 1** MDS‐UPDRS total score was significantly reduced in patients receiving gut microbiota interventions compared to placebo.

**Figure:**
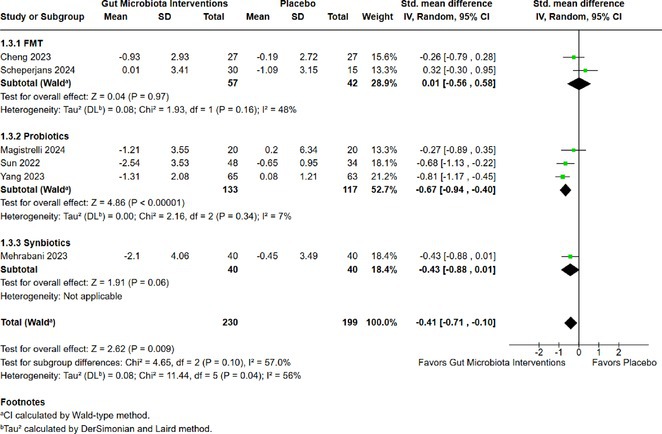
**FIGURE 2** Gut microbiota interventions significantly improved depression symptoms in PD patients, with probiotics showing the greatest efficacy.

**Figure:**
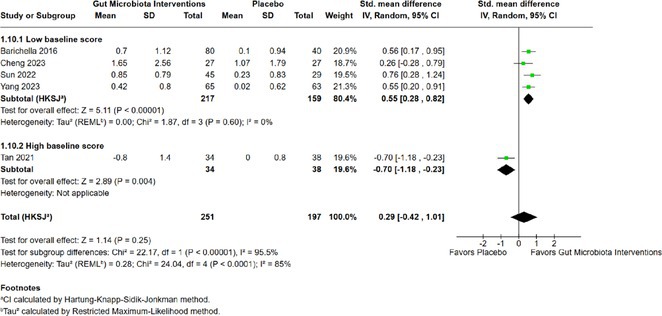
**FIGURE 3** Bristol Stool Form Scale (BSFS) was significantly increased in patients with low baseline score treated with gut microbiota interventions.

**Conclusion:** Gut microbiota interventions seem to alleviate both motor and non‐motor symptoms of PD patients. Further research exploring FMT and its impact on the disease's trajectory is needed.

**Disclosure:** Nothing to disclose.

## EPO‐275

### Amantadine extended‐release tablets for the treatment of parkinson's disease: A phase IV multicenter, single‐arm study

#### A. Yeole^1^; D. Nagarwal^2^; A. Barua^3^; P. Kumar^4^; B. Chaudhary^5^; A. Ansari^6^; K. Rakesh^7^; A. Verma^8^; N. Venkata sundarachary^9^; R. Shah^10^; M. Rajurkar^11^; S. Behera^12^; C. Bornare^11^; S. Sonowal ^11^; D. Sonawane^11^; S. Pandit^11^; P. Devkare^11^; D. Patil^11^; P. Ghadge^11^; L. Lakhwani^12^; S. Mehta^11^; S. Joglekar^13^

##### ^1^Surya Multispeciality Hospital, Nashik, India; ^2^Jawahar Lal Nehru Medical College, Ajmer, India; ^3^Guwahati Neurological Research Centre & Hospital, Guwahati, India; ^4^PMSSY Victoria Hospital, Bengaluru, India; ^5^Apex Hospitals Pvt. Ltd, Jaipur, India; ^6^City Neurology Centre, Varanasi, India; ^7^Excel Hospital, Secunderabad, India; ^8^GSVM Medical College, Kanpur, India; ^9^Guntur Medical College & Government General Hospital, Guntur, India, ^10^V. S. General Hospital, Ahmedabad, India, ^11^Sun Pharma Laboratories Limited, Mumbai, India, ^12^Ex Sun Pharma Laboratories Limited, Mumbai, India, ^13^Ex Sun Pharmaceutical Industries Limited, Mumbai, India

**Background and aims:** This subset analysis of a phase IV study was performed to assess the safety and efficacy of amantadine extended‐release (ER) tablets in Parkinson's Disease (PD) patients.

**Methods:** A subset of 141 patients from this single‐arm, open‐label multicenter, phase IV Indian study (CTRI/2023/04/051973), with PD was analyzed for safety and efficacy of amantadine ER tablet. The starting dose was 129 mg orally once; further uptitrated at weekly intervals to a maximum daily dose of 322 mg (administered as a 129 mg and 193 mg tablets) as per patient's response and tolerability. Treatment duration was up to 12 weeks depending on dose uptitration. Primary objective was safety assessment. Efficacy endpoints were change in MDS‐UPDRS score (Part I, II, III, IV and total score), OFF hours, ON hours with dyskinesia and PDQ‐39‐QOL.

**Results:** Total 29 treatment‐emergent adverse events occurred in 16 patients; most common was headache. The mean (±SD) MDS‐UPDRS total score decreased significantly (p<0.0001) from 71.24 (±34.17) at baseline across all visits to 47.08 (±29.13) at EOT. Also, the mean (±SD) MDS‐UPDRS part I, II, III and IV score significantly decreased from baseline till EOT (p<0.0001). Daily mean (±SD) off hours decreased significantly (p<0.0001) from 1.89 (±2.11) at baseline to 1.04 (±1.43) till EOT. There was significant decrease in mean (±SD) daily ON hours with dyskinesia from baseline till EOT (p<0.0001). The PDQ‐39‐QOL score decreased from 32.29 (±19.15) at baseline to 19.71 (±14.88) till EOT (p<0.0001).

**Conclusion:** Amantadine ER tablet was safe and efficacious in Parkinson disease.

**Disclosure:** The study was funded by Sun Pharma Laboratories Limited (SPLL).

## EPO‐276

### MR guided focused ultrasound dual lesioning for Parkinson disease

#### V. KRISHNAN ^1^; M. KARUPPANNASAMY^2^; A. VISWANATHAN^3^; R. KIRUPAKARAN^4^

##### ^1^NEUROLOGIST AND NEUROSONOLOGIST ROYAL CARE HOSPITAL COIMBATORE; ^2^NEUROSURGEON ROYAL CARE HOSPITAL COIMBATORE; ^3^NEUROLOGIST ROYAL CARE HOSPITAL COIMBATORE; ^4^NEUROSURGEON ROYAL CARE HOSPITAL COIMBATORE

**Background and aims:** lesioning procedures have become safe with advent of MRgFUS.

**Methods:** Dual lesioning of pallido thalamic tract and vim/vo for the past 2 yrs. The pallido thalamic tract was targeted at two sites, one medial and one lateral. The lateral target was located 6.5 to 8.5 mm away from the lateral border of the 3rd ventricle at half the distance from the AC‐PC in the same plane. The medial target was located 1.5 mm inferior, 1.5mm posterior and 1.5 mm medial to the lateral target. The subthalamic body, mammillothalamic tract and the internal capsule were marked on the planning MRI and fused later with the live MRI during lesioning. 7 targets were identified for the vim/vo by image based and by standard coordinates. Two are newly described royal care targets. As the vim/vo is a volume multiple lesions were placed monitoring the patient response.

**Figure:**
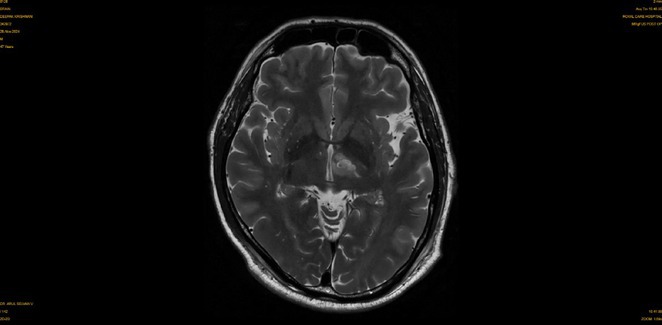
**FIGURE 1** MEDIAL PTT TARGET

**Figure:**
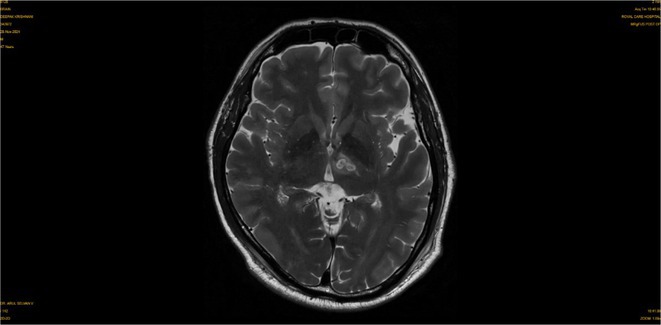
**FIGURE 2** LATERAL PTT TARGET

**Figure:**
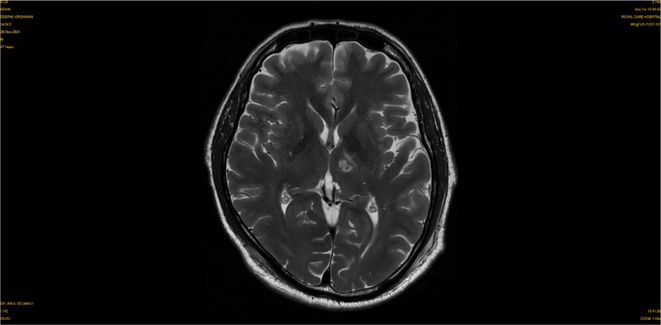
**FIGURE 3** VIM‐VO TARGET

**Results:** Total follow up has been for 2yrs. Results of 25 cases of dual lesioning are discussed. There was more than 70% reduction in the UPDRS motor scores. Some improvement in the non‐motor symptoms were also noted.

**Conclusion:** MRgFUS lesioning is safe and effective treatment for PD. Effects are immediate and are also lasting. There is a significant reduction in DOPA dosage. Dyskinesias disappear after PTT lesioning and do not reappear on DOPA challenge. Dual lesioning and sequential bilateral lesioning is also safe and effective. The craving for DOPA is substantially reduced. The quality of life improves over time.

**Disclosure:** “Nothing to disclose.”

## EPO‐277

### Movement disorders in GLUT1 deficiency syndrome: A systematic review of the literature

#### Z. Bercini ^1^; F. Cavallieri^2^; A. Gessani^1^; J. Rossi^2^; M. Bassi^3^; V. Fioravanti^2^; G. Di Rauso^2^; E. Monfrini^4^; A. Latorre^5^; E. Menozzi^5^; E. Mulroy^5^; M. Carecchio^6^; A. Di Fonzo^4^; R. Erro^7^; E. Moro^8^; F. Valzania^2^

##### ^1^Department of Biomedical, Metabolic and Neurosciences, University of Modena and Reggio Emilia, Modena, Italy; ^2^Neurology Unit, Neuromotor & Rehabilitation Department, Azienda USL‐IRCCS di Reggio Emilia, Reggio Emilia, Italy; ^3^Medical Library, Azienda Unità Sanitaria Locale‐IRCCS di Reggio Emilia, Reggio Emilia, Italy; ^4^Neurology Unit, Fondazione IRCCS Ca' Granda Ospedale Maggiore Policlinico, Milan, Italy; ^5^Department of Clinical and Movement Neurosciences, UCL Queen Square Institute of Neurology, London, UK; ^6^Movement Disorders Unit, Department of Neuroscience, University of Padua, Padua, Italy; ^7^Center for Neurodegenerative Diseases (CEMAND), Department of Medicine, Surgery and Dentistry, “Scuola Medica Salernitana”, University of Salerno, Salerno, Italy; ^8^Grenoble Alpes University, CHU of Grenoble, Division of Neurology, Grenoble Institute of Neurosciences, INSERM, Grenoble, France

**Background and aims:** Glut‐1 deficiency syndrome (GLUT1‐DS) is a rare brain energy failure syndrome caused by impaired glucose transport across brain tissue barriers. Movement disorders (MD) are a prominent feature of the disease. Here we describe in details the clinical presentation of movement disorders in GLUT1‐DS by performing a systematic review of the published cases.

**Methods:** We conducted a comprehensive and systematic review of the literature. The Preferred Reporting Items for Systematic Reviews and Meta‐Analyses (PRISMA) guidelines were followed. The search comprised five electronic databases: Medline, Embase, Cinahl, Scopus, Web of Science. Any case reports or case series which report a detailed clinical description of MD in GLUT1‐DS patients was included. Data collected from each study included patient demographic characteristics, GLUT1‐DS diagnosis, MD and treatments.

**Results:** The initial search yielded 881 publications. After duplicates’ removal, 606 titles were screened, and fifty‐nine articles reporting 76 patients (males: 40/76; age at GLUT1‐DS diagnosis 14.7 years [±12.70]) met the inclusion criteria. Ataxia was the most frequent MD (43/76) followed by paroxysmal dyskinesia (35/76), dystonia (20/76), tremor (11/76), chorea (7/76) and myoclonus (5/76). Pharmacological treatments determined heterogenous response, whereas ketogenic diet led mostly to complete remission or significant improvement (35/44), and partial or no clinical response in the minority of cases (5/44 and 4/44, respectively).

**Conclusion:** This review suggests that GLUT1‐DS can be associated with a wide range of MD particularly ataxia, paroxysmal dyskinesia and dystonia. The effects of pharmacological treatments were limited while ketonic diet was helpful in the majority of cases.

**Disclosure:** Nothing to disclose.

## MS and Related Disorders 2

## EPO‐278

### The role of retinal hyper‐reflecting foci in axonal damage and retinal vascular density in multiple sclerosis

#### A. Castiello ^1^; A. Carotenuto^1^; G. Cennamo^2^; M. Rinaldi^2^; A. Esposito^1^; G. Corsini^1^; V. Nicolella^1^; D. Ranucci^1^; M. Petracca^3^; M. Moccia^1^; R. Lanzillo^1^; V. Brescia Morra^1^; C. Costagliola^2^

##### ^1^Department of Neuroscience, Reproductive Science and Odontostomatology, Federico II University of Naples, Naples, Italy; ^2^Eye Clinic, Public Health Department, University of Naples Federico II, Naples, Italy; ^3^Department of Human Neurosciences, Sapienza University, Rome, Italy.

**Background and aims:** Optical Coherence Tomography (OCT) is a key tool in Multiple Sclerosis (MS), detecting neuro‐axonal atrophy, which correlates with brain atrophy. OCT Angiography (OCTA) allows assessment of retinal vascular density (VD), reduced in MS patients, especially in the foveal region and optic nerve head (ONH). Hyper‐reflecting foci (HRF) are potential markers of activated microglia. This study investigates the relationship between HRF, axonal damage, and VD in relapsing‐remitting MS (RRMS) patients.

**Methods:** We evaluated ganglion cell complex (GCC), retinal nerve fiber layer (RNFL) and VD in superficial and deep capillary plexuses, the ONH and radial peripapillary capillary plexus in RRMS patients. Patients with history of optic neuritis were excluded. HRF were defined as isolated, small‐size (<30 mm) elements with moderate reflectivity without any back shadowing. Association between OCT‐SD, OCT‐A and HRF was assessed through Correlations between SDOCT and OCTA parameters, using linear mixed model using age and sex as covariates and subject as random factor.

**Results:** We enrolled 19 RRMS patients (mean age 38.9 ± 14.6 years; median disease duration 5 years; median EDSS 2.5). The median number of HRF per subject was 4 (range 1–7). We found a positive inter‐eye correlation for HRF (coeff. 0.50, p=0.03). Number of HRF correlated with RNFL (correl. coeff.= ‐35.3, p=0.03) and with inside disc VD (correl. coeff. = ‐2.00, p=0.01).

**Conclusion:** HRF are primarily associated with axonal damage rather than vascular parameters, suggesting they reflect microglial activation driving chronic inflammation and neurodegeneration in MS. These findings position HRF as potential biomarkers for disease monitoring and therapeutic evaluation.

**Disclosure:** A.E. has received honoraria from Novartis. M.M. has received research grants from ECTRIMS‐MAGNIMS, the UK MS Society, and Merck, and honoraria from Biogen, BMS Celgene, Ipsen, Janssen, Merck, Novartis, Roche, and Sanofi‐Genzyme. M.P. has received research grants from the Italian MS Foundation and Baroni Foundation, honoraria from Health & Life and Biogen, and sponsorship for travel/meeting expenses from Novartis, Roche, and Merck. R.L. has received honoraria from Biogen, Merck, Novartis, Roche, and Teva. V.B.M. has received research grants from the Italian MS Society and Roche, and honoraria from Bayer, Biogen, Merck, Mylan, Novartis, Roche, Sanofi‐Genzyme, and Teva. A.C. has received research grants from Almirall, research grants from ECTRIMS‐MAGNIMS, and honoraria from Almirall, Biogen, Roche, Sanofi‐Genzyme, Merck, Ipsen, and Novartis. None of the other authors has any conflict of interest to disclose.

## EPO‐279

### Quantitative clinical observation of neurodegeneration in the case secondary progressive multiple sclerosis (SPMS)

#### A. Buniak ^1^; A. Mikitchuk^2^; O. Pereverzeva^1^

##### ^1^neurological department, Republican research and clinical center, Minsk, Belarus; ^2^Faculty of Radiophysics and Computer Technologies, Belarusian State University, Minsk, Belarus

**Background and aims:** Neurodegeneration in secondary progressive multiple sclerosis (SPMS) can be manifested by decreased MR density near ventricular horns, old lesions, “black holes” and “dirty” white matter (DWM) on magnetic resonance imaging (MRI). The process of density loss cannot be assessed using a segmentation approach. The evaluation of SPMS neurodegeneration by means of temporal differences of pre‐registrated and normalized MR‐images was observed.

**Methods:** MR‐data collected for patient with SPMS (2021 – 2024), FSPGR images (256x256x272) at 2021 are recorded to be reference. All others are co‐registrated and re‐sliced with reference (SPM12) to be located in the similar spatial basis. Then brightness of MR‐images is slice‐by‐slice normalized to the reference one (goal is minimization of brightness differences) to be in the same brightness basis. Consequently, temporal differences (calculated for each pair of years) are in the same spatial and brightness basis. This allows to sum brightness values in specified region of interest (ROI). These sums allow to quantitative compare images over time period. To assess the neurological symptoms, we used EDSS.

**Results:** This approach allows to analyze ventricle increase, DWM and lesion MR‐density reduction (fig. 1). In the case under study, ventricles expand faster, the areas of DWM and especially periventricular lesion density reduces slower (fig. 2,3). The EDSS score increased from 5.5 in 2021‐2022 to 6.5 in 2024 (fig. 3)

**Figure:**
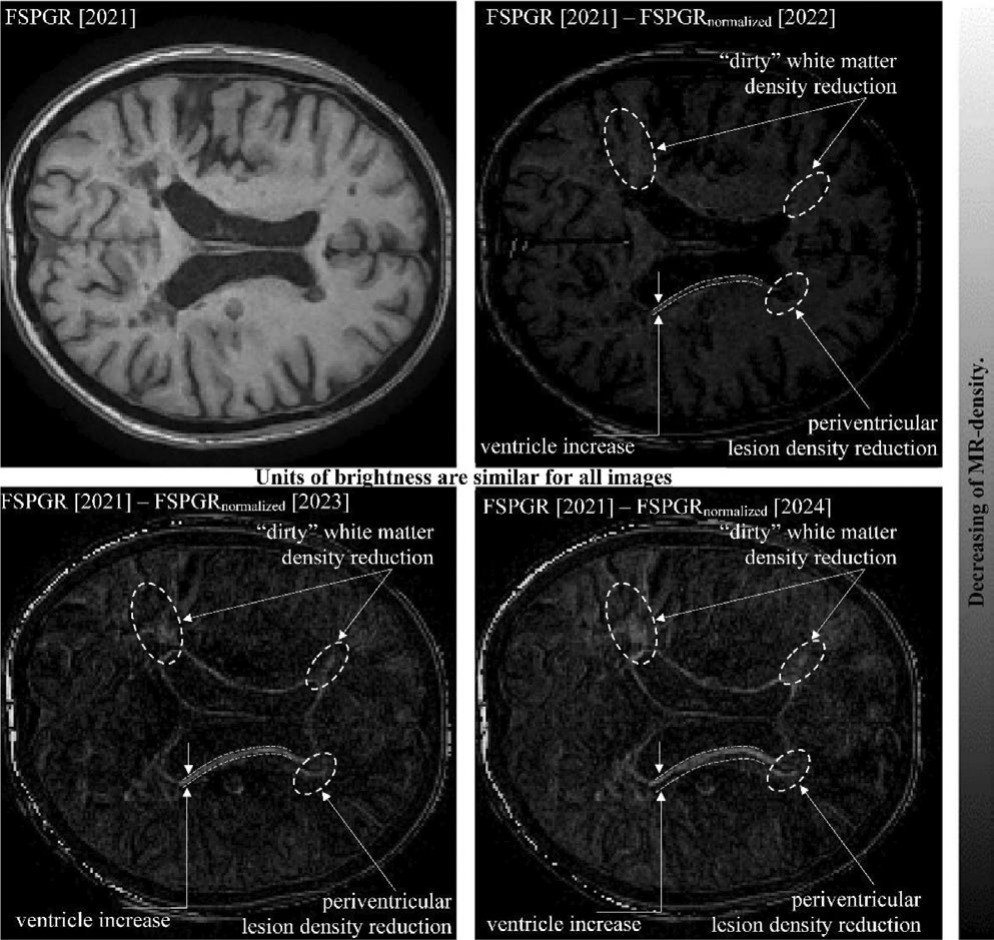
**FIGURE 1** Decreasing of MR‐density

**Figure:**
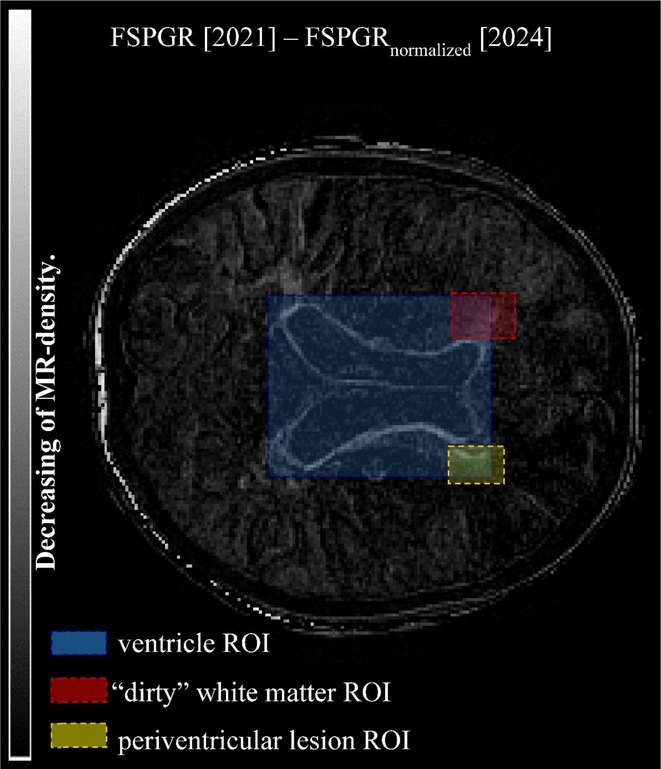
**FIGURE 2** ROI with MR‐density reduction

**Figure:**
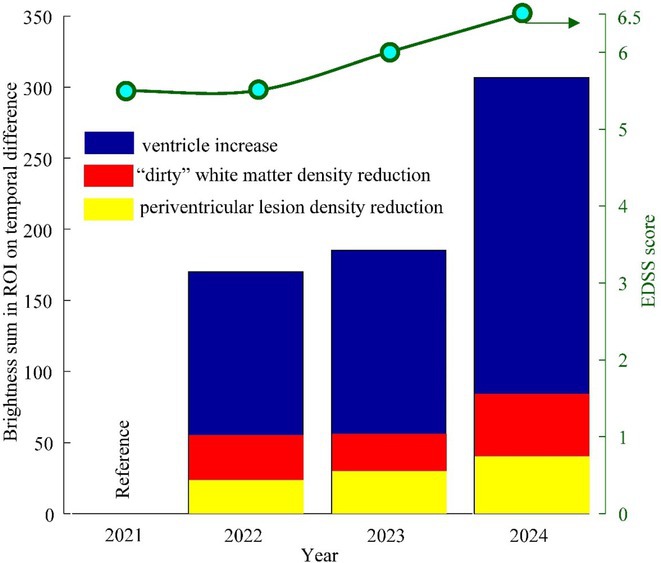
**FIGURE 3** Changes in brightness sum in ROI and EDSS score

**Conclusion:** Calculation of temporal differences of pre‐registrated and normalized MR‐images allows to visualize and score neurodegeneration quantitatively. Rapid grows of sum brightness values correlates well with EDSS score.

**Disclosure:** Nothing to disclose.

## EPO‐280

### The central vein sign: A valuable tool for differential diagnosis of demyelinating diseases

#### C. Oreja‐Guevara ^1^; J. Casablanca Mezquita^2^; I. Gómez‐Estevez^1^; E. Alba Suárez^1^; J. Alvarez‐Linera^3^; L. García‐Vasco^1^

##### ^1^Neurology, Hospital Clínico San Carlos, Madrid, Spain; ^2^Medicina, Universidad Complutense de Madrid; Spain; ^3^Radiology, Ruber Internacional, Madrid, Spain

**Background and aims:** The central vein sign (CVS), indicative of a vein within a white matter lesion visible (WMLs) on MRI, is considered suggestive of multiple sclerosis and will be incorporated into the new diagnostic criteria.

**Methods:** To investigate the utility of the central vein sign in clinical practice for patients with WMLs on brain MRI for the differential diagnosis.

**Results:** The study included 44 patients: 5 with undetermined diagnosis, 4 with confirmed MOGAD, 4 with confirmed NMO and 25 with confirmed RRMS having a disease duration of at least one year. All MS patients showed 70‐80% of WML CVS+ on cranial MRI and had at least 6 CVS+ lesions on T2*/FLAIR*. The 4 NMO patients showed no CVS+ lesions, and 3 out of 4 MOGAD patients had fewer than 6 CVS+ lesions on MRI. One MOGAD patient with 20 T2 lesions showed 9 CVS+ lesions but less than 40% of the total. Among the five individuals with unknown diagnoses, two had no CVS lesions: one was later diagnosed with migraine and small vessel disease, while the other tested positive for MOG antibodies. Two others displayed up to 20% CVS‐positive lesions: one was recently diagnosed with double‐negative NMOSD, and the other with MOGAD. One patient, presenting with headaches, had 45% CVS‐positive lesions; however, two lumbar punctures performed were negative for IgG OCBs, and the MRI findings did not meet the MAGNIMS criteria.

**Conclusion:** The CVS is a useful marker for ruling out MS in patients with WML and uncertain diagnoses in clinical practice

**Disclosure:** C Oreja‐Guevara received honoraria for speaking, consulting and serving on advisory boards from Alexion, Amgen, Biogen Idec, BMS, Horizon, Janssen, Merck, Novartis, Roche, Sanofi‐Genzyme, Sandoz, Viatris, Neuraxpharm and Teva. The rest of authors have nothing to disclose.

## EPO‐281

### Supporting brain‐healthy behaviours in MS through lifestyle education and intervention

#### C. Peel; J. Connor; S. Southward

##### Overcoming MS, Oxford, England

**Background and aims:** Evidence shows the importance of lifestyle for brain health, treatment and management, and preventing comorbidities in multiple sclerosis (MS) (Giovannoni 2024) yet awareness and application of lifestyle choices is not widespread (Wills 2024) and supportive education is essential (Bassetti 2022). Charity Overcoming MS developed education to inform and equip people living with MS to make sustainable lifestyle choices to improve their brain health and experience of MS.

**Methods:** Two education progammes ‘Pathways’ and ‘Retreats’ both inform and support an approach to positive living with MS encompassing nutrition, medication, physical activity, stress management and behaviour change. The education includes online group consultation, expert teaching from people with lived experience of MS, group discussion and peer support through online community. (Retreats also include a 3 day residential). Data are gathered pre‐ and post‐course.

**Results:** From 2022‐2024,198 people living with MS attended Retreats (n=78, 2 cohorts) or Pathways (n=120, 4 cohorts). Data across all six cohorts found marked improvements in perceived physical and mental wellbeing; subjective reporting rose by an average of 36% and 37% respectively. Confidence in understanding of, and adherence to the lifestyle medicine Program rose by an average of 44% and confidence in talking to friends and family about lifestyle choices by 31%.

**Conclusion:** Data from this education suggests people with MS engaging in tailored lifestyle education have improved understanding of, and likelihood of engaging with, healthy behaviours and that this improved self‐efficacy has a positive impact on their perceived health quality.

**Disclosure:** Nothing to disclose.

## EPO‐282

### Cognitive impairment in multiple sclerosis: An IMSCOGS overview of systematic reviews protocol

#### E. Baldin ^1^; M. Schettino^1^; C. De Santis^1^; M. Bassi^2^; D. Langdon^3^; S. Morrow^4^; L. Hancock^5^; M. Schoonheim^6^; C. Young^7^; F. Nonino^1^

##### ^1^IRCCS, Istituto delle Scienze Neurologiche di Bologna, Epidemiology and Statistics Unit, Cochrane Review Group Multiple Sclerosis and Rare Diseases of the CNS, Bologna, Italy; ^2^Azienda USL – IRCCS di Reggio Emilia; ^3^Royal Holloway, University of London, Egham, UK; ^4^Department of Clinical Neurosciences, Hotchkiss Brain Institute, University of Calgary; Calgary, AB, Canada; ^5^Neurological Institute, Cleveland Clinic; Cleveland, Ohio, USA; ^6^MS Center Amsterdam, Department of Anatomy and Neurosciences, Vrije Universiteit Amsterdam, Amsterdam Neuroscience, Amsterdam UMC, Amsterdam, The Netherlands; ^7^Institute of Systems, Molecular and Integrative Biology, University of Liverpool, Liverpool, UK; Walton Centre NHS Foundation Trust, Liverpool, UK

**Background and aims:** Cognitive impairment (CI) is a prevalent and disabling symptom in people with multiple sclerosis (PwMS), affecting various cognitive domains and contributing to poorer quality of life. However, the definitions of CI and the screening tools used in research and clinical settings vary widely. This overview of systematic reviews (SRs) aims to provide an outline of the current definitions of CI and the cognitive tests used to screen for CI in studies targeting PwMS. This overview of SRs is part of an International Multiple Sclerosis Cognition Society (IMSCOGS)‐European Committee for Treatment and Research in Multiple Sclerosis (ECTRIMS) collaborative project, aimed at reaching a formal consensus on how to define and assess CI in PwMS.

**Methods:** Relevant literature will be identified through a comprehensive search of peer‐reviewed SRs of diagnosis, intervention or prognosis from the following electronic databases: MEDLINE, Embase, PsychInfo, CINAHL. SRs including PwMS aged >=18, published since January 1, 2001, in English language will be considered. The overview of SRs will be conducted according to the JBI Reviewers’ Manual and reported following the PRIOR statement. SRs must provide a clear definition of acquired CI, disorder or dysfunction and may include individual tests or batteries to formally assess CI.

**Results:** This overview of SRs will summarize the definitions and the screening tools employed to assess CI in PwMS.

**Conclusion:** These findings may guide future researchers on improving practice.

**Disclosure:** This project is supported by ECTRIMS. EB, CDS, FN, MS are members of the Cochrane review group MS and rare diseases of the CNS, MMS is president of IMSCOGS, LH is co‐chair of steering committee for IMSCOGS, DL has received consultancy, sponsorship, lecture fees or research grants from Bayer, Merck, Novartis and BMS, SAM has served on advisory boards for Amgen, Biogen Idec, BMS/Celgene, EMD Serono, Novartis, Roche, Sanofi Genzyme, and has received research funds or investigator grant from Biogen Idec, MS Canada, National MS Society, and CIHR, CAY has received consultancy, sponsorship, lecture fees or research grants from Biogen, Merck, Roche, Vectura and BMS.

## EPO‐283

### The blood‐brain barrier in MS: correlation between clinical, CSF, MRI signature and albumin quotient in naïve patients

#### F. De Napoli^1^; V. Mauceri ^1^; A. Miscioscia^1^; A. Bertoldo^2^; E. Silvestri^2^; A. Marin^3^; P. Perini^1^; F. Rinaldi^1^; P. Gallo^1^; M. Puthenparampil^1^

##### ^1^Department of Neurosciences, University of Padova, Padua, Italy; ^2^Bioingeneer, Department of Information Engineering, University of Padova, Padua, Italy; ^3^Department of Biomedical Sciences, University of Padova, Padua, Italy

**Background and aims:** The alteration of the blood‐brain barrier (BBBD) plays a key role in the pathogenesis of Multiple Sclerosis (MS) and has been observed even before demyelinating lesions develop. The permeability of the BBB, measured as QAlb (AlbL/AlbS), correlates with central inflammatory load. This study aims to evaluate whether BBBD correlates with clinical, cerebrospinal fluid (CSF) and neuroradiological characteristics in relapsing‐remitting MS patients naїve to disease‐modifying therapies.

**Methods:** A retrospective sample of 166 RRMS patients and 30 controls was analyzed. All subjects underwent blood sampling, lumbar puncture, and 3T brain and spinal MRI. Patients were divided into two groups: BBBD– and BBBD+, with BBBD defined by QAlb>QAlbLim. CSF was examined for NfL and YKL‐40. The analysis included the EDSS score at baseline and during follow‐up (maximum of 8 years), brain volumes, and cerebral lesion volume. Spinal involvement was qualitatively characterized.

**Results:** Patients had higher QAlb values, and BBBD was detected in 38 patients (22.89%) and none of the controls. The BBBD+ group had a higher proportion of men. The EDSS score was higher in pBBBD+ at baseline and during follow‐up. No significant differences in clinical progression or occurrence of PIRA were found. CSF concentrations of NfL and YKL‐40 correlated positively with QAlb (*r* = 0.302, *r* = 0.237, p<0.005). pBBBD+ had a higher lesion load and white matter volume, no other significant differences were found in brain volumetrics or contrast enhancement.

**Figure:**
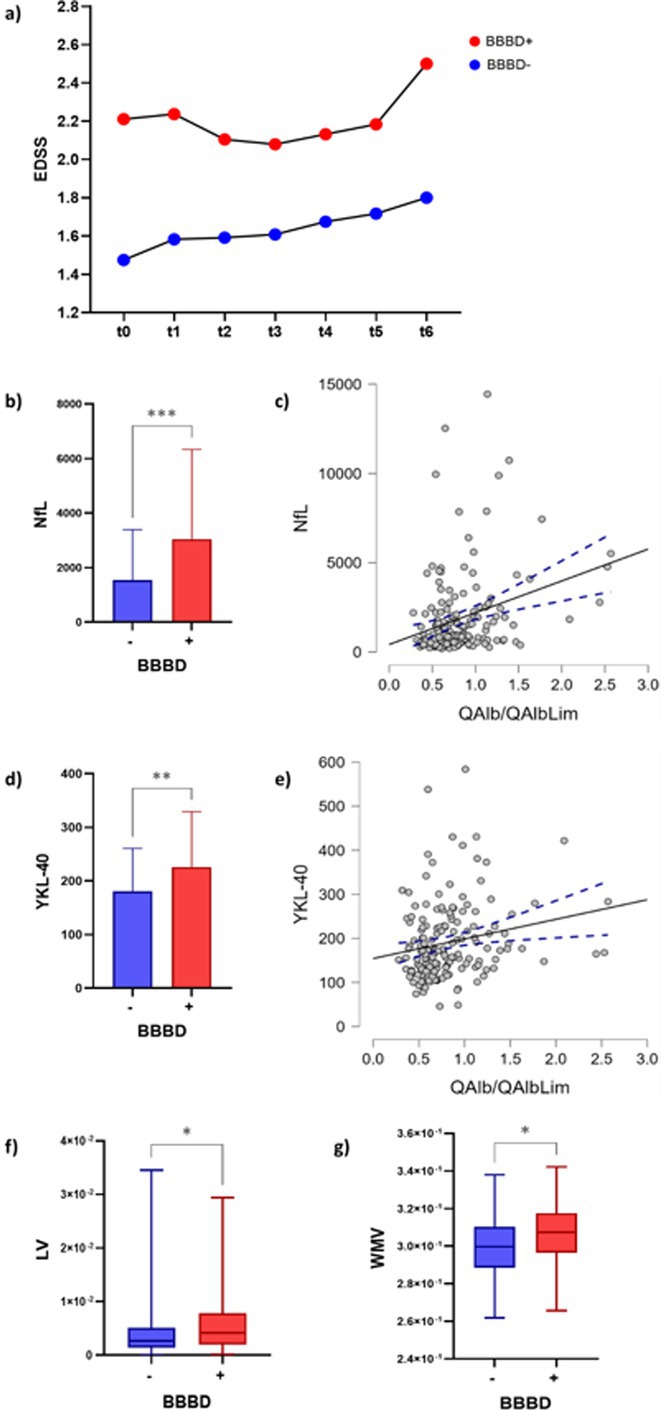
**FIGURE 1** EDSS was higher in pBBBD+ (a). CSF concentrations of NfL (b) and YKL‐40 (d), brain lesion volume (f), and white matter volume (g) were higher in pBBBD+. QAlb positively correlated with CSF concentrations of NfL (c) and YKL‐40 (e).

**Conclusion:** BBBD correlates with a higher neuroinflammatory burden, defined by brain lesion volume and CSF biomarkers, and is associated with worse clinical impairment.

**Disclosure:** Nothing to disclose.

## EPO‐284

### The volume of choroid plexus in MS: correlation with QAlb and clinical, CSF, MRI signature in naïve patients

#### F. De Napoli^1^; V. Mauceri ^1^; A. Miscioscia^1^; E. Barbuti^2^; A. Bertoldo^3^; E. Silvestri^3^; A. Marin^4^; P. Perini^1^; F. Rinaldi^1^; P. Gallo^1^; M. Puthenparampil^1^

##### ^1^Department of Neurosciences, University of Padova, Padua, Italy; ^2^Ospedale Sant'Andrea, University La Sapienza, Rome, Italy; ^3^Bioingeneer, Department of Information Engineering, University of Padova, Padua, Italy; ^4^Department of Biomedical Sciences, University of Padova, Padua, Italy

**Background and aims:** An emerging factor in MS is the alteration of the blood‐cerebrospinal fluid barrier (BCSB), which seems to play a significant role in the early stages of MS. The choroid plexuses (ChP) volume is considered a marker of BCSB activation and correlates with disease severity. This study aims to evaluate whether the volume of the ChP correlates with clinical, cerebrospinal fluid (CSF) and neuroradiological characteristics in relapsing‐remitting MS patients naїve to disease‐modifying therapies.

**Methods:** A retrospective sample of 10 controls and 50 MSRR patients was analyzed. All subjects underwent blood sampling, lumbar puncture, and 3T brain MRI. CSF was examined for QAlb (AlbL/AlbS), oligoclonal bands, NfL, and YKL‐40. The analysis included the EDSS score at baseline, brain volumes, and cerebral lesion volume. The volume of the ChP was obtained from manual segmentation at the level of the lateral ventricles in 3D‐T1 sequences. All brain volumes were normalized for total intracranial volume.

**Figure:**
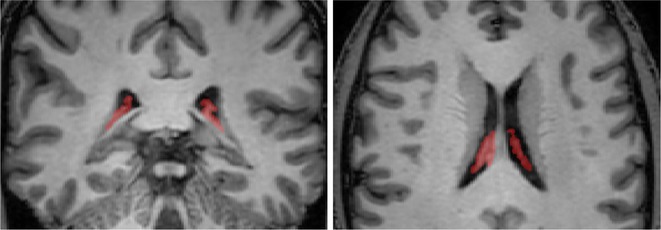
**FIGURE 1** Images of the segmentation process of the choroid plexuses in an RRMS patient, coronal (a) and axial (b) sections.

**Results:** The ChP volume was higher in patients compared to controls (p=0.035) and in MS patients it correlated positively with QAlb (r=0.338, p=0.016) and negatively with total brain volume, grey matter volume, and white matter volume (r=‐0.379, r=‐0.283, r=‐0.353, p<0.05). No correlation was detected with the other variables under analysis.

**Figure:**
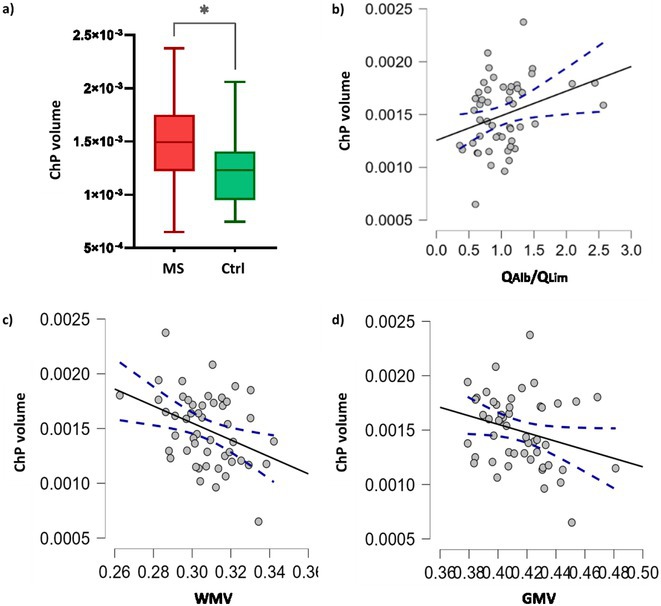
**FIGURE 2** The volume of the ChP was higher in MS patients compared to controls (a) and positively correlated with the QAlb (b). The volume of ChP negatively correlated with the white matter volume (WMV, c) and with the gray matter volume (GMV, d).

**Conclusion:** The volume of the choroid plexuses correlates with disease progression defined by the degree of brain atrophy.

**Disclosure:** Nothing to disclose.

## EPO‐285

### EEG microstate dynamics as biomarkers for neural network dysfunction in multiple sclerosis

#### G. Leodori; M. Mancuso; D. Maccarrone; A. Collura; F. Satriano; M. Fratino; M. Altieri; D. Belvisi; G. Ferrazzano; A. Conte

##### Department of Human Neurosciences, Sapienza University of Rome, Rome, Italy

**Background and aims:** Multiple Sclerosis (MS) progression involves brain network dysfunctions, but assessment tools are limited. EEG microstates, representing transient stable brain activity, provide a non‐invasive approach to investigate brain network dynamics. This study evaluated microstate metrics to differentiate healthy volunteers (HV) from MS patients and explore their associations with cognitive impairment (CI).

**Methods:** We compared 45 HVs to 46 MS patients and 57 CI to 31 non‐CI MS patients.

**Results:** TANOVA revealed no significant topographical differences between HV and MS. MS patients had significantly higher Class B explained variance (ExpVar), occurrence, and coverage but shorter durations for Classes C, F, and G. ExpVar of class B and D, Total ExpVar, and class F occurrence were the most relevant features for distinguishing groups, achieving 76.9% classification accuracy (sensitivity 73.9%, specificity 80%). We found significant topographical differences in Classes C, F, and G between CI and non‐CI patients. CI patients demonstrated significantly lower Class F ExpVar, duration, occurrence, and coverage. Class F ExpVar emerged as the sole predictor of cognitive impairment, with 64.8% classification accuracy (sensitivity 68.4%, specificity 58.1%). EDSS significantly positively correlated with Class B ExpVar, Occurrence, and Coverage, controlling for age and education.

**Figure:**
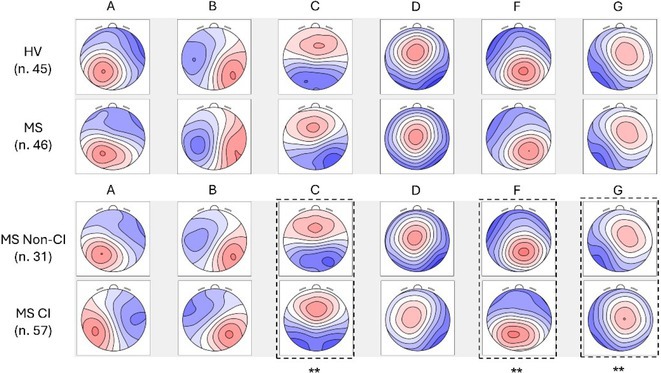
**FIGURE 1** Topographical maps of microstate classes (A–G) for healthy volunteers (HV), multiple sclerosis (MS) patients, and MS subgroups with (CI) and without (non‐CI) cognitive impairment. **: significant topographical differences, as tested by TANOVA (p < 0.01).

**Figure:**
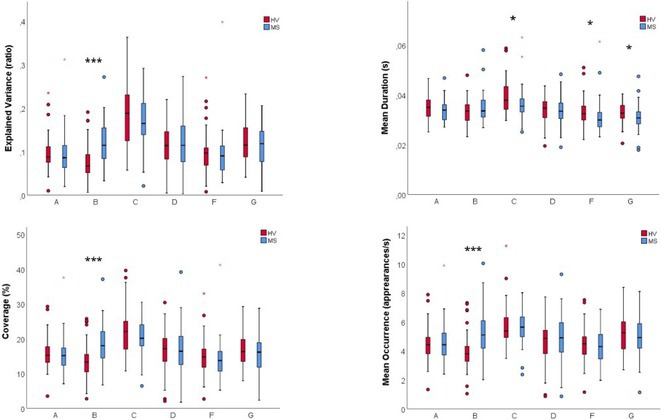
**FIGURE 2** Boxplots comparing microstate metrics across classes between HV (red) and MS patients (blu). Significant differences are indicated: ***p < 0.001, **p < 0.01, *p < 0.05.

**Figure:**
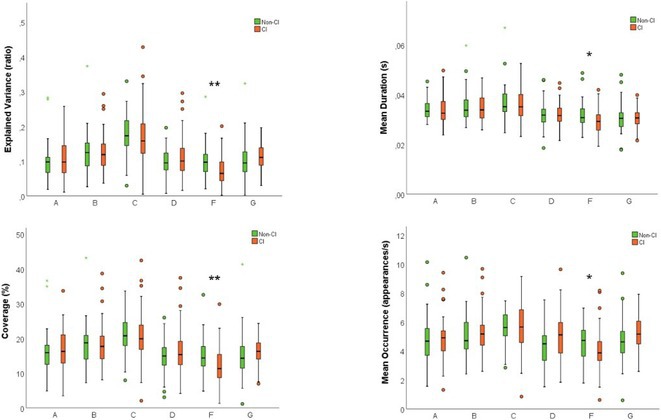
**FIGURE 3** Boxplots comparing microstate metrics across classes between Non‐CI (green) and CI (orange) MS patients. Significant differences are indicated: **p < 0.01, *p < 0.05.

**Conclusion:** Microstates reveal increased visual network (B) activity linked to disability and strong MS‐HC discrimination, with reduced stability in salience/DMN (C/F) and sensorimotor (G) networks. Salience network (F) hypo‐representation strongly associates with cognitive impairment. Microstates hold promise as biomarkers for MS progression and cognitive dysfunction, warranting longitudinal validation.

**Disclosure:** None

## EPO‐286

### First insights on the ocrelizumab route administration switch from IV to SC in MS: data from CONFIDENCE and trotzMS

#### M. Buttmann ^1^; S. Meuth^2^; S. Schmidt^3^; M. Weber^4^; S. Walter^5^; C. Ankirchner^5^; G. Ferreira^6^; S. Hieke‐Schulz^5^; J. Leemhuis^5^; T. Ziemssen^7^

##### ^1^Caritas‐Krankenhaus, Bad Mergentheim, Germany; ^2^Department of Neurology, University Clinic Düsseldorf, Heinrich‐Heine‐University Düsseldorf, Düsseldorf, Germany; ^3^Health Centre, St Johannes Hospital, Bonn, Germany; ^4^Institute of Neuropathology, Department of Neurology, University Medicine Göttingen, Göttingen, Germany; Fraunhofer‐Institute for Translational Medicine and Pharmacology ITMP, Göttingen, Germany; ^5^Roche Pharma AG, Grenzach‐Wyhlen, Germany; ^6^F. Hoffmann‐La Roche AG, Basel, Switzerland; ^7^Center of Clinical Neuroscience, Neurological Clinic, Carl Gustav Carus University Clinic, University of Technology, Dresden, Germany

**Background and aims:** The phase III OCARINA II study demonstrated that subcutaneous (SC) ocrelizumab (OCR) had a non‐inferior pharmacokinetic area under the curve (weeks 1–12) compared to intravenous (IV) OCR in people with relapsing (pwRMS) and primary progressive multiple sclerosis (pwPPMS). The SC formulation may offer a more convenient route of administration. We present preliminary real‐world experience with this switch.

**Methods:** We included pwMS from the German non‐interventional post‐authorization study CONFIDENCE (ML39632, EUPAS22951) who switched from OCR IV to SC. The incidence of adverse events (AEs), serious AEs (SAEs) and reasons for switching were collected. Additionally, we included pwMS from the trotzMS patient‐support program who switched from OCR IV to SC or newly started OCR SC and analyzed satisfaction and convenience using the Treatment Administration Satisfaction Questionnaire (TASQ).

**Results:** In CONFIDENCE, 31 pwMS switched from OCR IV to SC (RMS: n=24; PPMS: n=7) between 22/07 and 11/10/2024. One person experienced an AE (1/31; 3.2%), classified as urinary tract infection, which occurred in the PPMS group and was considered unrelated to OCR. No SAEs were reported. Reasons for switching included patient wish (16/31, 51.6%), physician wish (capacity reasons: 9/31, 29.0%; patient compliance: 1/31, 3.2%) and were missing for 5/31 pwMS (16.1%). Baseline characteristics and TASQ results of pwMS who switched from OCR IV to SC or newly starting OCR SC will be presented.

**Conclusion:** The preliminary real‐world experience of initiating/switching to OCR SC highlights the importance of treatment convenience for pwMS and showed no new safety signals after switching from OCR IV.

**Disclosure:** MB: Honoraria/travel: Biogen, BMS, Das Fortbildungskolleg, Florian Schmitz Kommunikation, Janssen, Merck, Novartis, RG Ärztefortbildung, Roche, Sandoz, Sanofi, Teva, Viatris SGM: Honoraria/travel: Academy2, Argenx, AstraZeneca, Bayer, BioNtech, Celgene, Datamed, Desitin, Diaplan, DIU, DPmed, Gen Medicine&Healthcare, IGES, Impulze GmbH, KWMedipoint, MedDay, Medmile, MICE, Mylan, Neuraxpharm, Neuropoint, OxfordPharmaGenesis, QuintilesIMS, Sanofi, Springer, STADA, Chugai, UCB, Viatris, Wings for Life int, Xcenda;research:BMBF, BfR, DFG, EKFS, G‐BA, Hertie Fdn, IZKF, DGN, Ministry of Culture&Science NRW, Daimler&Benz Fdn, dmsg, Peek&Cloppenburg Fdn, Hempel Fdn, German Alzheimer Society, Bayer, DGM, FME, GFFU, HERZ Burgdorf; honoraria/travel/research: Alexion, Almirall, Amicus, Argenx, BGP, Biogen, BMS, Demecan, Diamed, Genzyme, Hexal, Janssen, MerckSerono, Novartis, NovoNordisk, ONOPharma, Roche, Teva SS: Advisory Boards/honoraria/travel: F Hoffmann‐La Roche, Novartis, MerckSerono, Bayer, Biogen, Genzyme, Teva MSW: Research: DFG (WE3547/5‐1, WE3547/7‐1, SFBTRR274), Novartis, TEVA, Biogen, Roche, Merck, Uniklinik Göttingen(ProFutura);honoraria/travel:Biogen, MerckSerono, Novartis, Roche, TEVA, Bayer, Genzyme; editor: PLoSOne Employees: SW, CA, JL, SHS, Roche Pharma AG; GF, F Hoffmann‐La Roche AG JL: F Hoffmann‐La Roche AG shareholder TZ: Personal: Biogen, Roche, Merck, TEVA, NovoNordisk, Neuraxpharm, BMS, Novartis, Sanofi, Sandoz, Viatris; research: Genzyme, Novartis, Roche, Sanofi, Teva, Neuraxpharm.

## EPO‐287

### Self‐report of bladder issues in the UK MS Register

#### E. Craig^1^; R. Nicholas^2^; J. Rodgers^1^; D. Marcus^3^; R. Middleton ^1^

##### ^1^Disease Registers Group, FMHLS, Swansea University, Swansea, UK; ^2^Department of Brain Sciences, Imperial College London, UK; ^3^Department of Surgery & Cancer, Imperial College London, UK

**Background and aims:** The United Kingdom Multiple Sclerosis Register (UKMSR) has collected regular Patient Reported Outcomes and Clinical data from people with MS (pwMS) and clinicians since 2011. Following participant co‐creation we included more specific instrumentation related to bladder. We deployed a modified version of the PROMIS Bladder Short Form (mPBSF) and carried out linkage with existing demographics, epidemiology and most recent normalised Multiple Sclerosis Impact Scale motor component (MSIS29‐motor). Aim Assess the response and impact of bladder issues on the UKMSR population.

**Methods:** We emailed the active population of the UKMSR about the availability of the mPBSF. Standard 2 question instrument with 7 potential responses, expanded to include data about catheterisation, cystitis/Urinary Tract Infection (UTI), and treatments. Availability was 14/12/2024‐14/01/2025. We carried out logistic regression modelling on the cohort.

**Results:** 3,011 pwMS completed the instrument (52% RMS, 43% PMS, 5% Other MS). 801 had a UTI in the last 12 months (36%) With 57% having had a UTI treated with antibiotics. PMS patients were more likely to have a permanent indwelling catheter (9.3%) and higher disability (MSIS‐motor 60(±21)). Controlling for age MS type was significant (p<0.0001) in likelihood of having UTI/cystitis. There were significant differences (p<0.001) between PMS and RMS populations who had Severe or Moderate need to pass urine, and felt they had not completely emptied their bladders in the last 7 days.

**Conclusion:** In this community based cohort bladder problems and cystitis/UTI are major issues for MS. Given the impact that UTIs can have on outcome, more proactive management should be considered.

**Disclosure:** Nothing to disclose.

## EPO‐288

### Optimizing rapid inflammation control and risk management in highly active multiple sclerosis: The role of natalizumab

#### T. Sirito ^1^; C. Lapucci^2^; M. Cellerino^1^; V. Boccia^1^; N. Cavalli^1^; S. Al Qudsi^1^; A. Laroni^1^; A. Uccelli^1^; E. Capello^2^; M. Inglese^1^; G. Boffa^1^

##### ^1^Department of Neuroscience, Rehabilitation, Ophthalmology, Genetics, Maternal and Child Health (DiNOGMI), University of Genoa, Genoa, Italy; ^2^IRCCS Ospedale Policlinico San Martino, Genoa, Italy

**Background and aims:** The rapid initiation of disease‐modifying therapies (DMTs) in patients with Highly Active Multiple Sclerosis (HAMS) facilitates prompt suppression of inflammatory activity. However, this approach requires a careful balance between efficacy and safety, particularly when starting continuous high‐efficacy DMTs. The objectives of our study were to evaluate whether the early initiation of natalizumab (NTZ) in treatment‐naïve HAMS patients allows for a rapid suppression of inflammatory activity while allowing for vaccination for long term risk minimization. We assessed the risks of disease reactivation and the occurrence of progressive multifocal leukoencephalopathy (PML) after transitioning from NTZ to other treatments.

**Methods:** Baseline clinical, demographic, and MRI data were collected. Disease activity and safety outcomes were monitored throughout the follow‐up period.

**Results:** A total of 102 treatment‐naïve HAMS patients were included, 43 of whom were anti‐JCV positive at treatment initiation. All patients underwent a tailored immunization program (including live attenuated vaccines) during NTZ therapy without adverse events. During NTZ treatment, only 10 patients (9.8%) experienced subtle disease activity. 48 patients were switched to other therapies during the follow‐up with an average wash‐out period of 61 days. Of these patients, in only 2 cases was observed MRI activity near the time of the switch. No infection‐related adverse events, including PML, were reported.

**Conclusion:** The rapid initiation of NTZ in treatment‐naïve HAMS patients achieves robust and timely suppression of inflammatory activity, while also enabling safe vaccination protocols. This strategy offers a valuable therapeutic window for managing highly active disease, minimizing the risks of delayed treatment and infection‐related complications.

**Disclosure:** GB received personal compensations from Novartis, Sanofi Genzyme, Roche, BMS and Merck, unrelated to the present work. CL received travel grants from Roche, Merck, Sanofi and honoraria for speaking from Novartis, Roche, Merck, Horizon and BMS. MC received personal compensations from Novartis, Sanofi Genzyme, Teva and consulting fees from Zambon. MI received grants NIH, NMSS, FISM; received fees for consultation from BMS; Janssen, Roche, Genzyme, Merck, Biogen and Novartis. None of these personal compensations were related to this work. AL received fees for consultation from Roche, Genzyme, Merck, Biogen, Novartis, Bristol‐Myers Squibb TS, NC, SA, AU, VDB and EC have nothing to disclose.

## EPO‐289

### Fist‐Palm Test (FiPaT): a novel bedside test to screen for cognitive impairment in Multiple Sclerosis

#### U. De Marca ^1^; S. Cuoco^1^; S. Scannapieco^2^; F. Di Filippo^1^; M. Consalvo^1^; C. Giordano^1^; M. Rotolo^1^; A. Rienzo^1^; A. D'Amico^1^; M. Pellecchia^1^; M. Amboni^1^; R. Capuano^2^; M. Di Gregorio^2^; P. Barone^1^

##### ^1^Department of Medicine, Surgery and Dentistry “Scuola Medica Salernitana”, University of Salerno; ^2^Neurology Clinic, Medical Sciences Department, AOU San Giovanni di Dio e Ruggi d’Aragona, Salerno

**Background and aims:** Fist‐Palm Test(FiPaT) is a novel non‐verbalmotor task, able to screen for global cognitive status and to predict cognitive impairment. The aim of our study was to evaluate if FiPat could screen for cognitive status in patients with Multiple Sclerosis(pwMS).

**Methods:** One‐hundred‐eleven pwMS with mild disability(EDSS<2) and 28 age, sex and education matched Healthy‐Subjects(HS) underwent: Neurological assessment with EDSS and nine‐hole‐peg test(9HPT), FiPaT (defined altered if final score was>=1); Brief Repeatable Battery of Neuropsychological tests

**Results:** PwMS were more cognitive impaired than HS (28.8% pwMS vs 0% HS, p=0.001). FiPaT scores were higher in pwMS than HS (mean 0.69 vs 0.21, p=0.03); FiPaT was impaired in 30.6% pwMS and in 14.3% HS (p=0.08). PwMS with altered FiPaT were older (46 vs 39.4 years old, p=0.01) and showed higher times at dominant hand 9HPT(9HPT‐DH) (23.2 vs 21.1, p=0.005) than pwMS with normal FiPaT, whereas the two PwMS groups displayed no differences in gender, EDSS, disease duration, treatment type, non‐dominant hand 9HPT(9HPT‐NDH). Symbol digit modality Test(SDMT) (mean 0.24 vs ‐0.46, p<0.001); Selective–Reminding‐Test(SRT) Consistent Long‐Term‐Retrieval(SRT‐CLTR) (‐.51 vs ‐1.31, p=0.002), SRT‐Long‐Term‐Storage (SRT‐LTS)(‐.64 vs ‐1.17, p=0.03), SRT delayed(SRT‐D)(‐.52 vs ‐1.11, p=0.02) 10/36 Spatial Recall Test (SPART) (‐.25 vs ‐.88, p= 0.002) were significantly lower in pwMS with altered FiPaT. In pwMS, FiPaT was a predictor of SRT‐CLTR (FiPaT p=0.008; beta‐.27) and SPART (FiPaT p=0.002; beta‐.3), whereas FiPaT and 9HPT‐DH were predictors of SDMT (FiPaT p=0.002; beta ‐.3; 9HPT‐DH p0.004, beta ‐.24) independently from age, education, disease duration, EDSS, 9HPT‐NDH. ROC analysis, to evaluate the accuracy of FiPaT in identifying cognitive impairment in MS, showed an Area Under the Curve of 0.59 (95% conf. int. 0.49‐0.69) whit a sensitivity of 43.7% and specificity of 74.4%.

**Conclusion:** FiPaT could be a quick tool to screen cognitive status in pwMS.

**Disclosure:** Nothing to disclose.

## EPO‐290

### Baseline EDSS and age predict progression risk independently of relapse/MRI activity in natalizumab‐treated MS patients

#### V. Mauceri ^1^; M. Puthenparampil^1^; M. Passamonti^1^; M. Rozzi^1^; E. Basii^1^; F. Rinaldi^2^; M. Nosadini^3^; S. Sartori^3^; P. Gallo^1^; P. Perini^2^

##### ^1^Department of Neurosciences, University of Padua, Padua, Italy; ^2^Multiple Sclerosis Centre, Azienda Ospedaliera di Padova, Padua, Italy; ^3^Paediatric Neurology and Neurophysiology Unit, Department of Women's and Children's Health, University Hospital of Padova, Italy

**Background and aims:** Together with clinical relapse, Progression independent of relapse and MRI activity (PIRMA) is the main driver of disability accumulation in patients with MS (pwMS) and NTZ pwMS. The effect of Natalizumab (NTZ) on the risk of PIRMA is still unclear.

**Methods:** In this retrospective, longitudinal observational study, we included 288 NTZ‐treated pwMS. During NTZ therapy, all patients performed MRI and clinical evaluation (with EDSS) every 6 months. We defined the Progression independent of relapse and MRI activity (PIRMA) as patients with PIRA conditions and in absence of any evidence of M RI activity. Finally, sustained PIRMA required the persistence of the increased disability for 12 months.

**Results:** At the end of their follow‐up, 79 patients developed PIRA. Cox regression analysis demonstrated that both EDSS and age at baseline strongly predicted PIRMA event (H.R.: 1.770, p<0.001, and H.R.: 1.028, p=0.014 respectively). ROC analysis identified a cut off in the EDSS score of 4.0 (AUC 0.7332, p<0.0001) and survival analysis confirmed an increased risk of PIRMA in patients with high baseline EDSS value than in patients with low (log rank p<0.0001). ROC curve (AUC 0.6609, p<0.0001) identified a cut‐off of 42.5 yo to predict PIRMA and survival analysis revealed a higher risk of PIRMA in elder patients compared to younger (H.R. 2.910, 95% IC 1.688 – 5.020, p<0.0001).

**Figure:**
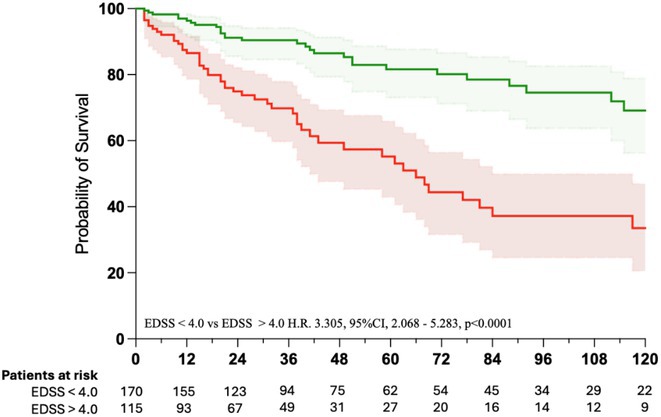
**FIGURE 1** The effect of EDSS on the risk of PIRMA

**Figure:**
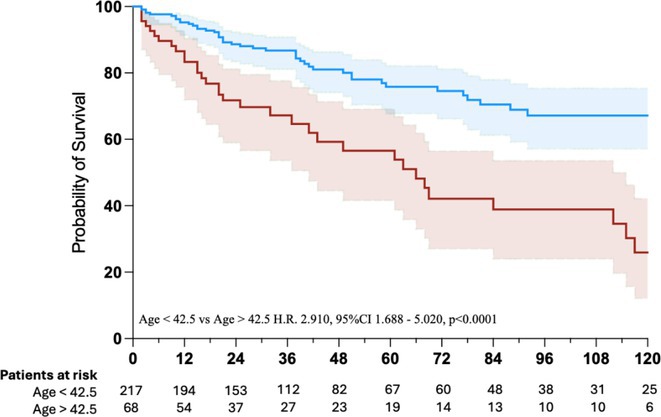
**FIGURE 2** The effect of age on the risk of PIRMA

**Conclusion:** The risk of sustained PIRMA in NTZ‐treated pwMS associated with baseline EDSS and age, supporting the early use of NTZ.

**Disclosure:** M.Pu., report grants from Almirall, Teva, Sanofi Genzyme, Merck Serono, Biogen Italy, Novartis; consultancy for Novartis, Biogen Italy, Sanofi Genzyme; board membership Sanofi Genzyme, Novartis, Biogen Italy. VAM, MP, MR, EB, MN and SS have nothing to disclose. RF report grants from Almirall, Teva, Sanofi Genzyme, Merck Serono, Biogen Italy, Novartis; consultancy for Novartis, Biogen Italy, Sanofi Genzyme. P.G. reports grant from Almirall, Teva, Sanofi Genzyme, Merck Serono, Biogen Italy, Novartis, Roche, Bristol Myers Squibb; consultancy for Novartis, Biogen Italy, Sanofi Genzyme, Roche, Bristol Myers Squibb; board membership Sanofi Genzyme, Novartis, Biogen Italy, Roche, Merck Serono, Bristol Myers Squibb. P.P. reports grants from Almirall, Teva, Sanofi Genzyme, Merck Serono, Biogen Italy, Novartis, Roche; consultancy for Novartis, Biogen Italy, Sanofi Genzyme, Roche.

## EPO‐291

### The clinical relevance of Hyper‐Reflective foci in the inner retina at the time of the diagnosis of Multiple Sclerosis

#### V. Mauceri ^1^; M. Puthenparampil^1^; M. Pengo^1^; E. Basili^1^; T. Torresin^3^; F. De Napoli^1^; P. Perini^2^; F. Rinaldi^2^; E. Pilotto^3^; E. Midena^3^; P. Gallo^1^

##### ^1^Department of Neuroscience, Università degli Studi di Padova, Padova, Italy; ^2^Day Hospital and Advanced Therapy in Neurological Disorders, Neurology Clinic, Azienda Ospedaliera di Padova, Padova, Italy; ^3^Ophthalmology Clinic, Azienda Ospedaliera di Padova, Padova, Italy

**Background and aims:** HyperReflective foci (HRF) increased in the inner retina (IR) of in patients with Multiple Sclerosis (pwMS). Their clinical prognostic relevance (marker of acute rather than chronic inflammation) is still unclear. The main objective consisted into the evaluation of the risk of disease activity based on HRS count at baseline.

**Methods:** Fifty‐seven pwMS were included in this retrospective, cohort single‐centre study. All patients were enrolled at clinical onset and were disease free. No evidence of optic nerve inflammation was acquired by means of clinical, radiological and OCT parameters. Patient were divided at baseline based on the MS treatment indicated by their neurologist as plat‐therapy pwMS (PTpwMS) and as High efficacy therapy pwMS (HETpwMS). Then, all patients that started a plat‐therapy (PT)were followed up for at least 24 months: the main outcome was the time to switch for lack of efficacy on inflammatory (clinical relapse and MRI new/enlarging/gadolinium‐enhancing lesion) outcomes. HRF count was expressed as the sum of both eyes in GCIPL, INL and IR (GCIPL+INL).

**Results:** At baseline HETPwMS had increased HRS count in all IR layer (HRF‐GCIPL: 19.0±5.3 vs 25.8±4.4; HRF‐INL 37.1±9.4 vs 50.2±9.9, HRF‐IR: 56.1±12.3 vs 76.0±12.2 all p<0.001) compared to PTpwMS. ROC analysis identified a best cut‐off in the IR‐HRS (75 foci), whose application off on PT pwMS identified an earlier switch (HR 6.5, 95% IC 1.5‐28.8, p=0.007).

**Figure:**
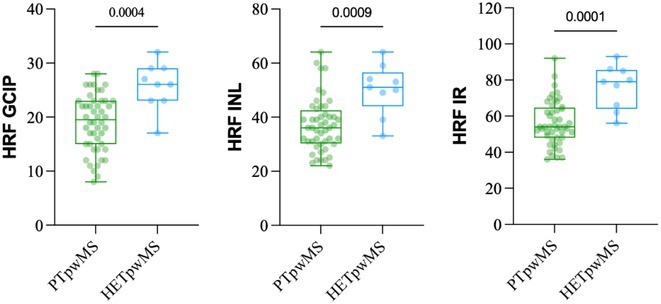
**FIGURE 1** HRF count in GCIPL, INL and IR.

**Figure:**
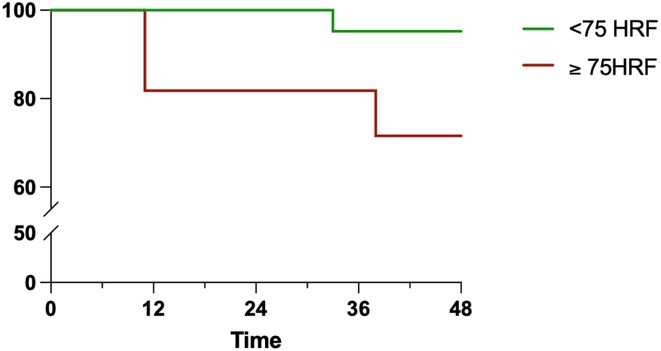
**FIGURE 2** Survival analysis on PTpwMS.

**Conclusion:** HRS might be a useful marker to predict the risk of acute demyelination in MS and might give clues to Neurologist for choosing HET earlier

**Disclosure:** M.Pu., report travel grants, consultancy, and board membership from Almirall, Teva, Sanofi Genzyme, Merck Serono, Biogen Italy, Novartis, Bristol Myers Squibb, Janssen, and Alexion. M.Pe, E.B., E.P., and E.M. have nothing to disclose. V.A.M reports travel grants from Sanofi Genzyme, Biogen, and Viatris. P.P. reports grants from Almirall, Teva, Sanofi Genzyme, Merck Serono, Biogen Italy, Novartis, Roche, Alexion, Janssen, Brystol Mayer Squibb; consultancy for Novartis, Biogen Italy, Sanofi Genzyme, Roche, Janssen, Brystol Mayer Squibb. RF report grants from Almirall, Teva, Sanofi Genzyme, Merck Serono, Biogen Italy, Novartis, consultancy for Novartis, Biogen Italy, Sanofi Genzyme. P.G. reports grant, consultancy, and board membership for Almirall, Teva, Sanofi Genzyme, Merck Serono, Biogen Italy, Novartis, Roche, Bristol Myers Squibb, Janssen, and Alexion.

## EPO‐292

### Pain in idiopathic longitudinally extensive transverse myelitis compared to neuromyelitis optica spectrum disorder

#### Y. Jin ^1^; D. Seo^2^; H. Yoon^1^; I. Jang^2^; L. Choi^2^; J. Kim^2^; W. Shin^2^; H. Lee^2^; S. Kim^2^; H. Jung^2^; J. Kim^2^; H. Kim^2^; Y. Lim^2^; E. Lee^2^

##### ^1^University of Ulsan College of Medicine, Seoul, Republic of Korea; ^2^Department of Neurology, Asan Medical Center, University of Ulsan, Seoul, Republic of Korea

**Background and aims:** Chronic pain is a common consequence of longitudinally extensive transverse myelitis (LETM), potentially exacerbated by inflammation. While pain in seropositive neuromyelitis optica spectrum disorder‐associated TM (NMOSD‐TM) has been frequently studied, less attention has been given to idiopathic LETM (I‐LETM), which lacks autoantibodies. We aimed to compare the prevalence and characteristics of chronic pain in I‐LETM and NMOSD‐TM.

**Methods:** We prospectively enrolled patients with I‐LETM or seropositive NMOSD‐TM (anti‐aquaporin‐4 antibodies) who were in the chronic phase, at least 3 months after their last clinical attack. LETM was defined as transverse myelitis (TM) affecting ≥3 spinal segments, with I‐LETM characterized by the absence of aquaporin‐4 and myelin oligodendrocyte glycoprotein antibodies. Pain was evaluated using the Pain DETECT Questionnaire (PDQ) and Short Form‐Brief Pain Inventory (SF‐BPI), while quality of life was assessed using EuroQoL‐5D (EQ‐5D) at baseline and follow‐up (6–12 months).

**Results:** Among 54 patients (I‐LETM: 9, NMOSD‐TM: 45; median age: 57 years), I‐LETM patients had fewer affected spinal segments (median, 4 vs. 10, p <0.001) and less cervical cord involvement (44% vs. 87%, p=0.012). Chronic pain was reported in all I‐LETM patients and 87% of NMOSD‐TM patients. Pain severity and neuropathic pain prevalence were comparable, though numbness was more severe in I‐LETM (median score: 4 vs. 0, p=0.027). Pain severity negatively correlated with quality of life and remained stable at follow‐up.

**Conclusion:** Chronic pain is highly prevalent and severe in both groups, suggesting that the presence of autoantibodies does not significantly influence LETM pain characteristics. Pain management should be prioritized for all LETM patients.

**Disclosure:** Nothing to disclose.

## Muscle and Neuromuscular Junction Disorder 2

## EPO‐293

### Understanding muscle biopsy pain: What to expect during and after

#### B. Labella ^1^; G. Brochier^1^; M. Beuvin^1^; A. Chanut^1^; A. Madelaine^1^; C. Labasse^1^; E. Lacene^1^; S. Leonard‐Louis^2^; G. Bassez^3^; T. Evangelista^1^

##### ^1^Neuromuscular Morphology Unit, Myology Institute, Groupe Hospitalier Universitaire Pitié‐Salpêtrière, 75013 Paris, France; ^2^Neuropathology Unit, Department of Neuropathology, Groupe Hospitalier Universitaire La Pitié‐Salpêtrière, 75013 Paris, France; ^3^Reference Center for Neuromuscular Disorders, Myology Institute, Neuropathology Unit, Department of Neuropathology, Groupe Hospitalier Universitaire La Pitié‐Salpêtrière, 75013 Paris, France

**Background and aims:** Open muscle biopsy (OBM) is a valuable diagnostic tool, but patients frequently express concerns about the surgical risks and potential discomfort. The aim of this observational study is to describe the characteristics of pain encountered during and after OBM and to assess the prognostic factors that may influence patient's pain perception.

**Methods:** Patients aged > 18 years who underwent OBM at the Pitie‐Salpetriere Hospital in Paris and provided informed consent were enrolled in the study. Clinical data and frailty assessment were collected prior to the intervention. Following OBM, patients completed a detailed questionnaire, including the numerical rating scale (NRS) for pain assessment, and the PHQ‐9 questionnaire. Follow‐up phone calls were performed at 15 and 30 days.

**Results:** Forty‐seven patients (13 males) were enrolled, with a mean pain score of 2.6 on the NRS. The most painful phase of the OBM was the sampling collection phase (24/47, 51.1%), followed by the local anesthesia phase (10/47, 21.3%). No major complications were observed in the follow‐up period, available for 36 patients. Fourteen patients (38.8%) reported mild pain (NRS 1‐3), lasting up to 48 hours (11/14), while four patients (11.1%) experienced a moderate pain (NRS 4‐6). Overall, only fourteen patients (38.8%) took an antalgic treatment after OBM, with paracetamol being the first line analgesic (12/14, 85.7%), yielding a good therapeutic response. Pre‐interventional anxiety wasn’t associated with higher pain perception during OBM.

**Conclusion:** Pain experienced during OBM is usually mild. After OBM, patients generally report little to no discomfort. Therefore, OBM is a safe and well‐tolerated procedure.

**Disclosure:** Nothing to disclose.

## EPO‐294

### Genome sequencing unveils new insights into LGMD in Tunisia: From misdiagnosis to accurate insights

#### I. Belhassen ^1^; S. Sakka^1^; P. Rodriguez Cruz^2^; M. Dammak^1^; C. Mhiri^1^

##### ^1^Neurology department Habib Bourguiba University Hospital, Sfax, Tunisia; ^2^Centro Nacional de Análisis Genómico (CNAG), Barcelona, Spain

**Background and aims:** Limb‐girdle muscular dystrophies (LGMD) are rare hereditary genetic disorders that affect the muscles of the pelvic and shoulder girdles. Due to the rarity of the disease and the phenotypic similarities between its 32 forms, achieving an accurate diagnosis of LGMD is often challenging.

**Methods:** A total of 48 patients presenting with progressive muscular weakness were included in this study. Targeted gene panel sequencing and whole exome sequencing (WES) were utilized for genetic analysis. Afterwards, Sanger sequencing and multiplex ligation‐dependent probe amplification (MLPA) were employed to validate the identified variants.

**Results:** Genome sequencing confirmed a diagnosis of dysferlinopathy (LGMDR2) for 28 patients, highlighting that this form is the most prevalent form of myopathy in Tunisia, contrary to previous assumptions that sarcoglycanopathy was more relevant. In addition, we confirmed diagnoses of LGMDR1 and LGMDR9 in 12 patients with the identification of new mutations. We also identified unreported rare forms of LGMD in Tunisia, including LGMDR11, LGMDR12, and Bethlem myopathy. Furthermore, we identified mutations that refined the diagnosis for some patients previously classified as having LGMD. Specifically, these patients carried mutations associated with McArdle disease, mitochondrial myopathy, and AMPD1 myopathy, which mimicked the phenotype of LGMD.

**Conclusion:** In conclusion, our study marks a turning point in the epidemiology of limb‐girdle muscular dystrophies (LGMD) in Tunisia, challenging the previously prevailing data in the region. By incorporating genome sequencing, we identified rare myopathies and corrected misdiagnoses, leading to more precise diagnoses and better treatment adjustments for affected patients

**Disclosure:** Nothing to disclose.

## EPO‐295

### Rapid onset of efficacy of Eculizumab in single‐center cohort of patients with refractory generalized Myasthenia Gravis

#### F. D'Anna; G. D'Alvano; V. Todisco; D. Marigliano; F. Trojsi; A. Tessitore; A. Bisecco

##### 1Department of Advanced Medical and Surgical Sciences – University of Campania “Luigi Vanvitelli”

**Background and aims:** Eculizumab is a humanized monoclonal antibody that targets complement protein C5 that has been approved in Italy for treatment of patients positive for anti‐acetylcholine receptor antibodies (AChR+) refractory generalized myasthenia gravis (gMG). The main objective of our study is to evaluate the time of efficacy onset of Eculizumab in the cohort of patients with refractory acetylcholine receptor antibody‐positive (AChR+) gMG.

**Methods:** All patients with refractory AChR+ gMG treated with eculizumab (900 mg/week for 4 weeks then 1200 mg the fifth week and then every 2 weeks) followed by our Department were included. Outcome measures were MyastheniaGravis‐Activities of Daily Living (MG‐ADL) scores, QuantitativeMyastheniaGravis (QMG) evaluations, number of exacerbations and adverse events. Data were collected before Eculizumab start (BL), 5‐weeks after (T1) and then at regular intervals of three months.

**Results:** Data were available for 6 adult patients (4F; 2M). Two patients had a history of thymoma surgically treated. The mean MG‐ADL score reduced from 7.5 at baseline to 2.6 at week 5 (p = 0.02). The mean QMG score dropped from 17.1 at baseline to 7.6 at week 5 (p = 0.004). This improvement was stationary at subsequent follow‐up. No meningococcal infections neither adverse drug reactions were reported. No patients required additional rescue therapy. One death was reported during FU and was considered unrelated to Eculizumab treatment.

**Conclusion:** This single‐center study confirm a rapid and sustained efficacy of Eculizumab in a real‐world setting in patients with refractory gMG and allow to hypothesize future clinical trials designed to evaluate the possible use of eculizumab in gMG exacerbations.

**Disclosure:** Nothing to disclose.

## EPO‐296

### Assessing efficacy and safety of gefurulimab in generalised Myasthenia gravis: Baseline characteristics from PREVAIL

#### F. Saccà ^1^; K. Gwathmey^2^; M. Masuda^3^; A. A. Habib^4^; S. Perić^5^; S. Rakhade^6^; J. Scholz^6^; S. Shang^6^; J. F. Howard^7^

##### ^1^University of Naples Federico II, Napoli, Italy; ^2^VCU Health, Richmond, USA; ^3^Tokyo Medical University, Tokyo, Japan; ^4^University of California, Irvine, USA; ^5^University Clinical Centre of Serbia & University of Belgrade, Belgrade, Serbia; ^6^Alexion, AstraZeneca Rare Disease, Boston, USA; ^7^University of North Carolina at Chapel Hill School of Medicine, Chapel Hill, USA

**Background and aims:** Complement component 5 (C5) inhibitors are effective treatments for anti‐acetylcholine receptor antibody‐positive (AChR‐Ab+) generalised myasthenia gravis (gMG). Gefurulimab (ALXN1720) is a new investigational C5 inhibitor designed for weekly subcutaneous (SC) self‐injection. The ongoing phase 3, multicentre, randomised, double‐blind, placebo‐controlled PREVAIL study is evaluating the efficacy and safety of gefurulimab in adults with AChR‐Ab+ gMG (NCT, NCT05556096; EudraCT, 2023‐508284‐77‐00). Here, we describe summary baseline characteristics of participants in the PREVAIL study.

**Methods:** Adult patients with AChR‐Ab+ gMG were randomised 1:1 to weekly SC self‐injection of gefurulimab or placebo. The study consists of an initial screening period (up to 4 weeks), a randomised controlled treatment period (26 weeks), and an open‐label extension (up to 105 weeks). Patients may continue previously prescribed allowed therapies, including immunoglobulins. The primary endpoint is change from baseline in Myasthenia Gravis Activities of Daily Living (MG‐ADL) total score at week 26. Secondary endpoints include change from baseline in Quantitative Myasthenia Gravis (QMG) total score and Myasthenia Gravis Composite (MGC) total score. Safety, pharmacokinetics, pharmacodynamics, immunogenicity, and quality of life are also assessed.

**Results:** As of 09 Dec 2024, 260 participants have been enrolled. At baseline (n=259), ~60% of participants were female and mean±SD MG‐ADL total score was 9.0±2.2. At first dose of study intervention (n=259), mean±SD age was 52.8±15.8yrs, and ~83% of patients were using any immunosuppressive therapy.

**Conclusion:** This study examines the potential of gefurulimab as an effective treatment for patients with AChR‐Ab+ gMG self‐administered once‐weekly as a SC injection. Additional baseline characteristics will be presented.

**Disclosure:** FS: speaking honoraria/ad board/consulting fees/PI‐clinical trials: Alexion, Amgen, argenx, AstraZeneca, Alexis, Biogen, Dianthus, Genpharm, Immunovant, JnJ, Leadiant, Lexeo, MedPharm, Medison, Neopharm Israel, Novartis, Prilena, Reata, RemeGen, Roche, Sandoz, Sanofi, Takeda, UCB, Zai Lab. KG: honoraria: AcademicCME, Alexion, AstraZeneca Rare Disease, Amgen, argenx, UCB. MM: honoraria/ad boards: Alexion Pharma GK, AstraZeneca Rare Disease, argenx, Asahi Kasei Medical, Hanall Biopharma, Japan Blood Products Organization, Takeda, UCB. AAH: research support: Alexion, AstraZeneca Rare Disease, argenx, Cabaletta, Genentech/Roche, Immunovant, Pfizer, Regeneron, UCB, Viela. SP: honoraria/research/travel grants/consulting fees: Adoc, Amgen, argenx, AstraZeneca, Berlin Chemie, Biogen Idec, Dianthus, Genesis, Immunabs, Kedrion, Medis, Ministry of Science of the Republic of Serbia, Mylan, Octapharma, Pfizer, Roche, Salveo, Sanofi, Swixx, Takeda, Teva Actavis, Vemax, Worwag. SR, JS, SS: Alexion, AstraZeneca Rare Disease employees; stock/stock options:AstraZeneca. JFH: research support/honoraria/consulting&nonfinancial fees: AcademicCME, Ad Scientiam, Alexion, AstraZeneca Rare Disease, Amgen, argenx, Biohaven, Biologix, Cartesian Therapeutics, CDC, CheckRare CME, CoreEvitas, Curie.bio, Medscape CME, EMD Serono, MGFA, Muscular Dystrophy Association, NIH, NMD Pharma, Novartis, PCORI, PeerView/Physicians’ Education Resource/PlatformQ CME, Regeneron, Sanofi, TG Therapeutics, Toleranzia AB, UCB, Zai Lab.

## EPO‐297

### Eculizumab as a new option for thymoma‐associated myasthenia gravis after thymectomy: A prospective case series

#### H. Wu ^1^; S. Luo^1^; J. Song^1^; C. Zhao^1^; X. Chu^2^; L. Pang^2^; L. Jin^1^; W. Jia^3^; H. Zhong^1^; R. Chen^1^; Z. Wu^4^

##### ^1^Huashan Rare Disease Center and Department of Neurology, Huashan Hospital, Shanghai Medical College, National Center for Neurological Disorders, Fudan University, Shanghai, China; ^2^Department of Cardiothoracic Surgery, Huashan Hospital, Fudan University, Shanghai, China; ^3^Department of Neurology, No.2 Affiliated Hospital, Kunming Medical University, Kunming, China; ^4^Faculty of Biology, University of Cambridge, Cambridge UK

**Background and aims:** The perioperative efficacy and safety of eculizumab in patients with thymoma‐associated myasthenia gravis (TAMG) after thymectomy have not been evaluated. This study aims to report a case series of TAMG who have eculizumab as an add‐on therapy for perioperative treatment.

**Methods:** This is a single‐centre observational prospective study. TAMG cases with thymoma burden and initiated eculizumab before thymectomy were enrolled. The MG‐activities of daily living (ADL) score, lymphocytic phenotypes, and adverse events were assessed pre‐thymectomy and post‐thymectomy.

**Results:** Seven TMG patients were finally recruited with a mean age of 53.0±14.95 years. The duration from MG onset and initiation of eculizumab to thymectomy was 7.17±6.21 months and 2.35±2.19 weeks, respectively. Upon eculizumab initiation, MG‐ADL score rapidly reduced from 9.83±6.52 to 5.50±6.38 by 1 week and 2.50±6.17 by 4 weeks. The thymectomy was performed successfully, and the patients were discharged from the hospital after recovery in 13.17±15.01 days. The percentages of CD3+CD4+Th lymphocytes in peripheral blood significantly declined from 45.14%±6.07% to 31.82%±6.77%(p<0.05), while there were no significant changes in CD3+CD8+Tc lymphocytes and CD19+B lymphocytes.

**Conclusion:** This small case series highlights the use of eculizumab in TAMG as a rapid symptom‐control treatment during the perioperative period. Future prospective cohort studies with a large sample size are expected to validate these findings, particularly for those TAMG with moderate to severe myasthenia before thymectomy.

**Disclosure:** Nothing to disclose.

## EPO‐298

### A real‐world experience with Efgartigimod for new‐onset generalized myasthenia gravis

#### C. Ma; Y. Zhu; J. Shen; R. Zhu

##### Department of Neurology, The First Affiliated Hospital of China Medical University, Shenyang, China

**Background and aims:** The real‐world experiences with efgartigimod, as reported in various studies confirmed that efgartigimod is effective across diverse subtypes of patients and can be integrated into personalized treatment strategies for myasthenia gravis (MG). But there remains a paucity of relevant evidence regarding its use in patients with new‐onset AChR antibody positive generalized MG.

**Methods:** We conducted a prospective study to evaluate the real‐world safety and efficacy of efgartigimod in 29 new‐onset AChR‐gMG patients with a three‐month follow‐up. The MG‐ADL, QMG score, dose of prednisone, laboratory data and adverse events were assessed at every follow‐up.

**Results:** At 4, 8, 12 weeks, the change in MG‐ADL score was 8.13±3.66, 7.41±4.22, and 6.37±4.67, respectively. 96% (28/29) of patients demonstrated an MG‐ADL response (ADL ≥ 2‐point) compared with baseline after one cycle and the time to response was 0.81 ± 0.53 weeks (5.67 ± 3.71 days). 52% (15/29) patients achieved MSE after one cycle, while 41% maintained MSE by 12 weeks. Moreover, 89% and 72% MG‐ADL responder was sustained for 8 and 12 consecutive weeks. Additionally, TMG patients presented worse response to efgartigimod and required to apply two infusion cycles. All patients were able to reduce their daily dose of steroids. The treatment was well tolerated with few adverse events reported.

**Conclusion:** Our study shows that efgartigimod is clinically beneficial and offers rapid symptom control for new‐onset AChR‐gMG patients. More aggressive application of efgartigimod in combination with corticosteroids may lead to a smoother therapy transition, which will further maintaining the favorable condition and improve longitudinal prognosis.

**Disclosure:** Nothing to disclose.

## EPO‐299

### Long‐term safety of rozanolixizumab treatment cycles in patients with generalised myasthenia gravis

#### J. Vissing ^1^; C. Antozzi^2^; A. Drużdż^3^; J. Grosskreutz^4^; A. Habib^5^; S. Sacconi^6^; K. Utsugisawa^7^; T. Vu^8^; F. Grimson^9^; N. Houston^10^; T. Tarancón^11^; V. Bril^12^

##### ^1^Department of Neurology, Rigshospitalet, University of Copenhagen, Copenhagen, Denmark; ^2^Neuroimmunology and Muscle Pathology Unit, Multiple Sclerosis Center, Fondazione Istituto di Ricovero e Cura a Carattere Scientifico, Istituto Nazionale Neurologico Carlo Besta, Milan, Italy; ^3^Department of Neurology, Municipal Hospital, Poznań, Poland; ^4^Precision Neurology of Neuromuscular Diseases, Department of Neurology, University of Lübeck, Lübeck, Germany; ^5^MDA ALS & Neuromuscular Center, Department of Neurology, University of California, Irvine, Orange, USA; ^6^Université Côte d’Azur, Peripheral Nervous System & Muscle Department, Pasteur 2 Hospital, Centre Hospitalier Universitaire de Nice, Nice, France; ^7^Department of Neurology, Hanamaki General Hospital, Hanamaki, Japan; ^8^Department of Neurology, University of South Florida Morsani College of Medicine, Tampa, FL, USA; ^9^UCB, Slough, UK, ^10^UCB, Dublin, Ireland, ^11^UCB, Madrid, Spain, ^12^University Health Network, Toronto, Canada

**Background and aims:** In the MycarinG study (MG0003/NCT03971422), one 6‐week cycle of rozanolixizumab was generally well tolerated and significantly improved myasthenia gravis (MG)‐specific outcomes versus placebo. After MycarinG, patients could receive additional cycles of rozanolixizumab in the open‐label extension study MG0007, which is now complete. We evaluate the long‐term safety of repeated rozanolixizumab treatment cycles in patients with generalised MG (gMG).

**Methods:** Safety data up to Cycle 13 were pooled for patients receiving >=1 rozanolixizumab cycle across MycarinG and MG0007 (NCT04650854).

**Results:** 188 patients received a total of 1094 cycles of rozanolixizumab 7mg/kg or 10mg/kg, equating to 310.25 years of exposure. Across all cycles, treatment‐emergent adverse events (TEAEs; mostly mild/moderate) occurred in 93.1% (n=175/188) of patients. Incidence varied across 13 cycles, from 37.5% (Cycle 12) to 74.5% (Cycle 1) in the 7mg/kg group and 44.4% (Cycle 12) to 88.9% (Cycle 5) in the 10mg/kg group (Table 1). Incidence of any TEAE did not increase with repeated cyclic treatment compared with Cycle 1. Most common TEAEs were headache (50.0%), diarrhoea (33.5%), COVID‐19 (21.8%) and pyrexia (20.7%). Overall incidence of infection or infestation (58.0%) and headache did not increase across cycles. Serious TEAEs occurred in 29.3% (n=55/188) of patients; events occurring in >1% of patients across all cycles were MG (9.6%), MG crisis (2.1%), COVID‐19 (1.6%), nephrolithiasis (1.1%) and pneumonia (1.1%). Four deaths occurred, all deemed unrelated to rozanolixizumab by investigators.

**Figure:**
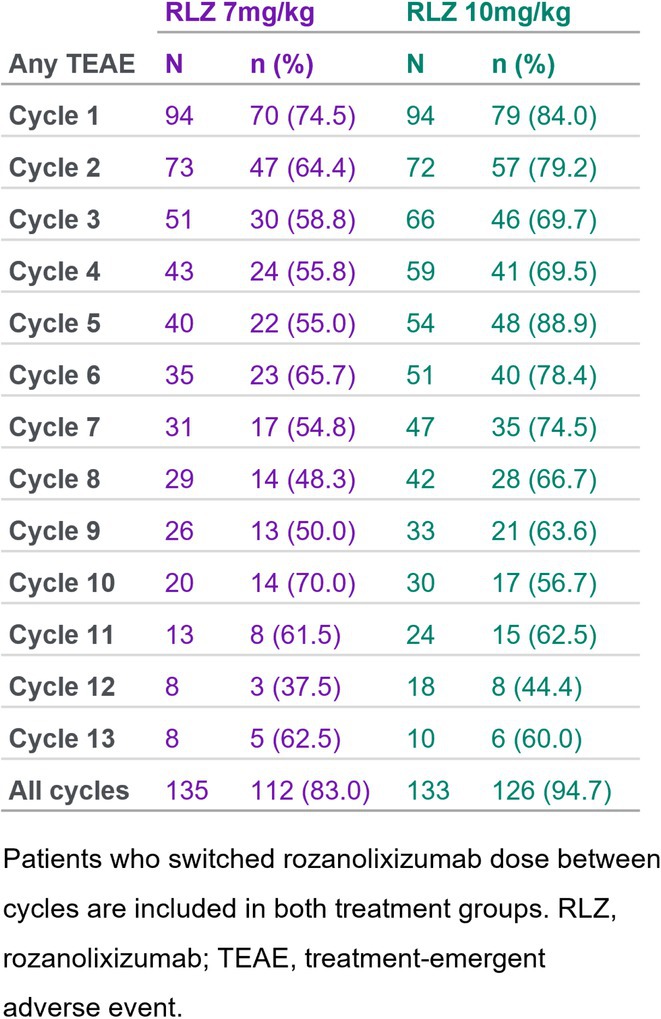
**TABLE 1** Incidence of TEAEs by treatment cycle.

**Conclusion:** Rozanolixizumab was generally well tolerated in patients with gMG with an acceptable and consistent safety profile across repeated treatment cycles.

**Disclosure:** This study was funded by UCB. Fiona Grimson, Niamh Houston and Thaïs Tarancón are employees and shareholders of UCB. Full disclosure of all industry relationships will be made during congress presentation if accepted.

## EPO‐300

### Assessment of patient‐reported outcomes from the phase 3 vivacity‐MG3 study of nipocalimab in gMG

#### E. Cortés Vicente^1^; S. Pease^2^; N. Imran^2^; K. Gandhi^2^; M. Ait‐Tihyaty^2^; I. Turkoz^3^; G. Coteur^4^; C. Gary^5^; Z. Choudhry^3^; S. Ramchandran^3^; J. Vissing ^6^

##### ^1^Unitat Patologia Neuromuscular, Servei Neurologia, Hospital Santa Creu i Sant Pau, Barcelona; ^2^Johnson & Johnson, Raritan, USA; ^3^Johnson & Johnson, Horsham, USA; ^4^Johnson & Johnson, Raritan, USA; IPATH Solutions, Wemmel, Belgium; ^5^Johnson & Johnson, Issy‐les‐Moulineaux, France; ^6^Department of Neurology, University of Copenhagen, Copenhagen, Denmark

**Background and aims:** Due to the heterogeneity of generalized myasthenia gravis (gMG), it is crucial to capture health‐related quality of life data, including treatment satisfaction for this rare condition. Examining patient‐reported outcomes (PROs) helps to better understand the overall impact of the disease and the effectiveness of treatments from the patients' perspectives. Nipocalimab+SOC demonstrated positive efficacy in Vivacity‐MG3 (NCT04951622) vs placebo+SOC in gMG. The analysis of comprehensive PRO measures from the Vivacity‐MG3 trial offers valuable insights into treatment satisfaction and overall disease status from the viewpoint of patients treated with nipocalimab+SOC vs placebo+SOC.

**Methods:** The efficacy analysis population included participants who were antibody‐positive for a gMG‐related pathogenic antibody (anti‐acetylcholine receptor [AChR], anti‐muscle‐specific tyrosine kinase [MuSK], or anti‐low density lipoprotein receptor‐related protein 4 [LRP4]). PROs were reported from week‐2 (W2) through week‐24 (W24) descriptively and included: EuroQol 5‐Dimension Visual Analogue Scale (EQ‐5D VAS), Patient Global Impression of Severity/Change‐Fatigue (PGIS/PGIC), and Treatment Satisfaction Questionnaire for Medication (TSQM‐9).

**Results:** EQ‐5D‐5L VAS mean (95% confidence interval [CI]) change‐from‐baseline scores were significantly improved for nipocalimab+SOC (11.1[7.1, 15.2]) vs placebo+SOC (1.3[‐2.2, 4.8]) by W2; improvement was sustained up to W24 (Figure). At W24, 56.5% of nipocalimab+SOC‐treated patients reported their fatigue as ‘much better’ or ‘moderately better’ since the start of study medication, a difference of 15.5% vs placebo (Table). Mean scores (95% CI) in TSQM‐9 Global Satisfaction domain at W24 were numerically higher in nipocalimab (65.7[59.4, 72.0]) vs placebo (56.1[50.1, 62.1]).

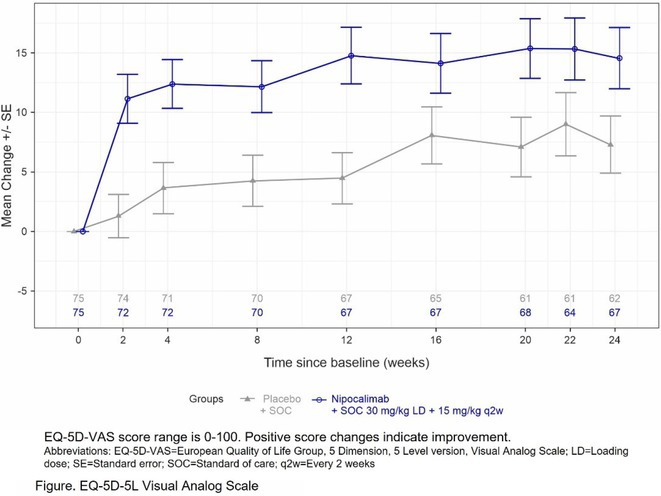

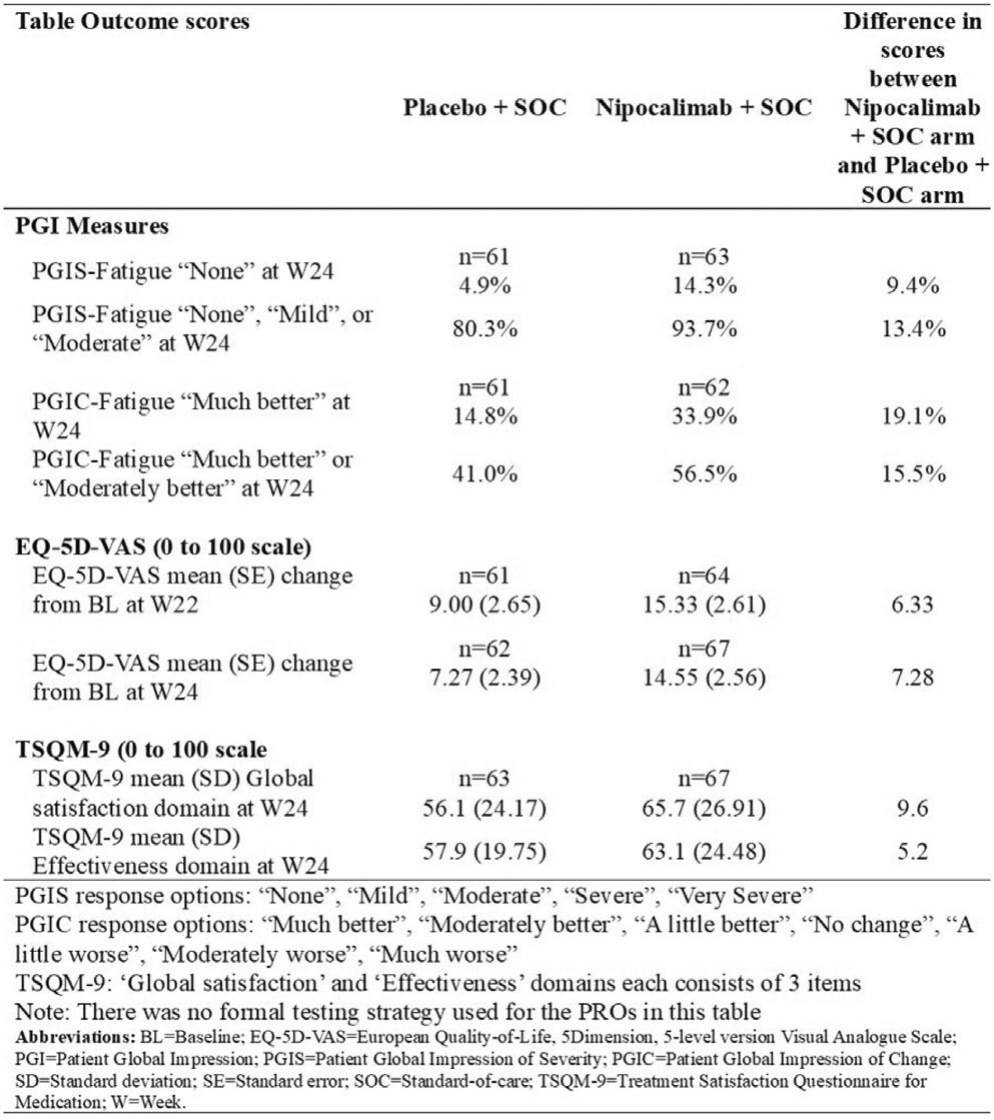

**Conclusion:** Nipocalimab‐treated patients reported numerically greater improvements on patient‐reported health status and treatment satisfaction compared with placebo‐treated patients.

**Disclosure:** This study was sponsored by Johnson & Johnson. Elena Cortés Vicente: Received consulting/advisory from Argenx BV, Alexion Pharmaceuticals Inc., Janssen Pharmaceuticals Inc., UCB Pharma SA. Sheryl Pease, Nida Imran, Kavita Gandhi, Maria Ait‐Tihyaty, Ibrahim Turkoz, Charlotte Gary, Zia Choudhry, and Sindhu Ramchandren: Employees of Johnson & Johnson, may hold stocks/stock options in Johnson & Johnson. Geoffroy Coteur: Owner of IPATH Solutions and received consultant fees from Johnson & Johnson. John Vissing: Participated in paid advisory boards for Alexion Pharmaceuticals Inc., Argenx BV, Dianthus Therapeutics, Horizon Therapeutics (now Amgen Inc.), Janssen, Regeneron, Roche, and UCB Pharma SA.

## EPO‐301

### Rehabilitation (R) and function of external respiration in outpatient myasthenia gravis (MG) patients

#### V. Haliyeuskaya; Y. Rushkevich; K. Malhina; S. Likhachev

##### Republican Scientific and Practical Center of Neurology and Neurosurgery, Minsk, Belarus.

**Background and aims:** It is important to diagnose respiratory disorders (RD) in MG that often stay unrecognized. The R‐possibilities in MG are limited and require special attitude.

**Methods:** The study was carried out in 36 outpatient MG patients (13(36%) men and 23(64%) women), 62.0[46.0;68.0] years, BMI 27.0[24.0;30.0]. All patients with generalized MG, 13(36%) with bulbar disorders and 23(64%) without, 35(97%) with the second and 1(3%) with the third MGFA class. The control group 14 patients (4(29%) men and 10(71%) women) without signs of neuromuscular pathology, 57.5[51.0;61.0] years, BMI 26.5[26.0;31.0]. Groups comparable in age (U, p=0.18), BMI (U, p=0.93), gender (χ2, p=0.61). The R‐complex included diaphragmatic breathing, exercises involving arms.

**Results:** When comparing spirometry in outpatient MG with control group significant decrease in Vital Capacity (VC) was found in MG: sitting 78,0[71,5;96,5]%/99,0[91,0;108,0]%, U, p=0.005 and lying 80,0[67,5;94,5]%/94,5[83,0;101,0]%, U, p=0.01. MG patients with low VC in the sitting position (n=21) underwent R. Significant increase was achieved in VC (72,0[69,0;78,0]%/82,0[77,0;89,0]%, W, p=0.01) and Inspiratory Reserve Volume (IRV) (1.14[1.02;1.56]l/1.65[0.96;1.93]l, W, p=0.03). Spirometry in MG patients before and after R were compared with spirometry in MG patients without R (n=35). VC and IRV before R comparable in both groups: 72,0[69,0;78,0]%/75,0[70,0;83,0]%, U, p=0.57 and 1.14[1.02;1.56]l/1.04[0.71;1.56]l, U, p=0.28. After R significant improvement was found: 82,0[77,0;89,0]%/75,0[70,0;83,0]%, U, p=0.04 and 1.65[0.96;1.93]l/1.04[0.71;1.56]l, U, p=0.02.

**Conclusion:** Preventive spirometry in outpatient MG patients is necessary for early diagnosis and active correction of latent RD.

**Disclosure:** Nothing to disclose.

## EPO‐302

### Respiratory studies in dystrophic myotonia patients

#### K. Malhina ^1^; Y. Rushkevich^1^; V. Haliyeuskaya^1^; A. Gusina^2^; S. Likhachev^1^

##### ^1^Republican Scientific and Practical Center of Neurology and Neurosurgery, Minsk, Belarus; ^2^Republican Scientific and Practical Center “Mother and Child”, Minsk, Belarus.

**Background and aims:** Breathing disorders during the day and at night are common non‐specific clinical manifestations in patients with dystrophic myotonia (DM) but often missed.

**Methods:** The study included 21 DM patients with identified mutation (2(10%) men, 19(90%) women): 17‐DM1, 4‐DM2, 44,0[36,0;50,0] years, BMI 24,0[21,0;29,0]. The control group 24 patients (3(13%) men, 21(87%) women), 40,0[33,0;55,0] years, BMI 26,5[23,0;31,0]. There was no statistical difference in age, gender and BMI. The external respiratory function (ERF) was performed using the MAС‐2 BM spirometer Belarus), and night respiratory monitoring was performed using the Pulsar portable pulseoximeter (Belarus).

**Results:** The results of spirometry and nocturnal pulseoximetry are presented in Tables 1 and 2. The following parameters were significantly reduced in 47,6% DM patients (47%): VC, l/min (U, p=0.001), VC, l % (U, p=0.003), FVC, l/min (U, p=0.04), FVC, l % (U, p=0.001), FEV1, l/min (U, p=0.003), FEV1, l % (U, p=0.004). A decrease in the main respiratory parameters of the pulseoximetry was observed in 42,8% DM patients: average total SpO2, % (U, p=0.001), minimum total SpO2, % (U, p=0.002) and average SpO2 during the sleep period, % (U, p=0.001), AHI, epis/hour (U, p=0.004), DI, epis./hour (U, p=0.02).

**Figure:**
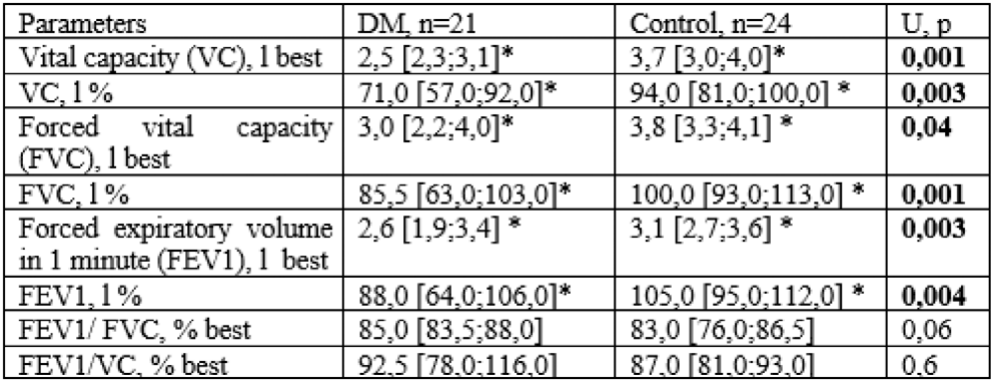
**TABLE 1** Spirometry parameters of patients with DM and the control group, Me[LQ;UQ].

**Figure:**
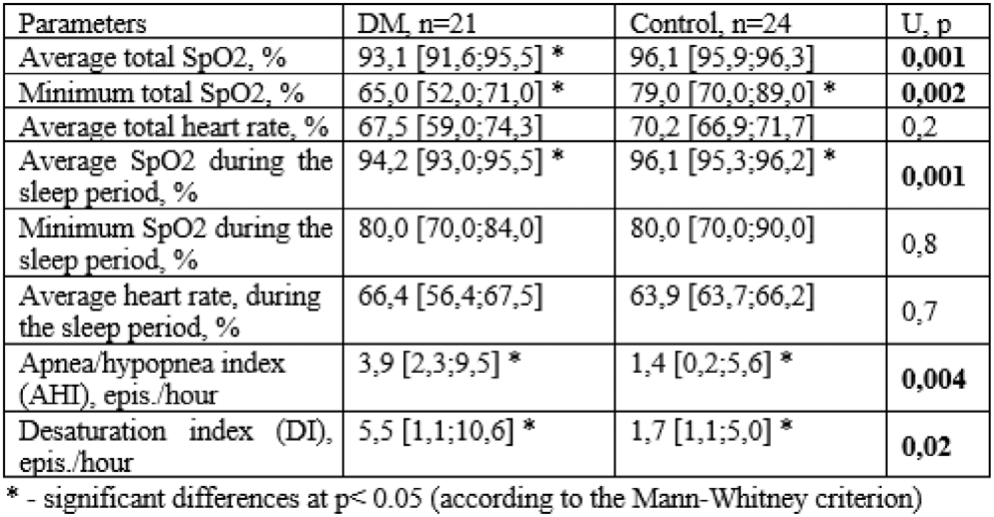
**TABLE 2** Parameters of overnight pulseoximetry in patients with DM and the control group, Me[LQ;UQ].

**Conclusion:** Respiratory disorders have been identified in DM patients: ERF, nocturnal hypoxemia and increased AHI. Persistent nocturnal hypoxemia, a result of sleep‐disordered breathing, leads to cardiovascular and pulmonary failure.

**Disclosure:** Nothing to disclose.

## EPO‐303

### Establishing the REaDY LGMD registry: A czech national database for limb girdle muscular dystrophies

#### L. Mensová ^1^; R. Mazanec^1^; J. Haberlová^2^; L. Juříková^3^; S. Voháňka^4^; J. Junkerová^5^; J. Staněk^6^; E. Ehler^7^; P. Ridzoň^8^; M. Svoboda^9^

##### ^1^Department of Neurology, 2nd Faculty of Medicine, Charles University and Motol University Hospital, Prague, Czechia; ^2^Department of Paediatric Neurology, 2nd Faculty of Medicine, Charles University and Motol University Hospital, Prague, Czechia; ^3^Department of Paediatric Neurology University Hospital in Brno and Faculty of Medicine Masaryk University, Brno, Czechia; ^4^Department of Neurology University Hospital in Brno and Faculty of Medicine Masaryk University, Brno, Czechia; ^5^Department of Neurology, University Hospital Ostrava, Ostrava, Czechia; ^6^Department of Paediatric Neurology, University Hospital Ostrava, Ostrava, Czechia; ^7^Department of Neurology, Pardubice Regional Hospital, Pardubice, Czechia; ^8^Department of Neurology, Thomayer University Hospital, Prague, Czechia; ^9^Institute of Bioanalysis and Statistics, Brno, Czechia

**Background and aims:** Limb Girdle Muscular Dystrophies (LGMD) are rare, genetically diverse neuromuscular disorders with prevalence estimates ranging from 1:14,500 to 1:123,000. Understanding their natural history and genetic background is crucial for patient monitoring and biomarker identification in clinical trials. To address these gaps, we established the REaDY LGMD National Registry in Czechia to: Collect epidemiological and genetic data, Gather longitudinal disease progression data, Set diagnostic and care standards, Support clinical trial feasibility studies.

**Methods:** Launched in June 2020, the registry collects clinical, genetic, and diagnostic data from seven neuromuscular centres. Patients can access their records and complete a Quality‐of‐Life survey (SF‐36). Operating under ethical approval and Czech Neurological Society oversight, it adheres to the TREAT‐NMD dataset and is a TGDOC member, enabling international data sharing.

**Results:** The registry includes 136 patients (58% men, 42% women), primarily aged 11 to 50. The most common LGMD subtypes are: LGMD R1 (calpain‐3 related) in 38 patients (28%), LGMD D4 (calpain‐3 related) in 27 patients (20%), FKRP‐related LGMD in 16 patients (12%). Detailed analysis of allele frequencies, age of onset, age at diagnosis, and clinical features will be presented in a subsequent poster.

**Figure:**
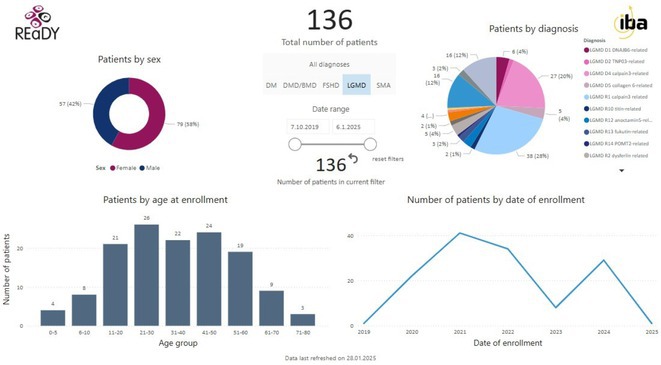
**FIGURE 1** REaDY LGMD basic statistics.

**Conclusion:** The registry continues collaborating with seven neuromuscular centres, with subtype distribution consistent with previous studies. Future plans include expanding enrolment, securing funding, and integrating data within the European TREAT‐NMD network.

**Disclosure:** Nothing to disclose.

## EPO‐304

### Bioequivalence, injection speed, and usability of subcutaneous efgartigimod PH20 administered with a prefilled syringe

#### M. Rongy ^1^; F. Borgions^1^; K. Allosery^1^; J. Noukens^2^; C. De Muynck^1^

##### ^1^argenx, Ghent, Belgium; ^2^Curare Consulting BV, Liempde, The Netherlands

**Background and aims:** Efgartigimod is a human immunoglobulin G1 (IgG1) antibody Fc fragment that reduces IgG levels through neonatal Fc receptor blockade. Efgartigimod administered subcutaneously (SC, coformulated with recombinant human hyaluronidase PH20) is approved for adult patients with generalized myasthenia gravis (gMG; US, EU) and chronic inflammatory demyelinating polyneuropathy (CIDP; US). The 1000‐mg fixed dose of efgartigimod SC is administered via separate vial and syringe (V+S). These studies evaluated the bioequivalence, safety, and usability of efgartigimod SC administered via prefilled syringe (PFS) vs V+S.

**Methods:** Bioequivalence of efgartigimod SC 1000 mg administered via PFS vs V+S was assessed in a phase 1, open‐label, randomized, 2‐period, crossover study. Seventy‐two healthy participants were randomized to receive 1 injection of efgartigimod SC via PFS or V+S in a crossover design. Separate studies tested injection speed and usability of efgartigimod SC administered via PFS.

**Results:** Bioequivalence between efgartigimod SC administered via PFS and V+S was established according to predefined criteria. Efgartigimod serum concentration vs time profiles were similar following a single injection with PFS or V+S. Most adverse events were mild to moderate, and no difference in the incidence of injection‐site reactions was observed. Rapid (20‐second) administration was feasible, and human factor validation studies determined that the PFS could be successfully administered by caregivers and participants with gMG or CIDP.

**Conclusion:** Efgartigimod SC was bioequivalent and demonstrated similar safety and usability profiles when administered via PFS or V+S. A PFS may offer a convenient treatment option for patients with gMG or CIDP.

**Disclosure:** This study was sponsored by argenx; MR, FB, KA, and CDM are employees of argenx; and JN is a consultant for argenx.

## EPO‐305

### Autoantibodies in myasthenia gravis: Cluster analysis, and clinical correlations

#### M. Qu; X. Rong; M. Lv; X. Sun; Y. Zhao; M. Liu

##### the Affiliated Hospital of Qingdao University

**Background and aims:** This study aims to explore autoantibody clusters and their correlations with clinical features in 644 myasthenia gravis (MG) patients.

**Methods:** Medical records of 664 MG patients were reviewed. Five autoantibodies (AChR, MuSK, titin, RyR and LRP4) were selected for cluster analysis. The various clinical manifestations were compared between clusters. Separate association analyses between individual autoantibodies and clinical manifestations as well as among different MGFA subtypes were also performed without prior clustering.

**Results:** Two separate autoantibody clusters were identified, with significantly in review different clinical manifestations. Cluster 1 (485 patients) was characterized by higher proportions of RyR‐, titin‐, and AChR‐, while cluster 2 (179 patients) had higher proportions of RyR+, titin+, and AChR+. Cluster 2 patients were older and had elevated QMG scores and odds of complications, particularly hypertension, diabetes, cardiovascular and cerebrovascular diseases, and eye conditions. Individual antibody analysis revealed male cases were more likely to be AChR+ and titin+, and older age was associated with AChR+, RyR+ and titin+. Among MGFA subtypes, significant differences were detected in AChR, MuSk, titin, complications, thymoma, and hypertension. As MG severity increased from type I to type V, AChR+, RyR+, and titin+ proportions peaked at stage IIa. MuSK+ patients were relatively rare and mostly present in the subtype b group. Type b patients had higher MuSk+ prevalence and increased cardiovascular and cerebrovascular disease incidence rates compared with type a cases.

**Conclusion:** Overall, cluster 2 features were less favorable to patients. This study provides valuable insights into the clinical and autoantibody profiles of Chinese MG patients.

**Disclosure:** Nothing to disclose.

## EPO‐306

### Empasiprubart vs immunoglobulin in chronic inflammatory demyelinating polyneuropathy: EMVIGORATE phase 3 study design

#### S. Rinaldi^1^; T. Brannagan^2^; P. Doneddu^3^; K. Gable^4^; K. Gorson^5^; M. Stettner^6^; O. Van de Steen ^7^; K. Budding^7^; I. Van de Walle^7^; S. Ellor^7^; M. Markov^7^; J. Allen^8^

##### ^1^Nuffield Department of Clinical Neurosciences, University of Oxford, Oxford, UK; ^2^Department of Neurology, Vagelos College of Physicians & Surgeons, Columbia University Irving Medical Center, Columbia University, New York, USA; ^3^Neuromuscular and Neuroimmunology Unit, IRCCS Humanitas Research Hospital, Rozzano, Milan, Italy; Department of Biomedical Sciences, Humanitas University, Milan, Italy; ^4^Neuromuscular Division, Department of Neurology, Duke University Medical Center, Duke University, Durham, USA; ^5^Department of Neurology, St. Elizabeth's Medical Center, Tufts University School of Medicine, Boston, USA; ^6^Department of Neurology and Center for Translational Neuro‐ and Behavioral Sciences (C‐TNBS), University Medicine Essen, Essen, Germany, ^7^argenx, Ghent, Belgium; ^8^Department of Neurology, University of Minnesota, Minneapolis, USA

**Background and aims:** Chronic inflammatory demyelinating polyradiculoneuropathy (CIDP) is a rare, immune‐mediated neuropathy characterised by progressive muscle weakness and sensory dysfunction. The complement system plays a key role in promoting macrophage‐mediated demyelination. Empasiprubart binds C2, blocking activation of classical and lectin complement pathways. EMVIGORATE will compare intravenous (IV) empasiprubart and IV immunoglobulin (IVIg) in CIDP.

**Methods:** This Phase 3, randomised, double‐blinded, double‐dummy study will randomise ~218 adults on stable maintenance IVIg to receive either empasiprubart IV or continue the stable IVIg dose in a 24‐week double‐blind treatment period (Part A), followed by a 24‐month open‐label period (Part B) and a 15‐month safety follow‐up (Figure). In Part A, participants will receive either empasiprubart IV plus IVIg placebo or empasiprubart IV placebo plus IVIg. In Part B, all participants will receive empasiprubart IV, while IVIg treatment will not be permitted. No study treatment will be administered during the safety follow‐up period.

**Figure:**
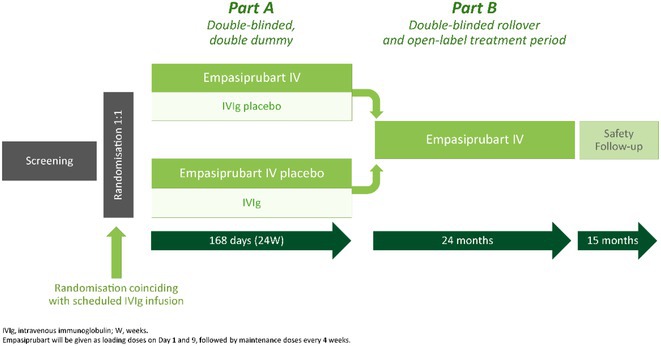
**FIGURE**. EMVIGORATE study design.

**Results:** The primary endpoint is the percentage of participants achieving ≥1‐point improvement versus baseline in adjusted Inflammatory Neuropathy Cause and Treatment (aINCAT) score at Week 24. Secondary endpoints (Week 24) include changes from baseline in Inflammatory Rasch‐Built Overall Disability Scale score, Medical Research Council sum score, dominant hand grip strength and Timed Up and Go, time to decrease of ≥1 point in aINCAT score, and Patient Global Impression of Change actual values.

**Conclusion:** This Phase 3 study will compare the efficacy and safety of empasiprubart IV and IVIg in CIDP, focusing on functional ability, muscle strength/function, gait performance, and patient‐reported outcomes.

**Disclosure:** TB: argenx, Janssen, Immunovant, Sanofi PED: argenx, CSL Behring, Kedrion KLG: Annexon Biosciences, argenx, CSL Behring, Grifols, Immunovant, Sanofi KG: Annexon Biosciences, argenx, Genentech, Momenta, Pfizer, UCB SR: Annexon Biosciences, argenx, the Beijing Association of Holistic and Integrated Medicine, British Medical Association, CSL Behring, Dianthus, Excemed, Fresenius, GBS/CIDP Foundation International, Guillain‐Barré syndrome and Related Inflammatory Neuropathies (GAIN) charity, Hansa Biopharma, the Irish Institute of Clinical Neuroscience, Medical Research Council (UK), National Institute of Health Research (NIHR), the Pathological Society of Great Britain Ireland, Peripheral Nerve Society, Takeda, UCB, the University of Oxford's John Fell Fund, Wellcome Trust MS: argenx, Bayer, Biogen Idec, Biotest, CSL Behring, Genzyme, Grifols, Immunovant, Kedrion, Merck, Novartis, Octapharma, PPTA, Roche, Sanofi‐Aventis, Teva, UCB JAA: Akcea Therapeutics, Alexion, Alnylam Pharmaceuticals, Annexon Biosciences, argenx, CSL Behring, Grifols, Immunovant, Immupharma, Johnson & Johnson, Pfizer, and Takeda OVdS, KBudding, IVdW, SE, and MM are employees of argenx.

## EPO‐307

### Quality of life in patients with generalised myasthenia gravis receiving rozanolixizumab: Post hoc analysis of MycarinG

#### S. Sacconi ^1^; C. Antozzi^2^; E. Cortes‐Vicente^3^; R. Pascuzzi^4^; K. Utsugisawa^5^; J. Vissing^6^; T. Vu^7^; J. Bloemers^8^; F. Grimson^9^; T. Tarancón^10^; V. Bril^11^

##### ^1^Université Côte d’Azur, Peripheral Nervous System & Muscle Department, Pasteur 2 Hospital, Centre Hospitalier Universitaire de Nice, Nice, France; ^2^Neuroimmunology and Muscle Pathology Unit, Multiple Sclerosis Center, Fondazione Istituto di Ricovero e Cura a Carattere Scientifico, Istituto Nazionale Neurologico Carlo Besta, Milan, Italy; ^3^Neuromuscular Diseases Unit, Hospital de la Santa Creu i Sant Pau, Barcelona, Spain; ^4^Neurology Department, Indiana University School of Medicine, Indiana University Health, USA; ^5^Department of Neurology, Hanamaki General Hospital, Hanamaki, Japan; ^6^Department of Neurology, Rigshospitalet, University of Copenhagen, Copenhagen, Denmark; ^7^Department of Neurology, University of South Florida Morsani College of Medicine, Tampa, USA; ^8^UCB, Brussels, Belgium; ^9^UCB, Slough, UK, ^10^UCB, Madrid, Spain, ^11^University Health Network, Toronto, Canada

**Background and aims:** Generalised myasthenia gravis (gMG) is a chronic autoimmune disease that can significantly impact many aspects of patients’ quality of life (QoL). In the MycarinG study (NCT03971422), rozanolixizumab significantly improved myasthenia gravis (MG)‐specific outcomes versus placebo in patients with gMG. In this post hoc analysis, we identified three themes of the MG‐QoL 15‐items revised (MG‐QoL 15r) tool to evaluate the impact of rozanolixizumab on QoL.

**Methods:** Patients received six weekly infusions of rozanolixizumab 7mg/kg, 10mg/kg or placebo to Day 43. We grouped the 15 items of MG‐QoL 15r (total score 0–30; higher scores reflect worse QoL) into three themes: physical (e.g., eating, walking; 0–14), social (e.g., hobbies, family; 0–8) and emotional (e.g., frustration, depression; 0–8). Descriptive analyses of the change from baseline (CFB) in scores at Day 43 for each theme were conducted.

**Results:** Overall, 200 patients received rozanolixizumab 7mg/kg (n=66), 10mg/kg (n=67) or placebo (n=67). Least squares mean (standard error) CFB in MG‐QoL 15r total score at Day 43 was greater for both rozanolixizumab groups than placebo – 7mg/kg: −4.35 (0.93), p=0.018; 10mg/kg: −5.81 (0.97), p<0.001; placebo: −2.11 (0.95). Mean scores CFB were greater for rozanolixizumab than placebo patients for all three themes – physical: −1.6, −2.4 and −0.6; social: −1.3, −1.5 and −0.3; emotional: −1.0, −1.4 and −0.4 for 7mg/kg, 10mg/kg and placebo, respectively.

**Conclusion:** Rozanolixizumab led to greater improvements in scores for the physical, social and emotional themes of the MG‐QoL 15r versus placebo, demonstrating the benefit of treatment across the spectrum of MG symptoms that affect patients’ QoL.

**Disclosure:** This study was funded by UCB. Jos Bloemers, Fiona Grimson, and Thais Tarancón are employees and shareholders of UCB. Full disclosure of all industry relationships will be made during congress presentation if accepted.

## Neuroimmunology 1

## EPO‐308

### Neurology crossroads: Unravelling the Enigma of NMDA encephalitis in patients with multiple sclerosis

#### A. Eissa ^1^; A. Spyropoulos^2^

##### ^1^Neurology Specialist Registrar in the North East of England Deanery; ^2^Neurology Consultant; Royal Victoria Infirmary, Newcastle Upon Tyne Hospitals, NHS Foundation Trust.

**Background and aims:** The co‐occurrence of multiple sclerosis and anti‐NMDAR encephalitis is rarely documented, with only a handful of case reports available. Our understanding of the shared pathogenic processes between these two conditions remains significantly limited.

**Methods:** We describe a trio series of NMDAR Encephalitis/MS overlap.

**Results:** Case 1: A 31‐year‐old female presented with NMDA encephalitis in 2019, experiencing anxiety, hallucinations, and paranoia. A relapse occurred two years later. Both episodes were treated with steroids, plasma exchange, and rituximab. Her 2021 relapse included neurological symptoms, revealing spinal and brain demyelinating lesions. Positive NMDAR antibodies and oligoclonal bands confirmed overlapping NMDA encephalitis and relapsing‐remitting MS. Treatment with Ocrevus stabilized both conditions, preventing further NMDA encephalitis relapses. Case 2: A 27‐year‐old female presented with gait ataxia and ophthalmoplegia. MRI showed cervical demyelination. CSF revealed unmatched oligoclonal bands. She later developed psychiatric symptoms. NMDAR antibodies were positive in serum and CSF. A left ovarian teratoma was discovered and removed, leading to NMDAR antibody disappearance. High‐dose steroids were administered. The patient experienced no further NMDAR encephalitis or MS relapses. Case 3: A 30‐year‐old woman diagnosed with relapsing‐remitting MS in 2019 developed NMDA receptor antibody encephalitis in 2020, with a relapse in 2021. After breakthrough relapses of her MS on Cladribine and natalizumab, she responded well to Ocrelizumab, started in November 2022. By June 2023, CSF NMDA antibodies disappeared, with no further disease activity.

**Conclusion:** These cases highlight the rarity and complexity of cases involving both MS and anti‐NMDAR encephalitis, emphasizing the need for thorough clinical vigilance and tailored treatment approaches.

**Disclosure:** Nothing to disclose.

## EPO‐309

### Evaluating cognitive outcomes in multiple sclerosis: Real‐world impact of ozanimod on processing speed using BICAMS

#### A. Zanghì; p. di filippo; C. Avolio; E. D'Amico

##### University of Foggia, Italy

**Background and aims:** Cognitive dysfunction represents a major burden in Multiple Sclerosis (MS). The impact on cognitive outcomes of ozanimod in real‐world settings remains to be fully elucidated.

**Methods:** In this single‐center observational study, we evaluated cognitive performance in 67 MS patients (74.6% female) receiving ozanimod (mean treatment duration 17.7 ± 3.0 months). Cognitive assessment was performed using the Brief International Cognitive Assessment for Multiple Sclerosis (BICAMS) battery, comprising Symbol Digit Modalities Test (SDMT), California Verbal Learning Test‐II (CVLT‐II), and Brief Visuospatial Memory Test‐Revised (BVMT‐R) collected at different time points.

**Results:** Analysis suggested significant improvement in SDMT Z‐scores (mean improvement 0.337, SD 0.638; Cohen's d = 0.42, p = 0.0111). Baseline SDMT z‐score emerged as the sole significant predictor of cognitive change (coefficient ‐0.345, p < 0.001), accounting for 32.4% of variance. CVLT‐II and BVMT‐R scores remained stable across time points.

**Conclusion:** This real‐world study suggests that ozanimod treatment is associated with significant improvement in information processing speed, independent of traditional prognostic factors. These findings complement existing clinical trial data and warrant further investigation through larger, multicenter studies with extended follow‐up periods to validate these cognitive benefits.

**Disclosure:** Nothing to disclose.

## EPO‐310

### Blood metabolites: Predictors of axonal damage and recovery biomarkers in multiple sclerosis

#### J. Rebeaud^1^; N. Phillips^1^; G. Thévoz^1^; M. Theaudin^1^; R. Du Pasquier^1^; J. Kuhle^2^; T. Collet^3^; C. Pot ^1^

##### ^1^Laboratories of Neuroimmunology, Center for Research in Neuroscience and Service of Neurology, Department of Clinical Neurosciences, Lausanne University Hospital and University of Lausanne, Lausanne, Switzerland; ^2^Multiple Sclerosis Centre and Research Center for Clinical Neuroimmunology and Neuroscience (RC2NB), Departments of Biomedicine and Clinical Research, University Hospital and University of Basel, Basel, Switzerland; ^3^Service of Endocrinology and Diabetology, Geneva University Hospitals, Geneva, Switzerland

**Background and aims:** Multiple sclerosis (MS) is a disorder with an unpredictable outcome at the time of diagnosis. Measurement of serum neurofilament light chain (sNfL) and glial fibrillary acidic protein (sGFAP) opened the field to new biomarkers for MS disease activity and progression. However, additional diagnostic and prognostic tools are needed. The aim of this study was to evaluate the predictive capacity of plasma metabolites, gut microbiota, and clinical/lifestyle factors on MS outcome measures including MS‐related fatigue, MS disability, and sNfL and sGFAP concentrations.

**Methods:** We conducted a prospective cohort study of 54 people with MS and collected anthropometric, biological, and lifestyle parameters. Untargeted metabolomics of plasma samples was performed. Fecal microbiota composition was assessed by 16S rRNA metagenomics sequencing. Nutritional, lifestyle parameters, including sleep and physical activity were obtained. We utilized the least absolute shrinkage and selection operator (LASSO) algorithm with ten‐fold cross‐validation to identify MS disease outcome parameters predictors based on plasma metabolomics, microbiota sequencing, and clinical and lifestyle measurements derived from questionnaires and anthropometric measurements.

**Results:** Circulating metabolites emerged as superior predictors for sNfL and sGFAP concentrations, while clinical and lifestyle data were associated with EDSS scores. Both plasma metabolites and clinical data significantly predicted MS‐related fatigue. Combining multiple multi‐omics data did not consistently improve predictive performance.

**Conclusion:** This study demonstrates that plasma metabolites are valuable predictors of sNfL and sGFAP, and fatigue in MS. Our findings suggest prioritizing metabolomics over other methods for more accurate prediction of MS disease.

**Disclosure:** This project was supported by grants from the Swiss MS Society (grant no. 2021‐13) and the Panacée Foundation. J.R., N.E.P., G.T., T.H.C. have nothing to disclose. J.K. received speaker fees, research support, travel support, and/or served on advisory boards by the Progressive MS Alliance, Swiss MS Society, Swiss National Research Foundation (320030‐189140), University of Basel, Biogen, Celgene, Merck, Novartis, Octave Bioscience, Roche, Sanofi. M.T. received travel grants, advisory board/lecture and consultancy fees from Biogen, Sanofi, Novartis, Merck and Roche. R.D.P. reports that the Lausanne University Hospital received speaker honoraria and travel grants for his activities with Biogen, Genzyme, Merck, Novartis, Roche, and Sanofi. None of them were related to this work. C.P. reports that the Lausanne University Hospital received speaker honoraria, travel grants and consulting services for her activities with Novartis, Roche, Biogen, Merck, Sanofi‐Aventis none related to this work.

## EPO‐311

### The unexpected guest: GABA A autoimmune encephalitis with GAD67 positivity

#### C. Bertolotti ^1^; M. Carasi^1^; R. Hoftberger^2^; F. Boso^1^; B. Giometto^1^

##### ^1^APSS Santa Chiara, UOC Neurologia and University of Trento; ^2^Division of Neuropathology and Neurochemistry (Department of Neurology), Medical University of Vienna

**Background and aims:** The diagnosis of autoimmune encephalitis (AE) is challenging because of overlapping phenotypes and variable testing availability: comprehensive antibody testing may detect rare reactivities such as GABA A and GAD67, but multiple positivity can occur. We present the case of a myeloma patient with ictal aphasia, cacosmia, cognitive impairment, multifocal brain lesions, oligoclonal bands, CSF monoclonal CD5‐ lymphocytes, but no corresponding antibodies on AE panels: brain biopsy was needed to confirm AE and CSF retesting revealed GABA A and GAD67 (but not GAD65) positivity.

**Methods:** This case was compared with literature reports: concurrent GABA A/GAD65 positivity may be common, while CSF antibodies exclusively against GAD67 are less studied and associated with heterogeneous, GAD65‐like, phenotypes (epilepsy, cerebellar ataxia, AE, stiff person syndrome).

**Results:** The significance of anti‐GAD67 antibodies remains elusive, due to limited diagnostic use and sparse literature. GAD67 autoimmunity may represent an epiphenomenon in syndromes like GABA A‐ AE, but a diagnostic and/or pathogenic role for GAD67 antibodies cannot be excluded, as they may potentially worsen the GABAergic dysregulation and perpetuate the inflammation and consequent neuronal damage.

**Conclusion:** Further research is needed to understand the nuances of multiple antibody positivity in AE, particularly when rare and less characterised antibodies such as GAD67 are involved. Brain biopsy and expert pathology review are essential not only for identifying rare antibody patterns, but also for speculating on the dominant drivers of the disease.

**Disclosure:** Nothing to disclose.

## EPO‐312

### Commercial tissue‐based assays are suboptimal to detect intracellular antibodies in autoimmune neurological syndromes

#### C. Milano ^1^; P. Businaro^2^; C. Papi^3^; L. Marmolejo^4^; L. Naranjo^5^; R. Ruiz Garcia^5^; L. Arlettaz^6^; M. Gastaldi^7^; E. Martinez‐Hernandez^4^; T. Armangué^8^; M. Guasp^4^; L. Sabater^4^; J. Dalmau^4^; F. Graus^4^; M. Spatola^4^

##### ^1^Neuroimmunology Program, FRCB‐IDIBAPS, University of Barcelona, Spain; La Caixa Research Institute, Barcelona, Spain; Department of Clinical and Experimental Medicine, University of Pisa, Pisa, Italy; ^2^Neuroimmunology Program, FRCB‐IDIBAPS, University of Barcelona, Spain; Department of Brain and Behavioral Sciences, University of Pavia, Pavia, Italy; Neuroimmunology Research Section, IRCCS Mondino Foundation, Pavia, Italy; ^3^Neuroimmunology Program, FRCB‐IDIBAPS, University of Barcelona, Spain; La Caixa Research Institute, Barcelona, Spain; 6. Department of Neuroscience, Catholic University of the Sacred Heart, Rome, Italy; ^4^Neuroimmunology Program, FRCB‐IDIBAPS, University of Barcelona, Spain; La Caixa Research Institute, Barcelona, Spain; ^5^Immunology Service, Biomedical Diagnostic Center, Hospital Clínic, Barcelona, Spain; ^6^Service d’Immuno‐Allergologie, Institut Central des Hôpitaux, Sion, Switzerland; ^7^Neuroimmunology Research Section, IRCCS Mondino Foundation, Pavia, Italy; ^8^Neuroimmunology Program, FRCB‐IDIBAPS, University of Barcelona, Spain; La Caixa Research Institute, Barcelona, Spain; Pediatric Neuroimmunology Unit, Sant Joan de Déu Children's Hospital, University of Barcelona, Barcelona, Spain

**Background and aims:** Current techniques to identify autoantibodies against intracellular neural antigens (IC‐abs) include tissue‐based assays (TBAs) alongside line‐blot or cell‐based assays (CBAs). Most clinical laboratories rely on commercially available TBAs, whose diagnostic accuracy has not been assessed. Here, we evaluated the performance of two commercial TBAs.

**Methods:** We tested samples from 100 patients with autoimmune neurological syndromes harbouring IC‐abs (determined by in‐house TBAs and line‐blot or CBAs) and from 50 negative controls. IC‐abs samples included sera (10 of each: Hu, Yo, Ri, SOX1, CV2, Ma2, Tr, amphiphysin, GAD65) or CSF (10 GFAP). Two commercial indirect immune‐fluorescent TBAs (INOVA and EUROIMMUN) were blindly evaluated by two experienced and three less‐experienced investigators; discordant results were re‐evaluated in an interrater discussion.

**Results:** The two experienced raters showed substantial agreement (>95% after interrater agreement) on negative or positive results. They correctly identified 118/150 (79%) and misclassified 28/150 (19%) samples with INOVA, whereas they correctly identified 106/150 (71%) and misclassified 39/150 (26%) samples with EUROIMMUN. Sensitivity was 73% for INOVA and 66% for EUROIMMUN. Specificity was 96% for INOVA and 88% for EUROIMMUN. Among the positive samples, antibody‐specific immunostaining patterns were correctly identified in 62/100 samples with INOVA and 55/100 with EUROIMMUN (p=0.39). Both TBAs failed to identify CV2 antibodies. INOVA better identified Ma2 antibodies (9/10 vs. 1/10, p=0.001), while EUROIMMUN better identified Hu/Ri antibodies (19/20 vs. 12/20, p=0.02). Less‐experienced raters showed higher rate of false positive results.

**Conclusion:** The performance of commercial TBAs for IC‐abs is suboptimal, particularly for CV2 (both kits), Ma2 (EUROIMMUN) and Hu/Ri (INOVA) antibodies.

**Disclosure:** M. Spatola receives research support from La Caixa Foundation (Junior Leader) and Spanish National Health Institute Carlos III (ISCIII) and co‐funded by the European Union (FIS grant PI23/01366). J. Dalmau receives research support from CaixaResearch Health 2022 (HR22‐00221), Spanish National Health Institute Carlos III (ISCIII) and co‐funded by the European Union (FIS grant PI23/00858), Cellex Foundation, Fundació Clínic per a la Recerca Biomèdica (FCRB) Programa Multidisciplinar de Recerca, Generalitat de Catalunya Department of Health (SLT028/23/000071), Edmond J Safra Foundation. He receives royalties from Euroimmun for the use of NMDA as an antibody test. He received a licensing fee from Euroimmun for the use of GABAB receptor, GABAA receptor, DPPX and IgLON5 as autoantibody tests; he has received a research grant from Sage Therapeutics. All the other authors report no disclosures.

## EPO‐313

### Risk assessment for progressive multifocal leukoencephalopathy using the two available tests

#### E. Alba Suárez; L. Franco Rubio; L. García Vasco; I. Gómez Estévez; L. López Trashorras; N. Rodríguez Albacete; P. Abizanda Saro; A. Aldaz Burgoa; M. Castro Hernández; L. Aguilera Carretero; E. Pizarro; M. García Rama; C. Oreja‐Guevara

##### CSUR Esclerosis Múltiple, Servicio de Neurología, Hospital Clínico San Carlos, Madrid, Spain

**Background and aims:** Patients treated with natalizumab are at an increased risk of PML caused by the reactivation of JC virus (JCV). The main risk factors include treatment duration, prior immunosuppression, and the presence of anti‐JCV antibodies in the blood. StratifyJCV™ is a Biogen risk stratification algorithm that combines anti‐JCV antibody status using an ELISA test, prior immunosuppression, and natalizumab treatment duration (by treatment year). Recently, Sandoz developed the ImmunoWELL™ JCV IgG test, which also uses a two‐step ELISA technique validated by the European Medicines Agency, demonstrating non‐inferiority compared to the StratifyJCV test with a sensitivity of 95%.

**Methods:** Eighty patients (72% women) with a diagnosis of relapsing‐remitting MS (RRMS) under natalizumab treatment and follow‐up at our center were recruited. Both risk stratification tests, StratifyJCV and ImmunoWELL, were performed simultaneously using the same sample.

**Results:** 34% of StratifyJCV tests were positive, whereas 60% of ImmunoWELL tests were positive. When comparing the results, 29% of them did not match, with StratifyJCV yielding negative results while ImmunoWELL was positive. Additionally, two patients showed differences greater than one point in the test results.

**Conclusion:** More than 25% of the results showed discrepancies between the two JCV antibody tests. The percentage of positive results was higher with ImmunoWELL. These discrepancias create difficulties for risk management strategies in conditions like PML.

**Disclosure:** Nothing to disclose.

## EPO‐314

### Immune checkpoint inhibitors trigger and exacerbate anti‐CV2/CRMP5 paraneoplastic neurological syndromes

#### G. Cereda ^1^; M. Villagrán‐García^2^; A. Farina^2^; M. Benaiteau^2^; A. Sautereau^3^; C. Birzu^2^; M. Bayrak^2^; G. Picard^2^; D. Psimaras^4^; V. Rogemond^2^; B. Joubert^2^; J. Honnorat^2^

##### ^1^French Reference Centre on Paraneoplastic Neurological Syndromes and Autoimmune Encephalitis, Hospices Civils de Lyon, Hôpital Neurologique, Bron, France; School of Medicine and Surgery, University of Milano‐Bicocca, Milan, Italy; ^2^French Reference Centre on Paraneoplastic Neurological Syndromes and Autoimmune Encephalitis, Hospices Civils de Lyon, Hôpital Neurologique, Bron, France; MeLiS‐UCBL‐CNRS UMR 5284. INSERM U1314, Université Claude Bernard Lyon 1, Lyon, France; ^3^CHSSM, Fontainebleau Hospital, Oncology Hematology Medicine Department, Fontainebleau, France; ^4^AP‐HP, Hospital Group Pitié‐Salpêtrière, Neuro‐oncology Department Paris, France; Inserm U1127, CNRS, Paris Brain Institute, Institut du Cerveau (ICM), Paris, France

**Background and aims:** Immune‐checkpoint inhibitors (ICI) may trigger or worsen paraneoplastic neurological syndromes (PNS). We described CV2/CRMP5‐PNS patients treated by ICI, compared post‐ICI cases with ICI‐naïve, and estimated the overall survival of ICI‐treated patients with CV2/CRMP5‐PNS, Hu‐, and Ma2‐PNS.

**Methods:** All patients positive for anti‐CV2/CRMP5 antibodies and treated with ICI in a French referral center (2016‐2024) were included.

**Results:** Fourteen ICI‐treated CV2/CRMP5‐PNS patients were included. Eight [median age, 73 years; 87.5% men] developed post‐ICI PNS after a median of 3.5 ICI cycles. Frequency and distribution of clinical phenotypes [isolated neuropathy (n=3), or multifocal involvement (encephalopathy, limbic, brainstem, cerebellar, ocular, neuropathy, and/or dysautonomia; n=5)] was similar to ICI‐naïve CV2/CRMP5‐PNS (n=48). Frequency of severe presentations [modified Rankin Scale (mRS) >3] at diagnosis was similar between post‐ICI and ICI‐naïve CV2/CRMP5‐PNS (63% vs 48%, p=0.7), but non‐significantly higher at last visit in post‐ICI patients (88% vs 54%, p=0.12). Anti‐CV2/CRMP5 antibodies were undetectable in one patient with a pre‐ICI serum sample. Among 6 patients with pre‐existing CV2/CRMP5‐PNS [median age, 66 years; 50% men], PNS worsened in 5 (83%) (median mRS increase of 1.5 points) after ICI. Median overall survival (22 months) was significantly longer in ICI‐treated CV2/CRMP5‐PNS compared to Hu‐ and Ma2‐PNS (4 months and 8 months, respectively, p=0.0069).

**Conclusion:** ICI may trigger the onset and exacerbate the progression of CV2/CRMP5‐PNS. Post‐ICI forms are clinically undistinguishable than ICI‐naïve. Pre‐ICI antibody negativity in one case challenges the role of baseline onconeural antibody testing as predictive biomarker for the development of PNS. Post‐ICI PNS have variable prognosis according to associated onconeural autoantibodies.

**Disclosure:** This work is supported by a public grant overseen by the Agence Nationale de la Recherche (ANR; French research agency) as part of the Investissements d’Avenir program (ANR‐18‐RHUS‐0012), also performed within the framework of the LABEX CORTEX of the Université Claude Bernard Lyon 1 (program Investissements d’Avenir, ANR‐11‐LABX‐0042, operated by the ANR) and supported by the European Reference Network RITA. G.S.C and A.F were awarded a research fellowship by the European Academy of Neurology. M.V‐G. is funded by a research grant from Fundación Alfonso Martín Escudero (Spain).

## EPO‐315

### Preoperative efgartigimod benefits myasthenia gravis patients undergoing thymectomy

#### X. Zhang^1^; H. Zhang^2^; J. Li^1^; Z. Pan^1^; Y. Liu^1^; Y. Xue^1^; X. Zheng^1^; X. Zhang^1^; G. Qi ^1^

##### ^1^Center of Treatment of Myasthenia Gravis, People's Hospital of Shijiazhuang, Shijiazhuang, China; ^2^Department of Neurology, Tianjin Neurology Institution, Tianjin Medical University General Hospital, Tianjin, China

**Background and aims:** Thymectomy is an effective way to alleviate muscle weakness for Myasthenia Gravis (MG) patients with and without thymoma. However, thymectomy may exacerbate MG. Efgartigimod as a neonatal Fc receptor blocker has been found to be effective in generalized MG, and this study aims to explore whether application of efgartigimod before surgery can also benefit MG patients who undergo thymectomy.

**Methods:** We retrospectively included 44 patients from Shijiazhuang People's Hospital via propensity score matching analysis (nearest neighbor 1:3 matching). Clinical features, quantitative antibody test results, Myasthenia Gravis Foundation of America (MGFA) type at different periods, thymic pathology, operation information, and muscle functional change as evaluated by both MG‐ADL and QMG scores were collected. Patients were divided into two groups: the efgartigimod group and the non‐efgartigimod group.

**Results:** Patients in the Efgartigimod group had a shorter time to reach surgical criteria (efgartigimod vs. non‐efgartigimod, 4 days vs. 55 days, p < 0.001) (Figure 1) and a shorter time to postoperative treatment in the ICU (36 h vs. 44 h, p = 0.007) compared with patients in the non‐efgartigimod group (Figure 2d). In addition, MG‐ADL (9 vs. 1, p = 0.002) and QMG scores (7 vs. 2, p < 0.001) showed greater functional improvement and greater decline in antibody titers (3.73 vs. 0.39, p < 0.001) (Figure 2a‐c). The efgartigimod group had a higher incidence of better postoperative MGFA type (Figure 3).

**Figure:**
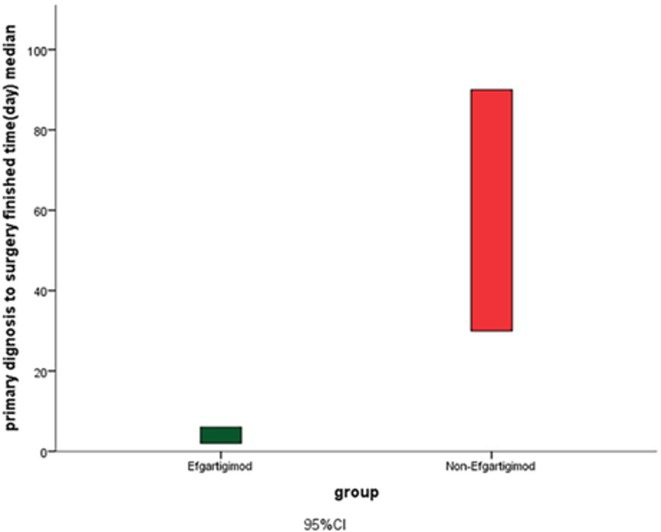
**FIGURE 1** Lower primary‐diagnosis‐to‐finished‐surgery time was observed in the efgartigimod group.

**Figure:**
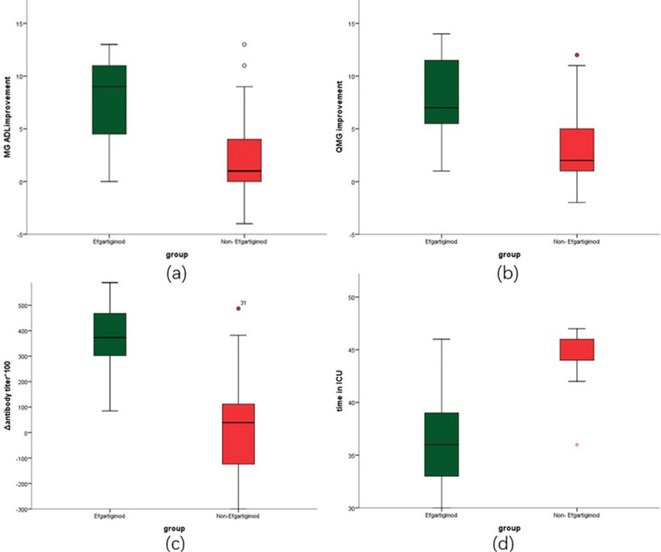
**FIGURE 2** (a‐c) Changes of MG ADL, QMG score and antibody titer after operation compared with those before operation. (d) Patients with preoperative efgartigimod spent less time in the ICU post‐surgery.

**Figure:**
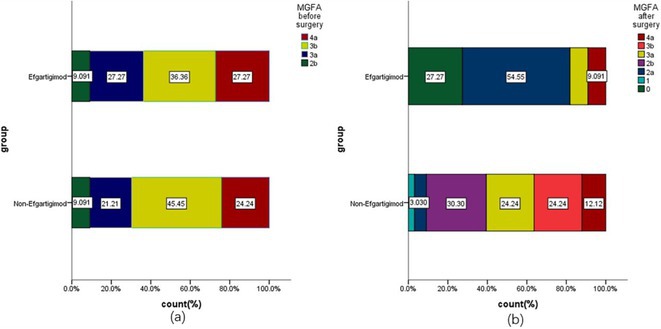
**FIGURE 3** (a) There were similar MGFA types before surgery in both groups; (b) Patients in the efgartigimod group obtained better MGFA types post‐surgery compared to the non‐efgartigimod group.

**Conclusion:** Using Efgartigimod is a good way for unstable MG patients to become more stable prior to surgery.

**Disclosure:** This work was supported by the National Natural Science Foundation of China (No.: 82274582), Central Guidance for Local Scientific and Technological Development Funding Projects (No.: 246Z7706G) and the Hebei Province Innovation Capability Enhancement Plan Project (No.: 20577715D).

## EPO‐316

### Tracking eye movements to detect motor and cognitive decline in Multiple Sclerosis: A novel approach

#### M. Eizaguirre ^1^; N. Ciufia^1^; A. Marinangeli^1^; L. Bacigalupe^1^; L. Ibarra^1^; L. Lapalma^1^; M. Casas^1^; G. Fernandez^2^; M. Shulz^2^; D. Verge^2^; R. Alonso^1^

##### ^1^Working Group on Demyelinating Diseases (CUEM). Neurology Division, Dr. J.M. Ramos Mejia Hospital, Buenos Aires, Argentina; ^2^ViewMind Inc, New York, USA

**Background and aims:** Multiple sclerosis (MS) is a neurological disorder characterized by physical and cognitive impairments. Abnormal eye movements, commonly observed in MS, may reflect underlying cognitive dysfunction. However, their relationship with disease severity remains unclear. This study investigates the association between motor and cognitive functions and eye movement parameters during the n‐back and Go/No‐Go tasks in individuals with MS.

**Methods:** A cross‐sectional study was conducted with 71 participants with MS (pwMS). Eye movements were tracked during the n‐back and Go/No‐Go tasks using a head‐mounted display with eye‐tracking technology. Parameters analyzed included saccade amplitude, gaze duration, task duration, number of fixations, single fixations, and refixations. Motor and cognitive functions were assessed with the Expanded Disability Status Scale (EDSS), Nine Hole Peg Test (NHPT), Timed 25‐Foot Walk (T25FW), Symbol Digit Modalities Test (SDMT), Paced Auditory Serial Addition Test (PASAT), California Verbal Learning Test (CVLT), and Brief Visuospatial Memory Test‐revised (BVMT‐R). Statistical analyses were performed, with significance set at p<0.05.

**Results:** Participants (mean age 40.2±12.03 years, disease duration 9.7±6.8 years) showed significant associations between eye movement patterns and disability. In the n‐back task, longer fixation duration correlated with higher disability and lower SDMT scores, while saccade amplitude was shorter with higher disability (p<0.05). In the Go/No‐Go task, increased refixations and reduced single fixations were linked to greater disability and poorer cognitive scores (p<0.05).

**Conclusion:** Eye movement parameters significantly correlate with motor and cognitive impairments in pwMS, highlighting their potential as biomarkers for monitoring MS progression.

**Disclosure:** María Barbara Eizaguirre, Natalia Ciufia, Aldana Marinangeli, Lucia Bacigalupe, Lucia Ibarra, Lucas Nicolas Lapalma, Magdalena Casas, Ricardo Alonso: nothing to disclose. Gerardo Fernandez, Danilo Verge, and Matías Shulz are employees of ViewMind.

## EPO‐317

### Microglial NFAT5 aggravates neuroinflammation via mediating NLRP6 inflammasome in experimental Ischemic stroke

#### H. Gan

##### Chongqing Medical University

**Background and aims:** Microglial activation triggers the inflammatory cascade and exacerbates brain injury following ischemic stroke. Middle cerebral artery occlusion (MCAO) model increased the expression of Nuclear factor of activated T cells 5 (NFAT5) in microglia. However, the role of microglial NFAT5 in ischemic stroke remains unclear.

**Methods:** The MCAO and Oxygen‐Glucose Deprivation/Reoxygenation (OGD/R) were utilized to emulate ischemia‐reperfusion injury. In addition, recombinant Adeno‐Associated Virus (rAAV) was employed for the specific silencing of microglial NFAT5 in vivo. In vitro, short hairpin RNA (shRNA) was utilized to knock down NFAT5.

**Results:** Here, our findings indicated that microglial NFAT5 knockdown reduced the expression of pro‐inflammatory factors, microglial activation, and neutrophil infiltration, ultimately ameliorating cerebral infarction and neurological deficits in mice following MCAO. Additionally, we treated hippocampal neuronal cells (HT22) with conditioned culture medium from a microglia cell line (BV2) to simulate microglia‐induced neuronal injury in vitro. We observed that NFAT5 knockdown attenuated the expression of pro‐inflammatory factors in BV2 cells and reduced apoptosis in HT22 cells. Previously, our published work reported that the NOD‐like receptor pyrin domain‐containing 6 (NLRP6) inflammasome contributes to inflammatory injury after MCAO. In this study, we discovered that NFAT5 promotes the transcriptional activity of the Nlrp6 promoter through its ‐1527bp to ‐1518bp element. Notably, our results also demonstrate that NFAT5 regulates the stability of NLRP6 mRNA via the 5'UTR of Nlrp6.

**Conclusion:** Our research indicates that the transcription factor NFAT5 may potentially exacerbates neuroinflammation and cerebral ischemia‐reperfusion injury by modulating the mRNA level of NLRP6 at both the transcriptional and post‐transcriptional stages.

**Disclosure:** Nothing to disclose.

## EPO‐318

### Treatment utilization and clinical outcomes by serostatus in a real‐world US generalized myasthenia gravis population

#### L. Miller‐Wilson^1^; J. Conyers^2^; S. Birija^2^; H. Connolly^2^; G. Gibson^2^; L. Lal ^1^; Y. Edwards^1^

##### ^1^Immunovant, Inc., New York, USA; ^2^Adelphi Real World, Bollington, UK

**Background and aims:** Generalized myasthenia gravis (gMG) is characterized by impaired transmission at the neuromuscular junction that is mostly driven by autoantibodies, including those directed against the acetylcholine receptor (AChR‐Ab+) and other targets (non‐AChR‐Ab+), though some patients are seronegative. This study describes treatment utilization and clinical outcomes in patients with gMG based on serostatus.

**Methods:** Data were from the Adelphi gMG II Disease Specific Programme™, conducted in the US from February–August 2024. Neurologists provided cross‐sectional and chart‐pulled patient data (demographics, Myasthenia Gravis‐Activities of Daily Living [MG‐ADL] score, clinical events, treatment utilization) in patients with varying serostatus.

**Results:** Fifty‐two neurologists provided data on 336 patients with gMG (mean [SD] time since diagnosis, 3.8 [5.6] years, 53.9% male, mean [SD] age, 54.9 [13.3] years). Most patients were AChR‐Ab+ (73.5%), 10.1% were non‐AChR‐Ab+, 11.3% were seronegative and 5.1% had unknown serostatus. Maintenance treatment was prescribed in 95.5% of AChR‐Ab+ patients (mean [SD] number of regimens used since diagnosis, 1.8 [1.0]), 94.1% of non‐AChR‐Ab+ patients (1.4 [1.0]), 89.5% of seronegative patients (1.4 [1.0]) and 52.9% of patients with unknown serostatus (0.6 [0.6]). Mean (SD) MG‐ADL scores in AChR‐Ab+, non‐AChR‐Ab+, seronegative, and unknown serostatus patients were 4.3 (3.3), 4.9 (4.0), 3.2 (3.1), and 3.8 (3.3), respectively. Since diagnosis, myasthenic crises or symptom exacerbations were reported in 45.8%, 39.4%, 27%, and 7.1% of patients, respectively.

**Conclusion:** Patients with gMG experience clinical events and activity impairment despite treatment and regardless of serostatus. Additional treatment options are needed for all patients to optimize clinical outcomes.

**Disclosure:** LAMW, LL and YE are employees of Immunovant, Inc., JC, SLB, HC and GG are employees of Adelphi Real World

## EPO‐319

### Kappa index in multiple sclerosis: A pivotal study to determine any correlation to recent disease activity

#### L. Paciolla ^1^; V. Ciampana^1^; A. Bianchi^1^; D. Ferrandi^2^; D. Vecchio^1^; C. Comi^1^

##### ^1^Neurology Unit, Department of Translational Medicine, University of Piemonte Orientale, Maggiore della Carità University‐Hospital, Novara, Italy; ^2^Neurology Unit, “Santi Antonio e Biagio e Cesare Arrigo” Hospital, Alessandria, Italy

**Background and aims:** kappa index (K‐index) is a marker of intrathecal synthesis widely studied for its diagnostic role in multiple sclerosis (MS). Increasing evidence suggests its prognostic value, yet the factors influencing K‐index levels require further exploration 2.3. This study aimed to investigate clinical and biological correlates of the K‐index, particularly its relationship with inflammatory activity and disease course.

**Methods:** This cross‐sectional study included 100 patients diagnosed with MS from two Italian centers. Data collection included cerebrospinal fluid (CSF) analyses, clinical parameters, and disease course characteristics. Linear regression models were used to evaluate associations between K‐index and variables such as recent clinical or radiological activity, time from disease onset, Link index, lambda index, and CSF cellularity.

**Results:** No significant correlation was observed between K‐index and recent disease reactivation (N=100; p=0.865), recent brain or spinal MRI with contrast‐enhancing lesions (N=32; p=0.394), time from disease onset (N=76; p=0.166), or lambda index (N=55; p=0.393). K‐index significantly correlated with Link index (N=87; p<0.001, estimate=95.219, 95% CI=57.67–132.77) and CSF cellularity (N=77; p<0.001, estimate=3.603, 95% CI=1.93–5.28).

**Conclusion:** Our findings suggest that the K‐index is not influenced by recent clinical relapse or the presence of contrast‐enhancing lesions, indicating that its value is more reflective of ongoing and temporally disseminated inflammation rather than acute disease reactivation. The significant correlation with the Link index and CSF cellularity supports its role as a marker of chronic intrathecal inflammation. Further studies are needed to confirm its prognostic utility and potential implications for therapeutic decision‐making in MS.

**Disclosure:** Nothing to disclose.

## EPO‐320

### Clinical features of patients with myasthenia gravis with initial worsening after high‐dose methylprednisolone pulse

#### M. Yamazaki; G. Watanabe; M. Saito; N. Tokashiki; T. Miyamoto; K. Tsukita; Y. Suzuki

##### Department of Neurology, National Hospital Organization Sendai Medical Center, Sendai, Japan

**Background and aims:** Myasthenia gravis (MG) is treated with high‐dose methylprednisolone pulse (HMP) as a rescue treatment for acute exacerbations. However, initial worsening may occur after treatment (often 2–5 days after administration). There are few reports on the characteristics and treatment strategies of MG patients with initial worsening after HMP. We investigated the clinical characteristics of MG patients with initial worsening following HMP.

**Methods:** We reviewed the electronic records of 123 MG patients admitted between April 2019 and June 2024, who underwent initial HMP. The patients were divided into two groups: 31 patients who developed an initial worsening (IW group) and 92 patients who did not (Non‐IW group). Clinical characteristics, such as gender, age, presence of thymoma, MG‐Activities of Daily Living scale, current and maximum prednisolone (PSL) doses, use of intravenous immunoglobulin/plasmapheresis (IVIg/PP) at HMP, and incidence of bulbar symptoms, were compared between the two groups.

**Results:** The IW group showed a higher frequency of bulbar symptoms [IW, n (%) vs. Non‐IW, n (%); 19 (61.3%) vs. 27 (29.7%); p < .0025] and the use of IVIg/PP [IW, n (%) vs. Non‐IW, n (%); 28 (90.3%) vs. 50 (54.4%); p < .0002]. The Non‐IW group had a higher frequency of ocular MG [IW, n (%) vs. Non‐IW, n (%); 3 (9.7%) vs. 38 (41.3%); p < .0009].

**Conclusion:** Initial worsening should be anticipated in generalized MG treated with HMP, even when IVIg/PP is combined. The dosage regimen of HMP should be determined considering clinical features of individual MG patients such as ocular or bulbar symptoms.

**Disclosure:** Dr. Genya Watanabe has received honoraria for lectures from Argenx Japan, UCB Japan, and Alexion Pharmaceuticals.

## EPO‐321

### Targeting ROS‐dependent NET formation with natural molecules: A potential approach for managing multiple sclerosis

#### I. Belhassen

##### Neurology Department, Habib Bourguiba University Hospital, Sfax, Tunisia

**Background and aims:** Neutrophil extracellular traps (NETs) are released by neutrophils as a defense mechanism, but in autoimmune diseases like multiple sclerosis (MS), excessive NET formation contributes to neuroinflammation and tissue damage.

**Methods:** Four natural molecules (oleuropein, phenylethanol, tyrosol, and tyrosol3) were tested for their capacity to inhibit NET formation in primary human neutrophils in vitro. To evaluate the potential cytotoxic effects of these molecules, we used the Trypan blue exclusion test. Since NET formation is primarily ROS‐dependent, the luminol‐amplified chemiluminescence assay was employed to assess intra‐ and extracellular ROS levels. Fluorescence microscopy, with Hoechst as a DNA intercalating dye, was used to visualize NET production.

**Results:** Our results showed that our four molecules do not have an acute cytotoxic effect on neutrophils maintained in culture for 48 hours. A high anti‐radical effect of these four molecules was translated by a strong inhibition of the production of superoxide anion by neutrophils. Fluorescence microscopy showed that the treatment of neutrophils with the four molecules has significantly reduced the production of NETs.

**Conclusion:** Our results revealed for the first time an inhibitory effect of four natural molecules on ROS‐dependent NET release. These four molecules could be considered in the future as a palliative treatment for patients with autoimmune diseases.

**Disclosure:** Nothing to disclose.

## Neuroimaging and Neurosonology

## EPO‐322

### Visual inspection of dorsolateral nigral hyperintensity in idiopathic REM sleep behaviour disorder

#### V. Tseriotis ^1^; T. Mavridis^2^; A. Adamou^3^; S. Konitsiotis^4^; M. Arnaoutoglou^5^; G. Chamko^4^; M. Wattjes^6^

##### ^1^Department of Neurology, Agios Pavlos General Hospital of Thessaloniki, Thessaloniki, Greece; ^2^Department of Neurology, Tallaght University Hospital (TUH), Dublin, Ireland; ^3^Institute for Diagnostic and Interventional Neuroradiology, Hannover Medical School, Hannover, Germany; ^4^Department of Neurology, University Hospital of Ioannina, Ioannina, Greece, ^5^1st Department of Neurology, University Hospital AHEPA, Faculty of Medicine Aristotle University of Thessaloniki, Thessaloniki, Greece; ^6^Department of Neuroradiology, Charité ‐ Universitätsmedizin Berlin, corporate member of Freie Universität Berlin and Humboldt‐Universität zu Berlin, Berlin, Germany

**Background and aims:** Early detection of idiopathic REM sleep behaviour disorder (iRBD) through neuroimaging can enhance prognostication and support the identification of phenoconversion to Parkinson's disease (PD). This study explores the clinical utility of dorsolateral nigral hyperintensity (DNH) loss on iron‐sensitive MRI in iRBD.

**Methods:** We searched MEDLINE, Scopus, Web of Science, ProQuest and Google Scholar for observational studies, assessed eligible studies with QUADAS‐2 and performed proportional and diagnostic test accuracy meta‐analysis. Heterogeneity was quantified with I2 statistics and investigated with meta‐regression.

**Results:** Among 349 search results, 5 studies of satisfying quality were eligible (420 patients, iRBD, n = 117, PD, n = 175, healthy controls, HCs, n = 128). Pooled difference in prevalence of STS loss between PD and iRBD was 0.37 [0.12; 0.72], with high heterogeneity (I2 = 94.6%). Pooled sensitivity and specificity for iRBD vs HCs differentiation were 0.49 [0.34; 0.64], I2 = 62.4%, and 0.91 [0.82; 0.95], I2 = 0.0%, respectively. Summary Area Under the Curve (SAUC) was 0.64. Meta‐regression eliminated heterogeneity, showing longer disease duration linked to increased sensitivity. Three studies investigated the capacity of DNH loss in detecting iRBD patients with abnormal nuclear scans, with sensitivity and specificity of 0.79 [0.65; 0.88], I2 = 0.0%, and 0.77 [0.54; 0.91], I2= 57.2%, respectively. SAUC was 0.80.

**Figure:**
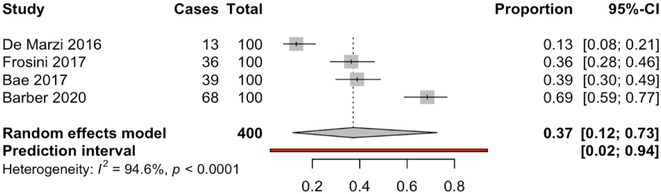
**FIGURE 1** Forest plot of meta‐analysis regarding the difference in prevalence of STS loss between PD and iRBD subjects (PD‐iRBD).

**Figure:**
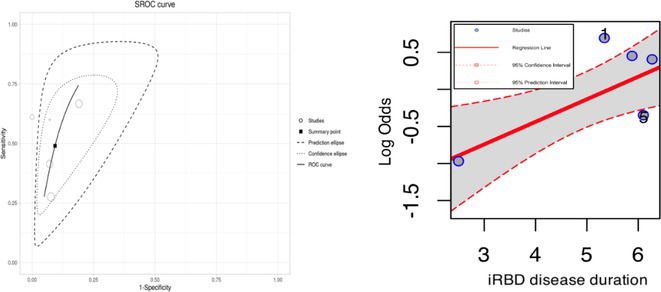
**FIGURE 2** Diagnostic accuracy meta‐analysis: Summary receiver‐operating characteristic curve (left) and meta‐regression analysis (right), with a significant positive association between sensitivity in differentiating iRBD from HCs and disease duration.

**Figure:**
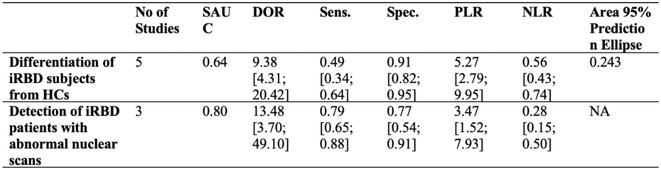
**TABLE 1** Pooled diagnostic accuracy measures. STS: swallow tail sign; iRBD: idiopathic REM sleep behavior disorder; HCs: healthy controls; SAUC: Summary Area Under the Curve; PLR: positive likelihood ratio; NLR: negative likelihood ratio; NA: not applicable.

**Conclusion:** Prevalence of DNH loss differs between iRBD and PD. Iron‐sensitive MRI may aid identification of iRBD among other sleep disorders, especially in association with disease duration, and may comprise a stratification tool for the conduction of nuclear scans.

**Disclosure:** Nothing to disclose.

## EPO‐323

### The tsiogkaspaeth grid for detection of neurological visual field defects: A validation study

#### Tsiogka^1^; E. Karmiris^2^; K. Chatzistefanou^1^; E. Samoli^3^; D. Papakonstantinou^1^; G. Karagiorgis ^4^; G. Spaeth^5^

##### ^1^1st Department of Ophthalmology, National and Kapodistrian University of Athens, School of Medicine, General Hospital of Athens “G. Gennimatas”, Athens, Greece; ^2^Ophthalmology Department, Hellenic Air Force General Hospital, Athens, Greece; ^3^Department of Hygiene, Epidemiology and Medical Statistics, Medical School, National and Kapodistrian University of Athens, Athens, Greece; ^4^Aeginitio Hospital, National and Kapodistrian University of Athens, Athens, Greece, ^5^5Glaucoma Service, Wills Eye Hospital, Sidney Kimmel College of Medicine, Thomas Jefferson University, Philadelphia, USA

**Background and aims:** The TsiogkaSpaeth (TS) grid is a new, low cost and easy to access portable test for visual field (VF) screening which could be used by clinicians in everyday clinical practice. Our study aimed to determine the validity of an innovative screening grid test for identifying neurological disease associated VF defects.

**Methods:** We enrolled two groups of participants: We assessed the one eye of 10 consecutive adult patients with different types of neurological disease associated VF defects and 10 eyes of controls in each group. The TS grid test was performed in each group. Sensitivity, specificity, and positive and negative predictive values of the TS grid scotoma area were assessed using the 24–2 VF Humphrey Field Analyser (HFA) as the reference standard.

**Figure:**
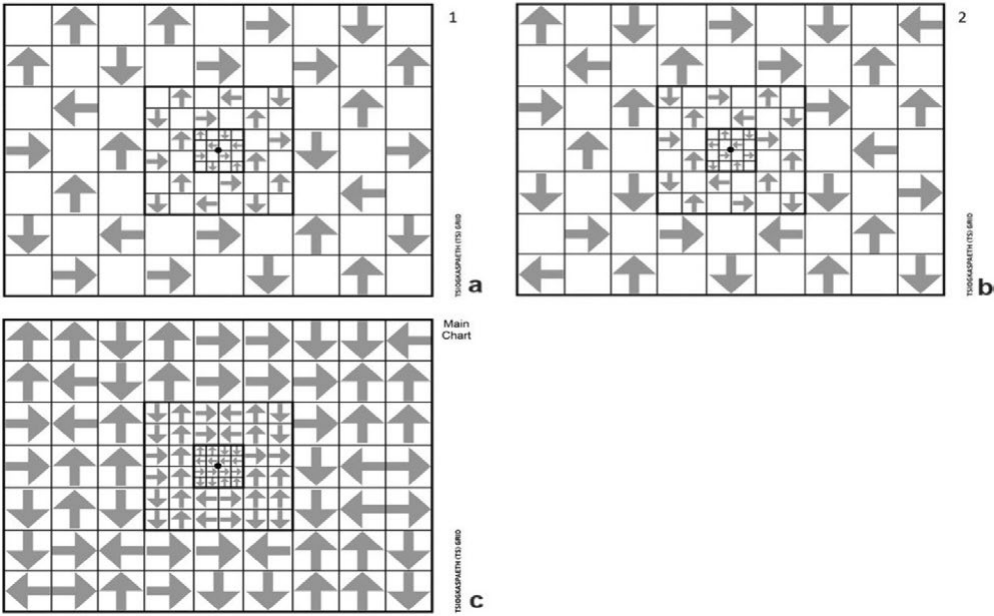
**FIGURE 1** TS grid 1 (a), TS grid 2 (b) and TS grid Main Reference Chart (c) for the clinical examination

**Results:** Sensitivity and specificity of the TS grid test were 100% and 90.91% respectively. The Area Under Curve was 0.9545 with 95% CI 0.87‐1.00. There was a significant correlation between the number of missed locations on the TS grid test and the Visual Field Index of the HFA 24‐2 (r = 0.9436, P < .0001).

**Figure:**
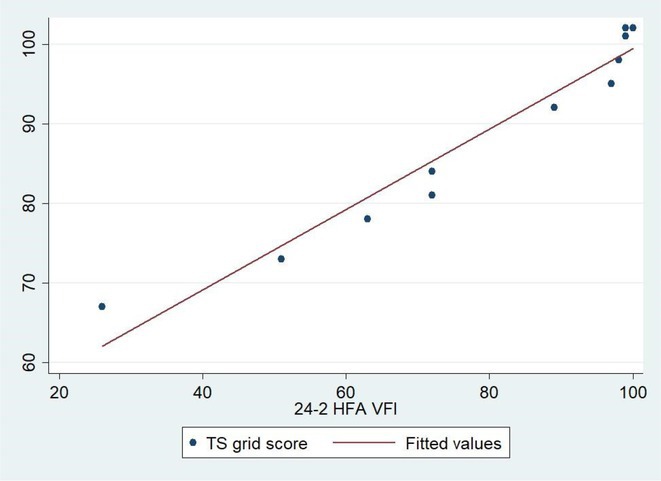
**FIGURE 2** Scatter plots of The TS grid score and HFA 24‐2 visual field parameters

**Figure:**
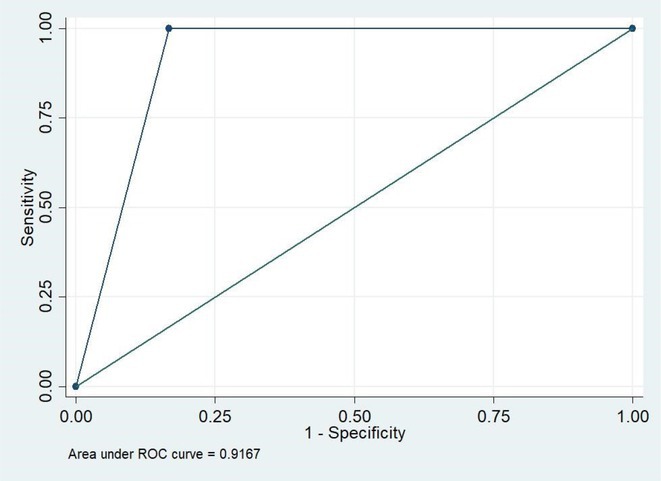
**FIGURE 3** TS grid ROC curve (blue line) and HVA 24‐2 ROC curve (green line) for detection of neurological disease associated VF defects

**Conclusion:** The sensitivity and specificity of the TS grid test were high in detecting VF defects in neurological disease. The TS grid test appears to be a reliable, low cost and easily accessed alternative to traditional VF tests in diagnosing typical neurological patterns of visual field defects. It would be useful in screening subjects for neurologically derived ocular morbidity in everyday clinical practice and in remote areas deprived of specialized health care services.

**Disclosure:** Nothing to disclose.

## EPO‐324

### Correlation between cerebral vasoreactivity and retinal vessel density in patients with internal carotid artery stenosis

#### A. Gaál ^1^; R. Stang^1^; H. Pál^1^; B. Csányi^2^; L. Illés^3^; Z. Mihály^4^; Z. Czinege^4^; T. Horváth^5^; D. Bereczki^1^; I. Kovács^3^; R. Debreczeni^1^

##### ^1^Semmelweis University, Department of Neurology; ^2^Semmelweis University, Doctoral College; ^3^Semmelweis University, Department of Ophtalmology; ^4^Semmelweis University, Deparmetment of Vascular and Endovascular Surgery; ^5^University of Physical Education, Research Center for Sport Physiology

**Background and Aims:** The retinal and cerebral circulation are developmentally, anatomically, and physiologically similar. We aimed to investigate the relationship between cerebral and retinal circulation in patients with atherosclerotic Internal Carotid Artery (ICA) stenosis.

**Methods:** 24 patients with significant ICA stenosis were consecutively enrolled. Cerebrovascular reactivity was estimated from the change in ipsilateral MCA blood flow velocity (measured by TCD) and resistance to the common carotid artery compression (CCC) test. Cerebral Arterial Resistance Transient Hyperemic Response Ratio ‐ CAR‐THRR (the change of flowresistance after CCC relative to the baseline value) and CARAUC (area under curve of CAR response to CCC) were calculated. Optical Coherence Tomography Angiography (OCTA) was performed to determine the vessel density (VD) on the papilla (P) whole image (WI) and in the peripapillary (PP) region for all vessel types (VDP‐WIall) and selectively for small vessels (VDP‐WIsmall) only.

**Results:** A significant, negative correlation was found between CAR‐THRR, CARAUC and VDPPsmall vessel type (*p* = 0.003; Spearman's *r* = −0.57), (*p* = 0.002; Spearman's *r* = −0.56), as well as between VDPPall vessel types (*p* = 0.01; Spearman's *r* = −0.48), (*p* = 0.03; Spearman's *r* = −0.46). There was also a significant, negative correlation between CAR‐THRR, CARAUC and VDP‐WIsmall (*p* = 0.01; Spearman's *r* = −0.52), (*p* = 0.01; Spearman's *r* = −0.54) and between VDP‐WIall (*p* = 0.02; Spearman's *r* = −0.45), (*p* = 0.006; Spearman's *r* = −0.47) too.

**Conclusion:** The study showed a significant correlation between decreased cerebrovascular reactivity and retinal functional vessel density in patients with ICA stenosis suggesting common mechanisms of action. The combined use of OCTA and TCD was found to be suitable for assessing the condition of cerebral vasculature in significant ICA stenosis.

**Disclosure:** Nothing to disclose.

## EPO‐325

### Association between stroke pattern and prior anticoagulant/antiplatelet use: A retrospective study

#### B. Yaralıoğlu ^1^; C. Aktan^1^; B. Kılboz^1^; A. Özer^1^; M. Karahoca^1^; Z. Arat^2^; O. Akan^1^

##### ^1^Department of Neurology, University of Health Sciences, Prof. Dr. Cemil Tascioglu City Hospital, Istanbul, Turkey; ^2^Department of Statistics, University of Health Sciences, Prof. Dr. Cemil Tascioglu City Hospital, Istanbul, Turkey

**Background and Aims:** The association between the structure of thrombus or thrombus source and infarct lesion patterns on diffusion‐weighted imaging (DWI) is a subject of interest. However, it is even more interesting whether the use of antiaggregants or anticoagulants affects the infarct pattern by changing thrombus formation. Our aim was to investigate the relation between prior antiplatelet or anticoagulant use and infarct patterns.

**Methods:** We retrospectively examined 938 stroke patients with etiologies including cardioembolic, small vessel disease (SVD), large‐artery atherosclerosis (LAA) using DWI. Infarct patterns were categorized as single cortical, medium/large corticosubcortical, subcortical less/more than 15 milimeters, borderzone, confluent and additional, small‐scattered lesions. Prior medication use were categorized as anticoagulant, antiplatelet, both, none. The association between infarct pattern and prior anticoagulant/antiplatelet use was investigated (Table 1).

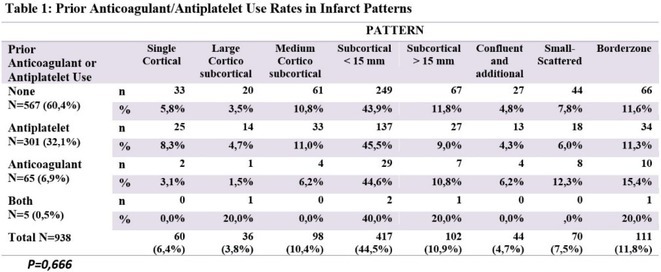

**Results:** Cardioembolic infarctions are known to cause small‐scattered, confluent and additional lesions as well as large single infarctions. Although anticoagulant/antiplatelet use was considered to affect thrombus structure, it was not found to be associated with a specific infarction pattern in our study. Small‐scattered and confluent were higher, large corticosubcortical lesions were lower in the anticoagulant group. It may be due to the fact that the patients with cardioembolic etiology were chosen from the ESUS/PAF+ subgroup.

**Conclusion:** Contrasting the literature, prior anticoagulant/antiplatelet use was not found to be associated with a specific infarction pattern, and may be explained by their etiology. To better understand the effects of anticoagulant/antiplatelet on the clotting mechanism and its association with infarct patterns, it may be useful to include patients not only ESUS/PAF also with known AF.

**Disclosure:** Nothing to disclose.

## EPO‐326

### Synthetic data integration in neurological research: Enhancing analysis accuracy and data privacy with EPICOSAI

#### E. Kanik

##### MedicReS, Quasar, İstanbul, Turkey

**Background and Aims:** Neurological research faces challenges such as data privacy concerns, small sample sizes, and complex datasets. Synthetic data offers a scalable solution, preserving confidentiality while replicating real‐world data. The EPICOSAI platform integrates Monte Carlo simulations with statistical distributions—normal, binomial, Bernoulli, and exponential—to generate synthetic datasets. This study explores EPICOSAI's potential to address data limitations and improve neurological research outcomes.

**Methods:** Key statistical properties demographic data and clinical variables were extracted from real datasets to guide synthetic data generation. Monte Carlo simulations modeled continuous variables (e.g., age, mean = 65 years, SD = 7 years) using the normal distribution, binary outcomes biomarker presence, 60%) using the binomial distribution, and time‐to‐event data (e.g., relapse time, mean = 2.5 years) using an exponential distribution. Validation confirmed fidelity, with less than 5% deviation in key metrics such as mean and variance.

**Results:** Synthetic datasets expanded sample sizes from 100 to 10,000 patients, enabling robust modeling of rare neurological conditions. Machine learning models trained on synthetic data achieved 90–95% accuracy, comparable to real data models. These datasets facilitated simulations of clinical scenarios, such as treatment efficacy in Alzheimer's disease, overcoming real‐world data limitations.

**Conclusion:** EPICOSAI provides scalable, privacy‐preserving synthetic data solutions that address critical challenges in neurological research. By ensuring data reliability and enabling advanced analyses, EPICOSAI supports impactful and ethical studies, establishing itself as a transformative tool in neurology.

**Disclosure:** The authors acknowledge EPICOSAI for providing the tools and resources necessary for generating synthetic data and conducting the analyses presented in this study.

## EPO‐327

### Diagnostic value of the ‘insular knife‐cut’ Sign in patients with suspected herpes simplex virus encephalitis

#### S. Marini^1^; G. Greco ^2^; M. Usai^3^; S. Falso^1^; D. Turilli^4^; S. Othmani^3^; R. Meloni^3^; P. Businaro^2^; M. Puci^5^; M. Marini^1^; A. Pichiecchio^6^; M. Paoletti^6^; P. Zara^3^; S. Masala^4^; G. Sotgiu^5^; M. Gastaldi^6^; P. Solla^3^; R. Iorio^1^; E. Sechi^3^

##### ^1^Department of Neuroscience, Catholic University of the Sacred Heart, Rome, Italy; ^2^Department of Brain and Behavioural Sciences, University of Pavia, Pavia, Italy; ^3^Neurology Unit, University Hospital of Sassari; Sassari, Italy; ^4^Radiology Unit, University Hospital of Sassari; Sassari, Italy; ^5^Clinical Epidemiology and Medical Statistics Unit, University Hospital of Sassari; Sassari, Italy; ^6^Neuroradiology Department, IRCCS Mondino Foundation, Pavia, Italy

**Background and Aims:** The “insular knife‐cut” sign, a sharp demarcation between insular FLAIR abnormalities and basal ganglia on axial images, is associated with herpes simplex virus encephalitis (HSVE). We assessed its prevalence and diagnostic accuracy in a cohort of patients with suspected HSVE.

**Methods:** A multi‐center, retrospective cohort of patients with suspected HSVE was carried out. Inclusion criteria were: (1) CSF sample tested with polymerase chain reaction for HSV‐1/2; and (2) acute brain MRI. We evaluated the relationship between insular knife‐cut sign and other clinical and radiological variables.

**Results:** A total of 188 patients were selected: 44 with HSVE, and 144 with alternative diagnoses (51 with autoimmune encephalitis; 22 with infectious encephalitis; 71 with other acute encephalopathies). The insular knife‐cut sign was found in 53% cases of HSVE and in 1% with alternative diagnoses (*p* < 0.001) at baseline. Specificity and sensitivity of the sign were 99.3% (95% CI, 96–100) and 52% (95% CI, 38–66), respectively. In eight HSVE patients the insular knife‐cut sign appeared on the subsequentMRIsobtained acutely, increasing sensitivity to 70.5% (95% CI, 56–82). On multivariate regression, the insular knife‐cut sign was the strongest independent predictor (odds ratio [95% CI]) of HSVE (42.4 [7.3–486.4]), followed by temporal pole involvement (12.9 [3.7–54.6]), abnormal brain MRI (7.4 [1.6–34.3]), and CSF pleocytosis (4.9 [1.4–19.4]).

**Conclusion:** Detection of the insular knife‐cut sign on MRI strongly predicts HSVE diagnosis in patients with suspected acute encephalitis. This marker could help early diagnosis and treatment of HSVE, especially when other diagnostic tests are equivocal/unavailable.

**Disclosure:** Nothing to disclose.

## EPO‐328

### Detection of microembolic signals in patients with carotid web

#### I. Alcobendas Liern; J. Botía Barberá; B. Lucio Ceballos; A. García Pastor; A. Iglesias Mohedano; M. Vales Montero; F. Díaz Otero; P. Vázquez Alén; Y. Fernández Bullido; A. Gil Nuñez

##### Neurology, Hospital General Universitario Gregorio Marañón, Madrid, Spain

**Background and Aims:** The mechanism by which the carotid web (CW) produces ischemic strokes is still not well understood. We describe the clinical characteristics and results of microembolic signal (MES) recording in a series of patients with CW and ischemic stroke or transient ischemic attack (TIA).

**Methods:** Patients admitted in our hospital between 2020 and 2024 with ischemic stroke or TIA and CW were identified. 60 minutes MES monitoring with transcranial Doppler (TCDX, Atys medical) was performed in MCA ipsilateral to CW.

**Results:** We included 9 patients, 5 women, 4 men. Median age 62 years (IQR 53 ‐ 74). Six patients suffered an ischemic stroke and three a TIA. CW was bilateral in 3 patients, ipsilateral to the vascular event in 3 and contralateral (asymptomatic) in 3 (table 1). MES monitoring was positive in three patients, all women. In two of the three MES+ cases, the CW was ipsilateral to the event and contralateral in the remaining case (patient with atrial fibrillation). The etiologic diagnosis at discharge was undetermined in 5 cases, cardioembolic in two, and small vessel in two (table 2). Carotid revascularization was not performed in any patient. With a median follow‐up of 8 months, no recurrences were recorded.

**Figure:**
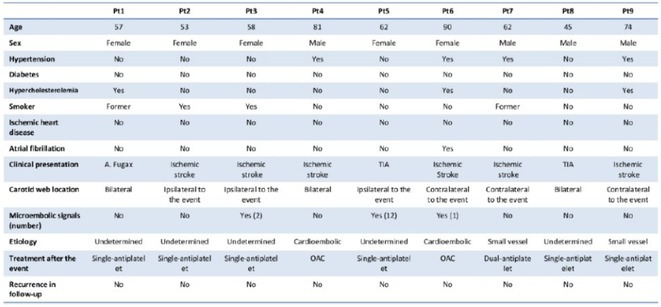
**FIGURE 1** Characteristics of included patients

**Figure:**
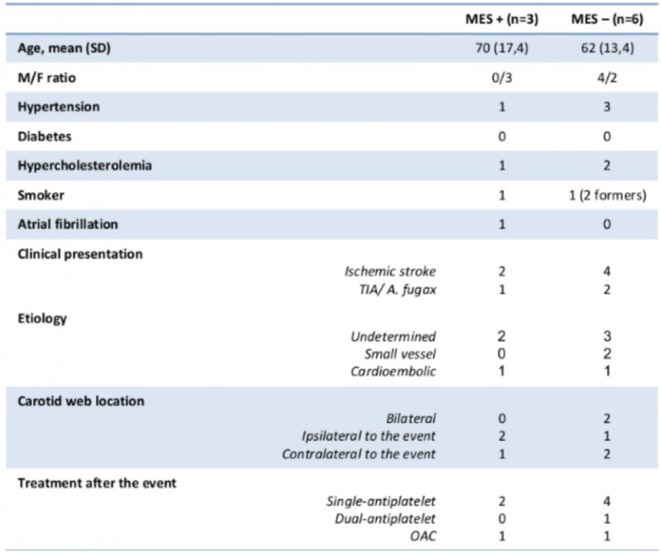
**FIGURE 2** Characteristics according to the presence or absence of MES

**Figure:**
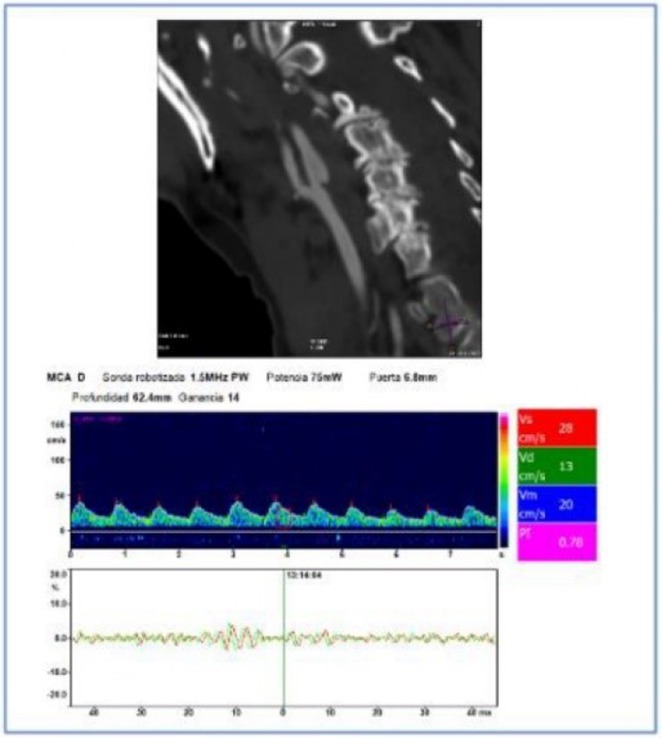
**FIGURE 3** CTA showing a CW in right ICA. MES (9 dB, 17 ms) detected during right MCA monitoring

**Conclusion:** Previous studies have described a high recurrence risk in patients with CW, which contrasts with what was observed in this series. It is difficult to establish a causal relationship between CW and ischemic stroke. MES monitoring with TCD could help to better understand the mechanism of cerebral ischemia in patients with CW.

**Disclosure:** Nothing to disclose.

## EPO‐329

### MRI features of central nervous system involvement in 104 Moroccan patients with Neuro‐Behcet's syndrome

#### J. Oumerzouk

##### Military Hospital Mohamed V of Rabat, Rabat, Morocco

**Background and Aims:** Behçet's disease (BD) is a recognized as a chronic relapsing heterogeneous multisystem inflammatory disorder and a vasculitis of unknown origin which has a peculiar epidemiology. Neurologic involvement has been reported to be less common than other systemic manifestations, but can cause substantial disability.

**Methods:** This is a retrospective study of 104 patients presenting with BD and neurological involvement, collected over a period of 23 years. The MR images of patients with BD, associated with neural parenchymal involvement in 86 cases (82.7%) and CVT in 18 cases (17.3%) were reviewed.

**Results:** Concerning parenchymal CNS pattern, brain MR imaging (MRI) showed hypointense lesions on T1‐weighted and hyperintense lesions on T2 and FLAIR‐weighted images, involving mostly the brainstem (84.8%), basal ganglia (68.6%), cerebral white matter (53.4%), internal capsule (47.6%) and thalamus (46.5%). For the extraparenchymal pattern, the brain MRI results of our series showed that the venous involvement was isolated superior sagittal sinus (SSS) thrombosis in 8 patients (44.4%), isolated lateral sinus (LS) thrombosis in 4 patients (22.2%), and concomitant SSS and right lateral sinus thrombosis in 3 patients (16.6%).

**Conclusion:** Brain MRI study including contrast and MR angiography (MRA) especially MR venography (MRV), are mandatory in categorizing the neurological involvement and both should be used routinely in cases of suspected NBD.

**Disclosure:** Nothing to disclose.

## EPO‐330

### Altered hippocampal volume in patients with cognitive impairment associated with post‐COVID‐19 condition

#### L. Adam ^1^; A. Dell'Orco^2^; M. Steinbrenner^1^; D. Steinbart^1^; L. Pechstein^1^; M. Scheel^2^; C. Franke^1^

##### ^1^Department of Neurology and Experimental Neurology, Charité Universitätsmedizin Berlin, Berlin, Germany; ^2^Institute for Neuroradiology, Charité Universitätsmedizin Berlin, Berlin, Germany

**Background and Aims:** The post‐acute sequelae of the SARS‐CoV‐2 infection are known as Long COVID or Post‐COVID‐19 Condition (PCC) and often manifests neurocognitive symptoms. Understanding the mechanisms driving this cognitive impairment is crucial for accurately characterizing the disease and developing effective therapeutic interventions. This study aims to investigate volumetric changes with subfield hippocampal analysis in PCC patients, building on prior evidence of structural brain alterations to better understand the neuroanatomical basis of cognitive impairment.

**Methods:** A dataset of 60 age‐ and sex‐matched PCC patients and 60 healthy controls (HC) was analyzed, with all PCC patients reporting cognitive deficits and reduced memory satisfaction based on MMQ scores. MRI scans were performed on all participants using a standardized protocol, and the data were processed with Freesurfer for hippocampal and amygdala subfield segmentation. Statistical analysis of hippocampal ROIs was conducted using ANCOVA, controlling for age, sex, and total intracranial volume, with false discovery rate (FDR) adjustments applied to *p*‐values. Multiple regression was employed to explore the relationships between volumetric brain measurements, neurocognitive performance, and the presence of autoantibodies in the cerebrospinal fluid of PCC patients.

**Results:** Significant volume reductions were observed in the hippocampal body, particularly in the CA4, subiculum, and molecular layer bilaterally, with FDR‐adjusted significance retained for the left CA3, dentate gyrus, and hippocampal fissure. No alterations were detected in the hippocampal head. Further modeling of imaging and clinical data is currently being conducted.

**Conclusion:** Reduced volume in several hippocampal body ROIs was found in PCC patients, suggesting susceptibility of the hippocampus to neuroinflammation following SARS‐CoV‐2 infection.

**Disclosure:** Nothing to disclose.

## EPO‐331

### Decision‐making made easy: Algorithmic approach to fetal and neonatal ventriculomegaly

#### R. Agarwal; A. Venkatesh; J. Gadupati; A. Jain

##### Ramaiah Medical College, Bengaluru, India

**Background and Aims:** Ventriculomegaly (VM) is a common neurodevelopmental anomaly characterized by cerebral ventricular enlargement. As a non‐specific sonographic finding, VM may be associated with a range of pathological and genetic conditions, making accurate diagnosis essential for appropriate prognosis and management. We aim to improve the differential diagnosis of VM by utilizing a structured, algorithmic approach which involves a step‐by‐step algorithmic framework that categorized etiology of VM based on imaging characteristics.

**Methods:** The diagnostic algorithm was developed through a systematic review of literature, analysis of clinical cases, and expert consultations. Imaging data from ultrasonography and magnetic resonance imaging (MRI) formed the basis for the algorithm's structured diagnostic flow. Key diagnostic steps included classifying the severity of VM, assessing associated anomalies, and categorizing potential etiologies. Validation was performed retrospectively on ten clinical cases, comparing algorithmic diagnoses with established clinical and imaging findings.

**Figure:**
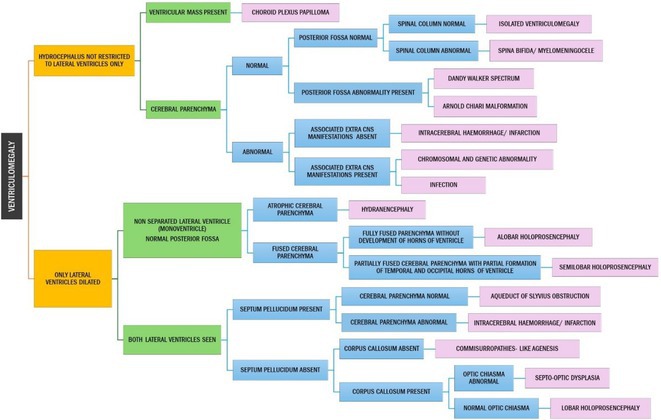
**FIGURE 1** Designed Algorithm for Fetal and Neonatal Ventriculomegaly

**Results:** The algorithm facilitated precise identification of various etiologies of VM, including semilobar holoprosencephaly, Dandy‐Walker malformation, intracerebral infarction, choroid plexus papilloma, corpus callosal agenesis, congenital infections (e.g., toxoplasmosis, cytomegalovirus), and congenital muscular dystrophy. Each diagnosis aligned with clinical and imaging findings, underscoring the algorithm's reliability.

**Conclusion:** This algorithmic approach offers a structured, evidence‐based tool for the differential diagnosis of VM, enabling clinicians to navigate its complex etiological spectrum. By integrating detailed imaging analysis with clinical findings, the algorithm supports early and accurate diagnosis, improves prognostication, and facilitates timely interventions, ultimately optimizing outcomes for affected neonates, fetuses, and their families.

**Disclosure:** Nothing to disclose.

## EPO‐332

### Is the T1‐w/T2‐w marker affected by iron deposition in patients with secondary progressive multiple sclerosis?

#### S. Gaździński ^1^; N. Hryniewicz^2^; E. Piątkowska‐Janko^3^; P. Kazulo^3^; K. Lipiński^3^; A. Karlińska^1^; Ł. Smoliński^4^; B. Błażejewska‐Hyżorek^4^; P. Bogorodzki^3^; P. Grieb^5^; M. Pawlak^6^; R. Rola^1^; D. Ryglewicz^1^; I. Kurkowska‐Jastrzębska^4^

##### ^1^Military Institute of Aviation Medicine, Warsaw, Poland; ^2^Nałęcz Institute of Biocybernetics and Biomedical Engineering, Polish Academy of Sciences, Warsaw, Poland; ^3^Warsaw University of Technology, Warsaw, Poland; ^4^Institute of Psychiatry and Neurology, Warsaw, Poland; ^5^Mossakowski Medical Research Centre, Polish Academy of Sciences, Warsaw, Poland; ^6^Poznan University of Medical Sciences, Poznań, Poland

**Background and Aims:** MRI and histological studies have shown altered brain iron levels in the brains of patients with multiple sclerosis (MS). Quantitative Susceptibility Mapping (QSM) provides quantitative distribution of susceptibility sources in tissue, especially iron in ferritin or deoxygenated hem. The ratio of T1‐w/T2‐w images is a marker of microstructural damage in MS that might be affected by paramagnetic ions in the tissue.

**Methods:** 22 patients with a diagnosis of secondary progressive MS (EDSS: 6.1 ± 1.1, 53.3 ± 8.1 years, 9M, duration of disease 18.3 ± 7.5 years) with various load of hypointense lesions on T1 and without enhancing lesions on MRI were included. High resolution T1‐w and T2‐w images were obtained at 3T. For QSM, a 3D multi‐echo gradient‐echo sequence (SWAN) was obtained (1 × 1 × 2 mm 39 TE values ranging from 4.5 to 48 ms). Susceptibility maps were calculated with MEDI. Regions of Interest (ROI) were outlined using SAMSEG.

**Results:** 12 patients had regions of increased susceptibility (Figure), among others 3 had rims (Figure 1), suggesting the presence of smoldering lesions. The corresponding mean values of T1‐w/T2‐w and susceptibility did not correlate in any ROI (e.g., Figure 2 and Figure 3).

**Figure:**
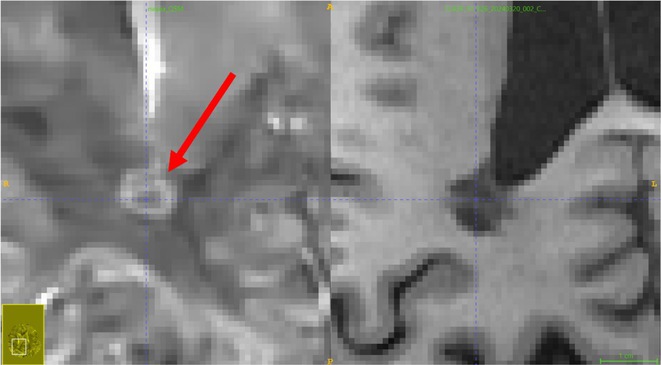
**FIGURE 1** Exemplary rim on QSM suggesting presence of a smoldering lesion and corresponding anatomy on T1‐w image.

**Conclusion:** The T1‐w/T2‐w ratio does not appear to be affected by tissue paramagnetic biometals, such as iron, associated with inflammatory states.

**Figure:**
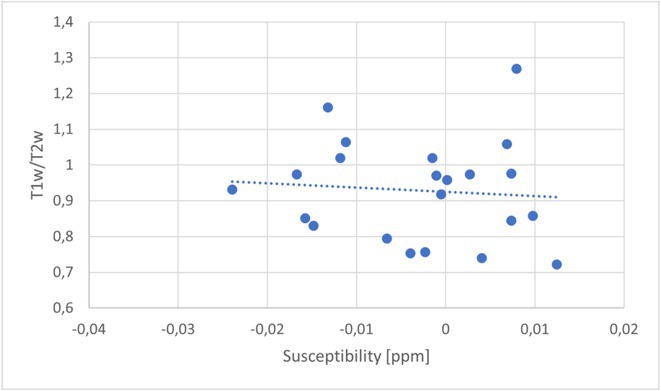
**FIGURE 2** No correlation in White Matter Signal Hyperintensities.

**Figure:**
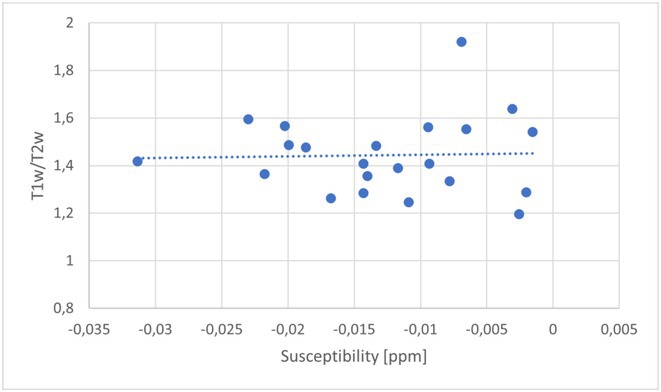
**FIGURE 3** No correlation in Normal Appearing White Matter.

**Disclosure:** Nothing to disclose. This study was supported by Medical Research Agency of Poland, grant 2021/ABM/02/00002‐00.

## EPO‐333

### Clinical and radiological correlations in patients with generalised epilepsy: A single center f‐MRI study

#### A. Giordano ^1^; M. Siciliano^1^; R. Matrullo^1^; F. Esposito^2^; M. Cirillo^2^; A. Tessitore^1^

##### ^1^Department of Advanced Medical and Surgical Sciences, University of Campania “Luigi Vanvitelli”, Naples, Italy; ^2^MRI Research Center SUN‐FISM, University of Campania “Luigi Vanvitelli”, Naples, Italy

**Background and Aims:** In patients with generalized epilepsy (PwGE) there is a bidirectional correlation between seizure outcome and comorbidities .The aims of the study are: a) to analyze the clinical features of a sample of PwGE b) to investigate the functional connectivity by using resting state (rs‐f‐MRI) c) to highlighted clinical and (functional) radiological correlations.

**Methods:** we enrolled 31 PwGE, 16 SFp and 15 DREp (3), and 15 age‐gender‐matched healthy controls (HCs). We used an ANOVA to compare the groups of PwGE and HCs on demographic, clinical, cognitive, and behavioural features. Moreover, we compared by one‐way ANOVA SFp and DREp subgroups with each other and the HCs. MRI at 3 Tesla was collected in all PwGE and HCs.

**Results:** DREp compared with SFp showed executive disfunction and behavioural abnormalities scoring worse to TMT and semantic fluency test, epitrack and apathy evaluation scale (AES). Moreover in DREp a reduction of functional connectivity of the limbic network (LN) and an increased functional connectivity of the salience network (SN) was detected. Correlation analyses showed a negative correlation between the scores to TMT test and LN functional connectivity; thus functional connectivity changes could represents a compensatory event to executive deficits in PwGE. Conversely a positive correlation between AES and SN connectivity was observed; this could represents an ineffective attempt to apathy.

**Figure:**
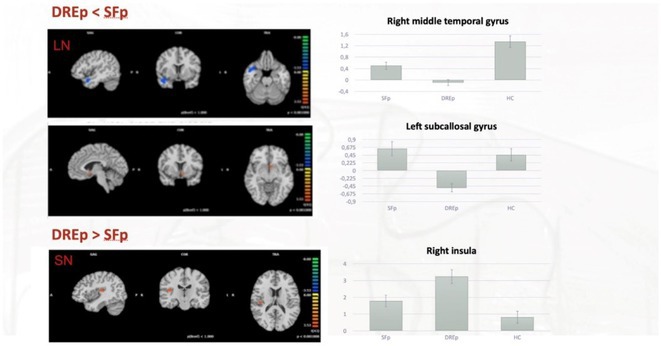
**FIGURE 1** MRI functional connectivity abnormalities in patients with treatment‐resistant generalized epilepsy (DREp) compared with seizure‐free patients (SFp)

**Figure:**
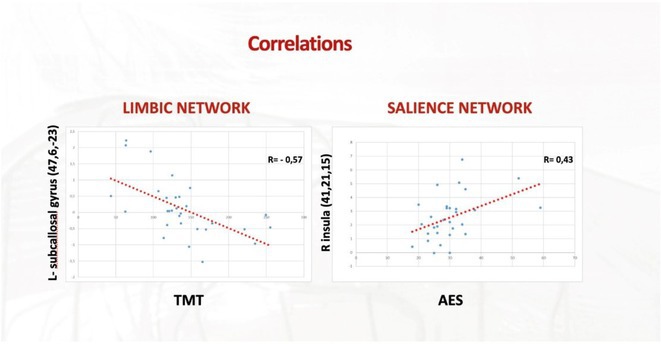
**FIGURE 2** Clinical and radiological correlations

**Conclusion:** In our single center rs‐fMRI study we confirmed both a bidirectional correlation between seizures outcome and cognitive/behavioural comorbidities and functional connectivity abnormalities between DREp and SFp. Moreover interesting clinical and radiological correlation were observed.

**Disclosure:** Nothing to disclose.

## EPO‐334

### AI‐detected brain atrophy pattern associated with progression independent of relapse activity in multiple sclerosis

#### V. Rinaldi ^1^; G. Moltoni^2^; L. Le Mura^1^; A. Romano^2^; M. Buscarinu^1^; M. Salvetti^1^; A. Bozzao^2^

##### ^1^Neurology Unit, Department of Neurosciences, Mental Health and Sensory Organs (NESMOS), Sapienza University of Rome, Rome, Italy; ^2^Neuroradiology Unit, Department of Neurosciences, Mental Health and Sensory Organs (NESMOS); Sapienza University of Rome, Rome, Italy

**Background and Aims:** Progression independent of relapse activity (PIRA) represents the main driver of clinical disability accrual in multiple sclerosis (MS). Brain atrophy is the MRI outcome that better correlates with disease progression, however it is difficult to detect at a subject‐level in the clinical practice. Also, its functional correlate still remains uncertain. The primary goal of this study was to identify the brain atrophy pattern (BAP) associated with PIRA using Artificial Intelligence (AI); secondarily, we aimed to assess the resting state functional connectivity (RS‐FC) correlated to this pattern and its implications on clinical performance.

**Methods:** We included MS‐patients treated with Natalizumab with PIRA (*n* = 14) and clinically stable (CS) (*n* = 10) disease. Patients underwent a motor/cognitive evaluation (EDSS/SDMT) and a brain MRI (MPRAGE,3D‐FLAIR,RS‐fMRI). Brain volumetrics was analyzed through an AI software (Pixyl) detecting global/regional atrophy. Comparisons of clinical/MRI volumetric measures between PIRA and CS groups were performed. We then assessed the RS‐FC of PIRA‐associated BAP through a seed‐based analysis using CONN‐toolbox.

**Results:** PIRA group showed a higher patients proportion with global/total WM atrophy than CS. No differences were found for total GM, however when analyzing the single GM regions, PIRA group showed a greater patients percentage with atrophy (88% vs. 40%; *p*‐value = 0.03241) of the left thalamus compared to CS. Patients with left thalamus atrophy (leftTA) showed a RS‐FC alteration of the Default Mode Network(DMN) and a lower SDMT‐score than those without leftTA.

**Conclusion:** AI enables to identify BAP associated to clinical phenotypes. PIRA is associated with leftTA, which correlates to a RS‐FC alteration of the DMN, possibly linked to decreased cognitive performance.

**Disclosure:** Rinaldi V. has nothing to disclose Moltoni G. has nothing to disclose Le Mura L. has nothing to disclose Romano A. has nothing to disclose Buscarinu C. has nothing to disclose Salvetti M. has nothing to disclose Bozzao A. has nothing to disclose.

## Neuro‐oncology

## EPO‐335

### A rare neurological immune‐related adverse event in a melanoma patient

#### A. Montalvo ^1^; A. Sousa^2^; L. Albuquerque^3^

##### ^1^Neurology Service, Department of Neuroscience and Mental Health, Local Health Unit of Santa Maria, Lisbon, Portugal; ^2^Oncology Service*,* Local Health Unit of Santa Maria, Lisbon, Portugal; ^3^Egas Moniz Study Center, University Neurology Clinic, Faculty of Medicine, University of Lisbon, Lisbon, Portugal

**Background and Aims:** Immune checkpoint inhibitors (ICI) are used as immunotherapy in different neoplasms. Neurological immune‐related adverse events (nirAE) are rare. Of these, headache/hypophysitis (2–9%), neuromuscular disorders (0.5–2.5%), encephalitis (0.5–1%), meningitis (<0.5%) and demyelinating disease (<0.5%) are usually considered. Symptoms typically present 6‐13 weeks after treatment initiation.

**Methods:** Case report.

**Results:** A 63‐year‐old woman with metastatic melanoma to the lymph nodes and lung (BRAF+) started nivolumab and ipilimumab. After 2 sessions, a rash, thyroiditis and grade 3 autoimmune hepatitis were identified. ICI were suspended and oral corticosteroids were administered for 8 weeks. Fourteen weeks after ICI start (11 weeks after stopping), the patient reported unsteadiness, and isolated gait ataxia was observed. Brain MRI showed multiple punctiform hyperintensities (1–5 mm size) in dark‐blood and T2/FLAIR with gadolinium enhancement. Lumbar puncture revealed 13 lymphocytes/mm^3^, without neoplastic cells, oligoclonal bands or antineuronal antibodies. Infectious serologies, MOG and AQP4 antibodies were negative. Clinical improvement was observed after another 6 weeks of corticosteroids. Melanoma treatment was switched to BRAF/MEK inhibitors. Brain MRIs showed a rapid regression of contrast enhancement and of most previous lesions (last MRI 12 months after symptom onset).

**Conclusion:** In this case, we hypothesize a nir‐AE with multifocal and monophasic inflammatory/demyelinating lesions. Although brain metastases were the main differential diagnosis, concomitant lymphocytic meningitis and systemic immune‐related adverse events strongly support that diagnosis. Neurological complaints may have initially been masked by steroids for the first ir‐AEs.

**Disclosure:** Nothing to disclose.

## EPO‐336

### Bridging neurology and oncology: Unraveling the complexities of primary central nervous system lymphoma

#### A. Costa ^1^; S. Moreira^1^; A. Câmara^2^; M. Gomes^3^; C. Morgado^1^; S. Marques^1^; J. Ferreira Pinto^1^; F. Sousa^1^

##### ^1^Neurology Department, Local Health Unit of Braga, Braga, Portugal; ^2^Neurology Department, Funchal Central Hospital, Madeira, Portugal; ^3^Neurorradiology Department, Local Health Unit of Braga, Braga, Portugal

**Background and Aims:** Primary central nervous system lymphomas are rare and, usually, with very poor prognosis. In these cases, the most described subtype is diffuse large B cell lymphoma. We intend to explore the clinical presentations of such cases.

**Methods:** Description of cases diagnosed with this pathology observed in neurology hospitalization regimen.

**Results:** We obtained nine cases. Five were confirmed as primary central nervous system lymphomas. No biopsy was performed in the others. Considering the confirmed ones, the clinical presentation was variable: motor deficits or gait impairment if brain located ones; headaches and peripheral face palsy in brainstem located one and, finally, a cauda equina lymphoma presented with progressive paraparesis. The lymphoma subtype confirmed in all was diffuse large B cell lymphoma. Two of them were treated with high dose methotrexate, meanwhile the two most recent ones were treated with MATRIX regimen. As for the non‐confirmed cases, they presented with state of consciousness alterations, motor deficits or gait impairment. Three of them didn’t perform biopsy considering their poor neurological status; one due to its difficult location. The only treatment performed was corticosteroids. The first group had a mean lifetime between diagnosis and obit of 1.75 months, while the other had a mean of 4.75 months. The patient diagnosed with cauda equina lymphoma is still alive and regained autonomous walk.

**Conclusion:** Despite the small sample, these cases show the challenges regarding this diagnosis considering its phenotypic variability. The poor prognosis associated highlights the extreme importance of an early diagnosis, allowing timely beginning of the treatment.

**Disclosure:** Nothing to disclose.

## EPO‐337

### Acute neurological sequelae in leptomeningeal carcinomatosis cases

#### A. Basel Pala; B. Dagdeviren Boz; K. Isik; M. Acikgoz; U. Celebi; E. Aciman Demirel; H. Atasoy

##### Zonguldak Bulent Ecevit University Faculty of Medicine, Zonguldak, Turkey

**Background and Aims:** Leptomeningeal carcinomatosis (LMC) is a neurological complication of various systemic cancers.

**Methods:** The data were retrospectively collected from the hospital database with patient consent.

**Results:** A 60‐year‐old female patient with a diagnosis of breast cancer who was receiving pembrolizumab presented with speech impairment. On examination, her speech was dysarthric, and her gait was ataxic. Cranial MRI findings were normal except for cerebral atrophy. She was admitted with a preliminary diagnosis of autoimmune cerebellitis and leptomeningeal disease. Lumbar puncture (LP) was performed. CSF glucose was low, and microprotein and microalbumin levels were elevated. CSF cytology showed suspicious results. The patient received radiotherapy with a preliminary diagnosis of leptomeningeal involvement and immune‐related encephalitis. A 51‐year‐old female with a diagnosis of metastatic breast cancer presented with complaints of dizziness. Diffusion MRI revealed diffusion restriction, and she was subsequently admitted to the hospital. During follow‐up, her consciousness deteriorated. A lumbar puncture (LP) was performed. CSF glucose levels were low, and malignant cells were observed in the CSF cytology. Control MRI showed contrast uptake in the cerebellum. The diagnosis of leptomeningeal carcinomatosis was confirmed.

**Conclusion:** Headache is the most common symptom of leptomeningeal carcinomatosis, with various neurological signs present. CSF cytology and imaging techniques are crucial for early diagnosis.In patients with a history of breast cancer presenting with stroke‐like neurological symptoms, leptomeningeal carcinomatosis should be considered in the differential diagnosis.In patients with a history of breast cancer presenting with stroke‐like neurological symptoms, leptomeningeal carcinomatosis should be considered in the differential diagnosis.

**Disclosure:** Nothing to disclose.

## EPO‐338

### Description of 2 cases of intravascular large B‐cell lymphoma (IVLBCL) with CNS involvement: A diagnostic challenge

#### E. Ginés Murcia; C. Lapeña López; M. Warnken Miralles; P. Mahiques Ochoa; L. Moreno Navarro; M. Farrerons Llopart; D. López Ros; A. Benavent Rojas; L. Montero Pardo; M. Castaño Pérez; L. Ruiz‐Escribano Menchén

##### Neurology, Hospital General Universitario Doctor Balmis, Alicante, Spain

**Background and Aims:** IVLBCL is a rare form of lymphoma, characterized by intravascular proliferation of lymphomatous cells, obstructing small and medium‐sized vessels. Neurological signs are diverse but present in up to 2/3 of patients and generally accompanied by constitutional symptoms. There are no specific laboratory or radiological findings, and histopathological diagnosis is required, unfortunately with autopsy in many cases.

**Methods:** We retrospectively analysed data from 2 patients diagnosed with IVLBCL in Hospital General Universitario Doctor Balmis in 2024. 1. A 66‐year‐old woman, history of pulmonary tuberculosis 20 years ago. Constitutional symptoms and post‐infectious paraparesis. MRI showed inflammatory ADEM like lesions in the brain and conus medullaris. 2. A 68‐year‐old woman, anticoagulated atrial fibrillation. Recurrent hospital admissions with encephalopathy and focal signs. MRI showed multiterritorial diffusion‐restricted lesions.

**Figure:**
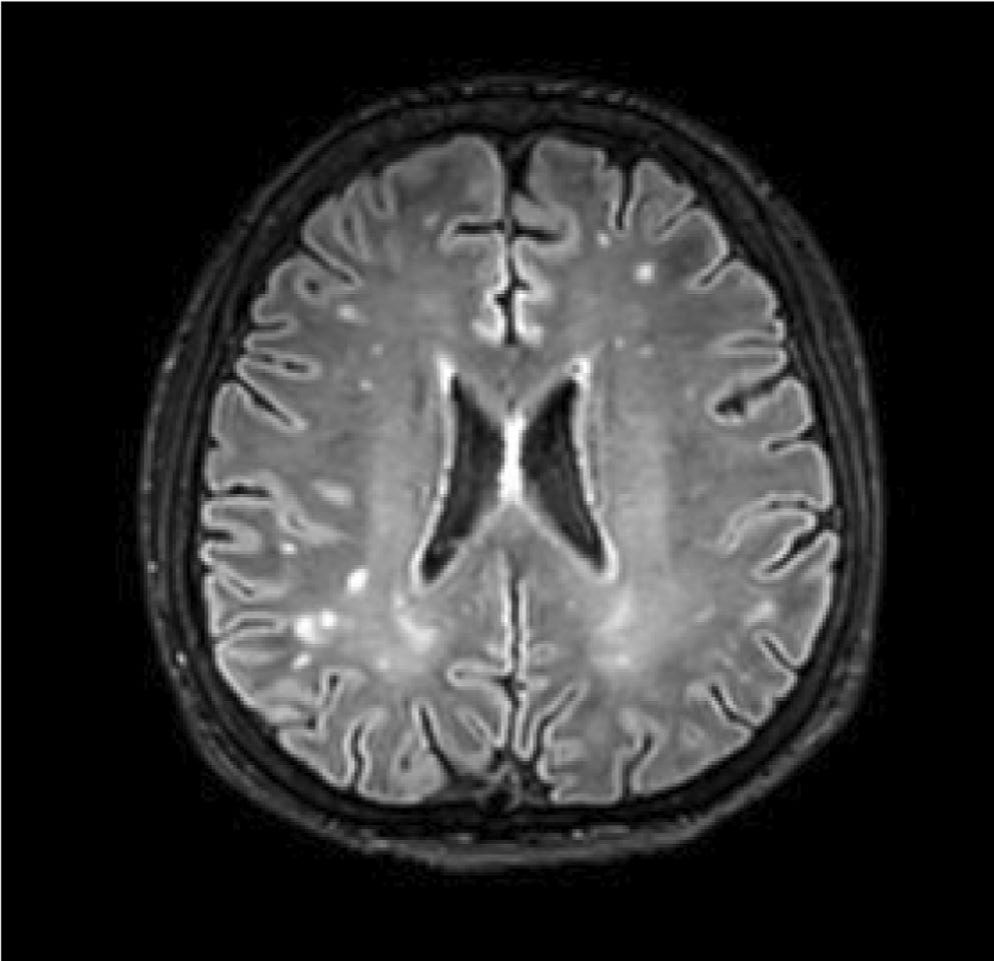
**FIGURE 1** Patient 1. Parenchymal lesions in both cerebral hemispheres, asymmetric, FLAIR hyperintense with an inflammatory appearance.

**Figure:**
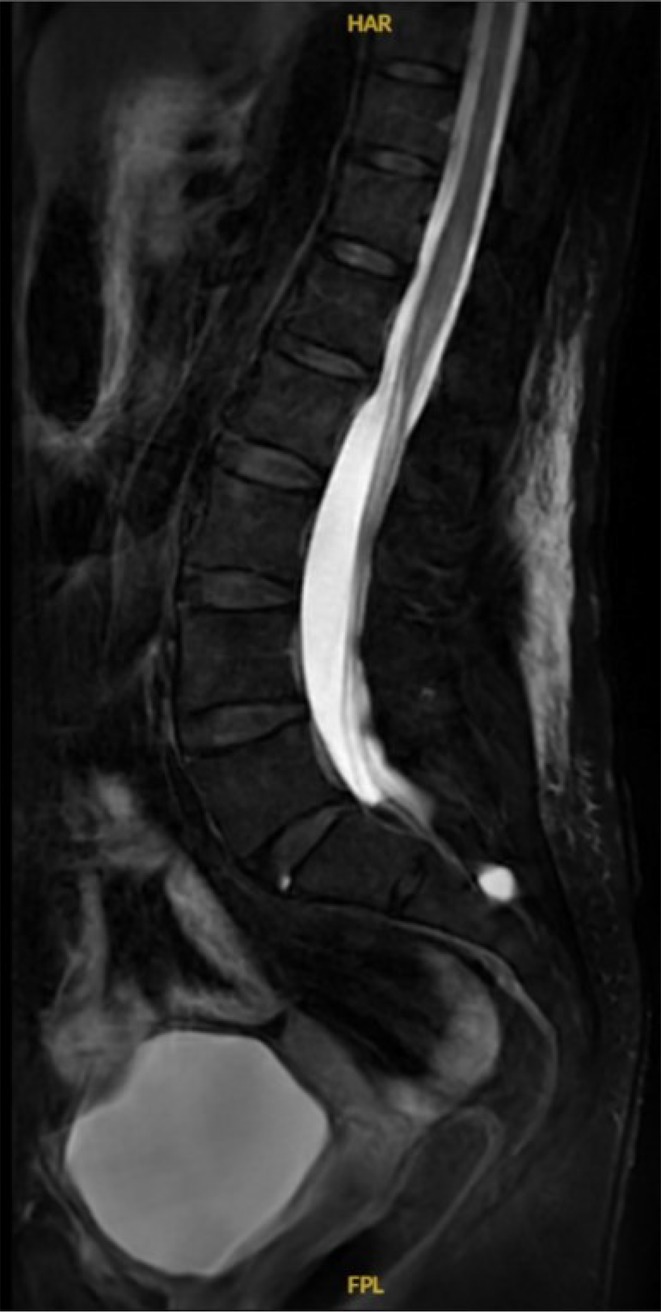
**FIGURE 2** Patient 1. Signal alteration of the conus medullaris, hyperintense on T2, involving the territory from T12 to L1, with central localization.

**Figure:**
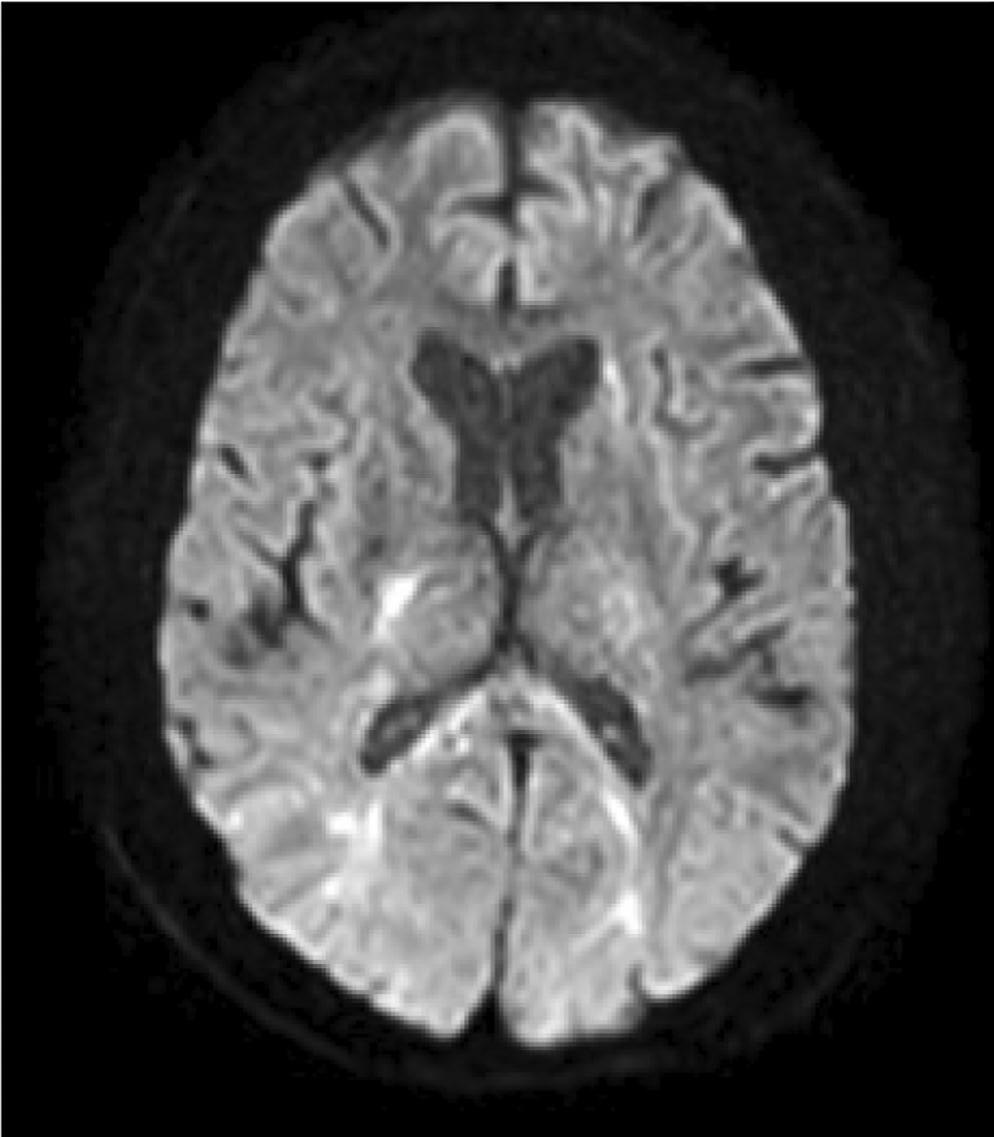
**FIGURE 3** Patient 2. Multiple areas of bilateral diffusion restriction, temporo‐occipital, in the corpus callosum and right basal ganglia, suggestive of ischemic foci of cardioembolic etiology.

**Results:** An extensive blood and CSF analitical study was negative (immunological, infectious, tumor markers, flow citometry…) in both. 1. CSF: mild hyperproteinorrachia. Body PET‐CT: chronic inflammatory lung changes. Clinico‐radiological improvement after high dose corticosteroids but worsening after tapering. New pulmonary infiltrates and mediastinal lymphadenopathy observed on PET‐CT. Lung biopsy confirmed the histopathological diagnosis of IVLBCL. 2. CSF: lymphocytic pleocytosis and mild hyperproteinorrachia. Body PET‐CT and vessel‐wall MRI showed no evidence of vasculitis. Brain biopsy of the right parietal lession confirmed the histopathological diagnosis of IVLBCL.

**Conclusion:** Neurologists must be aware of IVLBCL in patients with recurrent inflammatory or ischemic multifocal lesions with inadequate response to treatment. Early histopathological diagnosis and treatment are crucial in this fatal condition.

**Disclosure:** Nothing to disclose.

## EPO‐339

### Diagnosis and treatment of gestational hemangioblastoma: A systematic review of case reports and case series

#### Y. Hawas^1^; Y. Ali^2^; E. Salah^3^; A. Eltobgy^4^; K. Albakri^5^; H. Atwan ^5^

##### ^1^Faculty of Medicine, Tanta University, Gharbeya, Egypt; ^2^Faculty of Medicine, Zagazig University, Zagazig, Egypt; ^3^Faculty of Science, Ain Shams University, Cairo, Egypt; ^4^Faculty of Medicine, Al‐Azhar University for Girls, Cairo, Egypt; ^5^Faculty of Medicine, The Hashemite University, Zarqa, Jordan

**Background and Aims:** Tumor occurrence during pregnancy is rare, with breast cancer being the most common type. Hemangioblastomas in pregnancy, though less studied than meningiomas and gliomas, pose significant diagnostic and management challenges due to overlapping symptoms and the complexity of timing tumor resection. This study systematically reviews the clinical features, diagnostic implications, and maternal and fetal outcomes of hemangioblastoma in pregnant females, drawing on case reports and series.

**Methods:** A comprehensive search was conducted in PubMed, Scopus, WOS, and Cochrane databases from inception to November 2024. Screening identified 81 case reports and series papers, encompassing 116 pregnant females.

**Results:** The mean maternal age was 31.72 ± 6.27 years, and the mean gestational age at diagnosis was 20.18 ± 8.63 weeks. Among 116 cases, 42 patients were multigravida, and 58 had von Hippel‐Lindau syndrome. Hemangioblastomas were predominantly infratentorial (74 cases), with 62 central lesions, including 37 in the brain and 20 in the spine, primarily in the cerebellum (30 cases). Headache was the most common symptom (33 cases), followed by weakness (15 cases) and vomiting (14 cases). MRI was the primary diagnostic tool (83 cases). Obstetric complications were underreported, with a low mortality rate of one miscarriage and three preterm deliveries. Neurological outcomes improved in 50 patients, with only three deaths recorded.

**Conclusion:** Management strategies should be individualized, considering the patient's neurological status and gestational age. Symptomatic management is common, especially for spinal hemangioblastomas, while urgent tumor resection can be performed safely with thorough maternal and fetal monitoring.

**Disclosure:** Nothing to disclose.

## EPO‐340

### Emerging therapeutic strategies for leptomeningeal metastases: Targeting the tumor microenvironment and immune evasion

#### H. Tariq; D. Siddiqui; A. Khan

##### Punjab Medical College, Faisalabad, Pakistan

**Background and Aims:** Leptomeningeal metastases (LM) are a severe complication of advanced malignancies, characterized by the dissemination of cancer cells to the leptomeninges and cerebrospinal fluid (CSF). Prognosis remains dismal, with median survival ranging from 2 to 6 months. Traditional treatments like radiotherapy and systemic chemotherapy have shown limited success. Recent advances in understanding the tumor microenvironment and immune evasion mechanisms have led to the development of novel therapeutic strategies.

**Methods:** A review of studies published between 2015 and 2024 was conducted, including clinical trials, observational studies, and preclinical research. Data sources included PubMed, ClinicalTrials.gov, and oncology conference proceedings. Outcomes assessed were overall survival (OS), progression‐free survival (PFS), response rates, and adverse events.

**Results:** Intrathecal immune checkpoint inhibitors, such as nivolumab, improved median OS to 7.5 months compared to 4.2 months with standard care (HR: 0.65; *p* = 0.002). Nanoparticle‐based drug delivery systems increased CSF drug concentrations by 30%, extending PFS by 2.8 months (*p* = 0.01). CSF microenvironment modulation through CXCL12/CXCR4 axis targeting reduced CSF tumor cell count by 25% (*p* = 0.005). Adverse events were mostly mild (grade 1–2), with no significant increase in severe events (*p* = 0.45).

**Conclusion:** Therapies targeting the tumor microenvironment and immune evasion offer promising strategies for LM. Intrathecal immunotherapies and advanced drug delivery systems show potential to improve survival outcomes. Large‐scale trials are essential to confirm these findings and inform clinical practice.

**Disclosure:** No disclosure is to be made

## EPO‐341

### Isolated cranial hypertrophic pachymeningitis as the sole manifestation of mantle cell lymphoma

#### H. Algahtani ^1^; B. Shirah^2^

##### ^1^Neurology Section, Department of Medicine, Aseer Central Hospital, Abha, Saudi Arabia; ^2^Department of Neuroscience, King Faisal Specialist Hospital & Research Centre, Jeddah, Saudi Arabia

**Background and Aims:** Mantle cell lymphoma (MCL) constitutes approximately 5% of non‐Hodgkin lymphomas, typically presenting with generalized lymphadenopathy and extranodal involvement. CNS involvement in MCL is rare and associated with poor prognosis.

**Methods:** We report a 62‐year‐old immunocompetent female presenting with a two‐week history of headaches, dizziness, vertigo, slurred speech, and blurry vision.

**Results:** Imaging revealed diffuse dural thickening. Histopathological examination confirmed MCL infiltrating the dura mater, presenting as hypertrophic pachymeningitis.

**Conclusion:** This case represents the first documented instance of hypertrophic pachymeningitis as the sole manifestation of MCL. This case underscores the importance of considering MCL in differential diagnoses of hypertrophic pachymeningitis and highlights the diagnostic value of dural biopsy in such atypical presentations.

**Disclosure:** Nothing to disclose.

## EPO‐342

### Microsurgery vs. radiotherapy for vestibular schwannoma: A systematic review and meta‐analysis

#### M. Han ^1^; J. Moreira^2^; E. Goes de Albuquerque^2^; M. Lira^2^; H. Silva Junior^3^

##### ^1^Medical School of the University of São Paulo, São Paulo, Brazil; ^2^Medical Sciences Center, University Federal of Paraiba, João Pessoa, Brazil; ^3^University of Brasilia, Brasilia, Brazil

**Background and Aims:** Vestibular schwannomas are benign tumors arising from Schwann cells of the vestibulocochlear nerve. Treatment options include microsurgical resection (MS), which offers immediate tumor removal but carries risks such as cranial nerve deficits and prolonged recovery, and stereotactic radiosurgery (SRS), a minimally invasive approach like Gamma Knife or CyberKnife, designed to control tumor growth with reduced morbidity. While SRS is associated with improved auditory preservation and fewer complications, comparative data on short‐term outcomes, particularly within a one‐year follow‐up, remain limited.

**Methods:** A systematic search of the Cochrane Library, Embase, and MEDLINE was conducted through December 2024. Data extraction included study design, population characteristics, interventions, and outcomes. Risk of bias was assessed using the Cochrane Risk of Bias 2.0 tool. A random‐effects meta‐analysis was performed using R software (v4.3.1), with heterogeneity quantified by the *I* ^2^ statistic and publication bias assessed via funnel plots.

**Results:** From 1,319 records, 4 studies met inclusion criteria, analyzing 199 MS‐treated and 239 SRS‐treated patients. MS was associated with better hearing preservation rates (OR 0.55, 95% CI 0.36–0.84, *I* ^2^ = 92%) and lower incidences of tinnitus (OR 0.84) and deafness (OR 0.72). SRS reduced vertigo (OR 0.69) and headaches (OR 1.37). Heterogeneity across outcomes was noted.

**Figure:**
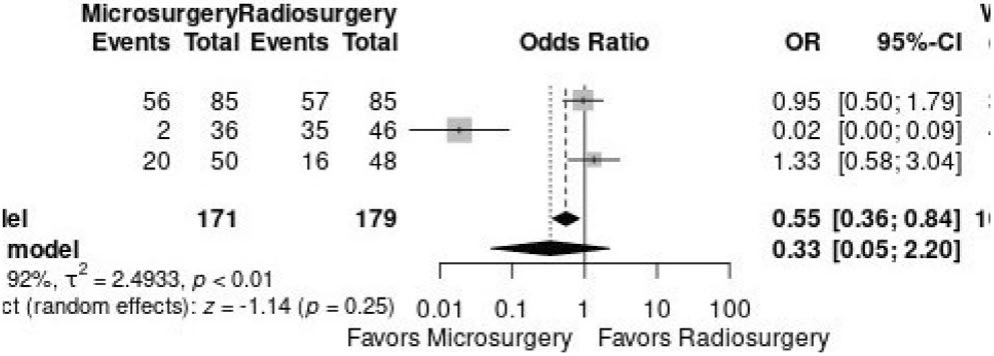
**FIGURE 1** Forest plots of the meta‐analysis on the proportion of patients with hearing serviceable after radiosurgery and microsurgery treatment.

**Figure:**
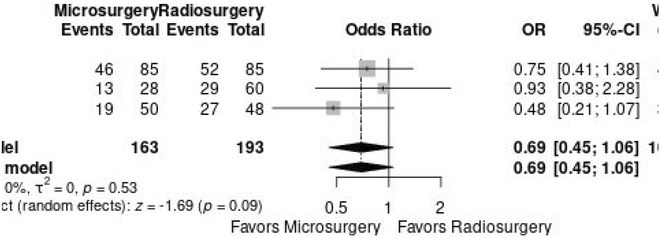
**FIGURE 2** Forest plots of the meta‐analysis on the proportion of patients with vertigo after radiosurgery and microsurgery treatment.

**Figure:**
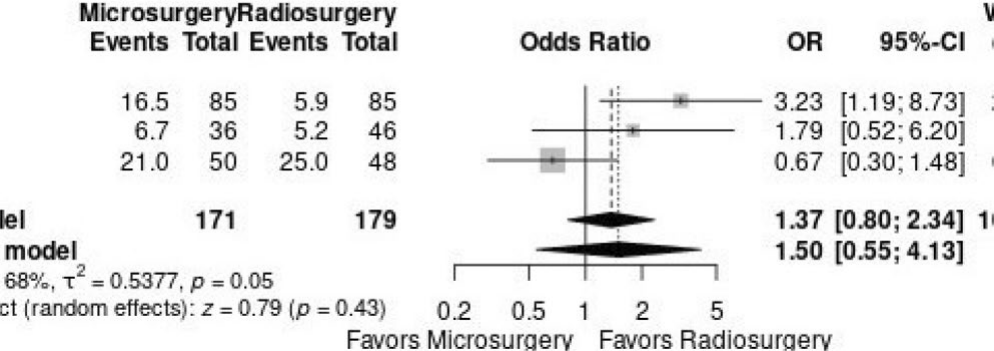
**FIGURE 3** Forest plots of the meta‐analysis on the proportion of patients with headache after radiosurgery and microsurgery treatment.

**Conclusion:** Both treatments offer specific benefits, highlighting the importance of individualized strategies for acoustic neuromas. Further high‐quality studies are needed to confirm these findings and guide clinical decision‐making.

**Disclosure:** Nothing to disclose.

## EPO‐343

### When the storm recedes and returns: Reactivation of ICANS in CAR‐T therapy and the critical role of timely intervention

#### M. El Harmochi Daoud; A. García Maruenda; P. Nieto Palomares; P. Gómez Ramirez; A. Sanchez Gomez; A. Herrera Ortega; M. Muñoz Pasadas; M. Usero Ruiz; M. Corrales Arroyo; A. Hernandez Gonzalez

##### Hospital General Universitario Ciudad Real, Ciudad Real, Spain

**Background and Aims:** Immune effector cell‐associated neurotoxicity syndrome (ICANS) is a recognized complication of CAR‐T therapy, primarily presenting with neurological symptoms. Although most cases occur early, delayed onset or reactivation of ICANS can pose significant challenges to recovery. This report describes a case of ICANS reactivation in a patient treated with CAR‐T therapy, with emphasis on its clinical progression, diagnostic findings, and management strategies.

**Methods:** A 39‐year‐old woman with grade IV follicular lymphoma underwent CAR‐T therapy on September 17 without immediate complications. Five days later, she developed aphasia and altered consciousness, necessitating ICU admission. Corticosteroid therapy resulted in significant improvement, and she was discharged. On December 22, she was readmitted with fever, renal failure, and hypoxia. Respiratory and renal function deteriorated but improved with methylprednisolone. Two days later, she experienced three tonic‐clonic seizures.

**Results:** CT imaging was unremarkable. Lumbar puncture revealed an opening pressure of 27 mmHg, no evidence of infection, and elevated IL‐6 at 6.7 pg/mL. MRI demonstrated asymmetric vasogenic cerebral edema involving supratentorial and infratentorial regions. Corticosteroid therapy escalation led to marked neurological improvement.

**Figure:**
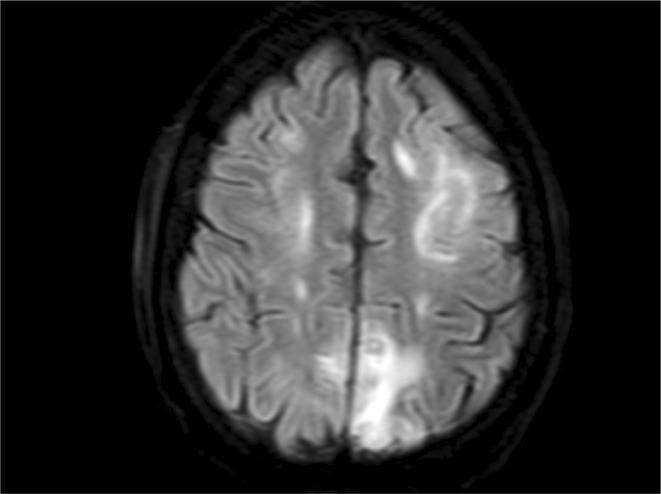
**FIGURE 1** Bilateral parasagital hypersignal noted, FLAIR.

**Figure:**
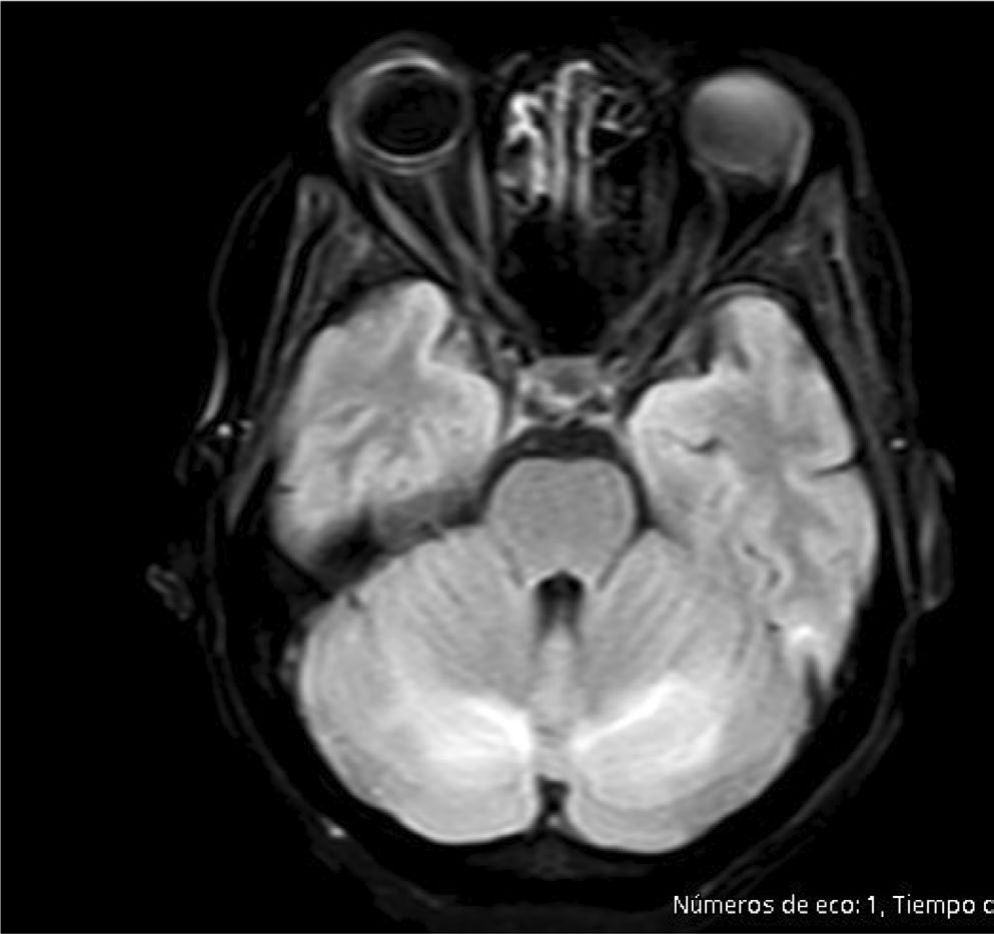
**FIGURE 2** Hypersignal in both cerebellar hemispheres, FLAIR.

**Figure:**
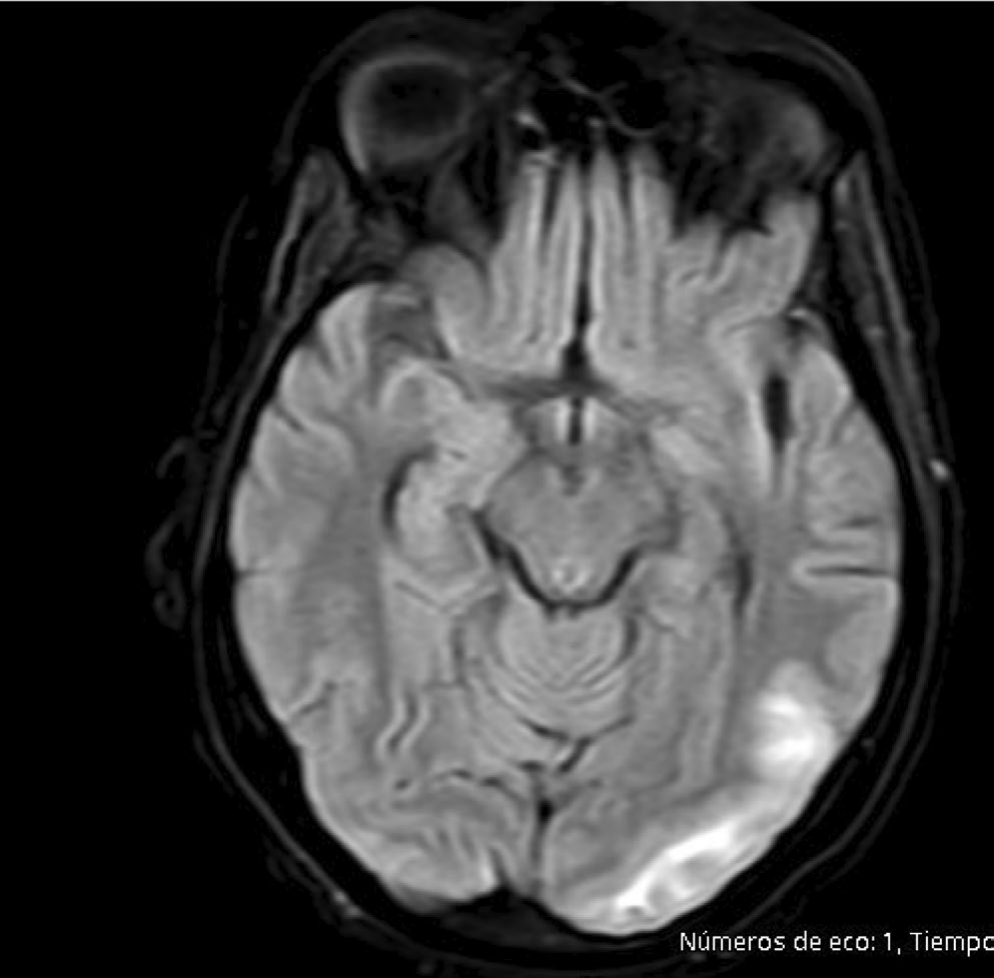
**FIGURE 3** Left temporoocipital hypersignal, FLAIR.

**Conclusion:** This case highlights the reactivation of ICANS following CAR‐T therapy. While ICANS and posterior reversible encephalopathy syndrome (PRES) share overlapping features, key differentiators include the patient's CAR‐T history, elevated inflammatory mediators (e.g., IL‐6), and multisystem involvement (respiratory, renal, cardiovascular). Prompt corticosteroid escalation is crucial for ICANS management. Antiepileptics may be used prophylactically or therapeutically, while tocilizumab is indicated only for severe cytokine release syndrome (CRS).

**Disclosure:** Nothing to disclose.

## EPO‐344

### The immune landscape of brain tumors: Implications for neuroimmunology and neuro‐oncology therapies

#### M. Alnahdi

##### King Abdulaziz University Hospital, Saudi Arabia

**Background and Aims:** The intersection of neuroimmunology and neuro‐oncology is an emerging field that investigates how the immune system influences brain tumor progression and how tumors evade immune detection. Brain tumors, including gliomas, glioblastomas (GBMs), and metastatic cancers, create an immunosuppressive environment that aids tumor growth and resistance to therapies. This review explores the complex relationship between the immune system and brain tumors, focusing on immune cells like microglia, macrophages, T cells, and dendritic cells, and their roles in either supporting or inhibiting tumor progression. We also discuss promising immunotherapies that target the tumor microenvironment, including immune checkpoint inhibitors, adoptive T‐cell therapy, and oncolytic viruses.

**Methods:** We conducted a systematic review of recent studies from 2015 to 2023, sourced from PubMed and Scopus. The review includes clinical trials, preclinical studies, and key articles on immune‐tumor interactions, immune evasion, and immunotherapy for brain tumors.

**Results:** Our findings show that brain tumors manipulate their microenvironment to suppress immune responses, using mechanisms like immune checkpoint activation and recruitment of immunosuppressive cells. Although immune checkpoint inhibitors and adoptive T‐cell therapies show potential, their clinical efficacy remains limited. Emerging combination therapies that integrate immune modulation with traditional treatments like chemotherapy and radiation offer new possibilities for improved outcomes.

**Conclusion:** A deeper understanding of immune‐tumor interactions is crucial for developing effective treatments. Combining immunotherapy with conventional therapies could lead to better outcomes for patients with aggressive brain cancers like GBM.

**Disclosure:** Nothing to disclose.

## EPO‐345

### Spinal metastasis in pleomorphic liposarcoma: A rare location for a rare malignancy

#### N. Lall ^1^; A. Kumar^1^; D. Joshi^1^; V. Singh^1^; A. Pathak^1^; A. Verma^1^; I. Dhal^2^

##### ^1^Institute of Medical Scineces, Banaras Hindu University, Varanasi, India; ^2^Mahamana Pandit Madan Mohan Malaviya Cancer Centre & Homi Bhabha Cancer Hospital, Varanasi, India

**Background and Aims:** Soft tissue sarcoma (STS) are rare mesenchymal tumors, originating from the non‐epithelial connective tissue. The most common subtype of liposarcoma is well differentiated and dedifferentiate liposarcoma. Abdominal wall is a rare site of origin of liposarcoma.

**Methods:** NA.

**Results:** We present a rare case of 58‐year‐old male presented with painless lump in left side lower abdomen for five months. Histopathological examination and immunohistochemistry of biopsy from the lump confirmed the diagnosis of dedifferentiated liposarcoma (vimentin and MDM2 strongly positive). 18FDGPET CT findings confirmed it to it to be a localized disease, FDG avid heterogeneously enhancing soft tissue mass with central necrosis noted at subcutaneous plane of left iliac fossa at anterior abdominal wall abutting anterior abdominal wall muscle with peripheral fat stranding measuring 6.5(AP) × 5.3(T) × 6.0(CC) cm (SUV max‐13.41). Patient underwent wide excision of mass and followed by adjuvant radiotherapy. Within a week of completion of radiotherapy patient developed acute backache and pain was radiating to lower back. Patient soon within a week developed acute paraparesis. MRI dorso‐lumbar spine revealed altered marrow signals with soft tissue component of D10 vertebra level, reaching into spinal canal and causing cord compression likely metastatic lesion.

**Figure:**
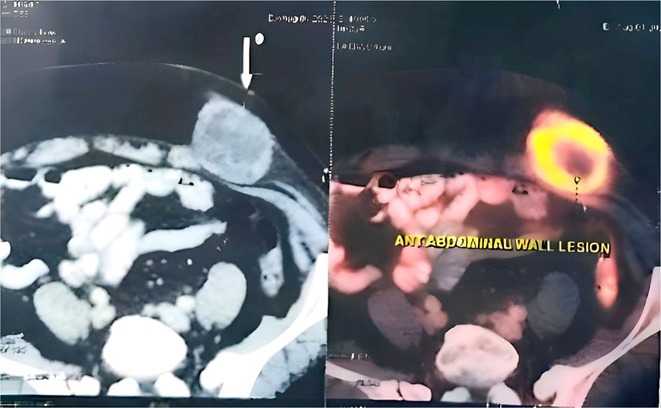
**FIGURE 1** CECT abdomen showing well‐defined oval hypodense lesion of size 5.3 × 5.2 × 4.2 cm seen in deep subcutaneous plane of anterior abdominal wall with maintained fat plane. (b) FDG avid heterogeneously enhancing soft tissue mass with central necrosis.

**Figure:**
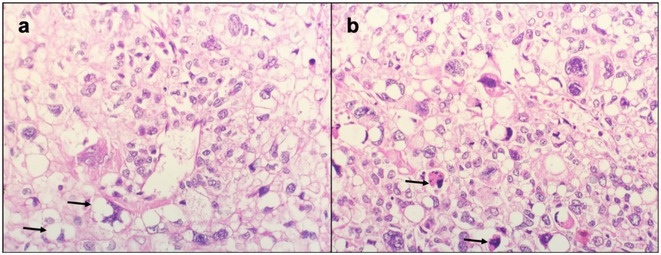
**FIGURE 2** shows sheets of tumor cells having marked nuclear pleomorphism. Cells have an abundant amount of clear cytoplasm. Scattered lipoblasts are noted (arrow), [H&E, 40×] (b) Numerous bizarre tumor cell nuclei are noted along with frequent multinucleated tumor

**Conclusion:** This unique case of localized abdominal wall dedifferentiated liposarcoma showed early dissemination to D10 vertebra and patient landed with acute paraparesis. Meticulous clinical examination and aid of MRI helped in earliest possible diagnosis. Patient received palliative radiotherapy to involved site of vertebra.

**Disclosure:** Nothing to disclose.

## EPO‐346

### Cognitive outcomes of sterotactic radiosurgery vs. stereotactic radiotherapy in skull base meningioma patients

#### R. Pontes Santos Silva ^1^; M. Vicente Campos Guimarães^2^; Ê. Lucas Froes de Araújo^3^; A. Luiza Costa Zaninotto^4^; V. Paglioni^2^; D.Godoy^5^; V. Trindade Gomes da Silva^2^; W. Silva Paiva^2^

##### ^1^Medicine Department, Catholic University of Pernambuco, Recife, Brazil; ^2^Neurosurgery Division, University of São Paulo, São Paulo, Brazil; ^3^Medicine Department, University of São Paulo, São Paulo, Brazil; ^4^Service of Interdisciplinary Neuromodulation, University of São Paulo, São Paulo, Brazil; ^5^Neuro‐Intensive Care Unit, Sanatorio Pasteur Medical Center, Catamarca, Argentina

**Background and Aims:** Meningiomas are the most common primary intracranial tumors, with their localization at the skull base posing a significant clinical challenge. While surgery is considered the gold standard treatment for meningiomas, alternatives such as stereotactic radiosurgery (SRS) and stereotactic radiotherapy (SRT) have emerged as less invasive and effective options. However, their long‐term cognitive impacts remain incompletely understood.

**Methods:** This study aimed to compare the effects of SRS and SRT on cognition in patients with skull base meningiomas, providing evidence to guide more informed therapeutic decisions that balance oncological efficacy with the preservation of quality of life. A quantitative, comparative, and prospective analysis was conducted at two tertiary care hospitals in São Paulo, Brazil. Cognitive evaluation included the Trail Making Test B (TMT‐B), administered pre‐treatment and repeated at 1 and 2 years post‐treatment, and the Brief Visuospatial Memory Test – Revised (BVMTR‐R), conducted at 6 and 18 months post‐treatment.

**Results:** The study sample consisted of 57 patients with skull base meningiomas, with 54.4% treated with SRT and 45.6% with radiosurgery RS. No statistically significant changes were observed in the cognitive test results or quality‐of‐life scores over time or between the two groups.

**Conclusion:** The results indicate that both SRS and SRT are equally effective and safe for preserving cognition in patients with skull base meningiomas, particularly in the domains of focused and alternating attention as well as visuospatial memory.

**Disclosure:** Nothing to disclose.

## EPO‐347

### Innovations in biomarker discovery for DMD: A systematic review of current research trends

#### S. Hassan ^2^; M. Medhat^2^; A. Esmail^2^; S. Elsenbawy^2^; N. Ali^1^; M. Elsayed^1^

##### ^1^MME Foundation, Mansoura, Egipt; ^2^Newgiza University, Alrehab, Egipt

**Background and Aims:** Duchenne muscular dystrophy (DMD) is a debilitating genetic disorder characterized by progressive muscle degeneration. Biomarkers play a critical role in advancing DMD management by facilitating early diagnosis, monitoring disease progression, and evaluating treatment responses. Recent advancements in biomarker discovery offer potential for improving patient care and accelerating therapeutic development.

**Methods:** A systematic search of PubMed, Embase, and Cochrane Library identified studies published up to 2024 that investigated biomarkers for DMD. Eligible studies included preclinical and clinical research evaluating biomarker utility in diagnosis, prognosis, and therapeutic monitoring. Data were synthesized to highlight current trends and key findings.

**Results:** Analysis of 30 studies identified promising biomarkers, including serum creatine kinase as a traditional diagnostic marker and microRNAs (e.g., miR‐206 and miR‐1) for monitoring muscle damage. Imaging biomarkers such as MRI‐based fat fraction quantification provided insights into disease progression. Novel biomarkers, including dystrophin quantification and inflammatory cytokines, demonstrated potential for evaluating treatment responses in clinical trials. Limitations included variability in biomarker standardization and validation.

**Conclusion:** Innovations in DMD biomarker discovery are transforming disease management by enabling precise monitoring and personalized therapeutic strategies. Continued research is essential to validate novel biomarkers and integrate them into clinical practice, advancing outcomes for patients with DMD.

**Disclosure:** Nothing to disclose.

## EPO‐348

### Primary CNS lymphoma with simultaneous brain and spinal cord involvement: A challenging rarity

#### T. Moncada Cordeiro; M. Couto; J. Rosa

##### Neurology Department, São José Local Health Unit, Lisbon, Portugal

**Background and Aims:** Primary central nervous system (CNS) lymphoma is a rare and aggressive neoplasm. Known risk factors include advanced age, immunosuppression, and Epstein‐Barr virus (EBV) infection. They are mostly diffuse large B‐cell lymphomas with predominant brain involvement.

**Methods:** Case report.

**Results:** A 50‐year‐old female patient, with known hypothyroidism and atrophic gastritis treated accordingly, developed a two‐month history of constitutional symptoms followed by psychomotor slowing, dysphagia, constipation, limb paresthesia, muscle weakness, and loss of walking ability. Brain MRI showed bilateral T2 and T2/FLAIR hyperintense thalamic lesions without diffusion restriction or gadolinium enhancement, and spinal cord lesions from C5 to D11. Laboratory results revealed positive EBV IgG serology and cerebrospinal fluid (CSF) pleocytosis. The remaining infectious study was unremarkable, and neuronal surface, anti‐AQP4, and anti‐MOG antibodies were negative. No CSF oligoclonal bands were detected. CSF cytology and immunophenotyping were normal. PET imaging showed diffuse brain hypometabolism and mild hypermetabolism in the cervical spinal cord. The patient underwent treatment with methylprednisolone, human immunoglobulin, plasmapheresis and rituximab. Despite imaging improvement of the spinal lesions, the brain lesions further expanded and there was clinical deterioration to coma. Brain biopsy confirmed a diffuse large B‐cell primary CNS lymphoma. Palliative care was prioritized, and the patient passed away five months after symptom onset.

**Figure:**
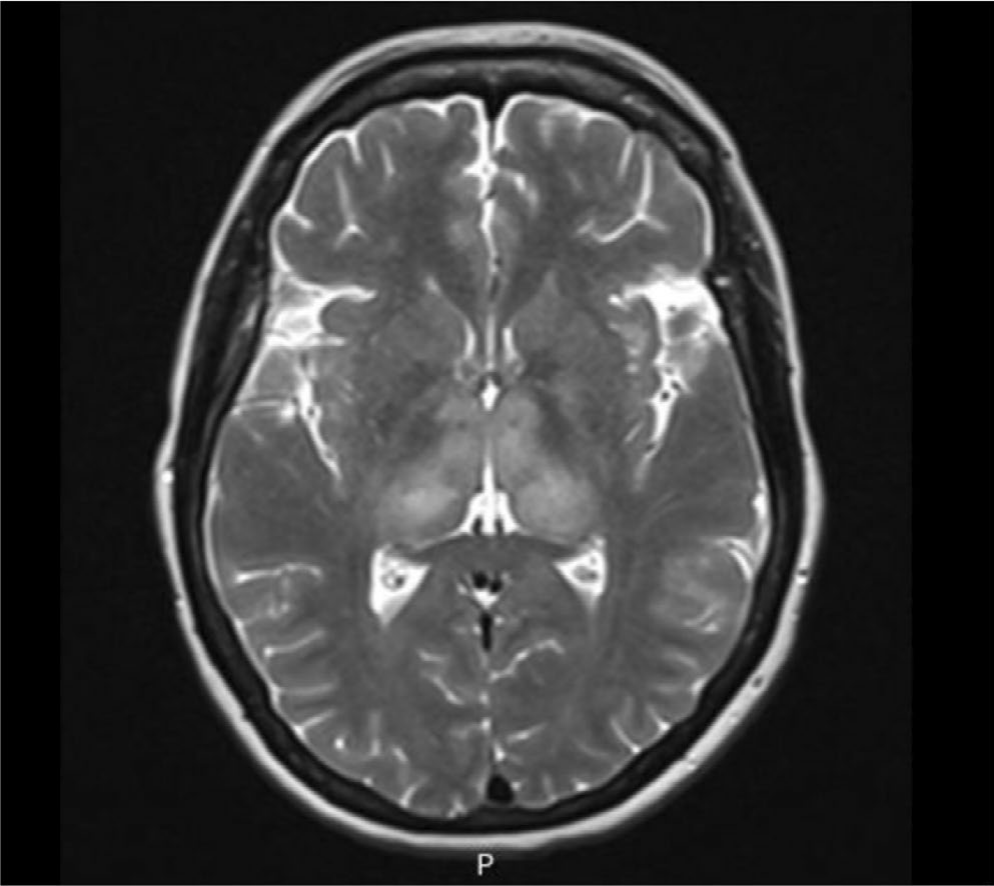
**FIGURE 1** Brain lesions in MRI T2 sequence

**Figure:**
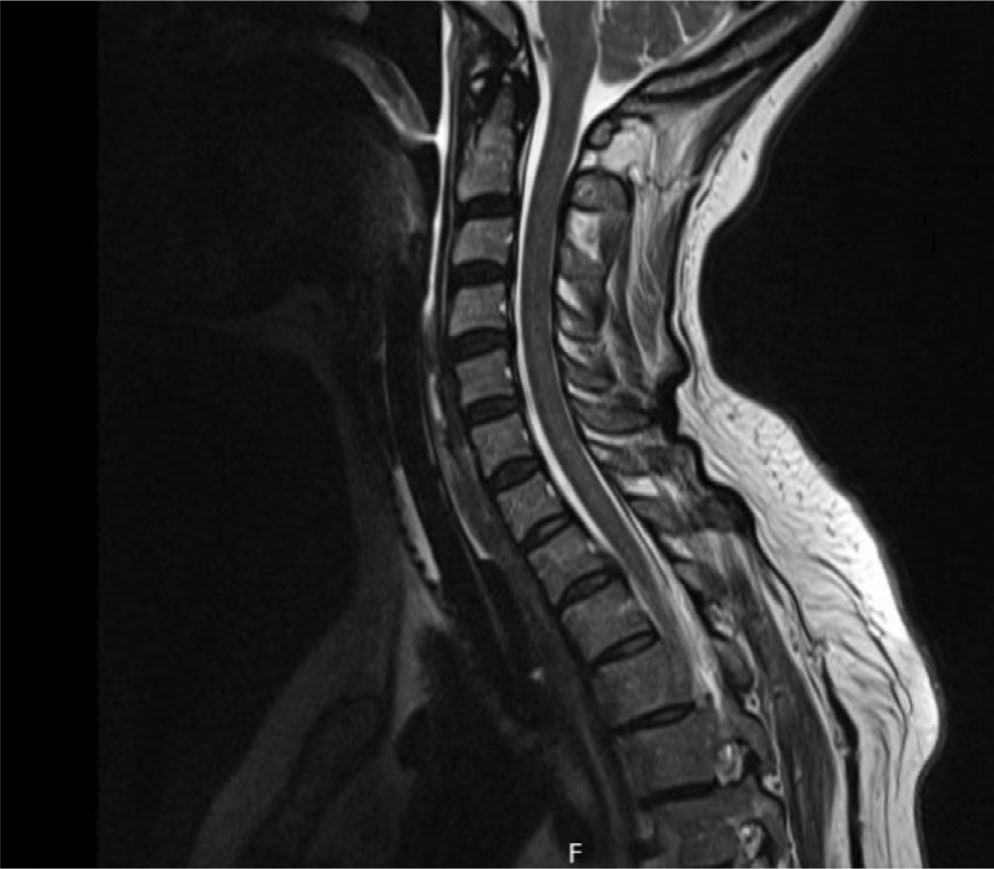
**FIGURE 2** Spinal cord lesions in MRI T2 sequence

**Conclusion:** This case highlights the diagnostic challenges posed by a rare presentation of primary CNS lymphoma. It also notes the importance of multidisciplinary collaboration to ensure a timely diagnosis and improved survival for these patients.

**Disclosure:** Nothing to disclose.

## EPO‐349

### Management of adult‐onset paraneoplastic opsoclonus‐myoclonus ataxia syndrome: Results from a “meta‐cohort”

#### V. Tseriotis ^1^; T. Mavridis^2^; H. Ariño Rodríguez^3^; A. Liampas^4^; S. Panagiotopoulos^5^; M. Arnaoutoglou^6^; G. Hadjigeorgiou^4^; G. Vavougios^4^; P. Mavropoulos^5^; C. Pourzitaki^5^; K. Lallas^7^; C. Tur^3^

##### ^1^Department of Neurology, Agios Pavlos General Hospital of Thessaloniki, Kalamaria, Thessaloniki, Greece; ^2^Department of Neurology, Tallaght University Hospital (TUH), Dublin, Ireland; ^3^Department of Neurology‐Neuroimmunology, Multiple Sclerosis Centre of Catalonia (Cemcat), Vall d'Hebron Barcelona Hospital Campus, Barcelona, Spain; ^4^Department of Neurology, Nicosia General Hospital, Strovolos, Nicosia, Cyprus; ^5^Laboratory of Clinical Pharmacology, Aristotle University of Thessaloniki, Thessaloniki, Greece; ^6^1st Department of Neurology, University Hospital AHEPA, Faculty of Medicine Aristotle University of Thessaloniki, Thessaloniki, Greece; ^7^Department of Medical Oncology, School of Medicine, Faculty of Health Sciences, Aristotle University of Thessaloniki, Greece

**Background and Aims:** Treatment guidelines of adult‐onset paraneoplastic opsoclonus‐myoclonus‐ataxia syndrome (pOMAS) are limited compared to paediatric cases, owing to its rarity and heterogeneous pathogenesis. We aim to systematically review individual cases of adult‐onset pOMAS to evaluate treatment efficacy.

**Methods:** We searched MEDLINE, SCOPUS and Web of Sciences through October 2024 with synonyms for “opsoclonus‐myoclonus‐ataxia syndrome”. Individual patient data were extracted from eligible pOMAS case reports/series. For analysis we grouped treatment into tumour‐directed (TD) and immunotherapy (IT). We dichotomised outcomes as clinical improvement or no benefit (relapse/non‐response) and used univariable and multivariable logistic regression models, adjusting for age and sex.

**Results:** We included 101 articles, pertaining to 141 patients. Lung cancer was the most common malignancy (52/141, 36.9%). Outcome was reported in 134 patients. Clinical improvement after any treatment was demonstrated in 91/134 patients (67.9%). TD and IT combination demonstrated significantly greater efficacy than IT alone (OR = 3 [1.09–8.38], *p* = 0.034). However, this finding was not sustained in multivariable analysis, with age being the sole significant predictor of clinical improvement (OR = 0.95 [0.92–0.99], *p* = 0.007). Gynecological‐ and breast‐cancer‐associated pOMAS was more likely to present clinical improvement (OR = 3.1 [1.11–9.66], *p* = 0.03 and OR = 4.44 [1.43–16.99], *p* = 0.01, respectively).

**Figure:**
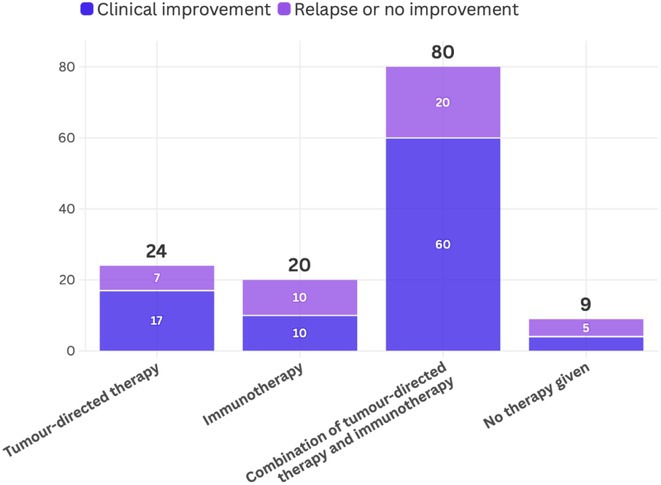
**FIGURE 1** Tumour‐directed treatment and immunotherapy combination demonstrated significantly greater efficacy than IT alone (OR = 3 [1.09–8.38], *p* = 0.034) in univariable analysis.

**Figure:**
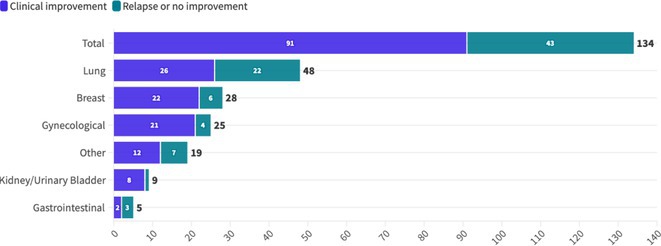
**FIGURE 2** Patients with gynaecological or breast cancer were more likely to present clinical improvement of OMAS symptomatology compared to patients with lung cancer (OR = 3.1 [1.11–9.66], *p* = 0.03 and OR = 4.44 [1.43–16.99], *p* = 0.01, respectively).

**Figure:**
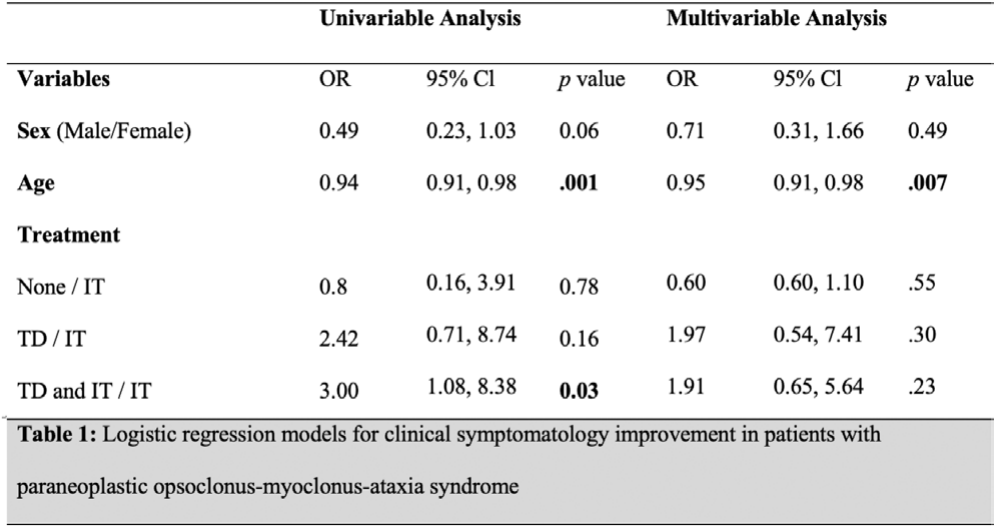
OR: Odds Ratio, CI: Confidence Intervals, TD: tumour‐directed therapy, IT: immunotherapy

**Conclusion:** We present the largest “meta‐cohort” of adult‐onset pOMAS. Despite indications for the superiority of TD and IT combination over IT alone, prognosis is mostly influenced by age and cancer type. Limitations related to study design and missing data, highlight the need for future registry‐based studies.

**Disclosure:** Nothing to disclose.

## Monday, June 23 2025

## Ageing and dementia 2

### EPO‐350

#### Abstract withdrawn

## EPO‐351

### 2 years of experience from the administration of adecanumab in a 33‐year‐old patient with genetic AD

#### A. Tsimpiktsioglou; Z. Gritsopoulou; S. Stasinou; M. Ioakeimidis; T. Doskas

##### Neurology Department, Athens Naval Hospital, Athens, Greece

**Background and Aims:** Aducanumab was the first etiological treatment for Alzheimer's disease (AD). It is a recombinant monoclonal antibody that targets beta‐amyloid plaques, though its results so far have been controversial. We present our two‐year experience with the infusions of this new treatment in a 35‐year‐old patient.

**Methods:** Case presentation.

**Results:** We present a 33‐year‐old patient with no family history of AD, who was examined in the Emergency Department in January 2021 after an episode of generalized seizures. The neurological examination revealed significant impairment of higher cognitive functions, with ideomotor apraxia being the most prominent feature. Her relatives reported behavioral disturbances and difficulty with work demands for approximately three years. Neuropsychological testing showed moderate cognitive decline, while Brain MRI revealed generalized cortical atrophy. The biological markers in the CSF indicated Alzheimer's disease, and genetic testing revealed a pathogenic mutation in PSEN1. After approval from the Geek National Organization for Medicines, treatment with Aducanumab (Aduhelm) was initiated, with one infusion per month and gradual titration, starting with the first dose in August 2021. She has received a total of 24 doses. She has tolerated the treatment without any adverse effects, and follow‐up MRI scans have shown no imaging findings related to β‐amyloid destruction (Amyloid‐Related Imaging Abnormalities), either in the form of hemorrhages (ARIA‐H) or brain edema (ARIA‐E).

**Conclusion:** We present a rare case of a genetic form of Alzheimer's disease with an exceptionally early onset, in which the first targeted treatment for the disease was administered for the first time in a European country.

**Disclosure:** Nothing to disclose.

## EPO‐352

### Genetic evidence for a link between frontotemporal dementia and Parkinson's disease: The case of RAB32 Ser71Arg

#### A. Panzavolta^1^; F. Cavallieri^2^; F. Valzania^2^; M. Radefeldt^3^; S. Lemke^3^; J. Paul^3^; F. Curado^3^; P. Bauer^3^; C. Beetz^3^; C. Cerami ^1^

##### ^1^Scuola Universitaria Superiore IUSS di Pavia, Italy; ^2^Azienda USL‐IRCCS di Reggio Emilia, Reggio Emilia, Italy; ^3^Centogene GmbH, Rostock, Germany

**Background and Aims:** Frontotemporal dementia (FTD) is a heterogeneous group of neurodegenerative disorders primarily affecting behavior and cognition, often overlapping with parkinsonism. A recently discovered gene associated with familiar Parkinson's disease (PD), i.e. RAB32, has been suggested as susceptibility gene in other neurodegenerative syndromes. Hereby, we report a case study on the first RAB32 Ser71Arg mutated patient with a FTD phenotype.

**Methods:** A case study on a 76‐year‐old female proband including family history, neurological examination, neuropsychological and instrumental evaluations, was conducted.

**Results:** Family history revealed five affected relatives with cognitive and/or motor phenotypes. The proband showed predominant executive and social cognition deficits and no motor impairments, fulfilling criteria for the behavioral variant of FTD (bvFTD). Genetic testing confirmed the RAB32 Ser71Arg mutation in a heterozygous state, with no variants in the main autosomic dominant FTD‐associated genes.

**Conclusion:** The RAB32 Ser71Arg mutation is implicated in a rare bvFTD phenotype, broadening the mutation's known clinical spectrum beyond PD. The high phenotypic variability within the proband's family supports incomplete penetrance mechanism and potential additional modifiers influencing disease expression. Strong collaboration between FTD and PD experts should be promoted to better capture the complexity of RAB32 Ser71Arg mutated cases.

**Disclosure:** MR, SL, JJP, FCu, PB and CB are employees of CENTOGENE GmbH. The other authors have nothing to disclose.

## EPO‐353

### Implementation of cerebrospinal fluid biomarkers for the diagnosis of Alzheimer's disease: A single‐center study

#### E. Conesa‐García ^1^; M. Martínez‐Zarco^1^; M. Tomás Orgaz^2^; E. Martínez‐Alonso^3^; J. Bermejillo‐Barrera^1^; B. Gómez‐Gozálvez^1^; M. Ruiz‐Perello^1^; F. Salazar‐Hernández^1^; J. Sánchez‐Villalobos^1^; A. Savolainen^1^; D. López‐Segura^1^

##### ^1^Neurology, Hospital Santa Lucía, Cartagena, España; ^2^Clinical Analysis, Hospital Santa Lucía, Cartagena, Spain; ^3^Cell Biology and Histology Department, Universidad de Murcia, Murcia, España

**Background and Aims:** Alzheimer's disease (AD) is a neurodegenerative disorder characterized by progressive cognitive decline, with cerebrospinal fluid (CSF) biomarkers serving as essential tools for early and accurate diagnosis. This study aims to retrospectively evaluate the application of CSF biomarkers in the diagnosis of AD within our hospital.

**Methods:** We conducted a retrospective observational study involving 116 patients evaluated for cognitive impairment. CSF samples were obtained via lumbar puncture and analyzed for amyloid‐beta 1‐42 (Aβ1‐42), the Aβ1‐42/Aβ1‐40 ratio, total tau (t‐tau), and phosphorylated tau 181 (p‐tau181), using CLEIA on the Lumipulse G600II Platform. Diagnostic categorization was based on established cut‐off values for these biomarkers. Statistical analysis was performed to calculate the predictive values of disease as well as the intrinsic efficacy parameters of the technique.

**Results:** Preliminary results show that 65% of patients had abnormal Aβ1‐42 levels, 46% exhibited reduced Aβ1‐42/Aβ1‐40 ratios, and elevated t‐tau and p‐tau181 levels were found in 31% and 27%, respectively. The biomarker demonstrating the highest positive predictive value (PPV) was p‐tau181 (92%), whereas Aβ1‐42 exhibited the lowest PPV (36%). Incorporating the Aβ1‐42/Aβ1‐40 ratio enhanced the PPV of Aβ1‐42 by 17%, corresponding to an approximate 30% reduction in the false‐positive rate. The prevalence of biomarker‐defined AD in this cohort was 25%.

**Conclusion:** Our findings support the implementation of CSF biomarkers as a reliable diagnostic tool for Alzheimer's disease, emphasizing the utility of Aβ1‐42/Aβ1‐40 ratio in routine clinical practice. This study highlights the importance of integrating biomarker analysis in diagnostic workflows for early and accurate identification of AD.

**Disclosure:** Nothing to disclose.

## EPO‐354

### Bernese brain health consultations: First experience

#### I. Filchenko; T. Monschein; G. Di Tanna; I. Penner; C. Bassetti

##### Department of Neurology, University Hospital, Inselspital, Bern, Switzerland

**Background and Aims:** The Swiss Brain Health Plan (Bassetti et al., 2023) emphasizes raising awareness and fostering interdisciplinary approaches to brain health. The Bernese Brain Health Consultations aimed to provide comprehensive care for patients, while promoting public awareness of brain health (BH). This analysis summarizes our first experience to guide future brain health initiatives.

**Methods:** We conducted 74 ambulatory BH consultations involving 69 patients at Inselspital Bern in 10/2023–07/2024. Of these, 56 patients provided general consent and were included in the current analysis of 61 consultations. Each one‐hour consultation provided individualized recommendations addressing key aspects of BH.

**Figure:**
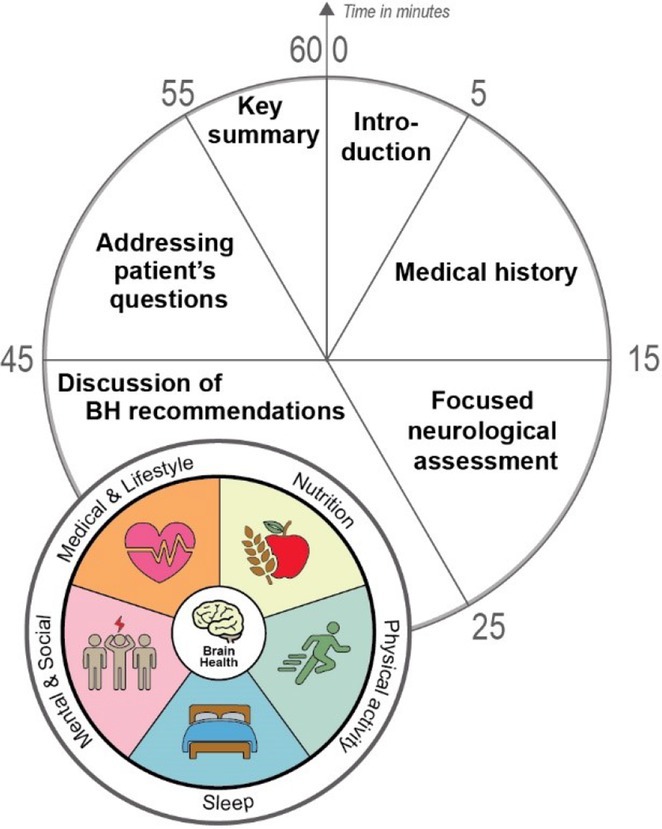
**FIGURE 1** BH consultation: suggested plan for one hour.

**Results:** The consultations included men and women equally (age: 55.1 ± 16.0 years; Figures 2 and 3). Patients had various diagnoses, including psychiatric, neurological, and cardiovascular disorders. Initial referrals originated from general practitioners, neurologists and other sources, including self‐referral, in nearly equal proportions. The recommendations of consultations were diverse, with a high prevalence of further referrals to other specialists (i.e., notably somnologists and specialists in cognitive neurology), additional diagnostics, or lifestyle interventions. Most patients did not require follow‐up BH consultations.

**Figure:**
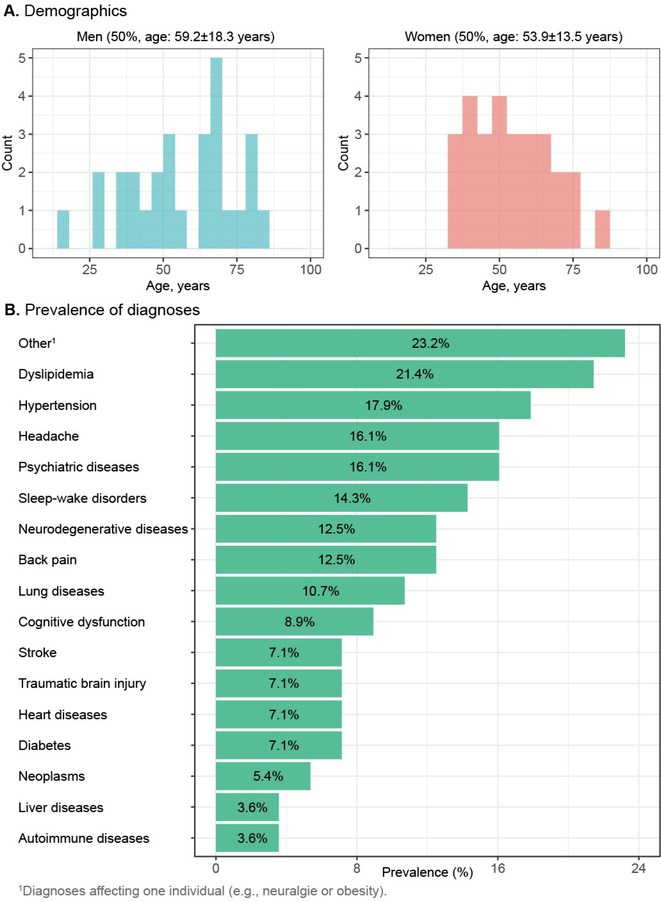
**FIGURE 2** Patient population (*n* = 56).

**Figure:**
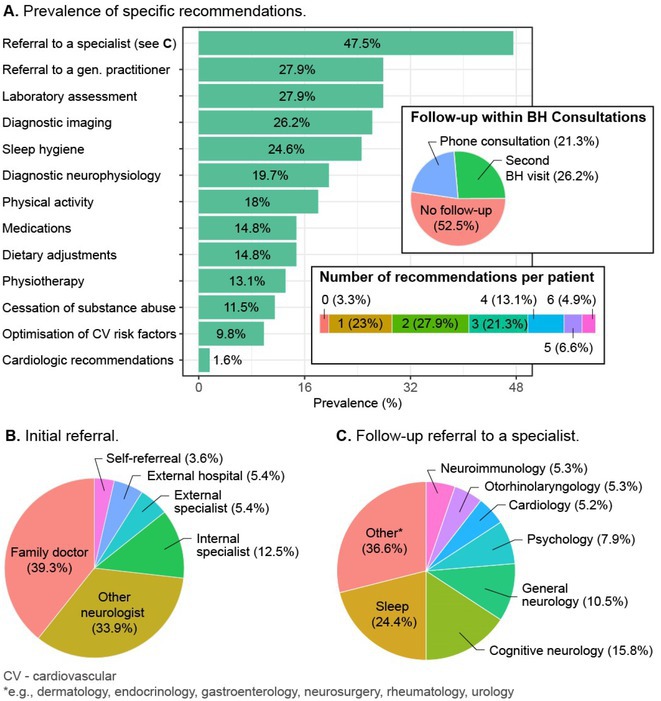
**FIGURE 3** BH recommendations (61 consultations in 56 patients).

**Conclusion:** This is the first description of the BH consultations. BH received high demand from general practitioners and non‐neurologic specialists. The complexity of BH recommendations highlights the importance of an interdisciplinary approach for BH. The experience with BH consultation lays foundation for the development of the Swiss Brain Health Questionnaire. Future efforts should focus on integrating BH into routine care and promoting strategies for BH in the general population, including healthy individuals.

**Disclosure:** Nothing to disclose.

## EPO‐355

### Yes, I'm forgetful – So what? Public perceptions of cognitive decline in Eastern Serbia: A survey study

#### J. Dragićević ^1^; L. Mihajlović Jovanović^1^; N. Ljubenović^2^

##### ^1^Health Center Knjaževac, Neuropsychiatry Department, Knjaževac, Serbia; ^2^Institute of Epidemiology, Military Medical Academy, Belgrade, Serbia

**Background and Aims:** Rural areas, with predominantly older populations, have a higher prevalence of dementia. A common clinical issue is that individuals with cognitive disturbances often seek care only after significant functional decline. We explored general awareness of cognitive decline and forgetfulness among residents of Knjaževac, Eastern Serbia.

**Methods:** The questionnaire designed by the authors for this study was given to the patients and caregivers in Health Center Knjaževac in Serbia. Exclusion criteria were previously diagnosed dementia or mild cognitive impairment.

**Results:** The sample included 745 participants (4.55% of Knjaževac's population), mean age 57.10 ± 14.12, of whom 68.7% lived in municipal areas, 26.7% in rural areas, and 4.56% received geriatric home care. Overall, 62.9% reported disturbances in one or more cognitive domains, and 47.6% of these considered the problem normal for their age. Only 30.7% of those who reported cognitive issues opted for a neurology examination. Among participants who declared memory problems, 58.1% believed frequent forgetfulness is due to lifestyle, 16.6% believed it is not, and 25.2% were unsure (*p* < 0.01). Furthermore, 15.0% were worried and 34.2% were sometimes worried, compared to only 4.9% and 11.3% of those without such problems (*p* < 0.001).

**Conclusion:** According to our findings, many people minimize or normalize cognitive issues. Improving knowledge about dementia is vital. This is crucial to increase knowledge about normal forgetfulness and dementia. Educational materials, information campaigns, and broader public awareness could support earlier dementia diagnosis.

**Disclosure:** Nothing to disclose.

## EPO‐356

### Astrocyte reactivity in Alzheimer's disease: Implications for blood‐brain barrier permeability

#### M. Poli ^1^; C. Bonomi^1^; F. Bernocchi^1^; A. Martorana^1^; N. Mercuri^2^; C. Motta^1^

##### ^1^Memory Clinic and Neurodegenerative Dementia Research Unit, Policlinico Tor Vergata, University of Rome “Tor Vergata” – viale Oxford 81, Rome, Italy; ^2^Neurology Unit, Policlinico Tor Vergata, University of Rome “Tor Vergata” – viale Oxford 81, Rome, Italy

**Background and Aims:** Astrocytes undergo structural and metabolic changes in Alzheimer's disease (AD) progression. These changes impact functions such as blood‐brain‐barrier (BBB) support (1), can be traced using biomarkers like CSF Glial Fibrillary Acidic Protein (GFAP) and lactate, and may vary by APOE genotype (2). This study examines the relationship between these astrocytic biomarkers and BBB permeability in AD, considering the effects of APOE genotype.

**Methods:** We enrolled 98 patients with biomarker‐confirmed AD and 17 age‐matched healthy controls (HC). CSF GFAP and Lactates, albumin quotient (Qalb) and APOE genotyping were measured. AD patients were subclassified as APOE‐ε4 when carrying at least one ε4 allele (*n* = 49), as APOE‐ε3 otherwise (*n* = 49). We performed Kruskal‐Wallis tests and multivariate regressions to verify the associations of CSF GFAP and Lactates with Qalb, adjusting for age, sex and p‐tau.

**Results:** There were no significant differences in terms of Qalb nor CSF astrocytic biomarkers across subgroups, with intact BBB throughout. CSF GFAP was negatively associated with Qalb in both APOE‐ε3 (β = −0.495, *p* = 0.016) and APOE‐ε4 (β = −0.482, *p* = 0.022), but not in HC. Conversely, CSF Lactates showed a moderate positive association with Qalb in APOE‐ε4 (β = 0.420, *p* = 0.002), but not in APOE‐ε3 (β = 0.219, *p* = 0.128) nor HC.

**Conclusion:** Our results underscore the multifaceted nature of astrocyte reactivity in AD, with structural and metabolic markers exhibiting distinct relationships with BBB permeability. Indeed, GFAP appears to be associated with a protective/compensatory mechanism aimed at maintaining BBB integrity (3), while the increase of CSF Lactates alongside BBB disruption in APOE‐ε4 patients might represent a genotype‐specific response to neuronal bioenergetic dysfunction.

**Disclosure:** Nothing to disclose.

## EPO‐357

### DCE‐MRI reveals impaired blood brain barrier in basal forebrain region in patients with Alzheimers disease

#### O. Lerch ^1^; D. Kala^2^; Z. Nedelska^1^; H. Hadzic^1^; J. Otáhal^2^; J. Hort^1^

##### ^1^Department of Neurology, Charles University, Second Faculty of Medicine and Motol University Hospital, Prague, Czechia; ^2^Department of Pathophysiology, Charles University, Second Faculty of Medicine, Prague, Czechia

**Background and Aims:** Blood brain barrier (BBB) dysfunctions is one of is one of the possible mechanisms contributing to onset of Alzheimer's disease (AD). Dynamic contrast enhanced magnetic resonance imaging (DCE‐MRI) is an imaging technique allowing regional assessment of BBB breakdown by estimating local metrics of capillary permeability such as K‐trans. We used DCE‐MRI to examine BBB dysfunction in regions affected early in the course of AD ‐ hippocampus, entorhinal cortex (EC) and basal forebrain (BF) nuclei.

**Methods:** A group of 43 participants – 22 biomarker negative cognitively unimpaired (CU) individuals and 21 biomarker positive patients with mild AD from Czech Brain Aging Study underwent DCE‐MRI. K‐trans maps were estimated using Patlak algorithm implemented within ROCKETSHIP software toolbox. Segmentations of hippocampal head, body and tail, anterolateral and posteromedial EC and BF nuclei were obtained using in house developed pipeline. Average K‐trans values were extracted for each region. Regional differences in BBB permeability between groups were assessed using ANCOVA adjusted for age, sex and ApoE4 positivity.

**Results:** Participants in the AD group had lower mean K‐trans in total BF (K‐transCU = 0.573 × 10^−3^ min^−1^; K‐transAD = 0.270 × 10^−3^ min^−1^, *p* = 0.002), anterior‐intermediate (K‐transCU = 0.833 × 10^−3^ min^‐1^; K‐transAD = 0.291 × 10^−3^ min^−1^, *p* = 0.002) and posterior (K‐transCU = 0.536 × 10^−3^ min^−1^; K‐transAD = 0.237 × 10^−3^ min^−1^, *p* = 0.004) part of nucleus basalis Meynerti. There were no other significant differences between regional BBB permeability in measured structures (*p* > 0.05).

**Conclusion:** Our data show regional reduction in BBB permeability in BF region in patients with mild AD. Regional BBB dysfunction may be one of the factors contributing to early AD related pathological changes in the BF area.

**Disclosure:** Nothing to disclose.

## EPO‐359

### Exploring trends in Alzheimer's disease mortality among aging diabetes patients in the United States over two decades

#### H. Ahmad^1^; M. Ali^2^; Prateek ^3^; F. Ahmed^4^; M. Kakakhel^5^; S. Joshi^6^; A. Sanan^7^; V. Chandak8

##### ^1^Department of medicine, Shalamar Medical and Dental College, Lahore, Pakistan; ^2^Department of Medicine, Jinnah Postgraduate Medical Center, Karachi, Pakistan; ^3^MBBS, All India Institute of Medical Sciences (AIIMS), Rishikesh, Uttrakhand, India; ^4^Duke University Hospital, Division of Cardiology, Durham, USA; ^5^Rehman Medical College, Charsadda, Pakistan; ^6^Yale New Haven Health, Norwalk, USA; ^7^MBBS, Khyber Medical College, Peshawar, Pakistan

**Background and Aims:** Alzheimer's disease poses a growing public health challenge, particularly among individuals with diabetes. This study investigates two‐decade trends in AD mortality rates among aging U.S. adults with diabetes, providing insights into the evolving burden.

**Methods:** Data from 1999 to 2022 were extracted from the CDC WONDER database. Age‐adjusted mortality rates (AAMR), annual percentage changes (APC), and average annual percentage changes (AAPC) were analyzed using Joinpoint regression for adults aged 65 and above.

**Results:** AD among diabetic patients resulted in 202,802 deaths, with AAMRs rising from 138.48 in 1999 to a peak of 264.35 in 2020 before declining to 223.47 in 2022, reflecting an AAPC of 2.06. The period from 2017 to 2020 saw significant increase in AAMR, with an APC of 9.74, followed by notable decline from 2020 to 2022 (−4.84). Alarmingly, females experienced higher AAMRs than males (198.53 vs. 191.07). AAMRs were highest among Non‐Hispanic (NH) Blacks (272.93), followed by Hispanics or Latinos (249.42), while the most pronounced increases were observed in NH Asians (AAPC: 4.28). The West region exhibited the highest AAMR (229.54), followed by the Midwest, South, and Northeast. Additionally, rural areas consistently reported higher AAMRs than urban areas (241.34 vs. 181.72). Among age groups, individuals aged 65–69 showed the highest increase (AAPC: 1.78). Mississippi reported highest AAMR (488) while Nevada reported the lowest (133.5).

**Figure:**
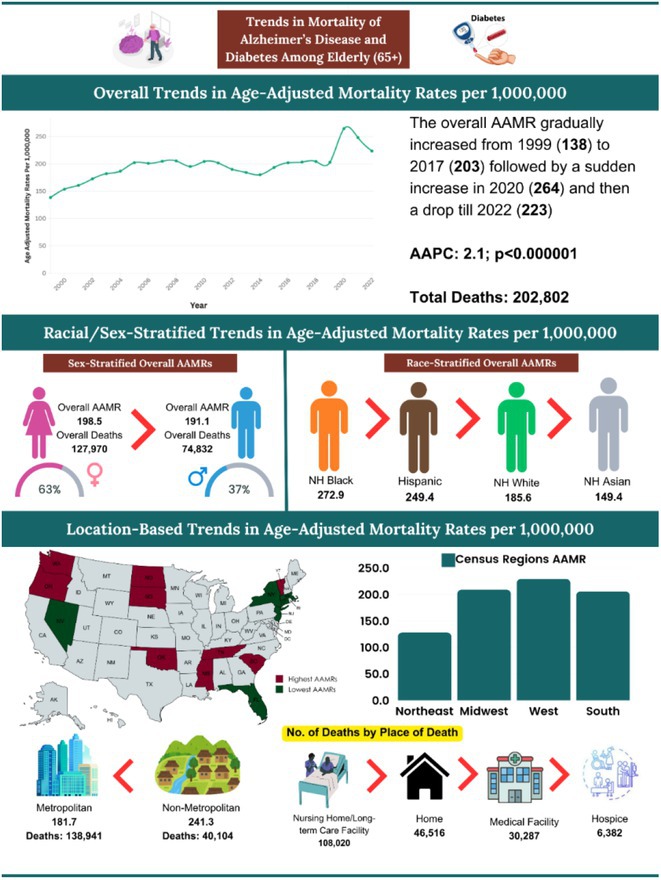
**FIGURE 1** Trends in Mortality of Alzheimer's Disease and Diabetes Among Elderly

**Figure:**
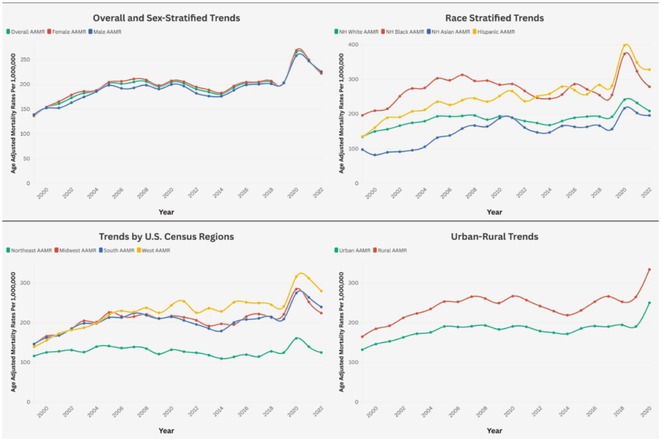
**FIGURE 2** Graphical Representation of Trends

**Conclusion:** AD and diabetes‐related mortality increased gradually since 1999, peaking in 2020, with higher rates observed in females, NH Blacks, the West region, and rural areas. The disparities warrant targeted intervention especially for vulnerable groups.

**Disclosure:** Nothing to disclose.

## EPO‐360

### Long‐term residential exposure to greenspace, bluespace, traffic, and air pollutants and Dementia risk: A cohort study

#### Q. Ji ^1^; Q. Liu^1^; Y. Xu^1^; M. Xu^2^; Y. Zhan^1^

##### ^1^Department of Epidemiology, School of Public Health (Shenzhen), Sun Yat‐Sen University, Shenzhen, China; ^2^School of Public Health (Shenzhen), Sun Yat‐Sen University, Shenzhen, China

**Background and Aims:** Residential air pollution‐related exposures have been implicated in dementia risk, but the underlying pathways remain unclear.

**Methods:** We analyzed 317,498 UK Biobank participants free of dementia at baseline. Residential exposures to pollutants, traffic, greenspace, and bluespace were assessed. Dementia outcomes included all‐cause dementia (ACD), Alzheimer's disease (AD), vascular dementia (VaD), and other dementias (O). Cox proportional hazards models evaluated exposure‐dementia associations, with mediation analysis on plasma metabolites and telomere length.

**Figure:**
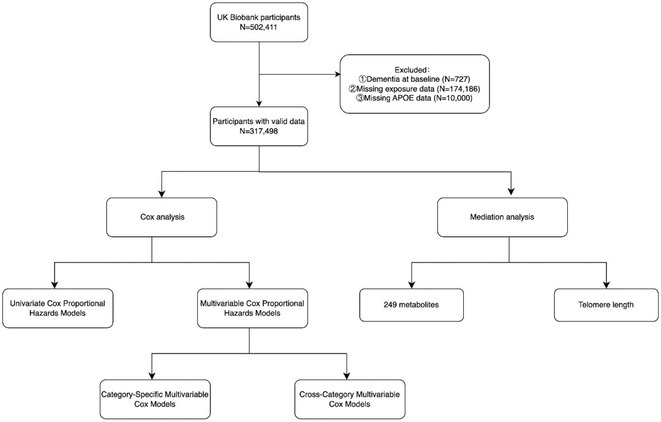
**FIGURE 1** Flowchart of the included participants.

**Results:** Pollutant exposures, especially NO₂ and PM10, were consistently associated with increased dementia risk, with age‐specific effects. Greenspace demonstrated protective effects, particularly for ACD and VaD, while traffic proximity significantly elevated VaD risk. Mediation analysis identified 49 metabolites linking PM2.5‐10 to ACD in younger participants, with Omega‐3% (33.3%) and S‐VLDL‐TG% (32.99%) as key mediators.

**Figure:**
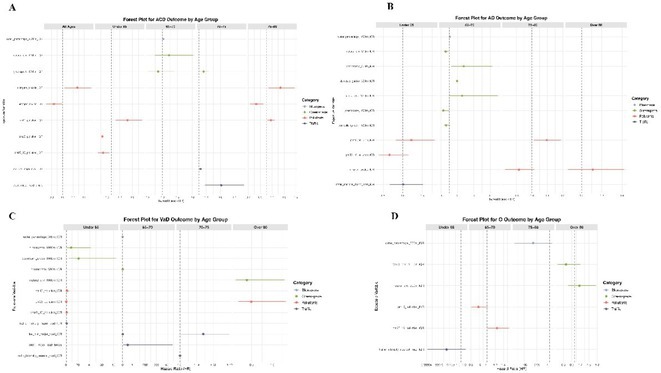
**FIGURE 2** Forest plots for cross‐category multivariate models.

**Figure:**
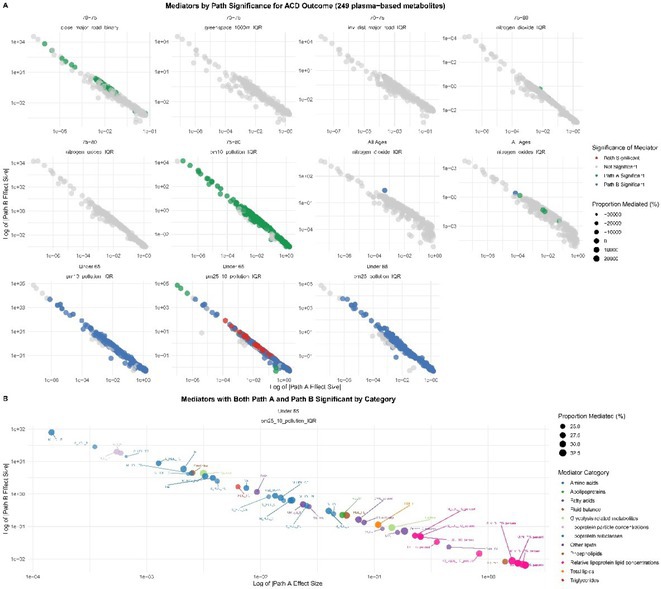
**FIGURE 3** Mediators by path significance for ACD outcome (249 plasma‐based metabolites).

**Conclusion:** Air pollution, especially particulate matter, is significantly associated with dementia risk, partially mediated by metabolic pathways, highlighting the need for environmental interventions.

**Disclosure:** Nothing to disclose.

## EPO‐361

### Carbon dots conjugated with procyanidin B2 function as diamagnetic CEST MRI theranostic agents for Alzheimer's disease

#### S. Wu

##### Department of Radiology, Second Affiliated Hospital of Xiamen Medical College, Xiamen, China

**Background and Aims:** Oxidative stress and neuroinflammation are consistently cited as primary pathological manifestations of Alzheimer's disease (AD). Natural procyanidins can mitigate AD pathological features, by reducing the accumulation of reactive oxygen species (ROS) and neuroinflammation. Implementing in vivo monitoring to assess the impact of antioxidant treatment can contribute to advancing our understanding of this pathophysiological process. Recently, carbon dots (C‐dots), which are discrete quasi‐spherical carbogenic nanoparticles measuring several nm in size, have emerged as a more biocompatible alternative to heavy metal‐based quantum dots. In this study, we developed a C‐dots integrated with procyanidin B2 (C‐dots@procyanidin B2) MRI theranostic agent for eliminating ROS in the brains of AD mice while simultaneously monitoring drug distribution.

**Methods:** We synthesized a new class MRI contrast agent C‐dots@ procyanidin B2. The C‐dots@ procyanidin B2 has chemical exchange saturation transfer (CEST), which enabled the use of CEST imaging for monitoring c‐dots distribution and provided the information for ROS and neuroinflammation accumulation.

**Figure:**
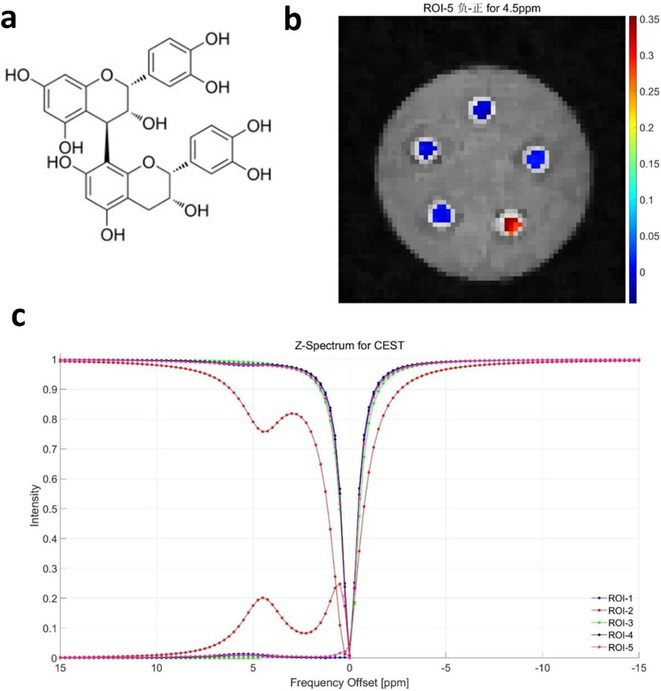
**FIGURE 1** We found procyanidin B2, which has a CEST effect near 4.5 ppm in aqueous solution, crucial for MRI contrast enhancement.

**Results:** Based on in vitro and in vivo studies, we demonstrate that C‐dots@procyanidin B2 exhibits CEST effects. Moreover, C‐dots@procyanidin B2 efficiently mitigates ROS levels. Furthermore, these C‐dots rapidly accumulate in the brains of AD mice and alleviate pathological features such as Aβ plaque deposition, neuronal loss, and neuroinflammation. Additionally, they significantly enhance learning ability and memory function in AD mice.

**Figure:**
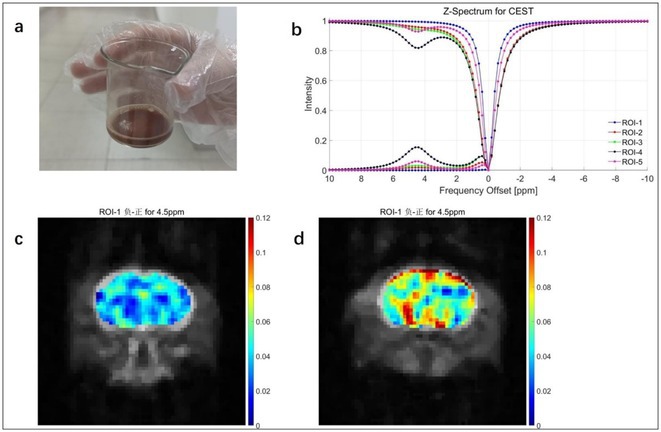
**FIGURE 2** The synthesis of AC‐dots@procyanidin B2 Agent and CEST MRI.

**Figure:**
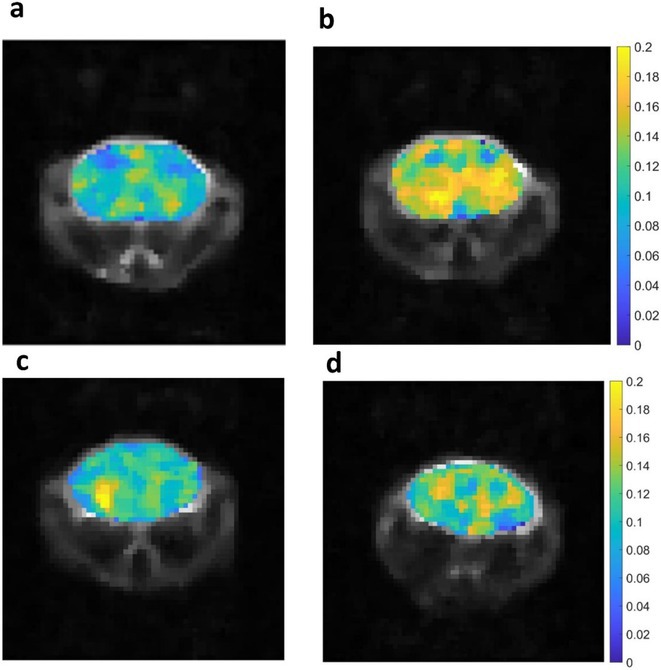
**FIGURE 3** Our in vivo studies using 6‐month‐old APP/PS1 mice demonstrated the efficacy of our nanoparticles. Thirty minutes post‐drug administration, CEST imaging revealed a significant increase in signal intensity in the therapy group (a,b) compared to the saline.

**Conclusion:** This study demonstrates that C‐dots@procyanidin B2 can attenuate the progression of AD by reducing levels of reactive oxygen species (ROS) and neuroinflammation accumulation. Additionally, its CEST effects offer a novel approach for developing theranostic agents targeting AD.

**Disclosure:** Nothing to disclose.

## Epilepsy 2

## EPO‐362

### Imaging of epilepsy in children in Abidjan, Cote D'Ivoire

#### A. Essoin‐De Souza ^1^; A. Abbe^1^; S. Agbo‐Panzo^2^; A. Beuseize^1^; N. Broh^1^; N. Yeo^1^; I. Diakite^1^; K. Kouassi^1^; M. Doumbia^1^

##### ^1^Teaching hospital of Treichville, Abidjan Cote D'Ivoire; ^2^Teaching Hospital of Cocody, Abidjan Cote D'Ivoire

**Background and Aims:** Epilepsy is a neurological chronic condition affecting 0.5 to 1% of children. Investigations carried often involve brain imaging. The overall aim of our study was to have a better understanding of the role of brain imaging in the management of child epilepsy, and to contribute to its accessibility in our country.

**Methods:** This was a retrospective, descriptive study of the records of children followed over a period of three (03) years, from January 2020 to January 2023. It included 129 children who had undergone brain imaging, representing 59.4℅ of children with epilepsy during this period.

**Results:** The average age was 5.3 years, and the most represented gender was male. The mean age of onset of seizures was 3 years and 2 months. Motor delay was noted in almost 30% of cases, and language delay in over 45%. Brain imaging performed in all our patients was pathological in 52.7% of cases: brain atrophy was the most frequent abnormality (48%), followed by anoxic‐ischemic lesions (29.7%). Predictive factors for pathological imaging were early age of onset, neonatal distress, delayed psychomotor development, focal seizures, and presence of a neurological deficit

**Conclusion:** Brain imaging remains essential in the search for the cause of epilepsy.

**Disclosure:** Nothing to disclose.

## EPO‐363

### Does age matter: Risk of an epilepsy diagnosis in an adult cohort referred to a first‐seizure clinic

#### A. Birkmose ^1^; C. Lackmann^2^; L. Pinborg^3^; K. Jensen^1^

##### ^1^Department of Neurology, Aalborg University Hospital, Aalborg, Denmark and Department of Clinical Medicine, Aalborg University, Aalborg, Denmark; ^2^Department of Neurology, Aalborg University Hospital, Aalborg, Denmark; ^3^Epilepsy Clinic and Neurobiology Research Unit, Department of Neurology, Copenhagen University Hospital – Rigshospitalet, Copenhagen, Denmark

**Background and Aims:** At the Epilepsy Clinic, Aalborg University Hospital, Denmark our clinical observations indicate a rising trend in the referral of older individuals for epilepsy evaluation. We aim to compare the likelihood of receiving an epilepsy diagnosis and the diagnostic yield of brain imaging and EEGs between different age groups.

**Methods:** This single‐center retrospective study include patients referred for epilepsy evaluation April 1, 2022–January 8, 2024, with a one‐year follow‐up available. Demographics, resulting diagnostic work‐up including CT/MRI brain imaging and electroencephalogram (EEG) and final diagnosis were registered. Descriptive data were calculated as proportions and the relative risk of epilepsy was modeled using Poisson regression with age as the primary predictor and presented using a cubic spline. OSL regression was used to compare the sensitivity and specificity of EEG between age groups.

**Results:** A total of 530 patients aged 18–95 years were included (mean age: 55, 56.7% male). Of patients referred to the clinic 158 (29.8%) of patients received an epilepsy diagnosis. Poisson regression curve indicated a rising trend in the risk of receiving an epilepsy diagnosis with advancing age. The risk of receiving an epilepsy diagnosis was significantly higher in patients aged >65 years compared to those aged 18‐64: RR = 1.36, 95% CI (1.04–1.76 *p*‐value: 0.02). MRI, CT‐scans of the brain and EEG were key diagnostic tools, with varying diagnostic yield across age groups.

**Conclusion:** Our study highlights the need for continuous attention to precise patient selection and specialized diagnostic work‐up of patients referred to First Seizure Clinics.

**Disclosure:** Nothing to disclose.

## EPO‐364

### Detection of clinical, molecular and radiological biomarkers in patients with progressive myoclonic epilepsy

#### A. Aydoğan ^1^; D. Yozlu^2^; Ö. Düzenli^2^; S. Danışman^2^; S. Susgun^3^; N. Korkmaz^3^; B. Bilge^4^; E. Yücesan^2^; N. Bebek^1^

##### ^1^Istanbul University Istanbul Faculty of Medicine Neurology Department, İstanbul, Turkey; ^2^Neurogenetics, Istanbul University‐Cerrahpasa Institute of Neurological Sciences, İstanbul, Turkey; ^3^Bezmialem Vakif University, Faculty of Medicine, Department of Medical Biology, İstanbul, Turkey; ^4^Department of Neuroscience, Istanbul University‐Cerrahpasa Institute of Neurological Sciences, İstanbul, Turkey

**Background and Aims:** Progressive myoclonic epilepsies (PME) are often diagnosed late due to the disease's insidious onset and lack of biomarkers, which accelerates progression. This study aimed to evaluate the clinical, molecular, and radiological findings of PME patients and identify potential biomarkers.

**Methods:** Seventeen patients aged over 18 with PME, obtained informed consent were included. Clinical findings, cerebrospinal fluid (CSF) neurofilament light chain (nFL) levels, and retrospective cranial MRI findings were analyzed. Expression levels of miRNAs linked to NHLRC1 and EPM2A genes (miR‐326 for NHLRC1 and miR‐383‐5p for EPM2A), key to Lafora Disease (LD) pathogenesis, were evaluated. Statistical analyses were conducted on the collected data.

**Results:** Nine patients (52.9%) were female, with a mean age of 34.8 ± 15.2 years. The most common genetic variant was LD‐NHLRC1. Disease duration averaged 22.2 ± 13.0 years, with other neurological symptoms appearing 5.7 ± 3.8 years post‐onset. Twelve patients (70.5%) were independent in walking. Frontal dysfunction (82.3%) dominated the neuropsychological profile. CSF nFL levels did not differ from reference values according to patients and age. However, miR‐326 and miR‐383‐5p expression levels were significantly reduced in patients compared to controls.

**Conclusion:** The p.Asp146Asn mutation in the NHLRC1 variant of LD is associated with a favorable course. Restrictive symptoms manifesting 5.7 years post‐disease onset highlight the importance of early diagnosis with suitable biomarkers. Normal CSF nFL levels suggest a slow neurodegenerative process via different mechanisms. The pronounced reduction in miR‐326 and miR‐383‐5p expression among non‐Lafora patients indicates shared pathophysiological processes in PMEs.

**Disclosure:** This study was supported by Istanbul University Scientific Research Projects Coordination Unit. Project ID: 40136.

## EPO‐365

### Comparison of subtotal hemispherectomy and other disconnective surgical techniques in epilepsy surgery

#### B. Turk ^1^; M. Iris^1^; H. Cerci^2^; M. Delil^1^; C. İsler^2^; S. Yeni^1^; C. Ozkara^1^; T. Tanriverdi^2^; M. Uzan^2^

##### ^1^Department of Neurology, Istanbul University‐Cerrahpasa, Faculty of Medicine, Istanbul, Turkey; ^2^Department of Neurosurgery, Istanbul University‐Cerrahpasa, Faculty of Medicine, Istanbul, Turkey

**Background and Aims:** Disconnective surgery may be an option for refractory epilepsy when the seizure onset zone is too large or resection is not feasible due to the risk of functional loss. Commonly used disconnective techniques today include Functional Hemispherotomy (FH), Posterior Quadrantectomy (PQ), and Subtotal Hemispherectomy (SH). The aim of this study is to retrospectively evaluate the outcomes of SH and other disconnective surgical techniques.

**Figure:**
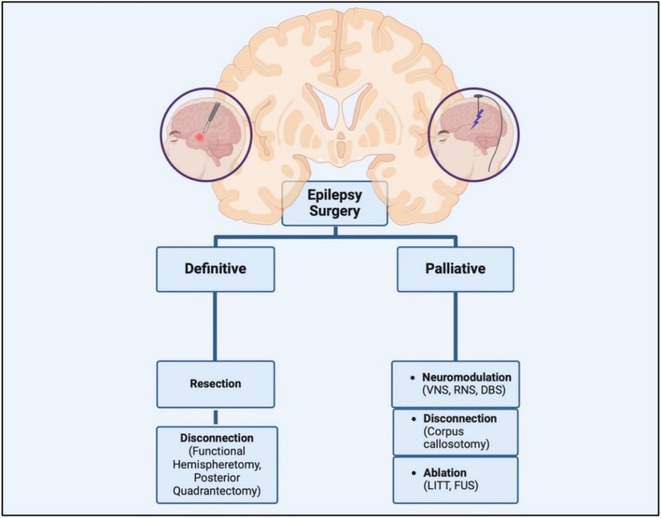
**FIGURE 1** Types of Epilepsy Surgery

**Methods:** Patient records from our center, from 2000 to 2022, were reviewed retrospectively. A total of 48 patients who underwent disconnective surgery and had at least 1 year of postoperative follow‐up were included in the study. Gender, age, age at seizure onset, disease duration, postoperative neurological complications, and postoperative seizure frequency were evaluated.

**Results:** The mean age of the 48 patients (20F, 28M) was 21.29 ± 12.67 years. 23 patients underwent FH, 17 patients underwent PQ, and 8 patients underwent SH. 31 patients were classified as Engel Class 1, 7 as Engel Class 2, 5 as Engel Class 3, and 5 as Engel Class 4. Of the patients who underwent SH, 7 achieved Engel Class 1, while 1 patient remained as Engel Class 4. Postoperative complications were observed in 11 patients. No significant differences were found between the groups regarding Engel classification and complication rates.

**Figure:**
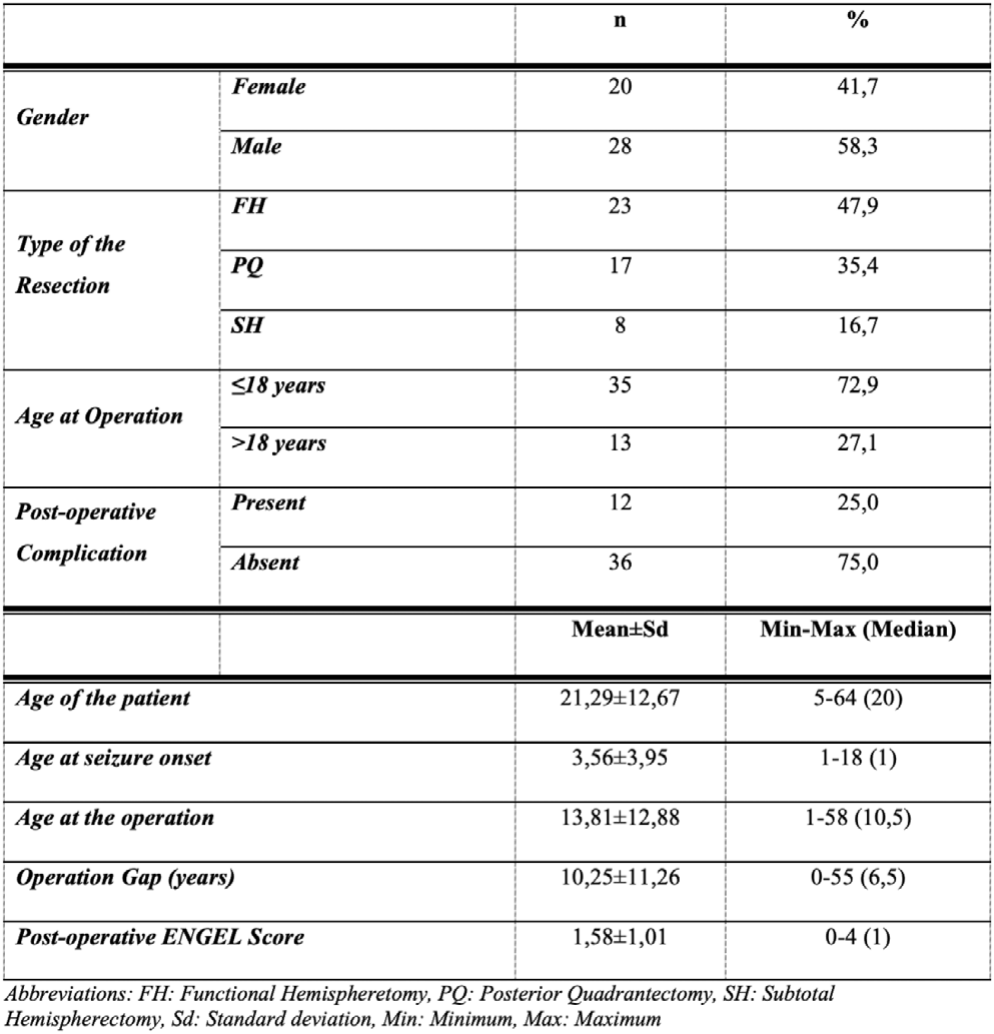
**TABLE 1** The Demographical Features of The Patients.

**Figure:**
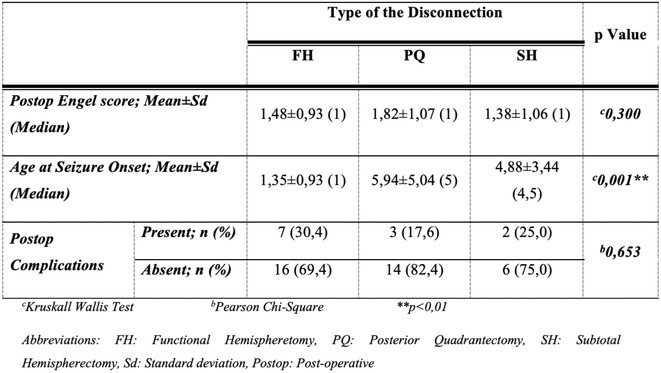
**TABLE 2** Analysis of Age at Seizure Onset, Postoperative Complications and Engel Scores According to the Types of Disconnection Surgery

**Conclusion:** The appropriate surgical disconnective procedure should be selected based on the seizure onset zone and individual patient characteristics. Patients with hemispheric structural anomalies but no paresis may be suitable candidates for SH.

**Disclosure:** Nothing to disclose.

## EPO‐366

### Effect of cenobamate on sudden unexpected death in epilepsy risk in a Spanish cohort of a phase 3 clinical trial

#### V. Villanueva^1^; J. Leach^2^; K. Thangavelu^3^; P. Pérez‐Domper^4^; E. Álvarez‐Barón ^5^

##### ^1^Hospital Universitari i Politècnic La Fe, Valencia, Spain; ^2^Angelini Pharma S.p.A., Rome, Italy; ^3^MeDaStats LLC, Tampa, USA; ^4^Angelini Pharma S.p.A., Barcelona, Spain; ^5^Angelini Pharma S.p.A., Madrid, Spain

**Background and Aims:** Effect of cenobamate on sudden unexpected death in epilepsy (SUDEP) accounts for 2‐17% of deaths in patients with epilepsy (Ficker 2000, Epilepsia 41 Suppl 2: S7–S12). SUDEP‐3 (score 0–4) and SUDEP‐7 (score 0–10) scales assess the potential risk of SUDEP. For each point reduction in SUDEP‐3, SUDEP odds decrease by 64%; for SUDEP‐7, per‐point odds decrease is 29% (Rasekhi 2021, Epilepsia 62(7):1536–1545).

**Methods:** NCT02535091 (C021, *N* = 1340) was a global, multicenter, phase 3, open‐label safety study of cenobamate as adjunctive treatment in adults with uncontrolled focal‐onset seizures (FOS). Efficacy data pre‐ and post‐cenobamate treatment were collected in in a multicenter retrospective observational study of the C021 Spanish cohort (*n* = 127). SUDEP‐3 and SUDEP‐7 risk scores (RS) were calculated for patients in that cohort before and after cenobamate treatment.

**Results:** At baseline, 76% and 24% patients had SUDEP‐3 RS of 2 and 3. After 2 years of cenobamate treatment, 6% had a RS point reduction of 3, 11% of 2, and 14% of 1 point; 69% remained stable (Figure 1). Using the SUDEP‐7 inventory, at baseline, 51% of patients had a RS of 1–3 and 49% had a RS of 4–8. After 2 years of cenobamate treatment, 1% had a RS point reduction of 4, 9% of 3, 9% of 2, and 30% of 1 point. 44% remained stable, and 7.5% increased SUDEP risk (Figure 2).

**Figure:**
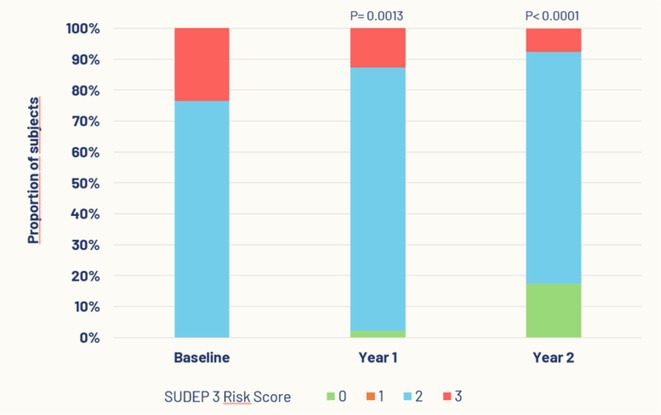
**FIGURE 1** Study subjects stratified by SUDEP 3 risk score.

**Conclusion:** Cenobamate treatment significantly reduced SUDEP risk in some Spanish cohort patients as measured by two SUDEP risk scales. The potential for reducing SUDEP risk should be considered when initiating/changing treatment in patients with uncontrolled FOS.

**Disclosure:** The original study C021 (NCT02535091) was supported by SK Life Science, Inc. (Paramus, NJ, USA), the Spanish cohort study was supported by Angelini Pharma Spain, and these analyses were supported by Angelini Pharma S.p.A. (Rome, Italy). VV: Consultant/advisor: Angelini Pharma, BIAL, Eisai, Esteve, GlaxoSmithKline, Jazz, Novartis, Sandoz, Takeda, UCB Pharma, Xenon; Speaker: Angelini Pharma, BIAL, Cevomed, Eisai, Esteve, Jazz, Newbridge, Paladin, UCB Pharma; Research support: Angelini Pharma, BIAL, Eisai, Jazz, UCB Pharma. JPL, PPD, EAB: Employees, Angelini Pharma. KT: Consultant: Angelini Pharma.

## EPO‐368

### Cortical myoclonus as an manifestation of familial myoclonic epilepsy of adults (FAME)

#### F. D'Anna ^1^; F. Brancati^2^; M. Cirillo^1^; A. Coppola^3^; V. Todisco^1^; L. Veneziano^4^; A. Giordano^1^; A. Tessitore^1^

##### ^1^Dipartimento di scienze mediche e chirurgiche avanzate Università degli studi della Campania “Luigi Vanvitelli”, Roma, Italy; ^2^Dipartimento di Scienze della Vita, della Salute e dell'Ambiente, Università degli Studi dell'Aquila, Roma, Italy; ^3^Dipartimento di Neuroscienze e Scienze Riproduttive ed Odontostomatologiche – Università degli Studi di Napoli Federico II; ^4^Istituto di Farmacologia Traslazionale di Roma, Roma, Italy

**Background and Aims:** A 51‐year‐old man reports acute onset 15 years ago of sudden, rapid, asymmetrical, appendicular and axial, asynchronous muscle contractions associated with subsequent generalized hyposthenia. Both father and brother presented the same symptomatology and episodic generalized tonic‐clonic seizures.

**Methods:** The patient underwent routine blood tests, brain MRI, electroencephalography, electroneuromyography, jerck‐locked back averaging analysis and genetic panel analysis for genetically determined epilepsies.

**Results:** Electroencephalography showed (a) isolated, brief clusters of punctate elements and subsequent slow wave clinically followed by upper limb muscle contraction (cortical myoclonus), (b) a photoparoxysmal response to ntermittent photic stimulation. Surface electromyography (long flexor and extensor carpal muscle) confirmed the presence of myoclonus. Genetic analysis showed a pathological pentameric expansion in heterozygosity (ATTTC)n in the STARD7 gene.

**Conclusion:** The abovementioned findings supported the diagnosis of Familial myoclonic epilepsy of the adult (FAME2). FAME is a genetically determined disorder with autosomal dominant and vertical transmission, with probable involvement of cerebellar structures, characterized by a heterogeneous syndromic cortex of cortical tremor, myoclonic jerks, occasional generalized tonic‐clonic and/or myoclonic seizures, and additional symptoms based on the disease phenotype (migraine, night blindness, cognitive impairement, psychiatric deseases). The clinical features of this disorder pose different differential diagnoses (essential tremor, juvenile myoclonic epilepsy, Progressive myoclonic epilepsy) because of the wide etiologic possibility of the myoclonic phenomenon; therefore, it is necessary to identify precise disease phenotypes, including in correlation with the mutated gene, and clinical, radiologic, and electrophysiologic dinstiguous elements to avoid misdiagnosis, inadequate therapeutic treatment, and worsening prognosis.

**Disclosure:** Nothing to disclose.

## EPO‐369

### Neurophysiological and clinical correlates of low‐dose perampanel in familial adult myoclonus epilepsy type 2

#### G. Senerchia ^1^; V. Iuzzolino^1^; P. Striano^2^; L. Bilo^1^; A. Coppola^1^; R. Dubbioso^1^

##### ^1^Department of Neurosciences (Reproductive Sciences and Odontostomatology), University of Naples “Federico II”, Naples, Italy; ^2^Department of Neurosciences, Rehabilitation, Ophthalmology, Genetics, Maternal and Child Health, Università Degli Studi di Genova, Genoa, Italy

**Background and Aims:** Familial adult myoclonus epilepsy (FAME) management relies on antiseizure medications (ASMs), which inadequately address myoclonus and cortical tremor. This study evaluates Perampanel (PER), an AMPA‐receptor antagonist, for treating FAME symptoms.

**Methods:** Fifteen FAME2 patients participated in an observational prospective study. They received up to 6 mg daily of PER and underwent Unified‐Myoclonus‐Rating‐Scale (UMRS) before and after treatment. Neurophysiological evaluations, including somatosensory evoked potentials (SEPs) and transcranial magnetic stimulation (TMS), assessed PER's impact on cortical glutamatergic excitatory and GABAergic inhibitory circuits.

**Results:** PER treatment significantly reduced UMRS total scores (*p* = 0.001) and action‐myoclonus subscores (*p* = 0.002), irrespective of disease duration, age at onset, or testing time (*p* > 0.05). Patients with more severe baseline myoclonus demonstrated significant improvements. Neurophysiological assessments revealed a PER‐induced decrease in sensorimotor hyperexcitability, characterized by diminished N33 amplitudes, attenuated glutamatergic facilitation, and enhanced GABAergic inhibition in the motor cortex. In conclusion, low‐dose PER is well tolerated and effective in alleviating myoclonus in FAME2 patients, supported by its modulatory effects on glutamatergic and GABAergic neuronal circuits. Plain Language Summary:

**Figure:**
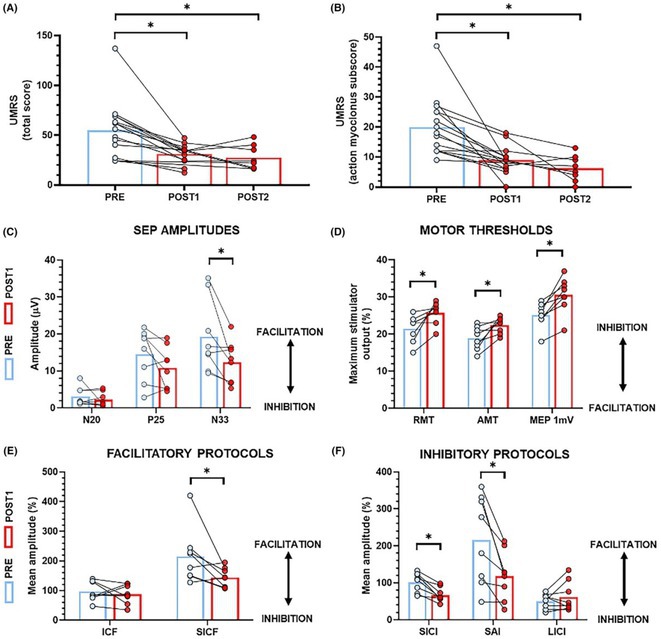
**FIGURE 1** The UMRS total score (A) and the action myoclonus subscore (B) were significantly reduced following PER (PER) treatment at both follow‐up assessments: POST1 (total score: *p* = 0.008; subscore: *p* = 0.031) and POST2 (total score: *p* = 0.006; subscore: *p* = 0.0)

**Figure:**
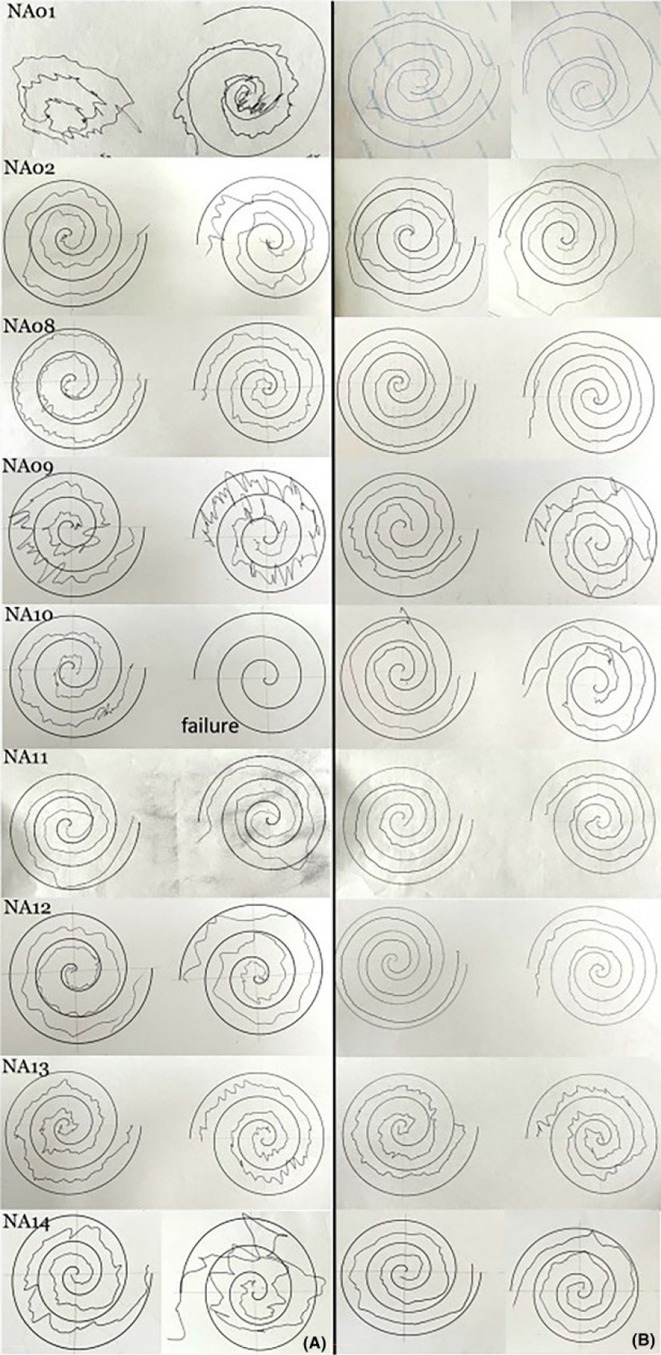
**FIGURE 2** (A) Spiral Archimedes drawing executed at baseline and (B) at POST‐1. Right‐hand drawing is on the right and left‐hand drawing is on the left. Note that subject NA10 could not initiate the task for the right hand at baseline.

**Conclusion:** This study investigated the effects of low‐dose perampanel in individuals with Familial Adult Myoclonus Epilepsy2 (FAME2), a hereditary condition characterized by epilepsy and tremors. Perampanel, an antiepileptic drug, blocks AMPA receptors in the brain, reducing excessive neural activity that causes seizures and abnormal movements. The results showed significant symptom improvement, which correlated with changes in brain activity as measured by neurophysiological tests. This study suggests that perampanel helps regulate abnormal brain signals and may help managing FAME2 symptoms.

**Disclosure:** Nothing to disclose.

## EPO‐370

### Real‐world experience with cannabidiol as add‐on treatment in patients with Lennox‐Gastaut syndrome

#### G. Bruschi ^1^; L. Licchetta^2^; C. Stipa^2^; V. Tontini^2^; B. Mostacci^2^; L. Muccioli^2^; V. Viola^1^; A. Boni^2^; D. Cordelli^3^; F. Bisulli^1^

##### ^1^Department of Biomedical and Neuromotor Sciences, University of Bologna, Bologna, Italy; ^2^IRCCS Institute of Neurological Sciences of Bologna, Reference Center for Rare and Complex Epilepsies – EpiCARE, Bologna, Italy; ^3^Department of Medical and Surgical Sciences (DIMEC), University of Bologna, Bologna, Italy

**Background and Aims:** The efficacy of Cannabidiol (CBD) has been established in several clinical trials and long‐term open‐label extension studies on Lennox‐Gastaut syndrome (LGS)1,2. This study evaluates the sustained effectiveness and safety of CBD in an adult cohort of LGS patients.

**Methods:** We retrospectively included all LGS patients referred to the adult Epilepsy Center of our Institute treated with CBD. Follow‐ups occurred at 3, 6, and 12 months. The primary endpoint was sustained effectiveness. Secondary endpoints included retention rate, improvement of behavioral/psychiatric disorders, and incidence of adverse effects (AEs).

**Results:** A total of 37 adult patients (M/F: 22/15, mean age: 36.5 ± 12 years) were included. All had refractory epilepsy previously treated with a mean of 11 anti‐seizure medications and had intellectual disability (ID); 21 (56.8%) had concomitant behavioral or psychiatric disorders. Eighteen patients (48.6%) showed a reduction in seizure frequency, and 10 patients (27%) reported improved quality of life. AEs were noted in 23 patients (60%), with drowsiness (29.7%) and elevated transaminases (10.8%) being the most common. At the last follow‐up, 16 patients (43.2%) continued CBD therapy at an average dose of 9.7 mg/kg/day: 12 (32.4%) with a sustained reduction in seizure frequency, and 4 (10.8%) with a transient or inadequate response. Conversely, 21 patients (56.8%) discontinued CBD due to long‐term inefficacy (29.7%) or AEs (27%) after 7.8 ± 6.7 months.

**Conclusion:** CBD appears to be a viable alternative in drug‐resistant adult patients. Among the responding patients, a significant percentage has shown an improvement of seizures frequency. Moreover, CBD is relatively well tolerated in our cohort.

**Disclosure:** Nothing to disclose.

## EPO‐371

### The prediction of seizure freedom in non‐lesional temporal lobe epilepsy based on a machine learning approach

#### P. Riha^1^; I. Dolezalova ^2^; R. Marecek^1^; M. Gajdos^1^; L. Vojtisek^1^; P. Coufal^1^; O. Strycek^2^; J. Kocvarova^2^; M. Pail^2^; M. Brazidl^1^; I. Rektor^1^

##### ^1^CEITEC‐Central European Institute of Technology, Multimodal and Functional Neuroimaging Research Group, Masaryk University, Brno, Czechia; ^2^Department of Neurology, St. Anne's University Hospital and Faculty of Medicine, Masaryk University, Brno, Czechia

**Background and Aims:** Introduction: Resective brain surgery represents the only therapeutic option that can lead to long‐term seizure cessation in patients with drug‐refractory epilepsy. In this study, we focused on predicting surgical seizure‐free outcomes in a group of patients with non‐lesional temporal lobe epilepsy (TLE).

**Methods:** Methods: We retrospectively identified a cohort of 19 drug‐resistant non‐lesional TLE patients who underwent surgical treatment and an extended pre‐surgical MRI protocol that included 25 imaging methods (IMs). Each IM was evaluated by three different metrics which were derived based on the known extent of resection, resulting in a total of 75 features for each patient. We then selected the 10 most discriminative features to construct three machine learning (ML) models: Multi‐Line Perceptron (MLP), Gaussian Naive Bayes (GNB), and Support Vector Machine (SVM). The performance of each model was assessed by its accuracy.

**Results:** Results: For 19 non‐lesional TLE patients, 10 IMs were selected as the most discriminative. The majority of the most discriminative features were based on a metric that compared tissue features within the resection to the immediate vicinity of the resection. The highest average accuracy of 80 % was obtained in the MLP and GNB model. The average accuracy of the SVM model was 70%. The highest accuracy of 89 % was present in the MLP model that included the nine most discriminative features.

**Conclusion:** Conclusion: Our study demonstrates that ML models can effectively predict surgical outcomes in patients with non‐lesional temporal lobe epilepsy, achieving an accuracy of up to 89% with the MLP model.

**Disclosure:** Nothing to disclose.

## EPO‐372

### Ictal and postictal central apnea in focal epilepsy due to NPLR3 pathogenic variants

#### M. Burani ^1^; G. Giovannini^2^; N. Orlandi^1^; M. Pugnaghi^2^; A. Vaudano^1^; A. Ballerini^1^; N. Biagioli^1^; L. Taruffi^1^; S. Scolastico^1^; L. Madrassi^1^; E. Micalizzi^3^; I. Florindo^4^; S. Meletti^1^

##### ^1^Department of Biomedical Metabolic Sciences and Neurosciences, University of Modena and Reggio Emilia, Modena, Italy; ^2^Neurophysiology Unit and Epilepsy Centre, Neuroscience Department, Modena AOU, Modena, Italy; ^3^Neurophysiology Unit, Department of Neuroscience, IRCCS San Martino Hospital, Genoa, Italy; ^4^Neurology Unit, University Hospital of Parma, Parma, Italy

**Background and Aims:** Recently, the association between mTOR pathway gene mutations and focal epilepsy with ictal and postictal central apnea and the increased risk of SUDEP has been provided in animal models. NPLR3, together with NPLR2 and DEPDC5, make the GATOR1 complex, which regulates mTOR signalling. Our group previously described a cohort of patients with DEPDC5 mutations who exhibited ictal and postictal central apnea (PICA). Here, we describe three new patients with pathogenic sequence variants in NPLR3 with MRI‐negative focal epilepsy who presented with PICA.

**Figure:**
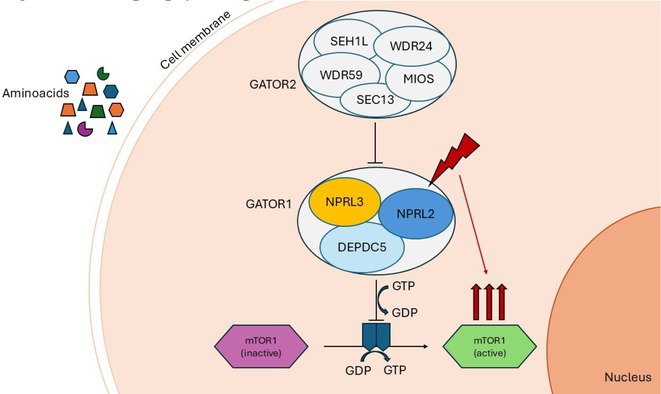
**FIGURE 1** A schematic overview of the GATOR1/2‐mTOR pathway. Mutations in GATOR genes lead to mTORC1 upregulation, resulting in hyperactivation of this pathway.

**Methods:** Three patients (two females; mean age 29.7 years), affected by focal epilepsy, were admitted to our Epilepsy Monitoring Unit in 2024 and underwent long‐term video‐EEG monitoring (LTVM) with cardiorespiratory polygraphy, neuropsychological tests, and 3T brain MRI with HARNESS protocol.

**Results:** During LTVM, a total of six seizures were recorded (50% during sleep), and in 83.3% of these, we observed ictal and postictal apnea with oxygen desaturation up to 79%. None of the patients were aware of respiratory distress and reported shortness of breath or poor sleep quality. None of them had known structural aetiology. Genetic testing (NGS panel) revealed a pathogenic mutation in NPLR3.

**Figure:**
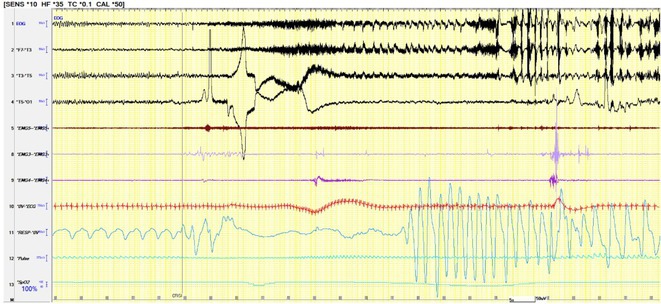
**FIGURE 2** 2 minutes of long‐term EEG monitoring of a temporal lobe seizures with a prolonged apnea (>30 seconds) right after the EEG seizure onset, shown in the RTA channel. The apnea is followed by marked tachypnea and oxygen desaturation.

**Conclusion:** These three new cases of peri‐ictal respiratory alteration associated with NPLR3 mutation confirm the association between mTOR pathway and central apnea, a recognized SUDEP risk factor. Furthermore, our findings highlight: 1) the need of respiratory polygraphy during LTVM in order to detect peri‐ictal breathing alterations; 2) the importance of offering genetic testing to patients with focal epilepsy without structural aetiology and peri‐ictal breathing disorders.

**Disclosure:** Nothing to disclose.

## EPO‐373

### Effectiveness, adherence and safety of cenobamate in patients with focal refractory epilepsy: Results of CiFES study

#### M. Ruiz Perelló; M. López López; F. Salazar Hernandez; B. Gomez Gozálvez; J. Bermejillo Barrera; J. Fajardo Sanchis; D. Vidal Mena; T. Espinosa Oltra; M. Martinez Zarco; M. Ortega Ortega; A. Savolainen; D. López Seguirá; E. Conesa Garcia; J. Garcia Carmona

##### Neurology, Hospital Santa Lucia de Cartagena, Murcia, España

**Background and Aims:** Objectives: We aimed to assess the effectiveness, adherence and safety of cenobamate (CEN) in a cohort of patients diagnosed with refractory focal epilepsy in clinical practice

**Methods:** Materials and methods: The CiFES (Cenobamate in Focal Epilepsy Study) study is an observational, retrospective study comparing the following variables up to 6 months after initiating CEN: number of monthly epileptic seizures, concomitant antiepileptic drugs (AEDs) compared as the number and as the corresponding Defined Daily Dose (DDD), the retention rate at 6 months and the side effects related with CEN withdrawal or dose adjustment

**Results:** Results: 41 patients were included. 27 (65.8%) were male, 71% between 30‐50 years of age, 57% diagnosed 10‐20 years ago, 48% with focal motor seizures and 4.13 ± 0.52 seizures per month. Treatment with CEN significantly reduced the number of epileptic seizures from baseline (13.12 ± 0.75) at 1 month (9.74 ± 0.42, *t* 1.40 = 4.863; *p* = 0.001), 3 months (1.96 ± 0.57, *t* 1.40 = 6.810; *p* = 0.001) and at 6 months (1.69 ± 0.48, *t* 1.40 = 6.938; *p* = 0.001). 5 (12%) patients were seizure‐free at 6 months. Furthermore, the number of concomitant AEDs was significantly lower after 6 months with CEN (2.57 ± 0.20 vs. 2.91 ± 0.23, *t* 1.40 = 2.676; *p* = 0.041) and the DDD significantly (*p* = 0.007) decreased from 5.87 ± 0.31 to 4.34 ± 0.29 after 6 months. 5 patients suffered side effects being dizziness the most reported (*n* = 2, 4.8%). Only 1 patient discontinued CEN after 6 months being the retention rate of 97% in the study period.

**Conclusion:** Conclusions: CEN is an effective and well tolerated antiepileptic treatment for patients with drug‐resistant focal epilepsy.

**Disclosure:** Nothing to disclose.

## EPO‐374

### Seizure outcomes and risk of post‐stroke epilepsy in patients with acute symptomatic post‐stroke seizures

#### N. Orlandi ^1^; G. Giovannini^2^; G. Bigliardi^2^; S. Maffei^2^; M. Pugnaghi^2^; A. Vaudano^2^; N. Biagioli^1^; S. Scolastico^1^; L. Madrassi^1^; M. Burani^1^; L. Taruffi^1^; S. Meletti^2^

##### ^1^Department of Biomedical, Metabolic and Neural Sciences, University of Modena and Reggio Emilia, Modena, Italy; ^2^Neurology Unit, OCB Hospital, AOU Modena, Italy

**Background and Aims:** Acute symptomatic seizures (AS) are considered among the most relevant factors for the development of post‐stroke epilepsy (PSE). Herein, we explored seizures’ outcome in a cohort of adult patients with AS after a first‐ever ischemic stroke.

**Methods:** Observational, single‐center, retrospective study of patients admitted to the Stroke Unit of Modena Academic Hospital (Italy) from January 1st 2004 to December 31st 2022. Acute (AS) and remote (RS) symptomatic seizures were defined according to ILAE definitions. Patients with AS were divided among those who experienced single seizures (SS), seizure cluster (SC) and status epilepticus (SE). Kaplan–Meier survival analyses and Cox proportional hazard regression models were used to estimate seizure freedom as longterm outcome.

**Results:** 49 patients (mean age: 69.2 y/o; 51% female) were included: 25 (51%) experienced SS, 9 (18%) SC and 15 (31%) SE. Overall, 8 patients (16%) developed RS (mean follow‐up: 55.8 months). Cumulative probability of seizure freedom at 5 years after stroke was 96% in SS, 69% in SC and 61% in SE sub‐groups, respectively. The risk of RS development was higher in case of SC (HR 7.8 95% CI 0.81–76.5; *p* = 0.08) and SE (HR 16.1 95% CI 1.7–153.2; *p* = 0.02) compared to SS. In 20 out of 49 patients, antiseizures medications were withdrew without further seizures (17 SS, 1 SC and 2 SE; *p* < 0.001).

**Conclusion:** In our cohort, the risk of PSE appeared to be influenced by the severity of seizures’ phenomena in the acute phase after stroke.

**Disclosure:** Nothing to disclose.

## EPO‐375

### Risk of seizure recurrence after antiseizure medications withdrawn in patients with acute post‐stroke seizures

#### N. Orlandi ^1^; G. Giovannini^2^; G. Bigliardi^2^; S. Maffei^2^; M. Pugnaghi^2^; A. Vaudano^2^; N. Biagioli^1^; S. Scolastico^1^; L. Madrassi^1^; M. Burani^1^; L. Taruffi^1^; S. Meletti^1^

##### ^1^Department of Biomedical, Metabolic and Neural Sciences, University of Modena and Reggio Emilia, Modena, Italy; ^2^Neurology Unit, OCB Hospital, AOU Modena, Modena, Italy

**Background and Aims:** The question of whether discontinue antiseizure medications (ASMs) in patients with acute symptomatic post‐stroke seizures (AS) represents an important issue. Herein, we explored seizures’ outcome in a cohort of adult patients with AS after a first‐ever ischemic stroke.

**Methods:** Observational, single‐center, retrospective study of patients admitted to the Stroke Unit of Modena Academic Hospital (Italy) from January 1st 2004 to December 31st 2022. Acute (AS; <1‐week from stroke) and remote (RS) seizures were defined according to ILAE classification. Patients with AS were further divided in single seizures (SS) and seizure cluster/status epilepticus (SC/SE). Kaplan–Meier survival analyses was used to estimate seizure freedom as longterm outcome.

**Results:** 49 patients (mean age: 69.2 y/o; 51% female) were included: 25 (51%) experienced SS and 24 (49%) SC/SE. Overall, 8 patients (16%) developed RS (55.8 months). In 23 patients, ASMs were withdrawn after a mean of 8 months since stroke: 68% of patients with SS (17/25) and 25% of patients with SC/SE (6/24) (*p* = 0.003). After ASMs withdrawn 3 out of 23 patients experienced RS, all in the SC/SE subgroup (follow‐up 55.5 months). Cumulative probability of seizure freedom at 1‐year after ASMs withdrawn resulted to be significantly lower in case of SC/SE compared to SS (44% vs 100%; *p* < 0.001).

**Conclusion:** The risk of post‐stroke epilepsy after ASMs withdrawn was higher in patients with acute symptomatic status epilepticus or seizure cluster, underlying the role of acute repetitive seizures in the development of the epileptogenic network.

**Disclosure:** Nothing to disclose.

## EPO‐376

### Predictors of good VNS response in 129 patients with generalized drug‐resistant epilepsy (DRE)

#### S. Aubert‐Conil ^1^; R. Carron^1^; D. Scavarda^2^; A. Paz Paredes^2^; N. Villeneuve^3^; A. Lepine^3^; F. Pizzo^1^; F. Bonini^1^; G. Daquin^1^; L. Vaugier^1^; J. Makhalova‐Scholly^1^; S. Lagarde^1^; A. Trebuchon^1^; F. Bartolomei^1^

##### ^1^Epileptology and Cerebral Rhythmology, Timone Hospital, Marseille, France; ^2^Pediatric Neurosurgery, Timone Hospital, Marseille, France; ^3^Pediatric Neurology, Timone Hospital, Marseille, France

**Background and Aims:** Identifying best candidates for VNS among all DRE patients remains challenging, as favorable prognostic factors are not clearly defined and only few large VNS cohorts are reported in the literature. The aim of this research is 1/ to analyze the VNS responder rate in our patients’ population and in its subgroups and 2/ to try to delineate favorable prognostic factors for VNS therapy in this population.

**Methods:** This single‐center (Marseille, France) retrospective study collected data from July 2000 to June 2024 from patients with generalized DRE who had VNS implantation with a follow up of at least one year. Univariate, multivariate, univariate logistic regression, stepwise and penalized logistic regression (LASSO) analyses were conducted.

**Results:** Among all DRE patients with VNS implantation (*n* = 401) and sufficient follow‐up, 129 patients had generalized DRE, most of them Lennox‐Gastaut Syndrome (LGS, *n* = 82) or Idiopathic Generalized Epilepsy (IGE, *n* = 27). Mean post‐implantation follow‐up was 8 years and 9 months (1–21 years). In the whole cohort (LGS + IGE patients), regardless of age, 43% were VNS responders (≥50% seizure reduction); 14% had 75–100 % seizure reduction. The variables better associated with a favorable VNS outcome were male gender (*p* = 0.00145) and older age at end of study and at implantation (*p* = 0.00585), regardless of epilepsy subtype.

**Conclusion:** VNS is a very good treatment option for generalized DRE. Older age and male gender are favorable prognostic factors in this study, but more extensive data analysis are required to confirm these findings.

**Disclosure:** Nothing to disclose.

## Cerebrovascular diseases 3

## EPO‐378

### Continuous blood pressure monitoring after thrombectomy for ischemic stroke – Experience of a stroke unit

#### A. Cabral ^1^; S. Casanova^1^; D. de Pinho^1^; L. Castro Rocha^1^; M. Rocha^2^; J. Novo^2^; L. Paredes^2^; H. Costa^2^; T. Gregório^2^; M. Veloso^1^; M. Ribeiro^3^; S. Castro^3^; M. Rodrigues^3^; A. Araújo^3^; P. Pires^3^; P. Barros^2^

##### ^1^Neurology Department, Local Health Unit of Gaia Espinho, Porto, Portugal; ^2^Stroke Unit, Local Health Unit of Gaia Espinho, Porto, Portugal; ^3^Interventional Neuroradiology Unit, Radiology Department, Local Health Unit of Gaia Espinho, Porto, Portugal

**Background and Aims:** Blood pressure (BP) control after endovascular treatment (EVT) for acute ischemic stroke (AIS) is essential. Elevated BP is linked to an increased risk of hemorrhagic transformation, yet the optimal BP target remains unclear. Most trials have relied on non‐invasive, intermittent BP monitoring (<100 measurements/24 h) and reported challenges in controlling BP.

**Methods:** We included AIS patients undergoing thrombectomy at our center. Continuous BP monitoring with an arterial catheter was performed, and individualized BP targets were defined. We conducted a descriptive analysis of BP control in the first 24 h post‐EVT and compared our findings with the literature.

**Results:** Among 58 patients, 36.2% received intravenous thrombolysis, and 79.3% achieved mTICI2c/3 recanalization. Each patient had a median of 1346 (IQR 178) BP measurements in the first 24 h. Overall, 6.6% of measurements exceeded targets, with 17.4% and 1.3% exceeding targets in strict (SBP <140 mmHg) and permissive (SBP 140–220 mmHg) groups, respectively. The standard deviation of SBP was 16 mmHg (IQR 6). Antihypertensive treatment was used in 37.9% of patients, and 19% required infusions. Symptomatic hemorrhagic transformation occurred in 1 patient (1.7%). At 3 months, 53.4% had mRS 0–2 or their previous mRS, with 10.3% mortality.

**Conclusion:** The lack of evidence of a clear BP target may result from trial methodologies. In the BP‐TARGET and PRAISE trials (<50–100 measurements/24 h), 35–50% and 15–30% of SBP measurements exceeded targets, respectively. In our sample, Continuous monitoring kept patients >90% of the time within BP targets, demonstrating its feasibility for effective BP control.

**Figure:**
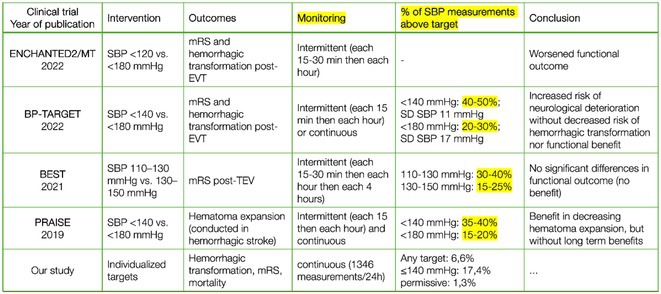
**FIGURE 1** Comparison between the results of our study and the results of clinical trials that have tested different blood pressure targets after endovascular therapy for acute ischemic stroke (and targets after hemorrhagic stroke in the case of the PRAISE trial).

**Disclosure:** Nothing to disclose.

## EPO‐379

### Red cell distribution width as inflammatory marker in correlation with intracranial artery calcification

#### D. Boshnjaku ^1^; F. Jashari^2^

##### ^1^Medical Faculty Skopje ‘Ss Cyril and Methodius,’ Skopje, North Macedonia; ^2^University Clinical Center of Kosovo, Clinic of Neurology, Pristina, Kosovo

**Background and Aims:** Evidence suggests that intracranial artery calcification and red cell distribution width (RDW) are independent risk factors for stroke. This study aims to explore the correlation between these risk factors and their ability to predict the severity of ischemic stroke.

**Methods:** We included 117 patients in this observational cohort study with a mean age of 67.3 ± 11.76 years (50.86% females), who had experienced an ischemic stroke or transient ischemic attack. RDW was recorded from blood samples, and types of intracranial artery calcification were evaluated using non‐contrast Computed Tomography (CT) of the head with a standardized method in the anterior and posterior brain arterial circulation systems.

**Results:** The total anterior intracranial artery calcification score was correlated with RDW. We found a significant correlation between RDW and total anterior intracranial artery calcification Persons correlation coefficient was 0.359 (*p* = 0.001).

**Figure:**
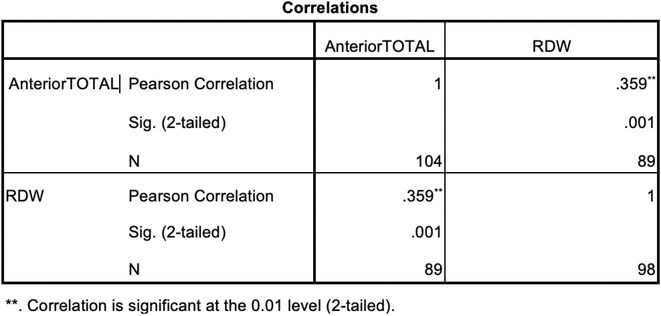
**TABLE 1** Table presentation of correlation between RDW and subtypes of calcification.

**Figure:**
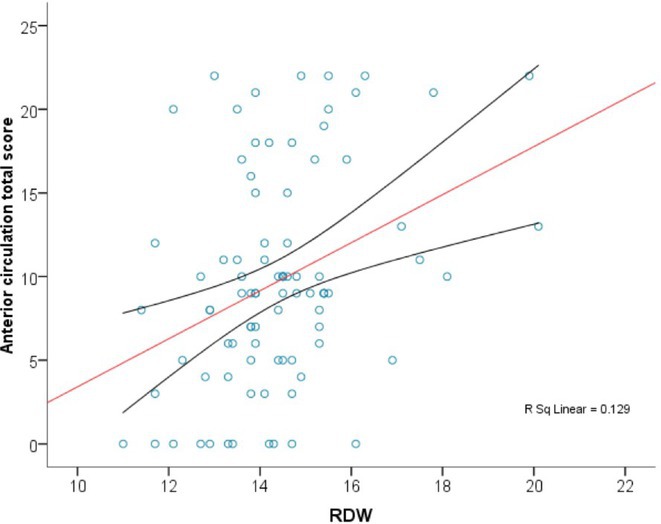
**FIGURE 1** Correlation between subtypes of artery calcification in anterior territory with RDW%

**Conclusion:** The correlation observed between RDW and cerebral artery circulation in the anterior system may be an important marker for the effects of systemic inflammation on intracranial artery calcification, which is an independent risk factor for ischemic stroke.

**Disclosure:** No.

## EPO‐380

### The role of post stroke inflammation in acute EEG alterations

#### G. Prandin; G. Furlanis; L. Mancinelli; F. Palacino; E. Vincis; E. Ricci; M. Quagliotto; P. Caruso; M. Naccarato; P. Manganotti

##### Clinical Unit of Neurology, Department of Medicine, Surgery and Health Sciences, University of Trieste, Trieste, Italy

**Background and Aims:** Post stroke inflammation is a well‐known complication which plays a significant role in outcome prediction. Previous studies found a strong association of both slow wave activity (SA) and epileptiform discharges (ED) with stroke outcome. However, there are few data about the relationship between inflammation and on acute phase EEG alteration.

**Methods:** we retrospectively analysed data of patients with an EEG recording after admission to the SU of Trieste. We excluded haemorrhagic strokes, stroke mimics, infratentorial ischemic strokes, factors which can alter the inflammatory biomarkers (infections, immunosuppressant drugs) and factors which can modify the EEG (previous strokes, antiseizure medication, cognitive impairment). The blood tests were collected after 24h the admission (e.g., C‐reactive‐protein (CRP), erythrocyte sedimentation rate (ESR), white blood count, neutrophil and lymphocyte count and their ratio (NLR)). We compared the stroke characteristics and risk factors between SA vs no‐SA and ED vs no‐ED.Then we performed a multivariate analysis (logistic regression).

**Results:** 316 patients were analysed 0.140 have SA and 67 ED. After multiple adjustment, NLR was not associated with SA (OR 0.98, CI 95% 0.89–1.08, *p* = 0.709), however NLR (OR 1.01, CI 95% 1.03–1.16, *p* = 0.002) remains significantly associated with ED. SA remains associated with some stroke characteristics as admission NIHSS (OR 1.15, CI 95% 1.07–1.24, *p* < 0.001) and haemorrhagic transformation (OR 4.23, CI 95% 1.26–14.21, *p* = 0.020).

**Figure:**
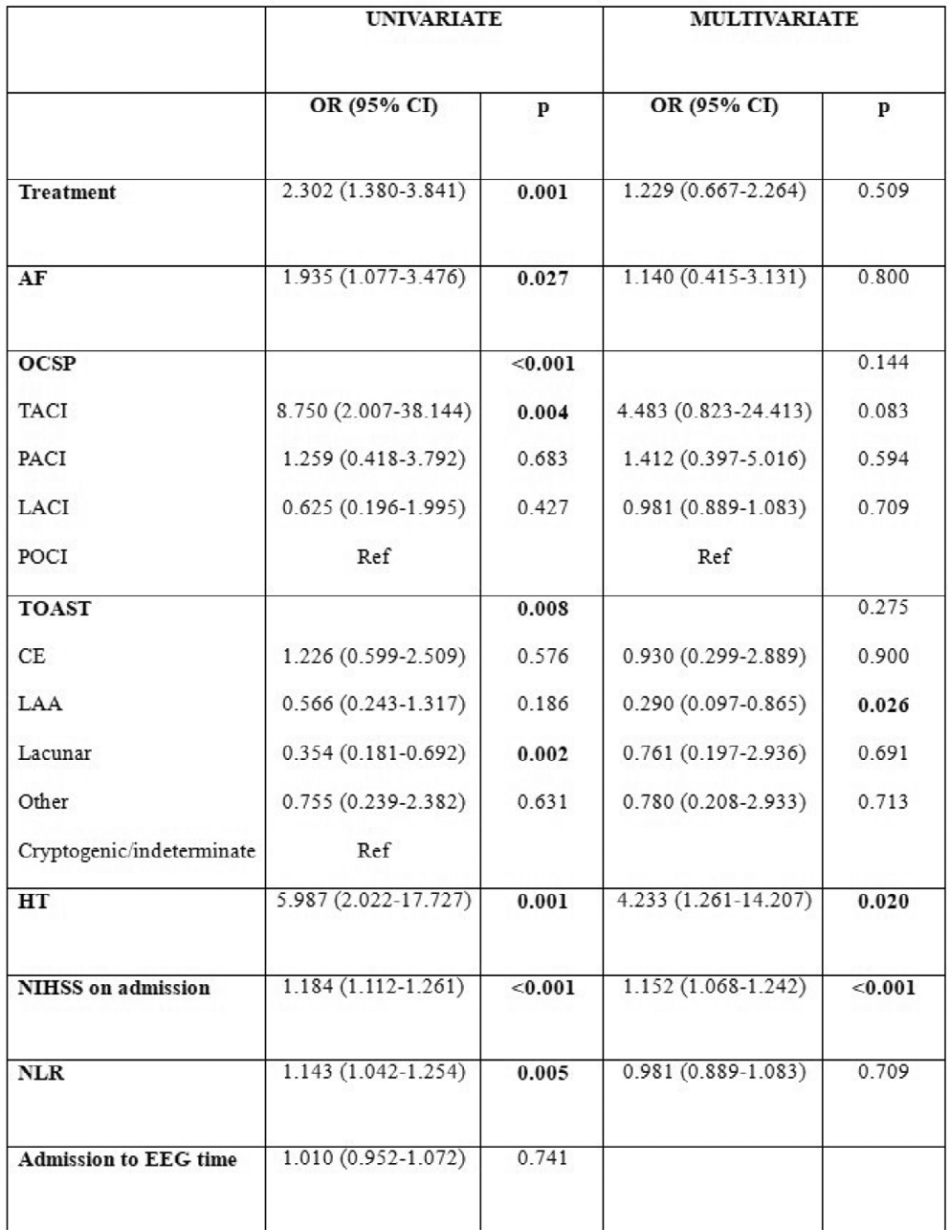
**TABLE 1** Univariate and multivariate regression for SA.

**Figure:**
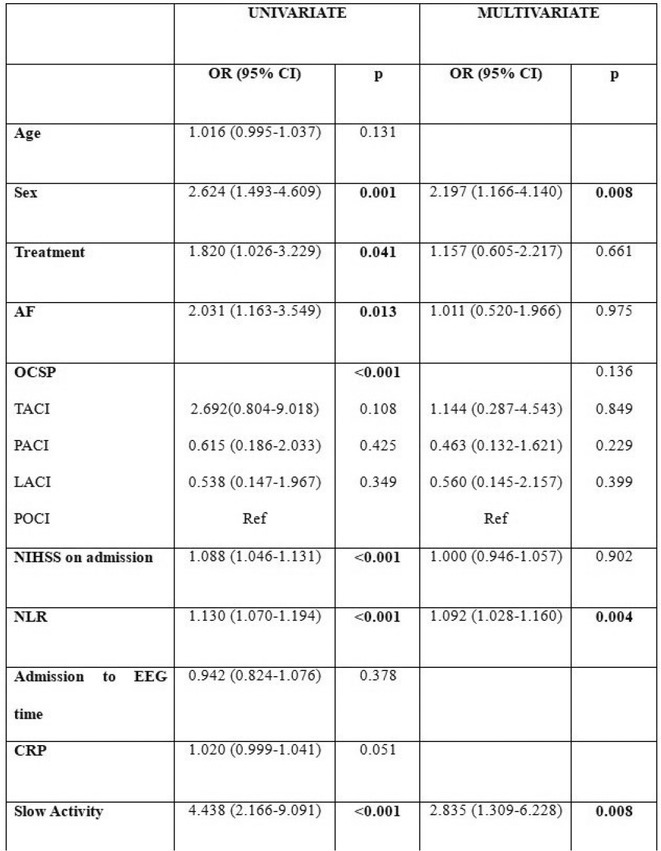
**TABLE 2** Univariate and multivariate regression for ED

**Figure:**
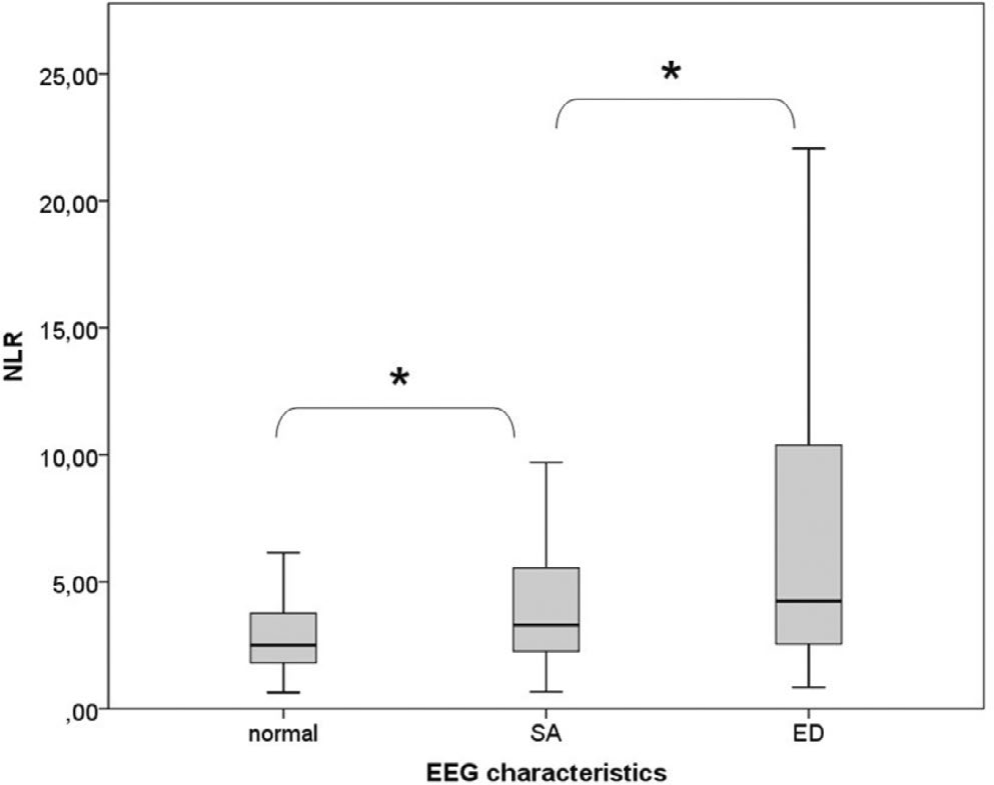
**FIGURE 1** NLR levels in the 3 groups. (*) if *p* < 0.01

**Conclusion:** post stroke inflammation may play different roles in acute EEG. NLR may be more related to the development of ED than SA. SA on EEG is more related to stroke severity and haemorrhagic transformation.

**Disclosure:** Nothing to disclose.

## EPO‐381

### Complex functional TCD examinations in patients with significant internal carotid artery stenosis

#### H. Pál ^1^; R. Magyar‐Stang^1^; B. Csányi^2^; A. Gaál^1^; Z. Mihály^3^; Z. Czinege^3^; P. Sótonyi^3^; T. Horváth^5^; B. Dobi^4^; D. Bereczki^1^; A. Koller^5^; R. Debreczeni^1^

##### ^1^Department of Neurology, Semmelweis University, Budapest, Hungary; ^2^János Szentágothai Neurosciences School of PhD Studies, Semmelweis University, Budapest, Hungary; ^3^Department of Vascular and Endovascular Surgery, Semmelweis University, Budapest, Hungary; ^4^HUN‐REN Neuroepidemiological Research Group, Budapest, Hungary; ^5^Research Center for Sport Physiology, Hungarian University of Sports Science, Budapest, Hungary

**Background and Aims:** In cases of 48 patients with significant Internal Carotid Artery (ICA) stenosis (ICAS‐70%<) a multimodal Transcranial Doppler US examination was performed before undergoing vascular surgery.

**Methods:** 48 patients were recruited. The applied four vasoactive stimuli were the Valsalva Maneuver (VM), Common Carotid artery Compression test (CCC), Hyperventilation (HV) and Breath Holding (BH) tests. The blood flow velocity changes of both MCA were registered by TCD. The patients' systemic hemodynamic parameters were also simultaneously recorded: ECG, continuous non invasive arterial blood pressure measurement and capnography. A number of time and amplitude variables were defined that took into account arterial blood pressure changes in addition to BFV changes. The Wilcoxon Matched Pair Test was used for the statistical analysis of the variables of the stenotic and contralateral side.

**Results:** Due to technical reasons VM of 34, HV‐BH test of 31 and CCC test of 26 patients were eveluated. In the case of HV‐BH tests the BHI did not show a significant difference regarding the stenotic and contralateral side (BHIICAop (mean (SD)): 2.36 ± 2.38, BHIICAnonop (mean (SD)): 2.33 ± 1.77 Wilcoxon Matched Pair Test *p* = 0.89), while in the dynamic tests (CCC and VM) both time and amplitude variables proved to be sensitive for detecting hemodynamic disturbances.

**Conclusion:** In cases of carotid stenosis requiring surgical treatment, we recommend the use of stimuli that induce pressure‐flow changes for preoperative ischemic risk assessment.

**Disclosure:** Nothing to disclose.

## EPO‐382

### AI‐based software to support mechanical thrombectomy transfer decision in low‐volume primary stroke centers

#### K. Koszarska ^1^; A. Kotlińska^1^; K. Wąchała^1^; S. Lepak^1^; K. Jucha^1^; M. Wiącek^2^; H. Bartosik‐Psujek^2^

##### ^1^Medical Collage of the University of Rzeszów, Rzeszów, Poland; ^2^Department of Neurology, Institute of Medical Sciences, Medical College of the University of Rzeszów, Rzeszów, Poland

**Background and Aims:** Incorporating artificial intelligence (AI)‐based tools into stroke workflows has been shown to significantly reduce the time to endovascular thrombectomy (EVT) in the drip‐and‐ship model. This study assesses their potential impact on low‐volume primary stroke centers.

**Methods:** We analyzed 95 consecutive anterior circulation stroke patients referred for EVT from 5 low‐volume primary stroke centers (≤12 referred patients/year) between January 2019 and April 2023. Remote transmission of radiological images was unavailable. Non‐contrast CT and CT angiography studies were retrospectively analyzed using Brainomix 360 software. Clinical data were extracted from our prospective database. EVT‐treatment decisions were simulated by two vascular neurologists blinded to outcomes.

**Results:** LVO was automatically detected in 77 (81.1%) patients. Median door‐in‐door‐out (DIDO) time was 104 minutes (interquartile range [IQR], 87–223), with time from CT angiography to EVT decision accounting for nearly half of it (median 50 minutes; IQR, 30–143). Seventy‐three patients (76.8%) could be readily qualified for EVT transfer based on AI‐generated imaging data. Transfer would be denied in 4 cases (4.2%) due to both extensive ischemic changes and poor collateral flow; all had unfavorable 3‐month outcome (modified Rankin scale of 5–6).

**Conclusion:** In approximately three‐quarters of anterior circulation EVT patients within a drip‐and‐ship model, referral decisions could be made based on AI‐generated neuroimaging data. This is particularly relevant in the workflow of low‐volume primary stroke centers, given the relatively long DIDO times. Additionally, AI‐based tools may help to prevent the transfer of a small percentage of patients at high risk of futile treatment.

**Disclosure:** Nothing to disclose.

## EPO‐383

### Comparative characteristics of stroke morbidity in Russia for 2015–2022 taking into account the impact of COVID‐19

#### M. Mitiukova ^1^; V. Shirokov^1^; A. Novikova^1^; A. Shastin^2^

##### ^1^Department of Neurology, F.F. Erisman Federal Research Center for Hygiene, Mytishchi, Moscow Region, Russian Federation; ^2^Department of Neurology, Yekaterinburg Medical Research Center for Prophylaxis and Health Protection in Industrial Workers, Yekaterinburg, Russian Federation

**Background and Aims:** All types of stroke have high levels of morbidity and disability, up to half of the patients become permanently disabled. The investigation of regional characteristics of incidence of stroke is a fundamental direction for evaluation and regulation of morbidity and mortality, especially in the context of influence on incidence of COVID‐19. Objective ‐ to study the dynamics of primary and general incidence of stroke in different regions of Russia and to compare morbidity levels in different periods from 2015 to 2022, taking into account the COVID‐19 pandemic.

**Methods:** By means of continuous statistical observation, official statistics of the primary and general morbidity of adult stroke in Russia in different regions were investigated. Cerebrovascular diseases, transient cerebral ischemic attacks, intracerebral and other intracerebral hemorrhages, brain infarctions and strokes not specified as hemorrhage or stroke were included. Calculated mean primary and general morbidity levels, variation range, standard deviation, coefficient of variation.

**Results:** In the period 2015‐2022, primary and general morbidity levels of individual subtypes of stroke had a different dynamics compared to the average long‐term indicators and during the pandemic of coronavirus infection.

**Figure:**
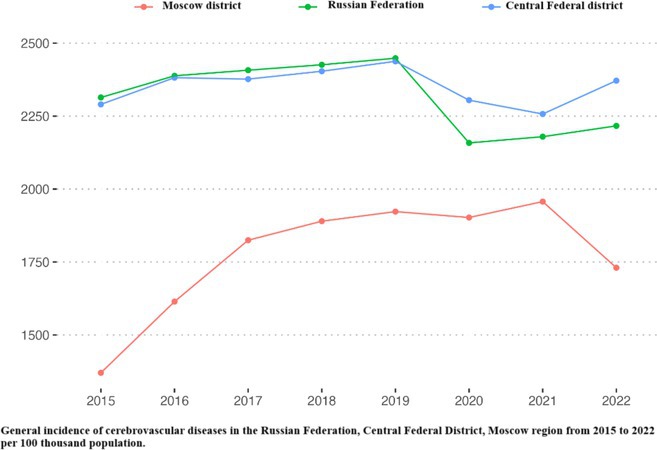
**FIGURE 1** Overall incidence of cerebrovascular diseases for 2015–2022

**Figure:**
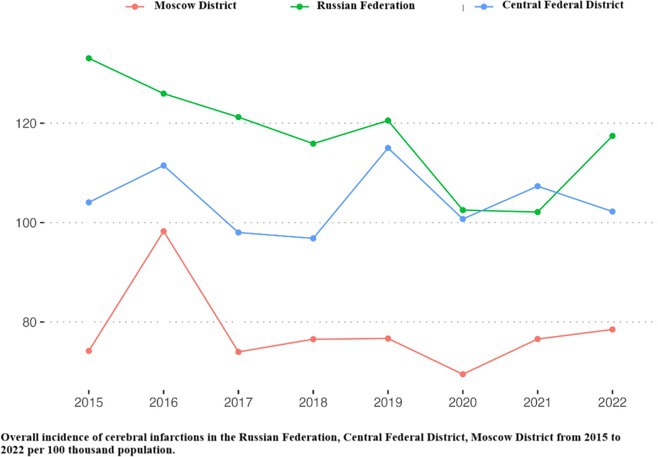
**FIGURE 2** Overall incidence of cerebral infarctions for 2015–2022

**Conclusion:** It is determined that for 2015–2022 the incidence rates are within a limited range of values, which shows the impact of various measures to reduce stroke morbidity in all studied areas. Fluctuations in stroke morbidity rates in the Russian Federation are treated as inaccurate morbidity estimates, incorrect diagnosis coding according to ICD‐10, especially in the conditional pandemic COVID‐19.

**Disclosure:** Nothing to disclose.

## EPO‐384

### One year real‐life cohort experience in bempedoic acid in secondary prevention of ischemic stroke

#### N. Mena García; G. Cabañas Engenios; M. Campos Jiménez; R. Pastor González; A. Cruz Culebras; R. Vera Lechuga; A. De Felipe Mimbreras; M. Matute Lozano; J. Masjuan Vallejo; S. García Madrona

##### Neurology Department, Ramón y Cajal University Hospital, Madrid, Spain

**Background and Aims:** Bempedoic acid (BA) antagonizes ATP citrate‐lyase, reducing cholesterol synthesis. Recently, it has been approved for the treatment of dyslipidemia, in addition to statin and ezetimibe. There is still not enough experience regarding its use in patients with ischemic stroke (IS). We aim to describe our experience in secondary prevention of stroke.

**Methods:** Prospective observational study of patients who started BA therapy as a secondary prevention of IS, treated in a single comprehensive Stroke Centre from September 2023 to December 2024.

**Results:** Fifty patients (42% female) were included, with a median of 70 years. All patients had multiple vascular risk factors (Table 1). Sixty‐five percent were atherothrombotic. We added BA to maximum tolerated statins +/‐ ezetimibe to achieve two LDL‐cholesterol (c‐LDL) targets (c‐LDL <55 mg/dL in atherothrombotic strokes, or c‐LDL <70 mg/dL in the other subtypes). The basal median c‐LDL was 79 mg/dL [IQ 37 (69.2–106.2)]. In most of them (78%), BA was combined with statins and ezetimibe, 16% received only ezetimibe, and 6% were on monotherapy. Table 2 shows the determination of c‐LDL levels at 3, 6 and 12 months. At 3 months, we observed a reduction of 17.4% in c‐LDL. These results were similar during the follow‐up with minimum decrease in efficacy. No treatment‐related adverse effects were reported. Furthermore, none of the patients suffered new vascular events.

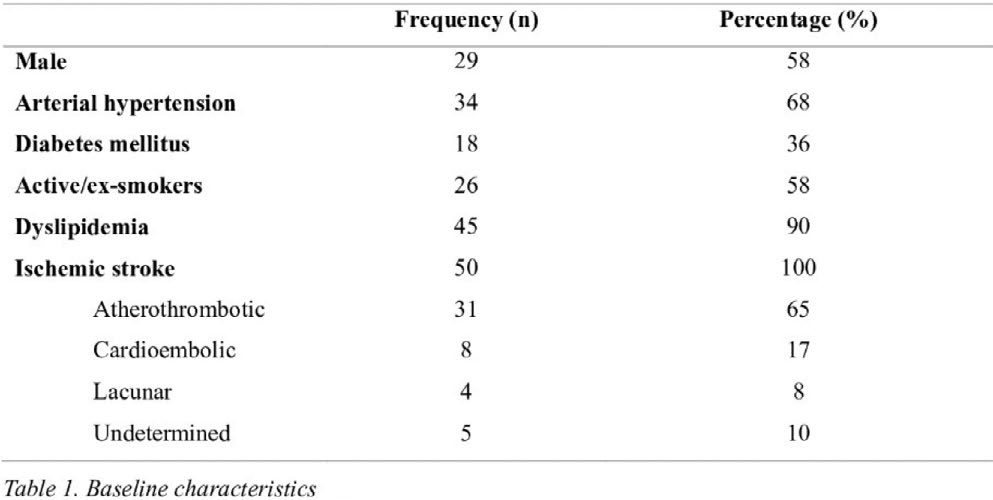

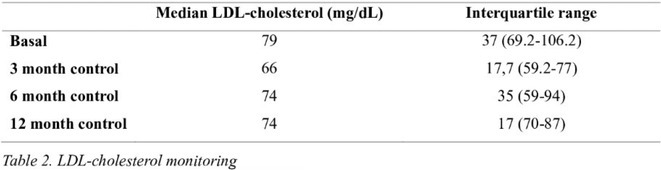

**Conclusion:** In our cohort, Bempedoic acid proved to be safe and effective in lowering LDL‐cholesterol in clinical practice. The efficacy of reduction in c‐LDL was similar to clinical trial.

**Disclosure:** Nothing to disclose.

## EPO‐385

### Good‐to‐excellent functional outcomes 90 days after large ischemic stroke stratified by ASPECTS in EVT: Meta‐analysis

#### N. Nhan ^1^; N. Vy^2^; H. Vinh^1^; T. Nghi^1^; L. Oláh^3^

##### ^1^University of Debrecen Medical School, Department of Medicine, Debrecen, Hungary; ^2^The University of Melbourne, Melbourne, Australia; ^3^University of Debrecen Medical School, Department of Neurology, Debrecen, Hungary

**Background and Aims:** Previous randomized controlled trials (RCTs) demonstrated the benefit of endovascular therapy (EVT) in achieving good outcomes for acute ischemic stroke with Alberta Stroke Program Early CT Scores (ASPECTS) ≤5, despite increased intracranial bleeding risk. However, data on 90‐day good‐to‐excellent outcomes for EVT in ASPECTS <3 versus 3–5 remain limited. This meta‐analysis compares 90‐day functional outcomes stratified into ASPECTS groups: 3–5, ≤3, and ≤5 (combined).

**Methods:** PubMed and Cochrane databases were searched for RCTs and observational studies comparing EVT versus medical management (MM) in acute ischemic stroke (ASPECTS ≤5), reporting modified Rankin Scale (mRS) 0–2 (good outcomes) and mRS 0–1 (excellent outcomes). Heterogeneity was assessed using I^2^, and random‐effects models were used where appropriate.

**Results:** Four studies (three RCTs, one cohort) with 1,225 patients (52.48% EVT) were included. At 90 days, EVT was superior to MM in achieving good outcomes in ASPECTS ≤5 (OR 3.10; 95% CI 1.63–5.88; *p* < 0.01) and ASPECTS 3–5 (OR 3.35; 95% CI 2.08–5.39; *p* < 0.01). Excellent outcomes were four times more likely with EVT in ASPECTS 3–5 (OR 4.01; 95% CI 2.02–7.94; *p* < 0.01) and three times more likely in ASPECTS ≤5 (OR 3.41; 95% CI 1.84–6.31; *p* < 0.01). However, ASPECTS ≤3 showed no significant differences in good (*p* = 0.33) or excellent (*p* = 0.60) outcomes between EVT and MM.

**Figure:**
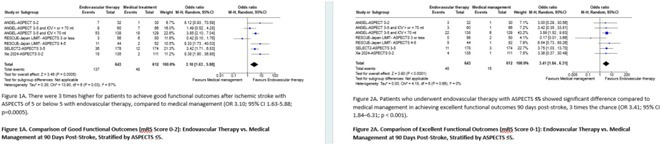
**FIGURE 1** Comparison of Functional Outcomes (mRS Score 0–2; versus 0–1): Endovascular Therapy vs. Medical Management at 90 Days Post‐Stroke, Stratified by ASPECTS ≤5.

**Figure:**
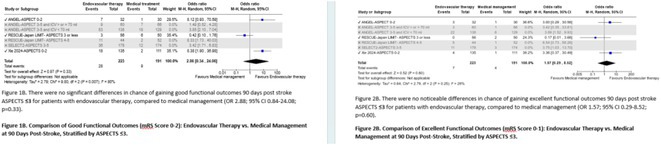
**FIGURE 2** Comparison of Functional Outcomes (mRS Score 0–2; versus 0–1): Endovascular Therapy vs. Medical Management at 90 Days Post‐Stroke, Stratified by ASPECTS ≤3.

**Figure:**
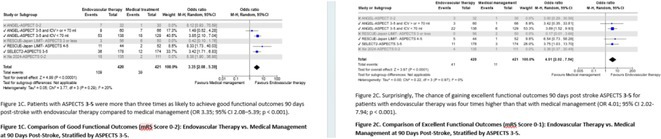
**FIGURE 3** Comparison of Functional Outcomes (mRS Score 0–2; versus 0–1): Endovascular Therapy vs. Medical Management at 90 Days Post‐Stroke, Stratified by ASPECTS 3–5.

**Conclusion:** EVT significantly improves good‐to‐excellent outcomes in ASPECTS 3–5 but shows no benefit over MM in ASPECTS <3. Further RCTs are warranted to explore EVT in ASPECTS ≤3.

**Disclosure:** Nothing to disclose.

## EPO‐386

### Herb formula Bao Yuan capsule promotes neurogenesis and neurological functional recovery in ischemic stroke mice model

#### Q. Du; R. Deng; J. Shen

##### School of Chinese Medicine, The University of Hong Kong, Pokfulam, Hong Kong

**Background and Aims:** Bao Yuan Capsule (BYC) is a patented Chinese medicine formula for health promotion and immunomodulation, but its neuroprotective effects remain unknown. In the present study, we tested the hypothesis that BYC could promote neurogenesis and neurological functional recovery in the mice model of ischemic stroke.

**Methods:** We firstly performed chemical identification studies by using QIT‐TOF‐MS technology. Then, we investigated the effects of BYC on improving the recovery of the neurological functions in transient middle cerebral artery occlusion (MCAO) ischemic mice.

**Results:** We tentatively characterized 36 compounds from the BYC extractions. BYC effectively improved locomotor ability, attenuated anxiety‐like behaviors, and enhanced the exploring behaviors, learning and memory capability in the transient MCAO mice. BYC treatment promoted the neural stem cell differentiations in the subventricular zone (SVZ) and subgranular zone (SGZ) of the MCAO mice. BYC also up‐regulated the expression of enzymes in oxidative phosphorylation of mitochondria, and its downstream Akt‐beta‐catenin signaling pathway in the hippocampus of the stroke mice. BYC significantly improved the mitochondrial functions in cultured mouse multipotent neural stem like C17.2 cells. BYC treatment promoted the neuronal differentiations in the C17.2 cells after exposed to oxygen‐glucose deprivation (OGD), whose effects were abolished by co‐treatments of ATP synthesis inhibitor oligomycin and PI3K/Akt inhibitor wortmannin. Moreover, Akt phosphorylation were dramatically reduced in oligomycin treated C17.2 in differentiation revealing that Akt might be the downstream mechanism of BYC induced neurogenesis.

**Conclusion:** BYC could promote neurogenesis and neurological functional recovery in post ischemic stroke treatment by regulating the mitochondrial functions.

**Disclosure:** Nothing to disclose.

## EPO‐387

### LA strain: A key to uncovering cardioembolic causes of transient ischemic attacks

#### S. Arnautu ^1^; D. Arnautu^1^; D. Jianu^2^

##### ^1^Internal Medicine Department, Victor Babes University of Medicine and Pharmacy, Timisoara, Romania; ^2^Neurology Department, Victor Babeș University of Medicine and Pharmacy, Timișoara, Romania

**Background and Aims:** Patients with transient ischemic attacks (TIA) often have undiagnosed paroxysmal atrial fibrillation (AF), a common cause of cardioembolic events. Since AF originates in the atria, this study investigated whether abnormalities in left atrial (LA) structure and function could help identify cardioembolic causes of TIA in patients with sinus rhythm but documented episodes of paroxysmal AF.

**Methods:** This study included 190 TIA patients, divided into two groups: those with confirmed paroxysmal AF (Group I) and those without (Group II), based on medical record assessments. Cardiac ultrasonography was performed during sinus rhythm, at least 14 days post‐TIA onset, to avoid the confounding effects of atrial stunning.

**Results:** Group I patients were older, more frequently female, had a history of stroke or TIA, and higher CHA2DS2‐VASc scores. They also exhibited increased LA volumes, reduced LA emptying fractions, and significantly altered LA deformation patterns. Multivariate logistic regression identified three independent predictors of paroxysmal AF: age >55 years, LA reservoir strain <−17%, and LA emptying fraction <51% (*p* < 0.0001).

**Figure:**
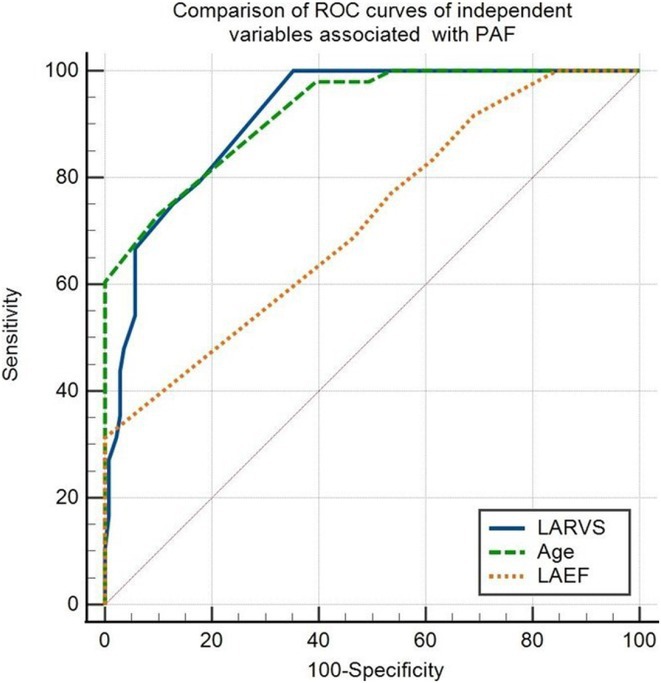
**FIGURE 1** ROC curves comparison

**Figure:**
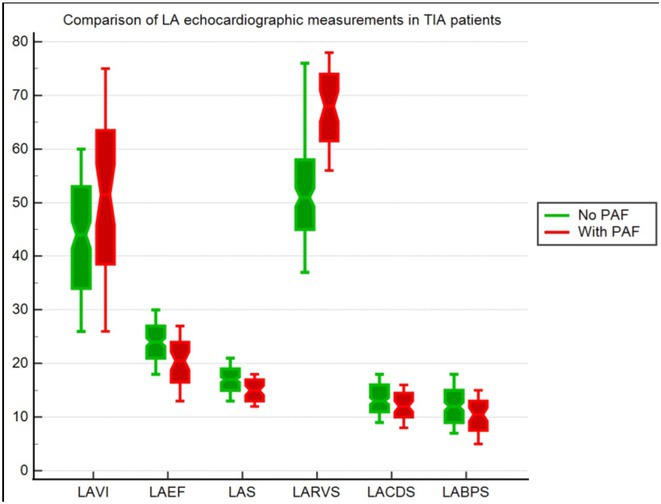
**FIGURE 2** Comparison of left atrial echocardiographic measurements in transient ischemic attack patients.

**Figure:**
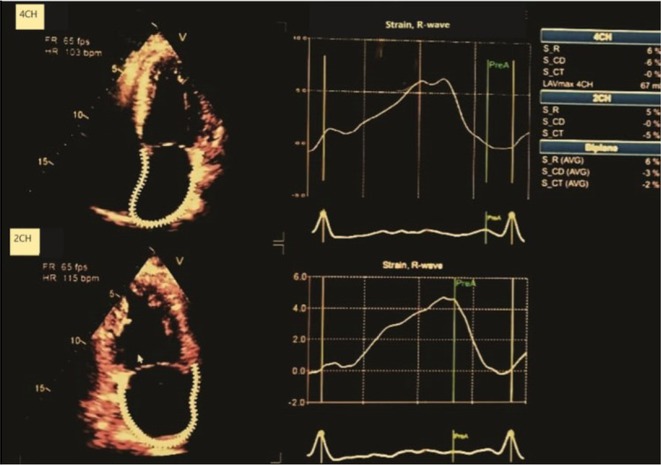
**FIGURE 3** Strain curves from left atrial speckle‐tracking, in apical four‐chamber and two‐chamber.

**Conclusion:** This research demonstrates that LA strains are independently associated with paroxysmal AF in TIA patients and may help identify cardioembolic origins of TIA. These findings have significant clinical implications, as LA 2D speckle‐tracking echocardiography (2D‐STE), not currently part of routine TIA evaluation, could provide a valuable tool for detecting atrial dysfunction and guiding targeted therapies.

**Disclosure:** Nothing to disclose.

## EPO‐388

### Effects of non‐invasive brain stimulation on cerebral blood flow in chronic ischemic stroke: A randomised control study

#### S. Narayan

##### Department of Neurology, JIPMER, Pondicherry, India

**Background and Aims:** Upper extremity impairments are common among stroke survivors. We studied the effect of repetitive transcranial magnetic stimulation (rTMS) and transcranial direct current stimulation (HD‐tDCS) on regional cerebral blood flow (CBF) in chronic ischemic stroke using single‐photon emission computed tomography (SPECT) imaging.

**Methods:** In a double‐blind, randomized controlled trial, 12 patients with chronic middle cerebral artery ischemic stroke with residual upper limb weakness were assigned to four groups of: combined real HD‐tDCS and rTMS (Group A), sham HD‐tDCS and real rTMS (Group B), real HD‐tDCS and sham rTMS (Group C), and sham HD‐tDCS and rTMS (Group D) administered for 10 consecutive days. Pre‐ and post‐treatment SPECT imaging was done using 99mTc‐ECD and acquired in Siemens Symbia T6 LEHR Collimator.

**Results:** No statistically significant changes in regional CBF were observed between the groups (*F* = 1.76, *p* = 0.23). Group A showed a maximum improved CBF (+1.67 ± 2.52) compared to Group B (−1.67 ± 1.15), Group C (+0.33 ± 2.31) and Group D (+1.33 ± 1.53). Compared to sham NIBS, the NIBS protocols showed no significant differences for the mean change in blood flow.

**Figure:**
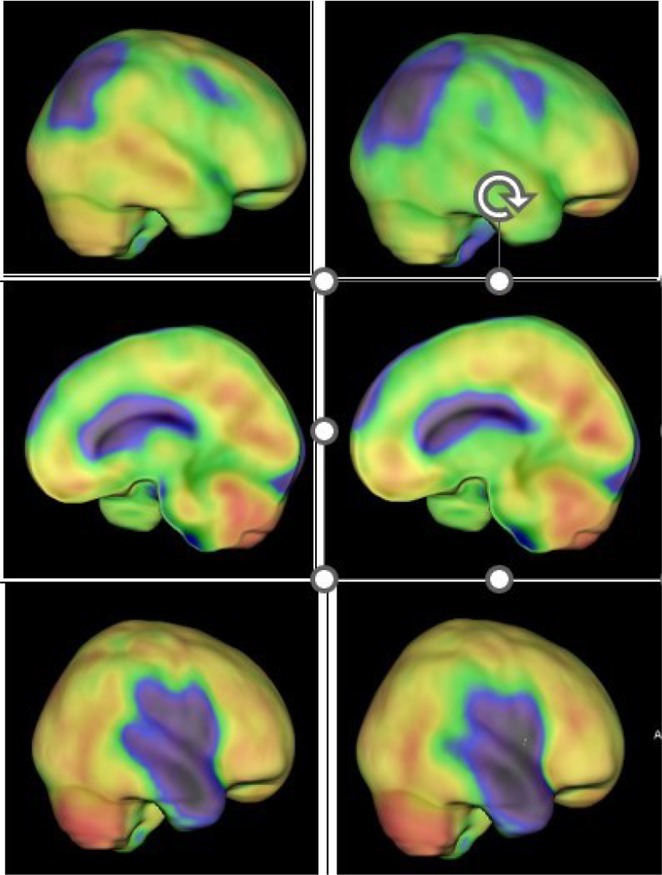
**FIGURE 1** GROUP B (Sham tDCS and Real rTMS)

**Figure:**
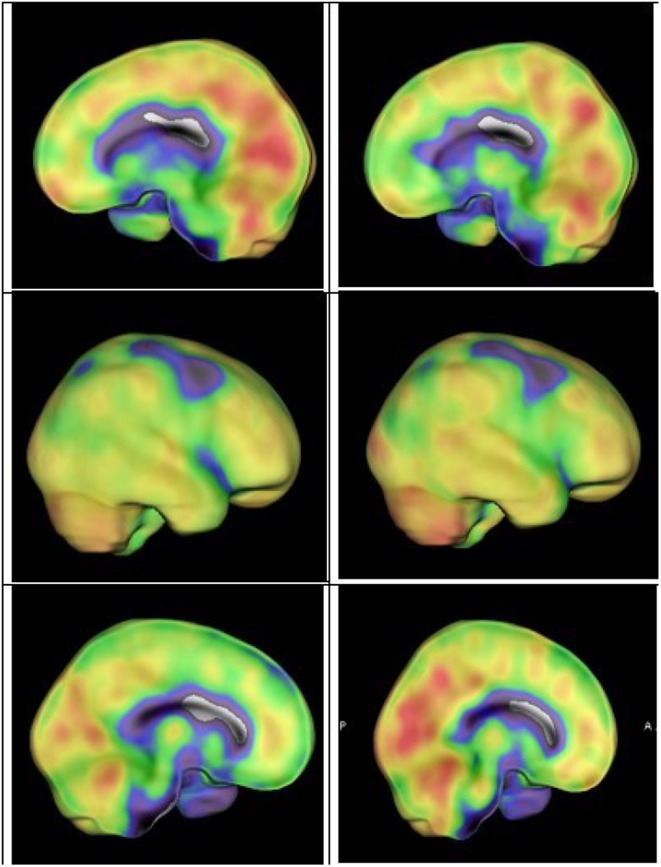
**FIGURE 2** GROUP C (Real tDCS and Sham rTMS)

**Conclusion:** This pilot study demonstrated the feasibility and safety, and potential of NIBS protocols for modifying the regional blood flow on the infarcted hemisphere and the potential for SPECT as a surrogate marker for NIBS induced modification of regional brain flow. Larger‐scale studies are warranted to validate these findings, which may give better insights into the physiological mechanisms underlying the effects of NIBS.

**Disclosure:** Nothing to disclose.

## EPO‐389

### Prognostic nutritional index as an indicator for in‐hospital mortality in patients with cerebral venous thrombosis

#### E. Uyar, A. Adıgüzel, Y. Kablan

##### Inonu University, Turgut Ozal Medical Center, Malatya, Turkey

**Background and Aims:** Prognostic nutritional index (PNI) combines albumin concentration and the lymphocyte count reflecting the nutrition, immunity status and inflammation. Several studies have reported that lower PNI is related to increased mortality in patients with various malignancies, cardiovascular diseases and ischemic stroke. However, little is known about PNI and its relationship with the mortality in cerebral venous thrombosis (CVT). Therefore, we evaluated the prognostic significance of the admission PNI for predicting in‐hospital mortality in patients with CVT.

**Methods:** This retrospective study included 50 consecutive patients admitted within 48 h of CVT onset to our clinic between January 2013 and February 2024. Patients were categorized as survivors discharged from the hospital and those who died during hospitalization. The groups were compared for demographics, risk factors, clinical symptoms, imaging characteristics, admission laboratorial parameters, and PNI. Multivariate logistic regression analysis was performed to confirm if lower PNI was associated with a hospital mortality.

**Results:** Patients who died in the hospital (*n* = 14) were significantly older compared to survivors. These patients were characterized by higher NIHSS scores, a higher prevalence of parenchymal lesion, and higher lymphocyte count. In addition, they exhibited significantly lower PNI value (*p* < 0.05). ROC curve analysis also showed that the PNI had a good predictive value for in‐hospital mortality with a cut‐off value of 41.45.

**Conclusion:** We suggested that lower PNI may serve as a valuable prognostic indicator for predicting in‐hospital mortality in patients with CVT.

**Disclosure:** Nothing to disclose.

## EPO‐390

### Impact of recanalisation level and the first pass effect on outcome in patients after M2 MCA occlusion thrombectomy

#### S. Pataky^1^; J. Fedorko^1^; P. Pedowski^1^; M. Skorvanek^2^; Z. Gdovinova ^2^

##### ^1^Department of Radiodiagnostics and Imaging Techniques, Faculty of Medicine, P.J. Safarik University, University Hospital Kosice, Košice, Slovakia; ^2^Department of Neurology, Faculty of Medicine, P.J. Safarik University, University Hospital Kosice, Košice, Slovakia

**Background and Aims:** Mechanical thrombectomy (MT) is modality of choice in treatment of acute ischemic stroke (AIS) and large vessel occlusion (LVO). Endovascular treatment of medium and distal vessel occlusions (DMVO) is currently under intensive scientific investigation. Aim of our study was to prove feasibility, effectivity and safety of MT in patients with a primary, isolated M2 MCA occlusion with focus on recanalisation level and first pass effect (FPE) as predictors.

**Methods:** We prospectively assessed 137 patients during the three years period, since July 2021 to July 2024 (Tab. 1). Primary outcome was defined by modified Rankin Scale (mRS) score 0 ‐ 2, secondary outcome included excellent functional independence (mRS 0–1) and successful recanalisation (mTICI 2c or 3). Safety outcomes included symptomatic intracerebral hemorrhage (sICH), any intracerebral (IC) hemorrhage and 90 days mortality.

**Figure:**
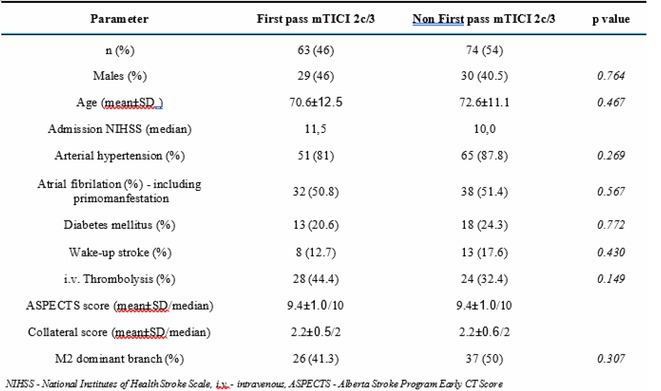
**FIGURE 1** Basic epidemiological parameters and clinical characteristics.

**Results:** We found that level of reperfusion is linked with better functional outcome, the correlation between good clinical outcome and good reperfusion level (TICI 2c or 3) reached statistical significance (*p* = 0.024) (Tab. 2). We failed to prove the importance of first pass effect (FPE) during MT of the M2 segment (Tab. 3). We also noticed a significant 31.3% mortality increase in the group of patients, where recanalisation of the occluded branch was insufficient.

**Figure:**
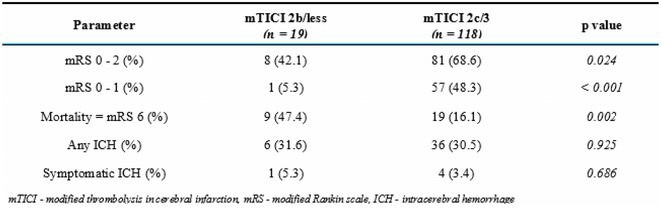
**FIGURE 2** General characteristics of outcomes divided by mTICI grade

**Figure:**
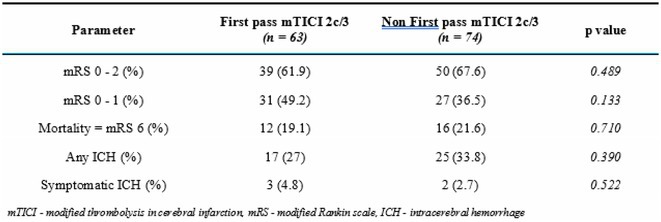
**FIGURE 3** General characteristics of outcomes divided according to presence of FPE.

**Conclusion:** We conclude, that MT is a powerful and effective treatment method for AIS caused by an occlusion of M2 segment in real life conditions. Patients have higher probability of long term good functional outcome in case that complete or near complete reperfusion is achieved.

**Disclosure:** Nothing to disclose.

Education in Neurology

## EPO‐391

### Teaching neurological emergencies through ward‐based simulation

#### A. Gupta ^1^; M. Elriedy^2^; S. Kilkie^3^

##### ^1^Department of General Internal Medicine, Epsom and St. Helier NHS Hospital NHS Trust, London, UK; ^2^Anaesthetic Department, School of Medicine, University of Nottingham, Nottingham, UK; ^3^University Hospitals of Derby and Burton NHS Trust, UK

**Background and Aims:** High‐fidelity simulation replicates medical scenarios for students to apply their skills safely. Many medical students experience ‘neurophobia’ when learning about neurological conditions. This ward‐based simulation programme aimed to evaluate confidence in managing neurological emergencies before and after working through a novel simulation programme.

**Methods:** Scenarios on epilepsy, spinal cord compression and meningitis were designed to run parallel on a simulated ward. Third‐year medical students were recruited to act as the patient. The scenarios were completed by final‐year medical students working at foundation year 1 doctor level. We collected a pre‐simulation data with students rating their confidence in neurological assessment and management on a Likert scale (1–5) and repeated this post‐simulation before analysis.

**Results:** In the first round of simulation, 16 students took part. There was a significant difference in reported confidence in recognising acute neurological deterioration and deficit prior to the simulation (M = 2.625, SD = 0.93) after the simulation (M = 3.875, SD = 0.70); *t*(15) = −5.84, *p* = 0.00003. All students agreed or strongly agreed that simulation is a good way to learn. There was a significant improvement in confidence when managing neurological emergencies before the simulation (M = 2.375, SD = 0.72) and after the simulation (M = 3.8125, SD = 0.66); *t*(15) = −6.45, *p* = 0.00001.

**Conclusion:** We found improved self‐rated confidence in neurological assessment and management. This exercise encourages students to apply clinical judgement. Our results are limited by the small sample size therefore, this simulation programme is ongoing, and further data is being collected. Multi‐centre data is also required to assess the wider impact of ward‐based simulation.

**Disclosure:** Nothing to disclose.

## EPO‐392

### Neurophobia and the perception of neurology among neurologists in Spain

#### I. Saldaña‐Inda^1^; Á. Lambea‐Gil ^2^

##### ^1^Hospital Universitari Arnau de Vilanova, Lleida, Spain; ^2^Hospital de la Santa Creu i Sant Pau, Barcelona, Spain

**Background and Aims:** Neurophobia has been well documented in medical students, residents and other specialties, but it has not been explored among neurologists. We aimed to study the awareness neurologists have of neurophobia, as well as our knowledge, fears and insecurities towards our specialty.

**Methods:** An online questionnaire was distributed through scientific regional societies of neurology. Respondents were questioned about neurophobia and its causes. They were also interrogated about the training received during residency and their perceived current knowledge and insecurities regarding the different subspecialties.

**Results:** 284 neurologists answered the survey. 60% were familiar with neurophobia. 249 (90%) thought neurophobia was prevalent among other specialties, 244 (88%) in residents, while only 151 (55%) identified neurophobia in medical students. Main reasons for neurophobia were the intrinsic difficulty of neurosciences (211, 74%) and the neurological examination (168%, 59%). Neurogenetics, palliative and sleep‐wake neurology were the weakest areas of training during the residency (85%, 84% and 77% respectively). However, neurologists felt the most insecure (Figure 1) in neurogenetics (142, 56%), ataxias (118, 47%), neuro‐oncology and neuromuscular diseases and (112, 44%). Reasons adduced were lack of exposure (177, 62%), training gaps during residency (137, 48%) or exclusive dedication to other pathologies (131, 46%).

**Figure:**
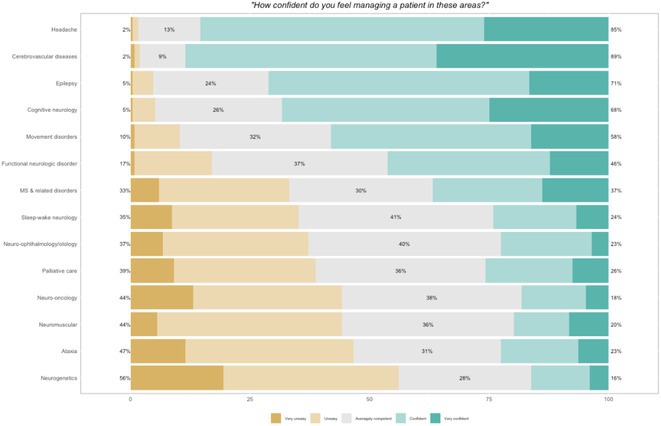
**FIGURE 1** Neurologists' confidence levels across different areas.

**Conclusion:** Neurophobia is not unknown to Spanish neurologists. We also identified a gap in residency training for palliative care, sleep‐wake neurology and genetics. However, it was neuromuscular diseases and ataxias where they felt the most insecure. One could argue “partial neurophobia” may exist among neurologists, caused by the lack of exposition more than training or theoretical knowledge.

**Disclosure:** Nothing to disclose.

## EPO‐393

### Exploring gender disparities in neurology education: Insights from Spanish medical schools

#### Á. Lambea‐Gil ^1^; S. Gil‐Navarro^2^; M. Jiménez^3^; P. Martínez‐Sánchez^4^; P. Mir^5^; C. Oreja‐Guevara^6^; J. Pascual‐Gómez^7^; Á. Pérez‐Sempere^8^; S. Santos‐Lasaosa^9^; A. Frank‐García^10^; E. Díez‐Tejedor^11^

##### ^1^Hospital de la Santa Creu i Sant Pau, Barcelona, Spain; ^2^Sociedad Española de Neurología, Barcelona, Spain; ^3^Hospital Quirónsalud Sagrado Corazón, Sevilla, Spain; ^4^Hospital Universitario Torrecárdenas, Almeria, Spain; ^5^Hospital Universitario Virgen del Rocío, Sevilla, Spain; ^6^Hospital Clínico San Carlos, Madrid, Spain; ^7^Hospital Universitario Marqués de Valdecilla, Santander, Spain; ^8^Hospital General Universitario Doctor Balmís, Alicante, Spain; ^9^Hospital Clínico Universitario Lozano Blesa, Zaragoza, Spain; ^10^Hospital Universitario La Paz, Madrid, Spain; ^11^Universidad Autónoma de Madrid, Madrid, Spain

**Background and Aims:** Gender disparities in university faculty roles is a concern across European higher education, with women often underrepresented in senior positions. Women comprise 54% of the Spanish Society of Neurology (SEN) membership, yet their representation within university medical programs remains underexplored. This study aims to analyze neurologists lecturing Neurology in Spanish medical schools, focusing on gender distribution, academic rank, and career progression.

**Methods:** From July to October 2024, a self‐administered survey was distributed to SEN members and medical schools. Data collected included demographics, teaching experience and academic roles. Bivariable and inferential analyses were conducted to assess relationships and statistical significance.

**Results:** Responses were received from 39 of 43 eligible medical schools. Among 217 respondents, 135 were actively lecturing Neurology, including 49 women (36%). The mean age was 53 ± 10 years, with women being younger than men (51 ± 8 vs. 55 ± 11, *p* = 0.047). Women had fewer years of teaching experience than men (10 ± 7 vs. 15 ± 11, *p* = 0.004). Gender distribution across academic ranks varied but did not reach statistical significance (*p* = 0.112): women held 25% of Professorships (1/4), 22% of Senior Lecturer roles (4/18), 43% of Lecturer roles (3/7), and 41% of Associate Lecturer or collaborator positions (41/101). None of the three Emeritus Professors were women.

**Conclusion:** Gender disparities are present in academic Neurology in Spain, with women underrepresented despite their substantial presence in the SEN. Although women hold fewer senior academic roles, these disparities did not reach statistical significance compared to their colleagues. Insight from other European countries would be of interest.

**Disclosure:** The authors are members of the SEN Council on “Neurology and University”.

## EPO‐394

### Immersive virtual reality education for electroencephalography interpretation

#### A. Creed^1^; D. Murphy^2^; C. McCafferty^3^; J. Chan^4^; S. Gomez Quintana^1^; A. Temko^1^; E. Popovici^1^; A. Factor ^3^

##### ^1^Department of Electrical and Electronic Engineering University College Cork, Cork, Ireland; ^2^Department of Computer Science, University College Cork, Cork, Ireland; ^3^Anatomy and Neuroscience Department, University College Cork, Cork, Ireland; ^4^Department of Applied Psychology, University College Cork, Cork, Ireland

**Background and Aims:** Electroencephalography (EEG) is an essential tool in neurology and neuroscience. Its interpretation is time‐consuming and requires specialised healthcare professionals for a medical diagnosis. This study introduces a novel Virtual Reality (VR)‐based EEG analysis platform that integrates visual and auditory modalities, and artificial intelligence (AI) to detect neonatal seizures.

**Methods:** An openly available neonatal EEG dataset from the University of Helsinki was used. An EEG analysis application on a VR headset was developed and evaluated. The developed VR environment integrates data and information streams to represent and analyse EEG data, comprised of 8‐channels, Fourier transforms, AI‐assisted sonification, and an AI‐informed 3D visualisation of the brain's seizure activity to detect seizures.

**Results:** A series of user studies were conducted, evaluating the system's usability, functionality, and overall user experience. User evaluations (20 participants) indicate high satisfaction and reduced cognitive load. Participants performed best when using both sound and visualisation (Combined mean = 7.60), followed by the visualisation alone (Visualisation mean = 6.10) and sound alone (Sound mean = 5.50). The comparative analysis between the two groups (with prior knowledge and no prior knowledge of EEG) shows a slight variation in performance across the three modalities.

**Conclusion:** This work introduces a VR‐based platform to facilitate EEG analysis for seizure detection using a multisensory representation, including 2D and AI‐assisted 3D visualisation and AI‐assisted sonification and analysis of EEG data. The initial results recommend the tool for applications in educational and clinical settings that require EEG analysis.

**Disclosure:** Qualcomm sponsored this work through a philanthropic gift UNI‐479522. All the authors don't have any conflict of interest to report.

## EPO‐395

### Focal epilepsy: Virtual patient simulation improves performance in diagnosis and management but uncovers inertia

#### L. Thevathasan^1^; C. Rohani‐Montez ^1^; K. Carpenter^1^; M. D'Amico^1^; S. Gupta^1^; J. Berrios^1^; C. Scot‐Smith^1^; B. Schmitz^2^

##### ^1^Medscape LLC, London, UK; ^2^Vivantes Humboldt‐Klinikum, Berlin, Germany

**Background and Aims:** Diagnosing focal epilepsy is challenging due to heterogeneous presentation, limited patient awareness and restricted access to diagnostic tools. Further, management is complicated by diverse aetiologies and numerous potential therapies. Using a patient simulation of a young female with focal seizures and co‐morbid anxiety and depression with partial control on 2 prior monotherapies, we assessed neurologists’ performance in diagnosing drug‐resistant focal epilepsy and managing nocturnal breakthrough seizures with appropriate combination therapy.

**Methods:** This CPD‐certified virtual simulation allowed European neurologists to select diagnostic and treatment options from a comprehensive database (available at: https://www.medscape.org/viewarticle/998607). After each decision, learners received clinical guidance (CG) based on evidence and faculty recommendations. Pre‐ and post‐CG decisions were analyzed using McNemar's test (*p* < 0.05 is significant). Data were gathered from March to December 2024.

**Results:** Ninety neurologists participated in the case simulation. Significant improvements were seen post‐guidance (CG) for identifying drug‐resistant epilepsy (21% to 65%, *p* < 0.001), managing epilepsy (36% to 69%, *p* < 0.001), and evaluating comorbid anxiety/depression (67% to 75%, *p* < 0.01). Analysing decision flows with a less‐stringent diagnostic definition, correct diagnosis and treatment rose from 24% to 29%, while incorrect decisions dropped from 46% to 36%. Among those choosing incorrect treatments, 87% showed inertia pre‐CG (no treatment change), decreasing to 74% post‐CG.

**Conclusion:** This study demonstrates the positive effect of online medical education through a virtual simulation on European neurologists’ performance in diagnosing and managing drug‐resistant focal epilepsy, but in‐depth analyses uncovered high levels of inertia in managing drug‐resistant epilepsy.

**Disclosure:** Developed through independent educational funding from Angelini Pharma.

## EPO‐396

### Virtual patient simulation improves diagnosis and management but uncovers low drug‐resistant epilepsy diagnosis rates

#### L. Thevathasan^1^; C. Rohani‐Montez ^1^; K. Carpenter^1^; M. D'Amico^1^; S. Gupta^1^; J. Berrios^1^; C. Scot‐Smith^1^; B. Schmitz^2^

##### ^1^Medscape LLC, London, UK; ^2^Vivantes Humboldt‐Klinikum, Berlin, Germany

**Background and Aims:** Diagnosing focal epilepsy is challenging due to heterogeneous presentation, limited patient awareness and restricted access to diagnostic tools. Further, management is complicated by diverse aetiologies and numerous potential therapies. Using a patient simulation of a 62‐year old male with post‐encephalitic focal seizures with poor control despite 3 different therapies, we assessed neurologists’ performance in diagnosing drug‐resistant focal epilepsy and managing frequent seizures with appropriate combination therapy.

**Methods:** This CPD‐certified virtual simulation allowed European neurologists to select diagnostic and treatment options from a comprehensive database (available at: https://www.medscape.org/viewarticle/998607). After each decision, learners received clinical guidance (CG) based on evidence and faculty recommendations. Pre‐ and post‐CG decisions were analyzed using McNemar's test (*p* < 0.05 is significant). Data were gathered from March to December 2024.

**Results:** 106 neurologists participated in the case simulation. Significant improvements were seen post‐guidance (CG) for identifying drug‐resistant epilepsy (16% to 56%, *p* < 0.001) and managing epilepsy (14% to 29%, *p* < 0.001). Analysing decision flows with a less‐stringent diagnostic definition, correct diagnosis and treatment rose from 0.9% to 13%, while incorrect decisions dropped from 93% to 60%. Among those with incorrect treatment, 61% showed inertia pre‐CG (no treatment change), decreasing to 46% post‐CG.

**Conclusion:** This study demonstrates the positive effect of online medical education through a virtual simulation on European neurologists’ performance in diagnosing and managing drug‐resistant focal epilepsy, but more in‐depth analyses uncover low levels of diagnosis and management of drug‐resistant epilepsy.

**Disclosure:** Developed through independent educational funding from Angelini Pharma.

## EPO‐397

### How does the publication of recommendations affect clinical practice? A comparative analysis of patient journeys

#### C. Festari^1^; C. Singh Solorzano^1^; S. Orini^2^; E. Castagna^3^; M. Cotta Ramusino^4^; F. D'antonio^3^; A. Di Crosta^5^; A. Masòtino^5^; F. Massa ^6^; A. Mazzonetto^7^; M. Panigutti^3^; N. Ravì^7^; M. Pievani^1^; L. Bonanni^5^; G. Bruno^7^; A. Cagnin^7^; R. Gatta^8^; G. Frisoni^9^

##### ^1^Laboratory of Alzheimer's Neuroimaging and Epidemiology, IRCCS Istituto Centro San Giovanni di Dio Fatebenefratelli, Brescia, Italy; ^2^Alzheimer's Unit, Memory Clinic, IRCCS Istituto Centro San Giovanni di Dio Fatebenefratelli, Brescia, Italy; ^3^Department of Human Neurosciences, Sapienza University of Rome, Rome, Italy; ^4^Unit of Behavioral Neurology and Dementia Research Center (DRC), IRCCS Mondino Foundation, Pavia, Italy; ^5^Department of Medicine and Aging Sciences, University G. d'Annunzio, Chieti‐Pescara, Italy; ^6^Department of Neuroscience, Rehabilitation, Ophthalmology, Genetics, Maternal and Child Health (DINOGMI), University of Genoa & IRCCS Ospedale Policlinico San Martino, Genoa, Italy; ^7^Neurology Unit, Department of Neuroscience, University of Padova, Padua, Italy; ^8^Department of Clinical and Experimental Sciences, Università degli Studi di Brescia, Brescia, Italy; ^9^University Hospitals of Geneva, Memory Centre, Division of Geriatrics and Rehabilitation & University of Geneva, Laboratory of Neuroimaging of Aging (LANVIE), Geneva, Switzerland

**Background and Aims:** Quantitative evaluation of the impact of recommendations publication on clinical practice has been poorly investigated in research. This study analysed the systematic changes in the clinical diagnostic process attributable to the publication of the Italian Intersocietal recommendations for biomarker‐based diagnosis of neurocognitive disorders (Boccardi, 2000).

**Methods:** Medical charts of new patients from three Italian memory clinics were reviewed for 2019 (pre‐recommendations, P1) and 2023 (post‐recommendations, P2). Sociodemographic and clinical data were extracted, and adherence to the recommendations was assessed using a modified Adherence Index (AI). The AI score, ranging from 0 to 5, evaluates diagnostic work‐up completeness, with higher scores indicating better adherence. Statistical analyses (Mann‐Whitney U and chi‐square tests) compared AI scores across periods.

**Results:** A total of 601 diagnostic work‐ups from P1 and 434 from P2 were reviewed. Adherence was computed only in diagnostic work‐up including biomarkers, i.e., 139 cases (23%) in P1 and 178 cases (41%) in P2. Over time, the IA score increased from 2.09 ± 1.04 to 2.28 ± 1.04 (*U* = 81; *p* = 0.047). P2 work‐ups featured a more thorough neuropsychological assessment (AI: 0.40 ± 0.36 in P1 vs. 0.48 ± 0.43 in P2, *U* = 9492; *p* < 0.001) and more adherent use of biomarkers prescription (X2(3) = 39.66; *p* < 0.001).

**Conclusion:** Adherence analysis highlighted significant changes in clinical diagnostic work‐ups following the recommendations release. AI is a straightforward‐to‐implement measure that provides quantitative indications of the impact of recommendations in actual settings. Process mining will allow for a more in‐depth analysis.

**Disclosure:** We acknowledge unrestricted grants from GE HealthCare LTD and Roche Diagnostics S.p.A. The funders had no role in the conception, design, and implementation of the project nor in data collection, data analysis, interpretation, or discussion of the results. Funders had no privileged access to the project's outputs at any stage.

## EPO‐398

### Inventory of the knowledge and training needs of professionals in EHPAD for Parkinson's disease

#### J. Thezenas‐Montauban ^1^; M. Heuzé‐De Raeve^2^

##### ^1^Groupement Hospitalier Eaubonne‐Montmorency, Eaubonne, France; ^2^Infirmière de Coordination, France

**Background and Aims:** Parkinson's disease is a major public health issue, due to its rapidly increasing incidence, which is associated with high levels of dependency and institutionalization. It requires personalized care. The aim of this study is to assess the initial knowledge of professionals working in residential care facilities for the elderly, in order to provide teams with knowledge to adapt to the profiles of their Parkinsonian patients.

**Methods:** A quantitative study was carried out using a nationally distributed questionnaire. 430 questionnaires were completed by paramedical staff, 92% of them by state‐qualified nurses and care assistants. 5 qualitative interviews were conducted with professionals to gain a deeper understanding of their perception of working and training conditions in residential care facilities.

**Results:** 71% of caregivers felt they knew the pathology. However, trembling (42%) is considered a diagnostic criterion. On the other hand, pain (12%), anxiety (10%) and depression (10%) are little recognized. The role of symptomatic treatments is accepted by 96% of caregivers. Little is known about their non‐oral administration. A desire for training (92%) is expressed on pathology (23%) and support (25%). Qualitative interviews underline the central role played by orderlies in dispensing medication, as well as the importance of their knowledge.

**Conclusion:** EHPAD professionals have an incomplete perception of the pathology. They express a need for training in how to support these patients. In the light of these results, it is essential to develop targeted actions to encourage appropriate care for Parkinson's patients.

**Disclosure:** The authors would like to thank Tilio Cognard, head of training at France Parkinson, for distributing and passing on the questionnaire feedback.

## EPO‐399

### The role of knowledge and information sources in empowering patients with multiple sclerosis

#### J. Cegarra Sánchez; A. López Santana; J. García Granado; A. Relloso de la Fuente; J. Rodríguez Santana; M. Pérez Vieitez; A. González Hernández

##### Neurology Department, Hospital Doctor Negrín, Las Palmas de Gran Canaria, Spain

**Background and Aims:** Empowering patients with multiple sclerosis (MS) is crucial for enhancing their management and quality of life. This study investigates how patient empowerment is reflected in a balance between different types of knowledge (emotional, rational, and spiritual). Additionally, it assesses the impact of verified and unverified information sources on this empowerment.

**Methods:** A sample of 75 MS patients was consecutively selected, and questionnaires designed to evaluate emotional, rational, and spiritual knowledge were administered. The influence of verified and unverified information sources on patients' decisions was also examined. Data were analysed using the SPSS 4.1 programme through multivariate regression and correlation analysis.

**Results:** Emotional knowledge demonstrated the greatest effect on patient empowerment (β = 0.86, *p* < 0.000), followed by rational (β = 0.77, *p* < 0.000) and spiritual (β = 0.73, *p* < 0.000). However, the balance between the three types was essential for achieving optimal levels of empowerment. Regarding information sources, verified ones had a greater impact (β = 0.31, *p* < 0.002) compared to unverified ones (β = 0.21, *p* < 0.023), although both were significant.

**Figure:**
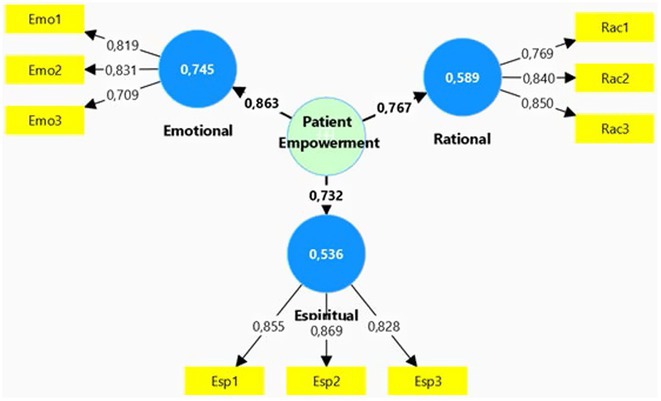
**FIGURE 1** Patient empowerment and kinds of knowledge

**Figure:**
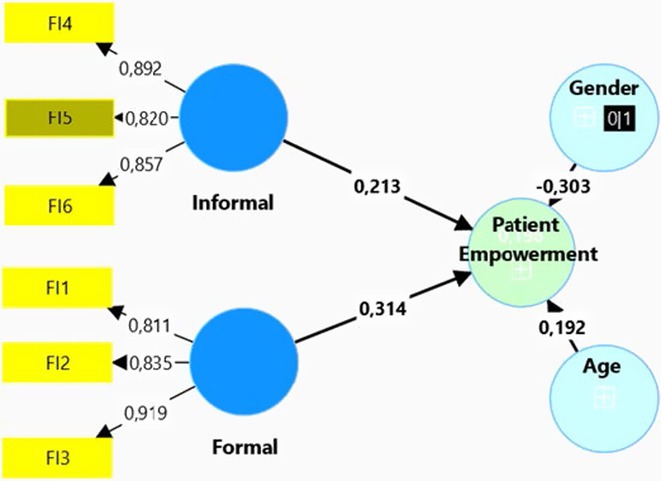
**FIGURE 2** Patient empowerment and sources of information (1)

**Figure:**
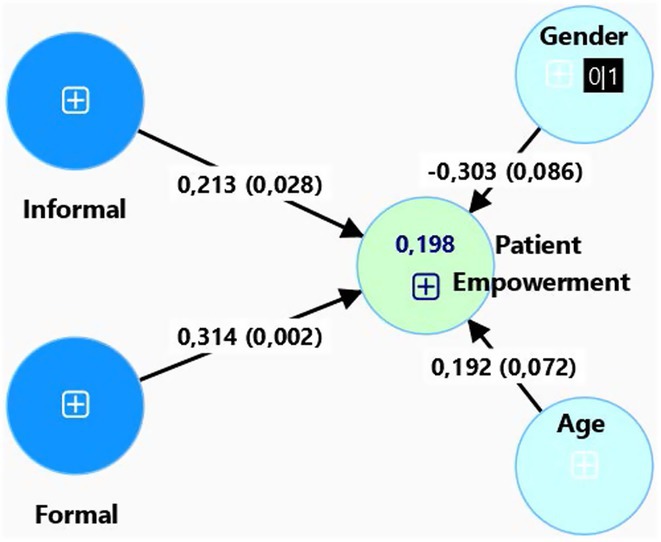
**FIGURE 3** Patient empowerment and sources of information (2)

**Conclusion:** Empowering MS patients requires a balance between emotional, rational, and spiritual knowledge, with emotional knowledge being the most influential, regardless of sex or age. We propose the development of an empowerment measurement scale and a filtering system to improve the quality of unverified information sources.

**Disclosure:** Nothing to disclose.

## EPO‐400

### Evaluation of Chat GPT's performance on the pediatric neurology specialty certificate examinations

#### K. Dzwilewski; M. Pietruszka; P. Rumiński; M. Zawadzka; M. Mazurkiewicz‐Bełdzińska

##### Department of Developmental Neurology, Gdańsk, Poland

**Background and Aims:** Artificial Intelligence (AI) is being utilized in many aspects of human life, including medicine. Our work focuses on analyzing the effectiveness of AI‐based language models in the context of solving the polish State Specialization Examination (SSE) in pediatric neurology.

**Methods:** The study evaluated the effectiveness of 2 language models: Chat GPT 3.5 and 4.0 in solving two past papers of SSE in pediatric neurology. For the study, questions were divided into 6 thematic groups. The point scores of both models were compared with the results of physicians taking the SSE in the given sessions and the difficulty index of each question.

**Results:** Chat GPT 4.0 achieved a passing score (60%) in both examination sessions. Considering the total points obtained in both examination sessions, Chat GPT 4.0 achieved similar scores (72%) to physicians (74%). The newer versions of Chat GPT outperformed (72%) its predecessor (48%). Chat GPT 4.0 performed best in the questions connected with metabolic disorders, headaches, CNS tumors, while doctors achieved the highest scores in all other categories.

**Figure:**
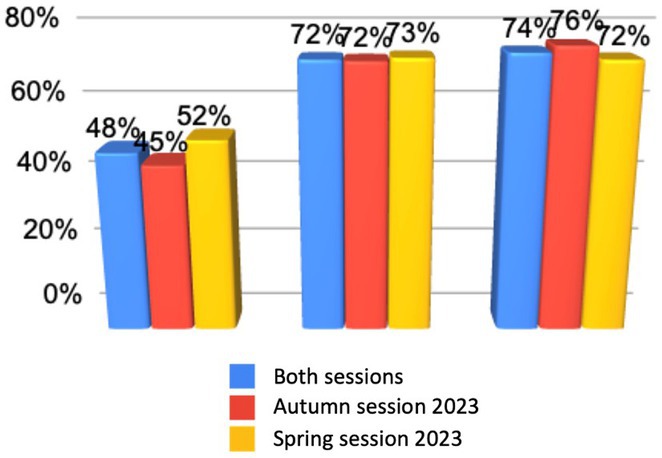
**FIGURE 1** Results of the SSE examinations in developmental neurology in the spring 2023 and fall 2023 sessions.

**Conclusion:** Chat GPT 4.0 outperformed its predecessor, probably due to significant enhancements, such as more advanced contextual understanding, greater language fluency, and a much larger base of learned information. Variations in the Chat GPT's performance in different categories may be a result of inadequate modeling by the engineers and the differences in availability of specialty‐specific materials in the training database. Nevertheless, the results presented in our work may indicate the potential utilization of artificial intelligence in the education and practice of pediatric neurologists.

**Disclosure:** Nothing to disclose.

## EPO‐401

### Educational potential of the HealUA mobile app for peer‐to‐peer neurology consultations during wartime in Ukraine

#### K. Potapova ^1^; M. Horiachok^2^; T. Ivanykovych^3^; M. Antoniv^4^; A. Nykonenko^5^; D. Khoptar^5^; O. Tytarenko^6^; I. Vereshchak^7^; S. Bielichenko^8^; B. Mamediiev^9^; A. Dzhemiliev^10^; S. Yaniuta^9^; N. Melnitchouk^4^

##### ^1^Department of Neurology, Bogomolets National Medical University, Kyiv, Ukraine; Global Medical Knowledge Alliance; Boston, USA; ^2^Bukovinian State Medical University, Chernivtsi, Ukraine; Global Medical Knowledge Alliance; Boston, USA; ^3^Danylo Halytsky Lviv National Medical University, Lviv, Ukraine; Global Medical Knowledge Alliance; Boston, USA; ^4^Department of Surgery, Division of Colorectal Surgery, Brigham and Women's Hospital, Harvard Medical School, Boston, USA; Global Medical Knowledge Alliance; Boston, USA; ^5^Bogomolets National Medical University, Kyiv, Ukraine; Global Medical Knowledge Alliance; Boston, USA; ^6^Vinnytsia National Pirogov Memorial Medical University, Vinnytsia, Ukraine; ^7^Department of Neurology, Center for Innovative Medical Technologies of the National Academy of Sciences of Ukraine, Kyiv, Ukraine; Global Medical Knowledge Alliance; Boston, USA; ^8^Division of Cardiac Surgery, Massachusetts General Hospital, Boston, USA; Global Medical Knowledge Alliance; Boston, USA; ^9^Global Medical Knowledge Alliance; Boston, USA; ^10^Department of Surgery, Brigham and Women's Hospital, Harvard Medical School, Boston, USA; Global Medical Knowledge Alliance; Boston, USA

**Background and Aims:** The Russian invasion of Ukraine has significantly disrupted healthcare, including specialized fields such as neurology. To address healthcare delivery, HealUA, a mobile app developed with Ukrainian physicians, enables verified doctors to engage in remote, peer‐to‐peer consultations, fostering both education and clinical support.

**Methods:** HealUA was created by the Global Medical Knowledge Alliance and Empat. The software enables verified doctors to submit clinical cases, obtain expert advice, and share their knowledge for education. The free app was distributed via app markets as well as marketed through physician networks, social media, and medical associations. Data from the app were evaluated using descriptive statistics.

**Figure:**
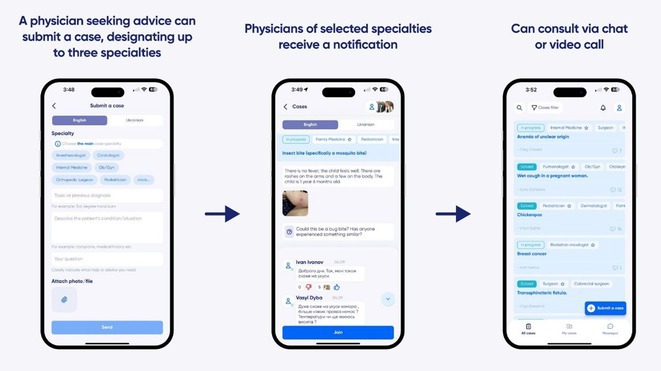
**FIGURE 1** How does HealUA work?

**Results:** Between May 2022 and January 2025, 4282 physicians joined HealUA, included 327 neurologists and 123 neurosurgeons 95% (*n* = 4038) came from Ukraine, 2% (*n* = 91) from the United States, and 3% (*n* = 113) from 33 different countries. Since May 2022, 577 requests have been sent using the app, with 55 categorized as “neurology” and 13 as “neurosurgery,” all of them have gotten answers. International specialists consulted on 24 (43.6%) of the neurological cases presented.

**Conclusion:** This study demonstrates the potential of the HealUA mobile app as a valuable tool for peer‐to‐peer neurology consultations and education during the war in Ukraine. The platform offers a significant wealth of clinical cases, including neurology and neurosurgery, facilitating international knowledge exchange and highlighting case‐based learning experiences. This innovative approach not only educates specialists in Ukraine, but also offers a model that can be repurposed in other countries facing crisis situations.

**Disclosure:** Nothing to disclose.

## EPO‐402

### Advancing cannabinoid delivery through nanotechnology and its implications for neurology

#### L. Mechtler ^1^; R. Ezra^2^; C. Ralyea^1^

##### ^1^Dent Neurologic Institute, Buffalo, USA; ^2^Capsoil Technologies, New York, USA

**Background and Aims:** In the evolving landscape of cannabinoid‐based medicine, neurologists must develop an understanding of cannabinoids to effectively incorporate them into treatment plans. Delivery method selection is critical for optimizing pharmacokinetic (PK) profiles, therapeutic efficacy, and patient outcomes. Cannabinoid therapies are available in oral, sublingual, inhalation, transdermal, and injectable modalities, each with unique strengths and limitations. Self‐NanoEmulsifying Drug Delivery Systems (SNEDDS) and nanoemulsion technologies offer promising solutions to overcome poor solubility and bioavailability challenges of lipophilic cannabinoids. This abstract highlights PK and permeation data from two studies: one evaluating an oral SNEDDS cannabis product versus an oil‐based tincture and another examining cannabidiol (CBD) dermal delivery using Capsoil Technology.

**Methods:** The first study was an open‐label crossover trial in nine subjects administered an oral SNEDDS formulation (8 mg THC, 8 mg CBD) or an oil‐based tincture with a 30‐day washout. The second study used an in vitro Franz cell diffusion model with human skin to evaluate CBD absorption of a Capsoil Nano‐emulsion versus a conventional CBD oil (10 mg/g CBD).

**Results:** The SNEDDS formulation demonstrated significantly higher Cmax values for THC (47.05 ng/mL vs. 12.66 ng/mL), CBD (10.62 ng/mL vs. 4.02 ng/mL), and their metabolites compared to the tincture. Bioavailability improvements ranged from 278.8% to 495.6%, supporting faster onset and prolonged therapeutic effects. The Capsoil Nano‐emulsion achieved a four‐fold increase in skin permeation and enhanced penetration across all layers, suggesting potential for effective transdermal applications.

**Conclusion:** These findings may support the use of SNEDDS‐based cannabinoid products in headache medicine and inform neurologists in developing effective treatment plans.

**Disclosure:** Laszlo Mechtler, MD, has served on advisory boards for, consulted for, and/or been a speaker for AbbVie, Allergen (now AbbVie), Amgen, Biohaven (now Pfizer), Currax Pharmaceuticals, Electrocore, Impel Pharmaceuticals, H.S. Lundbeck, Novartis, Promius Pharma, Teva, Theranica Bio‐electrics, Tonix Pharmaceuticals. Dr. Mechtler serves on the Board of Directors for the International Headache Society and Genomate Health and serves as an advisory board member for NeurodiscoveryAI, Craniometrix, and the New York State Athletic Commission. The institution of Dr. Mechtler has received research and/or educational support from Abbvie, Aeon BioPharma, Alder, Allergan (now AbbVie), American Migraine Foundation, Amgen, Alpheus Medical, Biohaven (now Pfizer), Boston Biomedical, Charlotte's Web, Currax Pharmaceuticals, Delmar Pharmaceuticals (now Kintara Therapeutics), Eli Lilly, H.S. Lundbeck, Miles for Migraine, Novartis, Orbis Pharma, Shiratronics, Teva, The Harry Dent Family Foundation, Inc, and Theranica.

## EPO‐403

### Uncovering knowledge and practice gaps in focal epilepsy amongst European neurologists

#### L. Thevathasan ^1^; C. Rohani‐Montez^1^; K. Carpenter^1^; C. Scot‐Smith^1^; S. Lattanzi^2^

##### ^1^Medscape LLC, London, UK; ^2^Marche Polytechnic University, Ancona, Italy

**Background and Aims:** Focal epilepsy poses diagnostic and therapeutic challenges due to varied clinical presentations, limited standardization, and evolving treatment paradigms. To identify and assess patterns relating to attitudes, knowledge and practice gaps, we conducted an educational survey among European neurologists.

**Methods:** A 27‐question online CME survey assessed knowledge (disease burden, predictors, clinical data) and case‐based competence. Conducted from April to December 2024, responses were de‐identified and aggregated. Two questions mirrored a 2021 Medscape survey, allowing result comparisons.

**Results:** A total of 135 neurologists completed the survey. A difference was observed between performance on knowledge‐based and competence‐based questions, with better results on case‐based clinical practice (Table 1). Comparing 2021 to 2024, similar results were seen on the same questions (Table 2). For a case of uncontrolled focal epilepsy on one ASM, 44% showed flexibility in choosing between two appropriate strategies, while 56% showed a definite preference: 39% for add‐on therapy and 17% for switching. Regarding 3rd generation ASMs, 61% opted for use after failure of 2 therapies, 13% after 3, and 16% based decisions on the specific ASM. Physicians demonstrated poor understanding of SUDEP risk factors and clinical trial data supporting current practice. However, management of focal epilepsy and comorbidities appeared stronger although half demonstrate inflexibility in their approach.

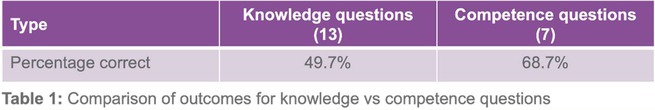

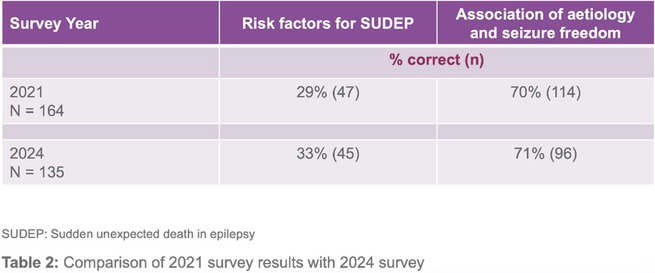

**Conclusion:** Whilst case‐based questions may have been easier or more intuitive to answer, this study uncovered persistent and significant gaps in knowledge and clinical practice. These findings highlight an urgent and targeted need for sustained knowledge‐based education to bridge critical gaps and enhance decision‐making.

**Disclosure:** Developed through independent educational funding from Angelini Pharma.

## EPO‐404

### Promoting brain health and awareness of brain‐related disorders among young people in Cameroon

#### M. Njohjam ^1^; E. Nongse^2^; S. Nji^3^

##### ^1^Department of Neurology, National University Hospital Center, Dakar, Senegal; ^2^Epilepsy Awareness, Aid and Research Foundation, Bamenda, Cameroon; ^3^University of Bamenda, Bamenda, Cameroon

**Background and Aims:** The burden of brain‐related disorders in Cameroon has been growing steadily, and brain health has been deteriorating among young people, mostly driven by increased substance abuse and a low level of public awareness of the importance of brain health. The objective of our project was to promote brain health and awareness of brain‐related disorders so as to destigmatise brain‐related disorders, leading to early detection and treatment.

**Methods:** We designed and implemented a peer‐led awareness‐raising initiative involving 25 young peer educators with healthcare and non‐healthcare backgrounds who were trained and empowered with knowledge on common neurological disorders and brain health. These trained peer educators subsequently organise awareness‐raising activities and workshops in schools and other public places using brain models and posters.

**Results:** Over a period of 24 months, we have reached over 15,000 young people in both rural and urban communities, engaging over 200 school authorities on brain health and establishing strong partnerships with local stakeholders. There has been an increase in the level of awareness of brain health and brain‐related disorders with growing interest in neurology and neuroscience among students and increased engagement from school authorities.

**Conclusion:** Youth‐led initiatives are important and feasible strategies to improve awareness of brain health and brain‐related disorders among young people in resource‐limited settings.

**Disclosure:** Nothing to disclose.

## Headache and pain

## EPO‐405

### Clinical profile of migraine in South Asian countries: A systematic review

#### A. Chapagain ^1^; A. Mishra^1^; R. Ojha^2^

##### ^1^Tribhuvan University Teaching Hospital, Kathmandu, Nepal; ^2^Department of Neurology, Tribhuvan University Teaching Hospital, Kathmandu, Nepal

**Background and Aims:** Migraine, a condition causing moderate to severe headaches, ranks as the second most burdensome neurological disorder among Asians regarding disability‐adjusted life years (DALYs). The paucity of knowledge from South Asia regarding migraine profiles made the commencement of this study imperative.

**Methods:** We conducted a systematic review per PRISMA guidelines. We screened English articles on descriptive studies done in the South Asian population with diagnosed adult migraine patients. The final review contained 36 articles.

**Figure:**
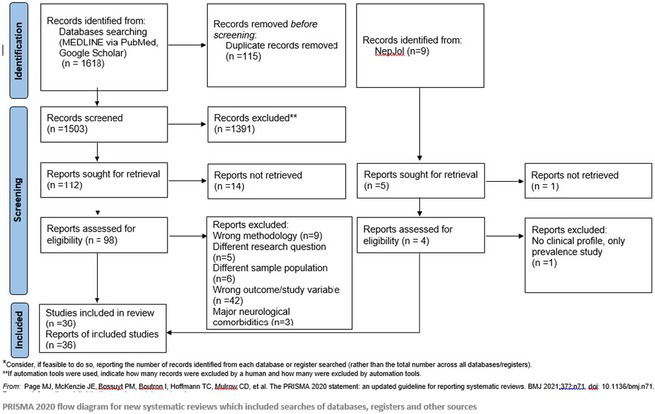
**FIGURE** PRISMA 2020 flow diagram of included studies.

**Results:** Migraineurs are mostly in their 30s, urban dwellers, with high female preponderance and family history. The commonest triggers were lack of sleep, stress, missed meals, specific smells/foods, and menstruation. Unilateral pulsatile headaches in the morning, and usually resolving within a day were found to be the most common occurrences. Migraine without aura was more commonly reported, with visual aura being the most prevalent. The most frequently associated features were photophobia, phonophobia, nausea, vomiting, vertigo, dizziness, neck pain, and vision problems. Most studies reported that medications like NSAIDs, acetaminophen, triptans, opioids, or combinations of these aid in relieving symptoms. While adequate sleep/rest and staying in a quiet room were commonly accepted non‐pharmacological relievers, few studies revealed patients found solace after vomiting, food intake, and a change in posture.

**Figure:**
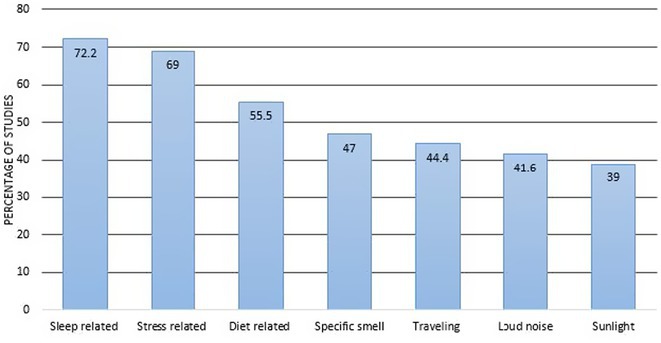
**FIGURE** Bar graph of common trigger factors of migraine.

**Conclusion:** As young females and urban dwellers are the common demographics, it highlights the role of genetics and environment in the etiology of migraine. Behavioral elements like stress, lack of sleep, fatigue, and diet habits being common triggers underscore the importance of identifying and preventing acute attacks with multispecialty care.

**Disclosure:** Nothing to disclose.

## EPO‐406

### The correlation between vitamin B12 serum levels and migraine: A case‐control study

#### A. Ahmed ^1^; S. Abdelsadek^3^; S. Tahoun^4^; F. Mansour^2^

##### ^1^Department of Neurology, The Royal Wolverhampton NHS Trust, Wolverhampton, UK; ^2^Department of Neurology, Faculty of Medicine for Boys, Al‐Azhar University, Cairo, Egypt; ^3^Department of Neurology, Faculty of Medicine for Girls, Al‐Azhar University, Cairo, Egypt; ^4^Department of Clinical Pathology, Faculty of Medicine for Girls, Al‐Azhar University, Cairo, Egypt

**Background and Aims:** Migraine represents the prevailing form of primary headache with no fully described aetiology and pathophysiology. This study aimed to assess the association between vitamin B12 serum levels and both chronic and episodic migraine.

**Methods:** This study was conducted as a case‐control study, including 90 migraineurs, divided into 48 with episodic migraine and 42 with chronic migraine as the case group, and 90 matched healthy participants as the control group. The serum level of vitamin B12 was measured using enzyme‐linked immunosorbent assay (ELISA) for all subjects. Its association with the Migraine Disability Assessment (MIDAS) scale and migraine attack severity, measured using the Visual Analog Scale (VAS), was analyzed.

**Results:** Migraineurs exhibited a notable reduction in serum vitamin B12 levels compared to the control group (243.97 ± 124.85 pg/mL vs. 302.69 ± 143.69 pg/mL, *p* = 0.014). Furthermore, chronic migraine patients had significantly lower serum vitamin B12 levels when compared to episodic migraine patients (202.7 ± 75.62 pg/mL vs. 269.17 ± 143.31 pg/mL, *p* = 0.026). A significant negative correlation was found between serum vitamin B12 levels and the severity of migraine attacks, as measured by the VAS (*r* = −0.407, *p* = 0.036).

**Conclusion:** The current study highlighted that vitamin B12 deficiency is highly associated with migraine and its severity. Further interventional research is highly recommended to investigate the potential causality of this association.

**Disclosure:** Nothing to disclose.

## EPO‐407

### Non‐pharmacological approaches to migraine management: Assessing the efficacy of repetitive transcranial magnetic stimulation

#### A. Fernandes ^1^; A. Alves^2^; J. Borges^2^; P. Sousa Martins^2^; R. Moreia^2^; A. Costa^1^

##### ^1^Neurology Service, Local Health Unit of São João, Porto, Portugal; ^2^Psychiatry Service, Local Health Unit of São João, Porto, Portugal

**Background and Aims:** In recent years, the potential neuromodulatory effect of Repetitive transcranial magnetic stimulation (rTMS) has been investigated as a therapeutic tool in migraine. We aim to assess the impact of unilateral rTMS targeting the dorsolateral or dorsomedial prefrontal cortex, performed according to protocols for treatment‐resistant major depression (TRMD) or obsessive‐compulsive disorder (OCD) respectively, on migraine control.

**Methods:** Clinical characterization of a cohort of patients who underwent rTMS in the last 10 months for TRMD or OCD, with a concomitant diagnosis of migraine. We conducted a questionnaire regarding migraine frequency, pain intensity, use of rescue analgesia, before and after rTMS.

**Results:** We included 8 female patients, including 5 patients with frequent episodic migraine and 3 with chronic migraine The median time elapsed since the last session was of 2 months (IQR:1–9.25). Half of our sample perceived a positive/very positive overall change after rTMS regarding migraine, including 3 patients with TRMD and one patient with OCD. Four patients reported simultaneous reduction in migraine frequency and the need for rescue analgesia, of whom 3 also reported a decrease in maximum pain intensity. Prior to rTMS the mean days‐per‐week with migraine was 3, which was significantly reduced to 1.7 days‐per‐week (*p* = 0.03). Worsening of migraine was observed in one patient, in terms of frequency and intensity of pain. Transient peri‐procedural headache was associated with lack of efficacy of rTMS regarding migraine (*p* = 0.071).

**Conclusion:** This preliminary investigation suggests a potential sustained benefit of rTMS in reducing the number and the severity of attacks in migraine.

**Disclosure:** Nothing to disclose.

## EPO‐408

### The Nordic chronic migraine trial of CGRP monoclonal antibody and onabotulinumtoxin A dual therapy (NorMig)

#### B. Bezgal ^1^; K. Wesnes^2^; K. Müller^3^; Z. Gadan^4^; H. Flemmen^5^; A. Poole^6^; M. Aalstad‐Johansen^7^; M. Bjørk^8^; K. Jakobsen^9^; A. Roy^10^; C. Sundal^11^; K. Devik^12^; A. Dueland^13^; L. Hofsøy Steffensen^14^; S. Mathisen^15^; Å. Hagen Morsund^16^; L. Stovner^17^; M. Matharu^18^; M. Toft^19^; H. Winther Schytz^20^; M. Linde^21^; T. Wisløff^22^; D. Dodick^23^; E. Tronvik^24^; A. Aamodt^25^

##### ^1^Department of Neurology, Oslo University Hospital, Oslo, Norway and Institute of Clinical Medicine, Faculty of Medicine, University of Oslo, Oslo, Norway; ^2^Norwegian Centre for Headache Research (NorHead), The Norwegian University of Science and Technology, Trondheim, Norway and Department of Neurology and Clinical Neurophysiology, St. Olav University Hospital, Trondheim, Norway; ^3^Department of Neurology, Sørlandet Hospital Trust, Kristiansand, Norway; ^4^Department of Neurology, Østfold Hospital Trust, Sarpsborg, Norway; ^5^Department of Neurology, Telemark Hospital Trust, Skien, Norway; ^6^Oslo Headache Centre, Oslo, Norway; ^7^Department of Neurology, Innlandet Hospital Trust, Lillehammer, Norway; ^8^Norwegian Centre for Headache Research (NorHead), The Norwegian University of Science and Technology, Trondheim, Norway, Department of Neurology, Haukeland University Hospital, Bergen, Norway, Institute of Clinical Medicine, University of Bergen, Bergen, Norway; ^9^Department of Neurology, Oslo University Hospital, Oslo, Norway; ^10^Institute of Clinical Medicine, Faculty of Medicine, University of Oslo, Oslo Norway; ^11^NeuroClinic Norway, Lillestrøm, Norway; ^12^Department of Neurology Namsos Hospital, Namsos, Norway, ^13^Sandvika Neuro Center, Sandvika, Norway; ^14^Department of Neurology, University Hospital of North Norway, Tromsø, Norway; ^15^Department of Neurology, Stavanger University Hospital, Stavanger, Norway; ^16^Department of Neurology, Molde Hospital, Molde, Norway; ^17^Norwegian Centre for Headache Research (NorHead), The Norwegian University of Science and Technology, Trondheim, Norway; ^18^National Hospital for Neurology and Neurosurgery, University College London Hospital, London, UK; ^19^Department of Neurology, Oslo University Hospital, Oslo, Norway and Institute of Clinical Medicine, Faculty of Medicine, University of Oslo, Oslo, Norway; ^20^Danish Headache Center, Department of Neurology, Rigshospitalet Glostrup, Copenhagen, Denmark and Faculty of Health and Medical Sciences, University of Copenhagen, Copenhagen, Denmark; ^21^Department of Neurology, Sahlgrenska University Hospital, Gothenburg, Sweden; ^22^Institute of Clinical Medicine, Faculty of Medicine, University of Oslo, Oslo Norway and Health Services Research Unit, Akershus University Hospital, Lørenskog, Norway; ^23^Mayo Clinic International, Arizona, USA; ^24^Norwegian Centre for Headache Research (NorHead), The Norwegian University of Science and Technology, Trondheim, Norway and Department of Neurology and Clinical Neurophysiology, St. Olav University Hospital, Trondheim, Norway; ^25^Department of Neurology, Oslo University Hospital, Oslo, Norway and Norwegian Centre for Headache Research (NorHead), The Norwegian University of Science and Technology, Trondheim, Norway

**Background and Aims:** There is tremendous evidence demonstrating the efficacy of calcitonin gene‐related peptide monoclonal antibodies (CGRP mAbs) in migraine patients. However, patients with chronic migraine considered as responders may still experience substantial disease burden. For this patient group, a combination of CGRP mAbs and onabotulinumtoxin A, might be beneficial. Although there are real‐world data supporting this combination therapy, evidence from randomized controlled trials is lacking. The aim of the NorMig trial is to assess the efficacy of dual therapy with CGRP mAbs and BTA compared to single therapy with CGRP mAbs in chronic migraine patients.

**Methods:** NorMig is an ongoing randomized placebo‐controlled, double‐blind multi‐centre phase III trial of CGRP mAbs and onabotulinumtoxin A versus treatment with CGRP mAbs and placebo in patients with chronic migraine. The trial is conducted in compliance with Guidelines of the International Headache Society for controlled trials of preventive treatment in chronic migraine. Primary outcome is the reduction of Monthly Migraine Days over 12 weeks of treatment with the study medication. Patients with chronic migraine aged 18‐70 years with indication for CGRP mAbs or onabotulinumtoxin A and no previous use of CGRP mAbs or onabotulinumtoxin A are included after 4‐week baseline registration.

**Figure:**
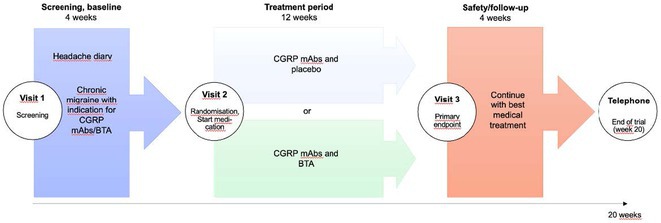
**FIGURE 1** Timeline for the trial. Duration 20 weeks.

**Results:** The trial is starting at Norwegian sites and is planned to be extended to centres in the Nordic countries to reach the sample size of 450 patients. Updated numbers of inclusions will be presented at the congress.

**Figure:**
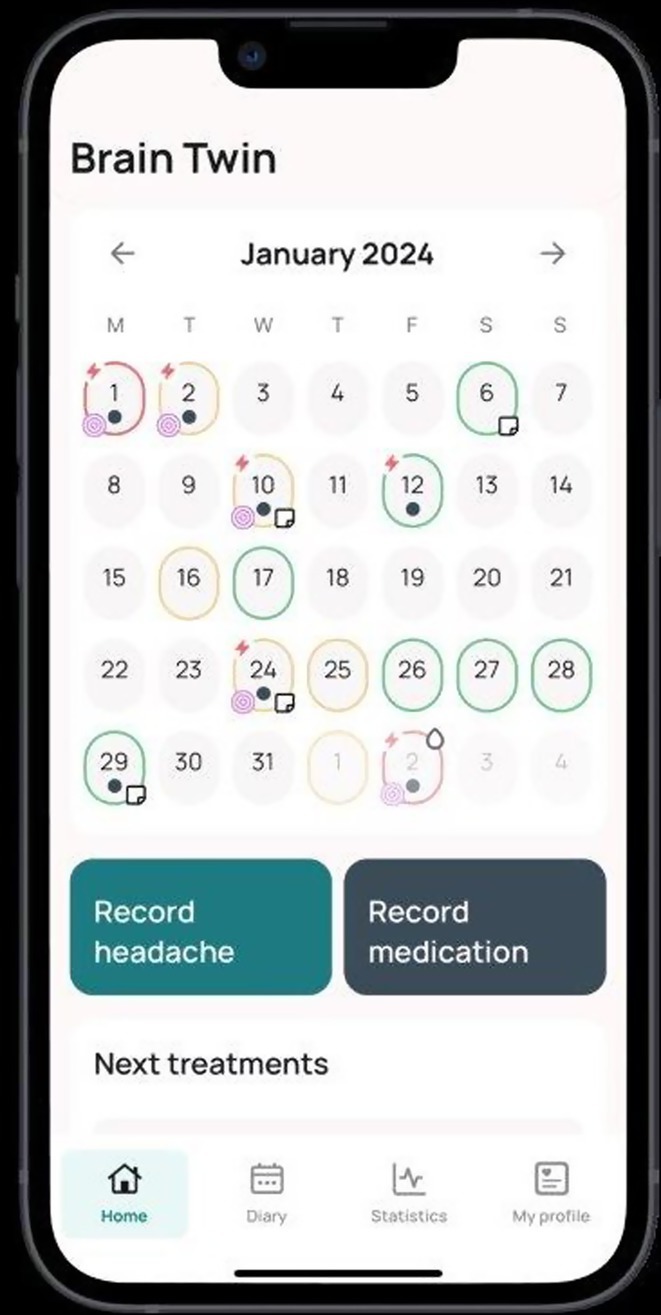
**FIGURE 2** Brain Twin app. Migraine tracker & headache diary.

**Figure:**
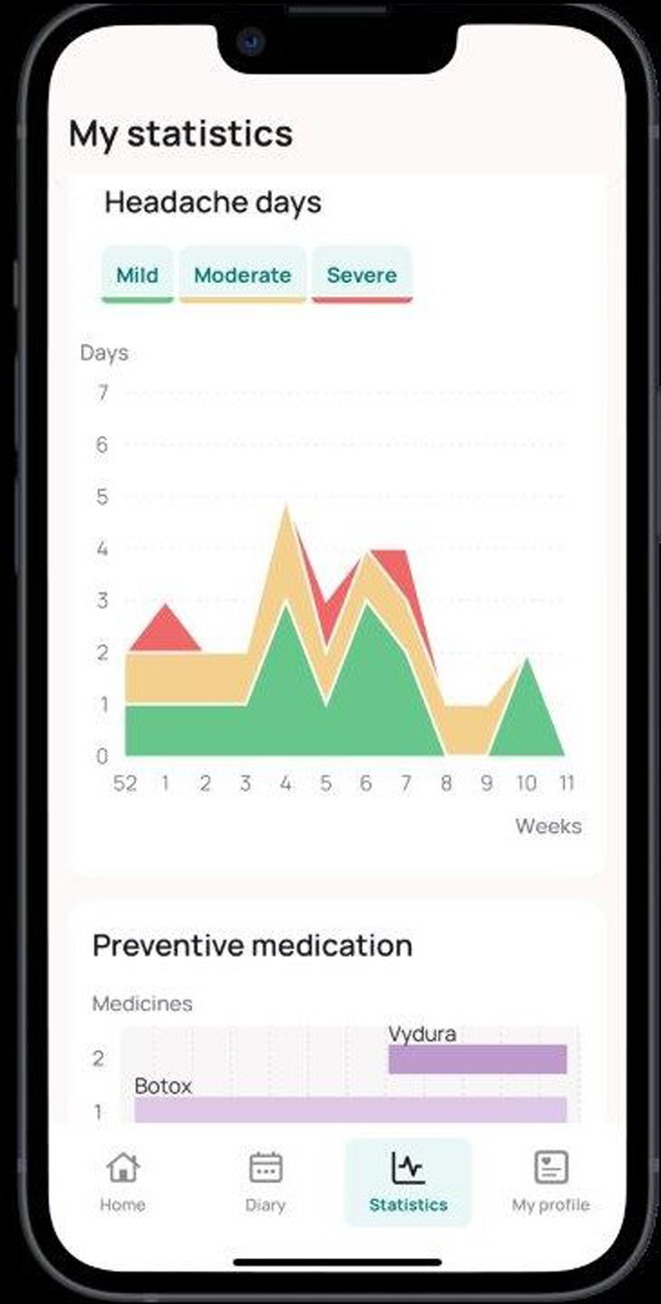
**FIGURE 3** Brain Twin app. Migraine tracker & headache diary.

**Conclusion:** The NorMig trial has the potential to change current treatment practice for chronic migraine and reduce migraine related disability.

**Disclosure:** Lecture presentations for Lundbeck and Abbvie. Attended Nordic Migraine Symposium arranged by Teva.

## EPO‐409

### Identification of barriers in migraine care: A national survey of primary care physicians in Singapore

#### C. Ng ^1^; J. Dang^1^; N. M.Raj^1^; C. Tan^1^; Y. Zhao^2^; Y. Idu Jion^1^

##### ^1^Department of Neurology, National Neuroscience Institute, Singapore; ^2^Zhao Neurology and Headache Clinic, Mount Elizabeth Hospital, Singapore

**Background and Aims:** Despite the high prevalence and disability burden of migraine globally, it remains underdiagnosed and management is suboptimal. This study aimed to identify clinical gaps and educational needs for migraine care in the primary care setting in Singapore.

**Methods:** A national cross‐sectional survey of primary care physicians in Singapore was conducted. The questionnaire evaluated confidence in diagnosing migraine and initiating prophylaxis, the frequency of addressing the impact of migraines on patients’ lives, comorbid conditions, counseling on medication overuse, and the top reasons for referring patients to neurologists.

**Results:** A total of 94 primary care physicians participated. Of these, 58.5% (55/94) were confident in diagnosing migraine, 29.8% (28/94) were somewhat confident, and 10.6% (10/94) were not confident. Confidence in initiating prophylaxis was lower, with 29.8% (28/94) confident, 35.1% (33/94) somewhat confident, and 31.9% (30/94) not confident. Regarding the impact of migraine, 36.2% (34/94) inquired often about burden of migraine, 40.4% (38/94) did so occasionally, and 22.3% (21/94) rarely. Physicians Inquired about comorbidities associated with migraine often in 41.5% (39/94), sometimes in 34% (32/94), and rarely in 21.3% (20/94). Counseling on medication overuse was provided often by 38.3% (36/94), occasionally by 31.9% (30/94), and rarely by 28.7% (27/94). Neurologist referrals were mainly for concerns about secondary headaches (51.1%), treatment‐resistant headaches (50%), patient preference for specialist opinions (39.4%) and diagnostic uncertainty (39.4%).

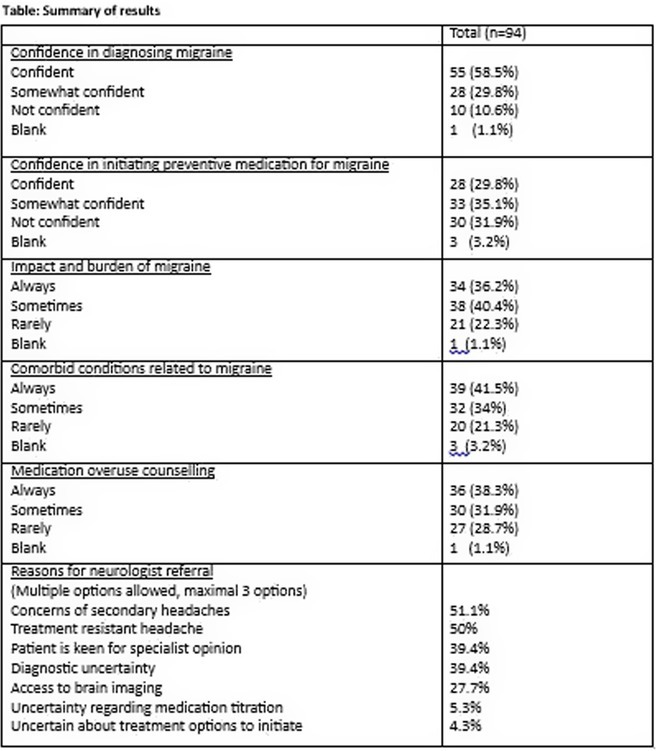

**Conclusion:** Primary care physicians in Singapore exhibited variable confidence and inconsistency in managing migraines. Structured training, robust education, adherence to guidelines, and clear workflows are crucial to improve outcomes for migraine patients.

**Disclosure:** Nothing to disclose.

## EPO‐410

### Headache attributed to low cerebrospinal fluid fistula: A clinical description with therapeutic particularities

#### E. Varas Martín ^1^; M. Pedraza Hueso^1^; M. Ros González^1^; M. Freire Lázaro^1^; C. Montero Grande^1^; P. Jiménez Caballero^1^; P. Puime Rey^1^; M. García Arteche^1^; D. García‐Azorín^2^; Á. Guerrero‐Peral^1^

##### ^1^Headache Unit, Neurology Department, University Clinical Hospital, Valladolid, Spain; ^2^Neurology Department, Rio Hortega Hospital, Valladolid, Spain

**Background and Aims:** Cerebrospinal fluid (CSF) fistula is one of the causes of headache attributed to low CSF pressure. We aim to describe a case managed with conservative treatment.

**Methods:** A 57‐year‐old woman with previous menstrually related migraine. Admitted to emergency room with a one‐week history of cervical stiffness and occipital headache.

**Results:** She was an informal caregiver of a disabled person, and pain began in relation to a transfer. Headache was oppressive, accompanied by nausea and photophobia, with complete resolution in supine position. Anesthetic blockade with 2% lidocaine was performed with transient resolution of the pain. A brain magnetic resonance imaging (MRI) showed pituitary and dural enhancement, with descent of the cerebellar tonsils. An axial MRI revealed linear epidural enhancement in the thoracic region, which was confirmed as a CSF fistula at T9‐T10, in a computed tomography myelography. Fistula was related to a herniated disc, tearing dural sac. Agreement was reached with neurosurgery and the patient, and conservative treatment with rest, hydration, and caffeine was offered. After 2 months, the patient remains paucisymptomatic.

**Figure:**
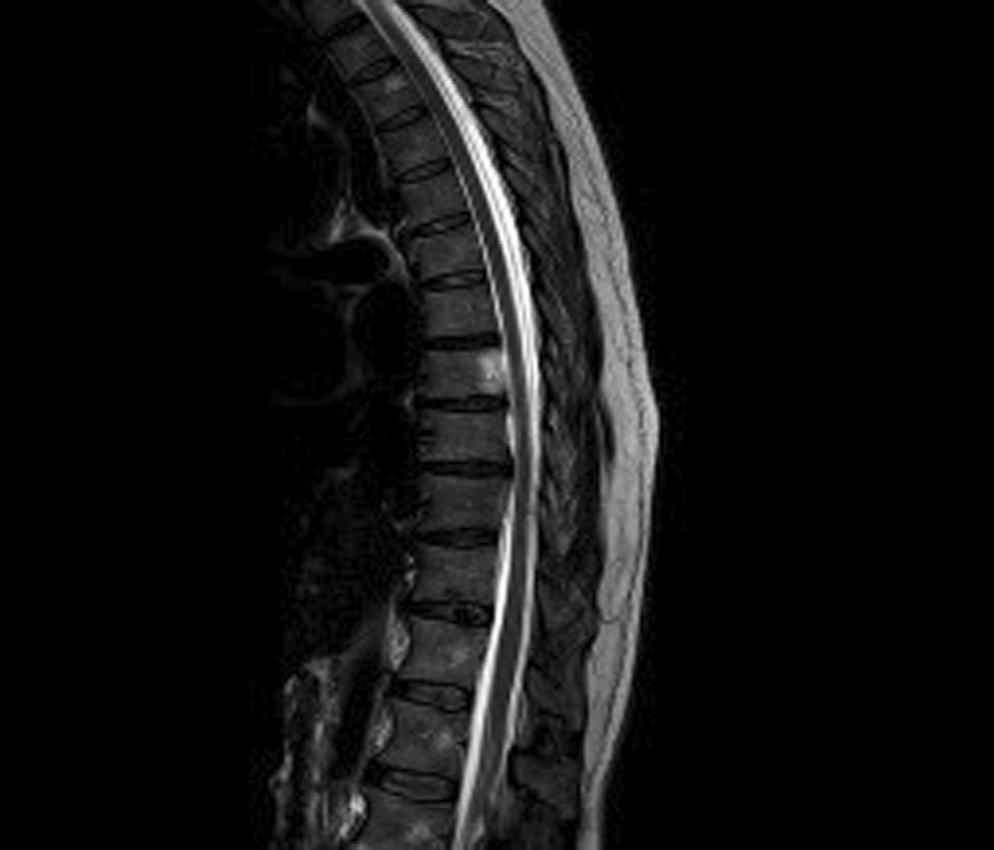
**FIGURE 1** Dural detachment in sagital spinal cord MRI

**Conclusion:** CSF fistula headache is a rare condition, but it should be taken into account in middle‐aged women carrying out physical activities without an adequate ergonomic approach. Conservative treatment may be enough for symptomatic control.

**Disclosure:** Nothing to disclose.

## EPO‐411

### Neuromodulation by transcranial direct current stimulation in the control of refractory neuropathic pain – Long effect

#### E. Carvalho; J. Valicek; P. Bastos

##### Sarah Network of Rheabilitation Hospitals, Brasilia, Brazil

**Background and Aims:** Neuropathic pain is highly prevalent and often difficult to treat. TDCS is effective in controlling neuropathic pain, but the late effect remains unknown.

**Methods:** Serie of cases with neuropathic pain refractory to more than one medication, in the rehabilitation program at SARAH‐BH. They underwent 2 cycles of 8 to 10 sessions of tDCS, anodal, daily, 20 minutes, 2mA in M1, with an interval of up to 6 months between cycles. They were assessed using the visual analogue pain scale (VAS). 22 patients who underwent two cycles of tDCS, with pain improvement, were interviewed at least 6 months after the last session to evaluate the late effect.

**Results:** TDCS sessions were performed in 50 patients; of these, 35 underwent 2 cycles. The diagnoses: traumatic (18)\non‐traumatic (4) tetraplegia, traumatic (18)\non‐traumatic (8) paraplegia and peripheral neuropathy (2). In Cycle 1, 31 (62%) showed an improvement greater than 30% in VAS quantification (D1‐ average 6.97; D10‐ average 4.43). 35 underwent cycle 2: 21 (60%) maintained or increased the initial improvement. LATE EFFECT: 22 were evaluated after average of 13.6 months (6 to 22 months) from the last session: 12 (55%) maintained late benefit; 8 (36%) reported that the improvement lasted a few months and the pain returned as before; 2 (9%) reported that the pain became worse.

**Conclusion:** TDCS is effective, as adjuvant therapy, in multidisciplinary program to control refractory neuropathic pain. The benefit reduces over time after stopping sessions, but 55% of responsive patients still report benefit after average of 13.6 months since the last session.

**Disclosure:** Nothing to disclose.

## EPO‐412

### Prevalence, characteristics and risk factors of migraine among students in Horus University: A cross‐sectional study

#### H. Zehry

##### Neurology Department, Horus University, Mansoura, Egypt

**Background and Aims:** Migraine is a common neurological disorder with a significant disease burden. A number of different factors can trigger migraine attacks as anxiety, stress, skipped meals and irregular sleep pattern. This study was conducted to estimate the prevalence of migraine and to determine its characteristics in students of Horus University, Egypt.

**Methods:** A cross‐sectional study was conducted using a self‐administered questionnaire. The study included 1339 students. Migraine‐related quality of life and disability were assessed using Migraine Specific Quality of life Questionnaire (MSQ) and Migraine Disability Assessment Scale (MIDAS) respectively

**Results:** The overall prevalence of migraine was 24%. The most frequent migraine triggers were mental stress, exertion, sleep disturbance and prolonged mobile use (74%, 72.7%, 68% and 55.8% respectively). Being a female, in the middle academic years and having low academic degrees were significant predictors of migraine among university students. Regarding migraine students, disability was significantly higher among females and students who don’t live with their families. Besides, their quality of life was significantly low among males, nonmedical students, students with low academic degrees and those with irregular physical exercise.

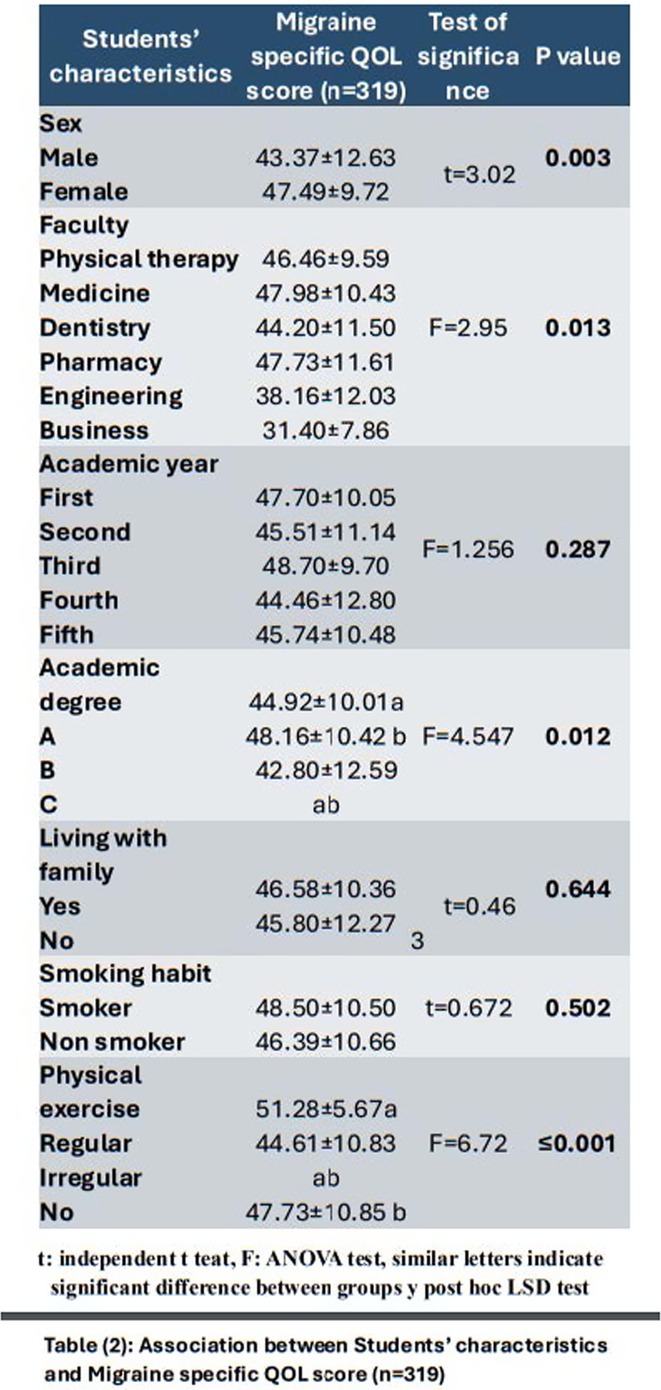

**Conclusion:** Migraine is highly prevalent among university students with significant disability and negative impact on their quality of life.

**Disclosure:** Nothing to disclose.

## EPO‐413

### Gender differences in physical activity, sleep and personal care among armenian population with chronic back pain

#### H. Sargsyan ^1^; M. Manukyan^2^; N. Aghasaryan^3^; N. Yeghiazaryan^4^

##### ^1^Erebouni MC Department of Neurology and Erebouni Pain Clinic, Yerevan, Armenia; ^2^Erebouni MC, Pain Clinic, Yerevan, Armenia; ^3^Erebouni MC, Department of Anesthesiology and Erebouni Pain Clinic, Yerevan, Armenia; ^4^Erebouni MC, Department of Neurology, Yerevan, Armenia

**Background and Aims:** Background: Chronic back pain has a profound impact on various aspects of daily life. The aim of this study is to investigate gender differences in physical activity, sleep patterns, personal care, and social life among patients with chronic back pain.

**Methods:** Methods: A cross‐sectional study included 1562 patients diagnosed with chronic back pain (CBP), with no prior spine surgery, from 3 multidisciplinary medical centers. Participants completed the Oswestry Low Back Pain Disability Questionnaire. To assess gender differences, the mean and standard deviation (STDEV) of ODI scores for each domain were calculated. The statistical comparison between males and females for each category was performed using the Student's T‐test with a two‐tailed distribution and two‐sample unequal variance (heteroscedastic).

**Results:** 967 women and 595 men were included. The overall total ODI% showed that men are significantly more affected than women (mean ODI% 40.1 + 20.32 in men versus mean 36.4+19 in women, *p* = 0.0005). The independent *T*‐tests conducted between two groups on all ODI criteria (pain intensity, ease of personal care, lifting, working, sitting, standing, sleeping, sex life, social life and traveling) showed no significant difference in pain intensity and social life, whereas personal care, physical activities, including sex life and travelling were more affected in men (see table 1&2).

**Figure:**
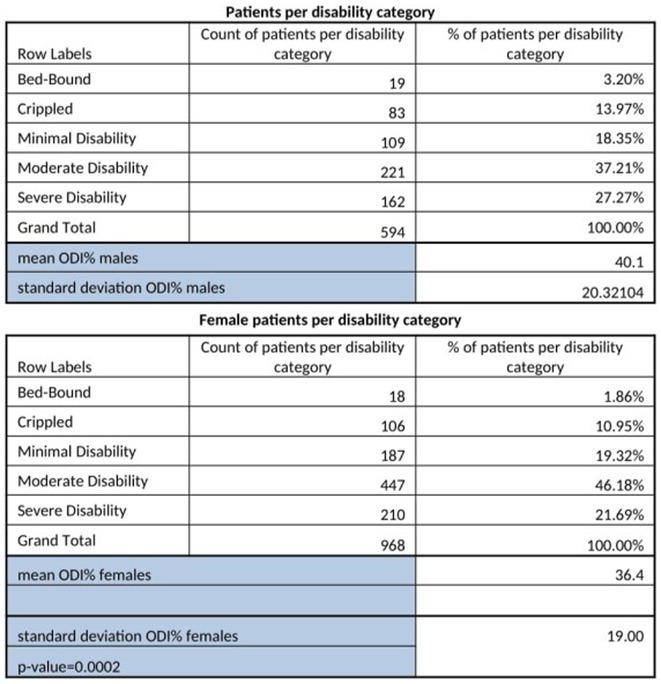
TABLE 1

**Figure:**
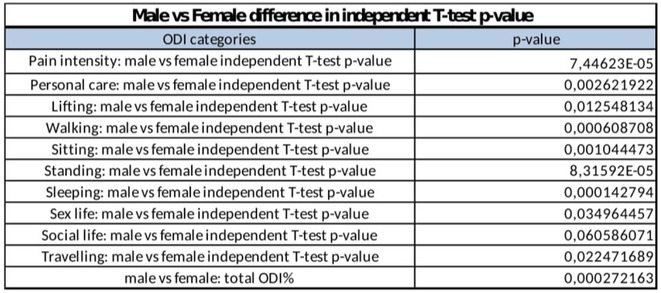
TABLE 2

**Conclusion:** Gender differences in chronic back pain disability are evident, with men being more severely affected. However, pain intensity and social life are equally affected in women and men. These findings highlight the importance of considering gender in the management of chronic back pain.

**Disclosure:** Nothing to disclose.

## EPO‐414

### Pharmacoepidemiology of idiopathic intracranial hypertension: An Austrian population based cohort study

#### N. Müller ^1^; N. Krajnc^1^; S. Zaic^1^; S. Macher^1^; C. Wöber^1^; W. Marik^2^; K. Novak^3^; B. Pemp^4^; B. Reichhardt^5^; G. Bsteh^1^

##### ^1^Department of Neurology, Medical University of Vienna, Vienna, Austria; ^2^Department of Neuroradiology, Medical University of Vienna, Vienna, Austria; ^3^Department of Neurosurgery, Medical University of Vienna, Vienna, Austria; ^4^Department of Ophthalmology, Medical University of Vienna, Vienna, Austria; ^5^Austrian Social Health Insurance Fund, Eisenstadt, Austria

**Background and Aims:** Idiopathic intracranial hypertension (IIH) is a rare disorder characterized by headaches and papilledema. This case‐control study utilized a large hospital‐based database to investigate IIH prevalence and treatment patterns, invasive and non‐invasive therapies

**Methods:** The Austrian health insurance register (>99% population coverage) was queried for patients discharged between 2016 and 2021 with ICD‐10 code G93.2 and/or acetazolamide (AZM) prescription. IIH was considered confirmed if G93.2 was assigned ≥2 times and AZM was prescribed ≥ once. Five obese controls (OBC, ICD‐10: E65/66/68) and five general population controls (GPC) were drawn from the register for each patient. Cumulative defined daily doses (cDDD) of prescribed medications and performed procedures were extracted.

**Results:** Of 5,969 patients identified, 114 met the criteria for confirmed IIH, yielding an estimated hospital‐based prevalence of 0.78 per 100,000 discharges in total and 1.34 per 100,000 female discharges. Compared to 114 GPC and 114 OBC matched for age and sex, IIH patients had higher prescription rates of furosemide (18.4%, cDDD: 0.52 mg/d) and topiramate (39.5%, 0.26 mg/d). Invasive procedures were more frequent in IIH, with lumbar punctures in 13% (vs. 0%) and ventriculoperitoneal shunting in 18.4% (vs. 0%). Optic sheath fenestration was not observed. Bariatric surgery rates were lower (4.4%) than in OBC (31.6%) but higher than in GPC (0%).

**Figure:**
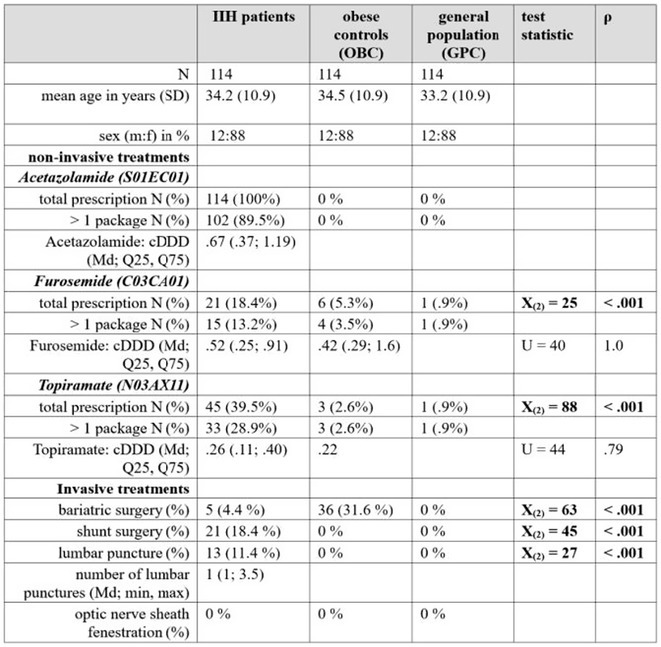
**TABLE 1** Demographics and frequency of invasive/non‐invasive therapies SD = standard deviation, Md = Median, Q25 = lower quartile, Q75 = upper quartile, m = male, f = female, cDDD = cumulative defined daily dose.

**Conclusion:** The estimated IIH prevalence is within the reported range for Middle Europe but likely underestimated due to reliance on hospital discharge data. Treatment patterns reflect guideline‐based management, though the high frequency of invasive procedures suggests a bias toward more severe cases.

**Disclosure:** Funding There was no funding to this research. Competing interests Nina Müller^1,2^, Nik Krajnc^1,2^, Sina Zaic^1,2^, Stefan Macher^1,2^, Christian Wöber^1,2^, Wolfgang Marik^2,3^, Klaus Novak^2,4^, Berthold Pemp^5^, Berthold Reichardt^6^, and Gabriel Bsteh^1,2^ NM: declares no conflict of interest relevant to this study NK: has participated in meetings sponsored by, received speaker honoraria or travel funding from BMC/Celgene, Merck, Novartis, Roche and Sanofi‐Genzyme. SZ: declares no conflict of interest relevant to this study SM: declares no conflict of interest relevant to this study CW: has received honoraria consultancy/speaking from Apomedica, Curelator, Eli Lilly, Grünenthal, Hermes, Novartis, Pfizer, Ratiopharm/Teva, and Stada WM: declares no conflict of interest relevant to this study. KN: declares no conflict of interest relevant to this study. BP: has received honoraria for consultancy/speaking from Chiesi, GenSight and Santen. BR: declares no conflict of interest relevant to this study. GB: has participated in meetings sponsored by, received speaker honoraria or travel funding from Biogen, Celgene/BMS, Lilly, Merck, Novartis, Roche, Sanofi‐Genzyme and Teva, and received honoraria for consulting Biogen, Celgene/BMS, Novartis, Roche, Sanofi‐Genzyme and Teva. He has received unrestricted research grants from Celgene/BMS and Novartis.

## EPO‐415

### Preventive migraine treatment in primary care and headache clinic: A follow‐up analysis in a Portuguese hospital

#### S. Moreira; A. Costa; A. Câmara; S. Gomes; J. Araújo; S. Varanda

##### Neurology, Local Health Unit Braga, Braga, Portugal

**Background and Aims:** Migraine treatment has evolved. This study compares the management of migraine patients referred to a Portuguese tertiary hospital in 2023 and 2017, analyzing referral patterns, treatments, and follow‐up.

**Methods:** Retrospective analysis. Data included referral sources, treatments, and follow‐up duration. Inclusion criteria: age ≥18; managed in primary care pre‐referral, no prior neurology follow‐up.

**Results:** In a Portuguese tertiary hospital, 22/85 first consultations in 2023 and 32/136 in 2017 met the criteria. In 2023, the majority were women (*n* = 17; 77.3%; mean age 41.6 years) as in 2017, (*n* = 31; 96.9%; mean age 33.9 years). Patients in 2023 were significantly older (*p* = 0.018). Preventive treatment criteria were met by 90.9% of patients in 2023 compared to 75% in 2017. The proportion of patients referred with prior preventive treatments increased in 2023 vs 2017 (77.3% vs 28.1%, *p* < 0.001). Patients in 2023 initiated/tried significantly more preventive treatments before referral (2.0 vs 0.34; *p* < 0.001). In primary care, the most prescribed preventives were topiramate (31.6%) and amitriptyline (26.3%) in 2023; propranolol (41.7%) and amitriptyline (25.0%) in 2017. In headache clinic, the mean number of preventive treatments attempted in 2023 was significantly higher compared to 2017 (1.3 vs. 0.7; *p* = 0.017). In 2023, topiramate (18.75%) and galcanezumab (18.75%) were most prescribed, while in 2017, propranolol (36.4%) and topiramate (27.3%). The mean follow‐up duration was longer in 2023 (452.0 vs 289.1 days; *p* = 0.004).

**Conclusion:** Migraine management improved with more preventive treatments initiated in primary care, greater use of advanced therapies, and longer follow‐up durations, reflecting significant progress in both primary and specialized care.

**Disclosure:** Nothing to disclose.

## EPO‐416

### Bilateral occipital nerve block in children with chronic migraine

#### T. Uyar Cankay

##### Istanbul Goztepe Prof.Dr. Suleyman Yalcın Research Hospital, Istanbul, Turkey

**Background and Aims:** Migraine is a common type of pain that has been recognized for thousands of years, yet its pathophysiology and morphological effects are not fully understood, and it can be remarkably resistant to treatments. Greater occipital nerve (GON) block is an effective and minimally invasive treatment option for primary headaches that can be used in patients older than 8 years, with relatively few side effects.

**Methods:** In this study, we retrospectively evaluated the efficacy of greater occipital nerve block in children and adolescents with migraine. We reviewed the medical records of patients aged 12 to 18 who had been diagnosed with migraine and treated with GON block. The GON block was performed bilaterally with 2 cc of 2% lidocaine weekly for 4 weeks, and monthly thereafter. Patients' headaches were assessed using a headache form, the PedMIDAS (Ped Migraine Disability Assessment) scale for evaluating migraine‐related disability in children, and the Headache Impact Test (HIT) forms at both baseline and after GON application.

**Results:** A total of 22 patients were evaluated in the study, all of whom received their first GON block. The mean age of the patients was 14.2 years (range 11–18). 17 patients were female and 5 were male. GON block showed significant efficacy, with improvements in frequency, severity (measured by VAS), PedMIDAS, and HIT scales.

**Conclusion:** GON block is an effective and safe treatment option for children and adolescents with migraine, with minimal side effects, and should be considered as a first‐line treatment option.

**Disclosure:** Nothing to disclose.

## EPO‐417

### Hemiplegic migraine attack misinterpreted as a stroke in postcoronarography – Case report

#### V. Bohotin ^1^; D. Spinu^1^; A. Spinu^1^; C. Bohotin^2^

##### ^1^Department of Neurology, Louis Pasteur Hospital, Chartres, France; ^2^CICAT‐28, France

**Background and Aims:** Hemiplegic migraine (HM) is a rare form of migraine with aura. The focal deficit can be misinterpreted as a stroke, making diagnosis challenging. This highlights the importance of a detailed patient history. However, obtaining this information can be difficult in the limited time available, as the benefit of brain reperfusion techniques is time‐dependen

**Methods:** We present a case of a patient who developed right hemiplegia, aphasia, and somnolence immediately following coronary angiography. A CT scan and carotid angiography performed promptly showed no abnormalities. The initial diagnosis was an incidental post‐coronary angiography ischemic stroke. However, a brain MRI performed a few days later revealed no evidence of stroke. The diagnosis of hemiplegic migraine (HM) was considered following a thorough history, provided by the patient's wife, which revealed multiple prior episodes and a history of migraines with aura that began at the age of 15. The patient had previously been hospitalized multiple times for motor deficits and coma of unknown origin, with numerous CT scans, brain MRIs, EEGs, and lumbar punctures consistently yielding no definitive findings.

**Results:** After admission, the motor deficit, aphasia, and somnolence fully resolved within 7 days. Cognitive assessments conducted during the acute phase and again 10 days later showed a remarkable improvement in test results. Genetic analysis identified a CACNA1A mutation (c.4523C>T/p.Ala1508Val)

**Conclusion:** Stroke mimics present a diagnostic challenge for all admissions to a stroke unit. Thorough investigations and a detailed patient history are crucial to avoid misdiagnosis, inappropriate medication prescriptions, and unnecessary tests

**Disclosure:** Nothing to disclose.

## EPO‐418

### Non‐headache features in migraine as predictors of super‐responders to prophylactic treatment

#### V. López Díaz; C. Díaz Garza; A. Espino Ojeda

##### Neurology, Tecnologico de Monterrey/Escuela de Medicina, Monterrey, Nuevo Leon, Mexico

**Background and Aims:** The non‐headache symptoms in migraine (NHS) could be classified as hypothalamic, autonomic, psychiatric, or hypersensitivities. These symptoms may occur during premonitory, ictal, or postdrome phases and could represent manifestations of different pathophysiological pathways. We aimed to look for a relationship between the NHS and the degree of response to preventive treatment in migraine.

**Methods:** Retrospective, cross‐sectional study included 58 migraine patients on prophylactic medication for at least one month. Data on demographics, headache characteristics, and non‐headache symptoms were collected via patient interviews and medical records. Patients were categorized into super‐responders (SR) if monthly headache days (MHD) decreased by at least 75% after preventive treatment. Statistical analyses were performed to identify associations between symptoms and treatment response.

**Results:** Among the 58 recruited patients (mean age 35 ± 11, 84% females), 37 (63%) were SR. There was no difference in basal MHD between SR and the others (17.7 vs 22.1 *p* 0.2) treatment used (topiramate in 51% vs 52%), number of preventive treatments used (SR 2 [RIQ 1–3] vs others 1[RIQ 1–3] *p* 0.7). Premonitory osmophobia was more frequent in SR (35% vs 9%, *p* 0.03). Ictal conjunctival injection was also more prevalent in SR (24% vs. 0%, *p* 0.02). There were no differences in the prodromic phase.

**Figure:**
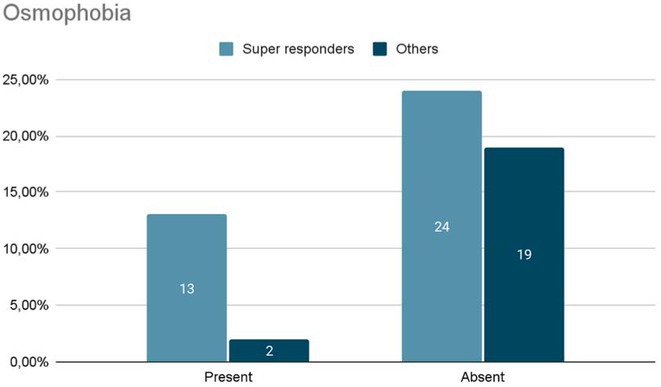
**FIGURE 1** The relationship between premonitory osmophobia and super‐response to migraine prophylaxis, with a treatment response rate of 75% or more (*p* = 0.03), highlighting its potential as a predictive factor.

**Conclusion:** The counterintuitive finding of conjunctival injection associated with a better response could be related to a predominantly peripherally sensitization, which could respond easily to treatment compared with central sensitization. Osmophobia was also associated with a better response rate.

**Disclosure:** Nothing to disclose.

## EPO‐419

### SS‐31 alleviated nociception and restored mitochondrial function in a headache mouse model via the Sirt3/Pgc‐1α loop

#### Z. Xiao

##### Department of Neurology, Renmin Hospital of Wuhan University, Wuhan, China

**Background and Aims:** Migraine, the second leading cause of disability, imposes significant socioeconomic burden. Mitochondrial dysfunction has been implicated in migraine, and Szeto‐Schiller peptide (SS‐31), a mitochondria‐targeted peptide, has shown promise in restoring mitochondrial function in various diseases. However, its potential effect on migraine remains unclear.

**Methods:** A headache mouse model was induced by repeated dural infusion of inflammatory soup (IS). The roles of the Sirt3/Pgc‐1α positive feedback loop in mitochondrial function and headache pathogenesis were examined. SS‐31 was administered, and mitochondrial function, ultrastructure, and nociceptive responses were assessed.

**Results:** IS infusion impaired mitochondrial function and homeostasis in the trigeminal nucleus caudalis (TNC). SS‐31 reversed these impairments and alleviated IS‐induced nociceptive responses. The effects of SS‐31 were partially attenuated by a Sirt3/Pgc‐1α inhibitor. Overexpression of Sirt3/Pgc‐1α enhanced their protein levels, indicating their positive feedback loop.

**Conclusion:** SS‐31 restores mitochondrial function and alleviates nociceptive responses in an IS‐induced headache model through the Sirt3/Pgc‐1α positive feedback loop.

**Disclosure:** Nothing to disclose.

## Movement disorders 4

## EPO‐420

### A novel variant in GNAO1 causes epilepsy and movement disorder with a variable phenotypic spectrum in the same family

#### M. Delgado‐Alvarado ^1^; E. Onecha^2^; J. Infante^3^; M. Rivera‐Sánchez^3^; S. Setién^4^; M. Misiego‐Peral^4^; J. Sánchez‐de la Torre^4^; Y. Jiménez´López^4^; D. Gallo‐Valentín^4^; J. Riancho^1^

##### ^1^Neurology Department, Hospital Sierrallana, Torrelavega, Spain. IDIVAL, Santander, Spain. CIBERNED, Madrid, Spain; ^2^Molecular Genetics Department, University Hospital Marqués de Valdecilla‐IDIVAL, Santander, Spain; ^3^Neurology Department, University Hospital Marqués de Valdecilla‐IDIVAL, Santander, Spain; ^4^Neurology Department, Sierrallana Hospital, Torrelavega, Spain

**Background and Aims:** We describe a family with a novel pathogenic variant in the GNAO1 gene and its phenotypic variability.

**Methods:** A 56‐year‐old woman with moderate intellectual disability since childhood and generalized convulsive epilepsy starting at 47, well controlled with levetiracetam, complained of tremor, beginning at 47. Physical exam showed short stature (135 cm), dysmorphic features, a characteristic voice, bilateral intentional tremor (+/‐ myoclonus), and cervical dystonia (Video 1A). Cranial MRI was normal. Genetic analysis revealed a heterozygous c.649G>T variant in GNAO1, likely pathogenic.

**Results:** The family includes 11 siblings with non‐consanguineous parents (figure 1). A 50‐year‐old sister (II.4) had mild intellectual disability, anxiety, and her first generalized seizure at 49. Examination showed short stature (135 cm), a characteristic voice, hyperreflexia, mild chorea, and mild cervical dystonia (Video 1B). The 91‐year‐old mother had mild intellectual disability, subtle orolingual chorea, mirror movements, short stature (139 cm), and no epilepsy (Video 1C). Another brother (II.3) and sister (II.12) were reportedly affected by intellectual disability, psychiatric disorders, and, in the case of the latter, epilepsy, but they were unavailable for evaluation. The affected sister and mother carried the GNAO1 variant, whereas two unaffected sisters (II.6, II.8) did not. The c.649G>T variant is a nonsense mutation causing a premature stop codon at position 217. Loss‐of‐function variants in GNAO1 are associated with neurodevelopmental disorders, epilepsy, and movement disorders. This variant is absent from dbSNP and gnomAD databases, with a high pathogenic CADD score (40.0).

**Figure:**
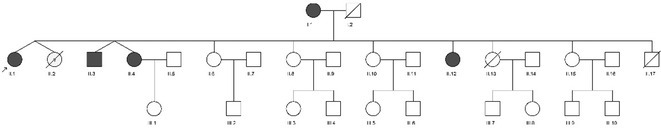
FIGURE 1

**Conclusion:** The c.649G>T mutation in GNAO1 is pathogenic, causing intellectual disability, epilepsy, and movement disorders with variable phenotypes.

**Disclosure:** Nothing to disclose.

## EPO‐421

### Safinamide significantly improves motor complications, motor and non‐motor symptoms in Parkinson's disease patients

#### C. Cattaneo

##### Medical Department Zambon SpA, Bresso, Italy

**Background and Aims:** Chronic levodopa treatment is associated with motor complications and non‐motor symptoms. Glutamate, besides other neurotransmitters, has been implicated in their development. Safinamide is multimodal drug with a dual mechanism of action, dopaminergic and glutamatergic, and has therefore the potential to improve these phenomena.

**Methods:** The effects of safinamide on motor complications, motor and non‐motor symptoms were investigated using the data from eight interventional, double‐blind, placebo‐controlled clinical trials performed in Caucasian and Asian patients. Outcomes included OFF time, ON time without troublesome dyskinesia, UPDRS III motor scores, PDQ‐39 mood and pain scores.

**Results:** Safinamide, compared to placebo, significantly improved ON time without troublesome dyskinesia (*p* < 0.0001), OFF time (*p* = 0.0001), motor symptoms (*p* < 0.0010), mood (*p* < 0.0009) and pain (*p* = 0.0014) with a good safety profile and without requiring any change in the concomitant dopaminergic therapy. These benefits were maintained also after a long‐term treatment (up to 2 years).

**Conclusion:** Safinamide, administered as add‐on therapy in fluctuating PD patients, improved motor symptoms, motor complications, mood, and pain without increasing troublesome dyskinesia. These effects may be explained by the modulation of glutamatergic hyperactivity. Further prospective studies are needed to fully explore its therapeutic potential.

**Disclosure:** Carlo Cattaneo is an employee of Zambon SpA.

## EPO‐422

### Treatment preferences in advanced Parkinson's disease: A discrete‐choice experiment subgroup analysis

#### R. Pahwa^1^; J. Domingos^2^; I. A. Malaty^3^; K. Ray Chaudhuri^4^; A. Antonini^5^; F. De Renzis^6^; P. Arija^7^; M. Heisen^8^; H. Penton^7^; C. H. Yan ^9^; E. Shirneshan^9^; M. Shah^9^; P. Kukreja^9^; J. Carlos Parra^9^; M. Boeri^10^

##### ^1^University of Kansas Medical Center, Kansas City, USA; ^2^Parkinson's Europe, Orpington, UK; Egas Moniz School of Health & Science, Almada, Portugal; ^3^University of Florida, Fixel Institute for Neurological Diseases, Gainesville, USA; ^4^Kings College, London, UK; ^5^Parkinson and Movement Disorders Unit, Study Centre for Neurodegeneration, Department of Neuroscience, University of Padova, Padova, Italy; ^6^Parkinson's Europe, Orpington, UK, ^7^Patient Centered Outcomes, OPEN Health, Rotterdam, The Netherlands; ^8^Heisen Health, Utrecht, The Netherlands; ^9^AbbVie Inc., North Chicago, USA; ^10^Preference Research and Scientific Lead for Patient Centered Outcomes, OPEN Health, UK

**Background and Aims:** Preferences for advanced Parkinson's Disease (aPD) treatments are often influenced by an individual's characteristics. This study aimed to identify which characteristics of people with aPD (PwaP) impact treatment preferences. Care partners (CPs) reported proxy preferences for PwaP.

**Methods:** A discrete‐choice experiment was conducted with 304 participants, requiring respondents to choose between pairs of hypothetical PD treatments with varying attribute levels (Table 1). Attribute Relative Importance (RI) was calculated using random‐parameter logit estimates. Four subgroups were assessed (Table 2).

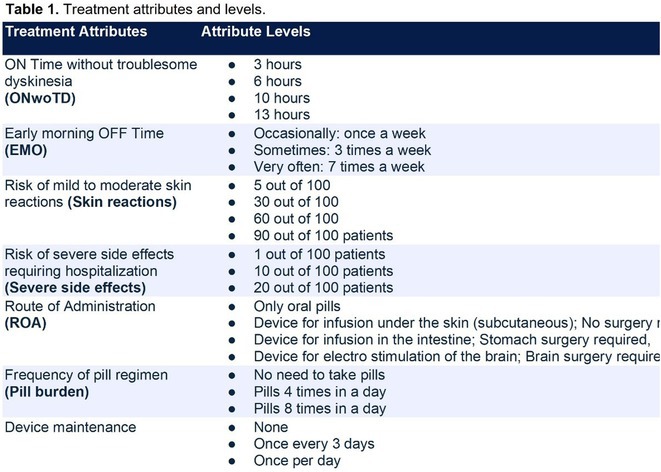

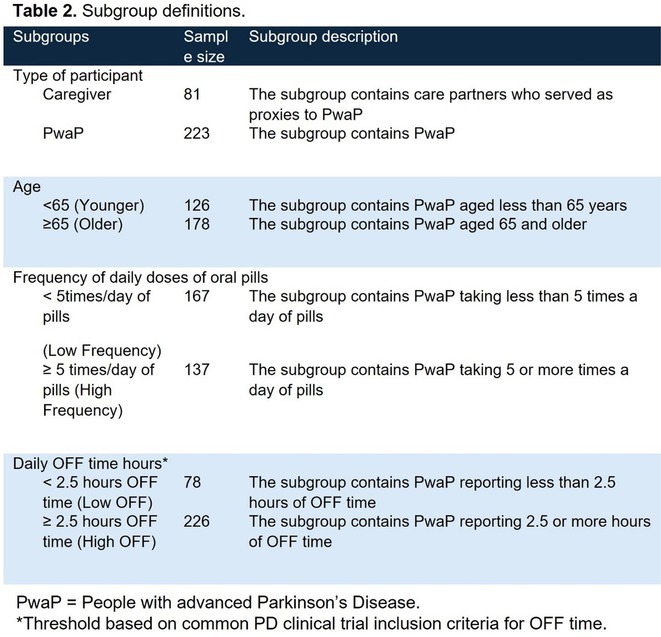

**Results:** PwaP had a mean age of 65.7 years (SD = 8.6), were diagnosed 10.0 years ago (SD = 4.2), and reported 4.0 OFF hours/day (SD = 2.4). PwaP received 29.6 (SD = 22.2) hours of CP support weekly. Across all subgroups, ON time without troublesome dyskinesia (ONwoTD) and route of administration (ROA) were main priorities. CPs valued ONwoTD significantly more than PwaP (RI = 35.9 vs. 22.8), while PwaP gave greater importance to risk of skin reactions (RI = 14.6 vs. 6.9) and ROA (RI = 36.6 vs. 30.5) than CPs. No statistically significant preferences linked to age, although younger participants emphasized ROA more than older participants (RI = 37.8 vs. 34.3). Conversely, older participants valued ONwoTD more (RI = 29.4 vs. 25.6). Those with higher pill frequencies prioritized ONwoTD more than those with lower frequencies (RI = 32.4 vs. 19.5). Self‐reported daily OFF time had no significant influence on preferences.

**Conclusion:** Treatment preferences for PwaP are diverse, with ROA and ONwoTD as key attributes. These insights can guide healthcare providers in understanding unique PwP and CP priorities, enabling more personalized and effective PD management, and enhancing adherence to treatment regimens.

**Disclosure:** RP has received fees, honoraria, and/or grants from AbbVie, ACADIA, Avid, Acorda, Adamas, Biotie, Civitas, Cynapses, Global Kinetics, Kyowa, Lundbeck, National Parkinson Foundation, Neurocrine, NIH/NINDS, Parkinson Study Group, Pfizer, Sage, Sunovion, Teva Neuroscience, and US World Meds. JD represents Parkinson's Europe. IM has received fees, honoraria, royalties, and/or grants from the Parkinson Foundation, Dystonia Coalition, AbbVie, Emalex, Medscape, Neuroderm, Praxis, Revance, Sage, Tourette Association of America, and Robert Rose Publishers. KRC has received fees, honoraria, and/or educational funds from AbbVie, Bial, Britannia, Britannia Bial, US Worldmeds, Otsuka, Medtronic, Zambon, Sunovion, Scion, and UCB. AA has received fees, honoraria, and/or grants from AbbVie, Bayer, Biopharma, Bial, Britannia, Ever Pharma, Horizon 2020, Italian Ministry of University and Research, Italian Ministry of Health, Jazz, Medscape, Next Generation EU ‐ National Center for Gene Therapy and Drugs, and Investment PE8 – Project Age‐It: “Ageing Well in an Ageing Society”, Roche, Theravance, UCB, and Zambon. FDR is employed by Parkinson's Europe. PA, HP, and MB are employees of OPEN Health. MH was employed by OPEN Health at the time of study conduct. OPEN Health received funding from AbbVie for the conduct of this study. OPEN Health received funding from AbbVie for the conduct of this study. CHY, ES, MS, PK, and JCP are employees of AbbVie and may own stocks/shares in the company.

## EPO‐423

### Clinical impact of nigrosome MRI–PET discrepancies on motor complications in Parkinson's disease

#### D. Park ^1^; M. Kim^2^; Y. Kim^1^; J. Yoon^1^

##### ^1^Ajou University School of Medicine, Suwon, Republic of Korea; ^2^Dongtan Sacred Heart Hospital, Hallym University College of Medicine, Hwaseong, Republic of Korea

**Background and Aims:** Accurate imaging markers for Parkinson's disease (PD) are key to both diagnosis and disease tracking. While FP‐CIT PET (a dopamine transporter scan) typically confirms nigrostriatal degeneration, MRI‐based nigrosome imaging has emerged as a useful complementary modality. Occasional discrepancies between these modalities—namely, cases in which FP‐CIT PET is abnormal but nigrosome imaging appears normal—have not been thoroughly characterised.

**Methods:** In this retrospective study, we analyzed 88 consecutive patients with clinically diagnosed PD, all showing abnormal FP‐CIT PET findings, who underwent MRI nigrosome imaging at nearly the same time as PET. Among them, seven cases showed discrepant findings between MRI and PET (MRI‐PET discrepant). Demographic and clinical data, including LEDD (levodopa equivalent daily dose) at baseline and follow‐up, wearing‐off events, levodopa‐induced dyskinesia (LID), and Unified Parkinson's Disease Rating Scale (UPDRS) scores, were collected. Kaplan–Meier analysis compared time to wearing‐off or LID onset between patients with consistent and discrepant findings.

**Results:** The MRI‐PET discrepant group had a shorter mean follow‐up duration (46.11 vs. 66.17 months, *p* = 0.044). No differences were found in baseline or follow‐up LEDD, wearing‐off incidence, or UPDRS scores. LID did not occur in patients with discrepant findings, though the small sample size limits conclusions. Kaplan–Meier curves showed no significant difference in time to wearing‐off or LID onset (*p* = 0.27).

**Figure:**
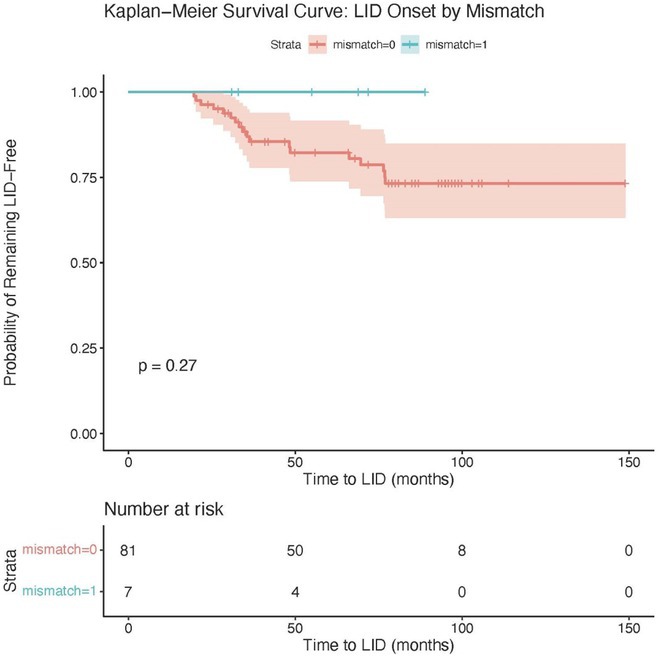
**FIGURE 1** Kaplan‐Meier curves comparing time to levodopa‐induced dyskinesia (LID) between MRI‐PET consistent (mismatch = 0, *n* = 81) and discrepant (mismatch = 1, *n* = 7) groups (*p* = 0.27). Shaded areas represent 95% confidence intervals.

**Conclusion:** MRI‐PET discrepancies are uncommon and suggest minimal clinical impact on wearing‐off or dyskinesia risk, though larger studies may help clarify the underlying physiology and prognostic significance.

**Disclosure:** Nothing to disclose.

## EPO‐424

### Women with Parkinson's disease – Menopause and the role of hormonal replacement therapy

#### K. Rukavina ^1^; V. Carvalho^3^; M. Gavriilaki^5^; I. Colonna^5^; N. Campese^7^; D. Carneiro^8^; M. Delgado Soares^10^; B. Nucera^11^; K. Smilowska^13^; F. Brogueira Rodrigues^14^; K. Aleksovska^2^; M. de Visser^15^; G. Arabia^16^

##### ^1^Movement Disorders Hospital, Beelitz‐Heilstätten, Germany; ^2^European Academy of Neurology; ^3^Egas Moniz Study Center, Faculty of Medicine, University of Lisbon, Lisbon, Portugal; ^5^1st Department of Neurology, AHEPA University Hospital, School of Medicine, Aristotle University of Thessaloniki, Thessaloniki, Greece; ^7^Department of Neurology, Medical University of Innsbruck, Innsbruck, Austria; ^8^Neurology Department, ULS Coimbra, Portugal; ^10^Neurology Department, São José Local Health Unit, Lisbon, Portugal; ^11^Department of Neurology, Hospital of Merano (SABES‐ASDAA), Franz Tappeiner Hospital, Merano, Italy; ^13^Department of Neurology, Regional Hospital of Santa Barbara, Poland; ^14^Laboratory of Clinical Pharmacology and Therapeutics, Faculty of Medicine, University of Lisbon, Lisbon, Portugal; ^15^Department of Neurology, Amsterdam University Medical Centre, Location University of Amsterdam, Amsterdam, The Netherlands; ^16^Neurologic Department, University of Catanzaro “Magna Graecia”, Catanzaro, Italy

**Background and Aims:** Women spend 40% of their lives in the postmenopause. Hormonal replacement therapy (HRT) substantially eases the postmenopausal symptom burden. In Women with Parkinson's disease (WwPD), the impact of HRT on the risk of developing PD and the rate of PD progression remains unclear. This systematic review (PROSPERO ID 636960) investigates 1) the impact of menopause‐related hormonal changes on the natural history of PD and 2) the impact of HRT on health‐related quality of life (HRQoL), motor and nonmotor symptoms (NMS) in WwPD in peri‐and postmenopausal period.

**Methods:** Eligible studies were identified through an electronic search of MEDLINE, Embase, and CENTRAL databases from inception to October 2024, hand‐search of the EAN and MDS abstract books (2019 to 2024) and cross‐checking of references. The titles and abstracts were screened, selected full texts will be assessed and the risk of bias evaluated (RoB2, ROBINS‐I, or JBI checklist for case series) independently by two authors. Disagreements will be resolved through consensus or by a third reviewer. Data extraction will follow using a pre‐established form, designed and pilot‐tested by the authors. Thematic coding, summary and descriptive statistical analysis will be conducted.

**Results:** The search returned 4799 records, 1705 duplicates were removed. After title and abstract screening, 141 articles were selected for full‐text screening. We synthetize available evidence on the impact of menopause/HRT on HRQoL, PD progression and burden of motor and NMS in WwPD.

**Conclusion:** Large RCTs are urgently needed to clarify the effects of HRT in women with Parkinson's disease and inform clinical decision‐making.

**Disclosure:** Nothing to disclose.

## EPO‐425

### Cluster analysis of patient characteristics associated with up to 100% “off” time improvement from two phase 3 trials

#### K. Dashtipour^1^; R. Pahwa^2^; B. Bergmans^3^; P. Odin^4^; M. Shah^5^; L. Bergmann ^5^; J. Homola^5^; C. Yan^5^; R. Gupta^5^; M. Soileau^6^

##### ^1^Loma Linda University Health System, Loma Linda, USA; ^2^University of Kansas Medical Center, Kansas City, USA; ^3^AZ St‐Jan Brugge, Brugge, Belgium and Ghent University Hospital, Ghent, Belgium; ^4^Skåne University Hospital, Lund University, Lund, Sweden; ^5^AbbVie Inc, North Chicago, USA; ^6^Texas Movement Disorder Specialists, Georgetown, USA

**Background and Aims:** As Parkinson's disease (PD) progresses, patients experience motor fluctuations at varying rates. Foslevodopa/foscarbidopa (LDp/CDp) is a soluble formulation of levodopa/carbidopa (LD/CD) prodrugs, administered as a 24‐hour/day continuous subcutaneous infusion. In randomised controlled and open‐label phase 3 trials, LDp/CDp demonstrated sustained “Off” time reductions. Given the heterogeneous nature and progression of PD, treatment responses also vary among patients. This analysis aimed to explore the baseline characteristics of patients who achieved “Off” time responses, including up to 100% improvement.

**Methods:** This post hoc analysis utilized cluster analysis to examine pooled patient demographics and baseline disease characteristics from two phase 3 trials: the 12‐week active‐controlled trial comparing LDp/CDp vs oral LD/CD (NCT04380142) and the 52‐week open‐label trial assessing LDp/CDp safety/efficacy (NCT03781167). Patients with non‐missing “Off” time data at baseline and Week 12 or Week 13 in their Hauser diaries (active‐controlled and open‐label trials, respectively) were grouped into clusters based on “Off” time improvement ranging from 0%–100%.

**Results:** Cluster analysis in the baseline population of 158 patients showed the largest cluster corresponded to those with 100% improvement (27% of patients; Table 1). Across clusters, most patients were aged > = 60–80 years with <1800 mg levodopa equivalent daily dose. The 100% improvement cluster was characterised by patients with <10 years PD duration and <5 hours/day “Off” time.

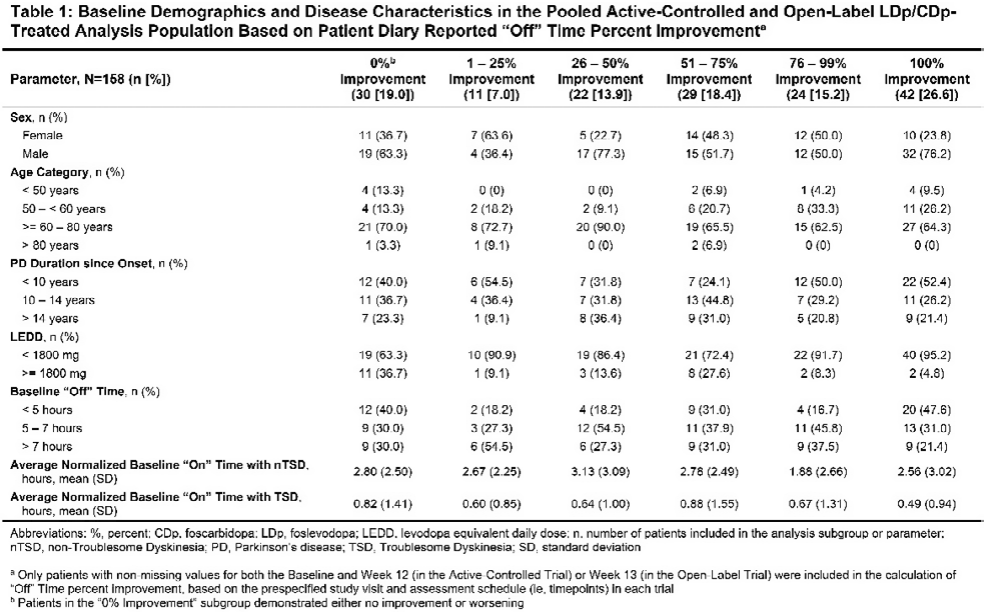

**Conclusion:** Cluster analysis identified patient profiles with 100% “Off” time improvement, characterised by shorter PD duration and less baseline “Off” time. These results highlight the potential for personalised approaches to optimise outcomes with LDp/CDp continuous infusion therapy in PD.

**Disclosure:** AbbVie Inc supported this analysis. Current/last 2 years financial disclosures: KD received advisor/consulting &/or speaker fees from AbbVie Inc, Acadia, Allergan, Amneal, Ipsen, Merz, Neurocrine, Sunovian, Supernus, & Teva. RP is an AbbVie Inc investigator & has received consulting &/or research support from Abbott, AbbVie Inc, ACADIA, Acorda, Adamas, Addex, Amneal, Biogen, Biohaven, Boston Scientific, CalaHealth, EIP, Global Kinetics, Impax, Impel, Intec, Kyowa, Lilly, Lundbeck, Mitsubishi, Neuraly, Neurocrine, Neuroderm, Neuropharma, Orbis, Parkinson's Foundation, Pharma 2B, PhotoPharmics, Prilenia, Roche, SIS, Sun Pharma, Sunovion, Teva, Theranexus, Theravance, US WorldMeds, & Voyager. BB has received advisor/speaker fees &/or grants from AbbVie Inc, EG, Ipsen, Merz, & Zambon. PO received: advisor/consultant &/or speaker fees from AbbVie Inc, Bial, Britannia, EVER Pharma, Nordic Infucare, STADA, & Zambon; &, PO's institution received research support from AbbVie Inc, Lund University Medical Faculty, Parkinsonfonden, SFO Multipark, Swedish Parkinson Academy, Swedish Research Council, & Health Care Region Skåne. MS, LB, JH, CHY, & RG are fulltime employees of AbbVie Inc & may hold AbbVie Inc stock/stock options. MJS received advisor/consulting/speaker fees &/or research/grant support from Abbott, AbbVie Inc, Amneal, Cerevel, CND Lifesciences, the HDSA, Jazz, Kyowa, Merz, Medtronic, Neurocrine, Praxis, Scion, Supernus, & Teva.

## EPO‐426

### Hospitalisation rates of people with Parkinson's disease in Austrian districts are associated with agricultural exposure

#### A. Santer ^1^; G. Fülöp^2^; A. Langer^3^; M. Ludwig^3^; H. Cetin^3^; F. Zimprich^3^; C. Brücke^3^; H. Zach^3^

##### ^1^Department of Neurology, Klinik Ottakring, Vienna, Austria; ^2^Gesundheit Oesterreich GmbH (GOEG), Vienna, Austria; ^3^Department of Neurology, Medical University of Vienna, Vienna, Austria

**Background and Aims:** Occupational exposure to pesticides is a known risk factor for Parkinson's disease. Previous studies have suggested that working in agriculture or living in rural areas may increase the risk of Parkinson's disease due to pesticide exposure, but the evidence remains inconsistent. This study explores whether there is an association between the hospitalization of people with Parkinson's disease and the proportion of individuals employed in agriculture and forestry across Austrian districts.

**Methods:** We acquired data on hospitalization rates of people with a main or secondary diagnosis of Parkinson's disease (ICD 10 G20‐G22) between 2015 and 2023 from the national hospital discharge register of the Gesundheit Oesterreich GmbH. The agricultural index, which indicates the proportion of the working population employed in agriculture and forestry in the respective districts, was obtained from Statistik Austria. We performed regression analysis to estimate whether hospitalisation rates could be predicted by the agricultural index in Austrian districts.

**Results:** We found that the cumulative agricultural index for the years 1981, 1991 and 2001 significantly predicted the rate of hospitalized patients with a main and secondary diagnosis of Parkinson's disease (ICD 10 G20‐G22) in the years 2015–2023 (*p* = 0.01).

**Conclusion:** The results indicate that the globally observed association between Parkinson's disease cases and agricultural employment may also be reflected within Austrian districts. Furthermore, the presented results suggest that the effects of agricultural by‐products may impact not only those employed directly in agriculture but also residents of rural areas with generally high agricultural employment rates.

**Disclosure:** Nothing to disclose.

## EPO‐427

### N‐acetyl‐L‐leucine for ataxia‐telangiectasia: A multinational double‐blind randomized placebo‐controlled crossover study

#### M. Strupp ^1^; M. Patterson^2^; J. Raymond^2^; B. Zanrucha^2^; A. Hatcher^2^; T. Fields^2^; T. Bremova‐Ertl^3^; K. Martakis^4^

##### ^1^Department of Neurology, Hospital of the Ludwig Maximilians University, Munich, Germany; ^2^IntraBio; ^3^Department of Neurology and Center for Rare Diseases, University Hospital Inselspital Bern, Switzerland; ^4^Department of Neuropediatrics, Justus Liebig University, Giessen, Germany

**Background and Aims:** Ataxia‐telangiectasia (A‐T) is a rare autosomal‐recessive cerebellar ataxia. The modified amino acid N‐acetyl‐L‐leucine has been associated with positive symptomatic and neuroprotective, disease‐modifying effects in observational clinical case studies and multinational clinical trials for various cerebellar ataxias and related lysosomal diseases. Most recently, N‐acetyl‐L‐leucine (NALL) demonstrated a significant improvement in neurological manifestations in Niemann‐Pick disease type C (NPC) in a Phase III double‐blind, randomized, placebo‐controlled crossover study (Study Code IB1001‐301). Here, we describe the implementation of this master study protocol for the treatment of adult and pediatric patients with A‐T (Sponsor Code IB1001‐303).

**Methods:** The IB1001‐303 protocol will enroll patients with a genetically confirmed diagnosis of A‐T patients aged 4 years and older across 11 trial sites. Patients are assessed during a baseline period and then randomized (1:1) to one of two treatment sequences: IB1001 followed by placebo or vice versa. Each sequence consists of a 12‐week treatment period.

**Results:** The primary efficacy endpoint is based on the Scale for the Assessment and Rating of Ataxia, and secondary outcomes include cerebellar functional rating scales, clinical global impression, and quality of life assessments.

**Conclusion:** The IB1001‐301 clinical trial served as the basis for the FDA approval of IB1001 (AQNEURSA) for the treatment of NPC. Utilizing the same master protocol, the IB1001‐303 placebo‐controlled cross‐over trial will evaluate the risk/benefit profile of IB1001 for A‐T to give information about the applicability of IB1001 as a therapeutic paradigm for other rare neurological disorders and potential label expansion of IB1001 for patients with A‐T.

**Disclosure:** Michael Strupp received speaker's honoraria from Abbott, Actelion, Auris Medical, Biogen, Eisai, Grünenthal, GSK, Henning Pharma, Interacoustics, MSD, Otometrics, Pierre‐Fabre, TEVA, UCB, Viatris. Consultant for Abbott, Actelion, AurisMedical, Decibel, Heel, IntraBio and Sensorion. Shareholder of IntraBio. Marc Patterson, Janelle Raymond, Bethany Zanrucha, and Asante Hatcher are employees of IntraBio. Tatiana Bremova‐Ertl received speaker's honoraria and consultancy fees from Actelion, Sanofi‐Genzyme and Zevra as well as blinded video‐rater fees from Intrabio.

## EPO‐428

### Current perspectives for wearable devices for Parkinson's disease: A systematic review

#### M. Valverde^1^; C. Moura^2^; L. Carretta^3^; M. Han ^4^; M. Nogueira^5^; M. Maximiano^2^; R. Silva^6^; M. Fernandes^7^; V. Mendonça^8^; M. Capitanio^9^; B. Pessoa^2^

##### ^1^Faculty of Medical and Health Sciences – FCMS/JF, Brazil; ^2^Fluminense Federal University, Brazil; ^3^Higher School of Sciences of Santa Casa de Misericórdia de Vitória (EMESCAM), Brazil; ^4^Faculty of Medicine, University of São Paulo, São Paulo, Brazil; ^5^University of Fortaleza, Fortaleza, Brazil; ^6^Federal University of Paraíba (UFPB), Brazil; ^7^Anhembi Morumbi University, São José dos Campos, Sao Paulo, Brazil; ^8^Bascom Palmer Eye Institute, Miami, USA; ^9^Community University of the Chapecó Region, Brazil

**Background and Aims:** Wearable devices have emerged as valuable tools for monitoring and managing symptoms of Parkinson's disease (PD). These technologies offer objective data to assess motor symptoms such as tremor, bradykinesia, and gait abnormalities. This systematic review aims to summarize the wearable devices currently available for PD management, and overview their prevalence and applications in clinical practice.

**Methods:** We conducted a systematic search of PubMed, Embase, and Cochrane Library databases to identify relevant studies. Initial screening included 3660 articles, of which 178 were selected based on inclusion criterea. Further evaluation 43 excluded studies that did not involve patient data or solely described the technology without clinical application.

**Results:** In total, 5,250 patients were included. The most commonly used devices were (e.g., accelerometer‐based sensors, gyroscopes, or smartwatches), primarily employed for continuous monitoring of gait and tremor. Most studies evaluated Gait abnormalities (51/135) 37%, followed by tremor (27/135) 20%, dyskinesia (17/135) 12%, and bradykinesia (16/135) 11%. These devices demonstrated significant potential for improving symptom tracking and treatment personalization. However, challenges such as device usability, cost, and integration into routine clinical workflows remain barriers to widespread adoption.

**Conclusion:** Wearable devices offer a promising avenue for enhancing the management of PD by providing objective, real‐time data on motor symptoms. Neurologists should familiarize themselves with these tools to integrate them into clinical practice, ultimately benefiting patient care and improving outcomes. Further research is warranted to standardize device use and optimize their utility in PD management.

**Disclosure:** Nothing to disclose.

## EPO‐429

### Deep brain stimulation in the mesencephalic locomotor region of the rat induces behaviours beyond just locomotion

#### M. Wilhelm; J. Hartig; T. Faupel; D. Sun; F. Hakimi; A. Sodmann; F. Schlott; S. Knorr; C. Ip; F. Fluri; J. Volkmann; M. Schuhmann; R. Blum

##### Department of Neurology, University Hospital Würzburg, Würzburg, Germany

**Background and Aims:** Deep brain stimulation (DBS) in the cuneiform nucleus (CnF), a subregion of the mesencephalic locomotor region (MLR), has been suggested for treating gait disturbances in severe Parkinson's disease (PD). However, recent studies observed no significant clinical effect of CnF‐DBS, but instead reported increased anxiety. This raised the question about neurobiological connections of the CnF to motor centres and neural circuits for fear and anxiety.

**Methods:** Here, we applied DBS to the CnF of rats and analysed acute DBS‐induced behaviour in an inversed open field. Unbiased behavioural analysis was conducted with DeepLabCut. Neural activity mapping was performed after DBS by quantifying de novo expression of the activity marker cFOS. Finally, we developed viral fibre tracers expressing plasma membrane‐bound green fluorescent protein (GFP) in glutamatergic neurons to visualize the ascending input and output matrix of the CnF area.

**Results:** DBS in the CnF induced acute behaviour at frequencies of 80‐130 Hz but not at 20‐60 Hz. DBS initiated gait within a second, but also induced subsequent periods of different behavioural states like running, tail rattling, rearing, and freezing. Neurons in the stimulated area expressed cFOS within 90 minutes. Fibre mapping of CnF neurons identified ascending projections to the substantia nigra, the subthalamic nucleus, different thalamic areas and the central amygdala.

**Figure:**
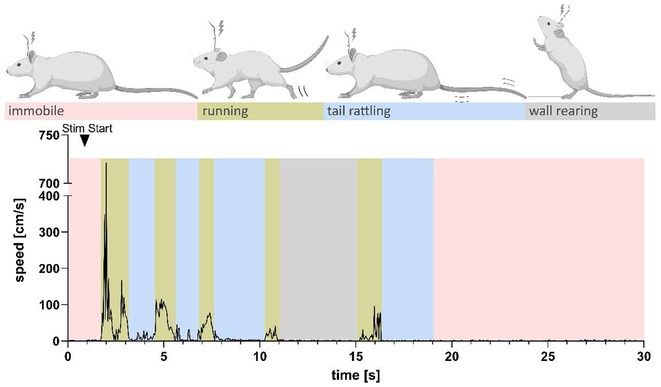
**FIGURE 1** Categorization of acute behaviour after HF‐DBS in the CnF.

**Figure:**
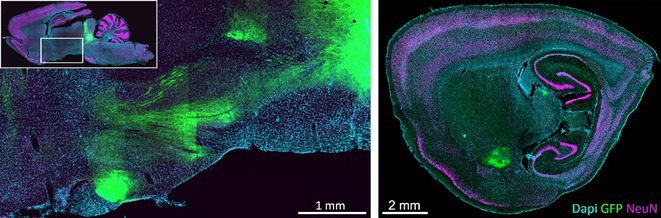
**FIGURE 2** Anterograde tracing visualizes ascending projections to higher order motor centres and brain areas involved in defensive behaviours.

**Conclusion:** Our study suggests that the CnF in the MLR integrates behaviours beyond just locomotion by ascending, glutamatergic projections to higher order motor centres and brain areas involved in defensive behaviours.

**Disclosure:** This project was funded by the Deutsche Forschungsgemeinschaft (DFG, German Research Foundation) Project‐ID424778381‐TRR 295. The authors declare no conflict of interest.

## EPO‐430

### Predictors of cognitive impairment and gait disorder after subthalamic deep brain stimulation in Parkinson's disease

#### O. Alenikova ^1^; A. Buniak^1^; E. Mikitchuk^2^; M. Dymkovskaya^1^; N. Alenikov^1^

##### ^1^Republican Research and Clinical Center of Neurology and Neurosurgery, Minsk, Belarus; ^2^Belarusian State University, Minsk, Belarus

**Background and Aims:** Cognitive impairment and gait disturbances are the most common complications after subthalamic nucleus stimulation (STN‐DBS) surgery, and the question of predisposing factors to these symptoms progression during STN‐DBS remains controversial.We aimed to determine the predictors of cognitive decline and gait disorder after STN‐DBS in PD.

**Methods:** A total of 22 PDpatients aged 45‐60 years were examined. MRI voxel‐based morphometry and tractography was applied before surgery. Neuropsychological testing was performed to identify frontal‐striatal, posterior cortical, and mixed subtypes of mild cognitive impairment (MCI) preoperatively and to assess cognitive function over the next 3–5 years after STN‐DBS surgery in PDpatients. The FOGQ assesses freezing of gait severity was used.

**Results:** In a retrospective analysis, PDpatients with preoperative frontostriatal or mixed MCI had greater cognitive decline in subsequent years compared with patients with posterior cortical MCI. These patients, along with a decrease in the density of the tracts connecting the frontal cortex with the caudate nucleus and putamen, had a more pronounced decrease in the volume of the orbitofrontal and ventromedial prefrontal cortex. The frontostriatal and/or mixed subtype MCI was also detected in patients who developed gait disturbances. A correlation was determined between the FOGQscore and the cortical areas volume related to the dorsal and ventral visual information processing system (p<0.03), as well as the frontal cortical areas (*p* < 0.02) responsible for planning and programming movements.

**Conclusion:** Thus, to select candidates for STN‐DBS surgery, it is necessary to take into account MRI morphometry and tractography data, as well as MCI subtypes prior to surgery.

**Disclosure:** Nothing to disclose.

## EPO‐431

### Genetically determined dystonic syndromes associated with elevated alpha‐fetoprotein

#### P. Havránková ^1^; L. Kunc^1^; J. Roth^1^; J. Rajmonová^1^; J. Necpál^2^; M. Skorvanek^3^; O. Ulmanová^1^; E. Tsoma^4^; M. Zech^5^; R. Jech^1^

##### ^1^Department of Neurology, 1st Faculty of Medicine and General University Hospital in Prague, Charles University, Prague, Czechia; ^2^Department of Neurology, Zvolen Hospital, Zvolen, Slovakia; ^3^Department of Neurology and Center for Rare Movement Disorders, Faculty of Medicine, P. J. Safarik University and University Hospital L. Pasteur, Kosice, Slovakia; ^4^Regional Clinical Centre of Neurosurgery and Neurology, Uzhhorod, Ukraine; ^5^Institute of Neurogenomics, Helmholtz Zentrum München, Munich, Germany

**Background and Aims:** Alpha‐fetoprotein (AFP) is a liver‐produced glycoprotein with an unknown function. Elevated levels are linked to autosomal recessive cerebellar ataxias, where atypical forms may primarily present with dystonia. The most common example is ataxia telangiectasia (AT) caused by ATM gene mutation.

**Methods:** We aimed to identify patients with dystonia and pathogenic mutations linked to AFP elevation and evaluate whether AFP could serve as a suitable biomarker for dystonia patients.

**Results:** The study included 669 patients diagnosed with dystonic syndromes who underwent whole‐exome sequencing. Pathogenic mutations associated with elevated AFP levels were identified in nine patients (five men). Among these were six cases of ATM gene mutations, two cases of SETX gene mutations, and one of an NGLY1 mutation. Dystonia was the only or dominant symptom in five patients, while ataxia was present in four patients. AFP‐level testing was performed in eight of nine patients with the pathogenic mutation, revealing increased levels in seven patients. In one patient with an ATM mutation, the AFP plasma level was within the normal range on repeated measurements, which is rare, but possible even with a diagnosis of ataxia telangiectasia.

**Conclusion:** We found a pathogenic mutation linked to AFP elevation in over 1 % of the patients, which we believe is significant enough to support the use of AFP as a biomarker for early‐onset dystonia. Supported by the grant: AZV Czech Republic: NW24‐04‐00067 and EU programme EXCELES: LX22NPO5107.

**Disclosure:** Nothing to disclose.

## EPO‐432

### Systematic review and meta‐analysis of neutralising antibodies to botulinum neurotoxin type A in multiple indications

#### U. Walter^1^; U. Walter^2^; P. Albrecht ^3^; P. Albrecht^4^; W. Carr^5^; H. Hefter^3^

##### ^1^Department of Neurology, Rostock University Medical Centre, Rostock, Germany; ^2^Deutsches Zentrum für Neurodegenerative Erkrankungen (DZNE) Rostock/Greifswald, Rostock, Germany; ^3^Department of Neurology, Medical Faculty and University Hospital, Heinrich‐Heine‐University Düsseldorf, Düsseldorf, Germany; ^4^Department of Neurology, Maria Hilf Clinics Mönchengladbach, Mönchengladbach, Germany; ^5^Allergy and Asthma Associates of Southern California, Southern California Research, Mission Viejo, USA

**Background and Aims:** Botulinum neurotoxin type A (BoNT‐A) is a biological treatment for cervical dystonia (CD), spasticity, and blepharospasm. Its biological nature means some patients can develop anti‐BoNT‐A neutralising antibodies (NAbs), which may reduce efficacy and, in some cases, cause secondary treatment failure. This meta‐analysis investigated the incidence of NAb‐positivity after treatment with commercially‐available BoNT‐A formulations.

**Methods:** A systematic review was conducted in April 2024, and meta‐analysis was performed of publications reporting data on immunogenicity after first‐line treatment with abobotulinumtoxinA, incobotulinumtoxinA or onabotulinumtoxinA (excluding original formulation) in patients with CD, spasticity or blepharospasm

**Results:** In total, 29 publications reporting 28 unique studies were identified. The proportion of patients with CD developing NAbs was significantly higher after treatment with abobotulinumtoxinA (2.1%; *p* = 0.02) versus incobotulinumtoxinA (0%). There was no difference in NAb positivity after onabotulinumtoxinA (1.5%) versus abobotulinumtoxinA (*p* = 0.72) or incobotulinumtoxinA (*p* = 0.07). The proportion of patients with spasticity developing NAbs after onabotulinumtoxinA (1.2%; *p* = 0.01) was significantly higher than with incobotulinumtoxinA (0%); there was no difference between abobotulinumtoxinA (0.5%) versus onabotulinumtoxinA (*p* = 0.84) or incobotulinumtoxinA (*p* = 0.12). Based on only three identified studies, the proportion of patients with blepharospasm developing NAbs was significantly higher after abobotulinumtoxinA (16.7%) than onabotulinumtoxinA (0%; *p* < 0.001) or incobotulinumtoxinA (0%; *p* = 0.002).

**Conclusion:** No patients exclusively treated with incobotulinumtoxinA developed persistent NAbs, supporting the low antigenicity of this BoNT‐A formulation. Clinicians must consider the potential effects of cumulative exposure to different BoNT‐A formulations for different indications. We recommend incobotulinumtoxinA to avoid developing immunogenicity, particularly for patients requiring higher doses and repeated treatments.

**Disclosure:** This study was funded by Merz Therapeutics GmbH.

## EPO‐433

### Secondary treatment failure with botulinum neurotoxin type A: A systematic review and meta‐analysis

#### U. Walter^1^; U. Walter^2^; P. Albrecht ^3^; P. Albrecht^4^; W. Carr^5^; H. Hefter^3^

##### ^1^Department of Neurology, Rostock University Medical Centre, Rostock, Germany; ^2^Deutsches Zentrum für Neurodegenerative Erkrankungen (DZNE) Rostock/Greifswald, Rostock, Germany; ^3^Department of Neurology, Medical Faculty and University Hospital, Heinrich‐Heine‐University Düsseldorf, Düsseldorf, Germany; ^4^Department of Neurology, Maria Hilf Clinics Mönchengladbach, Mönchengladbach, Germany; ^5^Allergy and Asthma Associates of Southern California, Southern California Research, Mission Viejo, USA

**Background and Aims:** Botulinum neurotoxin type A (BoNT‐A) treatment is recommended for cervical dystonia (CD) and spasticity. Since it is a biological therapy, some patients can develop anti‐BoNT‐A neutralising antibodies (NAbs). NAbs reduce efficacy and, in some cases, cause secondary treatment failure (STF). This meta‐analysis aimed to investigate the incidence of STF after treatment with commercially‐available BoNT‐A formulations for which long‐term data are available.

**Methods:** A systematic review identified publications reporting NAb‐induced STF after first‐line treatment with abobotulinumtoxinA, incobotulinumtoxinA or onabotulinumtoxinA (excluding original formulation) in patients with CD or spasticity. STF occurrence was analysed in a meta‐analysis using the DerSimonian‐Laird random‐effects method with Freeman‐Tukey double arcsine transformation. Differences in STF occurrence between the three formulations were evaluated through heterogeneity testing.

**Results:** In total, 18 studies assessed STF in CD or spasticity. Meta‐analysis showed that the proportions of patients with CD developing STF were significantly higher after treatment with abobotulinumtoxinA (9%; *p* < 0.001) or onabotulinumtoxinA (3%; *p* = 0.03) than with incobotulinumtoxinA (0%). There was no difference between the proportions of patients developing STF after abobotulinumtoxinA versus onabotulinumtoxinA (*p* = 0.08). The proportion of patients with spasticity developing STF after treatment with abobotulinumtoxinA (5%; *p* = 0.01) was also significantly higher than with incobotulinumtoxinA (0%). No patients treated exclusively with incobotulinumtoxinA developed NAb‐induced STF, irrespective of the treatment indication.

**Conclusion:** IncobotulinumtoxinA was associated with a significantly lower risk of developing STF compared with abobotulinumtoxinA (CD and spasticity) and onabotulinumtoxinA (CD). IncobotulinumtoxinA is recommended to avoid developing NAb‐induced STF, particularly for patients who require higher doses and repeated treatments.

**Disclosure:** This study was funded by Merz Therapeutics GmbH.

## EPO‐434

### Six‐month comparison of DBS‐STN, LCIG, CSCI and LECIG in advanced Parkinson's disease patients

#### M. Hero; V. Rački; N. Grozdanić; V. Vuletić

##### Clinic of Neurology, Clinical Hospital Center Rijeka, Rijeka, Croatia

**Background and Aims:** Parkinson's disease (PD) is a progressive neurodegenerative disorder characterized by motor and non‐motor symptoms. In advanced stages, individualized treatment strategies are needed to manage motor fluctuations and dyskinesias. This study compares the efficacy of deep brain stimulation of the subthalamic nucleus (DBS‐STN), levodopa/carbidopa intestinal gel (LCIG), levodopa/entacapone/carbidopa intestinal gel (LECIG), and continuous subcutaneous infusion (CSCI) with foslevodopa/foscarbidopa in advanced PD over six months.

**Methods:** We analyzed 51 PD patients treated at the Clinic of Neurology, Clinical Hospital Center Rijeka, between 2022 and 2024. Patients were assessed at baseline and after six months, using Unified Parkinson's Disease Rating Scale (UPDRS) part III and IV scores.

**Results:** After six months, DBS‐STN showed the highest efficacy, with a 51% improvement in UPDRS III and 53% in UPDRS IV scores. CSCI and LCIG demonstrated 30% improvement in both UPDRS III and IV scores, while LECIG improved UPDRS III by 29% and UPDRS IV by 22%.

**Conclusion:** These findings highlight DBS‐STN as the most effective option for managing motor symptoms and complications in advanced PD. While CSCI, LCIG, and LECIG offer viable alternatives, their efficacy is lower, particularly in motor complication management. CSCI, being the least invasive, is an important option influencing patient decisions. DBS should be prioritized for eligible patients, with subcutaneous and intestinal gel therapies reserved for those unsuitable for surgery. This study continues prospectively to further evaluate long‐term treatment outcomes.

**Disclosure:** Nothing to disclose.

## Movement disorders 5

## EPO‐435

### Genetic control of enzymatic reactive oxygen species formation may influence idiopathic Parkinson's disease progression

#### A. Törnell; D. von Below; H. Nissbrandt; F. Bergquist; K. Hellstrand; A. Martner

##### Departments of Microbiology and Immunology, Infectious Diseases, Neurology and Pharmacology, Sahlgrenska Academy, University of Gothenburg, Gothenburg, Sweden

**Background and Aims:** The NOX2 enzyme is highly expressed by microglia and generates reactive oxygen species (ROS) to eliminate pathogens. While excessive NOX2 activation has been linked to neurodegenerative diseases, the role of NOX2 in Parkinson's disease (PD) is not fully understood. We aimed to determine the impact of variation in genes encoding NOX2 activity on long‐term progression in patients with idiopathic PD. Single nucleotide polymorphisms (SNPs) at rs4673 and rs1049254 in CYBA, encoding the NOX2 subunit p22phox, influence NOX2 activity and impact on progression in multiple sclerosis and recovery from Guillain‐Barré syndrome.

**Methods:** We genotyped 93 patients with idiopathic PD for these SNPs. Blinded investigators reviewed patient records to determine time from motor onset to the occurrence of 25 disease progression milestones. The impact of CYBA SNP genotypes on PD progression was assessed using individual milestones and a composite measure of all milestones. Data were censored when 50% of events within each milestone had occurred to reduce the contribution of normal aging.

**Figure:**
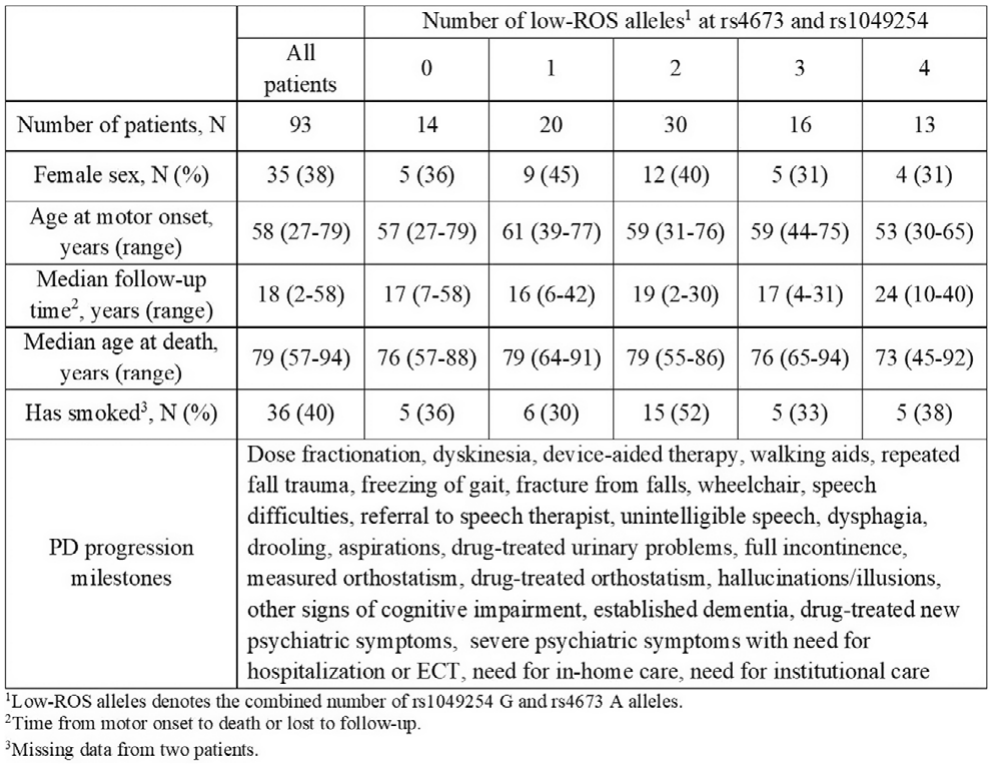
**TABLE 1** Patient characteristics.

**Results:** Patients carrying low‐ROS genotypes showed a delayed onset of several individual milestones and a reduced accumulation of clinical milestones over time (Figure 1‐2). These effects on milestone accumulation remained significant after adjusting for potential confounders (*p* = 0.006 for rs1049254 and *p* = 0.001 for low‐ROS alleles). Ten years after motor onset, patients with a homozygous high‐ROS genotype had acquired nearly three times as many clinical milestones as patients with a low‐ROS genotype.

**Figure:**
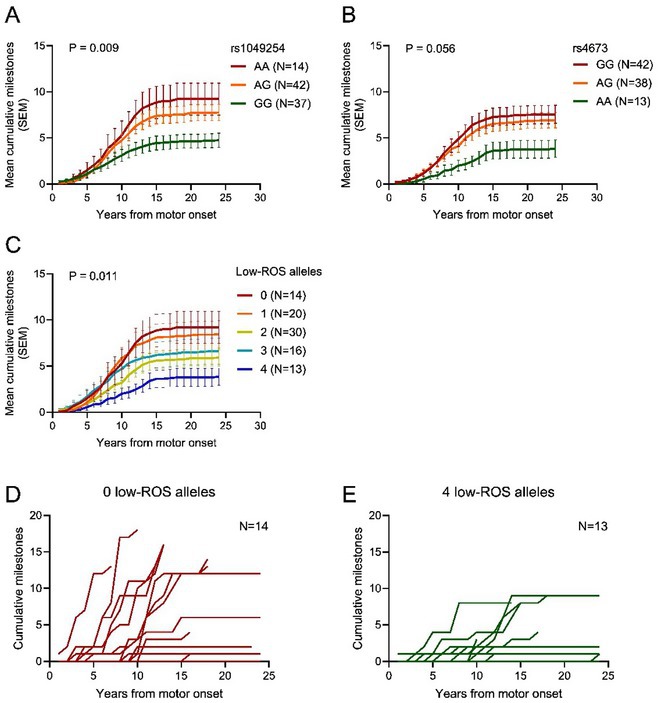
**FIGURE 1** (A–C) Mean cumulative milestones within each genotype group. (D, E) Cumulated events for individual patients (lines) with (D) 0 or (E) 4 low‐ROS alleles. (C–E) Combined rs1049254 G and rs4673 A alleles. Statistics by linear mixed‐effects model.

**Figure:**
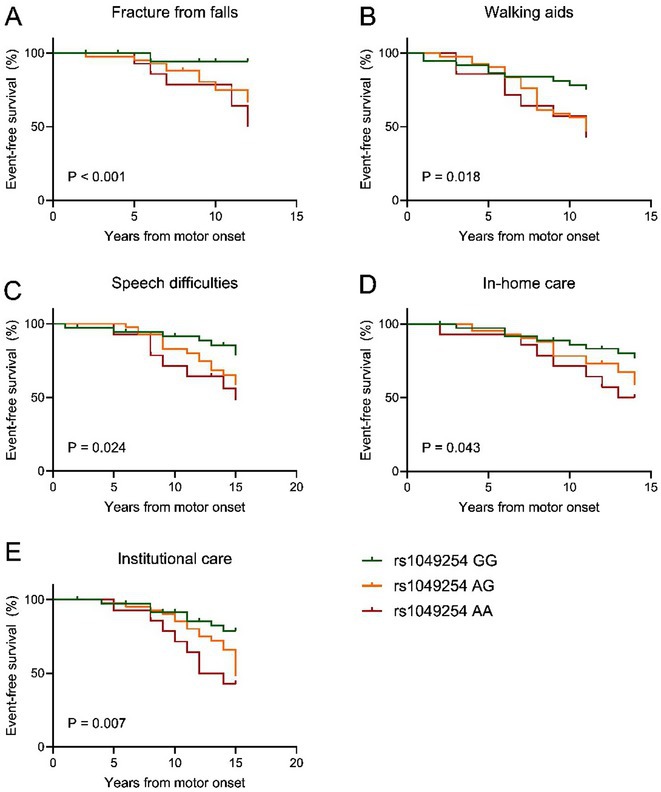
**FIGURE 2** Time from PD onset to reach the milestones (A) fracture from falls, (B) need for walking aids, (C) speech difficulties, (D) in‐home care or (E) institutional care. Patients grouped by rs1049254 genotype. Statistics by log‐rank test for trend.

**Conclusion:** These results suggest a role for NOX2 in PD progression and highlight the potential of pharmacologically targeting NOX2 to reduce neurodegeneration.

**Disclosure:** Nothing to disclose.

## EPO‐436

### Motor symptoms and stigma in Parkinson's disease

#### A. Sauerbier ^1^; V. Stopic^1^; S. Jost^1^; J. Haupt^1^; G. Fink^2^; H. Dafsari^1^; D. Gruber^3^; G. Ebersbach^3^; J. Kessler^1^; M. Barbe^1^

##### ^1^University of Cologne, Faculty of Medicine and University Hospital Cologne, Department of Neurology, Cologne, Germany; ^2^University of Cologne, Faculty of Medicine and University Hospital Cologne, Department of Neurology, Cologne, Germany and Cognitive Neuroscience, Institute of Neuroscience and Medicine (INM‐3), Research Centre Jülich, Jülich, Germany; ^3^Movement Disorders Hospital, Kliniken Beelitz GmbH, Beelitz‐Heilstätten, Germany

**Background and Aims:** Different motor symptoms have been described as being stigmatizing by Parkinson's disease (PD) patients. However, to date, findings are mainly based on qualitative research. Our aim was to address the association of motor symptoms and stigma by using the newly validated Parkinson's Disease Stigma Questionnaire (PDStigmaQuest).

**Methods:** This is a multi‐center prospective study. We compared felt stigma domain scores to other PDStigmaQuest domain scores using Wilcoxon signed‐rank tests. PDStigmaQuest domain scores and the total score were compared between early PD patients (disease duration ≤3 years) and other patients using Mann‐Whitney *U* tests. Spearman correlations between PDStigmaQuest total score and the following clinical characteristics were calculated: Hoehn and Yahr (HY), MDS‐UPDRS II, III, and IV, and PDQ‐39 SI.

**Results:** In total, 201 PD patients (34.3% female, mean age 64.4, median HY 2.0) were included. Felt stigma scores were higher than hiding (*p* < 0.001), rejection (*p* < 0.001), and patronization scores (*p*< 0.001). Early PD patients showed significantly higher scores in the hiding domain (*p* = 0.035) and substantially lower rejection domain scores than other PD patients (*p* = 0.004). PDStigmaQuest total score correlated significantly with the following clinical characteristics: HY (*r* = 0.15, *p* = 0.033), MDS‐UPDRS II activities of daily living (*r* = 0.29, *p* < 0.001), MDS‐UPDRS IV dyskinesia score (*r* = 0.23, *p* = 0.008), and PDQ‐39 SI (*r* = 0.61, *p* < 0.001).

**Conclusion:** These data provide evidence that stigma is a critical aspect for quality of life in PD. In particular, dyskinesia and impairment of ADLs were associated with stigma. This ongoing study is essential for a deeper understanding of stigma in PD and its management in the future.

**Disclosure:** Nothing to disclose.

## EPO‐437

### Subacute onset progressive gait disorder with recurrent falls: A case report

#### G. Hemicker; C. Cerejo; P. Ellmerer; F. Leys; E. Holzknecht; P. Mahlknecht; F. Krismer; B. Heim

##### Department of Neurology, Medical University of Innsbruck, Innsbruck, Austria

**Background and Aims:** A 72‐year‐old male with a history of microcytic anemia, obstructive sleep apneas, arterial hypertension, hypercholesterolemia, and nocturia, presented with progressive gait imbalance over the past year, worsening significantly in the last four months.

**Methods:** The patient reported recurrent falls, primarily backward and sideways, marked slowness in daily activities, and occasional non‐formed visual hallucinations. Neurological examination revealed prominent antecollis, significant hypomimia, mild hypophonia, dysarthria without palilalia, bilateral grasp reflexes, and slowed saccades without ophthalmoplegia. Motor findings included rapid hypokinesia and rigidity, brisk reflexes, normal muscle strength, and absent tremors. Postural reflexes were significantly impaired, requiring a walker for ambulation. MDS‐UPDRS III score was 56, with Hoehn & Yahr stage 4.

**Results:** Cognitive testing showed an MMSE score of 25/30, with deficits in verbal memory retrieval, visual naming, and psychomotor speed. Polysomnography confirmed central and mixed OSA. Laboratory findings included microcytic hypochromic anemia and folate deficiency. Brain MRI demonstrated marked mesencephalic atrophy, whereas the DAT scan was unremarkable. CSF analysis, autoimmune panels, and onconeural antibodies were negative; however, IgLON5 antibodies were detected in both serum and CSF.

**Conclusion:** A diagnosis of Anti‐IgLON5 antibody disease presenting as an atypical parkinsonism of progressive supranuclear palsy (PSP) phenotype was established. Anti‐IgLON5 disease is a rare autoimmune encephalopathy that bridges neurodegeneration and autoimmunity, often linked with tau‐related pathology. Clinical features are diverse, encompassing parasomnias, cognitive and psychiatric symptoms, atypical parkinsonism with falls, bulbar dysfunction, and autonomic failure. This case underscores the importance of considering Anti‐IgLON5 disease in patients with atypical parkinsonism and diagnostic inconsistencies.

**Disclosure:** Nothing to disclose.

## EPO‐438

### When phenotype outweighs genotype: Deep brain stimulation in ATP1A3‐related dystonia

#### I. Alexandratou ^1^; E. Hainque^2^; C. Foucard^1^; E. Roze^2^; S. Navarro^3^; C. Ewenczyk^2^; E. Apartis^4^; M. Vidailhet^2^; C. Delorme^2^

##### ^1^Assistance Publique‐Hôpitaux de Paris, Service de Neurologie, Hôpital Pitié‐Salpêtrière, Paris, France; ^2^Sorbonne Université, Institut du Cerveau‐Paris Brain Institute‐ICM, Inserm, CNRS, AP‐HP, Institut de Neurologie, Hôpital Salpetriere, France; ^3^AP‐HP, Hôpitaux Universitaires La Pitié Salpêtrière‐Charles Foix, Service de Neurochirurgie, Paris, France; ^4^Inserm U1127, UMR S 1127, CNRS UMR 7225, Faculté de Médecine, Institut du Cerveau, Sorbonne Université, Paris, France; Département de Neurophysiologie DePAS, Hôpital Saint‐Antoine, AP‐HP, Paris, France

**Background and Aims:** ATP1A3‐related movement disorders are often severe, resistant to pharmacological treatments, and thus present a considerable therapeutic challenge. Historically, patients with ATP1A3 mutations have shown poor responses to deep brain stimulation (DBS) targeting the globus pallidus internus (GPi). Here, we describe a patient with an ATP1A3 mutation who achieved a remarkable therapeutic improvement with GPi DBS.

**Methods:** We evaluated a 24‐year‐old woman with a genetically confirmed ATP1A3 mutation (p.Arg756Leu), presenting with refractory myoclonic jerks and segmental dystonia, which significantly impaired her quality of life. She also had mild cerebellar ataxia but no intellectual impairment. Brain MRI was normal except for mild cerebellar atrophy. Polygraphic EMG recording showed subcortical myoclonus and mobile dystonia. Following a multidisciplinary evaluation, bilateral GPi DBS was implemented.

**Figure:**
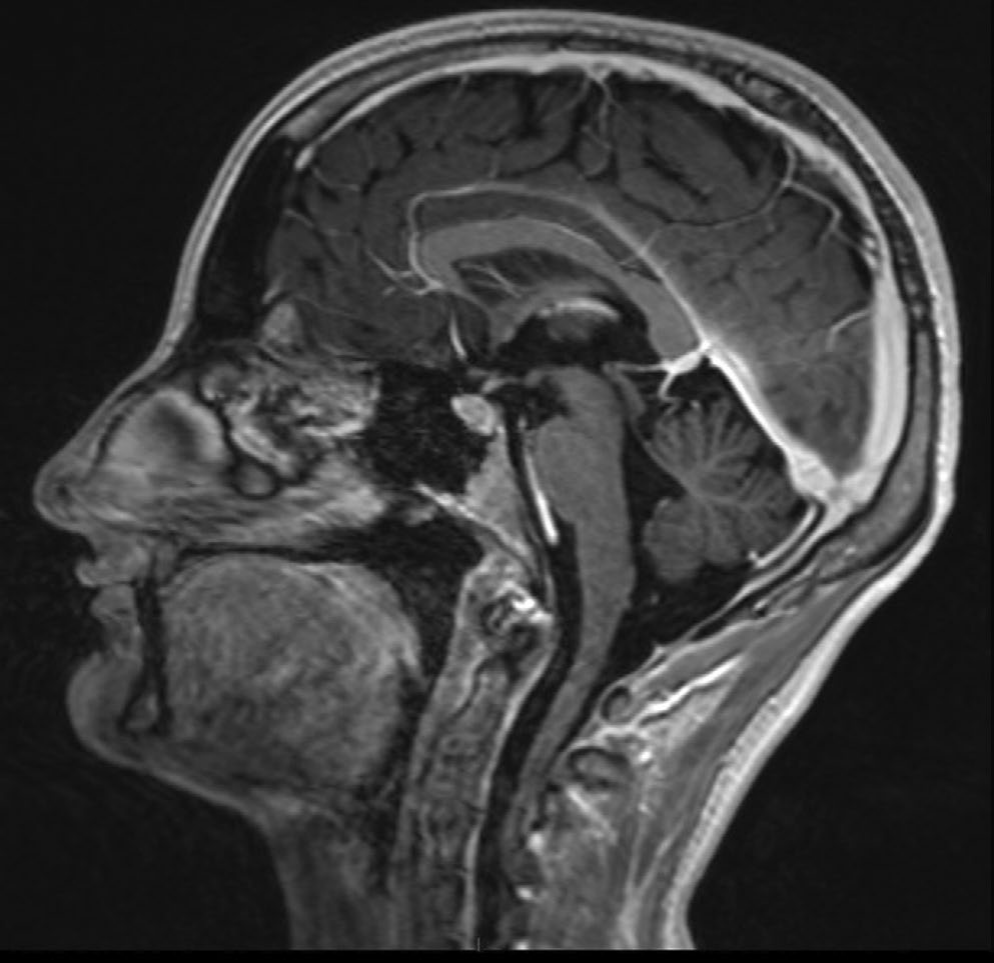
**FIGURE 1** Preoperative Brain MRI

**Figure:**
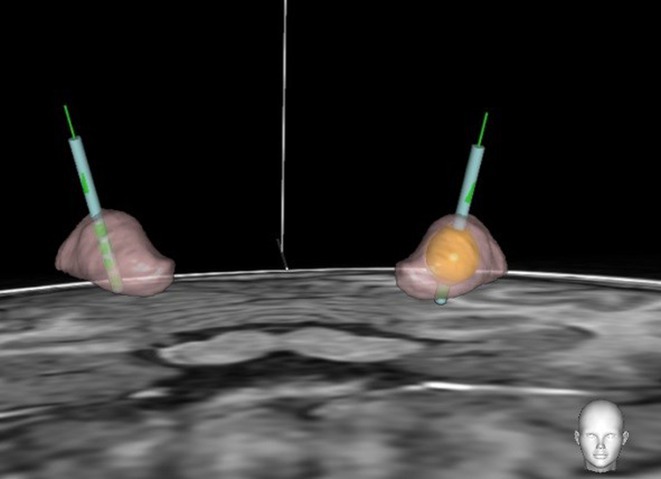
**FIGURE 2** VOLUME OF TISSUE ACTIVATED (VTA) LEFT

**Figure:**
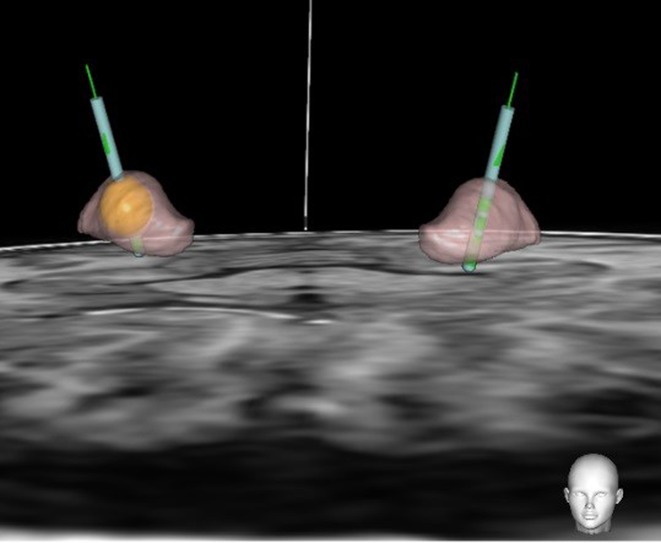
**FIGURE 3** VOLUME OF TISSUE ACTIVATED (VTA) RIGHT

**Results:** The patient exhibited a profound and sustained reduction in myoclonus and dystonic postures following DBS, with significant functional and motor improvement. At last follow‐up one year after surgery, clinical global impression improvement (CGI‐I) was rated 2/7 (much improved) by both the patient and clinicians. The patient did not have any side effects from DBS.

**Conclusion:** This case highlights two key insights. First, the clinical spectrum of ATP1A3 mutations extends beyond established syndromic classifications, necessitating careful phenotypic characterization for personalized management. Second, we emphasize the importance of considering phenotype first when evaluating a patient for GPi DBS, even when the genotype generally suggests a poor response. Patients with prominent myoclonus without fixed dystonia and without significant lesions on brain MRI may respond well to GPi DBS, regardless of the underlying etiology.

**Disclosure:** Nothing to disclosure.

## EPO‐439

### Staged bilateral MR‐guided focused ultrasound pallidothalamic tractotomy for Parkinson's disease

#### L. Pan; X. He; Z. Zeng; Y. Bai

##### Departments of Neurosurgery, PLA General Hospital, Beijing, China

**Background and Aims:** Since 2011, pallidothalamic tractotomy (PTT) has been employed as a therapeutic intervention for Parkinson's disease (PD) using magnetic resonance‐guided focused ultrasound (MRgFUS). We aimed to investigate the Safety and Effectiveness of Staged Bilateral PTT‐MRgFUS for PD.

**Methods:** Thirteen consecutive patients suffering from chronic (mean disease duration 9.0 years) and therapy‐resistant PD were treated unilaterally with PTT MRgFUS. Eleven received operation of the second side. The primary endpoints comprised Unified Parkinson's Disease Rating Scale (UPDRS) scores assessed during both on‐ and off‐medication states, along with adverse events recorded at baseline, 1 week, 1 month, 3 months, 6 months, and 12 months post‐treatment.

**Results:** The mean duration between baseline UPDRS score and 1 year after the second side was 13.5 months. The UPDRS Part III score off‐medication at 1 year after the first PTT was reduced by 37% (*p* = 0.0002) compared to that at baseline on‐medication and 16% after the second PTT (*p* = 0.02). Percentage reductions of the mean scores comparing 1 year off‐ with baseline on‐medication examinations were 83% for tremor, 63% for rigidity, and 57% for hypobradykinesia. Adverse events such as hypophonia (29%) and fatigue (29%) were mild and improved in post‐treatment 3 months.

**Conclusion:** Our results suggest MRgFUS PTT was a safe and effective intervention for PD patients, in varying symptoms.Additional large‐scale studies and long‐term outcomes evaluation are needed.

**Disclosure:** Nothing to disclose.

## EPO‐440

### Aperiodic spectral component as a potential new marker and input signal for aDBS

#### L. Šmahovská ^2^; M. Bočková^1^; M. Lamoš^1^

##### ^1^Brain and Mind Research Program, Central European Institute of Technology, Masaryk University, Brno, Czechia; ^2^First Department of Neurology, Masaryk University School of Medicine, St. Anne's Hospital, Brno, Czechia

**Background and Aims:** New stimulation devices enable to record and analyze the local field potentials (LFPs) anytime, even during the deep brain stimulation therapy (DBS). The aim of our work was to study individual differences in LFPs, their evolution in time and to evaluate the influence of DBS on parameters of aperiodic spectral component.

**Methods:** LFP were recorded from the subthalamic nucleus (STN) in Parkinson´s disease patients (PD) (*n* = 23) during a simple experimental paradigm that included 5 minutes of resting state and a short gait during DBS “off” and “on” conditions (“off” medication). Classical spectral analysis was performed using Fast Fourier Transform (FFT). Progression in time could be already evaluated analyzing control measurement after one year (*n* = 15). Finally, the analysis of the aperiodic component was performed by fitting oscillations and one‐over‐F (FOOOF) approach.

**Results:** Typical beta power peaks, that represent a well‐known correlate of PD main motor symptoms could be detected in the majority of our group of patients. Moreover, the frequency slowing of beta peak was detected in some cases comparing baseline and control measurement. Aperiodic slope and offset were significantly modified by DBS (*p* = 0.0003 and *p* = 0.001) tested by Wilcoxon signed rank test.

**Conclusion:** Beta power sensing is a well‐established method in the DBS field, but has its limitations in the clinical practice. Evaluation of the aperiodic component in LFPs has the potential to better reflect the pathological activity of the neuronal network and might serve as a new clinical marker for adaptive DBS.

**Disclosure:** Nothing to disclose.

## EPO‐441

### Preliminary outcomes of MRgFUS thalamotomy or subthalamotomy for Parkinson's disease: A retrospective study

#### R. Garcia‐Ramos ^1^; A. Fernández^1^; C. Ribacoba^1^; E. Lopez Valdes^1^; C. Perez^3^; A. Lopez Frias^3^; A. Trondin^2^; M. Yus^3^

##### ^1^Movement Disorders Unit, Neurology Department, Clinico San Carlos Hospital, Madrid, Spain; ^2^Neurosurgery Department, Clinico San Carlos Hospital, Madrid, Spain; ^3^Radiology Department, Clínico San Carlos Hospital, Madrid, Spain

**Background and Aims:** Tremor‐dominant Parkinson's disease (TDPD) responds only partially to levodopa. Unilateral thalamotomy and subthalamotomy using magnetic resonance‐guided focused ultrasound (MRgFUS) have shown improvements in motor symptoms and it could be an alternative to deep brain stimulation (DBS). We present our initial experience of TDPD patients treated with MRgFUS.

**Methods:** This is a retrospective study (2023–2024) of patients TDPD treated with MRgFUS‐STN or MRgFUS‐VIM which were evaluated at 1 and 6 months post‐treatment. Change MDS‐UPDRS motor part III score for the treated hemibody and total MDS‐UPDRS III score. Treatment parameters were collected. All adverse events occurring during were documented. Data were analyzed with SPSS.

**Results:** Among 11 patients who underwent thalamotomy (mean age: 72.1 years; disease duration: 5.9 years), MDS‐UPDRS III improvement for the treated hemibody was 63.3%, and MDS‐UPDRS‐III total score improvement was 34.09%; 5 patients experienced side effects at one month, of which 1 was permanent. In 10 patients with subthalamotomy (mean age: 68.6 years; disease duration: 7.5 years), improvements were 38.6% for the treated hemibody and 23% overall MDS‐UPDRS‐III; 8 patients experienced side effects at one month, of which 2 were permanent. SDR mean was 0.50. The mean coordinates of the subthalamus are: ML 12 mm; AP 7.83 mm; SI −3.83 mm and for thalamotomy are: ML 14.71 mm; AP: 6.25 mm and SI 1mm. Mean of sonications in thalamus was 7.5 and 9.62 in subthalamus.

**Conclusion:** Thalamotomy appears to be slightly more effective in controlling motor symptoms with fewer side effects than subthalamotomy. Further studies with longer follow‐ups are needed.

**Disclosure:** Nothing to disclose.

## EPO‐442

### Hyperkinetic movement disorders expand the phenotypic spectrum of OPA1 variants: Case reports and literature review

#### M. Pocora ^1,2,3^; A. Di Fonzo^4^; S. Correia^3^; M. Fournier^3^; F. Pollet‐Villard^5^; C. Bruni‐Gauthier^5^; E. Moro^3^

##### ^1^Department of Brain and Behavioural Sciences, University of Pavia, Pavia, Italy; ^2^Headache Science and Rehabilitation Unit, IRCCS C. Mondino Foundation, Pavia, Italy; ^3^Grenoble Alpes University, Division of Neurology, CHU of Grenoble, Grenoble Institute of Neurosciences, Grenoble, France; ^4^Neurology Unit, Foundation IRCCS Ca' Granda Ospedale Maggiore Policlinico, Milan, Italy; ^5^Division of Ophthalmology, CHU of Grenoble, Grenoble, France

**Background and Aims:** Mutations in optic atrophy protein 1 (OPA1) gene are rare and are associated with dominant optic atrophy (DOA), They encompass multisystemic phenotypes, termed “DOA‐plus” syndromes, often involving a constellation of neurological symptoms. Nevertheless, hyperkinetic movement disorders (HMDs) have been very rarely reported. We herein report two cases of HMDs linked to OPA1 variant.

**Methods:** Two patients presenting with HMDs underwent comprehensive multimodal evaluations, including genetic testing, within a long‐term follow‐up.

**Results:** Patient n. 1 (66 years old, male) presented with a long history of left‐predominant intentional tremor in the upper‐limbs, musician's dystonia and writer's cramp. Patient n. 2 (38 years old, female) had longstanding perioral dyskinesias, dystonic postures of both hands, and subtle bradykinesia. Electrophysiological studies revealed sensorineural deafness in case 1 and axonal sensory neuropathy in case 2. In both cases, genetic testing showed a heterozygous OPA1 variant, c.1149A>G (p.lle383Met), that was classified as pathogenic according to the American College of Medical Genetics (ACMG) criteria. A complete ophthalmological examination was then performed, although in the absence of subjective visual complaints.

**Conclusion:** Recognition of atypical presentations, such as HMDs, and the absence of visual symptoms in OPA1 mutation carriers emphasizes the importance of broader genetic testing in patients with HMDs. Early identification may facilitate management, enabling timely interventions to patients and their children to address emerging complications like optic atrophy or other systemic manifestations.

**Disclosure:** The authors have nothing to disclose with regard to the present research.

## EPO‐443

### Subclinical gait and postural changes are associated with a “malignant” phenotype in early Parkinson's disease patients

#### N. Piramide; R. De Micco; L. Donisi; M. Siciliano; I. Giordano; V. Sant'Elia; F. Esposito; A. Tessitore

##### Department of Advanced Medical and Surgical Sciences, University of Campania “Luigi Vanvitelli”, Naples, Italy

**Background and Aims:** The presence of autonomic dysfunction, REM sleep behaviour disorders (RBD) and cognitive/behavioural symptoms has been associated with a so‐called “malignant” phenotype in patients with Parkinson's disease (PD), according to the evidence of a more rapid disease progression. These symptoms have been also consistently associated with increased risk of falls. We aimed at investigating the association between severity of these symptoms and the presence of subtle gait and postural alterations in early non‐demented PD patients.

**Methods:** Fifteen early PD patients (disease duration <5 years, Hoehn & Yahr ≤2.5) and 15 age and sex‐matched healthy controls (HC) were consecutively enrolled. Gait and balance parameters were acquired using six Opal V2R wearable sensors during the Timed up and go in single/dual task (TUG‐ST/DT) conditions. Motor symptoms was evaluated using the Unified Parkinson's disease Rating scale part III (UPDRS‐III). Nonmotor symptoms were assessed by means of the Scales for Outcomes in Parkinson's Disease ‐ Autonomic Dysfunction, Beck Depression index and RBD single question. A II‐level neuropsychological assessment was also performed. A “malignant” composite score (MCS) was created as numeric indicator of global nonmotor symptoms severity.

**Results:** Higher MCS significantly correlated with worse sit‐to‐stand performance at TUG‐ST detected by wearable sensors. No correlations have been found between the MCS and UPDRS‐III.

**Conclusion:** Our findings revealed that wearable sensors could provide useful information for the characterization of early PD patients. These subclinical changes are already associated with presence of autonomic dysfunction, RBD, cognitive and behavioural symptoms, suggesting a potential role to predict worse clinical outcome.

**Disclosure:** Nothing to disclose.

## EPO‐444

### Effects of subcutaneous foslevodopa‐foscarbidopa on motor and nonmotor symptoms in Parkinson's disease

#### O. Ciaramaglia; R. De Micco; M. D'Anna; C. Cabato; V. Sant'Elia; M. Siciliano; F. Pagliuca; A. Tessitore

##### Department of Advanced Medical and Surgical Sciences, University of Campania “Luigi Vanvitelli”, Naples, Italy

**Background and Aims:** Foslevodopa/foscarbidopa is a new combination administered 24‐hour/day continuous subcutaneous infusion (CSCI) for patients with fluctuating Parkinson's disease (PD).

**Methods:** We analyzed the effect of switching from oral L‐dopa to CSCI on motor and nonmotor symptoms over a 3‐month treatment period. From April to December 2024, 28 PD patients were implemented on CSCI in our site. Longitudinal data were available for 20 PD patients. At baseline and after 3 months, we evaluated motor symptom severity using the Unified Parkinson's Disease Rating Scale (UPDRS) part III; nonmotor symptoms burden with the NonMotor Symptom Scale (NMSS); treatment‐related motor and nonmotor complications with the UPDRS part IV and Nonmotor Fluctuation Assessment questionnaire (NoMoFa), respectively; sleep quality with the Parkinson's Disease Sleep Scale 2 (PDSS‐2); patient‐reported quality of life using the Parkinson's Disease Questionnaire 8 (PDQ‐8). At each visit, the total levodopa equivalent daily dose (LEDD) was calculated. Within‐subject longitudinal differences on demographic and clinical variables between the two timepoints were assessed by means of one‐way repeated‐measures ANOVA analyses.

**Results:** Compared to baseline, significant changes were found in NMSS, PDSS‐2 and PDQ‐8 after 3 months of treatment with CSCI. Significant increase in total wake‐up time spent in OFF state without troublesome dyskinesia was also observed at follow‐up. No significant changes were observed in total LEDD (oral vs CSCI). Infusion site adverse events were common and generally well‐tolerated after 3 months of treatment.

**Conclusion:** Our data reveal that, along with motor fluctuation stabilization, treatment with CSCI may significantly improve nonmotor symptoms burden in PD patients.

**Disclosure:** Nothing to disclose.

## EPO‐445

### Neuroacanthocytosis: Clinical and genetic heterogeneity in a cohort of 11 cases

#### O. López‐Lombardía

##### Neurology, Hospital de la Santa Creu i Sant Pau, Barcelona, Spain

**Background and Aims:** Neuroacanthocytosis (NA) is a rare disease with approximately 1,000 cases reported worldwide. It encompasses a heterogeneous group of disorders characterized by neurological and psychiatric manifestations. This study describes the clinical and genetic characteristics of a cohort of 11 NA patients followed at the Movement Disorders Unit of Hospital Sant Pau.

**Methods:** Demographic, genetic, clinical, and neuroimaging data were analyzed. Descriptive statistics were calculated for age at symptom onset, age at diagnosis, and diagnostic delay.

**Results:** The cohort included 10 males (91%) and 1 female (9%), with a mean age at diagnosis of 41.4 years (SD ± 11.5) and a mean age at symptom onset of 34.8 years (SD ± 13.2). The average diagnostic delay was 4.8 years (SD ± 7.6), ranging from 0 to 22 years. The most frequent genetic diagnoses involved variants in VPS13A and XK. Initial symptoms included chorea (27%), tics (27%), and behavioral disorders (18%). Self‐injurious behaviors and oromandibular dystonia were observed in 8 of the 11 patients (73%). Most patients exhibited psychiatric features, such as anxiety and depression, while one‐third demonstrated cognitive impairments. Neuroimaging findings frequently showed caudate volume reduction. Two patients underwent deep brain stimulation (DBS) with good clinical response.

**Conclusion:** This study highlights the clinical and genetic heterogeneity of NA, emphasizing the importance of early diagnosis to optimize clinical management. The high prevalence of self‐injurious behaviors and oromandibular dystonia emerges as a key distinguishing feature The positive outcomes observed with DBS in two cases suggest it may be a valuable therapeutic option in selected patients.

**Disclosure:** Nothing to disclose.

## EPO‐446

### Is there body district‐specificity in sequence‐specific implicit motor learning in Parkinson's disease?

#### P. Ortelli ^1^; M. Rizzo^2^; A. Bottari^2^; A. Scarton^2^; F. Bombieri^2^; M. Fiorio^2^; L. Sebastianelli^1^; D. Ferrazzoli^1^; S. Pogliaghi^2^

##### ^1^Department of Neurorehabilitation, Hospital of Vipiteno (SABES‐ASDAA), Teaching Hospital of the Paracelsus Medical Private University (PMU), Vipiteno‐Sterzing, Italy; ^2^Department of Neurosciences, Biomedicine and Movement Sciences, University of Verona, Italy;

**Background and Aims:** Motor learning alterations are associated with Parkinson's disease (PD). Body district‐specificity remains unexplored and could have implications for rehabilitation. We investigated sequence‐specific implicit motor learning (iML) differences in alternative task movements in PD patients vs age‐matched controls (HCs).

**Methods:** Thirty PD participants (67.6 ± 8.0 years, 1.9 ± 0.7 H&Y) and 30 HCs (69.6 ± 5.2 years) performed three Serial Reaction Time Tasks (SRTTs) differing for the adopted body part: Hands, Arms, Feet. Visual‐motor reaction time (RT) was recorded in response to visual stimuli. Eight blocks consisting of 4 repetitions of 12‐stimuli sequence were presented: random sequence order was practiced in block one and eight (R1, R8); a fixed 12‐stimuli sequence was performed in blocks from 2 to 7 (S2‐S7). RTs were corrected for Errors (cRT) and reprocessed as percentage, where the mean RT of R1 represented 100% (cRTR1%). The responses curve (cRTR1‐R8Curve), General Practice (GP, cRT% different from R1), Sequence Learning (SLcRT% from R8), and iML (% difference between R8 and S7) were compared among groups and body districts by two‐way RM‐ANOVA.

**Results:** PD patients showed slower cRTs for each test (*p* < 0.05), but both groups completed the repeated sequence faster than the random trials (*p* < 0.05), indicating that GP, SL, and iML did not differ across testing modalities and groups.

**Conclusion:** PD patients are able to gain an iML level comparable to HCs. The iML acquired in PD is consistent across different body regions. Therefore, SRTT could be a tool for studying iML in PD and may be used as neurorehabilitation outcome.

**Disclosure:** Nothing to disclose.

## EPO‐447

### Short‐term heart rate variability recordings as a marker of Parkinson's disease severity

#### M. Delgado‐Alvarado ^1^; S. Stavrakis^2^; C. de Dios^3^; M. Misiego‐Peral^4^; Y. Jiménez‐López^4^; J. Riancho^1^; S. Setién‐Burgués^4^; J. Sánchez‐de la Torre^4^; D. Gallo‐Valentín^4^; J. Infante^5^; M. Gómez‐España^6^; R. López‐Maza^4^; E. Aurrecoechea^6^; L. Riancho‐Zarrabeitia^6^

##### ^1^Neurology Department, Sierrallana Hospital‐IDIVAL, Torrelavega, Spain. CIBERNED, Madrid, Spain; ^2^University of Oklahoma Health Sciences, Oklahoma City, USA; ^3^Medicines Faculty, University of Cantabria, Spain; ^4^Neurology Department, Sierrallana Hospital, Torrelavega, Spain; ^5^Neurology Department, University Hospital Marqués de Valdecilla‐IDIVAL, Santander, Spain; ^6^Rheumatology Department, Sierrallana Hospital‐IDIVAL, Torrelavega, Spain

**Background and Aims:** Heart rate variability (HRV) reflects autonomic function and can be easily assessed using short‐term recordings. Despite their practicality, few studies have evaluated HRV in PD, and none have explored its relationship with disease severity. This study aimed to investigate the association between short‐term HRV measures and PD severity.

**Methods:** We conducted a cross‐sectional study, recruiting consecutive PD patients from outpatient clinics. A 5‐minute ECG recording was performed in the supine position after 10 minutes of rest, fasting, and medication abstinence overnight. HRV analysis was conducted in time and frequency domains using Kubios software.

**Results:** The patients (*n* = 40) had a mean age of 70.5 ± 7.5 years, disease duration of 6.5 ± 5 years, and a UPDRS III score of 29.1 ± 14.4. Significant inverse correlations were observed between UPDRS III scores and mean RR interval (*ρ* = −0.457; *p* = 0.021), standard deviation of NN intervals (SDNN, *ρ* = −0.436; *p* = 0.035), low‐frequency power (*ρ* = −0.428; *p* = 0.042), and total power (*ρ* = −0.454; *p* = 0.028), even after Bonferroni adjustment. Linear regression identified UPDRS III as an independent predictor of mean RR (β = −0.388; *p* = 0.008), SDNN (β = −0.304; *p* = 0.05), and SD2 (β = −0.309; *p* = 0.046) when adjusted for age. Patients were stratified by Hoehn and Yahr (H&Y) stage (≤2: *n* = 20; >2: *n* = 20). The more severe group had significantly lower RR (*p* = 0.032), SDNN (*p* = 0.014), low‐frequency power (*p* = 0.019), high‐frequency power (*p* = 0.045), total power (*p* = 0.013), and SD2 (*p* = 0.012), even after adjusting for age.

**Conclusion:** Short‐term HRV recordings may provide a simple method to assess dysautonomia in PD. Reduced HRV measures were observed in patients with greater disease severity, highlighting autonomic impairment.

**Disclosure:** Nothing to disclose.

## EPO‐448

### Biocollection effort in Parkinson's disease, parkinsonism and neurodegenerative disorders: The PADUA‐CESNE cohort

#### G. Bonato ^1^; V. Misenti^1^; M. Campagnolo^1^; A. Emmi^4^; M. Carecchio^1^; S. Cauzzo^3^; R. Manara^5^; E. Fiorenzato^6^; R. Biundo^6^; V. D'Onofrio^1^; A. Guerra^1^; L. Salviati^7^; G. Musso^8^; A. Antonini^1^

##### ^1^Parkinson and Movement Disorders Unit, Centre for Rare Neurological Diseases (ERN‐RND), Department of Neuroscience, University of Padova, Padova, Italy; ^3^Padova Neuroscience Center (PNC), University of Padova, Padova, Italy; ^4^Institute of Human Anatomy, Department of Neuroscience, University of Padova, Padova, Italy; ^5^Neuroradiology Unit, DIMED, University Hospital of Padova, Padova, Italy; ^6^Psychology Department, University Hospital of Padova, Padova, Italy; ^7^Clinical Genetic Unit, University Hospital of Padova, Padova, Italy; ^8^Department of Medicine – DIMED, University of Padova, Padova, Italy

**Background and Aims:** Recently, the potential role of fluid and tissue‐based biomarkers in Parkinson's disease (PD) and neurodegenerative disorders has emerged; further work is needed to integrate them with other clinical and instrumental data, and translating them into clinical practice. We describe PADUA‐CESNE biocollection program aimed at studying disease mechanisms and aiding in diagnosis‐prognosis‐therapy definition.

**Methods:** Standardized assessment and biocollection protocol.

**Results:** Patients undergo neurological evaluation to confirm clinical diagnosis of PD, parkinsonism or other neurodegenerative disorder. Biocollection program includes: ‐ clinical data (motor and non‐motor scales, extensive neuropsychological assessment), ‐ genetic testing (NGS Illumina NextSeq550 custom gene panel, CNV, MLPA) to define genetic diagnosis and select patients for target‐therapy trials, ‐ skin/tissue biopsy (immunohistochemistry: phosphorylated alpha‐synuclein pSyn SER129, alpha‐synuclein oligomers Clone 5G4, phosphorylated Tau pTau Clone AT8, inflammatory markers; RT‐QuIC assay), to define disease pathology at biological level, ‐ fluid biomarkers collection (SIMOA test: serum GFAP, NFL, pTau181; chemiluminescence: plasma pTau217 and CSF 1‐42/1‐40 beta‐amyloid, pTau181 and total Tau; RT‐QuIC assay for synuclein in serum, CSF and plasma) to monitor disease progression, ‐ structural and functional neuroimaging (MRI, fMRI, DAT‐Scan, PET), ‐ neurophysiology evaluation (HD‐EEG) to measure cortical connectivity. 204 serum samples, 165 plasma samples, 66 skin biopsies, 22 fibroblast cultures, 203 MRIs, 135 EEG, 400 DNA samples were collected so far from patients and healthy controls.

**Conclusion:** Biocollection effort is a fundamental approach to study PD and neurodegenerative disorders, obtaining cohorts with integrated multimodal data and comprehensive biomarker profiles for each patient, to improve diagnosis, prognosis and personalized medicine.

**Disclosure:** Nothing to disclose.

## EPO‐449

### Amantadine ER tablets for treatment of drug induced extrapyramidal reactions: A phase IV multicenter, single‐arm study

#### A. Yeole^1^; D. Nagarwal^2^; A. Barua^3^; P. Kumar^4^; B. Chaudhary^5^; A. Ansari^6^; K. Rakesh^7^; A. Verma^8^; N. Venkata Sundarachary^9^; R. Shah^10^; M. Rajurkar^11^; S. Behera^12^; C. Bornare^11^; Sonowal ^11^; D. Sonawane^11^; S. Pandit^11^; P. Devkare^11^; D. Patil^11^; P. Ghadge^11^; L. Lakhwani^12^; S. Mehta^11^; S. Joglekar^13^

##### ^1^Surya Multispeciality Hospital, Nashik, India; ^2^Jawahar Lal Nehru Medical College, Ajmer, India; ^3^Guwahati Neurological Research Centre & Hospital, Guwahati, India; ^4^PMSSY Victoria Hospital, Bengaluru, India; ^5^Apex Hospitals Pvt. Ltd, Jaipur, India; ^6^City Neurology Centre, Varanasi, India; ^7^Excel Hospital, Secunderabad, India; ^8^GSVM Medical College, Kanpur, India; ^9^Guntur Medical College & Government General Hospital, Guntur, India; ^10^V. S. General Hospital, Ahmedabad, India; ^11^Sun Pharma Laboratories Limited, Mumbai, India; ^12^Ex Sun Pharma Laboratories Limited, Mumbai, India; ^13^Ex Sun Pharmaceutical Industries Limited, Mumbai, India

**Background and Aims:** This subset analysis of a phase IV study was performed to assess the safety and efficacy of amantadine extended‐release (ER) tablets in Indian patients with drug‐induced extrapyramidal reactions.

**Methods:** A subset of 22 patients from this single‐arm, multicenter, phase IV study (CTRI/2023/04/051973), with drug‐induced extrapyramidal reactions was analyzed for safety and efficacy of amantadine ER tablet. The starting dose was 129 mg orally once; further uptitrated at weekly intervals to a maximum daily dose of 322 mg (administered as 129 mg and 193 mg tablets) as per patient's response and tolerability. Treatment duration was upto 8 weeks depending on dose uptitration. Primary objective was safety assessment. Efficacy endpoints were change from baseline in Extrapyramidal Symptom Rating Scale (ESRS) total score and ESRS part I, II, III and IV subscores.

**Results:** A total of 8 treatment‐emergent adverse events (TEAEs) occurred in 6 patients. The most common TEAE was hyperchlorhydria. All the TEAEs were non‐serious and were resolved without sequelae at the end of the study. The total mean (±SD) ESRS score decreased significantly (*p* < 0.0001) from 38.52 (±13.14) at baseline to 19.62 (±11.83) end of treatment (EOT). Also, the mean (±SD) ESRS part I, II, III and IV sub scores significantly decreased gradually from baseline till EOT (*p* < 0.05).

**Conclusion:** Amantadine ER tablet was safe and efficacious in Parkinson disease with the convenience of once‐daily dosing.

**Disclosure:** The study was funded by Sun Pharma Laboratories Limited (SPLL).

## MS and related disorders 3

## EPO‐450

### Biosimilars education in neurology: Knowledge gains through online CME

#### A. Stan ^1^; L. Fairley^1^; T. Barras^1^; M. Magyari^2^

##### ^1^Medscape Education Global, London, UK; ^2^Danish Multiple Sclerosis Center, Department of Neurology, Copenhagen University Hospital, Rigshospitalet, Copenhagen, Denmark

**Background and Aims:** We developed an online Continuing Medical Education (CME) activity titled: “Biosimilars 101: From Neurologists, for Neurologists”. We hypothesized that participation in this education would lead to improved knowledge of the key features of biologic and biosimilar medicines and the similarities and differences between them.

**Methods:** Neurologists participated in a 30‐minute video discussion between 3 experts with accompanying slides (https://www.medscape.org/viewarticle/996834). Educational effect was assessed using a repeated‐pair design with pre‐/post‐assessment. Three multiple choice questions assessed knowledge and 1 question (Likert‐type scale) assessed confidence. A paired samples t‐test was conducted for significance testing on overall average number of correct responses and for confidence rating, and a McNemar's test was conducted at the learning objective level (5% significance level, *p* <.05). Cohen's d with correction for paired samples estimated the effect size of the education on number of correct responses (<.20 modest, .20‐.49 small, .59‐.79 moderate, ≥.80 large). The CME activity launched on 9/27/2023, and the data were collected through 2/27/2024.

**Results:** A total of 621 neurologists participated, of which 33 completed all the pre‐ and post‐activity questions during the study period. Overall, 45% improved their knowledge of biosimilars (*p* < 0.001) indicating a considerable effect of the education (Cohen's *d* = 0.73) (see Table). Overall, 42% had a measurable improvement in confidence in their knowledge about biosimilars.

**Figure:**
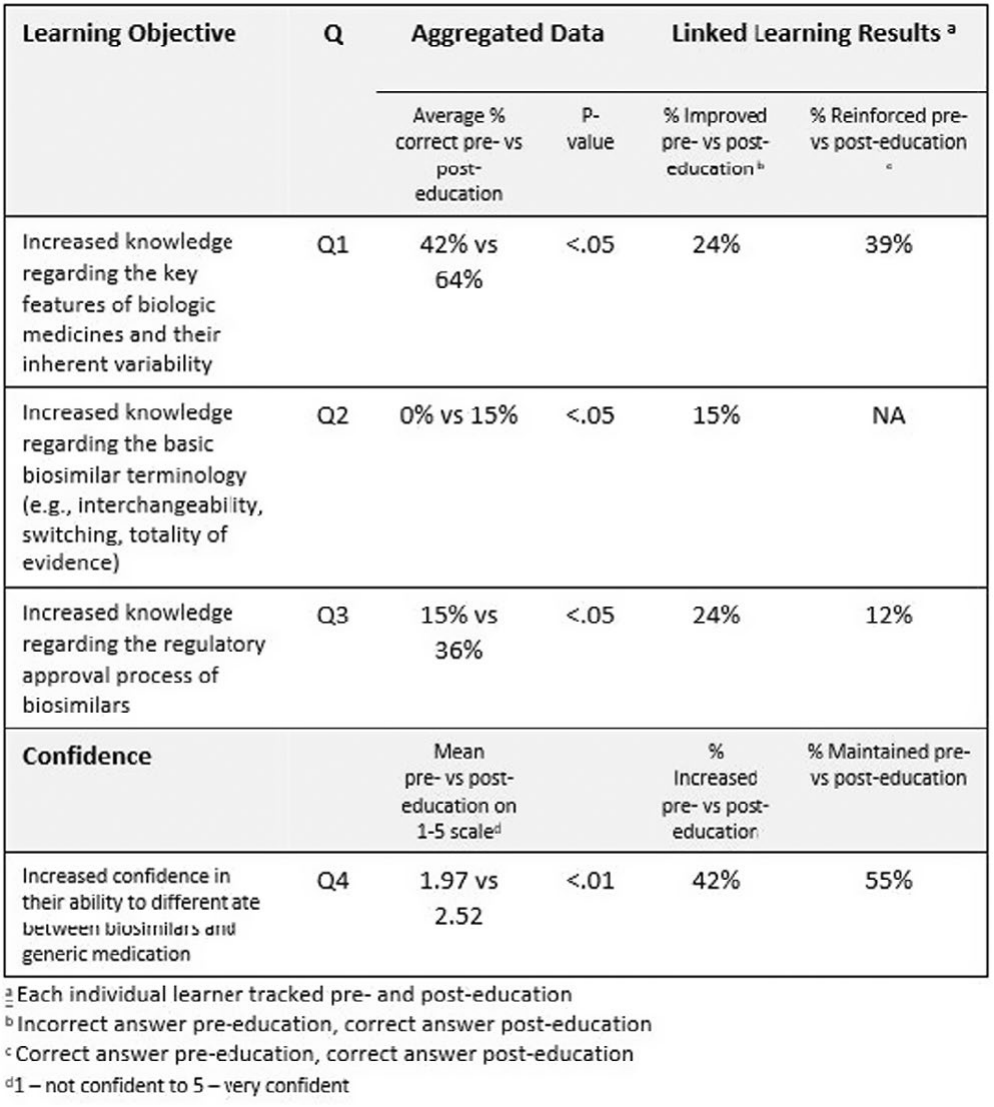
**FIGURE 1** Impact of education on’ knowledge of biosimilars

**Conclusion:** This online CME activity significantly improved neurologists’ knowledge of biosimilars, however substantial gaps remain which should be addressed in future medical education.

**Disclosure:** Nothing to disclose.

## EPO‐451

### Probiotics supplementation for information process speed in multiple sclerosis

#### A. Naseri ^1^; S. Sanaie^2^; S. Rahnemayan^2^; R. Mosaddeghi‐Heris^2^; M. Talebi^2^; M. Talebi^2^

##### ^1^Student Research Committee, Tabriz University of Medical Sciences, Tabriz, Iran; ^2^Neurosciences Research Center (NSRC), Tabriz University of Medical Sciences, Tabriz, Iran

**Background and Aims:** Probiotic supplementation, through the gut‐brain axis, is suggested to enhance clinical outcomes in patients with Multiple sclerosis (MS) and also found to improve cognitive function. This study investigated the effects of probiotic supplementation on information process speed (IPS) in minimally disabled relapsing‐remitting MS (RRMS) patients.

**Methods:** In this parallel, randomized, double‐blind, placebo‐controlled trial, 90 RRMS patients, with Expanded Disability Status Scale (EDSS) <4, received either the probiotics supplementation (Lactocare®) or a placebo twice daily for four months. Visual IPS was assessed using the Symbol Digit Modalities Test (SDMT), and the three‐second version of the Paced Auditory Serial Addition Test (PASAT‐3) was utilized to assess auditory IPS. Analysis of covariance (ANCOVA) ‐adjusted based on before values‐ was conducted using the SPSS software with 95% confidence intervals and 0.05 level of significance for *p*‐value.

**Results:** Sixty participants completed the trial (29 in the probiotics group, 31 in the placebo group). Median disease duration was 60 [IQR: 93] and 48 [IQR: 63] in the probiotics and placebo groups, respectively and most of the participants were females (72.4% and 80.6% in the probiotics and placebo groups, respectively). Based on the ANCOVA, the estimated mean and standard error for SDMT in probiotics was 46.80 ± 1.20 and in the placebo group, it was 47.47 ± 1.12 (*p*‐value: 0.68). For the PASAT‐3, these values were 47.97 ± 1.38 and 46.78 ± 1.25 in the probiotics and placebo groups, respectively (*p*‐value: 0.53).

**Conclusion:** Supplementation with a seven‐strain probiotics supplementation does not result in a significant improvement in IPS in minimally disabled RRMS patients.

**Disclosure:** Nothing to disclose.

## EPO‐452

### Radiological isolated syndrome: Models for predicting multiple sclerosis

#### D. Cetinkaya Tezer; I. Gungor Dogan; S. Demir

##### Neurology Department, Health Science University Sancaktepe Şehit Prof. Dr. İlhan Varank Training and Research Hospital, Istanbul, Turkey

**Background and Aims:** Radiologically isolated syndrome (RIS) represents a preclinical stage of Multiple Sclerosis (MS) identified by incidental MRI findings in individuals without neurological symptoms. According to the criteria proposed in 2023, fewer lesions in typical spaces are sufficient for diagnosis. This study aims to characterize asymptomatic individuals with MRI‐detected demyelinating lesions and proposes two additional terms: “preRIS” and “preMS.”

**Methods:** A retrospective analysis of patient records identified 96 individuals with demyelinating MRI lesions typical for MS. These were classified into three groups: preRIS, RIS, and preMS. PreRIS denotes lesions insufficient for dissemination in space and time criteria, while preMS fulfills these criteria. Demographic and clinical features were analyzed.

**Results:** Among 96 patients (67 females, 69.8%; mean age: 37.3 ± 10.6 years), 35 were classified as preRIS, 50 as RIS, and 11 as preMS. During a mean follow‐up of 3.97 ± 3.26 years, 26 patients (27%) converted to MS, with the preMS group showing the highest conversion rate and preRIS the lowest (*p* < 0.001). The mean time from RIS to MS diagnosis was 26.59 months (median: 12.67 months; range: 1.2–87.7 months). Common initial attacks included cerebellar symptoms and optic neuritis.

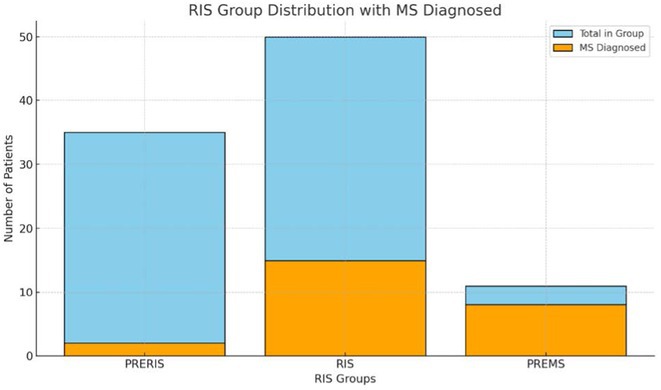

**Conclusion:** As a result of the study, preMS group demonstrated a significantly higher risk of MS conversion. Subgrouping RIS patients may refine risk stratification, improve monitoring, and inform early intervention strategies.

**Disclosure:** Nothing to disclose.

## EPO‐453

### Neuropsychological differences between NMOSD, MOGAD and MS

#### D. Ranucci ^1^; A. Spiezia^1^; G. Corsini^1^; A. Esposito^1^; V. Nicolella^1^; A. Castiello^1^; M. Petracca^2^; M. Moccia^3^; V. Brescia Morra^1^; A. Carotenuto^1^; R. Lanzillo^1^

##### ^1^Department of Neurosciences, Reproductive Sciences and Odontostomatology, University of Naples Federico II, Naples, Italy; ^2^Department of Human Neurosciences, Sapienza University of Rome, Rome, Italy; ^3^Department of Molecular Medicine and Medical Biotechnology, Federico II University of Naples, Naples, Italy

**Background and Aims:** Neuromyelitis optica spectrum disorder (NMOSD) and myelin oligodendrocyte glycoprotein antibody‐associated disease (MOGAD) are rare inflammatory diseases. While cognitive involvement in Multiple Sclerosis (MS) is well‐established, cognitive disability in NMOSD and MOGAD remain underexplored.

**Methods:** We enrolled 11 NMOSD (7 NMOSD + 4 MOGAD) and 11 SM patients (clinic‐demographic data of the patients are reported in Table1). Patients were assessed clinically with EDSS, cognitively with BICAMS (from which we obtained the total cerebral functional score, CFS), and for depression with the Beck Depression Inventory II (BDI‐II). SM patients were matched to NMOSD patients for age, education, sex and EDSS. Comparisons between‐groups for Symbol Digit Modalities Test (SDMT), California Verbal Learning Test (CVLT), Brief Visuospatial Memory Test (BVMT) and BDI‐II were performed through the Student's *t*‐test.

**Figure:**
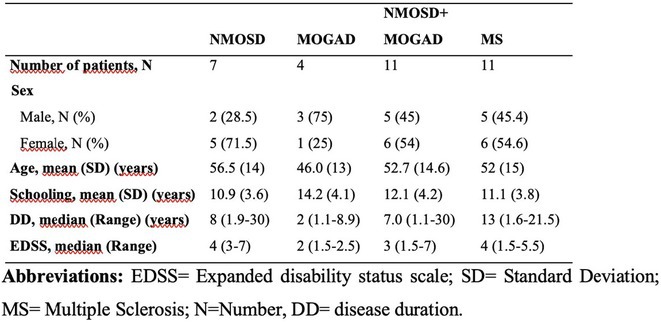
**TABLE 1** Clinical characteristics of patients with NMOSD, MOGAD, MS and in the combined NMOSD + MOGAD group.

**Results:** MS and NMOSD patients were similar for age, sex, education and EDSS. Disease duration was longer in MS patients vs NMOSD+MOGAD group (median = 13, range = 1.6–21.5 vs median = 7.0, range = 1.1–30; *p* = 0.02). SM patients had worse SDMT, BVMT and CFS scores while there was no difference for CVLT and BDI‐II. Comparing MS to NMOSD patients confirms the significant difference for SDMT and BVMT scores, whereas no differences were reported comparing MS to MOGAD patients.

**Conclusion:** Patients with MS show greater deficits in visuospatial memory and attention/information processing speed domains than patients with NMOSD or MOGAD. This finding can be explained by the greater cortical involvement and cerebral white matter diffuse abnormalities more frequently affecting MS compared to NMOSD and MOGAD.

**Disclosure:** A.E. has received honoraria from Novartis. M.M. has received research grants from ECTRIMS‐MAGNIMS, the UK MS Society, and Merck, and honoraria from Biogen, BMS Celgene, Ipsen, Janssen, Merck, Novartis, Roche, and Sanofi‐Genzyme. M.P. has received research grants from the Italian MS Foundation and Baroni Foundation, honoraria from Health & Life and Biogen, and sponsorship for travel/meeting expenses from Novartis, Roche, and Merck. R.L. has received honoraria from Biogen, Merck, Novartis, Roche, and Teva. V.B.M. has received research grants from the Italian MS Society and Roche, and honoraria from Bayer, Biogen, Merck, Mylan, Novartis, Roche, Sanofi‐Genzyme, and Teva. A.C. has received research grants from Almirall, research grants from ECTRIMS‐MAGNIMS, and honoraria from Almirall, Biogen, Roche, Sanofi‐Genzyme, Merck, Ipsen, and Novartis. None of the other authors has any conflict of interest to disclose.

## EPO‐454

### Ocrelizumab efficacy, safety, adherence, and retention in cypriot multiple sclerosis patients

#### E. Kkolou ^1^; K. Kleopa; G. Pitsas; A. Adamou; A. Koupparis; M. Pantzaris

##### Neurology Clinics, The Cyprus Institute of Neurology and Genetics, Nicosia, Cyprus

**Background and Aims:** We retrospectively evaluated real‐world efficacy, safety, adherence, and retention of ocrelizumab (OCR) in adult cypriot multiple sclerosis (MS) patients.

**Methods:** Data from forty‐five patients, encompassing two years prior and two years following OCR introduction, were analyzed. Thirty patients (67%) had Relapsing Remitting (RR) MS, whereas fifteen (33%) Primary Progressive (PP) MS.

**Results:** In RRMS patients (mean age at time of OCR initiation 42, mean MS duration 10 years, mean EDSS 3.7), mean Annual Relapse Rate (ARR) during the two years before OCR was 0.9 and mean EDSS progression 0.4. Two years after OCR, mean ARR was 0.1 and mean EDSS progression was 0.1, signifying reductions of 89% (*p* = 0.003) and 75% (*p* = 0.017) respectively compared to baseline. In PPMS patients (mean age at time of OCR initiation 51, mean MS duration 4 years, mean EDSS 5), the mean EDSS progression during the two years before OCR was 1.3. Two years after OCR, mean EDSS progression was 0.6, signifying a reduction of 54% compared to baseline (*p* = 0.134). Two patients (4%) discontinued OCR, one due to family planning and another due to disease progression. Forty‐one patients (95% of completers) adhered to treatment, completing five or six OCR infusions in 24 months. Most frequent Adverse Drug Reactions (ADRs) were infections (11%), infusion‐related reactions (4%) and gastrointestinal events (4%).

**Conclusion:** Ocrelizumab significantly reduced relapse rates and delayed disease progression in RRMS patients and considerably delayed disease progression in PPMS patients. ADRs were generally mild and transient, leading to high adherence and retention to therapy.

**Disclosure:** Nothing to disclose.

## EPO‐455

### Bruton tyrosine kinase inhibitors in relapsing multiple sclerosis: A systematic review and meta‐analysis

#### E. Bittencurt Thomaz de Assis ^1^; H. Kalaiarasan Swamy^1^; M. Otero de Melo dos Reis^2^; B. César Miranda Matos^2^; A. Stepanov Nikolaevich^1^; A. Turdieva^1^; J. Papaterra Limongi^3^

##### ^1^Baltic Federal University, Kaliningrad, Russian Federation; ^2^Universidad Nacional de Rosario, Rosario, Argentina, ^3^University of São Paulo, São Paulo, Brazil

**Background and Aims:** Bruton tyrosine kinase (BTK) inhibitors are oral drugs now being developed for the treatment of relapsing multiple sclerosis (RMS), offering potential benefits by targeting both B‐cells and innate immune responses.

**Methods:** This systematic review and meta‐analysis included three studies with four groups analyzing BTK inhibitors for RMS. A comprehensive search of PubMed, Embase, and Cochrane databases was conducted following PRISMA guidelines. Risk of bias was assessed, and a meta‐analysis was performed using Review Manager 4.1.

**Results:** A total of 2,687 patients were included, with BTK inhibitor dosages ranging from 25 mg to 150 mg per day. BTK inhibitors significantly reduced relapse rates compared to placebo and standard treatment, with a pooled rate ratio of 0.50 (95% CI: 0.44–0.57, *p* < 0.00001) and minimal heterogeneity (*I* ^2^ = 0%), indicating a consistent 50% reduction in relapse risk across studies. It also reduced T2 lesion volume compared to placebo and standard treatment (MD: −1.10, 95% CI: −1.83 to −0.37, *p* = 0.003, *I* ^2^ = 26%). However, there was no significant effect on T1 gadolinium‐enhancing lesion reduction (MD: 0.10, 95% CI: −0.13 to 0.33, *p* = 0.39), with high heterogeneity observed (*I* ^2^ = 73%). Evobrutinib, tolebrutinib, and remibrutinib were all well tolerated, with manageable side effects.

**Figure:**
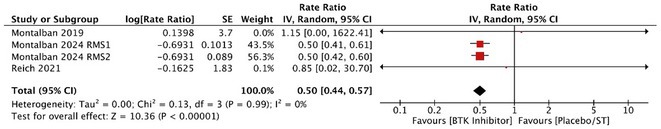
**FIGURE 1** BTK – Relapse Rate

**Figure:**
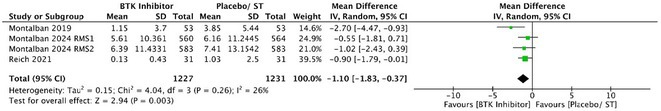
**FIGURE 2** BTK – T2 lesion

**Figure:**
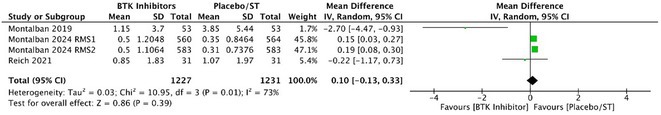
**FIGURE 3** BTK – T1 lesions

**Conclusion:** This meta‐analysis shows that BTK inhibitors reduce relapse rates and MRI‐detected T2 lesion volume, suggesting potential benefits for RMS control. However, further research is needed to confirm long‐term efficacy.

**Disclosure:** Nothing to disclose.

## EPO‐456

### Impact of treatment change on long‐term disease outcomes in multiple sclerosis

#### N. Yapıcı^1^; E. Kaya ^2^; U. Samadzade^3^; Y. Simsek^3^; I. Kara^4^; T. Kahraman^5^; S. Ozakbas^3^

##### ^1^Turgutlu State Hospital, Manisa, Turkey; ^2^Dokuz Eylul University, Faculty of Medicine, Department of Neurology, Izmir, Turkey; ^3^Izmir University of Economics, Medical Point Hospital, Izmir, Turkey; ^4^Izmir University of Economics, Graduate School, Izmir, Turkey; ^5^Katip Celebi University, Faculty of Health Sciences, Departmant of Physiotherapy and Rehabilitation, Izmir, Turkey

**Background and Aims:** Disease‐modifying therapies (DMTs) alter the course of MS through immunosuppression or immunomodulation. Despite considerations of the side effect profiles and effectiveness of DMTs during clinical follow‐up, determining the right medication at the right time can still be challenging for clinicians.The aim of this study was to compare relapse rates among people with MS who switched DMTs due to side effects with those who switched DMTs for other reasons, such as lack of efficacy, pregnancy, or scheduled stop.

**Methods:** A retrospective analysis was conducted on a cohort of 337 people diagnosed with MS who had undergone treatment changes and were followed up for 10 years. Patients were divided into two groups based on the reason for treatment discontinuation: side effects (*n* = 253, 75%) and lack of efficacy/pregnancy/scheduled stop (*n* = 84, 25%). Demographic and clinical characteristics were compared between the two groups using the Mann‐Whitney U test. The primary outcome measure was the number of relapses after treatment discontinuation.

**Results:** There were no significant differences in sex, age, disease duration, disease type, or Expanded Disability Status Scale (EDSS) scores between the two groups (*p* > 0.05). However, patients who discontinued DMT due to side effects experienced significantly more relapses after switching medications [median = 2, interquartile range (IQR) = 0–3] compared to those who discontinued due to pregnancy or inefficacy (median = 1, IQR = 0–2) (*p* < 0.001).

**Conclusion:** Patients who discontinued DMTs due to side effects experienced a higher number of relapses after switching medications compared to those who discontinued due to pregnancy or inefficacy. This highlights the importance of careful consideration of treatment decisions, especially when managing adverse effects, to optimize patient outcomes.

**Disclosure:** Nothing to disclose.

## EPO‐457

### Dual diagnosis of multiple sclerosis and Parkinson's disease

#### F. Javed ^1^; M. Idris^2^; H. Kearney^2^; S. Hutchinson^2^; N. Tubridy^2^; W. Shields^2^; M. Mckenna^2^; A. Mullen^2^; B. Dwyer^2^

##### ^1^Tallaght University Hospital, Dublin, Ireland; ^2^St. James Hospital, Dublin, Ireland

**Background and Aims:** This case report describes two patients with a rare dual diagnosis of MS and Parkinson's disease. Both were initially diagnosed with MS, but later developed Parkinsonism. A dopamine transporter scan confirmed reduced uptake, highlighting the need for ongoing clinical surveillance.

**Methods:** This case report contains two patient reports of such a dual diagnosis. In both cases, the patients had been diagnosed clinically with MS, and this was supported by MRI and CSF analysis.

**Results:** Case 1: A 58‐year‐old woman with MS developed Parkinson's disease (PD) 15 years later. Treatment with IFN‐β, Levodopa, and Gabapentin improved symptoms but posed challenges with fatigue and worsening PD. Case 2: A 62‐year‐old man with MS and a family history of PD developed PD 2 years post‐MS diagnosis. Ofatumumab and PD treatment yielded good outcomes with no MS progression.

**Conclusion:** The two cases highlight the rare co‐occurrence of MS and PD, posing diagnostic and treatment challenges due to overlapping symptoms. Neuroinflammation and immune dysregulation may link both diseases. DaTSCAN aids diagnosis, and future therapies like NLRP3 inhibitors hold promise.

**Disclosure:** Nothing to disclose.

## EPO‐458

### Switching from fingolimod to B cell‐depleting therapy in multiple sclerosis: A systematic review and meta‐analysis

#### A. Machado Silva^2^; Chaves do Vale ^1^; M. Guedes Almino Pessoa^3^; Menegaz de Almeida^5^; P. de Souza Wagner^6^; F. Alves de Paiva^7^; D. Yukiko Kogawa^4^

##### ^1^Medicine, Catholic University of Pernambuco, Brazil; ^2^Medicine, University City of Sao Paulo, Brazil; ^3^Medicine, Catholic University of Pernambuco, Brazil; ^4^Medicine, Universidad de Buenos Aires, Argentina, ^5^Medicine, Federal University of Mato Grosso, Brazil; ^6^Medicine, Federal University of Santa Catarina, Brazil, ^7^Medicine, Federal University of Amazonas, Brazil

**Background and Aims:** MS is a chronic disorder with a relapsing‐remitting phenotype (RRMS). Fingolimod reduces relapse frequency, but a switch to ATZ, RTX, OCR, or cladribine may be needed if relapse prevention is insufficient.

**Methods:** MEDLINE, Embase, and Cochrane Library were searched. HR and RR were calculated using a random‐effects model, both with 95% CI. Heterogeneity was assessed with the *I* ^2^ test.

**Results:** Switching from fingolimod to each B cell‐depleting therapy reduces the annual relapse rate (ARR), with an effect of 0.2806 (95% CI 0.1356 to 0.4255; *p* = 0.15). Furthermore, the risk of experiencing a first relapse after switching from fingolimod to a B cell‐depleting therapy was significantly higher compared to patients who were treatment‐naive (HR 5.2384; 95% CI 2.6839 to 10.2243; *p* < 0.001). Expanded Disability Status Scale (EDSS) reduction was observed in the pooled analysis with an effect of 2.2932 (95% CI 1.4141 to 3.1723; *p* = 0.20).

**Figure:**
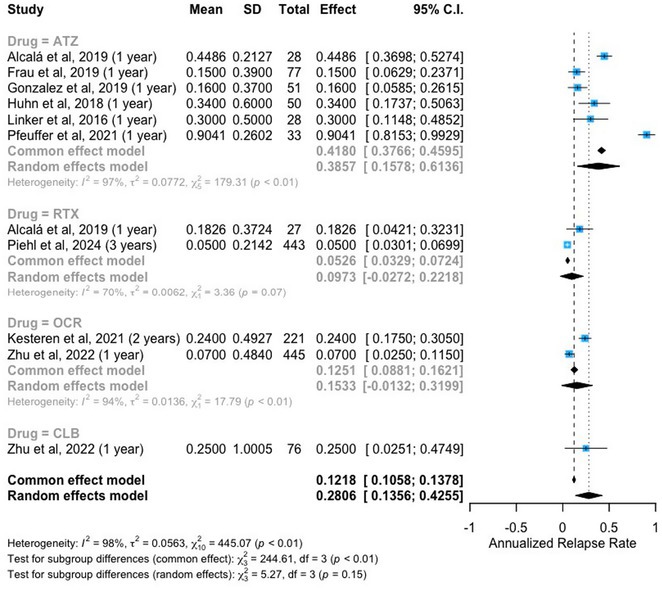
**FIGURE 1** Annualized Relapse Rate

**Figure:**
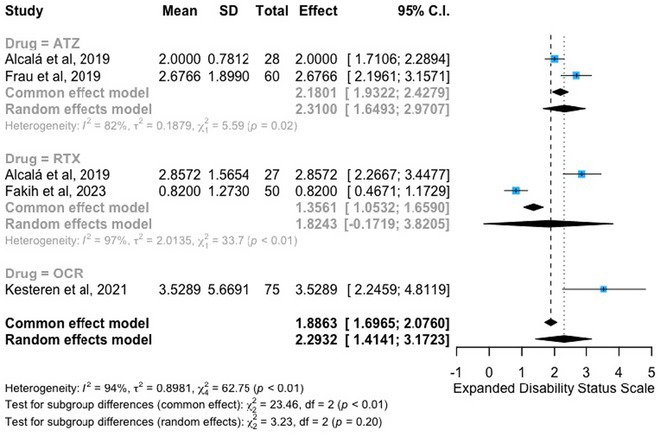
**FIGURE 3** Expanded Disability Status Scale

**Figure:**
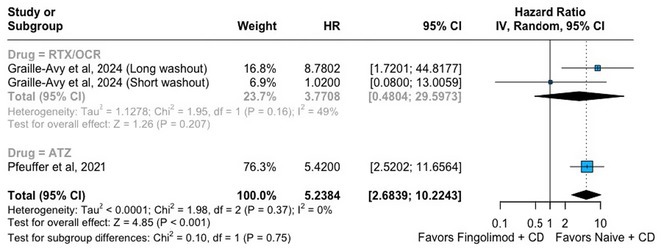
**FIGURE 2** Time to First Relapse

**Conclusion:** ATZ most effectively reduces relapse rates and improves disability in patients switching from fingolimod, while the others therapies provide limited benefit.

**Disclosure:** Nothing to disclose.

## EPO‐459

### Social determinants of health in multiple sclerosis in Italy: A scoping review

#### M. Ponzano; M. Fedriga; A. Signori; M. Sormani

##### Department of Health Sciences, University of Genoa, Genoa, Italy

**Background and Aims:** Social determinants of health (SDH) can influence outcomes related to multiple sclerosis (MS) and the relationship between SDH and MS is complex, due to the interplay between a wide range of factors and to potentially bidirectional associations. Inequities can be also seen in countries with an universal health‐care system such as Italy. This scoping review aims to identify and synthesize the existing and emerging literature on SDH in MS Italian population, exploring a wide range of determinants (sex, gender and sexuality; race and ethnicity; education; employment; socioeconomic status; domestic abuse; health‐care access; food access; air pollution; social support).

**Methods:** This review followed the methodological guidelines for scoping reviews. Eleven PubMed search strings were defined and works exploring SDH in MS Italian patients over the past decade were included, together with emerging literature from the ECTRIMS congress.

**Results:** A total of 214 works (284 SDH findings) were included. More than one‐third of the works focused on sex and gender while just one on domestic abuse; articles often presented results on several SDH simultaneously, especially for education and employment (*N* = 14) and health‐care access and social support (*N* = 10). This work showed associations between a wide range of SDH and MS and revealed several unmet needs and disparities in the Italian MS population.

**Figure:**
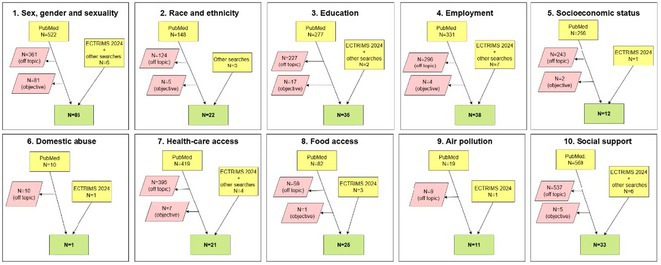
**FIGURE 1** Flow diagram – study selection process

**Figure:**
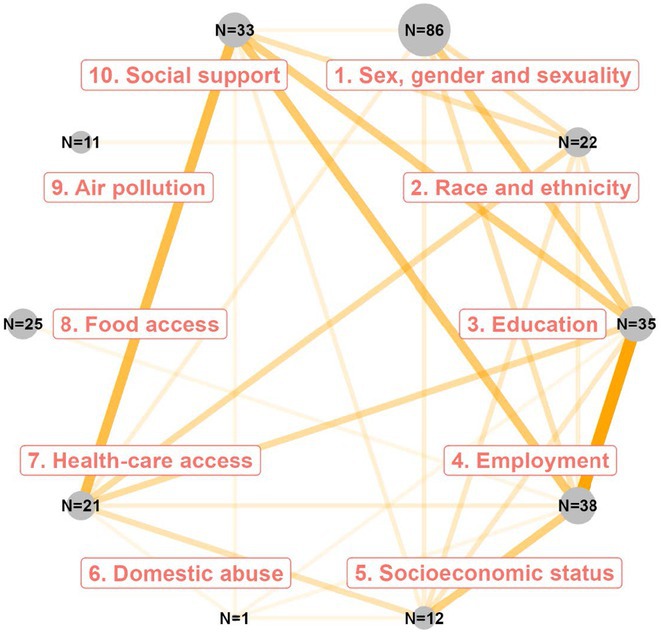
**FIGURE 2** Relationship between the included studies

**Conclusion:** Disproportionality in thematic areas of research was observed and some gaps were identified. Findings from this review demonstrate the importance of accounting for SDH in the health context and can potentially guide future interventions from the Italian sociopolitical perspective.

**Disclosure:** Nothing to disclose.

## EPO‐460

### Reasons for switching initial disease modifying therapies in early relapsing MS: Lateral vs. escalation strategies

#### M. Peters^1,2^; D. Ellenberger^1^; F. Fneish^1^; N. Frahm^1^; P. Flachenecker^3^; F. Paul^4^; H. Temmes^5^; K. Hellwig ^6^; A. Stahmann^1^

##### ^1^German MS‐Registry, MS Forschungs‐ und Projektentwicklungs‐ gGmbH (MS Research and Project Development gGmbH [MSFP]), Hannover, Germany; ^2^German MS‐Registry, Gesellschaft für Versorgungsforschung mbH (Society for Health Care Research [GfV]), Hannover, Germany; ^3^Neurological Rehabilitation Center Quellenhof, Bad Wildbad, Germany; ^4^Experimental and Clinical Research Center, Max Delbrueck Center for Molecular Medicine and Charité – Universitätsmedizin Berlin, Berlin, Germany; ^5^Deutsche Multiple Sklerose Gesellschaft, Bundesverband e.V. (German Multiple Sclerosis Society [DMSG]), Hannover, Germany; ^6^St. Josef Hospital, Ruhr University, Department of Neurology, Bochum, Germany

**Background and Aims:** The number of treatment options for relapsing multiple sclerosis (MS) has increased considerably in recent years. Switches within disease‐modifying therapies (DMTs) are common clinical practice. This study analyses reasons for switching from DMT of mild/moderate efficacy (MME‐DMTs) either lateral (within the same efficacy category) or through escalation (to highly effective therapies, HE‐DMTs).

**Methods:** Data from the German MS Register (as of 1‐Sep‐2024) included patients with relapsing‐remitting MS (RRMS) on initial MME‐DMT switching their DMT. Inclusion criteria were MS diagnosis ≥2016, initial DMT ≥2018 and discontinuation of the initial DMT. In the absence of a reported reason for DMT discontinuation, a surrogate for lack of therapy efficacy was used to evaluate switch reasons.

**Results:** The study population included 622 MS patients (73.6% female). Of these, 389 switched laterally to another MME‐DMT and 233 escalated to HE‐DMTs (Figure 1). The median age at start of the first DMT was 32.7 [25% quartile; 75% quartile: 25.8; 40.2] years. Median time to switch was 0.75 years for lateral and 1.25 years for escalation switch, with most switches occurring within the first two years (Figure 2). A lack of DMT efficacy was the primary reason for escalation switches (71.5%), while adverse events dominated lateral switches (53.8%). Patient requests and pregnancy‐related reasons were more common in lateral switches (Table 1).

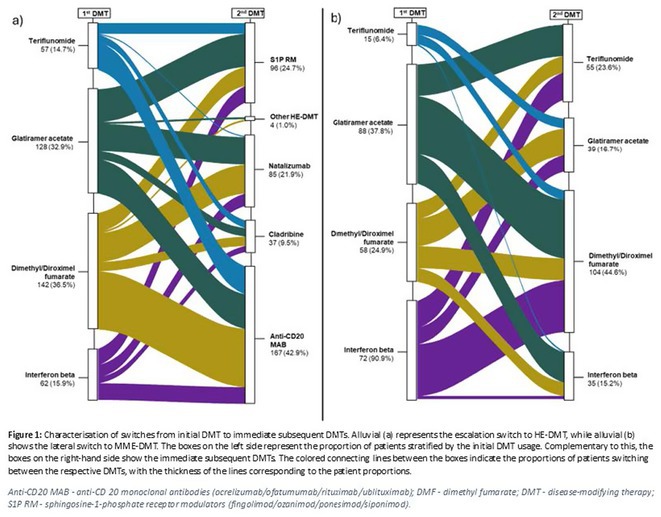

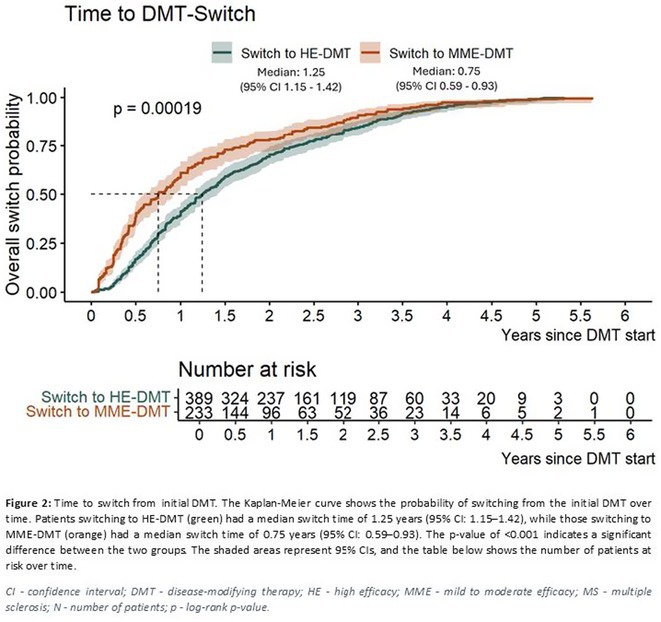

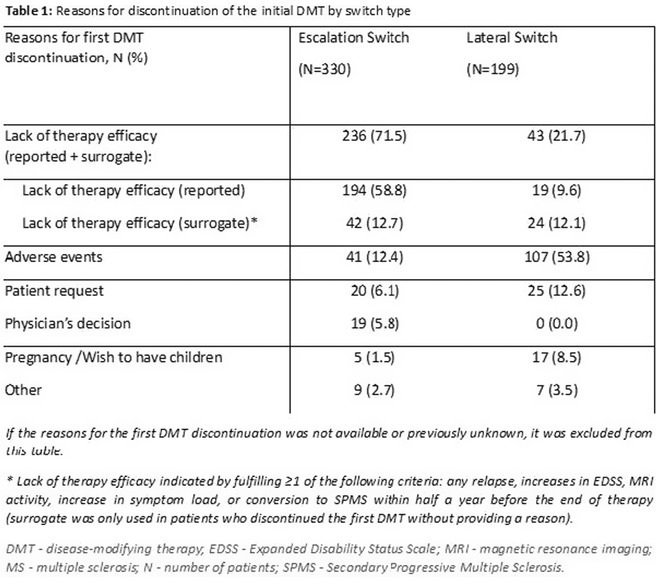

**Conclusion:** Reasons for escalation switches are primarily disease activity and progression (lack of efficacy). Escalation switches occur later compared to lateral switches. The findings highlight the differing clinical contexts and patient priorities influencing therapy decisions.

**Disclosure:** MP, DE, and FF have nothing to disclose. KH, AS, NF, PF, FP and HT have received speaking fees, travel support, honoraria from advisory boards and/or financial support for research activities from industry sponsors.

## EPO‐461

### Pseudocoloring and homomorphic filtering of product of T2 and weighted SWAN registered MRIs for CVS visualization

#### O. Pereverzeva ^1^; A. Mikitchuk^2^; A. Buniak^1^

##### ^1^Neurological department, Republican research and clinical center, Minsk, Belarus; ^2^Faculty of Radiophysics and Computer Technologies, Belarusian State University, Minsk, Belarus

**Background and Aims:** Revision of McDonald criteria’2024 takes into account recent advances in imaging of multiple sclerosis (MS). Revisions are likely to be published, new diagnostic markers include the central vein sign (CVS). CVS is studied only during study in‐depth because either specialized routine as T2*, or analysis of multimodal magnetic resonance imaging (MRI). Visualization enhancement, especially, express method without need in both artificial intelligence and hardware‐based MRIs, is very actual.

**Methods:** The golden standard of CVS visualization is hardware‐based T2* MRI, but very similar result can be obtained by means of T2 and SWAN (magnitude and phase) processing (fig. 1). The set of MRIs (1.5 T and 3.0 T) was collected for 18 patients with diagnosed MS.

**Figure:**
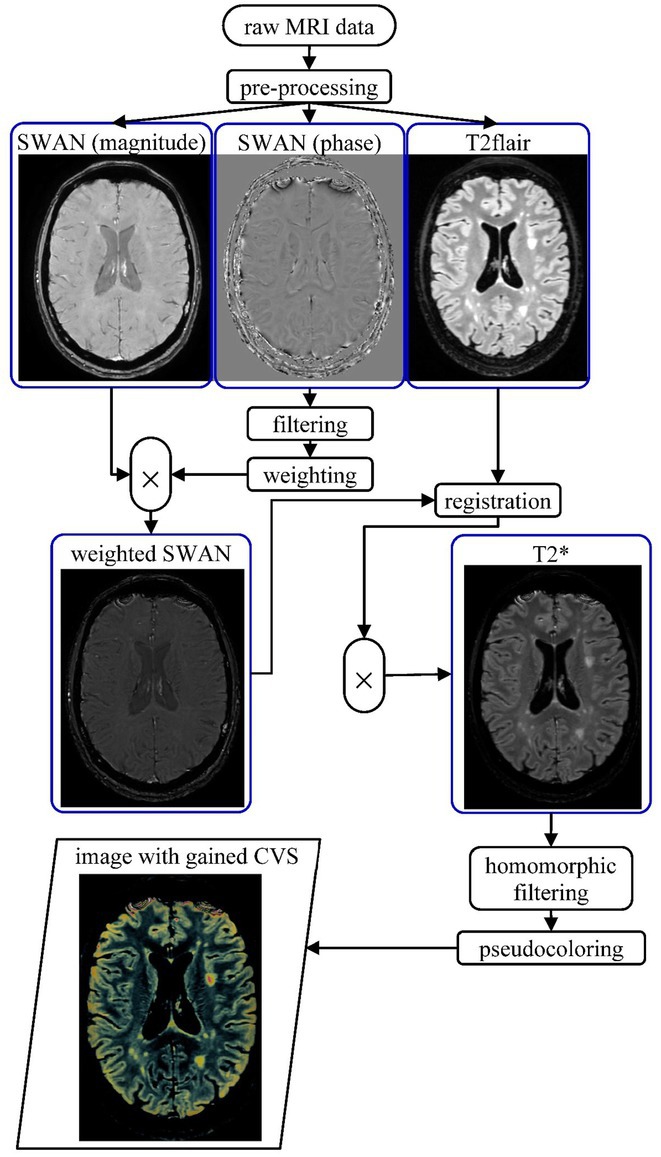
**FIGURE 1** T2 and SWAN (magnitude and phase) processing scheme

**Results:** Each MRI is performed for 120–272 slices of both T2FLAIR and SWAN. Low‐frequency fluctuations are filtered from SWAN phase simultaneously with local correction algorithms to reduce artifacts. The magnitude is multiplied by the filtered phase. Result image contains both magnitude and phase information, after it is registrated (SPM12) with T2 one and they are multiplied on order to form T2*. Homomorphic filtering of T2* allows to simultaneously normalize the brightness across an image and increase contrast, after non‐monotonic pseudocoloring is applied. This allows to enhance visualization of “coffee bean”, “central dot” and classical CVS patterns (fig. 2).

**Figure:**
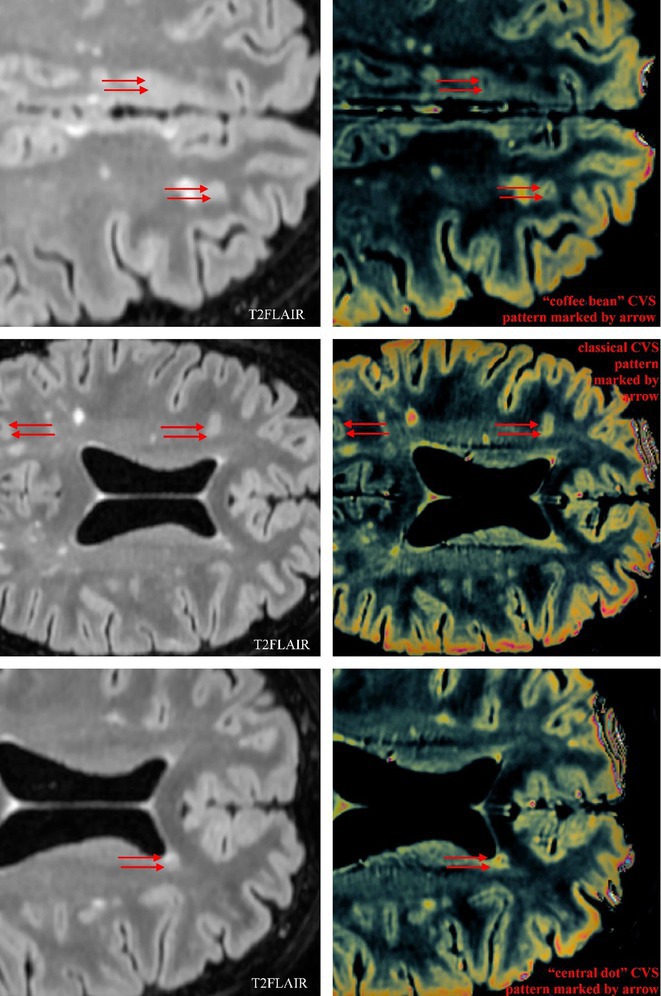
**FIGURE 2** Improved visualization of “coffee bean”, “central dot” and classical CVS patterns

**Conclusion:** This method enhances CVS visualization for both 1.5 and 3.0T MRIs, the approach is verified by direct comparison with registrated SWAN images.

**Disclosure:** Nothing to disclose.

## EPO‐462

### Application of phase stretch transform to standard MRI protocol for central vein sign visualization enhancement

#### O. Pereverzeva ^1^; A. Mikitchuk^2^; A. Buniak^1^

##### ^1^Neurological department, Republican research and clinical center, Minsk, Belarus; ^2^Faculty of Radiophysics and Computer Technologies, Belarusian State University, Minsk, Belarus

**Background and Aims:** The revision’2024 of McDonald criteria demonstrates the shift towards considering the pathomorphological basis of multiple sclerosis (MS). In patients with typical lesions in one topography, the presence of 6 lesions with central vein sign (CVS) plus dissemination in time is sufficient to diagnose MS. However, simplified magnetic resonance imaging (MRI) typically provide only T1/T2 routines. Especially, less than 3 T MRI, CVS is visualized poorly. In this paper, the image processing of phase stretch transform (PST) is adopted (with zero‐learning approach) in order to enhance CVS visualization.

**Methods:** PST does not add additional info to image, it only gains valuable spatial frequencies. By so doing, “super‐resolution” can be achieved by suppression of MRI‐artefacts. Set of MRIs (34–272 slices of T2 each) was collected for 37 patients with diagnosed MS.

**Results:** MRIs are pre‐processed in order to obtain T2flair images, after PST based on non‐linear filtering of images phase in Fourier domain is applied. Before processing, the real‐valued strength and warp of the phase profile are sweeped in order realize fine tuning of PST parameters, pre‐learn nonlinear filter for “super‐resolution” (fig. 1). PST gains valuable spatial frequencies for both 1.5 and 3.0T MRIs, it allows to enhance visualization of “coffee bean”, “central dot” and classical CVS patterns (fig. 2).

**Figure:**
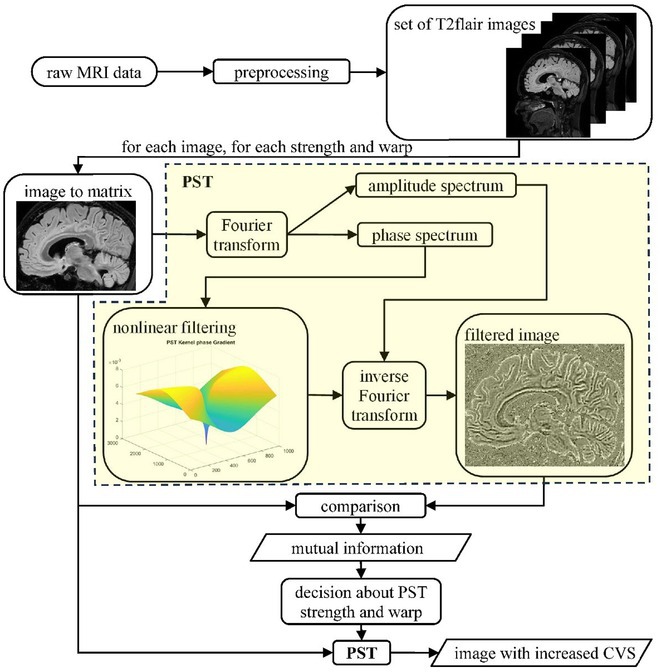
**FIGURE 1** Image processing scheme

**Figure:**
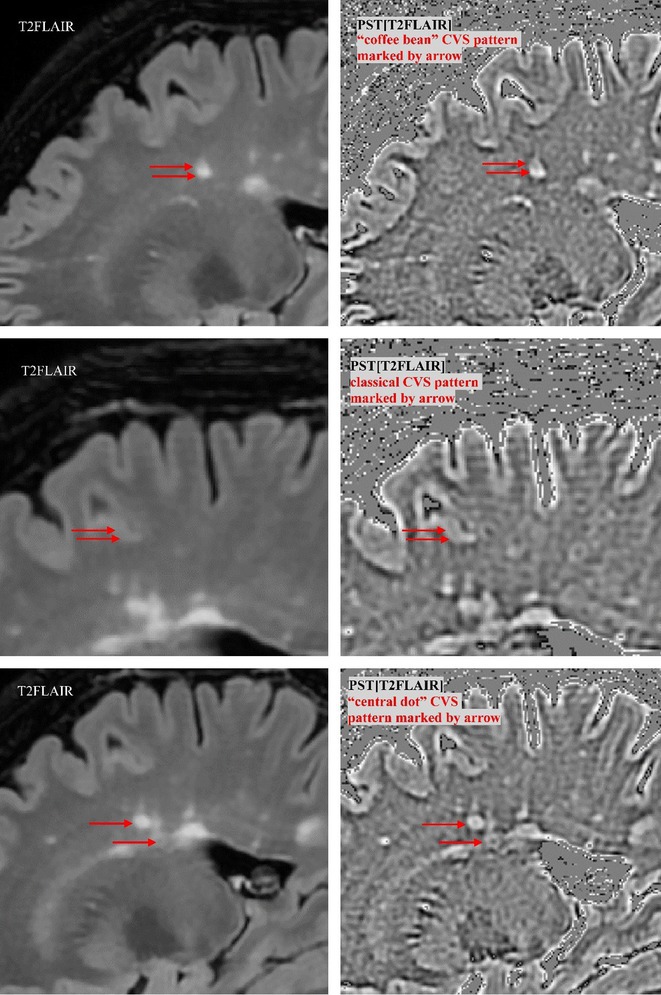
**FIGURE 2** Improved visualization of “coffee bean”, “central dot” and classical CVS patterns

**Conclusion:** PST visualization enhancement is verified by direct comparison with registrated SWAN for CVS. That allows to fulfil McDonald criteria’2024 even based on simplified MRI protocol.

**Disclosure:** Nothing to disclose.

## EPO‐463

### Lesions in critical brain regions increase the risk of migraine in multiple sclerosis

#### P. Gklinos ^1^; M. Evangelopoulos^1^; G. Velonakis^2^; D. Mitsikostas^1^

##### ^1^First Department of Neurology, Eginition University Hospital, National and Kapodistrian University of Athens, Athens, Greece; ^2^Department of Radiology, General University Hospital “Attikon”, National and Kapodistrian University of Athens, Athens, Greece

**Background and Aims:** An increased prevalence of migraine in people with MS (pwMS) has been documented over the past decade, with one of the leading explanations being the presence of lesions within regions, critical for pain modulation.

**Methods:** PwMS fulfilling the 2017 Mc Donald criteria were recruited prospectively in the study from the outpatient MS clinic of Eginition University Hospital, Athens, Greece. Patients underwent a detailed neurological examination and assessment for primary headache disorders. Brain MRI scans were obtained and assessed. Odd‐ratios (ORs) were calculated to examine the potential association of lesions within pain‐perceiving brain regions and primary headache disorders.

**Results:** A total of 96 participants were included in the study. PwMS with a lesion in the periaqueductal gray (PAG) were more than four times more likely to experience migraine compared to those without a lesion in this region (OR = 4.7; 95% CI: 1.62–13.57; *p* < 0.05). Although not statistically significant, thalamic or cortical lesions were also associated with migraine (OR = 6.9; 95% CI: 1.48–31.80; and OR = 5.8; 95% CI: 1.3–26.03; respectively).

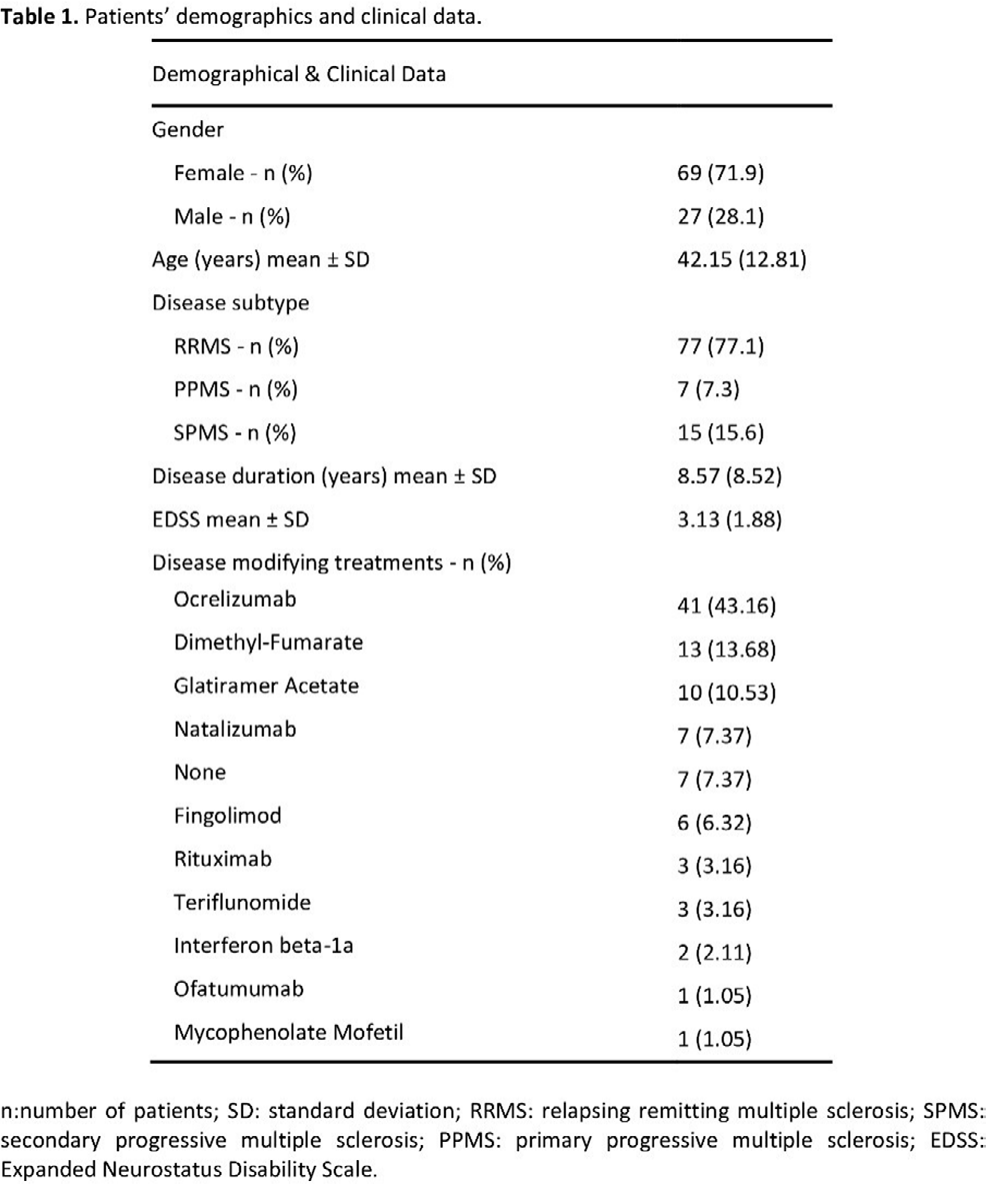

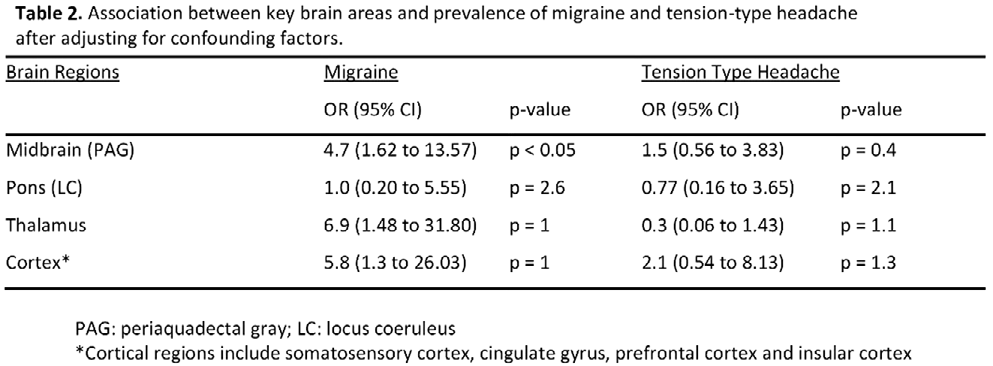

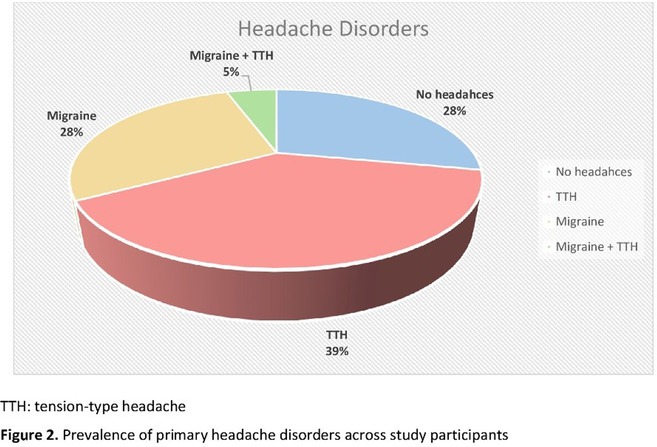

**Conclusion:** Lesions within critical brain regions are associated with migraine and are possibly the leading cause of the increased prevalence of migraine in pwMS.

**Disclosure:** Nothing to disclose.

## EPO‐464

### CIDP: A case series of 7 patients treated with Efgartigimod

#### Z. Zheng ^1^; Y. Tang^2^

##### ^1^Guangdong Hospital of Traditional Chinese Medicine, Guangzhou, China; ^2^The Second Clinical Medical School of Guangzhou University of Chinese Medicine, Guangzhou, China

**Background and Aims:** Chronic inflammatory demyelinating polyneuropathy (CIDP) is a rare immune‐mediated demyelinating disease of the peripheral nervous system, characterized by symmetrical proximal and distal limb weakness and sensory dysfunction. Efgartigimod, as a neonatal Fc receptor (FcRn) antagonist, has demonstrated potential therapeutic efficacy in some autoimmune diseases. Therefore, the aim of this study was to investigate the clinical application and efficacy of Efgartigimod in the treatment of CIDP patients.

**Methods:** We conducted a study on seven CIDP patients treated with Efgartigimod shock therapy. Prior to treatment, these patients underwent comprehensive neuroelectrophysiological assessments. The results revealed that all seven patients exhibited demyelinating changes combined with axonal injury, which met the diagnostic criteria for CIDP. Concurrently, the INCAT scores of these seven patients were meticulously recorded both prior to and following the treatment.

**Results:** Among the seven patients treated with Efgartigimod, five showed significant improvement in their INCAT scores. A paired *t*‐test was employed, revealing a statistically significant difference in the scores before and after treatment (*t* = 0.0214, *p* < 0.05).

**Conclusion:** Efgartigimod appears to possess therapeutic potential for CIDP patients, especially in enhancing limb function and nerve conduction. This report provides preliminary clinical evidence for the application of Efgartigimod in CIDP treatment, offering a reference for further research and clinical practice.

**Disclosure:** Nothing to disclose.

## Muscle and neuromuscular junction disorder 3

## EPO‐465

### Mexiletine paediatric investigation plan, PIP4 study: Efficacy findings in children aged 0–

#### C. Barnérias^1^; A. Isapof^2^; J. Hogrel^3^; N. Adetoro^4^; A. Zozyla‐Weidenfeller ^5^

##### ^1^Hôpital Necker‐Enfants Malades, Paris, France; ^2^CHU Paris Est – Hôpital d'Enfants Armand‐Trousseau, Paris, France; ^3^Institute of Myology, Paris, France; ^4^Lupin Pharmaceuticals, Baltimore, USA; ^5^Lupin Neurosciences, Zug, Switzerland

**Background and Aims:** The PIP explores mexiletine use in children with myotonic dystrophy (DM) or non‐dystrophic myotonia (NDM). Here, efficacy findings in PIP4 are presented.

**Methods:** PIP4 (EudraCT2019‐003757‐28): 12‐week, open‐label, non‐comparative study of mexiletine in sequential cohorts. Cohort 1: 12–<18 years; Cohort 2: 6–<12 years. Study design: 4 weeks’ screening; 4 weeks’ mexiletine 62, 83 or 167 mg once‐daily titrated to maximum 3‐times‐daily; 4 weeks’ maintenance (best‐tolerated dose). Efficacy endpoints (baseline to end of study): relaxation time measured by handgrip dynamometer; patient‐reported visual–analogue scale [VAS] 0–100 scores [stiffness, pain, weakness/fatigue]); Myotonia Behavioural Scale (MBS); Pediatric Quality of Life InventoryTM (PedsQL); Clinical Global Impression (CGI).

**Results:** PIP4 Cohort 1 (*N* = 7): 2 with DM1, 5 with NDM (mean age 13 years; 4 female; max dose range 186–500 mg). Cohort 2 (*N* = 5) with NDM (mean age 8 years; 3 female; max dose range 186–249 mg). Mexiletine treatment improved relaxation time (all cohorts, Figure 1). PIP4 VAS scores (*n* = 10): stiffness, 33.7–78.5% improvement; pain improvements, 85.4% (Cohort 1); 61.3% (combined cohort); weakness/fatigue, 43.2–60.2% improvements. MBS scores improved (*n* = 8; 67%), were stable (*n* = 3; 25%) or worsened (*n* = 1; 8%) Overall, MBS scores decreased across cohorts (Figure 2). Improvements were observed for PedsQL (Figure 3), especially physical domain (most impacted at baseline) and among older children. PedsQL neuromuscular scores also improved, especially in Cohort 1. For CGI, mexiletine was rated very efficient (25%), good (58%), fair (17%) (all cohorts).

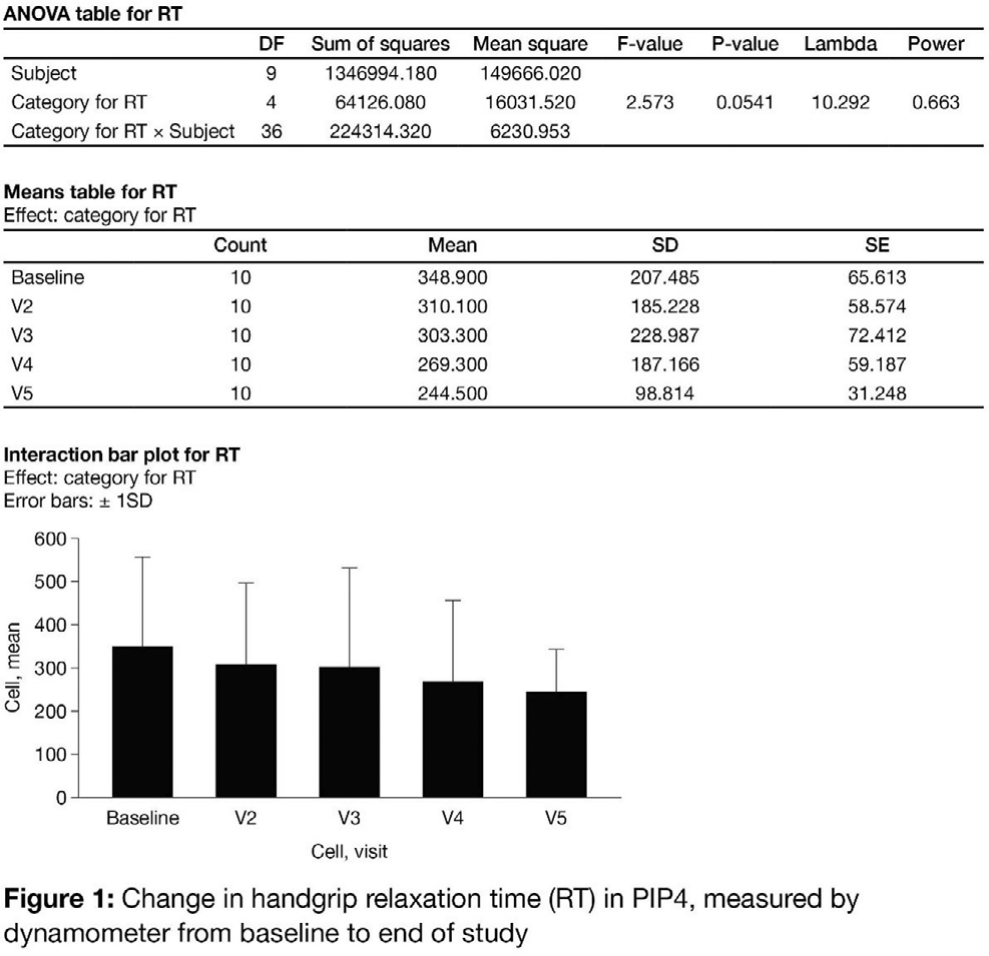

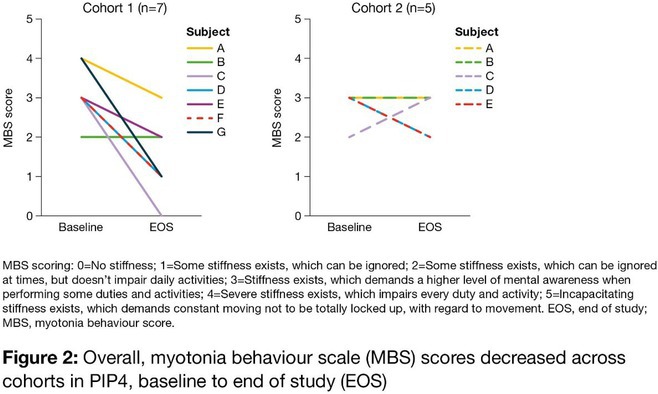

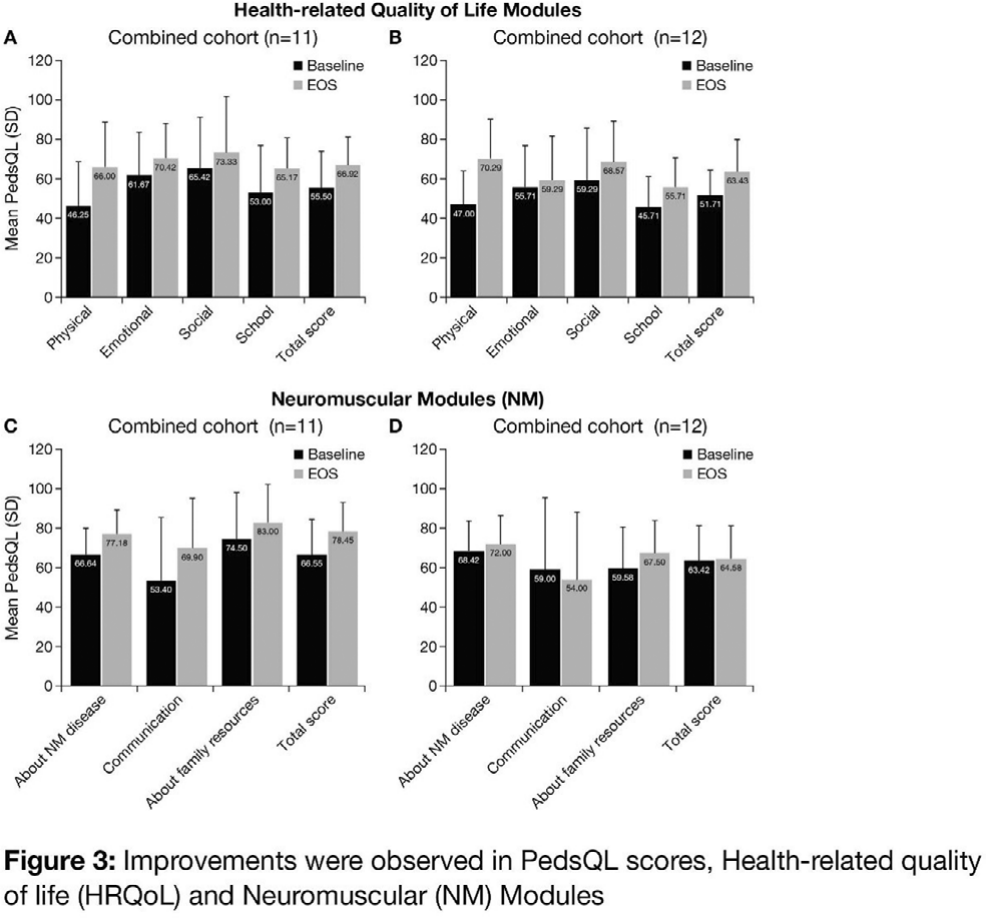

**Conclusion:** PIP4 confirms that mexiletine is efficacious treatment for myotonia in children aged 6‐<18 years. PIP4 completers are being followed for ≥2 years in PIP7.

**Disclosure:** CB; AI; J‐YH; NA: consultancy fees from Lupin AZ‐W: Lupin employee.

## EPO‐466

### Mexiletine paediatric investigation plan, PIP4 study: Safety and pharmacokinetic findings in children with myotonia

#### C. Barnérias^1^; A. Isapof^2^; H. Pentikis^3^; N. Adetoro^4^; A. Zozyla‐Weidenfeller ^5^

##### ^1^Hôpital Necker‐Enfants Malades, Paris, France; ^2^CHU Paris Est – Hôpital d'Enfants Armand‐Trousseau, Paris, France; ^3^SAJE Consulting, Baltimore, Maryland, USA; ^4^Lupin Pharmaceuticals, Baltimore, USA; ^5^Lupin Neurosciences, Zug, Switzerland

**Background and Aims:** The PIP explores safety of mexiletine treatment in children with myotonic disorders.

**Methods:** PIP4 (EudraCT2019‐003757‐28) was a 12‐week open‐label exploration of mexiletine in sequential cohorts. Cohort 1: 12–<18 years; Cohort 2: 6–<12 years. Design: 4 weeks’ screening; 4 weeks’ mexiletine 62, 83 or 167 mg once‐daily titrated to maximum 3‐times‐daily; 4 weeks’ maintenance (best‐tolerated dose). Primary endpoints: Safety, pharmacokinetics (PK), tolerability, adverse‐event (AE) profiling including ECG, baseline–end of study (EOS).

**Results:** Cohort 1: *N* = 7(mean age 13y; 4 female); cohort 2: *N* = 5(mean age 8 years; 3 female). Table 1 shows maintenance dose/body weight. Mexiletine exposure, ≥53 days (all subjects continue in PIP7 ≥24‐month extension). ECGs were normal excluding one abnormal baseline assessment (not clinically significant). Mexiletine was well tolerated. All AEs and TEAEs were mild; most resolved without intervention and were unrelated. No subjects reported dose modifications. TEAEs were reported in *n* = 6 (86%) Cohort 1, *n* = 1 (20%), Cohort 2: overall *N* = 7 (58%). No deaths, serious TEAEs, or TEAEs leading to study discontinuation were reported. Most frequent TEAEs: abdominal pain and nausea. Physical examinations and haematological, biochemical, and muscle‐function assessments revealed no clinically significant changes. PK data confirm paediatric mexiletine exposure, consistent with well‐established adult posology. PK modelling adequately described mexiletine concentration data, showing good agreement between observed and predicted concentrations. Paediatric doses required to achieve mexiletine concentrations are similar to adult doses (Figure 1). Bootstrap analysis indicates the model is robust (Table 2).

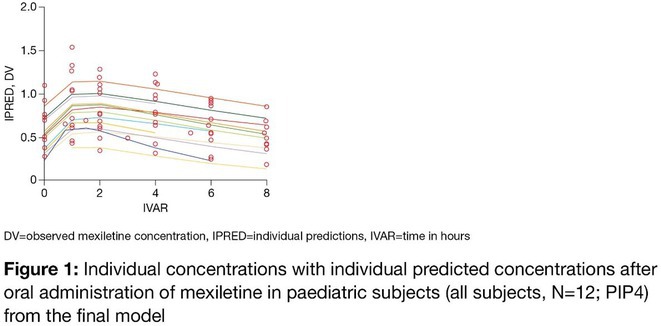

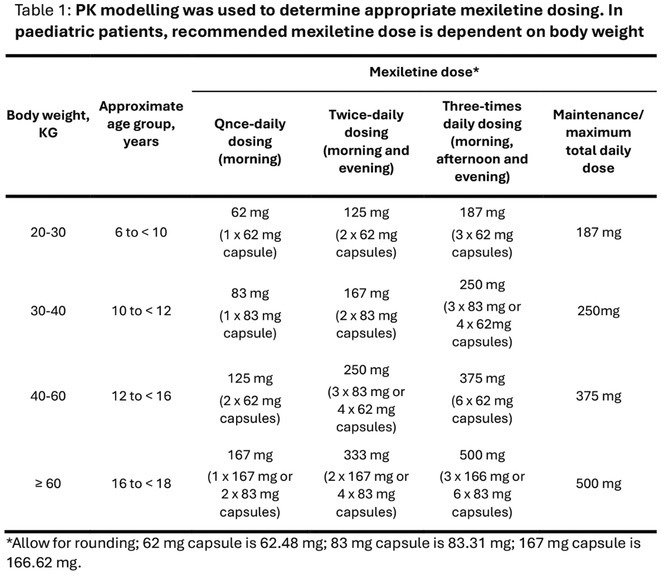

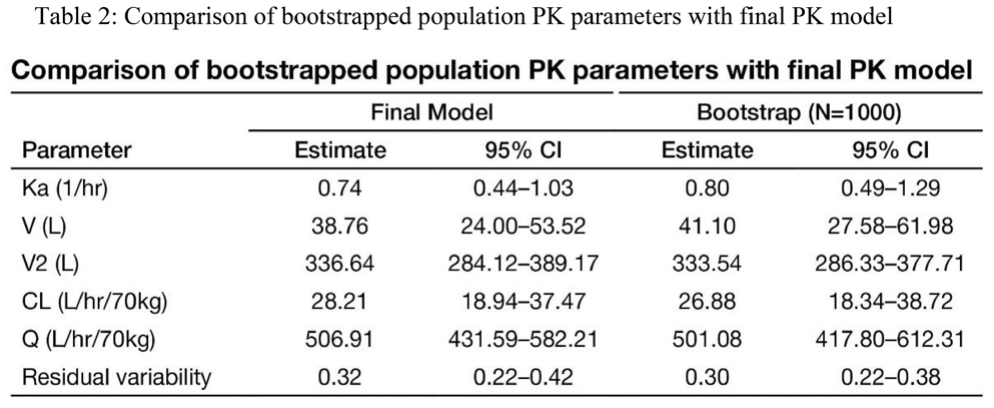

**Conclusion:** No unexpected safety findings were observed: safety profile was consistent with NaMuscla® SMPC. No events resulted in mexiletine discontinuation. PK analyses confirm paediatric mexiletine dosing.

**Disclosure:** CB; AI; HP; NA: consultancy fees from Lupin AZ‐W: Lupin employee.

## EPO‐467

### Comprehensive analysis of efgartigimod: The real‐world safety evaluation for myasthenia gravis from the FAERS database

#### Y. Zhou

##### Department of Neurology, Second Affiliated Hospital of Army Medical University, Chong Qing, China

**Background and Aims:** Efgartigimod, a neonatal Fc receptor (FcRn) antagonist, is used to treat myasthenia gravis (MG). As the FDA's first approved FcRn antagonist for the treatment of MG, comprehending its post‐marketing safety evaluation in the real‐world context is essential.

**Methods:** This research collected data on efgartigimod in the real‐world from the FAERS database from the fourth quarter of 2021 to the second quarter of 2024. To disproportionally analyze the adverse events (AEs) related to efgartigimod, we employed the methods of reporting odds ratio (ROR), proportional reporting ratio (PRR), multi‐item gamma Poisson shrinker (MGPS), and Bayesian confidence propagation neural network (BCPNN). We used the Weibull distribution to model the risk of AEs over time.

**Results:** The study analyzed 12,757 AE reports related to efgartigimod. The dataset revealed a higher incidence of reports from females compared to males, with the peak reporting observed in the 65 to 85 years age cohort. Some adverse reactions were documented in the instructions, such as Dyspnea, Urinary tract infection, Headache, Feeling abnormal, and Respiratory tract infection. In the meantime, we also identified some potential adverse reactions which were not mentioned in the instructions, including Nausea, Diarrhea, Nasopharyngitis, Arthralgia, Herpes zoster, Influenza, and so on.

**Conclusion:** Our study found some new AE signals of efgartigimod and might enhance clinical surveillance and risk detection. However, because of the limitations of the data sources and analysis methods, these results require additional validation through extensive data analysis and ongoing research to further investigate and confirm.

**Disclosure:** Nothing to disclose.

## EPO‐468

### Efficacy of nusinersen treatment in type 1, 2, and 3 spinal muscular atrophy: Real‐world data from a single‐center study

#### A. Lemska; P. Ruminski; J. Szymarek; S. Studzińska; M. Mazurkiewicz‐Bełdzinska

##### Department of Developmental Neurology, Medical University of Gdańsk, Poland

**Background and Aims:** Spinal muscular atrophy (SMA) is a genetic neuromuscular disorder caused by the absence of the SMN1 gene, leading to muscle weakness and atrophy. It is classified into types 0‐4, based on symptom onset and severity. Recent treatments, including nusinersen, onasemnogene abeparvovec, and risdiplam, have significantly improved SMA prognosis. This study evaluated the safety and efficacy of nusinersen in pediatric SMA types 1, 2, and 3 in a real‐world setting.

**Methods:** His prospective, observational, single‐center study involved 23 pediatric patients with genetically confirmed SMA over a 22‐month period. Participants received intrathecal nusinersen loading doses followed by maintenance doses. Functional assessments were made using the CHOP‐INTEND scale, and clinical data were collected during routine visits. Adverse events were also recorded.

**Results:** Of the 37 initial patients, 23 were analyzed due to treatment changes. Significant improvements in CHOP‐INTEND scores were seen, with an average increase of 4.2 points at 6 months, rising to 17.8 points at 22 months. By study end, all patients showed stabilization or improvement, with significant clinical progress in several. Nusinersen was well‐tolerated, with post‐lumbar puncture headache and back pain as common adverse events.

**Conclusion:** Nusinersen significantly improves motor function in pediatric SMA types 1, 2, and 3. Early and ongoing treatment is crucial for sustained improvements, supporting nusinersen as an effective therapy. Further research is needed to optimize long‐term outcomes.

**Disclosure:** The authors declare no conflicts of interest.

## EPO‐469

### Abstract withdrawn

## EPO‐470

### Sustained minimal symptom expression in generalised myasthenia gravis: A 120‐week post hoc analysis of RAISE‐XT

#### C. Hewamadduma ^1^; S. Bresch^2^; M. Freimer^3^; R. Juntas‐Morales^4^; M. Leite^5^; A. Maniaol^6^; K. Utsugisawa^7^; T. Vu^8^; M. Weiss^9^; B. Boroojerdi^10^; F. Grimson^11^; N. Savic^12^; J. Howard^13^

##### ^1^Academic Neuroscience Unit, Sheffield Teaching Hospitals NHS Foundation Trust & Sheffield Institute for Translational Neuroscience (SITraN), University of Sheffield, Sheffield, UK; ^2^Service de Neurologie, Hospital Pasteur, Centre Hospitalier Universitaire de Nice, Nice, France; ^3^Department of Neurology, The Ohio State University Wexner Medical Center, Columbus, USA; ^4^Department of Neurology, Vall d’Hebron University Hospital, Passeig de la Vall d’Hebron, Barcelona, Spain; ^5^Nuffield Department of Clinical Neurosciences, University of Oxford, Oxford, UK; ^6^Department of Neurology, Oslo University Hospital, Oslo, Norway; ^7^Department of Neurology, Hanamaki General Hospital, Hanamaki, Japan; ^8^Department of Neurology, University of South Florida Morsani College of Medicine, Tampa, USA; ^9^Department of Neurology, University of Washington Medical Center, Seattle, USA; ^10^UCB, Monheim, Germany; ^11^UCB, Slough, UK; ^12^UCB, Bulle, Switzerland; ^13^Department of Neurology, The University of North Carolina at Chapel Hill, Chapel Hill, USA

**Background and Aims:** Minimal symptom expression (MSE), defined as a myasthenia gravis activities of daily living (MG‐ADL) score of 0 or 1, is a rigorous measure of therapeutic efficacy in myasthenia gravis (MG). This post hoc analysis of RAISE‐XT (NCT04225871), a Phase 3, open‐label extension study of the complement component 5 inhibitor zilucoplan, assessed the durability of MSE response.

**Methods:** Adults with anti‐acetylcholine receptor antibody‐positive generalised MG who completed a qualifying double‐blind, placebo‐controlled study (NCT03315130/NCT04115293 [RAISE]) could opt to enter RAISE‐XT and self‐administer once‐daily subcutaneous injections of zilucoplan 0.3 mg/kg. The cumulative proportion of patients who achieved MSE (MG‐ADL score of 0 or 1 without rescue therapy) at any time during zilucoplan treatment up to Week 120 and the proportion of time spent in MSE up to Week 120 were assessed post hoc (interim data cut‐off: 11 November 2023).

**Results:** Of 200 patients enrolled in RAISE‐XT, 183 received zilucoplan 0.3mg/kg or placebo in the double‐blind studies. The cumulative proportion of patients who achieved MSE at any time from the start of zilucoplan treatment up to Week 120 in the zilucoplan 0.3 mg/kg/zilucoplan 0.3 mg/kg and placebo/zilucoplan 0.3mg/kg groups was 61% and 64%, respectively. After first achieving MSE during zilucoplan treatment, patients maintained their MSE response for a median (range) of 80.8% (0.8–100.0%) of their remaining time in the study up to Week 120. Treatment‐emergent adverse events were experienced by 97.0% (*n* = 194/200) of patients; most were mild or moderate.

**Conclusion:** Zilucoplan demonstrated sustained efficacy, as shown by maintenance of MSE response up to 120 weeks of treatment.

**Disclosure:** This study was funded by UCB. Babak Boroojerdi, Fiona Grimson and Natasa Savic are employees and shareholders of UCB. Full disclosure of all industry relationships will be made during congress presentation if accepted.

## EPO‐471

### Real‐world and recent clinical trials in NDM show consistent myotonia improvements with mexiletine

#### E. Matthews ^1^; S. Vicart^2^; V. Vivekanandam^3^; S. Sedehizadeh^4^; A. Rosenbohm^5^; C. Tard^6^; D. Jayaseelan^3^; C. Schneider‐Gold^7^; J. Hogrel^8^; A. Zozyla‐Weidenfeller^9^

##### ^1^Neurosciences and Cell Biology Research Institute, St George's University of London, London, UK; ^2^Assistance Publique‐Hôpitaux de Paris, Sorbonne Université, INSERM, Service of Neuro‐Myology, Muscle Channelopathies Reference Center and UMR 974, Institute of Myology, University Hospital Pitié‐Salpêtrière, Paris, France; ^3^Centre for Neuromuscular Disorders, The National Hospital for Neurology and Neurosurgery, London, UK; ^4^Nottingham University Hospitals NHS Trust, Nottingham, UK; ^5^Universitätsklinikum Ulm, Klinik für Neurologie, Ulm, Germany; ^6^CHRU Lille, Lille, France; ^7^St. Josef‐Hospital Klinikum der Ruhr Universität Bochum, Bochum, Germany; ^8^Institute of Myology, Paris, France; ^9^Lupin EMEA, Zug, Switzerland

**Background and Aims:** Studies evaluating mexiletine treatment for myotonia are generating a growing body of patient‐reported evidence: data encompass subjective parameters of physical health/‐quality‐of‐life (QoL) that inform real‐world mexiletine use. However, individual studies contain low participant numbers and use different methodologies. To investigate signals of mexiletine efficacy, we reviewed key outcomes reported in three datasets evaluating treatment of adults with non‐dystrophic myotonia (NDM): MYOMEX (N = 25), MEND (N = 60) and the Post Authorisation Safety Study (PASS; *N* = 53).

**Methods:** Study characteristics were compared (Table 1). Spearman correlations between baseline and post‐mexiletine treatment data for myotonia‐associated symptoms (stiffness [VAS/daily IVR]; locking and pain [INQoL, SF‐36, respectively]; myotonia behaviour scale, MBS] were investigated. Changes were tested using the Wilcoxon signed rank test.

**Results:** Despite between‐study methodological differences, including absence of/variation in comparator arms, consistent benefits were observed in patients receiving mexiletine. Reduction in patient‐reported stiffness (on VAS/IVR) and improved QoL (e.g. reduction in locking/pain [INQOL] or bodily pain [SF‐36]) were seen across the studies: (Tables 2, 3). Notably, MYOMEX data showed that although patients still reported locking at study end, its overall impact on QoL significantly reduced. Some PROs strongly correlated with baseline VAS stiffness assessment. Statistical changes were observed following mexiletine treatment e.g. VAS and INQoL locking domain; *p* = 0.0249 and *p* = 0.0154, respectively (PASS).

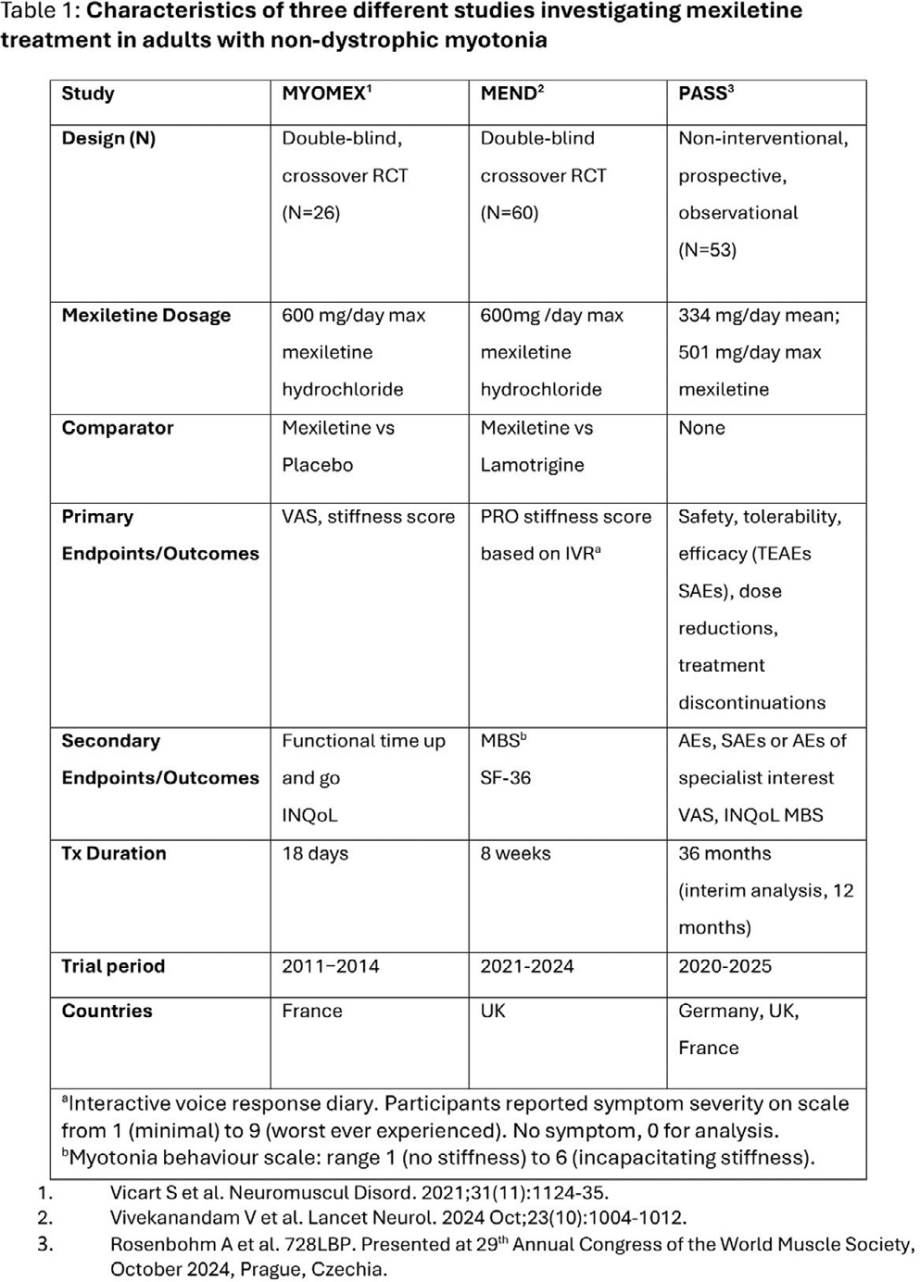

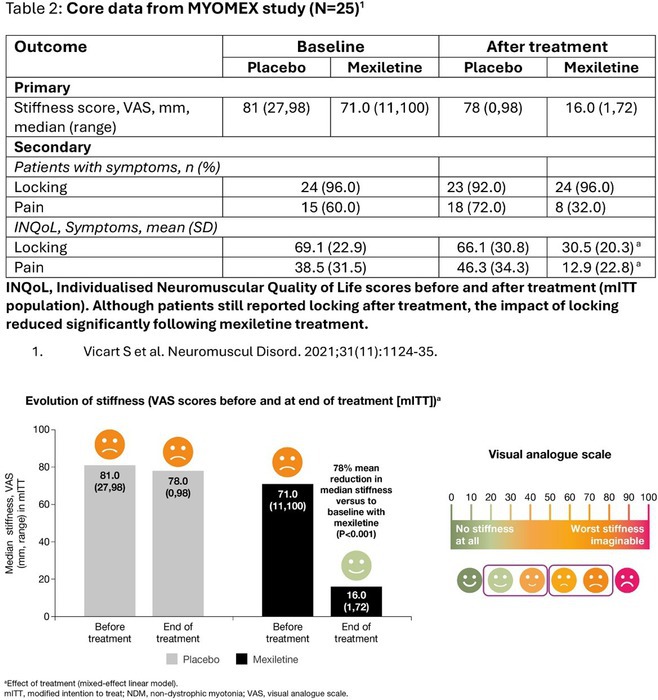

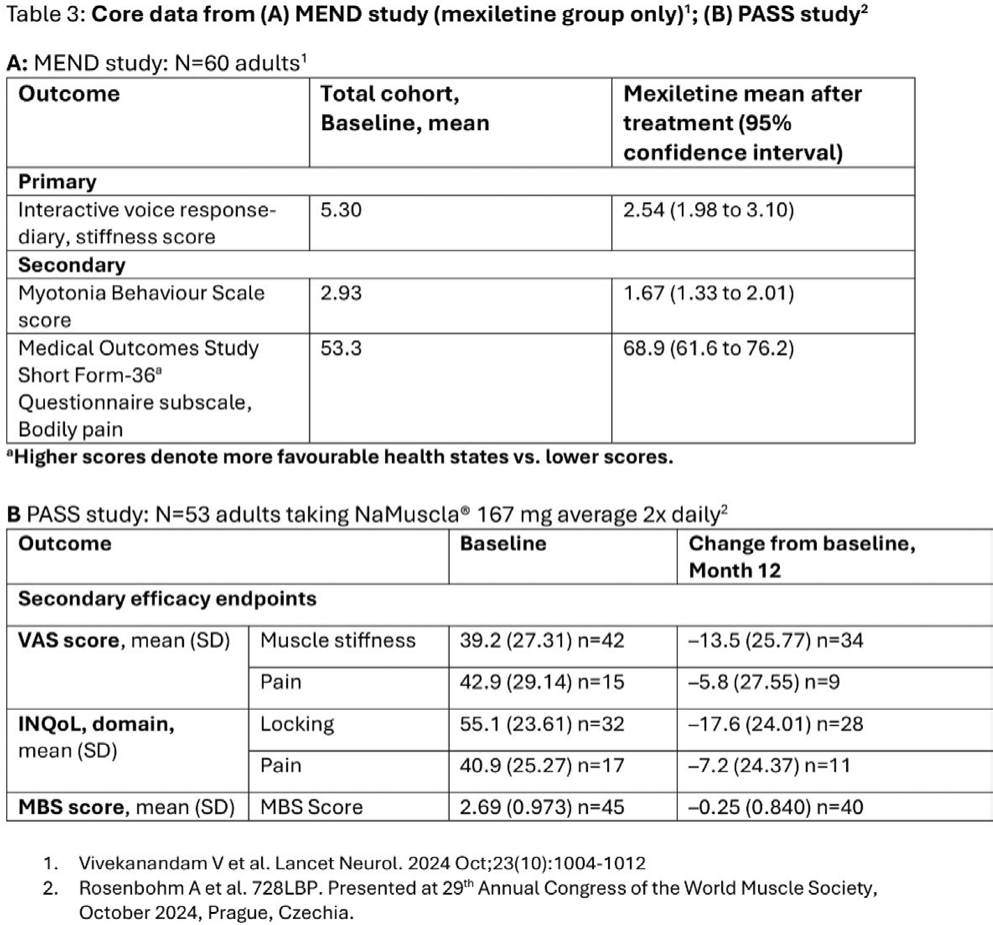

**Conclusion:** Together, evidence from 3 studies (*N* = 138 adults with NDM) illustrate the consistent impact of mexiletine on myotonia‐associated patient‐reported outcomes, especially stiffness and locking. Data strengthen the evidence base for mexiletine treatment, further validating the meaningful QoL benefits that symptom improvements bring to people with myotonia.

**Disclosure:** EM, SV, VV, SS, AR, CT, DJ, CS‐GG, J‐YH: Consultancy fees from Lupin AZ‐W: Employee of Lupin.

## EPO‐472

### Glial fibrillary alfa protein level as a potential biomarker in patients with myasthenia gravis

#### E. Saluvēra ^1^; A. Grosmane^2^; M. Roddate^2^; V. Krutovs^2^; I. Glāzere^2^; G. Ķauķe^2^; V. Žukova^2^; I. Roze^2^; K. Blennow^3^; H. Zetterberg^3^; N. Kurjāne^2^; V. Ķēniņa^2^

##### ^1^Rīga Stradiņš University, Latvia; ^2^Rīga Stradiņš University, Latvia; Pauls Stradiņš Clinical University Hospital, Department of Neurology, Latvia; ^3^Department of Psychiatry and Neurochemistry, Institute of Neuroscience and Physiology, the Sahlgrenska Academy at the University of Gothenburg, Mölndal, Sweden; Clinical Neurochemistry Laboratory, Sahlgrenska University Hospital, Mölndal, Sweden

**Background and Aims:** At present, no reliable biomarkers are available that correlate with myasthenia gravis (MG) disease severity, highlighting the need for novel biomarkers to evaluate both disease progression and treatment efficacy. Plasma glial fibrillary acidic protein (GFAP) is a protein that is not only expressed in central nervous system, but also in denervated neuromuscular junctions by terminal Schwann cells. This study aims to investigate GFAP concentration in the MG patient group and compare it to the control group.

**Methods:** The study included 48 patients diagnosed with MG and 40 healthy controls. The disease clinical classification was based on the MGFA classification. The blood samples from both groups were taken during outpatient visits in 2024 and measured with a Simoa.

**Results:** There were 15 males and 33 females with mean age 46.0(SD ± 10.8) years, mean disease duration 102.8 (SD ± 96.1) months in the MG patient group. Control group consisted of 11 males and 29 females with mean age 41.5 (SD ± 11.1) years. Median sGFAP concentration was 87.0 pg/mL (IQR = 61.5 pg/mL) in MG patient group and 87.5 pg/mL (IQR = 48.5) in control group. There was no significant difference between both groups (*U* = 870.500, *p* = 0.668). Analysing the association between sGFAP (H = 9.452, *p* = 0.150) and severity score MGFA no correlations were found.

**Conclusion:** There is no significant difference in sGFAP levels between patient group and control group. No correlation was observed between sGFAP levels and disease severity. Therefore, sGFAP could not serve as a potential biomarker also it does not appear to be effective for assessing disease severity or monitoring treatment efficacy.

**Disclosure:** Nothing to disclose.

## EPO‐473

### Myasthenic crisis – 15‐years' single neuromuscular center experience

#### E. Sobieszczuk; P. Szczudlik; K. Badowski; A. Opuchlik; B. Szyluk; C. Rajczewska‐Oleszkiewicz; A. Kostera‐Pruszczyk

##### Department of Neurology, Medical University of Warsaw, Warsaw, Poland, ERN EURO‐NMD

**Background and Aims:** Myasthenic crisis (MC) is a life‐threatening episode developing in 15–20% MG patients.

**Methods:** Retrospective analysis of all MC patients between 2009 and 2024.

**Results:** There were 89 MCs in 77 MG patients (F 48.3%); 14% had >2 MCs. Mean age at MC was 63.6 + 18.3 years, 73% had late onset MG. 62.9% had AChR‐MG, 5.6% MuSK‐MG, 10.1% had history of thymoma. Mean MG duration before MC was 50.7 + 72.3 months. In four (4.8%) MC was the initial MG presentation. 18 (20%) of cases had previous MC; they were younger at MG onset, had longer ICU stay (*p* < 0.05) and MG duration (*p* < 0.01). Before MC, 60.7% of patients were treated with glucocorticosteroids, 25.8% with nonsteroidal immunosuppressants. 48.3% patients received IVIg, 34.8% PLEX, 11.2% both. Mean length of mechanical ventilation (MV) was 12.9 + 8.6 days. Mean length of ICU stay was 24.6 + 18.5 days, longer in treated with IVIG (28.6 + 24), IVIG+PLEX (30.8 + 14.8) than with PLEX (18.5 + 6.8), *p* < 0.05. In 38.2% MC was preceded by infection, 13.5% therapy change. CRP and WBC were increased at admission in 50.6 and 41.6% respectively; 22.5% had anaemia, 22.5% bacteriuria. MC was complicated by pneumonia 33.7%, urinary infections 19.1%, myocardiac infarct (9%), pulmonary embolism (7.9%), critical care neuropathy (5.6%). In 3.4% PEG, 4.5% tracheostomy, 4.5% nasogastric tube were maintained after extubation. Mortality was 4.5% (*N* = 4), age at death 77.5 + 4.8 years. Higher WBC count at admission predicted longer MV.

**Conclusion:** MC in gMG patients is most often triggered by infection or therapy change, with 4.5% mortality in our cohort.

**Disclosure:** Nothing to disclose.

## EPO‐474

### Myasthenia gravis activities of daily living and myasthenia gravis quality‐of‐life questionnaires: Estonian versions

#### L. Väli ^1^; A. Perve^2^

##### ^1^Department of Neurology, University of Tartu, Tartu, Estonia; ^2^University of Tartu, Tartu, Estonia

**Background and Aims:** Evaluation of quality of life and impact of the disease on the daily life has become essential in healthcare. The aim of the study was to translate, adapt and validate the myasthenia gravis‐specific activities of daily living (MG‐ADL) scale and the 15‐item myasthenia gravis quality‐of‐life (MG‐QOL15) questionnaire into the Estonian language.

**Methods:** Translation and adaption of the questionnaires were performed. The validation protocol included the MG‐QOL15, MG‐ADL, myasthenia gravis composite score (MGCS) and quantitative myasthenia gravis (QMG) score. We used the Cronbach α to test internal consistency of the questionnaires, Cohen's weighted kappa to test short‐term test–retest reproducibility, and Spearman's correlation between the questionnaries and MGCS and QMG score for construct validity.

**Results:** Twenty‐six patients were enrolled into the study. The mean MG‐ADL score was 5.9 ± 3.5, with *α* = 0.76 and test–retest scores 0.52–0.87. The mean MG‐QOL15 score was 22.2 ± 15.4, with *α* = 0.97 and test–retest scores 0.57–0.89. The MG‐ADL showed strong correlation with the MG‐QOL15 (*r* = 0.77, *p* < 0.0001), the MGCS (*r* = 0.82, *p* < 0.0001) and moderate with the QMG score (*r* = 0.64, *p* = 0.0002). The MG‐QOL15 had moderate correlation with the MGCS (*r* = 0.63, *p* = 0.0003) and the QMG score (*r* = 0.56, *p* = 0.002).

**Figure:**
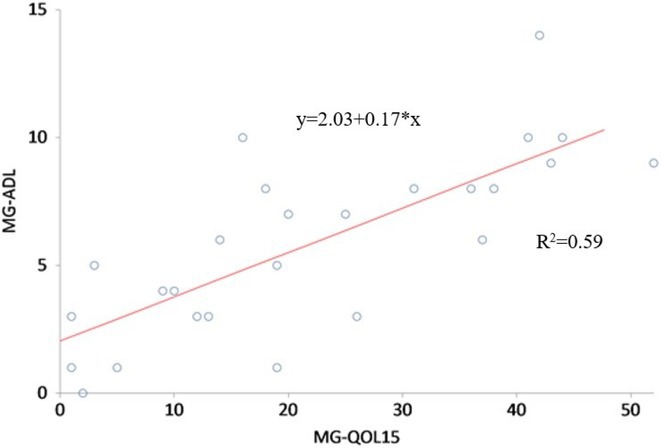
**FIGURE 1** Correlation between MG‐ADL and MG‐QOL15.

**Figure:**
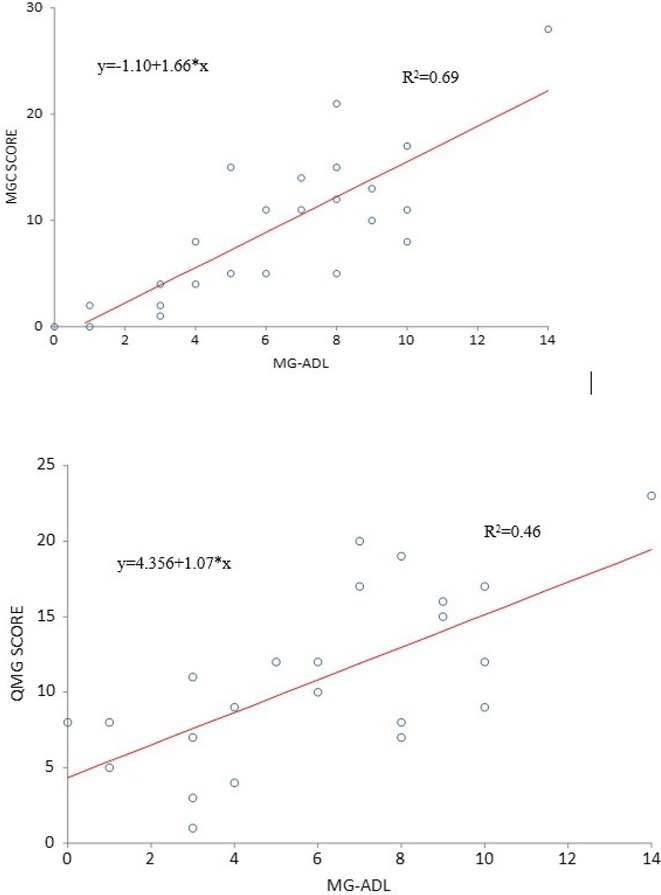
**FIGURE 2** Relationships between (a) MG‐ADL and myasthenia gravis composite (MGC) score and (b) MG‐ADL and quantitative myasthenia gravis (QMG) score.

**Figure:**
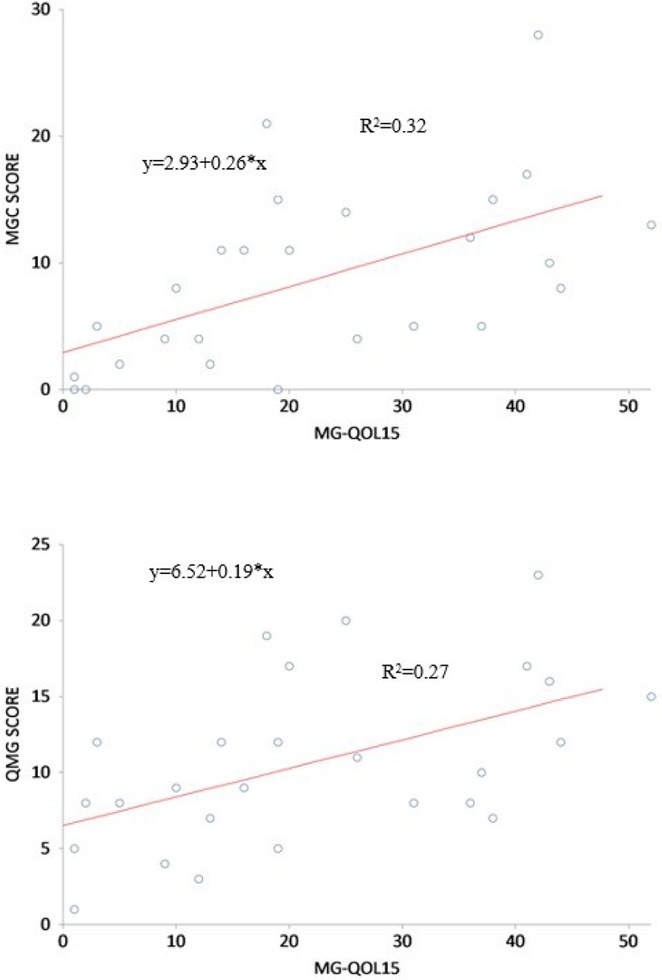
**FIGURE 3** Relationships between (a) MG‐QOL15 and myasthenia gravis composite (MGC) score (b) MG‐QOL15 and quantitative myasthenia gravis (QMG) score.

**Conclusion:** The Estonian versions of the MG‐ADL and MG‐QOL15 are valid and reliable self‐reported scales for monitoring patients in clinical practice, their disease severity and the impact of the disease on their lives.

**Disclosure:** Nothing to disclose.

## EPO‐475

### Effect of zilucoplan on rescue therapy use in patients with generalised myasthenia gravis: RAISE‐XT post hoc analysis

#### M. Leite ^1^; S. Bresch^2^; C. Hewamadduma^3^; A. Maniaol^4^; J. Schmidt^5^; K. Utsugisawa^6^; B. Boroojerdi^7^; F. Grimson^8^; N. Savic^9^; J. Howard^10^

##### ^1^Nuffield Department of Clinical Neurosciences, University of Oxford, Oxford, UK; ^2^Service de Neurologie, Hospital Pasteur, Centre Hospitalier Universitaire de Nice, Nice, France; ^3^Academic Neuroscience Unit, Sheffield Teaching Hospitals NHS Foundation Trust & Sheffield Institute for Translational Neuroscience (SITraN), University of Sheffield, Sheffield, UK; ^4^Department of Neurology, Oslo University Hospital, Oslo, Norway; ^5^Department of Neurology and Pain Therapy, Immanuel Clinic Rüdersdorf, University Hospital Brandenburg Medical School, Berlin, Germany; ^6^Department of Neurology, Hanamaki General Hospital, Hanamaki, Japan; ^7^UCB, Monheim, Germany; ^8^UCB, Slough, UK; ^9^UCB, Bulle, Switzerland; ^10^Department of Neurology, The University of North Carolina at Chapel Hill, Chapel Hill, USA

**Background and Aims:** In the Phase 3 RAISE study (NCT04115293), zilucoplan, a complement component 5 inhibitor, demonstrated clinically meaningful improvements in myasthenia gravis (MG)‐specific outcomes versus placebo in patients with anti‐acetylcholine receptor antibody‐positive generalised MG (gMG); improvements were sustained during long‐term use in the ongoing open‐label extension study, RAISE‐XT (NCT04225871). This post hoc analysis assessed rescue therapy use with long‐term zilucoplan treatment.

**Methods:** Adults who completed a qualifying double‐blind, placebo‐controlled study (Phase 2 [NCT03315130] or RAISE) could enter RAISE‐XT to self‐administer daily treatment.subcutaneous zilucoplan 0.3mg/kg. If the investigator deemed rescue therapy necessary, patients could receive intravenous immunoglobulin or plasma exchange concomitantly with zilucoplan. A cycle of rescue was defined as > = 1 treatments within a 7‐day period. Incidence of rescue therapy per 100 patient‐years at risk (PYAR) was assessed post hoc. The primary endpoint was incidence of treatment‐emergent adverse events (TEAEs; data cut‐off: 11 November 2023).

**Results:** During the double‐blind periods, rate of rescue therapy use was 31.19 and 78.16 events per 100 PYAR for patients who received zilucoplan 0.3mg/kg (*n* = 4/101) and placebo (*n* = 13/103), respectively. During RAISE‐XT (median [range] exposure: 2.2 [0.1–5.6] years; *N* = 200), rate of rescue therapy use was 22.64 events per 100 PYAR. Overall, 17.2% (*n* = 16/93) of patients who received zilucoplan 0.3mg/kg and 20.0% (*n* = 18/90) of patients who received placebo in the double‐blind studies received rescue therapy during RAISE‐XT. TEAEs occurred in 97.0% (*n* = 194/200) of patients.

**Conclusion:** Rescue therapy use was lower with zilucoplan versus placebo in the double‐blind studies. Improvement in gMG disease fluctuations requiring rescue therapy was sustained with long‐term zilucoplan treatment.

**Disclosure:** This study was funded by UCB. Babak Boroojerdi, Fiona Grimson and Natasa Savic are employees and shareholders of UCB. Full disclosure of all industry relationships will be made during congress presentation if accepted.

## EPO‐476

### A phase 1/2 study design to assess safety and efficacy of YTB323 in treatment‐resistant generalised myasthenia gravis

#### M. Meriggioli^1^; M. Stangel ^2^; J. Whittle^1^; J. F. Howard Jr.^3^

##### ^1^Novartis Biomedical Research, Cambridge, USA; ^2^Novartis Biomedical Research, Basel, Switzerland; ^3^Department of Neurology, University of North Carolina School of Medicine, Chapel Hill, USA

**Background and Aims:** Generalised myasthenia gravis (gMG) is an IgG autoantibody‐mediated chronic autoimmune disorder affecting the neuromuscular junction, leading to fatigable muscle weakness variably involving ocular, bulbar, respiratory and limb muscles. B‐cells are implicated in gMG pathophysiology as they produce IgG autoantibodies. YTB323 is an investigational, autologous CD19‐directed chimeric antigen receptor (CAR)‐T cell therapy which targets a broad population of the B‐cell lineage. Here, we report the design of a phase 1/2 study to assess the safety, efficacy, and cellular kinetics of YTB323 in treatment‐resistant gMG patients.

**Methods:** This is an open‐label, multicentre, non‐confirmatory study, with a single‐dose design and a sentinel cohort of 3 patients, followed by an expansion cohort of 12 patients. The study will enrol patients aged 18–65 years, diagnosed with gMG (Myasthenia Gravis Foundation of America disease class III–IVa) who are either acetylcholine receptor positive (AChR+), or muscle‐specific kinase positive (MuSK+), with a Myasthenia Gravis Activities of Daily Living (MG‐ADL) score ≥6, and persistent MG symptoms despite adequate treatment courses with at least two different non‐steroidal immunosuppressive drugs. The primary endpoint is the frequency and severity of adverse events and the change from baseline in safety parameters. Key secondary endpoints include an assessment of the MG‐ADL and Quantitative MG (QMG) scores, and the pharmacokinetics of YTB323.

**Results:** Approximately 15 participants with gMG will be treated with YTB323. Following a 2‐year core study, there will be a long‐term follow‐up study for 13 years.

**Conclusion:** This study will provide the scientific evidence needed for further development of YTB323 in treatment‐resistant gMG patients.

**Disclosure:** Matthew Meriggioli, Martin Stangel and JoAnn Whittle are Novartis employees. Prof. James Howard Jr. has received honoraria/consulting fees from AcademicCME, Alexion AstraZeneca Rare Disease, Amgen, Biohaven Ltd, CheckRare CME, CoreEvitas, Curie.bio, Medscape CME, Merck EMB Serono, Novartis Pharma, PeerView CME, Physicians' Education Resource (PER) CME, PlatformQ CME, Regeneron Pharmaceuticals, Sanofi US, TG Therapeutics, Toleranzia AB, UCB Pharma, and Zai Labs. Prof. Howard has also received personal compensation for participating on advisory boards with Alexion AstraZeneca Rare Disease, argenx, Novartis, UCB (Ra Pharma), Merck EMB Serono, Amgen, Sanofi, Toleranzia AB.

## EPO‐477

### Real‐world utilization of myasthenia gravis treatments and the influence of demographics on therapy selection

#### N. Streicher

##### Department of Neurology, Georgetown University, Washington DC, USA

**Background and Aims:** Treatment approaches for myasthenia gravis (MG) vary based on provider preferences, healthcare access, and disease severity. However, demographic factors such as age, gender, race, and ethnicity may also impact treatment selection. Understanding these trends is essential for identifying disparities and optimizing care.

**Methods:** A retrospective chart review was conducted using electronic medical records from the MedStar Neurology and Neurosurgery network. Data included the use of corticosteroids, intravenous immunoglobulin (IVIG), C5 inhibitors, FcRn inhibitors, B‐cell depleting therapies, and antimetabolites.

**Results:** Corticosteroids were the most prescribed therapy, followed by IVIG, while C5 and FcRn inhibitors were used less frequently. Older patients were more likely to receive corticosteroids and antimetabolites, while younger patients were more likely to receive C5 and FcRn inhibitors. Women were more frequently treated with multiple therapies. White patients had higher IVIG utilization and were more likely to receive combination therapies. Hispanic and Black patients were less likely to receive advanced therapies.

**Conclusion:** Real‐world MG treatment utilization shows significant demographic disparities. Corticosteroids remain the most widely used therapy, while IVIG, C5 inhibitors, and FcRn inhibitors were prescribed less frequently in Hispanic and Black patients, raising concerns about healthcare access. White and non‐Hispanic patients had greater access to IVIG and combination therapies. The influence of age and gender was also evident, with older patients more likely to receive corticosteroids and younger patients more likely to receive newer biologic treatments. These findings highlight the need to investigate the causes of these disparities and develop strategies for equitable MG treatment.

**Disclosure:** Nothing to disclose.

## EPO‐478

### Overlap syndrome: Myositis, myocarditis, and myasthenia gravis secondary to checkpoint inhibitor treatment: Case series

#### R. Sanjinez Arana

##### Neurology, Hospital Italuano de Buenos Aires, Buenos Aires, Argentina

**Background and Aims:** Checkpoint inhibitors (ICP) are now standard therapy for advanced cancers, but can trigger autoimmune adverse effects. A rare but severe overlap of myositis, myocarditis, and myasthenia gravis (MG), termed the “3M Triad,” has been reported, carrying a poor prognosis.

**Methods:** We describe 2 clinical cases of patients who developed myositis, myocarditis, and MG following ICP infusion.

**Results:** Patient 1: A 73‐year‐old man receiving durvalumab for advanced squamous cell lung cancer. Following the initial injection, he suffered left eyelid ptosis, pain, neck muscle weakness, and dyspnea 27 days later In suspected ICP‐related myasthenic syndrome, he received 2 mg/kg immunoglobulin and 1 mg/kg prednisone. He was admitted to the ICU for symptoms and lab results (CPK 1800, troponins 380, and fresh third‐degree atrioventricular block). Myocarditis suspicions led to a temporary pacemaker and greater corticosteroid doses. Intubation and forced breathing killed him 14 days after admission from bacterial and cardiac issues. Patient 2: A 76‐year‐old pembrolizumab‐treated advanced ductal breast cancer patient. 23 days after the injection, she suffered right eyelid ptosis, pain, lower limb weakness, and dyspnea. The ECG indicated ST‐segment depression and elevated troponins. Coronary angiography removed significant obstructions. Symptom progression sent her to the cardiac ICU. Strong corticosteroids and 2 mg/kg immunoglobulin were given for five days. Despite treatment, her fragility and troponin levels increased, requiring mycophenolate and plasmapheresis. She died 25 days after admission.

**Conclusion:** Overlap syndrome of myositis, myocarditis, and myasthenia gravis is rare with ICP immunotherapy. Early diagnosis is essential for complete therapy due to its high mortality.

**Disclosure:** Nothing to disclose.

## EPO‐479

### Clinical implications of a very late presentation in myasthenia gravis – Real world experience in a tertiary center

#### A. Fernandes; G. Nadais; F. Silveira; L. Braz; M. João Pinto

##### Neurology Service, Local Health Unit of São João, Porto, Portugal

**Background and Aims:** Myasthenia Gravis (MG) is an immune‐mediated disease with a bimodal incidence: early‐onset (<50 years) and late‐onset (>50 years). Recently, a subgroup with very late‐onset (≥65 years) was described; however, its clinical relevance concerning disease course and prognosis remain uncertain.

**Methods:** We conducted a retrospective cohort study comprising all patients diagnosed with MG currently followed in the neuromuscular disorders unit consultation of a tertiary center. Clinical and paraclinical parameters were evaluated.

**Results:** Ninety‐two patients were included, of which 51 (55.4%) were female, with a current mean age of 59.9 ± 18.0 years. Concerning the age of onset, 23 (25.6%) patients were classified as very late‐onset. Gender distribution and number of patients with minimal manifestation status or better did not statistically differ between the late and very late onset subgroups. Myasthenic crisis and refractoriness were more frequent in the late‐onset subgroup versus the very late‐onset, but no statistical difference was found (p = 0.221 and p = 0.650 respectively). We did not find statistically significant differences in acetylcholine receptor antibody titers between the groups. No significant difference was observed in the prevalence of autoimmune co‐pathology among the subgroups.

**Conclusion:** Our study suggests that the recently established subgroup of very late‐onset may not be clinically distinct from late‐onset MG, as these patients seem to exhibit a similar clinical trajectory.

**Disclosure:** All authors: No conflicts of interest to report.

## Neuroimmunology 2

## EPO‐480

### Correlation of serum cytokines in patients with epilepsy of unknown etiology as a marker of neuroinflammation

#### Y. Lopez Moreno^1^; A. Sarmiento Pita ^1^; P. Cabezudo García^1^; G. García Martín^1^; N. Lundhal^2^; P. Serrano Castro^1^

##### ^1^Hospital Regional Universitario de Málaga, Málaga, Spain; ^2^Instituto de Investigación Biomédica de Málaga y Plataforma en Nanomedicina (IBIMA Plataforma BIONAND), Málaga, Spain

**Background and Aims:** Interest in epilepsy associated with autoimmunity (EAA) and the underlying immune‐mediated mechanism is increasing, given the possibility that, in these patients (often resistant to antiepileptic drugs), immunotherapy (IT) may offer a therapeutic opportunity. Additionally, aspects of immunity not mediated by antibodies, such as the role of cytokines in these patients, are starting to be studied.

**Methods:** A total of 119 patients were recruited between January and November 2023 in a multicenter study conducted in Andalusia. Serum levels of 5 cytokines related to neuroinflammation in epilepsy patients (IL1b, IL4, IL6, IL10, and TNF‐alpha) were measured using SIMOA, an ultrasensitive ELISA technique for analyzing molecules in biological samples.

**Results:** In patients with epilepsy associated with anti‐GAD 65 antibodies, a higher median of TNF‐alpha was observed compared to the rest of the sample (any anti‐GAD 65‐negative epilepsy), as well as compared to epilepsy cases without immunoreactivity (in which no antibodies were found in serum using advanced techniques such as indirect immunofluorescence on murine tissue). No differences were found in the median levels of cytokines in relation to drug resistance or absolute drug resistance (defined as failure of more than 6 antiepileptic drugs).

**Conclusion:** In patients with anti‐GAD65‐associated epilepsy, a significant increase in circulating TNF‐alpha was observed compared to other patients, suggesting a potential target for treatment. Further studies and analysis of other subgroups are needed to determine differences that will allow for better characterization of neuroinflammation and EAA.

**Disclosure:** Nothing to disclosure.

## EPO‐481

### Frequency of LRP4 antibodies in a consecutive cohort of suspected myasthenia gravis patients

#### A. Malvaso ^1^; P. Businaro^1^; S. Masciocchi^2^; C. Morandi^2^; S. Scaranzin^2^; E. Zardini^2^; D. Franciotta^3^; M. Gastaldi^2^

##### ^1^Department of Brain and Behavioral Sciences, Neuroimmunology Research Unit, IRCCS Mondino Foundation, University of Pavia, Pavia, Italy; ^2^Neuroimmunology Research Unit, IRCCS Mondino Foundation, Via Mondino 2, 27100, Pavia, Italy; ^3^UOM, Laboratory of Clinical Pathology, APSS, Santa Chiara Hospital, Trento, Italy

**Background and Aims:** Autoantibodies to the lipoprotein receptor‐related protein‐4 (anti‐LRP4) have been reported in a minority of patients with Myasthenia Gravis (MG), with potential pathogenic role and uncertain clinical relevance. However, LRP4 antibodies frequency varies substantially according to the type of assay used for their detection. We aim to investigate the prevalence of anti‐LRP4 in a consecutive cohort of patients with suspected MG.

**Methods:** A consecutive cohort of 333 suspected MG, compared to 68 disease controls (38 ALS and 30 NPH) and 55 healthy controls were tested. We implemented and validated both fixed and live cell‐based assays (CBA) expressing full‐length LRP4. All samples were tested in parallel with validated AchR and MUSK fixed and live‐CBA. Seronegative MG (SNMG) diagnosis was assessed through clinical history, electromyography and pyridostigmine response.

**Results:** The LRP4‐CBAs were validated by demonstrating their surface expression by staining of the live cells with a commercial antibody targeting extracellular epitopes of LRP4. Overall, 32% (*n* = 108) were positive for either anti‐AChR/MuSK, while 0/333 were positive for LRP4 as well as in the control groups. Among 225 triple‐negative patients, a diagnosis of SNMG was established in 11% (*n* = 26). The median age of SNMG at sampling was 63 years (range: 32–80), and 46% (*n* = 12) were female. Thirty percent (*n* = 8) of SNMG patients were collected at disease onset, and 58% (*n* = 15) had generalized MG.

**Conclusion:** Using our implemented and validated CBA, LRP4 antibodies seem to be exceedingly rare, thus questioning their clinical relevance in routine clinical practice. Standardization studies are warranted to understand the actual clinical impact of requesting LRP4 testing.

**Disclosure:** Nothing to disclose.

## EPO‐482

### Distinguishing NMOSD and MOGAD: A systematic review and a meta‐analysis of clinical and imaging differences

#### A. Rechtman; O. Zveik; T. Friedman‐Korn; A. Vaknin‐Dembinsky

##### Department of Neurology and Laboratory of Neuroimmunology and the Agnes‐Ginges Center for Neurogenetics, Hadassah‐Hebrew University Medical Center, Ein–Kerem, Israel

**Background and Aims:** This study systematically compares the clinical and imaging characteristics of neuromyelitis optica spectrum disorder (NMOSD) and myelin oligodendrocyte glycoprotein antibody‐associated disease (MOGAD) to identify distinguishing features that enhance diagnostic accuracy and inform therapeutic strategies.

**Methods:** A systematic review of PubMed was conducted, selecting studies with NMOSD and MOGAD patient cohorts (*n* > 5 per group). Data extraction focused on clinical and imaging parameters. Random‐effects models calculated odds ratios and standardized mean differences, with heterogeneity assessed using the *I* ^2^ statistic.

**Results:** A total of 2859 articles were screened, of which 131 provided relevant clinical data. NMOSD patients exhibited older age of onset and female predominance. MOGAD patients more frequently presented with bilateral optic neuritis and infectious prodromes, whereas NMOSD was associated with more frequent myelitis relapses, area postrema symptoms, and co‐existing autoimmune disorders. Ethnic differences were observed, with MOGAD patients being more likely Caucasian, while NMOSD had a higher proportion of African‐descendent patients. Imaging differences included a higher prevalence of area postrema and medulla lesions in NMOSD, whereas cerebellar and pontine lesions were more common in MOGAD. Juxtacortical lesions were predominant in MOGAD, while NMOSD showed a trend toward periventricular lesions.

**Figure:**
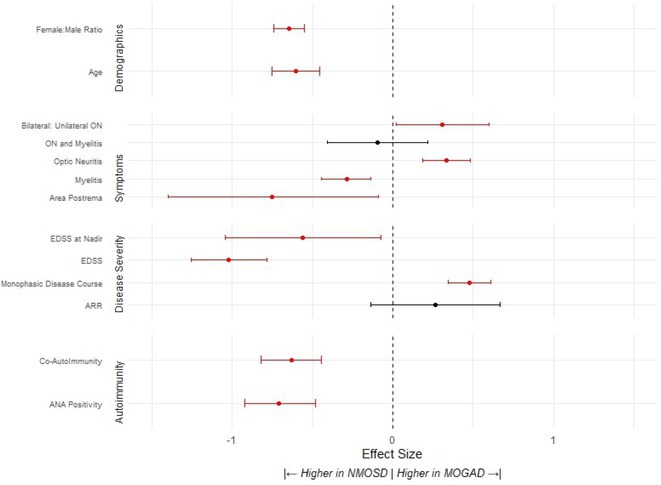
**FIGURE 1** Clinical Differences between MOGAD and NMOSD patients

**Figure:**
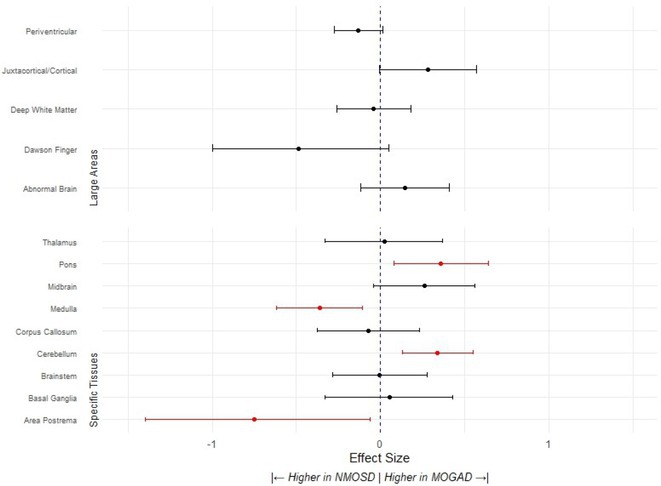
**FIGURE 2** Imaging Differences Between MOGAD and NMOSD patients

**Conclusion:** This meta‐analysis highlights distinct clinical and imaging patterns in NMOSD and MOGAD, reflecting significant differences in disease pathogenesis. These findings emphasize the need for tailored diagnostic and therapeutic approaches to address the unique characteristics of each disorder. A deeper understanding of these differences could provide valuable insights into the underlying pathophysiology, ultimately contributing to improved management and treatment strategies for both conditions.

**Disclosure:** Nothing to disclose.

## EPO‐483

### Clinical and serological characteristics of MOGAD: A retrospective single center cohort study

#### D. Cetinkaya Tezer^1^; I. Gungor Dogan^1^; B. Tasdelen ^1^; S. Kendirli Aslan^1^; F. Yadi^1^; O. Gulacti^2^; M. Yigit^2^; S. Demir^1^

##### ^1^Neurology Clinic, Sancaktepe Şehit İlhan Varank Training and Research Hospital, İstanbul, Turkey; ^2^My Gene and Cell Therapies Biotechnology, İstanbul, Turkey

**Background and Aims:** Myelin Oligodendrocyte Glycoprotein Antibody‐Associated Disorder (MOGAD) is a rare inflammatory demyelinating disease of the central nervous system. This study aims to address gaps in knowledge by examining the relationship between anti‐MOG IgG titers, demographic characteristics, and clinical variables in MOGAD patients.

**Methods:** A retrospective study was conducted with 61 MOGAD patients, diagnosed per the 2023 consensus criteria, monitored at the Neuroimmunology Clinic of Sancaktepe Research and Training Hospital. Associations between age, gender, anti‐MOG antibody levels (measured using an in‐house flow cytometric live cell‐based assay), cerebrospinal fluid (CSF) parameters, serological markers, and clinical features were analyzed.

**Results:** The cohort included 61 patients (32 females, 29 males) with a mean diagnosis age of 41.60 ± 13.21 years (excluding those under 18). Presentations included optic neuritis (ON) in 25 patients, myelitis in 17, and other subtypes in 19. Fourteen patients had at least one relapse. Anti‐MOG titers showed no significant associations with gender, age, relapse rates, or attack types. Male patients had significantly higher CSF protein levels (*p* = 0.013). ON relapsed in 80% of ON cases, and all myelitis relapses presented as myelitis. Tocilizumab had higher seroconversion rates to seronegativity compared to azathioprine and rituximab (*p* = 0.040).

**Conclusion:** This study highlights the heterogeneity of MOGAD and the potential role of tocilizumab as a superior therapeutic option. The relapse patterns suggest consistent clinical trajectories, particularly in ON and myelitis. However, the higher CSF protein levels in males may indicate gender‐related differences. The retrospective design and single‐center cohort limit generalizability, emphasizing the need for larger, multi‐center studies to validate these findings.

**Disclosure:** Nothing to disclose.

## EPO‐484

### Combined central and peripheral demyelination study and the relationship with neurofascin antibody and mog antibody

#### C. Turkok ^1^; S. Karsıdag^1^; M. Ozdag^1^; S. Demir^2^; R. Türkoglu^3^; A. Vural^4^; N. Bulbul^1^

##### ^1^Department of Neurology, Sultan Abdulhamid Han Training and Research Hospital, Istanbul, Turkey, ^2^Department of Neurology, Sancaktepe Prof. Dr. Ilhan Varank Training and Research Hospital, Istanbul, Turkey, ^3^Department of Neurology, Haydarpasa Numune Training and Research Hospital, Istanbul, Turkey, ^4^Department of Neurology, Koç University Hospital, Istanbul, Turkey

**Background and Aims:** This study aims to determine the rate of combined central and peripheral demyelination (CCPD), compare its clinical, radiological, and electrophysiological features with CNS demyelinating diseases and CIDP, and assess the role of Neurofascin‐155 (NF‐155) and myelin oligodendrocyte glycoprotein (MOG) antibodies.

**Methods:** We analyzed CNS demyelinating and CIDP patients, identifying CCPD cases. Neuroradiological imaging, laboratory findings, and electrophysiological exams were assessed. Hughes scores measured disability and treatment response in CCPD and CIDP groups, while EDSS was used for CNS cases. NF‐155 and MOG antibodies were tested using a live cell‐based assay. Statistical analysis was performed using SPSS.

**Results:** Among 122 patients, 10 had CCPD. While no significant age difference was found between CCPD and CNS groups, CCPD was more common in younger patients than CIDP (*p* < 0.05). CCPD showed distinct clinical features, with myelitis as the most frequent CNS involvement and variant CIDP as the most common PNS diagnosis. CCPD patients had higher spinal T2 lesion prevalence, elevated CSF protein, and OCB negativity (*p* < 0.05). Steroid/IVIG unresponsiveness was also significant (*p* < 0.05). Hughes scores improved post‐treatment (*p* < 0.05). MOG antibodies were detected in four CCPD patients and NF‐155 in two, linking them to CCPD.

**Conclusion:** CCPD presents unique yet overlapping features with CNS demyelinating diseases and CIDP. Anti‐MOG and anti‐NF‐155 antibodies contribute to its pathogenesis, highlighting the need for targeted diagnostic and therapeutic approaches.

**Disclosure:** Nothing to disclose.

## EPO‐485

### Assessment of a commercial tissue‐based assay for detecting neural surface antibodies in autoimmune encephalitis

#### C. Papi ^1^; C. Milano^2^; L. Arlettaz^3^; P. Businaro^4^; L. Marmolejo^5^; L. Naranjo^6^; E. Martinez‐Hernandez^5^; T. Armangué^7^; M. Guasp^5^; R. Ruiz Garcia^6^; M. Gastaldi^8^; R. Iorio^9^; L. Sabater^5^; F. Graus^10^; J. Dalmau^5^; M. Spatola^5^

##### ^1^Neuroimmunology Program, FRCB‐IDIBAPS, University of Barcelona, Spain; Caixa Research Institute (CRI), Barcelona, Spain; Department of Neuroscience, Catholic University of the Sacred Heart, Rome, Italy; ^2^Neuroimmunology Program, FRCB‐IDIBAPS, University of Barcelona, Spain; Caixa Research Institute (CRI), Barcelona, Spain; Department of Clinical and Experimental Medicine, University of Pisa, Pisa, Italy; ^3^Service d’Immunologie et Allergologie, Institut Central des Hôpitaux, Hôpital du Valais, Sion, Switzerland; ^4^Neuroimmunology Program, FRCB‐IDIBAPS, University of Barcelona, Spain; Department of Brain and Behavioral Sciences, University of Pavia, Pavia, Italy; Neuroimmunology Research Section, IRCCS Mondino Foundation, Pavia, Italy; ^5^Neuroimmunology Program, FRCB‐IDIBAPS, University of Barcelona, Spain; Caixa Research Institute (CRI), Barcelona, Spain; ^6^Immunology Service, Biomedical Diagnostic Center, Hospital Clínic, Barcelona, Spain; ^7^Neuroimmunology Program, FRCB‐IDIBAPS, University of Barcelona, Spain; Caixa Research Institute (CRI), Barcelona, Spain; Pediatric Neurology Unit, Neurology Department, Sant Joan de Deu Children's Hospital, Barcelona, Spain; ^8^Neuroimmunology Research Section, IRCCS Mondino Foundation, Pavia, Italy; ^9^Department of Neuroscience, Catholic University of the Sacred Heart, Rome, Italy; ^10^Neuroimmunology Program, FRCB‐IDIBAPS, University of Barcelona, Spain

**Background and Aims:** The diagnosis of autoimmune encephalitis (AE) relies on the detection of neural surface antibodies (NSAbs). The recommended diagnostic strategy includes a combination of tissue‐based assays (TBAs) and cell‐based assays (CBAs). While specialized centers use in‐house TBAs, many clinical laboratories rely on commercial TBAs, whose accuracy remains undetermined.

**Methods:** We included 92 CSF and 99 serum samples from AE patients with NSAbs (20 AMPAR, GABAAR, GABABR, IgLON5, LGI1, NMDAR, CASPR2; 19 mGluR5, 17 DPPX, 15 mGluR1) confirmed by in‐house TBAs and CBAs, along with 50 CSF and 50 sera from negative controls. We evaluated the performance of a commercial indirect immunofluorescence (IIF)‐TBA (EUROIMMUN). Slides were evaluated (positive/negative) by two experienced investigators (FG, JD); if discordant, an interrater discussion was conducted.

**Results:** The two raters were concordant in classifying 94% (133/142) of CSF and 88% (131/149) of sera. For CSF samples, 75% (106/142) were correctly identified, while 19% (27/142) were misclassified. Among sera, 66% (98/149) were correctly identified, while 22% (33/149) misclassified. The poorest performance was observed in detecting NMDAR, GABAAR, and mGluR5 Abs (not identified in 5/10, 6/10, and 5/9 sera and in 4/10, 5/10, 5/10 CSF samples, respectively). The sensitivity of the commercial IIF‐TBA was 84% for CSF and 76% for serum; the specificity was 72% for CSF and 73% for serum.

**Conclusion:** The diagnostic performance of EUROIMMUN IIF‐TBA for NSAbs is suboptimal. NMDAR‐Abs can be missed in 50% of cases. Our findings suggest that this commercial TBA should not be used alone to screen for NSAbs.

**Disclosure:** M. Spatola receives research support from La Caixa Foundation (Junior Leader) and Spanish National Health Institute Carlos III (ISCIII) and co‐funded by the European Union (FIS grant PI23/01366). J. Dalmau receives research support from CaixaResearch Health 2022 (HR22‐00221), Spanish National Health Institute Carlos III (ISCIII) and co‐funded by the European Union (FIS grant PI23/00858), Cellex Foundation, Fundació Clínic per a la Recerca Biomèdica (FCRB) Programa Multidisciplinar de Recerca, Generalitat de Catalunya Department of Health (SLT028/23/000071), Edmond J Safra Foundation. He receives royalties from Euroimmun for the use of NMDA as an antibody test. He received a licensing fee from Euroimmun for the use of GABAB receptor, GABAA receptor, DPPX and IgLON5 as autoantibody tests; he has received a research grant from Sage Therapeutics. All the other authors report no disclosures relevant to the abstract.

## EPO‐486

### Ublituximab and CD 19+ B cells rapid depletion after the first infusion: An early real‐world experience

#### E. D'Amico; F. De Pasquale; P. Di Filippo; A. Zanghì

##### University of Foggia, Italy

**Background and Aims:** Ublituximab, a third‐generation glycoengineered‐chimeric monoclonal antibody, targets a unique epitope on the CD20‐antigen and has a great antibody‐dependent‐cellular‐cytotoxicity.

**Methods:** A monocentric, real‐world Italian experience on three patients undergoing the first ublituximab infusion. The treatment protocol included an initial dose of 150/mg on day 1, followed by a second‐dose of 450/mg two‐weeks later. Blood samples were obtained before and at the end of the first split infusion (PRE/POST T0), after 7 days (T1), and after the second split dose (14 days) (POST‐T2). Lymphocytic count and immunophenotype were collected.

**Results:** Three patients were enrolled, median age 49 years (Q1:Q3; 46–54.5); median Expanded‐Disability‐Status‐Scale score 3.5 (Q1:Q3; 3.0–4.0). All patients were naïve to disease‐modifying therapies and had high active disease course. Blood samples ‘results are reported below. Patient 1: PRE‐T0‐absolute‐lymphocyte‐count 0.83 103/μL, CD19+B‐cells‐count 100 cells/μL; POST‐T0‐absolute‐lymphocyte‐count 0.40 103/μL, CD19+B cells‐count 3 cells/μL, T1‐absolute‐lymphocyte‐count 0.85 103/μL, CD19+B‐cells‐count 0 cells/μL, POST‐T2absolute‐lymphocyte‐count 0.60 103/μL, CD19+B cells‐count 1 cells/μL. Patient 2: PRE‐T0‐absolute‐lymphocyte‐count 1.83 103/μL, CD19+B‐cells‐count 191 cells/Ul; POST‐T0‐absolute‐lymphocyte‐count 0.14 103/μL, CD19+B‐cells‐count 0 cells/μL;T1‐absolute‐lymphocyte‐count 1.57 103/μL, CD19+B‐cells‐count 0 cells/μL; POST‐T2‐absolute‐lymphocyte‐count 0.54 103/μL, CD 19+ B cells count 0 cells/μL. Patient 3: PRE‐T0 absolute lymphocyte count 2.34 103/μL, CD 19+ B cells count 197 cells/μL; POST‐T0 absolute lymphocyte count 0.24 103/μL, CD 19+B cells count 0 cells/μL; T1 absolute lymphocyte count 1.83 103/μL, CD19+B‐cells‐count 0 cells/μL; POST‐T2 (not still available).

**Conclusion:** This study provides the first immunological profiling of CD19+ B cells depletion in patients receiving ublituximab after initial infusion. Further data are needed to validate them in larger cohorts.

**Disclosure:** Nothing to disclose.

## EPO‐487

### Real‐world experience of efgartigimod in juvenile myasthenia gravis in China: A multicenter retrospective study

#### J. Lin; B. Bu; Z. Li

##### Department of Neurology, Tongji Hospital, Tongji Medical College, Huazhong University of Science and Technology, Wuhan, Hubei, China

**Background and Aims:** Therapeutic decisions for juvenile myasthenia gravis (JMG) rely mostly on pediatric retrospective studies or adult MG guidelines. Here, we investigated the effectiveness and safety of efgartigimod in JMG.

**Methods:** In this multicenter, retrospective study involving 12 centers in China, participants with JMG (<18 years old) who received at least one dose of intravenous efgartigimod were enrolled. The clinical status was analyzed using MG‐related Activities of Daily Living (MG‐ADL) scores, Quantitative Myasthenia Gravis (QMG) score, and MG composite (MGC) scales.

**Results:** Seventeen JMG patients (3 male, 14 female) were included with some refractory JMG, seronegative MG, ocular MG, and myasthenic crisis. The average age pre‐efgartigimod treatment was 13.41 ± 2.96 years, with a median disease duration of 23 months (range, 1.5–166 months). At baseline, most of the patients (88.2%) were classified as MGFA classes II to V. Fifteen patients (88.2%) were anti‐AChR antibody‐positive. Most patients (16/17) received at least one immunomodulatory treatment. Following efgartigimod administration, 70.6% showed therapeutic progress at V1. By V2, 73% achieved significant improvement; 91.7% had remission, and 8 (66.7%) achieved minimal symptom expression at V4. Compared to baseline, the MG‐ADL and QMG scores decreased on average by 30% and 30.1% respectively at V1 and increased to 87.6% and 71.8% by V4. Efgartigimod improved symptoms across all muscle groups. No medication‐associated allergic reactions, infections, or serious adverse events were reported.

**Conclusion:** Efgartigimod is safe and rapidly effective in managing JMG patients, especially in some refractory JMG and seronegative MG, while providing more proof for treatment options.

**Disclosure:** No conflicts of interest

## EPO‐488

### Reactive pleocytosis after repeated lumbar puncture – Implications for clinical practice

#### M. Schmidauer ^1^; F. Foettinger^2^; K. Berek^1^; M. Auer^1^; R. Barket^1^; F. Di Pauli^1^; N. Krajnc^2^; L. Stichaller^2^; S. Zaic^2^; A. Zinganell^1^; F. Deisenhammer^1^; J. Walde^3^; G. Bsteh^2^; H. Hegen^1^

##### ^1^Department of Neurology, Medical University of Innsbruck, Innsbruck, Austria; ^2^Department of Neurology, Medical University of Vienna, Vienna, Austria; ^3^Department of Statistics, Faculty of Economics and Statistics, University of Innsbruck, Austria

**Background and Aims:** Lumbar puncture (LP) is a routine clinical procedure and in some cases repeatedly performed for diagnostic or therapeutic reasons. The impact of repeated LP on cerebrospinal fluid (CSF) findings is not clear.

**Methods:** Patients with non‐inflammatory neurological disease (NIND) and at least two consecutive lumbar punctures (LP) were included. Longitudinal changes in CSF white blood cell count (WBC), CSF total protein (TP) and CSF/serum albumin quotient (Qalb) were assessed depending on the time interval between the LP.

**Results:** A total of 73 patients with a median age of 35 years (25th–75th percentile: 25–45) and a female predominance of 75% had second LP after 6 (3–19) days. Twenty (27%) patients developed pleocytosis with an increase of WBC count to 8 (6–15) with a maximum of 30. Patients with pleocytosis had the follow‐up LP significantly earlier than patients without pleocytosis, 3.5 (3–7) versus 7 (3–28) days. The majority of patients (90%) with CSF pleocytosis had the second LP within 10 days. Further repeated LP in a subgroup of patients revealed similar findings. CSF TP and Qalb slightly increased in patients with pleocytosis.

**Conclusion:** Mild “reactive” CSF pleocytosis occurs in approximately one third of patients after repeated LP mostly when performed within 10 days.

**Disclosure:** Nothing to disclose.

## EPO‐489

### Comparative the efficacy of efgartigimod and intravenous immunoglobulin on AQP‐4 IgG positive NMOSD during acute attacks

#### J. Liu; M. Li; X. Xu; K. Dai; R. Dong; J. Liu; L. Yang; Y. Jiang; F. Peng

##### The Third Affiliated Hospital of Sun Yat‐sen University, Guangzhou, China

**Background and Aims:** The aim of our study was to evaluate the efficacy of efgartigimod and intravenous immunoglobulin (IVIG) on Aquaporin‐4 (AQP4) IgG positive Neuromyelitis optica spectrum disorders (NMOSD) patients during acute attacks.

**Methods:** A retrospective case‐control study was designed to compare the clinical outcomes of 13 NMOSD patients treated with efgartigimod at a dose of 20 mg/kg on the first and fifth day and 20 NMOSD patients treated with IVIG at 0.4 g/kg/day for 5 days. Follow‐up outcome information for patients is documented at 6 months post‐discharge.

**Results:** Compared with IVIG, efgartigimod could improve NMOSD patients' symptoms at acute attacks, the Expanded Disability Status Scale (EDSS) scores were significantly improved from 3.0 at admission to 2.5 at discharge (*p* < 0.001). The serum IgG levels were obviously decreased in NMOSD patients treated with efgartigimod (*p* < 0.001). AQP‐4 antibody in five NMOSD patients were found to turn negative after efgartigimod treatment.

**Conclusion:** The efficacy of efgartigimod is comparable to IVIG therapy in improving acute symptoms of AQP4‐IgG‐positive NMOSD. Efgartigimod could be an elegant alternative to IVIG therapy, and no serious adverse events were observed during infusion.

**Disclosure:** The authors declare that they have no competing interests

## EPO‐490

### Characteristics of recently diagnosed MS patients treated with ofatumumab in Switzerland: The KOSMOS study population

#### R. Hoepner^1^; P. Roth^2^; C. Berger^3^; A. Czaplinski^4^; L. Diem^5^; M. Théaudin^6^; C. Zecca^7^; D. Eyer^8^; M. Arzt ^8^

##### ^1^Department of Neurology, Inselspital, Bern University Hospital and University of Bern, Bern, Switzerland; ^2^University Hospital Zurich and University of Zurich, Zurich, Switzerland; ^3^Praxis für Augenheilkunde und Neurologie, Sargans, Switzerland; ^4^Bellevue Medical Group and Klinik für Neurologie Hirslanden, Zurich, Switzerland; ^5^Neurocenter, Luzerner Kantonsspital, Lucerne, Switzerland; ^6^Department of Clinical Neurosciences, Service of Neurology, Lausanne University Hospital and University of Lausanne, Lausanne, Switzerland; ^7^Multiple Sclerosis Center, Neurocenter of Southern Switzerland, EOC and Faculty of biomedical Sciences, Università della Svizzera Italiana, Lugano, Switzerland; ^8^Novartis Pharma Schweiz AG, Rotkreuz, Switzerland

**Background and Aims:** Real‐world data evaluating the effect of ofatumumab on people with multiple sclerosis (pwMS) in a routine medical setting are still missing, especially for recently diagnosed pwMS. The goal of KOSMOS (Kesimpta® [Ofatumumab] in Swiss Multiple sclerosis patients – an Observational Study) is to close this evidence gap.

**Methods:** KOSMOS (COMB157GCH01/NCT05285904) is a non‐interventional study aiming to investigate the impact of ofatumumab on an early RMS population under routine medical care. The study recruited adult patients within 5 years of RMS diagnosis who had received ofatumumab under Swiss Kesimpta label for 3 months or longer prior to inclusion. Patients had to be willing and able to participate and provide written informed consent. The primary endpoint of KOSMOS is NEDA‐3 (“no evidence of disease activity” defined as lack of relapses, MRI lesions, and disability worsening) at 12 months, as compared to the standard‐of‐care arm of a phase‐3b study, STHENOS (COMB157G3301/NCT04788615). Other endpoints include adherence and persistence to ofatumumab, patient‐reported outcomes, and immune cell population dynamics and serum biomarkers.

**Results:** A total of 107 pwMS were included across 18 centres (3 university hospitals, 3 other hospitals, 12 private practices). Here, we will present baseline data including demographic characteristics, medical history, previous disease‐modifying therapies, clinical and MRI parameters, as well as patient‐reported physical and psychological impact of MS (MSIS‐29).

**Conclusion:** This baseline analysis will characterize data of an early Swiss RMS population under routine medical care with ofatumumab. Results from the KOSMOS study will provide insights into the effectiveness of ofatumumab in a real‐world scenario.

**Disclosure:** This study was fully funded by Novartis Pharma Schweiz AG. RH received speaker/advisor honorary from Merck, Novartis, Roche, Biogen, Alexion, Sanofi, Janssen, Bristol‐Myers Squibb, Teva/Mepha and Almirall. He received research support within the last 5 years from Roche, Merck, Sanofi, Biogen, Chiesi, and Bristol‐Myers Squibb. PR has received honoraria for lectures or advisory board participation from Alexion, Bristol‐Myers Squibb, Boehringer Ingelheim, Debiopharm, Galapagos, Merck Sharp and Dohme, Laminar, Midatech Pharma, Novocure, QED, Roche, Sanofi and Servier and research support from Merck Sharp and Dohme and TME Pharma. AC received compensations and grants from Novartis, Roche, Merck, Biogen, TEVA, Almirall, Sanofi. CHUV (employer) received for MT speaker honoraria, fees for advisory boards and travel grants from Novartis. Ente Ospedaliero Cantonale (employer) received compensation for CZ's speaking activities, consulting fees, or grants from Abbvie, Alexion, Almirall, Biogen, Bristol Meyer Squibb, Eisai, Lilly, Lundbeck, Merck, Merz, Novartis, Organon, Pfizer, Sandoz, Sanofi, Teva Pharma, Roche. CZ is recipient of a grant for senior researchers provided by AFRI (Area Formazione accademica, Ricerca e Innovazione), EOC. DE and MEA are employees of Novartis Pharma Schweiz AG. CB and LD have nothing to disclose related to this submission.

## EPO‐491

### Five‐year outcomes in MS patients based on Type 2 and Type 3 oligoclonal band patterns

#### S. Alizada ^1^; U. Samadzada^4^; N. Yapici^1^; S. Cevik^2^; E. Zengin^4^; C. Baba^3^; S. Ozakbas^4^

##### ^1^Department of Neurology, Dokuz Eylul University, Izmir, Turkey; ^2^Institute of Health Sciences, Dokuz Eylul University, Izmir, Turkey; ^3^Urla State Hospital, Neurology, Izmir, Turkey; ^4^Izmir University of Economics, Medical Point Hospital, Izmir, Turkey

**Background and Aims:** Oligoclonal bands (OCBs) are essential biomarkers in multiple sclerosis (MS), providing insights into disease activity and progression. However, the clinical significance of different OCB patterns, particularly Type 2 and Type 3, remains unclear. Understanding their impact on long‐term outcomes may help refine prognostic assessments in MS. The study aims to compare the clinical characteristics and five‐year outcomes of multiple sclerosis patients with Type 2 and Type 3 oligoclonal band positivity.

**Methods:** This retrospective cohort study included 1,090 MS patients with Type 2 OCB positivity and 180 with Type 3 OCB positivity. Clinical variables, including age of onset, gender, Expanded Disability Status Scale (EDSS) scores, relapse rates, and transition to secondary‐progressive MS (SPMS), were analyzed at years two and five.

**Results:** At year two, the mean EDSS score was significantly lower in the Type 3 OCB group (1.11 ± 1.51) compared to the Type 2 group (1.51 ± 1.67; *p* < 0.05). By year five, EDSS scores increased in both groups (Type 3: 1.38 ± 1.83; Type 2: 1.7 ± 1.91), but the difference was no longer statistically significant. No significant differences were observed in relapse rates or SPMS transition.

**Conclusion:** Type 3 OCB positivity was associated with lower disability at year two, but this difference was not sustained at year five. Further studies incorporating cognitive and motor outcomes are needed to clarify the long‐term prognostic value of OCB patterns in MS.

**Disclosure:** Nothing to disclose.

## EPO‐492

### Antineuronal antibody screening in the absence of neurological involvement in high‐risk oncological patients

#### S. Stavropoulou De Lorenzo ^1^; A. Bokas^2^; E. Kesidou^1^; N. Grigoriadis^1^; I. Michailidou^1^; C. Bakirtzis^1^; H. Alexopoulos^3^

##### ^1^2nd Department of Neurology, School of Medicine, Aristotle University of Thessaloniki, Thessaloniki, Greece; ^2^Department of Medical Oncology, Theageneio Cancer Hospital, Thessaloniki, Greece; ^3^Department of Cell Biology and Biophysics, Faculty of Biology, National and Kapodistrian University of Athens, Athens, Greece

**Background and Aims:** The incidence of paraneoplastic neurological syndromes (PNS) has significantly increased due to the widespread administration of immune checkpoint inhibitors (ICIs), inducing the production of antineuronal autoantibodies. The aim of this study is the detection of antineuronal autoantibodies in patients with small cell lung cancer (SCLC) in the absence of neurological signs and/ or symptoms.

**Methods:** 10 patients were included in this study. For the detection of antineuronal autoantibodies, tissue‐based assay (TBA), line immunoassay (LIA), and enzyme‐linked immunosorbent assay (ELISA) using patients’ blood sera were performed.

**Results:** The male: female ratio was 7:3, the mean age of participants was 69.8 years (SEM ± 2.98 years), and the mean disease duration was 7.6 months (SEM ± 2.13 months). 70% of patients were diagnosed with extensive SCLC, whereas 30% were diagnosed with limited SCLC. 50% of patients were treated with ICIs and 30% of patients had received ICIs for ≥6 months. Antibody screening with TBA revealed the presence of antineuronal autoantibodies in 50% of patients. The presence of anti‐GAD65 antibodies was identified using LIA, whereas anti‐GAD65 titers were measured using ELISA (102.8 IU/mL). The presence of low‐titer anti‐GAD65, prior to ICIs initiation, was attributed to diabetes mellitus type 2, according to the patient's medical history.

**Conclusion:** Antineuronal autoantibodies were detected in the serum of a significant proportion of patients with SCLC in the absence of neurological signs and/ or symptoms. According to the currently available literature, the association between the presence of antineuronal autoantibodies and the development of PNS remains elusive.

**Disclosure:** Nothing to disclose.

## EPO‐493

### Clinical disability in coexisting NMOSD and systemic autoimmune diseases

#### Y. Martinez Lopez ^1^; E. Tetlalmatzi‐Azuara^2^; A. García‐Sarreon^2^; J. Vargas‐Rodríguez^2^; M. Coutinho‐Thomas^1^; T. Corona^3^; J. Flores Rivera^1^; V. Rivas‐Alonso^1^

##### ^1^Neuroimmunology Department, National Institute of Neurology and Neurosurgery, Mexico City, Mexico; ^2^Neurology Department, National Institute of Neurology and Neurosurgery, Mexico City, Mexico; ^3^Clinical Laboratory of Neurodegenerative Disease, National Institute of Neurology and Neurosurgery, Mexico City, Mexico

**Background and Aims:** Neuromyelitis optica spectrum disorder (NMOSD) is an autoimmune central nervous system disease that leads to significant clinical disability with each relapse. Its association with other autoimmune systemic diseases (ASD) is recognized, but the impact on clinical disability remains unclear.

**Methods:** Methods: A cross‐sectional study was conducted on NMOSD patients diagnosed by the 2015 criteria, with follow‐ups from January 2002 to December 2023. ASD was diagnosed by rheumatologists, and sociodemographic and clinical data were collected. Multivariate Cox regression identified factors associated with reaching an Expanded Disability Status Scale (EDSS) score of 6 or higher. Objective: To assess clinical disability in patients with coexisting NMOSD and ASD.

**Results:** Among 170 patients, 28 (16.5%) had coexistent‐ASD. The clinical course was relapsing in 75% and monophasic in 25%. The most prevalent ASDs were systemic lupus erythematosus (35.7%) and Sjögren's syndrome (32.1%). Factors associated with higher clinical disability included AQP4‐IgG positivity (HR = 2.82, *p* < 0.05*), age at NMOSD diagnosis (HR = 1.06, *p* < 0.05*), coexistent‐ASD (HR = 1.68, *p* = 0.36), and first‐attack neurological symptom (HR = 1.16, *p* = 0.44).

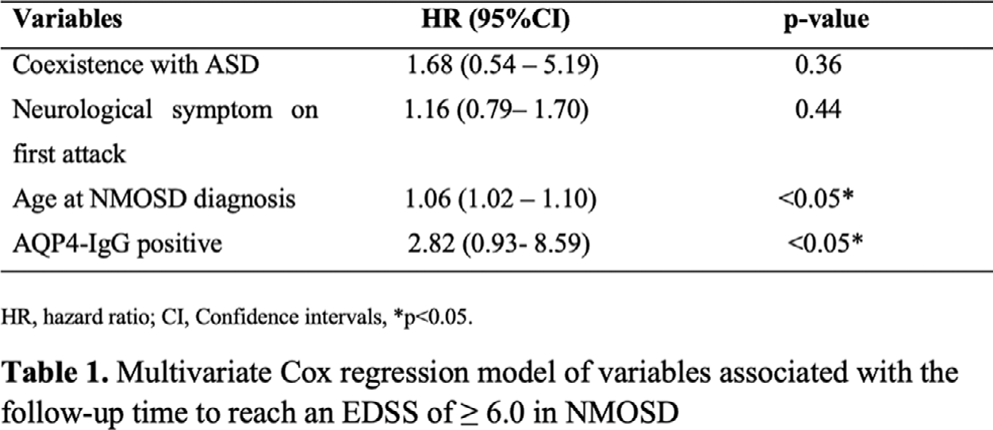

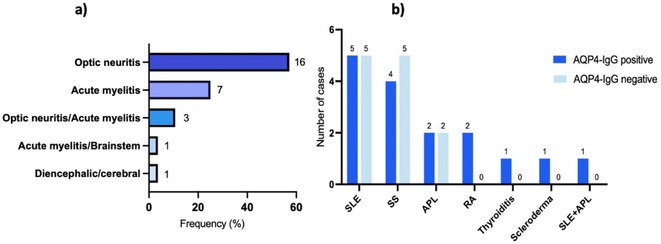

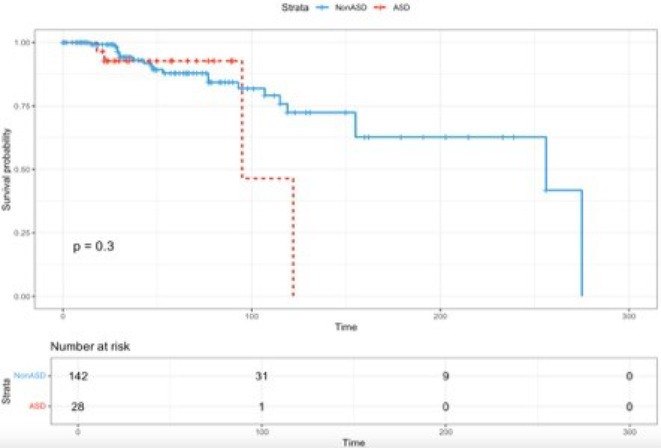

**Conclusion:** The coexistence of NMOSD and ASD impacts clinical disability, making early identification and evaluation crucial for effective therapy and reducing long‐term disability.

**Disclosure:** Nothing to disclose.

## Neurogenetics

## EPO‐494

### The effect of melatonin MTNR1A and MTNR1B receptor gene mutations in chronic insomnia

#### B. Oguz Selcuk ^1^; A. Sahbaz^2^; D. Kırac^2^; F. Mayda Domac^1^

##### ^1^University of Health Sciences, Erenkoy Mental Health and Neurological Disorders Training and Research Hospital, Neurology Department, Istanbul, Turkey; ^2^Department of Medical Biology, Yeditepe University, Faculty of Medicine, Istanbul, Turkey

**Background and Aims:** Insomnia is a common sleep disorder characterized by symptoms such as difficulty in falling asleep, maintaining sleep, or waking up early. When these complaints occur at least three times a week for three months and cause functional problems, it is called chronic insomnia. Our study aims to investigate genetic factors in the etiology of chronic insomnia by examining rs2119882 and rs4753426 mutations in the MTNR1A and MTNR1B genes.

**Methods:** 100 patients diagnosed with chronic insomnia and 100 healthy controls were included in our study. DNA isolation was performed from peripheral venous blood samples. Mutations rs2119882 in the MTNR1A gene and rs4753426 in the MTNR1B gene were investigated by real‐time polymerase chain reaction method.

**Results:** 200 participants (100 patients, 100 controls) were examined. While no significant difference was found between the groups in the rs2119882 polymorphism, the homozygous mutant (CC) genotype was more frequent in the patient group. In the rs4753426 polymorphism, the homozygous mutant genotype was found only in the patient group and was statistically significant (p<0.001). Additionally, when allele distributions between patient and control groups were compared, a statistically significant difference was found for the C allele in the rs4753426 polymorphism in the patient group (p<0.05).This result, showing a similar outcome to the higher presence of the CC genotype in the patient group, suggests that the C allele may predispose to the formation of insomnia.

**Conclusion:** Our study suggests that the rs4753426 mutation in the MTNR1B gene, particularly the CC genotype, may predispose to insomnia. This study may guide gene therapy studies.

**Disclosure:** Nothing to disclose.

## EPO‐495

### Mitochondrial ataxia: The Italian experience

#### C. Bernardini; A. Meli; P. Lopriore; V. Montano; M. Mancuso

##### Neurological Institute, Department of Clinical and Experimental Medicine, University of Pisa, Pisa, Italy

**Background and Aims:** This retrospective study assessed the prevalence and characteristics of ataxic syndrome in 764 patients genetically or clinically diagnosed with late‐onset (age >16) primary mitochondrial disease (PMD).

**Methods:** Data from the Italian database of mitochondrial diseases were analyzed including clinical, neurophysiological, neuroimaging, and genetic information.

**Results:** Ataxia was observed in 63 patients (33 females), with a mean PMD onset age of 36.38 and ataxic syndrome onset at 39.86. Ataxia preceded PMD diagnosis in 7 cases and coincided with it in 28 cases. We observed cerebellar ataxia in 26 patients, pure sensory ataxia in 10 and spinocerebellar ataxia in 27 cases. Electroneurography showed axonal sensory neuropathy in 24 patients, axonal sensory motor involvement in 12, and normal findings in 10. MRI findings included cerebellar (47.6%), brainstem (14.3%) and global cerebral atrophy (50.8%), white matter hyperintensities (42.9%), lactate peak on spectroscopy (17.5%), and basal ganglia abnormalities (22.2%). Genetic analysis identified mtDNA variants in 25 patients (mostly m.8344A>G in MT‐TK and m.3243A>G in MT‐TL1), single mtDNA deletions in 3 patients, and nDNA gene variants in the remaining cases (16 POLG1, 3 OPA1, 2 C10ORF2, 1 AARS2, 1 DARS2, 1 PMPCA).

**Conclusion:** As ataxic syndrome frequently occurs at the onset of PMD and given the increasing prevalence of PMDs, mitochondrial etiology should be included in adult‐onset ataxia diagnostic flowchart. Early identification of this etiology can be crucial for addressing any concurrent medical conditions that may arise in PMDs patients, as well as for potential target therapies.

**Disclosure:** Nothing to disclose.

## EPO‐496

### Genetic mutations in early onset dementia

#### C. Linguetta ^1^; F. Della Pia^1^; R. De Rosa^1^; C. Criscuolo^2^; E. Salvatore^3^

##### ^1^Department of Neuroscience, Reproductive Sciences and Dentistry, University Federico II, Naples, Italy; ^2^Centers for Cognitive Disorders and Dementia, Neurology University Hospital Federico II, Naples, Italy; ^3^Department of Advanced Biomedical Sciences, University Federico II, Naples, Italy

**Background and Aims:** Early‐onset dementia (EOD) is a heterogeneous group of neurodegenerative disorders with an important genetic component. This study aimed to assess the prevalence of genetic mutations in symptomatic patients and explore correlations with disease onset, clinical phenotype, and cognitive impairment severity.

**Methods:** We enrolled patients with dementia onset before 65 years, evaluated clinically, through brain imaging, and cerebrospinal fluid (CSF) biomarker analysis (Aβ42, t‐tau, p‐tau, Aβ42/Aβ40). Then, it was performed a genetic testing with 52 genes implicated in EOD. Fifty patients from our University Hospital's Center for Cognitive Disorders and Dementias (CCDD) were analyzed in 2023‐24.

**Figure:**
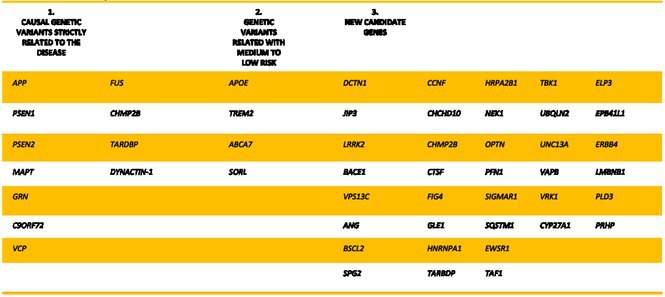
**FIGURE 1** The genes’ list with his subdivision in three categories is reported in the table

**Results:** Genetic testing identified pathogenic mutations in 15% of cases, consistent with literature data. These included autosomal dominant Alzheimer's disease (ADAD), frontotemporal dementia (FTD), behavioral variant FTD (FTDbv) and Cerebrotendinous xanthomatosis (CTX). No clear genotype‐phenotype correlation emerged, likely due to the limited sample size. Among seven mutation‐positive patients, two were women with a positive family history: a 38‐year‐old with PSEN1 and a 63‐year‐old with MAPT. The five men included a 67‐year‐old with GRN mutation and memory‐executive dysfunction, a 54‐year‐old with MAPT and stuttering onset, a 59‐year‐old with C9ORF72 and severe psychiatric symptoms, a 47‐year‐old with CYP27A1 and progressive dementia, and a 55‐year‐old with TBK1

**Conclusion:** Genetic mutations were found in a minority of EOD cases, underscoring the need for further research. Advances in genetic testing, biomarkers, and therapies are crucial for improving diagnosis, prognosis, and patient management. Understanding EOD's genetic basis is key to precision medicine and targeted interventions.

**Disclosure:** Nothing to disclose.

## EPO‐498

### A case report of a de novo TAOK1 variant associated with childhood‐onset action tremor

#### M. Krygier ^1^; M. Pietruszka^1^; M. Limanówka^1^; P. Rumiński^1^; K. Dzwilewski^1^; M. Mazurkiewicz‐Bełdzińska^1^; M. Chylińska^2^; M. Zech^3^

##### ^1^Department of Developmental Neurology, Medical University of Gdansk, Gdansk, Poland; ^2^Department of Adult Neurology, Medical University of Gdansk, Gdansk, Poland; ^3^Institute of Human Genetics, School of Medicine and Health, Technical University of Munich, Munich, Germany; Institute of Neurogenomics, Helmholtz Zentrum München, Munich, Germany; Institute for Advanced Study, Technical University of Munich, Garching, Germany

**Background and Aims:** The aim of this study was to investigate the potential genetic basis of childhood‐onset tremor in a 15‐year old patient.

**Methods:** We conducted a clinical and genetic evaluation of a 15‐year‐old patient with symptoms of essential tremor. The patient was assessed through neurological examination, electromyography and magnetic resonance imaging as well as metabolic tests. Trio exome sequencing was performed on the patient and his parents to identify potential genetic etiology. The identified variant was further analyzed using available genomic databases.

**Results:** The patient presented with a bilateral upper limb postural and kinetic tremor, initially observed in early childhood and progressively worsening with age. The tremor was exacerbated by stress and emotions. Neurological examination revealed no other significant findings. EMG confirmed a kinetic tremor with a frequency of 6–7 Hz. Exome sequencing identified a de novo heterozygous frameshift variant in TAOK1 (NM_020791.4: c.952del, p.Gln318Argfs*9), classified as likely pathogenic based on ACMG criteria. This variant was absent in both GnomAD and ClinVar databases. The patient's tremor significantly improved with propranolol treatment.

**Conclusion:** This case supports the association between TAOK1 mutations and tremor, specifically childhood‐onset, action tremor, even in the absence of other neurodevelopmental symptoms. Further studies involving larger patient cohorts are needed to explore the frequency of TAOK1 mutations in hereditary essential tremor.

**Disclosure:** Funding sources: This work was supported by funding from the EJP RD (EJP RD Joint Transnational Call 2022) and the German Federal Ministry of Education and Research (BMBF, Bonn, Germany), awarded to the project PreDYT (PREdictive biomarkers in DYsTonia, 01GM2302). This research was also supported by a “Schlüsselprojekt” grant from the Else Kröner‐Fresenius‐Stiftung (2022_EKSE.185). In addition, this study (M.Z.) has received funding from the Federal Ministry of Education and Research (BMBF) and the Free State of Bavaria under the Excellence Strategy of the Federal Government and the Länder, as well as by the Technical University of Munich ‐ Institute for Advanced Study. M.Z. receives research support from the German Research Foundation (DFG 458949627; ZE 1213/2‐1).

## EPO‐499

### Unraveling the genetic basis of heterogeneous neurologic and other phenotypes through genomic data reanalysis

#### M. Drakos; S. Zapri; A. Metaxas; E. Skoula; P. Tsasmea; I. Tsiverdis; L. Mathioudakis; I. Zaganas

##### Neurology and Neurogenetics Laboratory, School of Medicine, University of Crete, Heraklion, Crete, Greece

**Background and Aims:** Next Generation Sequencing (NGS), particularly Whole Exome Sequencing (WES), has revolutionized rare disease diagnostics by enabling earlier and more precise molecular diagnoses. As genomic databases expand and analytical tools improve, regular reanalysis of NGS data in undiagnosed Mendelian disorders is strongly recommended, as it can lead to new diagnoses in previously unsolved cases. In this study, we aimed to evaluate the effectiveness of re‐analyzing pre‐existing whole‐exome sequencing (WES) data from patients with rare diseases for whom a molecular cause has not yet been identified.

**Methods:** We analyzed WES data from 243 undiagnosed individuals using the Genome‐Phenome Analysis Platform (GPAP) and in‐house analysis pipelines. The study cohort included cases referred to the Neurology and Neurogenetics Lab (NNL) at the University of Crete between 2014 and 2024, which remained unsolved after previous analyses. All participants were Caucasians residing in Greece, presenting with diverse clinical features, primarily neurological (72.4% of cases) and developmental manifestations.

**Results:** A causative variant was identified in 29 patients, resulting in a diagnostic yield of 11.9%. Of the causative variants detected, 24 were novel. The remaining cases were classified into two groups for further analysis: those with a potential diagnosis (11.1%) and those without a diagnosis (76.9%), based on the strength of evidence supporting a genetic cause.

**Conclusion:** Our reanalysis provided a genetic diagnosis for 29 previously unsolved rare disease cases. We identified 24 novel pathogenic variants and uncovered two ultra‐rare syndromes—Verheij and White‐Sutton syndromes—each with approximately 60 and 100 reported cases in the literature, respectively.

**Disclosure:** Nothing to disclose.

## EPO‐500

### Prevalence of intronic repeat expansions in RFC1 in Slovak patients with idiopathic late‐onset ataxia

#### M. Ostrožovičová ^3^; R. Curro^4^; N. Dominik^4^; J. Necpal^5^; A. Lackova^1^; V. Han^1^; L. Trckova^2^; M. Rizig^4^; A. Cortese^4^; H. Houlden^4^; M. Skorvanek^1^

##### ^1^Department of Neurology, P.J. Safarik University, Kosice, Slovakia; ^2^Department of Neurology, University Hospital of L. Pasteur, Kosice, Slovakia; ^3^Laboratory of Clinical Neurosciences, University Scientific Park MEDIPARK, P. J. Safarik University, Kosice, Slovakia; ^4^Department of Neuromuscular Disease, UCL Queen Square Institute of Neurology, London, UK; ^5^Department of Neurology, Zvolen Hospital, Zvolen, Slovakia

**Background and Aims:** Biallelic repeat expansions in RFC1 cause Cerebellar ataxia, neuropathy and vestibular areflexia (CANVAS) in multiple populations. Therefore, this study aims to investigate the frequency of CANVAS among Slovak patients with idiopathic late‐onset spinocerebellar ataxia [1].

**Methods:** DNA samples from 51 cases from 2 tertiary movement disorders centres (Kosice and Zvolen, Slovakia) involved in the CEGEMOD consortium [2] were analysed with Flanking PCR and Repeat Primed PCR for (AAGGG)n, (AAAGG)n and (AAAAG)n RFC1 repeat expansions. Southern blot confirmed positive cases [3].

**Results:** We identified one patient positive for biallelic pathogenic (AAGGG)n repeat expansion (1/51 = 1.96%). The patient, an 82‐year‐old female, developed generalised chorea at 73y old, unresponsive to antipsychotic treatment changes (initiated for depression and anxiety). Within five years, she also developed dysarthria, slight dysphagia, nystagmus, slow vertical saccades, appendicular and gait ataxia, followed by EMG‐verified sensory‐motor axonal‐demyelinating neuropathy and vestibular areflexia with abnormal HIT. Additionally, she complained about mild cough and memory issues. MOCA score was 24p. Brain MRI showed significant cerebellar atrophy. The family history was negative. Previous genetic testing for Huntington's disease, SCA1,2,3,6,7,17, C9orf72 and WES was negative. We did not identify any (AAAGG)n or (AAAAG)n repeat expansions in our study group.

**Conclusion:** Our data suggest that though rare, CANVAS should be included in the standard clinical genetic testing of patients with spinocerebellar ataxia in Slovakia, especially to prevent delayed diagnosis as initial symptoms may vary. To our knowledge, this is the first patient with CANVAS reported from Slovakia.

**Disclosure:** This project was supported by the Slovak Grant and Development Agency under contract APVV‐22‐0279 and by the EU Renewal and Resilience Plan “Large projects for excellent researchers” under grant No. 09I03‐ 03‐V03‐00007 References: 1. Dominik, N. et al. Normal and pathogenic variation of RFC1 repeat expansions: implications for clinical diagnosis. Brain 146, 5060–5069 (2023). 2. Ostrozovicova, M. et al. Central European Group on Genetics of Movement Disorders. Eur. J. Neurol. 31, e16165 (2024). 3. Ronco, R. et al. Truncating variants in RFC1 in cerebellar ataxia, neuropathy, and vestibular areflexia syndrome. Neurology 100, e543–e554 (2023).

## EPO‐501

### The complex molecular landscape of Parkinson disease in Turkey – Genotype‐phenotype correlations in a genetic cohort

#### T. Gül‐Demirkale ^1^; Ö. Öztop‐Çakmak^2^; G. Genç^3^; N. Şimşek‐Erdem^4^; H. Uysal^5^; O. Doğu^6^; G. Kızıltan^7^; G. Yalçın‐Çakmaklı^8^; B. Elibol^8^; S. Ertan^2^; J. Trinh^9^; C. Klein^9^; A. Başak^1^

##### ^1^Suna and Inan Kıraç Foundation, Neurodegeneration Research Laboratory (NDAL)‐KUTTAM, School of Medicine, Koç University, Istanbul, Turkey; ^2^Department of Neurology, School of Medicine, Koç University, Istanbul, Turkey; ^3^Department of Neurology, University of Health Sciences, Istanbul, Turkey; ^4^Department of Neurology, Private Termessos Hospital, Antalya, Turkey; ^5^Department of Neurology, School of Medicine, Akdeniz University, Antalya, Turkey; ^6^Department of Neurology, Faculty of Medicine, Mersin University, Mersin, Turkey; ^7^Department of Neurology, Cerrahpaşa Medical Faculty, Istanbul University, Istanbul, Turkey; ^8^Department of Neurology, School of Medicine, Hacettepe University, Ankara, Turkey; ^9^Institute of Neurogenetics, University of Lübeck, Lübeck, Germany

**Background and Aims:** Parkinson Disease (PD) is the second most prevalent neurodegenerative disorder (NDD) characterised by its cardinal motor signs and non‐motor symptoms. Due to its ancient history, Turkey's population is admixed. Also genetically underrepresented, it needs more thorough investigations.

**Methods:** A cohort comprising 209 PD patients with either family history or early age of onset and 21 patients with atypical PD was analyzed by WES and conventional methods. Seven unsolved patients were re‐annotated to investigate candidate gene variants, not associated with any disease in OMIM.

**Results:** Ninety‐three patients were identified with rare or novel variants in 27 different genes that are causative (12 PD‐ and 13 NDD‐associated genes) and/or risk factors for PD (GBA1 and GLUD2) with a diagnostic yield of 40.4% (Figure 1,2). Fifteen and 23 patients were detected with heterozygous variants in the possible risk genes PRKN/PINK1 and TENM4, respectively. Further ten possible candidate genes were identified by re‐annotating WES data and analysing AMP‐PD and PPMI.

**Figure:**
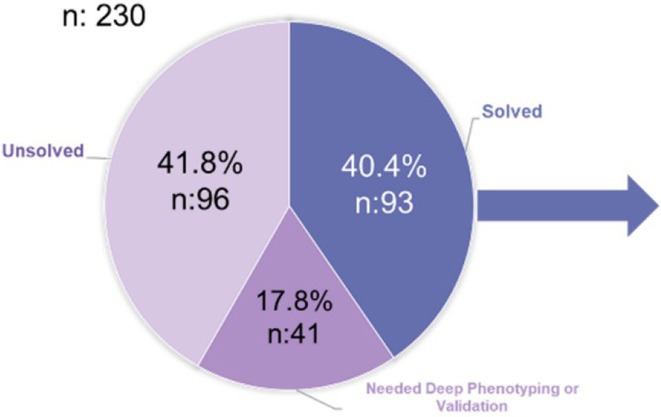
**FIGURE 1** The diagnostic yield of the study cohort.

**Figure:**
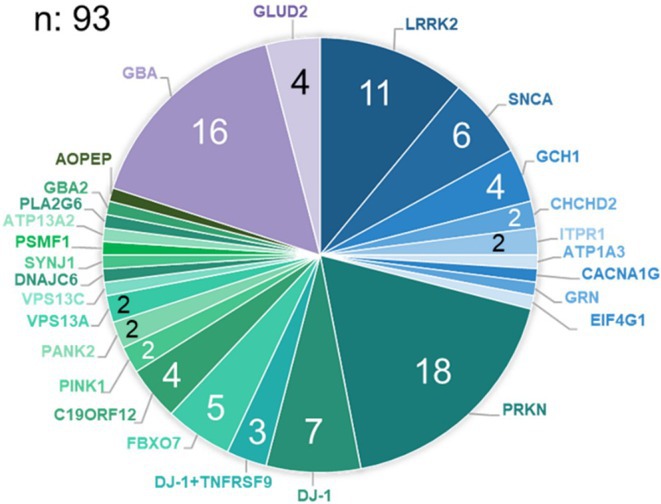
**FIGURE 2** Genes with pathogenic variants identified in the patients solved. Autosomal dominantly inherited genes are in bluish colours, autosomal recessive genes are in greenish hues, and the risk factors are in purple.

**Conclusion:** To the best of our knowledge, this is the most comprehensive study on the molecular structure of PD in Turkey. The PRKN gene was identified as the most frequently mutated gene in the predominantly genetic cohort under study. Detection of reported variants in genes associated with other NDD, points to the genetic heterogeneity within and overlap across these diseases. Our results are expected to contribute to new knowledge of the genetic and mechanistic factors underlying PD in Turkey.

**Disclosure:** This study was supported by Suna and İnan Kıraç Foundation and Koç University Research Center for Translational Medicine (KUTTAM).

## EPO‐502

### CANVAS: Not the usual pentanucleotide

#### V. Gioiosa ^1^; M. Barghigiani^2^; A. Tessa^2^; F. Santorelli^2^; C. Casali^1^

##### ^1^Department of Medico‐Surgical Sciences and Biotechnologies, University of Rome Sapienza, Latina, Italy; ^2^IRCCS Stella Maris Foundation, Pisa, Italy

**Background and Aims:** Cerebellar Ataxia, Neuropathy, and Vestibular Areflexia Syndrome affects approximately 1 in 20,000 individuals (1). The most common cause is a biallelic expansion of the AAGGG pentanucleotide in the second intron of the RFC1 gene. In December 2023, a study suggested that additional pentanucleotides expansions beyond speciphic sizes can also be causative of the disease (2). Until then, expanded alleles other than AAGGG had only been found in control groups (3). Only isolated cases of this condition have been identified, no family studies having been reported.

**Methods:** A family of 6 siblings underwent neurological examination, electrophysiological assessment (ENG‐EMG), and blood sampling for genetic investigations, at the Polo Pontino/ICOT of Rome Sapienza University. Pathogenic expansion of the (AAGGG)n, and the expansion of the (AAAGG) ≥600 was investigated.

**Results:** The 4 symptomatic patients exhibited cerebellar ataxia, nystagmus, hypoacusis, and sensory axonal neuropathy. The onset was late‐onset, with a slowly progressive course. They showed compound heterozygosity with the pathogenic pentanucleotide expansion AAGGG and the AAAGG pentanucleotide expansion exceeding 600 repeats. The samples from healthy controls showed only the presence of the pathogenic AAGGG pentanucleotide in heterozygosity.

**Figure:**
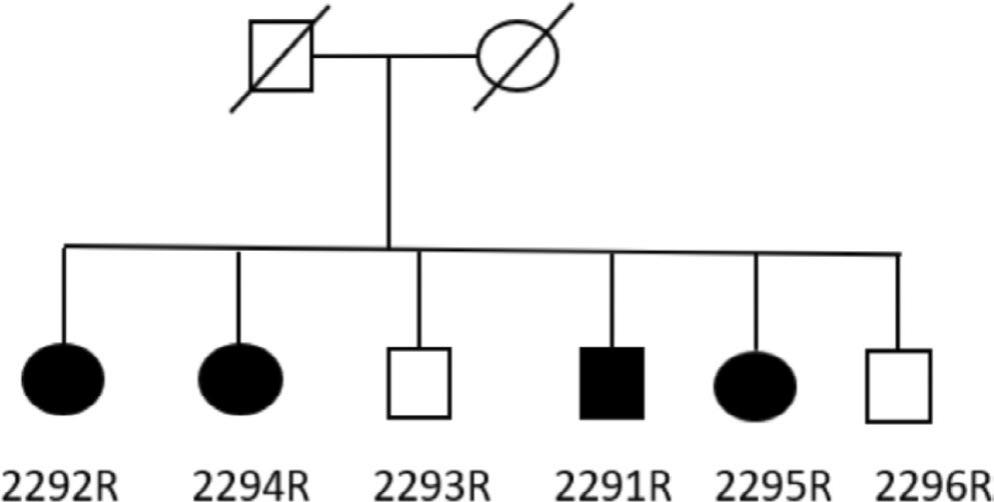
**FIGURE 1** FAMILY TREE

**Figure:**
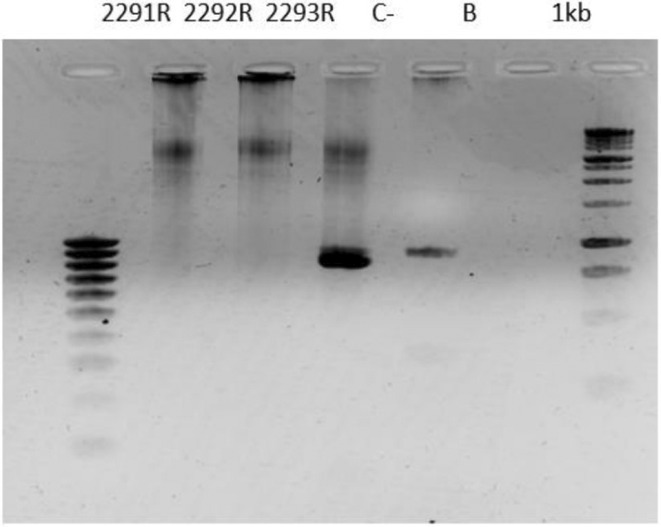
**FIGURE 2** electrophoresis

**Figure:**
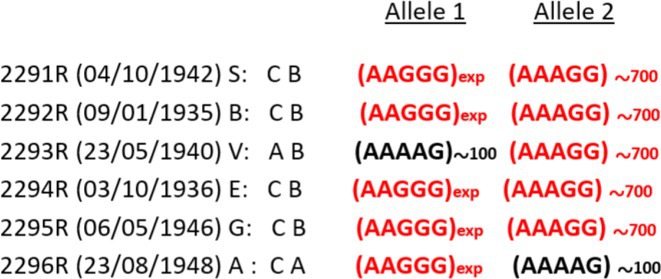
**FIGURE 3** Alleles

**Conclusion:** CANVAS is usually diagnosed after reassessing ataxic patients with no family history, a late‐onset and sensory axonal neuropathy, frequently accompanied by a cough. In our family the clinical presentation was similar with little intrafamilial variability and without clear vestibular issues and cough. This study presents an Italian family with four siblings affected by CANVAS caused by compound heterozygosity of the AAGGG pentanucleotide expansion and the AAAGG pentanucleotide expansion exceeding 600 repeats.

**Disclosure:** Nothing to disclose.

## EPO‐503

### Genetic landscape of primary dystonic syndromes: Insights from next‐generation sequencing in a cohort of 65 patients

#### V. Busco ^1^; A. Cimmino^1^; P. Sanginario^1^; M. Petracca^1^; D. Genovese^1^; A. De Biase^1^; M. Pomponi^2^; D. Tiziano^2^; P. Calabresi^1^; A. Bentivoglio^1^; G. Di Lazzaro^1^

##### ^1^Neurology Unit, Fondazione Policlinico Universitario Agostino Gemelli IRCCS, Rome, Italy; ^2^Istituto di Medicina Genomica, Fondazione Policlinico Universitario Agostino Gemelli IRCCS, Rome, Italy

**Background and Aims:** Dystonia, a movement disorder, can manifest in isolation or combination and involves various genetic causes. TOR1A (DYT1) was the first gene linked to primary dystonia, but advances in gene sequencing have identified many other implicated genes [1,2]. This study aimed to examine the prevalence of genetic variants associated with dystonic syndromes and characterize their clinical phenotypes in 65 patients with primary dystonic syndrome.

**Methods:** We studied 65 patients with primary dystonic syndrome, analyzing their Next‐Generation Sequencing (NGS) dystonia panel to identify mutations and assess their association with compatible clinical phenotypes.

**Results:** NGS panels of 26 of these patients (40%) were negative. In 39 patients (60%) variants were detected. A total of 62 variants were reported, among which 13 were classified as pathogenetic/likely pathogenetic, and 49 as variants of unknown significance (VUS). Six patients received a genetic diagnosis (ATM, PRRT2, TH, GNAL, GLB1 and SGCE). Four patients presented VUS in ANO3, CIZ1, TOR1A, ATP7B and had clinical signs and a segregation of the variants compatible with the related clinical phenotype. The remaining twenty‐nine patients had inconclusive results.

**Conclusion:** Overall, 9% of patients achieved a definitive genetic diagnosis, with an additional 6% having probable diagnoses. Despite the modest diagnostic yield, the study highlights the role of genetic analysis in identifying atypical phenotypes and providing tailored patient care. The predominance of VUS underscores challenges in variant interpretation and family counseling. The increasing accessibility of NGS panels enhances diagnostic accuracy, yet pre‐test counseling remains critical to prepare patients for possible inconclusive results.

**Disclosure:** Nothing to disclose.

## EPO‐504

### A presentation of PDGFRB‐related parkinsonism without cranial CT calcifications

#### V. Busco ^1^; A. Cimmino^1^; M. Petracca^1^; M. Pomponi^2^; D. Tiziano^2^; P. Calabresi^1^; A. Bentivoglio^1^; G. Di Lazzaro^1^

##### ^1^Neurology Unit, Fondazione Policlinico Universitario Agostino Gemelli IRCCS, Rome, Italy; ^2^Istituto di Medicina Genomica, Fondazione Policlinico Universitario Agostino Gemelli IRCCS, Rome, Italy

**Background and Aims:** Mutations in PDGFRB are linked to Primary Familial Brain Calcification (PFBC), a neurodegenerative disorder marked by basal ganglia calcifications [1]. Tadic et al. provided a systematic review of neuroimaging in PFBC with known gene mutations, showing calcifications on cranial CT in all cases [2].

**Methods:** We present a 46‐year‐old man with young onset parkinsonism and likely pathogenic PDGFRB variants, but without CT‐detected calcifications. Symptoms began at age 39 with asymmetric resting tremor and bradykinesia, minimally responsive to dopaminergic treatment. He had also developed anxiety and gambling issues prior to dopaminergic therapy, with a family history of hearing loss (father) and late‐onset Parkinson's disease (paternal grandfather).

**Results:** Magnetic resonance imaging was normal. Genetic testing (Next Generation Sequencing) revealed a Variant of Uncertain Significance (VUS) in PDGFRB (c.2325C>G, shared with the father); a second PDGFRB VUS (c.763G>A) and a LRRK2 VUS (c.7337G>A), both shared with the mother. Neither the patient nor his parents exhibited calcifications on cranial CT. PDGFRB variants follow an autosomal dominant inheritance pattern with variable expressivity. The VUS PDGFRB:c.2325C>G, present in father and likely in the affected paternal grandfather, is a null variant in a gene where loss of function is a known disease mechanism, suggesting its pathogenicity. However, parkinsonism associated with PDGFRB mutations in the absence of cranial CT calcifications has never been described before [2].

**Conclusion:** This case could highlight a novel presentation of PDGFRB mutation‐linked parkinsonism without CT calcifications, expanding the phenotypic spectrum of PDGFRB‐associated disorders. Further studies are needed to clarify the pathogenicity of these variants and their clinical manifestations.

**Disclosure:** Nothing to disclose.

## EPO‐505

### Delineation clinical phenotype spectrum in autosomal recessive SLC9A1‐related ataxia

#### Y. Mecheri ^1^; R. Maroofian^2^; S. SLC9A1 study group^3^

##### ^1^Department of Neurology, Dr Benbadis University Hospital, Constantine, Algeria; ^2^Department of Neuromuscular Diseases, UCL Queen Square Institute of Neurology, London, UK; ^3^Various International Research Centers

**Background and Aims:** Biallelic pathogenic variants in SLC9A1 gene have been linked to Lichtenstein‐Knorr syndrome (LKS) based on the consecutive report of six affected individuals. In the current study we provide expanded clinical description with functional studies for a total of 35 patients from 23 families including updated data for previously reported patients.

**Methods:** We used the GeneMatcher platform and extensive worldwide data sharing. Clinical data was collected MRI was reviewed by a pediatric neuroradiologist. All subjects gave informed consent for the publication of clinical and genetic information according to the declaration of Helsinki.

**Results:** Our cohort (mean age 16 +/‐ 13 years (range 4–51)) predominantly presented moderate‐to severe ataxia starting mostly in early childhood involving gait, trunk and limbs with dysmetria (97%) and tremor (60%). Intellectual disability (93%) was mainly mild to moderate. Amelogenesis imperfecta (91%), motor delay (91%), speech delay (76%) and cognitive impairment (86%) were particularly frequent. Sensory‐neural hearing loss (69%) was mainly severe to profound with onset mostly before the age of one year. Other features included hyporeflexia (77%) and hypotonia (59%), Seizures (50%) Abnormal eye movements (36%), dyskinetic movements (31%), dystonia (24%). Cerebellar atrophy was observed in most patients. Of the 16 variants identified, 13 were novel and functional studies were performed in eight these variants and had deleterious effects.

**Figure:**
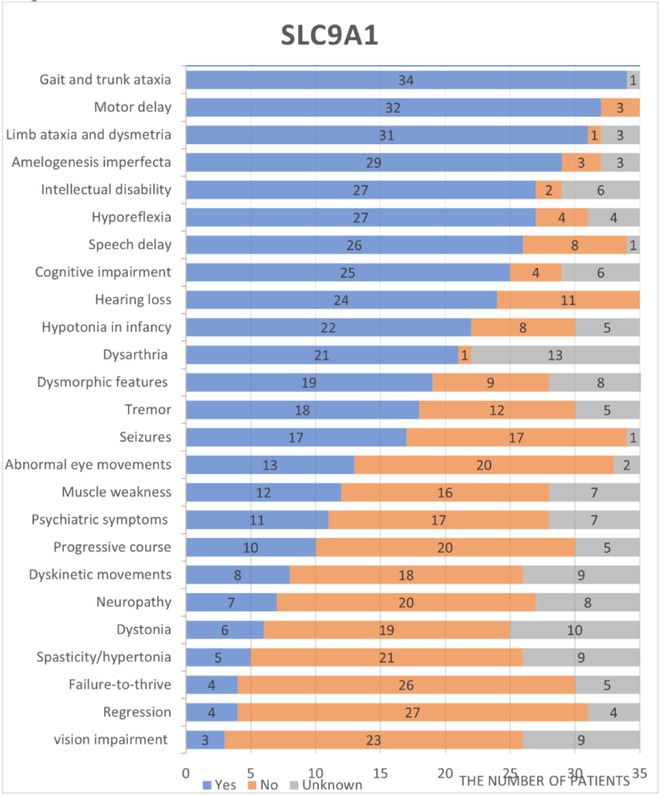
**FIGURE 1** Clinical features of patients with SLC9A1‐related ataxia

**Conclusion:** The association of hearing loss, amelogenesis imperfecta, intellectual disability, motor delay and cerebellar atrophy allow to suspect SLC9A1‐linked ataxia.

**Disclosure:** Nothing to disclose.

## EPO‐506

### Vestibulo‐ocular reflex gain and vestibular evoked potentials in patients with spinocerebellar ataxia

#### Z. Calic ^1^; A. Dyball^2^; D. Herrero^3^; N. Young^2^; S. Rosengren^2^; K. Kumar^4^; M. Welgampola^2^

##### ^1^Department of Neurophysiology, Liverpool Hospital, Australia; ^2^Institute of Clinical Neurosciences, Royal Prince Alfred Hospital, Camperdown, Australia; ^3^Faculty of Medicine of Clinica Alemana, Universidad del Desarrollo, Santiago de Chile, Chile; ^4^Concord Repatriation General Hospital, Department of Neurology, Concord, Australia

**Background and Aims:** The spinocerebellar ataxias (SCAs) are a genetically heterogeneous group of autosomal dominantly inherited progressive disorders characterised by loss of balance and coordination.

**Methods:** 16 patients (SCA3 *n* = 6, SCA6 *n* = 4, SCA7 *n* = 2, SCA14 *n* = 2, SCA34 *n* = 2) with mean age 56.0 ± 11.1 years (5F/11M), were recruited from Outpatient Balance Neurology Clinic. Video Head‐Impulse test was used to assess vestibulo‐ocular reflex (VOR) function in all three canal planes and cervical and ocular‐Vestibular‐Evoked Myogenic Potentials (c‐ and oVEMP) were used to assess otolith function.

**Results:** Mean International Co‐operative Ataxia Rating Scale (ICARS) was 30.1 ± 15.0 and Scale for the assessment and rating of ataxia mean (SARA) score was 10.2 ± 5.5. Saccadic pursuit was present in 13 (81%) patients, and loss of VOR suppression in 11 (69%) patients. Air‐conducted cVEMPS were absent in 4 (25%) patients and bone‐conducted oVEMPS were absent in 5 (31%) patients. Horizontal, anterior and posterior canal (HC, AC, PC) VOR‐gains were 0.63 ± 0.2, 0.67 ± 0.3, 0.60 ± 0.3. HC, AC and PC VOR‐gain were reduced in 12 (75%) patients, 10 (62%) and 11 (68%) patients. All patients with SCA3 had VOR‐gain reduced in all six‐canals. Patients with SARA scores ≥10 had significantly lower HC VOR‐gain compared to the patients with mild disease severity (0.49 vs 0.76, *p* = 0.015). There was a negative correlation between SARA score and HC VOR‐gain (*r* = ‐4.001, *p* = 0.034).

**Conclusion:** In patients with SCAs reduction in the VOR‐gain is common and is associated with higher degree of functional impairment.

**Disclosure:** Nothing to disclose.

## Neuro‐ophthalmology/neuro‐otology

## EPO‐507

### Type III cryoglobulinemia presenting with acute cochleovestibular loss

#### A. Costa ^1^; F. Bartolomeu^2^; M. Amorim^2^; D. Damas^3^; S. Matos^3^; A. Inês Martins^3^; A. Jorge^3^; J. Lemos^3^; J. Lemos^4^

##### ^1^Department of Neurology, Local Health Unit of Trás‐os‐Montes and Alto Douro, Vila Real, Portugal; ^2^Department of Otorhinolaryngology, Local Health Unit of Coimbra, Coimbra, Portugal; ^3^Department of Neurology, Local Health Unit of Coimbra, Coimbra, Portugal; ^4^Faculty of Medicine, Coimbra University, Coimbra, Portugal

**Background and Aims:** Type III cryoglobulinemia is a mixed cryoglobulinemia involving polyclonal immunoglobulins that form immune complexes, depositing in small to medium‐sized vessels and causing end‐organ damage. Symptoms include purpura, arthralgias, weakness, and rarely, cochleovestibular loss.

**Methods:** We describe a type III cryoglobulinemia patient with sequential acute cochleovestibular loss.

**Results:** A 26‐year‐old female presented with sudden painless left‐sided hearing loss and imbalance. The use of ototoxic agents was ruled out. Her medical history was notable for a previous episode of acute cochleovestibular loss on the right side 2 years prior (characterized by right moderate sensorineural hearing loss on audiogram, right gain reduction on VHIT and normal brain and inner ear MRI), without an established etiology. An audiogram confirmed acute left‐sided sensorineural hearing loss, and VHIT demonstrated reduced gain values bilaterally, now indicating new‐onset left vestibular nerve dysfunction. Brain and inner ear MRI were again unremarkable. Autoimmune assessment revealed the presence of cryoglobulins (polyclonal IgM), indicative of type III cryoglobulinemia. Other laboratory studies were unremarkable. She was treated with a 125 mg bolus of methylprednisolone and a 5‐day course of 60 mg deflazacort and was putted on aspirin. Imbalance fully improved spontaneously, while the hearing loss remained unchanged after one month.

**Conclusion:** This case highlights inner ear dysfunction in type III cryoglobulinemia, manifesting as sequential cochleovestibular loss. Although audiovestibular symptoms are uncommon, they occur in up to one‐third of mixed cryoglobulinemia patients, possibly due to cryoglobulin precipitation in inner ear vessels causing vasculitis. Mixed cryoglobulinemia should be considered in unexplained cochleovestibular loss.

**Disclosure:** Nothing to disclose.

## EPO‐508

### Central positional downbeat nystagmus responsive to clonazepam in SCA3

#### D. Damas ^1^; A. Sobral‐Pinho^2^; S. Matos^1^; A. Martins^1^; A. Jorge^1^; J. Lemos^1^

##### ^1^Neurology Department, Coimbra University Hospital, Coimbra, Portugal; ^2^Neurology Department, Ocidental Lisbon Local Health Unit, Lisbon, Portugal

**Background and Aims:** Ocular motor phenotypes in spinocerebellar ataxias (SCA) have been progressively described, with some features predominating over others, depending on genotype. As an example, central positional nystagmus is extremely rare in SCA3, while being present in ~80% of SCA6 patients. The treatment of CPN poses a clinical challenge, as only a few drugs have been anecdotally reported to improve CPN in some (but not in others) SCA patients.

**Methods:** Case report of SCA3 patient with symptomatic CPN and positional square‐wave‐jerks with a striking response to clonazepam.

**Results:** A 28‐year‐old SCA3 male patient presented with 3‐year positional vertigo. Exam in upright position showed square‐wave‐jerks and microflutter, gaze evoked nystagmus, impaired pursuit, hypometric saccades, impaired head impulses, partial upgaze restriction and divergence/convergence insufficiency. During positional testing, head‐hanging maneuver showed after ~5sec latency, torsional square wave jerks ~5sec followed by paroxysmal downbeat nystagmus ~10sec, associated with intense vertigo and patient's unwillingness to repeat maneuvers. Positional central downbeat nystagmus and torsional square wave jerks were diagnosed and prescribed with oral clonazepam 0.5 mg once daily. Two months later, the patient reported complete resolution of positional vertigo. Head‐hanging maneuver showed no nystagmus or saccadic intrusions, indicating optimal response to treatment.

**Conclusion:** Oral clonazepam might have abated central positional nystagmus in the current case by restoring cerebellar nodulus/uvula inhibitory output on vestibular nuclei, through GABA‐related circuitry. Given the scarce literature, multicentric studies on SCA‐related CPN are most needed to help guide treatment. Similar treatment efficiency on positional saccadic intrusions, a yet largely neglected entity, equally deserves further research.

**Disclosure:** Nothing to disclose.

## EPO‐509

### A diagnostic challenge: Differentiating thyroid orbitopathy from carotid‐cavernous fistula

#### E. Akalin Akkas; I. Kalyoncu Aslan; E. Gozke

##### Department of Neurology, Fatih Sultan Mehmet Training and Research Hospital, Istanbul, Turkey

**Background and Aims:** Carotid‐cavernous fistula (CCF) is an abnormal connection between the internal carotid artery (ICA) and the cavernous sinus, affecting cranial nerves III, IV, V1, V2, and VI. It presents with vision‐threatening symptoms like proptosis, blurry vision, chemosis, headache, and ophthalmoplegia. Early‐stage CCF can mimic other conditions, such as thyroid‐associated ophthalmopathy (TAO), presenting with diplopia, progressive proptosis, conjunctival congestion, and chemosis.

**Methods:** A 67‐year‐old male presented with left eye redness lasting three months, without trauma or significant medical history. Physical examination revealed left‐sided proptosis, chemosis, restricted eye movements, and dilated episcleral vessels. Thyroid function tests showed mildly suppressed TSH and elevated thyroid antibodies, suggestive of thyroiditis. Thyroid ultrasonography identified a solid nodule and increased vascularity in a heterogeneous thyroid gland. Imaging findings included hyperintensity in the retrobulbar tissues on MRI, thickened medial and inferior rectus muscles, and a prominent ophthalmic vein. MR venography revealed narrowing and discontinuity in the superior sagittal sinus. Diagnostic angiography confirmed an indirect CCF originating from meningeal branches of the right ICA and external carotid arteries (ECA), draining into the left cavernous sinus.

**Figure:**
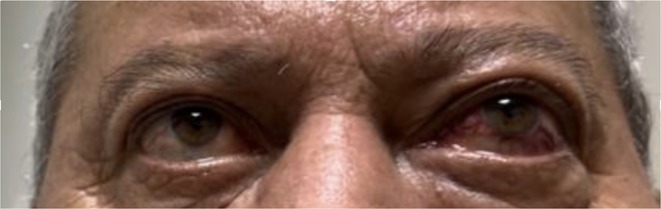
**FIGURE 1** Bilateral exophthalmos, more pronounced on the left side, along with proptosis and chemosis

**Figure:**
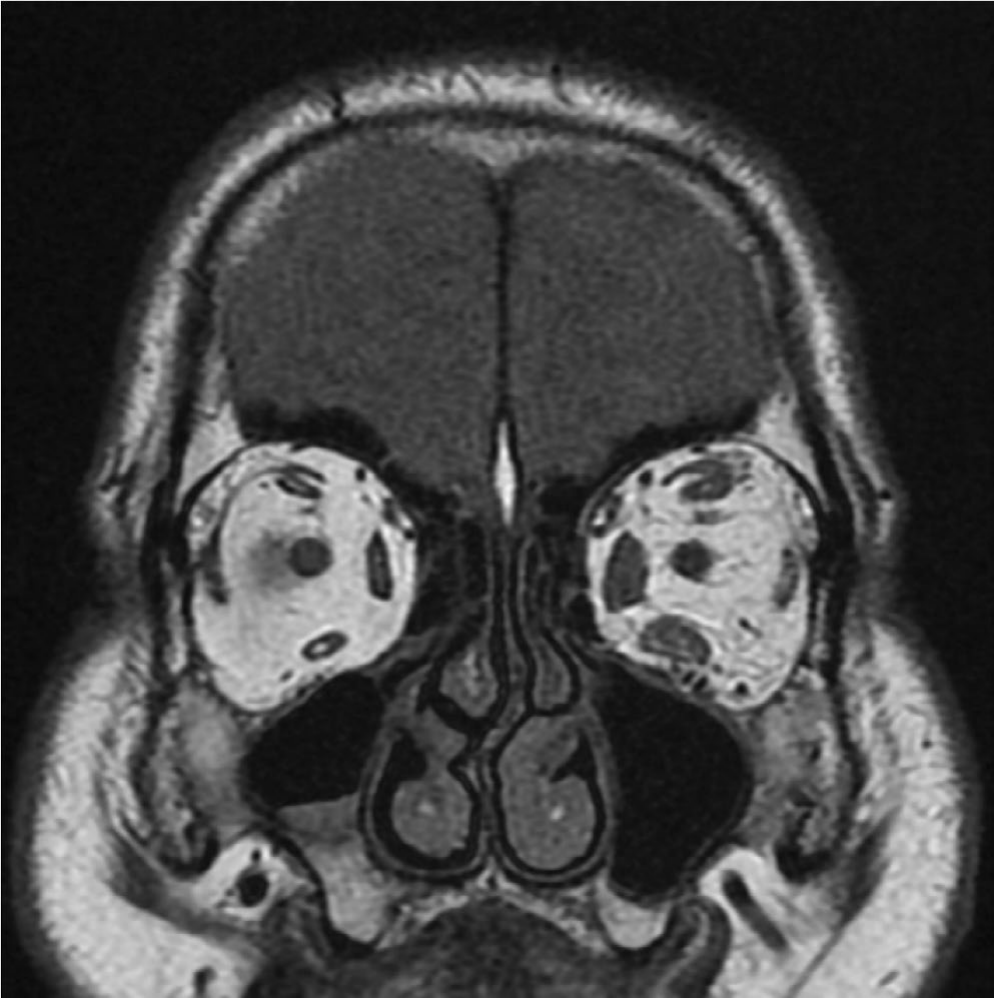
**FIGURE 2** Orbital MRI demonstrated thickening of the medial and inferior rectus muscles in the left orbit

**Figure:**
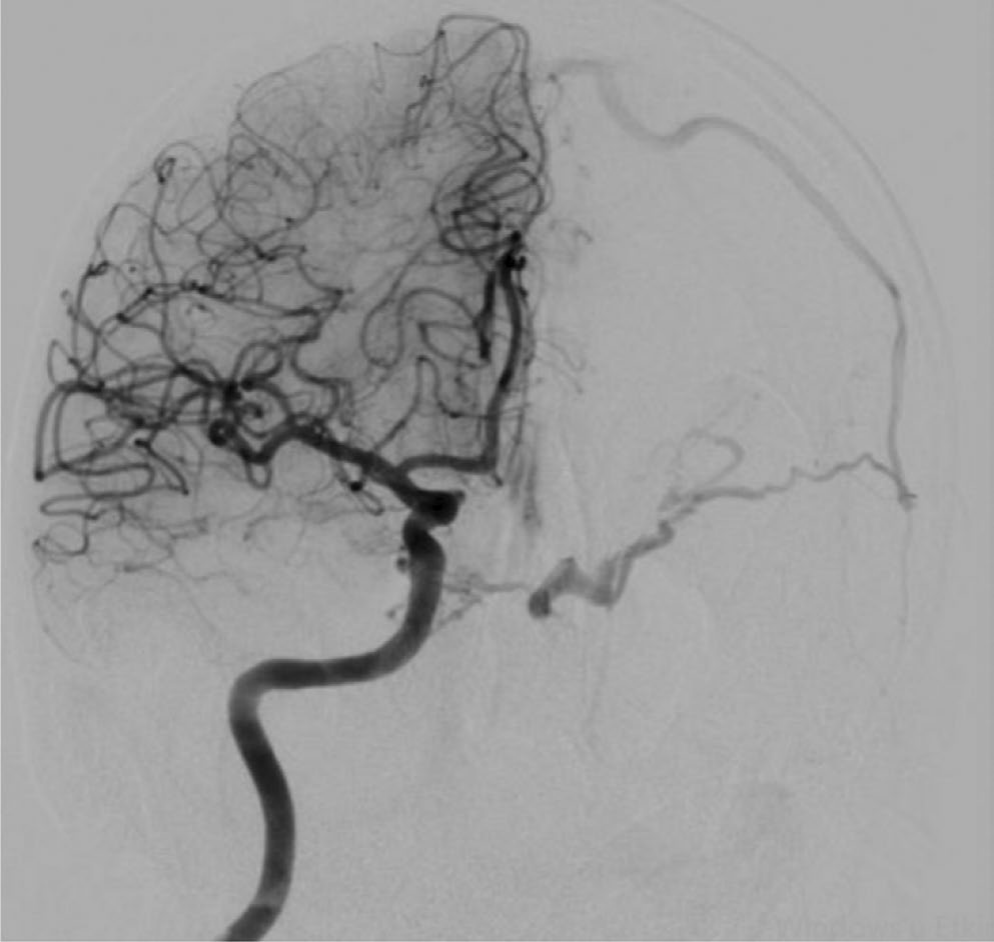
**FIGURE 3** DSA confirmed an indirect CCF originating from meningeal branches of the right ICA and ECA, draining into the left cavernous sinus.

**Results:** This case highlights the diagnostic challenges of distinguishing CCF from TAO, emphasizing the importance of cerebral angiography as the gold standard for diagnosis. Endovascular intervention, the preferred treatment, successfully resolves most cases.

**Conclusion:** Clinicians should maintain a high index of suspicion for CCF in patients with atypical orbital symptoms to ensure timely and accurate management.

**Disclosure:** Nothing to disclose.

## EPO‐510

### Vestibular agnosia in elderly subjects with small vessel disease and imbalance – Perliminary results

#### F. Honegger^1^; C. Stieger^2^; B. Semungal^3^; H. Rust ^1^

##### ^1^Vestibular Neurology Unit, Department of Neurology, University of Basel Hospital, Basel, Switzerland; ^2^Division of Neuro‐Otology, Department of ORL, University of Basel Hospital, Basel, Switzerland; ^3^Centre for Vestibular Neurology, Imperial College London, London, UK

**Background and Aims:** Vestibular agnosia (VA) is a brain disconnection syndrome that manifests with imbalance and an altered sensation of self‐motion perception despite an intact peripheral vestibular system. VA can be measured and quantified employing vestibular psychophysics. Only recently VA was described for the first time in acute traumatic brain injury. VA has been reported to occur in elderly patients in whom there is disconnection within the brain due to small vessel disease (SVD). In an ongoing exploratory prospective single‐centre study we assess elderly patients with imbalance and dizziness for vestibular perceptual thresholds (VPTs) and balance control.

**Methods:** 4 patients with SVD (Fazekas 2–3) and imbalance/ falls were assessed, 2 males, 2 females (age 75, 76, 81, 82 years). 4 elderly controls were assessed, 1 male, 3 females (age 72, 74, 79, 89 years). VPTs were determined for yaw‐plane rotations with randomly presented half cosine stimuli (0.1 Hz). Peripheral vestibular function, balance control, handedness, reaction times, cognition and self‐reported symptoms of dizziness, anxiety and depression were assessed.

**Results:** 4 patients with SVD and imbalance had elevated VPTs (range 6.3–19.9°/s; cut‐off 5°/s for controls, mean age 42.3 years) despite intact peripheral vestibular function and abnormal balance control. 4 controls had VPTs within a low normal range (0.8–2.7°/sec). 3 patients indicated only slight handicap because of dizziness.

**Conclusion:** VPTs in elderly subjects over the age of 70. VPTs might be within normal ranges and severe as a biomarker for imbalance and risk of falls in elderly subjects.

**Disclosure:** HMR has received funding from Freiwillige Akademische Gesellschaft Basel (FAG), lecture fees from Forum für medizinische Fortbuildung (FomF) and Volkshochschule Basel CS has nothing to disclose BMS has nothing to disclose FH has nothing to disclose.

## EPO‐511

### Biomarkers in patients with optic neuritis: Demographic, clinic and prognostic features

#### H. Özdemir

##### Ege University Medical School, Department of Neurology, İzmir, Turkey

**Background and Aims:** The new optic neuritis (ON) classification leads to a change in how ON patients are grouped. Our aim is to appraise the clinical features and prognoses of patients with autoimmune ON not associated with MS.

**Methods:** Patients referred to our neuro‐ophthalmology laboratory were enrolled to this retrospective study. Patients with ON associated with MS were excluded. The remaining patients were divided into three groups: aqauaporin‐4 (AQP4) antibody ON group, myelin oligodendrocyte glycoprotein (MOG) antibody ON group, and seronegative ON group. The patients were examined on admission, one month after acute treatment, and at the third‐year follow‐up. We compared demographic, clinical, radiologic, laboratory data, and treatment responses among these three groups.

**Results:** The study included 92 patients with 120 eyes. The older age of onset, bilateral simultaneous involvement of the optic nerves, severe vision loss at onset, and need for aggressive treatment were more common in the AQP‐ON and the MOG‐ON groups than the seronegative ON group (*p* = 0.01, *p* = 0.003, *p* = 0.011, *p* = 0.007, *p* < 0.001, *p* < 0.001, respectively). The presence of optic disc edema was a significant feature of MOG‐ON as well as long‐length contrast enhancement on MRI (*p* = 0.003, *p* = 0.002). Additional autoimmune antibodies and CNS lesions outside the optic nerve were the features of AQP4‐ON patients (*p* < 0.001, *p* = 0.015). Generalized estimating equations analysis revealed that the presence of AQP4 antibody, increased age and recurrence were associated with visual acuity over time (*p* = 0.014, *p* = 0.002, *p* = 0.016, respectively).

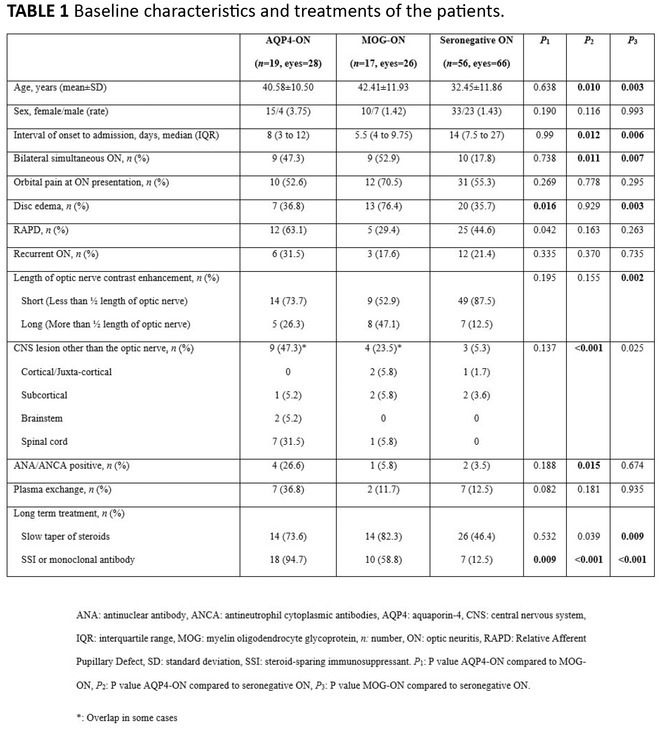

**Figure:**
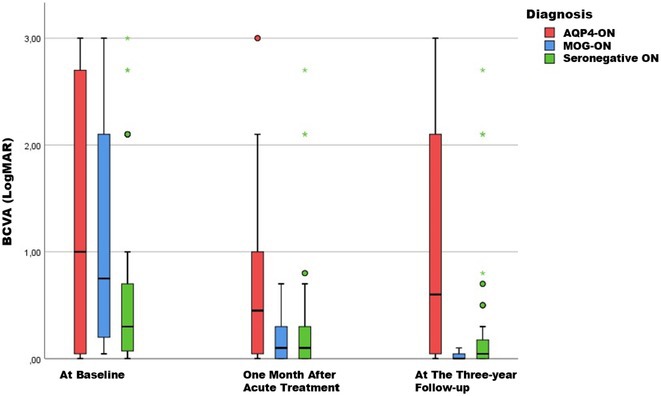
**FIGURE 1** A clustered boxplot graphic showing patients’ visual acuities. BCVA: best corrected visual acuity, LogMAR: Logarithm of the Minimum Angle of Resolution.

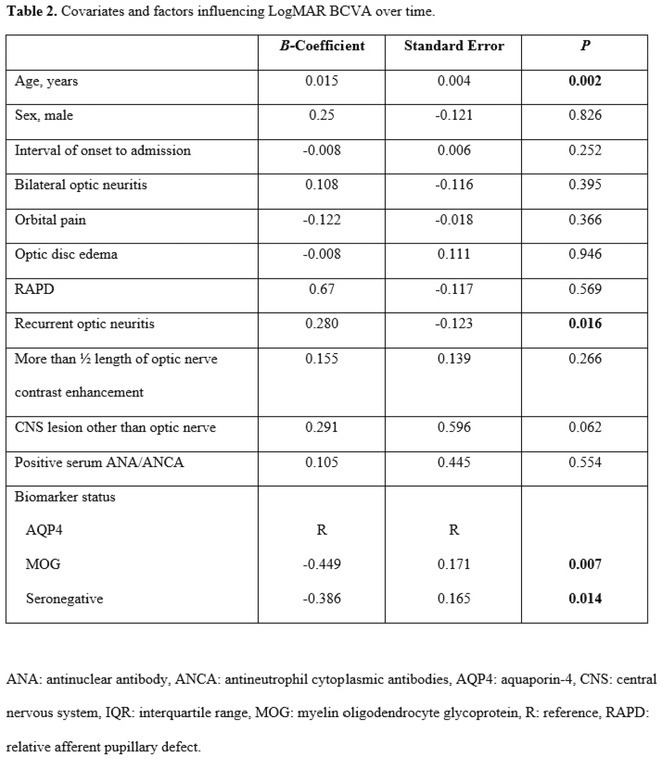

**Conclusion:** The association of serum biomarker status with demographic, clinical features, and visual outcomes indicate the importance of biomarker detection in these patients.

**Disclosure:** Nothing to disclose.

## EPO‐512

### Ocular dysmotility in orbital neoplasms

#### M. Kang^1^; H. Yang^2^; N. Kim^2^; J. Hwang ^2^

##### ^1^Department of Ophthalmology, Kyung Hee University Hospital, Seoul, Repuplic of Korea; ^2^Department of Ophthalmology, Seoul National University College of Medicine, Seoul National University Bundang Hospital, Seongnam, Republic of Korea

**Background and Aims:** To analyze the pattern of ocular motility disturbances caused by orbital tumors, and investigate factors associated with the severity of misalignment or motility limitation.

**Methods:** The medical records of 68 patients with orbital neoplasm were retrospectively reviewed. The location, type, and size of tumor were noted. The limitation of extraocular muscle (EOM) movement was quantitatively scored for upgaze, downgaze, abduction, and adduction by the severity of limitation on a grading scale. The maximum range of abduction, adduction, supraduction and infraduction were measured by image analysis based on 9 gaze photographs using the 3D Strabismus Photo Analyzer.

**Results:** Twenty patients(29%) patients had extraconal orbital tumors (60%), while 8 had intraconal lesions(40%).The mean size of extraconal tumors was significantly larger than intraconal tumors (*p* = 0.010), and there were no significant differences in tumor size between each four quadrants. In 75% of patients with extraconal neoplasms, ocular motility was limited towards the action of the adjacent EOM. Conversely, in cases with intraconal neoplasms, ocular motility was limited towards the opposite direction of the nearest EOM action in 75% cases. Compared with the normal fellow eye, significant limitations of ocular motility were observed in adduction (*p* = 0.043) and supraduction (*p* = 0.002). The size of intraconal tumors was the only significant factor related to ocular motility limitation (*p* = 0.010).

**Conclusion:** A significant movement restrictions in upgaze and adduction varied depending on whether the tumor was located extraconally or intraconally. The size of orbital tumor in the intraconal region was the only factor related to ocular motility limitation.

**Disclosure:** Nothing to disclose.

## EPO‐513

### Spatial orientation impairment in patients with bilateral vestibulopathy and persistent postural‐perceptual dizziness

#### J. Gerb ^1^; V. Oertle^2^; S. Becker‐Bense^2^; T. Brandt^3^; M. Dieterich^4^

##### ^1^German Center for Vertigo and Balance Disorders, LMU University Hospital; Department of Neurology, LMU University Hospital, Munich, Germany; ^2^German Center for Vertigo and Balance Disorders, LMU University Hospital, Munich, Germany; ^3^German Center for Vertigo and Balance Disorders, LMU University Hospital; Graduate School of Systemic Neuroscience, LMU Munich; Hertie Senior Professor for Clinical Neuroscience, LMU Munich, Munich, Germany; ^4^German Center for Vertigo and Balance Disorders, LMU University Hospital; Graduate School of Systemic Neuroscience, LMU Munich; Department of Neurology, LMU University Hospital; Munich Cluster for Systems Neurology (SyNergy), Munich, Germany

**Background and Aims:** Spatial orientation abilities typically decrease with higher age, cognitive impairment or vestibular disorders. Bilateral vestibulopathy (BVP) is known to be associated with poorer performance in orientation and navigation tasks. Less is known about possible visuospatial deficits in persistent postural‐perceptual dizziness (PPPD).

**Methods:** 32 patients with BVP (17 females), 43 with PPPD (25 females) and 32 healthy controls (HC, 15 females) participated in a clinical bedside test investigating spatial orientation abilities (3D real‐world pointing task, 3D‐RWPT). This test includes a cognitive (mental rotation) and a vestibular paradigm (passive whole‐body rotation without visual feedback). All participants had normal cognitive testing results and were younger than 65 years. Participants additionally filled out self‐reports of subjective spatial abilities and spatial orientation discomfort. PPPD and HC had normal peripheral‐vestibular function.

**Figure:**
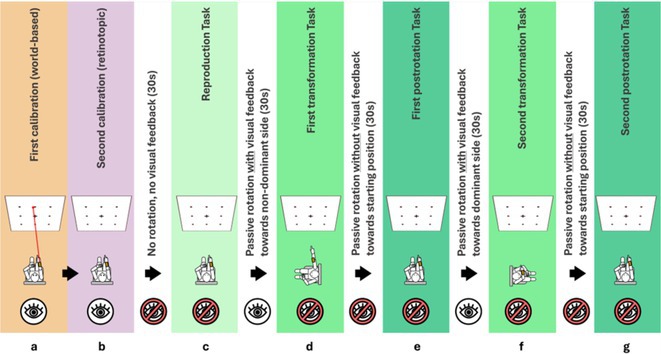
**FIGURE 1** Overview of the 3D‐RWPT. The full test consists of two calibration paradigms (a, b) and five test paradigms (c–f).

**Results:** Patients with BVP and PPPD showed substantially larger angular deviations (i.e., lower accuracy) in the 3D‐RWPT compared to HC (BVP: 9.62 ± 3.21°, PPPD: 9.16 ± 3.85, HC: 7.77 ± 2.86°; *p* 0.03*), especially in the subtasks which rely on vestibular function (BVP: 8.11 ± 5.51°, PPPD: 6.62 ± 4.46, HC: 4.45 ± 2.33°; *p* < 0.01**). All cohorts had comparable levels of self‐assessed spatial abilities, while both BVP and PPPD patients showed higher levels of spatial orientation discomfort.

**Conclusion:** PPPD patients exhibited measurable visuospatial deficits in the 3D‐RWPT, although these deficits were not as pronounced as in BVP patients. Since especially the vestibular subtasks were affected, one might speculate that PPPD patients show a reduced use of vestibular input and rely more on visual input. Both PPPD and BVP patients reported spatial orientation discomfort.

**Disclosure:** Nothing to disclose.

## EPO‐514

### Downbeat nystagmus, symptom of a sensorimotor syndrome of vertical spatial disorientation

#### J. Gerb ^1^; V. Oertle^2^; S. Becker‐Bense^2^; E. Kierig^1^; M. Dieterich^3^; T. Brandt^4^

##### ^1^German Center for Vertigo and Balance Disorders, LMU University Hospital; Department of Neurology, LMU University Hospital, Munich, Germany; ^2^German Center for Vertigo and Balance Disorders, LMU University Hospital, Munich, Germany; ^3^German Center for Vertigo and Balance Disorders, LMU University Hospital; Graduate School of Systemic Neuroscience, LMU Munich; Department of Neurology, LMU University Hospital; Munich Cluster for Systems Neurology (SyNergy), Munich, Germany; ^4^German Center for Vertigo and Balance Disorders, LMU University Hospital; Graduate School of Systemic Neuroscience, LMU Munich; Hertie Senior Professor for Clinical Neuroscience, LMU Munich, Munich, Germany

**Background and Aims:** Downbeat nystagmus (DBN), the most common form of acquired central fixation nystagmus, causes vertical oscillopsia, imbalance and postural instability in the anterior‐posterior direction. So far, DBN‐related alterations of the internal representation of space have not been investigated.

**Methods:** 20 patients with DBN (13 females, mean age 69.97 ± 10.17 years) and 20 age‐, sex‐, handedness‐, and cognition‐matched participants (13 females, mean age 69.87 ± 9.52 years) without peripheral‐vestibular deficits underwent a spatial orientation bedside test (3D real‐world pointing task, 3D‐RWPT) based on whole‐arm pointing at a target matrix. For both cohorts, the subjective haptic straight ahead (SHA) with eyes closed was measured. Additionally, quantitative (horizontal and vertical angular deviation) and qualitative performance markers (morphology of target matrix) were investigated. DBN intensity was measured using videooculography.

**Figure:**
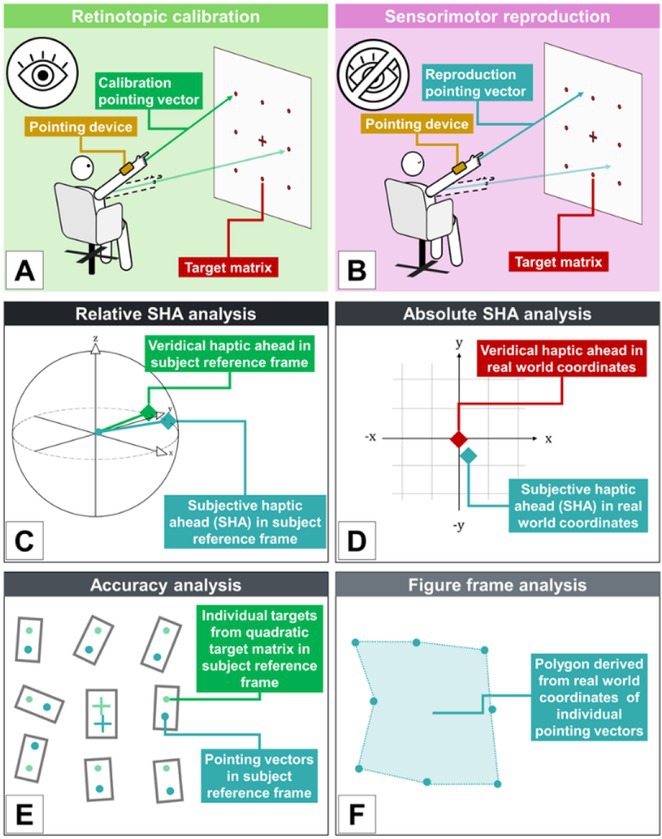
**FIGURE 1** Depiction of the 3D‐RWPT and the analyses performed in this study.

**Results:** Compared to the control cohort, the DBN cohort exhibited an absolute (real‐world coordinates) and relative (subject reference frame) SHA downward shift. DBN patients showed vertically (but not horizontally) decreased pointing accuracy (Welch's *t*‐test azimuth/horizontal deviation: n.s.; polar/vertical deviation: *t* = 3.85, *p* < 0.001***). DBN intensity was not robustly associated with 3D‐RWPT performance or postural swaying patterns.

**Conclusion:** DBN is associated with a SHA downward shift in a pointing task performed without visual feedback. This is contradirectional to prior research investigating the subjective visual ahead, which is shifted upwards in DBN. These findings indicate that DBN‐associated spatial impairment is not solely caused by oscillopsia and thus an ocular motor syndrome, but persists even without visual input, hinting at central‐vestibular alterations of spatial perception of head and body in space.

**Disclosure:** Nothing to disclose.

## EPO‐515

### Unveiling dual diagnoses: Papilledema and pseudopapilledema in a series of patients

#### L. Silva ^1^; A. Jorge^1^; S. Matos^1^; D. Damas^1^; B. Silva^2^; A. Martins^1^; J. Lemos^1^

##### ^1^Neurology Department, Coimbra University Hospital Centre, Coimbra, Portugal; ^2^Faculty of Medicine, Coimbra University, Coimbra, Portugal

**Background and Aims:** Papilledema indicates the presence of bilateral optic disc edema caused by intracranial hypertension (IH), and is potentially associated with sight‐ and/or life‐threatening conditions. Pseudopapilledema consists of a disk elevation associated with underneath tilted disks/high myopia (TD), optic disk drusens (ODD) or other variants, which carries no relevant risk.

**Methods:** This study describes a retrospective series of patients demonstrating the rare instance of presenting with both pseudopapilledema and papilledema.

**Results:** We included 7 patients (4 males, mean age 34.5 ± 18.2 years). Presenting symptom/sign leading to neuro‐ophthalmic assessment included: headache (2), transient monocular vision loss (2), pulsatile tinnitus (1), seizure (1) and incidental fundoscopic finding (2). Pseudopapilledema consisted of: OOD (4), TD (2) and ODD plus TD (1). OCT showed retinal nerve fiber layer thickening in 6, and ODD in 5 patients. The initial presumptive neuro‐ophthalmic diagnosis was: definite papilledema and pseudopapilledema (3), definite pseudopapilledema and possible papilledema (2), definite pseudopapilledema (1), and definite papilledema (1). In the first two groups, CSF opening pressure and papilledema improvement after oral acetazolamide confirmed the concurrent presence of papilledema associated with idiopathic IH and pseudopapilledema. In the patient with presumptive pseudopapilledema (ODD and meningioma with apparently no imaging signs of IH), follow‐up retinography clearly showed papilledema resolution while leaving unchanged baseline ODD. In the patient with presumptive papilledema, after complete resolution of the papilledema with oral acetazolamide for idiopathic IH, baseline TD were now apparent.

**Conclusion:** The presence of pseudopilledema should never preclude the active exclusion of papilledema, as both can occur simultaneously.

**Disclosure:** Nothing to disclose.

## EPO‐516

### Unrecognized benign paroxysmal positional vertigo in frail in‐patients

#### L. Martinkovic ^1^; O. Cakrt^2^; K. Kucerova^2^; S. Koutna^2^; M. Kvapil^3^; K. Slaby^2^; J. Jerabek^1^

##### ^1^Department of Neurology, Second Faculty of Medicine, Charles University and Motol University Hospital, Prague, Czechia; ^2^Department of Rehabilitation and Sports Medicine, Second Faculty of Medicine, Charles University and Motol University Hospital, Prague, Czechia; ^3^Department of Geriatrics, Second Faculty of Medicine, Charles University and Motol University Hospital, Prague, Czechia

**Background and Aims:** Dizziness is a common complaint, its incidence increases with age and is often associated with balance disorders and predispose the patient to falling. Benign paroxysmal positional vertigo (BPPV) is an important cause which can easily be treated but is frequently not recognized by professionals. We sought to determine the prevalence of unrecognized BPPV in hospitalized elderly patients.

**Methods:** Patients admitted to Department of Geriatrics were enrolled in the study. A questionnaire was used to obtain uniform and detailed history, patients with previous diagnosis of BPPV were excluded. A clinical neuro‐otologic examination (including Romberg test, spontaneous and gaze nystagmus) was performed, as well as Dix‐Hallpike and Supine‐Roll test. Prevalence was tested with Fisher's exact test.

**Results:** We evaluated a sample of 77 patients (53 women) during 3‐month period (mean 79.1 ± 10.3 years). There was no statistically significant difference in age or sex between groups. 41 (53.2 %) of them reported a fall in the past six months and 38 (49.4 %) dizziness. Definite BPPV was found in 8 patients (10.1 %). Most (87.5%) referred symptom was dizziness during standing up and a sensation of spinning in general (all *p* < 0.05). Only half of them reported positional‐related symptoms after head movement or rolling in the bed (*p* < 0.05).

**Conclusion:** These data indicated that unrecognized BPPV is quite common in elderly in‐patient population. Only half of them reported positional related symptoms, but most of them nonspecific dizziness. Diagnostic maneuvers should be part of the essential examination algorithm and successful treatment of BPPV may reduce morbidity and mortality.

**Disclosure:** Nothing to disclose.

## EPO‐517

### Bedside assessment of square‐wave jerks in movement disorders: Evaluating reliability with quantitative oculography

#### M. Kouvli; I. Stamelos; G. Armenis; V. Constantinides; C. Koros; S. Papageorgiou; L. Stefanis; E. Anagnostou

##### Department of Neurology, Eginition Hospital, National and Kapodistrian University of Athens, Greece

**Background and Aims:** Square‐wave jerks (SWJs) are clinically observable fixation intrusions that occur with increased frequency in various neurological conditions, particularly movement disorders. Although SWJs are included in the diagnostic criteria for progressive supranuclear palsy, the reliability of their clinical detectability at the bedside has never been formally evaluated.

**Methods:** Patients with various movement disorders were prospectively examined by a neurologist specializing in movement disorders. The number of SWJs during one minute of straight‐ahead fixation was counted at the bedside. Each patient also underwent binocular video‐oculography while fixating on a visual target in the primary position, with SWJs detected. Offline using custom‐written MATLAB software. Physicians operating the oculography setup were blinded to the clinicians' SWJ counts, and vice versa.

**Results:** A total of 21 patients (17 female, mean age: 64 years, range: 36–78 years) with various movement disorders, including Parkinson's disease, Parkinson‐plus syndromes, cerebellar ataxia and Stiff‐Person Syndrome, were included in the study. The agreement between bedside and apparatus‐based SWJ counts was poor, as indicated by an intraclass correlation coefficient (ICC) of 0.268 (95% CI: −0.900 to 0.709). Bedside counting underestimated SWJ counts in 17 patients and overestimated them in 4 patients. When using a threshold of >10 SWJ/min to define abnormality, the agreement between bedside assessment and oculography remained poor, with a Cohen's kappa of 0.106 (*p* > 0.05).

**Conclusion:** Counting SWJs at the bedside does not appear to align well with quantitative measurements obtained via videooculography. Clinical assessment tends to underestimate SWJ rates in most cases, though there are a few notable exceptions where it overestimates them.

**Disclosure:** Nothing to disclose.

## EPO‐518

### Unidirectional visual motion exposure does not modify cVEMP amplitude in vestibular migraine

#### E. Cortese^1^; A. Grigore^2^; P. Castro ^3^; M. Ozen^2^; N. Koohi^2^; D. Kaski^2^

##### ^1^SENSE Research Unit, Department of Clinical and Movement Neurosciences, Institute of Neurology, University College London, London, UK; Audiology Department, School of Speech‐Language Pathology and Audiology, Faculty of Medicine, Universidad de Valparaiso; ^2^SENSE Research Unit, Department of Clinical and Movement Neurosciences, Institute of Neurology, University College London, London, UK; ^3^School of Allied Health Sciences, Faculty of Health and Life Sciences, De Montfort University, Leicester, UK; Universidad del Desarrollo, Escuela de Fonoaudiologia, Facultad de Medicina Clinica Alemana, Santiago, Chile

**Background and Aims:** Vestibular migraine (VM) is the leading cause of non‐positional episodic vestibular complaints. It results from an altered state in the brain that disrupts sensory processing across various modalities. A reliance on clinical history alone for diagnosis dictates a need for bedside biomarkers for the early diagnosis of this condition, particularly in emergency settings where misdiagnosis rates are highest.

**Methods:** A cross‐sectional study utilizing non‐probabilistic sampling was conducted. Seventeen healthy controls (mean age = 34, age range = 21–55; 13 females) and seventeen patients (mean age = 38.7, age range = 20–64; 15 females) diagnosed with VM were recruited from acute vertigo clinics at the UCLH. The study protocol involved repeated cVEMP (cervical vestibular evoked myogenic potentials) measurements, before and after a Unidirectional Visual Motion Stimuli presented through VR (Virtual Reality) goggles.

**Results:** No significant differences in amplitude response (μv) were found between groups at baseline. A statistically significant difference in Right ‐ Left cVEMP amplitude asymmetry between groups was noted. SPANOVA revealed no significant effects for maximum amplitude. No significant correlation was found between questionnaire‐based symptom burden scores and changes in cVEMP amplitude following visual stimulation, in either group.

**Conclusion:** The study found that a prolonged unidirectional visual stimulation does not affect cVEMP responses in vestibular migraine patients, making it unsuitable as a bedside diagnostic biomarker for VM.

**Disclosure:** Nothing to disclose.

## EPO‐519

### Visual assessment in cerebral venous thrombosis: a prospective retinographic, perimetric and tomographic study

#### S. Matos ^1^; A. Martins^1^; A. Jorge^1^; D. Damas^1^; I. Pais^1^; B. Silva^1^; P. Fonseca^2^; J. Sousa^1^; F. Silva^1^; C. Nunes^3^; M. Castelo Branco^4^; J. Sargento‐Freitas^1^; J. Lemos^1^

##### ^1^Neurology Department, Coimbra University Hospital Centre, Portugal; ^2^Ophthalmology Department, Coimbra University Hospital Centre, Portugal; ^3^Neuroradiology Department, Coimbra University Hospital Centre, Portugal; ^4^Coimbra Institute for Biomedical Imaging and Translational Research, Portugal

**Background and Aims:** Papilledema has been reported in 30‐80% of cerebral venous thrombosis (CVT) patients. Optical coherence tomography (OCT) and orbital ultrasound may be used to portray papilledema in CVT. We aim to quantitatively assess visual function of CVT patients at presentation.

**Methods:** CVT patients were prospectively recruited and separated based on the presence versus absence of papilledema. We analysed symptoms, visual function (OCT, automated perimetry, and retinography), affected sinuses (3T MRI), orbital and transcranial ultrasound.

**Results:** 39 CVT patients were recruited (9 male; mean age 40.21 ± 13.17). Sigmoid (79.5%) and transverse (76.9%) sinuses were the most affected. Symptoms included headache (97.4%), tinnitus (23.1%), focal neurological deficits (20.5%), transient visual obscurations (5.1%), vision loss (2.6%), whereas no patient had diplopia. Confrontational visual fields were abnormal in 2 (5.1%), papilledema was present in 13 (30.8%), and no patient had 6th nerve palsy. Perimetry was abnormal in 8 (20.5%) patients. The presence of focal neurological deficits was significantly associated with papilledema (*p* = 0.029) and the same trend was observed with tinittus (*p* = 0.066). Intracranial pulsatility (IC) index positively correlated with RNFL thickness in the left eye (right IC, *r* = 0.376, *p* = 0.020; left IC, *r* = 0.501, *p* = 0.001). The extent of CVT, deep venous system involvement, and symptom duration did not predict the presence of papilledema.

**Conclusion:** Mild papilledema is present in around one‐third of CVT patients and is not associated with relevant visual field loss. The presence of focal neurological deficits and increased IC predicted papilledema. Longitudinal assessment of CVT patients may further help evaluate the role of quantitative visual measures in predicting long‐term outcomes.

**Disclosure:** Nothing to disclose.

## EPO‐520

### Individual disease trajectories in childhood‐onset subacute/dynamic eyes by age at onset from the Case Record Survey‐2

#### T. Klopstock ^1^; B. Leroy^2^; P. Yu‐Wai‐Man^3^; J. van Everdingen^4^; M. Krawczynski^5^; C. Lamperti^6^; V. Carelli^7^; X. Llòria^8^

##### ^1^Friedrich Baur Institute at the Department of Neurology, LMU University Hospital, LMU Munich, Munich, Germany; ^2^Center for Medical Genetics, Ghent University Hospital, Ghent, Belgium; ^3^John van Geest Centre for Brain Repair, and MRC Mitochondrial Biology Unit, Department of Clinical Neurosciences, University of Cambridge, Cambridge, UK; ^4^Department of Neuro‐Ophthalmology, The Rotterdam Eye Hospital (OZR), BH Rotterdam, The Netherlands; ^5^Department of Medical Genetics, Poznan University of Medical Sciences, Poznan, Poland; ^6^Department of Medical Genetics and Neurogenetics, Fondazione IRCCS Istituto Neurologico Carlo Besta, Milano, Italy; ^7^IRCCS Istituto di Scienze Neurologiche di Bologna, Bologna, Italy; ^8^Chiesi Farmaceutici S.p.A., Parma, Emilia‐Romagna, Italy

**Background and Aims:** Childhood‐onset Leber hereditary optic neuropathy (LHON) is a distinct form of LHON with varied clinical presentation and often better outcomes. Improved understanding of the clinical course of childhood‐onset LHON is essential for optimising management in this patient subgroup. Here, we present individual visual acuity (VA) trajectories by age at symptom onset (OoS) in childhood‐onset subacute/dynamic (S/D) eyes from the Case Record Survey‐2 (CRS‐2; NCT02796274).

**Methods:** Retrospective clinical data were extracted from case records of LHON patients. Eligibility: aged ≥12 years at enrolment, m.11778G>A, m.3460G>A, or m.14484T>C mitochondrial DNA mutation, OoS after 1999 and ≥2 VA assessments within 5 years of OoS and prior to idebenone use. Baseline (BL) was first VA assessment after OoS; S/D eyes were those with BL≤1 year after OoS.

**Results:** A total of 51 S/D eyes were aged <15 years at OoS with a mean (± standard deviation) follow‐up time from BL of 48.0 ± 55.2 months. Distribution of age at OoS was: 6–<9 years: 27.5% (*n* = 14); 9–<12 years: 33.3% (*n* = 17); 12–<15: 39.2% (*n* = 20). Individual VA trajectories by age at OoS are shown in Figure 1. High variability in VA trajectories was observed within and between age at OoS subgroups. Rapid VA decline in the first 6 months was infrequently observed, and many eyes with follow‐up >1 year remained on‐chart (<1.68 logMAR) throughout the chronic phase.

**Figure:**
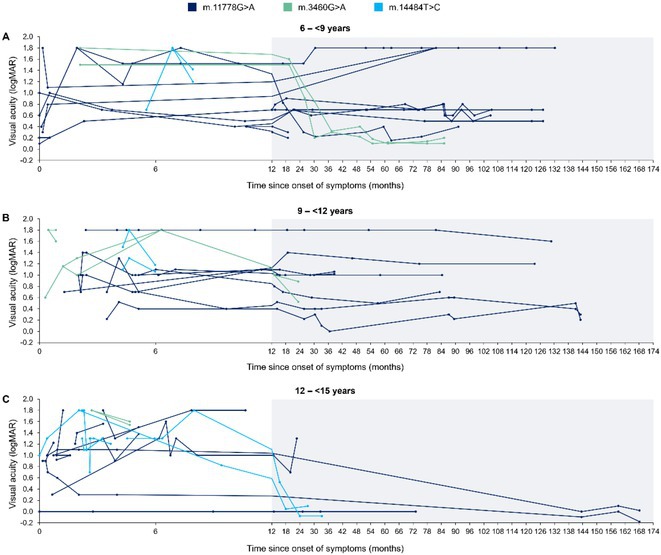
**FIGURE 1** Individual VA trajectories after OoS in childhood‐onset S/D eyes that were (A) 6.

**Conclusion:** High variability in the trend of vision loss over time in childhood‐onset eyes from CRS‐2, irrespective of age at OoS, emphasises the importance of regular follow‐up and individualised management for patients with childhood‐onset LHON.

**Disclosure:** The CRS‐2 study was funded by Santhera Pharmaceuticals. TK, PYWM, and VC received research support and/or personal compensation from Santhera Pharmaceuticals, Chiesi Farmaceutici S.p.A., and GenSight Biologics. PYWM also received consultation fees from Neurophth. BPL received consulting fees and travel support from GenSight Biologics. JvE, MK and CL have nothing to declare. XL is an employee of Chiesi Farmaceutici S.p.A. Medical writing support was provided by nspm ltd, Switzerland, and funded by Chiesi Farmaceutici S.p.A.

## Tuesday, June 24 2025

## Clinical neurophysiology

## EPO‐521

### The role of lower motor neuron dysfunction in cutaneous silent period alterations in ALS

#### A. Abramova; A. Broutian; M. Zakharova

##### Research Center of Neurology, Moscow, Russian Federation

**Background and Aims:** The aim of this study was to evaluate cutaneous silent period (CSP) in patients with amyotrophic lateral sclerosis (ALS), and investigate its correlation with lower motor neuron (LMN) dysfunction.

**Methods:** CSP was recorded from the abductor pollicis brevis (APB) muscle using strong electrical stimuli applied to the index finger of the least affected hand. LMN dysfunction was assessed using the compound muscle action potential (CMAP) and motor unit number index (MUNIX) in APB. Sixty patients with definite ALS, as defined by the El Escorial criteria (disease duration 15.5 [10; 24] months) were included, with 30 (50%) presenting with spinal‐onset ALS and 30 (50%) with bulbar‐onset ALS. The control group consisted of 48 age‐ and sex‐matched healthy subjects.

**Results:** CSP onset latency (*p* < 0.01) and duration (*p* < 0.05) were significantly prolonged in ALS patients as compared to controls. No statistically significant differences in CSP parameters were found between subgroups with spinal‐ and bulbar‐onset. CSP duration correlated positively with disease duration (*r* = 0.63, *p* < 0.05) but not with ALSFRS‐R score. MUNIX and CMAP values in ALS patients were significantly lower than in controls (*p* < 0.05). CSP onset latency showed no correlation with CMAP or MUNIX of APB, though a weak positive correlation was observed between CSP duration and MUNIX (r = 0.31, *p* < 0.05).

**Figure:**
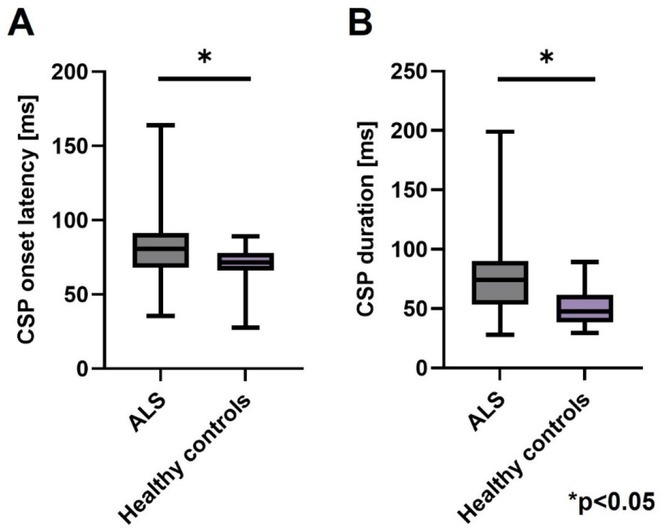
**FIGURE 1** Comparison of CSP onset latency (A) and duration (B) between the ALS group and healthy controls.

**Conclusion:** Prolonged onset latency and duration of CSP in ALS has been previously reported, with some authors attributing this to upper motor neuron involvement. Our study suggests that LMN dysfunction may contribute to CSP alterations in ALS, although further research on CSP in patients with lower MUNIX values is needed,

**Disclosure:** The authors have nothing to disclose.

## EPO‐522

### Clinical, neurophysiological and imaging features of lissencephaly

#### C. Guedes Vaz ^1^; M. Pereira^1^; R. Samões^1^; J. Chaves^1^; T. Temudo^2^; I. Carrilho^2^; S. Figueiroa^2^; J. Freitas^3^; G. Videira^3^; R. Chorão^3^

##### ^1^Serviço de Neurologia, Centro Hospitalar Universitário de Santo António, ULSdSA, Porto, Portugal; ^2^Serviço de Neuropediatria, Centro Hospitalar Universitário de Santo António, ULSdSA, Porto, Portugal; ^3^Serviço de Neurofisiologia, Centro Hospitalar Universitário de Santo António, ULSdSA, Porto, Portugal

**Background and Aims:** Lissencephalies are a rare and heterogenous group of cortical malformations, defined by different MRI and EEG patterns. We aim to characterize a cohort of lissencephaly patients of a tertiary center and explore potential associations between MRI findings, EEG patterns, and clinical presentation.

**Methods:** Retrospective analysis of clinical, imagiological and electroencephalographic data of a consecutive sample of lissencephaly patients followed at a tertiary center.

**Results:** Nineteen patients were included, 63.2% (*n* = 12) female, with a median age at diagnosis of 11 months (IQR 3 years). Genetic mutations were confirmed in 68.4% (*n* = 13), most commonly in the DCX gene (38.5%). The average time between imaging and genetic diagnosis was 6.3 ± 5.1 years. Seventeen patients developed epilepsy (89.5%) at a median age of 2 years (IQR 6.2 years), with tonic seizures being most frequent (*n* = 11, 64.7%). The most common EEG pattern (*n* = 6; 31.6%) was characterized by diffuse high amplitude alpha/beta activity. Patients with this pattern or with normal EEG had a better functional status (*p* < 0.001) and more commonly had non‐refractory epilepsy (*p* = 0.03). Focal or multifocal epileptiform activity was observed in 11 patients (57.9%). The anterior gradient was the most frequent neuroimaging pattern (*n* = 9, 47.4%) and was not associated with epilepsy severity or functional status.

**Conclusion:** Nearly all lissencephaly patients developed early‐onset epilepsy. Despite the typically symmetrical cortical abnormalities, focal or multifocal epileptiform activity was common. In our cohort, the EEG pattern was associated with epilepsy severity and functional status. The neuroimaging pattern was independent of clinical or electroencephalographic aspects.

**Disclosure:** Nothing to disclose.

## EPO‐523

### Trigeminal reflex abnormalities distinguish between classical and idiopathic trigeminal neuralgia

#### G. De Stefano ^1^; C. Mollica^2^; C. Leone^1^; E. Galosi^1^; G. Di Pietro^1^; P. Falco^1^; N. Esposito^1^; D. Litewczuk^1^; E. Evangelisti^1^; F. Caramia^1^; A. Truini^1^; G. Di Stefano^1^

##### ^1^Department of Human Neuroscience, Sapienza University of Rome, Rome, Italy; ^2^Department of Statistical Sciences, Sapienza University of Rome, Rome, Italy

**Background and Aims:** Primary trigeminal neuralgia (TN) is a representative neuropathic facial pain condition classified into classical (associated with neurovascular compression), and idiopathic (unknown etiology). Differentiating between classical and idiopathic TN based on clinical and neurophysiological findings remains challenging. In this clinical and neurophysiological study, we aimed to identify predictive clinical and neurophysiological variables that may distinguish between the two types of TN.

**Methods:** We retrospectively analyzed clinical records and neurophysiological data from 114 patients with primary TN (84 classical TN, 30 idiopathic TN). We implemented a logistic regression model to identify predictive variables for classical and idiopathic TN.

**Results:** Thelogistic regressionmodel showed that a trigeminal reflex latency asymmetry longer than 0.5ms between the affected and unaffected sides was predictive of classical TN (*p* < 0.05). Additionally, combined involvement of the second and third trigeminal divisions was predictive of idiopathic TN (*p* < 0.05).

**Figure:**
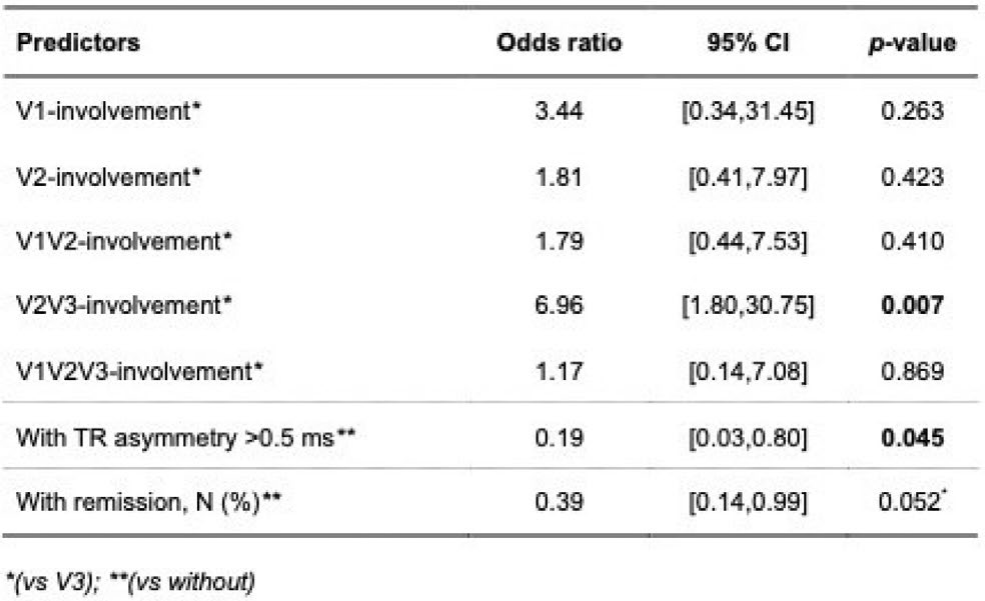
**TABLE 1** Significant predictors of idiopathic TN according to the logistic regression analysis.

**Figure:**
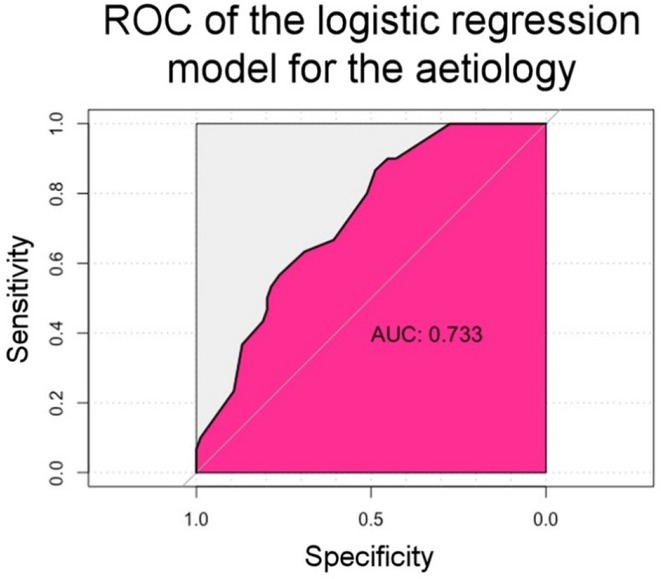
**FIGURE 1** ROC of the logistic regression model for the aetiology

**Conclusion:** Our findings suggesting that latency asymmetry in trigeminal reflexes differentiate between classical and idiopathic TN probably reflects the association of classical TN with neurovascular compression, while idiopathic TN may involve other factors affecting trigeminal nerve fibers.

**Disclosure:** Nothing to disclose.

## EPO‐524

### Neurophysiological biomarkers in adults with type III spinal muscular atrophy

#### G. Libelli ^1^; A. Nuredini^1^; A. D'Orsi^1^; E. Siena^2^; S. Romano^1^; P. Anceschi^1^; I. Allegri^3^; E. Chierici^3^; E. Saccani^3^

##### ^1^Unit of Neurology, Department of Medicine and Surgery, University of Parma, Parma, Italy; ^2^Neurology Unit, Department of General and Specialized Medicine, Hospital of Vaio, Fidenza, Italy; ^3^Neurology Unit, Department of General and Specialized Medicine, University Hospital of Parma, Parma, Italy

**Background and Aims:** This study aimed to evaluate the feasibility and tolerability of MUSIX (Motor Unit Size Index) and MUNIX (Motor Unit Number Index) as potential biomarkers of disease progression in patients with spinal muscular atrophy (SMA) and to identify the most reliable target muscle. It also sought to establish correlations between neurophysiological outcomes and changes in motor scores (RULM and HFMSE).

**Methods:** Four patients with SMA type III (mean age 35.5 ± 15 years) were enrolled. MUNIX and MUSIX were assessed in various limb muscles, and the results were compared to those from healthy controls. A potential correlation between neurophysiological measurements and functional motor scales was subsequently analyzed.

**Figure:**
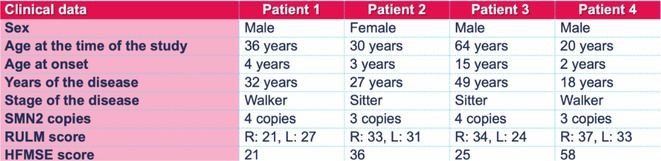
**TABLE 1** Clinical data.

**Results:** MUSIX values were higher in SMA patients compared to healthy controls, suggesting greater neurogenic damage and active reinnervation, while the compound muscle action potential (cMAP) amplitude and MUNIX values were lower. The cMAP amplitude of less affected muscles approached the normal range, whereas MUNIX and MUSIX values did not, highlighting their greater sensitivity as neurophysiological parameters. A significant inverse correlation was observed between MUSIX values in the abductor pollicis brevis (APB) muscle and the ipsilateral RULM score (*p* < 0.05). Furthermore, MUSIX demonstrated an inverse correlation with muscle strength and the degree of disability, while MUNIX revealed a direct correlation.

**Figure:**
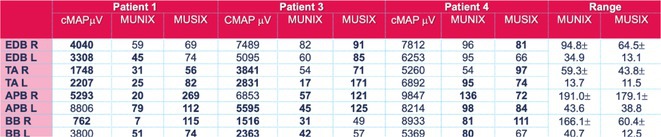
**TABLE 2** Neurophysiological parameters with pathological values in bold

**Conclusion:** In conclusion, this study utilized an innovative neurophysiological approach to assess a cohort of adult SMA patients. The findings suggest that these techniques, particularly MUSIX, hold promise as outcome measures in this population.

**Disclosure:** Nothing to disclosure.

## EPO‐525

### The long‐term effects of repetitive magnetic stimulation in stroke hand rehabilitation depend on hemispheric dominance

#### J. Veldema

##### Department of Sport Science, Faculty of Psychology and Sports Science, Bielefeld University, Bielefeld, Germany

**Background and Aims:** This randomised, double‐blind study investigated the long‐term effects of 1Hz rTMS over the contralesional M1 in stroke patients with a moderate hand motor disability.

**Methods:** 40 stroke patients received 1 Hz rTMS/sham rTMS followed by motor training of the affected hand over three weeks. The hand motor function (Wolf Motor Function Test, Motor Evaluation Scale for Upper Extremity in Stroke Patients, Finger Tapping) and neural networks (Motor Evoked Potentials) were tested (i) immediately prior, (ii) immediately after, and (iii) six months after the intervention.

**Results:** Significant intervention‐induced reduction of corticospinal excitability was detected after 1 Hz rTMS (in comparison to sham rTMS), independent of lesion location. The motor function of the affected hand improved after verum rTMS only in patients with lesioned dominant hemisphere (*n* = 17). Patients with the lesion within the non‐dominant hemisphere (*n* = 23) did not benefit from this intervention.

**Conclusion:** The impact of hemispheric differences on rehabilitation‐induced effects in stroke subjects was only insufficiently investigated up to now. Future studies should devote more attention to this relevant topic.

**Disclosure:** Nothing to disclose.

## EPO‐526

### Not everything that is rhythmic is a seizure: EEG characteristics during cognitive fluctuations of LBD

#### L. Taruffi ^1^; G. Giovannini^2^; N. Orlandi^1^; M. Pugnaghi^2^; M. Burani^1^; N. Biagioli^1^; S. Scolastico^1^; L. Madrassi^1^; A. Ballerini^1^; A. Vaudano^1^; S. Meletti^1^

##### ^1^Department of Biomedical, Metabolic and Neural Sciences, University of Modena and Reggio Emilia, Modena, Italy; ^2^Neurology Unit, OCB Hospital, AOU Modena, Modena, Italy

**Background and Aims:** Lewy Body Dementia (LBD) is associated with EEG slowing, but its characteristics and relationship with the ictal‐interictal continuum during cognitive fluctuations (CF) remain underexplored.

**Methods:** A comprehensive literature review was conducted on PubMed with the MeSH terms “Dementia with Lewy Body” and “Electroencephalogram.” From 184 articles, studies addressing CF and EEG findings were selected. Additionally, two new case reports from our institution initially admitted for suspected NCSE/seizures were included.

**Results:** Nine studies involving 170 LBD patients consistently reported EEG background slowing during CF, with one case NCSE pattern. Three studies described cognitive impairment in LBD later diagnosed as seizures, successfully treated with anti‐seizure medications (ASMs). Four studies identified rhythmic patterns outside CF, including Frontal Intermittent Rhythmic Delta Activity (FIRDA) in 18 patients and Generalized Rhythmic Delta Activity (GRDA) in 14 patients. One study reported an increased prevalence of interictal epileptiform discharges in LBD. Personal cases: i) a 76‐year‐old woman presented with long‐lasting episodes of altered mental status. Initially, NCSE was diagnosed and ASMs started. Long‐term video‐EEG monitoring of the episodes fulfilled EEG criteria for ictal‐interictal continuum / possible NCSE according to ACNS terminology; ii) an 82‐year‐old woman with episodes of confusion underwent prolonged EEG monitoring showing bilateral frontotemporal slow‐wave activity. Neither case showed a clinical or EEG response to ASMs. Both patients were later diagnosed with LBD.

**Conclusion:** EEG background rhythms during CF in LBD commonly show rhythmic theta/delta patterns. However, long‐term video‐EEG studies remain scarce. Our cases show how EEG findings alone can be misleading, prompting misdiagnosis of NCSE.

**Disclosure:** Nothing to disclose.

## EPO‐527

### High‐density surface electromyograms reveal the diffusion of the botulinum neurotoxin effect

#### M. Gagliardi ^1^; L. Malimpensa^2^; M. Fröhlich^1^; C. Esposito^2^; A. Conte^2^; G. Fabbrini^2^; G. Leodori^2^; D. Belvisi^2^; G. Ferrazzano^2^; T. Vieira^1^

##### ^1^Laboratory for Engineering of the Neuromuscular System (LISiN), Department of Electronics and Telecommunication, Politecnico di Torino, Turin, Italy; ^2^Department of Human Neurosciences, Sapienza University, Rome, Italy

**Background and Aims:** The successful administration of Botulinum Toxin depends on the diffusion of its silencing effect rather than on its dispersion within the targeted tissue. Quantifying the distribution of muscle fibres responding to BT is therefore imperative if the muscle region under BT effect is to be assessed. Here we investigate the potential of assessing the diffusion of the BT effect based on surface electromyograms detected from multiple skin location: the HD‐sEMG.

**Methods:** Grids with 64 electrodes were used to sample HD‐sEMG, bilaterally from gastrocnemius medialis muscles. M wave were elicited through supramaximal stimulation of the tibial posterior nerve, before (T0), at four (T1), and at 12 weeks post‐injection. Our reasoning is that action potentials would not be triggered by nerve stimulation only in the muscle fibres under the BT effect. We hypothesise the amplitude of M waves detected on skin locations covering the silenced fibres would be sensitive to the BT. Contrarily, no change would be expected for the contralateral muscle.

**Results:** Figure 1 shows M waves at the three evaluation points for a single subject. A notable T0‐T1 reduction in M wave amplitude was observed for the spastic though not for the control muscle. This difference was evident only for 12‐grouped electrodes (Figure 2).

**Figure:**
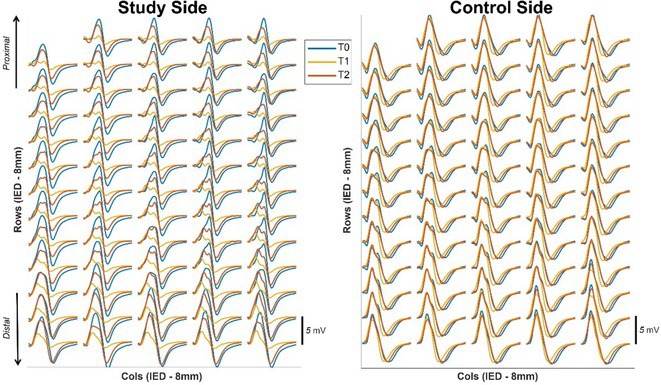
**FIGURE 1** This figure shows the raw M‐waves recorded with two 64‐electrode grids at T0, T1, and T2. The left panel (study side) shows reduced amplitudes at T1 (BT peak effect) with partial recovery at T2. The right panel (control side) shows consistent amplitudes.

**Figure:**
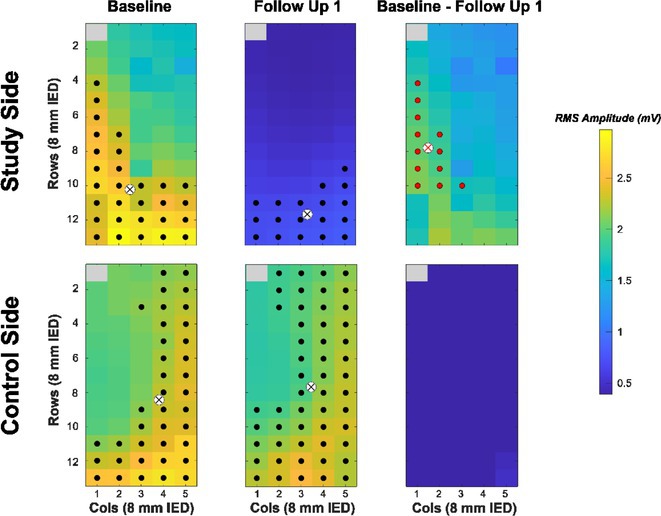
**FIGURE 2** This figure shows RMS values as colormaps and segmented channels (>70% max amplitude) at T0, T1, and their difference. On the study side, 12 channels active at T0 disappear at T1, indicating BT's effect. Control side colormaps remain consistent.

**Conclusion:** HD‐sEMG proved sensitive to the diffusion of the BT effect. Presumably, the electrodes providing M waves with reduced amplitude after injection reflect the location of the silenced fibres: we are collecting follow‐up data from other subjects to further this possibility.

**Disclosure:** This study was carried out within the «BREAKing BONDS» project – funded by the Ministero dell’Università e della Ricerca – within the PRIN 2022 program (D.D. 104 – 02/02/2022). This manuscript reflects only the authors’ views and opinions and the Ministry cannot be considered responsible for them.

## EPO‐528

### Posterior interosseous neuropathy secondary to parosteal lipoma: A case report and literature review

#### M. Ryan ^1^; E. Dolan^1^; G. O'Connor^1^; B. McNamara^2^

##### ^1^Department of Neurology, Cork University Hospital, Cork, Ireland; ^2^Department of Neurophysiology, Cork University Hospital, Cork, Ireland

**Background and Aims:** Parosteal lipomas are a relatively rare cause of compressive posterior interosseous neuropathy. The objective of this case report is to raise awareness of this highly treatable entity and underline the importance of neurophysiological assessment in localising the site of injury.

**Methods:** Case report and review of literature.

**Results:** A 58‐year‐old right‐handed woman presented with a six‐week history of progressive right arm pain, wrist drop and clawing of right hand. Examination revealed moderate weakness of right wrist extension and right finger extension and flexion with normal reflexes and sensory examination. Nerve conduction studies demonstrated absent right radial motor response and intact right superficial radial sensory response. Needle electromyography showed severe subacute partial denervation in right extensor digitorium communis and right extensor indicus, with occasional neurogenic units in her right triceps. MRI cervical spine showed severe narrowing of the exit neural foramina bilaterally at C6‐C7 level, with left nerve root abutment. MRI right forearm revealed a non‐enhancing 5.2 x 6.5cm parosteal lipoma wrapping around the ulnar aspect of the proximal radius. She underwent surgical excision of the lipoma with resultant improvement in right upper limb function.

**Conclusion:** Posterior interosseous neuropathies due to lipoma are rarely encountered in clinical practice but important to recognise as they are eminently treatable with early surgical intervention. This case highlights the clinical utility of neurophysiological studies in temporally differentiating between a more indolent chronic right C7 radiculopathy and a symptomatic subacute compressive right posterior interosseous neuropathy.

**Disclosure:** Nothing to disclose.

## EPO‐529

### Multimodal transoperative neurophysiological monitoring in carotid surgery. Ten years of experience

#### R. Garcia Santos ^1^; M. Collado Corona^2^; S. Cohen Mussali^3^; C. Ramirez Cerda^3^; J. Valdes Flores^3^

##### ^1^Department of Neurophysiology, ABC Medical Center. Mexico City, Mexico; ^2^National Institute of Neurology and Neurosurgery “Manuel Velasco Suarez” Mexico City, Mexico; ^3^Department of Vascular Sugery, ABC Medical Center. Mexico City, Mexico

**Background and Aims:** Carotid surgery (CS) is a surgical procedure to treat carotid stenosis (CAS) and paraganglioma (PG). There is a high risk of stroke during carotid artery cross occlusion and secondary embolism when performing selective bypass (BS) as well as cranial nerve (CN) injury. The use of multimodal intraoperative neurophysiological monitoring (MNFIOM) using somatosensory evoked potentials and electroencephalography can be used to monitor cerebral perfusion during CC, is useful in determining the need and timing of BS. Locating and mapping NC VII (3 branches), IX and X and monitoring their function during surgery minimizes postoperative deficit.

**Methods:** Observational, retrospective, cross‐sectional study of consecutive patients undergoing CS at ABC Medical Center from 2012 to 2023.

**Figure:**
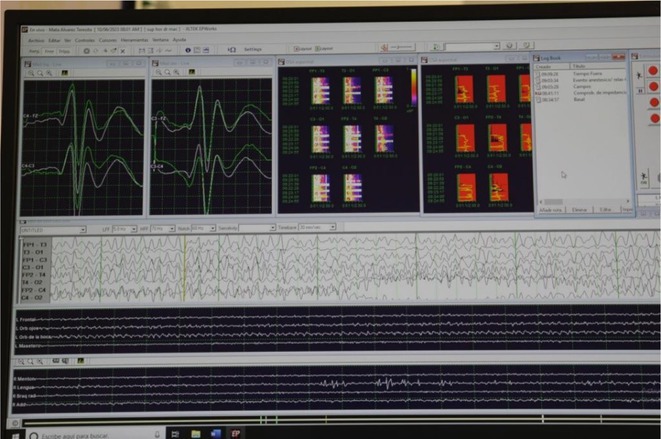
**FIGURE 1** Top Left: PESS Top Right Center: EEG Frequency Scale Center: Short EEG Bottom: Free Cranial Nerve Needle EMG

**Results:** Eighty‐eight patients were included, 51 (58%) male, median age 62.7 years. CC was performed in 43.18% (38) for CG and 56.82% (50) for symptomatic CAS (71) 80.68%, and 16 severe asymptomatic (18.18%). Fifty‐six left carotid arteries (63.64%) were operated on using MNFIOM, with an average operative time of 82 minutes and an average clamping time of 21 minutes. Only one case presented with stroke, which was thrombolyzed with total recovery at 6 months. In addition, BS was performed in 10 cases by neurophysiological indication, the complications were transient facial paralysis (7) and swallowing problems in (7) patients.

**Figure:**
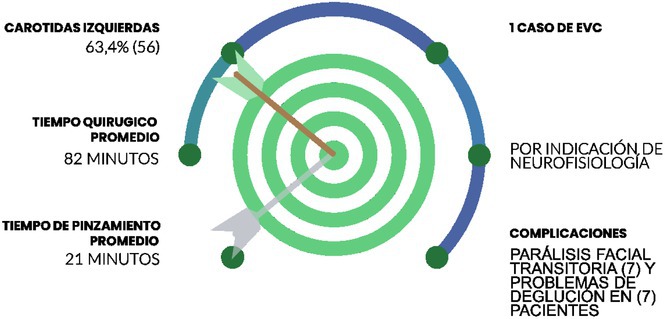
**FIGURE 2** Left Carotids 63.4% (56) Average Surgery Time 82 minutes Average Clamping Time 21 minutes 1 Stroke Intraoperative Mechanical Thrombectomy 10 Carotid Bypasses Indicated by Neurophysiology Complications Transient Facial Paralysis (7) Swallowing Proble

**Figure:**
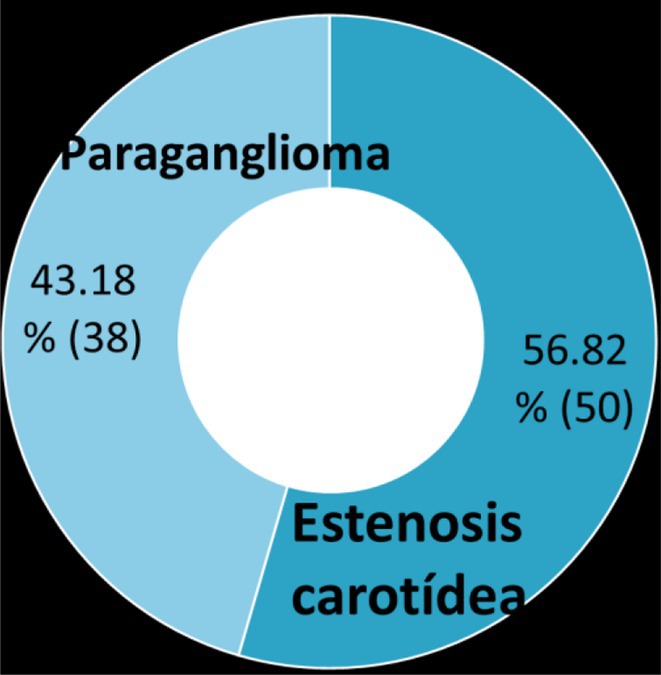
**FIGURE 3** Here's the updated translation with the appropriate labels: Etiologies (Left Pie Chart): Paraganglioma: 43.18% (38) Carotid Stenosis: 56.82% (50) Symptoms (Right Pie Chart): Asymptomatic: 18.18% (16) Symptomatic: 80.68% (71)

**Conclusion:** This study stressed that the routine use of MNFIOM is sensitive and specific to prognosticate and minimize any postoperative neurological deficits. It helps to monitor cerebral perfusion during surgery, predict the need for BS after cross‐occlusion and preserve NCs.

**Disclosure:** Nothing to disclose.

## EPO‐530

### Abstract withdrawn

## EPO‐531

### Analysis of intraoperative neurophysiological monitoring in neuromuscular and idiopathic scoliosis surgery

#### Y. Park

##### Gangnam Severance Hospital, Seoul, Republic of Korea

**Background and Aims:** There are few comparative studies of intraoperative neurophysiological monitoring (IONM) between neuromuscular scoliosis (NS) and idiopathic scoliosis (IS). We made comparative analysis, especially in patients with no postoperative motor deterioration.

**Methods:** We reviewed 53 IONM records of scoliosis operation (NS: 25, IS: 28). The maximum percentage of amplitude decrement of MEPs (ΔMEPampMax) and SEPs (ΔSEPampMax), and the maximum percentage of prolonged SEPs latency (ΔSEPlatMax) compared to baseline value were analyzed. The preoperative motor score (Motorpre) of both lower extremities by the International Standards for Neurological Classification of Spinal Cord Injury were calculated using the MRC grade. Cobb's angle (Cobb'spre), corrected Cobb's angle (ΔCobb's) were measured. The maximum and minimum systolic BP (SBP) and diastolic BP (DBP) during surgery.

**Results:** NS showed significantly lower height, weight, and Motorpre, compared to IS. NS had larger Cobb'spre, longer fixation level and operation duration, and more bleeding amount than IS (Table 1). However, there were no significant differences of ΔMEPampMax, ΔSEPampMax, or ΔSEPlatMax between two groups. By Pearson's correlation analysis, ΔSEPampMax were significantly correlated with operation duration (*p* = 0.01) in NS. In addition, ΔSEPampMax were correlated with DBPMin (*p* = 0.04) and MEPampMax (*p* = 0.01) in IS. The ΔSEPlatMax was correlated with SBPMax (*p* < 0.01) and ΔSEPampMax (*p* < 0.01) in NS. On the linear regression analysis, bleeding amount, SBPMax, and DBPMax were significant contributing factors for ΔSEPlatMax in NS (Table 2).

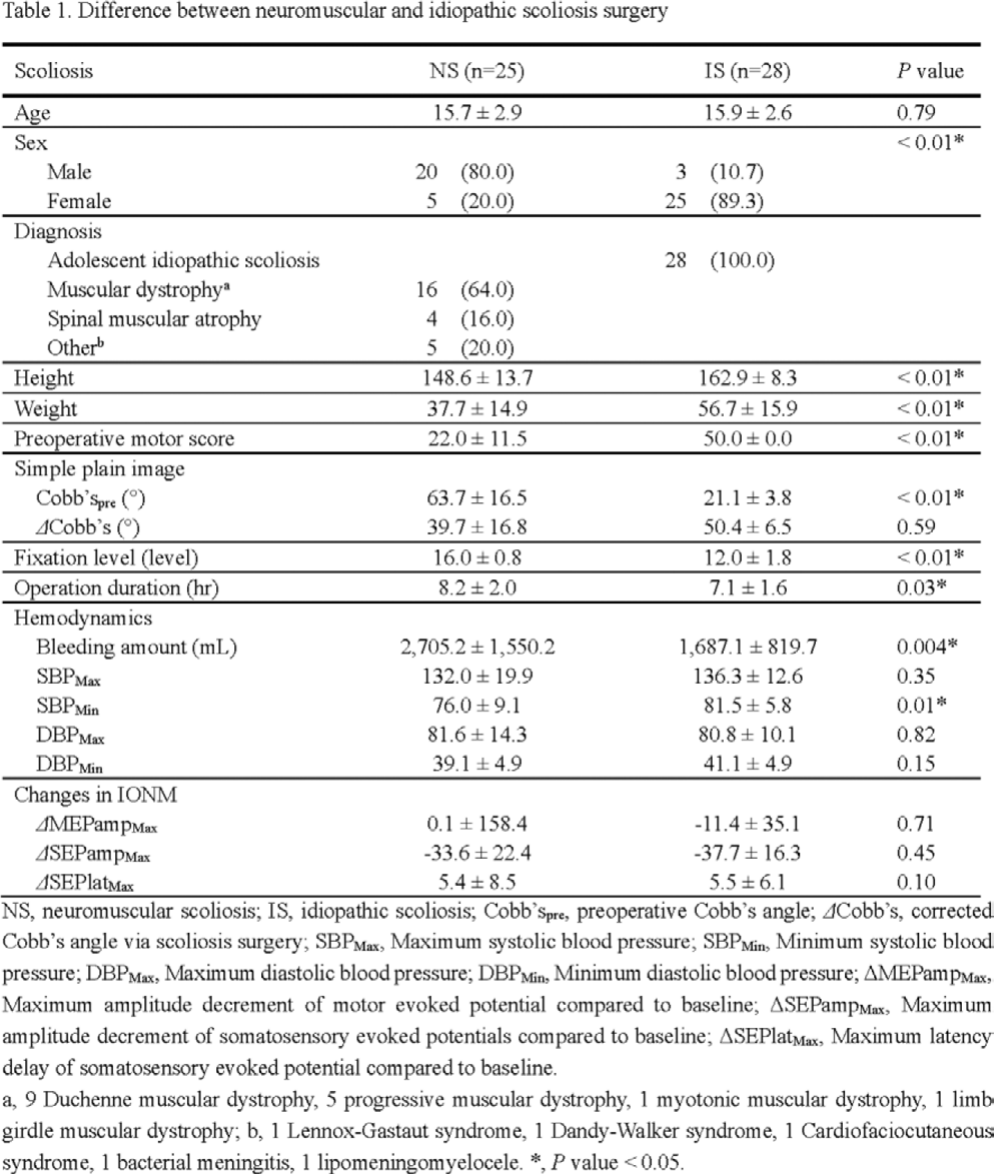

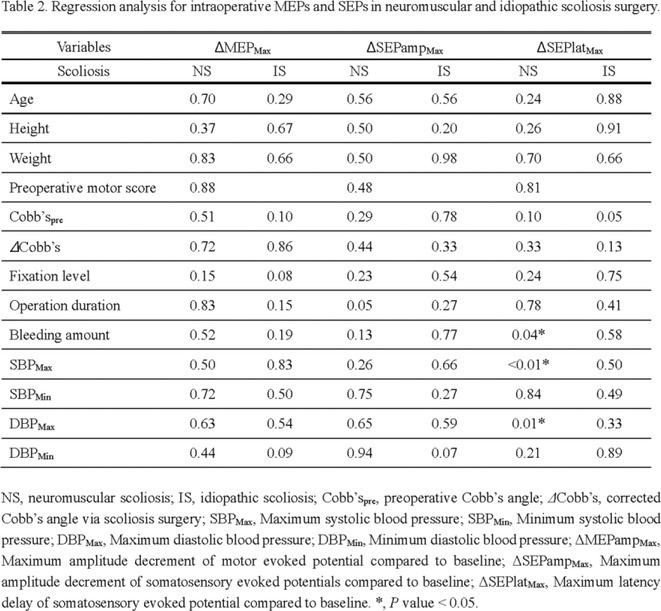

**Conclusion:** These results represent that the bleeding amount and the following hemodynamics are important factors to make the SEP latency prolonged in NS.

**Disclosure:** Nothing to disclose.

## Coma, trauma, and neurocritical care

## EPO‐532

### Feasibility study of predicting functional outcome after cardiac arrest, evaluating the effects of trial‐prognostication

#### A. Lagebrant

##### Department of Clinical Sciences Lund, Lund University, Lund, Sweden

**Background and Aims:** This study aims to evaluate the safety of predicting functional outcome after cardiac arrest by evaluating the effects of trial‐prognostication in the yet largest randomized cardiac arrest cohort.

**Methods:** Retrospective analysis of the prospective Targeted Hypothermia versus Targeted Normothermia after Out‐of‐Hospital Cardiac Arrest (TTM2)‐trial. Patients with available functional outcome at six months were included. The prognostic accuracy of conservative TTM2‐trial criteria and the effects of hypothermia were assessed.

**Results:** We included 1829/1861 (98.3%) patients (Fig. 1). Trial prognostication according to conservative TTM2‐trial criteria was performed in 881 patients at median 110 (IQR: 98–133) hours with sedation weaned off for median 13 (IQR: 1–48) hours (Table 1). Withdrawal of life‐sustaining therapy (WLST) was performed in 324/881 (36%) and 345/948 (36%) of the trial‐prognosticated and non‐prognosticated patients at median 141 (IQR: 109–195) and 53 (IQR: 21–86) hours, respectively. Neurological cause to WLST was more common and performed at later timepoints in the trial‐prognosticated patients than in the non‐prognosticated cohort, 270/324 (83%) at median 133 (IQR: 105–172) hours vs 78/345 (23%) at 90 (IQR: 74–112) hours, respectively (*p* < 0.001). Poor functional outcome was found in 476/811 (54%) and 509/948 (54%) in trial‐prognosticated and non‐trial prognosticated patients, respectively. TTM2‐trial prognostication identified 47% (95% CI: 42–52%) of poor outcome patients (Table 1). Patients randomized to hypothermia woke up significantly later than normothermic patients, at median 72 (IQR; 47–119) hours vs 61 (IQR: 45–102) hours, respectively (*p* = 0.00186) (Fig. 1).

**Figure:**
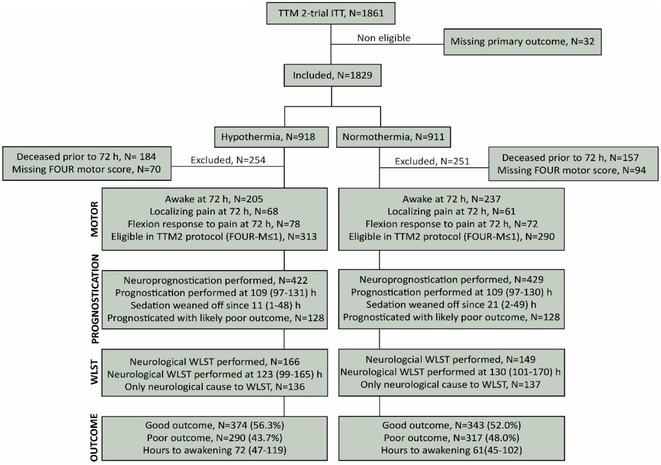
FIGURE 1

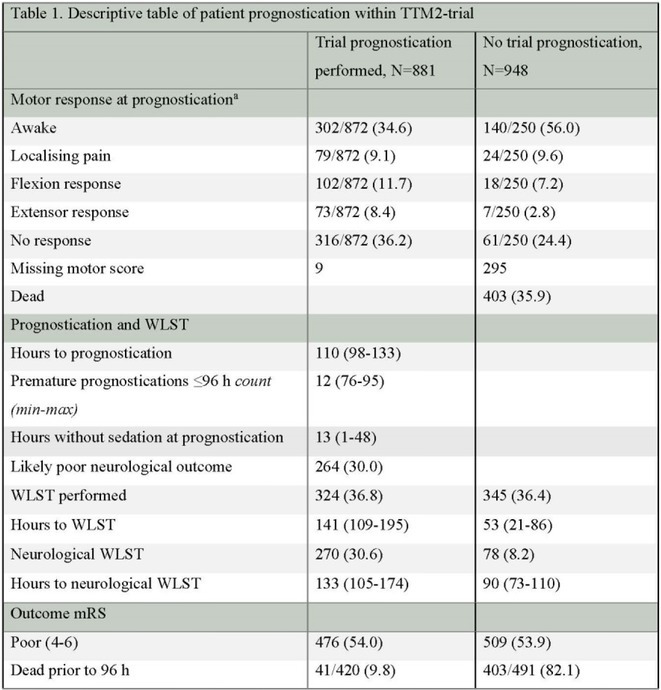

**Conclusion:** Trial prognostication is associated with higher rate of neurological WLST and delayed WLST compared to non‐prognosticated patients. Hypothermia is associated with longer time to awakening.

**Disclosure:** Nothing to disclose.

## EPO‐533

### A consensus conference on care pathways for individuals with post‐anoxic disorder of consciousness (CaPIADoC)

#### A. Estraneo ^1^; A. Magliacano^1^; F. De Bellis^1^; A. Amantini^1^; S. Lavezzi^2^; A. Grippo^3^; O. CapiaDoC study group^4^

##### ^1^IRCCS Fondazione Don Carlo Gnocchi ONLUS, Florence, Italy; ^2^Unit of Severe Brain Injury Rehabilitation, Department of Neuroscience, S. Anna University Hospital, Ferrara, Italy; ^3^Neurophysiology Unit, Careggi University Hospital, Florence, Italy; ^4^The CapiaDoC study group encompasses 22 professionals from diverse disciplines and representing 9 scientific societies along with 2 associations of patients’ families

**Background and Aims:** Accurately evaluating the level of consciousness and promptly identifying neurological complications in intensive care settings are pivotal for proper prognostication and personalized treatment in patients with post‐anoxic disorders of consciousness (DoC). This Consensus Conference, organized across multiple Italian scientific societies, aimed to tackle ongoing debates surrounding diagnostic and prognostic strategies.

**Methods:** Twelve working groups, comprising 22 professionals from diverse disciplines and representing 9 scientific societies along with 2 associations of patients’ families, performed a systematic review of the literature to address 12 key questions ‐ 5 focusing on diagnosis and 7 on prognosis. The included studies were assessed for quality of evidence using the Oxford Centre for Evidence‐Based Medicine Levels of Evidence. Based on these findings and expert opinion, a jury comprising members from scientific societies and family associations developed recommendations.

**Results:** Out of 1,219 articles initially identified, 21 were included in the analysis. Each working group provided insights into the strengths and weaknesses of the evidence addressing their specific question. A key recommendation was to adopt a multimodal approach, integrating validated clinical tools, neurophysiological tests, and neuroimaging techniques for diagnostic and prognostic evaluations, enabling tailored treatment plans. Additionally, the use of standardized terminology and diagnostic criteria was strongly encouraged to ensure uniformity and appropriateness in managing patients.

**Conclusion:** This multidisciplinary Consensus Conference has established the first set of operational recommendations to guide clinical practice for patients with post‐anoxic DoC. Periodic updates will be required to incorporate new evidence and enhance the current guidelines over time.

**Disclosure:** No disclosures.

## EPO‐534

### Impact of fever prevention on acute cerebrovascular injury outcomes: A meta‐analysis of randomized controlled trials

#### L. Violeta Rodrigues de Matos^1^; A. Menegaz de Almeida^2^; C. Grieger^3^; K. Violeta Rodrigues de Matos^4^; C. Maia^1^; M. De Lima^5^; A. Maldonado^5^; D. Texeira^5^; G. Ferreira^5^; A. Lydia Machado Silva ^6^

##### ^1^Federal University of Amazonas, Brazil; ^2^Federal University of Mato Grosso, Brazil; ^3^Nilton Lins University; ^4^Metropolitan University of Manaus, Brazil; ^5^Cesumar University; ^6^University City of São Paulo, Brazil

**Background and Aims:** Fever occurrence in patients with acute vascular brain injury, may contribute to poorer outcomes. However, the effectiveness of the interventions in improving functional recovery and reducing mortality is still unclear.

**Methods:** A systematic search was conducted in the MEDLINE, Cochrane, Web of Science, and Embase databases for Randomized Controlled Trials investigating patients with Acute Vascular Brain Injury and the effects of fever prevention. Data were analyzed using the inverse variance method with 95% confidence intervals (CIs). Heterogeneity was assessed using the I^2^ statistic. Statistical analysis was performed using RStudio, version 4.3.2.

**Results:** We included 9 studies and 9,687 patients. Compared with standard care, fever prevention achieved better rates of brain injury (45%–42%; OR 1.11; 95% CI 1.01–1.22; *p* = 0.029227; *I* ^2^ = 0%). The outcome of fever burden was statically desfavorable to the fever prevention group (MD = −0.63; 95%; CI −1.80 to 0.53; *p* = 0.285593; *I* ^2^ = 96%), while there was a lower incidence of sepsis (RR = 0.68; 95% CI 0.37−1.27; *p* = 0.65; *I* ^2^ = 0%). The outcome of pneumonia was statistically favorable to standard care group (RR = 1.10; 95% CI 0.90−1.34; *p* = 0.361892; *I* ^2^ = 0%). Also resulted of death was not statistically favorable for both groups (15% vs 17% RR = 0.96; 95% CI 0.81−1.12; *p* = 0.592765; *I* ^2^ = 46%).

**Figure:**
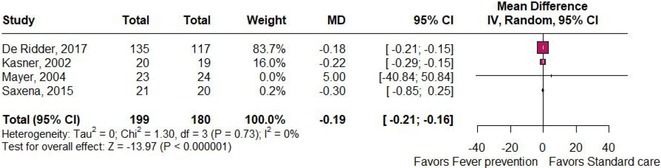
**FIGURE 1** Temperature

**Figure:**
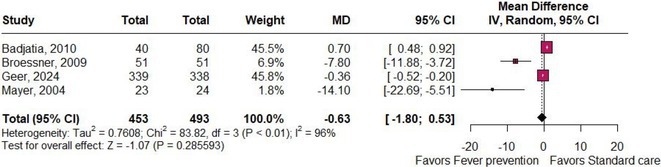
**FIGURE 2** Ferver burden

**Figure:**
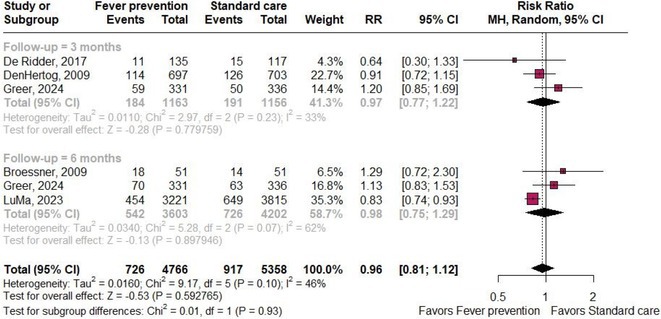
**FIGURE 3** mRS = 6

**Conclusion:** This meta‐analysis demonstrated that the use of antipyretics or temperature control devices for effective fever control resulted in decreased complications like sepsis and pneumonia in patients with acute vascular brain injury.

**Disclosure:** Nothing to disclose.

## EPO‐535

### Spontaneous eyes movement in comatose patients: radiological findings, etiology and outcome

#### A. Frochaux ^1^; J. Lee^2^; A. Rossetti^3^; V. Alvarez^4^

##### ^1^Department of Medicine, eHnv, Yverdon‐les‐Bains and University of Lausanne, Lausanne, Switzerland; ^2^Department of Neurology, Brigham and Women's Hospital, Harvard Medical School, Boston, USA; ^3^Department of Clinical Neurosciences, CHUV and University of Lausanne, Lausanne, Switzerland; ^4^Department of Neurology, Hôpital du Valais, Sion, Switzerland

**Background and Aims:** Spontaneous eye movements (SEM) in comatose patients often signal a poor prognosis; they are usually classified as vertical (bobbing, dipping) and horizontal (ping pong, periodic alternating gaze). The aim is to assess neurological diagnosis, radiological correlations, and the outcome associated with SEM.

**Methods:** Observational study involving two cohorts of adult patients. One comprises patients prospectively collected from a community hospital's ICU (Sion, Switzerland) between 2016 and 2020. The second one is a retrospective cohort from a university hospital (Boston, USA) identified based on a keyword search in patients’ medical files data. Patients with vertical and horizontal eyes movements were compared.

**Results:** We studied 85 patients (21 patients in the prospective and 64 patients in the retrospective cohort) (Table 1). Median age was 56.8 years (range: 18–92). The most frequent diagnoses were cerebral anoxia (41.2%), posterior fossa stroke (22.4%), and traumatic brain injury (7.1%); other conditions accounted for 29.3%. Vertical SEM were more common (82.4%) than horizontal. Multifocal/diffuse and posterior fossa lesions were more frequent in vertical movements, while anoxia was more prevalent in horizontal SEM. Imaging was unremarkable in 20% of cases in both groups. The overall prognosis was poor, with a median mRS of 6 (range 0−6) without difference between the two groups. Four patients only reached a mRS ≤2 at discharge.

**Figure:**
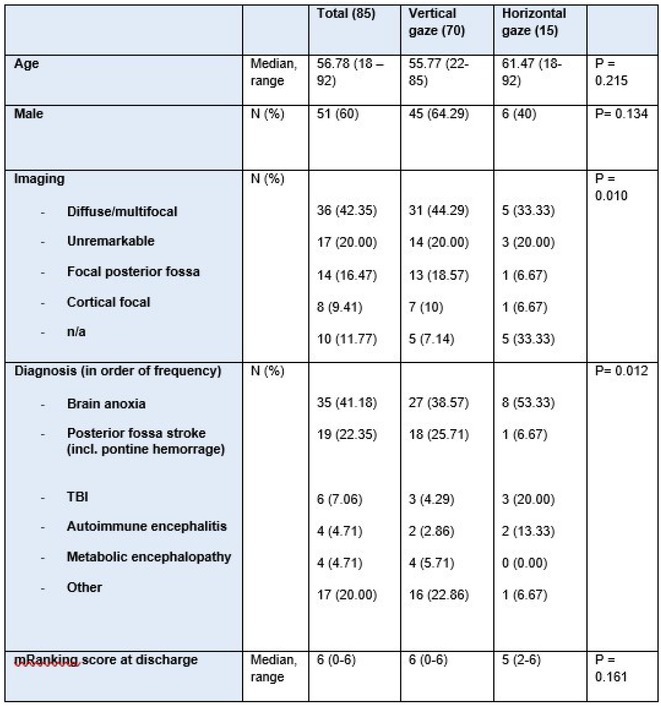
**FIGURE 1** Comparison of comatose patients with vertical and horizontal spontaneous eyes movements. Other diagnostic includes 3 bacterial meningitis, 3 brain tumor; 2 SDH, 2 multifocal embolic strokes, 1 status epilepticus, 1 SAH, and 5 non available n/a.

**Conclusion:** Despite different movement axis and the more frequent correlation of vertical SEM with diffuse and posterior fossa lesions than horizontal SEM, both are strongly associated with extreme poor prognosis.

**Disclosure:** Nothing to disclose.

## EPO‐536

### Performance of four different EWS for predicting adverse outcomes in hospitalized patients with neurological conditions

#### C. Arellano González ^1^; L. Niembro Muñoz^1^; J. Sanchez Zavala^1^; J. Aguilar Gerez^2^

##### ^1^Department of Internal Medicine, Médica Sur Hospital, Mexico City, Mexico; ^2^Department of Internal Medicine, National Institute of Medical Sciences and Nutrition Salvador Zubirán, Mexico City, Mexico

**Background and Aims:** Early warning systems (EWS) are crucial for detecting clinical deterioration. They reduce mortality rates and facilitate transfers to critical areas without requiring emergency procedures. No EWS has been specifically designed for hospitalized patients with neurological conditions. We aimed to evaluate different EWS and create a new EWS for neurological diagnosis.

**Methods:** We conducted a retrospective study. The sample was subdivided into neurological and neurosurgical. The performance of the EWS, was assessed at different times of hospitalization, in predicting the composite outcomes (urgent transfer to a critical area and in‐hospital death). The AUC was calculated, and calibration was analyzed through probability plots. We developed a new predictive score; the sample was randomly divided into derivation and validation datasets. A multivariate logistic regression was applied to evaluate the association between physiological parameters and clinical outcomes. Subsequently we assess the discriminatory performance of the new EWS.

**Results:** The evaluated EWS performed below the levels reported in the literature. mREMS demonstrated the best predictive ability for composite outcomes. All patients, neurological and neurosurgical. AUC: 0.68 (0.602–0.758), 0.754 (0.649–0.86) and 0.646 (0.551–0.74) respectively. The proposed model was superior to all EWS to predict the composite outcome for neurological patients. AUC of new model 0.782 (0.611–0.953).

**Figure:**
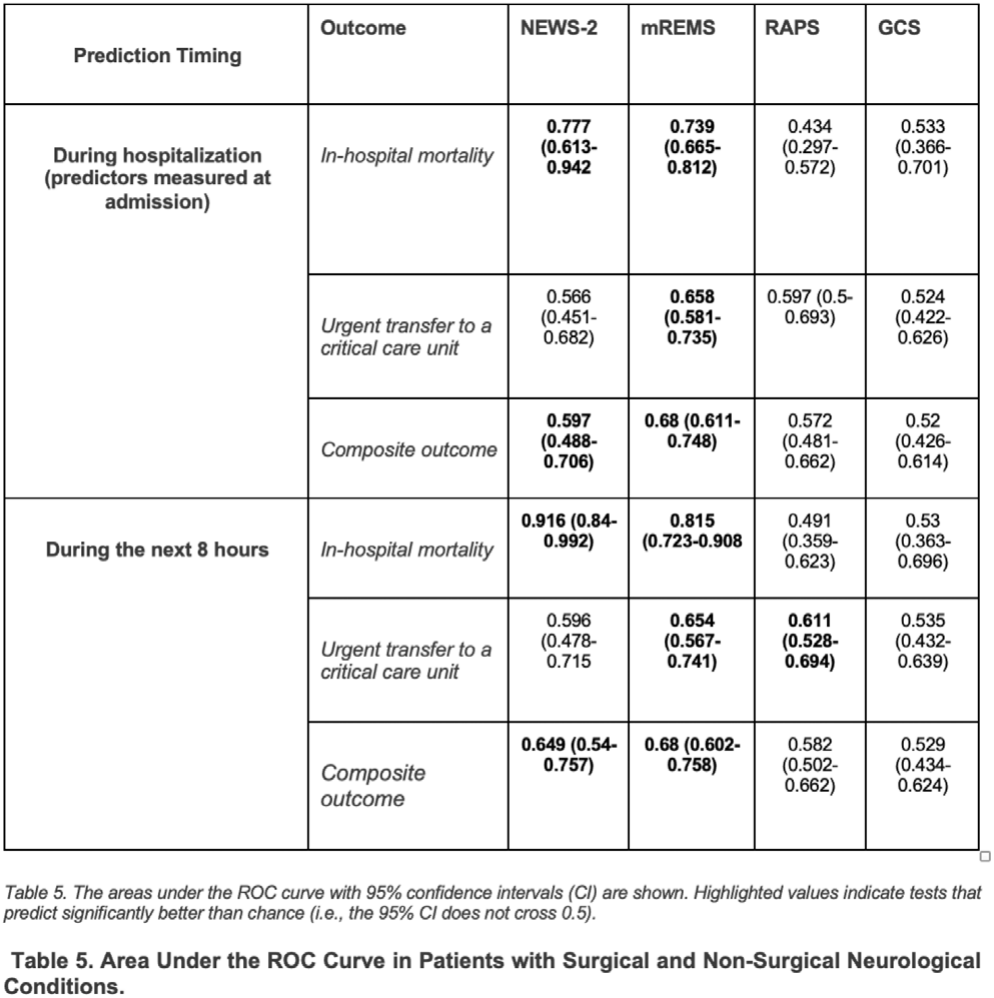
Table. for the 4 EWS studied, with the area under the ROC curve for the composite outcome anda secondary outcomes, for all patients.

**Figure:**
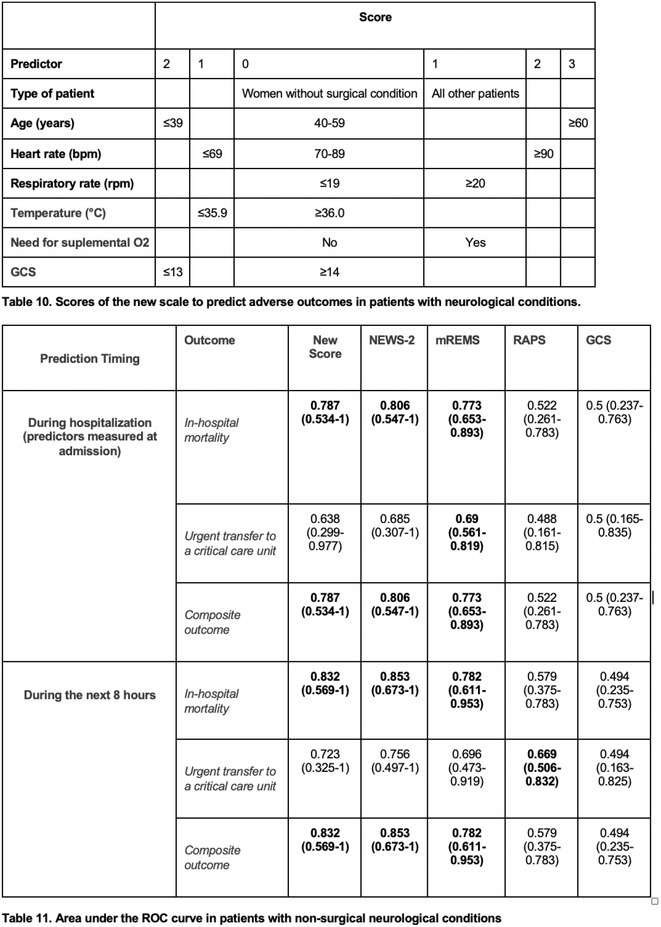
Table with the new proposed EWS score and table with the area under the ROC curve for the composite outcome anda secondary outcome, for neurological patients.

**Figure:**
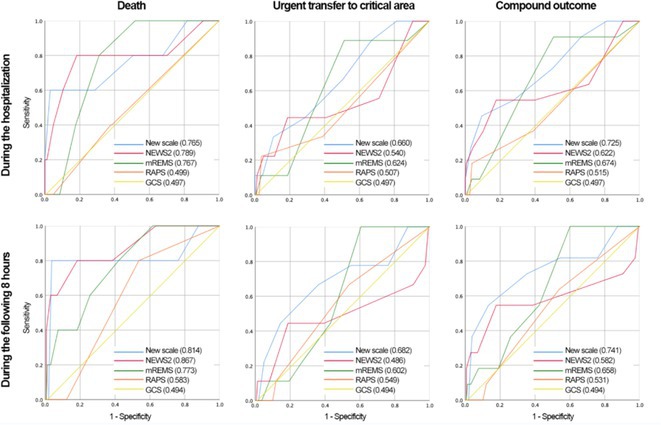
Figure with ROC curve for 4 EWS and new EWS proposed.

**Conclusion:** EWS in neurological diagnosis, can improve, if adjusted to include specific clinical variables. From the EWS use in today's practice; mREMS system is recommended for all neurological conditions due to its superior predictive power. For neurological patients they could benefit more from the new EWS score proposed in this paper.

**Disclosure:** Nothing to disclose.

## EPO‐537

### Neuroendoscopy is better than conventional cranitomy in treating intracranial hemhorrage

#### D. Clemente; M. Misaghi; A. Soares; K. Toledo; N. Santos; A. Paladino

##### Medicine, Universidade da Região de Joinville, Joinville, Brazil

**Background and Aims:** Intracranial Hemorrhage has high mortality and morbidity, and there's still doubts in the medical community about what should be the standard approach. When compared to conventional craniotomy, neuroendoscopy is less invasive and may be a viable alternative.

**Methods:** PubMed, EMBASE, and Cochrane databases were systematically searched for studies that compared craniotomy to neuroendoscopy in intracranial hemorrhage and reported: (1) surgery/anesthesia time; (2) hematoma drainage rate; (3) blood loss rate; (4) mortality rate; (5) complication/infection rate; (6) Modified Rankin Scale. Heterogeneity was examined with I2 statistics. P values of < 0.05 were considered statistically significant.

**Results:** A total of 6 randomized controlled trials (RCTs) and 13 retrospective studies (non‐RCTs) involving 1922 patients were included in the final analysis. Minimum follow up was 1 month post operation. Operation time (SMD −3.32; 95% CI [−4.08] to [−2.55]; *p* < 0.00001), Evacuation rate (SMD 1.18; 95% CI 0.73–1.62; *p* < 0.00001), Blood loss (SMD −3.34; 95% CI [−4.30] to [−2.39]; *p* < 0.00001), Mortality (RR 0.69; 95% CI 0.53–0.90; *p* = 0.006), Infection rate (RR 0.53; 95% CI 0.40–0.71; *p* < 0.0001) were all in favour of the efficacy of neuroendoscopy when compared to craniotomy. Rebleeding (RR 0.62; 95% CI 0.37–1.07; *p* = 0.09) and Modified Rankin Scale (SMD ‐0.44; 95% CI [−1.01] to [0.12]; *p* = 0.13) were not considered statistically significant.

**Conclusion:** These findings suggest that neuroendoscopy has superior efficacy to conventional craniotomy as a drainage procedure in patients with intracranial bleeding.

**Disclosure:** Nothing to disclose.

## EPO‐538

### Clinical features of neurological disorders in Hatay after the February 6 earthquake

#### D. Yavuz Demiray; B. Usta Balıkel; T. Duman

##### Neurology Department, Mustafa Kemal University, Hatay, Turkey

**Background and Aims:** This study aimed to evaluate the clinical features, mood, and sleep quality of patients with Alzheimer's Disease (AD), Epilepsy, Multiple Sclerosis (MS), and Parkinson's Disease (PD) followed in the Neurology Outpatient Clinic at Hatay Mustafa Kemal University Hospital after the February 6 earthquake.

**Methods:** A total of 200 patients (50 from each group) participated. Depression, anxiety, and sleep quality were assessed using the Beck Depression Inventory (BDI), Beck Anxiety Inventory (BAI), and Pittsburgh Sleep Quality Index (PSQI).

**Results:** Among AD patients, 78% experienced clinical deterioration, with 84% showing depression, 90% anxiety, and all having poor sleep quality. A negative correlation was found between MMSE scores and sleep quality (*p* = 0.042). In the epilepsy group, 54% had increased seizure frequency, with higher seizure occurrence among those staying in containers (*p* = 0.041). Depression and anxiety were present in 46%, and 60% had poor sleep quality. In the MS group, 62% reported seizures after the earthquake, and depression, anxiety, and poor sleep quality were found in 52%, 50%, and 70%, respectively. Low education levels were associated with higher depression and anxiety (*p* < 0.05). For PD patients, 64% experienced clinical deterioration, linked to diagnosis duration, Hoehn‐Yahr stage, and container housing (*p* < 0.05). Depression, anxiety, and poor sleep quality were found in 56%, 68%, and 86%, respectively.

**Conclusion:** Clinical deterioration was observed across all groups, strongly associated with increased depression, anxiety, and poor sleep quality. These findings highlight the significant impact of the earthquake on the health and well‐being of patients with neurological conditions.

**Disclosure:** Nothing to disclose.

## EPO‐539

### Sepsis associated encephalopathy‐prevalence and related clinical factors

#### M. Siemiński; K. Krzyżaniak; R. Krion; A. Szymczyk; A. Wsciślak

##### Department of Emergency Medicine, Medical University of Gdansk, Gdansk, Poland

**Background and Aims:** Sepsis‐associated encephalopathy (SAE) is a common complication of sepsis, significantly increasing mortality among patients and potentially leading to long‐term neurological deficits. The study aimed to determine the frequency and clinical factors related to development of SAE in acute phase of sepsis.

**Methods:** Sepsis was diagnosed based on a SOFA scale score ≥ 2. SAE was diagnosed in situations of acute worsening of cognitive functions and consciousness, temporarily related to symptoms of sepsis. Data on patients clinical course, mortality and laboratory tests were retrospectively collected.

**Results:** 443 cases of sepsis were identified with 240 cases of SAE. The most common neurological impairments were communicative disorders (97.6%), confusion (76.8%), and drowsiness (60%). 125 septic patients died, including 109 with SAE. Procalcitonin levels were higher in patients with SAE. Patients developing SAE were more severely burdened by comorbidities.

**Figure:**
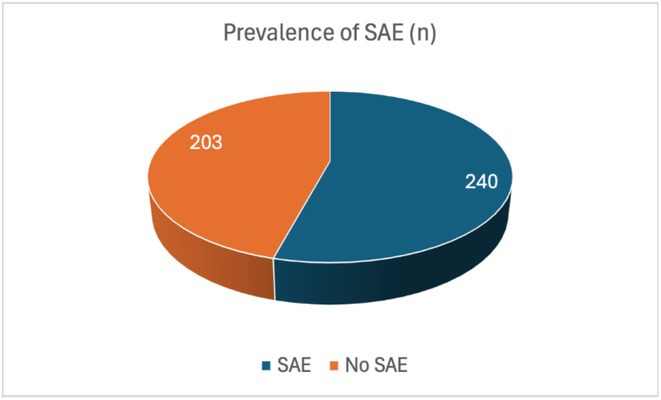
**FIGURE 1** Prevalence of SAE in septic patients

**Figure:**
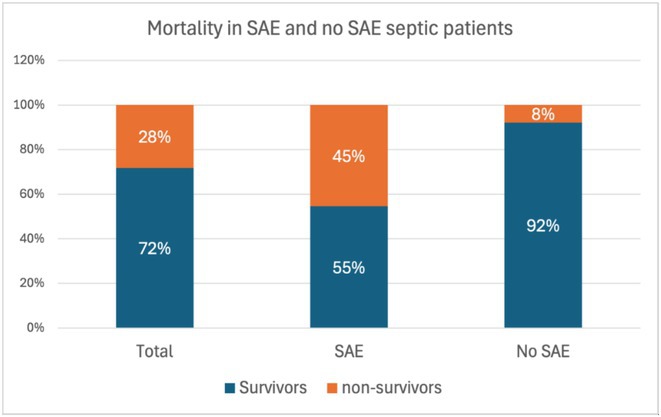
**FIGURE 2** Mortality in SAE and Non‐SAE groups of septic patients

**Conclusion:** SAE is a frequent but still under diagnosed neurological complication of sepsis strongly increasing probability of patient's death. Our data suggest that development of SAE is related to intensity of generalized inflammatory process measured with procalcitonin. Risk of SAE increases in parallel to burdens of comorbidities.

**Disclosure:** Nothing to disclose.

## EPO‐540

### Stimulants for disorders of consciousness in ICU patients: Randomized, placebo‐controlled, cross‐over trial

#### M. Othman ^1^; K. Hansen^1^; M. Nielsen^2^; S. Hyttel‐Sørensen^3^; M. Møller^3^; P. Møller‐Sørensen^4^; J. Hauerberg^5^; S. Sigurdsson^6^; C. Wamberg^7^; T. Itenov^7^; K. Møller^6^; T. Andersen^8^; J. Kjaergaard^9^; D. Kondziella^1^

##### ^1^Department of Neurology, Copenhagen University Hospital ‐ Rigshospitalet, Copenhagen, Denmark; ^2^Department of Forensic Medicine, Faculty of Health and Medical Sciences, University of Copenhagen, Denmark; ^3^Department of Intensive Care, Copenhagen University Hospital – Rigshospitalet, Copenhagen, Denmark; ^4^Department of Cardiothoracic Anaesthesiology, Copenhagen University Hospital – Rigshospitalet, Copenhagen, Denmark; ^5^Department of Neurosurgery, Copenhagen University Hospital – Rigshospitalet Copenhagen, Denmark; ^6^Department of Neuroanaesthesiology, Copenhagen University Hospital – Rigshospitalet, Copenhagen, Denmark; ^7^Department of Anaesthesia and Intensive Care, Copenhagen University Hospital – Bispebjerg and Frederiksberg Hospital, Copenhagen, Denmark; ^8^Department of Applied Mathematics and Computer Science, Cognitive Systems, Danish Technical University (DTU), Lyngby, Denmark; ^9^Department of Cardiology, Copenhagen University Hospital ‐ Rigshospitalet, Copenhagen, Denmark

**Background and Aims:** In the intensive care unit (ICU), there is a critical need for therapeutic strategies that promote consciousness recovery. We evaluated apomorphine and methylphenidate in brain injury patients with acute disorders of consciousness (DoC), hypothesizing that the stimulants would enhance consciousness biomarkers assessed by automated pupillometry (I) and improve clinical signs of consciousness (II).

**Methods:** We randomized 50 ICU patients with DoC (14 women; mean age 63 ± 10 years; 48 with non‐traumatic brain injuries) into strata of three consecutive treatment sessions with apomorphine, methylphenidate or placebo. We administered 112 study medications: 36 apomorphine, 39 methylphenidate and 37 placebo. Automated pupillometry recordings (*n* = 590) from 48 (96%) patients were analyzed.

**Results:** Cognitive load during mental arithmetic was identified in 70 (12%) recordings. Seven (15%) patients fulfilled criteria for cognitive motor dissociation. Apomorphine (OR 1.35, 95% CI: 0.93–1.96) and methylphenidate (OR 1.29, 95% CI: 0.89–1.86) did not significantly increase cognitive load per pupillometry. In total, 10 (20%) patients showed improved clinical arousal at least once: 1 after placebo, 4 after apomorphine (OR 5.04, 95% CI: 0.56–120.7), and 7 after methylphenidate (OR 9.96, 95% CI: 1.36–235.8). Changes toward higher consciousness levels followed 1 placebo, 4 apomorphine (OR 5.67, 95% CI 0.63–169.46), and 3 methylphenidate doses (OR 3.41, 95% CI 0.34–88.00).

**Conclusion:** While pupillometry revealed no significant effects on covert cognition, stimulants may have triggered clinical arousal in some patients. Replication is needed. Our results should guide future pharmacological trials aimed at improving arousal and consciousness recovery. DoC patients may have cerebral reserves that can be activated in the ICU.

**Disclosure:** Nothing to disclose.

## EPO‐542

### Decompressive craniectomy versus conservative management for spontaneous intracranial hemorrhage: A meta‐analysis

#### R. Reis de Oliveira^1^; Y. Picanço Silva ^2^; I. Simon Petry^3^; M. Lee Han^4^; M. de Bastos Maximiano^5^; U. Ansari^6^; R. Leal Dias da Silva^7^; J. dos Santos Monteiro^8^; L. Neves Cordeiro Cavalcanti^9^

##### ^1^Federal University of Pará, Brazil; ^2^Healthcare Institution of South Iceland, Selfoss, Iceland; ^3^University of Southern Santa Catarina, Brazil; ^4^Faculty of Medicine of the University of São Paulo, São Paulo, Brazil; ^5^Federal Fluminense University, Niterói, Rio de Janeiro, Brazil; ^6^Temple University, Philadelphia, Pennsylvania, USA; ^7^Federal University of Rio de Janeiro, Rio de Janeiro, Brazil; ^8^University of Pernambuco, Pernambuco, Brazil; ^9^Federal University of Pará, Pará, Brazil

**Background and Aims:** Spontaneous intracerebral hemorrhage (sICH) is a significant contributor to stroke‐related morbidity and mortality worldwide. Despite recent advancements in pharmacological and surgical management, outcomes remain challenging. Recent studies suggest that decompressive craniectomy (DC) may offer benefits over best medical treatment (BMT) in certain sICH cases. This study aims to compare DC versus BMT with respect to neurological function, morbidity, and mortality in patients with sICH.

**Methods:** PubMed, EMBASE, Cochrane and Web of Science databases were searched for randomized and observational studies comparing DC with conservative management alone for treatment of patients with sICH. The outcomes analyzed were modified Rankin Scale (mRS), mortality at 30 days, overall mortality and length of hospital stay. Odds ratio (OR) and mean difference (MD) were calculated for binary and continuous outcomes, respectively.

**Results:** Of the 1182 studies initially identified, 8 met the inclusion criteria for quantitative analysis. These studies included 746 patients, with 345 undergoing DC and 375 receiving conservative management. BMT alone was associated to a poor neurological function (mRS of 5–6) (OR 0.44; 95% CI 0.24–0.78; *p*‐value 0.005; *I* ^2^ = 39,8%), higher mortality at 30‐days and at last follow up (OR 0.36; 95% CI 0.19–0.66; *p*‐value 0.001; *I* ^2^ = 0%), (OR 0.33; 95% CI 0.21–0.52; *p*‐value < 0.001; *I* ^2^ = 34,8%), respectively. Length of hospital stay was superior in the DC cohort, but without statistical significance (MD 16.05; 95% CI −3.24 to 35.34; *p*‐value 0.1; *I* ^2^ = 92.9%).

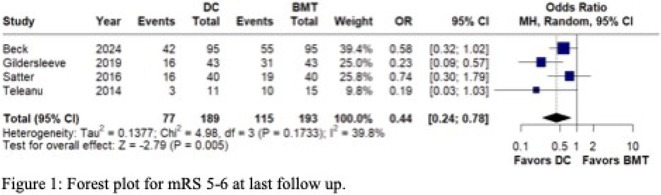

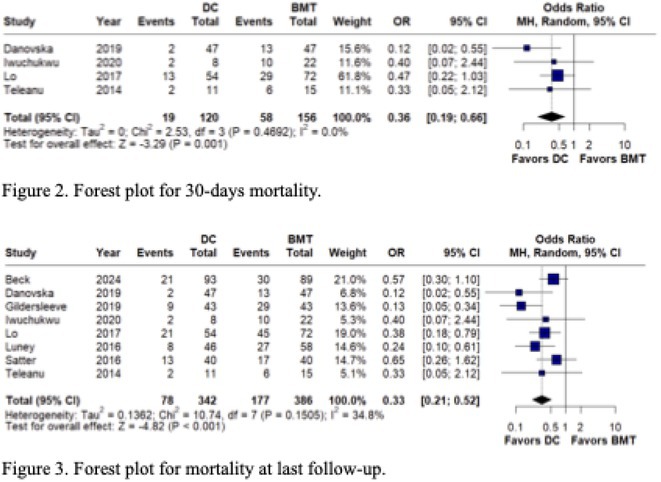

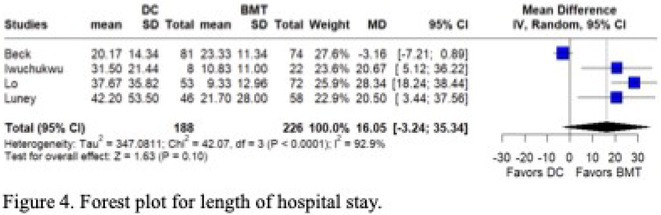

**Conclusion:** In patients with sICH, DC is associated with a reduced mortality and better neurological function, despite superior length of hospital stay, when compared to best medical treatment alone.

**Disclosure:** Nothing to disclose.

## EPO‐543

### Intraoperative salvage red blood cell transfusion on total blood transfusion in spine surgery: A meta‐analysis

#### G. da Silva Semione^1^; Y. Picanco Silva ^2^; P. Łajczak⁩^3^; L. Tamara Alves Carretta^4^; A. R. Ahmed^5^; I. Calisaya‐Madariaga^6^; F. F. Marques^7^; P. Guimarães Marcarini^8^

##### ^1^Departament of Medicine, University of West of Santa Catarina, Joaçaba, Brazil; ^2^Healthcare Institution of South Iceland, Selfoss, Iceland; ^3^Faculty of Medical Sciences in Zabrze, Medical University of Silesia, Katowice, Poland; ^4^Escola Superior de Ciências da Santa Casa de Misericórdia de Vitória, Vitória, Brazil; ^5^Jinnah Medical and Dental College, Karachi, Pakistan; ^6^Department of Neurosurgery, Hospital Nacional Edgardo Rebagliati Martins, Lima, Peru; ^7^NOESC (Universidade do Oeste de Santa Catarina), Brazil; ^8^Higher School of Sciences of the Holy House of Mercy of Vitória (EMESCAM), Vitória, Brazil

**Background and Aims:** Blood transfusion is a common practice in spine surgery, associated with potential complications. Intraoperative salvage red blood cell (SRBC) transfusion is a blood conservation technique that reinfuses a patient's own blood collected during surgery. This study aims to assess the impact of intraoperative SRBC transfusion on total blood transfusion volume in spine surgery.

**Methods:** PubMed, Embase, Web of Science, and the Cochrane Library were searched for randomized and observational studies evaluating the intraoperative use of SRBCs. Outcomes assessed included autologous transfusion rates, estimated blood loss, intraoperative allogeneic transfusion rates, operative time, postoperative allogeneic transfusion rates, and total transfusion requirements. Odds ratios (ORs) were calculated for binary outcomes, while mean differences (MDs) were calculated for continuous outcomes.

**Results:** A total of 14 studies involving 1,660 patients were included in the meta‐analysis. 44% underwent ISRBC. No significant difference was seen between ISRBC and control for AT (MD 456.99 mL; 95% CI 369.90–564.58; *I* ^2^ = 97%), IAT (MD ‐0.33; 95%CI ‐0.81 to 0.14; *p* = 0.17; *I* ^2^ = 97%), PAT (MD ‐0.24; 95% CI ‐0.49 to 0.02; *p* = 0.07; *I* ^2^ = 76%), PT (OR 0.57; 95% CI 0.30‐1.11; *p* = 0.1; *I* ^2^ = 67%) and TT (OR 0.92; 95% CI 0.43‐1.98; *p* = 0.836; *I* ^2^ = 83%). However, EBL (MD 150.76mL; 95%CI 36.62‐264.9; *p* < 0.01; I2 = 87%) and OT (MD 19.87 h; 95% CI 3.02‐36.73; *p* = 0.02; *I* ^2^ = 87%) significantly favored control.

**Conclusion:** Our results suggest no significant reduction in allogeneic transfusion rates with ISRBC transfusion in spine surgery. However, ISRBC was associated with increased estimated blood loss and operative time. Further research is needed to determine the clinical significance of these findings.

**Disclosure:** Nothing to disclose.

## Cerebrovascular diseases 4

## EPO‐544

### Prognostic factors in vascular motor aphasia after music therapy

#### A. Jouonang Teugang ^1^; N. Diagne^2^; F. Niakam Mbouleup^1^; G. Tsemo Yimta^1^; J. Badiane^2^; M. Ngoule^1^; A. Mondomobe^1^; E. Tonga^1^; C. Bangweni^1^; N. Mundih Njohjam^1^; M. ndiagne^1^; M. Sene^1^; G. Diop^1^

##### ^1^Neurology Department, Fann University Hospital, Dakar, Senegal; ^2^Physical Medicine and Rehabilitation Department, Fann University Hospital, Dakar, Senegal

**Background and Aims:** Aphasia is a language disorder linked to brain damage or dysfunction, characterized by difficulties in expression and/or comprehension. Aphasia is common after a stroke and may require lengthy, costly, and sometimes inappropriate treatments, which can hinder the patient's socio‐professional life. The use of self‐administered rehabilitation methods, such as music therapy, could improve the functional outcomes of aphasic patients.

**Objectives:** To determine the prognostic factors of post‐stroke motor aphasia treated with music therapy.

**Methods:** This was a prospective, bi‐center cohort study including patients with verbal expression disorders of vascular origin, without a history of motor aphasia or cognitive disorders. Aphasia was assessed using the LAST score. Patients were randomly divided into three groups: GBI1 (speech therapy), GBI2 (music therapy), and GNBI (no rehabilitation). Sessions were conducted for up to 3 months, with assessments made at admission, at month 1, and at month 3.

**Results:** A total of 55 patients were included, predominantly male, with an average age of 61 years. Broca's aphasia accounted for 2/5 of the study population. The mean LAST scores at 1 month were 8.7 for GBI1, compared to 11.4 for GBI2 and 6.3 for GNBI. At 3 months, the scores were 10.55, 13.4, and 6, respectively. The prognostic factors for vascular motor aphasia treated with music therapy included the musical environment, the type of stroke, and the automatic series. These factors were associated with the LAST score dimensions of naming, repetition, and automatic series.

**Conclusion:** Music therapy improves aphasia of vascular origin.

**Disclosure:** Nothing to disclose.

## EPO‐545

### Evaluation of risk factors in stroke patients with paroxysmal atrial fibrillation (PAF) and development of a risk score

#### B. Yaralıoğlu ^1^; A. Özer^1^; B. Balaban Kocas^2^; S. Yıldız^2^; S. Ucler Yaman^1^; O. Akan^1^

##### ^1^Department of Neurology, University of Health Sciences, Prof. Dr. Cemil Tascioglu City Hospital, Istanbul, Turkey; ^2^Department of Cardiology, University of Health Sciences, Prof. Dr. Cemil Tascioglu City Hospital, Istanbul, Turkey

**Background and Aims:** Our study aims to develop a new scoring system to assess the risk of paroxysmal atrial fibrillation (PAF) in stroke patients, helping to identify the correct patient group and prevent recurrent strokes.

**Methods:** 940 stroke patients included and divided into two groups: 261 with PAF, 679 non‐PAF. Logistic regression analysis was performed to assess risk factors.

**Results:** In our study, stroke history, temporal summation in the anterior/posterior circulation, gender, age, hypertension, dyslipidemia, ischemic heart disease, APC on ECG, MRS>2, NIHSS>5 were identified as risk factors for PAF. Infarction in the MCA and in multiple‐vascular areas was more likely to be associated with PAF (*p* < 0.001). Infarction in the inferior division of the MCA and the isolated occipital/occipitotemporal areas increased the risk for PAF. All cortical borderzone ischemias were seen in the PAF group. The ischemic patterns associated with PAF were confluent‐additional and small‐scattered lesions (*p* < 0.001). Infarctions in the cortical, bilateral and anterior/posterior circulations, elevated T4, moderately reduced ejection fraction, left atrial enlargement, moderate/greater mitral insufficiency or accompanying mitral stenosis were additional parameters that increased the risk of PAF. Risk‐reducing factors included smoking, basilar, lateral thalamic and internal borderzone infarction. After determining the parameters in the score, a user interface was designed using the free licensed Python language (v.3.9.0), creating a new risk scoring system for PAF.

**Figure:**
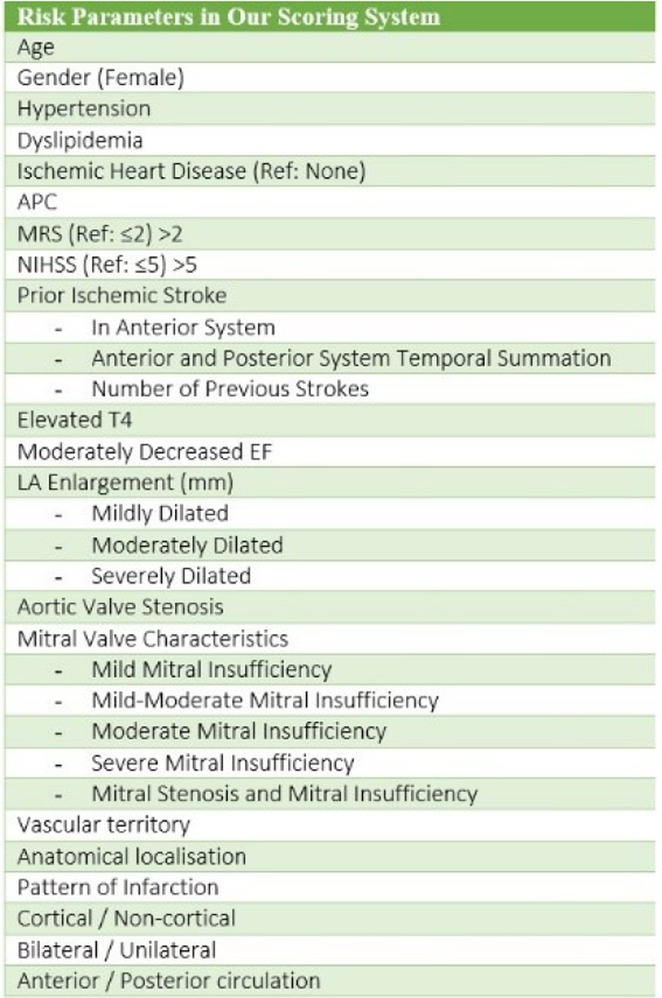
**FIGURE 1** Risk parameters in our scoring system

**Figure:**
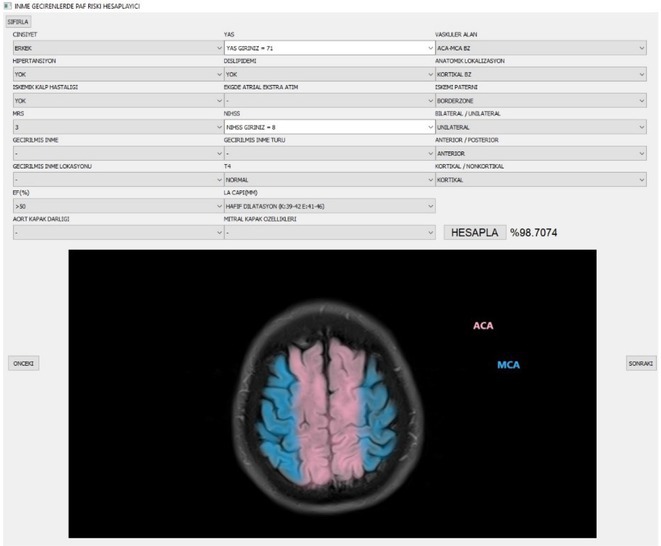
**FIGURE 2** An example of PAF risk calculator

**Figure:**
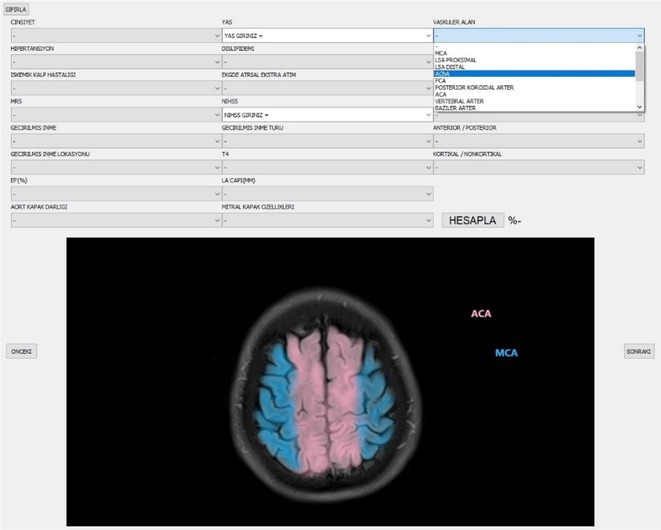
**FIGURE 3** An example of PAF risk calculator

**Conclusion:** While existing scoring systems for PAF in ESUS patients are limited, our study introduces a novel approach by incorporating detailed topographical stroke features. Our scoring system can quickly identify patients who need advanced monitoring and anticoagulation, helping to prevent recurrent strokes.

**Disclosure:** Nothing to disclose.

## EPO‐546

### Differences in serum elabela‐apelin levels in acute and chronic ischemic stroke patients with different etiologies

#### B. Yarci; Ö. Öcek; P. Ortan; S. Deger; K. Kuzu; G. Bozkaya

##### Neurology/Izmir Bozyaka Training and Research Hospital, Izmir, Turkey

**Background and Aims:** Apelin and elabela are components that play crucial roles in angiogenesis, with potential neuroprotective effects. This study aims to investigate serum apelin and elabela levels in ischemic stroke patients of different etiologies in acute and chronic periods and compare them with healthy individuals.

**Methods:** This study includes 126 patients diagnosed with ischemic cerebrovascular disease (ICVD) and 30 healthy controls. Patients were classified according to the TOAST classification into cardioembolic stroke (CES), large artery atherosclerosis (LAA), and small vessel occlusion (SVO) groups. Serum apelin and elabela levels were measured within 48 hours and at 6‐month follow‐up.

**Results:** Serum elabela levels were significantly lower in ICVD patients and subgroups during acute phase compared to healthy controls. In all patient groups, serum elabela levels significantly decreased over 6 months. Serum apelin levels didn’t differ significantly between patient groups and healthy controls in acute phase. However, SVO patients had significantly lower apelin levels compared to other subgroups. Serum apelin levels decreased significantly in the LAA and CES groups, while no change was observed in the SVO group. In chronic phase, serum elabela levels were significantly lower in SVO compared to CES patients.

**Figure:**
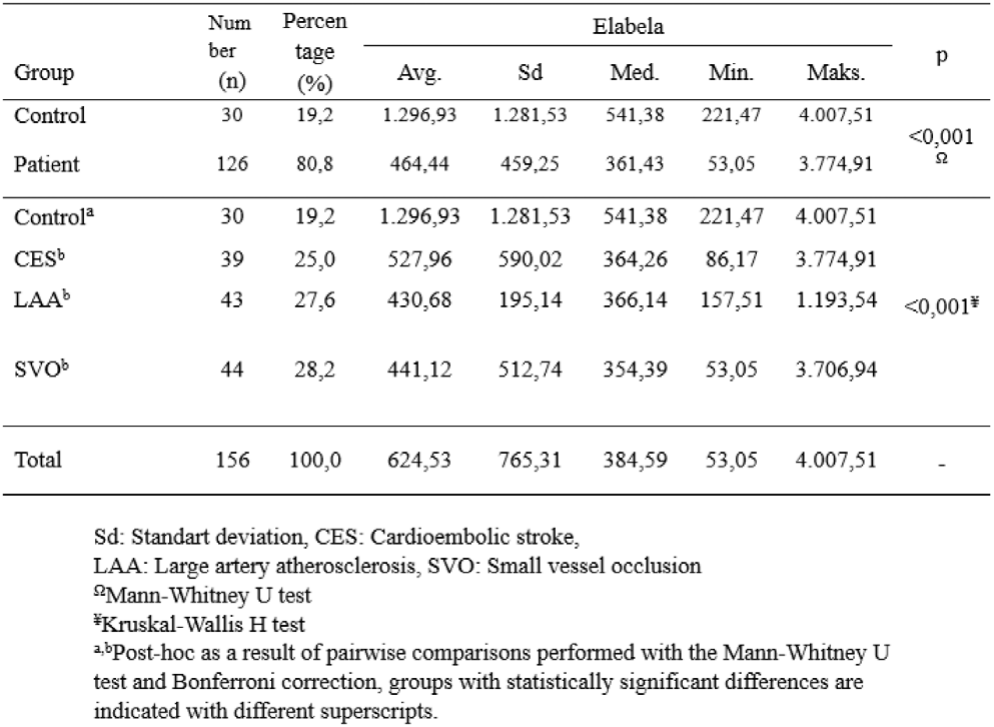
**TABLE 1** Serum elabela comparison between patient and healthy control groups.

**Figure:**
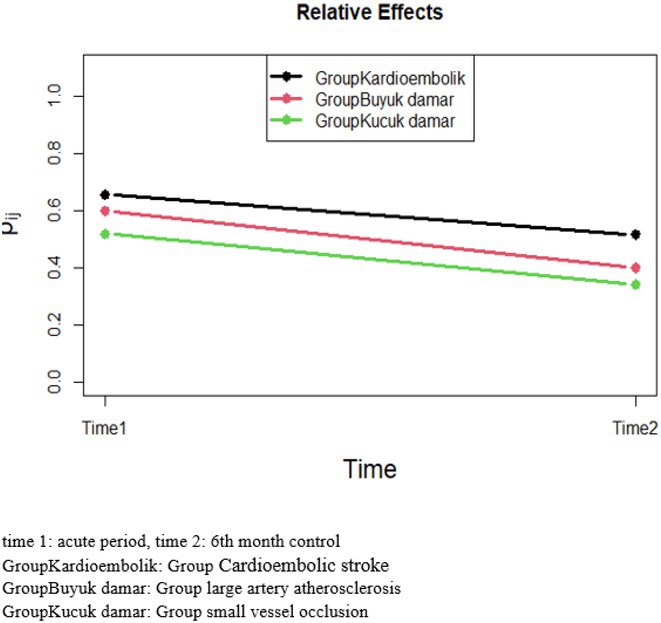
**FIGURE 1** Temporal change of serum elabela level for each subgroup

**Figure:**
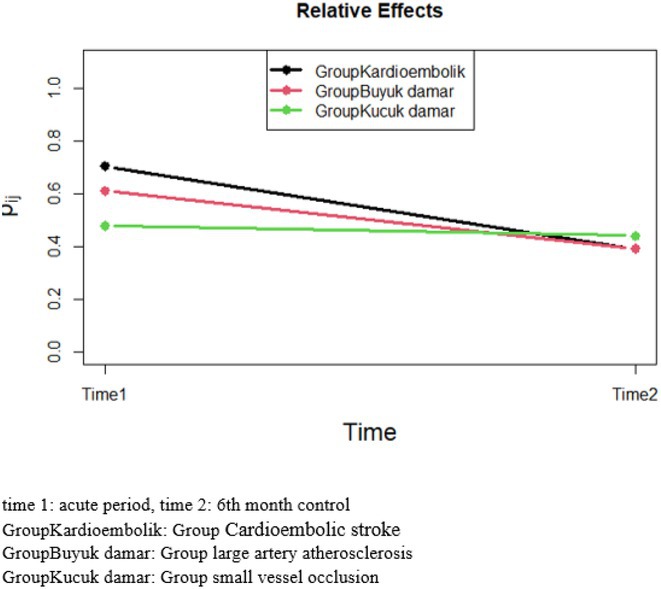
**FIGURE 2** Temporal change of serum apelin level for each subgroup

**Conclusion:** This study is the first to compare apelin and elabela levels in ICVD patients and examine their temporal changes. The fact that serum elabela levels are found to be lower in all ISVH subgroups compared to healthy controls and are related to temporal course of ISVH suggest that this parameter may be valuable for stroke.

**Disclosure:** Nothing to disclose.

## EPO‐547

### Two‐year follow‐up of bilateral spontaneous cervical carotid artery dissection with NOTCH1 mutation: A case report

#### D. Kim ^1^; J. Shin^2^; H. Jeong^2^; E. Oh^2^

##### ^1^Department of Neurology, Dong‐A University Hospital, Busan, Republic of Korea; ^2^Department of Neurology, Chungnam National University Hospital, Daejeon, Republic of Korea

**Background and Aims:** Spontaneous cervical artery dissection (CAD) is a rare but significant cause of stroke, with genetic predispositions such as mutations in the NOTCH1 gene being even less common. NOTCH 1 mutations have been associated with arterial fragility, particularly in coronary and recurrent extracranial artery dissections. This report presents a case of bilateral cervical internal carotid artery dissection (ICAD) presenting with pulsatile tinnitus, exploring the potential role of a NOTCH1 mutation in vascular pathology.

**Methods:** A 46‐year‐old male presented with bilateral pulsatile tinnitus without ischemic symptoms. Audiometric evaluation revealed no evidence of hearing impairment.

**Results:** Magnetic resonance (MR) and cerebral angiography showed severe stenosis in the distal right internal carotid artery (ICA) and fusiform dilatation with intimal flaps in the left ICA with mild proximal stenosis. Next‐generation sequencing (NGS) subsequently identified a heterozygous NOTCH1 mutation (p. Asn685Ile). Echocardiography ruled out coronary artery involvement. The patient was treated with dual antiplatelet therapy, and follow‐up imaging over two years showed persistent but stable dissections bilaterally.

**Conclusion:** NOTCH1 related vascular dissections are exceedingly rare, with only a few cases documented in the literature. This case adds to the limited evidence of the mutation's role in arterial fragility and site‐specific vascular involvement. Advanced imaging and genetic testing are crucial for the diagnosis and management of spontaneous CAD. This patient's stable vascular condition over two years implies that NOTCH1 mutations may predispose to dissection without rapid progression. Larger studies are needed to confirm the association and inform personalized management strategies.

**Disclosure:** Nothing to disclose.

## EPO‐548

### New discoveries in amyloid‐related imaging abnormalities with hemorrhage and anti‐amyloid beta monoclonal antibodies

#### D. An ^1^; Y. Xu^2^

##### ^1^Department of Neurology, Nanjing Drum Tower Hospital, Chinese Academy of Medical Sciences & Peking Union Medical College, Nanjing, China; ^2^Department of Neurology, Nanjing Drum Tower Hospital, Affiliated Hospital of Medical School, Nanjing, China

**Background and Aims:** Amyloid‐related Imaging Abnormalities (ARIA) are adverse effects that occur during amyloid beta monoclonal antibody treatment for Alzheimer's disease, including edema‐type ARIA and hemorrhage‐type ARIA (ARIA‐H). Few retrospective analyses have compared ARIA‐H incidence among individual monoclonal antibodies, and a comprehensive comparison is currently lacking. After the approval of these antibodies, research has mainly focused on dosing frequency and drug dosage, leaving it unclear whether ARIA‐H is associated with the specific characteristics of different monoclonal antibodies.

**Methods:** We compared the characteristics of 7 monoclonal antibodies marketed and under study. We chose five variables: (1) Types of Amyloid Beta Binding, (2) Polymer Affinity, (3) Binding Epitope, (4) Fc Subtype, (5) Amyloid Beta Clearance Rate.

**Results:** The risk of ARIA‐H, from highest to lowest, is as follows: Donanemab, Aducanumab, Bapineuzumab, Lecanemab, Gantenerumab, Crenezumab, Solanezumab. Besides, ARIA‐H is associated with the characteristics of monoclonal antibodies. (1) More mature Amyloid Beta clearance is associated with a higher risk of ARIA‐H. (2) Lower clearance of Amyloid Beta oligomers is associated with a higher risk of ARIA‐H. (3) Amyloid Beta clearance closer to the N‐terminus is associated with a higher risk of ARIA‐H. (4) Monoclonal antibodies with an IgG4 structure are more likely to cause ARIA‐H than those with an IgG1 structure. (5) Faster achievement of Amyloid Beta clearance thresholds is associated with a higher risk of ARIA‐H.

**Conclusion:** This research enhances our understanding of ARIA‐H and may guide future monoclonal antibody drug development, which may improve the cognition and overall prognosis of Alzheimer's disease patients.

**Disclosure:** Nothing to disclose.

## EPO‐549

### Quantitative EEG can predict early functional stroke outcome after mechanical thrombectomy

#### K. Stojić ^1^; M. Kovačević^2^; T. Švabić‐Međedović^2^; I. Berisavac^2^; U. Mirčić^3^; P. Stanarčević^2^; D. Jovanović^2^; K. Trajković^3^; N. Vojvodić^2^

##### ^1^Medical Faculty, University of Belgrade, Belgrade, Serbia; ^2^Neurology Clinic, University Clinical Center of Serbia, Medical Faculty, University of Belgrade, Belgrade, Serbia; ^3^Center for Radiology, University Clinical Center of Serbia, Belgrade, Serbia

**Background and Aims:** Stroke caused by large artery occlusion (LAO) in the anterior circulation is a leading cause of morbidity and mortality, but mechanical thrombectomy (MT) improves functional outcomes. Quantitative electroencephalography (qEEG) allows objective assessment of functional damage and may predict clinical outcomes. This study aimed to evaluate qEEG parameters in predicting early functional clinical outcome in MT‐treated anterior circulation LAO stroke patients.

**Methods:** We included consecutive LAO anterior circulation stroke patients treated with MT at the Neurology Clinic, University Clinical Center of Serbia, between July 20 and November 20, 2024. EEG was performed within 72h post‐MT using cap electrodes (10‐10 system) and NicoletOne software. Delta/alpha ratio (DAR) and (delta+theta)/(alpha+beta) ratio (DTABR) were calculated via Fast Fourier Transformation. Ischaemic volume was measured using computed tomography 24h post‐MT. Early functional outcome was assessed on exmission using modified Rankin Scale (mRS), categorizing patients as having poor outcomes (mRS>2) or favourable outcomes (mRS< = 2). Statistical analysis (SPSS V.26) included Mann‐Whitney for group differences, logistic regression for predictive values, and Spearman's coefficient for correlation, with p<0.05 for significance.

**Results:** The study included 30 patients (Table 1). DTABR was significantly higher in patients with mRS>2 than mRS< = 2 (0.14 vs. 0.03, *p* = 0.008), while DAR showed no significant difference between groups. A DTABR cut‐off at >0.1 had a positive predictive value of 83.3%, 95% CI [62.9‐95.6%] for unfavourable outcomes at exmission, with odds ratio 7.0 95% CI [1.3‐37.9]. No significant correlation was found between DAR, DTABR and ischaemia volume.

**Figure:**
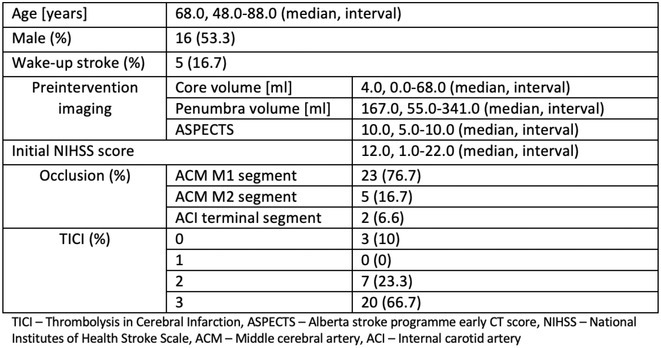
TABLE 1

**Conclusion:** qEEG is a promising tool for predicting functional outcomes in MT‐treated stroke patients.

**Disclosure:** Nothing do disclose.

## EPO‐550

### A cryptogenic stroke associated with non‐infectious Endocarditis and antiphospholipid syndrome

#### M. Cholakova; N. Mihnev; I. Staikov

##### Acibadem City Clinic UMHAT Tokuda, Sofia, Bulgaria

**Background and Aims:** Ischemic stroke is the most common neurological complication of endocarditis. Cardiac myxoma (CM) is the most frequent type of cardiac neoplasm.The antiphospholipid syndrome (APS) occurs most commonly in the systemic lupus erythematosus.

**Methods:** Testing for thrombophilia, laboratory test, neurological exam, echocardiography, magnetic resonance tomography (MRI).

**Results:** We present a clinical case42‐year old female admitted with weakness in the left foot. She weak up with difficulties in performing dorsal and plantar flexion evaluated3/5with manual muscle testing. No pathological reflexes were found.Firstly, peripheral nerve damaged was observed. After MRIof the head was performed showing ischemic stroke in the high right frontal parietal area (image 1, 2, 3, 4). After consultation with cardiologist and echocardiography, it was found endocarditis of mitral valve with mild mitral regurgitation. Patient was tested for thrombophilia with positive antiphospholipid antibodies (anticardiolipin antibodies (ACL)/+/‐beta2Glicoprotein I antibodies/+/). Several sets with blood cultures were taken‐all of them were negative and blood tests were negative for inflammation (normal levels for C‐reactive protein, normal count for leucocytes). A surgery was performed for mitral valve with prosthesis. Biopsy of the tumor valve formation showed mitral valve myxoma (image 5). Only few clinical cases have described the coexistence of atrial myxoma and APS which was found in our patient. Interleukin‐6 (IL‐6) could be produced by myxoma and trigger an immunological reaction leading to the secondary APS.

**Figure:**
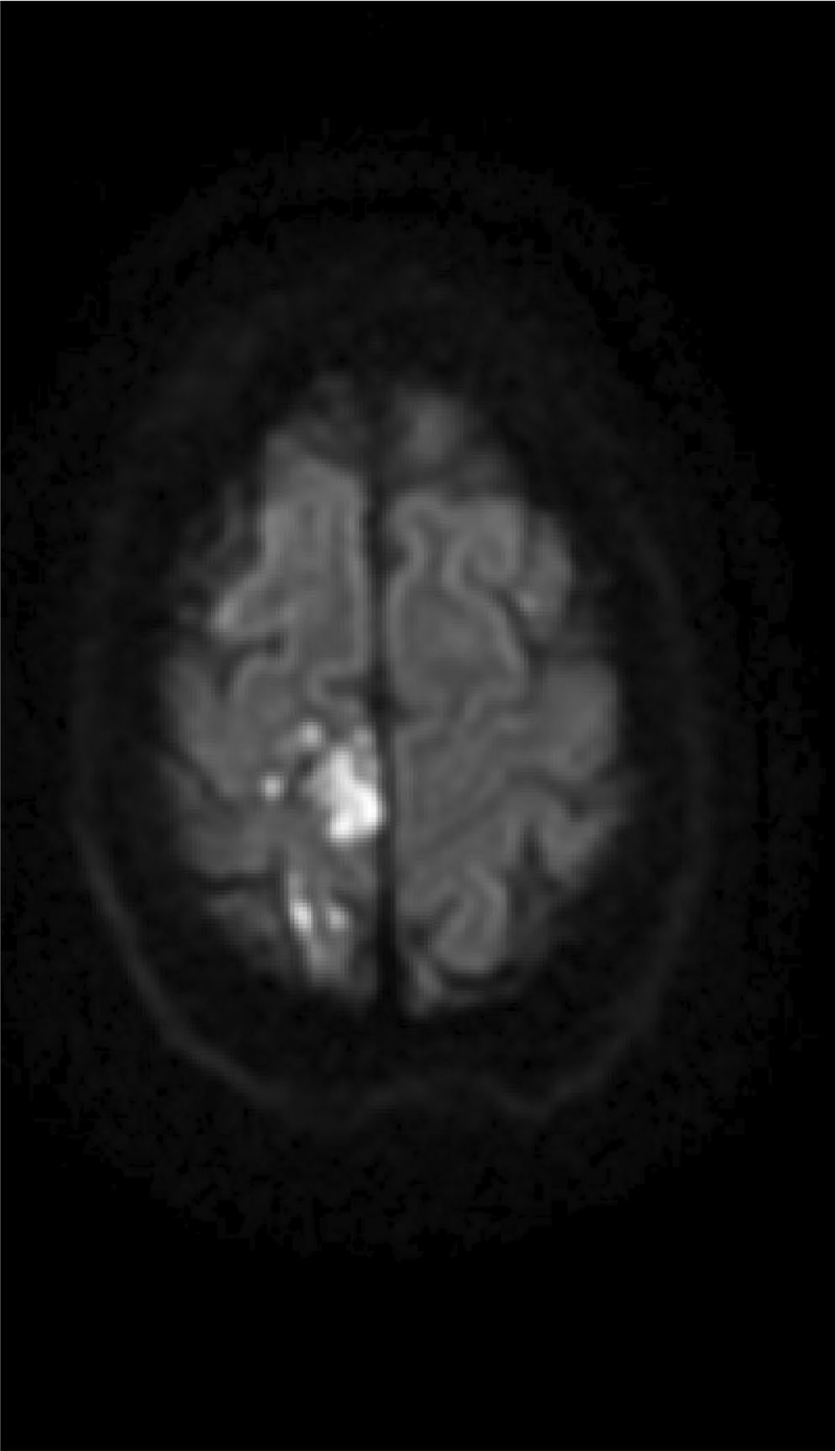
image 1

**Figure:**
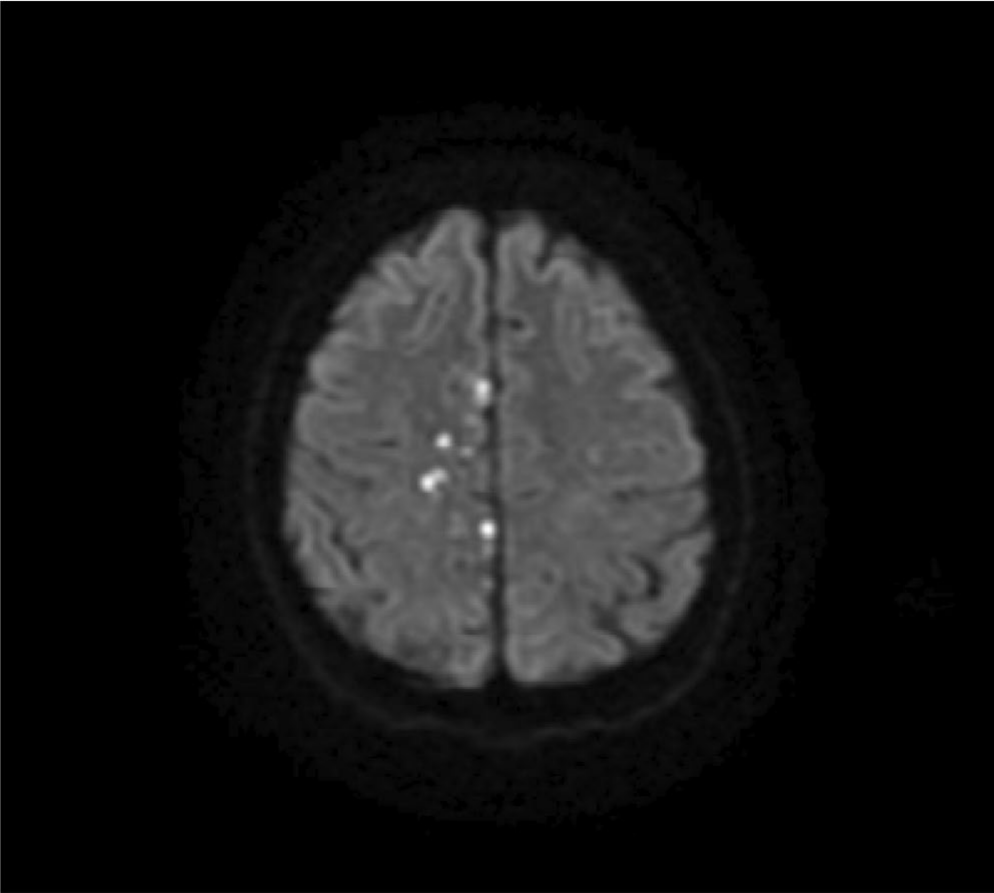
image 2

**Figure:**
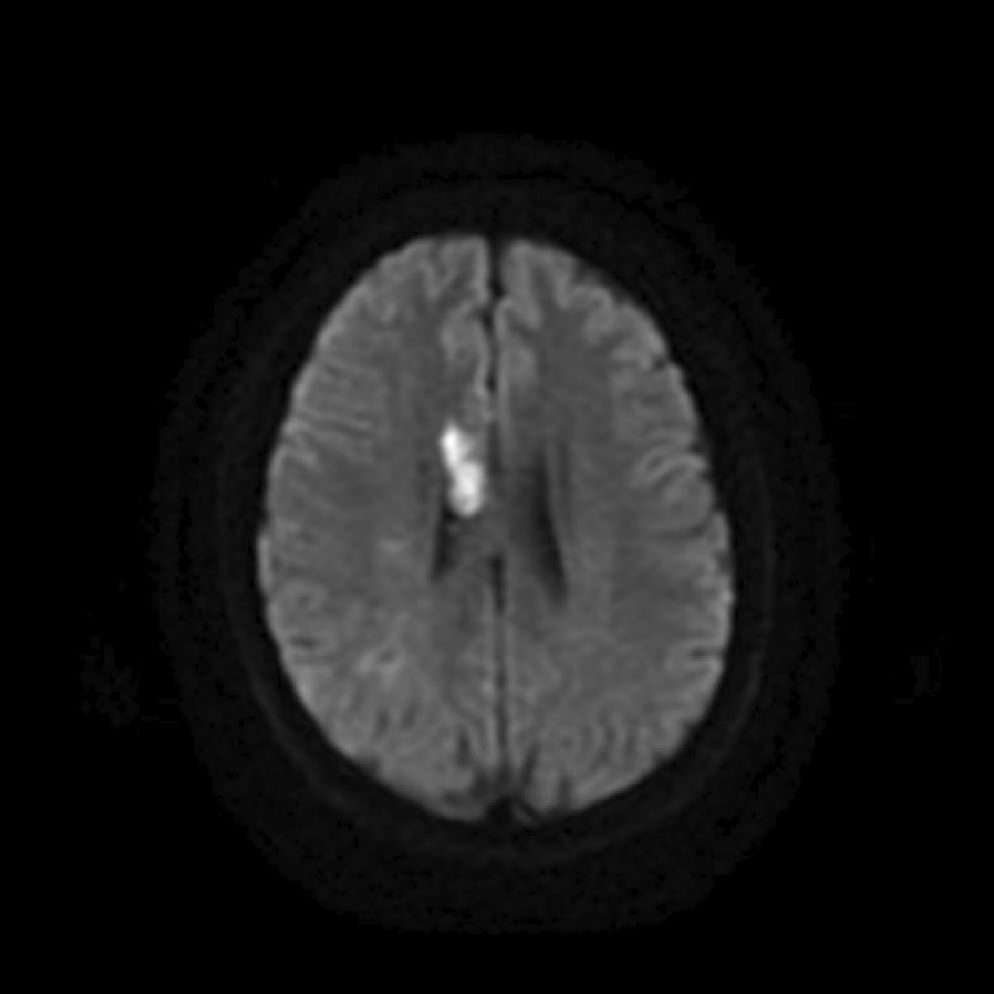
image 3

**Conclusion:** Endothelial injury from circulating cytokines such as tumour necrosis factor or interleukins in a hypercoagulable state patient causes platelets aggregation in the affected valves.It is mandatory to check them for underlying malignancy,systemic lupus erythematosus,antiphospholipid antibody syndrome.Non infectious endocarditis should be excluded in young adults with cryptogenic stroke.

**Disclosure:** Nothing.

## EPO‐551

### Risk factors for the epileptic seizures in cerebral venous sinus thrombosis

#### M. Vasilieva ^1^; I. Vasilieva^2^; I. Plesca^3^; S. Groppa^1^

##### ^1^Nicolae Testemitanu State University of Medicine and Pharmacy, Laboratory of Neurobiology and Medical Genetics, Chisinau, Republic of Moldova; ^2^Nicolae Testemitanu State University of Medicine and Pharmacy, Department of Laboratory Medicine, Chisinau, Republic of Moldova; ^3^Nicolae Testemitanu State University of Medicine and Pharmacy, Department of Neurology, Chisinau, Republic of Moldova

**Background and Aims:** Cerebral venous sinus thrombosis (CVST) constitutes 0.5‐1% of all strokes in all age groups. Epileptic seizures (ES) occur in 12‐31.9% of patients with CVST, while up to 44.3% of patients may have ES in the early stage of the disease. Our objective is to determine the risk factors for ES in CVST.

**Methods:** A retrospective study from 2010‐2024 in the Emergency Institute was performed. 50 patients were included in the study. The patients with a past history of epilepsy and structural lesions other than CVST were excluded.

**Results:** Our patients were divided into groups: with ES (14 patients with acute early seizures (28%)) and without ES (36 patients (72%)). The median age was 40 years. Females are more commonly (64.3%) involved in developing ES in CVST. ES group include‐28.6% with Status Epilepticus (SE), and 71.4%‐focal with secondary bilateral seizures. Sagittal superior sinus (57.2%), Galen Vein (28.6%), and cavernous sinus (14.3%) involvement were more common in the ES group. Sagittal inferior sinus (55.56%), transverse sinus (27.75%), and sigmoid sinus (16.67%) involvement were in the non‐ES group. The altered state of consciousness (35.7%) presents a risk factor for the ES. Hemorrhagic infarction, in the frontal cortex was present in 35.7% of the ES group.

**Conclusion:** Risk factors for acute ES in CVST are sagittal superior sinus, Galen Vein, and cavernous sinus involvement. Galen Vein thrombosis was seen to be included in patients with SE. Altered state of consciousness, hemorrhagic infarction in the frontal cortex and female gender are predisposing factors for ES.

**Disclosure:** Nothing to disclose.

## EPO‐552

### Endovascular or medical treatment for acute stroke due to medium‐vessel occlusion: Which is the best option?

#### M. Campos Jiménez; R. Pastor González; N. Mena García; G. Gabañas Engenios; M. Matute Lozano; S. García Madrona; A. De Felipe Mimbrera; R. Vera Lechuga; J. Masjuan Vallejo; G. García Alcántara; A. Cruz Culebras

##### Stroke Unit, Neurology Department, Ramón y Cajal University Hospital, Madrid, Spain

**Background and Aims:** Medium‐vessel occlusion (MeVO) accounts for 30% of acute ischemic stroke cases. Deciding on treatment with mechanical thrombectomy (MT) remains a challenging task. This study aimed to compare three clinical strategies.

**Methods:** We conducted a retrospective analysis at a stroke center from April 2023 to April 2024. Patients with MeVO involving occlusions in segments M2/M3/M4, A2/A3, or P1/P2 were included. Patients treated with endovascular thrombectomy (EVT), intravenous thrombolysis (IVT), or medical management were compared using multivariable logistic regression. The primary outcome was to assess functional independence, defined as a modified Rankin Scale (mRS) score of 0–2 at 3 months, and treatment safety.

**Results:** A total of 138 patients were included in the study. Thirty‐one patients (22.5%) received medical management without reperfusion, 48 (34.8%) underwent IVT only, and 59 (42.8%) underwent MT with or without IVT. The mean age was 73 years. The most common occlusion site was M2 (42.8%), followed by M3 (15.9%). No significant differences were found in functional outcomes as measured by mRS at 3 months (P = 0.713). A lower baseline NIHSS score was significantly associated with favorable functional outcomes (OR 1.18, 95% CI 1.03–1.36) across all groups.

**Conclusion:** This study did not find significant differences in functional outcomes among patients with medium‐vessel occlusions treated with EVT, IVT, or medical management. Larger registries are needed to define the optimal strategy for this patient group.

**Disclosure:** Nothing to disclose.

## EPO‐553

### The reliability of modified Rankin Scale assessed at discharge from the stroke unit for the actual functional outcome

#### N. Pożarowszczyk; M. Nowak; H. Ziąbska; I. Kurkowska‐Jastrzębska; M. Karliński

##### II Neurology Department, Institute of Psychiatry and Neurology, Warsaw, Poland

**Background and Aims:** This study aimed to evaluate the reliability of the mRS score obtained at discharge from the stroke unit (SU) compared to the assessment shortly after discharge when patients had already encountered challenges of home environment or more complex tasks in a neurological rehabilitation ward (NRW). Special emphasis was placed on distinguishing independence (mRS 0‐2) from dependence (mRS 3‐5) and no significant disability (mRS 0‐1) from disability (mRS 2‐5).

**Methods:** We enrolled 116 acute ischaemic stroke discharged from SU with residual deficits (mRS 1–4) from October 2020 to June 2022, assessed by a certified rater at discharge and 7–21 days later. The agreement was reported as Krippendorff's alpha.

**Results:** Of 109 analysed patients 61 (56%) were transferred to NRW. The agreement between mRS assessments at discharge from SU and shortly after was low overall (alpha 0.34) and in patients discharged home (alpha 0.41), while very low in patients transferred to NRW (alpha 0.10). At discharge home: 21% of patients initially assessed as mRS 0‐1 turned out 2‐5; 9% of those assessed as mRS 0‐2 turned out 3‐5; 35% of assessed as mRS 3‐4 were scored 0‐2. At discharge to NRW: none was initially assessed as mRS 0‐1; 10% of those assessed as mRS 0‐2 turned out 3‐4; 35% of assessed as 3‐4 turned out 0‐2.

**Conclusion:** The agreement between mRS assessments at discharge from SU and shortly after is modest. This points to the risk of bias when SU‐based mRS scores are used to represent long‐term functional outcome.

**Disclosure:** Nothing to disclose.

## EPO‐554

### Newly diagnosed diabetes mellitus and prediabetes in a stroke unit

#### R. Rodrigues ^1^; M. Dias da Costa^1^; A. Paula^1^; M. Rosário^1^; T. Pinho e Melo^1,2^; A. Fonseca^1,2^

##### ^1^Department of Neurosciences and Mental Health, Local Health Unit of Santa Maria, Lisbon, Portugal; ^2^Egas Moniz Study Center, University Neurology Clinic, Faculty of Medicine, University of Lisbon, Lisbon, Portugal

**Background and Aims:** Diabetes mellitus and prediabetes are modifiable vascular risk factors for ischemic stroke that double the risk of stroke and are associated with poorer functional prognosis, and increased risk of recurrent cerebrovascular events. The objetives were to determine the prevalence of newly diagnosed diabetes and prediabetes in patients with acute ischemic stroke admitted to the stroke unit of a university hospital and to evaluate whether there is an association with stroke etiology.

**Methods:** Retrospective observational study of a prospectively included sample of adult patients admitted to a stroke unit due to ischemic stroke or TIA during the second semester of 2023. A chi‐square test was performed to assess the association between newly diagnosed diabetes or prediabetes and stroke etiology according to TOAST classification.

**Results:** A total of 199 patients were included, 37 of whom had a prior diagnosis of diabetes. Among the included patients, 53.3% were newly diagnosed with diabetes (7.5%) or prediabetes (92.5%). Of the latter group, 51.4% showed a high risk of progression to diabetes. Regarding ischemic stroke etiology, the majority were cardioembolic, followed by large artery atherosclerosis and small vessel occlusion. No statistically significant association was found between new diagnosis of diabetes or prediabetes and a specific stroke etiology.

**Conclusion:** The high prevalence of newly diagnosed prediabetes and diabetes in our sample, corroborates data indicating that these conditions are frequently underdiagnosed in patients with acute ischemic stroke. Including glycated hemoglobin measurement in stroke unit protocols allows for better secondary prevention through tailored management of these vascular risk factors.

**Disclosure:** Nothing to disclose.

## EPO‐555

### The two most interesting polymorphic sites in the 9r21 chromosomal locus associated with arteriovenous malformations

#### S. Erkinova

##### Department of Neurology, Pediatric Neurology and Medical Genetics, Tashkent Pediatric Medical Institute, Tashkent city, Uzbekistan

**Background and Aims:** An aberrant tangle of arteries and veins that diverts blood from the arterial bed into the venous, excluding the capillary network, is known as an arteriovenous malformation (AVM). The two most interesting polymorphic sites in the 9r21 chromosomal locus are rs7865618 in the CDKN2A gene and rs1333040 in the CDKN2B gene. The CDKN2A/B gene is involved in cell cycle regulation and affects a number of physiological processes, including aging, apoptosis, stem cell self‐renewal, and tissue remodeling. In certain hereditary disorders, dysregulation of these genes has been connected to aberrant vascular development. Theoretically, mutations in these genes may contribute to the creation of AVMs if they affect the integrity or repair of the normal vessel wall. This study aims to investigate the contribution of allelic polymorphisms of the CDKN2B and CDKN2A genes to the genetic susceptibility to the development of AVM in Uzbek citizens.

**Methods:** 95 individuals from Tashkent's clinical centers were included in the study group. Using competing TaqMan probes, polymorphic gene variants were identified by real‐time PCR.

**Results:** According to data, patients with the GG genotype have a roughly two‐fold increased risk of developing an AVM compared to those with the GA and AA genotypes for the polymorphic locus rs7865618 of the CDKN2A gene (OR = 1.915, CI = [1.158‐3.167], *p* = 0.01).

**Conclusion:** Thus, for Uzbek citizens, genotype GG rs7865618 of the CDKN2A gene is a risk factor for the development of AVM.

**Disclosure:** Nothing to disclose.

## EPO‐556

### Prognostic impact of malnutrition assessed by bioelectrical impedence vector analysis in acute ischemic stroke

#### S. Dal Bello ^1^; L. Ceccarelli^1^; Y. Tereshko^3^; G. Gigli^2^; M. Valente^1^; G. Merlino^3^

##### ^1^Clinical Neurology Unit, Santa Maria della Misericordia University Hospital, Udine, Italy; ^2^Department of Medical Area, University of Udine, Udine, Italy; ^3^SOSD Stroke Unit, Department of Head‐Neck and Neuroscience, Azienda Sanitaria Universitaria Friuli Centrale (ASUFC), Udine, Italy

**Background and Aims:** Until now, the assessment of nutritional status in stroke patients has been mainly based on laboratory‐based composite scores. Bioelectrical Impedance Analysis (BIA) and Bioelectrical Impedance Vector Analysis (BIVA) are non‐invasive, cost‐effective, and rapid methods that accurately provide body composition, nutritional and hydration status. The aim was to compare the ordinal distribution of the mRS scores 90 days after an acute ischemic stroke, distinguishing between malnourished and non‐malnourished patients according to the BIVA malnutrition parameter.

**Methods:** We conducted a single‐centre prospective observational study on all patients admitted for acute ischemic stroke to our Centre between April 1st 2024 and September 30th 2024. We applied the IPW statistical technique and ordinal logistic regression to compare mRS scores in malnourished and non‐malnourished patients.

**Results:** We analysed 195 ischemic stroke patients using the BIVA. Of these, 37 patients (19%) were malnourished at the time of admission to our Stroke Unit. The ordinal distribution of mRS scores 90 days after the ischemic stroke was higher in patients who were malnourished upon Stroke Unit admission according to BIVA parameters (cOR 3.34; *p* = 0.001). Even after accounting for relevant covariates, malnutrition remained an independent predictor of unfavourable outcomes (acOR 2.79; *p* = 0.005), along with NIHSS score at admission (acOR 1.19; *p* < 0.001), intravenous thrombolysis (acOR 0.28, *p* < 0.001), absolute lymphocyte count (cOR 1.01; *p* = 0.027), and albumin concentration (cOR 0.82; *p* < 0.001).

**Conclusion:** Malnutrition, assessed through BIVA at the time of admission to the Stroke Unit, is associated with worse clinical outcomes at 90 days following the ischemic cerebrovascular event.

**Disclosure:** Nothing to disclose.

## EPO‐557

### Eagle syndrome

#### T. Unt

##### Neurology and Psychiatry Clinic, Stroke Department, West Tallinn Central Hospital, Tallinn, Estonia

**Background and Aims:** Eagle syndrome is a rare condition characterized by elongated styloid processes or calcified stylohyoid ligaments that may impinge on adjacent anatomical structures, leading to diverse symptoms. It was first described in 1937 by Watt W. Eagle with classical symptoms of throat pain, dysphagia, and referred otalgia, typically exacerbated by head movement or palpation. This case highlights an unusual presentation with syncopal episodes resulting from elongated styloid process’ pressure on internal carotid artery.

**Methods:** Case report.

**Results:** A 59‐year‐old man presented to the emergency department with recurrent syncopes which had increased in frequency, now presenting monthly. Episodes started with hazy vision, general weakness, sensory disturbances on left side of the head after which he lost conciousness for few seconds. He also reported occasionally hearing pulsating noise while falling asleep and occasional throat discomfort. Neurological and cardiovascular examinations were unremarkable. CT scan of the brain was normal, CT‐angiography with 3D reconstruction revealed bilateral elongated styloid processes, the right one being in direct contact with right internal carotid artery in extracranial portion causing significant compression and ~50% narrowing of its lumen. Patient was referred to vascular surgeon for follow‐up and additional testing. Repeat imaging after 3 months was without dynamics and at the moment patient remains on watchful waiting surveillance.

**Figure:**
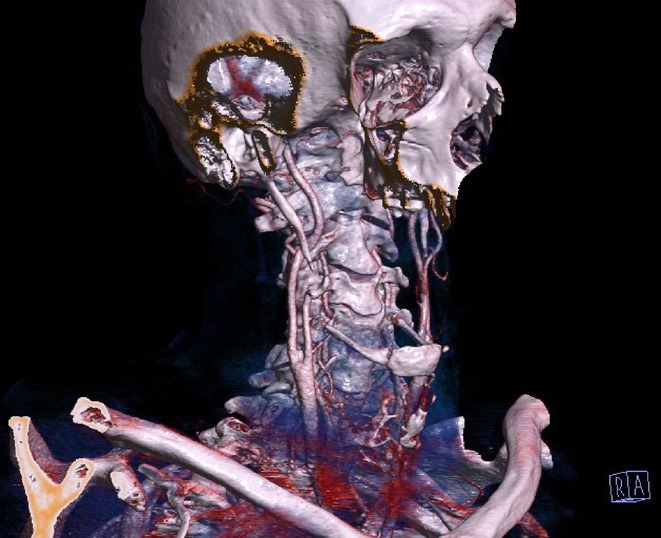
Image 1 3D reconstruction of CT angiography showing conflict of elongated styloid process and internal carotid artery on the right side.

**Conclusion:** This case underscores the need for heightened awareness of Eagle syndrome in patients presenting with atypical symptoms such as syncopal episodes. Early diagnosis and appropriate interventions can significantly alleviate symptoms and improve patient outcomes.

**Disclosure:** Nothing to disclose.

## Cerebrovascular diseases 5

## EPO‐558

### Shifting mortality trends in stroke patients: Analysis of U.S. national data from the CDC WONDER database

#### A. Elgenidy ^1^; O. Alomari^2^; A. Afifi^3^; A. Hassan^1^

##### ^1^Department of Neurology, Faculty of Medicine, Cairo University, Cairo, Egypt; ^2^Hamidiye International School of Medicine, University of Health Sciences, Istanbul Turkey; ^3^University of Toledo Medical Center, Ohio, USA

**Background and Aims:** Stroke remains a leading cause of mortality worldwide, yet recent trends in stroke‐related deaths have shown variation across time, gender, and stroke subtypes. This study aims to analyze national stroke mortality trends to identify temporal patterns and demographic disparities.

**Methods:** We conducted a retrospective analysis of stroke‐related mortality in the U.S. from 1999 to 2020 using the CDC WONDER database. Joinpoint regression was employed to assess trends in stroke‐related deaths, calculating the annual percent change (APC) with 95% confidence intervals (CIs).

**Results:** Mortality declined significantly from 1999 to 2013 in individuals <45 years (‐1.88%/year) and from 1999 to 2012 in those 45–64 years (‐2.29%/year), followed by an increase (2018–2020: +3.82%/year). In ≥65 years, a sharp decline (1999–2006: ‐7.24%/year) slowed, with a slight rise from 2012 to 2020 (+0.45%/year). Black individuals exhibited a significant decline from 2002 to 2012 (‐4.70%, 95% CI: ‐5.44 to ‐4.36), followed by a subsequent rise (1.22%, 95%CI: 0.69 to 1.92). Stroke subtype analysis revealed a consistent decline in hemorrhagic stroke mortality, while mortality from cerebral infarction showed a sharp increase from 2014 to 2017 (37.43%, 95% CI: 26.78 to 44.51). Gender analysis indicated an early decline in stroke mortality for both males and females, but a significant reversal in recent years, particularly in males (Slope 4: 0.0421, *p* < 0.0001).

**Figure:**
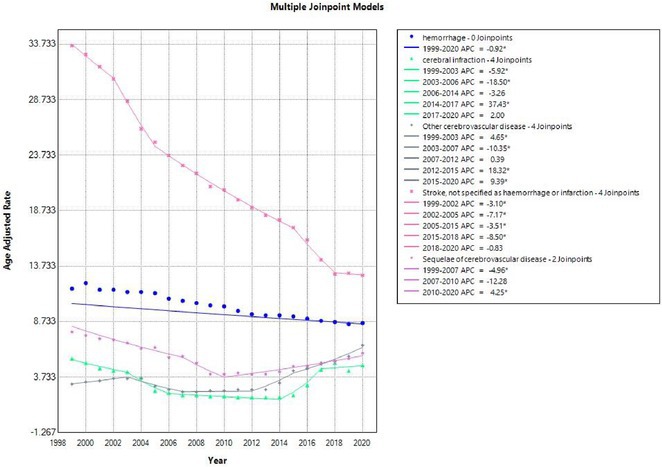
**FIGURE 1** Trend analysis for stroke patients subgrouped by the Cause

**Conclusion:** While stroke mortality rates declined substantially between 1999 and 2012, recent data suggest a plateau or reversal in progress, particularly among middle‐aged adults, Black populations, and those with ischemic stroke.

**Disclosure:** Nothing to disclose.

## EPO‐559

### Intracerebral hemorrhage in the young: Clinical characteristics and outcomes in a university hospital stroke registry

#### A. Gujjar ^1^; T. Siddiqui^2^; G. Al‐Sarmi^3^; A. Al‐Qassabi^2^; H. Al‐Abri^2^; S. Raniga^4^; F. Al‐Azri^4^; A. Al‐Asmi^1^

##### ^1^Department of Medicine (Neurology Unit), College of Medicine and Health Sciences, Sultan Qaboos University, Muscat, Oman; ^2^Department of Medicine(Neurology Unit), Sultan Qaboos University Hospital, Muscat, Oman; ^3^Oman Medical Specialty Board, Muscat, Oman; ^4^Department of Radiology and Molecular Imaging, Sultan Qaboos University Hospital, Muscat, Oman

**Background and Aims:** Intracerebral hemorrhage (ICH) is a severe stroke syndrome. While commonly encountered >60 years age, ICH is also occurs in younger individuals, with varied characteristics. We compared the clinical and outcome profiles of ICH among patients <50 and >50 years age in a University Stroke‐Registry at Oman.

**Methods:** Hospital records were reviewed for demographics, risk‐factors, cause, clinical characteristics and outcomes. Univariate and logistic regression analyses were utilized to compare profiles among younger (18‐50 yr ICHy) and older (>50 yr ICHo) cohorts.

**Results:** Among 1483 patients with stroke over 10 years, 263 had ICH (17.7%) (age: 62+13 yrs; M:F::1.7:1). 79/263 (30%) had ICHy (age: 44.8+6 yrs; M:F::1.8:1) while 183/263(70%) were older (>50 yr; ICHo: age 65.5+10; M:F::1.6:1). Underlying causes of ICHy were hypertension or hypertensive‐emergency (HTN) in 91%. Causes of ICHo were hypertension (73%), cerebral small‐vessel‐disease (15.8%) and cerebral‐amyloid‐angiopathy (5%). Altered sensorium, hemiparesis, aphasia were presenting symptoms in both. Mean ICH volumes (30.5 + 41 mL vs 27.7 + 34 mL) were similar. 22.8% of patients died. Good outcomes (MRS 0‐3) were more prevalent in ICHy than ICHo (38% vs 26.6%, *p* = 0.012). Gender, age, altered sensorium and hemiparesis were independent predictors of mortality (*p* < 0.02). We explored the utility of a modified ICH‐score with lower age cut‐offs for improved outcome predictability.

**Conclusion:** Up to 30% of all ICH patients are <50 years of age. Hypertensive crisis was the most common precipitating cause of ICHy. While ICH volumes and mortality are similar to older patients, good outcomes are higher in ICHy. Improved control of hypertension to prevent hypertensive crises may have higher impact for ICH prevention in young adults.

**Disclosure:** Nothing to disclose.

## EPO‐560

### Prevalence of post stroke delirium and associated risk factor, a multicenter prospective study, Addis Ababa Ethiopia

#### B. Ketema

##### Tikur Anbessa Specialized Hospital, Addis Ababa, Ethiopia

**Background and Aims:** Post‐stroke delirium is a frequent and significant complication of stroke. The impact of post‐stroke delirium on stroke recovery is substantial. It leads to prolonged hospital stays, increased dependence and mortality rates. As a result, early identification and prompt treatment of post‐stroke delirium are imperative for optimizing outcomes in stroke patients.

**Methods:** We performed a prospective observational cross sectional study, including all the stroke patients admitted to respective study areas during the study period. A total of 101 participants who fulfilled the inclusion criteria were involved in this study. Data was collected using interviewer administered Questionnaire with well tested and validated tool, Patients were assessed for Delirium within 48 hour of admission and subsequently screened every 12 hours.

**Results:** Out of 101 patients 26(25.7%) had Post stroke Delirium. Majority 56 (55.4%) of the patients were females. The mean (SD) age of the study participants was 56.05 ± 15.38 years, the mean time in day's until the occurrence of delirium is 3 ± 1 days. Multivariable logistic regression analysis showed that, Age greater than 60(AOR = 19.1, 95% CI (1.7‐211) *p* = 0.016, Presence of Sepsis (AOR = 8.3, 95% CI (1.2‐56) *p* = 0.029, Presence of Polypharmacy (AOR = 157, 95% CI (10.2‐244) *p* = 0.0001, Presence of Electrolyte Derangement (AOR = 65.2, 95% CI (3.4‐124.1) *p* = 0.005 were statistically significant risk factors.

**Conclusion:** Our Study showed that Post Stroke Delirium occurs in a quarter of patients admitted with Diagnosis of Acute Stroke, and the Identified risk factors were Age greater than 60, Polypharmacy, Presence of Sepsis and Electrolyte Derangement, Medical professionals responsible for caring for acute stroke patients should be vigilant in identifying those at higher risk of developing post‐stroke delirium.

**Disclosure:** Nothing to disclose.

## EPO‐561

### Possible sleep quality factors predictors of wake‐up strokes

#### C. Cunha ^1^; P. Simões^2^; I. Carvalho^1^; C. Fernandes^1^; J. Sousa^1^; F. Barros^1^; D. Damas^1^; C. Silva^1^; J. Sargento‐Freitas^1^; A. Brás^1^

##### ^1^Neurology department, Coimbra University Hospital Centre, Coimbra, Portugal; ^2^Faculty of Medicine of the University of Coimbra, Coimbra, Portugal

**Background and Aims:** Wake‐up stroke (WUS) physiopathology is not completely understood but studies report a possible sleep association.

**Methods:** We conducted a prospective observational study including all patients admitted to the neurology department for acute stroke during a 4‐month period. We excluded patients due to hemorrhagic stroke, impaired language or disagreement with the written consent. Selected 81 patients that were screened about vascular risk factors. Sleep quality was assessed using a self‐fulfilled questionnaire, STOP‐BANG Sleep Apnea Questionnaire, Epworth Sleep Scale, and Insomnia Severity Index. Clinical and demographic data was collected, and statistical analysis performed.

**Results:** The sample included 81 patients (43 men) with a mean age of 73.7 ± 12.9years at symptoms’ onset; 18.52% patients (*n* = 15) had WUS. NIHSS score was higher in WUS than non‐wake‐up stroke (NWUS) patients (9.73 ± 4.25 vs. 6.65 ± 4.81, *p* = 0.013). Cervical perimeter showed a mean of 39.8 ± 3.64cm and was significantly higher in WUS patients (43.6 ± 2.44 vs. 39.0 ± 3.32, *p* < 0.001). Daytime sleepiness was significantly more frequent in WUS patients (53.3% vs. 25.8%, *p* = 0.037). Pre‐stroke sleep characteristics demonstrated no statistically significant difference. Logistic regression analysis showed a significantly higher cervical perimeter in patients with WUS (OR = 1.586, 95% CI = 1.21‐2.08, *p* < 0.001).

**Conclusion:** We found an association between WUS and cervical perimeter and daytime sleepiness, suggesting an underdiagnosis of sleep disorders. Understanding WUS physiopathology and risk factors might help optimize prevention strategies.

**Disclosure:** Nothing to disclose.

## EPO‐562

### Intravenous thrombolysis in patients with acute ischemic stroke: Clinical experience from two egyptian centers

#### E. Abed

##### Neurology Department, Al‐Azhar University, Cairo, Egypt

**Background and Aims:** We aimed to identify the barriers that prevent the utilization of rt‐PA in a proper time for reducing delays for revascularization therapies.

**Methods:** This retrospective study was conducted on patients with acute ischemic stroke and were treated with Intravenous rt‐PA within 4.5 hours of the onset. The data was collected from an archiving system of Stroke Units of Al‐Azhar University and ALmaadi Military Hospital between May 2014 and April 2021.

**Results:** A total of 167 patients (mean age of 62.55 ± 9.94 years) and included 94 males (56.2%) were included. 88 patients (52.6%) were treated within 0‐3 hours and 79 patients (47.3%) within 3‐4.5 hours from stroke onset. Lack of knowledge about emergency calling was reported in 46 patients (27.5%). Door‐to‐needle time ≤60 minutes was achieved by only 32.8% (42/128) of patients who arrived within 0‐2 hours of their symptom onset compared to 48.7% (19/39) of those who arrived at the emergency department within 2‐3.5 hours of their symptom onset. The reported complications included symptomatic intracerebral hemorrhage (10.7 %), asymptomatic intracerebral hemorrhage (2.9 %) and hematuria (2.3 %). However, pneumonia due to COVID‐19 infection was the cause of death in 15 patients (8.9%) during the era of the COVID‐19 pandemic.

**Figure:**
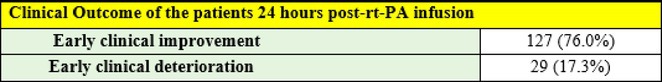
Clinical characterstics

**Figure:**
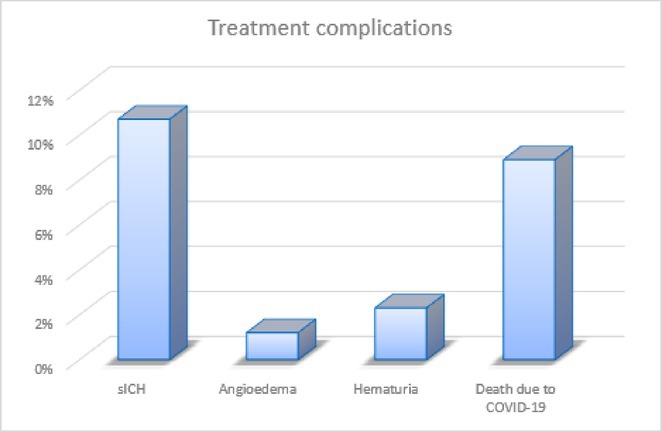
**FIGURE 1** Complications

**Figure:**
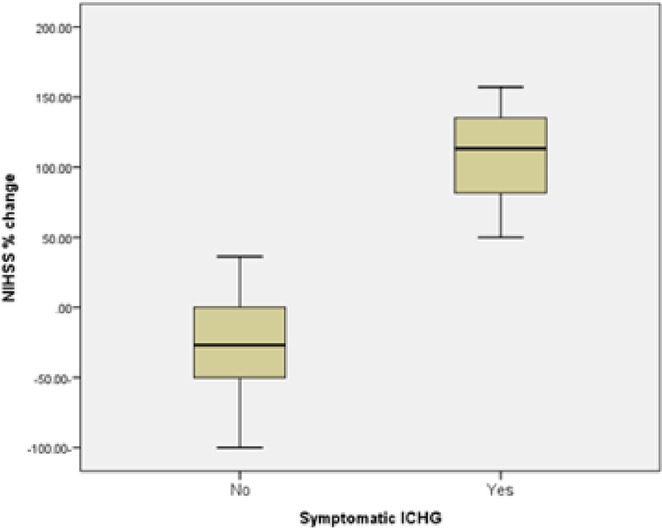
**FIGURE 2** Complication

**Conclusion:** The extremely low number of stroke patients receiving rt‐PA in developing countries was attributed to several barriers including a lack of public awareness, and Inaccessibility to emergency medical services especially restrictions and logistic rules of many hospitals during COVID‐19 pandemic.

**Disclosure:** Nothing to disclose.

## EPO‐563

### Clinical features in the occlusion of artery of Percheron. A challenging diagnosis

#### F. Salazar Hernández; C. Sánchez‐Vizcaíno Buendía; M. Ruiz Perelló; B. Gómez Gozálvez; J. Bermejillo Barrera; A. Savolainen; D. López Segura; D. Vidal Mena; J. Fajardo Sanchís; M. Martínez Zarco; J. García‐Carmona; M. Ortega Ortega

##### Neurology, Hospital Santa Lucía de Cartagena, Murcia, España

**Background and Aims:** Occlusion of the artery of Percheron (AOP) is a rare cause of bilateral thalamic stroke, posing significant diagnostic challenges due to its low incidence and diverse clinical‐radiological presentations. This study aimed to improve the understanding and early recognition of AOP stroke by describing its clinical features, diagnostic approach, and outcomes.

**Methods:** A retrospective review was conducted on patients discharged from the Hospital General Universitario Santa Lucía de Cartagena (2013–2024) with a diagnosis of bilateral thalamic stroke or AOP occlusion confirmed by MRI. Demographic data, clinical presentation, imaging findings, and outcomes were analyzed.

**Results:** The study included 12 patients (66% male, median age 73.5 years). Cardioembolism was the predominant etiology (58.3%), and the main risk factors were atrial fibrillation (50%) and hypertension (33%). Median NIHSS at presentation was 4, with consciousness decline being the most prevalent symptom (91.6%), primarily somnolence (73%). Cognitive‐behavioral impairment (66%) and vertical gaze palsy (50%) were common, although only 33% presented the classic triad. MRI revealed bilateral paramedian and anterior thalamic infarctions with midbrain involvement in 41.6%, correlating with more severe consciousness decline and triad prevalence. CT scans were initially normal in 75% but showed abnormalities on review in 50%. At six months, half of the patients had favorable outcomes (mRS ≤ 2), while 16.7% experienced fatal outcomes.

**Figure:**
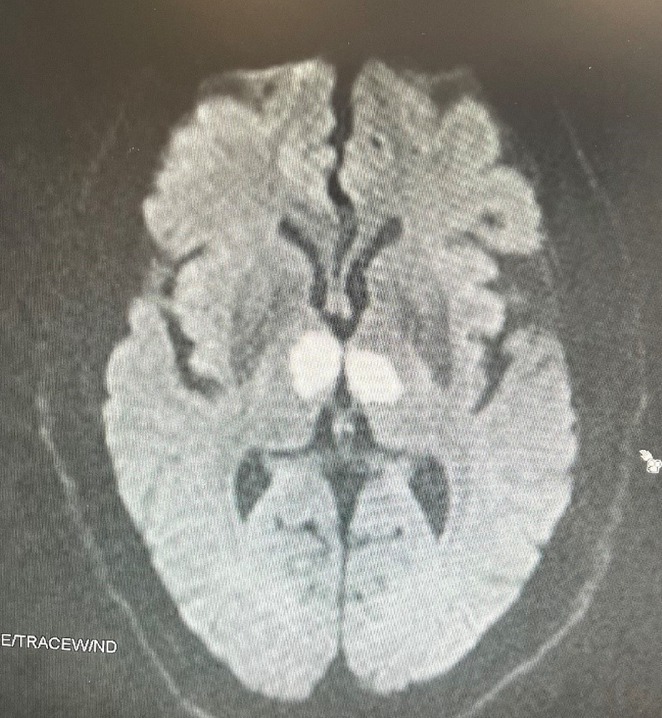
**FIGURE 1** Bilateral paramedian and anterior thalamic infarction

**Figure:**
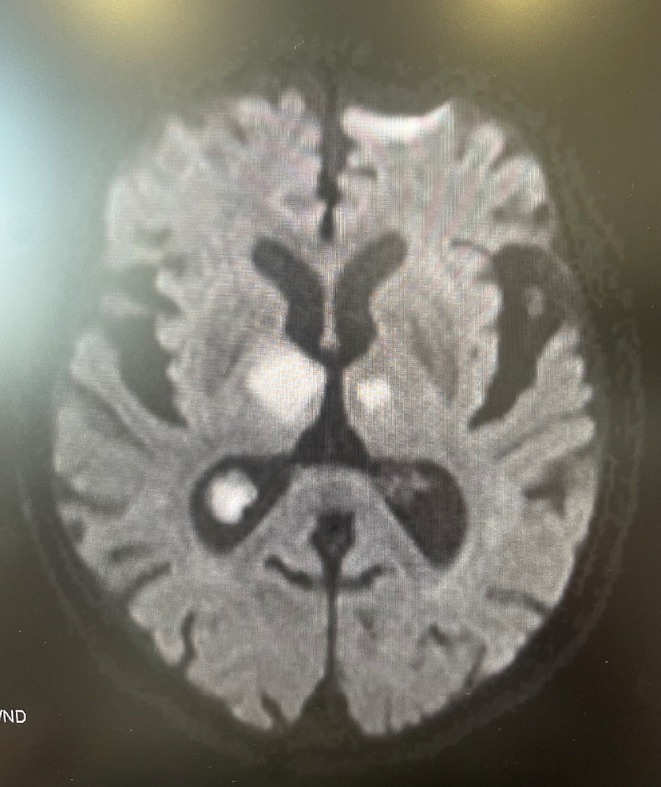
**FIGURE 2** Bilateral paramedian and anterior thalamic infarction 2

**Conclusion:** AOP stroke requires high clinical suspicion, particularly in patients with unexplained consciousness decline and associated neurological deficits. Early MRI is critical for diagnosis, although early CT signs and clinical expertise may guide prompt recognition and management.

**Disclosure:** Nothing to disclose.

## EPO‐564

### Impact of adherence to key performance indicators (KPI) on acute stroke outcome in a tertiary hospital in Tanzania

#### H. Musalam ^1^; T. Kahwa^2^; A. Nagri^3^; R. Shamge^1^; P. Adebayo^2^

##### ^1^Department of Medicine, Neurology Section, Aga Khan Hospital, Dar es Salaam, Tanzania; ^2^Department of Medicine, Neurology Section, Aga Khan University Dar es Salaam, Tanzania; ^3^Department of Medicine, Neurology Section, Muhimbili University of Health and allied Sciences, Dar es Salaam, Tanzania

**Background and Aims:** Adherence to key performance indicators has been associated with shorter lengths of hospital stay, reduced medical complications and lower‐case fatality among stroke patients. This study explored the impact of adherence to key performance indicators for acute stroke management in Aga Khan hospital, Dar es Salaam.

**Methods:** This was a prospective longitudinal observational study. Stroke patients, 18‐years and above admitted via the accident and emergency unit of the hospital between February 2018 and January 2023 were evaluated. Patients’ demographics, stroke types, and ten KPIs were profiled. KPI adherence and its relationship with prolonged hospital stay, in‐hospital mortality and complication were analyzed. Analysis was performed using spss V22. Level of significance was set at *p* ≤ 0.05.

**Results:** 155 acute stroke patients (M: F; 113:42) with mean age (SD) of 57.9 (13.63) were admitted via the ER within the study period. 121(78.1%) had ischemic stroke while 34 (21.9) patients had hemorrhagic stroke. Code stroke was activated in 143 patients (92.3%). 120 (77.45) had a brain CT within 25 minutes of arrival at ER. 80 (51.9%) had complete (100%) compliance with stroke KPIs. Intrahospital complication was noted in 23(14.8%), 7 days case fatality was 4 (2.6%). 46 (29.7%) patients had prolonged hospital stay (> 7 Days). Non‐compliance with KPI was not associated with intrahospital mortality (*p* = 0.948) and prolonged hospital stay (*p* = 0.134) but was associated with intrahospital complications (*p* = 0.006).

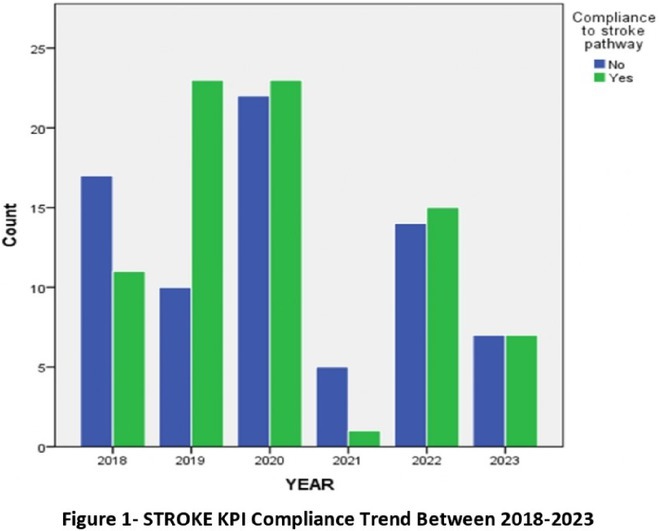

**Conclusion:** Continuous quality measurement, education and improvement in stroke care delivery should be reinforced for optimal stroke outcomes.

**Disclosure:** Nothing to disclose.

## EPO‐565

### Cerebral venous congestion presented with aphasia in a hemodialysis patient

#### H. Jeong ^1^; J. Shin^1^; D. Choi^2^

##### ^1^Department of Neurology, Chungnam National University hospital, Daejeon, Republic of Korea; ^2^Department of Nephrology, Chungnam National University Hospital, Daejeon, Republic of Korea

**Background and Aims:** Cerebral venous congestion results from obstructed venous outflow, like thrombosis or dural arteriovenous fistulas (AVF). We report a rare case of an AVF in a dialysis patient causing reversed internal jugular vein (IJV) flow and aphasia from left hemisphere congestion.

**Methods:** A 54‐year‐old male presented to the emergency department with three days of language impairment. He had been on hemodialysis via an AVF in the left arm for 9 years without complications. Neurological examination revealed reduced speech fluency and impaired comprehension, but no other focal deficits. Brain MRI showed hemosiderosis and disseminated microbleeds in the left hemisphere and left cerebellum, while TOF‐MRA showed prominent flow in in the left transverse sinus and IJV. Left internal carotid artery DSA showed delayed venous drainage through the right transverse sinus into the right IJV, but no dural arteriovenous fistula. Duplex sonography revealed reversed blood flow in the left IJV, which normalized upon AVF compression. These findings led to a diagnosis of venous congestion in the left hemisphere due to IJV reflux.

**Results:** Angiography of the AVF revealed severe stenosis in the left brachiocephalic trunk, with predominant venous drainage into the left IJV. Angioplasty for severe stenosis in the left brachiocephalic trunk resolved the patient's language impairment.

**Conclusion:** Neurological issues in hemodialysis patients typically include stroke or uremic encephalopathy. Reversed IJV flow due to vascular stenosis in the hemodialysis access route resulting in cerebral venous congestion is rare. This condition was successfully managed through angioplasty of the brachiocephalic vein.

**Disclosure:** Nothing to disclose.

## EPO‐566

### Nurse detection of atrial fibrillation in stroke unit patients with suspected cardioembolic stroke on admission

#### J. Han ^1^; B. Kim^2^

##### ^1^Department of Nursing, Asan Medical Center, Seoul, Republic of Korea; ^2^Department of Neurology, Asan Medical Center, Seoul, Republic of Korea

**Background and Aims:** Detecting atrial fibrillation (AF) is crucial for understanding stroke mechanisms and preventing secondary strokes. This study aims to evaluate the effectiveness of nurses' detection of AF in a stroke unit and its influence on the occurrence of subsequent cerebral infarctions.

**Methods:** Acute ischemic stroke patients admitted to ASU and diagnosed as embolic stroke of undetermined source (ESUS) was enrolled. Cardiac workups including cardiac rhythm monitoring during admission to the ASU was performed. Factors associated with detection of AF detection and AF detection by nurse in the ASU were investigated.

**Results:** Among 235 ESUS patients, 114 (48.5%) had AF. Hypertension (OR 1.913), admission heart rate (OR 1.016), and NIHSS score (OR 1.069) were significantly associated with AF detection. Nurses in the ASU identified 24 suspicious AF cases (10.2%), confirming 54.2%. Among them, higher Troponin I levels were associated with nurse detection (*p* = .005). Screening indicators such as premature atrial contraction (92.9% vs. 0.0%) and short runs of atrial tachycardia (28.6% vs. 8.0%, *p* = 0.031) on holter monitoring were significantly more prevalent in the nurse‐detected group. Of the 112 nurses, those with 10+ years of experience detected 48.9% of cardiac abnormalities (*p* = 0.006), with 83.3% confirmed as AF (*p* = .044). Nurse detection led to shorter stays (7.38 vs. 10.12 days, *p* = .030) and lower costs ($217.40 vs. $462.60, *p* = .001), and avoided invasive tests like loop recorders.

**Conclusion:** Nurse detection of AF led to shorter hospital stays, reduced costs, and minimized invasive testing. Experience in neurology improved AF detection rates.

**Disclosure:** Nothing to disclose.

## EPO‐567

### Hypernatremia in the acute ischemic stroke undergoing endovascular thrombectomy and its influence on functional outcomes

#### K. Haruna ^1^; K. Maekawa^1^; N. Nomura^1^; K. Ota^1^; S. Hosoki^1^; N. Ohara^1^; T. Ohta^2^; M. Kawamoto^1^

##### ^1^Department of Neurology, Kobe City Medical Center General Hospital, Japan; ^2^Department of Neurosurgery, Kobe City Medical Center General Hospital, Japan

**Background and Aims:** The potential influence of hypernatremia, a known mortality risk factor in intensive care settings, on functional and neurological outcomes following acute ischemic stroke remains unclear. This study aimed to evaluate the association between hypernatremia and functional and neurological outcomes in patients with acute ischemic stroke.

**Methods:** This retrospective study investigated patients with acute ischemic stroke meeting all the followings: undergoing endovascular thrombectomy at our hospital from October 2018 to December 2023, achieving successful reperfusion, and premorbid modified Rankin Scale (mRS) 0–3. Hypernatremia was defined as peak serum sodium > = 146 mmol/L during hospitalization. Patients were matched by the presence of hypernatremia using propensity‐score matching. Primary outcome was mRS at 3 months.

**Results:** Following propensity score matching (*n* = 152), compared to the non‐hypernatremia group, the hypernatremia group exhibited significantly worse outcomes in mRS at both 3‐month follow‐up (median 4 [IQR 3–5] vs 3 [1–4], *p* < 0.001) and discharge (4 [4–5] vs 3 [2–4], *p* < 0.001), and higher National Institutes of Health Stroke Scale (NIHSS) score at discharge (13 [3–23] vs 4 [1–12], *p* < 0.001). Shift analysis of mRS revealed that hypernatremia was independently associated with worse functional outcomes at 3 months (adjusted odds ratio 3.36 [95% confidence interval 1.86–6.15, *p* < 0.001) and at discharge (2.93 [1.63–5.35], *p* < 0.001).

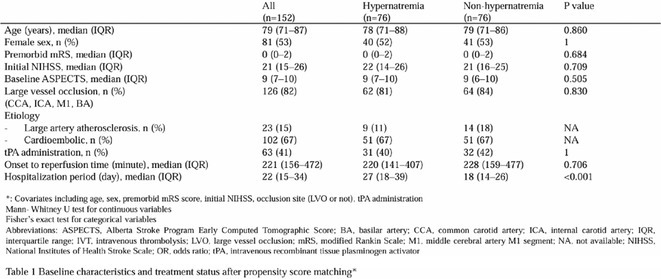

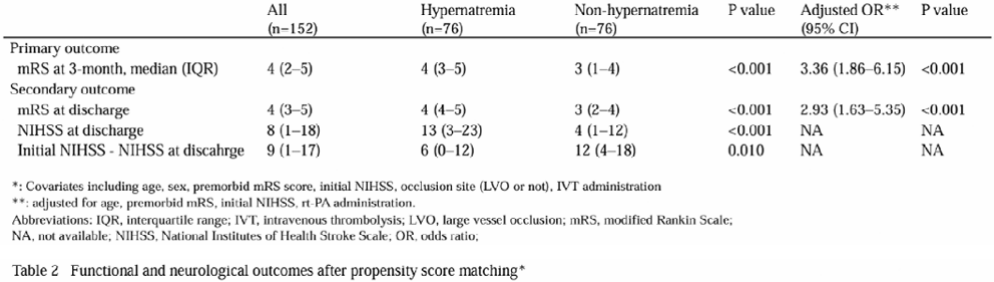

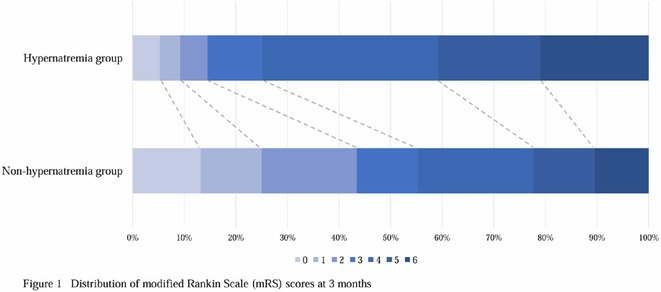

**Conclusion:** These findings indicate that hypernatremia in acute ischemic stroke settings may be associated with poorer functional outcomes and negative influence on neurological status, suggesting the importance of appropriate sodium management in preventing possible secondary brain injury.

**Disclosure:** The authors declare that they have no disclosure.

## EPO‐568

### Exploring results of mechanical thrombectomy in nonagenarians with ischaemic stroke and anterior circulation occlusion

#### M. Delgado‐Romeu; Ó. López‐Lombardía; P. Carbonell‐Fernández; C. Morales‐González; M. Borrell‐Pic; A. Martínez‐Domeño; M. Guasch‐Jiménez; G. Ezcurra‐Díaz; J. Fernández‐Vidal; L. Prats‐Sánchez; A. Ramos‐Pachón; J. Martí‐Fàbregas; P. Camps‐Renom; Á. Lambea‐Gil

##### Stroke Unit, Department of Neurology, Hospital de la Santa Creu i Sant Pau, Barcelona

**Background and Aims:** Data on very elderly patients treated with mechanical thrombectomy (MT) for acute ischemic stroke (AIS) is scarce. Our aim is to explore the safety and efficacy of MT in patients aged ≥90y who suffer AIS due to large vessel occlusion in the anterior circulation (LVO‐AC).

**Methods:** Retrospective observational study of a series of consecutive patients aged ≥90y diagnosed of AIS admitted to a Comprehensive Stroke Centre (2020‐2024). Patients with mRS ≤3, <24h from symptom‐onset and LVO‐AC (TICA/M1/M2/A1/Tandem) were included. MT was compared with best medical treatment (BMT), including intravenous thrombolysis (IVT). The primary efficacy outcome was 3‐month mRS (favourable if ≤3), and safety outcomes included hemorrhagic transformation (PH2/symptomatic) and 3‐month mortality. Analysis was adjusted just for baseline NIHSS to avoid overfitting.

**Results:** We included 31 patients (mean age 92+/‐2.2y, 66.7% women). Median baseline NIHSS was 17 (IQR 8–20). 18 patients received MT and 9 IVT. Patients treated with MT had higher NIHSS (18 [IQR 17‐22] vs.7 [IQR 5‐16]) and more TICA/M1 occlusions (14 vs. 3). Among patients treated with MT, 38.9% achieved 3‐month mRS≤3, compared to 66.7% in those receiving BMT. Adjusting for baseline NIHSS, no statistically significant differences were found (*p* = 0.725). No significant differences were observed in hemorrhagic transformation (MT 5.6%, BMT 0%; *p* = 0.99) or 3‐month mortality (MT 44.4%, BMT 16.7%; *p* = 0.235).

**Conclusion:** In our sample, nonagenarians with LVO‐AC who received MT had worse basal NIHSS and more proximal occlusions. MT was safe, but considering the relevant differences in our cohorts we did not find differences on functional outcomes and further studies are needed.

**Disclosure:** Nothing to disclose.

## EPO‐569

### Central nervous system vasculitis as an etiology of stroke code activation: A case series

#### P. Gutiérrez Bedia; A. Maruri Pérez; J. Obregón Galán; M. Malaret Segurado; J. Ortega Macho; L. Lopez Trashorras; A. Aldaz Burgoa; L. Franco Rubio; P. Abizanda Saro; N. Rodríguez Albacete; J. Egido; C. Gómez‐Escalonilla; P. Simal

##### Neurology Department. Hospital Clínico San Carlos, Madrid, Spain

**Background and Aims:** Central nervous system vasculitis (CNSV) may present as acute ischemic stroke, prompting stroke code activation. However, evidence regarding safety and efficacy of reperfusion therapies—such as intravenous thrombolysis and mechanical thrombectomy—in this context remains limited.

**Methods:** We report a series of five patients with CNSV (two women, aged 35‐63 years old) who triggered stroke code activation, focusing on their initial management, complications related to reperfusion therapy, and clinical outcomes.

**Results:** All patients had an initial NIHSS scores of 2‐6, and ASPECTS score of 10. One received intravenous thrombolysis, two underwent mechanical thrombectomy with intracranial stent placement followed by dual antiplatelet therapy, and the remaining two were managed with dual antiplatelet therapy alone. During hospitalization, four patients received intravenous methylprednisolone followed by immunosuppressive therapy (three with primary CNS angiitis and one with giant cell arteritis). The fifth patient was treated with intravenous penicillin and antiplatelet therapy for meningovascular syphilis. No reperfusion‐related complications were observed. At three‐month follow‐up, three patients achieved functional independence.

**Figure:**
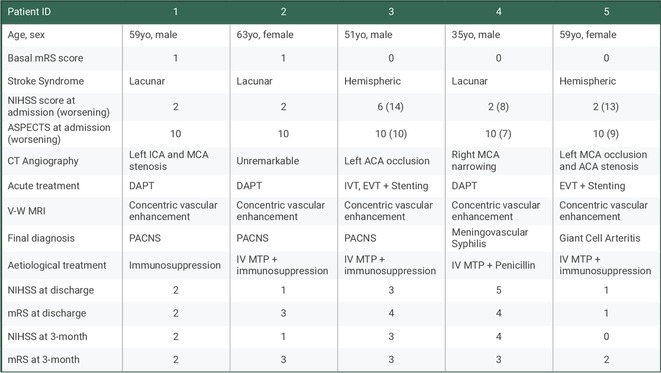
**TABLE 1** This table summarises the characteristics of five patients with CNS vasculitis who triggered stroke code activation. Three patients presented acute worsening during admission; new NIHSS and ASPECTS are reported using brackets.

**Conclusion:** CNSV is a rare cause of stroke code activation. In this series, reperfusion therapies showed a favorable safety profile. However, further studies are necessary to better define their role in CNSV‐related stroke.

**Disclosure:** Nothing to disclose.

## EPO‐570

### Factors associated with carotid atheromatosis in asymptomatic population

#### S. Lozano‐Veiga ^1^; P. Vera‐Andrés^1^; D. Escobar‐Segura^1^; J. Vega^2^; C. Alonso‐Rodríguez^2^; Á. Ximénez‐Carrillo^1^; P. Bugidos‐Martín^1^; C. Albalat‐Sanleon^1^; C. Ramos‐Martín^1^; J. Alonso‐Maroto^1^; E. Cañada‐Lahoz^1^; R. Berbegal‐Serralta^1^; J. Vivancos‐Mora^1^; S. Trillo‐Senín^1^

##### ^1^Neurology, Hospital Universitario de La Princesa, Madrid, Spain; ^2^Radiology, Hospital Universitario de La Princesa, Madrid, Spain

**Background and Aims:** Carotid atherosclerosis is the leading cause of atherothrombotic stroke, yet no screening guidelines exist for asymptomatic individuals. This study aimed to identify variables linked to carotid atheromatosis without stenosis (CAWS) and hemodynamically significant carotid stenosis >50% (HSCS) in an asymptomatic population regarding cerebrovascular and retinal events.

**Methods:** A retrospective observational study was conducted with prospective data collection from patients who underwent Echo‐Doppler of the supraaortic trunks between 2021 and 2022 at a Spanish hospital. Patients with formal indications for the test due to cerebral or retinal ischemic symptoms were excluded. The association of HSCS and CAWS with medical history and analytical parameters was analyzed.

**Results:** Among 261 patients (mean age 70.9 ± 12.4 years, 55.9% women), 16 (6.1%) had HSCS and 157 (60.2%) had CAWS. CAWS was associated with male sex (*p* = 0.012), advanced age (*p* < 0.001), hypertension (*p* = 0.002), diabetes (*p* = 0.036), smoking (*p* = 0.016), ischemic heart disease (*p* = 0.018), small‐vessel cerebrovascular disease (*p* = 0.023), and lower glomerular filtration rate (*p* = 0.007). HSCS was linked to male sex (*p* = 0.040), smoking (*p* = 0.001), ischemic heart disease (*p* < 0.001), peripheral vascular disease (*p* = 0.001), and elevated triglyceride levels (*p* = 0.044). Multivariate analysis independently associated CAWS with male sex (*p* = 0.029), advanced age (*p* < 0.001), and smoking (*p* = 0.007), while HSCS was linked to smoking (*p* = 0.015), ischemic heart disease (*p* = 0.001), and elevated triglycerides (*p* = 0.030).

**Conclusion:** These findings highlight known vascular risk factors for CAWS and HSCS. Future studies should evaluate their potential in developing screening systems for at‐risk populations.

**Disclosure:** Nothing to disclose.

## EPO‐571

### Outcomes predictors in patients with basilar artery occlusion and NIHSS

#### S. Bellavia

##### Università Cattolica del Sacro Cuore, Italy

**Background and Aims:** Treatment of basilar artery occlusions (BAO) is controversial for patients presenting with a NIHSS<10. Current ESO recommendations favor best medical management (BMM) over mechanical thrombectomy (MT) in this subgroup of patients. Nonetheless, recent evidence suggests the potential benefit of MT over BMM. In this retrospective multicenter study, we analyzed a large cohort of BAO patients with a baseline NIHSS<10 that received MT, to identify variables influencing the 3 months outcome.

**Methods:** The prospective database of 10 European stroke centers were screened. Patients older than 18 years old, presenting with BAO and a NIHSS<10, treated with MT, were included. Patients were divided into two groups according to the dichotomized 90‐day modified Rankin Scale score (mRS, 0‐2 vs. 3‐6) and univariate and binary logistic regression (BLR) analysis were performed.

**Results:** 116 patients were collected. Among them, 82 patients achieved a 90‐day mRS of 0‐2. Based on univariate analysis results and clinical reasoning, 9 variables were included in the BLR model. Among them, a distal occlusion site and a higher baseline pcASPECTS were independent predictors of 90‐day mRS of 0‐2. Conversely, higher pre‐event mRS score, atherosclerotic etiology and hemorrhagic complications were independent predictor of poor clinical outcome.

**Conclusion:** Among BAO patient with NIHSS<10, the site of occlusion, the baseline pcASPECTS, the pre‐event mRS and the etiology behind the event appear to be independent predictors of outcome after MT. Future, prospective studies are needed to confirm our observations.

**Disclosure:** Nothing to disclose.

## Movement disorders 6

## EPO‐572

### NXRN1 syndrome: Presentation as myoclonic ataxia

#### A. Gómez González; E. Morales García; M. Gómez Heredia; C. Ortega Hiraldo; F. Pérez Errazquin

##### Hospital Universitario Virgen de la Victoria, Málaga, Spain

**Background and Aims:** The NRXN1 gene encodes a membrane protein belonging to the neurexin family, which is primarily expressed in the brain and plays a fundamental role in synaptic function. This syndrome is characterized by global developmental delay, severe intellectual disability, and the absence of expressive language. Other common features include muscular hypotonia, seizures, autistic behavior, and stereotyped movements.

**Methods:** A case of a 22‐year‐old woman with a history of autism spectrum disorder and developmental delay.

**Results:** We report the case of a female patient who presented at a movement disorder clinic with ataxia and distal myoclonus in the upper limbs. On examination, she exhibited generalized hyperreflexia and a wide‐based gait with an inability to perform tandem walking. MRI findings were normal. Genetic testing revealed that she was a heterozygous carrier of a pathogenic deletion affecting at least intron 18 and exon 19 of the NRXN1 gene (NM_001135659.2). Family segregation studies are currently pending.

**Conclusion:** We continue to expand our understanding of the NRXN1 syndrome spectrum. Further knowledge about its clinical presentation and earlier diagnosis is crucial to improving the quality of life for these patients.

**Disclosure:** Nothing to disclose.

## EPO‐573

### Examination of the frequency and characteristics of impulse control disorders in Wilson's disease

#### A. Nikolic ^1^; V. Markovic^2^; V. Kostic^2^; N. Dragasevic Miskovic^2^; I. Petrovic^2^; M. Jecmenica Lukic^2^; I. Stankovic Tutus^2^; A. Tomic Pesic^2^; N. Kresojevic^2^

##### ^1^Faculty of Medicine, University of Belgrade, Belgrade, Serbia; ^2^Neurology Clinic, Department for Neurodegenerative Diseases, University Clinical Center of Serbia, Belgrade, Serbia

**Background and Aims:** Wilson's disease is a rare hereditary disorder that leads to copper accumulation, primarily in the liver and brain, due to impaired excretion. Impulse control disorder (ICD) is characterized by an inability to resist urges, resulting in repetitive behaviors, often associated with basal ganglia disorders. The aim of our study was to determine the frequency and characteristics of impulse control disorders and other psychiatric symptoms in Wilson's disease.

**Methods:** This observational study included consecutive patients on stable chelation therapy, examined at the Neurology Clinic, UKCS, Belgrade. Patients were assessed using the following scales and questionnaires: Impulsive‐Compulsive Disorders Questionnaire, Beck Depression Inventory, Hamilton Anxiety Scale, Apathy Scale, Impulsivity Scale, and the Obsessive‐Compulsive Symptoms Questionnaire. Demographic data were collected, and disease severity was assessed using the Unified Wilson's Disease Rating Scale. ICD diagnosis was based on established criteria.

**Results:** A total of 32 patients were examined, with an average age of 41.97 ± 10.8 years and disease duration of 16.7 ± 9.6 years. Nine patients (28%) had ICDs, including pathological gambling, binge‐eating, and mixed impulse control disorder. No significant differences were found between groups with and without ICDs regarding disease severity, behavioral, and cognitive features. A logistic regression model for ICD diagnosis was significant (*p* = 0.034), correctly classifying 84% of patients, with higher apathy scores as a significant predictor (OR 0.730, 95% CI 0.548‐0.971, *p* = 0.031).

**Conclusion:** ICDs affect 28% of Wilson's disease patients, with those exhibiting severe apathy at higher risk.

**Disclosure:** Keywords: Wilson's disease; impulse control disorder; impulsivity; apathy.

## EPO‐574

### The role of serotonergic mechanisms in the progression of Parkinson's disease in the population of Uzbekistan

#### D. Pulatova

##### Department of Neurology, Tashkent Medical Academy, Tashkent, Uzbekistan

**Background and Aims:** This study explores the relationship between serotonin levels and both motor and non‐motor symptoms in Parkinson's disease (PD), highlighting serotonergic dysfunction's role in disease progression.

**Methods:** A cohort of 150 PD patients and 150 matched healthy controls were assessed. Motor symptoms were evaluated using the Unified Parkinson's Disease Rating Scale (UPDRS), while non‐motor symptoms, including depression, anxiety, sleep disturbances, and cognitive impairment, were analyzed. Plasma and cerebrospinal fluid (CSF) serotonin levels were measured using high‐performance liquid chromatography (HPLC), and correlations with symptom severity were examined.

**Results:** Serotonin levels in both plasma and CSF were significantly lower in PD patients compared to controls (*p* < 0.05). Reduced serotonin was strongly linked to more severe non‐motor symptoms, particularly depression and anxiety (*p* < 0.01), as well as cognitive impairment (*p* < 0.05). However, no significant association was found between serotonin levels and motor symptom severity.

**Conclusion:** Serotonergic dysfunction in PD is closely associated with non‐motor symptoms, especially mood and cognitive impairments. These findings highlight the need for further research into serotonin‐targeted therapies to improve the management of PD‐related non‐motor symptoms.

**Disclosure:** Nothing to disclose.

## EPO‐575

### Fecal microbiota transplantation for motor symptoms in Parkinson's disease: A systematic review and meta‐analysis

#### M. Otero de Melo dos Reis^1^; E. Bittencurt Thomaz de Assis ^2^; H. Kalaiarasan Swamy^2^; K. Amorim Alves^1^; A. Stepanov Nikolaevich^2^; J. Papaterra Limongi^3^

##### ^1^Universidad Nacional de Rosario, Argentina; ^2^Baltic Federal University, Kaliningrad, Russian Federation; ^3^University of São Paulo, São Paulo, Brazil

**Background and Aims:** Fecal Microbiota Transplantation (FMT) has emerged as a potential therapeutic intervention for managing motor symptoms in Parkinson's Disease (PD). This study aimed to synthesize evidence on the impact of FMT on motor symptom improvement in PD, specifically assessed by the MDS‐UPDRS Part III.

**Methods:** This systematic review and meta‐analysis included 3 studies investigating the efficacy of FMT for motor symptoms in PD. A comprehensive search was conducted across PubMed, Embase, Web of Science, Scopus, and Cochrane databases, adhering to PRISMA guidelines. Eligible studies evaluated FMT's impact on motor symptoms, measured by the MDS‐UPDRS Part III score. The meta‐analyses were performed using Review Manager 4.1. Risk of bias was assessed, and heterogeneity was explored.

**Results:** A total of 143 participants were included, 78 in the FMT group and 65 in the placebo group. FMT significantly improved motor symptoms compared to placebo (MD ‐2.75; 95% CI: ‐4.03, ‐1.47, *p* < 0.0001) in the MDS‐UPDRS Part III score. Low heterogeneity was observed across studies (*I* ^2^ = 5%, *p* = 0.35). Bruggeman et al. contributed the largest weight (85.5%) to the pooled estimate due to its robust sample size and lower variance. Risk of bias was moderate across included studies.

**Figure:**
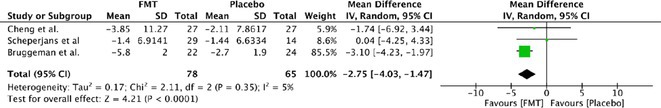
**FIGURE 1** FMT in MDS‐UPDRS Part III Scores

**Conclusion:** FMT shows promise in improving motor symptoms in patients with PD, as evidenced by a significant reduction in MDS‐UPDRS Part III scores. These findings support the therapeutic potential of gut microbiota modulation in PD. However, further studies are needed to validate these results and explore long‐term outcomes.

**Disclosure:** Nothing to disclose.

## EPO‐576

### Serum IGF‐1 and IGF‐2 as biomarkers in idiopathic Parkinson's disease: Correlation with disease stages

#### E. Cakir; A. Özyılmaz; M. Alpay; S. Uyurca

##### Neurology Department, Duzce University/Duzce, Turkey

**Background and Aims:** Idiopathic Parkinson's Disease (IPD) is a progressive neurodegenerative disorder characterized by tremor, rigidity, akinesia, and postural instability. Dysfunction in lysosomal autophagy, involving proteins like IGF‐1(insulin like growth factor) and IGF‐2, contributes to neuroinflammation and neuronal death. Reliable biomarkers for IPD diagnosis and monitoring remain elusive. This study investigates serum IGF‐1 and IGF‐2 levels to evaluate their biomarker potential.

**Methods:** Eighty‐four individuals (43 IPD patients, 41 controls) aged 18–79 were included. Diagnoses followed UK Brain Bank Criteria; severity was assessed with Hoehn & Yahr (H&Y) and UPDRS (Unified Parkinson's Disease Rating Scale). Serum IGF‐1 and IGF‐2 levels were measured using ELISA. Statistical analyses included t‐tests, Mann‐Whitney U, Chi‐square, and Spearman correlation, with *p* < 0.05 considered significant.

**Results:** Serum IGF‐2 levels were significantly higher in patients compared to controls (*p* = 0.006), while IGF‐1 levels showed no significant difference. Both IGF‐1 and IGF‐2 levels displayed negative correlated with disease duration (*p* = 0.044 and *p* = 0.008). Although IGF‐1 and IGF‐2 levels appeared elevated at H&Y stage 2, the differences were not statistically significant. No significant associations were observed between IGF levels and UPDRS scores or medication use.

**Figure:**
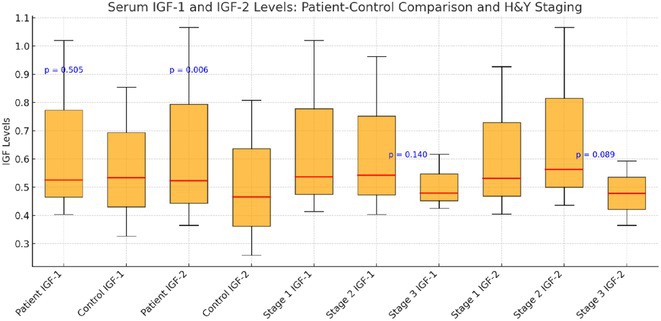
**FIGURE 1** Serum ıgf‐ and ıgf2 levels patients and control comparison and staging.

**Conclusion:** Elevated serum IGF‐2 levels indicate its potential as a biomarker for IPD. These findings contribute to a better understanding of the role of IGF‐1 and IGF‐2 in IPD pathophysiology, suggesting that further multicenter studies are needed to clarify their diagnostic and therapeutic potential.

**Disclosure:** Funding: This study was funded by the Scientific Research Projects Commission of Düzce University under project number 2020.04.03.1108. The funding body had no role in the study design, data collection, analysis, interpretation, or manuscript writing. Conflict of Interest: The authors declare no conflicts of interest regarding this research. There are no financial, personal, or professional relationships that could have influenced the findings of this study. Ethical Approval: This study was conducted in accordance with the principles of the Declaration of Helsinki and was approved by the Ethics Committee of Düzce University Faculty of Medicine (Approval Number: 2023/52). Informed Consent: Written informed consent was obtained from all participants before their inclusion in the study.

## EPO‐577

### Spectral domain and angiography optical coherence tomography in atypical parkinsonisms and Parkinson's disease

#### G. Cennamo; L. Di Maio; G. Riccio; R. Coppola; L. Baratto; A. Giglio; F. Manganelli; G. De Michele; C. Costagliola; A. De Rosa

##### Department of Neurosciences and Reproductive and Odontostomatological Sciences, Federico II University, Naples, Italy

**Background and Aims:** Research recently focused on identifying early and accurate biomarkers to differentiate Parkinson's disease (PD) from other degenerative parkinsonisms, as Progressive Supranuclear Palsy (PSP) and Multiple System Atrophy (MSA). We aimed to investigate changes in the retinal structure and choroidal vascular network (CVN) in PSP and MSA patients in comparison to PD and controls (Ctrl).

**Methods:** Spectral Domain‐Optical Coherence Tomography (SD‐OCT) was used to examine the ganglion cell complex (GCC), retinal nerve fiber layer (RNFL) and subfoveal choroidal thickness, and OCT Angiography (OCTA) for the vessel density (VD) of retinal and CVN assessment.

**Results:** We analyzed 22 eyes from 11 PSP, 14 from 7 MSA, 48 from 24 PD patients, and 50 from 25 Ctrl. In comparison to Ctrl, we observed decreased GCC thickness among PSP (*p* = 0.001) and PD patients (*p* = 0.003), and reduced RNFL thickness in all three groups of patients (PD *p* = 0.043; PSP p<0.001; MSA *p* < 0.001). PD subjects showed lower values in VD of superficial capillary plexus (SCP, *p* = 0.013) and radial peripapillary capillary plexus (RPC, *p* = 0.014) in comparison to Ctrl, whereas MSA and PSP patients did not differ from them. Both groups presented significantly decreased RNFL thickness and higher VD of RPC plexus in comparison to PD group (*p* < 0.001).

**Figure:**
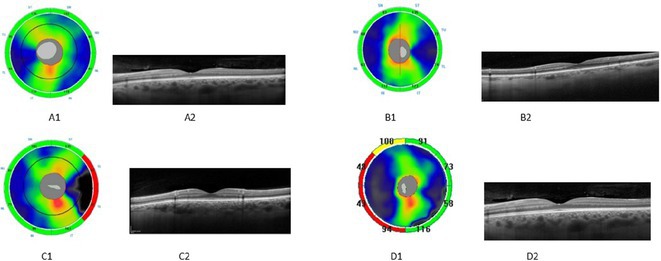
**FIGURE 1** The figure shows normal RNFL thickness in Ctrl (A1), mild reduction in PD (B1), and severe reduction in PSP (C1) and MSA (D1) at SD‐OCT. The subfoveal choroidal thickness was normal in Ctrl, PSP and MSA (A2, C2, D2), and slightly decreased in PD (B2).

**Figure:**
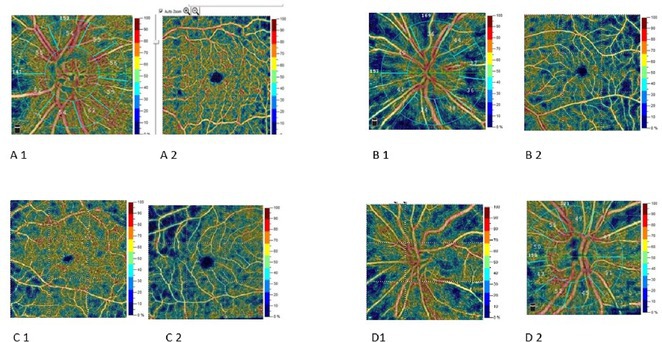
**FIGURE 2** The figure shows preserved vessel density of RPC (A1) and SCP (A2) in a control subject and in PSP and MSA patients (C1, C2; D1, D2), and decreased vessel density of RPC and SCP in a PD patient (B1, B2), at the OCTA exam.

**Conclusion:** Compared to PD, the retina structural damage in PSP and MSA appears to be similar but more severe, whereas the CVN appears to be preserved. Our preliminary results should be confirmed in a larger series of patients to test whether OCTA can be used to differentiate degenerative parkinsonisms early.

**Disclosure:** Nothing to disclose.

## EPO‐578

### Opicapone in very elderly population: Safety overview of post‐marketing data

#### I. Peixoto; D. Lopes; D. Martins; H. Brigas; H. Gama; J. Holenz

##### Research & Development Division, BIAL‐Portela & Cª, S.A., S. Mamede do Coronado, Portugal

**Background and Aims:** Opicapone (OPC) is a catechol‐O‐methyltransferase inhibitor effective as adjunctive therapy to Levodopa for Parkinson's disease patients with motor fluctuations. There is limited clinical trial data in very elderly patients (85 years or older). This analysis primarily aims to evaluate the safety profile in this subgroup, considering data from 8 years of post‐marketing experience.

**Methods:** OPC global safety database was searched for valid post‐marketing reports (spontaneous reports, health authorities, literature reports, non‐interventional studies) from launch (March 2016) to December 2024, in patients with age equal or superior to 85 years old. Expectedness was assessed based on the most recent European Summary of Product Characteristics for OPC.

**Results:** Cumulatively, 354 adverse events (AEs) were collected from 161 valid safety reports: 90 from female patients (55.9%) and 68 from male (42.2%) (unknown: 3). The 2 countries with most reports were Japan (93; 57.8%) and United States of America (37; 23.0%), with less than 10 reports in each of the remaining countries. The 3 most common Preferred Terms (PTs) were “dyskinesia” (22; 6.2%), “hallucination, visual” and “hallucination” (both 14; 4.0%); all 3 considered expected. 135 AEs led to drug withdrawn: “dyskinesia” (12; 8.9 %) and “hallucination, visual” and “nausea” (both 8; 5.9%) were the 3 most common. 250 AEs were non‐serious (70.6%).

**Conclusion:** Most of the AEs were non‐serious. The 3 most reported PTs were considered expected, in line with the known safety profile of OPC. OPC seems to maintain an adequate safety profile in patients with 85 years or older.

**Disclosure:** Supported by Bial‐Portela & Cª, S.A.

## EPO‐579

### Sleep disorders in Parkinson's disease: Bridging nocturnal challenges and daytime impacts

#### l. bouafia; M. Mhiri; Y. Saad; R. Ben dhiaa; N. Gouta; M. Frih‐Ayed

##### Neurology Department, Monastir University Hospital Fattouma Bourguiba, Monastir, Tunisia

**Background and Aims:** Sleep disorders are a prevalent non‐motor symptom in Parkinson's disease (PD) that significantly impair quality of life. This study aims to evaluate sleep quality and the prevalence of excessive daytime sleepiness (EDS) and nocturnal difficulties in PD patients.

**Methods:** We included PD patients diagnosed and followed in the neurology department of Monastir over one year. Disease stages were classified using the Hoehn & Yahr Scale (HYS), while severity was assessed with the Unified Parkinson's Disease Rating Scale (UPDRS). Sleep quality was evaluated using the Pittsburgh Sleep Quality Index (PSQI), Parkinson's Disease Sleep Scale (PDSS), and Epworth Sleepiness Scale (ESS). Polysomnography (PSG) was conducted in 29 patients with significant sleep disturbances (ESS ≥10 and/or PSQI ≥7).

**Results:** Among 100 patients (62% men, 38% women; mean age: 66 ± 10 years), sleep disorders were identified in 69%. The most frequent were parasomnias (66.3%), snoring (62%), insomnia (56%), and hypersomnia (46%). Poor sleep quality was observed in 52% of patients, with a median PSQI of 7 [IQR: 3–10]. Hypersomnia correlated significantly with higher UPDRS motor scores (*p* = 0.002), while no association was found between insomnia and disease severity.

**Figure:**
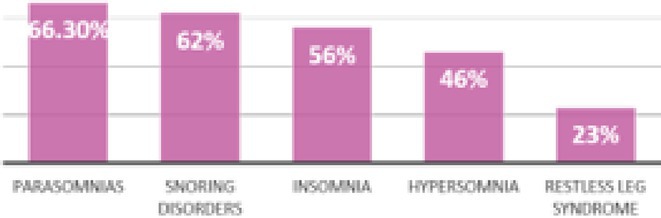
**FIGURE 1** Prevalence and types of sleep disorders in PD patients

**Conclusion:** Sleep disorders are highly prevalent and impactful in PD, emphasizing the importance of systematic screening and targeted management. The correlation between hypersomnia and advanced motor impairment suggests a potential role of disease progression and treatment side effects. Addressing sleep issues in PD could improve patient quality of life and overall disease outcomes.

**Disclosure:** Nothing to disclosure.

## EPO‐580

### Causes of death in anti‐IgLON5 disease: A novel case report and systematic literature review

#### L. Gattermeyer‐Kell ^1^; T. Howischer^1^; S. Hirschbichler^2^; P. Katschnig‐Winter^1^; M. Kögl^1^; S. Franthal^1^; C. Enzinger^1^; R. Höftberger^3^; P. Schwingenschuh^1^

##### ^1^Department of Neurology, Medical University of Graz, Graz, Austria; ^2^Karl Landsteiner University of Health Sciences, Krems, Austria; ^3^Division of Neuropathology and Neurochemistry, Department of Neurology, Medical University of Vienna, Vienna, Austria

**Background and Aims:** Anti‐IgLON5 disease is a neurological disorder characterized by both autoimmune and neurodegenerative pathomechanisms. Reported mortality rates vary between 19 and 59%, with the most common causes of death being sudden death, central hypoventilation, dysphagia, and aspiration. However, the high rate of largely unclear sudden deaths calls for further research in this area.

**Methods:** We performed a systematic literature search on causes of death in anti‐IgLON5 disease following PRISMA guidelines. We also present a new case that was followed up in our clinic until death.

**Results:** Of 258 publications with anti‐IgLON5 disease reported in the literature, 21 publications discussing 61 cases which reported causes of death were included in the analysis. The most common cause of death was death due to complications like pneumonia or falls (36.1 %), followed by sudden death (32.8%), either happening during sleep, wakefulness, or unknown times. Other causes include respiratory, cardiac, and unknown causes. The patient presented here as a novel case report was additionally diagnosed with cardiac amyloidosis and died from a cardiac cause of sudden death.

**Conclusion:** We found a strikingly high number of reports of sudden death among the specified causes of death. A progressive neurodegenerative process in the brain stem causing central hypoventilation is generally assumed as major causative factor. The case first reported here had concomitant cardiac amyloidosis which may raise the question whether unrecognised cardiac causes ‐ which are not routinely screened for in this population ‐ might represent another cause of sudden death, which would have important therapeutic implications.

**Disclosure:** Nothing to disclose.

## EPO‐581

### Parkinson's disease cardiovascular symptoms and the influence of DBS

#### M. Bočková ^1^; R. Panovský^3^; J. Halámek^5^; M. Lamoš^2^; P. Jurák^5^; V. Kincl^4^

##### ^1^Department of Neurology, St. Anne's University Hospital, Masaryk University, Brno, Czechia; ^2^Central European Institute of Technology, Brno, Czechia; ^3^International Clinical Research Center, St. Anne's University Hospital, Faculty of Medicine, Masaryk University, Brno, Czechia; ^4^Department of Internal Medicine/Cardiology, St. Anne's University Hospital, Faculty of Medicine, Masaryk University, Brno, Czechia; ^5^Institute of Scientific Instruments of the Czech Academy of Sciences, Brno, Czechia

**Background and Aims:** The known impairments of the cardiovascular system in Parkinson's disease (PD) are orthostatic hypotension, chronotropic insufficiency, and reduced heart rate variability. Other dysfunctions, i.e. arrhythmias and heart morphology changes, are still the subject of research. Dopaminergic treatment does not influence these symptoms but less is known about the possible impact of deep brain stimulation (DBS).

**Methods:** Thirty PD patients without known cardiac comorbidities underwent bicycle ergometry, electrocardiogram Holter monitoring and CMR (cardiac MRI). Exercise and CMR parameters were compared with controls. The effect of DBS was evaluated, where available.

**Results:** PD patients had lower baseline systolic blood pressure (SBP) (117.8 vs. 128.3 mmHg, *p* < 0.01), peak SBP (155.8 vs. 170.8 mmHg, *p* < 0.05), and lower heart rate increase (49.7 vs. 64.3 beats per minute, *p* < 0.01) during exercise. PD patients had higher indexed left and right ventricular end‐diastolic volumes (68.5 vs. 57.3, *p* = 0.003 and 73.5 vs. 61.0 mL/m^2^, respectively) and also indexed left and right ventricular end‐systolic volumes (44.1 vs. 39.0, *p* = 0.013 and 29.0 vs. 22.0 mL/m^2^, *p* = 0.013). A high prevalence of atrial fibrillation (26.7%) was found. Our pilot data indicate that DBS might positively influence particularly the heart rate variability and the occurrence of arrythmias

**Conclusion:** Our study showed that PD is linked with weaker blood pressure and heart rate reaction during exercise, increased myocardial mass and heart volumes compared to controls, and a high prevalence of atrial fibrillation. DBS might improve some of the cardiac symptoms.

**Disclosure:** Nothing to disclose.

## EPO‐582

### Exploring the impact of diagnosis for people with Parkinson's disease and their caregivers in Tanzania

#### M. Giblin ^1^; N. Fothergill‐Misbah^2^; C. Dotchin^3^; M. Breckons^2^; H. Wilson^1^; M. Dekker^4^; J. Rogathe^4^; S. Urasa^4^; D. Mushi^4^; R. Walker^3^

##### ^1^Faculty of Medical Sciences, Newcastle University, Newcastle upon Tyne, UK; ^2^Population Health Sciences Institute, Newcastle University, Newcastle upon Tyne, UK; ^3^Department of Medicine, North Tyneside General Hospital, Newcastle upon Tyne, UK; ^4^Department of Medicine, Kilimanjaro Christian Medical Centre, Moshi, Tanzania

**Background and Aims:** People with Parkinson's (PwP) and their caregivers in sub‐Saharan Africa (SSA) are under‐represented in research investigating the lived experience of Parkinson's disease (PD). Previous research has highlighted the different challenges that PwP face in Tanzania, including stigmatising perceptions of symptoms and limited access to PD services and medicines. This research aims to understand the impact that a diagnosis has on PwP and their caregivers in Tanzania, to inform future policy and practice to better support people impacted by PD in Tanzania.

**Methods:** Qualitative data were collected using semi‐structured interviews with PwP and caregivers in the Kilimanjaro region of northern Tanzania. Purposeful maximal variation sampling was used to recruit participants. Audio recordings were translated and transcribed and reflexive thematic analysis was applied.

**Results:** Twelve PwP and eight caregivers were interviewed. Data from interviews identified that a diagnosis shifted uncertainty from being driven by the unknown cause of symptoms and complex diagnostic journeys to the challenge of navigating a diagnosis of PD with limited information. Most participants reported acceptance and relief upon receiving a diagnosis. A diagnosis had varied impacts on hope for PwP and their caregivers, however consistent hope manifested through spirituality.

**Conclusion:** Receiving a diagnosis of PD is important to PwP and caregivers in Tanzania. It provides a sense of legitimacy and is a gateway to accessing medicines. Increasing awareness of PD and removing the financial barriers to healthcare would increase diagnoses. This should be accompanied by better availability of specialist neurological and informal services for PwP.

**Disclosure:** Research received funding from Transforming Parkinson's Care in Africa (TraPCAf), a National Institute for Health and Care Research (NIHR) funded project (Award ID NIHR133391).

## EPO‐583

### Friedreich's ataxia patient pathway in Europe

#### J. Vallortigara^1^; J. Greenfield^2^; B. Hunt^2^; C. Reinhard^3^; H. Graessner^3^; D. Hoffman^4^; A. Federico^5^; V. Quoidbach^6^; S. Morris^7^; P. Giunti ^1^

##### ^1^Ataxia Centre, Department of Clinical and Movement Neurosciences, UCL Queen Square Institute of Neurology, Queen Square House, Queen Square, London WC1N 3BG, UK; ^2^Ataxia UK, London, UK; ^3^Centre for Rare Diseases and Institute of Medical Genetics and Applied Genomics, University Hospital Tübingen, Tübingen, Germany; ^4^Takeda Pharmaceuticals, Cambridge, USA; ^5^Department of Medicine, Surgery and Neurosciences, Medical School, University of Siena, Italy; ^6^European Brain Council, Brussels, Belgium; ^7^Primary Care Unit, Department of Public Health and Primary Care, University of Cambridge, Cambridge, UK

**Background and Aims:** Friedreich's ataxia (FA) is a rare progressive and multi systemic neurodegenerative disorder characterized by loss of coordination, typically resulting in loss of ambulation. Cardiomyopathy, diabetes mellitus, and scoliosis are common and serious manifestations of the disease.

**Methods:** This project explores the FA patient pathways and compares their care experiences attending specialist ataxia centres (SAC) compared with care in non–specialist settings. We collected data in the UK, Germany and Italy using a patient survey, to gather information about the diagnosis and the management of the ataxias in specialist and non‐specialist settings.

**Results:** Our population is patients with FA over 16 years old, living in UK (*N* = 27), Germany (*N* = 14) and Italy (*N* = 56) with a confirmed diagnosis of FA. This cohort consists of three groups defining different pathways: currently attending SAC (UK *N* = 3; Germany *N* = 13; Italy *N* = 30), never attended SAC (UK *N* = 12; Germany *N* = 0; Italy *N* = 5), and used to attend SAC (UK *N* = 3; Germany *N* = 1; Italy *N* = 10). We investigated the impact of SAC attendance on symptom management and care delivered to patients and found that some outcomes showed a favourable association with specialist care. All data on the patient pathway will be discussed.

**Conclusion:** This study shows the value of SAC specifically for the management of complex rare conditions like FA. We understand better FA patients’ journey including the burden of the disease and their pathway in the care system. SAC are the best service to deliver new treatment that are FDA approved.

**Disclosure:** Nothing to disclose.

## EPO‐584

### Opsoclonus‐myoclonus‐ataxia syndrome in pregnancy with severe disease course and complete recovery: A case report

#### S. Chowdhury ^1^; L. Vaithianathar^2^

##### ^1^Neurology, Nottingham University Hospitals NHS Foundation Trust, Nottingham, UK; ^2^Neurology, University Hospitals Derby & Burton NHS Foundation Trust, UK

**Background and Aims:** Opsoclonus‐myoclonus‐ataxia syndrome (OMAS) is a rare immune mediated neurological disorder characterized by opsoclonus (rapid, oscillatory eye movement), myoclonic jerks mostly in the face and limbs, cerebellar ataxia, tremors, and encephalopathy. OMAS is rare in adults and exceedingly rarer in pregnancy, as only a few cases in pregnancy have been reported. To the best of our knowledge, this is the fifth presented case of OMAS with a severe disease course and complete reversibility of neurological symptoms in a pregnant woman.

**Methods:** We report and discuss a challenging case of OMAS which presented at 36 weeks gestation in a 38 year old lady. Despite extensive infectious and malignancy evaluation, an underlying etiology was not readily apparent thus we treated presumptively for an idiopathic autoimmune OMAS with high dose intravenous steroids and intravenous immunoglobulin (IVIG).

**Results:** A significant clinical improvement was seen post‐IVIG and upon childbirth through emergency C‐section. The diagnostic workup showed a normal MRI neuroaxis, and positive CSF oligoclonal bands. At 6 months’ follow up, there was evidence of further clinical improvement with a resolution of the movement disorder and opsoclonus.

**Conclusion:** The autoimmune response in OMAS is thought to occur by molecular mimicry with neuronal cell surface antigens from an infective agent. A preceding infection was absent in this case therefore we hypothesize that the immune trigger was the pregnancy.

**Disclosure:** Nothing to disclose.

## EPO‐585

### Using zebrafish model to investigate complex hereditary spastic paraplegia caused by variants in Kennedy pathway genes

#### S. Wang

##### School of Biological Science, University of Manchester, Manchester, UK

**Background and Aims:** Hereditary Spastic Paraplegia (HSP) is a wide and heterogeneous group of inherited degenerative and neurodevelopmental disorders. 19 genes of spastic paraplegia genes (SPG) are involved in membrane phospholipid metabolism. The Kennedy pathway is one of the major biosynthetic sources of PE. EPT1 gene encodes ethanolaminephosphotransferases converting CDP‐ethanolamine to PE in PE branch of the pathway. The bi‐allelic loss of function mutations in EPT1 have been described to cause a complex autosomal recessive HSP, SPG81.

**Methods:** CRISPR‐Cas9 technique was used to create ept1 ex5 knock‐out mutants and crispants. A series of phenotypic characterization, RNA sequencing and lipidomic profiling on these zebrafish models were conducted in the zebrafish model.

**Figure:**
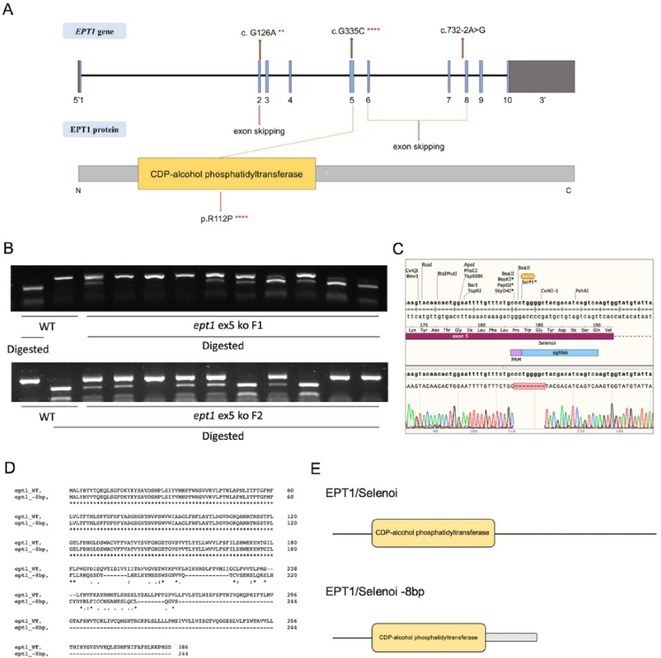
**FIGURE 1** EPT1 gene and crispr guide design

**Results:** CRISPR‐Cas9 technique was used to create ept1 ex5 knock‐out mutants and crispants. A series of phenotypic characterization, RNA sequencing and lipidomic profiling on these zebrafish models were conducted in the zebrafish model.

**Figure:**
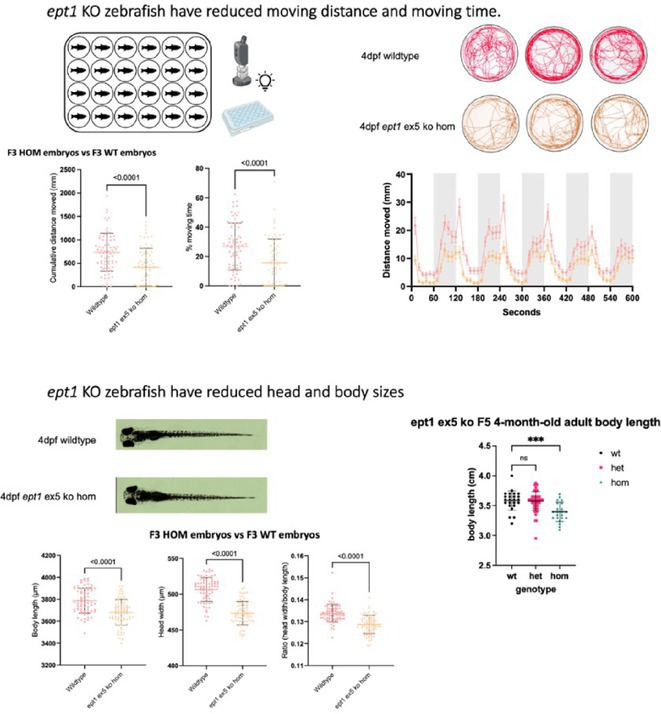
**FIGURE 2** Movement and developmental changes in the knockouts

**Figure:**
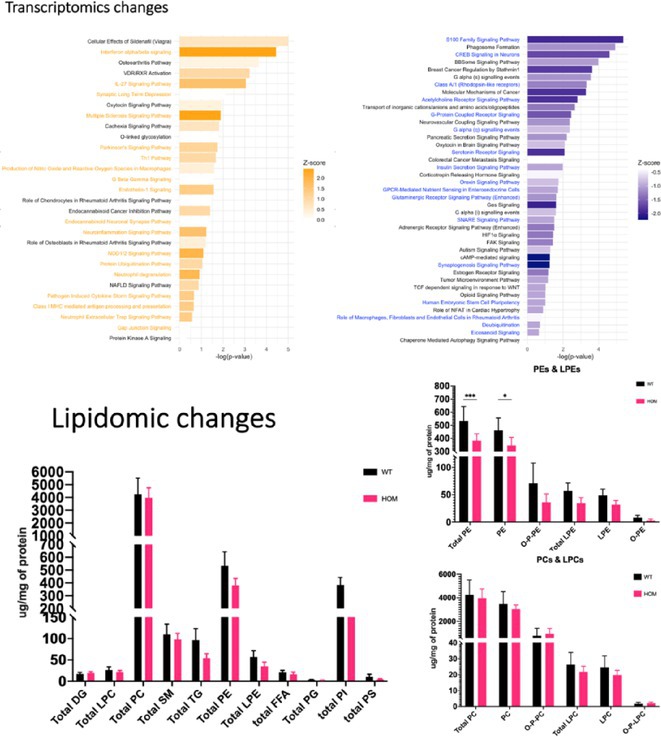
**FIGURE 3** Transcriptomic and Lipidomic changes in the knockouts

**Conclusion:** CRISPR‐Cas9 technique was used to create ept1 ex5 knock‐out mutants and crispants. A series of phenotypic characterization, RNA sequencing and lipidomic profiling on these zebrafish models were conducted in the zebrafish model.

**Disclosure:** Nothing to disclose.

## EPO‐586

### A quantitative investigation of the handwriting kinematics in response to STN‐DBS in Parkinson's disease patients

#### Z. Yavuz; H. Önder; B. Çetin; S. Çomoğlu

##### Neurology Clinic, Etlik City Hospital, Ankara, Turkey

**Background and Aims:** Micrographia is a disabling feature in Parkinson's disease (PD), yet its underlying mechanisms remain unclear. While STN‐DBS is effective in improving motor symptoms, its impact on handwriting kinematics is not well established. We aimed to investigate handwriting changes, particularly micrographia, in PD patients with STN‐DBS under different stimulation conditions.

**Methods:** We prospectively evaluated consecutive PD patients with STN‐DBS who visited our movement disorders center between October and December 2023. Demographic and clinical parameters, along with MDS‐UPDRS‐III scores in stimulation “off” and “on” conditions, were recorded. Handwriting parameters—letter area, writing time, and pressure—were analyzed using a digital graphic tablet (Wacom Intuos Pro‐Large) and an electronic pen. Micrographia‐related metrics were assessed across four stimulation conditions: bilateral, left, right, and no stimulation.

**Results:** Twenty patients (mean age: 56.7 ± 11.4 years, F/M = 7/13) were included. The median motor improvement rate with stimulation was 0.37. No significant differences in handwriting parameters were observed across stimulation conditions. However, longer writing duration (stim‐off) correlated with higher HAM‐A scores (CC = 0.662, *p* = 0.007) and HDRS scores (CC = 0.642, *p* = 0.005), suggesting an influence of anxiety and depression on handwriting performance.

**Conclusion:** STN‐DBS did not induce significant changes in handwriting kinematics, suggesting that micrographia may result from non‐dopaminergic dysfunctions resistant to DBS. These findings provide insights into the pathophysiology of micrographia and the differential effects of DBS on fine motor control.

**Disclosure:** Nothing to disclose.

## Movement disorders 7

## EPO‐587

### Epidemiological and clinical profiles in Huntington's disease: Analysis from Pauls Stradiņš Clinical University Hospital

#### A. Baborikina ^1^; K. Lazsovska^1^; R. Valante^1^; M. Mašinska^2^

##### ^1^Department of Neurology, Pauls Stradiņš Clinical Universtiy Hospital, Riga, Latvia; ^2^Clinic of Medical Genetics and Prenatal Diagnosis, Children's Clinical University Hospital, Riga, Latvia

**Background and Aims:** Huntington's disease (HD) is a genetically determined neurodegenerative disorder. While motor symptoms are hallmark features, non‐motor manifestations—such as cognitive impairment, psychiatric disturbances, and behavioral changes—pose significant challenges in disease management.

**Methods:** Epidemiological data were collected from local HD patients. Functional independence was assessed using the Total Functional Capacity (TFC) scale, and non‐motor symptoms, including psychiatric and cognitive impairments, were evaluated using the Non‐Motor Symptoms Scale (MD‐NMS).

**Results:** This study included 40 patients (47.5% male) observed between 2014 and 2024. Among them, five were asymptomatic, and ten died. The median age was 48 years (range: 23–76 years), and symptom onset occurred at a median age of 40 years (range: 29–47 years). Paternal inheritance was identified in 50% of cases, and maternal inheritance in 10%, 40% left unknown. Motor and psychiatric symptoms were the earliest and most disruptive. The median TFC score was 4 (IQR: 1–7), with 40.9% of patients classified in stage 3. Among 17 participants in the MD‐NMS questionnaire, the mean total score was 156.29 (± 42.90). Depression (mean: 23.59 ± 10.57), cognition (mean: 21.94 ± 11.42), and impulse control (mean: 19.94 ± 9.85) were the most affected domains.

**Conclusion:** Motor and psychiatric symptoms significantly impair HD patients’ functional independence. Non‐motor symptoms further reduce quality of life, highlighting the need for comprehensive management strategies addressing both motor and non‐motor manifestations.

**Disclosure:** ChatGPT was used to enhance readability.

## EPO‐588

### Essential tremor patient motivations and barriers for advanced treatment: From USA to Europe

#### A. Sanchez ^1^; R. Martuscello^2^; K. Gant^2^; C. Ferrer^1^; A. Grinspan^2^

##### ^1^Medical Affairs, Insightec Europe GmbH, Munich, Germany; ^2^Medical Affairs, Insightec, Inc., Miami, USA

**Background and Aims:** Essential tremor (ET) impairs activities of daily living (ADL). Medications are ineffective in up to 50% of patients. Deep brain stimulation (DBS) and MR‐guided focused ultrasound (MRgFUS) are included in advanced therapies guidelines. 6,000 MRgFUS procedures have been performed in Europe, over 22,000 worldwide, surpassing DBS for ET in the USA. To better understand living with tremor and motivations for advanced treatments, we explored patient‐reported data from Insightec's USA‐educator team.

**Methods:** Compliant with US‐Regulations, the Insightec educator team provides information about tremor management, MRgFUS technology, and treatment eligibility to interested patients. From August 2015 to December 2024, patient‐reported data were collected via questionnaires on tremor impact, medication satisfaction, clinical care, and motivations for advanced treatment.

**Results:** Out of 145,286 responders, 63,925 (44%) had an ET formal diagnosis. 98.76% of those interested in MRgFUS have tried medication, 80% being dissatisfied (Figure 1). 80% reported moderate‐severe impact on ADLs. Tremor is mainly managed by general neurologists (46%) or primary care physicians (PCP) (43%), regardless of severity (Figure 2). Barriers to MRgFUS include low tremor severity and travel requirements. Respondents largely denied objection to MRgFUS‐procedural considerations (Figure 3).

**Figure:**
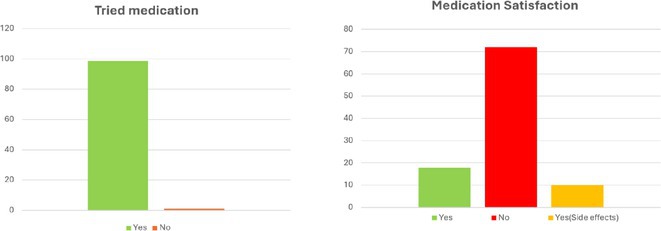
**FIGURE 1** Representation of patients interested in MRgFUS who have tried medication, and their satisfaction with it.

**Figure:**
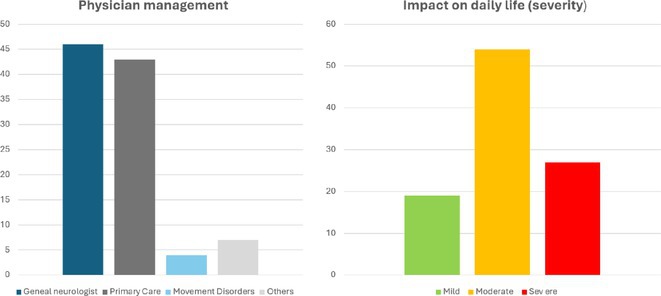
**FIGURE 2** Representation of professionals in charge of patients’ management, and impact on daily life.

**Figure:**
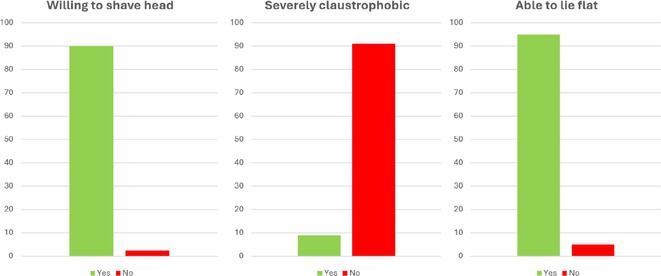
**FIGURE 3** Each of the three categories listed (willing to shave head, being severely claustrophobic or having the ability to lay flat) are potential disqualification questions when considering MRgFUS.

**Conclusion:** This analysis demonstrates that among patients contacting our USA‐educator team, more than 5000 per year are dissatisfied with pharmacotherapies and are interested in advanced treatments. These, in combination with high MRgFUS‐procedural considerations acceptance may explain its growing adoption worldwide. Awareness and knowledge around ET's burden and advanced therapies’ options are critical for neurologists and PCPs. Some barriers remain and further investigation is needed to understand if they translate to European Patients.

**Disclosure:** The authors are all Insightec employees.

## EPO‐589

### Long‐term effectiveness of safinamide vs. rasagiline in Parkinson's disease: A real‐world retrospective study

#### A. Sánchez Rodríguez; J. González Ardura; B. Castaño García

##### Neurology Department, University Hospital of Cabueñes, Gijón, Spain

**Background and Aims:** Parkinson's disease (PD) management involves levodopa and other treatments, including MAO‐B inhibitors like safinamide and rasagiline. This study assesses their real‐world use and effectiveness in Spanish PD patients.

**Methods:** Using IQVIA's Electronic Medical Records (EMR) database, this non‐interventional, longitudinal retrospective study analysed adults treated with N04 ‐ Anti Parkinson drugs between July 2022 and July 2023. 151,264 projected patients were grouped based on rasagiline or safinamide treatment, with a follow‐up period of 24 months before and after their relevant index‐prescription.

**Results:** Most patients were males aged > = 76 years with comorbidities. Rasagiline did not demonstrate improvements in comorbid conditions. However, safinamide exhibited benefits after 24 months, especially in pain (reducing from 25% to 21%). Regarding concomitant medications, safinamide patients experienced reductions in the use of antipsychotics, hypnotics/sedatives, and analgesics by 2%, 6%, and 3%, respectively. Conversely, rasagiline patients increased the use of these medications by 2%, 1%, and 1%, respectively. After 24 months, 91% of safinamide patients remained persistent in their medication use, compared to 85% of rasagiline patients. Additionally, rasagiline patients increased their levodopa dose by 21% over 12 months. In contrast, safinamide patients maintained a steady dose throughout the same follow‐up period, with their dosage varying only 2%. Also, rasagiline treatment led to a 4% increase in Dopamine Agonist Levodopa Equivalent Dose (LED‐DA) while safinamide reduced it in 1%.

**Conclusion:** Safinamide may offer a better treatment option for PD patients, with higher persistence rates, better comorbidity management, stabilized levodopa dosage, and reduced use of additional medications compared to rasagiline.

**Disclosure:** The research conducted in this study received commercial and institutional support from Zambon.

## EPO‐590

### Imaging features and clinical outcomes in patients with ventriculomegaly and gait impairment

#### C. Espinoza Vinces ^1^; I. Avilés‐Olmos^1^; M. Jiménez‐Vázquez^2^; M. Calvo‐Imirizaldu^2^; G. Martí‐Andrés^3^; M. Luquin^1^

##### ^1^Department of Neurology, Clínica Universidad de Navarra, Pamplona, Spain; ^2^Department of Radiology, Clínica Universidad de Navarra, Pamplona, Spain; ^3^Department of Neurology, University Hospital of Navarra, Pamplona, Spain

**Background and Aims:** Ventriculomegaly, a key feature of idiopathic normal pressure hydrocephalus (iNPH), can also occur in neurodegenerative diseases like Alzheimer's disease (AD), Parkinson's disease (PD), dementia with Lewy bodies (DLB), and progressive supranuclear palsy (PSP). Imaging measures such as Evans's index (EI), Callosal angle (CA), and Disproportionately Enlarged Subarachnoid Space Hydrocephalus (DESH) are helpful, but differences in neurodegenerative contexts remain unclear

**Methods:** A retrospective study analyzed 55 patients with gait impairment and 40 matched controls (mean age 74.1 ± 5.4 years). Brain MRIs assessed ventriculomegaly using EI (>0.3) and CA (<90°), alongside DESH scoring. Patients were classified into neurodegeneration (PSP, AD, DLB, PD), iNPH, and controls based on diagnostic criteria, cognitive tests, CSF biomarkers, and ¹^8^F‐Dopa‐PET, with ≥3 years of follow‐up. Kruskal‐Wallis and Bonferroni tests were applied for statistical analysis

**Results:** Of patients with ventriculomegaly, 44% met criteria for PSP, 14% for AD, 4% for DLB, 1 for PD, and 36% for iNPH. The CA was lower in neurodegeneration (ND) patients compared to iNPH and controls (79.9 ± 5.2 vs 87.5 ± 2.0 vs 129.7 ± 3.6, *p* < 0.001). EI was similar between ND and iNPH groups (Table 1). The DESH index was higher in ND patients compared to iNPH and controls (6.5 ± 1.3 vs 4.5 ± 0.9 vs 0.10 ± 0.3, *p* < 0.001).

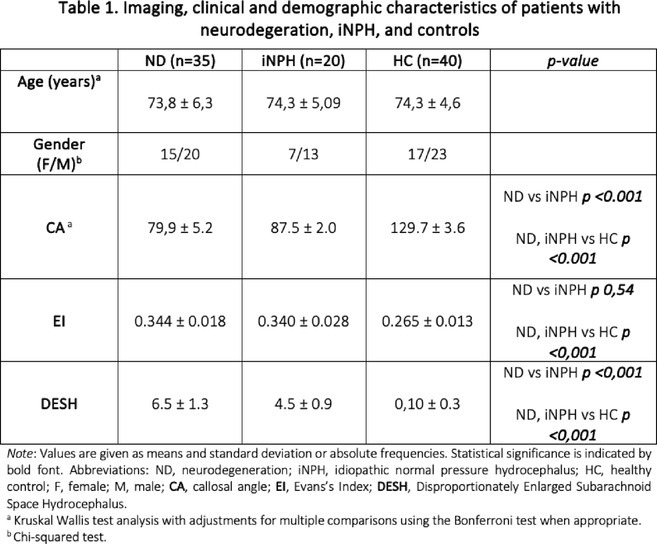

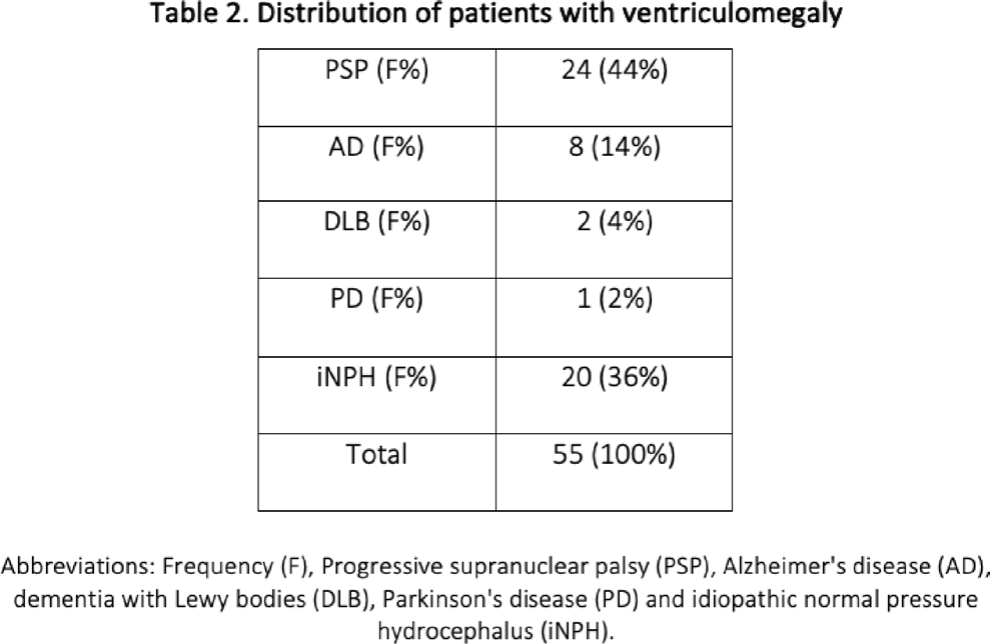

**Conclusion:** Our findings indicate that patients with ventriculomegaly and neurodegeneration have a lower CA and higher DESH. These observations may help in assessing neurodegeneration but should be interpreted cautiously when considering shunting decisions

**Disclosure:** Nothing to disclose.

## EPO‐591

### The relation between upper limbs action tremor asymmetry, midline tremor and gait in essential tremor

#### C. Terravecchia; R. Terranova; A. Messina; D. Caruso; G. Donzuso; C. Cicero; G. Mostile; A. Nicoletti

##### Department of Medical, Surgical Sciences and Advanced Technologies “G.F. Ingrassia”, Section of Neurosciences, University of Catania, Catania, Italy

**Background and Aims:** Upper limbs action tremor represents the clinical hallmark of Essential Tremor (ET), which showed varying degrees of asymmetry. However, the possible role of the upper limbs action tremor asymmetry in the context of the broader ET motor phenotypical spectrum has never been addressed to date. The aim of the present study was to assess the possible relation between upper limbs action tremor asymmetry and other motor aspects which may characterize the ET syndrome.

**Methods:** Clinical tremor scores and instrumental kinematic gait parameters were assessed. An asymmetry index (AI) was computed based on clinical severity of each upper limbs action tremor component. Symmetric (S‐ET, AI = 0) and Asymmetric (A‐ET, AI≠0) patients were defined and compared based on the most asymmetric action tremor component.

**Results:** Thirty‐seven tremor patients [8 pure ET (21.6%) and 29 ET‐plus (78.4%)] were enrolled. Forward outstretched (FO) postural tremor represented the action tremor subtype showing the greater clinical asymmetry across the whole tremor population. No significant differences on action tremor AIs were reported between pure ET and ET‐Plus. Based on FO tremor AI, two patients’ subgroups were defined: A‐ET (*N* = 21, 56.8%) and S‐ET (*N* = 16, 43.2%), the latter showing higher midline tremor severity (i.e. head and voice) and worse instrumental gait parameters.

**Conclusion:** The study highlights the possible role of upper limbs action tremor asymmetry as an adjunctive feature to be considered in ET clinical phenotyping.

**Disclosure:** Nothing to disclose.

## EPO‐592

### Impact of beta glucocerebrosidase gene mutation on quality of life and activities of daily living in Parkinson's disease

#### E. Strujić ^1^; L. Kusić^2^; T. Klepo^2^; A. Poturak^5^; J. Kragujević^4^; V. Pekić^6^; S. Matoša^3^; T. Gilman Kuric^3^; Z. Popović^3^; T. Mirošević Zubonja^3^; S. Tomić^3^

##### ^1^Department of Neurology, University Hospital Centre Osijek, Osijek, Croatia; ^2^Faculty of Medicine, University of J. J. Strossmayer in Osijek, Osijek, Croatia; ^3^Department of Neurology, University Hospital Centre Osijek, Osijek, Croatia; Faculty of Medicine, University of J. J. Strossmayer in Osijek, Osijek, Croatia; ^4^Department of Neurology, University Hospital Centre Osijek; Faculty of Dental Medicine and Health, University of J. J. Strossmayer in Osijek, Osijek, Croatia; ^5^Department of Neurology, University Hospital Centre Osijek, Osijek; Department of Gastroenterology, University Hospital Centre Osijek, Osijek, Croatia; ^6^Department of Neurology, University Hospital Centre Osijek, Osijek, Croatia; Faculty of Medicine, University of J. J. Strossmayer in Osijek, Osijek, Croatia; Faculty of Dental Medicine and Health, University of J. J. Strossmayer in Osijek, Osijek, Croatia

**Background and Aims:** The aim of this study was to investigate the impact of the beta glucocerebrosidase (GBA) gene mutation on quality of life (QoL) and activities of daily living (ADL) in patients with idiopathic Parkinson's disease (PD).

**Methods:** This cross‐sectional study included patients with idiopathic PD. Patient data were obtained from the Movement Disorders Clinic at the University Hospital Centre Osijek. QoL and ADL were assessed using the Movement Disorder Society – Unified Parkinson`s Disease Rating Scale (MDS‐UPDRS) II and Parkison`s Disease Questionnairre‐8 (PDQ‐8) questionnaires through a telephone survey.

**Results:** The study included 51 patients with idiopathic PD (17 female, 34 male) that performed genetic testing for GBA mutation. Genetic testing identified 5 (9.8%) carriers of the heterozygous beta GBA mutation. There was no statistically significant correlation between age and measures of ADL and QoL in both patients with (*p* = 0.94; *p* = 0.55) and without GBA mutation (*p* = 0.13; *p* = 0.89). In patients without GBA mutation, a negative correlation was observed between age and sleep disturbances, sialorrhea and tremor (*p* = 0.01, *p* = 0.005, *p* = 0.04). Episodes of “freezing of gait” were more frequent among carriers of GBA mutation (*p* = 0.05). No significant differences in QoL or ADL were observed between GBA gene mutation carriers and non‐carriers.

**Conclusion:** In this cohort, GBA mutation did not significantly influence QoL or ADL. However, further research with a larger sample size should be done to better evaluate the role of the beta GBA mutation on PD patients' lifestyles.

**Disclosure:** Nothing to disclose.

## EPO‐593

### Real‐world and long‐term safety of MR‐guided focused ultrasound in movement disorders: A comprehensive review

#### G. Frazzetta ^1^; G. Schiff^2^; N. Hemo^2^; C. Ferrer^1^; A. Grinspan^3^; A. Sokolov^2^

##### ^1^Medical Office, Insightec Europe GmbH, Munich, Germany; ^2^Medical Office, Insightec Ltd, Haifa, Israel; ^3^Medical Office, Insightec, Inc., Miami, USA

**Background and Aims:** Magnetic resonance–guided focused ultrasound (MRgFUS) is CE‐approved for unilateral treatment of Parkinson's disease (PD) and both unilateral and staged‐bilateral treatments of essential tremor (ET) and neuropathic pain (NP). To date, over 22,000 MRgFUS procedures have been performed globally, with over 6,000 conducted in Europe. This analysis provides a comprehensive review of safety outcomes reported since MRgFUS approval.

**Methods:** We reviewed long‐term safety data from published literature, including clinical trials, manufacturer‐reported safety data, and user surveys for MRgFUS treatments in ET and PD.

**Results:** In ET pivotal trials, adverse events (AEs) were predominantly mild (74% unilateral, 85% bilateral) with no severe AEs reported. Common AEs included paresthesias (38% unilateral, 33% bilateral) and gait disturbances (36% unilateral, 24% bilateral), often resolving within six months (48% and 52%, respectively). Dysarthria and taste disturbances were more frequent after bilateral MRgFUS (2% vs. 24% and 5% vs. 20%, respectively). Similarly, in unilateral PD treatments, AEs were mostly mild, with paresthesias (0–35%), gait disturbances (3–26%), and dysarthrias (3–19%) typically resolving within six months. Long‐term follow‐ups showed no worsening of AEs. Post‐marketing surveillance of 19,850 procedures reported 193 AEs (1%), primarily paresthesias and gait disturbances.

**Conclusion:** Real‐world data confirm the safety of unilateral and staged‐bilateral MRgFUS, consistent with prior literature and without unexpected events. This analysis reinforces the favorable safety profile of MRgFUS and its role as an advanced therapy for movement disorders.

**Disclosure:** All authors are employees of Insightec.

## EPO‐594

### SL‐START: A multicentre observational study of sublingual apomorphine titration and usage schemes in routine practice

#### J. Kassubek ^1^; P. Themann^2^; C. Maganete^3^; G. Harrison‐Jones^3^; I. Pijuan Jiménez^3^

##### ^1^Department of Neurology, University Hospital Ulm, Ulm, Germany; ^2^Department of Neurology and Parkinson, KTW Klinik am Tharandter Wald GmbH, Halsbruecke, Germany; ^3^BIAL‐Portela & Cª, S.A, Porto, Portugal

**Background and Aims:** People with Parkinson's disease (PD) experiencing motor fluctuations often suffer problems such as morning akinesia, delays in time‐to‐ON, and unpredictable OFF episodes with their levodopa regimen. Apomorphine sublingual film (SL‐APO) is taken on demand for a quick and reliable transition from OFF to ON. SL‐APO requires titration for optimal effect while balancing tolerability. The SL‐START study aims to characterize how SL‐APO is titrated and used in routine practice.

**Methods:** SL‐START is a prospective, observational, 6‐month study of SL‐APO to be conducted at 12 sites across Germany. Patients (≥18y) with a PD diagnosis and experiencing intermittent OFF episodes not sufficiently controlled by oral antiparkinsonian medication are eligible if they have been prescribed SL‐APO for the management of OFF episodes.

**Results:** Patients will be assessed at baseline, during titration, and after 3 and 6 months. The primary objective is to identify the different titration schemes, including number of days and OFF episodes needed to achieve the optimal SL‐APO dose and the percentage of patients who achieve an optimal dose. Details of the SL‐APO regimen, use of domperidone, and other PD medications will be documented. Clinician and Patient Global Impressions of Change and satisfaction with SL‐APO will also be captured. Safety and tolerability will be assessed via adverse event reporting and the number of treatment discontinuations.

**Conclusion:** SL‐START will inform on the factors of SL‐APO titration that are important for patient retention and the titration strategies which work best in real‐world practice.

**Disclosure:** Funded by BIAL.

## EPO‐595

### ROSSINI: Study in progress assessing long‐term foslevodopa/foscarbidopa effectiveness and safety in Parkinson's disease

#### W. Jost^1^; T. Henriksen^2^; L. Bergmann ^3^; P. Kukreja^3^; R. Gupta^3^; M. Shah^3^; J. Parra^3^; S. Caughlin^3^; F. Bergquist^4^

##### ^1^Parkinson‐Klinik Ortenau, Wolfach, Germany; ^2^Movement Disorder Clinic, University Hospital of Bispebjerg, Copenhagen, Denmark; ^3^AbbVie Inc., North Chicago, Illinois, USA; ^4^Department of Pharmacology, University of Gothenburg, Gothenburg, Sweden

**Background and Aims:** Clinical trials demonstrated the efficacy/safety of foslevodopa/foscarbidopa (LDp/CDp) in patients with advanced Parkinson's disease (PD) and uncontrolled motor fluctuations. LDp/CDp has not been studied in real‐world settings. The ROSSINI (Real‐world Outcomes with continuouS Subcutaneous levodopa INfusIon) study is assessing the real‐world effectiveness and safety/tolerability of LDp/CDp and evaluating whether outcomes are similar to those in clinical trials and sustained in the long‐term.

**Methods:** ROSSINI is a prospective, observational, multicountry, dual‐cohort, open‐label study (NCT06107426) conducted in adults with levodopa‐responsive PD. Patients received LDp/CDp per approved local label/reimbursement regulations. Enrolling patients are naive to LDp/CDp (cohort A) or transitioning from LDp/CDp open‐label extension studies (cohort B; NCT04379050, NCT04750226). The primary endpoint is change from baseline to month 36 in “Off” time (measured by Movement Disorder Society‐Unified PD Rating Scale Part IV item 4.3 [cohort A] and Hauser diaries [cohort B]). ROSSINI is also assessing quality of life, motor/nonmotor symptoms, and safety. Effectiveness outcomes are being evaluated during 7‐10 study visits over 3 years. Analyses are being conducted separately for each cohort.

**Results:** ROSSINI will enrol ≈450 patients (cohort A, *n* = 300; cohort B, *n* = 150) across 77 sites in Europe, Australia, and North America. Baseline characteristics of the first 102 patients (all non‐US [US enrolment had not yet started]) enrolled in cohort A were reflective of patients with advanced PD and uncontrolled motor fluctuations (Table).

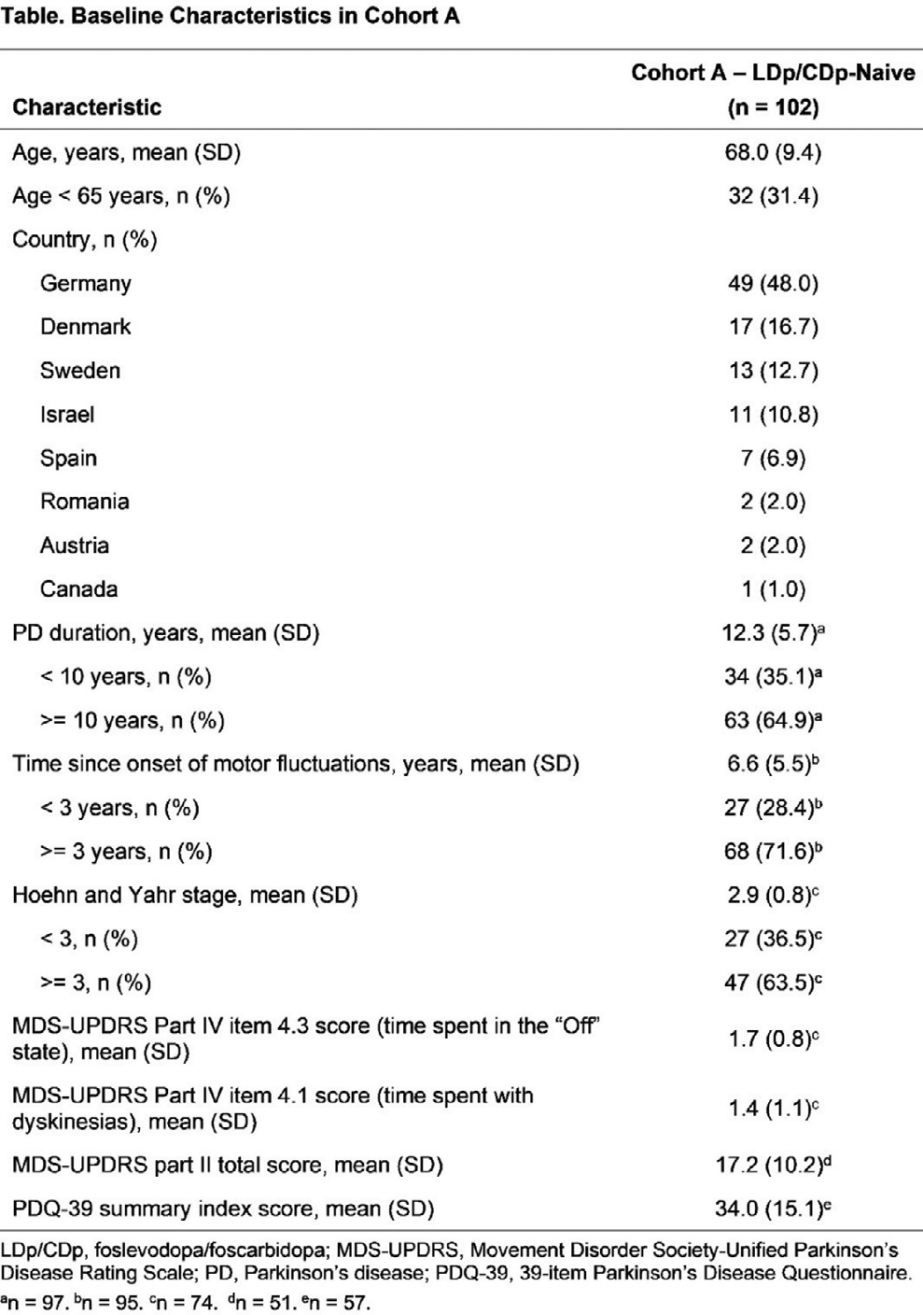

**Conclusion:** Final ROSSINI study results will extend findings from previous studies by providing up to 3 years of data on the real‐world effectiveness and safety/tolerability of LDp/CDp in advanced PD.

**Disclosure:** WHJ serves as an advisor and speaker for AbbVie, Bial, Desitin, Stada, UCB, and Zambon. TH has served as a speaker for AbbVie, Britannia, Convatec, Nordic Infucare, and Lundbeck, and is a primary investigator for the M15‐741 and M15‐737 studies sponsored by AbbVie and the Elegance study sponsored by Brittania. She is also a board member of a data monitoring committee at a study sponsored by Lundbeck. LB, PK, RG, MS, JCP, and SC are full‐time employees of AbbVie, and may hold AbbVie stock and/or stock options. FB has received financial compensation for lectures and advisory services as well as in‐kind donations of PKG reports for clinical studies from GKC, and has received honoraria for lectures and advisory boards from AbbVie.

## EPO‐596

### COMT‐inhibitors clinical experience in early motor fluctuations. Twelve months analysis of REONPARK study

#### L. Lopez‐Manzanares ^1^; J. Garcia‐Caldentey^2^; D. Vilas Rolán^3^; B. Solano Vila^4^; M. Mata Álvarez‐Santullano^5^; J. Blanco Ameijeiras^6^; I. Tegel Ayuela^6^; M. Rodríguez‐de Miguel^6^

##### ^1^Hospital Universitario La Princesa, Madrid, Spain; ^2^Centro Neurológico Oms 42, Palma, Spain; ^3^Hospital Universitario Germans Trias i Pujol, Badalona, Barcelona, Spain; ^4^Hospital Santa Caterina, Girona, Spain; ^5^Hospital Universitario Infanta Sofia, San Sebastián de los Reyes, Universidad Europea de Madrid, Spain; ^6^Laboratorios Bial S.A. Madrid, Spain

**Background and Aims:** In Parkinson's disease (PD), COMT inhibitor (iCOMT) usage extend the elimination half‐life of levodopa and reduce peak‐trough variations, contributing to optimize the dose of levodopa and stabilize plasma levels [1]. Available iCOMT include opicapone, entacapone and tolcapone. Although evidence indicates comparable iCOMT efficacy in reducing off‐time and increasing on‐time in both patients with recent and long‐standing motor fluctuations, some studies suggest a benefit of earlier initiation of iCOMT [2‐6]. The REONPARK study aims to evaluate iCOMT effectiveness and tolerability in alleviating motor complications associated with L‐dopa treatment in PD patients with early motor fluctuations (EMF, signs of end‐of‐dose motor fluctuations within ≤2 years) in clinical practice.

**Methods:** REONPARK study is a Spanish iCOMT registry for PD patients treated with L‐dopa/DDCI and EMF. Presenting here an interim analysis up to 12 months post iCOMT initiation.

**Results:** 89 evaluable patients (mean ± SD: 64.6 ± 10.2 years old; 4.8 ± 3.1 years PD duration; 463.8 ± 191.7 mg/daily L‐dopa; MDS‐UPDRS III: 29 ± 14.5; MDS‐UPDRS IV: 4.5 ± 1.9) initiated iCOMT (98.9% opicapone; 1.1% entacapone). After 3, 6 and 12 months the motor symptoms and motor complications (fluctuations and dyskinesias) were reduced (Table 1). Mean OFF time decreased from 3.8 ± 2.6h at baseline to 1.9 ± 2.2 h (*p* < 0.001) after 12 months (Figure 1). Meanwhile, OFF severity seems to be reduced since the functional impact of the fluctuations was reduced, and patients experiencing no impact increased from 12.4% to 47.7% (Figure 2).

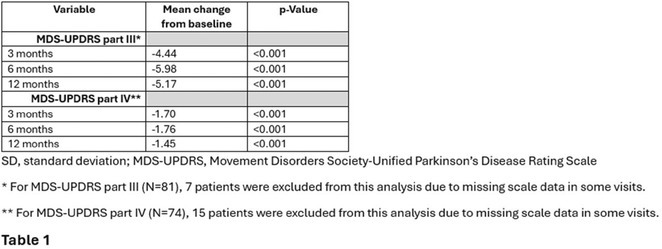

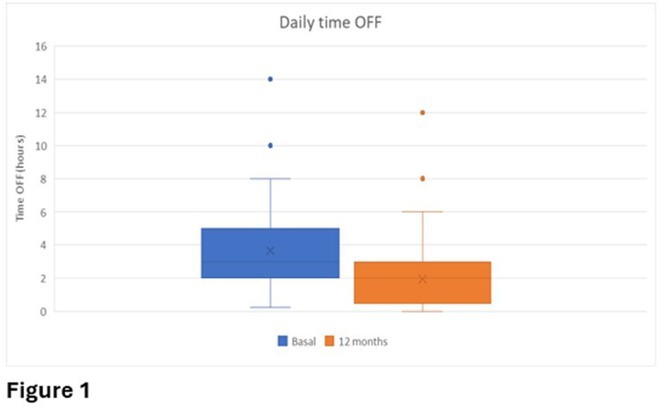

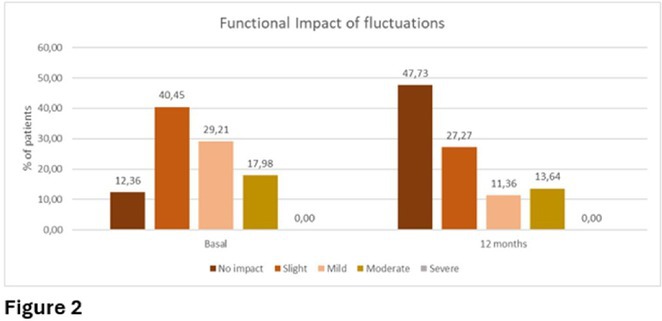

**Conclusion:** iCOMT (mainly opicapone) demonstrated a reduction on OFF time, improving the functional impact of fluctuations after up to 12 months treatment in real‐world practice

**Disclosure:** Study supported by Laboratorios Bial S.A LLM received honoraria in the past 5 years from Abbott, AbbVie, Bial, Biogen, Esteve, Italfarmaco, Orion, STADA and Zambon. JGC received honoraria in the past 5 years from Bial, Zambon, Nutricia and Schwabe. DVR has received honoraria for educational presentations and consulting services in the past 5 years from Bial and Zambon. BSV has received honoraria for educational presentations and consulting services in the past 5 years from Bial and Zambon. MMA has received honoraria for educational presentations and consulting services in the past 5 years from Stada, Abbvie, Orion Pharma, Italfarmaco, Ever, Bial, Zambon, Esteve and Exeltis. JBA, ITA and MRM are employees of Bial.

## EPO‐597

### The non‐motor characteristics of idiopathic Parkinson's disease in northern Tanzania, focusing on mood and cognition

#### M. Prakash ^1^; C. Dotchin^2^; E. Scott^1^; K. Harrington^1^; J. Josephat^3^; M. Dekker^3^; D. Mushi^3^; R. Walker^2^

##### ^1^Newcastle University Medical School, Newcastle Upon Tyne, UK; ^2^Northumbria Healthcare NHS Foundation Trust, Newcastle Upon Tyne, UK; ^3^Neurology, Kilimanjaro Christian Medical Centre, Moshi, Tanzania

**Background and Aims:** This is an observational study set in the Hai district of Northern Tanzania. It aims to describe the non‐motor symptom (NMS) profile of idiopathic Parkinson's disease (PD) in a rural setting in Sub‐Saharan Africa, focusing on the domains of mood and cognition.

**Methods:** A door‐to‐door survey identified PD cases in the district. The following validated assessment tools were used to ascertain the participants’ NMS burden: the Non‐Motor Symptom Questionnaire (NMSQ), Movement Disorder Society Unified PD Rating Scale (MDS‐UPDRS), Identification and Intervention for Dementia in Elderly Africans (IDEA) screening tool for cognitive impairment (CI), and Hospital Anxiety and Depression Scale (HADS). Statistical analysis was done to identify relationships within the dataset.

**Results:** 100% of the cohort (*n* = 29) experienced NMS. The MDS‐UPDRS cohort mean (+ SD) for total NMS score was 15.1 (+ 1.7) out of 52, and 15 people had CI. MDS‐UPDRS data highlighted depressed (*n* = 12), anxious (*n* = 13), and apathetic (*n* = 11) moods. The IDEA screen picked up 9 participants with signs of dementia. Participants experienced an average of 10 out of 30 symptoms within the NMSQ. 13 (48%) participants had borderline or abnormal results for both anxiety and depression in the HADS. There was a 100% treatment gap for mood and cognitive symptoms.

**Conclusion:** The study underscores a high prevalence of NMS in this cohort. PD patients in Tanzania are commonly affected by mood and cognitive symptoms, which are strongly associated with one another. Further research with a larger cohort will strengthen these conclusions and emphasise the need to develop accessible NMS therapies.

**Disclosure:** The research received funding from Transforming Parkinson's Care in Africa, a National Institute for Health and Care Research funded project (Award ID NIHR133391).

## EPO‐598

### Glymphatic dysfunction, cognitive and sleep disorders in patients with Parkinson's disease

#### O. Alenikova; O. Zmachynskaya; L. Parkhach; M. Dymkovskaya; N. Alenikov

##### Republican Research and Clinical Center of Neurology and Neurosurgery, Minsk, Belarus

**Background and Aims:** Non‐motor symptoms, including sleep and cognitive disturbances, are common in Parkinson's disease (PD). Sleep maintains brain homeostasis and promotes waste clearance via the glymphatic system (GS). Sleep fragmentation with reduced slow wave sleep (SWS) impairs cognition and promotes neurodegenerative changes. We investigated glymphatic dysfunction in relation to the brain bioelectrical activity during sleep, cognitive abilities and MRI structural alteration in PD.

**Methods:** Participants included 26 non‐demented PD patients aged 45‐60 years and 30 age‐matched controls. Polysomnography with quantitative EEG spectral power analysis was used for sleep assessment. Diffusion tensor image analysis with perivascular space (DTI‐ALPS) index was calculated to investigate the GS. We also used MRI voxel‐based morphometry and neuropsychological tests.

**Results:** Objective and subjective sleep disorders indicators were worse in the PDpatient. Additionally, they showed SWA reductions with greater decrease in frontal regions during NREM compared to controls (figure). A number of PDpatients showed mild cognitive impairment (MCI) and worse neuropsychological test scores. Along with the reduction in the brain structures volume related to the frontostriatal and posterior‐cortical MCI subtype, PD patients had a reduced DTI‐ALPS index compared to controls (table1). A relationship was found between DTI‐ALPS index and frontal delta spectral power during N3, subjective sleep disturbances, as well as cognitive function in PD patients. Glymphatic influx correlates positively with decreased volume in the brain structures involved in executive function and memory formation (table2).

**Figure:**
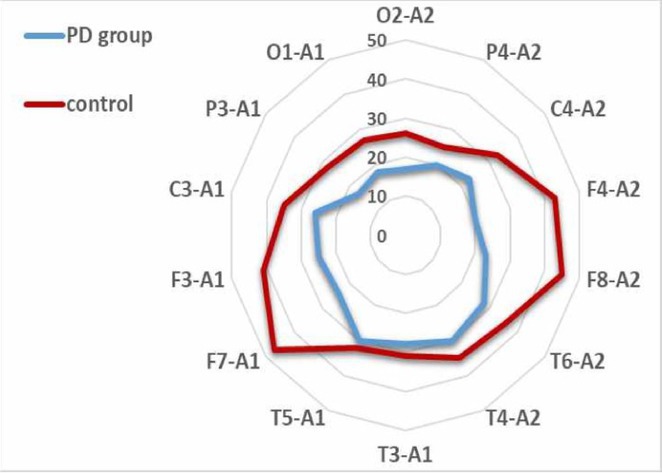
**FIGURE 1** Distribution of relative delta‐band power (SWA 1‐4 Hz (%) between groups during slow wave sleep.

**Figure:**
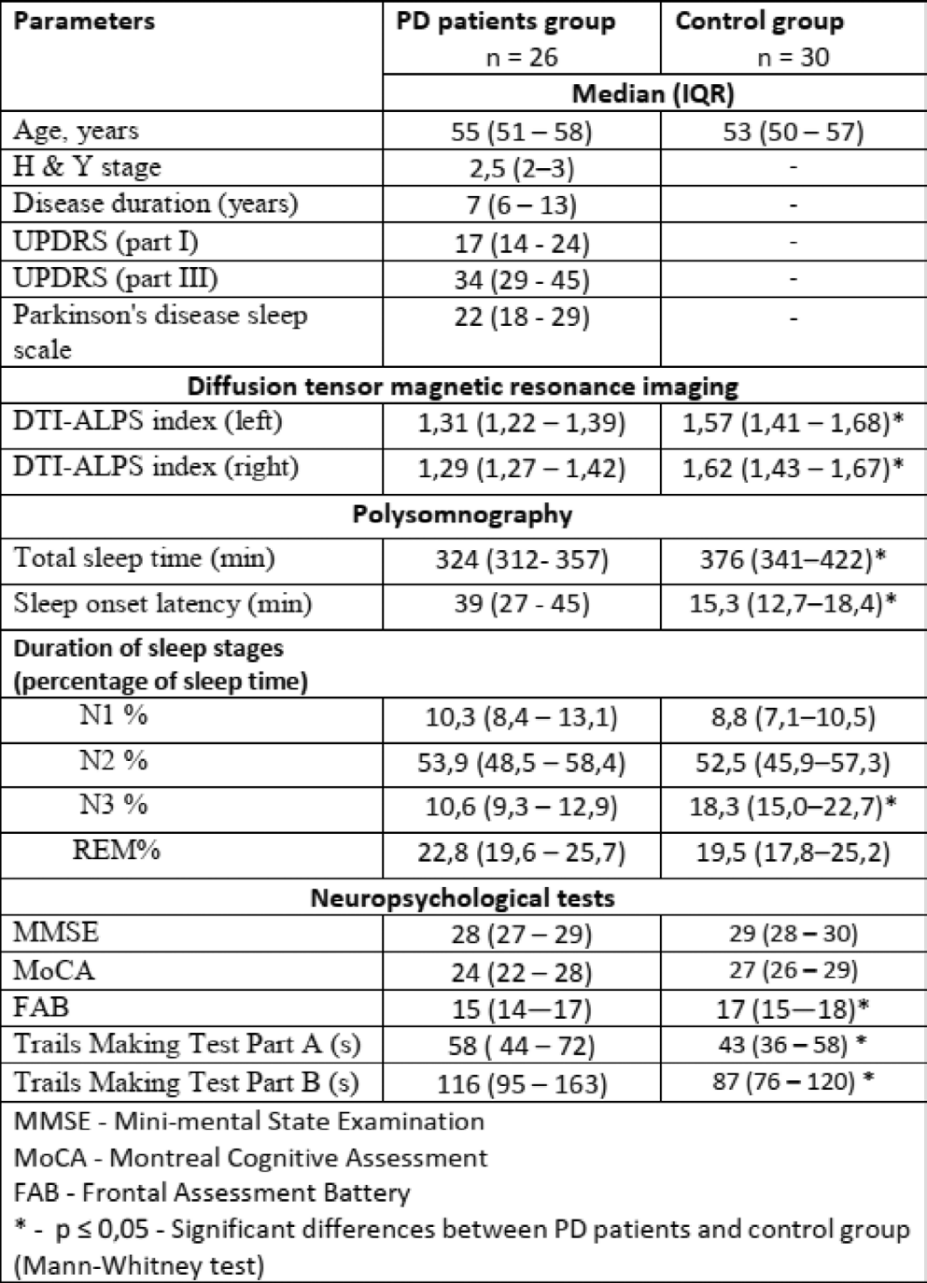
**TABLE 1** Comparative assessment of the PD patients group and the controls.

**Figure:**
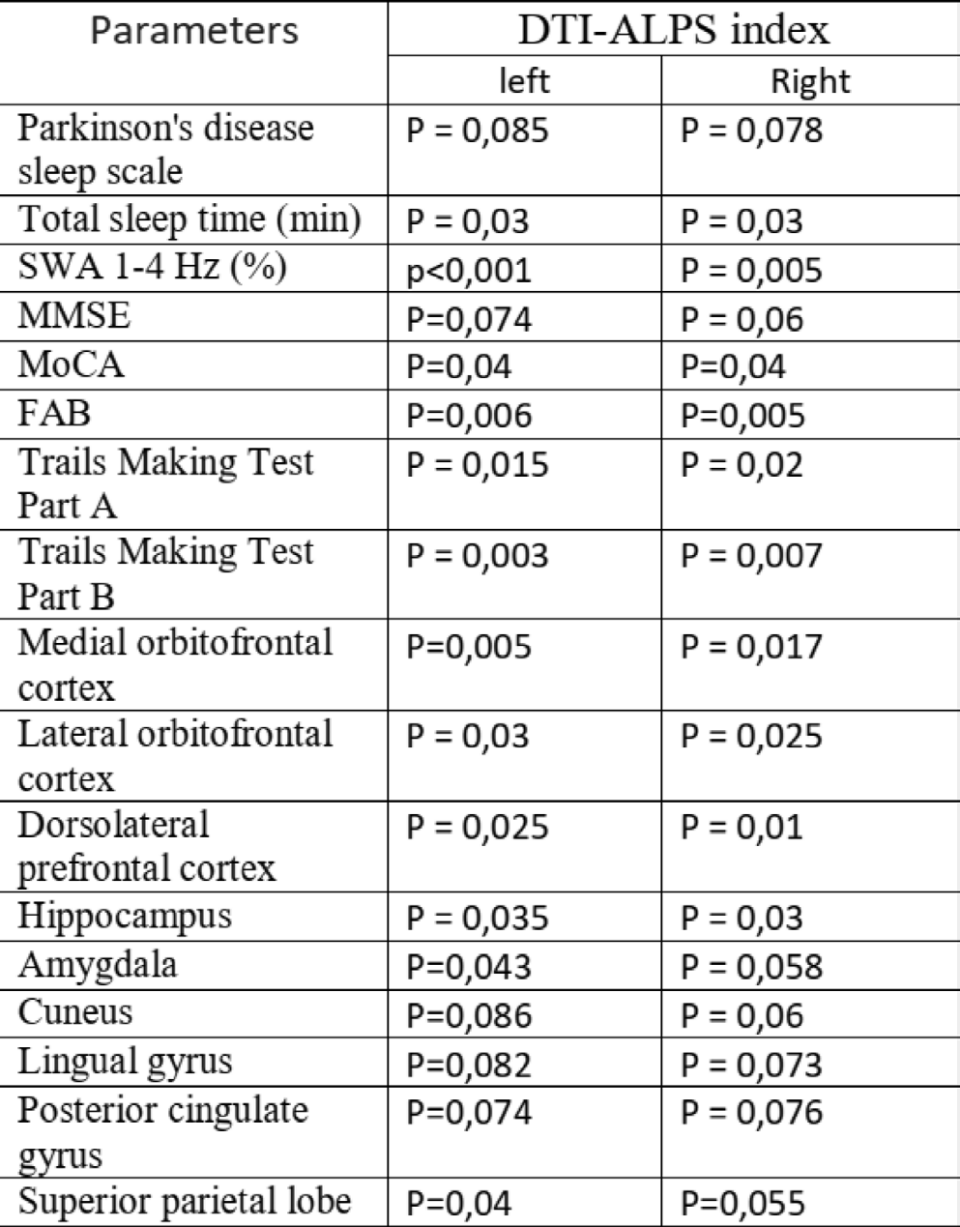
**TABLE 2** Statistical significance of the relationship between neuropsychological tests, sleep parameters, MRI voxel‐based morphometry data and DTI‐ALPS index according to Spearman correlation analysis.

**Conclusion:** The links between glymphatic clearance, sleep architecture and cognitive dysfunction are of interest and provide a basis for developing therapeutic strategies to prevent disease progression.

**Disclosure:** Nothing to disclose.

## EPO‐599

### Opicapone improves end‐of‐dose neuropsychiatric fluctuations in patients with Parkinson's disease

#### R. De Fiores ^1^; I. Martino^1^; A. Quattrone^2^; B. Vescio^3^; G. Arabia^1^; A. Gambardella^1^; M. Morelli^1^

##### ^1^Institute of Neurology, Department of Medical and Surgical Sciences, Magna Graecia University, Catanzaro, Italy; ^2^Neuroscience Research Center, Magna Graecia University, Catanzaro, Italy; ^3^Biotecnomed S.C.aR.L. Catanzaro, Italy

**Background and Aims:** Non‐motor fluctuations (NMF) represent one of the main complications that patients with Parkinson's disease (PD) may experience during long‐term levodopa treatment. Opicapone (OPC), a COMT inhibitor indicated for end‐of‐dose motor fluctuations (MF), has not yet been extensively investigated for the management of NMF. We aim to evaluate the efficacy of OPC on end‐of‐dose neuropsychiatric fluctuations, the most frequent and severe NMF.

**Methods:** We assessed 15 PD patients (8 males/7 females; mean age ± SD: 69.5 ± 7.1 years; disease duration: 7.2 ± 1.7 years) with end‐of‐dose MF and NMF, confirmed by 19‐item Wearing‐Off Questionnaire (WOQ‐19). For each patient, we identify the first end‐of‐dose deterioration period through MDS‐UPDRS‐III administered every 30 minutes over two consecutive days. On the third day, a comprehensive clinical and neuropsychological battery was administered during this designated period. Subsequently, OPC was prescribed. After 6 months, patients were re‐evaluated using the same baseline assessments during the same end‐of‐dose period.

**Results:** At 6‐month follow‐up, PD patients showed a significant improvement in the following tests: WOQ‐19 (*p* < 0.001), total MDS‐UPDRS and each of its four parts (*p* < 0.001), NMSS scores (*p* < 0.001), neuropsychological tests assessing executive functions/attention (Weigl's, *p* < 0.001; FAS fluency, *p* < 0.001; STROOP, *p* = 0.003) and mood related symptoms (BDI‐II, HAM‐A; both *p* < 0.001). In contrast, there were no significant differences in the scores of Visual Search (*p* = 0.033), RAVLT‐I (*p* = 0.225), and RAVLT‐D (*p* = 0.136).

**Conclusion:** OPC improved end‐of‐dose fluctuations in anxiety, depression, and executive functions/attention, where dopamine plays a critical role, while less dopamine‐dependent domains, such as memory and visuospatial abilities, showed no significant changes.

**Disclosure:** Nothing to disclose.

## EPO‐600

### Neurophysiological and clinical effect of botulinum toxin in essential blepharospasm

#### Y. Tereshko; S. Versace; C. Dalla Torre; G. Merlino; M. Valente; C. Lettieri

##### University Hospital Santa Maria della Misericordia, Clinical Neurology department, Udine, Italy

**Background and Aims:** Botulinum toxin Type A might exert a central effect in distonias; however, the literature regarding its possible central effect in this setting is scarce. This study aims to investigate the central effect of this drug by the means of the amplitude ratio obtained with blink reflex recovery cycle before and after the treatment in patients affected by blepharospasm.

**Methods:** We treated 13 patients affected by essential blepharospasm. We performed a baseline (before botulinum toxin type A therapy) and a final evaluation 5 weeks after treatment. During the baseline and final evaluation, 3 clinical scales were administered (Blepharospasm Disability Scale, Jankovic Rating Scale, and Blepharospasm Severity Scale) and a R2 blink reflex recovery cycle was performed on both right and left side, using interstimulus intervals of 200, 300, 500 and 1.000 milliseconds. The amplitude ratios between the unconditioned R2 and conditioned R2 were calculated for each interval.

**Results:** The amplitude ratio of the blink reflex recovery cycle, when compared to the baseline, was significantly reduced after the treatment for every interstimulus interval applied. This data was confirmed considering both eyes and each eye individually. There was also a significant improvement in the scales used to quantify the therapeutic clinical outcome.

**Figure:**
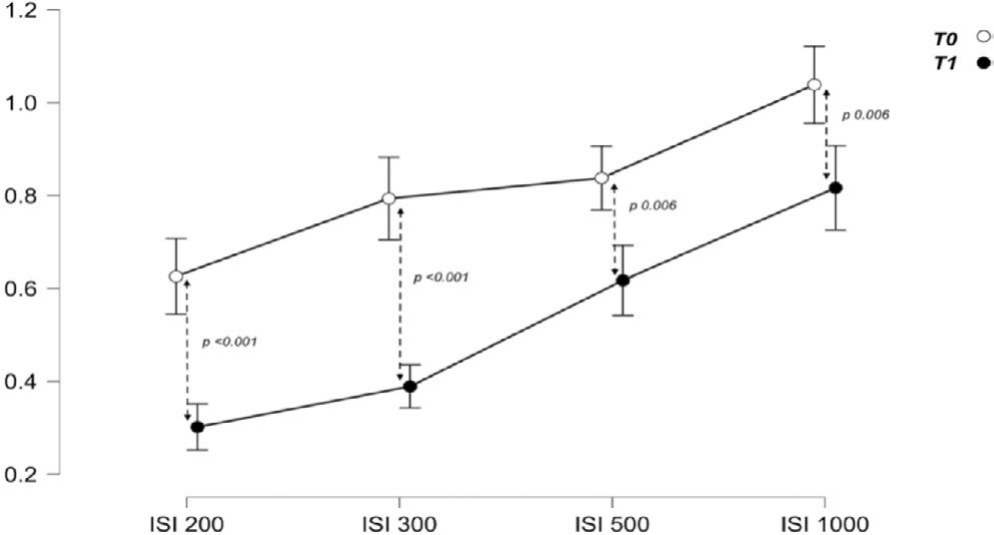
**FIGURE 1** shows the plot line (with 95% confidence intervals) of the amplitude ratio at different paired pulses ISI (200 ms, 300 ms, 500 ms, and 1000 ms), considering both eyes. The RM ANOVA analysis confirmed a significant increase in the amplitude ratio with the inc.

**Conclusion:** The significant reduction of the amplitude ratios observed after the treatment with botulinum toxin type A could imply a central effect of this drug on reducing the hyperexcitability of the circuits underlying this form of dystonia.

**Disclosure:** Nothing to disclose.

## Movement disorders 8

## EPO‐601

### Psychiatric comorbidity in patients with dystonia – Data from a retrospective analysis of adult patients in Bulgaria

#### S. Shtereva ^2^; V. Todorov^2^; A. Atanasov^1^; N. Kalaydzhiev^1^; T. Evdenova^1^; D. Gadzhalova^1^; H. Papazova^1^; I. Milanov^2^; D. Bogdanova^2^

##### ^1^University Hospital for Active Treatment in Neurology and Psychiatry Sv. Naum, Sofia, Bulgaria; ^2^Medical University, Sofia, Bulgaria

**Background and Aims:** Dystonia is the third most common movement disorder worldwide. The aim of our study was to analyze the non‐motor psychiatric symptoms of an adult cohort of patients with dystonia in our hospital.

**Methods:** Data were retrospectively collected from all patients with dystonia hospitalized in University Hospital for Active Treatment in Neurology and Psychiatry, Sofia, Bulgaria in 2024. Psychiatric comorbidity, sex, age at onset, body distribution, disability, coexistence of tremor, duration of the disease and type of treatment were analyzed. Patients with dystonia with different etiologies were included – idiopathic, tardive, functional dystonia and dystonia in cerebral atrophy and cerebral small vessel disease.

**Results:** A total of 60 patients were hospitalized with dystonia in our hospital. Almost half of the patients ‐ 25 (41.7%) were diagnosed with additional psychiatric disorder. The most common comorbidity was mixed anxiety‐depressive disorder (7), followed by schizophrenia (2), bipolar (2), panic (2) and major depressive (2) disorder. One patient had a somatic symptom disorder. Nine of the patients were prescribed a psychiatric therapy from a specialist but either refused to show or didn’t bring the official medical documentation. Ten (6%) of the patients had a tardive dystonia and five patients were diagnosed with functional dystonia.

**Conclusion:** Psychiatric disorders are highly prevalent in patients with dystonia. They appear to be under‐recognized and undertreated. Complex approach with the participation of a neurologist and a psychiatrist is necessary.

**Disclosure:** Nothing to disclose.

## EPO‐602

### Impact of acupuncture on lowering accidental injury in patients with Parkinson's disease: A nationwide cohort study

#### C. Lin; H. Chou; M. Wang; S. Lin

##### Department of Chinese Medicine, Taipei City Hospital, Renai Branch, Taipei, Taiwan

**Background and Aims:** Patients with Parkinson's disease (PD) face a heightened risk of accidental injuries due to motor and non‐motor symptoms that impair mobility and balance. Acupuncture, known for improving balance and gait, has not been thoroughly studied for its role in injury prevention among PD patients. This study aimed to evaluate the impact of acupuncture on reducing accidental injury risk in this population.

**Methods:** A nationwide retrospective cohort study was conducted using Taiwan's National Health Insurance Research Database, including patients newly diagnosed with PD between 2001 and 2012. Propensity score matching was employed to balance demographics, comorbidities, and medication usage between patients who received acupuncture and those who did not. The incidence of accidental injuries was assessed, and adjusted hazard ratios (HRs) were calculated using Cox proportional hazards models.

**Results:** Among 24,467 PD patients, 32% underwent acupuncture. The acupuncture group experienced a lower incidence of accidental injuries (15.2%) compared to the non‐acupuncture group (17.1%). Adjusted analyses indicated that acupuncture was associated with a 33% reduced risk of injury (adjusted HR: 0.67; 95% CI: 0.42–0.85). The protective effect was consistent across most subgroups, except for patients aged under 60, those in less urbanized areas, or those with high insurance coverage.

**Figure:**
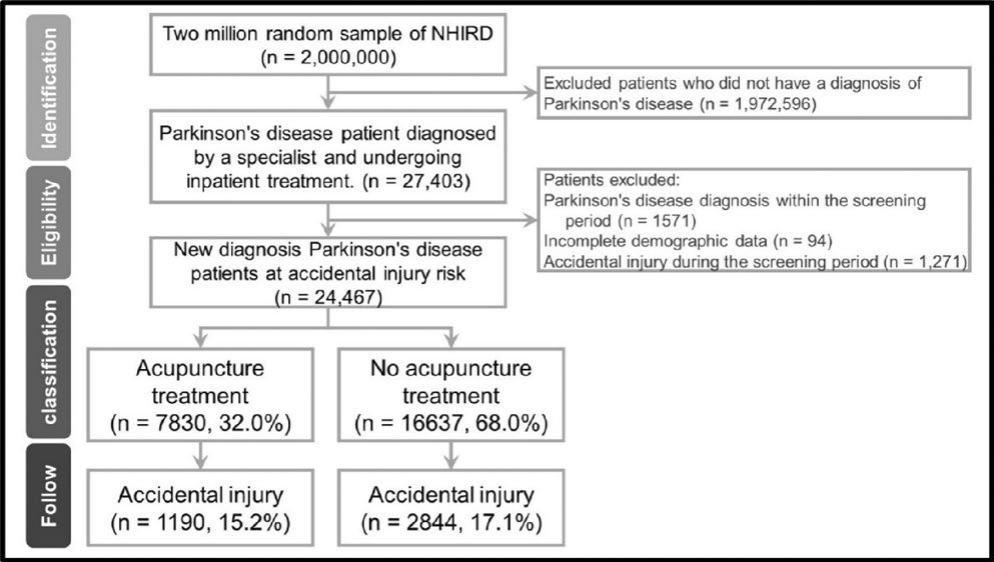
**FIGURE 1** Flowchart depicting the selection of participants and assessment of accidental injuries in a study on Parkinson's disease patients.

**Figure:**
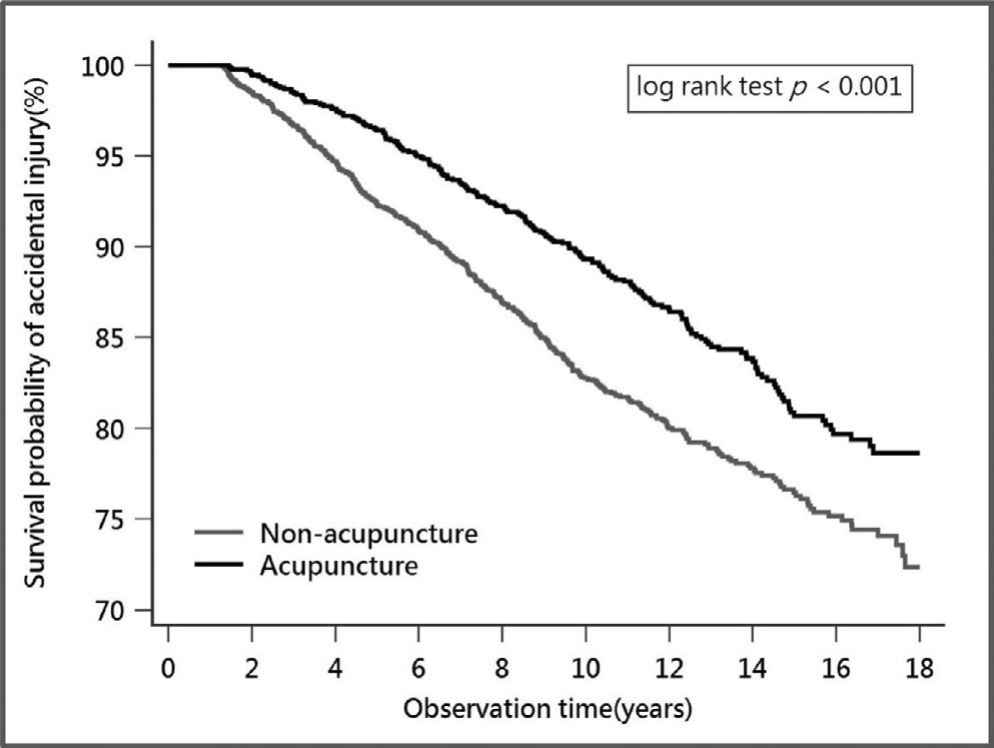
**FIGURE 2** Kaplan‐Meier analysis of Parkinson's disease patients based on acupuncture treatment.

**Figure:**
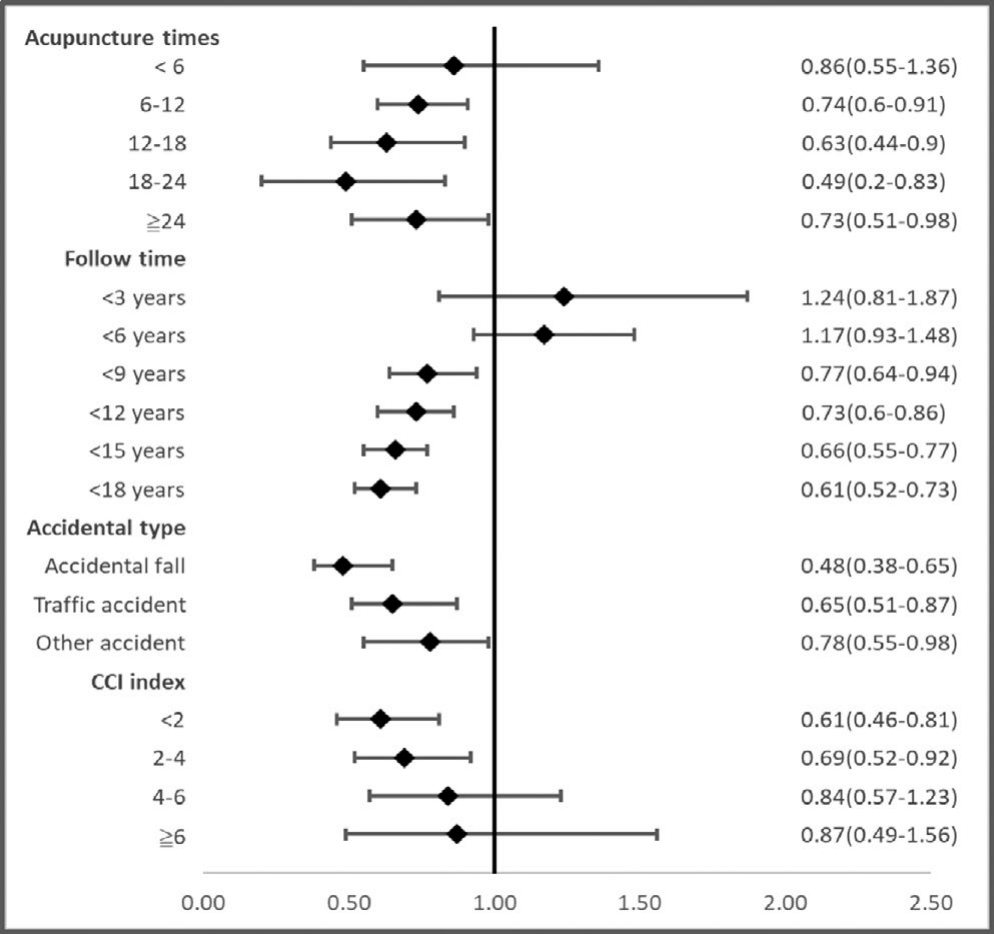
**FIGURE 3** Adjusted hazard ratios for accidental injuries across different acupuncture frequencies, follow‐up periods, and injury types; CCI index, Charlson Comorbidity Index.

**Conclusion:** Acupuncture significantly reduces the risk of accidental injuries in patients newly diagnosed with PD, suggesting its potential as a preventive strategy. Incorporating acupuncture into standard PD care may enhance safety and quality of life for these patients.

**Disclosure:** Nothing to disclose.

## EPO‐603

### Motor, nonmotor and cognitive predictors of early treatment‐related motor fluctuations in Parkinson's disease patients

#### C. Cabato; R. De Micco; M. Siciliano; F. Ambrosio; O. Ciaramaglia; F. Pagliuca; V. Sant'Elia; A. Tessitore

##### Department of Advanced Medical and Surgical Sciences, University of Campania “Luigi Vanvitelli”, Naples, Italy

**Background and Aims:** Treatment‐related motor fluctuations may emerge within few years after levodopa initiation but may go unrecognized at office visits, particularly in patients with a shorter disease duration. We investigated motor, nonmotor and cognitive predictors associated with early development of motor fluctuations after 2 years of treatment.

**Methods:** The study sample was recruited from a longitudinal project enrolling drug‐naïve PD. Patients underwent an extensive motor, nonmotor and cognitive assessments by means of validated scales at the time they were diagnosed with PD. After the baseline assessments, all patients were prescribed with dopaminergic treatment and yearly clinically assessed. At the 2‐year follow‐up, 73 patients have developed early signs of wearing‐off, defined as having at least 1 h of daily OFF time for at least 4 weeks (PD early‐fluctuators, PD‐EF) and were automatically matched with 77 patients without motor fluctuations (PD non‐fluctuators, PD‐NF). Baseline motor, nonmotor and cognitive data were compared between the study groups. A multivariate regression model was used to explore clinical baseline predictors of treatment‐related motor fluctuations at 2‐year follow‐up.

**Results:** At baseline, compared to PD‐NF, PD‐EF were presenting higher severity of pain, depression, autonomic dysfunction and worse performances in memory, executive and visuospatial cognitive domains.

**Conclusion:** Our findings demonstrated that specific nonmotor and cognitive features may characterize drug‐naïve PD patients more prone to develop early treatment‐related fluctuations. Identifying at‐risk PD population prior to starting dopaminergic treatment may help clinical management and foster prevention strategies.

**Disclosure:** Nothing to disclose.

## EPO‐604

### Brain networks alterations based on EEG pattern assignment in at‐risk of DLB subjects

#### D. Ondracek ^1^; M. Lamos^1^; D. Ondracek^2^; L. Brabenec^1^; K. Mitterova^1^; I. Rektorova^1^; I. Rektorova^2^

##### ^1^Brain and Mind Research Program, CEITEC, Masaryk University, Brno, Czechia; ^2^First Department of Neurology, Faculty of Medicine, Masaryk University and St. Anne's University Hospital, Brno, Czechia

**Background and Aims:** EEG microstates (EEG MS) provide spatial and temporal characteristics of large‐scale brain networks. While EEG patterns described by Bonanni's group improve early diagnosis of dementia with Lewy bodies (DLB), EEG MS may enhance our understanding of early network changes.

**Methods:** Altogether 117 medication‐naïve subjects at risk of DLB (mean age… ± …) underwent a 5‐minute recording of high‐density resting state scalp EEG. Participants were sorted based on their EEG pattern assignment into three groups: pattern 1 (normal EEG), pattern 2 (early DLB) and pattern 5 (advanced DLB). We compared time coverage, mean duration and occurrence of individual EEG MS across EEG pattern groups.

**Results:** We identified altogether 5 EEG MS. We observed higher time coverage (*p* = 0.003 and *p* < 0.001) and higher occurrence (*p* < 0.001 and *p* < 0.001) of MS A (engaging mostly temporal cortices and representing sensory and arousal networks) in DLB EEG patterns 2 and 5 compared to pattern 1, respectively. Conversely, the same comparison revealed lower time coverage (*p* = 0.004 and *p* = 0.004) and lower mean duration (*p* < 0.001 and *p* = 0.005) of the EEG MS C (engaging anterior cingulate and insular cortices ‐ representing salience network) in those with DLB EEG patterns.

**Conclusion:** We demonstrated that DLB‐related EEG patterns were presented in almost half of at‐risk of DLB/prodromal DLB subjects. Those with pathological EEG patterns as compared to those with normal EEG showed increased excitability of the sensory/arousal networks and decreased involvement of the salience network.

**Disclosure:** Nothing to disclose.

## EPO‐605

### Parkinson's disease and the metabolic syndrome: An integrated analysis of complex clinical and biochemical profiles

#### D. Akramova; G. Rakhimbaeva; D. Bobamuratova; B. Ibodov; M. Akramova

##### Tashkent Medical Academy, Tashkent, Uzbekistan

**Background and Aims:** Parkinson's disease (PD) and metabolic syndrome are two complex and multidimensional diseases, the combination of which has a significant impact on the quality and life of patients. This study aims to link these two conditions to determine how their combination affects metabolic and neurodegenerative functions.

**Methods:** The study design was based on a retrospective analysis, in which data from patients in two different clinical groups were studied: 62 patients diagnosed with PD only and 32 patients diagnosed with PC and metabolic syndrome. Clinical data, neuropsychological test results, and biochemical parameters were collected and analyzed. Independent samples *t*‐test, Mann‐Whitney *U* test, and χ^2^ test were used for data analysis.

**Results:** The clinical manifestations of PD were higher in the group with metabolic syndrome. In this group, the mean score on the MDS‐UPDRS scale was 134.8 ± 6.1, and on the PD‐CRS scale was 65.1 ± 2.1, both of which were considered significant at the *p* < 0.01 level. The levels of glucose and urea in the blood were also significantly higher in the metabolic syndrome group (glucose 9.3 ± 0.55 mmol/L, *p* < 0.001; urea 8.3 ± 0.54 mmol/L, *p* < 0.05).

**Conclusion:** The results of this study demonstrate that the co‐occurrence of metabolic syndrome and PCa produces complex clinical and biochemical profiles. Integrated management of these two conditions may lead to the development of new treatment strategies aimed at slowing disease progression and improving the overall health of patients.

**Disclosure:** Nothing to disclose.

## EPO‐606

### Genetic influences on Parkinson's disease clinical phenotype according to gene function and number of mutated variants

#### C. Gaiga ^1^; G. Ermanis^1^; D. De Monte^1^; C. Del Regno^1^; M. Scanni^1^; A. Bernardini^1^; G. Pellitteri^1^; E. Belgrado^2^; M. Mucchiut^2^; E. Betto^3^; G. Gigli^1^; G. Damante^3^; M. Valente^1^; F. Janes^1^

##### ^1^Clinical Neurology Unit, Santa Maria della Misericordia University Hospital, Udine, Italy; ^2^Neurology Unit, Santa Maria della Misericordia University Hospital, Udine, Italy; ^3^Institute of Medical Genetics, Azienda Sanitaria Friuli Centrale (ASUFC), Udine, Italy

**Background and Aims:** Parkinson's disease (PD) clinical phenotype may be influenced by genetic risk factors. We wanted to investigate the link between genetic characteristics and symptoms in a selected population of PD patients.

**Methods:** We collected clinical and genetic data of 143 PD patients with age at onset (AAO) < = 60 years or with positive familial history for parkinsonism (FH+). We investigated if the distribution of clinical characteristics differed significantly between subjects with negative test (GT‐) and positive test patients categorised according to the main function(s) of each mutated gene(s), and we evaluated the correlation between 12.5th percentiles and number of one‐mutation and more‐than‐one‐mutations carriers per percentile.

**Results:** We found no significant differences between positive and GT‐ patients when comparing AAO (*p* = 0.872), FH+ (*p* = 0.764) and each symptom. When considering patients divided in functional classes, we discovered in “lysosomal” class patients a higher prevalence of orthostatic hypotension (*p* = 0.038) and, in “protein synthesis” class, a higher AAO (*p* = 0.012) than in GT‐ subjects. We also detected a significant correlation between 12.5th percentiles’ mean AAO and number of more‐than‐one‐mutations carriers per percentile (*R* = ‐0.796; *p* = 0.018).

**Figure:**
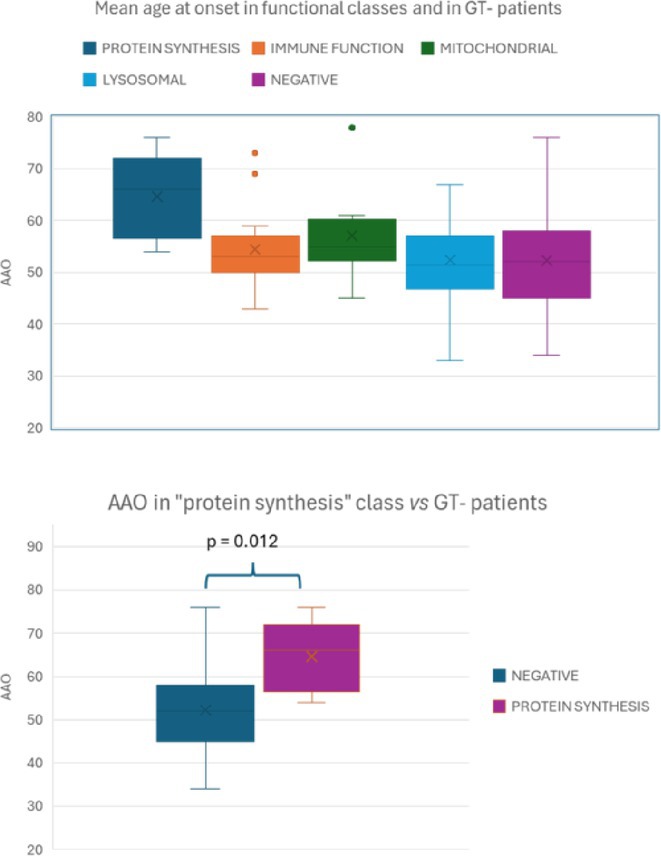
**FIGURE 1** Top graph: mean AAO in functional classes and in GT‐ patients. Bottom graph: mean AAO between “protein synthesis” patients and GT‐ patients; the difference in AAO between these two categories proved to be significant (*p* = 0.012).

**Figure:**
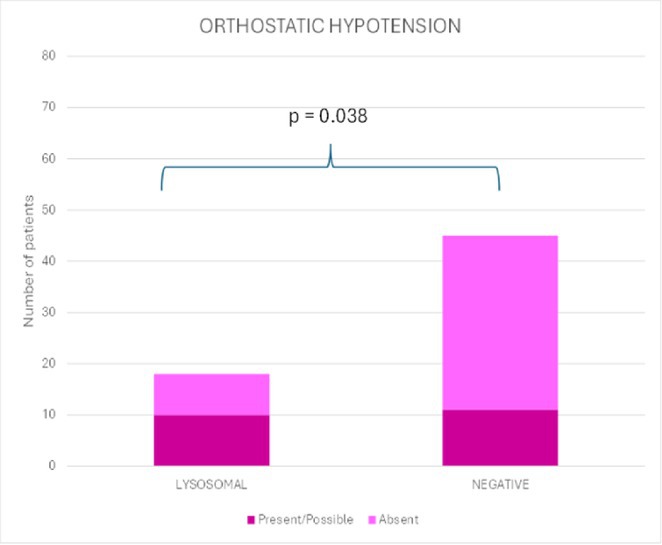
**FIGURE 2** Distribution of orthostatic hypotension in patients with “lysosomal” mutation(s) in comparison with GT‐ patients, revealing a significant prevalence in mutated patients.

**Figure:**
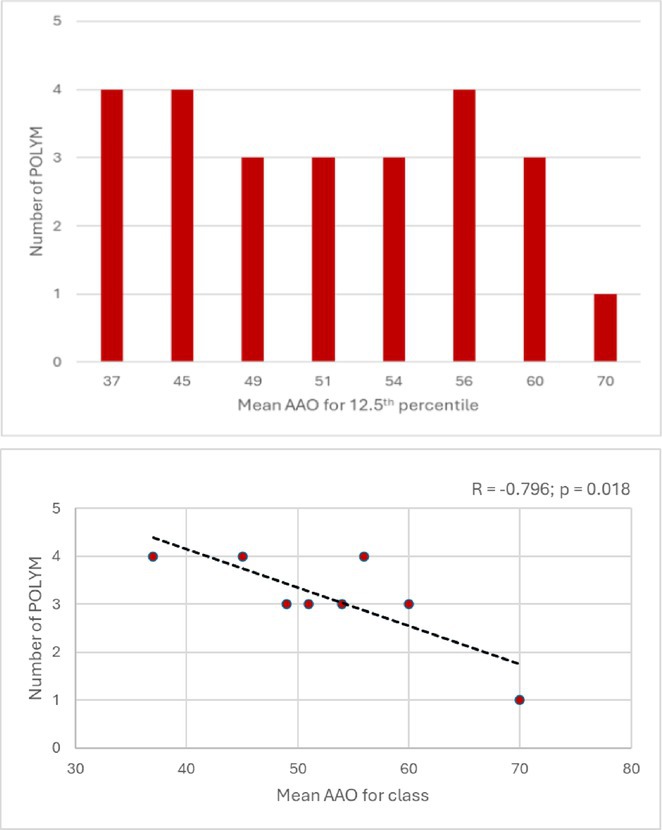
**FIGURE 3** Distribution of more‐than‐one‐mutations carriers (POLYM) and mean AAO for 12.5th percentile.

**Conclusion:** Based on our data, earlier AAO and FH+ seem not related to positive result at genetic test. We saw in “lysosomal” class mutations a higher prevalence of orthostatic hypotension and in “protein synthesis” subjects a higher AAO than in GT‐ subjects. We also observed an inverse association between AAO and chance of obtaining more than one mutation in positive genetic test results, possibly reflecting an additive role in favouring PD onset.

**Disclosure:** Nothing to disclose.

## EPO‐607

### Constipation and fecal biomarkers in a de novo Parkinson disease cohort

#### S. Enriquez ^1^; L. Melgarejo^1^; M. Millet^1^; A. López^1^; M. Arrué^1^; D. Samaniego^1^; M. Martinez^1^; J. Hernandez^2^; A. Laguna^1^

##### ^1^Val d´Hebron Institut de Recerca, Neurodegenerative Diseases Group, Barcelona, Spain; ^2^Movement disorders Unit, Vall d´Hebron University Hospital, Barcelona, Spain

**Background and Aims:** Parkinson's disease (PD) is the second growing neurodegenerative disorder characterized by motor and non‐motor symptoms, including significant gastrointestinal (GI) dysfunction, particularly in early stages. This study aimed to explore blood, CSF and fecal biomarkers in a de novo PD cohort.

**Methods:** Clinical assessments included demographics, motor status by the Unified Parkinson's Disease Rating Scale, University of Pennsylvania Smell Identification Test, Montreal Cognitive Assessment, SCOPA‐AUT. Calprotectin, zonulin and interleukines and alfa syn levels total and aggregated in blood and CSF and stools were measured. Data were analyzed using R.

**Results:** 81 patients were included: 19 controls, 16 treated PD and 46 de novo PD. 42% women and mean age in all groups were 57 (+‐2.9). Total UPDRS was higher in PD treated (57.6+‐3) than in de novo PD and controls (table 1). Hiposmia and constipation was higher in de novo PD than healthy controls, and SCOPA‐Aut was higher in PD treated than de novo PD. Serum calprotectine (intestinal permeability) was slightly higher in de novo PD than controls, Aggregated alfa syn was detected also in blood, CSF and stool of patients with PD (figure 1).

**Figure:**
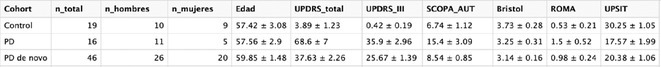
TABLE 1

**Figure:**
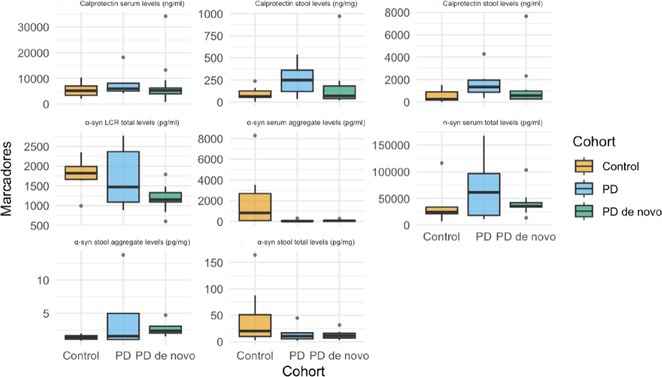
FIGURE 1

**Conclusion:** This study highlights the relevance of GI dysfunction in de novo PD. Innovative and less invasive biomarkers in stool could could aid in early diagnosis and classify clinical subgroups. Further longitudinal studies are needed to validate these findings and explore therapeutic implications.

**Disclosure:** Nothing to disclose.

## EPO‐608

### Longterm effect of EMG‐guided botulinum toxin (BoNT) treatment of complex facial dystonia with initial progression involving oromandibular dystonia

#### H. Biernat; S. Bech; A. Stark; A. Løkkegaard

##### Bispebjerg and Frederiksberg University Hospital, Neurological Department, Copenhagen, Denmark

**Background and Aims:** Oromandibular dystonia (OMD) is a rare form of dystonia with involuntary movements of the jaw muscles, causing masticatory problems. In some patients, the OMD progresses, in‐volving eyelid spasms (Meige syndrome). Symptoms may also progress to a more wide‐spread dystonia. These case‐presentations demonstrate distinct dystonic manifestations that illustrate a progression of a primary OMD to a wider head and neck involvement. The ob‐jective is to illustrate the diagnostic and therapeutic benefits of employing EMG guidance for BoNT treatment, preceded by standardized evaluation.

**Methods:** Two patients with oromandibular dystonia initially progressing with involvement of head and neck area with amongst other OMD, were assessed with: • Clinical and orofacial examination • Muscle Strength (jaw opening force, hand grip strength) • Sensibility (oral stereognosis and two‐point discrimination) • Burke‐Fahn Marsden Dystonia rating scale, Pain Numeric rating Scale Treatment with needle‐EMG‐guided BoNT was based on the assessment. The patients were treated regularly, every three months.

**Results:** Case 1 symptoms onset in 2009. EMG guided treatment regime involved mm. digastricii, mm. thyerohyoideus and mm. obicularis oculi until 2013, when the treatment could be re‐duced to aesthetic treatment with 47.5 U INCO‐BoNT in mm. obicularis oculii bilat and plat‐ysma. Case 2 had symptom onset in 2012. EMG guided treatment regime in. mm. orbicularis oris superior and inferior and dxt. M. pteryogoideus lateralis until 2019, henceforth estehtic treatment with 15 U INCO‐BoNT in mm orbicularis oris inferior.

**Conclusion:** Conclusion: Standardized protocolled EMG‐guided treatment of complex facial dystonia provides long‐term benefit with stabilization of progression of symptoms.

**Disclosure:** Nothing to disclose.

## EPO‐609

### Association of urate with dopaminergic integrity and motor function in REM sleep behavior disorder: A multicenter study

#### J. Jung ^1^; H. Na^2^; S. Lee^3^; E. Kim^4^; K. Ji^1^; M. Kang^1^; E. Chung^1^; P. Lee^2^; S. Kim^1^

##### ^1^Department of Neurology, Busan Paik Hospital, Inje University College of Medicine, Busan, Republic of Korea; ^2^Department of Neurology, Yonsei University College of Medicine, Seoul, Republic of Korea; ^3^Department of Nuclear Medicine, Busan Paik Hospital, Inje University College of Medicine, Busan, Republic of Korea; ^4^Department of Psychology, Vanderbilt University, Nashville, Tennessee, USA

**Background and Aims:** Research on urate in idiopathic REM sleep behavior disorder (iRBD), a prodromal stage of synucleinopathies, is limited. This study examined the relationship between serum urate levels, presynaptic dopamine neuronal integrity, and motor deficits in iRBD.

**Methods:** We enrolled 18 patients with polysomnography (PSG)‐confirmed iRBD who underwent positron emission tomography (PET) scans to assess dopamine transporter (DAT) availability in the posterior putamen. Relationships between serum urate levels, mean posterior putaminal DAT availability, and motor deficits were analyzed using Unified Parkinson's Disease Rating Scale Part III (UPDRS‐III) scores and subthreshold parkinsonism status. For validation, 39 additional PSG‐confirmed iRBD patients with serum urate measurements and dopamine transporter PET scans were recruited from another center.

**Results:** Serum urate levels were positively correlated with mean posterior putaminal DAT uptake (*n* = 18; γ = 0.508, *p* = 0.032), a relationship that remained significant after adjusting for confounders such as age, sex, BMI, and alcohol use (β [SE] = 0.323 [0.094], *p* = 0.005). Although no significant association was found with UPDRS‐III scores (γ = ‐0.449, *p* = 0.061), iRBD patients without subthreshold parkinsonism had higher serum urate levels than those with subthreshold parkinsonism (median [IQR], mg/dL = 5.2 [3.3] vs. 3.8 [1.7], *p* = 0.040). Validation confirmed the positive correlation between serum urate and posterior putaminal DAT uptake (*n* = 39; γ = 0.488, *p* = 0.002).

**Conclusion:** Elevated serum urate levels are associated with preserved posterior putaminal dopaminergic neurons and motor function in iRBD, suggesting a potential neuroprotective role of urate in early synucleinopathies.

**Disclosure:** Nothing to disclose.

## EPO‐610

### Motor phenotype as a factor in sleep disturbances in Parkinson's disease

#### J. Trejo‐Ayala ^2^; H. Trujillo‐Guerra^1^; B. Chávez‐Luévanos^3^; I. Estrada‐Bellmann^2^

##### ^1^Servicio de Neurología, Monterrey, Mexico; ^2^Clínica de Parkinson y Trastornos del Movimiento, Monterrey, Mexico; ^3^Laboratorio y Unidad de Monitoreo de Epilepsia y Sueño, Mexico

**Background and Aims:** In Parkinson's disease (PD), sleep disturbances such as insomnia or REM sleep behaviour disorder (RBD) are common. It is known that motor phenotypes exhibit heterogeneity in cognitive, psychiatric, and sleep symptoms.

**Methods:** A cross‐sectional study was conducted from June 2022 to January 2025 in patients from a movement disorders clinic, using clinical surveys and the Movement Disorders Society Unified Parkinson's Disease Rating Scale (MDS‐UPDRS). Patients were divided into three motor phenotype groups: postural instability and gait difficulty (PIGD), tremor‐dominant (TD), and indeterminate (INDT). Parametric statistics were applied.

**Results:** A total of 138 patients were included: 64 PIGD (46.4%), of whom 51 (79.7%) presented sleep disturbances; 53 TD (38.4%), with 43 (81.1%) showing disturbances; and 21 INDT (15.2%), with 19 (90.5%) affected. Sleepiness was the most prevalent symptom across all three groups (41 (64.1%), 27 (50.9%), and 14 (66.7%), respectively). No significant differences (*p* > 0.05) were found in the presence of sleep disturbances RBD, insomnia, or sleepiness with respect to motor phenotype.

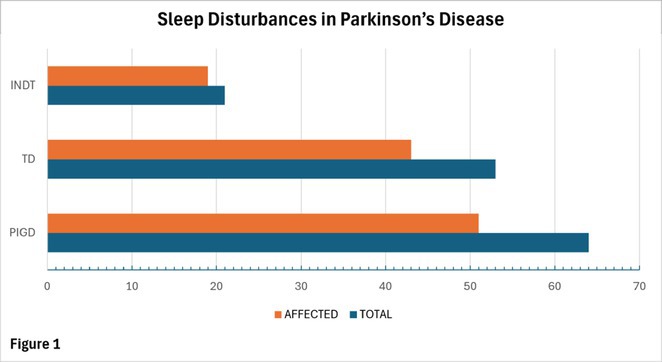

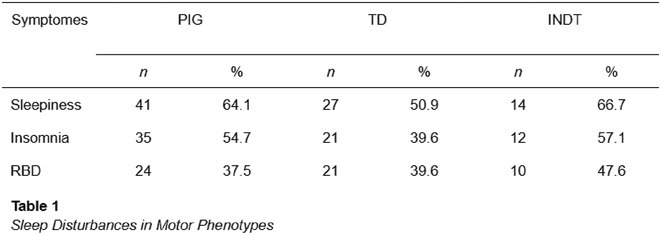

**Conclusion:** Our results suggest that sleep disturbances are common symptoms in PD patients. Motor phenotype does not appear to be a determining factor in the presence of sleep disturbances. This highlights the need to individualise patient care, taking into account comorbidities and symptoms, with particular emphasis on assessing sleepiness.

**Disclosure:** Nothing to disclose.

## EPO‐611

### Double limb support as core feature of Parkinson's disease with mild motor fluctuations

#### L. Colombo ^1^; A. Pilotto^1^; C. Zatti^1^; A. Rizzardi^1^; C. Hansen^2^; A. Magliozzi^1^; R. Romijnders^2^; M. Rizzetti^3^; W. Maetzler^2^; A. Padovani^1^

##### ^1^Department of Clinical and Experimental Sciences, Neurology Unit, University of Brescia, Italy; ^2^Department of Neurology, Christian‐Albrechts‐University of Kiel, Kiel, Germany; ^3^Parkinson's disease Rehabilitation Center, FERB European Fundation Biomedical Research, Trescore Balneario hospital Bergamo, Italy

**Background and Aims:** Motor fluctuations are common in Parkinson's Disease (PD), and are associated with increased risk of falls and other complications. The aim of this study was to evaluate the presence of specific gait alternations during ON phases in patients with motor fluctuations (PD‐F) using Mobile Health Technology (MHT), compared with patients without motor fluctuations (PD‐NF) in groups of matched patients for motor and non‐motor severity.

**Methods:** The study enrolled matched PD patients with and without motor fluctuations, who underwent extensive clinical assessment and supervised MHT evaluation in ON state at normal and fast paces and during dual‐task performance.

**Results:** 60 subjects were included, 30 PD‐F and 30 PD‐NF. In the ON‐phase gait analysis, PD‐F exhibited a longer double limb support time compared to PD‐NF patients during normal, fast, and dual‐task walking conditions, with similar gait parameters were comparable.

**Conclusion:** Therefore, we conclude that the increased double limb support time may indicate greater postural instability in patients with motor fluctuations.

**Disclosure:** Nothing to disclose.

## EPO‐612

### Skin biopsy as a biomarker for parkinsonism: Differentiating synucleinopathies and tauopathies – A systematic review

#### K. Nesrine ^1^; A. Leila^2^; K. Nourchene^3^

##### ^1^University Hospital of Bordeaux, Bordeaux, France; ^2^University of Pegaso, Napoli, Italy, University of Camerino, School of Advanced Studies, Macerata, Italy; ^3^Faculty of Medicine of Tunis, Tunisia

**Background and Aims:** The differentiation between synucleinopathies and tauopathies remains a significant challenge due to overlapping clinical presentations. Accurate biomarkers are needed to improve diagnostic precision. Skin biopsy has emerged as a minimally invasive method capable of detecting pathological seed aggregates. The aim of the study was to evaluate the effectiveness of skin biopsy in differentiating synculeinopathies and tauopathies.

**Methods:** A systematic literature review was conducted using PubMed, Web of Science, and Scopus. The following combination of MeSH terms and Boolean operators was used: ((Parkinson*)AND(tauopathies)AND(synucleinopathies))AND((skin)OR(cutaneous))AND((diagnosis)AND(specificity)). The search focused on articles published between 2014 and 2024. The main outcome was to assess the diagnostic accuracy and specificity of skin biopsy in distinguishing between synucleinopathies and tauopathies in patients exhibiting motor symptoms.

**Figure:**
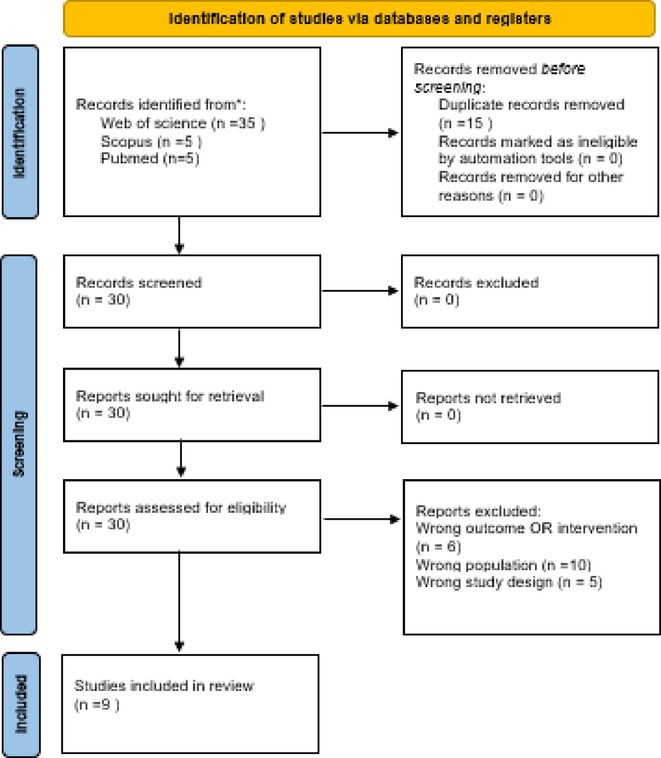
**FIGURE 1** Prisma flow diagram

**Results:** From 35 studies identified, we selected 9 papers. The clinical features were dominated by parkinsonism. Of these studies, 4 used multiple biopsy sites targeting the lower leg, forearm, and cervical region. For synucleinopathies, skin biopsy demonstrated a high sensitivity and specificity in detecting seeds aggregates, with pathological tau also observed at lower levels. Tauopathies were diagnosed with a similar average of sensitivity and specificity. The Skin tau, using TauK18 and TauK19 as reaction substrates for 4R and 3R isoforms were used to differentiate both proteinopthies. Higher concentrations of synuclein seed aggregates were found in the cervical region, while tau aggregates were predominantly located in limbs

**Conclusion:** Our review highlights the potential of skin biopsy as a promising non‐invasive diagnostic tool for synucleinopathies and tauopathies.

**Disclosure:** Nothing to disclose.

## EPO‐613

### Comparison of specialist ataxia centres with non‐specialist services for ataxia care, resource use and costs in Italy

#### J. Vallortigara^1^; J. Greenfield^2^; B. Hunt^2^; A. Filla^3^; A. Federico^4^; M. Litani^5^; D. Hoffman^6^; S. Morris^7^; P. Giunti ^1^

##### ^1^Ataxia Centre, Department of Clinical and Movement Neurosciences, UCL Queen Square Institute of Neurology, Queen Square House, Queen Square, London, UK; ^2^Ataxia UK, London, UK; ^3^Department of Neurosciences, Reproductive and Odontostomatological Sciences, Federico II University, Naples, Italy; ^4^Department of Medicine, Surgery and Neurosciences, Medical School, University of Siena; ^5^A.I.S.A Onlus, Sestri Levante (GE), Italy; ^6^Takeda Pharmaceuticals, Cambridge, USA; ^7^Primary Care Unit, Department of Public Health and Primary Care, University of Cambridge, Cambridge, UK

**Background and Aims:** The ataxias are rare complex neurological disorders that represent a challenge for the clinicians to diagnose and manage. This study explored the patient pathways, costs associated and care satisfaction of individuals attending specialist ataxia centres (SAC) compared with non–specialist settings in Italy.

**Methods:** A patient survey was distributed to people with ataxia in Italy during May – September 2021 to gather information about the diagnosis and management of the ataxias in SAC and non‐specialist settings including patients’ satisfaction. We compared mean resource use for each contact type and health service costs per patient, stratifying patients by whether they were currently attending a SAC or had never attended a SAC.

**Results:** We had 174 participants in the survey; 44% of participants saw a neurologist within 6 months of seeking medical advice, and 56% went to a SAC as first referral. Traveling was a top reason why people stopped going to a SAC and why people never attend one. People attending SAC reported that such centres delivered better service in most aspects (coordinating referrals, offering opportunities to take part in research, communications with social care professionals).

**Conclusion:** There was a trend towards patients’ appreciation for the delivery of service in SAC compared to non‐SAC. However, we identified difficulties in attendance and adherence despite the higher number of SACs in Italy compared to other countries (UK and Germany). We believe that combination of face‐to‐face and telemedicine could improve the continuation of the patients attendance to SAC.

**Disclosure:** Nothing to disclose.

## EPO‐614

### Essential tremor patient journey, from caseload to advanced therapies: A neurology survey

#### A. Sanchez Fraga ^1^; C. Tengelin^1^; K. Gant^2^; P. Crivelli^1^; C. Ferrer^1^; A. Grinspan^2^

##### ^1^Insightec Europe GmbH, Munich, Germany; ^2^Insightec Inc., Miami, USA

**Background and Aims:** Recent evidence on disease burden, tremor classification and treatment guidelines, including advanced therapies like magnetic resonance‐guided focused ultrasound (MRgFUS), has changed the essential tremor (ET) landscape. We investigated neurologist perceptions on ET caseload, tremor severity, advanced therapy and standard protocol use in Europe.

**Methods:** An anonymized web survey was conducted between 16‐01‐23 and 24‐02‐23, following relevant data privacy legislation and guidelines. General neurologists (GN) and movement disorder neurologists (MDN) were asked about ET patients’ caseload, severity, prescription of advanced therapies and use of standardized protocols.

**Results:** The survey was completed by 68 MDN and 156 GN. Neurologists reported that 10.1 ± 4.5% of their patients experienced ET; 62.5 ± 6.7% of those presented a moderate to severe condition (Figure 1). Prescriptions for advanced therapies were deep brain stimulation (mild 3.3 ± 1.3%; moderate 14.8 ± 3.6%; severe 82.0 ± 4.8%); MRgFUS (mild 7.7 ± 2.2%; moderate 26.4 ± 9.1%; severe 66.0 ± 11.4%). Earlier prescription and higher variations, apart from severity, were observed between the MDN and GN groups for MRgFUS (Figure 2). Neurologists envisaged prescribing MRgFUS to 27.1 ± 3.6% of their patients (Figure 3) with most neurologists (>70%) being satisfied. Only 37.5 ± 16% of neurologists reported adhering to standardized pathways/protocols (Figure 3).

**Figure:**
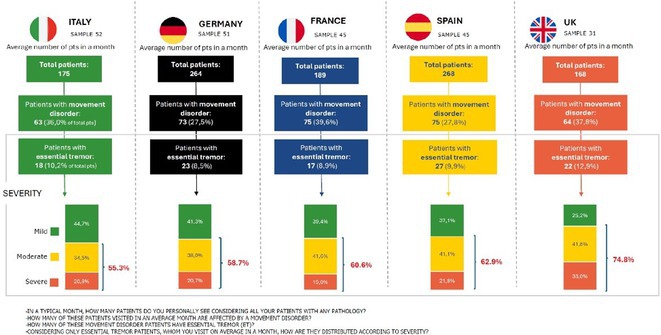
**FIGURE 1** Origin and severity of essential tremor patients referred to general and movement disorder neurologists in Europe (Italy, Germany, France, Spain, and the UK). The sample number of neurologists per country is reported.

**Figure:**
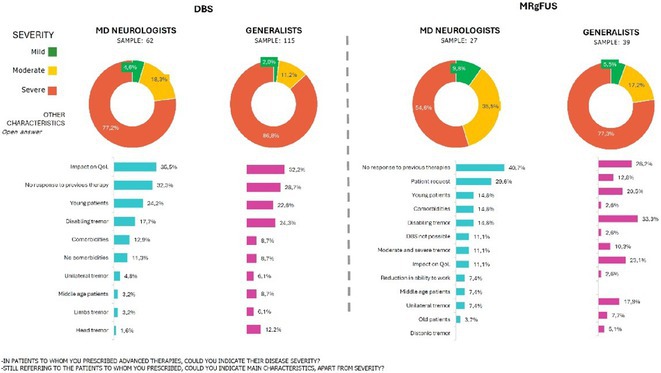
**FIGURE 2** Severity and disease characteristics of patients who were prescribed advanced therapies for essential tremor. The sample number of neurologists is reported.

**Figure:**
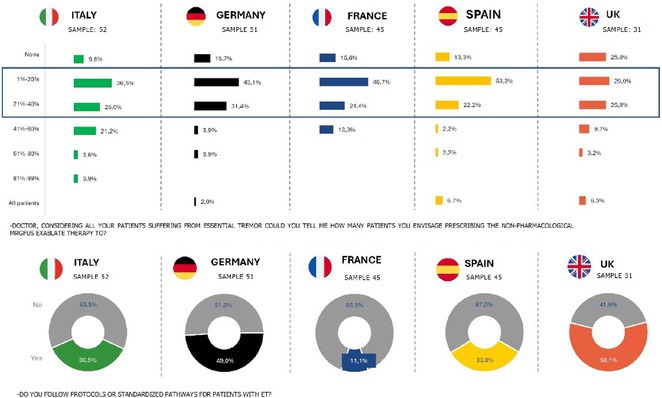
**FIGURE 3** Proportion of patients with essential tremor (ET) likely to be prescribed MRgFUS by neurologists in Europe and the proportion of neurologists adhering to protocols/standardised pathways. The sample number of neurologists per country is reported.

**Conclusion:** In Europe, the ET caseload is high among neurologists, with most patients presenting moderate‐to‐severe symptoms. Nevertheless, access to advanced therapy remains low with a heterogenous patient profile. This may be related to the absence of standardized protocols. Further study is necessary to explain geographical differences and further work should focus on creating and disseminating protocols to enhance patient access to the appropriate therapies.

**Disclosure:** The authors are all Insightec employees. To carry out the survey, support was provided by Stethos Srl, Italy, Milan. Writing assistance was provided by Content Ed Net, Madrid, Spain. The survey responses were used for market research purposes.

## MS and related disorders 4

## EPO‐615

### Treatment landscape and patient journey in MS: Experience from central military emergency hospital in Bucharest, Romania

#### C. Vlaicu ^1^; I. Caloianu^2^; C. Sîrbu^2^

##### ^1^Neurology Department, Central Military Emergency Hospital “Dr. Carol Davila”, Bucharest, Romania; ^2^Neurology Department, University of Medicine and Pharmacy “Carol Davila”, Bucharest, Romania

**Background and Aims:** Multiple sclerosis (MS) management requires timely access to diverse therapeutic options. We conducted a retrospective study spanning 2000–2021 to analyze treatment patterns, obstacles, and outcomes among 805 MS patients treated at Central Military Emergency Hospital “Dr. Carol Davila” (SUUMC) in Bucharest, Romania.

**Methods:** Data on therapy types, duration, changes, hospitalizations, and costs were extracted from medical records. Descriptive statistics and trend analysis were employed.

**Results:** Platform therapies were most commonly utilized (59%), followed by intermediary active drugs (24%), and highly active therapies (17%). 15.6% of patients changed treatment, primarily due to adverse reactions (54%) or lack of efficacy (39%). The most commonly changed drugs were platform therapies, first being recombinant human interferon beta‐1b, followed by recombinant human interferon beta‐1a and glatiramer acetate, and were most frequently substituted with teriflunomide and natalizumab. The mean time until treatment change was 5.2 years. Moreover, 7.4% of patients transitioned to territorial centers, reflecting geographical dynamics. Day hospitalizations increased from 860 (2011) to 1518 (2021), mirroring evolving care needs. Treatment costs rose from 7.3 million lei (2011) to 15 million lei (2021), totaling approximately 119 million lei (approximately 24 million euros) over the study period.

**Conclusion:** The introduction of diverse therapies expanded treatment options for MS patients at SUUMC, allowing tailored management aligned with disease type and activity. Despite challenges such as delayed drug availability and treatment changes, our center witnessed improved patient access to appropriate therapies, reflected in increased hospitalizations and associated costs.

**Disclosure:** Nothing to disclose.

## EPO‐616

### Impact of single or multiple spinal regions pain on disability levels in multiple sclerosis: A meta‐analysis study

#### D. Onan ^1^; H. Arikan^2^

##### ^1^Department of Physiotherapy and Rehabilitation, Faculty of Health Sciences, Yozgat Bozok University, Yozgat, Turkey; ^2^Department of Physiotherapy and Rehabilitation, Faculty of Health Sciences, Tokat Gaziosmanpaşa University, Tokat, Turkey

**Background and Aims:** Pain is a common complaint in multiple sclerosis (MS), significantly impacts daily activities. Spinal pain (cervical, thoracic, or lumbal) can impair trunk stabilization and mobility. Clinicians may observe that pain in multiple spinal regions may increase disability, but does this pattern hold true from a broader perspective through meta‐analysis? This study aimed to evaluate differences in disability between MS patients experiencing pain in a single spinal region (SSR) and those with pain in multiple spinal regions (MSR).

**Methods:** A systematic search was conducted in PubMed, Web of Science, and Scopus databases up to December 2024 (Figure‐1). Studies were included if they assessed disability using the Expanded Disability Status Scale (EDSS) and reported spinal pain in MS patients. Subgroup analyses were conducted using the “meta” package in RStudio, applying both common‐effect model (CEM) and random‐effect models (REM). PROSPERO number is CRD42025632240.

**Figure:**
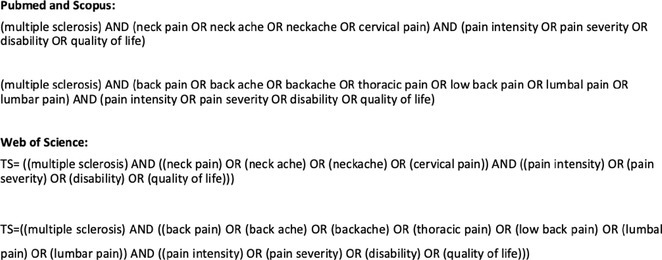
**FIGURE 1** Keyword searched in databases

**Results:** Seven studies met the inclusion criteria (Figure‐2). The SSR group (336 patients) had a mean EDSS score of 3.70 (common‐effect) and 3.90 (random‐effect) with moderate heterogeneity (*I* ^2^ = 82.2%). The MSR group (201 patients) scored 4.19 (common‐effect) and 4.39 (random‐effect) with high heterogeneity (*I* ^2^ = 99%). A significant difference between groups was found in the CEM (χ^2^ = 357.75, *p* < 0.0001), but not in REM (χ^2^ = 0.52, *p* = 0.4698). CEM results showed a higher disability in MSR pain patients compared to SSR pain patients (Figure‐3).

**Figure:**
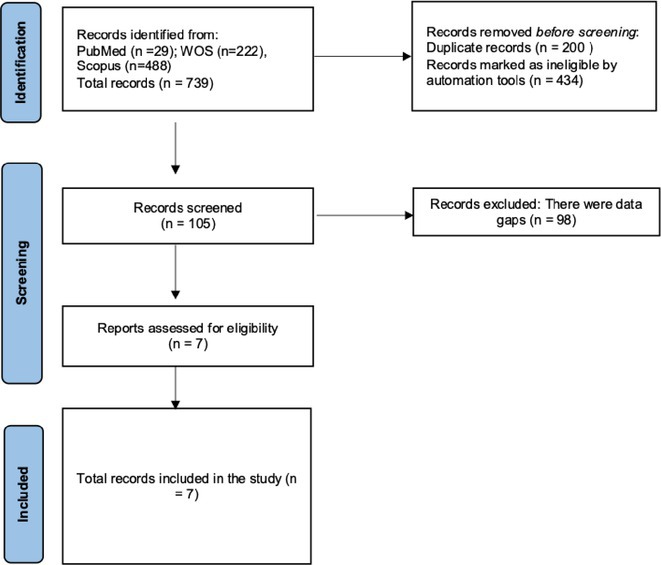
**FIGURE 2** The PRISMA flowchart of the literature search

**Figure:**
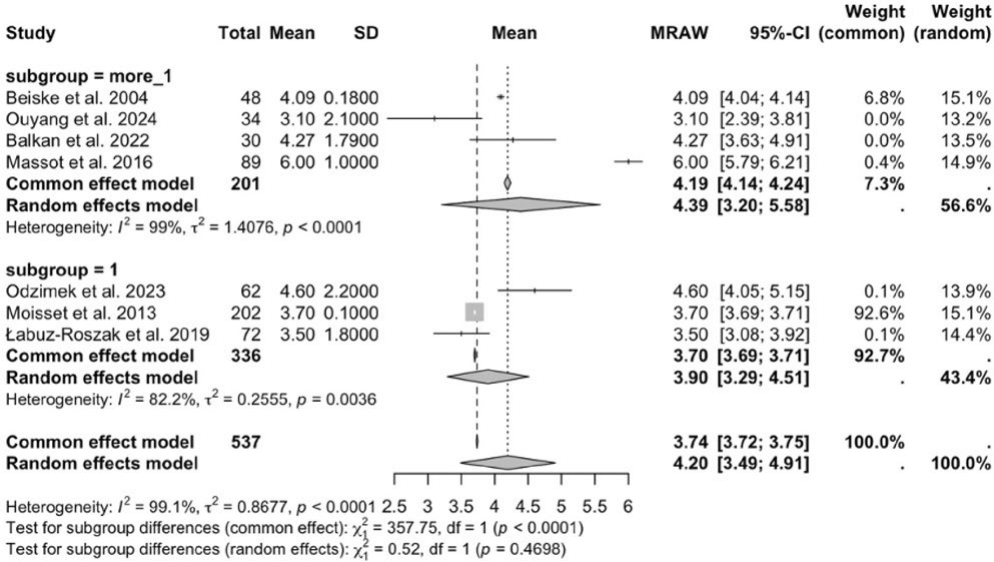
**FIGURE 3** Forest Plot of MS disability and subgroups

**Conclusion:** Patients with MSR pain may have a higher disability. While CME demonstrated significant differences, REM did not confirm them, likely due to heterogeneity. Therefore, future studies should focus on heterogeneity sources and spinal pain management.

**Disclosure:** Nothing to disclose.

## EPO‐617

### Gut microbiome at the onset of multiple sclerosis: An Italian cohort study

#### E. Virgilio ^1^; S. Rolla^1^; A. Maglione^1^; I. Ferrocino^2^; R. Rosso^1^; R. Lanzillo^3^; C. Cordioli^4^; F. Masuzzo^1^; D. Tortarolo^5^; S. Gepiro Contaldo^5^; M. Beccuti^5^; F. Cordero^5^; M. Clerico^1^

##### ^1^Department of Clinical and Biological Sciences, AOU San Luigi Gonzaga, University of Turin, Italy; ^2^Department of Agricultural, Forestry and Food Sciences, University of Turin, Italy; ^3^Department of Neurosciences, Reproductive Sciences and Odontostomatology, Federico II University, Naples, Italy; ^4^Spedali Civili di Brescia, Multiple Sclerosis Centre, Brescia, Italy; ^5^Department of Computer Science, University of Turin, Italy

**Background and Aims:** Multiple Sclerosis (MS) etiopathogenesis is uncertain. Gut microbiota possibly affects brain activity, producing active metabolites. However, its composition in early disease stages remains undetermined. We aimed to explore differences in gut microbiota composition at disease onset compared with healthy donors (HD) and identify differences based on negative prognostic factors such as high lesion burden.

**Methods:** 53 MS patients (pMS) at the first clinical attack and 18 healthy controls (HC), matched on age, sex, diet, BMI, and lifestyle, were recruited in a multicenter case‐control study. Stool, blood samples and radiological‐clinical data were collected at baseline. DNA isolated from stools was subjected to shotgun metagenomic sequencing strategy; peripheral blood mononuclear cells were isolated and analyzed by flow cytometry to identify T helper (Th)17 and T regulatory cells. Taxonomic classification was performed using Kraken2. Results were processed using Bayesian re‐estimation of abundance to provide accurate estimates.

**Results:** The overall gut microbiome structure of pMS did not differ from HC's, as indicated by α‐ and β‐diversity between the two groups. However, several species in different taxa were found up‐ or down‐regulated by comparing differential abundances between pMS and HC. Among clinical and radiological variables, glucocorticoid intake and MRI lesion burden were associated with microbiome diversity variations in pMS. Furthermore, some species correlated with increased pathogenic Th17 cells in the blood

**Conclusion:** Our results suggest that pMS displays a moderate gut microbial dysbiosis in the early stages, influencing not only the autoimmune peripheral response but also MS disease course.

**Disclosure:** Nothing to disclose.

## EPO‐618

### Functional evaluation of MS exacerbation treatment duration

#### E. Babych; Y. Solodovnikova; K. Yarovaya

##### Department of Neurology and Neurosurgery, Odesa National Medical University, Odesa, Ukraine

**Background and Aims:** Treatment of multiple sclerosis (MS) exacerbations has evolved, with pulse therapy of methylprednisolone for 3‐5 consequent days as the standard approach, followed by plasma exchanges if needed. Still, longer pulse therapies regimens exist with no consensus or specific guidelines regarding the optimal duration of such treatments. This study aimed to evaluate the correlation between the Multiple Sclerosis Functional Composite (MSFC) dynamics and methylprednisolone therapy duration in patients with MS.

**Methods:** 27 MS patients (11 males, 16 females) aged 22–50 years (mean 33, SD ± 7.29) with baseline EDSS scores of 1.5–5.0 (mean 3.59, SD ± 1.08) were assessed. MSFC was measured at hospitalization and after pulse therapy with methylprednisolone (1 g/day for 3, 5, or 7 days). MSFC score formulas were used for calculations and Jamovi v. 2.3.28.0 Windows with χ^2^ test and binomial logistic regression was used for statistical analysis.

**Results:** 14 participants showed MSFC improvement at discharge, while 13 deteriorated. Among the patients who underwent 7‐day therapy, all deteriorated. Among improved patients, 10 received 5‐day therapy, and 4 received 3‐day therapy. The χ^2^ test revealed a significant correlation between therapy duration and MSFC results (χ^2^ = 7.3, *p* = 0.026). Reducing the duration of therapy by 1 day increases the chances of improvement approximately 3 times (OR = 1/0.333 ≈ 3).

**Conclusion:** A 7‐day pulse therapy in MS exacerbations does not show additional benefits compared to 3‐ or 5‐day regimens. Shorter pulse therapies should be considered.

**Disclosure:** Nothing to disclose.

## EPO‐619

### Measuring frailty in multiple sclerosis: A systemic review of current frailty indices

#### F. Föttinger ^1^; M. Aicher^2^; R. Hoepner^2^; G. Bsteh^1^

##### ^1^Medical University of Vienna, Department of Neurology, Vienna, Austria; ^2^University Hospital of Bern, Department of Neurology, Bern, Switzerland

**Background and Aims:** Frailty in multiple sclerosis (MS) is an emerging concept linked to outcomes such as disability progression and disease activity, but standardized assessment methods are still lacking. This study evaluates existing evidence on frailty indices in MS.

**Methods:** A systematic review was conducted of studies published between 2014–2024 that reported original data or systematic reviews on frailty assessments in patients with MS (pwMS). Searches were performed in MEDLINE, EMBASE, and the Cochrane Library using the terms “multiple sclerosis” and “frailty.” Quality of evidence was assessed by four raters using the GRADE approach.

**Results:** From 817 screened studies, 10 met inclusion criteria, encompassing 1,941 pwMS and three frailty indices: Fried Frailty (FF), Frailty Index (FI), and Tilburg Frailty Indicator (TFI). FI, analyzed in 8 studies (1,653 patients), showed correlations with higher relapse likelihood (adjusted OR = 0.69; *p* < 0.01, inverse), and greater disability (EDSS; β = 0.47, *R* ^2^ = 0.26, *p* < 0.001). Evidence quality was low due to bias and imprecision. FF and TFI were linked to higher disability (TFI: β = 0.57, *R* ^2^ = 0.35, *p* < 0.001), with TFI additionally associated with poorer quality of life (*p* < 0.001) and reduced autonomy (*p* = 0.017), though evidence quality was very low due to limited studies and high risk of bias.

**Conclusion:** This review highlights the potential of frailty indices in MS, yet reveals significant gaps in validation and applicability, with routine clinical use being premature. Further research is needed to develop and validate MS‐specific frailty assessments.

**Disclosure:** Nothing to disclose.

## EPO‐620

### Measuring comorbidity in multiple sclerosis: A systemic review of current comorbidity scales

#### F. Föttinger ^1^; R. Hoepner^2^; M. Aicher^2^; G. Bsteh^1^

##### ^1^Medical University of Vienna, Department of Neurology, Vienna, Austria; ^2^University Hospital of Bern, Department of Neurology, Bern, Switzerland

**Background and Aims:** Comorbidity assessment in multiple sclerosis (MS) is crucial due to its impact disability progression and quality of life. However, comprehensive and systematic methods for assessing comorbidities in MS populations remain underexplored. This systematic review aimed to identify current comorbidity scales used in MS populations and assess their suitability for clinical implementation based on the quality of evidence.

**Methods:** Studies published between 2014–2024 that reported original data or systematic reviews on measures of comorbidity in patients with MS (pwMS) were included applying search terms “multiple sclerosis” and “comorbidity”. Information sources included MEDLINE, EMBASE, and the Cochrane Library. Quality of evidence was assessed by four raters using the GRADE approach.

**Results:** From 3,178 screened studies, 31 met inclusion criteria, analyzing data from 234,089 MS patients. Four comorbidity scales were identified: Charlson Comorbidity Index (CCI), Elixhauser Comorbidity Index (ECI), Self‐Administered Comorbidity Questionnaire (SCQ), and Self‐Reported Comorbidity Questionnaire for MS (SRQ‐MS). The CCI was most used and showed relevant associations with MS‐specific outcomes (e.g. increased risk of reaching disability milestones from CCI ≥1 [HR 1.23–1.62, *p* < 0.001]) but had moderate to low evidence quality due to limited validation and reliance on retrospective study designs. Similarly, the ECI and SCQ lacked direct validation and demonstrated very low evidence quality. The SRQ‐MS, validated for MS populations, showed moderate evidence quality but faced inconsistencies across studies.

**Conclusion:** Current comorbidity scales lack validation for routine use in MS populations. Further research is needed to develop and standardize MS‐specific comorbidity assessment for clinical and research purposes.

**Disclosure:** Nothing to disclose.

## EPO‐621

### Relationship between OND and ONSD measured by TOS and clinical, radiological, electrophysiological parameters in MS

#### G. Çakmakcı ^1^; M. Çetiner^2^; G. Akdağ^2^; Ş. Atlanoğlu^3^; S. Gültekin Irgat^4^; F. Akkoyun Arıkan^5^; S. Canbaz Kabay^6^

##### ^1^Gemlik State Hospital, Bursa, Turkey; ^2^Department of Neurology, Faculty of Medicine, Kutahya Health Sciences University, Kutahya, Turkey; ^3^Department of Radiology, Faculty of Medicine, Kutahya Health Sciences University, Kutahya, Turkey; ^4^Department of Ophthalmology, Faculty of Medicine, Kutahya Health Sciences University, Kutahya, Turkey; ^5^Park Hayat Hospital, Kutahya, Turkey; ^6^Dokuz Eylül University, Faculty of Medicine, Department of Neurology, Izmir, Turkey

**Background and Aims:** This study aimed to evaluate the reliability of optic nerve diameter (ONSD) and optic nerve sheath diameter (ONSD) measurements made with transorbital sonography (TOS) and MRI in patient groups that may progress with subclinical optic atrophy over time, such as MS.

**Methods:** A total of 102 patients (81 RRMS, 19 SPMS, 2 PPMS), and 34 healthy controls were included in the study. On the same day, each patient underwent TOS, orbital MRI, VEP, and OCT examinations for each eye.

**Results:** OND, ONSD, and OND/ONSD ratio measured by TOS were significantly lower in the MS patient group compared to the healthy controls (*p* < 0.001). Furthermore, in SPMS group, TOS measurements of OND and ONSD were significantly lower compared to the RRMS group (*p* < 0.001). In MS patients with an EDSS score >2, OND and ONSD measurements obtained by TOS were significantly lower (*p* < 0.001). A moderate positive correlation was found between the OND and ONSD measurements obtained by TOS and pRNFL thicknesses (G, T, TS, TI quadrants). No significant difference was found between the mean differences of OND and ONSD measurements obtained using TOS and MRI.

**Conclusion:** This study demonstrated the relationship and reliability of OND and ONSD measurements performed using TOS and MRI in patient groups that may exhibit subclinical optic atrophy over time, such as those with MS. These results indicate that OND and ONSD measurements obtained by TOS may be reliable methods for the early detection of disability and optic atrophy in SPMS patients, as well as those with RRMS.

**Disclosure:** Nothing to disclose.

## EPO‐622

### Longitudinal changes in sleep quality and their predictors in patients with multiple sclerosis

#### H. Yun ^1^; E. Lee^2^; D. Seo^2^; Y. Jin^1^; I. Jang^2^; L. Choi^1^; J. Kim^1^; W. Shin^1^; H. Lee^1^; S. Kim^1^; H. Jung^1^; J. Kim^1^; H. Kim^1^; Y. Lim^1^

##### ^1^University of Ulsan College of Medicine, Seoul, Republic of Korea; ^2^Department of Neurology, Asan Medical Center, University of Ulsan, Seoul, Republic of Korea

**Background and Aims:** Sleep disturbances are common in patients with multiple sclerosis (PwMS) and significantly impact quality of life (QoL). However, the longitudinal courses and clinical factors influencing these disturbances remain unclear.

**Methods:** PwMS in remission were prospectively recruited from a single tertiary medical center. Sleep quality was measured using the Pittsburgh Sleep Quality Index (PSQI) at baseline and after 6–12 months. Poor sleep was defined as a PSQI score >5, with significant worsening characterized by a score increase of ≥3. QoL and MS symptoms were evaluated using the EQ‐5D‐5L index and Functional Systems (FS) score. Factors associated with baseline and future PSQI scores were analyzed. Predictors of future PSQI scores and significant sleep changes were also investigated.

**Results:** Of 116 participants (mean age: 46 years), 54% and 53% were classified as poor sleepers at baseline and follow‐up, respectively. Poor sleep was independently associated with reduced QoL. Mean PSQI scores remained stable over follow‐up, while 24 (20.7%) patients experienced significant worsening. Baseline cerebral and optic FS scores independently predicted both baseline and future PSQI scores.

**Conclusion:** Sleep disturbances are common and significantly impactful in PwMS over time. Cerebral and optic dysfunction are key predictors, underscoring the need to prioritize targeted sleep care.

**Disclosure:** Nothing to disclose.

## EPO‐623

### Rituximab for the treatment of multiple sclerosis: A retrospective observational study of 50 cases from Morocco

#### J. Oumerzouk

##### Military Hospital Mohamed V of Rabat‐Morocco, Rabat, Morocco

**Background and Aims:** Rituximab (RTX) showed to be effective and relatively safe in the treatment of relapsing‐remitting and progressive forms of multiple sclerosis (MS), both in the phase II setting and in some observational studies.

**Methods:** We report a retrospective observational study to describe the effectiveness and safety of off‐label rituximab in the treatment of a population of Moroccan MS patients including 50 relapsing‐remitting (RRMS) and progressive multiple sclerosis (PMS) subjects.

**Results:** Our study showed that the RTX treatment was associated with the mean ARR decreasing by 0.72 at one year follow up. EDSS scores improved after 1 year of treatment with RTX by a score of 0.5‐1.0 in 31 (62%) patients and remained stable in the second year of therapy. EDSS score remained same in 12 patients (24%), of which 9 had RRMS and 3 SPMS. EDSS worsened after 2 years from RTX in 7 (14%) patients (5 SPMS, 2PPMS). Follow up MRI Brain with contrast at one year, show new T2 lesions in 6 patients (12%), with no enhancing lesions either old or new. Concerning safety issues in our patients, we observed a frequency of infusion associated reactions inferior to the data reported in other studies. Majority of patients (98%) tolerated RTX infusion well.

**Conclusion:** RTX could be an effective and safe treatment in RRMS. Some selected PMS patients could also benefit from this treatment.

**Disclosure:** Nothing to disclose.

## EPO‐624

### Primary histiocytic sarcoma of the central nervous system mimicking multiple sclerosis: A case report

#### L. Casado ^1^; A. Zabalza^2^; A. Vilaseca^2^; B. Rodriguez^2^; E. Martinez^5^; C. Auger^3^; H. Ariño^2^; L. Brieva^4^; X. Montalban^2^

##### ^1^Neurology Resident, Hospital Universitari Vall d’Hebrón, Universitat Autònoma de Barcelona, Barcelona, Spain; ^2^Department of Neuroimmunology CEMCAT, Hospital Universitari Vall d’Hebrón, Universitat Autònoma de Barcelona, Barcelona, Spain; ^3^Section of Neuroradiology, Hospital Universitari Vall d’Hebrón, Universitat Autònoma de Barcelona, Barcelona, Spain; ^4^Neurology Department, Hospital Universitario Arnau de Vilanova, Institut de Recerca Biomèdica de Lleida‐IRBLleida, Lleida, Spain; ^5^Department of Neuropathology, Hospital Universitari Vall d’Hebrón, Universitat Autònoma de Barcelona, Barcelona, Spain

**Background and Aims:** Primary histiocytic sarcoma (HS) affecting the central nervous system (CNS) is an exceptionally rare lymphohematopoietic tumour with poor prognosis due to challenging diagnosis, aggressive clinical course and lack of standardised treatment guidelines.

**Methods:** Case description of a patient who developed primary HS of the CNS.

**Results:** A 50‐year‐old man presented in July 2022 progressive blurred vision. MRI in January 2023 showed three white matter lesions (WML) highly suggestive of an inflammatory demyelinating disease, located in the left medial and right superior cerebellar peduncles and the right temporal periventricular. Cerebrospinal fluid analysis showed no pleocytosis, negative microbiology findings and positive oligoclonal bands. A diagnosis of multiple sclerosis (MS) was made, he was treated with intravenous methylprednisolone and started Ofatumumab in April 2023. After 2 months of treatment, the patient presented a progressive clinical decline with gait instability, diplopia, dysmetria, vomiting, anorexia and weight loss. A follow‐up MRI in June 2023 showed enlargement of the previously known lesions and new WML. Due to the atypical disease progression, a cerebral biopsy was made showing non‐necrotizing granulomas, leading to empirical treatment against Nocardia, Actinomycosis and Tuberculosis. Despite antibiotics, the disease progressed both clinically and radiologically. A second biopsy was performed, in which the morphological and immunohistochemical profile confirmed HS with TP53 and PMS2 mutated. Chemotherapy with the MATRIX protocol achieved partial response; however, the patient died from urinary sepsis complications.

**Figure:**
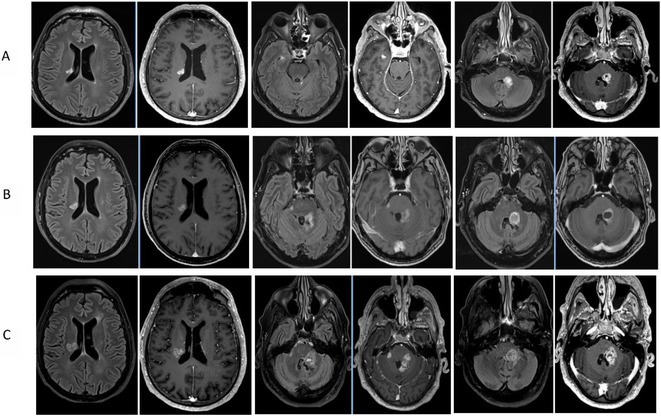
**FIGURE 1** Progression within the months of the WML located in right temporal periventricular region and cerebellar peduncles. Contrast‐enhanced T1 and T2‐FLAIR acquired in (A) September 2023, (B) November 2023 and (C) January 2024.

**Conclusion:** Despite fulfilling MS diagnostic criteria, cases with atypical disease progression despite appropriate treatment, should prompt consideration of alternative etiologies, including rare malignancies like primary CNS HS.

**Disclosure:** Nothing to disclose.

## EPO‐625

### Long‐term prognostic value of early NEDA‐3 vs NEDA‐4 status in relapsing‐remitting multiple sclerosis

#### M. Minetti ^1^; V. Bazzurri^2^; E. Tsantes^3^; F. Porpora^1^; R. Bersani^1^; F. Granella^1^; E. Curti^3^

##### ^1^Neurosciences Unit, Department of Medicine and Surgery, University of Parma, Parma, Italy; ^2^Neurology Unit, Emergency Department, Guglielmo da Saliceto Hospital, Piacenza, Italy; ^3^Neurology Unit, Department of General and Specialized Medicine, Parma University Hospital, Parma, Italy

**Background and Aims:** Aim of the study was to evaluate the long‐term prognostic value of maintaining NEDA‐3 and NEDA‐4 in the first two years of DMTs in people with recent diagnosis of RR multiple sclerosis.

**Methods:** All patients with early RRMS referred to Parma (IT) Centre between January 2015 and December 2018, taking DMT and with 5‐year follow‐up were included. Patients who stopped therapy within two years for reason other‐than‐efficacy were excluded. NEDA‐3 was the absence of relapses, MRI activity and disability progression. NEDA‐4 was NEDA‐3 plus annualized brain volume loss (SIENA) inferior to 0.4%.

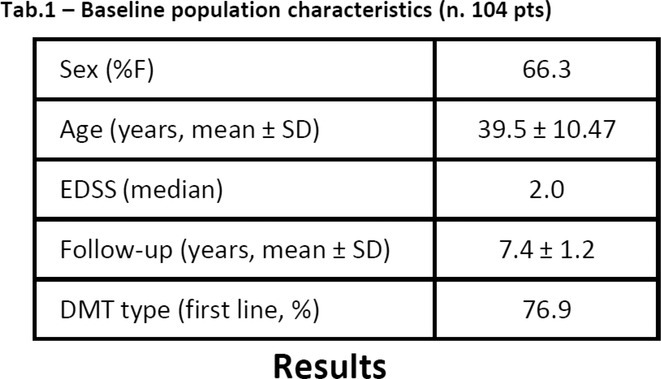

**Results:** Included 104 patients (66.3% female, mean age 39.5y, median EDSS 2.0, mean follow‐up 7.4y, first‐line DMT in 76.9%). Patients with 2‐year NEDA‐3 (35.6%) and NEDA‐4 (17.3%) had significantly less CDA events than non‐NEDA patients (NEDA‐3 24.3% and NEDA‐4 11.1% vs 41.8% non‐NEDA). CDA reduction was still more significant with 6md‐NEDA (6md‐NEDA‐3 22.6% and 6md‐NEDA4 10% vs 49% non‐NEDA). Correlation between CDA and NEDA status was more evident between RAW and NEDA‐3 (RAW 2.7% in NEDA‐3 vs 23.9% non‐NEDA) and between PIRA events and NEDA‐4 and 6md‐NEDA‐4 (PIRA 10% in NEDA‐4 vs 27.5 % non‐NEDA). NEDA‐4 outperformed NEDA‐3 in terms of CDA and PIRA events.

**Figure:**
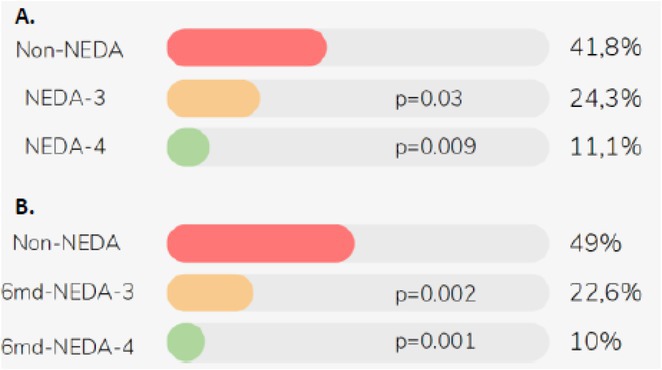
**FIGURE 1** CDA events related to NEDA (A) and 6md‐NEDA (B) status.

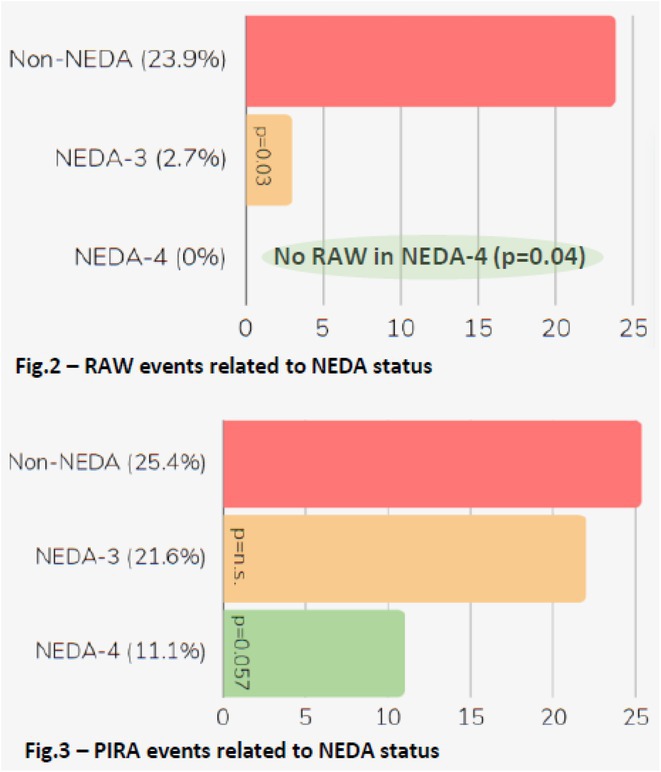

**Conclusion:** NEDA status has been proposed as long‐term prognostic factor in MS. In our work NEDA‐3 and NEDA‐4 in the two first years of DMT showed a protective role against CDA in the long term, NEDA‐4 exerting a stronger action, especially against PIRA. Our data underlined the importance of obtaining early NEDA‐status, especially NEDA‐4.

**Disclosure:** Nothing to disclose.

## EPO‐626

### Metabolomics of CSF and quantitative MRI data by AI based software Pixyl.Neuro.MS® in multiple sclerosis

#### M. Zido; P. Mikulenka; I. Stetkarova

##### Neurology department, 3rd Faculty of Medicine Charles University and University Hospital Kralovske Vinohrady, Prague, Czechia

**Background and Aims:** Metabolomics is a method of analytical chemistry to detect/measure presence of small molecules in investigated medium. Metabolomics of cerebrospinal fluid (CSF) may represent a new source of prognostic and/or diagnostic biomarkers of multiple sclerosis (MS). For example, histidine is significantly reduced in early stages of MS compared to controls. Aim of this study was to evaluate possible connection between amino/fatty acid concentrations in CSF and volume of MS specific brain lesions extracted by an AI based software.

**Methods:** We used clinical and paraclinical data of MS patients from our database as well as metabolomics results extracted from our previous study (1). We analysed brain MRI through AI software Pixyl.Neuro.MS® (1.8.X, Pixyl SAS, Grenoble, France) and extracted lesion volumes. We did correlation analyses among EDSS values, MRI lesion volumes and amino/fatty acid concentrations to judge their potential relationship.

**Results:** We have analysed metabolomic results, EDSS values and MRI data of 21 MS patients in early stages of their disease. We observed statistically significant correlation between lesion volumes and baseline EDSS (*r* = 0.65; *p* = 0.001) and EDSS after 1 year (*r* = 0.67; *p* = 0.002). We did not observe any significant correlation between concentrations of histidine, arginine, serine, choline, tyrosine, spermidine, glutamate, oleic, linoleic, palmitic acid and brain lesion volume.

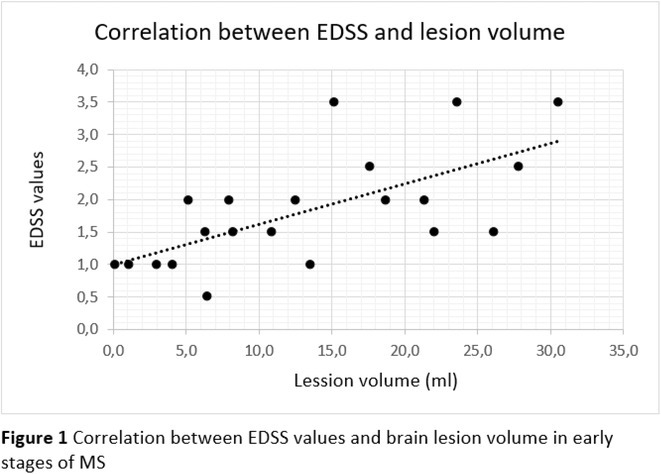

**Conclusion:** The volume of brain lesions in early stages of MS reflects the clinical state of patients in terms of EDSS values, but it did not show any significant connections with metabolomics results. However, it still needs further investigation.

**Disclosure:** Nothing to disclose.

## EPO‐627

### Intrathecal synthesis of Epstein‐Barr virus antibodies 15 years before multiple sclerosis onset. A case report

#### M. Guerrero‐Fernández ^1^; C. Serrano‐Alvárez^2^; J. Foronda‐Abengoa^3^; J. Gutiérrez‐Fernández^4^; A. Solozano‐Puerto^1^

##### ^1^Department of Microbiology, School of Medicine and PhD Program in Clinical Medicine and Public Health, University of Granada‐ibs, Granada, Spain; ^2^Department of Anesthesiology and Reanimation. University Hospital San Cecilio, Granada, Spain; ^3^Department of Neurology. University Hospital of Jaén, Spain; ^4^Laboratory of Microbiology, Virgen de las Nieves University Hospital‐ibs, Granada, Spain

**Background and Aims:** Epstein‐Barr virus (EBV) is considered the etiology of multiple sclerosis (MS). There is no consensus on the time between infection and clinical symptoms. A recent study has demonstrated that EBV infection previously was present in all cases of pediatric MS patients in the Netherlands, with b lood antibodies used as a screening and discriminating test.

**Methods:** A case‐control study was conducted in paired serum and CSF samples from 76 MS patients and 75 control subjects. The study participants were recruited between January 2002 and April 2005 at the San Cecilio University Hospital in Granada, Spain, for an EBV DNA and antibody testing. The control group included patients scheduled for minor surgery under epidural anesthesia with no previous neurological symptoms. A comprehensive review of a ll clinical records of MS patients as well as control patients in this study has been conducted over the past year.

**Results:** In the course of the study review, one patient recruited from the surgical control group was identified with a high titer of intrathecal EBV antibodies in a lumbar puncture performed in 2003. In 2017 this patient (a 46‐ year‐old female) consulted with paresthesias and Lhermitte·s sign as her first neurological symptoms. A brain MRI revealed demyelinating lesions disseminated in space with Gd enhancement. A CSF analysis revealed a leukocyte count 1 cell/ul. albumin 24.9 mg/dl, lgG 4 .43 mg/dl, and presence of oligoclonal bands.

**Conclusion:** When a diagnostic lumbar puncture is conducted for suspected demyelinating disease this test may be a valuable screening option.

**Disclosure:** Nothing to disclosure.

## EPO‐628

### Evaluation of treatment responses and prognosis in Marburg variant multiple sclerosis: A systematic review

#### O. Uwishema

##### Department of Research and Education, Oli Health Magazine Organization, Kigali, Rwanda

**Background and Aims:** Marburg variant multiple sclerosis (MS) represents an acute, fulminant form of MS, characterized by its aggressive nature, leading to significant disability or death. Due to its rarity, there is a scarcity of data regarding effective management strategies, highlighting the urgency for a comprehensive review of treatment outcomes.

**Methods:** A systematic literature review was conducted following PRISMA guidelines. Four databases (PubMed, Scopus, Embase, and Web of Science) were searched for case reports on Marburg variant MS up to September 2024. From an initial 1633 records, 18 studies were selected based on predefined inclusion and exclusion criteria. Data on demographics, clinical presentation, radiological findings, and therapeutic interventions were extracted and analyzed qualitatively.

**Results:** The review included 18 patients with Marburg variant MS, with a mean age of 34.9 years, predominantly female (88.9%). Lumbar puncture results showed lymphocytic pleocytosis in 71%, elevated protein in 64%, and positive oligoclonal bands in 50% of cases. MRI findings included hyperintense lesions on T2/FLAIR, most commonly in periventricular white matter. Treatment regimens involved high‐dose intravenous steroids (100%), plasmapheresis (67%), and cyclophosphamide (61%), followed by maintenance with monoclonal antibodies like ocrelizumab, rituximab, or alemtuzumab. Outcomes showed significant improvement in 78% of patients, complete remission in 11%, and a mortality rate of 11% within weeks of diagnosis.

**Conclusion:** This review underscores the potential for clinical and radiological improvement in Marburg variant MS with aggressive, multimodal treatment. However, the high early mortality rate emphasizes the critical need for swift and effective therapeutic interventions. Further research is needed to optimize treatment protocols.

**Disclosure:** Nothing to disclose.

## EPO‐629

### Gut microbiota alterations in young adults with relapsing‐remitting multiple sclerosis onset

#### S. Talapbek kyzy; T. Mikchail; S. Nikolay

##### RUDN University, Moscow, Russian Federation

**Background and Aims:** The gut microbiota functions as an independent organ influencing all body systems and contributing to various diseases. Its role in multiple sclerosis (MS) pathogenesis and the potential to modify the disease course via microbiota remain unclear. Objective: To evaluate gut microbiota composition in young individuals with relapsing‐remitting multiple sclerosis (RRMS) onset and compare it to a control group.

**Figure:**
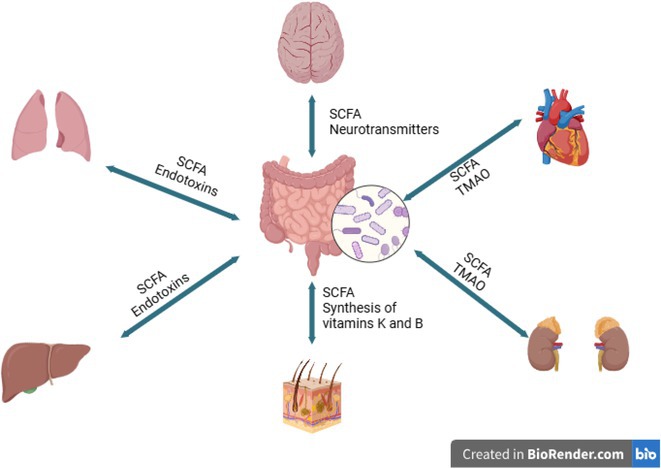
**FIGURE 1** Bidirectional gut microbiota‐organ relationships. SCFA – short‐chain fatty acids, TMAO – trimethylamine‐N‐oxide. Illustrated using the app https://app.biorender.com/.

**Methods:** The study included 14 RRMS patients and 14 healthy volunteers. Fecal samples were analyzed using mass spectrometry (Osipov method) to measure microbial markers, including fungi and viruses. Statistical analysis was conducted using Statistica 12 and Microsoft Excel. Normality was assessed via Kolmogorov‐Smirnov and Shapiro‐Wilk tests, and group differences were evaluated with the Mann‐Whitney *U*‐test (*p* ≤ 0.05).

**Results:** RRMS patients exhibited a significant predominance of Clostridium difficile and Propionibacterium spp. compared to healthy controls, with an absence of Kingella spp. and Pseudomonas aeruginosa. Fungal flora was quantitatively higher in RRMS patients, while the median Herpes spp. concentration was greater in healthy controls.

**Figure:**
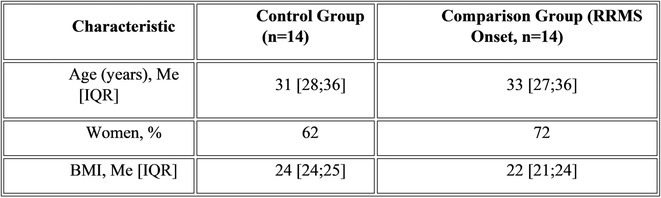
**TABLE 1** Demographic data of the Study Group.

**Figure:**
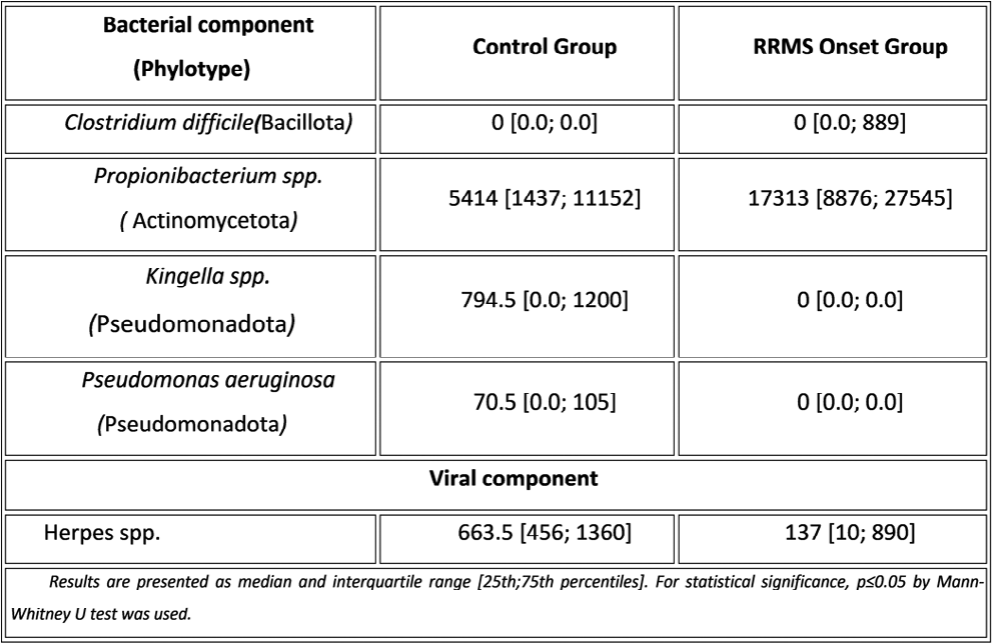
**TABLE 2** Concentration of Microbial Markers in Fecal Analysis in the Control Group and Patients with RRMS at Onset (105 cells/gram).

**Conclusion:** Significant differences were found in the bacterial, fungal, and viral components of the gut microbiota between RRMS patients and healthy individuals. However, further studies are needed to identify the “key microbiota” associated with MS and its potential as a therapeutic target.

**Disclosure:** Nothing to disclose.

## MS and related disorders 5

## EPO‐630

### Multiple sclerosis delayed diagnosis: Prevalence, causes and prognostic value

#### A. Belkina

##### Outpatient Department, Research Center of Neurology, Moscow, Russian Federation

**Background and Aims:** Over 83% of countries have barriers to early diagnosis of multiple sclerosis (MS), such as the lack of awareness of MS symptoms among patients, medical professionals and low availability of diagnostic tests. According to the Russian MS registry, only 36.2% patients were initially correctly diagnosed with MS.

**Methods:** We analyzed the prevalence and causes of diagnostic delay in 97 patients with MS, clinically isolated syndrome (CIS) and radiologically isolated syndrome (RIS) referred to the Research Center of Neurology from October 2023 to December 2024. We compared our data with the results the Russian MS registry, as well as studies conducted in Prague and in Egypt.

**Results:** The median time to diagnosis was 10 months, which is significantly longer than in Prague and Egypt. The rate of delayed diagnosis was 66%, which is 13% higher than in the Czechia study and 17% higher than in the Egypt. In the timely diagnosis group the median EDSS score was 2 whereas in delayed diagnosis group the median EDSS score was 3.

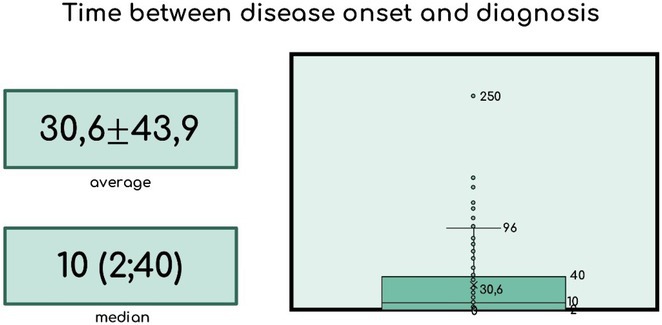

**Figure:**
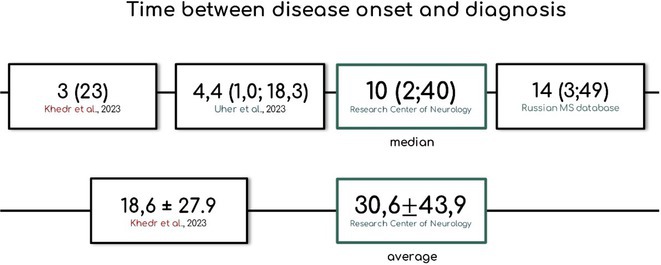
**FIGURE 1** Time between disease onset and diagnosis: comparison with other studies

**Figure:**
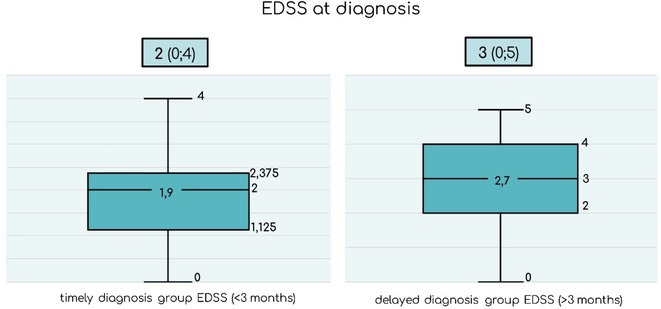
**FIGURE 2** EDSS at diagnosis depending on diagnostic delay

**Conclusion:** Thus, the time to diagnosis in the Russian data was significantly longer than in foreign studies. It can be suggested that the 1‐month achievable timeframe for MS diagnosis set by the consensus criteria is currently unrealistic. A diagnosis period of less than 3 months correlates with a lower degree of disability. The introduction of normative MS diagnosis timeframes into clinical guidelines could help reduce the frequency of delayed diagnosis and overcome one of the barriers to early DMT prescription.

**Disclosure:** Nothing to disclose.

## EPO‐631

### Tract density as additional marker of multiple sclerosis brain reorganization

#### A. Buniak ^1^; A. Mikitchuk^2^; O. Pereverzeva^1^

##### ^1^Neurological Department, Republican Research and Clinical Center, Minsk, Belarus; ^2^Faculty of Radiophysics and Computer Technologies, Belarusian State University, Minsk, Belarus

**Background and Aims:** The recent articles concerning tract‐wise disconnection and tract‐centered view in explaining multiple sclerosis (MS) disability are shown to be of great importance. Tract density (TD) is extensively studied as a potential MS marker. Statistical properties of TD distribution as a marker of brain reorganization for RRMS, PPMS, SPMS, active SPMS are analyzed.

**Methods:** MRI is performed with GE MR750w 3T as follows: resolution 2.0 mm (256x256), slice thickness 2.0 mm, 128 directions of b‐value 4000 s/mm^2^. Tractography is realized by DSI Studio with 4th Runge‐Kutta method, 100 million tracts are calculated without sub‐voxel interpolation. For each slice, the state of disorder is evaluated with Shannon entropy, entropy values over all slices are summarized.

**Results:** SPMS (dieses duration (DD) 15y) is shown to be characterized by the lowest TD entropy values (fig. 1), both sum and distribution in the space of brain (fig. 2): RRMS (DD 3y) is characterized by TD higher entropy, which sum exceeds on more than 50% the SPMS one; PPMS (DD 5y) is characterized by higher TD entropy, which sum exceeds on more than 20% the RRMS case; ASPMS (DD 14y) is shown to be characterized by the highest TD entropy, which sum exceeds on more than 20% the PPMS one (fig. 1,2).

**Figure:**
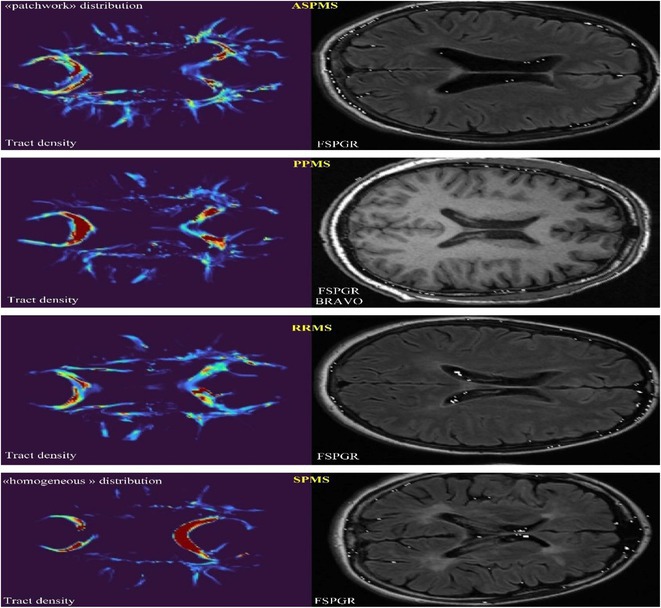
**FIGURE 1** TD entropy values in different forms of MS

**Figure:**
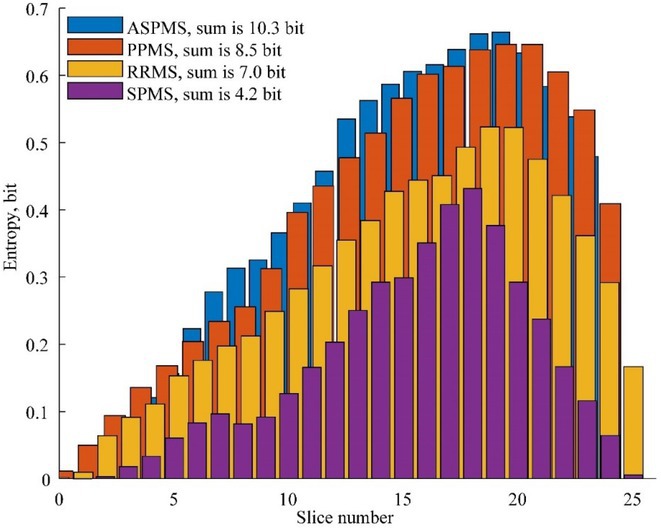
**FIGURE 2** Sum and distribution TD entropy in the space of brain in different forms of MS

**Conclusion:** Low state of TD disorder in the case of SPMS indicates the lowest brain reorganization, intensity of brain reorganization increases in the following order: RRMS, PPMS, ASPMS, all of them are characterized by «patchwork» TD distribution. Further studies are required.

**Disclosure:** Nothing to disclose.

## EPO‐632

### Metacognition in multiple sclerosis: Assessing MCQ‐30 reliability and its clinical implications

#### A. Rienzo ^1^; S. Cuoco^1^; R. Capuano^2^; S. Scannapieco^2^; M. Consalvo^1^; C. Giordano^1^; M. Rotolo^1^; U. De Marca^1^; A. D'Amico^1^; M. Pellecchia^1^; P. Barone^1^; M. Di Gregorio^2^

##### ^1^Department of Medicine, Surgery and Dentistry “Scuola Medica Salernitana”, University of Salerno, Salerno, Italy; ^2^Neurology Clinic, Medical Sciences Departement, AOU San Giovanni di Dio e Ruggi d’Aragona, Salerno, Italy

**Background and Aims:** Metacognition, the ability to monitor and regulate thoughts, emotions and behaviors, plays a crucial role in psychological health. Its dysfunction is generally associated with higher levels of depression, anxiety and fatigue in the general population. This study aims to explore the relationship between Metacognition Questionnaire (MCQ‐30) and its five subscales (PositiveBeliefsAboutWorry [POS], NegativeBeliefsAboutUncontrollabilityAndDanger [NES], CognitiveConfidence [CC], Need To Control Thoughts [NC], CognitiveSelfConsciousness [CSC]) with these symptoms in patients with Multiple Sclerosis (PwMS).

**Methods:** Sixty‐five cognitively preserved PwMS underwent clinical (EDSS) and psychological evaluations (MCQ‐30, Beck Depression Inventory [BDI], State‐Trait‐Anxiety‐Inventory [STAI], Fatigue Severity Scale [FSS]). Spearman's correlations were calculated between the MCQ‐30 total score (MCQ‐30‐ts), subscales and individual items. Gender differences and convergent validity with other psychological scales were also analyzed.

**Results:** Clinical and demographic data are shown in Tab.1. The internal consistency of MCQ‐30 and its subscale was high, except for CSC (Tab.2). No gender differences were found in MCQ‐30‐ts or its subscales. Significant correlations were observed between the MCQ‐30‐ts and all items, except items 5 and 12 (rho: 0.26–0.71). All subscales correlated with all items: POS (rho > 0.55, *p* < 0.05), NES (rho > 0.55, *p* < 0.05), CC (rho > 0.79, *p* < 0.05), NC (rho > 0.49, *p* < 0.05) and CSC (rho > 0.41, *p* < 0.05). The MCQ‐30‐ts and all subscales showed significant intercorrelations (coefficient between 0.41–0.82). As shown in Tab.3, MCQ‐30‐ts, POS, NES, CC and NC correlated with STAI, BDI and FSS while MCQ‐30‐ts and CC also correlated with EDSS.

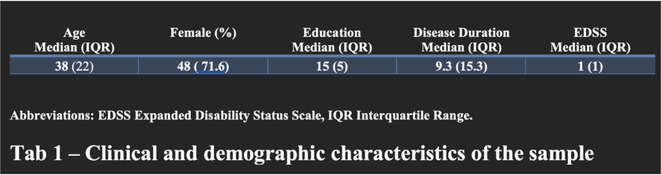

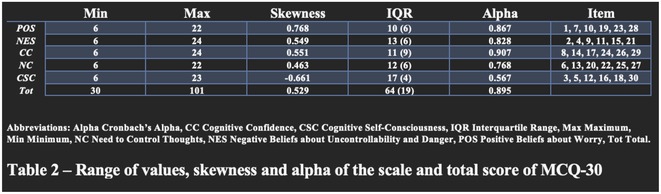

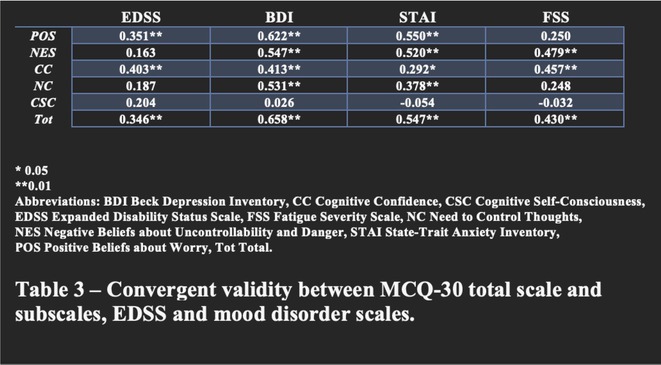

**Conclusion:** The MCQ‐30 is a reliable and valid tool for assessing metacognition in PwMS. Increased metacognitive dysfunction is associated with worsening symptoms of anxiety, depression and fatigue.

**Disclosure:** Quattropani, M. C., Lenzo, V., Mucciardi, M., and Toffle, M. E. (2014). Psychometric properties of the Italian version of the short form of the metacognitions questionnaire (MCQ‐30). BPA Appl. Psychol. Bull. 62.

## EPO‐633

### The effect of dual‐task on upper extremity functions in multiple sclerosis patients

#### A. Çakmakci ^1^; E. Okur^1^; S. Canbaz Kabay^2^

##### ^1^Kutahya Health Sciences University, Kütahya, Turkey; ^2^Dokuz Eylul University, İzmir, Turkey

**Background and Aims:** Multiple Sclerosis is a neurodegenerative, chronic, autoimmune disorder of the central nervous system and affects daily living activities. Daily living activities often require dual‐task performance. Assessments that include dual tasks can show the impairments in PwMS' daily life. Although upper extremity dysfunctions are frequently seen in PwMS, research on the effect of dual‐tasking on upper extremity functions and the dual‐task interference is limited. This study aimed to investigate the effect of dual‐tasking on upper extremity functions in PwMS and compare it with HC.

**Methods:** This case‐control design study was conducted with 30 PwMS and 30 HC. Age and sex‐matched controls were also randomly selected from the general population. The Edinburgh Handedness Questionnaire measured participants' hand preferences. Disability status is assessed using the EDSS, and cognitive status is assessed using the BICAMS. MMDT was used to evaluate the upper extremity function of the participants. The DTQ was used to evaluate the difficulties experienced during dual‐task.

**Results:** There was no difference between single‐task placement times, single‐task turning times, dual‐task turning times, and dual‐task effects of PwMS and HC (*p* > 0.05). However, it was found that there was a statistically significant difference between the verbal fluency and turning errors made by PwMS and HC during dual‐task performance and the dual‐task questionnaire scores (*p* < 0.05).

**Conclusion:** The results of this study demonstrate that PwMS exhibits upper extremity performance similar to healthy controls under dual‐task conditions. This finding provides reassurance about the potential for PwMS to lead everyday lives.

**Disclosure:** Nothing to disclose.

## EPO‐634

### Cortical excitability and cognitive impairment in individuals with multiple sclerosis

#### C. Siniscalchi; L. Mollo; R. Dubbioso; F. Manganelli; R. Iodice

##### Department of Neurosciences, Reproductive Sciences and Odontostomatology University of Naples Federico II Naples, Italy

**Background and Aims:** This study investigates cortical excitability and cognitive impairment in individuals with Multiple Sclerosis (MS) compared to matched healthy controls, aiming to explore their interrelationship and implications for disease pathology and management.

**Methods:** We assessed 44 individuals diagnosed with MS and a matched control group of 44 healthy participants. Cortical excitability was measured using transcranial magnetic stimulation (TMS), while cognitive performance was evaluated using standardized neuropsychological tests targeting memory, attention, and executive function. Demographic and clinical variables, including disease duration and disability scores, were analyzed to control for potential confounders.

**Results:** Participants with MS exhibited significantly altered cortical excitability parameters, including reduced motor threshold and increased intracortical facilitation, compared to healthy controls (*p* < 0.05). Cognitive assessments revealed deficits in attention, memory, and executive functioning among the MS group, with moderate to strong correlations observed between cortical excitability measures and cognitive performance (*r* = 0.4–0.6, *p* < 0.01). These relationships persisted after adjusting for age, education, and disease severity.

**Conclusion:** This study highlights the interplay between cortical excitability and cognitive impairment in individuals with MS, suggesting a potential neurophysiological basis for cognitive deficits in this population. These findings underscore the importance of integrating neurophysiological assessments into clinical evaluations and exploring interventions targeting cortical excitability to mitigate cognitive decline in MS.

**Disclosure:** Nothing to disclose.

## EPO‐635

### Discontinuation of disease‐modifying therapies in patients with multiple sclerosis: A retrospective case‐control study

#### G. Cabañas Engenios ^1^; N. Mena García^1^; P. Garay Albízuri^1^; A. Llanes Ferrer^1^; A. Sánchez Asensio^1^; J. Romero Ferro^1^; L. Gil Martínez^1^; M. Campos Jiménez^1^; R. Pastor González^1^; F. Rodríguez Jorge^1^; S. Sainz De la Maza^1^; E. Monreal Laguillo^1^; L. Villar^2^; J. Masjuan Vallejo^1^; L. Costa‐Frossard^1^; J. Chico García^1^

##### ^1^Neurology Department, Ramón y Cajal University Hospital, Madrid, Spain; ^2^Immunology Department, Ramón y Cajal University Hospital, Madrid, Spain

**Background and Aims:** Currently, there is no consensus on the strategies of discontinuation of disease‐modifying therapy (DMT) in multiple sclerosis (MS) patients. This study evaluates confirmed disability progression (CDP) and evidence of disease activity‐3 (EDA‐3) after one year of DMT discontinuation compared with a control group.

**Methods:** Single‐centre retrospective observational case‐control study, including patients diagnosed with MS who had discontinued DMT and had a minimum follow‐up of one year (June 2013‐December 2024). Controls without DMT discontinuation were selected from a prospectively collected MS patients database by propensity score matching by age, sex, MS type and DMT. Controls exclusion criteria included less than one year follow‐up and no clinical or radiological stability for at least 3 years. We classified patients according to VIAADISC scale. This scale predicts the risk of activity after DMT discontinuation according to age, and clinical and MRI data.

**Results:** After propensity score matching, 32 patients who discontinued DMT and 44 controls who did not discontinue DMT were included. No statistically significant differences were found between the two groups regarding age (median 54.2 vs 51.3 years), sex, MS phenotype, DMT, EDSS and VIAADISC score. After one year, there were no significant differences between the two groups in terms of CDP (OR 0.55, 0.07‐2.85, *p*‐value = 0.69) and EDA‐3 (OR 1.05, 0.37‐2.92, *p*‐value = 0.6). There were no differences regarding new MRI lesions (*p*‐value = 0.18), although slightly more relapses in the control group were observed (*p*‐value = 0.04).

**Conclusion:** Discontinuation of DMT in selected MS patients showed no higher risk of disease activity and disability accumulation after one year.

**Disclosure:** Nothing to disclose.

## EPO‐636

### Myelin oligodendrocyte glycoprotein antibody‐associated disease manifesting as idiopathic intracranial hypertension

#### H. Doopadahalli Sathyanarayana ^1^; M. Ahmed Gamea^1^; A. Ghassana^1^; G. Anwar^2^; Y. Mousa^3^; S. Base^1^; T. Joseph^1^

##### ^1^Department of Neurology, Burjeel Hospital, Abu Dhabi, UAE; ^2^Department of Ophthalmology, Burjeel Hospital, Abu Dhabi, UAE; ^3^Department of Radiology, Burjeel Hospital, Abu Dhabi, UAE

**Background and Aims:** This case report highlights the diagnostic challenges in a female presenting with mild visual disturbances and headache, initially diagnosed with idiopathic intracranial hypertension (IIH) but later identified as having Myelin Oligodendrocyte Glycoprotein Antibody‐Associated Disease (MOGAD).

**Methods:** A 37‐year‐old woman with no significant medical history presented with a week‐long history of visual disturbances, bifrontal headaches, and pain associated with eye movement Ophthalmological assessment revealed bilateral papilledema without focal neurological deficit. Her MRI and MRV of the brain were normal. Given the clinical suspicion IIH, the patient was recommended for a lumbar puncture, which she declined. Consequently, treatment with acetazolamide. During her follow‐up, an acute exacerbation of her visual disturbances was observed Anti‐MOG antibodies was positive, which was pivotal in confirming the diagnosis of MOGAD. The patient received intravenous methylprednisolone for five days, resulting in significant symptom relief for six months. However, she later developed right‐sided facial numbness repeat MRI, which revealed enhancement of the right trigeminal nerve and a small enhancing lesion in the right pontine region, without evidence of associated mass effect.

**Figure:**
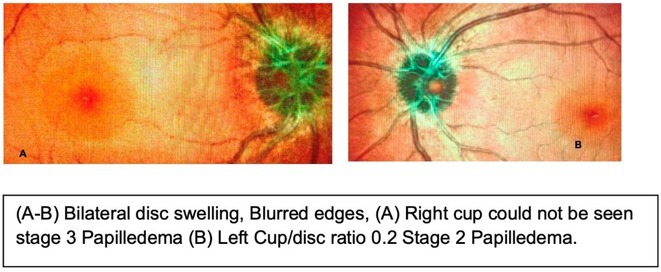
**FIGURE 1** Fundus Pic

**Figure:**
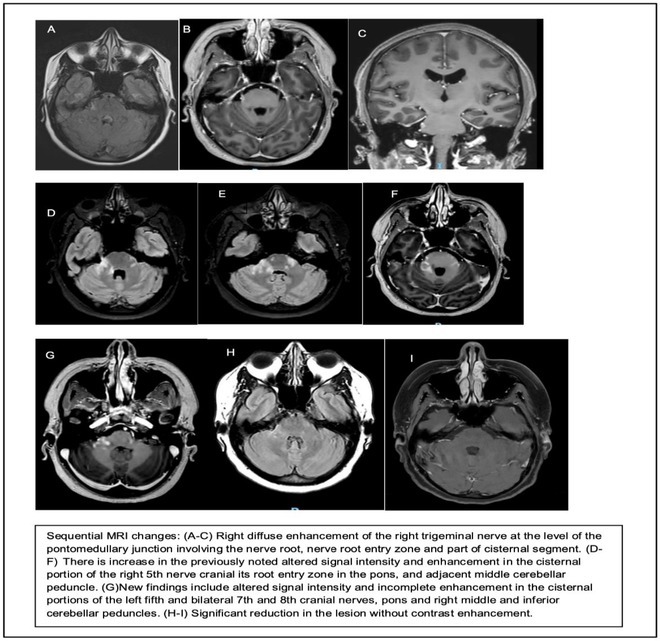
**FIGURE 2** MRI Brain

**Results:** The combination of clinical findings, imaging results, and laboratory data led to the diagnosis of MOGAD. The patient received five additional doses of IVMP, and oral steroids complemented by the initiation of azathioprine as a disease‐modifying therapy.

**Conclusion:** This case illustrates the necessity of a broad differential diagnosis in patients presenting with visual disturbances and papilledema. A comprehensive and multi‐disciplinary approach is essential for the effective management of MOGAD, ensuring accurate diagnostics and improved patient outcomes.

**Disclosure:** Nothing to disclose.

## EPO‐637

### Efficacy of eculizumab and pharmacogenetic modulation by rs17611 in Chinese AQP4‐IgG‐positive relapsing neuromyelitis optica spectrum disorder: Implications for precision dosing

#### L. Zou; F. Li; B. Sun; H. Yang; J. Li; S. Chen; Y. Yong

##### Department of Neurology, Xiangya Hospital Central South University, Changsha, China,Hospital of Xinjiang Medical University, Urumqi, China

**Background and Aims:** Complement‐mediated pathogenesis underpins Neuromyelitis Optica Spectrum Disorders (NMOSD), with C5‐driven membrane attack complex (MAC) formation being a therapeutic target. Eculizumab, a humanized anti‐C5 monoclonal antibody, has been shown to significantly reduce relapse risk by blocking terminal complement activation. However, emerging evidence suggests that genetic polymorphisms modulating C5 cleavage dynamics—particularly rs17611, a risk allele enhancing neutrophil elastase (HNE)‐mediated proteolysis into dysfunctional C5a/C5b variants—may critically alter MAC assembly efficiency. This study bridges a critical knowledge gap by evaluating eculizumab's clinical efficacy and its interplay with rs17611 pharmacogenetics in Chinese AQP4+ NMOSD patients, aiming to refine personalized therapeutic paradigms.

**Methods:** In this prospective observational study, five AQP4‐IgG‐seropositive NMOSD patients experiencing acute attacks received eculizumab at Xiangya Hospital, Central South University (August 2024–March 2025). Neurological recovery was longitudinally quantified using the Expanded Disability Status Scale (EDSS), while rs17611 genotyping was performed via whole‐exome sequencing. Genotype‐phenotype correlations were analyzed using non‐parametric statistics (Mann‐Whitney U test).

**Results:** rs17611 C>T variant carriers (*n* = 2) exhibited superior EDSS improvement (median reduction: 1.5 points, range: 1.0–2.0) versus wild‐type patients (*n* = 3; median: 1.0 point, range: 1.0–1.5; *p* = 0.042). The rs17611 C>T variant group (*n* = 2) exhibited notable decreases in complement C3 (Δmedian = −32.5%), IgA (Δmedian = −23.9%), and IgM (Δmedian = −63.4%) following treatment, while wild‐type controls (*n* = 3) demonstrated increases in complement C4 (Δmedian = +54.1%) and C3 (Δmedian = +20.0%). No treatment‐related infections or mortality occurred, confirming short‐term safety.

**Figure:**
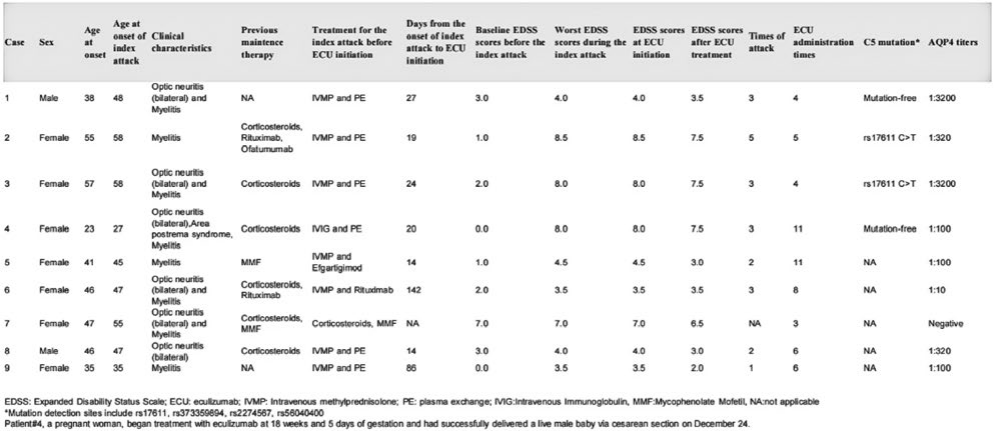
**TABLE 1** Demographic, baseline characteristics and changes in EDSS scores.

**Conclusion:** We pioneer the discovery that rs17611—a putative risk allele in NMOSD pathogenesis—paradoxically predicts enhanced therapeutic response to eculizumab, likely through attenuated MAC formation via HNE‐dominant C5 cleavage. This dual‐context genetic effect challenges conventional pharmacogenetic frameworks and unveils a novel biomarker for precision dosing in NMOSD. Crucially, our findings advocate for genotype‐guided escalation protocols in Asian populations, warranting validation in larger cohorts and mechanistic studies on C5 fragment bioactivity.

**Disclosure:** Approved by the Institutional Review Board of Xiangya Hospital. All participants provided written informed consent with anonymized data handling, adhering to the Declaration of Helsinki.

## EPO‐638

### The real‐world effectiveness of natalizumab in relapsing‐remitting multiple sclerosis in Algeria

#### N. Hecham ^1^; O. Meheni^2^; S. Benhamada^3^; S. Bourokba^4^; N. Lakri^5^; N. Oudrer^1^; A. Aidi^1^; S. Nouioua^6^; N. Toubal^4^; A. Fekraoui^3^; M. Tazir^7^

##### ^1^Department of Neurology Oran Hospital, Algeria; ^2^Department of epidemiology, Mustapha Bacha Hospital, Algiers, Algeria; ^3^Department of Neurology Ibn Badis Hospital, Constantine, Algeria; ^4^Department of Neurology Annaba; ^5^Department of Neurology Ain Naadja Hospital, Algiers, Algeria; ^6^Department of Neurology El Maham Hospital, Cherchell, Algeria; ^7^Neuroscience Laboratory, Algiers University, Algeria

**Background and Aims:** Natalizumab therapy for MS patients has been demonstrated to be highly effective in several clinical trials. Data in Algerian population are needed on long‐term effect of natalizumab (NTZ) in relapsing‐remitting multiple sclerosis (RRMS).

**Methods:** We conducted a multicentre, observational and retrospective study at five department of neurology reported RRMS patients who initiated natalizumab ‡12 months prior to study conduction.

**Results:** 167 patients were included, with a mean age of 26.1 ± 7.6 years at diagnosis, and a mean age of 31.8 ± 9.4 years at NTZ initiation. The median number of infusions was 36. The Mean EDSS scores remained unchanged up to 3 years. It was 3.7 ± 1.6 (range, 0–5, median 4) at baseline and it was 3.4 ± 2.1 (range, 0–5, median 4) at the follow‐up. The mean annualized relapse rate (ARR) previous, during and after NTZ was 2.3 ± 1.4, 0.4 ± 0.6 and 0.3 ± 0.5, respectively. 52.7% of patients remained relapse free and reached NEDA3 at 3 years. RIO 3 at 3 years was also achieved in 67% of cases. One case of progressive multifocal leucoencephalopathy was reported. Patients with an EDSS of 4 at the start of treatment had a better outcome, with a lower annual relapse rate, stabilisation of disability and no new T2 lesions on MRI.

**Conclusion:** Our data confirm natalizumab's overall safety profile and the low relapse rate and stabilised disability levels in Algerian RRMS patients treated with natalizumab in clinical practice.

**Disclosure:** Nothing to disclose.

## EPO‐639

### Assessment of multiple sclerosis awareness and knowledge among Saudi population

#### N. Albekairy

##### King Saud bin Abdulaziz University for Health Sciences (KSAU‐HS), Riyadh, Saudi Arabia

**Background and Aims:** Although the Middle East region falls in the low‐to‐moderate multiple sclerosis (MS) prevalence zone, a lack of knowledge about MS symptoms may cause patients to miss the opportunity to reap the benefits of early interventions. This study examines the awareness and knowledge of MS in Saudi population to help better build targeted campaigns that aid in early interventions.

**Methods:** We did a convenience sample cross sectional study, assessing people's knowledge on MS from various regions of Saudi Arabia using a previously validated questionnaire.

**Results:** A total of 544 individuals have completed the survey. Majority (81.8 %) of participants ages fall between 18 and 45 years old, and females made up 45.2% of the sample. Also, over half of the participants are college students. According to our survey analysis, 72.6% had previously heard about the disease mainly through social media platforms (56.1%). Moreover, only 29% of participants knew someone with MS. Nevertheless, the majority (90.6%) believed that MS sufferers required support, and 53.3% were able to correctly answer questions on its symptoms. However, less than one third knew about the different treatment modalities. Also, majority (89.5%) believed that MS patients have a compromised professional life; and over half of the participants agreed that it is linked to depression.

**Conclusion:** The Saudi public's understanding of Multiple Sclerosis as a disease, its prevalence, and its treatment remains inadequate. However, they are slightly aware of certain elements in the condition's pathophysiology. Nevertheless, programs to raise public awareness should help increase MS knowledge in Saudi Arabia.

**Disclosure:** Nothing to disclose.

## EPO‐640

### Migraine and tension‐type headache are associated with multiple sclerosis: A case‐control study

#### P. Gklinos ^1^; M. Evangelopoulos^1^; G. Velonakis^2^; D. Mitsikostas^1^

##### ^1^First Department of Neurology, Eginition UniversityH ospital, National and Kapodistrian University of Athens, Athens, Greece; ^2^Research Unit of Radiology and Medical Imaging, National and Kapodistrian University of Athens, Athens, Greece

**Background and Aims:** Over the past decades there has been increased scientific interest in the prevalence of headache disorders among people with MS (pwMS). Although the latest data suggest an association between migraine and multiple sclerosis, studies have been providing inconsistent results largely due to methodological shortcomings. The aim of this study is to investigate the prevalence of primary headache disorders in pwMS and healthy controls (HCs) and examine the potential association between the two conditions.

**Methods:** Ninety‐six pwMS from Eginition University Hospital, Athens, Greece and 96 matched HCs were recruited prospectively. Both groups were assessed for headache disorders using a semi‐structured questionnaire and diagnosed according to the International Classification for Headache Disorders 3 (ICHD‐3) criteria. A multivariable logistic regression model adjusted for age and sex, evaluated the association between MS and headache disorders.

**Results:** A higher prevalence of primary headache disorders in pwMS (71.9%) compared to HCs (43.8%) was observed. Specifically, 28.1% of pwMS had migraine, and 38.5% had tension‐type headache (TTH). PwMS were significantly more likely to be diagnosed with any primary headache disorder (OR = 4.54; 95% CI: 2.28 to 9.04; *p* = 1.7), migraine (OR = 2.21 95% CI: 1.05 to 4.62; *p* < 0.05), and TTH (OR = 2.16 95% CI: 1.16 to 4; *p* < 0.05) compared to HCs.

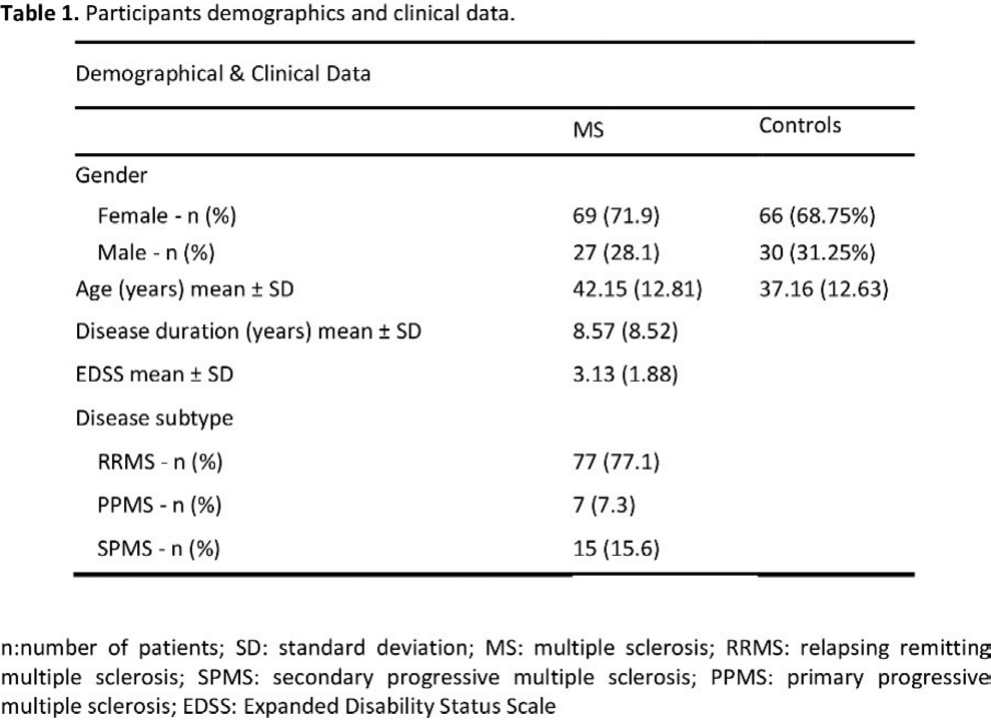

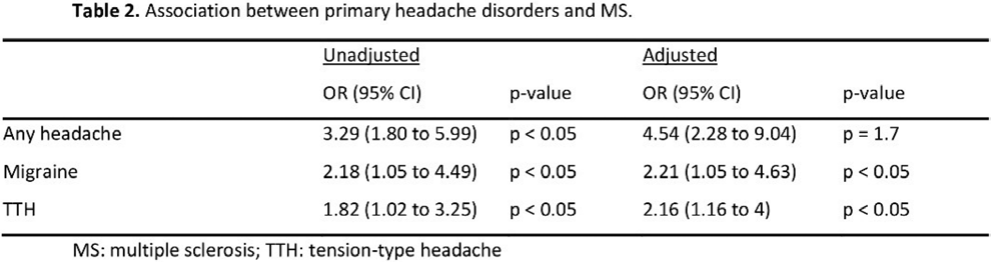

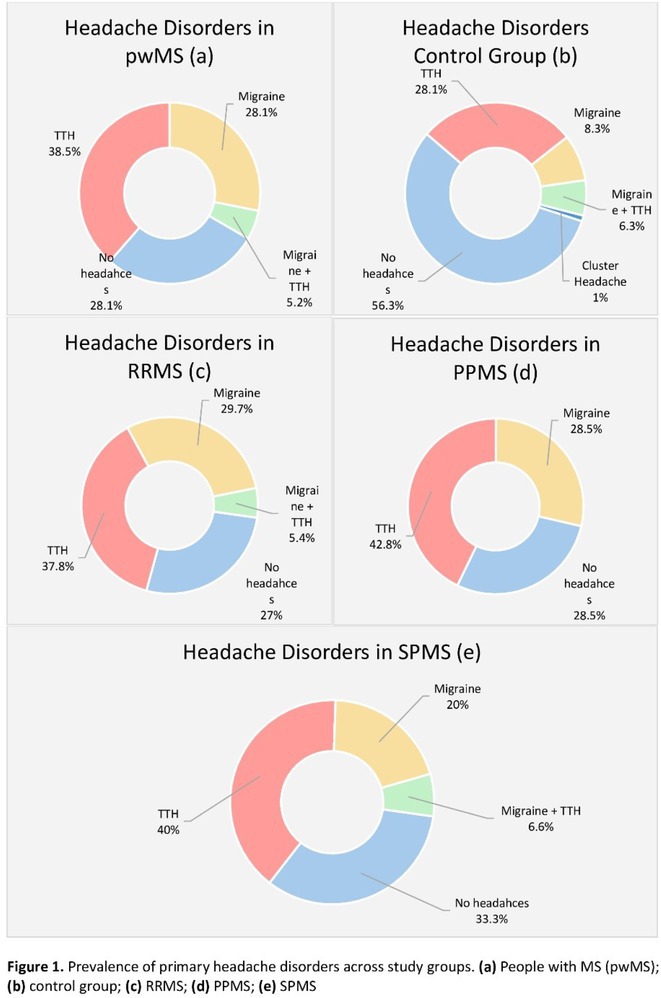

**Conclusion:** Our study suggests a strong association of primary headache disorders and MS suggesting that MS‐related changes may increase susceptibility to headache and highlights the need for targeted headache management in pwMS. Prospective longitudinal studies are needed to draw more robust conclusions.

**Disclosure:** Nothing to disclose.

## EPO‐641

### Education on the rationale for BTK inhibitors significantly improves knowledge, confidence, and intention to learn more

#### S. Rohani‐Montez ^1^; L. Fairley; B. J. Hon^1^; P. Guedj^1^; V. Popescu^2^

##### ^1^Medscape Education Global. London, UK; ^2^Hasselt University, Pelt, Belgium

**Background and Aims:** Neurologists are unfamiliar with Bruton tyrosine kinase inhibitors (BTKis) and their relevance for future MS practice. We sought to improve knowledge of the rationale for BTKis in MS by providing expert‐led education.

**Methods:** Neurologists participated in an online CME divided into 6 video + slide segments with synchronized slides.1 The education effects were assessed using a 3‐question, repeated pairs, pre‐assessment/post‐assessment study design. One question assessed confidence. Differences pre‐ to post‐assessment were evaluated using McNemar's test. *p* ≤ 05 is significant. The activity launched in June 2024; data were collected over 8 weeks.

**Results:** 598 neurologists participated, with 78 completing all pre‐ and post‐assessment questions. Significant overall improvements were seen, with a 43% correct response rate pre‐assessment vs 63% post‐assessment; *p* < 0.001, *N* = 78. Specifically, significant improvements were observed in knowledge that BTK inhibition within the CNS has a pharmacological effect on B cells and microglia; and that T1‐ and T2‐weighted MRI can be used longitudinally to measure slowly expanding lesions, which may be driven by smoldering inflammation. After participating, 45% of neurologists had measurable improved confidence in describing the role of BTK inhibition within the CNS. 47% of neurologists said they intend to learn more about MS pathophysiology, and 19% said they would learn more about BTK inhibition in MS.

**Conclusion:** This study demonstrates the success of online, multi‐component, multi‐faculty, video‐based CME in improving knowledge and intention to acquire more knowledge about a new class of therapy for MS. Improved knowledge on BTKis should result in increased confidence in their future implementation of these therapies.

**Disclosure:** Nothing to disclose for all authors except Veronica Popescu, MD, PhD, has the following relevant financial relationships: Consultant or advisor for: Almirall; Biogen; Medtronic, Inc.; Merck; Novartis; Roche; Sanofi‐Genzyme; Teva Pharmaceutical Industries Ltd. Speaker or member of speakers bureau for: Biogen; Merck; Novartis; Roche; Sanofi‐Genzyme.

## EPO‐642

### Cladribine impacts on clinical, radiological, cognitive and physical assessments of people with multiple sclerosis

#### S. Ozakbas ^1^; E. Kaya^2^; Y. Simsek^1^; A. Ozdogar^3^

##### ^1^Izmir University of Economics, Medical Point Hospital, Izmır, Turkey; ^2^Dokuz Eylul University, Faculty of Medicine, Neurology, Izmır, Turkey; ^3^Van Yuzuncu Yıl University, Faculty of Health Sciences, Physiotherapy, Van, Turkey

**Background and Aims:** This abstract comprehensively evaluates the efficacy and safety of cladribine treatment in MS, focusing on cognitive and physical outcomes besides clinical outcomes.

**Methods:** A cohort of participants diagnosed with MS underwent treatment with cladribine (n = 173), with cognitive and physical assessments (n = 32) included in the study. The physical and cognitive assessments were conducted at baseline (T0) and follow‐up (first year = T1, second year = T2). Cognitive function was evaluated using the Brief International Cognitive Assessment for Multiple Sclerosis (BICAMS) battery, which included the California Verbal Learning Test (CVLT), Brief Visuospatial Memory Test‐Revised (BVMTR), and Symbol Digit Modalities Test (SDMT) while physical function was assessed through the Timed 25‐Foot Walk (T25FW) and Nine‐Hole Peg Test (N‐HPT). The Expanded Disability Status Scale (EDSS), disease duration, age, presence of new attacks and the new MRI findings of the participants were recorded.

**Results:** The mean age and disease duration were 36.75 ± 11.44 and 8.33 ± 6.85. Results revealed significant improvements in CVLT, BVMTR, and SDMT tests at the follow‐up interval. However, no significant differences were observed in N‐HPT. Fourteen participants had new attacks, while sixteen participants had new MRI findings.

**Conclusion:** These findings suggest that cladribine treatment is beneficial and safe for cognitive function in pwMS. However, further research is warranted to elucidate its impact on physical function. The significant efficacy demonstrated by cladribine treatment in treating MS is complemented by its favourable safety profile, as evidenced by the small number of new relapses and the emergence of new MRI findings.

**Disclosure:** Nothing to disclose.

## EPO‐643

### Maraviroc‐responsive progressive multifocal leukoencephalopathy‐associated immune reconstitution inflammatory syndrome

#### S. Canbaz Kabay ^1^; G. Çakmakcı^2^; G. Akdağ^3^; M. Çetiner^3^

##### ^1^Dokuz Eylül University, Faculty of Medicine, Department of Neurology, Izmir, Turkey; ^2^Gemlik State Hospital, Bursa, Turkey; ^3^Department of Neurology, Faculty of Medicine, Kutahya Health Sciences University, Kutahya, Turkey

**Background and Aims:** PML is an opportunistic infection of the brain caused by the human polyomavirus 2. Overall, PML is associated with severe disability and a relatively high mortality.

**Methods:** A 45‐year‐old male patient who had been receiving natalizumab therapy for MS for 3 years presented with complaints of apathy, disorientation, and weakness in the left arm and leg for 10 days.

**Results:** Brain MRI revealed lesions consistent with PML. The serum anti‐JCV index was 3.93, CSF protein 37.5 mg/dl, and CSF JCV DNA 621 copies/ml. The patient was evaluated as consistent with a preliminary diagnosis of PML and underwent plasmapheresis. Following clinical and radiological progression, the patient was treated with 1 g/day of intravenous methylprednisolone under the preliminary diagnosis of IRIS. Based on the diagnosis of PML and PML‐associated IRIS, peroral maraviroc at a dose of 2x300 mg was initiated. Radiological and clinical improvement were observed. At the 6th month of maraviroc treatment, the CSF JCV index decreased to 10 copies/ml, and no JCV DNA was detected in the CSF at 1 year.

**Figure:**
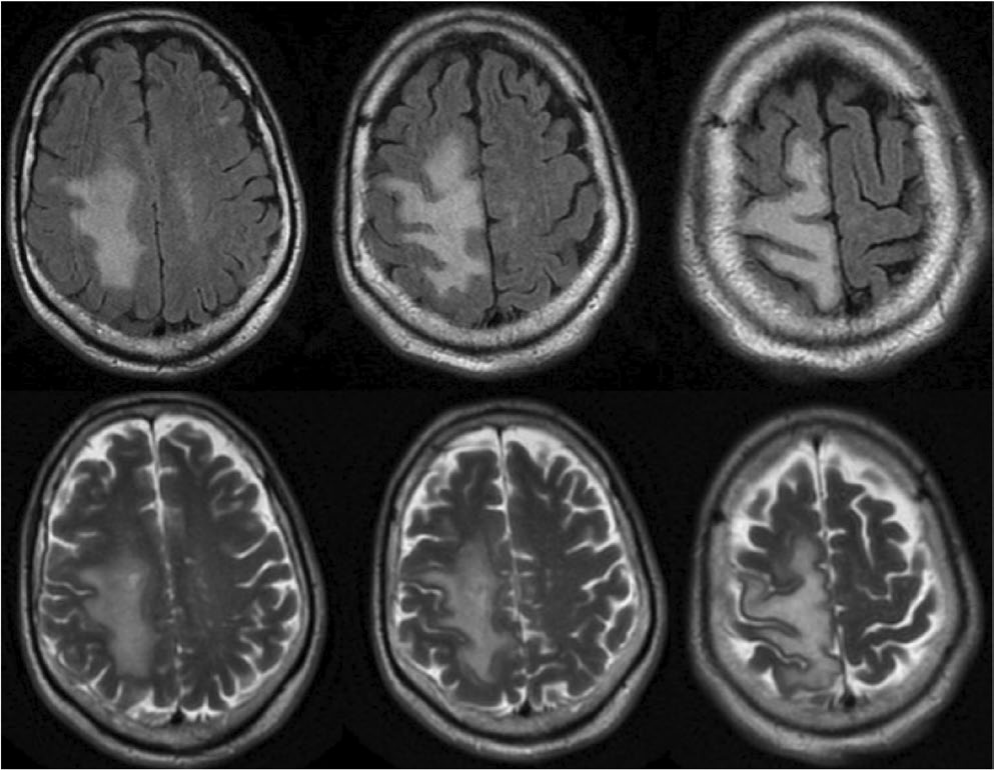
**FIGURE 1** Axial FLAIR and T2‐weighted MRI before maraviroc treatment.

**Figure:**
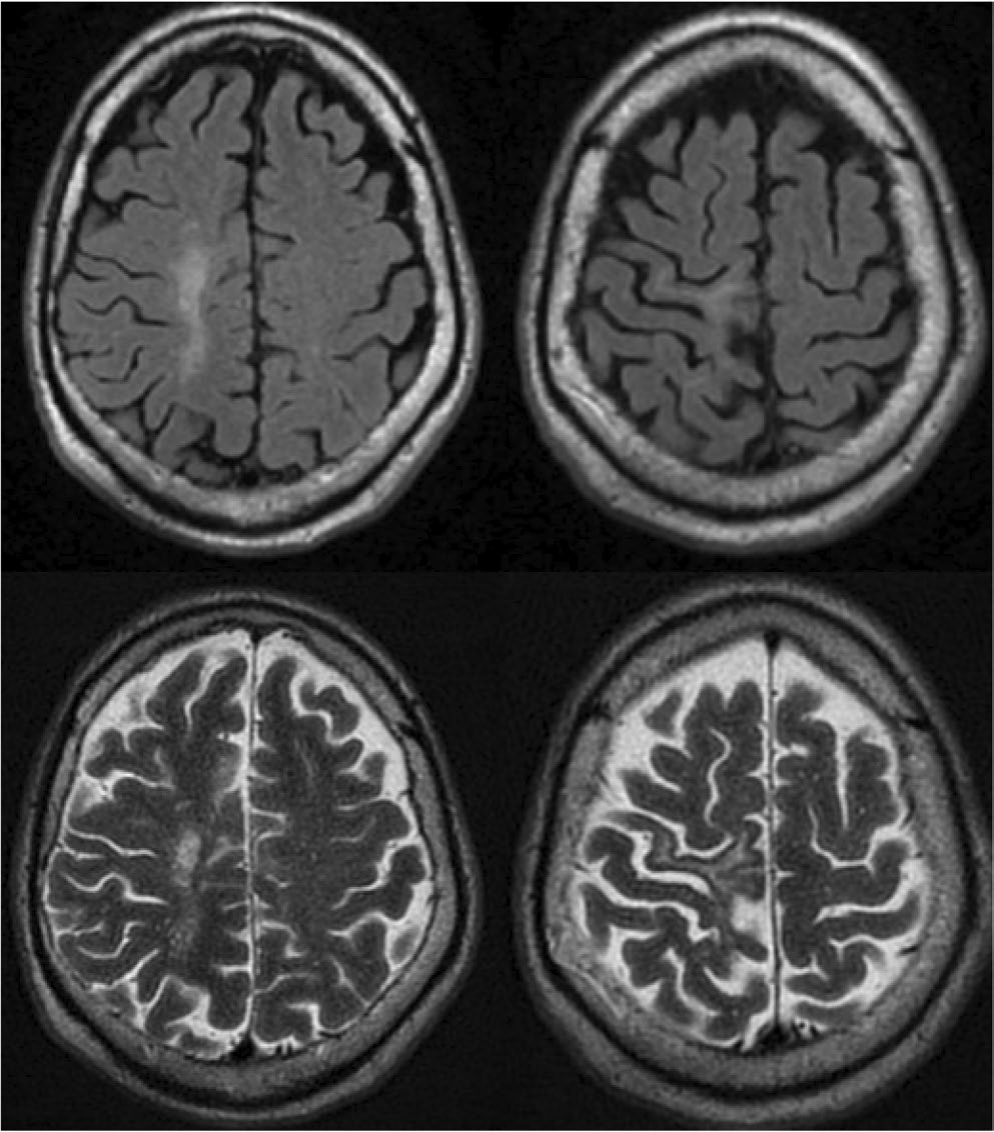
**FIGURE 2** Axial FLAIR and T2‐weighted MRI after maraviroc treatment.

**Conclusion:** In patients with natalizumab‐associated PML, prognosis largely depends on the timing of diagnosis and the size of the lesions. Additionally, a condition known as PML‐associated IRIS may also develop. Immune checkpoint inhibitors, T‐cell therapies and and CCR5 agonists such as Maraviroc are among the treatment options. Distinguishing cases of PML‐associated IRIS from MS relapses and administering appropriate treatments in a timely manner are crucial for reducing mortality and morbidity.

**Disclosure:** Nothing to disclose.

## EPO‐644

### Gender does not impact autonomic dysfunction among people with multiple sclerosis

#### S. Chnitir; A. Souissi; A. Gharbi; Y. Abida; A. Atrous; I. Kacem; A. Gargouri; R. Gouider

##### Department of Neurology, Razi Universitary Hospital, Tunis, Tunisia

**Background and Aims:** During Multiple Sclerosis (MS), autonomic nervous system (ANS) dysfunction is described in 45 to 85% of cases. This study aims to assess the presence of ANS dysfunction among people with MS and to evaluate potential gender differences.

**Methods:** A cross‐sectional study was conducted in the Department of Neurology of Razi Hospital (Tunisia) and included patients diagnosed with MS according to the 2017 McDonald criteria. Patients were divided into two groups according to gender. ANS was evaluated via the Composite Autonomic Symptom Score (COMPASS)‐31. Six autonomic domains were evaluated: orthostatic intolerance, vasomotor, secretomotor, bowel, bladder and pupillomotor. Expanded Disability Status Scale (EDSS) score was used to assess the degree of disability. A *p*‐value of <0.05 was considered statistically significant.

**Results:** A total of 82 patients with MS were enrolled with a female predominance (*n* = 64, 78%). Mean age (33.7 years ± 9.7 vs 37.9 years ± 11.8; *p* = 0.1), disease duration (7.8 years ± 5.7 vs 9.5 years ± 7.5; *p* = 0.2), and EDSS score (2.2 ± 1.9 vs 3 ± 2.1; *p* = 0.1) were comparable between females and males. ANS dysfunction was highly reported among females (*n* = 62, 97%) and males (*n* = 16, 89%) (*p* = 0.2). COMPASS‐31 scores were higher among females but did not reach a statistically significant difference (22.9 ± 14.4 vs 18.1 ± 13.7; *p* = 0.2). Females exhibited higher scores in orthostatic intolerance, secretomotor, bowel, bladder and pupillomotor functions, but none of these differences reached statistical significance (*p* > 0.05).

**Conclusion:** Autonomic dysfunction is highly prevalent among people with MS and appears to be independent from gender.

**Disclosure:** Nothing to disclose.

## Neuroimmunology 3

## EPO‐645

### Diagnostic delay and misdiagnosis of multiple sclerosis: Data from the iConquerMS patient registry

#### A. Solomon ^1^; S. Weinstein^2^; R. Shinohara^3^; S. Aoun^4^; S. Hollie^5^; S. Alessandra^6^

##### ^1^Larner College of Medicine at the University of Vermont, Department of Neurological Sciences; ^2^Department of Epidemiology and Biostatistics, College of Public Health, Temple University, Philadelphia, PA; ^3^Penn Statistics in Imaging and Visualization Center, Department of Biostatistics, Epidemiology, and Informatics, Perelman School of Medicine, University of Pennsylvania, Philadelphia, PA; ^4^University of Western Australia, Perron Institute for Neurological and Translational Science, La Trobe University, Australia; ^5^Accelerated Cure Project, Waltham, MA; ^6^Unit of Neuroepidemiology, Fondazione IRCCS Istituto Neurologico Carlo Besta, Milan, Italy

**Background and Aims:** Recent data from research cohorts suggest that time to diagnosis of MS has diminished relative to revisions to MS diagnostic criteria, yet some studies have not replicated this finding more broadly. Several recent studies suggest that misdiagnosis of initial symptoms of MS are a frequent contributor to diagnostic delay.

**Methods:** A patient survey was available to participants of iConquerMS during from November 12, 2021, through December 22, 2021.

**Results:** There were 428 participants. Diagnostic delay was median of 2.0 months (mean of 22.8 months, range: 0–32.9 years). 173/428 (40.4%) reported misdiagnosis of symptoms that were later diagnosed as due to MS. Diagnostic delay evaluated by epoch proximal to revisions to MS diagnostic criteria decreased over time. Misdiagnosis was associated with a longer delay to MS diagnosis (*p* < 0.001). 208 reported earlier symptoms retrospectively recognized as referable to MS that were not clinically evaluated. Diagnostic delay from these symptoms was a median of 5.4 years and mean 8.9 years (range: 0–47.4 years).

**Conclusion:** Diagnostic delay was prevalent and associated with frequent misdiagnosis of initial symptoms of MS. Half the participants reported earlier symptoms that were not clinically evaluated and were later attributed to MS. Future studies tracing the diagnostic journey of patients with MS are needed to understand and prevent patient‐specific and healthcare system‐related causes of diagnostic delay.

**Disclosure:** AJS: has received research funding from Bristol Meyers Squibb; personal compensation for consulting, advisory boards, or non‐promotional speaking from Bristol Meyers Squibb, EMD Serono, Horizon Therapeutics, Kiniksa Pharmaceuticals, Octave Bioscience, and TG Therapeutics; contract research with Sanofi, Actelion, Genetech/Roche, and Novartis. SMW: reports no disclosures. SMA: reports no disclosures. HS: reports no disclosures. RTS: personal compensation for consulting to Octave Bioscience and the American Medical Association. AS: has received personal fees for advisory board and non‐promotional speaking for Almirall and Merck.

## EPO‐646

### Central vein sign in mitochondrial cytopathy, due to mutation in the NUBPL gene

#### Â. Seke

##### Neurology Service, Santo António Hospital, CHUdSA; ICBAS, University of Porto, Porto, Portugal

**Background and Aims:** Introduction: The central vein sign on MRI has been considered specific to multiple sclerosis and is part of the new diagnostic criteria (2024). The NUBPL mutation consists of the substitution of a T for a C at position 545 resulting in the replacement of a valine for an alanine at position 182. They have previously been associated with mitochondrial complex I deficiency, including ataxia, dysarthria, spasticity, and cognitive deterioration, usually early.

**Methods:** A 52‐year‐old accountant presented cognitive complaints evolving over a year, including disorganization and delays in completing professional and domestic tasks. She has a history of psoriatic arthritis, treated with adalimumab, and was referred to Neurology due to MRI‐detected white matter changes, raising suspicion of demyelinating disease secondary to anti‐TNF therapy. Family history revealed a sister with learning difficulties, behavioral changes, motor deficits, ataxia, cognitive decline, and epilepsy, who died at 43.

**Results:** Findings included cerebral MRI with supratentorial white matter hypersignal on T2/FLAIR, some ovoid and periventricular lesions, and multifocal subcortical frontal‐parietotemporal expression. No cortical, posterior fossa, or corpus callosum involvement was noted. Lesions displayed T1‐weighted hyposignal, and SWI sequences showed central linear hypointensities with a “central vein” sign. Genetic testing confirmed a deleterious NUBPL gene mutation, consistent with her sister's case. Neuropsychological assessment revealed attentional and executive difficulties. No oligoclonal bands.

**Conclusion:** The patient exhibits isolated cognitive impairments, atypical white matter lesions for MS, progressive supratentorial white matter hyperintensity, and a confirmed NUBPL gene mutation. Although the central vein sign strongly suggests MS, it can also occur in other conditions.

**Disclosure:** Nothing to disclose.

## EPO‐647

### Diagnostic and therapeutic challenges in LGI1 autoimmune encephalitis with paraneoplastic features

#### C. Ballester Martinez; I. Hernando Jimenez; M. Herrezuelo Lafuente; M. Gilot Sancho; M. Elguezabal Garcia; C. Treviño Peinado; N. Huertas Gonzalez; V. Hernando Requejo; C. Cemillan Fernandez; N. Juarez Torrejon

##### Department of Neurology, Hospital Universitario Severo Ochoa, Madrid, Spain

**Background and Aims:** Autoimmune encephalitis associated with LGI1 antibodies is a rare and potentially severe condition, often linked to paraneoplastic syndromes. Its nonspecific symptoms, including behavioral disturbances, cognitive decline and seizures, complicate timely diagnosis. Prompt recognition is essential, as its paraneoplastic nature may reflect an underlying malignancy requiring targeted management.

**Methods:** We report the case of a 56‐year‐old woman with a history of anxiety and depression treated with sertraline since 2020. In March 2024, she presented with diarrhea and hyponatremia, initially attributed to SIADH, prompting sertraline discontinuation. Over the following months, her family observed worsening mood, confusion and memory deficits, resulting in multiple emergency visits. By June, she exhibited erratic behavior, anxiety, visual hallucinations, and worsening memory, prompting admission to Neurology. Examination revealed temporal disorientation, impaired short‐term memory, bradypsychia, confabulations, a persistent glabellar reflex, and myoclonic intrusions.

**Results:** Diagnostic workup showed a normal MRI and CSF (including tau/beta‐amyloid ratio and absence of 14‐3‐3 protein). EEG confirmed epileptiform activity, which resolved after dual treatment with brivaracetam and valproic acid. PET‐FDG was compatible with encephalitis, while serum LGI1 antibodies were positive. Immunoglobulins and corticosteroids achieved significant clinical stabilization, with marked improvement in memory (MoCA 30/30) and functional independence at six months. Further CSF analysis revealed oligoclonal bands with an IgG lambda monoclonal component. Flow cytometry identified a B‐cell lymphoproliferative syndrome (CD5+), consistent with marginal zone lymphoma. Hematology favored monitoring and potential Rituximab therapy if needed.

**Conclusion:** This case highlights the diagnostic challenges of LGI1 encephalitis and its paraneoplastic implications, emphasizing the need for individualized, interdisciplinary care.

**Disclosure:** Nothing to disclose.

## EPO‐648

### Clinical spectrum of anti‐Gq1b antibody syndrome

#### C. Kumbhar ^1^; R. Kulkarni^2^; S. Pujari^2^; V. Deshpande^2^

##### ^1^Manipal Hospital, Baner, Pune, MH, India; ^2^Deenanath Mangeshkar Hospital, Pune, India

**Background and Aims:** Antibody based studies revealed Miller Fisher Syndrome and Bickerstaff Brainstem Encephalitis were commonly linked to Anti‐Gq1b antibody, although with certain additional clinical features which were not a part of the core features of Miller Fisher or Bickerstaff Encephalitis. Our study aims to study the spectrum of Anti‐Gq1b related disease.

**Methods:** Twenty one newly diagnosed Anti‐GQ1b antibody positive patients between 2019 and 2024 were included in the study.

**Results:** 63% patients had an antecedent illness; 6/18 (28%) had diarrhea and 7/18 (33%) had URTI. 17/18 patients had ophthalmoplegia (85%), 13/18 (72%) had ataxia, 12/18 (66%) had areflexia while 1/18 (5%) had hyperreflexia. 13/18 (63%) had ocular motor nerve involvement, 10/18 (55%) had lower cranial nerves involvement and 6/18 (33%) had bifacial involvement. 7/18 (39%) patients had motor weakness. CSF routine microscopy was abnormal in 5/18 (28%) patients. NCV studies were abnormal in 6/18 (33%).

**Conclusion:** Antecedent illness is not uncommon in Anti‐Gq1b (60‐70%); URTI is most common followed by gastroenteritis. Partial Gq1b syndromes and Overlap Gq1b antibody syndrome commoner than Classic MFS/Bickerstaff Encephalitis. Ophthalmoplegia is the most common presenting and sign, followed by ataxia and areflexia. Limb weakness is seen in upto 40% as a part of Anti‐Gq1b Antibody Related Disease/Overlap syndrome. Bulbar palsy can occur in 40‐70% of patients, although not a part of the classic triad. CSF albumin‐cytological dissociation can be present in 27‐40% patients. CSF leucocytosis is rare. NCV was abnormal in 28% cases but can be abnormal in upto 80% patients. Intravenous Immunoglobulin is the treatment of choice and 80‐95% patients show good recovery.

**Disclosure:** Nothing to disclose.

## EPO‐649

### Resolution of myelin oligodendrocyte glycoprotein‐IgG‐associated disorders after treatment with efgartigimod

#### D. Liu ^1^; J. Huang^1^; Y. Yuan^1^; Y. Liu^2^; S. Hu^1^; Y. Huang^2^

##### ^1^Department of Neurology, The Third Xiangya Hospital, Central South University, Changsha 410013, China; ^2^Department of Radiology, The Third Xiangya Hospital, Central South University, Changsha, China

**Background and Aims:** Myelin oligodendrocyte glycoprotein antibody‐associated disease (MOGAD) is a distinct central nervous system inflammatory disease. Documented studies or reports on the use of efgartigimod for the treatment of MOGAD are currently lacking.

**Methods:** The patient was admitted to the hospital for fever and headache, altered consciousness, and unsteady walking with weakness in the limbs. Magnetic resonance imaging revealed abnormal signals within the brain tissue and intracranial enhancement of several leptomeninges. Cerebrospinal fluid examination revealed a slight increase in the protein concentration (0.681 g/L). The anti‐MOG antibody titer in the CSF was 1:100. It was serologically negative with IgG‐oligoclonal bands categorized as type II, suggesting intrathecal IgG synthesis in the central nervous system. Treatment with intravenous methylprednisolone 1000 mg once a day failed to completely control the symptoms of the patient. Thus, 10 mg/kg of efgartigimod was administered intravenously once a week for 3 weeks. The serum anti‐MOG antibody and IgG concentrations decreased, and the clinical symptoms improved.

**Results:** A case of MOGAD accompanied by encephalitis, with the anti‐MOG antibody titer in the CSF was 1:100, where the patient was administered efgartigimod and subsequently exhibited favorable therapeutic outcomes. To our knowledge, this treatment for MOGAD is the first attempt currently.

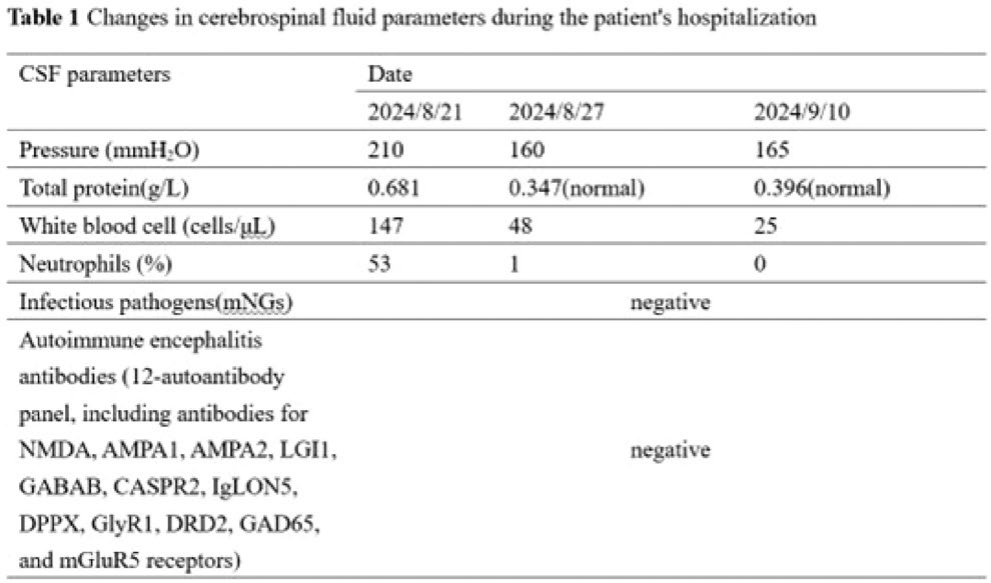

**Figure:**
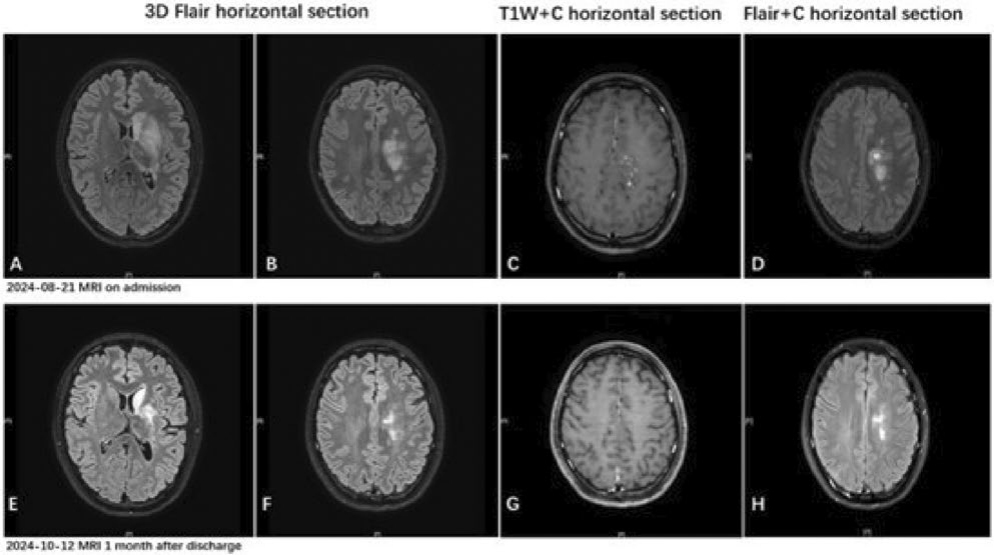
**FIGURE 1** The MRI at admission, the review MRI during hospital stay, and the review MRI at one month post‐discharge.

**Figure:**
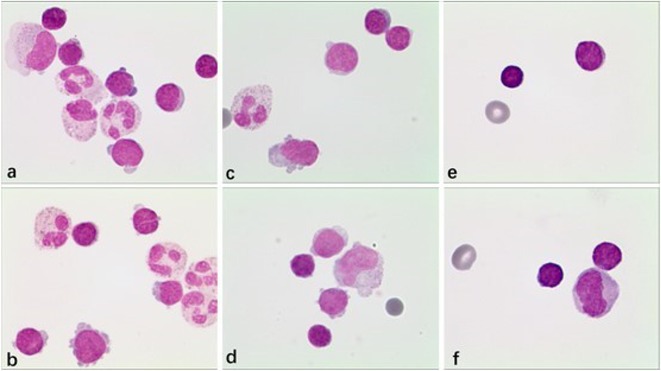
**FIGURE 2** The CSF at admission, the review during hospital stay, and on discharge.

**Conclusion:** The combination of efgartigimod with methylprednisolone yielded positive outcomes and is a promising therapeutic strategy for MOGAD.

**Disclosure:** Nothing to disclose.

## EPO‐650

### Safety and efficacy of cladribine in patients discontinuing fingolimod due to elevated liver enzymes: FinClad study

#### M. Sonmez^1^; M. Yetkin ^2^; D. Arslan Mehdiyev^1^; M. Köseoğlu^3^; S. Mungan^4^; N. Kale^5^; M. Terzi^6^; A. Tunç^7^; E. Koc^8^; S. Sen^6^; S. Ozben^9^; T. Yoldaş^10^; V. Cilingir^11^; D. Kotan^7^; D. Aksoy^12^; F. Kurtuluş Aydın^1^; B. Kocer^13^; M. Demir^3^; M. Cam^14^; P. Öztürk^15^; Y. Ekmekyapar Fırat^16^; S. Ömerhoca^5^; M. Ercan^13^; J. Skuljec^17^; R. Pul^18^

##### ^1^Department of Neurology, Ankara Etlik City Hospital, Ankara, Turkey; ^2^Department of Neurology, Erciyes University, Kayseri, Turkey; ^3^Department of Neurology, University of Health Sciences, Istanbul Kanuni Sultan Suleyman Training and Research Hospital, Istanbul, Turkey; ^4^Department of Neurology, University of Health Sciences Ankara City Hospital, Ankara, Turkey; ^5^Department of Neurology, University of Health Sciences Bağcılar Training and Research Hospital, Istanbul, Turkey; ^6^Department of Neurology, Ondokuz Mayis University, Faculty of Medicine, Samsun, Turkey; ^7^Department of Neurology, Sakarya University, Faculty of Medicine, Sakarya, Turkey; ^8^Department of Neurology, Uludag University, Faculty of Medicine, Bursa, Turkey; ^9^Department of Neurology, University of Health Sciences, Antalya Training and Research Hospital, Antalya, Turkey; ^10^Department of Neurology, Ankara Yıldırım Beyazıt University, Faculty of Medicine, Ankara, Turkey; ^11^Department of Neurology, Van Yüzüncü Yıl University, Faculty of Medicine, Van, Turkey; ^12^Department of Neurology, Tokat Gaziosmanpasa University, Faculty of Medicine, Tokat, Turkey; ^13^Department of Neurology, Gazi University Faculty of Medicine, Ankara, Turkey; ^14^Department of Neurology, Canakkale University, Faculty of Medicine, Canakkale, Turkey; ^15^Department of Neurology, Ankara Yıldırım Beyazıt University Yenimahalle Training and Research Hospital, Ankara, Turkey; ^16^Department of Neurology, SANKO University School of Medicine, Gaziantep, Turkey; ^17^Department of Neurology/NeuroScience Lab, University Hospital Essen, Essen, Germany; ^18^Department of Neurology, University Medicine Essen, Essen, Germany; Center for Translational Neuro‐ and Behavioral Sciences, University Hospital Essen, Essen, Germany

**Background and Aims:** Fingolimod is an effective disease‐modifying therapy (DMT) for relapsing‐remitting multiple sclerosis (RRMS), but elevated liver enzyme levels often necessitate treatment discontinuation. Cladribine, an oral DMT with comparable efficacy, has a more favorable hepatic safety profile. However, data on the safety and short‐term efficacy of switching from fingolimod to cladribine in patients with liver enzyme elevation remain limited.

**Methods:** This retrospective, multicenter study included 45 RRMS patients (aged 18–65) who transitioned from fingolimod to cladribine due to elevated liver enzymes (AST and ALT > 3× ULN). Clinical and demographic data, liver function tests (LFTs), and disease activity parameters were collected at six time points: before fingolimod initiation, at fingolimod discontinuation, immediately before cladribine initiation, and at 1, 2, and 3 months post‐treatment. Disease activity was assessed based on relapses and MRI findings (new/enlarging T2 or gadolinium‐enhancing lesions). Statistical analyses included repeated‐measures ANOVA and logistic regression.

**Results:** AST and ALT levels significantly declined after cladribine initiation (p < 0.001). No serious liver‐related adverse events were reported. Disease activity remained controlled in 86.7% of patients; five had relapses, and one showed radiological activity. Longer washout duration (>9 weeks) was associated with increased disease activity (p = 0.011). Younger age was a potential risk factor. EDSS scores remained stable.

**Conclusion:** Cladribine is a safe and effective option for RRMS patients discontinuing fingolimod due to elevated liver enzymes. Short washout periods and close monitoring are essential to minimize disease reactivation.

**Disclosure:** Nothing to disclose.

## EPO‐651

### Physician‐ and patient‐reported outcomes by disease severity in a United States real‐world Myasthenia Gravis population

#### L. Miller‐Wilson^1^; J. Conyers^2^; S. Birija^2^; H. Connolly^2^; G. Gibson^2^; L. Lal ^1^; Y. Edwards^1^

##### ^1^Immunovant, Inc., New York, USA; ^2^Adelphi Real World, Bollington, UK

**Background and Aims:** Myasthenia gravis (MG) is a rare neuromuscular condition causing muscle weakness and fatigue. This study describes physician‐ and patient‐reported outcomes in patients with MG stratified by MG Foundation of America (MGFA) severity classification.

**Methods:** Physicians provided patient‐level data via the Adelphi MG II Disease Specific Programme™ (DSP) from February–August 2024; a different cohort of patients (PAT) self‐reported data in October 2024. Information on patient demographics, MGFA classification, clinical outcomes, and MG‐Activities of Daily Living (MG‐ADL) was collected.

**Results:** Fifty‐two physicians reported on 390 DSP patients; 243 PAT patients self‐reported. DSP patients had a mean (SD) age of 55.1 (13.7) years, a mean (SD) time since diagnosis of 3.8 (5.6) years, and 46.9% were female. PAT patients had a mean (SD) age of 49.1 (14.6) years, self‐reported experiencing symptoms for a mean (SD) of 13.0 (2.2) years, and 87.7% were female. The proportions of MGFA class I, class II, and class III/IV/V patients in the DSP population were 20.5%, 63.3% and 16.2%, respectively; corresponding proportions in the PAT cohort were 6.6%, 30.0%, and 63.4%, respectively. Clinical and MG‐ADL outcomes by MGFA classification are in Table 1. MG‐related hospitalizations in the year prior to the survey occurred in 7.1%, 16.3% and 18.3% of MGFA class I, II, and III/IV/V DSP patients, respectively; corresponding proportions in the PAT cohort were12.5%, 23.3% and 48.7%.

**Figure:**
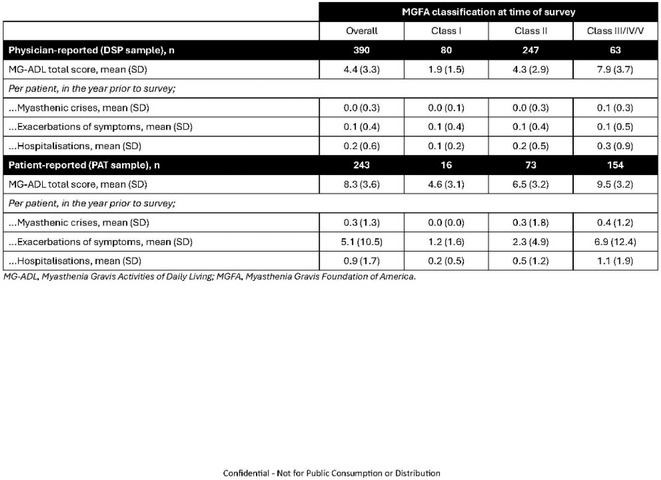
**TABLE 1** MG‐ADL total scores derived from physician‐ and patient‐reporting by MGFA classification at time of survey.

**Conclusion:** Increased activity impairment and worse clinical outcomes were observed with higher MGFA classification in both cohorts. Treatments delivering greater improvements for patients with moderate‐to‐severe MG are needed.

**Disclosure:** LAMW, LL and YE are employees of Immunovant, Inc., JC, SLB, HC and GG are employees of Adelphi Real World.

## EPO‐652

### Two cases of progressive multifocal leukoencephalopathy linked to siponimod in multiple sclerosis

#### M. Grávalos ^1^; R. Carvajal^1^; A. Zabalza^1^; B. Rodríguez‐Acevedo^1^; M. Tintoré^1^; C. Auger^2^; A. Rovira^2^; X. Montalban^1^

##### ^1^Department of Neurology‐Neuroimmunology, Vall Hebron University Hospital and Multiple Sclerosis Centre of Catalonia (Cemcat) & Universitat Autònoma de Barcelona, Pg. Vall d’Hebron, 119‐129, 08035, Barcelona, Spain; ^2^Section of Neuroradiology, Vall Hebron University Hospital & Universitat Autònoma de Barcelona, Pg. Vall d’Hebron, 119‐129, 08035, Barcelona, Spain

**Background and Aims:** Sphingosine‐1‐phosphate (SP1) modulators, used in multiple sclerosis (MS), have been linked to progressive multifocal leukoencephalopathy (PML), predominantly with fingolimod and only one reported case with Siponimod.

**Methods:** We report one confirmed and another probable case of PML associated with Siponimod in MS patients treated at a tertiary hospital in 2024.

**Results:** Case 1: A 65‐year‐old male with secondary progressive MS (SPMS), previously treated with interferon beta‐1a (INF‐B), natalizumab (discontinued in 2012) and fingolimod, initiated Siponimod in March 2023. In July 2024, he developed severe left hemiparesis. Magnetic resonance imaging (MRI) revealed a fronto‐parietal juxta‐subcortical white matter lesion, highly suggestive of PML, which was later confirmed by biopsy. Treatment with pembrolizumab stabilized his clinical condition, with partial neurological recovery and lesion reduction on follow‐up MRI. Case2: A 48‐year‐old male with SPMS, previously treated with INF‐B and dimethylfumarate, began Siponimod in 2023. One year after he reported headaches and visual disturbances initially diagnosed as migraine. In July 2024, MRI revealed a new occipital juxtacortical lesion, highly suggestive of PML. JC virus in cerebrospinal fluid was negative twice and biopsy was deferred due to clinical stability. Siponimod was discontinued, but the patient developed myelitis shortly after. Teriflunomide was initiated, with stability of the occipital lesion but persistence of progressive MS‐related disability.

**Figure:**
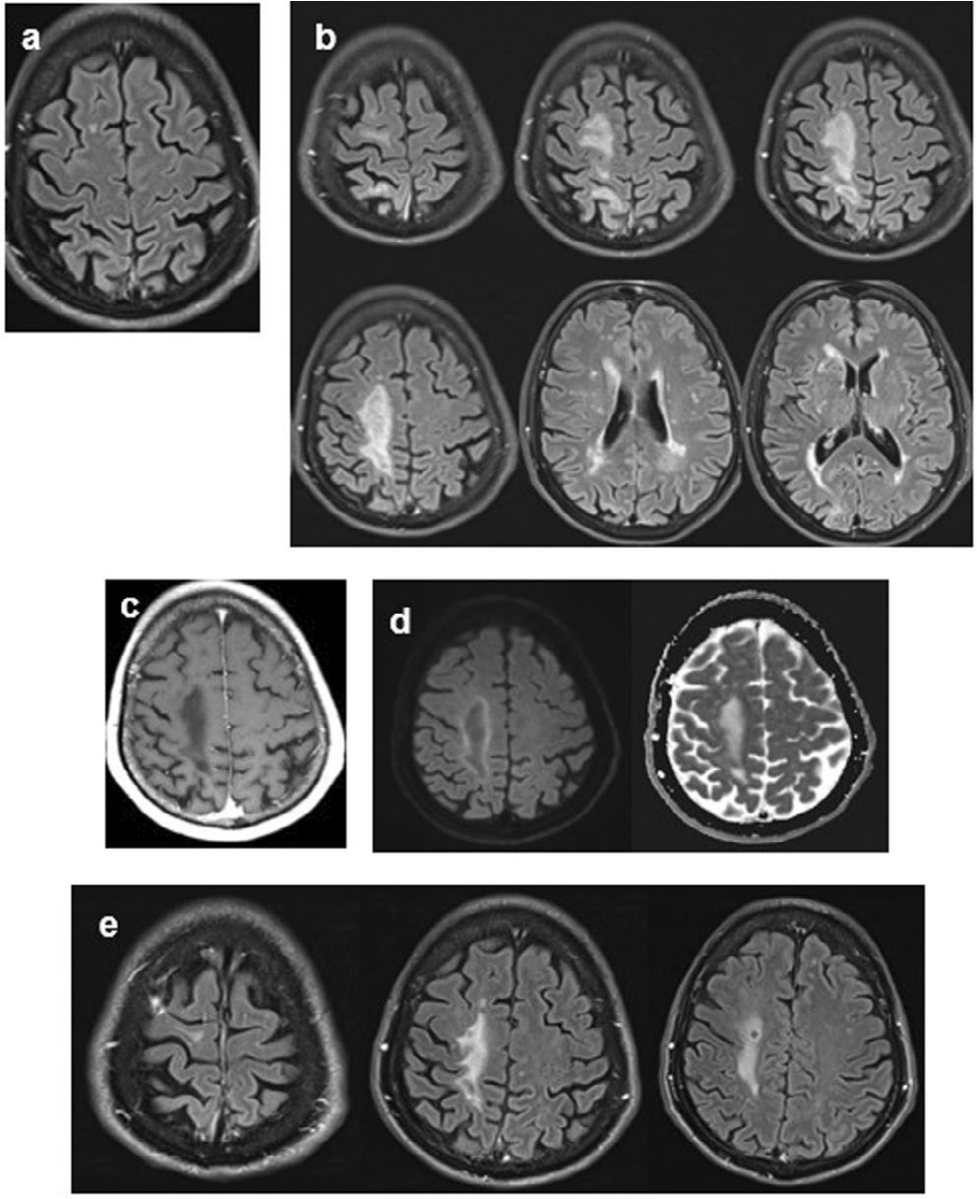
**FIGURE 1** Case 1. Baseline brain MRI (a). Right fronto‐parietal juxta‐subcortical white matter lesion hyperintense on FLAIR (b), with no contrast enhancement (c) and mild peripheral diffusion restriction on DWI (d). Reduction in size in follow‐up MRI (d).

**Figure:**
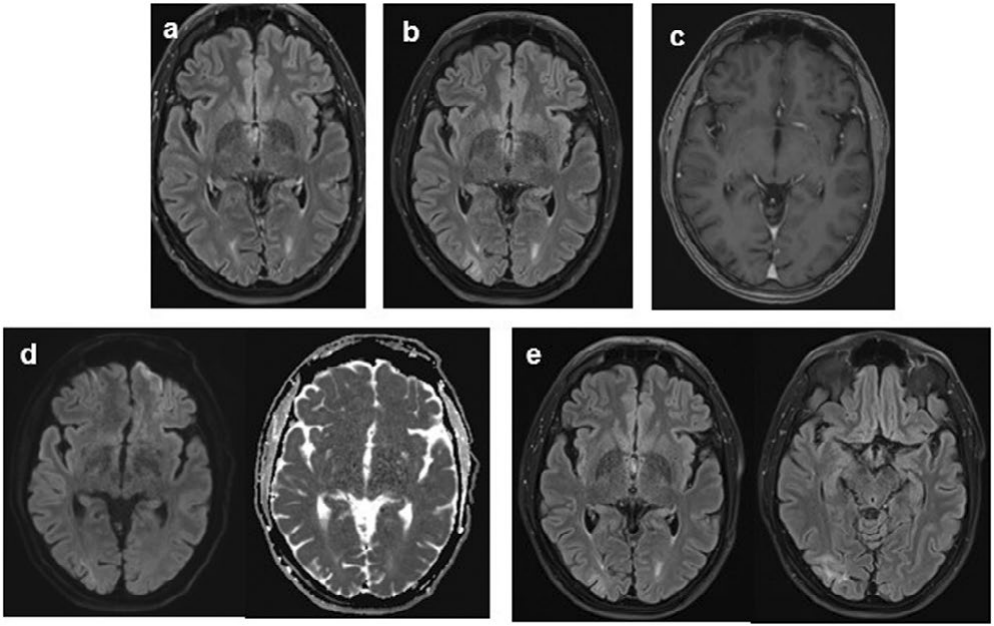
**FIGURE 2** Case 2. Baseline brain MRI (a). Hyperintense right occipital juxtacortical lesion (b), with no contrast enhancement (c) and mild peripheral diffusion restriction on DWI (d). Stability on follow‐up MRI (e).

**Conclusion:** Newer S1P modulators, such as Siponimod, now have long follow‐up data in real‐world settings, with safety remaining a critical concern. Our cases highlight the importance of MRI in detecting rare but severe complications, such as PML. Continued vigilance is essential to mitigate risks.

**Disclosure:** Nothing to disclose.

## EPO‐653

### Incidence of COVID‐19 in the phase 3 Myasthenia Gravis inebilizumab trial randomized controlled period

#### R. Nowak^1^; K. Utsugisawa^2^; M. Benatar^3^; E. Ciafaloni^4^; M. Leite ^5^; J. Vissing^6^; Q. Li^7^; F. Tang^7^; C. Najem^7^; S. Cheng^7^; J. Howard Jr.^8^

##### ^1^Yale University, New Haven, USA; ^2^Hanamaki General Hospital, Hanamaki, Japan; ^3^University of Miami Miller School of Medicine, Miami, USA; ^4^University of Rochester, Rochester, USA; ^5^University of Oxford, Oxford, UK; ^6^University of Copenhagen, Copenhagen, Denmark; ^7^Amgen, Thousand Oaks, USA; ^8^University of North Carolina, Chapel Hill, USA

**Background and Aims:** B‐cell‐depleting therapies have the potential to lessen humoral responses. It is unclear whether this leads to a higher risk of Coronavirus Disease 2019 (COVID‐19) incidence. This post‐hoc analysis of the Myasthenia Gravis Inebilizumab Trial (MINT) examined whether inebilizumab treatment is associated with an increased risk of COVID‐19 infection in participants with generalized Myasthenia Gravis (gMG).

**Methods:** MINT (NCT04524273) was a phase 3 study which evaluated the efficacy and safety of inebilizumab in adult patients with gMG. First participant was enrolled August 30, 2020, after COVID‐19 was initially reported (December 2019) and before the first vaccine received approval (December 2020). Participants were given 300mg of intravenous inebilizumab or placebo on RCP Day‐1, Day‐15, and Day‐183 (AChR+ only). Descriptive statistics/analysis completed.

**Results:** A total of 238 participants were randomized (placebo 119, inebilizumab 119). Considering the timeline of trial enrollment and vaccine availability, 4 (3.4%) placebo‐treated participants and 9 (7.6%) inebilizumab‐treated participants received a COVID‐19 vaccine on/after the 1st dose. COVID‐19 infection was reported in 19 (16.0%) placebo‐treated and in 21 (17.7%) inebilizumab‐treated participants during the RCP. Most infections were in participants <65 years of age (100% placebo, 90.5% inebilizumab). Most of the reported infections were Grade 1 [placebo: 8/19 (42.1%); inebilizumab: 10/21 (47.6%)] or Grade 2 [placebo: 8/19 (42.1%); inebilizumab: 8/21 (38.1%)]. COVID‐19 related hospitalizations occurred in 4 placebo‐treated participants and 4 inebilizumab‐treated participants with a mean ± SD hospitalization length of 10.8 ± 8.8 days and 22.8 ± 25.9 days, respectively.

**Figure:**
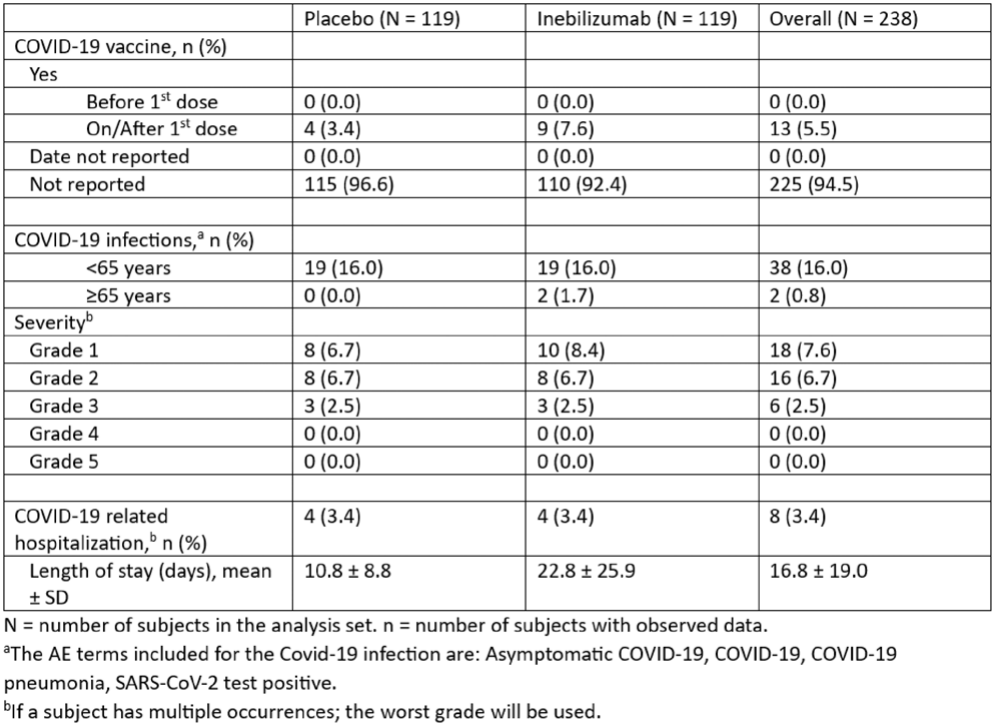
**TABLE 1** COVID‐19 in MINT clinical trial (randomized control period).

**Conclusion:** Inebilizumab treatment did not increase the incidence of COVID‐19 in participants with gMG.

**Disclosure:** RJN research support from NIH, Genentech, Alexion, argenx, Annexon Biosciences, UCB S.A., the MGFA Inc., Janssen, Immunovant, Grifols & Amgen Inc. Consultant/advisor to Alexion, argenx, Cabaletta Bio., Cour, UCB S.A., Immunovant, Janssen & Amgen Inc. KU consultant to UCB, argenx, Janssen, Amgen Inc., Chugai, Hanall BioPharma, Merck & Mitsubishi Tanabe. Honoraria from Argenx, Alexion, UCB & the Japan Blood Products Organization. MB research support from Immunovant & Alexion. Consultant to Alexion, Cartesian, Amgen Inc., Immunovant, Sanofi, Takeda, & UCB. EC advisor/consultant to Alexion, argenx, Biogen, Amicus, Pfizer, Italfarmaco, Sarepta, Janssen, NS Pharma & Roche. MIL funded by NHS & University of Oxford. Grants from Myaware, Muscular Dystrophy UK & the University of Oxford. Honoraria/travel from Biogen, Novartis, UCB & the Guthy‐Jackson Charitable Foundation. Advisory boards for UCB, argenx & Amgen Inc. JV advisory boards for Regeneron, UCB, argenx, Alexion, Amgen Inc., Dianthus Therapeutics, Janssen & Roche. FT, CN, SC are employees/stockholders of Amgen Inc. JFH funding from Ad Scientiam, Alexion, argenx, Cartesian, CDC, Prevention, MGFA, MDA, NIH, PCORI & UCB; honoraria/consulting from Academic CME, Alexion AstraZeneca RD, argenx, Biohaven, Biologix, CheckRare CME, Curie.bio, F. Hoffmann‐LaRoche, Amgen Inc., Medscape CME, Merck EMB Serono, NMD Pharma, Novartis, PeerView CME, Physicians' Education Resource CME, PlatformQ CME, Regeneron, Sanofi, UCB, & Zai Labs.

## EPO‐654

### Movement disorders and limbic encephalitis: An uncommon presentation of autoimmune GFAP astrocytophaty

#### M. Lozano López; G. Lafuente Gómez; J. Sosa Luis; L. Portela Martínez; R. Boto Martínez; J. García Domínguez; A. Contreras Chicote

##### Neurology, Hospital General Universitario Gregorio Marañon, Madrid, Spain

**Background and Aims:** Antibodies targeting glial fibrillary acidic protein (GFAP), an intermediate filament protein in adult astrocytes, have recently been identified as a cause of autoimmune meningoencephalomyelitis. This condition demonstrates significant clinical heterogeneity, with impaired consciousness, confusion and headache being the most commonly described symptoms. We describe a distinctive presentation of autoimmune GFAP astrocytopathy.

**Methods:** A 63‐year‐old male with no significant medical history presented with a progressive 3‐month history of tremor, myoclonus, gait disturbances, and cognitive decline.

**Results:** On examination, he exhibited somnolence, disorientation, limited speech, generalized bradykinesia, myoclonus, positional tremor in both arms, left‐sided rigidity, left‐sided pyramidal signs, bilateral clonus, and frontal release signs. Brain MRI revealed T2‐FLAIR cortical and juxtacortical hyperintensities in the temporal lobes and bilateral temporomesial regions. CSF analysis demonstrated mononuclear‐predominant pleocytosis (30 cells, 90% mononuclear) and elevated protein levels (102 mg/dL) with negative microbiological and cytological studies. These findings were consistent with autoimmune limbic encephalitis. Given the suspicion, treatment with high‐dose methylprednisolone (1 g/day, 5 days) and IV immunoglobulins (2 g/kg, 5 days) were followed by corticosteroid tapering, achieving improvement. Rituximab (1 g, twice, 2 weeks apart) was added to sustain the response. Autoimmune GFAP astrocytopathy was confirmed by anti‐GFAP antibodies detected via cell‐based assay at a reference laboratory. Systemic evaluation for neoplasia is negative, and the patient remains clinically stable 2 months post‐treatment.

**Figure:**
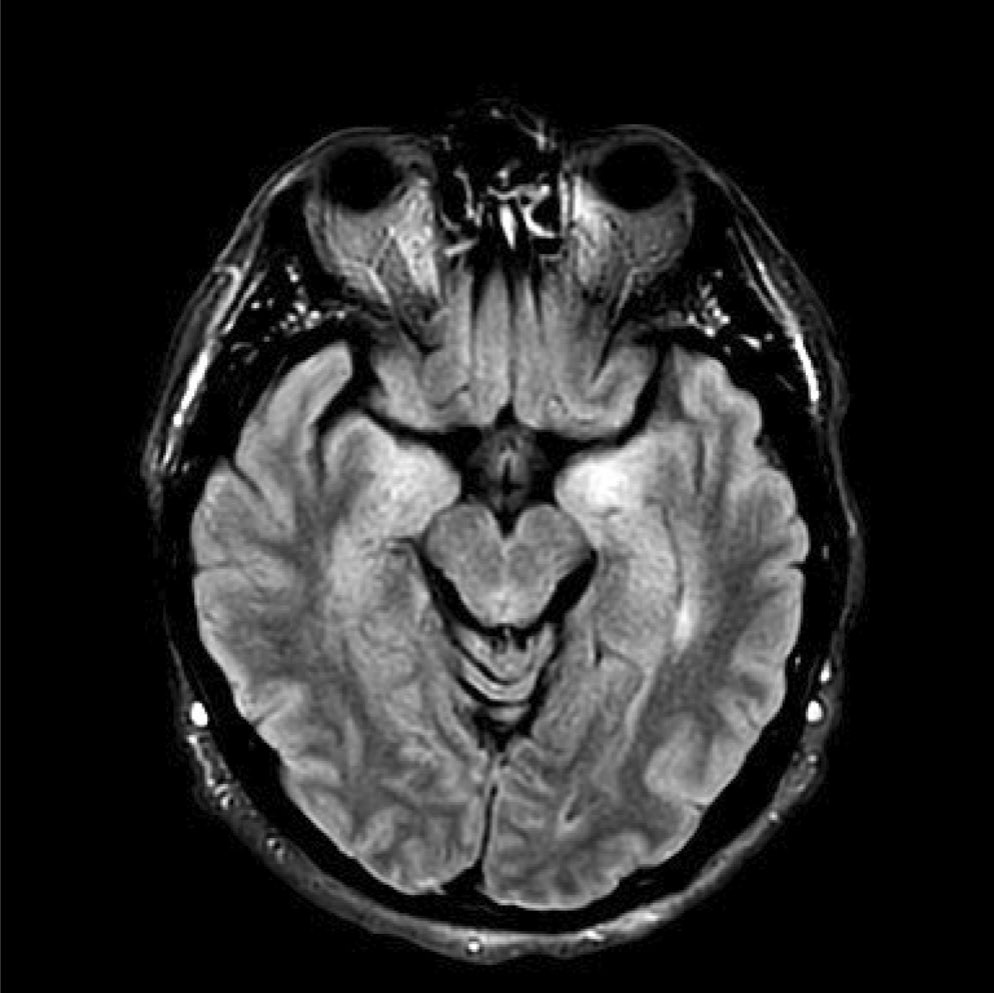
**FIGURE 1** Brain RM T2‐FLAIR revealing hyperintensities in bilateral temporomesial regions.

**Figure:**
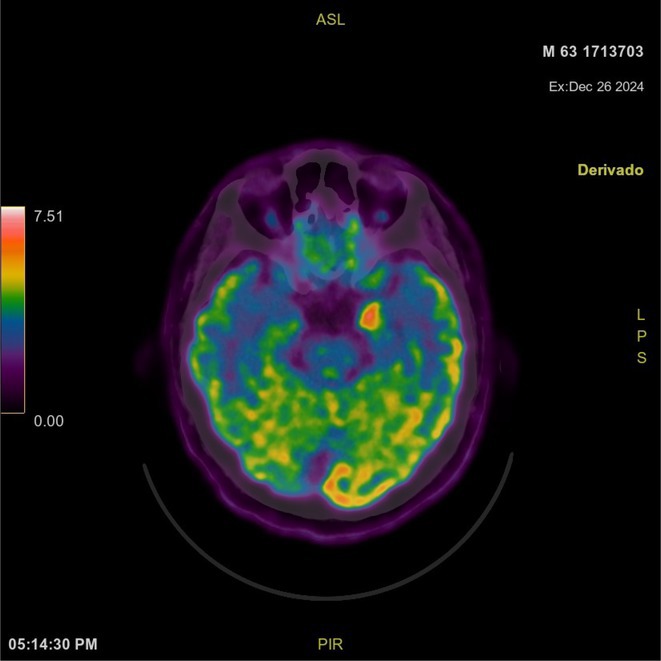
**FIGURE 2** Brain PET/CT scan revealing enhancement in the medial area of the left temporal lobe.

**Conclusion:** This case highlights the need to consider autoimmune GFAP astrocytopathy in patients with movement disorders or limbic encephalitis. Increased awareness of its clinical and radiological features may facilitate earlier diagnosis and treatment.

**Disclosure:** Nothing to disclose.

## EPO‐656

### Function and inflammation of the blood‐brain barrier after a primary exposure to SARS‐CoV‐2

#### R. Santos Silva ^1^; L. Bernardo‐Menezes^2^; E. Azevedo^2^; A. Černohorská^3^; M. Cavalcante^2^; F. de Albuquerque^2^; M. Ferreira^4^; J. de Magalhães^2^; F. Travassos^5^; C. de Morais^2^; C. Bresani‐Salvi^2^

##### ^1^Catholic University of Pernambuco, Recife, Brazil; ^2^Aggeu Magalhães Institute, Fiocruz, Recife, Brazil; ^3^Second Faculty of Medicine, Charles University, Prague, Czechia; ^4^Neurology Department, Hospital da Restauração, Recife, Brazil; ^5^Fernando Travassos Laboratory, Recife, Brazil

**Background and Aims:** SARS‐CoV‐2 can damage the blood‐brain barrier (BBB) and lead to several central nervous system (CNS) disorders, however, its involvement remains poorly understood.

**Methods:** We analysed serum and cerebrospinal fluid (CSF) samples from 39 adults admitted to a neurological emergency in Northeastern Brazil with acute neurological complaints. Patients had no clinical signs of COVID‐19 and were classified into three groups: CNS syndrome and SARS‐CoV‐2 exposure (*n* = 16), exposed without a CNS syndrome (*n* = 17), and unexposed to SARS‐CoV‐2 (*n* = 7). Inflammatory cytokines (interleukins‐IL 6/8) and chemokines (CCL 2, CXCL 5/8/9/10) were measured using flow cytometry, as well as protein profile via gel electrophoresis. BBB function was assessed using CSF:serum coefficients of albumin (QAlb), gamaglobulin (QGgb), while intrathecal inflammation was evaluated through cytokine CSF:serum coefficients.

**Results:** CSF:serum coefficients of IL‐6, IL‐8, and CXCL8 were higher in both exposed groups when compared with the unexposed group. Higher coefficients of CXCL5 and CXCL10 were observed in CNS syndromes patients exposed to SARS‐CoV‐2, compared to the other two groups. No significant differences were observed in QAlb, QGgb, and QGgb:QAlb indices.

**Conclusion:** These findings suggest no evidence of BBB breakdown or intrathecal immunoglobulin production following subclinical SARS‐CoV‐2 exposure. However, a systemic profile of inflammation plus intrathecal production of chemokines involved in the modulation of immune cells were evident in CNS manifestations after primary exposure to SARS‐CoV‐2, even in the absence of COVID‐19 symptoms.

**Disclosure:** Nothing to disclose.

## EPO‐657

### Dysautonomia preceding the diagnosis of LGI1 encephalitis requiring cardioneuroablation

#### Y. Kwon ^1^; D. Kiss^2^; P. Jansky^3^; M. Elisak^3^; H. Mojzisova^3^

##### ^1^Second Faculty of Medicine, Charles University and Motol University Hospital, Prague, Czechia; ^2^Department of Cardiology, University Hospital Brno, Brno, Czechia; ^3^Department of Neurology, Second Faculty of Medicine, Charles University and Motol University Hospital, Prague, Czechia

**Background and Aims:** Encephalitis associated with antibodies against Leucine‐rich glioma inactivated 1 protein (LGI1) commonly manifests with cognitive impairment, psychiatric symptoms, and epileptic seizures, including the pathognomonic faciobrachial dystonic seizures (FBDS). However, rarely, patients may initially present with dysautonomic symptoms. We report a patient who presented initially with bradyarrhythmia requiring close cardiac monitoring with subsequent cardioneuroablation prior to the diagnosis of LGI1 encephalitis.

**Methods:** Case Report.

**Results:** A 39‐year‐old previously healthy man presented with an 8‐day history of recurrent loss of consciousness. He was referred to emergency cardiology service and was diagnosed with recurrent asystole due to vagotonia. A cardioneuroablation was performed and was successful in treating the patient's syncopes. Two weeks after the procedure, the patient developed short‐term memory impairment and episodes of confusion with migrating paraesthesias with impaired consciousness, implying the possibility of focal unaware seizures. Electroencephalogram (EEG) demonstrated focal slowing in the fronto‐temporal region bilaterally with episodes of lateralised periodic discharges. Magnetic resonance imaging (MRI) findings included enlargement of the left hippocampus and parahippocampal gyrus, consistent with signs of unilateral limbic encephalitis. Cerebrospinal fluid (CSF) analysis showed lymphocytic predominant pleocytosis, no infectious agents, and ultimately the serum was positive for antibodies against LGI1. The patient initially did not respond well to methylprednisolone. Then, five cycles of immunoadsorption therapy were administered, resulting in clinical improvement.

**Conclusion:** We present to our knowledge the first case of a patient with LGI1 encephalitis whose severe dysautonomia (prolonged asystole with loss of consciousness) required cardioneuroablation, which successfully treated his syncopes.

**Disclosure:** KYS (medical student) has nothing in relation to this manuscript to disclose. KD has nothing in relation to this manuscript to disclose. JP has nothing in relation to this manuscript to disclose. EM and MH disclose participation in Roche trial recruiting patients with LGI1 encephalitis.

## Neuroepidemiology and ethics in neurology

## EPO‐658

### Prevalence estimation of tremor syndromes in Hungary based on the National Health Insurance Fund database

#### A. Papp ^1^; Á. Berki^1^; P. Vinnai^2^; A. Ajtay^1^; D. Bereczki^1^; L. Erőss^3^; G. Tamás^1^

##### ^1^Department of Neurology, Semmelweis University, Budapest, Hungary; ^2^HUN‐REN Neuroepidemiology Research Group, Semmelweis University, Budapest, Hungary; ^3^Department of Neurosurgery and Neurointervention, Semmelweis University, Budapest, Hungary

**Background and Aims:** Essential tremor is one of the most common movement disorders, and its differential diagnosis is often challenging. The published prevalence of essential tremor is variable worldwide and lacking in Central Europe. We aimed to estimate its prevalence in Hungary and to explore the use of the available therapeutic approaches recommended by the international guidelines.

**Methods:** We collected data from the National Health Insurance Fund database and the pharmacy database of medication refills from all pharmacies throughout the country registered between 2010 and 2020. By matching the specified codes of the International Classification of Diseases and the individually tailored combination of interventions, we attempted to exclude Parkinson's disease and other tremor‐evoking conditions.

**Results:** We estimated the prevalence of essential tremor age‐standardized to the European Standard Population to be 378–388/100,000. After excluding patients with possible Parkinsonian syndromes, we found that 36.4% of the patients with tremor did not take any medication during the study period. Most of the rest used alprazolam, followed by propranolol for the longest period; the alprazolam‐propranolol combination was the most preferred. Deep brain stimulation and ablative surgery were chosen for less than 0.5% of the patients.

**Conclusion:** Our strict methods underestimate the prevalence of essential tremor in Hungary; however, the results do not differ considerably from the international results. Given the limitations of the medication therapy, expanding and improving neurosurgical interventions may help to provide a better quality of life to patients with essential tremor.

**Disclosure:** Nothing to disclose.

## EPO‐659

### Ethical challenges in neurology: Navigating clinical and technological dilemmas

#### A. Chagiashvili

##### MD, Resident Neurologist, Lecturer at Tbilisi State university (TSU), Research Department, Tbilisi, Georgia

**Background and Aims:** Neurology presents unique ethical challenges due to the complex nature of neurological disorders and advancements in neuro technologies. Key concerns include informed consent, patient autonomy, and the ethical use of emerging treatments. This study explores ethical dilemmas in clinical practice and research within neurology, focusing on neurodegenerative diseases, neuro ethics, and novel neuro technologies.

**Methods:** A systematic review was conducted of literature from journals such as Neurology, Brain, The Lancet Neurology, Neuroethics, Journal of Medical Ethics, Frontiers in Neurology, American Journal of Bioethics, Journal of Alzheimer’s Disease, and Journal of Clinical Neuroscience. Articles were selected based on their relevance to ethical issues, including informed consent in cognitively impaired patients, the ethical implications of neuro technologies like deep brain stimulation and neuro prosthetics, decision‐making in end‐of‐life care, and ethical concerns in neurological research. Data were analysed thematically to identify recurring ethical challenges in clinical and research contexts.

**Results:** The review identified key ethical issues: (1) Informed consent and decision‐making in cognitively impaired patients, (2) Ethical concerns with neuro technologies, (3) End‐of‐life decisions in neurological diseases, and (4) Research ethics regarding vulnerable populations. Additionally, the potential ethical dilemmas surrounding neuroenhancement and brain‐computer interfaces were discussed.

**Conclusion:** Ethical challenges in neurology require adaptive frameworks that balance scientific progress with patient autonomy and dignity. As neuro technologies evolve, ongoing interdisciplinary discussions are necessary to develop comprehensive ethical guidelines for clinical practice and research.

**Disclosure:** Nothing to disclose.

## EPO‐660

### Maternal prenatal nut and seafood consumption and child neuropsychological function from 4 to 15 years of age

#### A. Pinar‐Martí ^1^; N. Ayala‐Aldana^1^; M. Foraster^2^; J. Julvez^1^

##### ^1^Clinical and Epidemiological Neuroscience (NeuroÈpia), Institut d’Investigació Sanitària Pere Virgili (IISPV), Reus, Spain; ^2^PHAGEX Research Group, Blanquerna School of Health Science, Universitat Ramon Llull (URL), Barcelona, Spain

**Background and Aims:** Understanding the role of maternal diet in early brain development is critical, as pregnancy represents a period of significant vulnerability and growth for the developing brain. The aim of this study was to assess the long‐term association of maternal nuts and seafood consumption during pregnancy with neuropsychological function in the offspring up to 15 years of age considering the potential mediation of omega‐3 fatty acids.

**Methods:** Conducted as part of the population‐based birth cohort study of the Spanish Childhood and Environment (INMA) Project, it followed 1737 mother‐child pairs from pregnancy until the children reached 15 years of age. Maternal dietary intake was evaluated using a semi‐quantitative food frequency questionnaire, and children's neuropsychological function was measured through standardized computer‐based tests. Linear mixed‐effects regression models were used to assess the association of nuts and seafood with all neuropsychological outcomes, while generalized structural equation modelling was used for mediation analyses.

**Results:** Results showed that higher maternal nut consumption was significantly linked to improved attention (HRT‐SE β = −0.05 milliseconds (ms), 95% CI = −0.09; −0.00, *p* for trend = 0.041) and working memory (d2’ β = 0.05, 95% CI = 0.00; 0.09, *p* for trend = 0.043, and d3’ β = 0.06, 95%CI = 0.02; 0.11, *p* for trend = 0.007) in offspring. Similarly, greater consumption of large fatty fish was associated with better attention (HRT‐SE β = −0.06 ms, 95% CI = −0.10; −0.02, *p* for trend = 0.004; and HRT β = −0.04 ms, 95% CI = −0.08; −0.00, *p* for trend = 0.032, respectively) and fluid intelligence (β = 0.08, 95% CI = 0.02; 0.13, *p* for trend = 0.006). Omega‐3 fatty acids mediated 8‐14% of these effects on attention.

**Conclusion:** Findings highlight the crucial role of maternal diet and omega‐3 intake in supporting long‐term cognitive development in children and adolescents.

**Disclosure:** Nothing to disclose.

## EPO‐661

### Multiple sclerosis from onset to secondary progression: A 30‐years Italian register study

#### A. Zanghì ^1^; M. Copetti^2^; E. D'Amico^1^; I. Registry^3^

##### ^1^University of Foggia, Italy; ^2^IRCSS Casa Sollievo della Sofferenza, San Giovanni Rotondo, Italy; ^3^MS Italian Registry, Italy

**Background and Aims:** Three decades have passed since the initial approval of disease modifying therapies (DMTs). Ongoing discussion is focused on fundamental aspects of the disease, highlighting a growing division between successes in managing relapsing Multiple Sclerosis (MS) and the persistent challenges posed by disease progression.

**Methods:** A cohort study on prospectively acquired data from the Italian MS register. The primary outcome was to describe the MS disease course from onset to secondary progression (SP) defined according to a data driven algorithm over 30‐years follow up and according to five different eras of disease onset.

**Results:** A total cohort of 9,958 patients was analyzed; 1,364 converted to SP after a mean of 8·5 (SD 5·5) years. A higher rate of patients converting to SP had never been exposed to DMTs (135, 9.9% vs 424, 5.2%) than non‐converting ones. The treatment coverage was also lower in patients converting to SP than non‐converting ones 58·4 (SD 31.5) vs 73·6 (SD 27.6). The 10‐years SP incidence rate was 1·26 (1.19–1.32) overall. The rates showed a downward trend among the different era: from 1st era 1·98 (1.73–2.27) to 5th era 1·15 (0.97–1.35). In the multivariable Cox model 10% increase of treatment coverage was associated to 19% lower risk to convert to SP (10% HR = 0.89, 95% CI = 0.87–0.90).

**Figure:**
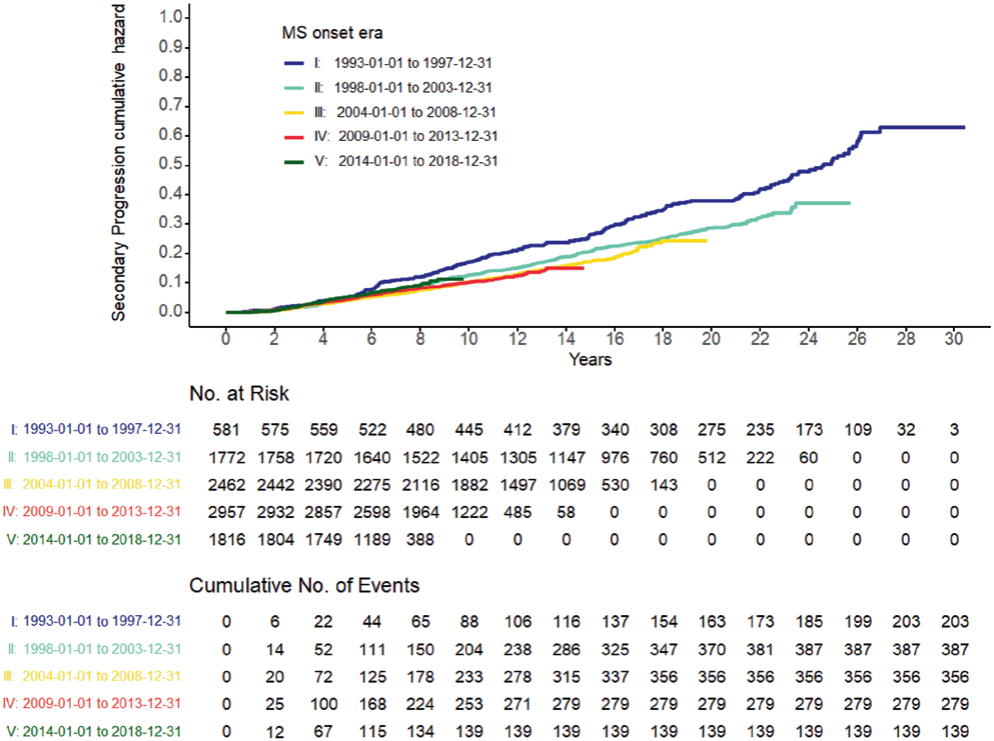
**FIGURE 1** Kaplan‐Meier curves for time to SPMS conversion according to disease onset era

**Figure:**
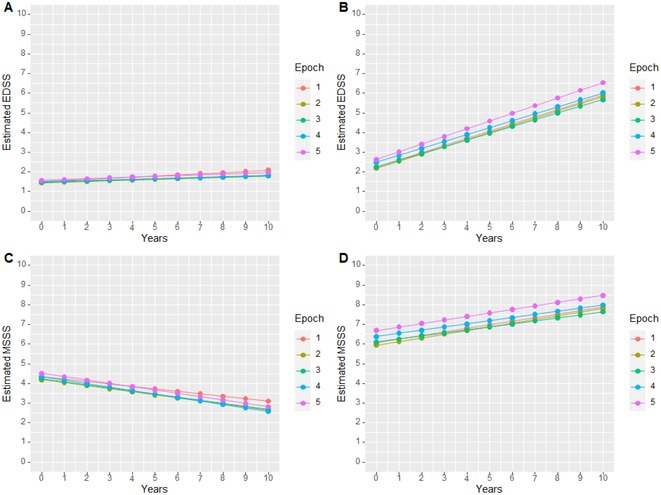
**FIGURE 2** Ten years longitudinal trajectories of EDSS and MSSS for converting to SP vs non converting patients (A) EDSS in non‐converting patients (B) EDSS in converting to SP patients (C) MSSS in non‐converting patients (D) MSSS in converting to SP patients E.

**Conclusion:** Further research is needed to explore the roles of inflammation and neurodegeneration in MS progression. These findings could inform clinical practice and health policy, emphasizing the need for continued therapeutic advancements.

**Disclosure:** Nothing to disclose.

## EPO‐663

### Exploring migration patterns and experiences of EAN RRFS members

#### D. Vlahovic ^1^; A. Accorroni^2^; N. Vashchenko^3^; A. Gonzalez‐Martinez^4^; K. Krzywicka^5^; L. Cuffaro^6^; V. Carvalho^7^; G. Sferruzza^8^

##### ^1^Neurology Clinic, University Clinical Centre of Vojvodina, Novi Sad, Serbia; ^2^Geneva Memory Center, Division of Geriatrics, Department of Rehabilitation and Geriatrics, Geneva University Hospitals, Geneva, Switzerland; ^3^I.M. Sechenov First Moscow State Medical University, Moscow, Russian Federation; ^4^Department of Neurology and Immunology, Hospital Universitario de la Princesa & Instituto de Investigación Sanitaria Princesa (IIS‐Princesa), Madrid, Spain; ^5^University Medical Center Groningen, The Netherlands, ^6^Università degli Studi di Milano‐Bicocca, Milano, Italy; ^7^Department of Neurosciences and Mental Health (Neurology) Hospital Santa Maria‐CHLN; ^8^Vita‐Salute San Raffaele University, Milan, Italy

**Background and Aims:** Trainees and young professionals are essential to advancing neurology and neuroscience, with migration significantly impacting their careers, and health systems. The EAN Residents and Research Fellows Section (RRFS) Migration Survey examined migration trends among its members, focusing on motivations, challenges, and personal and professional outcomes.

**Methods:** An anonymous online questionnaire was distributed to RRFS members from June to September 2024, collecting quantitative and qualitative data on demographics, migration history, and perceived impacts. Statistical and thematic analyses were conducted.

**Results:** The survey included 273 participants, with a median age of 31 years (IQR 5); 63.37% identified as female. Most were residents (59.23%), followed by young neurologists (20.77%) and PhD students (7.69%). 78.4% were from Europe, 16.14% from Asia, and 4.06% from Africa. 24.42% had already migrated, 27.91% were considering it, and 34.8% had considered but decided against it. Most migration occurred within Europe (52.38%), followed by Asia to Europe (14.29%) and Africa to Europe (6.35%). The primary motivations for migration were better working conditions and expanded education in emerging neurological fields (73.47%). Main challenges were language barriers and differences in teaching methods (52.35%). 30.64% would not return, but 83.87% would migrate again, and 56.45% would return if conditions improved. Respondents recommended expanding training, mentorship, and addressing workforce shortages to retain young professionals.

**Conclusion:** This survey underscores the significant role migration plays in shaping the careers of RRFS members, with implications for professional development and possibly for health professional shortage. Addressing integration challenges and improving conditions could support these professionals and mitigate long‐term workforce shortages.

**Disclosure:** Nothing to disclose.

## EPO‐664

### Abstract withdrawn

## EPO‐665

### Trends in ischemic stroke mortality among adults with atrial fibrillation in the US from 1999 to 2020

#### M. Ahmed ^1^; E. Mahboob^2^; M. Samad^2^; H. Ansari^2^; S. Chaudhry^3^; S. Samad^2^; R. Ahmed^4^; G. Imbianozor^5^; A. Alareed^6^; T. Hashmi^1^; H. Jain^7^

##### ^1^Rawalpindi Medical University, Rawalpindi, Pakistan; ^2^Dow University of Health Sciences, Karachi, Pakistan; ^3^ABWA Medical College, Faisalabad, Pakistan; ^4^National Heart & Lung Institute, Imperial College London, UK; ^5^Royal Wolverhampton NHS Trust, New Cross Hospital, Wolverhampton, UK; ^6^University Hospital Southampton NHS Foundation Trust; ^7^AIIMS Jodhpur, India

**Background and Aims:** Ischemic stroke (IS), a sequela of Atrial Fibrillation (AF), continues to be the world's second leading cause of death. Our study aims to identify demographic and regional disparities in mortality from IS amongst adult US population with AF.

**Methods:** Data from the National Vital Statistics System (1999–2020) was analyzed using CDC WONDER. IS deaths with AF as a contributing factor were identified, with results reported as age‐adjusted mortality rates (AAMR) per 100,000. Joinpoint regression analyzed trends and annual percentage changes (APCs).

**Results:** There were 130,937 IS deaths (AAMR = 2.7, 95% CI: 2.7–2.8). Females had higher AAMR (2.87) than males (2.45). Non‐Hispanic Whites (NHW) had the highest AAMR (2.8), followed by Non‐Hispanic Blacks (NHB) (2.06), Non‐Hispanic Asian/Pacific Islanders (NH‐API) (2.05), and Hispanics (1.8). Non‐Hispanic American Indian/Alaska Natives (NH‐AIAN) had the lowest (1.4). The West had the highest regional AAMR (3.3), with rural areas showing higher rates (3.1) than urban areas (2.6). Overall AAMR declined from 1999 to 2020 (APC: ‐0.4). NHW and NH‐API showed declines, while NHB and Hispanics had rising rates.

**Figure:**
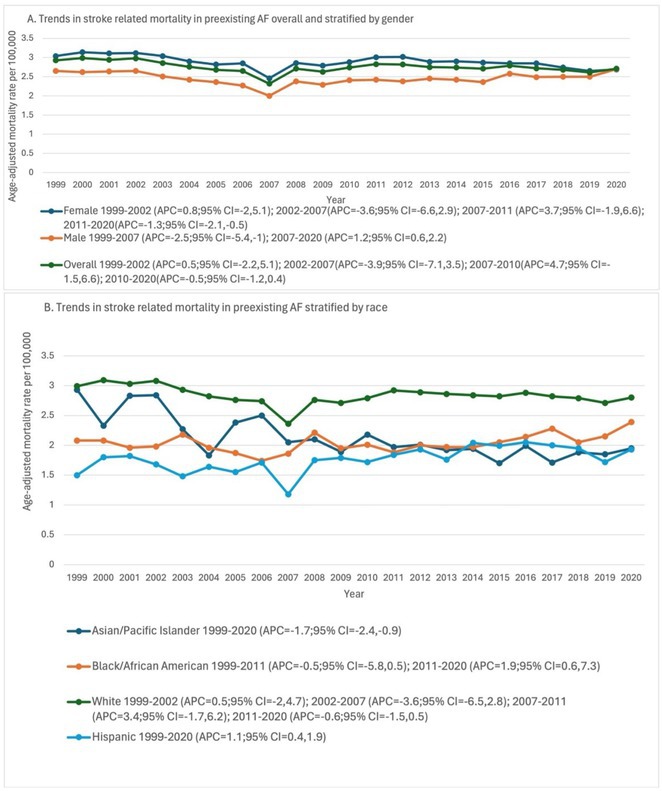
**FIGURE 1** Trends in mortality from ischemic stroke in adult US patients with atrial fibrillation from 1999 to 2020. (Panel A) Age‐adjusted mortality rates stratified by overall population and sex, (Panel B) Age‐adjusted mortality rates stratified by race

**Conclusion:** Our study reveals significant disparities in IS‐related mortality with females, NHW, and residents in the West as well as rural areas exhibiting higher mortality rates. These findings highlight the need of focused interventions and thoughtful healthcare resources allocation to enhance outcomes for the vulnerable populations.

**Disclosure:** NA.

## EPO‐666

### No increased risk of amyotrophic lateral sclerosis after traumatic head injury

#### L. Levison^1^; P. Jepsen^2^; P. Jepsen^3^; J. Blicher ^4^

##### ^1^Department of Neurology, Aarhus University Hospital, Aarhus, Denmark; ^2^Department of Clinical Epidemiology, Aarhus University Hospital, Aarhus, Denmark; ^3^Department of Hepatology and Gastroenterology, Aarhus University Hospital, Aarhus, Denmark; ^4^Department of Neurology, Aalborg University Hospital, Aarhus, Denmark

**Background and Aims:** Studies have identified an association between traumatic head injury and amyotrophic lateral sclerosis (ALS), but the causal relation remains uncertain. We aimed to determine whether hospital–diagnosed traumatic head injury is an ALS risk factor.

**Methods:** In this population‐based case‐control study, we used Danish nationwide health registers from 1980 to 2021 to identify hospital–diagnosed ALS patients. Each patient was matched 1:10 with individuals from the general population by age, sex, and calendar year. We used conditional logistic regression to examine the odds ratio (OR) of ALS associated with having prior hospital‐diagnosed traumatic head injury.

**Results:** Traumatic head injury was observed in 4.7% of 5,943 ALS cases vs 3.7% of 59,426 controls, with an OR of 1.3 (95% CI, 1.1–1.4). However, this association was caused by head injuries sustained shortly before the ALS diagnosis was made. Thus, the OR was 4.5 (95% CI, 2.8–7.3) within the 6 months prior to ALS diagnosis, and declined to 2.4 (95% CI, 1.4–4.0) 6 to 12 months prior to ALS diagnosis. Going further back in time, more than 3 years prior to ALS diagnosis, we found no association between traumatic head injury and ALS (OR,1.1 (95% CI, 1.0–1.3).

**Figure:**
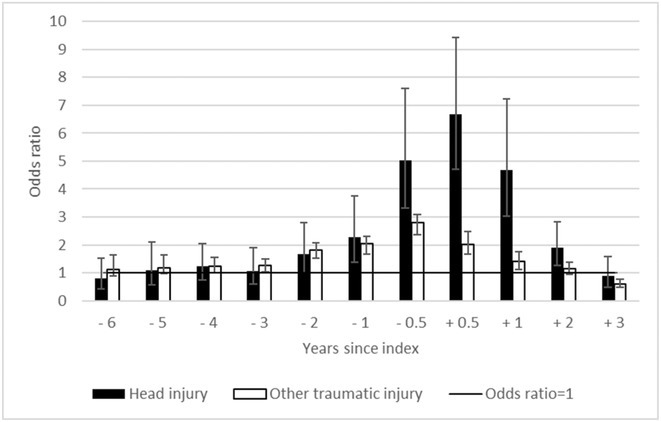
**FIGURE 1** Amyotrophic lateral sclerosis risk according to timing of traumatic head injury and other traumatic injuries.

**Conclusion:** There was a strong association of ALS with head injury experienced ≤1 year before ALS diagnosis, however our results suggest that this is due to reverse causation. Consequently, we do not consider hospital‐diagnosed traumatic head injury an ALS risk factor.

**Disclosure:** Nothing to disclose.

## EPO‐667

### Missing medical data in neurological emergency care compromise patient safety and healthcare resources

#### L. Krey ^1^; Z. Rabea^1^; O. Krause^2^; S. Greten^1^; J. Heck^3^; A. Boeck^1^; S. Petri^1^; F. Wegner^1^; M. Klietz^1^

##### ^1^Department of Neurology, Hannover Medical School, Hannover, Germany; ^2^Center for Geriatric Medicine, Hospital DIAKOVERE Henriettenstift, Hannover, Germany; ^3^Institute for Clinical Pharmacology, Hannover Medical School, Hannover, Germany

**Background and Aims:** We observed complications in the acute care of patients in the neurological emergency department (ED) when patients arrived without proper medical information in our clinical practice. The aim of this study was to assess systematically the frequency and consequences of incomplete medical data upon ED admission.

**Methods:** We assessed the availability and accuracy of medical data of all patients upon arrival to our neurological ED. Complications in emergency treatment due to missing medical information were recorded. Furthermore, we investigated whether initially missing data affect the inpatient stay of patients admitted via the ED.

**Results:** Our data show that medical data of 27% of the 272 included patients were incomplete upon admission. In this group, phone calls to gather information caused relevant delays, as they were necessary in 57% of those cases (vs. 22% in patients with complete data, p < 0.0001). Furthermore, unnecessary diagnostic procedures were performed in 5% of these patients, thus compromising patient safety. We show that even the inpatient stay was affected by initially missing data. Retrospectively, 5% of hospitalizations could have been avoided if all medical information had been available upon ED admission.

**Conclusion:** The acute care and the inpatient care of neurological patients is complicated by missing medical information. Our data show that this can compromise patient safety and lead to a waste of medical resources. We postulate that the implementation of a digital data management system in Germany could help to improve patient safety and facilitate efficient patient care in the ED and beyond.

**Disclosure:** Nothing to disclose.

## EPO‐668

### Equality in neuromuscular research: Analysis of 20 years of clinical trials

#### L. Fontanelli ^1^; G. Vadi^2^

##### ^1^Health Science Interdisciplinary Center, Sant’Anna School of Advanced Studies, Pisa, Italy; ^2^Department of Clinical and Experimental Medicine, University of Pisa, Pisa, Italy

**Background and Aims:** Equitable access to clinical research is a critical imperative, yet underrepresentation of non‐white and non‐male individuals remains a widespread issue, constituting a widespread lack of diversity in clinical trials.

**Methods:** In this study, we conducted a systematic analysis of clinical trials on neuromuscular diseases registered on ClinicalTtrials.gov over the last 20 years to assess the representation of sex, race and ethnicity.

**Results:** Two thousand fifty‐four studies were screened for eligibility and 469 studies were included in the analysis. Race was reported in standard terminology in 223 studies, encompassing 17860 patients. Thirtynine (0.2%) patients were American Indian or Alaska Native, 8.4% were Asian, 2.2% were Black, 0.2% were Native Hawaian or other pacific islander, 83.5% were White; 0.6% patients were listed as more than one race and 4.8% as unknown. Race and ethnicity distributions per diseases are shown in Fig.1. We observed a significant increase in study reporting race over time (*p* < 0.001). However, year did not significantly influence the racial composition nor the ethnicity in the clinical studies over time (*p* = 1) (Fig. 2). All the studies reported sex amongst two categories (i.e.: male, female), while one observational study reported gender without sex. Most patients, both in observational and in interventional studies were male (60.1% and 60.4%, respectively) (Fig. 3).

**Figure:**
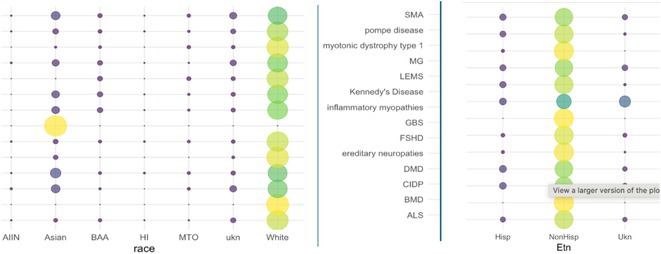
**FIGURE 1** Race and ethnicity distribution across neuromuscular diseases. The majority of people are non hispanic white.

**Figure:**
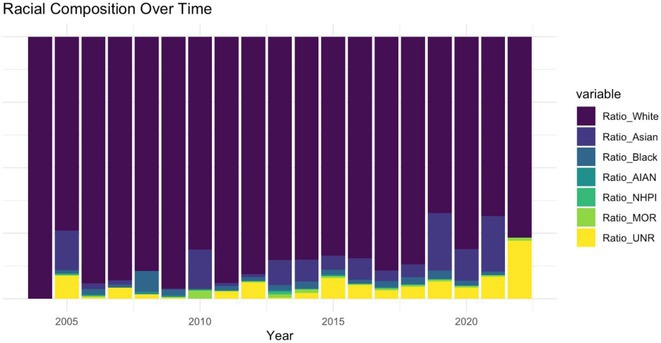
**FIGURE 2** Race composition over time. We did not find any significant difference of racial composition over time.

**Figure:**
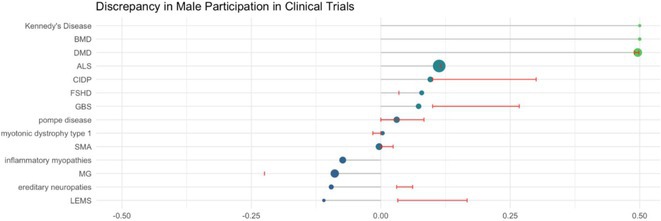
**FIGURE 3** Sex distribution across neuromuscular diseases. Zero means equal number of male and female. Disease male/female ratio is represented by red lines.

**Conclusion:** This study quantify sex, race and ethnicity disproportion in neuromuscular diseases trials. Over time, race reporting increases, while majority of patients continue to be non hispanic white. While the majority of people are male, the distribution varies across diseases.

**Disclosure:** Nothing to disclose.

## EPO‐669

### Epidemiology of functional neurological disorders: A systematic review

#### O. Mounir Alaoui ^1^; E. Roze^2^; A. Daubigney^3^; H. Amieva^4^; B. Garcin^1^

##### ^1^Department of Neurology, Hopital Avicenne, APHP, Bobigny, France; ^2^Department of Neurology, Hopital Pitié Salpêtrière, APHP, Paris, France; ^3^Equipe Mobile de NeuroPsychiatrie, Pôle de Neurosciences Cliniques, CHU de Bordeaux et Pôle de Psychiatrie Générale et Universitaire du Centre Hospitalier Charles Perrens, Bordeaux, France; ^4^Inserm U1219 Bordeaux Population Health Center, University of Bordeaux, Bordeaux, France

**Background and Aims:** Fnctional neurological disorders (FND) are characterized by motor, sensory, and/or cognitive symptoms that relate to functional rather than structural abnormalities. Although these disorders are common in neurology, their epidemiology is not well‐documented.

**Methods:** Following the Preferred Reporting Items for Systematic Reviews and Meta‐analyses guidelines, we searched PubMed and Embase for articles reporting on the incidence or prevalence of FND in adults, published in French or English between 1972 and 2022. We used The Joanna Briggs Institute Prevalence Critical Appraisal Tool to assess the quality of the studies. This study was registered under the ID CRD42023434331 in the PROSPERO database.

**Results:** Out of 4,260 screened articles, 27 were included, primarily from India, the US, and Europe. The prevalence estimates for hysteria, conversion disorders or FND ranged from 37.2 to 6,900 per 100,000, with an incidence ranging from 10.7 to 186 per 100,000. Functional dissociative seizures had a prevalence of 23.8 to 890 per 100,000 and an of 0.91 to 4.9 per 100,000. Functional motor disorders had an incidence of 3.9 to 5.0 per 100,000. Most cases involved young women. Only 8 studies were rated as high quality. Overall, the rapidly changing nosology and diagnostic criteria complicate the interpretations of existing data.

**Conclusion:** Our findings highlight the urgent need for large‐scale, rigorous studies targeting the multiple forms of FND to obtain reliable epidemiological data that are essential to develop an adequate health policy for FND.

**Disclosure:** Nothing to disclose.

## EPO‐670

### The age at onset of LRRK2‐related Parkinson's disease across ancestries and countries of origin

#### T. Lüth ^1^; B. Laabs^2^; S. Sendel^3^; I. König^2^; A. Caliebe^3^; A. Noyce^4^; L. Screven^5^; S. Bardien^6^; M. Farrer^7^; C. Klein^1^; S. Ben Sassi^8^; J. Trinh^1^; G. Global Parkinson's Genetics Program^9^

##### ^1^Institute of Neurogenetics, University of Lübeck, Lübeck, Germany; ^2^Institute of Medical Biometry and Statistics, University of Lübeck, Lübeck, Germany; ^3^Institute of Medical Informatics and Statistics, Kiel University and University Hospital Schleswig‐Holstein, Kiel, Germany; ^4^Centre for Preventive Neurology, Wolfson Institute of Population Health, Queen Mary University of London, London, UK; ^5^National Institute on Aging, Bethesda, USA; ^6^Division of Molecular Biology and Human Genetics, Faculty of Medicine and Health Sciences, Stellenbosch University and South African Medical Research Council/Stellenbosch University Genomics of Brain Disorders Research Unit, Cape Town, South Africa; ^7^Department of Neurology, University of Florida, Gainesville, USA; ^8^Faculty of Medicine of Tunis, Tunisia/Neurology's Department, Mongi Ben Hmida National Institute of Neurology, Tunis, Tunisia; ^9^Global Parkinson's Genetics Program

**Background and Aims:** The LRRK2 p.G2019S pathogenic variant has reduced penetrance and presents with a wide range of age at onset (AAO) in patients with Parkinson's disease (PD). Herein, we investigate cumulative incidence differences of LRRK2‐PD across ancestries and countries.

**Methods:** We included *N* = 922 unrelated LRRK2 p.G2019S variant carriers (affected: *N* = 762, unaffected: *N* = 160) from the Global Parkinson's Genetics Program (GP2) in addition to cohorts from the Israeli Ashkenazi Jewish and Tunisian Arab‐Berber population. The sex‐adjusted Cox proportional‐hazards model and Kaplan‐Meier analysis were applied to examine differences in cumulative incidence between ancestries derived from genetic data and countries.

**Results:** We observed ancestry‐specific differences, as there was a median five‐year younger AAO of LRRK2‐PD in the North African (HR = 1.48, *p* = 7.0e‐4) compared to the European ancestry group. In contrast, the median AAO was five years older in the Ashkenazi Jewish ancestry group (HR = 0.61, *p* = 4.0e‐6). Secondly, the country also showed differences. Patients from Israel (HR = 1.59, *p* = 4.0e‐6) and Tunisia (HR = 2.57, *p* < 2.0e‐16) had a median 5‐year and 10‐year younger AAO compared to patients from the USA, respectively. Thirdly, when focusing only on individuals with Ashkenazi Jewish ancestry, persons from Israel still had a younger AAO than those from the USA (HR = 1.82, *p* = 1.5e‐8). Analogously, assessing only persons from the USA, the Ashkenazi Jewish ancestry group still had an older AAO than the European (HR = 0.51, *p* = 1.3e‐6).

**Conclusion:** Our results provide evidence that a person's ancestry and country of origin are associated with the AAO of LRRK2‐PD. This highlights the impact of both genetic and environmental factors on LRRK2‐PD AAO.

**Disclosure:** This project was supported by the DFG RU ProtectMove (DFG FOR2488) and by a DFG Heisenberg Grant to JT. Data used in the preparation of this abstract were obtained from Global Parkinson's Genetics Program (GP2). GP2 is funded by the Aligning Science Across Parkinson's (ASAP) initiative and implemented by The Michael J. Fox Foundation for Parkinson's Research (https://gp2.org). For a complete list of GP2 members see https://gp2.org.

## EPO‐671

### Impact of hospital teaching status, insurance status, race, & income on mortality in subarachnoid hemorrhage (2019–2021)

#### Z. Krayem ^1^; M. Chohan^3^; M. Ahmed^2^

##### ^1^Rowan Virtua School of Osteopathic Medicine, Stratford, USA; ^2^AtlantiCare Regional Medical Center, Atlantic City, USA; ^3^Jefferson Health, Philadelphia, USA

**Background and Aims:** Subarachnoid Hemorrhage (SAH) is a stroke caused by artery rupture, leading to bleeding in the brain. SAH can have profound neurologic effects, including severe disability and death if not promptly diagnosed and treated. This study examines mortality differences based on hospital teaching status, payer status, income, and race in the United States (US).

**Methods:** Data was sourced from 2019 to 2021 National Inpatient Sample (NIS), representing a 20% stratified sample of US community hospitals. Mortality rates were compared across rural, urban non‐teaching, and urban‐teaching hospitals. Statistical analyses were performed using Chi Square and Logistic regression in StataNow18.

**Results:** Of 137,537 SAH admissions, 88.6% were treated at urban‐teaching hospitals, 9.1% at urban non‐teaching hospitals, and 2.3% at rural hospitals. The overall mortality rate was 23.5%, highest at rural (26.8%, OR = 1.21) and urban non‐teaching hospitals (25.7%, OR = 1.15), and lowest at urban‐teaching hospitals (23.2%) (*p* < 0.05). Self‐pay patients had the highest mortality rate (30.0%, OR = 1.33), followed by Medicare (24.4%) and Medicaid (23.7%), while private insurance had the lowest (19.8%, OR = 0.77). Native Americans had the highest mortality rate (30.6%, OR = 1.49), followed by Asian/Pacific Islanders (27.1%, OR = 1.25), compared to Whites (22.9%) (*p* < 0.05). Mortality decreased with increasing income, from 24.8% in the lowest income quartile to 22.5% in the highest (*p* < 0.05).

**Figure:**
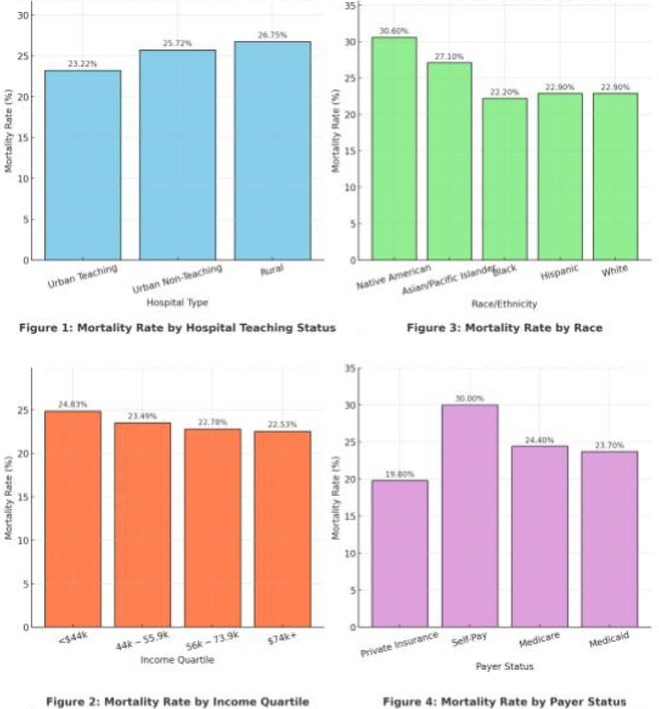
**FIGURE 1** Mortality Rates by Hospital Teaching Status, Race, Income, and Payer Status: Hospital death rates were highest among Native American patients, uninsured individuals, and those treated in non‐teaching hospitals.

**Conclusion:** This study reveals disparities in mortality among patients with SAH across hospital teaching status, socioeconomic status, and race. Notably, mortality rates were highest in non‐teaching hospitals, which likely reflects the substantial impact of limited resources and access to specialized care in these settings.

**Disclosure:** Nothing to disclose.

## Motor neurone diseases & spinal cord and root disorders

## EPO‐672

### Spontaneous intracranial hypotension: A tertiary centre experience

#### A. Neves ^1^; M. Pinto^1^; I. Santos Neto^2^; T. Oliveira Pedro^3^; L. Braz^1^; J. Fonseca^3^; P. Pereira^4^; M. Gomes^5^; P. Trigo Barbosa^5^; J. Guimarães^1^

##### ^1^Neurology Department, Local Health Unit of São João, Porto, Portugal; Department of Clinical Neurosciences and Mental Health, Faculty of Medicine, University of Porto, Porto, Portugal; ^2^Northern Rehabilitation Centre, Local Health Unit of Vila Nova de Gaia e Espinho, Porto, Portugal; ^3^Neuroradiology Department, Local Health Unit of São João, Porto, Portugal; ^4^Neurosurgery Department, Local Health Unit of São João, Porto, Portugal; Department of Clinical Neurosciences and Mental Health, Faculty of Medicine, University of Porto, Porto, Portugal; ^5^Anesthesiology Department, Multidisciplinary Chronic Pain Unit, Local Health Unit of São João, Porto, Portugal

**Background and Aims:** Spontaneous intracranial hypotension (SIH) requires a systematic and early therapeutic approach to prevent complications. The aim of this study is to describe patients with SIH followed up in a tertiary hospital.

**Methods:** Retrospective observational study including adults with HIE in a tertiary centre (2017–2024). We describe demographic parameters, clinical presentation, complementary diagnostic methods, treatments and outcomes.

**Results:** Fourteen of the 22 patients were female (64%), with a mean age of 42 (23‐57) years. In thirteen patients, a trigger was identified (Valsalva manoeuvre or minor trauma). All of them presented with orthostatic headache, associated with nausea/vomiting (82%) and neck pain (73%). Six patients had neurological examination alterations and eleven (50%) had complications – unilateral (*n* = 1) and bilateral (*n* = 9) subdural haematoma, cerebral venous thrombosis (*n* = 2) and high convexity subarachnoid haemorrhage (*n* = 1). All underwent brain computed tomography/magnetic resonance (MRI), thirteen proceed with myelo‐MRI. No aetiology was identified in twelve patients, with dural defect being the most common cause (*n* = 9). All were treated conservatively according to internal protocol, with complete resolution in four patients. Eighteen patients required a non‐targeted blood patch, with 11 requiring more than one. Three patients underwent a blood patch directed at the point of fistula. Two patients required neurosurgical intervention. After discharge, two patients were left with residual headaches.

**Conclusion:** Half of the patients had pre‐treatment complications and the majority did not respond to conservative treatment. The authors advocate an interdisciplinary, protocol‐based approach that allows early symptomatic relief and minimises complications.

**Disclosure:** Nothing to disclose.

## EPO‐673

### Relative leukopenia in C9ALS

#### A. Ghezzi ^1^; G. Gianferrari^1^; E. Zucchi^1^; I. Martinelli^2^; N. Ticozzi^3^; N. Fini^2^; J. Mandrioli^1^

##### ^1^Department of Biomedical, Metabolic and Neural Sciences, University of Modena and Reggio Emilia, Modena, Italy; ^2^Neurology Unit, Azienda Ospedaliero Universitaria di Modena, Modena, Italy; ^3^Department of Neurology and Laboratory of Neuroscience, IRCCS Istituto Auxologico Italiano, Milan, Italy

**Background and Aims:** Hexanucleotide repeat expansions in C9ORF72 are the most frequent genetic cause of familial and sporadic amyotrophic lateral sclerosis (ALS). Among the functions attributed to C9ORF72 there's a role in inflammation and immunity. Knock‐out of C9ORF72 in preclinical models resulted in autoimmune and neuroinflammatory phenotypes without clear signs of neurodegeneration. Considering the established role of C9ORF72 in immunity, we searched for alterations in the blood cells count (BCC) in C9ALS patients.

**Methods:** We retrospectively collected the BCC and the main clinical parameters from 92 C9ALS patients and 184 sex, age and diagnostic delay‐matched sporadic ALS controls as close as possible to diagnosis, carefully avoiding samples drawn in cases of concomitant infections and other acute conditions that could temporarily modify the BCC.

**Results:** We found a significant reduction for C9ALS in the global leukocyte count and in all the subpopulations (neutrophils, lymphocytes, monocytes, basophils, eosinophils), as well as a significant increase in neutrophils to eosinophils ratio, lymphocytes to eosinophils ratio and monocyte to eosinophils ratio. Despite the time from diagnosis to sampling was shorter in C9ALS patients compared to sporadic patients, no correlation with the sampling delay was found. No significant correlation with the main clinical parameters and with overall survival was found.

**Conclusion:** Our work shows that C9ALS is characterized by a relative leukopenia that involves both the myeloid and in the lymphoid lines, which only partially overlap with what is known from previous studies on animals and humans, suggesting that immune dysregulation in C9ALS could differ from that of sporadic ALS patients.

**Disclosure:** Nothing to disclose.

## EPO‐674

### A nationwide survey of prevalence and symptom burden of pseudobulbar affect in amyotrophic lateral sclerosis

#### C. Steenkjaer ^1^; J. Storgaard^2^; L. Levison^2^; J. Blicher^1^

##### ^1^Department of Neurology, Aalborg University Hospital, Denmark; ^2^Department of Neurology, Aarhus University Hospital, Denmark

**Background and Aims:** Our aim was to determine diagnostic prevalence of pseudobulbar affect (PBA) and symptom burden in patients with amyotrophic lateral sclerosis (ALS) in Denmark.

**Methods:** In this nationwide cross‐sectional survey, we included citizens diagnosed with ALS who had a hospital contact in Denmark between January 2010 and July 2024 according to the Danish National Patient Register. Patients were invited between August and November 2024 to complete an online survey. The survey contained questions including known PBA diagnosis, treatment and quantification of symptoms through the CNS‐Lability Scale (CNS‐LS). A CNS‐LS score of 13 or higher served as a threshold for possible symptoms of PBA.

**Results:** As shown in Figure 1, 679 patients were invited, of whom 157 patients with ALS completed the survey. 12.1% reported known PBA and the entire study population had a mean CNS‐LS score of 11.3 ± 4.8. Patients with known PBA had a higher mean CNS‐LS score (17.2 ± 4.7) compared to those not diagnosed with PBA (10.5 ± 4.3, *p* < 0.001). Patients with known PBA were more likely to receive antidepressant medication (47.4% compared to 15.2%, *p* = 0.002). In treated PBA, patients were predominantly treated with citalopram (78%). In the entire study population, 30.6% scored 13 or higher in the CNS‐LS. Of those not known with PBA, 23.2% scored 13 or higher.

**Figure:**
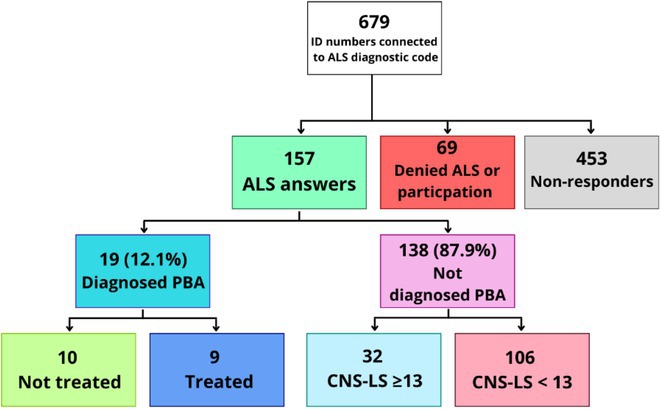
**FIGURE 1** Flowchart of recruitment and highlighted survey response findings. ALS: amyotrophic lateral sclerosis, PBA: Pseudobulbar Affect, CNS‐LS: CNS‐Lability Scale.

**Conclusion:** While the proportion of known PBA among the ALS population was relatively low, the proportion of patients with symptoms of possible PBA was markedly higher. These findings may indicate under‐ or misdiagnosis of PBA among ALS patients, leading to lack of recommended symptomatic treatment.

**Disclosure:** Nothing to disclose.

## EPO‐675

### iRNA nanoparticles in ALS mouse model treatment

#### D. Kuzin; D. Labunskiy; S. Kiryukhina; N. Kurgaev; D. Baranov

##### Ogarev Mordovia State Universitykyzmindi, Saransk, Russian Federation

**Background and Aims:** Amyotrophic lateral sclerosis (ALS) is caused by the progressive death of nerve cells in the spinal cord and brain. This rare disease causes weakness, loss of muscle mass, and eventually paralysis. There is currently no treatment for ALS, and existing therapies can only slightly slow the progression of the disease

**Methods:** The therapeutic agent in this study was a small interfering RNA (siRNA) molecule designed to suppress the expression of the tau protein, which is believed to play a key role in neurodegenerative changes. The base material for the nanoparticles was a polymer consisting of chains of lactic and glycolic acid. It has been previously discovered that mesenchymal stromal cells, stem cells that can turn into bone, muscle, fat, and other tissues, can also replace dead motor neurons in spinal cord of ALS patients. siRNA nanoparticles significantly enhance the therapeutic effect of mesenchymal stromal cells. We studied surface properties of the nanoparticles to maximize their penetration across the intact BBB in transgenic SOD1 mutant mice, analysed locomotor activity of tyese mice before and after electrophoresis with siRNA nanoparticles, also IL2, and IL6 cytokines, recombinant proteins of adhesion molecules were evaluated.

**Results:** iRNA nanoparticles causes better locomotion activity, normalization of IL2, and IL6 cytokines, recombinant proteins of adhesion molecules SOD1 mutant mice serum.

**Figure:**
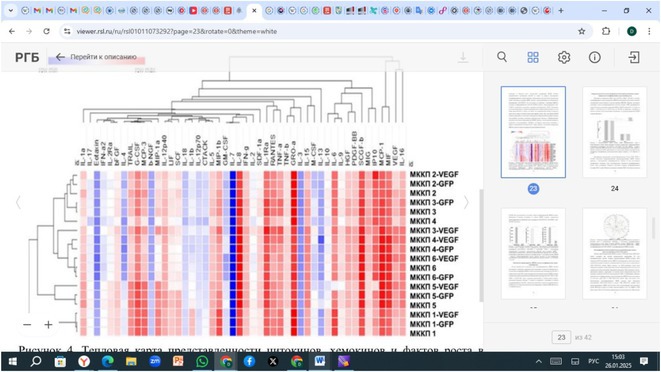
**FIGURE 1** Cytokines in SOD1 mice

**Figure:**
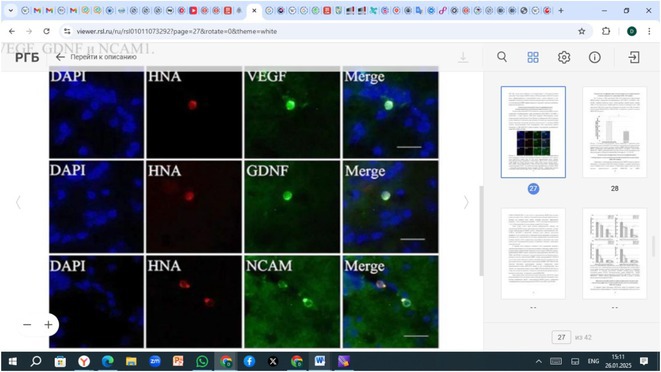
**FIGURE 2** Recombinant proteins in SOD 1mice

**Conclusion:** This led to the identification of a unique nanoparticle design that increased the transport of encapsulated siRNA across the intact BBB and significantly improved the uptake of the drug by brain cells.

**Disclosure:** Nothing to disclose.

## EPO‐676

### Sphingosine‐1‐phosphate as a predictor of neurological deterioration in spinal bulbar muscular atrophy

#### J. Park ^1^; J. Kim^2^

##### ^1^Department of Neurology, School of Medicine, Kyungpook National University Chilgok Hospital, Daegu, Republic of Korea; ^2^College of Pharmacy, Yeungnam University, Gyeongsan, Republic of Korea

**Background and Aims:** Spinal and bulbar muscular atrophy (SBMA) is a genetic motor neuron disease that show slowly progressive muscular weakness and atrophy, caused by increased CAG repeats in the exon 1 of the androgen receptor gene. There are clinical markers that are used to predict neurological deterioration but the lack of quantitative biomarkers limits the sensitive evaluation. This study aimed to identify potential biomarkers associated with SBMA progression.

**Methods:** Plasma samples from 21 SBMA patients were collected at baseline and 3‐4 years post‐diagnosis, were employed to untargeted and targeted metabolomics using liquid chromatography‐mass spectrometry. The levels of identified metabolites were further analyzed in relation to the rate of disease progression, which was defined by changes in ALSFRS‐R scores during the follow‐up period.

**Results:** Plasma S1P concentrations emerged as a promising marker for diagnosing and monitoring SBMA progression, as identified through targeted and untargeted metabolomics. Plasma S1P levels showed a significant decrease over the follow‐up period in the fast progression group, defined by ALSFRS‐R changes greater than ‐3 points. Additionally, follow‐up S1P concentrations showed positive correlations with ALSFRS‐R that showed negative correlation with serum creatine kinase levels.

**Conclusion:** Plasma S1P showed a promising diagnostic marker for SBMA. Despite the inherent variability in S1P levels requiring careful interpretation, it might be a novel marker reflecting neurological progression. More studies are needed to understand the underlying patho‐mechanism of S1P and its regulation in SBMA, to strengthen the validity of these findings.

**Disclosure:** Nothing to disclose.

## EPO‐677

### Spontaneous spinal cord infarction: A rare disease with less frequent aetiologies

#### A. Morgadinho; M. Nascimento; L. Pereira; P. Pereira; M. Rodrigues

##### Department of Neurology, Hospital Garcia de Orta, Almada, Portugal

**Background and Aims:** Spinal cord infarction is a rare condition, most often seen as a surgical complication. Spontaneous causes closely resemble those of ischaemic stroke. This case highlights the challenges in finding the etiology of spinal cord infarction.

**Methods:** N/A.

**Results:** A 60‐year‐old woman with a history of hypertension presented with sudden onset of severe abdominal pain, followed by bilateral sensory loss up to the umbilicus and reduced muscle strength in the lower limbs. Surgical acute abdomen and trauma were excluded. Neurological examination revealed flaccid hyperreflexic grade 2 paraparesis and anaesthesia with a sensory level at D7, raising suspicion of spinal cord involvement. Thoracic‐abdominal‐pelvic CT angiography excluded aortic dissection but identified pulmonary thromboembolism (PTE). The patient was outside the therapeutic window for thrombolysis, and anticoagulation was initiated for PTE. During inpatient care, MRI confirmed infarction of the anterior spinal artery spanning levels D8 to D12. Transcranial Doppler ultrasound, performed to detect microembolic signals, identified a right‐to‐left shunt, and transoesophageal echocardiography confirmed the presence of patent foramen ovale (PFO). Anticoagulation was complicated by lower bowel bleeding, prompting a colonoscopy that revealed a colonic adenocarcinoma, subsequently resected. At discharge, following rehabilitation, the patient had not yet achieved autonomous gait.

**Conclusion:** Two less common potential causes of spinal cord infarction: paradoxical embolism through a PFO in the context of PTE, and a prothrombotic state induced by neoplasia, implicated in the occurrence of PTE and spinal cord ischemia. Management in such cases should consider PFO closure and the maintenance of long‐term anticoagulation.

**Disclosure:** Nothing to disclose.

## EPO‐678

### Subacute myelopathy due to nitrous oxide inhalation: An emerging etiology among young adults. A case series

#### L. Giramé‐Rizzo ^1^; A. Vilaseca^4^; J. Mayol Traveria^1^; M. Alanís Bernal^1^; E. Saez de Gordoa^3^; A. Martí Carretero^2^; J. Castilló^4^; A. Zabalza^4^; C. Auger^3^; X. Montalban^4^

##### ^1^Neurology Department, Vall d’Hebron University Hospital, Universitat Autònoma de Barcelona, Spain; ^2^Internal Medicine Department, Vall d’Hebron University Hospital, Universitat Autònoma de Barcelona, Spain; ^3^MRI unit, Vall d’Hebron University Hospital, Barcelona, Spain; ^4^MS Centre of Catalonia (Cemcat), Vall d’Hebron University Hospital, Universitat Autònoma de Barcelona, Spain

**Background and Aims:** Inhaling nitrous oxide (N_2_O) can result in functional deficiency of vitamin B12, which may cause neurological toxicity, including myelopathy. The objective is to describe two cases of subacute myelopathy caused by N_2_O inhalation.

**Methods:** We report two patients diagnosed with subacute myelopathy due to N_2_O inhalation admitted to our center.

**Results:** A 33‐year‐old male with a 4‐week‐progressive‐ascending hypoesthesia following a stocking‐and‐glove pattern associated with areflexia and dysautonomia. Slightly decreased B12 levels (291 pg/mL). Brain and spinal cord MRI showed a longitudinally extensive spinal cord lesion from C1 to T10, mainly involving dorsal columns, mimicking Neuromielitis optica spectrum disorder lesions. A 20‐year‐old male with a 3‐month‐progressive‐ascending hypoesthesia with a sensory level in T10–T12 associated with areflexia and sensory ataxia. Decreased B12 levels (153 pg/mL). Despite a normal MRI, the neurophysiological study revealed severe involvement of dorsal columns and pyramidal tract in the lower limbs, alongside axonal sensorimotor polyneuropathy. Both patients disclosed prior use of inhaled nitrous oxide (N_2_O) in a binge pattern before the onset of symptoms and other potential etiologies were excluded. The 1st case was treated with 1g of intravenous methylprednisolone once daily for 5 days and B12 supplementation, while the 2nd only received B12 supplementation. In the subsequent days, both patients experienced remarkable clinical improvement, presenting only mild symptoms by the time of discharge.

**Conclusion:** N_2_O is a rare cause of subacute myelopathy in young individuals and should be taken into account in the differential diagnosis due to its reversibility when treated promptly.

**Disclosure:** Nothing to disclose.

## EPO‐679

### Neutrophil percentage‐to‐albumin ratio as a possible biomarker for disease progression in amyotrophic lateral sclerosis

#### L. Ferullo ^1^; E. Olivieri^1^; L. Poli^2^; B. Risi^3^; F. Caria^3^; V. Bozzoni^2^; B. Labella^1^; T. Merati^1^; A. Padovani^1^; M. Filosto^1^

##### ^1^Department of Clinical and Experimental Sciences, University of Brescia, Brescia, Italy; ^2^Unit of Neurology, ASST Spedali Civili, Brescia, Italy; ^3^NeMO‐Brescia Clinical Center for Neuromuscular Diseases, Brescia, Italy

**Background and Aims:** recent studies highlight the role of serum inflammatory markers in Amyotrophic Lateral Sclerosis (ALS) and the neutrophil‐to‐lymphocyte ratio (NLR) has proposed as a reliable marker. Recently, the neutrophil percentage‐to‐albumin ratio (NPAR) has proposed as a marker of inflammation, demonstrating prognostic utility in ischemic stroke, intracerebral haemorrhage, and cognitive decline. This study investigates its role in ALS.

**Methods:** we retrospectively analyzed 3,263 ALS patients with NPAR values recorded within ±90 days of recruitment from PRO‐ACT. The median age was 57 years (IQR: 49–75); 62.7% were male and 37.3% female. During follow‐up, 28.7% of patients died or underwent tracheostomy. NPAR values were stratified into tertiles. Statistical comparisons between groups were conducted with Chi‐square and Kruskal‐Wallis test as appropriate. Logistic regression was employed to evaluate associations between tertiles and clinical outcomes. Patient survival was assessed via Cox regression.

**Results:** NPAR showed significant differences across tertiles in age at symptom onset, ALSFRS‐R score, disease progression rate (DPR) at recruitment, and follow‐up days (*p* < 0.001). Higher NPAR values were associated with later diagnoses, lower ALSFRS‐R scores, faster DPR, and shorter follow‐up. Logistic regression revealed significant associations with clinical outcomes (*p* < 0.001). Cox regression showed reduced survival in group with higher NPAR (OR: 1.32; CI: 1.12–1.54), even after adjusting for age, gender, ethnicity, and site of symptom onset. The work was done using a public database (PRO‐ACT).

**Conclusion:** in a large cohort of ALS patients, NPAR appears to be a potential accessible blood biomarker for predicting clinical outcomes, supporting the role of neuroinflammation in ALS.

**Disclosure:** Nothing to disclose.

## EPO‐680

### West Yorkshire miniseries cases studies of split‐hand feature in appendicular and bulbar onset MND and MND mimics

#### M. Thura ^1^; A. Al‐Samak^3^; K. Lwin^1^; A. Thet^2^; S. Tun^2^

##### ^1^Department of Neurophysiology, Calderdale Royal Hospital, Halifax, UK; ^2^Department of Neurophysiology, Pinderfields General Hospital, Wakefield, UK; ^3^Department of Neurophysiology, Harrogate District Hospital, Harrogate, UK

**Background and Aims:** Split‐hand phenomenon is reportedly seen in 70% of ALS in early stages of MND. A neurophysiological tool to support a clinical diagnosis of a MND appeared as split hand index (CMAP APB X FDI/CMAP ADM) = <5.2 and two CMAP ratios of thenar/hypothenar muscles (APB/ADM = <0.6, FDI/ADM = <0.9, ADM/APB >1.7). We assessed the utility of this in our cases.

**Methods:** Data were collected from the records of patients diagnosed as possible MND in our neurophysiology clinics from (8/2020 to 11/2024).

**Results:** Twelve appendicular onset (AO) MND patients and eight bulbar onset (BO) cases were included. There were four cases of Hirayama. None of the bulbar onset MND cases and Hirayama's cases revealed the split hand phenomenon. All AO cases did. AO cases were seen six months to a year from symptoms onset. BO cases were seen within a year. Dysarthria, dysphagia, dysphonia and respiratory failure were seen in BO cases and four of them died in two months to two years later. One patient never developed appendicular symptoms. The others developed appendicular weakness in three months. AO cases started as weakness and cramps in one limb and spread to involve all four limbs. Three patients developed bulbar symptoms and one died. Four are still surviving. Split hand index were negative for all bulbar and Hirayama cases.

**Conclusion:** Any test to support the clinical diagnosis of MND is valuable. We found those ratios and index helpful in appendicular onset MND. They were negative in our series for bulbar onset MND and MND mimics cases.

**Disclosure:** Nothing to disclose.

## EPO‐681

### Subacute combined degeneration in relation to nitrous oxide abuse: An emerging disorder

#### R. Boto Martinez; I. Catalina Alvarez; A. Contreras Chicote; A. Alungulese; M. Lozano Lopez; L. Portela Martinez; J. Sosa Luis; J. Garcia Dominguez

##### Neurology Department, Hospital General Universitario Gregorio Marañon, Madrid, Spain

**Background and Aims:** Neurological symptoms resulting from recreational use of nitrous oxide (N2O), also known as laughing gas, are an emerging social problem in Europe. This drug is easily available at nightclubs and online shops, producing an epidemic that affects young adults. We report six patients with neurological impairment after excessive N_2_O consumption.

**Methods:** We identified all patients with final diagnosis of neurological disorder due to N_2_O abuse, presenting to a tertiary hospital in Spain, in the year 2023–2024.

**Results:** Total of six cases: Young men (19–30 years old) with a history of both chronic and high‐dose N_2_O inhalation. The predominant feature in all of them was ascending paresthesias in the lower limbs and associated gait ataxia. Altered MRI signal as myelopathy was observed in four out of six patients. Demyelinating features in the motor nerves were found in four of the six cases. One patient developed visual impairment in the form of retinitis. Additional tests ruled out other etiologies. Vitamin B12 treatment was initiated in five patients. Three patients discontinued N_2_O use with partial clinical improvement, persisting mild sensitive symptoms.

**Conclusion:** Neurological disorders due to N_2_O have been linked to vitamin B12 deficiency through interference with its metabolism, leading to demyelination. Empirical treatment consists of hydroxocobalamin injections, with uncertain functional prognosis. It is imperative to raise awareness among clinicians about the risks associated with this practice, including visual loss (not reported in existing literature) and the importance of considering N2O abuse in the differential diagnosis of central and/or peripheral demyelinating diseases in young patients.

**Disclosure:** Nothing to disclose.

## EPO‐683

### Cramp‐fasciculation syndrome and anti‐N‐methyl‐D‐aspartate receptor positivity: A case report

#### V. Tseriotis ^1^; K. Eleftheriadou^1^; F. Lioliou^1^; P. Papadopoulos^1^; C. Maskalidis^2^; C. Manika^2^; M. Belimezi^3^; T. Mavridis^4^; S. Koukou^1^

##### ^1^Department of Neurology, Agios Pavlos General Hospital of Thessaloniki, Thessaloniki, Greece; ^2^Department of Radiology, Agios Pavlos General Hospital of Thessaloniki, Kalamaria, Thessaloniki, Greece; ^3^Diagnostic Department, Hellenic Pasteur Institute, Vasilissis Sofias 127, 11521, Athens, Greece; ^4^Department of Neurology, Tallaght University Hospital (TUH), Dublin, Ireland

**Background and Aims:** Anti‐NMDAR antibodies, traditionally associated with anti‐NMDAR encephalitis, have also been identified in cases of optic neuritis and myelitis, leading to the broader classification of NMDAR spectrum disorder. Here, we present a case of confirmed anti‐NMDAR positivity in both cerebrospinal fluid and serum, manifesting as cramp‐fasciculation syndrome (CFS) with atypical radiological features, including myelitis and leptomeningeal enhancement.

**Methods:** A 63‐year‐old male patient presenting with painful cramps, spontaneous fasciculations and back pain was admitted and evaluated with a full neurological examination, nerve conduction studies (NCS) and electromyography (EMG), brain and spinal cord MRI, lumbar puncture, and serum/cerebrospinal fluid (CSF) analysis for infectious and autoimmune diseases.

**Figure:**
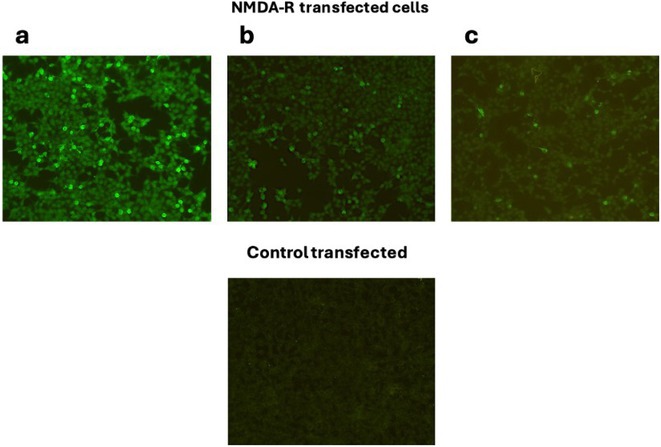
**FIGURE 1** Indirect Immunofluorescence Assay of transfected EU90 cells expressing NMDAR and control transfected cells. a, b: Serum 1:10 and 1:320; c: CSF, undiluted, followed by incubation with anti‐human IgG fluorescent antibody.

**Results:** Neurological examination was remarkable for increased deep‐tendon reflexes and clonus in both lower limbs but there was no muscle weakness. Despite normal NCS, EMG revealed acute‐subacute and chronic denervation in upper and lower limbs (especially tibialis anterior). MRI indicated a short‐segment myelitis lesion at T6 level, with leptomeningeal enhancement. Serum and CSF analysis excluded infectious causes. Type IV oligoclonal bands were detected. An extensive auto‐antibody screening with cell‐ and tissue‐based assays revealed anti‐NMDAR positivity in serum and CSF. Despite relapses, there was clinical and radiological response to immunotherapy (intravenous corticosteroids and plasmapheresis).

**Figure:**
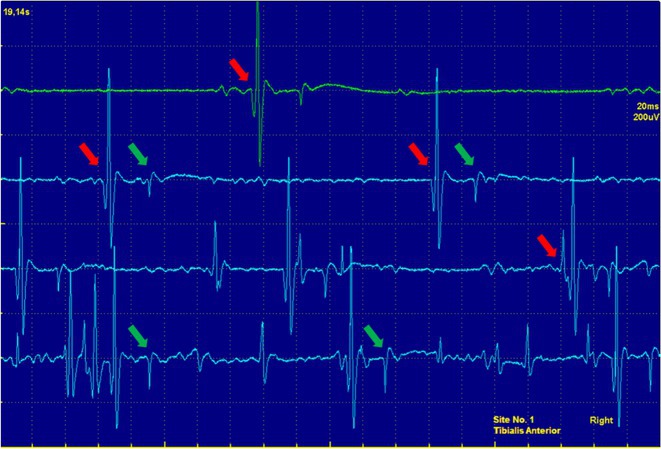
**FIGURE 2** Electromyography with abnormal spontaneous activity. Positive sharp waves (green arrows) and fibrillation potentials (red arrows)

**Figure:**
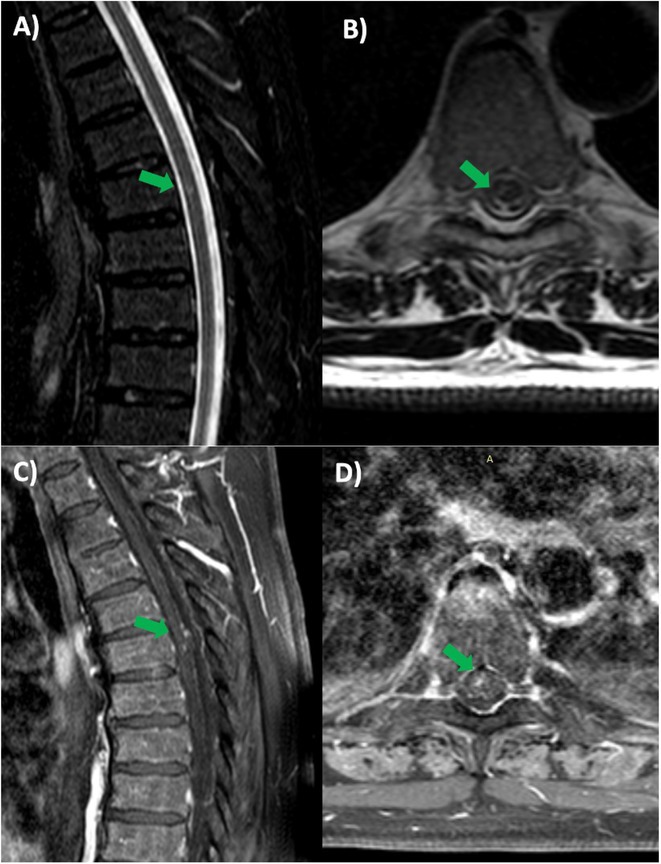
**FIGURE 3** Lesion at T6 level on thoracic spinal cord MRI at first admission A. STIR, B. T2WI, C and D. T1WI with fat suppression after intravenous administration of contrast agent

**Conclusion:** Our anti‐NMDAR case presents atypical clinical and imaging features. Although the exact mechanism remains unclear, cramp‐fasciculation‐like phenotype and leptomeningeal enhancement suggest spinal nerve root involvement. We support the need for increased awareness of the syndrome's diverse phenotypes.

**Disclosure:** Nothing to disclose.

